# Correction: Komiyama et al. RNA-seq and Mitochondrial DNA Analysis of Adrenal Gland Metastatic Tissue in a Patient with Renal Cell Carcinoma. *Biology* 2022, *11*, 589

**DOI:** 10.3390/biology11071037

**Published:** 2022-07-11

**Authors:** Tomoyoshi Komiyama, Hakushi Kim, Masayuki Tanaka, Sanae Isaki, Keiko Yokoyama, Akira Miyajima, Hiroyuki Kobayashi

**Affiliations:** 1 Department of Clinical Pharmacology, Tokai University School of Medicine, Isehara 259-1193, Kanagawa, Japan; hkobayas@is.icc.u-tokai.ac.jp; 2 Department of Urology, Tokai University Hachioji Hospital, Tokyo 192-0032, Tokyo, Japan; 3 Medical Science College Office, Tokai University, Isehara 259-1193, Kanagawa, Japan; matanaka@tokai-u.jp (M.T.); sanaei@tsc.u-tokai.ac.jp (S.I.); kekoyoko@tsc.u-tokai.ac.jp (K.Y.); 4 Department of Urology, Tokai University School of Medicine, Isehara 259-1193, Kanagawa, Japan; akiram@tokai.ac.jp

The authors would like to make the following correction to the published paper [1].

The authors apologize for any inconvenience caused and state that the scientific conclusions are unaffected. This correction was approved by the Academic Editor. The original publication has also been updated.

## Error in Affiliation

In the original publication, there were some errors in the affiliation(s) for Masayuki Tanaka, Sanae Isaki, Keiko Yokoyama and Akira Miyajima. The corrected affiliations appear below.

Tomoyoshi Komiyama ^1,^*^,†^, Hakushi Kim ^2,^*^,†^, Masa **yuki Tanaka ^3^, Sanae Isaki ^3^, Keiko Yokoyama ^3^, Akira Miyajima ^4^ and Hiroyuki Kobayashi ^1^**

^1^  Department of Clinical Pharmacology, Tokai University School of Medicine, Isehara 259-1193, Kanagawa, Japan; hkobayas@is.icc.u-tokai.ac.jp
^2^  Department of Urology, Tokai University Hachioji Hospital, Tokyo 192-0032, Tokyo, Japan
^3^  Medical Science College Office, Tokai University, Isehara 259-1193, Kanagawa, Japan; matanaka@tokai-u.jp (M.T.); sanaei@tsc.u-tokai.ac.jp (S.I.); kekoyoko@tsc.u-tokai.ac.jp (K.Y.)
^4^  Department of Urology, Tokai University School of Medicine, Isehara 259-1193, Kanagawa, Japan; akiram@tokai.ac.jp

* Correspondence: komiyama@tokai-u.jp (T.K.); qblong888@gmail.com (H.K.); Tel.: +81-463-93-1121 (T.K.)
† These authors contributed equally to this study.
