# Supplementary material for: RNA-seq and Mitochondrial DNA Analysis of Adrenal Gland Metastatic Tissue in a Patient with Renal Cell Carcinoma
Source: Biology (Basel). 2022 Apr 13;11(4):589. doi: 10.3390/biology11040589 (PMC9030821; doi:10.3390/biology11040589)
Supplement: Supplementary file 1 [file biology-11-00589-s001.zip › Komiyama_Supplymentary.pdf]

Supplementary Materials

Supplementary Tables

Table S1. Primer set used for amplifying the whole mitochondrial DNA region.

Primer	Forward/Reverse	Sequence (5'–3')
homoF1	F	AGGACTATTCCTAGCCATGCACTACTCA
homoR1	R	AAATAATAGGATGAGGCAGGAATCAAAGAC
homoF2	F	GAACTGTATCCGACATCTGGTTCCTACT
homoR2	R	TTTAGCTCAGAGCGGTCAAGTTAAGTTG
homoF3	F	TATGAAACTTAAGGGTCGAAGGTGGATT
homoR3	R	GCCATCTTAACAAACCCTGTTCTTG
homoF4	F	ATCCAATAACTTGACCAACGGAACA
homoR4	R	AAGGATTATGGATGCGGTTGCTT
homoF5	F	CCACCTATCACACCCCATCCTAA
homoR5	R	CAGTTGCCAAAGCCTCCGATT
homoF6	F	CAATGCTTCACTCAGCCATTTTACCT
homoR6	R	TGTAGACCTACTTGCGCTGCATGT
homoF7	F	GATGCATACACCACATGAAACATCCTA
homoR7	R	GTGATTGGTGGGTCATTATGTGTTGT
homoF8	F	ACTTCTTACCACAAGGCACACCTACA
homoR8	R	GGCAATAGGCACAATATTGGCTAAGA
homoF9	F	GATAATCATATTTACCAAATGCCCCTCATT
homoR9	R	GCAGTTCTTGTGAGCTTTCTCGGTAA
homoF10	F	CACTCACCCACCACATTAACAACA
homoR10	R	GGCTGTTAGAAGTCCTAGGAAAGTGACAG
homoF11	F	GCATTAGCAGGAATACCTTTCCTCACA
homoR11	R	CAAGAATAGGAGGTGGAGTGCTGCTA
Homo_mt_Gap_1F	F	GGCCACCAATGGTACTGAAC
Homo_mt_Gap_1R	R	GCAATGAATGAAGCGAACAG
Homo_mt_Gap_2F	F	TTAGACTGAGCCGAATTGGT
Homo_mt_Gap_2R	R	AGCCTCTGTTGTCAGATCCA
Homo_mt_Gap_3F	F	TCGGAGGACAACCAGTAAGC
Homo_mt_Gap_3R	R	GCACTCTTGTGCGGGATATT
HUMmt-148F	F	ATCCCATTATTTATCGCACCT
HUMmt-1038R	R	CTATTGTGTGTTCAGATATGTT
HUMmt-1038F	F	ACATATCTGAACACACAATAGCT
HUMmt-1914F	R	AGACGAGCTACCTAAGAACAGCT
HUMmt-2022R	F	AACTAAGATTCTATCTTGGACA
HUMmt-2850R	R	TGGTGAAGTCTTAGCATGTACT
HUMmt-4885F	F	ATCTCAATCATATACCAAATCT
HUMmt-5606R	R	ATTTGCGTTCAGTTGATGCAGA
HUMmt-13601F	F	TATAGCACTCGAATAATTCTT
HUMmt-14133F	F	ACTCCTAATCACATAACCTATT
HUMmt-14201R	R	TGATTAGTAGTAGTTACTGGTT
HUMmt-15052R	R	AGTAGAGAAATGATCCGTAATA
F817-837	F	GGGAAACAGCAGTGATTAACC
R1235-1255	R	CAAGAGGTGGTGAGGTTGATC
F1557-1579	F	GAGGAGACAAGTCGTAACATGGT
R2341-2360	R	ATCTGACGCAGGCTTATGC

F2592-2613	F	CGTGCAAAGGTAGCATAATCAC
R3071-3091	R	ACTCCGGTCTGAACTCAGATC
F5536-5556	F	ACAGACCAAGAGCCTTCAAAG
R6015-6032	R	AGCTCGGCTCGAATAAGG
F6818-6838	F	CATATTTCACCTCCGCTACCA
R7419-7440	R	GTATACGGGTTCTTCGAATGTG
F3274	F	ACAGTCAGAGGTTCAATTCCTCTTCT
ND1-R	R	CTGAGAATCCAAAATTCTCCGTG
ND1-seqR	R	CCTGATCAGAGGATTGAGTAAACG
ND4-seqF	F	CTGAACGCAGGCACATACTTC
ND4-seqR	R	ATAAGTGGAGTCCGTAAAGAGGTATCT
ND5-seqF	F	CCAACTGTTCATCGGCTGAGA
ND5-seqR	R	TGCTAGGAGGAGGCCTAGTAGTG

Supplementary Figures

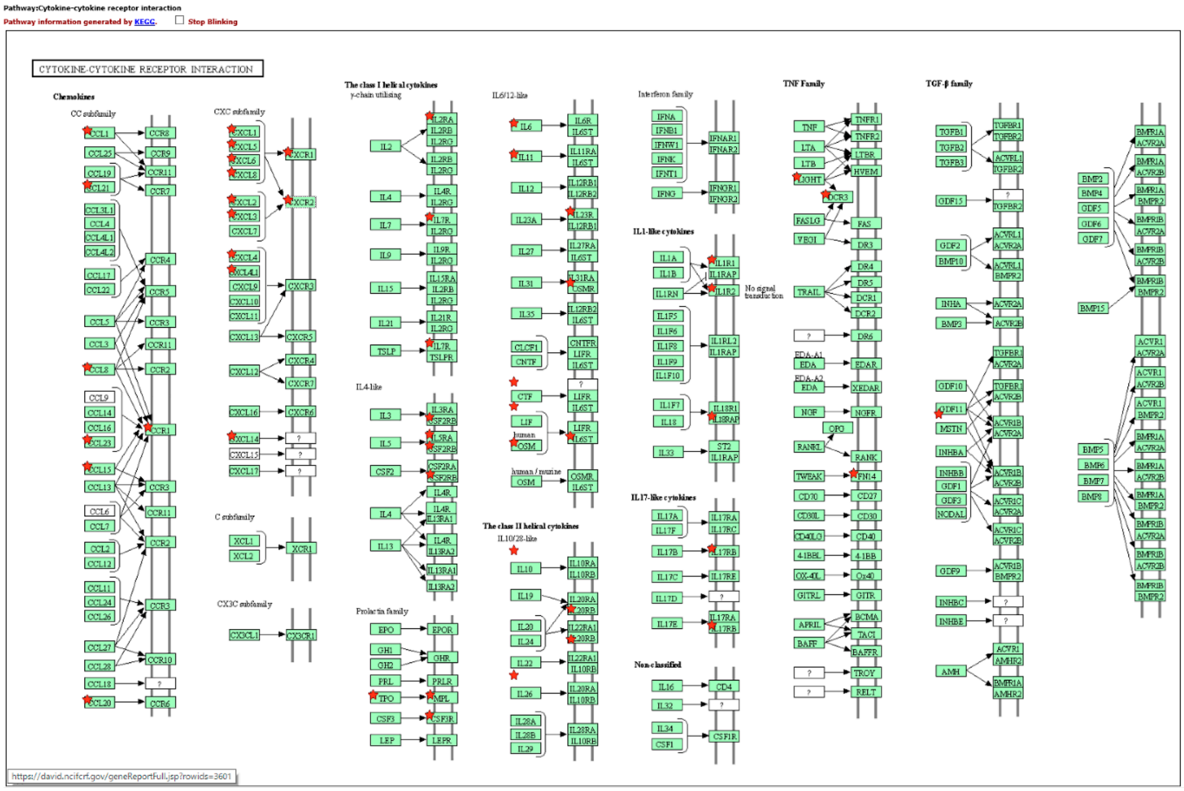

Figure S1. K58-M using the Kyoto Encyclopedia of Genes and Genomes (KEGG) analysis; K58-M: Fold change ≥ 2 upregulated 2,243 genes (2,152 genes*). * Number of genes corresponds to DAIVID.

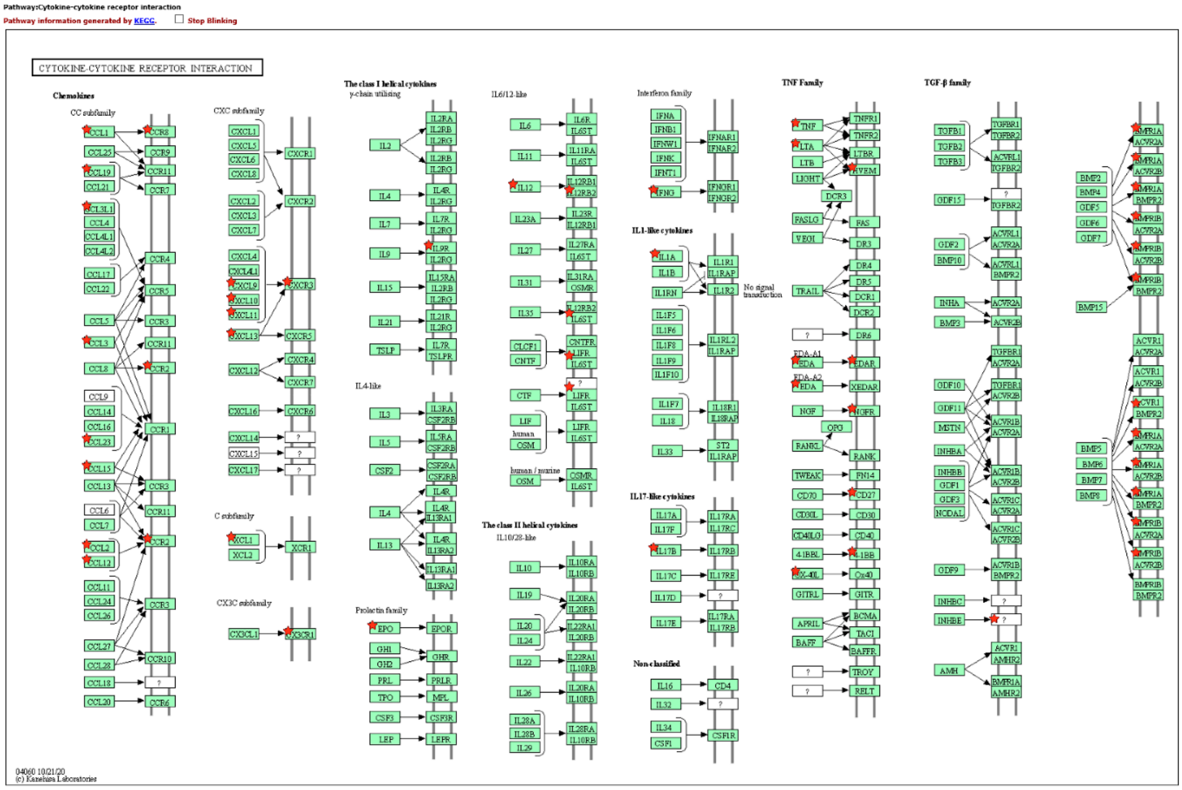

Figure S2. K58-R using the Kyoto Encyclopedia of Genes and Genomes (KEGG) analysis; K58-R: Fold change ≥ 2 upregulated 1,897 genes (1,810 genes*). * Number of genes corresponds to DAIVID.
