# Supplementary material for: RNA-seq and Mitochondrial DNA Analysis of Adrenal Gland Metastatic Tissue in a Patient with Renal Cell Carcinoma
Source: Biology (Basel). 2022 Apr 13;11(4):589. doi: 10.3390/biology11040589 (PMC9030821; doi:10.3390/biology11040589)
Supplement: Supplementary file 1 [file Table S2. Gene expression analysis_list of genes..pdf › Table S2. Gene expression analysis_list of genes..pdf]

Table S2. Gene expression analysis: list of genes.

tracking_id	clas	nea	gene_id	gene_short_name	tss_id	locus	length	cove	link_for_IGV	K58-R:B07467					K58-M:B07468					K58-M:B07468 K58-R:B07467	
										count	FPKM	conf_lo	conf_hi	status	count	FPKM	conf_lo	conf_hi	status	status	log2(fold_
ENSG0000000	-	-	ENSG0000000000	'TSPAN6	-	X:100627108-100639991	-	-	IGV_LINK	464.496	13.3564	4.37341	22.3394	OK	954.914	27.3651	11.3722	43.3579	OK	OK	1.0348
ENSG0000000	-	-	ENSG0000000000	'TNMD	-	X:100584801-100599885	-	-	IGV_LINK	0	0	0	0	OK	1.03122	0.04987	0	0.299128	OK	NOTEST	inf
ENSG0000000	-	-	ENSG0000000041	'DPM1	-	20:50888918-50958555	-	-	IGV_LINK	415.04	25.7201	0	57.8283	OK	474.363	29.4958	0	67.5363	OK	OK	0.197612
ENSG0000000	-	-	ENSG0000000045	'SCYL3	-	1:169662006-169894267	-	-	IGV_LINK	363.645	6.28923	0	13.29	OK	303.18	5.46196	0	12.5641	OK	OK	-0.20346
ENSG0000000	-	-	ENSG0000000046	'C1orf112	-	1:169662006-169894267	-	-	IGV_LINK	101.821	2.35946	0	7.84206	OK	61.8735	1.468	0	6.35202	OK	OK	-0.68461
ENSG0000000	-	-	ENSG0000000093	'FGR	-	1:27612063-27635277	-	-	IGV_LINK	808.747	25.966	6.60474	45.3273	OK	671.327	22.6572	5.32412	39.9902	OK	OK	-0.19666
ENSG0000000	-	-	ENSG0000000097	'CFH	-	1:196651877-196747504	-	-	IGV_LINK	585.711	9.53876	3.09165	15.9859	OK	5839.83	96.1284	27.7766	164.48	OK	OK	3.33309
ENSG0000000	-	-	ENSG0000000103	'FUCA2	-	6:143450806-143511690	-	-	IGV_LINK	931.901	25.8454	7.25543	44.4353	OK	838.386	23.168	6.478	39.8581	OK	OK	-0.15777
ENSG0000000	-	-	ENSG0000000108	'GCLC	-	6:53497340-53617171	-	-	IGV_LINK	583.772	19.2853	1.62387	36.9468	OK	654.828	23.55	0	47.6897	OK	OK	0.288221
ENSG0000000	-	-	ENSG0000000116	'NFYA	-	6:41026910-41099976	-	-	IGV_LINK	533.346	10.5982	0	22.3461	OK	295.962	5.40304	0	11.7287	OK	OK	-0.97197
ENSG0000000	-	-	ENSG0000000146	'STPG1	-	1:24307555-24472976	-	-	IGV_LINK	179.398	4.87061	0	17.2364	OK	160.871	4.19721	0	15.1095	OK	OK	-0.21467
ENSG0000000	-	-	ENSG0000000146	'NIPAL3	-	1:24307555-24472976	-	-	IGV_LINK	1396.4	18.233	5.44096	31.025	OK	2034.61	27.1367	8.29694	45.9765	OK	OK	0.573698
ENSG0000000	-	-	ENSG0000000149	'LAS1L	-	X:65512581-65534775	-	-	IGV_LINK	925.113	21.5087	4.59617	38.4212	OK	774.45	18.4168	4.6655	32.1681	OK	OK	-0.2239
ENSG0000000	-	-	ENSG0000000156	'ENPP4	-	6:46129992-46146699	-	-	IGV_LINK	382.07	4.80782	1.63578	7.97986	OK	267.087	3.36178	1.04499	5.67858	OK	OK	-0.51616
ENSG0000000	-	-	ENSG0000000161	'SEMA3F	-	3:49940006-50189075	-	-	IGV_LINK	1134.57	21.6865	0	61.5383	OK	917.79	19.8899	0	58.2752	OK	OK	-0.12476
ENSG0000000	-	-	ENSG0000000162	'CFTR	-	7:117465783-117874139	-	-	IGV_LINK	12.6064	1.46641	0	7.73535	OK	12.3747	1.55553	0	6.68806	OK	OK	0.085111
ENSG0000000	-	-	ENSG0000000162	'ANKIB1	-	7:92246233-92401384	-	-	IGV_LINK	1230.58	17.612	4.52649	30.6975	OK	820.855	12.2057	2.41634	21.995	OK	OK	-0.52901
ENSG0000000	-	-	ENSG0000000163	'CYP51A1	-	7:92112150-92180725	-	-	IGV_LINK	268.613	6.19933	1.0951	11.3036	OK	246.463	5.15915	1.06187	9.25643	OK	OK	-0.26498
ENSG0000000	-	-	ENSG0000000163	'KRIT1	-	7:92198968-92246166	-	-	IGV_LINK	847.086	20.8463	4.02682	37.6657	OK	649.241	16.5218	2.69836	30.3453	OK	OK	-0.33542
ENSG0000000	-	-	ENSG0000000201	'RAD52	-	12:912076-1495933	-	-	IGV_LINK	282.968	13.4512	0	40.739	OK	178.402	7.82697	0	28.0529	OK	OK	-0.78121
ENSG0000000	-	-	ENSG0000000207	'MYH16	-	7:99238828-99325826	-	-	IGV_LINK	7.75776	0.2773	0	1.15198	OK	8.2498	0.316216	0	1.22686	OK	NOTEST	0.189462
ENSG0000000	-	-	ENSG0000000233	'BAD	-	11:64269829-64289500	-	-	IGV_LINK	600.257	49.2024	3.05259	95.3521	OK	633.172	52.541	3.14907	101.933	OK	OK	0.094717
ENSG0000000	-	-	ENSG0000000254	'LAP3	-	4:17577191-17614571	-	-	IGV_LINK	3581.18	123.308	48.53	198.086	OK	3901.12	131.965	52.8562	211.073	OK	OK	0.097884
ENSG0000000	-	-	ENSG0000000258	'CD99	-	X:2691178-2741309	-	-	IGV_LINK	1431.31	80.5891	34.7435	126.435	OK	2227.45	129.162	51.815	206.51	OK	OK	0.68053
ENSG0000000	-	-	ENSG0000000258	'HS3ST1	-	4:11393149-11429765	-	-	IGV_LINK	117.336	0.874101	0	1.93394	OK	278.431	4.54234	0	12.3898	OK	OK	2.37757
ENSG0000000	-	-	ENSG0000000272	'AOC1	-	7:150824626-150861504	-	-	IGV_LINK	2360.3	55.4641	23.432	87.4961	OK	18773.5	444.333	59.2264	829.439	OK	OK	3.00201
ENSG0000000	-	-	ENSG0000000274	'WNT16	-	7:121325366-121341104	-	-	IGV_LINK	2.90916	0.055142	0	0.196824	OK	2.06245	0.039091	0	0.158739	OK	NOTEST	-0.4963
ENSG0000000	-	-	ENSG0000000274	'HECW1	-	7:42954134-43566001	-	-	IGV_LINK	1.93944	0.021875	0	0.632237	OK	65.9984	1.86172	0	5.46735	OK	OK	6.41122
ENSG0000000	-	-	ENSG0000000282	'MAD1L1	-	7:1815792-2233243	-	-	IGV_LINK	362.675	10.5405	1.94892	19.1322	OK	353.71	11.0214	0.927979	21.1148	OK	OK	0.064356
ENSG0000000	-	-	ENSG0000000283	'LASP1	-	17:38869858-38921770	-	-	IGV_LINK	4018.52	60.9602	23.8617	98.0587	OK	4084.68	61.1203	21.3076	100.933	OK	OK	0.003784
ENSG0000000	-	-	ENSG0000000291	'SNX11	-	17:48103356-48123074	-	-	IGV_LINK	459.648	13.3549	3.54041	23.1694	OK	461.989	13.0818	2.91839	23.2452	OK	OK	-0.02981
ENSG0000000	-	-	ENSG0000000293	'TMEM176A	-	7:150791284-150805120	-	-	IGV_LINK	8687.73	449.134	0	1050.4	OK	15777.7	824.063	0	2028.83	OK	OK	0.875607
ENSG0000000	-	-	ENSG0000000305	'M6PR	-	12:8681599-8949955	-	-	IGV_LINK	4028.22	139.44	0	282.917	OK	4548.73	156.832	4.45079	309.213	OK	OK	0.169572
ENSG0000000	-	-	ENSG0000000309	'KLHL13	-	X:117897812-118117340	-	-	IGV_LINK	106.669	2.08503	0.071055	4.099	OK	239.244	4.9117	1.17865	8.64474	OK	OK	1.23615
ENSG0000000	-	-	ENSG0000000313	'CYP26B1	-	2:72129237-72148038	-	-	IGV_LINK	10.6669	0.14188	0	0.487261	OK	46.4051	0.588578	0	1.36825	OK	OK	2.05256
ENSG0000000	-	-	ENSG0000000314	'ICA1	-	7:8113183-8344516	-	-	IGV_LINK	330.675	13.3752	2.4248	24.3257	OK	386.709	16.2459	2.14067	30.3511	OK	OK	0.280511
ENSG0000000	-	-	ENSG0000000324	'DBNDD1	-	16:90004864-90047773	-	-	IGV_LINK	314.189	9.51659	0	20.3842	OK	131.997	4.00769	0	11.1467	OK	OK	-1.24768
ENSG0000000	-	-	ENSG0000000339	'ALS2	-	2:201700553-201781189	-	-	IGV_LINK	585.711	5.36382	1.95678	8.77087	OK	513.55	4.77422	1.57657	7.97186	OK	OK	-0.168
ENSG0000000	-	-	ENSG0000000340	'CASP10	-	2:201182880-201229406	-	-	IGV_LINK	631.288	9.52263	1.02994	18.0153	OK	922.946	13.8576	1.76412	25.9511	OK	OK	0.541246
ENSG0000000	-	-	ENSG0000000340	'CFLAR	-	2:201116103-201176687	-	-	IGV_LINK	5878.96	86.354	0	190.125	OK	7265.79	115.751	0	253.356	OK	OK	0.422689
ENSG0000000	-	-	ENSG0000000343	'TFPI	-	2:187001875-187565760	-	-	IGV_LINK	1739.68	48.3236	2.21493	94.4323	OK	4007.34	114.913	4.3402	225.487	OK	OK	1.24975
ENSG0000000	-	-	ENSG0000000350	'NDUFAF7	-	2:37167819-37324808	-	-	IGV_LINK	324.856	11.9101	0	31.3418	OK	274.306	11.8197	0	32.0906	OK	OK	-0.011
ENSG0000000	-	-	ENSG0000000375	'RBM5	-	3:49940006-50189075	-	-	IGV_LINK	3026.5	72.2989	0	172.791	OK	2420.38	63.4616	0	151.63	OK	OK	-0.18809
ENSG0000000	-	-	ENSG0000000398	'MTMR7	-	8:17224963-17413528	-	-	IGV_LINK	19.3944	0.335201	0	2.19362	OK	37.1241	0.646971	0	3.52968	OK	OK	0.948674
ENSG0000000	-	-	ENSG0000000398	'SLC7A2	-	8:17497087-17570573	-	-	IGV_LINK	440.253	3.6492	1.14437	6.15402	OK	1233.35	9.72153	3.67278	15.7703	OK	OK	1.4136
ENSG0000000	-	-	ENSG0000000405	'ARF5	-	7:127580617-127602144	-	-	IGV_LINK	1445.73	107.078	13.7452	200.411	OK	1186.94	84.0595	22.0962	146.023	OK	OK	-0.34918
ENSG0000000	-	-	ENSG0000000413	'SARM1	-	17:28357580-28407197	-	-	IGV_LINK	334.232	6.60202	0	26.5283	OK	329.719	6.59993	0	27.1311	OK	OK	-0.00046
ENSG0000000	-	-	ENSG0000000414	'POLDIP2	-	17:28347176-28357522	-	-	IGV_LINK	1608.77	47.3388	20.3718	74.3059	OK	1865.49	55.2104	23.7272	86.6936	OK	OK	0.221916
ENSG0000000	-	-	ENSG0000000439	'PLXND1	-	3:129555174-129606818	-	-	IGV_LINK	2840.31	74.719	4.31639	145.122	OK	4525.02	112.592	0	226.217	OK	OK	0.59156
ENSG0000000	-	-	ENSG0000000445	'AK2	-	1:32986951-33080996	-	-	IGV_LINK	3221.41	132.227	38.4541	226	OK	3961.97	169.308	0	348.457	OK	OK	0.356627
ENSG0000000	-	-	ENSG0000000446	'CD38	-	4:15778274-15853230	-	-	IGV_LINK	537.225	8.13006	0.562698	15.6974	OK	436.208	6.32796	1.41393	11.242	OK	OK	-0.36153
ENSG0000000	-	-	ENSG0000000447	'FKBP4	-	12:1946053-2941140	-	-	IGV_LINK	1238.33	26.5517	0	70.2707	OK	1095.16	24.7822	0	67.2707	OK	OK	-0.0995
ENSG0000000	-	-	ENSG0000000448	'KDM1A	-	1:23019447-23177808	-	-	IGV_LINK	1010.45	22.5026	1.64015	43.365	OK	967.289	21.8549	1.14219	42.5676	OK	OK	-0.04213
ENSG0000000	-	-	ENSG0000000453	'RBM6	-	3:49940006-50189075	-	-	IGV_LINK	3193.29	67.0922	0	152.402	OK	2540.85	55.09	0	122.355	OK	OK	-0.28436
ENSG0000000	-	-	ENSG0000000466	'CAMKK1	-	17:3860314-3894891	-	-	IGV_LINK	265.703	4.50556	1.38907	7.62205	OK	300.086	5.02942	1.63749	8.42135	OK	OK	0.158687
ENSG0000000	-	-	ENSG0000000470	'RECQL	-	12:21437614-21501669	-	-	IGV_LINK	1082.21	24.3773	2.2078	46.5468	OK	1107.54	23.5123	2.78019	44.2443	OK	OK	-0.05213
ENSG0000000	-	-	ENSG0000000476	'VPS50	-	7:93232339-93361121	-	-	IGV_LINK	323.887	6.59252	0.635498	12.5495	OK	338.242	9.12917	0.328869	17.9295	OK	OK	0.469652
ENSG0000000	-	-	ENSG0000000477	'HSPB6	-	19:35754565-35758079	-	-	IGV_LINK	8.72748	0.38297	0	0.993271	OK	14.4371	0.634007	0	1.52188	OK	OK	0.727267
ENSG0000000	-	-	ENSG0000000477	'ARHGAP33	-	19:35774531-35797902	-	-	IGV_LINK	1006.57	26.3894	0	53.5041	OK	599.142	15.3226	0	31.9601	OK	OK	-0.7843
ENSG0000000	-	-	ENSG0000000477	'NDUFAB1	-	16:23581001-23596356	-	-	IGV_LINK	718.563	74.1617	30.4014	117.922	OK	887.885	92.1444	38.3657	145.923	OK	OK	0.313223
ENSG0000000	-	-	ENSG0000000479	'PDK4	-	7:95583498-95596491	-	-	IGV_LINK	1219.91	21.9323	5.82203	38.0426	OK	6688.53	124.878	18.4768	231.279	OK	OK	2.50939
ENSG0000000	-	-	ENSG0000000480	'SLC22A16	-	6:110424686-110476641	-	-	IGV_LINK	0.969721	0.186148	0	0.783954	OK	2.06245	0.135553	0	0.565206	OK	NOTEST	-0.45759
ENSG0000000	-	-	ENSG0000000483	'ZMYND10	-	3:50341105-50346852	-	-	IGV_LINK	102.79	3.98763	0.106662	7.8686	OK	83.5292	2.97049	0	6.17366	OK	OK	-0.42483
ENSG0000000	-	-	ENSG0000000484	'ABCB5	-	7:20615206-20777038	-	-	IGV_LINK	14.5458	0.550184	0	1.45872	OK	0	0	0	0	OK	NOTEST	'-inf
ENSG0000000	-	-	ENSG0000000484	'ARX	-	X:25003693-25016420	-	-	IGV_LINK	5.81832	0.121286	0	2.38928	OK	17.5308	0.365595	0	2.67492	OK	NOTEST	1.59183
ENSG0000000	-	-	ENSG0000000486	'SLC25A13	-	7:96120219-96322147	-	-	IGV_LINK	469.345	11.9859	0.941284	23.0305	OK	585.736	15.5131	0	38.2115	OK	OK	0.372152
ENSG0000000	-	-	ENSG0000000486	'ST7	-	7:116952445-117230103	-	-	IGV_LINK	536.579	17.5254	4.08123	30.9695	OK	417.646	12.6642	2.62247	22.7059	OK	OK	-0.4687
ENSG0000000	-	-	ENSG0000000489	'CDC27	-	17:47117702-47189422	-	-	IGV_LINK	622.561	17.5411	0	35.5893	OK	687.827	20.6208	0	41.9738	OK	OK	0.23336
ENSG0000000	-	-	ENSG0000000493	'SLC4A1	-	17:44248384-44268141	-	-	IGV_LINK	0.969721	0.043356	0	0.439701	OK	4.1249	0.132969	0	0.666062	OK	NOTEST	1.61679
ENSG0000000	-	-	ENSG0000000494	'CALCR	-	7:93424486-93574730	-	-	IGV_LINK	8.72748	0.22335	0	0.581532	OK	16.4996	0.385216	0	0.883638	OK	NOTEST	0.786361
ENSG0000000	-	-	ENSG0000000496	'HCCS	-	X:11111300-11123078	-	-	IGV_LINK	235.642	10.6868	2.55757	18.816	OK	355.773	16.0245	4.66396	27.3851	OK	OK	0.584455
ENSG0000000	-	-	ENSG0000000497	'DVL2	-	17:7225340-7234545	-	-	IGV_LINK	467.405	16.2464	2.79027	29.7025	OK	449.614	15.1503	3.14448	27.1561	OK	OK	-0.10078
ENSG0000000	-	-	ENSG0000000500	'PRSS22	-	16:2852726-2859726	-	-	IGV_LINK	0	0	0	0	OK	2.06245	0.141037	0	0.713987	OK	NOTEST	inf
ENSG0000000	-	-	ENSG0000000500	'UPF1	-	19:18831937-18868236	-	-	IGV_LINK	1729.01	20.8751	5.75746	35.9928	OK	1983.05	24.1205	6.82073	41.4203	OK	OK	0.208476
ENSG0000000	-	-	ENSG0000000502	'SKAP2	-	7:26667061-26995239	-	-	IGV_LINK	439.283	10.2261	0	21.2508	OK	1014.73	23.8866	0.601684	47.1715	OK	OK	1.22394
ENSG0000000	-	-	ENSG0000000502	'SLC25A5	-	X:119466033-119471319	-	-	IGV_LINK	2464.06	129.827	53.5588	206.095	OK	2329.54	121.375	47.0908	195.659	OK	OK	-0.09712
ENSG0000000	-	-	ENSG0000000505	'MCUB	-	4:109560204-109703583	-	-	IGV_LINK	250.188	11.9855	0	27.1089	OK	262.962	12.2812	0	28.7868	OK	OK	0.035172
ENSG0000000	-	-	ENSG0000000507	'HOXA11	-	7:27181509-27193448	-	-	IGV_LINK	8.72748	0.250364	0	0.639049	OK	2.06245	0.059197	0	0.209553	OK	NOTEST	-2.08043
ENSG0000000	-	-	ENSG0000000507	'POLR2J	-	7:102464928-102478907	-	-	IGV_LINK	450.92	33.759	8.41518	59.1027	OK	538.299	40.2656	9.95818	70.573	OK	OK	0.254277
ENSG0000000	-	-	ENSG0000000510	'DHX33	-	17:5282264-5469060	-	-	IGV_LINK	292.856	3.57359	0	10.3752	OK	283.587	3.45715	0	10.0308	OK	OK	-0.04779
ENSG0000000	-	-	ENSG0000000510	'MEOX1	-	17:43640387-43661954	-	-	IGV_LINK	11.6366	0.298841	0	0.716155	OK	11.3435	0.463592	0	1.15644	OK	NOTEST	0.633479
ENSG0000000	-	-	ENSG0000000510	'THSD7A	-	7:11180901-11832198	-	-	IGV_LINK	222.066	1.23289	0.170816	2.29496	OK	259.869	1.64958	0	4.15548	OK	OK	0.420058
ENSG0000000	-	-	ENSG0000000515	'LIG3	-	17:34927858-35121522	-	-	IGV_LINK	411.161	6.88827	0	19.4225	OK	382.584	6.36615	0	19.5656	OK	OK	-0.11372
ENSG0000000	-	-	ENSG0000000517	'RPAP3	-	12:47661248-47706061	-	-	IGV_LINK	321.947	7.7159	0.927207	14.5046	OK	266.056	6.00284	1.04593	10.9597	OK	OK	-0.36219
ENSG0000000	-	-	ENSG0000000518	'ACSM3	-	16:20610242-20925006	-	-	IGV_LINK	439.283	16.4785	0	40.3618	OK	459.926	20.8683	0	56.0393	OK	OK	0.340724
ENSG0000000	-	-	ENSG0000000518	'REXO5	-	16:20610242-20925006	-	-	IGV_LINK	70.7896	1.81883	0	9.07359	OK	65.9984	1.92196	0	11.4807	OK	OK	0.079572
ENSG0000000	-	-	ENSG0000000519	'CIAPIN1	-	16:57428168-57487327	-	-	IGV_LINK	495.527	25.3305	0	94.9045	OK	688.858	27.2258	0	89.8337	OK	OK	0.1041
ENSG0000000	-	-	ENSG0000000520	'SPPL2B	-	19:2252251-2354806	-	-	IGV_LINK	1463.22	48.4939	0	145.593	OK	2007.44	65.5075	0	198.653	OK	OK	0.433856
ENSG0000000	-	-	ENSG0000000523	'FAM214B	-	9:35104111-35116341	-	-	IGV_LINK	538.195	12.3092	2.45261	22.1658	OK	645.547	15.0972	3.15129	27.0432	OK	OK	0.294546
ENSG0000000	-	-	ENSG0000000524	'COPZ2	-	17:48026166-48038030	-	-	IGV_LINK	220.127	19.635	2.86802	36.4021	OK	219.651	19.0438	5.30272	32.7848	OK	OK	-0.04411
ENSG0000000	-	-	ENSG0000000524	'PRKAR2B	-	7:107044648-107161811	-	-	IGV_LINK	87.2748	1.37676	0.090349	2.66317	OK	38.1553	0.800381	0	2.07247	OK	OK	-0.78251
ENSG0000000	-	-	ENSG0000000530	'MSL3	-	X:11758158-11775753	-	-	IGV_LINK	768.988	24.9743	4.49471	45.4538	OK	729.076	22.5475	3.86137	41.2335	OK	OK	-0.14748
ENSG0000000	-	-	ENSG0000000533	'CREBBP	-	16:3725053-3880726	-	-	IGV_LINK	1953.02	22.7258	0	52.5619	OK	2042.86	20.9279	0	48.0876	OK	OK	-0.11891
ENSG0000000	-	-	ENSG0000000537	'TSPOAP1	-	17:58301227-58417595	-	-	IGV_LINK	1910.35	46.3933	0	112.034	OK	1014.73	22.2502	0	57.1029	OK	OK	-1.0601
ENSG0000000	-	-	ENSG0000000538	'MPO	-	17:58269855-58280935	-	-	IGV_LINK	4.8486	0.248965	0	1.10577	OK	3.09367	0.057325	0	0.68775	OK	NOTEST	-2.1187
ENSG0000000	-	-	ENSG0000000542	'PON1	-	7:95297675-95324707	-	-	IGV_LINK	3.87888	0.479701	0	1.26807	OK	1.03122	0.024272	0	0.335396	OK	NOTEST	-4.30477
ENSG0000000	-	-	ENSG0000000543	'GCFC2	-	2:75646782-75710989	-	-	IGV_LINK	303.523	7.1752	0	15.2069	OK	239.244	8.27421	0	21.2048	OK	OK	0.205602
ENSG0000000	-	-	ENSG0000000544	'WDR54	-	2:74421677-74425755	-	-	IGV_LINK	494.557	29.6221	10.2413	49.003	OK	743.513	47.8282	17.9386	77.7179	OK	OK	0.691188
ENSG0000000	-	-	ENSG0000000546	'CROT	-	7:87345680-87399795	-	-	IGV_LINK	192.974	4.5323	0	9.11278	OK	215.526	5.06585	0	10.2211	OK	OK	0.16056
ENSG0000000	-	-	ENSG0000000547	'ABCB4	-	7:87401696-87480435	-	-	IGV_LINK	110.548	2.56671	0	6.7168	OK	361.96	8.07284	0	17.9286	OK	OK	1.65315
ENSG0000000	-	-	ENSG0000000548	'KMT2E	-	7:105013424-105399308	-	-	IGV_LINK	1871.56	20.3374	0	41.9118	OK	1529.31	19.8428	0	47.5557	OK	OK	-0.03552
ENSG0000000	-	-	ENSG0000000548	'RHBDD2	-	7:75842601-75888926	-	-	IGV_LINK	699.168	27.4484	9.7992	45.0976	OK	876.541	34.1825	12.5905	55.7745	OK	OK	0.316536
ENSG0000000	-	-	ENSG0000000551	'SOX8	-	16:981807-986979	-	-	IGV_LINK	2.90916	0.041195	0	0.121609	OK	9.28102	0.141979	0	0.339941	OK	NOTEST	1.78513
ENSG0000000	-	-	ENSG0000000570	'IBTK	-	6:82169982-82247754	-	-	IGV_LINK	898.931	11.0236	4.00591	18.0413	OK	558.924	6.87255	2.48225	11.2629	OK	OK	-0.68168
ENSG0000000	-	-	ENSG0000000580	'ZNF195	-	11:3336720-3379222	-	-	IGV_LINK	382.07	12.1763	1.96546	22.3871	OK	449.614	13.4908	2.52427	24.4574	OK	OK	0.147906
ENSG0000000	-	-	ENSG0000000581	'MYCBP2	-	13:76990659-77327050	-	-	IGV_LINK	1535.07	24.4048	0	62.5867	OK	993.07	18.0825	0	46.8913	OK	OK	-0.43257
ENSG0000000	-	-	ENSG0000000581	'FBXL3	-	13:76990659-77327050	-	-	IGV_LINK	1096.75	22.5466	0	52.3146	OK	1120.94	22.9178	0	48.9286	OK	OK	0.023556
ENSG0000000	-	-	ENSG0000000584	'ITGAL	-	16:30472657-30523185	-	-	IGV_LINK	2392.3	40.0027	1.04869	78.9567	OK	1211.69	20.7996	0.205848	41.3934	OK	OK	-0.94354
ENSG0000000	-	-	ENSG0000000588	'PDK2	-	17:50094736-50129882	-	-	IGV_LINK	867.9	73.035	8.00137	138.069	OK	791.981	69.4738	7.32044	131.627	OK	OK	-0.07212
ENSG0000000	-	-	ENSG0000000588	'ITGA3	-	17:50055967-50090481	-	-	IGV_LINK	3510.39	52.9797	0	111.863	OK	3633.01	49.7282	6.5561	92.9004	OK	OK	-0.09137
ENSG0000000	-	-	ENSG0000000588	'ZFX	-	X:24146224-24216255	-	-	IGV_LINK	479.042	5.61819	0.049092	11.1873	OK	479.52	5.63324	0	12.0782	OK	OK	0.003861
ENSG0000000	-	-	ENSG0000000589	'LAMP2	-	X:120427826-120469365	-	-	IGV_LINK	2317.63	70.9757	26.0578	115.894	OK	3287.55	102.122	38.2995	165.945	OK	OK	0.524899
ENSG0000000	-	-	ENSG0000000596	'ITGA2B	-	17:44372179-44389505	-	-	IGV_LINK	38.7888	1.9316	0	6.27974	OK	62.9047	1.53408	0	3.84789	OK	OK	-0.33243
ENSG0000000	-	-	ENSG0000000598	'ASB4	-	7:95478443-95540232	-	-	IGV_LINK	0	0	0	0	OK	1324.09	21.4256	9.01694	33.8343	OK	OK	inf
ENSG0000000	-	-	ENSG0000000600	'GDE1	-	16:19501688-19522145	-	-	IGV_LINK	783.534	20.2125	5.53643	34.8886	OK	1279.75	36.1484	10.707	61.5899	OK	OK	0.838684
ENSG0000000	-	-	ENSG0000000601	'C19orf60	-	19:18571729-18621039	-	-	IGV_LINK	593.469	42.9008	0	147.016	OK	694.014	50.3658	0	147.879	OK	OK	0.231441
ENSG0000000	-	-	ENSG0000000601	'CRLF1	-	19:18571729-18621039	-	-	IGV_LINK	30.0613	1.93754	0	21.2633	OK	27.8431	2.06284	0	23.563	OK	OK	0.090405
ENSG0000000	-	-	ENSG0000000602	'OSBPL7	-	17:47807371-47821834	-	-	IGV_LINK	514.922	12.2558	0.685902	23.8257	OK	566.143	11.4331	1.5813	21.285	OK	OK	-0.10024
ENSG0000000	-	-	ENSG0000000604	'TMEM98	-	17:32927909-32945106	-	-	IGV_LINK	512.982	21.7333	6.51515	36.9515	OK	416.615	19.4822	5.79879	33.1656	OK	OK	-0.15775
ENSG0000000	-	-	ENSG0000000604	'YBX2	-	17:7281666-7294615	-	-	IGV_LINK	26.1825	1.3786	0	4.52161	OK	25.7806	0.643163	0	2.40204	OK	OK	-1.09994
ENSG0000000	-	-	ENSG0000000605	'KRT33A	-	17:41346091-41350812	-	-	IGV_LINK	0	0	0	0	OK	0	0	0	0	OK	NOTEST	0
ENSG0000000	-	-	ENSG0000000606	'MAP3K14	-	17:45190930-45317040	-	-	IGV_LINK	1524.4	23.1707	0	52.9317	OK	2044.92	29.2478	0	60.9859	OK	OK	0.336025
ENSG0000000	-	-	ENSG0000000607	'ABCC8	-	11:17392884-17476845	-	-	IGV_LINK	8.72748	0.483782	0	1.75111	OK	3.09367	0.271851	0	1.31942	OK	NOTEST	-0.83154
ENSG0000000	-	-	ENSG0000000611	'CACNG3	-	16:24255552-24362801	-	-	IGV_LINK	0	0	0	0	OK	0	0	0	0	OK	NOTEST	0
ENSG0000000	-	-	ENSG0000000611	'TMEM132A	-	11:60913165-60952530	-	-	IGV_LINK	876.627	24.1965	0	71.706	OK	1301.41	29.9877	0	75.9659	OK	OK	0.309572
ENSG0000000	-	-	ENSG0000000612	'AP2B1	-	17:35568108-35868891	-	-	IGV_LINK	3913.79	45.4066	0	103.575	OK	3710.35	42.4917	5.11855	79.8648	OK	OK	-0.09572
ENSG0000000	-	-	ENSG0000000612	'TAC1	-	7:97731907-97740472	-	-	IGV_LINK	0	0	0	0	OK	0	0	0	0	OK	NOTEST	0
ENSG0000000	-	-	ENSG0000000619	'ZNF263	-	16:3263742-3318852	-	-	IGV_LINK	742.806	18.9261	0	37.9366	OK	743.513	18.8909	0.187917	37.594	OK	OK	-0.00268
ENSG0000000	-	-	ENSG0000000621	'CX3CL1	-	16:57372457-57385048	-	-	IGV_LINK	4120.34	74.2698	30.0812	118.458	OK	2281.07	41.6678	16.7598	66.5758	OK	OK	-0.83384

ENSG0000000	-	-	ENSG0000000628	'SPATA20	-	17:50532542-50555852	-	-	IGV_LINK	2124.66	50.6697	17.1093	84.2301	OK	3285.48	79.7601	26.3262	133.194	OK	OK	0.654543
ENSG0000000	-	-	ENSG0000000628	'CACNA1G	-	17:50556206-50631940	-	-	IGV_LINK	0.969721	0.008492	0	0.15907	OK	2.06245	0.017794	0	0.16976	OK	NOTEST	1.06732
ENSG0000000	-	-	ENSG0000000632	'TNFRSF12A	-	16:3014711-3022383	-	-	IGV_LINK	214.308	13.4646	3.64512	23.2841	OK	595.017	38.8063	14.8545	62.758	OK	OK	1.52712
ENSG0000000	-	-	ENSG0000000637	'DLX6	-	7:96955140-97014066	-	-	IGV_LINK	6.78804	0.19475	0	0.621594	OK	2.06245	0.114016	0	0.601897	OK	NOTEST	-0.77239
ENSG0000000	-	-	ENSG0000000643	'MAP3K9	-	14:70722525-70809534	-	-	IGV_LINK	66.9107	0.375431	0	0.815086	OK	45.3739	0.266825	0	0.622459	OK	NOTEST	-0.49265
ENSG0000000	-	-	ENSG0000000645	'RALA	-	7:39623482-39708124	-	-	IGV_LINK	500.376	10.9873	3.55403	18.4205	OK	574.392	13.102	4.13306	22.0709	OK	OK	0.253954
ENSG0000000	-	-	ENSG0000000645	'BAIAP2L1	-	7:98252378-98401068	-	-	IGV_LINK	204.611	3.45169	0	10.3879	OK	393.928	7.91491	0	19.7384	OK	OK	1.19727
ENSG0000000	-	-	ENSG0000000645	'KDM7A	-	7:140084745-140177035	-	-	IGV_LINK	697.229	5.02352	0.411696	9.63535	OK	531.081	3.63557	1.38603	5.88511	OK	OK	-0.46652
ENSG0000000	-	-	ENSG0000000646	'ETV1	-	7:13891227-13991425	-	-	IGV_LINK	124.124	3.24039	0	7.73353	OK	23.7182	0.65579	0	2.03547	OK	OK	-2.30486
ENSG0000000	-	-	ENSG0000000653	'AGK	-	7:141529202-141702153	-	-	IGV_LINK	718.563	14.0094	0	33.1493	OK	621.829	13.338	0	28.3315	OK	OK	-0.07086
ENSG0000000	-	-	ENSG0000000653	'ALDH3B1	-	11:68008577-68030461	-	-	IGV_LINK	831.05	18.9589	6.69519	31.2226	OK	956.977	22.2438	8.60005	35.8875	OK	OK	0.230527
ENSG0000000	-	-	ENSG0000000655	'TTC22	-	1:54779711-54801267	-	-	IGV_LINK	201.702	4.05165	0.835858	7.26743	OK	309.367	5.96879	1.66977	10.2678	OK	OK	0.55893
ENSG0000000	-	-	ENSG0000000657	'PHTF2	-	7:77798791-77957503	-	-	IGV_LINK	332.614	6.10588	0	14.4013	OK	287.712	4.80458	0.319844	9.28931	OK	OK	-0.34579
ENSG0000000	-	-	ENSG0000000660	'CCL26	-	7:75769532-75789896	-	-	IGV_LINK	0	0	0	0	OK	1.03122	0.164938	0	0.665535	OK	NOTEST	inf
ENSG0000000	-	-	ENSG0000000660	'FARP2	-	2:241356242-241509730	-	-	IGV_LINK	514.922	8.68735	0	24.3937	OK	404.24	8.95193	0	29.3215	OK	OK	0.043283
ENSG0000000	-	-	ENSG0000000661	'USH1C	-	11:17493894-17544416	-	-	IGV_LINK	3197.17	97.7136	20.5811	174.846	OK	3656.72	117.52	11.1905	223.849	OK	OK	0.266273
ENSG0000000	-	-	ENSG0000000662	'GGCT	-	7:30496620-30634033	-	-	IGV_LINK	228.864	13.6448	0	37.5686	OK	225.838	13.8594	0	38.3491	OK	OK	0.022514
ENSG0000000	-	-	ENSG0000000663	'DBF4	-	7:87833567-87909541	-	-	IGV_LINK	100.851	3.42109	0	9.49292	OK	97.9664	3.35602	0	9.02771	OK	OK	-0.0277
ENSG0000000	-	-	ENSG0000000663	'TBXA2R	-	19:3594505-3606840	-	-	IGV_LINK	49.4557	1.23823	0	2.58508	OK	43.3114	1.05708	0	2.21815	OK	OK	-0.2282
ENSG0000000	-	-	ENSG0000000665	'IFRD1	-	7:112422967-112491062	-	-	IGV_LINK	629.349	20.2053	4.91846	35.4921	OK	408.365	13.8659	2.66971	25.0621	OK	OK	-0.54319
ENSG0000000	-	-	ENSG0000000665	'LGALS14	-	19:39704305-39709444	-	-	IGV_LINK	0.969721	0.090859	0	0.482549	OK	0	0	0	0	OK	NOTEST	'-inf
ENSG0000000	-	-	ENSG0000000669	'COX10	-	17:14069495-14208677	-	-	IGV_LINK	201.702	4.89168	0.744471	9.03889	OK	262.962	6.05119	1.52047	10.5819	OK	OK	0.306889
ENSG0000000	-	-	ENSG0000000670	'GTF2IRD1	-	7:74453789-74602604	-	-	IGV_LINK	448.981	9.17563	0.407461	17.9438	OK	501.175	9.60443	1.51234	17.6965	OK	OK	0.065893
ENSG0000000	-	-	ENSG0000000671	'PAF1	-	19:39385851-39391195	-	-	IGV_LINK	846.566	26.8316	6.04724	47.6159	OK	1136.41	35.2308	10.4689	59.9927	OK	OK	0.392906
ENSG0000000	-	-	ENSG0000000671	'VPS41	-	7:38722962-38932394	-	-	IGV_LINK	1124.88	23.2823	1.27226	45.2924	OK	1123	24.5265	1.10228	47.9508	OK	OK	0.075107
ENSG0000000	-	-	ENSG0000000674	'ARHGAP44	-	17:12665889-12991643	-	-	IGV_LINK	167.762	2.75359	0	5.81053	OK	129.934	1.80271	0.140653	3.46477	OK	OK	-0.61114
ENSG0000000	-	-	ENSG0000000674	'ELAC2	-	17:12992390-13018187	-	-	IGV_LINK	962.932	22.5026	6.07507	38.9301	OK	1341.62	33.6555	8.35219	58.9587	OK	OK	0.58075
ENSG0000000	-	-	ENSG0000000674	'SCIN	-	7:12570124-12660179	-	-	IGV_LINK	273.329	5.34811	1.22028	9.47593	OK	227.901	4.5711	0.402665	8.73953	OK	OK	-0.22649
ENSG0000000	-	-	ENSG0000000675	'ARSD	-	X:2903969-2929351	-	-	IGV_LINK	1219.91	19.0146	0	39.2338	OK	1480.84	20.7517	0.95971	40.5437	OK	OK	0.126121
ENSG0000000	-	-	ENSG0000000675	'PNPLA4	-	X:7898246-7927739	-	-	IGV_LINK	229.824	8.7564	0	19.4574	OK	263.994	11.4224	0.220337	22.6245	OK	OK	0.383459
ENSG0000000	-	-	ENSG0000000678	'MYH13	-	17:10291819-10373130	-	-	IGV_LINK	0	0	0	0	OK	13.4059	0.131559	0	0.302139	OK	NOTEST	inf
ENSG0000000	-	-	ENSG0000000683	'ADIPOR2	-	12:1688573-1788678	-	-	IGV_LINK	1276.15	19.7564	6.06035	33.4524	OK	1311.72	19.5645	7.8562	31.2728	OK	OK	-0.01408
ENSG0000000	-	-	ENSG0000000683	'CDKL3	-	5:134114710-134371047	-	-	IGV_LINK	70.3846	4.89113	0	39.9151	OK	49.6109	4.37115	0	45.2014	OK	OK	-0.16216
ENSG0000000	-	-	ENSG0000000700	'UPP2	-	2:157876701-158136154	-	-	IGV_LINK	7.75776	0.463173	0	1.32098	OK	12.3747	0.472397	0	1.34891	OK	NOTEST	0.028449
ENSG0000000	-	-	ENSG0000000703	'PRSS21	-	16:2817179-2826304	-	-	IGV_LINK	6.78804	0.566805	0	1.56224	OK	5.15612	0.635887	0	1.84505	OK	OK	0.165919
ENSG0000000	-	-	ENSG0000000704	'MARK4	-	19:45071499-45305283	-	-	IGV_LINK	574.075	10.1261	0	25.0634	OK	579.548	14.3401	0	40.9588	OK	OK	0.501971
ENSG0000000	-	-	ENSG0000000706	'PROM1	-	4:15960242-16084378	-	-	IGV_LINK	3422.14	62.461	13.6985	111.224	OK	1.03122	0.099118	0	1.16523	OK	OK	-9.29959
ENSG0000000	-	-	ENSG0000000708	'CCDC124	-	19:17933015-17943991	-	-	IGV_LINK	727.29	52.8885	20.2038	85.5732	OK	559.955	40.8568	16.0596	65.654	OK	OK	-0.37238
ENSG0000000	-	-	ENSG0000000712	'CEACAM21	-	19:41545191-41586844	-	-	IGV_LINK	125.094	9.24241	0	19.4619	OK	77.3419	6.42703	0	14.1375	OK	OK	-0.52412
ENSG0000000	-	-	ENSG0000000716	'PAFAH1B1	-	17:2593209-2685615	-	-	IGV_LINK	1504.75	34.2365	0	68.5792	OK	1460.71	29.9914	0.777192	59.2056	OK	OK	-0.19099
ENSG0000000	-	-	ENSG0000000717	'NOS2	-	17:27756765-27894752	-	-	IGV_LINK	40.7283	0.573317	0	1.69608	OK	12.3747	0.186455	0	0.83789	OK	OK	-1.6205
ENSG0000000	-	-	ENSG0000000717	'DNAH9	-	17:11598430-11997510	-	-	IGV_LINK	121.215	2.50877	0	6.95068	OK	138.184	3.80307	0	10.2389	OK	OK	0.600187
ENSG0000000	-	-	ENSG0000000720	'KIAA0100	-	17:28455751-28645454	-	-	IGV_LINK	2891.71	33.9546	0	108.524	OK	3800.06	42.4136	0	137.86	OK	OK	0.320923
ENSG0000000	-	-	ENSG0000000721	'SLC13A2	-	17:28455751-28645454	-	-	IGV_LINK	4.8486	0.107641	0	2.67745	OK	3.09367	0.08123	0	3.02915	OK	NOTEST	-0.40615
ENSG0000000	-	-	ENSG0000000723	'GAS7	-	17:9910608-10198551	-	-	IGV_LINK	562.438	5.27397	1.40858	9.13937	OK	445.489	4.20894	1.17411	7.24376	OK	OK	-0.32543
ENSG0000000	-	-	ENSG0000000725	'TRAPPC6A	-	19:45071499-45305283	-	-	IGV_LINK	299.644	30.4637	0	86.0011	OK	269.15	26.951	0	76.1021	OK	OK	-0.17675
ENSG0000000	-	-	ENSG0000000726	'MATK	-	19:3777969-3802129	-	-	IGV_LINK	91.1537	2.68803	0.046694	5.32937	OK	138.184	5.09397	0.200927	9.98702	OK	OK	0.922242
ENSG0000000	-	-	ENSG0000000730	'CEACAM7	-	19:41673306-41706976	-	-	IGV_LINK	0	0	0	0	OK	0	0	0	0	OK	NOTEST	0
ENSG0000000	-	-	ENSG0000000731	'CD79B	-	17:63928739-63932354	-	-	IGV_LINK	109.578	5.702	0	11.4443	OK	109.31	5.66898	0.811008	10.527	OK	OK	-0.00838
ENSG0000000	-	-	ENSG0000000731	'SCN4A	-	17:63938553-63972918	-	-	IGV_LINK	30.0613	0.221968	0	0.640247	OK	36.0929	0.266536	0	0.662311	OK	NOTEST	0.263979
ENSG0000000	-	-	ENSG0000000734	'ST7L	-	1:112466540-112671619	-	-	IGV_LINK	303.523	10.9789	0	35.7167	OK	313.492	9.53339	0	34.6324	OK	OK	-0.20368
ENSG0000000	-	-	ENSG0000000735	'TKTL1	-	X:154257537-154330350	-	-	IGV_LINK	5.81832	0.140605	0	0.767932	OK	1.03122	0.024292	0	0.556244	OK	NOTEST	-2.53308
ENSG0000000	-	-	ENSG0000000737	'PAX6	-	11:31509699-32105755	-	-	IGV_LINK	35.8797	0.323899	0	2.4065	OK	39.1865	0.415732	0	4.3678	OK	NOTEST	0.360108
ENSG0000000	-	-	ENSG0000000737	'RPUSD1	-	16:784973-800737	-	-	IGV_LINK	309.341	10.8399	0	22.0067	OK	323.805	11.464	0.621662	22.3063	OK	OK	0.08075
ENSG0000000	-	-	ENSG0000000738	'RHBDF1	-	16:58058-76355	-	-	IGV_LINK	270.552	8.9065	1.08172	16.7313	OK	197.995	5.76795	0.438657	11.0973	OK	OK	-0.6268
ENSG0000000	-	-	ENSG0000000739	'LUC7L	-	16:188968-229463	-	-	IGV_LINK	1174.33	47.0693	15.4796	78.659	OK	819.824	31.398	10.9704	51.8255	OK	OK	-0.58412
ENSG0000000	-	-	ENSG0000000740	'CACNA2D2	-	3:50347329-50504244	-	-	IGV_LINK	24.243	0.266245	0	1.38156	OK	25.7806	0.288867	0	1.50148	OK	NOTEST	0.117648
ENSG0000000	-	-	ENSG0000000751	'BAIAP3	-	16:1333600-1351911	-	-	IGV_LINK	79.5171	1.67041	0	8.60562	OK	56.7174	1.49968	0	5.80424	OK	OK	-0.15554
ENSG0000000	-	-	ENSG0000000752	'TSR3	-	16:1333600-1351911	-	-	IGV_LINK	409.222	22.8891	6.36786	39.4104	OK	420.74	24.9842	7.21875	42.7497	OK	OK	0.126356
ENSG0000000	-	-	ENSG0000000754	'PIGQ	-	16:566994-584136	-	-	IGV_LINK	1136.95	32.668	9.00417	56.3319	OK	1105.87	31.1253	0	239.561	OK	OK	-0.06979
ENSG0000000	-	-	ENSG0000000754	'CRAMP1	-	16:1612324-1702280	-	-	IGV_LINK	733.236	7.6059	0	26.7013	OK	798.386	9.37196	0	34.0761	OK	OK	0.301233
ENSG0000000	-	-	ENSG0000000786	'TEAD3	-	6:35473596-35497076	-	-	IGV_LINK	132.852	2.84427	0.455377	5.23317	OK	139.215	2.92017	0.519135	5.32121	OK	OK	0.037996
ENSG0000000	-	-	ENSG0000000790	'SELE	-	1:169662006-169894267	-	-	IGV_LINK	28.1219	0.833869	0	5.39504	OK	59.811	0.915117	0	3.28265	OK	OK	0.134135
ENSG0000000	-	-	ENSG0000000792	'DNAJC11	-	1:6624865-6701924	-	-	IGV_LINK	1007.54	21.9657	3.98198	39.9494	OK	1035.35	22.5899	3.96827	41.2114	OK	OK	0.040422
ENSG0000000	-	-	ENSG0000000793	'FMO3	-	1:171090876-171117819	-	-	IGV_LINK	9.69721	0.290752	0	0.858992	OK	30.9367	0.921946	0	1.99986	OK	OK	1.66489
ENSG0000000	-	-	ENSG0000000794	'MYLIP	-	6:16129124-16148248	-	-	IGV_LINK	797.11	21.1229	7.55301	34.6927	OK	832.199	22.0007	7.73642	36.265	OK	OK	0.058745
ENSG0000000	-	-	ENSG0000000795	'NOX1	-	X:100843323-100874345	-	-	IGV_LINK	0.969721	0.111877	0	0.490663	OK	4.1249	0.105026	0	0.433446	OK	NOTEST	-0.09116
ENSG0000000	-	-	ENSG0000000796	'E2F2	-	1:23506429-23531220	-	-	IGV_LINK	77.5776	0.871164	0.00928	1.73305	OK	57.7486	0.870942	0	2.44002	OK	OK	-0.00037
ENSG0000000	-	-	ENSG0000000801	'PSMB1	-	6:170535116-170553341	-	-	IGV_LINK	2103.32	148.538	64.6555	232.42	OK	1345.75	96.5003	42.0206	150.98	OK	OK	-0.62223
ENSG0000000	-	-	ENSG0000000805	'SYN1	-	X:47561099-47630305	-	-	IGV_LINK	6.78804	0.40379	0	8.54401	OK	6.18735	0.116295	0	2.20371	OK	NOTEST	-1.79582
ENSG0000000	-	-	ENSG0000000808	'JARID2	-	6:15246295-15522040	-	-	IGV_LINK	376.252	3.79828	1.3325	6.26407	OK	400.115	4.04002	1.44118	6.63885	OK	OK	0.089015
ENSG0000000	-	-	ENSG0000000808	'CDKL5	-	X:18425582-18672109	-	-	IGV_LINK	381.1	2.5585	0	6.76581	OK	209.339	1.43704	0	4.31759	OK	OK	-0.8322
ENSG0000000	-	-	ENSG0000000811	'CAMK1G	-	1:209583716-209613938	-	-	IGV_LINK	118.306	3.33825	0	6.8527	OK	14.4371	0.358997	0	0.888145	OK	OK	-3.21705
ENSG0000000	-	-	ENSG0000000812	'CDK11A	-	1:1702729-1745992	-	-	IGV_LINK	479.567	32.0574	0	70.8044	OK	571.034	33.1587	1.0994	65.2179	OK	OK	0.048729
ENSG0000000	-	-	ENSG0000000813	'NADK	-	1:1751231-1780457	-	-	IGV_LINK	1284.88	37.1995	4.829	69.5701	OK	2083.07	60.7165	12.9761	108.457	OK	OK	0.706803
ENSG0000000	-	-	ENSG0000000819	'TFAP2B	-	6:50818722-50847613	-	-	IGV_LINK	0	0	0	0	OK	2.06245	0.175754	0	0.561432	OK	NOTEST	inf
ENSG0000000	-	-	ENSG0000000819	'TFAP2D	-	6:50713827-50772988	-	-	IGV_LINK	0	0	0	0	OK	0	0	0	0	OK	NOTEST	0
ENSG0000000	-	-	ENSG0000000822	'DLEC1	-	3:38039204-38137242	-	-	IGV_LINK	22.3036	0.588282	0	5.65886	OK	30.9367	0.389787	0	2.03538	OK	OK	-0.59382
ENSG0000000	-	-	ENSG0000000825	'CYTH3	-	7:6161775-6272644	-	-	IGV_LINK	402.434	5.78956	0.67619	10.9029	OK	269.15	5.20285	0	11.4857	OK	OK	-0.15415
ENSG0000000	-	-	ENSG0000000827	'ADAM22	-	7:87934142-88202889	-	-	IGV_LINK	341.342	2.38188	0.196973	4.5668	OK	341.335	2.57585	0.119313	5.03239	OK	OK	0.112947
ENSG0000000	-	-	ENSG0000000828	'SYPL1	-	7:106090502-106112576	-	-	IGV_LINK	1413.85	49.5536	16.0158	83.0913	OK	1725.24	62.5105	19.4354	105.586	OK	OK	0.33511
ENSG0000000	-	-	ENSG0000000828	'CYB561	-	17:63432303-63446378	-	-	IGV_LINK	898.931	21.5613	5.31201	37.8106	OK	902.322	20.4707	5.73118	35.2102	OK	OK	-0.07488
ENSG0000000	-	-	ENSG0000000829	'SPAG9	-	17:50962173-51120865	-	-	IGV_LINK	2934.37	43.4255	3.13654	83.7144	OK	2011.92	28.4029	2.14956	54.6563	OK	OK	-0.6125
ENSG0000000	-	-	ENSG0000000830	'CELSR3	-	3:48636468-48662915	-	-	IGV_LINK	68.8502	1.34918	0	4.10261	OK	84.5604	1.11837	0	2.95967	OK	OK	-0.27068
ENSG0000000	-	-	ENSG0000000831	'AASS	-	7:122075646-122144280	-	-	IGV_LINK	284.128	5.60091	1.68223	9.51959	OK	111.372	2.5035	0.033236	4.97376	OK	OK	-1.16171
ENSG0000000	-	-	ENSG0000000832	'PLEKHG6	-	12:6310435-6328506	-	-	IGV_LINK	37.8191	1.55744	0	5.07941	OK	160.871	3.75835	0.227474	7.28923	OK	OK	1.27092
ENSG0000000	-	-	ENSG0000000832	'SS18L2	-	3:42547968-42665854	-	-	IGV_LINK	142.549	13.2026	0	51.6086	OK	128.903	11.8659	0	42.2408	OK	OK	-0.15399
ENSG0000000	-	-	ENSG0000000838	'MPND	-	19:4343526-4360086	-	-	IGV_LINK	300.613	13.6234	1.81043	25.4363	OK	334.117	14.4455	2.3464	26.5446	OK	OK	0.084537
ENSG0000000	-	-	ENSG0000000839	'MGST1	-	12:16347141-16610594	-	-	IGV_LINK	1313	114.055	39.4926	188.618	OK	6416.28	563.504	162.887	964.121	OK	OK	2.30469
ENSG0000000	-	-	ENSG0000000840	'CRY1	-	12:106991363-107123314	-	-	IGV_LINK	238.551	5.04305	0.295309	9.79079	OK	221.713	4.40796	0.345214	8.4707	OK	OK	-0.19419
ENSG0000000	-	-	ENSG0000000843	'PGLYRP1	-	19:45995460-46023065	-	-	IGV_LINK	0	0	0	0	OK	0	0	0	0	OK	NOTEST	0
ENSG0000000	-	-	ENSG0000000844	'NFIX	-	19:12995607-13098796	-	-	IGV_LINK	712.745	11.8105	3.66013	19.9608	OK	832.199	16.0747	4.96413	27.1854	OK	OK	0.44473
ENSG0000000	-	-	ENSG0000000851	'ST3GAL1	-	8:133454847-133571940	-	-	IGV_LINK	1172.39	11.6245	0.959983	22.289	OK	1864.45	19.9738	0	42.5903	OK	OK	0.780938
ENSG0000000	-	-	ENSG0000000851	'MMP25	-	16:3046680-3060726	-	-	IGV_LINK	98.9115	2.51314	0	7.36295	OK	200.058	5.42613	0	13.8135	OK	OK	1.11043
ENSG0000000	-	-	ENSG0000000851	'IL32	-	16:3065296-3087100	-	-	IGV_LINK	3547.24	263.468	105.671	421.266	OK	4802.41	356.451	140.253	572.648	OK	OK	0.436074
ENSG0000000	-	-	ENSG0000000871	'PKD1	-	16:2039814-2135898	-	-	IGV_LINK	1441.97	32.2039	0	89.6096	OK	1259.13	29.178	0	81.9289	OK	OK	-0.14236
ENSG0000000	-	-	ENSG0000000873	'MAPK8IP2	-	22:50568860-50613981	-	-	IGV_LINK	13.5761	0.224717	0	2.2695	OK	14.4371	0.199267	0	1.75372	OK	NOTEST	-0.17341
ENSG0000000	-	-	ENSG0000000883	'MED24	-	17:40019096-40100725	-	-	IGV_LINK	1184.03	43.2638	0	98.2719	OK	1277.69	40.8521	0	90.64	OK	OK	-0.08275
ENSG0000000	-	-	ENSG0000000885	'RHOBTB2	-	8:22687658-23083619	-	-	IGV_LINK	327.766	6.99103	0	37.9192	OK	255.744	4.90868	0	34.2069	OK	OK	-0.51017
ENSG0000000	-	-	ENSG0000000886	'HEATR5B	-	2:36968382-37084342	-	-	IGV_LINK	1228.64	10.5795	3.8985	17.2605	OK	859.01	7.65687	0.739229	14.5745	OK	OK	-0.46644
ENSG0000000	-	-	ENSG0000000895	'SEC62	-	3:169939352-169998373	-	-	IGV_LINK	2040.29	58.1578	15.1011	101.214	OK	2226.41	65.7991	20.6255	110.973	OK	OK	0.178097
ENSG0000000	-	-	ENSG0000000898	'RPS20	-	8:56067294-56074581	-	-	IGV_LINK	13363.7	1654.24	495.264	2813.22	OK	13581.2	1689.17	502.25	2876.09	OK	OK	0.030147
ENSG0000000	-	-	ENSG0000000930	'CSDE1	-	1:114716912-114758676	-	-	IGV_LINK	8687.73	172.583	29.0516	316.115	OK	8138.43	161.046	28.8005	293.292	OK	OK	-0.09982
ENSG0000000	-	-	ENSG0000000933	'UBE3C	-	7:157138912-157269372	-	-	IGV_LINK	1157.85	18.9808	0	39.9804	OK	1298.31	18.9977	0	38.3821	OK	OK	0.001281
ENSG0000000	-	-	ENSG0000000941	'REV3L	-	6:111299027-111606278	-	-	IGV_LINK	567.286	7.15427	0	20.2268	OK	290.805	6.1605	0	16.9863	OK	OK	-0.21576
ENSG0000000	-	-	ENSG0000000969	'TENM1	-	X:123959232-124963817	-	-	IGV_LINK	1253.85	5.55643	0.150287	10.9626	OK	463.02	2.05437	0	4.36616	OK	OK	-1.43546
ENSG0000000	-	-	ENSG0000000970	'PAX7	-	1:18631005-18748866	-	-	IGV_LINK	0	0	0	0	OK	0	0	0	0	OK	NOTEST	0
ENSG0000000	-	-	ENSG0000000972	'MASP2	-	1:11012343-11047233	-	-	IGV_LINK	103.76	2.57997	0	10.4989	OK	59.811	1.48794	0	7.21024	OK	OK	-0.79404
ENSG0000000	-	-	ENSG0000000976	'IYD	-	6:150368891-150405969	-	-	IGV_LINK	3.87888	0.106125	0	0.644261	OK	1.03122	0.006783	0	0.455635	OK	NOTEST	-3.96779
ENSG0000000	-	-	ENSG0000000978	'FAM76A	-	1:27725978-27763122	-	-	IGV_LINK	202.672	6.20096	0.40288	11.999	OK	166.027	6.63741	0	13.9252	OK	OK	0.098129
ENSG0000000	-	-	ENSG0000000979	'TRAF3IP3	-	1:209756031-209784559	-	-	IGV_LINK	1125.85	37.4594	6.49711	68.4217	OK	728.045	27.0888	2.25077	51.9268	OK	OK	-0.46763
ENSG0000000	-	-	ENSG0000000983	'POMT2	-	14:77274955-77331597	-	-	IGV_LINK	361.706	12.016	0	26.5464	OK	422.802	12.6301	0	29.2482	OK	OK	0.071904
ENSG0000000	-	-	ENSG0000000984	'VTA1	-	6:142147161-142224689	-	-	IGV_LINK	464.496	14.9784	3.07026	26.8865	OK	365.054	11.2924	2.02529	20.5596	OK	OK	-0.40753
ENSG0000000	-	-	ENSG0000000995	'MLXIPL	-	7:73593193-73624543	-	-	IGV_LINK	416.01	10.2923	1.36184	19.2227	OK	401.147	10.2624	1.04674	19.478	OK	OK	-0.0042
ENSG0000000	-	-	ENSG0000000995	'BAZ1B	-	7:73440397-73522278	-	-	IGV_LINK	1641.74	16.4763	6.61077	26.3418	OK	1649.96	16.3706	6.69224	26.0489	OK	OK	-0.00929
ENSG0000001	-	-	ENSG0000001001	'RANBP9	-	6:13615326-13711564	-	-	IGV_LINK	533.346	11.9531	0	24.9187	OK	446.52	11.0121	0	23.1295	OK	OK	-0.1183
ENSG0000001	-	-	ENSG0000001003	'ETV7	-	6:36354090-36491143	-	-	IGV_LINK	568.256	23.1265	0	46.6898	OK	82.498	3.35704	0	10.5619	OK	OK	-2.78429
ENSG0000001	-	-	ENSG0000001007	'SPRTN	-	1:231332752-231355023	-	-	IGV_LINK	192.005	4.46644	0	11.2142	OK	156.746	2.68993	0	6.01127	OK	OK	-0.73156
ENSG0000001	-	-	ENSG0000001016	'METTL13	-	1:171781663-171814023	-	-	IGV_LINK	540.134	14.4385	0.500761	28.3762	OK	665.14	18.749	3.5417	33.9564	OK	OK	0.376897
ENSG0000001	-	-	ENSG0000001021	'DYRK4	-	12:4562203-4851927	-	-	IGV_LINK	304.013	28.9205	0	67.9949	OK	317.617	27.4618	0	63.1443	OK	OK	-0.07467
ENSG0000001	-	-	ENSG0000001024	'ZNF207	-	17:32266175-32381886	-	-	IGV_LINK	3091.47	77.1269	13.0173	141.236	OK	2839.99	77.32	12.0849	142.555	OK	OK	0.003608
ENSG0000001	-	-	ENSG0000001025	'UQCRC1	-	3:48599001-48610976	-	-	IGV_LINK	1547.67	71.0471	23.4692	118.625	OK	1489.09	74.2687	21.7165	126.821	OK	OK	0.063978
ENSG0000001	-	-	ENSG0000001027	'STARD3NL	-	7:38178221-38230671	-	-	IGV_LINK	842.687	34.6772	11.8793	57.4751	OK	796.106	31.545	12.4825	50.6075	OK	OK	-0.13658
ENSG0000001	-	-	ENSG0000001027	'CD9	-	12:6199714-6238271	-	-	IGV_LINK	3337.78	209.968	80.1225	339.813	OK	2725.53	176.015	66.7499	285.28	OK	OK	-0.25447
ENSG0000001	-	-	ENSG0000001028	'HHATL	-	3:42692662-43060211	-	-	IGV_LINK	0.969721	0.103214	0	1.75028	OK	2.06245	0.219584	0	3.16082	OK	NOTEST	1.08913
ENSG0000001	-	-	ENSG0000001029	'NCAPD2	-	12:6491885-6531955	-	-	IGV_LINK	1003.66	17.4394	0	47.4311	OK	1025.04	16.7024	0	47.1266	OK	OK	-0.0623
ENSG0000001	-	-	ENSG0000001029	'IFFO1	-	12:6538374-6556083	-	-	IGV_LINK	1039.54	25.3233	8.82962	41.8169	OK	1094.13	26.1539	10.0557	42.2521	OK	OK	0.04656
ENSG0000001	-	-	ENSG0000001031	'GIPR	-	19:45668243-45683724	-	-	IGV_LINK	64.9713	2.13749	0	5.3294	OK	56.7174	4.29281	0	30.4676	OK	OK	1.006
ENSG0000001	-	-	ENSG0000001031	'PHF7	-	3:52410656-52423641	-	-	IGV_LINK	99.8812	5.64654	0	12.3865	OK	88.6853	4.80102	0	10.092	OK	OK	-0.23403
ENSG0000001	-	-	ENSG0000001031	'SEMA3G	-	3:52433052-52445085	-	-	IGV_LINK	64.9713	1.10949	0	2.95209	OK	402.178	4.96665	1.3245	8.6088	OK	OK	2.16237
ENSG0000001	-	-	ENSG0000001032	'NISCH	-	3:52455117-52493071	-	-	IGV_LINK	1755.19	29.9925	3.88012	56.1048	OK	1859.3	33.3255	3.9153	62.7357	OK	OK	0.152026
ENSG0000001	-	-	ENSG0000001032	'STAB1	-	3:52495337-52770949	-	-	IGV_LINK	2649.28	43.3116	0	114.452	OK	12376.8	133.075	0	331.14	OK	OK	1.61942

ENSG0000001	-	-	ENSG0000001036	'FUZ	-	19:49766967-49817376	-	-	IGV_LINK	542.074	28.7376	0	64.319	OK	510.456	26.1855	0	66.485	OK	OK	-0.13418
ENSG0000001	-	-	ENSG0000001037	'SLC6A13	-	12:220620-262873	-	-	IGV_LINK	1055.06	44.8276	9.78214	79.873	OK	475.395	21.108	2.31206	39.904	OK	OK	-1.08659
ENSG0000001	-	-	ENSG0000001040	'IDS	-	X:149476989-149550510	-	-	IGV_LINK	3590.86	43.0615	0	87.2243	OK	3448.42	45.6558	0	93.1081	OK	OK	0.084398
ENSG0000001	-	-	ENSG0000001043	'PRSS3	-	9:33750465-33920404	-	-	IGV_LINK	1.93944	0.137758	0	2.11761	OK	19.5933	1.70635	0	10.8627	OK	OK	3.6307
ENSG0000001	-	-	ENSG0000001053	'ZNF200	-	16:3188211-3236221	-	-	IGV_LINK	187.156	4.98554	0.695801	9.27527	OK	211.401	6.31108	0.342596	12.2796	OK	OK	0.340139
ENSG0000001	-	-	ENSG0000001061	'CD4	-	12:6786857-6820808	-	-	IGV_LINK	3256.32	67.1365	23.7461	110.527	OK	3697.97	74.7938	29.077	120.511	OK	OK	0.155822
ENSG0000001	-	-	ENSG0000001062	'LRRC23	-	12:6867118-6923698	-	-	IGV_LINK	355.121	17.9525	0	133.138	OK	458.374	22.1862	0	159.561	OK	OK	0.305478
ENSG0000001	-	-	ENSG0000001067	'BTK	-	X:101349446-101390796	-	-	IGV_LINK	716.623	24.9815	1.3381	48.625	OK	552.737	18.2871	0	36.5835	OK	OK	-0.45004
ENSG0000001	-	-	ENSG0000001070	'HFE	-	6:26087280-26098343	-	-	IGV_LINK	336.493	9.23798	1.2596	17.2164	OK	350.616	10.7581	1.68748	19.8288	OK	OK	0.219777
ENSG0000001	-	-	ENSG0000001080	'SCMH1	-	1:41014589-41242154	-	-	IGV_LINK	1178.21	29.7135	2.01108	57.4159	OK	1295.22	33.0471	2.90155	63.1926	OK	OK	0.153403
ENSG0000001	-	-	ENSG0000001081	'FYN	-	6:111660331-111873452	-	-	IGV_LINK	973.599	32.5408	7.6899	57.3917	OK	829.105	34.7177	8.10029	61.3351	OK	OK	0.09342
ENSG0000001	-	-	ENSG0000001081	'HIVEP2	-	6:142748442-142956698	-	-	IGV_LINK	1046.33	6.14399	2.29167	9.99631	OK	490.863	3.75799	0	14.1472	OK	OK	-0.70921
ENSG0000001	-	-	ENSG0000001093	'FMO1	-	1:171247579-171285978	-	-	IGV_LINK	482.921	18.5125	1.24301	35.782	OK	1115.79	43.5076	7.21565	79.7995	OK	OK	1.23277
ENSG0000001	-	-	ENSG0000001100	'ELOA	-	1:23743154-23778287	-	-	IGV_LINK	675.895	11.5601	3.00058	20.1196	OK	806.418	14.0924	3.95708	24.2278	OK	OK	0.285764
ENSG0000001	-	-	ENSG0000001100	'LYPLA2	-	1:23790969-23795539	-	-	IGV_LINK	1077.36	42.8094	17.1655	68.4533	OK	1197.25	49.8617	19.1291	80.5943	OK	OK	0.220004
ENSG0000001	-	-	ENSG0000001102	'CLCN6	-	1:11761786-11848345	-	-	IGV_LINK	984.812	13.3224	0	29.5632	OK	812.565	11.0109	0	25.3787	OK	OK	-0.27492
ENSG0000001	-	-	ENSG0000001102	'MRC2	-	17:62626030-62693597	-	-	IGV_LINK	1112.27	16.4138	5.93341	26.8942	OK	836.323	12.2428	4.47372	20.0119	OK	OK	-0.42298
ENSG0000001	-	-	ENSG0000001105	'NME1-NME2	-	17:51153535-51171747	-	-	IGV_LINK	221.216	14.6931	0	59.941	OK	78.6187	5.22893	0	32.1011	OK	OK	-1.49055
ENSG0000001	-	-	ENSG0000001108	'SLC6A7	-	5:150189956-150290291	-	-	IGV_LINK	0.969721	0.024197	0	0.237775	OK	3.09367	0.0489	0	0.261053	OK	NOTEST	1.01501
ENSG0000001	-	-	ENSG0000001110	'TSPAN9	-	12:3077354-3286564	-	-	IGV_LINK	2312.78	36.6528	9.10421	64.2013	OK	3512.35	65.4042	3.57879	127.23	OK	OK	0.835462
ENSG0000001	-	-	ENSG0000001111	'BTBD7	-	14:93237549-93333092	-	-	IGV_LINK	624.5	8.10628	0	19.3436	OK	463.02	6.69994	0	18.6851	OK	OK	-0.27489
ENSG0000001	-	-	ENSG0000001113	'APBA3	-	19:3750818-3761699	-	-	IGV_LINK	645.74	25.3443	5.65753	45.0311	OK	658.868	26.2187	6.12136	46.3161	OK	OK	0.048934
ENSG0000001	-	-	ENSG0000001114	'MKS1	-	17:58205436-58268518	-	-	IGV_LINK	417.95	18.4106	3.95035	32.8708	OK	418.677	18.2155	3.86974	32.5612	OK	OK	-0.01537
ENSG0000001	-	-	ENSG0000001119	'ABHD5	-	3:43286511-43734371	-	-	IGV_LINK	88.2446	3.66114	0	13.1479	OK	149.528	7.79995	0	24.8631	OK	OK	1.09117
ENSG0000001	-	-	ENSG0000001120	'ANOS1	-	X:8528873-8732186	-	-	IGV_LINK	145.458	2.09876	0	5.14774	OK	135.09	1.67127	0	4.01205	OK	OK	-0.32859
ENSG0000001	-	-	ENSG0000001124	'AKAP8L	-	19:15353384-15419141	-	-	IGV_LINK	1512.76	64.4997	1.64431	127.355	OK	1156	50.0104	0	105.981	OK	OK	-0.36706
ENSG0000001	-	-	ENSG0000001125	'MBTD1	-	17:51177424-51260163	-	-	IGV_LINK	634.197	8.2346	0.811553	15.6577	OK	473.332	5.72591	0.808564	10.6433	OK	OK	-0.52419
ENSG0000001	-	-	ENSG0000001126	'UTP18	-	17:51260527-51297936	-	-	IGV_LINK	538.195	18.0168	6.8308	29.2029	OK	508.394	17.2597	6.44724	28.0721	OK	OK	-0.06194
ENSG0000001	-	-	ENSG0000001127	'RNF216	-	7:5620046-5781739	-	-	IGV_LINK	760.261	8.84681	3.06777	14.6259	OK	915.728	11.6196	4.47332	18.7659	OK	OK	0.393331
ENSG0000001	-	-	ENSG0000001129	'TTC19	-	17:15976559-16353656	-	-	IGV_LINK	886.73	21.0346	0	51.5978	OK	851.837	19.8174	0	45.9198	OK	OK	-0.08599
ENSG0000001	-	-	ENSG0000001130	'PTBP1	-	19:797074-812327	-	-	IGV_LINK	3646.15	80.5354	16.2783	144.792	OK	3529.88	84.3008	8.07933	160.522	OK	OK	0.065924
ENSG0000001	-	-	ENSG0000001133	'DPF1	-	19:38211005-38229714	-	-	IGV_LINK	0.969721	0.147903	0	0.852883	OK	1.03122	0.041848	0	0.624977	OK	NOTEST	-1.82143
ENSG0000001	-	-	ENSG0000001134	'SYT7	-	11:61515312-61581148	-	-	IGV_LINK	5.81832	0.088873	0	0.434974	OK	0	0	0	0	OK	NOTEST	'-inf
ENSG0000001	-	-	ENSG0000001137	'LARS2	-	3:45388505-45549421	-	-	IGV_LINK	250.188	3.73233	0.18735	7.2773	OK	293.899	4.45761	0.312201	8.60302	OK	OK	0.256196
ENSG0000001	-	-	ENSG0000001140	'PIK3C2A	-	11:17074388-17207983	-	-	IGV_LINK	957.114	9.39041	0	45.0011	OK	819.824	7.94156	0	73.3821	OK	OK	-0.24177
ENSG0000001	-	-	ENSG0000001142	'PLAUR	-	19:43646094-43670547	-	-	IGV_LINK	212.369	10.4354	2.30081	18.5699	OK	556.861	29.5296	9.20532	49.8539	OK	OK	1.50068
ENSG0000001	-	-	ENSG0000001142	'ANLN	-	7:36324220-36453791	-	-	IGV_LINK	127.033	2.2865	0	6.37724	OK	203.151	3.03417	0	6.89284	OK	OK	0.408161
ENSG0000001	-	-	ENSG0000001145	'WIZ	-	19:15419979-15449951	-	-	IGV_LINK	940.629	16.287	3.64358	28.9304	OK	1031.22	18.251	3.09895	33.4031	OK	OK	0.164258
ENSG0000001	-	-	ENSG0000001145	'RABGAP1	-	9:122940832-123104866	-	-	IGV_LINK	1109.97	15.6639	0	31.3556	OK	927.071	12.6524	0.058747	25.2461	OK	OK	-0.30804
ENSG0000001	-	-	ENSG0000001146	'DCN	-	12:91140483-91183123	-	-	IGV_LINK	20.3641	0.873906	0	3.05266	OK	62.9047	2.98325	0	6.65306	OK	OK	1.77134
ENSG0000001	-	-	ENSG0000001147	'QPCTL	-	19:45687453-45703989	-	-	IGV_LINK	173.58	5.21331	0	17.796	OK	234.088	6.46203	0	20.2762	OK	OK	0.309789
ENSG0000001	-	-	ENSG0000001148	'PPP5C	-	19:46346993-46392981	-	-	IGV_LINK	974.569	30.8828	7.08938	54.6763	OK	952.852	29.8873	9.38547	50.3892	OK	OK	-0.04727
ENSG0000001	-	-	ENSG0000001152	'CEP68	-	2:65056365-65130106	-	-	IGV_LINK	447.041	4.85613	0	11.3418	OK	414.552	4.57736	0	12.6232	OK	OK	-0.08529
ENSG0000001	-	-	ENSG0000001156	'MAP4K3	-	2:39249265-39717963	-	-	IGV_LINK	350.069	5.88313	0	13.139	OK	325.867	5.11511	0	10.9713	OK	OK	-0.20182
ENSG0000001	-	-	ENSG0000001159	'ZBTB32	-	19:35704526-35717038	-	-	IGV_LINK	18.4247	0.737941	0	1.79413	OK	13.4059	0.484502	0	1.24415	OK	OK	-0.607
ENSG0000001	-	-	ENSG0000001160	'TYROBP	-	19:35904400-35908295	-	-	IGV_LINK	2690	388.668	160.184	617.152	OK	2664.69	386.709	164.833	608.585	OK	OK	-0.00729
ENSG0000001	-	-	ENSG0000001163	'TMEM159	-	16:20933110-21180616	-	-	IGV_LINK	743.776	31.5079	10.0635	52.9523	OK	436.208	20.9274	5.5596	36.2951	OK	OK	-0.59032
ENSG0000001	-	-	ENSG0000001167	'GABRA3	-	X:152138882-152451358	-	-	IGV_LINK	0	0	0	0	OK	0	0	0	0	OK	NOTEST	0
ENSG0000001	-	-	ENSG0000001204	'BRCA1	-	17:43044294-43211689	-	-	IGV_LINK	179.398	4.09498	0	24.3665	OK	203.151	4.71103	0	27.4255	OK	OK	0.202187
ENSG0000001	-	-	ENSG0000001206	'ERCC1	-	19:45379633-45478828	-	-	IGV_LINK	1508.89	65.2807	0	133.596	OK	1302.44	59.0657	0.248873	117.883	OK	OK	-0.14433
ENSG0000001	-	-	ENSG0000001212	'CD22	-	19:35319260-35347355	-	-	IGV_LINK	122.185	4.32758	0	9.91813	OK	175.308	5.38003	0	11.5707	OK	OK	0.314052
ENSG0000001	-	-	ENSG0000001217	'SEMA3B	-	3:50267557-50277546	-	-	IGV_LINK	132.852	3.03398	0.266323	5.80164	OK	937.384	22.4964	8.31286	36.6799	OK	OK	2.89041
ENSG0000001	-	-	ENSG0000001217	'MBTPS2	-	X:21839635-21885424	-	-	IGV_LINK	319.987	4.49293	1.22849	7.75737	OK	509.688	7.52005	2.40732	12.6328	OK	OK	0.743085
ENSG0000001	-	-	ENSG0000001221	'PRICKLE3	-	X:49175263-49186528	-	-	IGV_LINK	164.852	6.38293	0.484431	12.2814	OK	169.121	6.45137	0.715305	12.1874	OK	OK	0.015387
ENSG0000001	-	-	ENSG0000001222	'LTF	-	3:46435644-46485234	-	-	IGV_LINK	37.8191	1.24303	0	3.24257	OK	213.464	5.80977	0.415503	11.204	OK	OK	2.22461
ENSG0000001	-	-	ENSG0000001223	'EXTL3	-	8:28600468-28755599	-	-	IGV_LINK	425.707	3.77169	1.19638	6.347	OK	620.797	5.52761	2.00567	9.04954	OK	OK	0.551444
ENSG0000001	-	-	ENSG0000001250	'NR1H4	-	12:100473707-100564413	-	-	IGV_LINK	871.779	29.5174	11.2535	47.7814	OK	1318.94	46.8999	17.3036	76.4962	OK	OK	0.668017
ENSG0000001	-	-	ENSG0000001266	'ELOVL5	-	6:53267397-53349179	-	-	IGV_LINK	1217	30.0453	8.17664	51.914	OK	1332.34	31.3598	10.1484	52.5712	OK	OK	0.061777
ENSG0000001	-	-	ENSG0000001277	'ALOX5	-	10:45374175-45453121	-	-	IGV_LINK	1999.56	68.3057	13.9898	122.622	OK	2316.13	75.4014	14.6394	136.163	OK	OK	0.142585
ENSG0000001	-	-	ENSG0000001281	'KDM5D	-	Y:19703864-19744939	-	-	IGV_LINK	1304.27	25.9728	3.15403	48.7916	OK	1025.04	19.0819	1.86843	36.2954	OK	OK	-0.4448
ENSG0000001	-	-	ENSG0000001282	'CALCOCO1	-	12:53708516-53727745	-	-	IGV_LINK	4182.4	113.98	26.0813	201.88	OK	4773.54	131.418	26.1591	236.677	OK	OK	0.205378
ENSG0000001	-	-	ENSG0000001296	'UBR7	-	14:93184950-93229215	-	-	IGV_LINK	707.896	11.9754	3.26187	20.689	OK	658.953	11.6738	1.61627	21.7314	OK	OK	-0.0368
ENSG0000001	-	-	ENSG0000001298	'MAP4K5	-	14:50418500-50668331	-	-	IGV_LINK	458.678	7.71297	0	16.9963	OK	369.179	6.45893	0	14.8573	OK	OK	-0.25599
ENSG0000001	-	-	ENSG0000001301	'EHD3	-	2:31234336-31269447	-	-	IGV_LINK	300.613	3.79551	1.25021	6.34081	OK	238.213	3.00843	0.889137	5.12772	OK	OK	-0.33529
ENSG0000001	-	-	ENSG0000001327	'PSMC4	-	19:39971004-39981441	-	-	IGV_LINK	843.657	36.1685	12.9249	59.412	OK	1025.04	43.0014	18.1684	67.8344	OK	OK	0.249651
ENSG0000001	-	-	ENSG0000001328	'MAN2B2	-	4:6575174-6623362	-	-	IGV_LINK	1149.12	12.5931	3.7417	21.4444	OK	1350.9	14.291	5.1291	23.4529	OK	OK	0.182478
ENSG0000001	-	-	ENSG0000001329	'SLC7A14	-	3:170418864-170860380	-	-	IGV_LINK	0	0	0	0	OK	2.06245	0.011706	0	0.252668	OK	NOTEST	inf
ENSG0000001	-	-	ENSG0000001329	'CLDN11	-	3:170418864-170860380	-	-	IGV_LINK	10.6669	0.732058	0	1.88803	OK	10.3122	0.41791	0	1.14097	OK	OK	-0.80877
ENSG0000001	-	-	ENSG0000001330	'SLC25A39	-	17:44319624-44324870	-	-	IGV_LINK	1687.31	70.8275	28.7817	112.873	OK	1724.21	70.6101	28.979	112.241	OK	OK	-0.00443
ENSG0000001	-	-	ENSG0000001336	'MVP	-	16:29806105-29848039	-	-	IGV_LINK	6141.24	155.081	0	324.779	OK	6947.94	168.585	1.44642	335.723	OK	OK	0.120458
ENSG0000001	-	-	ENSG0000001337	'NUB1	-	7:151341698-151413354	-	-	IGV_LINK	2046.11	59.5939	14.2139	104.974	OK	1743.8	53.9751	12.0056	95.9446	OK	OK	-0.14287
ENSG0000001	-	-	ENSG0000001337	'PGM3	-	6:83067665-83198932	-	-	IGV_LINK	692.38	13.1444	0	32.3223	OK	529.018	11.4359	0	32.531	OK	OK	-0.20088
ENSG0000001	-	-	ENSG0000001339	'RWDD2A	-	6:83067665-83198932	-	-	IGV_LINK	206.55	3.25922	0	9.44294	OK	115.497	1.82305	0	5.11437	OK	OK	-0.83817
ENSG0000001	-	-	ENSG0000001344	'CLK1	-	2:200853008-200864744	-	-	IGV_LINK	3491.96	98.8406	25.4201	172.261	OK	2378	68.2784	20.5825	115.974	OK	OK	-0.53367
ENSG0000001	-	-	ENSG0000001350	'POLR3B	-	12:106357657-106889316	-	-	IGV_LINK	73.6988	1.01289	0	3.01054	OK	76.3106	1.04908	0	3.24489	OK	OK	0.050656
ENSG0000001	-	-	ENSG0000001352	'ANGEL1	-	14:76762188-76826246	-	-	IGV_LINK	595.263	9.87289	0	30.2701	OK	546.06	8.98178	0	23.2754	OK	OK	-0.13647
ENSG0000001	-	-	ENSG0000001356	'RNF14	-	5:141943584-141990291	-	-	IGV_LINK	1012.39	36.0483	0.956469	71.1401	OK	1319.97	47.4556	1.30814	93.6031	OK	OK	0.396648
ENSG0000001	-	-	ENSG0000001356	'DNASE1L1	-	X:154389954-154421726	-	-	IGV_LINK	846.566	24.5848	0	163.032	OK	988.945	28.7728	0	146.045	OK	OK	0.226941
ENSG0000001	-	-	ENSG0000001357	'DDX11	-	12:31020762-31104791	-	-	IGV_LINK	681.714	27.9674	3.15231	52.7824	OK	717.733	28.7299	3.38809	54.0718	OK	OK	0.03881
ENSG0000001	-	-	ENSG0000001358	'HEBP1	-	12:12927725-13000273	-	-	IGV_LINK	554.68	31.3729	12.1432	50.6027	OK	694.014	39.0651	15.9855	62.1447	OK	OK	0.316359
ENSG0000001	-	-	ENSG0000001358	'GPRC5A	-	12:12890781-12917937	-	-	IGV_LINK	5.81832	0.148282	0	0.588151	OK	251.619	5.46208	0	11.817	OK	OK	5.20303
ENSG0000001	-	-	ENSG0000001361	'MAMLD1	-	X:150361421-150514178	-	-	IGV_LINK	108.609	1.80024	0	3.69227	OK	78.3731	1.16126	0	2.35244	OK	OK	-0.6325
ENSG0000001	-	-	ENSG0000001372	'CD6	-	11:60971679-61020377	-	-	IGV_LINK	564.377	13.3145	0.488282	26.1408	OK	384.647	8.69197	0.199974	17.184	OK	OK	-0.61525
ENSG0000001	-	-	ENSG0000001381	'TACC3	-	4:1721489-1745176	-	-	IGV_LINK	685.592	17.6877	5.40357	29.9719	OK	814.668	21.4491	5.52089	37.3772	OK	OK	0.278165
ENSG0000001	-	-	ENSG0000001412	'UFL1	-	6:96358373-96555276	-	-	IGV_LINK	806.807	11.7883	3.14012	20.4364	OK	548.612	8.03111	1.8218	14.2404	OK	OK	-0.55368
ENSG0000001	-	-	ENSG0000001413	'POLA2	-	11:65261761-65305589	-	-	IGV_LINK	337.463	12.5355	1.35947	23.7115	OK	333.086	10.9226	1.20735	20.6379	OK	OK	-0.19869
ENSG0000001	-	-	ENSG0000001416	'ZC3H3	-	8:143437654-143541453	-	-	IGV_LINK	476.133	9.15679	2.2822	16.0314	OK	537.268	10.1593	3.1237	17.1949	OK	OK	0.149891
ENSG0000001	-	-	ENSG0000001421	'CAPN1	-	11:65177605-65212006	-	-	IGV_LINK	2590.12	59.7998	18.9787	100.621	OK	3532.98	81.0486	22.7515	139.346	OK	OK	0.438647
ENSG0000001	-	-	ENSG0000001425	'ACPP	-	3:132317366-132368298	-	-	IGV_LINK	34.9099	1.13432	0	2.54988	OK	46.4051	2.37558	0	5.81127	OK	OK	1.06646
ENSG0000001	-	-	ENSG0000001464	'MDH1	-	2:63121382-63827843	-	-	IGV_LINK	1930.71	100.623	28.2478	172.998	OK	2562.59	127.703	43.7258	211.68	OK	OK	0.343831
ENSG0000001	-	-	ENSG0000001482	'SLC30A9	-	4:41990471-42090457	-	-	IGV_LINK	923.174	23.7284	5.72807	41.7288	OK	1160.13	27.5396	8.07145	47.0078	OK	OK	0.214892
ENSG0000001	-	-	ENSG0000001491	'MTMR11	-	1:149928650-149936869	-	-	IGV_LINK	2179.93	65.925	19.8196	112.03	OK	2335.72	72.9997	19.9916	126.008	OK	OK	0.147065
ENSG0000001	-	-	ENSG0000001491	'COX15	-	10:99659505-99756134	-	-	IGV_LINK	876.627	25.3662	0	55.6082	OK	597.079	16.3134	0	35.0247	OK	OK	-0.63685
ENSG0000001	-	-	ENSG0000001513	'CCDC88C	-	14:91271322-91417844	-	-	IGV_LINK	763.17	12.3718	0	25.6185	OK	503.238	9.29954	0	20.5627	OK	OK	-0.41183
ENSG0000001	-	-	ENSG0000001515	'YAF2	-	12:42157103-42238349	-	-	IGV_LINK	211.399	13.6529	0	29.649	OK	277.4	22.6499	0	45.7489	OK	OK	0.730294
ENSG0000001	-	-	ENSG0000001517	'ZMYND11	-	10:134464-254637	-	-	IGV_LINK	1028.87	22.4401	7.27767	37.6025	OK	649.672	14.3436	3.59383	25.0933	OK	OK	-0.64567
ENSG0000001	-	-	ENSG0000001528	'WAS	-	X:48676595-48691427	-	-	IGV_LINK	637.106	28.875	5.58405	52.1659	OK	681.64	28.9123	9.4235	48.401	OK	OK	0.001861
ENSG0000001	-	-	ENSG0000001541	'DPEP1	-	16:89613307-89638456	-	-	IGV_LINK	9.69721	0.516447	0	1.56525	OK	8.2498	0.316933	0	1.13192	OK	NOTEST	-0.70444
ENSG0000001	-	-	ENSG0000001547	'BID	-	22:17734137-17774770	-	-	IGV_LINK	547.892	21.2241	4.4913	37.957	OK	591.923	23.8877	5.1859	42.5894	OK	OK	0.170559
ENSG0000001	-	-	ENSG0000001547	'MATR3	-	5:138946719-139331677	-	-	IGV_LINK	4218.51	99.7104	4.03569	195.385	OK	3711.09	88.246	2.12034	174.372	OK	OK	-0.17621
ENSG0000001	-	-	ENSG0000001552	'NPC1L1	-	7:44512534-44541315	-	-	IGV_LINK	1.93944	0.023485	0	0.098939	OK	2.06245	0.024981	0	0.10037	OK	NOTEST	0.089077
ENSG0000001	-	-	ENSG0000001553	'XYLT2	-	17:50346091-50365232	-	-	IGV_LINK	699.168	13.0397	4.74672	21.3327	OK	705.358	12.9202	0	26.7574	OK	OK	-0.01328
ENSG0000001	-	-	ENSG0000001556	'RGPD5	-	2:109792757-109857695	-	-	IGV_LINK	5.81832	0.364441	0	1.32486	OK	12.3747	0.610178	0	1.72669	OK	OK	0.743544
ENSG0000001	-	-	ENSG0000001559	'STMN4	-	8:27235322-27258420	-	-	IGV_LINK	0.969721	0.065724	0	0.389659	OK	0	0	0	0	OK	NOTEST	'-inf
ENSG0000001	-	-	ENSG0000001567	'NUDCD3	-	7:44379120-44490880	-	-	IGV_LINK	1654.34	39.2577	5.15499	73.3605	OK	1899.52	44.2136	6.13217	82.2951	OK	OK	0.171514
ENSG0000001	-	-	ENSG0000001608	'ISL1	-	5:51383390-51394738	-	-	IGV_LINK	0	0	0	0	OK	0	0	0	0	OK	NOTEST	0
ENSG0000001	-	-	ENSG0000001639	'CHDH	-	3:53283427-53846390	-	-	IGV_LINK	1254.82	9.90691	0.583834	19.23	OK	2158.35	20.1495	0	45.1276	OK	OK	1.02424
ENSG0000001	-	-	ENSG0000001640	'IL20RA	-	6:136999970-137045180	-	-	IGV_LINK	0	0	0	0	OK	0	0	0	0	OK	NOTEST	0
ENSG0000001	-	-	ENSG0000001649	'CLCA1	-	1:86468367-86500289	-	-	IGV_LINK	0	0	0	0	OK	0	0	0	0	OK	NOTEST	0
ENSG0000001	-	-	ENSG0000001660	'CLCA4	-	1:86547077-86693203	-	-	IGV_LINK	0	0	0	0	OK	0	0	0	0	OK	NOTEST	0
ENSG0000001	-	-	ENSG0000001686	'GLT8D1	-	3:52495337-52770949	-	-	IGV_LINK	508.134	24.2935	0	77.8007	OK	550.674	25.3356	0	131.029	OK	OK	0.060595
ENSG0000001	-	-	ENSG0000001726	'ATP2C1	-	3:130850594-131381475	-	-	IGV_LINK	2109.14	39.7747	6.37344	73.1759	OK	1759.27	35.0357	3.28627	66.7852	OK	OK	-0.18302
ENSG0000001	-	-	ENSG0000001742	'IGF1	-	12:102120184-102480645	-	-	IGV_LINK	49.4557	0.893175	0	5.53751	OK	623.891	15.3542	0	37.3464	OK	OK	4.10355
ENSG0000001	-	-	ENSG0000001748	'SLC38A5	-	X:48458536-48470256	-	-	IGV_LINK	65.941	3.34076	0	6.99783	OK	655.859	27.728	8.55651	46.8994	OK	OK	3.0531
ENSG0000001	-	-	ENSG0000001779	'RALBP1	-	18:9475008-9538116	-	-	IGV_LINK	1250.94	17.6437	7.35084	27.9365	OK	1315.84	19.9717	6.96047	32.983	OK	OK	0.178809
ENSG0000001	-	-	ENSG0000001818	'RUFY3	-	4:70703746-70807315	-	-	IGV_LINK	1071.54	40.6138	7.76446	73.4631	OK	1839.71	67.8585	12.9609	122.756	OK	OK	0.74056
ENSG0000001	-	-	ENSG0000001823	'CNTN1	-	12:40692441-41072418	-	-	IGV_LINK	0	0	0	0	OK	0	0	0	0	OK	NOTEST	0
ENSG0000001	-	-	ENSG0000001828	'SLC11A1	-	2:218382028-218396894	-	-	IGV_LINK	322.917	8.60979	0.796994	16.4226	OK	851.792	28.7339	1.60532	55.8624	OK	OK	1.7387
ENSG0000001	-	-	ENSG0000001840	'WWTR1	-	3:149517234-149736714	-	-	IGV_LINK	1917.14	50.7287	0.91345	100.544	OK	2182.07	59.0634	0.362092	117.765	OK	OK	0.219462
ENSG0000001	-	-	ENSG0000001851	'AGPS	-	2:177227594-177559299	-	-	IGV_LINK	855.293	13.1732	0	33.5002	OK	879.635	13.3787	0	38.2669	OK	OK	0.022336
ENSG0000001	-	-	ENSG0000001860	'ZNF806	-	2:132309308-132318736	-	-	IGV_LINK	0	0	0	0	OK	0	0	0	0	OK	NOTEST	0
ENSG0000001	-	-	ENSG0000001861	'CXorf56	-	X:119538148-119565408	-	-	IGV_LINK	329.705	13.3553	2.28807	24.4225	OK	367.116	13.9318	2.70897	25.1546	OK	OK	0.060972
ENSG0000001	-	-	ENSG0000001862	'ATP1A2	-	1:160115758-160143591	-	-	IGV_LINK	53.3346	0.686449	0	1.77604	OK	77.3419	0.933216	0	2.02771	OK	OK	0.443059
ENSG0000001	-	-	ENSG0000001869	'TTC27	-	2:32628031-32821051	-	-	IGV_LINK	367.524	8.25567	1.877	14.6343	OK	386.709	8.06474	2.81103	13.3185	OK	OK	-0.03376
ENSG0000001	-	-	ENSG0000001886	'ZNF582	-	19:56219669-56393545	-	-	IGV_LINK	45.5769	2.1932	0	7.13563	OK	24.7494	1.36491	0	5.09227	OK	OK	-0.68424
ENSG0000001	-	-	ENSG0000001910	'VSIG2	-	11:124747471-124752238	-	-	IGV_LINK	34.9099	1.98394	0	4.27902	OK	17.5308	0.993359	0	2.27428	OK	OK	-0.99798
ENSG0000001	-	-	ENSG0000001914	'PHLDB1	-	11:118606439-118679690	-	-	IGV_LINK	1931.68	34.2487	2.60061	65.8967	OK	1803.61	30.7317	1.89517	59.5682	OK	OK	-0.15632
ENSG0000001	-	-	ENSG0000001916	'MARCO	-	2:118942165-118994660	-	-	IGV_LINK	30.0613	1.0172	0	2.16605	OK	36.0929	1.22217	0	2.55265	OK	OK	0.264833
ENSG0000001	-	-	ENSG0000001918	'CYP24A1	-	20:54153448-54173973	-	-	IGV_LINK	2338	81.576	7.42347	155.729	OK	10136.9	354.88	9.19068	700.57	OK	OK	2.12112
ENSG0000001	-	-	ENSG0000001948	'PRDM11	-	11:45095805-45235292	-	-	IGV_LINK	219.157	2.59958	0	5.40445	OK	138.184	1.82922	0	4.64204	OK	OK	-0.50705
ENSG0000001	-	-	ENSG0000001950	'SYT13	-	11:45240300-45286319	-	-	IGV_LINK	490.679	5.56221	2.11005	9.01436	OK	397.022	4.50158	1.60672	7.39644	OK	OK	-0.30523
ENSG0000001	-	-	ENSG0000001954	'SNAI2	-	8:48917767-48921740	-	-	IGV_LINK	34.9099	1.07069	0	2.21346	OK	41.249	1.26591	0	2.58097	OK	OK	0.241624

ENSG0000001	-	-	ENSG0000001958	'CD74	-	5:150401636-150412929	-	-	IGV_LINK	138452	7037.96	0	18406.7	OK	93038.1	4694.04	0	11711.8	OK	OK	-0.58433
ENSG0000001	-	-	ENSG0000001999	'HGF	-	7:81699005-81770438	-	-	IGV_LINK	263.764	6.36685	0	13.1366	OK	175.308	4.11425	0	8.62189	OK	OK	-0.62995
ENSG0000001	-	-	ENSG0000001999	'ZRANB1	-	10:124917142-125161170	-	-	IGV_LINK	277.435	2.83114	0	8.46914	OK	199.265	2.03387	0	4.74547	OK	OK	-0.47716
ENSG0000002	-	-	ENSG0000002012	'NCDN	-	1:35433489-35567274	-	-	IGV_LINK	529.467	9.74938	0	23.1528	OK	628.016	11.8012	0	32.2749	OK	OK	0.275545
ENSG0000002	-	-	ENSG0000002018	'ADGRA2	-	8:37784190-37849904	-	-	IGV_LINK	651.652	6.67886	1.65052	11.7072	OK	395.99	4.11853	0.439108	7.79796	OK	OK	-0.69747
ENSG0000002	-	-	ENSG0000002021	'CCT8L1P	-	7:152445476-152447150	-	-	IGV_LINK	0.969721	0.036397	0	0.142557	OK	0	0	0	0	OK	NOTEST	'-inf
ENSG0000002	-	-	ENSG0000002025	'ZFP64	-	20:52051662-52204308	-	-	IGV_LINK	239.521	5.45522	0	11.1657	OK	291.837	5.83455	1.73075	9.93836	OK	OK	0.096986
ENSG0000002	-	-	ENSG0000002042	'MNAT1	-	14:60734741-61083733	-	-	IGV_LINK	167.762	11.9449	0	26.1089	OK	173.246	12.9048	0	29.3723	OK	OK	0.111514
ENSG0000002	-	-	ENSG0000002057	'SAMD4A	-	14:54567096-54793315	-	-	IGV_LINK	639.046	7.08621	0.859601	13.3128	OK	770.325	9.7686	0	20.6771	OK	OK	0.463138
ENSG0000002	-	-	ENSG0000002063	'RUNX3	-	1:24899510-24965121	-	-	IGV_LINK	788.383	12.4553	3.67482	21.2359	OK	768.263	13.0988	2.66991	23.5278	OK	OK	0.072673
ENSG0000002	-	-	ENSG0000002092	'MRE11	-	11:94415577-94499583	-	-	IGV_LINK	707.896	9.40538	1.75335	17.0574	OK	679.577	10.0949	1.04215	19.1476	OK	OK	0.102064
ENSG0000002	-	-	ENSG0000002130	'PLEKHB1	-	11:73646177-73662819	-	-	IGV_LINK	105.7	3.30109	0.024461	6.57771	OK	176.339	5.76637	0.812982	10.7198	OK	OK	0.804723
ENSG0000002	-	-	ENSG0000002135	'SERPINB1	-	6:2832331-2842006	-	-	IGV_LINK	1610.71	57.52	15.1855	99.8546	OK	1479.81	53.5159	11.29	95.7418	OK	OK	-0.1041
ENSG0000002	-	-	ENSG0000002146	'CYP3A43	-	7:99828012-99866102	-	-	IGV_LINK	4.8486	0.238051	0	1.00128	OK	1.03122	0.071173	0	0.702821	OK	NOTEST	-1.74187
ENSG0000002	-	-	ENSG0000002148	'SLC7A9	-	19:32830508-32869766	-	-	IGV_LINK	216.248	8.72357	1.97463	15.4725	OK	503.238	19.7975	7.22791	32.3672	OK	OK	1.18233
ENSG0000002	-	-	ENSG0000002157	'SPAST	-	2:32063610-32157637	-	-	IGV_LINK	174.55	3.47904	0.195956	6.76213	OK	178.402	3.84335	0.414154	7.27255	OK	OK	0.143675
ENSG0000002	-	-	ENSG0000002164	'NRXN3	-	14:78170372-79868290	-	-	IGV_LINK	172.61	2.49494	0.209422	4.78045	OK	85.5917	1.14398	0	2.48486	OK	OK	-1.12494
ENSG0000002	-	-	ENSG0000002176	'OSBPL5	-	11:3087115-3166739	-	-	IGV_LINK	383.04	9.76656	1.27471	18.2584	OK	371.241	7.90783	0.970168	14.8455	OK	OK	-0.30457
ENSG0000002	-	-	ENSG0000002177	'AQR	-	15:34851781-34969839	-	-	IGV_LINK	791.292	9.38991	0	20.0409	OK	837.355	9.62205	0	19.9821	OK	OK	0.035233
ENSG0000002	-	-	ENSG0000002182	'CPS1	-	2:210477681-210679107	-	-	IGV_LINK	8.72748	0.635387	0	1.8691	OK	534.175	7.00955	0	14.3784	OK	OK	3.46361
ENSG0000002	-	-	ENSG0000002185	'C8B	-	1:56929209-58546802	-	-	IGV_LINK	0	0	0	0	OK	0	0	0	0	OK	NOTEST	0
ENSG0000002	-	-	ENSG0000002226	'FHL1	-	X:136146701-136211359	-	-	IGV_LINK	1850.23	53.8478	19.2047	88.491	OK	6278.1	184.12	51.1282	317.112	OK	OK	1.77369
ENSG0000002	-	-	ENSG0000002227	'RTFDC1	-	20:56468584-56526142	-	-	IGV_LINK	1903.27	75.5806	27.8582	123.303	OK	2071.72	86.755	32.0801	141.43	OK	OK	0.198932
ENSG0000002	-	-	ENSG0000002235	'GABRA1	-	5:161847062-161899981	-	-	IGV_LINK	0	0	0	0	OK	0	0	0	0	OK	NOTEST	0
ENSG0000002	-	-	ENSG0000002255	'NLRP2	-	19:54923508-55001142	-	-	IGV_LINK	53.3346	1.8238	0	4.6812	OK	44.3427	1.3908	0	3.6351	OK	OK	-0.39104
ENSG0000002	-	-	ENSG0000002256	'SLC45A4	-	8:141207165-141308305	-	-	IGV_LINK	525.589	7.50276	0	15.9247	OK	535.206	8.1819	0	16.6442	OK	OK	0.125015
ENSG0000002	-	-	ENSG0000002284	'RNF10	-	12:120503273-120577594	-	-	IGV_LINK	1698.95	53.1823	0	108.765	OK	2399.66	72.6442	0	148.136	OK	OK	0.449902
ENSG0000002	-	-	ENSG0000002297	'ZNF839	-	14:102317376-102362916	-	-	IGV_LINK	140.609	3.68369	0	8.87114	OK	93.8415	3.30671	0	9.5788	OK	OK	-0.15575
ENSG0000002	-	-	ENSG0000002304	'ZDHHC6	-	10:112374017-112446917	-	-	IGV_LINK	1129.72	37.9213	0	80.8743	OK	653.797	21.4947	0	48.8369	OK	OK	-0.81903
ENSG0000002	-	-	ENSG0000002317	'GRAMD1B	-	11:123358427-123627774	-	-	IGV_LINK	393.707	5.0966	0	10.3165	OK	284.618	3.05338	0.405829	5.70093	OK	OK	-0.73913
ENSG0000002	-	-	ENSG0000002319	'RNH1	-	11:494511-507300	-	-	IGV_LINK	2118.84	72.4412	26.304	118.579	OK	2360.47	81.0277	27.504	134.551	OK	OK	0.161603
ENSG0000002	-	-	ENSG0000002322	'NDUFS1	-	2:206114816-206162928	-	-	IGV_LINK	1285.29	25.8369	0	82.9089	OK	1443.25	30.6236	0	81.7428	OK	OK	0.24521
ENSG0000002	-	-	ENSG0000002328	'RB1CC1	-	8:52622455-52745843	-	-	IGV_LINK	662.319	8.48277	0	18.695	OK	662.046	7.5695	0	16.1559	OK	OK	-0.16434
ENSG0000002	-	-	ENSG0000002331	'ERP44	-	9:99979178-100099040	-	-	IGV_LINK	1050.21	12.6423	5.48633	19.7984	OK	1103.41	13.2861	5.77791	20.7942	OK	OK	0.07165
ENSG0000002	-	-	ENSG0000002333	'ALAS1	-	3:52198085-52214327	-	-	IGV_LINK	461.587	14.3807	1.62159	27.1399	OK	440.333	12.3258	4.15573	20.4958	OK	OK	-0.22246
ENSG0000002	-	-	ENSG0000002344	'BIRC3	-	11:102317449-102339403	-	-	IGV_LINK	3208.81	42.2009	12.2588	72.1429	OK	11198.1	151.135	20.2516	282.018	OK	OK	1.84049
ENSG0000002	-	-	ENSG0000002351	'AKAP11	-	13:42272152-42323267	-	-	IGV_LINK	973.599	5.6289	2.42948	8.82831	OK	664.109	3.84002	1.56897	6.11107	OK	OK	-0.55174
ENSG0000002	-	-	ENSG0000002357	'GLRX2	-	1:193096467-193106114	-	-	IGV_LINK	35.8797	3.74716	0	7.64962	OK	92.8102	9.91871	0.998995	18.8384	OK	OK	1.40436
ENSG0000002	-	-	ENSG0000002360	'SNAPC1	-	14:61681040-61796428	-	-	IGV_LINK	159.034	3.60883	0	9.87587	OK	274.306	6.22747	0	17.8859	OK	OK	0.787117
ENSG0000002	-	-	ENSG0000002369	'DERA	-	12:15911171-16037282	-	-	IGV_LINK	562.438	33.1498	9.41489	56.8848	OK	923.978	52.2957	13.1339	91.4576	OK	OK	0.657692
ENSG0000002	-	-	ENSG0000002373	'STRAP	-	12:15882390-15903478	-	-	IGV_LINK	944.508	33.1261	12.9011	53.3511	OK	1040.51	35.9779	14.3878	57.568	OK	OK	0.119144
ENSG0000002	-	-	ENSG0000002383	'ABCC2	-	10:99782731-99852192	-	-	IGV_LINK	360.736	3.95554	1.36567	6.54542	OK	1886.11	20.7299	9.17084	32.289	OK	OK	2.38977
ENSG0000002	-	-	ENSG0000002389	'DEF6	-	6:35297851-35321771	-	-	IGV_LINK	823.293	29.3003	4.22963	54.371	OK	591.923	23.3685	1.73182	45.0052	OK	OK	-0.32635
ENSG0000002	-	-	ENSG0000002390	'PLEKHO1	-	1:150149182-150164720	-	-	IGV_LINK	2219.69	85.2324	26.3339	144.131	OK	1379.78	50.4969	15.0495	85.9444	OK	OK	-0.75521
ENSG0000002	-	-	ENSG0000002390	'GCLM	-	1:93885204-93909456	-	-	IGV_LINK	228.854	6.09738	0	13.0959	OK	689.89	25.3992	2.08847	48.71	OK	OK	2.05852
ENSG0000002	-	-	ENSG0000002404	'UBR2	-	6:42564061-42693504	-	-	IGV_LINK	1375.06	10.0843	4.48307	15.6854	OK	785.793	5.76361	2.26713	9.26009	OK	OK	-0.80706
ENSG0000002	-	-	ENSG0000002442	'EHD2	-	19:47607523-47743134	-	-	IGV_LINK	2579.46	42.846	16.7508	68.9413	OK	5145.81	92.2291	20.0633	164.395	OK	OK	1.10606
ENSG0000002	-	-	ENSG0000002452	'DEPDC1	-	1:68474151-68538627	-	-	IGV_LINK	31.0311	0.417149	0	1.11762	OK	39.1865	0.689962	0	2.05756	OK	OK	0.725953
ENSG0000002	-	-	ENSG0000002486	'CCDC28A	-	6:138725210-138793319	-	-	IGV_LINK	767.049	40.3413	16.8468	63.8357	OK	665.14	34.9363	14.116	55.7565	OK	OK	-0.20753
ENSG0000002	-	-	ENSG0000002503	'RRAGD	-	6:89364635-89412270	-	-	IGV_LINK	986.206	17.6963	3.11724	32.2753	OK	320.711	7.37594	0	15.2026	OK	OK	-1.26255
ENSG0000002	-	-	ENSG0000002515	'HSF2	-	6:122399545-122433119	-	-	IGV_LINK	164.852	3.82753	0.840181	6.81489	OK	95.9039	2.681	0	5.6378	OK	OK	-0.51364
ENSG0000002	-	-	ENSG0000002529	'PHF20	-	20:35771973-35950381	-	-	IGV_LINK	634.197	11.972	0	26.3923	OK	646.578	9.98879	0	21.3509	OK	OK	-0.26128
ENSG0000002	-	-	ENSG0000002542	'HSD17B6	-	12:56712427-56787790	-	-	IGV_LINK	6.78804	0.282847	0	9.18898	OK	5.15612	0.186531	0	5.89182	OK	NOTEST	-0.60061
ENSG0000002	-	-	ENSG0000002543	'NR1H3	-	11:47239301-47269032	-	-	IGV_LINK	928.992	48.6348	2.05562	95.2141	OK	1584.99	76.57	0	154.161	OK	OK	0.654789
ENSG0000002	-	-	ENSG0000002570	'TYMP	-	22:50523567-50532580	-	-	IGV_LINK	6196.65	231.602	34.0734	429.13	OK	4938.94	182.664	50.0955	315.233	OK	OK	-0.34245
ENSG0000002	-	-	ENSG0000002577	'NCAPH2	-	22:50508215-50523472	-	-	IGV_LINK	851.415	29.7336	10.4417	49.0255	OK	710.514	24.7171	8.86279	40.5714	OK	OK	-0.26659
ENSG0000002	-	-	ENSG0000002577	'TOMM34	-	20:44910061-44960486	-	-	IGV_LINK	481.951	14.3747	0	30.4798	OK	372.272	11.1101	0	23.9114	OK	OK	-0.37165
ENSG0000002	-	-	ENSG0000002579	'SEC63	-	6:107867755-107959986	-	-	IGV_LINK	1291.67	19.7975	0	40.5741	OK	971.414	15.7184	0	31.8074	OK	OK	-0.33287
ENSG0000002	-	-	ENSG0000002580	'KPNA6	-	1:32108037-32176568	-	-	IGV_LINK	1283.91	10.0417	4.42862	15.6548	OK	1599.43	12.7498	5.20219	20.2975	OK	OK	0.344476
ENSG0000002	-	-	ENSG0000002602	'VIM	-	10:17214238-17237593	-	-	IGV_LINK	51249.7	1708.49	0	3592.63	OK	42339	1412.21	20.128	2804.3	OK	OK	-0.27476
ENSG0000002	-	-	ENSG0000002603	'RTEL1-TNFRSF6B	-	20:63657809-63743505	-	-	IGV_LINK	548.354	5.85318	0	15.4929	OK	526.463	5.65328	0	14.7275	OK	OK	-0.05013
ENSG0000002	-	-	ENSG0000002610	'FAS	-	10:88879733-89015785	-	-	IGV_LINK	779.655	31.4877	0	69.1636	OK	584.705	23.7971	0	55.4514	OK	OK	-0.40401
ENSG0000002	-	-	ENSG0000002629	'RNASET2	-	6:166409363-166957191	-	-	IGV_LINK	10691.9	585.556	124.45	1046.66	OK	6188.8	344.881	72.6409	617.121	OK	OK	-0.76371
ENSG0000002	-	-	ENSG0000002650	'CD44	-	11:35138869-35232402	-	-	IGV_LINK	1742.59	45.209	7.67795	82.74	OK	5896.54	137.202	19.9659	254.438	OK	OK	1.60162
ENSG0000002	-	-	ENSG0000002655	'KCNG1	-	20:50999369-51023129	-	-	IGV_LINK	0.969721	0.068784	0	0.408562	OK	4.1249	0.141616	0	0.560192	OK	NOTEST	1.04184
ENSG0000002	-	-	ENSG0000002665	'AGPAT4	-	6:161129978-161274061	-	-	IGV_LINK	293.825	3.5647	0	8.21567	OK	187.683	1.91512	0	4.37564	OK	OK	-0.89634
ENSG0000002	-	-	ENSG0000002675	'SLAMF7	-	1:160739056-160754821	-	-	IGV_LINK	1253.85	27.3019	11.2054	43.3984	OK	546.549	12.0154	3.93149	20.0993	OK	OK	-1.18412
ENSG0000002	-	-	ENSG0000002695	'BTN3A1	-	6:26402236-26415216	-	-	IGV_LINK	4743.87	106.467	27.6233	185.31	OK	1833.52	40.8151	13.3609	68.2693	OK	OK	-1.38323
ENSG0000002	-	-	ENSG0000002700	'MIPEP	-	13:23730188-23902655	-	-	IGV_LINK	344.251	8.92585	2.86051	14.9912	OK	378.46	9.95678	3.16723	16.7463	OK	OK	0.15769
ENSG0000002	-	-	ENSG0000002707	'PRKCH	-	14:61187558-61658696	-	-	IGV_LINK	1098.69	22.3792	5.2523	39.5061	OK	714.639	16.4089	0	33.1187	OK	OK	-0.44768
ENSG0000002	-	-	ENSG0000002764	'INSRR	-	1:156806242-156881850	-	-	IGV_LINK	5.81832	0.06653	0	0.370787	OK	8.2498	0.094356	0	0.472998	OK	NOTEST	0.504092
ENSG0000002	-	-	ENSG0000002769	'IFNGR1	-	6:137197483-137219449	-	-	IGV_LINK	2875.22	83.2619	35.6972	130.827	OK	1898.49	56.9702	23.7871	90.1534	OK	OK	-0.54745
ENSG0000002	-	-	ENSG0000002784	'B4GALT7	-	5:177600099-177610347	-	-	IGV_LINK	428.616	15.5998	4.21652	26.983	OK	639.359	23.6674	6.26084	41.0739	OK	OK	0.601373
ENSG0000002	-	-	ENSG0000002786	'SH2D2A	-	1:156806242-156881850	-	-	IGV_LINK	231.763	9.21641	1.74959	16.6832	OK	252.65	11.8947	2.39578	21.3936	OK	OK	0.368043
ENSG0000002	-	-	ENSG0000002811	'VRK2	-	2:57907650-58241686	-	-	IGV_LINK	273.461	9.18258	0.30624	18.0589	OK	196.964	6.95564	0	15.2074	OK	OK	-0.40072
ENSG0000002	-	-	ENSG0000002813	'TNFRSF1B	-	1:12166942-12209228	-	-	IGV_LINK	1777.5	29.2826	11.2644	47.3008	OK	2442.97	39.5567	16.1109	63.0025	OK	OK	0.433879
ENSG0000002	-	-	ENSG0000002820	'VEZT	-	12:95217745-95302790	-	-	IGV_LINK	958.084	25.2874	6.0119	44.563	OK	1278.72	35.3228	7.7657	62.88	OK	OK	0.48218
ENSG0000002	-	-	ENSG0000002827	'POU2F2	-	19:42086109-42196585	-	-	IGV_LINK	452.859	6.67644	0	15.0113	OK	344.429	5.37131	0	12.5612	OK	OK	-0.3138
ENSG0000002	-	-	ENSG0000002831	'BRD9	-	5:795605-919357	-	-	IGV_LINK	832.99	20.3246	3.62565	37.0235	OK	813.884	19.6838	4.91488	34.4527	OK	OK	-0.04622
ENSG0000002	-	-	ENSG0000002852	'SNX1	-	15:64094122-64146090	-	-	IGV_LINK	2758.85	62.2335	14.3966	110.07	OK	2806.99	70.9904	17.2888	124.692	OK	OK	0.189932
ENSG0000002	-	-	ENSG0000002883	'TBPL1	-	6:133952169-134052636	-	-	IGV_LINK	164.852	10.3629	0.801559	19.9243	OK	90.7478	5.04763	0	10.7913	OK	OK	-1.03775
ENSG0000002	-	-	ENSG0000002915	'ARNTL2	-	12:27332853-27446625	-	-	IGV_LINK	144.488	1.88046	0	3.81841	OK	112.404	1.79036	0	3.84675	OK	OK	-0.07084
ENSG0000002	-	-	ENSG0000002936	'BCLAF1	-	6:136256626-136289851	-	-	IGV_LINK	1706.71	41.2975	0.763674	81.8314	OK	1261.19	34.9363	1.67531	68.1972	OK	OK	-0.24133
ENSG0000002	-	-	ENSG0000002936	'SLC39A9	-	14:69380122-69462388	-	-	IGV_LINK	908.628	14.2533	1.87952	26.627	OK	1041.54	15.9454	3.434	28.4567	OK	OK	0.161846
ENSG0000002	-	-	ENSG0000002953	'ANK1	-	8:41653219-41896762	-	-	IGV_LINK	435.405	4.09593	0.274347	7.91751	OK	76.3106	0.883231	0	2.95119	OK	OK	-2.21333
ENSG0000002	-	-	ENSG0000002955	'IBSP	-	4:87799580-87812435	-	-	IGV_LINK	1.93944	0.079578	0	0.243703	OK	5.15612	0.21174	0	0.519047	OK	NOTEST	1.41186
ENSG0000002	-	-	ENSG0000002963	'TFB1M	-	6:154733324-155314493	-	-	IGV_LINK	182.307	10.4879	0	30.5858	OK	121.685	8.01551	0	23.6389	OK	OK	-0.38786
ENSG0000002	-	-	ENSG0000002972	'RABEP1	-	17:5282264-5469060	-	-	IGV_LINK	728.26	10.8463	0	32.6539	OK	666.171	8.79123	0	24.6072	OK	OK	-0.30307
ENSG0000002	-	-	ENSG0000002999	'HMGB3	-	X:150980508-150990775	-	-	IGV_LINK	108.609	5.68672	0	12.4421	OK	108.279	5.06431	0	11.3829	OK	OK	-0.16723
ENSG0000003	-	-	ENSG0000003006	'NUP160	-	11:47778086-47848555	-	-	IGV_LINK	1017.24	12.6526	2.54634	22.7589	OK	918.821	11.0017	2.74784	19.2555	OK	OK	-0.20172
ENSG0000003	-	-	ENSG0000003011	'BAK1	-	6:33540045-33593338	-	-	IGV_LINK	557.589	15.9365	6.28913	25.5839	OK	441.364	12.678	4.59985	20.7561	OK	OK	-0.33001
ENSG0000003	-	-	ENSG0000003030	'MUSK	-	9:110668770-110801620	-	-	IGV_LINK	0	0	0	0	OK	1.03122	0.022522	0	0.377968	OK	NOTEST	inf
ENSG0000003	-	-	ENSG0000003041	'IKZF2	-	2:212581356-213152427	-	-	IGV_LINK	258.915	2.94277	0	6.95682	OK	140.247	1.54964	0	3.79906	OK	OK	-0.92524
ENSG0000003	-	-	ENSG0000003058	'GRN	-	17:44345085-44353102	-	-	IGV_LINK	7114.84	245.488	57.0868	433.888	OK	10305	335.79	66.8971	604.683	OK	OK	0.45191
ENSG0000003	-	-	ENSG0000003100	'FAM13B	-	5:137814332-138051961	-	-	IGV_LINK	887.294	13.0277	0.39135	25.664	OK	712.576	8.82426	1.27223	16.3763	OK	OK	-0.56203
ENSG0000003	-	-	ENSG0000003108	'ARHGAP31	-	3:119294372-119420714	-	-	IGV_LINK	1106.45	8.34534	0	18.2726	OK	959.039	7.00483	0	15.0581	OK	OK	-0.25262
ENSG0000003	-	-	ENSG0000003169	'CENPQ	-	6:49463377-49493107	-	-	IGV_LINK	50.4255	1.82108	0.009596	3.63256	OK	48.4676	1.75166	0.000412	3.50291	OK	OK	-0.05607
ENSG0000003	-	-	ENSG0000003169	'SARS	-	1:109213917-109238169	-	-	IGV_LINK	2505.76	89.0279	36.9754	141.08	OK	2266.63	82.4787	33.8916	131.066	OK	OK	-0.11024
ENSG0000003	-	-	ENSG0000003182	'RANBP3	-	19:5916138-5978142	-	-	IGV_LINK	996.873	35.5914	3.16174	68.021	OK	976.57	34.4819	2.59325	66.3705	OK	OK	-0.04569
ENSG0000003	-	-	ENSG0000003221	'ARID4A	-	14:58298384-58395641	-	-	IGV_LINK	552.741	7.99873	0	16.2539	OK	413.521	5.67311	0	11.8334	OK	OK	-0.49563
ENSG0000003	-	-	ENSG0000003238	'EIPR1	-	2:3188924-3377882	-	-	IGV_LINK	319.038	12.0503	3.43206	20.6686	OK	360.929	14.0777	4.01453	24.1409	OK	OK	0.22434
ENSG0000003	-	-	ENSG0000003244	'PNPLA6	-	19:7522625-7561764	-	-	IGV_LINK	1111.3	24.4308	0	51.6511	OK	1411.75	27.7525	0	59.3066	OK	OK	0.183913
ENSG0000003	-	-	ENSG0000003274	'IFT88	-	13:20567068-20691437	-	-	IGV_LINK	494.557	13.8929	2.26434	25.5214	OK	460.958	12.1819	1.39005	22.9738	OK	OK	-0.18961
ENSG0000003	-	-	ENSG0000003301	'ALG1	-	16:4882506-5097808	-	-	IGV_LINK	372.594	13.7786	0	30.6736	OK	576.661	25.7531	0	65.7926	OK	OK	0.902317
ENSG0000003	-	-	ENSG0000003303	'ZCCHC8	-	12:122471599-122501073	-	-	IGV_LINK	779.655	17.8311	6.62297	29.0392	OK	676.484	15.8614	5.08654	26.6362	OK	OK	-0.16888
ENSG0000003	-	-	ENSG0000003305	'ABCF2	-	7:151207836-151227230	-	-	IGV_LINK	630.318	12.8694	4.67197	21.0669	OK	829.105	18.6789	2.46363	34.8942	OK	OK	0.537464
ENSG0000003	-	-	ENSG0000003310	'CHPF2	-	7:151232488-151277896	-	-	IGV_LINK	649.713	10.6094	2.49008	18.7286	OK	969.351	15.9362	3.74647	28.126	OK	OK	0.586974
ENSG0000003	-	-	ENSG0000003312	'LRRC7	-	1:69568397-70205620	-	-	IGV_LINK	6.78804	0.220983	0	1.43405	OK	10.3122	0.404834	0	2.42617	OK	NOTEST	0.873393
ENSG0000003	-	-	ENSG0000003317	'FUT8	-	14:65410591-65744121	-	-	IGV_LINK	426.677	13.524	2.50205	24.5459	OK	734.232	22.5221	5.52699	39.5172	OK	OK	0.735822
ENSG0000003	-	-	ENSG0000003317	'UBA6	-	4:67612651-67701179	-	-	IGV_LINK	546.922	7.26274	0	17.1124	OK	487.769	8.22082	0	21.2105	OK	OK	0.178768
ENSG0000003	-	-	ENSG0000003332	'GAB2	-	11:78215296-78574874	-	-	IGV_LINK	787.413	7.67352	1.74324	13.6038	OK	1002.35	10.0898	1.62368	18.5559	OK	OK	0.394935
ENSG0000003	-	-	ENSG0000003362	'ATP6V0A1	-	17:42458843-42522611	-	-	IGV_LINK	2342.84	47.0897	9.02544	85.154	OK	3608.42	73.7562	14.9357	132.577	OK	OK	0.647354
ENSG0000003	-	-	ENSG0000003380	'PIAS1	-	15:68054178-68257211	-	-	IGV_LINK	740.866	16.3751	0	39.3839	OK	657.922	15.8346	0	39.6203	OK	OK	-0.04842
ENSG0000003	-	-	ENSG0000003386	'SLC4A7	-	3:27372720-27484420	-	-	IGV_LINK	806.807	11.8469	0	23.929	OK	1093.1	18.1903	0	36.7353	OK	OK	0.618652
ENSG0000003	-	-	ENSG0000003405	'APBA2	-	15:28884482-29118315	-	-	IGV_LINK	69.8199	1.4365	0	3.12537	OK	304.211	5.90823	1.79466	10.0218	OK	OK	2.04017
ENSG0000003	-	-	ENSG0000003415	'MAP2K3	-	17:21284671-21315240	-	-	IGV_LINK	582.802	18.4817	5.90791	31.0554	OK	721.857	22.5241	7.23696	37.8112	OK	OK	0.285372
ENSG0000003	-	-	ENSG0000003423	'EFCAB1	-	8:48710788-48735311	-	-	IGV_LINK	5.81832	0.494022	0	1.45462	OK	6.18735	0.225978	0	0.869969	OK	NOTEST	-1.12839
ENSG0000003	-	-	ENSG0000003451	'TMSB10	-	2:84821649-84907008	-	-	IGV_LINK	15452.5	2691.71	668.447	4714.97	OK	16086.1	2812.16	652.052	4972.27	OK	OK	0.063159
ENSG0000003	-	-	ENSG0000003453	'ASTE1	-	3:130850594-131381475	-	-	IGV_LINK	189.095	4.41029	0	14.7934	OK	134.059	3.4466	0	13.4725	OK	OK	-0.3557
ENSG0000003	-	-	ENSG0000003467	'RNF19A	-	8:100157905-100336218	-	-	IGV_LINK	2209.99	78.353	0	160.593	OK	4015.59	131.866	0	270.652	OK	OK	0.751014
ENSG0000003	-	-	ENSG0000003469	'PEX3	-	6:143450806-143511690	-	-	IGV_LINK	229.824	9.56734	0	26.3393	OK	180.464	8.06624	0	23.8559	OK	OK	-0.24622
ENSG0000003	-	-	ENSG0000003471	'GABARAPL2	-	16:75528531-75623300	-	-	IGV_LINK	1268.39	82.5474	25.9305	139.164	OK	1309.66	85.0903	25.9271	144.253	OK	OK	0.043773
ENSG0000003	-	-	ENSG0000003497	'MYOC	-	1:171600620-171652683	-	-	IGV_LINK	19.3944	0.703483	0	2.39058	OK	15.4684	0.499619	0	1.85595	OK	OK	-0.49369
ENSG0000003	-	-	ENSG0000003511	'SH3YL1	-	2:217729-278283	-	-	IGV_LINK	869.839	44.1428	0	94.6811	OK	862.104	46.0732	0	97.0953	OK	OK	0.061748
ENSG0000003	-	-	ENSG0000003514	'FAM136A	-	2:70295974-70302090	-	-	IGV_LINK	667.168	20.9971	7.68879	34.3055	OK	860.042	25.6587	10.1224	41.1951	OK	OK	0.289258
ENSG0000003	-	-	ENSG0000003540	'VCL	-	10:73995192-74151063	-	-	IGV_LINK	1169.48	12.4131	1.56483	23.2614	OK	863.135	9.26996	0	18.7846	OK	OK	-0.42123
ENSG0000003	-	-	ENSG0000003549	'DEPDC1B	-	5:60596911-60700190	-	-	IGV_LINK	50.4255	1.44365	0	2.96596	OK	42.2802	1.39432	0	3.25031	OK	OK	-0.05016
ENSG0000003	-	-	ENSG0000003566	'DAPK2	-	15:63907035-64072033	-	-	IGV_LINK	73.6988	2.20907	0	5.53573	OK	157.777	4.79359	0	11.1332	OK	OK	1.11767
ENSG0000003	-	-	ENSG0000003568	'NSMAF	-	8:58583503-58659844	-	-	IGV_LINK	723.411	18.9649	2.20362	35.7262	OK	580.58	16.7048	1.30081	32.1088	OK	OK	-0.18307
ENSG0000003	-	-	ENSG0000003568	'ADSS	-	1:244352634-244452134	-	-	IGV_LINK	646.804	16.4898	5.12546	27.8542	OK	520.769	12.4242	3.66092	21.1876	OK	OK	-0.40842
ENSG0000003	-	-	ENSG0000003572	'STAP1	-	4:67558727-67607337	-	-	IGV_LINK	26.1825	1.23477	0	2.67529	OK	43.3114	1.9795	0	4.01556	OK	OK	0.680899
ENSG0000003	-	-	ENSG0000003586	'TIMP2	-	17:78852976-78925387	-	-	IGV_LINK	1733.86	30.2947	12.8848	47.7045	OK	2123.29	36.8406	15.8745	57.8068	OK	OK	0.282235

ENSG0000003	-	-	ENSG0000003592	'RFC1	-	4:39287455-39366375	-	-	IGV_LINK	1486.58	25.175	2.43438	47.9157	OK	1306.56	25.9868	1.4606	50.513	OK	OK	0.045784
ENSG0000003	-	-	ENSG0000003605	'TBC1D23	-	3:100260999-100325251	-	-	IGV_LINK	474.193	8.21657	2.42348	14.0097	OK	671.327	11.0151	4.33547	17.6948	OK	OK	0.422877
ENSG0000003	-	-	ENSG0000003625	'CUL3	-	2:224464681-224585397	-	-	IGV_LINK	741.836	19.4957	5.04494	33.9465	OK	969.351	26.7265	7.62835	45.8247	OK	OK	0.455116
ENSG0000003	-	-	ENSG0000003644	'MYOM2	-	8:2045039-2165552	-	-	IGV_LINK	209.46	13.7562	0	36.1582	OK	245.432	16.6231	3.39911	29.8471	OK	OK	0.273106
ENSG0000003	-	-	ENSG0000003647	'OTC	-	X:37349274-38688920	-	-	IGV_LINK	0	0	0	0	OK	0	0	0	0	OK	NOTEST	0
ENSG0000003	-	-	ENSG0000003653	'CYP46A1	-	14:99684303-99727301	-	-	IGV_LINK	24.243	0.925198	0	2.34686	OK	17.5308	0.54277	0	1.46659	OK	OK	-0.76942
ENSG0000003	-	-	ENSG0000003654	'ZZZ3	-	1:77562415-77683419	-	-	IGV_LINK	755.412	13.7924	0	27.682	OK	702.264	11.6494	1.35047	21.9483	OK	OK	-0.24362
ENSG0000003	-	-	ENSG0000003656	'SLC18A1	-	8:20144854-20183206	-	-	IGV_LINK	42.6677	1.54237	0	3.51617	OK	12.3747	0.554729	0	1.48483	OK	OK	-1.4753
ENSG0000003	-	-	ENSG0000003667	'USP2	-	11:119355214-119381726	-	-	IGV_LINK	40.7283	1.51149	0	3.71989	OK	89.7166	2.39779	0	5.27918	OK	OK	0.66574
ENSG0000003	-	-	ENSG0000003682	'CASR	-	3:122183682-122291629	-	-	IGV_LINK	86.3051	0.492558	0.014845	0.970272	OK	100.029	0.570948	0.025921	1.11598	OK	OK	0.213065
ENSG0000003	-	-	ENSG0000003704	'TUBG2	-	17:42659304-42667006	-	-	IGV_LINK	464.496	19.109	6.29534	31.9227	OK	484.676	20.0668	6.70323	33.4303	OK	OK	0.070555
ENSG0000003	-	-	ENSG0000003724	'RPL26L1	-	5:172954785-172969771	-	-	IGV_LINK	299.644	31.1602	9.47364	52.8467	OK	343.398	36.4267	10.2121	62.6413	OK	OK	0.225292
ENSG0000003	-	-	ENSG0000003728	'FLT4	-	5:180601505-180649624	-	-	IGV_LINK	680.744	7.20511	2.12267	12.2875	OK	730.107	7.88616	2.03468	13.7376	OK	OK	0.130303
ENSG0000003	-	-	ENSG0000003747	'NSUN2	-	5:6599238-6633291	-	-	IGV_LINK	1103.54	35.0454	7.33097	62.7597	OK	1242.63	37.2729	9.16775	65.378	OK	OK	0.088902
ENSG0000003	-	-	ENSG0000003763	'FBXO42	-	1:16246838-16352454	-	-	IGV_LINK	624.5	10.644	0.864788	20.4232	OK	742.482	14.4207	0	30.644	OK	OK	0.438101
ENSG0000003	-	-	ENSG0000003774	'MFAP3	-	5:153990127-154445850	-	-	IGV_LINK	354.918	16.0366	0	42.9938	OK	455.801	15.2298	0	40.0538	OK	OK	-0.07447
ENSG0000003	-	-	ENSG0000003775	'MRI1	-	19:13764531-13778462	-	-	IGV_LINK	831.05	36.4162	1.32569	71.5067	OK	814.668	35.1248	0.8688	69.3807	OK	OK	-0.05209
ENSG0000003	-	-	ENSG0000003789	'METTL1	-	12:57762333-57818704	-	-	IGV_LINK	204.611	14.838	0	37.0012	OK	468.176	32.3308	0	80.7374	OK	OK	1.12362
ENSG0000003	-	-	ENSG0000003796	'HOXC8	-	12:53981508-54056030	-	-	IGV_LINK	54.3043	1.78292	0	6.82625	OK	42.2802	1.38908	0	5.26029	OK	OK	-0.36012
ENSG0000003	-	-	ENSG0000003800	'AGA	-	4:177430769-177442503	-	-	IGV_LINK	256.006	16.667	2.14283	31.1912	OK	258.837	16.4892	1.02425	31.9541	OK	OK	-0.01548
ENSG0000003	-	-	ENSG0000003821	'PI4K2B	-	4:25160640-25279092	-	-	IGV_LINK	161.016	2.69463	0.208795	5.18047	OK	237.196	3.97088	0.686291	7.25546	OK	OK	0.559367
ENSG0000003	-	-	ENSG0000003821	'BOD1L1	-	4:13568737-13627723	-	-	IGV_LINK	1557.37	20.9577	0.96138	40.9541	OK	1072.47	17.8929	0	36.98	OK	OK	-0.22809
ENSG0000003	-	-	ENSG0000003827	'MAT2B	-	5:163503113-163519336	-	-	IGV_LINK	1996.65	62.8171	21.2321	104.402	OK	1861.36	61.2582	21.3966	101.12	OK	OK	-0.03625
ENSG0000003	-	-	ENSG0000003829	'TLL1	-	4:165873257-166103895	-	-	IGV_LINK	224.005	3.24239	0	8.00128	OK	24.7494	0.436761	0	1.35957	OK	OK	-2.89214
ENSG0000003	-	-	ENSG0000003835	'EDC4	-	16:67873022-67888855	-	-	IGV_LINK	1425.86	33.898	1.47251	66.3235	OK	1349.18	29.4707	3.58584	55.3556	OK	OK	-0.20192
ENSG0000003	-	-	ENSG0000003838	'TRIO	-	5:14143701-14532128	-	-	IGV_LINK	2052.9	30.4019	0	67.8104	OK	2690.47	46.2706	0	98.2103	OK	OK	0.605934
ENSG0000003	-	-	ENSG0000003842	'VCAN	-	5:83471464-83582303	-	-	IGV_LINK	661.349	7.14402	0	15.6193	OK	617.704	11.27	0	24.0943	OK	OK	0.65768
ENSG0000003	-	-	ENSG0000003853	'CLEC16A	-	16:10944487-11182189	-	-	IGV_LINK	487.769	5.85677	1.73502	9.97851	OK	578.517	9.38862	1.69572	17.0815	OK	OK	0.680808
ENSG0000003	-	-	ENSG0000003894	'MSR1	-	8:16107877-16567490	-	-	IGV_LINK	1509.85	36.6677	12.6701	60.6653	OK	2075.86	51.6065	16.9151	86.2979	OK	OK	0.493043
ENSG0000003	-	-	ENSG0000003906	'CDH1	-	16:68737224-68835548	-	-	IGV_LINK	1758.1	26.8241	9.12754	44.5207	OK	678.546	10.581	0	23.2898	OK	OK	-1.34205
ENSG0000003	-	-	ENSG0000003912	'SKIV2L2	-	5:55307759-55535050	-	-	IGV_LINK	968.751	31.6417	0	76.2647	OK	894.072	23.8796	0	57.9026	OK	OK	-0.40605
ENSG0000003	-	-	ENSG0000003913	'DNAH5	-	5:13690330-13944543	-	-	IGV_LINK	299.644	1.09264	0.294426	1.89085	OK	246.463	0.934624	0.128193	1.74106	OK	OK	-0.22536
ENSG0000003	-	-	ENSG0000003931	'ZFYVE16	-	5:80408012-80479350	-	-	IGV_LINK	794.201	11.299	0	23.9576	OK	696.077	9.41696	0	19.4925	OK	OK	-0.26287
ENSG0000003	-	-	ENSG0000003952	'RIPOR1	-	16:67517861-67546788	-	-	IGV_LINK	1954.96	38.7943	2.50603	75.0826	OK	2162.48	53.1914	0	126.283	OK	OK	0.455348
ENSG0000003	-	-	ENSG0000003953	'C6	-	5:41142233-41261438	-	-	IGV_LINK	18.4247	0.307338	0	0.96581	OK	47.4363	0.929595	0	2.32251	OK	OK	1.59678
ENSG0000003	-	-	ENSG0000003956	'RAI14	-	5:34656236-34832627	-	-	IGV_LINK	690.441	10.6738	0.156759	21.1908	OK	707.42	10.0025	1.1795	18.8255	OK	OK	-0.09371
ENSG0000003	-	-	ENSG0000003960	'SOX30	-	5:157625678-157671480	-	-	IGV_LINK	14.5458	0.26512	0	0.616261	OK	18.562	0.338425	0	0.725338	OK	NOTEST	0.352189
ENSG0000003	-	-	ENSG0000003965	'PNKP	-	19:49818278-49888749	-	-	IGV_LINK	745.506	44.6993	0	139.094	OK	776.512	50.7544	0	172.467	OK	OK	0.18328
ENSG0000003	-	-	ENSG0000003998	'BEST2	-	19:12751701-12758458	-	-	IGV_LINK	0	0	0	0	OK	0	0	0	0	OK	NOTEST	0
ENSG0000004	-	-	ENSG0000004019	'PHLPP2	-	16:71623707-71724701	-	-	IGV_LINK	273.461	2.17464	0.346063	4.00322	OK	263.994	2.13074	0.265638	3.99585	OK	OK	-0.02942
ENSG0000004	-	-	ENSG0000004027	'SPDL1	-	5:169583633-169604778	-	-	IGV_LINK	188.126	5.22328	0.360155	10.0864	OK	166.027	5.27322	0	11.0012	OK	OK	0.013726
ENSG0000004	-	-	ENSG0000004034	'STAU2	-	8:73420003-73878910	-	-	IGV_LINK	771.924	30.9723	0	65.5228	OK	741.765	27.6452	1.12924	54.1613	OK	OK	-0.16395
ENSG0000004	-	-	ENSG0000004048	'PQLC2	-	1:19312325-19329300	-	-	IGV_LINK	225.945	8.69762	2.04194	15.3533	OK	257.806	9.79172	2.32846	17.255	OK	OK	0.170941
ENSG0000004	-	-	ENSG0000004053	'CTNS	-	17:3636467-3661542	-	-	IGV_LINK	314.189	7.2724	1.40717	13.1376	OK	409.396	9.27885	2.38042	16.1773	OK	OK	0.351515
ENSG0000004	-	-	ENSG0000004060	'RTN4R	-	22:20241414-20283246	-	-	IGV_LINK	19.3944	0.615288	0	1.46354	OK	36.0929	1.26863	0	2.65891	OK	OK	1.04394
ENSG0000004	-	-	ENSG0000004063	'PHF23	-	17:7235027-7239722	-	-	IGV_LINK	429.586	15.5683	5.2426	25.894	OK	460.958	18.044	4.37161	31.7163	OK	OK	0.212904
ENSG0000004	-	-	ENSG0000004073	'CDH10	-	5:24487099-24644978	-	-	IGV_LINK	0	0	0	0	OK	0	0	0	0	OK	NOTEST	0
ENSG0000004	-	-	ENSG0000004093	'INPP4A	-	2:98444853-98594390	-	-	IGV_LINK	640.985	7.62277	0	15.746	OK	507.363	5.40194	0.222004	10.5819	OK	OK	-0.49684
ENSG0000004	-	-	ENSG0000004135	'RAB27B	-	18:54717859-54898083	-	-	IGV_LINK	29.0916	0.40078	0	1.43479	OK	94.8727	1.41155	0	4.74762	OK	OK	1.81639
ENSG0000004	-	-	ENSG0000004135	'PSMA4	-	15:78540404-78552419	-	-	IGV_LINK	1458.46	99.5861	40.2918	158.88	OK	1245.72	90.2264	29.6114	150.841	OK	OK	-0.1424
ENSG0000004	-	-	ENSG0000004151	'MYO16	-	13:108596151-109208007	-	-	IGV_LINK	14.5458	0.148037	0	0.507595	OK	18.562	0.212868	0	0.803657	OK	NOTEST	0.524006
ENSG0000004	-	-	ENSG0000004180	'LSG1	-	3:194584010-194672477	-	-	IGV_LINK	753.473	19.4585	0	43.1662	OK	977.601	24.6417	0	56.6981	OK	OK	0.340701
ENSG0000004	-	-	ENSG0000004188	'PARP3	-	3:51942344-51948867	-	-	IGV_LINK	580.863	19.8697	4.72712	35.0123	OK	488.801	16.3028	3.30961	29.2961	OK	OK	-0.28545
ENSG0000004	-	-	ENSG0000004198	'TNC	-	9:115019577-115118257	-	-	IGV_LINK	561.468	7.19332	0	16.5302	OK	134.059	1.84701	0	5.23728	OK	OK	-1.96147
ENSG0000004	-	-	ENSG0000004198	'THAP3	-	1:6624865-6701924	-	-	IGV_LINK	248.248	13.2634	0	31.5683	OK	230.994	12.8591	0	37.0988	OK	OK	-0.04466
ENSG0000004	-	-	ENSG0000004206	'RIPOR3	-	20:50586107-50691528	-	-	IGV_LINK	4287.13	98.9551	16.9525	180.958	OK	1936.64	41.7007	8.12095	75.2804	OK	OK	-1.2467
ENSG0000004	-	-	ENSG0000004208	'TDP1	-	14:89954938-90044768	-	-	IGV_LINK	241.46	5.45212	1.19536	9.70888	OK	274.306	7.48816	1.37256	13.6038	OK	OK	0.457795
ENSG0000004	-	-	ENSG0000004228	'AIFM2	-	10:70052795-70132934	-	-	IGV_LINK	395.646	7.50926	1.98125	13.0373	OK	412.49	8.30917	2.09806	14.5203	OK	OK	0.146034
ENSG0000004	-	-	ENSG0000004230	'C2orf83	-	2:227610089-227648606	-	-	IGV_LINK	0	0	0	0	OK	0	0	0	0	OK	NOTEST	0
ENSG0000004	-	-	ENSG0000004231	'SPATA7	-	14:88384923-88554733	-	-	IGV_LINK	192.974	6.21071	0.131746	12.2897	OK	197.995	6.45446	0	13.1695	OK	OK	0.055537
ENSG0000004	-	-	ENSG0000004242	'MED17	-	11:93661638-93814963	-	-	IGV_LINK	530.868	11.1227	0	31.7493	OK	450.468	9.75461	0	32.428	OK	OK	-0.18935
ENSG0000004	-	-	ENSG0000004244	'RETSAT	-	2:85342087-85391752	-	-	IGV_LINK	1537.01	32.2346	6.32301	58.1462	OK	2629.62	55.1797	14.0648	96.2945	OK	OK	0.775527
ENSG0000004	-	-	ENSG0000004249	'CAPG	-	2:85394747-85418432	-	-	IGV_LINK	4163.98	273.076	89.9651	456.186	OK	4006.31	254.52	89.539	419.502	OK	OK	-0.10152
ENSG0000004	-	-	ENSG0000004275	'AP2S1	-	19:46838135-46850992	-	-	IGV_LINK	734.078	65.275	23.1262	107.424	OK	906.447	77.0715	30.5545	123.589	OK	OK	0.239668
ENSG0000004	-	-	ENSG0000004278	'USH2A	-	1:215622893-216423396	-	-	IGV_LINK	9.69721	0.029208	0	0.29478	OK	43.3114	0.130461	0	0.422017	OK	NOTEST	2.1592
ENSG0000004	-	-	ENSG0000004281	'ZPBP	-	7:49773660-50159830	-	-	IGV_LINK	0.969721	0.059238	0	0.524662	OK	1.03122	0.055916	0	0.515343	OK	NOTEST	-0.08325
ENSG0000004	-	-	ENSG0000004283	'TG	-	8:132866957-133134903	-	-	IGV_LINK	27.1522	0.752979	0	6.71903	OK	107.247	2.20386	0	8.6938	OK	OK	1.54935
ENSG0000004	-	-	ENSG0000004298	'ADAM28	-	8:24294039-24912073	-	-	IGV_LINK	1043.42	34.497	3.17861	65.8155	OK	962.133	29.5536	0	62.7595	OK	OK	-0.22314
ENSG0000004	-	-	ENSG0000004303	'BARX2	-	11:129375939-129452279	-	-	IGV_LINK	962.932	32.9116	14.0095	51.8137	OK	632.141	21.7044	8.27772	35.1312	OK	OK	-0.60061
ENSG0000004	-	-	ENSG0000004309	'DCUN1D1	-	3:182938073-182985953	-	-	IGV_LINK	393.707	9.97353	0.344397	19.6027	OK	389.803	9.74922	0	22.0419	OK	OK	-0.03282
ENSG0000004	-	-	ENSG0000004314	'JADE2	-	5:134524311-134583230	-	-	IGV_LINK	2027.69	25.3975	6.32425	44.4707	OK	1113.72	17.6296	1.97672	33.2824	OK	OK	-0.52669
ENSG0000004	-	-	ENSG0000004335	'ZIC2	-	13:99981771-99986773	-	-	IGV_LINK	0	0	0	0	OK	10.3122	0.290051	0	1.4011	OK	NOTEST	inf
ENSG0000004	-	-	ENSG0000004346	'LCP2	-	5:170232446-170298227	-	-	IGV_LINK	1762.95	62.5869	17.8188	107.355	OK	1328.22	46.2687	15.5672	76.9702	OK	OK	-0.43582
ENSG0000004	-	-	ENSG0000004351	'TRIT1	-	1:39841021-39883511	-	-	IGV_LINK	411.161	17.2449	3.21717	31.2727	OK	409.396	18.2907	3.09616	33.4852	OK	OK	0.084935
ENSG0000004	-	-	ENSG0000004359	'ADRB1	-	10:114044055-114046908	-	-	IGV_LINK	8.72748	0.18349	0	0.417608	OK	1.03122	0.02169	0	0.081182	OK	NOTEST	-3.08059
ENSG0000004	-	-	ENSG0000004401	'GUCA2B	-	1:42153420-42155824	-	-	IGV_LINK	2.90916	0.386236	0	1.03665	OK	0	0	0	0	OK	NOTEST	'-inf
ENSG0000004	-	-	ENSG0000004409	'CUL7	-	6:43037616-43075099	-	-	IGV_LINK	1515.67	16.5798	1.56432	31.5952	OK	951.821	10.8075	0.025115	21.5898	OK	OK	-0.61739
ENSG0000004	-	-	ENSG0000004411	'CTNNA1	-	5:138610966-138935034	-	-	IGV_LINK	6555.31	125.615	28.3131	222.918	OK	8762.32	167.877	25.7275	310.027	OK	OK	0.418393
ENSG0000004	-	-	ENSG0000004444	'PHKA2	-	X:18890295-18984598	-	-	IGV_LINK	4933.94	111.374	0	240.079	OK	5613.99	112.54	0	246.475	OK	OK	0.015029
ENSG0000004	-	-	ENSG0000004445	'CNTLN	-	9:17134981-17503923	-	-	IGV_LINK	241.46	2.74514	0.549209	4.94107	OK	221.713	3.05318	0	6.69447	OK	OK	0.153434
ENSG0000004	-	-	ENSG0000004452	'EPHA3	-	3:89107523-89482134	-	-	IGV_LINK	83.396	1.16338	0.03167	2.29509	OK	312.461	3.96995	0.950008	6.98989	OK	OK	1.7708
ENSG0000004	-	-	ENSG0000004457	'HSPA5	-	9:125234852-125241330	-	-	IGV_LINK	4992.12	75.3138	30.8193	119.808	OK	7107.2	107.256	40.5398	173.972	OK	OK	0.510073
ENSG0000004	-	-	ENSG0000004660	'DSG2	-	18:31498042-31556911	-	-	IGV_LINK	1231.55	12.2655	5.33688	19.1942	OK	947.696	9.44046	3.9429	14.938	OK	OK	-0.37768
ENSG0000004	-	-	ENSG0000004664	'GEMIN8	-	X:14008278-14029893	-	-	IGV_LINK	334.554	19.8626	6.36563	33.3596	OK	353.71	21.6502	6.58745	36.7129	OK	OK	0.124321
ENSG0000004	-	-	ENSG0000004665	'OFD1	-	X:13734744-13769353	-	-	IGV_LINK	1504.04	30.8054	6.16137	55.4495	OK	1411.75	29.0026	6.29205	51.7132	OK	OK	-0.087
ENSG0000004	-	-	ENSG0000004665	'GPM6B	-	X:13771030-13938638	-	-	IGV_LINK	189.095	4.35298	0.096662	8.60929	OK	71.1545	1.62273	0	3.75003	OK	OK	-1.42358
ENSG0000004	-	-	ENSG0000004677	'MAGEC2	-	X:142202344-142205290	-	-	IGV_LINK	0	0	0	0	OK	0	0	0	0	OK	NOTEST	0
ENSG0000004	-	-	ENSG0000004688	'PREX2	-	8:67952117-68237030	-	-	IGV_LINK	642.925	5.05167	0	14.1587	OK	1752.05	18.1088	0	49.4594	OK	OK	1.84186
ENSG0000004	-	-	ENSG0000004705	'WDR37	-	10:1049537-1132297	-	-	IGV_LINK	597.348	8.35345	2.94549	13.7614	OK	403.209	5.67106	1.41049	9.93163	OK	OK	-0.55875
ENSG0000004	-	-	ENSG0000004718	'YTHDC2	-	5:113513682-113595285	-	-	IGV_LINK	885.355	9.85432	0.947241	18.7614	OK	857.979	10.255	0.314823	20.1952	OK	OK	0.057497
ENSG0000004	-	-	ENSG0000004723	'CTPS2	-	X:16588002-16712936	-	-	IGV_LINK	347.16	8.23555	0	17.9146	OK	291.837	4.7941	0.494359	9.09385	OK	OK	-0.78061
ENSG0000004	-	-	ENSG0000004724	'ATP6V1H	-	8:53715556-53843558	-	-	IGV_LINK	563.408	18.9742	5.84545	32.1029	OK	700.202	22.3489	7.65693	37.0409	OK	OK	0.236168
ENSG0000004	-	-	ENSG0000004731	'POLR2B	-	4:56907875-57205510	-	-	IGV_LINK	1566.1	36.8307	0	177.707	OK	1467.43	32.7221	0	175.087	OK	OK	-0.17065
ENSG0000004	-	-	ENSG0000004734	'FAM214A	-	15:52577841-52709817	-	-	IGV_LINK	619.651	16.8056	3.93285	29.6784	OK	797.137	20.8138	4.6583	36.9692	OK	OK	0.308591
ENSG0000004	-	-	ENSG0000004736	'ARAP2	-	4:35948220-36274220	-	-	IGV_LINK	776.746	8.92433	0.393245	17.4554	OK	436.208	4.52033	0.158563	8.8821	OK	OK	-0.98132
ENSG0000004	-	-	ENSG0000004741	'TPR	-	1:186296278-186375693	-	-	IGV_LINK	2857.77	22.876	0	52.0694	OK	2210.95	21.3813	0	51.2805	OK	OK	-0.09748
ENSG0000004	-	-	ENSG0000004745	'CP	-	3:149129583-149222055	-	-	IGV_LINK	14231.6	334.002	0	686.115	OK	41722.3	994.664	0	2368.25	OK	OK	1.57435
ENSG0000004	-	-	ENSG0000004757	'KIAA0556	-	16:27550132-27780369	-	-	IGV_LINK	671.047	9.86979	0	21.1078	OK	840.448	12.1109	0	26.8679	OK	OK	0.295219
ENSG0000004	-	-	ENSG0000004757	'DTNBP1	-	6:15522800-15663058	-	-	IGV_LINK	401.464	20.099	5.70584	34.4921	OK	229.963	10.4815	2.87032	18.0927	OK	OK	-0.93928
ENSG0000004	-	-	ENSG0000004759	'XK	-	X:37349274-38688920	-	-	IGV_LINK	5.81832	0.065143	0	1.11041	OK	21.6557	0.242515	0	2.34142	OK	NOTEST	1.8964
ENSG0000004	-	-	ENSG0000004761	'ANO2	-	12:5531868-5946232	-	-	IGV_LINK	30.0613	0.769926	0	1.97399	OK	53.6237	2.71885	0	6.8083	OK	OK	1.8202
ENSG0000004	-	-	ENSG0000004762	'C12orf4	-	12:4487727-4538508	-	-	IGV_LINK	393.707	7.4308	1.32302	13.5386	OK	385.678	8.12752	0.962941	15.2921	OK	OK	0.129297
ENSG0000004	-	-	ENSG0000004763	'SCML1	-	X:17737448-17754988	-	-	IGV_LINK	316.129	6.99937	1.27254	12.7262	OK	283.587	6.01186	0.784227	11.2395	OK	OK	-0.21942
ENSG0000004	-	-	ENSG0000004764	'WWC3	-	X:10015561-10144478	-	-	IGV_LINK	976.509	9.07576	1.75981	16.3917	OK	913.665	8.41514	1.73836	15.0919	OK	OK	-0.10903
ENSG0000004	-	-	ENSG0000004764	'ARHGAP6	-	X:11137542-11665701	-	-	IGV_LINK	307.401	4.21447	1.07353	7.35542	OK	177.371	2.66488	0.255506	5.07425	OK	OK	-0.66128
ENSG0000004	-	-	ENSG0000004766	'FAM184B	-	4:17614630-17781512	-	-	IGV_LINK	362.675	3.16905	0	7.24719	OK	271.212	2.37027	0	5.6854	OK	OK	-0.419
ENSG0000004	-	-	ENSG0000004784	'MAP4	-	3:47850689-48089272	-	-	IGV_LINK	3660.69	45.4078	0	94.255	OK	3900.09	45.4813	9.72008	81.2425	OK	OK	0.002332
ENSG0000004	-	-	ENSG0000004793	'GOPC	-	6:117288299-117602542	-	-	IGV_LINK	648.743	8.32478	2.6062	14.0434	OK	381.553	4.86889	1.39324	8.34454	OK	OK	-0.77382
ENSG0000004	-	-	ENSG0000004793	'ROS1	-	6:117288299-117602542	-	-	IGV_LINK	0	0	0	0	OK	0	0	0	0	OK	NOTEST	0
ENSG0000004	-	-	ENSG0000004802	'USP28	-	11:113797873-113875570	-	-	IGV_LINK	415.04	11.3446	1.08569	21.6035	OK	401.147	11.5814	0.530159	22.6327	OK	OK	0.029807
ENSG0000004	-	-	ENSG0000004805	'HDAC9	-	7:18086948-19002416	-	-	IGV_LINK	218.187	6.90628	0	15.1308	OK	113.435	3.44364	0	8.20948	OK	OK	-1.00398
ENSG0000004	-	-	ENSG0000004814	'TSPAN17	-	5:176647386-176659054	-	-	IGV_LINK	776.746	18.0204	5.73307	30.3077	OK	1034.32	23.8227	8.4396	39.2058	OK	OK	0.402707
ENSG0000004	-	-	ENSG0000004816	'NOP16	-	5:176365467-176416569	-	-	IGV_LINK	422.798	25.91	0	65.7546	OK	465.082	29.444	0	80.5947	OK	OK	0.184467
ENSG0000004	-	-	ENSG0000004834	'CC2D2A	-	4:15469864-15601557	-	-	IGV_LINK	475.163	12.9809	0.383729	25.5781	OK	484.676	14.2559	1.03962	27.4723	OK	OK	0.13517
ENSG0000004	-	-	ENSG0000004839	'RRM2B	-	8:102204501-102239118	-	-	IGV_LINK	918.325	16.2447	1.23942	31.2499	OK	1440.62	23.2905	4.68955	41.8915	OK	OK	0.519777
ENSG0000004	-	-	ENSG0000004840	'ZNF800	-	7:127346789-127431924	-	-	IGV_LINK	280.249	8.55047	1.17674	15.9242	OK	289.774	8.90423	1.01846	16.79	OK	OK	0.058488
ENSG0000004	-	-	ENSG0000004846	'TNFRSF17	-	16:11927372-11976643	-	-	IGV_LINK	15.5155	1.06447	0	3.63065	OK	13.4059	0.920974	0	4.37771	OK	OK	-0.2089
ENSG0000004	-	-	ENSG0000004847	'SNX29	-	16:11976736-12611044	-	-	IGV_LINK	2767.58	22.9326	1.39031	44.4749	OK	2596.62	21.2943	1.10768	41.4809	OK	OK	-0.10693
ENSG0000004	-	-	ENSG0000004854	'LMO3	-	12:16347141-16610594	-	-	IGV_LINK	0	0	0	0	OK	17.5308	0.874199	0	15.1181	OK	OK	inf
ENSG0000004	-	-	ENSG0000004854	'MRPS10	-	6:42206800-42217865	-	-	IGV_LINK	314.189	9.13884	3.0625	15.2152	OK	310.399	9.03394	3.00661	15.0613	OK	OK	-0.01666
ENSG0000004	-	-	ENSG0000004854	'GUCA1A	-	6:42155405-42180056	-	-	IGV_LINK	0	0	0	0	OK	0	0	0	0	OK	NOTEST	0
ENSG0000004	-	-	ENSG0000004864	'RSF1	-	11:77659995-77821017	-	-	IGV_LINK	785.307	12.0384	0	27.6135	OK	674.537	11.5922	0	27.1224	OK	OK	-0.05449
ENSG0000004	-	-	ENSG0000004870	'VPS13D	-	1:12230066-12512047	-	-	IGV_LINK	1187.91	9.72328	0	20.6469	OK	859.01	8.18803	0	17.2838	OK	OK	-0.24793
ENSG0000004	-	-	ENSG0000004874	'CELF2	-	10:10798396-11336675	-	-	IGV_LINK	1322.7	14.5616	3.79145	25.3317	OK	1028.13	11.7004	2.8643	20.5365	OK	OK	-0.31561
ENSG0000004	-	-	ENSG0000004882	'FAM120A	-	9:93446493-93566107	-	-	IGV_LINK	2530.97	41.4536	7.52214	75.385	OK	2797.71	41.0303	9.82806	72.2325	OK	OK	-0.01481
ENSG0000004	-	-	ENSG0000004899	'R3HDM1	-	2:135531454-135725270	-	-	IGV_LINK	394.676	9.50073	0	19.4902	OK	376.397	7.75318	0	16.2342	OK	OK	-0.29325
ENSG0000004	-	-	ENSG0000004908	'COL9A2	-	1:40300486-40317816	-	-	IGV_LINK	458.678	10.2971	2.94773	17.6464	OK	146.434	3.20935	0.221586	6.19712	OK	OK	-1.68188
ENSG0000004	-	-	ENSG0000004913	'KITLG	-	12:88492792-88598218	-	-	IGV_LINK	171.641	2.92167	0.23224	5.61109	OK	282.556	4.50111	0.858347	8.14388	OK	OK	0.623491
ENSG0000004	-	-	ENSG0000004916	'ERCC8	-	5:60873830-60945073	-	-	IGV_LINK	70.2442	2.74795	0	6.34347	OK	84.3971	2.77439	0	5.82293	OK	OK	0.013814
ENSG0000004	-	-	ENSG0000004919	'ADAMTS6	-	5:65148735-65481920	-	-	IGV_LINK	34.9099	0.468579	0	1.55117	OK	35.0616	0.412169	0	1.14074	OK	NOTEST	-0.18506
ENSG0000004	-	-	ENSG0000004923	'H6PD	-	1:9234774-9271337	-	-	IGV_LINK	2439.82	15.3551	6.71478	23.9954	OK	6331.72	41.308	15.3723	67.2436	OK	OK	1.4277
ENSG0000004	-	-	ENSG0000004924	'VAMP3	-	1:7771268-7781432	-	-	IGV_LINK	1432.28	43.6822	11.5421	75.8224	OK	1817.02	53.4466	17.9342	88.9591	OK	OK	0.291052

ENSG0000004	-	-	ENSG0000004924	'PER3	-	1:7784319-7853512	-	-	IGV_LINK	904.749	13.7534	0	31.0326	OK	569.236	7.45959	0	16.8102	OK	OK	-0.88262
ENSG0000004	-	-	ENSG0000004924	'UTS2	-	1:7784319-7853512	-	-	IGV_LINK	273.461	16.3715	0	41.089	OK	163.965	9.68112	0	24.4546	OK	OK	-0.75794
ENSG0000004	-	-	ENSG0000004924	'TNFRSF9	-	1:7915893-7943165	-	-	IGV_LINK	305.462	6.01402	0	12.2098	OK	128.903	2.52419	0	5.30327	OK	OK	-1.25251
ENSG0000004	-	-	ENSG0000004928	'EPN3	-	17:50532542-50555852	-	-	IGV_LINK	0.969721	0.141037	0	3.9052	OK	13.4059	1.44746	0	15.9087	OK	OK	3.35938
ENSG0000004	-	-	ENSG0000004932	'LTBP1	-	2:32946971-33399509	-	-	IGV_LINK	670.077	7.96794	1.60778	14.3281	OK	415.584	4.44339	1.48913	7.39766	OK	OK	-0.84255
ENSG0000004	-	-	ENSG0000004944	'RCN1	-	11:31509699-32105755	-	-	IGV_LINK	2745.28	79.8528	22.3632	137.342	OK	3939.28	114.747	32.9009	196.593	OK	OK	0.523041
ENSG0000004	-	-	ENSG0000004954	'ELN	-	7:74027788-74069907	-	-	IGV_LINK	56.2438	1.75422	0	4.79347	OK	100.029	2.17989	0	5.14581	OK	OK	0.313425
ENSG0000004	-	-	ENSG0000004954	'RFC2	-	7:74231498-74254458	-	-	IGV_LINK	474.193	17.7919	6.59373	28.9901	OK	555.83	22.5583	8.1674	36.9493	OK	OK	0.342438
ENSG0000004	-	-	ENSG0000004961	'ARID1B	-	6:156776359-157210779	-	-	IGV_LINK	1370.22	16.495	0.444058	32.546	OK	944.602	12.8848	0	26.5883	OK	OK	-0.35636
ENSG0000004	-	-	ENSG0000004965	'CLPTM1L	-	5:1317743-1345099	-	-	IGV_LINK	1821.14	82.2946	21.2523	143.337	OK	2769.87	116.148	28.7321	203.565	OK	OK	0.4971
ENSG0000004	-	-	ENSG0000004975	'NEDD4L	-	18:58044366-58401540	-	-	IGV_LINK	1614.58	48.7848	0	187.869	OK	917.79	20.7982	0	46.1291	OK	OK	-1.22997
ENSG0000004	-	-	ENSG0000004976	'FOXP3	-	X:49235466-49264826	-	-	IGV_LINK	210.429	5.57801	0	11.3753	OK	182.527	4.75809	0	9.87679	OK	OK	-0.22937
ENSG0000004	-	-	ENSG0000004976	'PPP1R3F	-	X:49269842-49301461	-	-	IGV_LINK	104.73	3.51006	0	9.79387	OK	57.7486	1.22626	0	3.48412	OK	OK	-1.51723
ENSG0000004	-	-	ENSG0000004986	'HEXB	-	5:74627405-74867854	-	-	IGV_LINK	1788.16	88.5311	0	194.669	OK	2576	118.731	0.592417	236.869	OK	OK	0.423439
ENSG0000004	-	-	ENSG0000004988	'PTCD2	-	5:72219408-72368395	-	-	IGV_LINK	296.734	2.97618	0	8.43552	OK	280.493	4.81059	0	15.363	OK	OK	0.692753
ENSG0000005	-	-	ENSG0000005003	'NEXMIF	-	X:74732848-74925485	-	-	IGV_LINK	2.90916	0.015686	0	0.23922	OK	0	0	0	0	OK	NOTEST	'-inf
ENSG0000005	-	-	ENSG0000005013	'JKAMP	-	14:59429021-59576831	-	-	IGV_LINK	529.467	22.4522	3.86326	41.0411	OK	613.579	25.2219	5.63869	44.805	OK	OK	0.167819
ENSG0000005	-	-	ENSG0000005016	'DKK3	-	11:11963105-12009769	-	-	IGV_LINK	352.009	10.9674	1.50833	20.4265	OK	225.838	5.8361	0.850624	10.8216	OK	OK	-0.91015
ENSG0000005	-	-	ENSG0000005032	'ARHGEF5	-	7:144186082-144380632	-	-	IGV_LINK	303.523	4.7438	0	10.1812	OK	259.869	4.44919	0	10.0224	OK	OK	-0.0925
ENSG0000005	-	-	ENSG0000005034	'NFE2L3	-	7:26152239-26187125	-	-	IGV_LINK	803.898	16.5479	5.61287	27.4829	OK	547.58	11.2781	3.9277	18.6285	OK	OK	-0.55312
ENSG0000005	-	-	ENSG0000005039	'MCUR1	-	6:13786556-13814568	-	-	IGV_LINK	444.132	7.80301	0	16.6394	OK	196.964	4.35146	0	9.95127	OK	OK	-0.84253
ENSG0000005	-	-	ENSG0000005040	'LIMA1	-	12:50175787-50283546	-	-	IGV_LINK	2417.51	48.4044	10.764	86.0448	OK	1507.65	28.8946	7.69525	50.094	OK	OK	-0.74434
ENSG0000005	-	-	ENSG0000005042	'LETMD1	-	12:51047961-51060424	-	-	IGV_LINK	1790.1	77.5644	24.33	130.799	OK	1819.08	77.7637	24.8042	130.723	OK	OK	0.003703
ENSG0000005	-	-	ENSG0000005043	'SLC4A8	-	12:51351246-51515763	-	-	IGV_LINK	157.095	2.44408	0	11.691	OK	85.5917	0.858952	0	4.13871	OK	OK	-1.50864
ENSG0000005	-	-	ENSG0000005055	'LAMC3	-	9:131009081-131094473	-	-	IGV_LINK	672.986	10.4378	0.008762	20.8668	OK	71.1545	1.28702	0	3.34941	OK	OK	-3.01971
ENSG0000005	-	-	ENSG0000005062	'PTGER3	-	1:70852352-71047808	-	-	IGV_LINK	539.165	11.6968	1.26716	22.1265	OK	2287.26	52.2895	17.8641	86.7148	OK	OK	2.1604
ENSG0000005	-	-	ENSG0000005073	'TNIP3	-	4:121131407-121227466	-	-	IGV_LINK	32.9705	1.14113	0	2.49735	OK	20.6245	0.713035	0	1.78525	OK	OK	-0.67842
ENSG0000005	-	-	ENSG0000005074	'MAPK9	-	5:180233142-180292099	-	-	IGV_LINK	792.262	19.5146	2.94673	36.0825	OK	852.823	20.7385	3.4924	37.9845	OK	OK	0.087755
ENSG0000005	-	-	ENSG0000005076	'COL23A1	-	5:178237617-178590555	-	-	IGV_LINK	6269.24	148.422	56.0929	240.751	OK	2729.65	63.6961	26.3817	101.01	OK	OK	-1.22043
ENSG0000005	-	-	ENSG0000005082	'BCAR1	-	16:75226073-75268053	-	-	IGV_LINK	601.227	12.2702	3.36584	21.1745	OK	1071.44	24.6174	5.73687	43.498	OK	OK	1.00453
ENSG0000005	-	-	ENSG0000005100	'FAM160A2	-	11:6211334-6234711	-	-	IGV_LINK	1435.19	40.7405	13.1792	68.3017	OK	1232.31	33.7625	9.13638	58.3887	OK	OK	-0.27104
ENSG0000005	-	-	ENSG0000005110	'HERPUD1	-	16:56932047-56944863	-	-	IGV_LINK	3246.62	153.69	38.5381	268.842	OK	3091.61	139.072	42.1443	235.999	OK	OK	-0.14419
ENSG0000005	-	-	ENSG0000005112	'HOMER3	-	19:18899513-18946831	-	-	IGV_LINK	482.921	24.9696	0	63.1855	OK	456.833	23.4201	0	62.1552	OK	OK	-0.09243
ENSG0000005	-	-	ENSG0000005118	'RAD51	-	15:40686723-40732339	-	-	IGV_LINK	58.1832	2.59933	0	7.476	OK	55.6861	2.70796	0	7.48921	OK	OK	0.059067
ENSG0000005	-	-	ENSG0000005134	'POLQ	-	3:121431426-121546641	-	-	IGV_LINK	69.8199	0.87067	0	2.79394	OK	50.53	0.456595	0	1.44628	OK	OK	-0.93121
ENSG0000005	-	-	ENSG0000005138	'PIK3CB	-	3:138652698-138834938	-	-	IGV_LINK	915.416	18.5177	1.66766	35.3676	OK	853.854	14.2957	2.4187	26.1727	OK	OK	-0.37332
ENSG0000005	-	-	ENSG0000005152	'CYBA	-	16:88643282-88651152	-	-	IGV_LINK	3136.08	270.069	107.702	432.436	OK	4321.86	374.442	151.606	597.279	OK	OK	0.471418
ENSG0000005	-	-	ENSG0000005159	'THOC3	-	5:175906938-176034680	-	-	IGV_LINK	178.429	15.6142	1.14856	30.0798	OK	262.962	22.1252	3.62647	40.6239	OK	OK	0.502832
ENSG0000005	-	-	ENSG0000005162	'HEBP2	-	6:138402584-138697288	-	-	IGV_LINK	1069.6	28.101	0	60.1394	OK	729.076	19.9172	0	43.4223	OK	OK	-0.49661
ENSG0000005	-	-	ENSG0000005182	'MPHOSPH9	-	12:123152319-123272334	-	-	IGV_LINK	479.926	13.8787	0	38.0088	OK	464.05	14.5421	0	41.3571	OK	OK	0.067368
ENSG0000005	-	-	ENSG0000005212	'PLEKHA5	-	12:19129751-19376400	-	-	IGV_LINK	1948.17	31.2296	11.6997	50.7594	OK	1873.74	31.9845	10.0076	53.9613	OK	OK	0.034459
ENSG0000005	-	-	ENSG0000005234	'PRSS8	-	16:31131432-31135762	-	-	IGV_LINK	113.457	4.29022	0.32564	8.25481	OK	90.7478	3.17246	0.106831	6.23809	OK	OK	-0.43545
ENSG0000005	-	-	ENSG0000005272	'SIKE1	-	1:114769478-114780685	-	-	IGV_LINK	495.527	13.7243	0	33.0693	OK	523.862	19.3374	0	45.5376	OK	OK	0.494659
ENSG0000005	-	-	ENSG0000005274	'RRP12	-	10:97356357-97426076	-	-	IGV_LINK	433.465	8.33829	0.127011	16.5496	OK	377.428	5.72698	1.12073	10.3332	OK	OK	-0.54198
ENSG0000005	-	-	ENSG0000005279	'FNIP2	-	4:158769137-159038760	-	-	IGV_LINK	782.564	9.0402	0	18.8612	OK	873.448	8.74259	0.595693	16.8895	OK	OK	-0.04829
ENSG0000005	-	-	ENSG0000005280	'MSMO1	-	4:165327622-165343160	-	-	IGV_LINK	232.733	7.14092	1.37896	12.9029	OK	621.829	18.8292	6.58803	31.0704	OK	OK	1.39879
ENSG0000005	-	-	ENSG0000005284	'TTC17	-	11:43311962-43494933	-	-	IGV_LINK	2523.21	62.2432	1.01946	123.467	OK	2426.47	49.8954	2.09776	97.6931	OK	OK	-0.31901
ENSG0000005	-	-	ENSG0000005285	'ALX4	-	11:44260443-44310166	-	-	IGV_LINK	2.90916	0.029394	0	0.078894	OK	0	0	0	0	OK	NOTEST	'-inf
ENSG0000005	-	-	ENSG0000005310	'FSTL4	-	5:133160437-133612564	-	-	IGV_LINK	7.75776	0.189076	0	0.743643	OK	15.4684	0.423225	0	1.15743	OK	NOTEST	1.16246
ENSG0000005	-	-	ENSG0000005325	'FOXN3	-	14:89124870-89954777	-	-	IGV_LINK	1269.36	16.9226	0	40.0369	OK	917.79	12.04	0	27.107	OK	OK	-0.49112
ENSG0000005	-	-	ENSG0000005332	'METTL24	-	6:110180140-110358272	-	-	IGV_LINK	20.3641	1.21253	0	5.12639	OK	6.18735	0.368859	0	2.2131	OK	OK	-1.71688
ENSG0000005	-	-	ENSG0000005337	'AKR7A2	-	1:19303964-19312146	-	-	IGV_LINK	1144.27	55.1424	23.5132	86.7715	OK	811.574	39.6515	15.9058	63.3971	OK	OK	-0.47579
ENSG0000005	-	-	ENSG0000005337	'MRTO4	-	1:19210500-19260128	-	-	IGV_LINK	553.71	16.5149	0	37.8296	OK	612.548	18.2701	0	42.527	OK	OK	0.145714
ENSG0000005	-	-	ENSG0000005343	'NNAT	-	20:37492471-37527931	-	-	IGV_LINK	11.6366	0.583788	0	3.54864	OK	7.21857	0.392926	0	3.27508	OK	OK	-0.57119
ENSG0000005	-	-	ENSG0000005350	'USE1	-	19:17215345-17219829	-	-	IGV_LINK	470.314	43.1662	13.2121	73.1204	OK	600.173	54.3692	19.5008	89.2377	OK	OK	0.332887
ENSG0000005	-	-	ENSG0000005352	'MCF2L2	-	3:183178042-183428778	-	-	IGV_LINK	96.0023	1.69119	0	3.94766	OK	94.8727	2.0697	0	6.28075	OK	OK	0.291381
ENSG0000005	-	-	ENSG0000005370	'NRIP2	-	12:1946053-2941140	-	-	IGV_LINK	430.556	9.71161	0	29.4692	OK	244.4	5.35441	0	18.3323	OK	OK	-0.85898
ENSG0000005	-	-	ENSG0000005374	'LAMA3	-	18:23689442-23955066	-	-	IGV_LINK	426.677	3.78509	0.508551	7.06163	OK	709.483	6.56442	2.14221	10.9866	OK	OK	0.794341
ENSG0000005	-	-	ENSG0000005377	'AP5M1	-	14:57200506-57298742	-	-	IGV_LINK	368.494	6.46056	0	14.7861	OK	348.554	6.63474	0	15.699	OK	OK	0.038381
ENSG0000005	-	-	ENSG0000005390	'ANAPC4	-	4:25377212-25418498	-	-	IGV_LINK	687.532	15.6266	5.41185	25.8413	OK	635.235	15.393	4.42663	26.3593	OK	OK	-0.02173
ENSG0000005	-	-	ENSG0000005391	'KCNQ1	-	11:2444683-2861568	-	-	IGV_LINK	304.492	5.65293	0	11.6436	OK	288.743	6.12419	0	13.5339	OK	OK	0.115519
ENSG0000005	-	-	ENSG0000005411	'TRAPPC3	-	1:36136569-36180849	-	-	IGV_LINK	899.901	45.9805	1.80442	90.1566	OK	971.414	53.2473	0	115.497	OK	OK	0.211687
ENSG0000005	-	-	ENSG0000005411	'THRAP3	-	1:36224415-36305357	-	-	IGV_LINK	2757.89	44.0566	6.86053	81.2526	OK	2889.49	45.4488	9.48203	81.4156	OK	OK	0.044886
ENSG0000005	-	-	ENSG0000005414	'PHPT1	-	9:136844414-136860799	-	-	IGV_LINK	1908.41	165.819	36.2797	295.358	OK	2101.64	200.259	53.8992	346.618	OK	OK	0.272255
ENSG0000005	-	-	ENSG0000005417	'ENTPD2	-	9:137048097-137054045	-	-	IGV_LINK	62.0621	2.06454	0.037506	4.09157	OK	80.4355	2.93728	0.168251	5.70631	OK	OK	0.508661
ENSG0000005	-	-	ENSG0000005421	'LY75	-	2:159712456-159904749	-	-	IGV_LINK	664.696	6.51145	0	14.2925	OK	819.948	8.7337	0	22.4238	OK	OK	0.423613
ENSG0000005	-	-	ENSG0000005426	'ARID4B	-	1:235131182-235344532	-	-	IGV_LINK	1078.36	16.687	0	37.6254	OK	814.688	11.8256	0	27.0598	OK	OK	-0.49681
ENSG0000005	-	-	ENSG0000005427	'OPN3	-	1:241532133-241802133	-	-	IGV_LINK	313.978	13.3306	0	29.9791	OK	773.003	25.8849	0	63.6572	OK	OK	0.957374
ENSG0000005	-	-	ENSG0000005428	'SDCCAG8	-	1:243256033-243851079	-	-	IGV_LINK	458.678	16.3874	0	41.5429	OK	432.083	15.3741	0	36.8018	OK	OK	-0.09208
ENSG0000005	-	-	ENSG0000005435	'PTPRN	-	2:219289622-219309648	-	-	IGV_LINK	8.72748	0.569214	0	1.98424	OK	22.6869	1.26628	0	3.54604	OK	OK	1.15356
ENSG0000005	-	-	ENSG0000005439	'HHAT	-	1:210328251-210676296	-	-	IGV_LINK	182.307	3.07209	0.455732	5.68845	OK	229.963	3.90791	1.04589	6.76994	OK	OK	0.347179
ENSG0000005	-	-	ENSG0000005452	'KIF1B	-	1:10210804-10381603	-	-	IGV_LINK	1103.03	6.67406	1.50995	11.8382	OK	1053.93	6.23549	2.5109	9.96008	OK	OK	-0.09806
ENSG0000005	-	-	ENSG0000005459	'FOXC1	-	6:1609971-1613897	-	-	IGV_LINK	90.184	1.35404	0.141696	2.56638	OK	45.3739	0.681461	0	1.36905	OK	OK	-0.99057
ENSG0000005	-	-	ENSG0000005461	'TBC1D22A	-	22:46762616-47175699	-	-	IGV_LINK	867.9	19.6933	6.84003	32.5465	OK	1214.78	28.318	11.538	45.0979	OK	OK	0.524014
ENSG0000005	-	-	ENSG0000005465	'SYNE2	-	14:63852982-64338599	-	-	IGV_LINK	3351.35	34.9514	4.96377	64.9391	OK	2704.9	33.0416	8.48913	57.5941	OK	OK	-0.08107
ENSG0000005	-	-	ENSG0000005469	'PLEKHH1	-	14:67447083-67600287	-	-	IGV_LINK	353.948	5.81562	0	14.9437	OK	205.214	3.05261	0	8.09862	OK	OK	-0.92989
ENSG0000005	-	-	ENSG0000005479	'ATP9A	-	20:51596513-51768634	-	-	IGV_LINK	504.255	3.68695	1.32152	6.05237	OK	582.642	4.21807	1.64279	6.79335	OK	OK	0.194157
ENSG0000005	-	-	ENSG0000005479	'SPO11	-	20:57329758-57343994	-	-	IGV_LINK	0	0	0	0	OK	0	0	0	0	OK	NOTEST	0
ENSG0000005	-	-	ENSG0000005480	'CBLN4	-	20:55997439-56005472	-	-	IGV_LINK	54.3043	1.07325	0.011571	2.13493	OK	40.2178	0.795168	0	1.60845	OK	OK	-0.43265
ENSG0000005	-	-	ENSG0000005493	'CHRDL2	-	11:74696428-74731385	-	-	IGV_LINK	4.8486	0.217125	0	0.808466	OK	0	0	0	0	OK	NOTEST	'-inf
ENSG0000005	-	-	ENSG0000005496	'FAM168A	-	11:73400486-73598189	-	-	IGV_LINK	1859.92	17.132	5.37646	28.8875	OK	1565.4	14.0417	4.56381	23.5195	OK	OK	-0.28698
ENSG0000005	-	-	ENSG0000005496	'RELT	-	11:73376263-73397474	-	-	IGV_LINK	426.677	7.04643	1.62401	12.4688	OK	424.865	7.86145	1.51162	14.2113	OK	OK	0.157903
ENSG0000005	-	-	ENSG0000005498	'GALC	-	14:87710418-87993665	-	-	IGV_LINK	702.078	12.4076	3.00568	21.8096	OK	904.384	16.2718	4.86324	27.6804	OK	OK	0.391147
ENSG0000005	-	-	ENSG0000005504	'NOP58	-	2:202265715-202303666	-	-	IGV_LINK	808.747	27.3678	9.25508	45.4804	OK	809.512	28.4609	8.69701	48.2247	OK	OK	0.056503
ENSG0000005	-	-	ENSG0000005507	'SZRD1	-	1:16352574-16437424	-	-	IGV_LINK	1651.43	47.5061	7.69608	87.3161	OK	1576.74	50.1739	7.89471	92.4532	OK	OK	0.078826
ENSG0000005	-	-	ENSG0000005511	'KCNH2	-	7:150944960-150978315	-	-	IGV_LINK	6.78804	0.144906	0	0.387055	OK	5.15612	0.107165	0	0.288219	OK	NOTEST	-0.43529
ENSG0000005	-	-	ENSG0000005513	'CUL1	-	7:148696466-148801036	-	-	IGV_LINK	1193.73	23.3417	9.78885	36.8945	OK	1189	23.4181	10.0099	36.8263	OK	OK	0.004715
ENSG0000005	-	-	ENSG0000005514	'FAM114A2	-	5:153990127-154445850	-	-	IGV_LINK	398.555	15.5522	0	38.15	OK	524.894	20.2665	0	48.2802	OK	OK	0.381983
ENSG0000005	-	-	ENSG0000005516	'CYFIP2	-	5:157260121-157575775	-	-	IGV_LINK	2372.91	38.0023	0.361113	75.6436	OK	1815.99	29.1727	0	59.8714	OK	OK	-0.38147
ENSG0000005	-	-	ENSG0000005520	'TAB2	-	6:149217925-149411613	-	-	IGV_LINK	1249.97	18.2448	7.07839	29.4112	OK	1109.6	16.5053	5.77937	27.2313	OK	OK	-0.14455
ENSG0000005	-	-	ENSG0000005521	'GINM1	-	6:149566293-149591748	-	-	IGV_LINK	691.411	21.336	8.44634	34.2256	OK	417.646	12.7203	4.65904	20.7815	OK	OK	-0.74616
ENSG0000005	-	-	ENSG0000005533	'EIF2AK2	-	2:37084450-37157065	-	-	IGV_LINK	1134.57	10.5705	0	26.1195	OK	1143.63	11.922	0	29.8484	OK	OK	0.173576
ENSG0000005	-	-	ENSG0000005548	'USP36	-	17:78787380-78841441	-	-	IGV_LINK	1271.3	21.7011	3.53575	39.8664	OK	1262.22	20.4201	3.90077	36.9394	OK	OK	-0.08778
ENSG0000005	-	-	ENSG0000005560	'KMT2C	-	7:152134921-152436005	-	-	IGV_LINK	1518.58	11.6175	0	23.9921	OK	1219.94	10.5382	0	21.2415	OK	OK	-0.14067
ENSG0000005	-	-	ENSG0000005573	'MCOLN3	-	1:84999146-85133138	-	-	IGV_LINK	13.5761	0.297454	0	0.769084	OK	8.2498	0.180891	0	0.487356	OK	NOTEST	-0.71755
ENSG0000005	-	-	ENSG0000005581	'CCDC85A	-	2:55952157-56386173	-	-	IGV_LINK	27.1025	0.400941	0	1.1418	OK	54.941	0.813014	0	1.95543	OK	OK	1.01989
ENSG0000005	-	-	ENSG0000005591	'PUM2	-	2:20248690-20352234	-	-	IGV_LINK	1819.2	22.0676	3.8104	40.3248	OK	1528.28	17.9562	3.80611	32.1064	OK	OK	-0.29744
ENSG0000005	-	-	ENSG0000005595	'MRPL43	-	10:100969457-100994401	-	-	IGV_LINK	920.265	64.6169	15.3203	113.914	OK	496.019	36.9928	5.82416	68.1615	OK	OK	-0.80467
ENSG0000005	-	-	ENSG0000005595	'ITIH4	-	3:52812974-52897596	-	-	IGV_LINK	235.582	7.54822	0	21.6922	OK	438.323	15.0177	0	37.0989	OK	OK	0.99246
ENSG0000005	-	-	ENSG0000005595	'ITIH1	-	3:52777591-52792068	-	-	IGV_LINK	12.6064	0.919104	0	2.54183	OK	353.71	10.5257	0.18892	20.8624	OK	OK	3.51754
ENSG0000005	-	-	ENSG0000005605	'HPF1	-	4:169729464-169757953	-	-	IGV_LINK	232.733	13.8012	3.28921	24.3133	OK	257.806	14.1604	3.66134	24.6594	OK	OK	0.037063
ENSG0000005	-	-	ENSG0000005609	'ZFR	-	5:32354349-32444761	-	-	IGV_LINK	1292.64	23.3141	5.46019	41.1681	OK	1194.16	22.989	2.5614	43.4167	OK	OK	-0.02026
ENSG0000005	-	-	ENSG0000005627	'ZNF280C	-	X:130202710-130268899	-	-	IGV_LINK	60.1227	0.759471	0	1.55265	OK	52.5925	0.722965	0	1.47531	OK	OK	-0.07107
ENSG0000005	-	-	ENSG0000005629	'NPFFR2	-	4:72031803-72148067	-	-	IGV_LINK	0	0	0	0	OK	0	0	0	0	OK	NOTEST	0
ENSG0000005	-	-	ENSG0000005648	'PHF21B	-	22:44881161-45009999	-	-	IGV_LINK	0	0	0	0	OK	0	0	0	0	OK	NOTEST	0
ENSG0000005	-	-	ENSG0000005655	'TRAF1	-	9:120902392-120929173	-	-	IGV_LINK	2201.27	23.8099	9.26096	38.3588	OK	1083.82	11.883	4.74447	19.0215	OK	OK	-1.00266
ENSG0000005	-	-	ENSG0000005658	'RC3H2	-	9:122844555-122905341	-	-	IGV_LINK	969.721	10.2644	0	21.7041	OK	728.045	6.00123	0.372471	11.63	OK	OK	-0.77431
ENSG0000005	-	-	ENSG0000005673	'IL17RB	-	3:53846579-53865800	-	-	IGV_LINK	786.443	20.2161	6.63569	33.7964	OK	2668.81	74.3383	28.7102	119.966	OK	OK	1.8786
ENSG0000005	-	-	ENSG0000005697	'TRAF3IP2	-	6:111299027-111606278	-	-	IGV_LINK	1192.76	36.148	5.4704	66.8257	OK	723.92	22.5641	4.80586	40.3224	OK	OK	-0.67989
ENSG0000005	-	-	ENSG0000005699	'GYG2	-	X:2828787-2882820	-	-	IGV_LINK	5.81832	0.14447	0	0.578193	OK	57.7486	1.0111	0	2.11314	OK	OK	2.80709
ENSG0000005	-	-	ENSG0000005701	'DCBLD2	-	3:98714329-98901689	-	-	IGV_LINK	319.038	4.81626	0	12.0765	OK	211.401	2.49119	0	6.18683	OK	OK	-0.95108
ENSG0000005	-	-	ENSG0000005714	'SERPINB3	-	18:63647578-63726432	-	-	IGV_LINK	0	0	0	0	OK	0	0	0	0	OK	NOTEST	0
ENSG0000005	-	-	ENSG0000005725	'SOAT1	-	1:179293713-179358680	-	-	IGV_LINK	1111.3	19.7038	0	42.1094	OK	1472.59	25.6265	0	53.7089	OK	OK	0.379162
ENSG0000005	-	-	ENSG0000005729	'PKP2	-	12:32790744-32896840	-	-	IGV_LINK	458.678	8.23872	0.972307	15.5051	OK	934.29	20.1504	3.24463	37.0561	OK	OK	1.29031
ENSG0000005	-	-	ENSG0000005746	'MSH4	-	1:75796881-75913238	-	-	IGV_LINK	2.90916	0.052998	0	0.142245	OK	16.4996	0.300693	0	0.670379	OK	NOTEST	2.50429
ENSG0000005	-	-	ENSG0000005759	'F7	-	13:113105787-113120681	-	-	IGV_LINK	0	0	0	0	OK	0	0	0	0	OK	NOTEST	0
ENSG0000005	-	-	ENSG0000005760	'GDI2	-	10:5765222-5842132	-	-	IGV_LINK	4035.98	107.328	42.5272	172.128	OK	2980.24	80.0126	32.5973	127.428	OK	OK	-0.42372
ENSG0000005	-	-	ENSG0000005765	'PRDM1	-	6:106045422-106325791	-	-	IGV_LINK	458.678	7.91452	0	17.9061	OK	506.331	8.20747	0	17.3912	OK	OK	0.052436
ENSG0000005	-	-	ENSG0000005766	'ATG5	-	6:106045422-106325791	-	-	IGV_LINK	403.404	8.97204	0	18.6223	OK	294.93	6.10398	0	13.1246	OK	OK	-0.55569
ENSG0000005	-	-	ENSG0000005770	'TMCC3	-	12:94567123-94650562	-	-	IGV_LINK	513.952	6.29817	0.563231	12.0331	OK	736.295	9.12901	2.44327	15.8148	OK	OK	0.535526
ENSG0000005	-	-	ENSG0000005775	'PITHD1	-	1:23778404-23788232	-	-	IGV_LINK	588.62	25.6881	9.67234	41.7038	OK	634.203	26.4432	9.87668	43.0097	OK	OK	0.041799
ENSG0000005	-	-	ENSG0000005793	'MTA3	-	2:42494568-42756947	-	-	IGV_LINK	580.863	13.731	2.4665	24.9955	OK	651.734	17.5198	3.71412	31.3254	OK	OK	0.351545
ENSG0000005	-	-	ENSG0000005805	'USP13	-	3:179652754-179789401	-	-	IGV_LINK	136.731	1.23298	0.005839	2.46013	OK	128.903	1.29672	0	3.11961	OK	OK	0.072711
ENSG0000005	-	-	ENSG0000005806	'ATP11B	-	3:182793499-182921938	-	-	IGV_LINK	917.356	8.70228	2.65897	14.7456	OK	797.137	6.72237	2.29966	11.1451	OK	OK	-0.37242
ENSG0000005	-	-	ENSG0000005808	'LAMC2	-	1:183186237-183244900	-	-	IGV_LINK	286.068	3.28519	0.954248	5.61612	OK	225.838	2.75682	0.235357	5.27829	OK	OK	-0.25297
ENSG0000005	-	-	ENSG0000005809	'CDK14	-	7:90335222-91210590	-	-	IGV_LINK	840.748	17.5313	0	43.2021	OK	892.01	17.9733	0	47.9769	OK	OK	0.035928
ENSG0000005	-	-	ENSG0000005826	'SEC61A1	-	3:128051640-128408646	-	-	IGV_LINK	3879.85	66.5382	17.2193	115.857	OK	5537.68	93.2366	22.0608	164.412	OK	OK	0.486713
ENSG0000005	-	-	ENSG0000005827	'PPP1R12A	-	12:79574978-79942712	-	-	IGV_LINK	832.02	20.9003	0	45.7426	OK	702.264	12.4662	0	26.1122	OK	OK	-0.7455
ENSG0000005	-	-	ENSG0000005833	'RASGRF1	-	15:78959946-79090773	-	-	IGV_LINK	21.3339	0.373101	0	1.20454	OK	23.7182	0.727644	0	2.17229	OK	OK	0.963665
ENSG0000005	-	-	ENSG0000005840	'CAMK2B	-	7:44217149-44334577	-	-	IGV_LINK	25.2127	0.462113	0	1.57438	OK	2.06245	0.061819	0	0.802035	OK	NOTEST	-2.90213
ENSG0000005	-	-	ENSG0000005845	'CROCC	-	1:16739937-16972979	-	-	IGV_LINK	688.502	13.5475	0	34.806	OK	657.922	16.263	0	38.8363	OK	OK	0.263563
ENSG0000005	-	-	ENSG0000005860	'POLR3E	-	16:22297374-22335103	-	-	IGV_LINK	714.684	18.5999	3.2981	33.9017	OK	925.009	26.5372	2.60199	50.4724	OK	OK	0.512721

ENSG0000005	-	-	ENSG0000005866	'ATP2B4	-	1:203626560-203744081	-	-	IGV_LINK	2907.22	21.1987	7.53654	34.8608	OK	3529.88	28.9685	7.567	50.3699	OK	OK	0.450509
ENSG0000005	-	-	ENSG0000005867	'ZC3H11A	-	1:203795653-203854999	-	-	IGV_LINK	3217.41	46.7102	8.1071	85.3132	OK	2814.12	38.6582	10.5991	66.7174	OK	OK	-0.27296
ENSG0000005	-	-	ENSG0000005872	'RIOK2	-	5:97089074-97437217	-	-	IGV_LINK	303.523	10.454	0	22.153	OK	311.43	13.4128	0	27.9208	OK	OK	0.359548
ENSG0000005	-	-	ENSG0000005879	'YIPF1	-	1:53851718-53889834	-	-	IGV_LINK	544.013	22.3554	7.46329	37.2475	OK	499.113	20.3348	6.796	33.8736	OK	OK	-0.13667
ENSG0000005	-	-	ENSG0000005880	'NDC1	-	1:53765459-53838860	-	-	IGV_LINK	284.128	3.22729	1.02533	5.42926	OK	277.4	3.35143	0.655644	6.04721	OK	OK	0.054451
ENSG0000005	-	-	ENSG0000005886	'DGKG	-	3:186046307-186362237	-	-	IGV_LINK	126.155	4.45965	0	15.6894	OK	185.62	4.60614	0	14.048	OK	OK	0.046629
ENSG0000005	-	-	ENSG0000005912	'FLYWCH1	-	16:2911936-2954276	-	-	IGV_LINK	1458.46	26.9232	9.28671	44.5597	OK	1202.41	28.0357	2.91402	53.1573	OK	OK	0.058412
ENSG0000005	-	-	ENSG0000005914	'UNKL	-	16:1351922-1414751	-	-	IGV_LINK	691.411	24.9397	0	107.679	OK	548.612	19.9944	0	94.3452	OK	OK	-0.31885
ENSG0000005	-	-	ENSG0000005937	'TBXAS1	-	7:139561569-140020325	-	-	IGV_LINK	1080.27	38.6184	0	109.267	OK	1223.03	44.2636	0	126.304	OK	OK	0.196833
ENSG0000005	-	-	ENSG0000005937	'PARP12	-	7:140023743-140063721	-	-	IGV_LINK	1312.03	48.7766	9.69169	87.8616	OK	1038.44	39.7993	7.20617	72.3924	OK	OK	-0.29345
ENSG0000005	-	-	ENSG0000005957	'ALDH18A1	-	10:95605928-95656706	-	-	IGV_LINK	947.417	18.6438	5.56602	31.7217	OK	1147.75	23.5347	6.51455	40.5549	OK	OK	0.336092
ENSG0000005	-	-	ENSG0000005958	'TARBP1	-	1:234391312-234479103	-	-	IGV_LINK	1244.15	26.5517	7.48084	45.6226	OK	720.826	16.2249	4.74778	27.7019	OK	OK	-0.7106
ENSG0000005	-	-	ENSG0000005969	'GATB	-	4:151670503-151761023	-	-	IGV_LINK	509.103	15.1581	4.83475	25.4814	OK	603.267	17.8627	6.23363	29.4918	OK	OK	0.236867
ENSG0000005	-	-	ENSG0000005972	'MXD1	-	2:69893559-69942945	-	-	IGV_LINK	445.102	5.05049	0	10.1649	OK	945.633	11.1036	1.40818	20.799	OK	OK	1.13653
ENSG0000005	-	-	ENSG0000005975	'CDK17	-	12:96278260-96400560	-	-	IGV_LINK	471.284	13.5092	0.299592	26.7188	OK	501.175	12.7941	1.48782	24.1004	OK	OK	-0.07846
ENSG0000005	-	-	ENSG0000005976	'DNAJC25	-	9:111631351-111670246	-	-	IGV_LINK	136.524	3.44775	0	7.96435	OK	192.496	4.88207	0	10.6723	OK	OK	0.50184
ENSG0000005	-	-	ENSG0000005980	'SLC2A3	-	12:7919229-7936275	-	-	IGV_LINK	1647.56	31.5169	7.27058	55.7633	OK	2124.32	42.5335	11.9162	73.1508	OK	OK	0.432472
ENSG0000005	-	-	ENSG0000005991	'PSD	-	10:102402616-102423136	-	-	IGV_LINK	28.1219	1.20803	0	4.45955	OK	16.4996	0.689821	0	2.79578	OK	OK	-0.80836
ENSG0000006	-	-	ENSG0000006006	'CTDP1	-	18:79679800-79756623	-	-	IGV_LINK	398.555	7.27257	2.02696	12.5182	OK	423.833	7.82435	2.35717	13.2915	OK	OK	0.105506
ENSG0000006	-	-	ENSG0000006013	'YBX3	-	12:10699088-10723312	-	-	IGV_LINK	4742.9	233.348	0	479.315	OK	5795.48	313.867	0	660.003	OK	OK	0.427668
ENSG0000006	-	-	ENSG0000006014	'STYK1	-	12:10618938-10674318	-	-	IGV_LINK	14.5458	0.290476	0	1.0902	OK	11.3435	0.363346	0	1.35548	OK	NOTEST	0.322923
ENSG0000006	-	-	ENSG0000006023	'WNK1	-	12:752592-911452	-	-	IGV_LINK	3042.98	32.6988	0	77.923	OK	3425.73	39.5555	0	100.602	OK	OK	0.274644
ENSG0000006	-	-	ENSG0000006030	'RPS17P5	-	6:50857254-50857662	-	-	IGV_LINK	0	0	0	0	OK	0	0	0	0	OK	NOTEST	0
ENSG0000006	-	-	ENSG0000006033	'CCAR1	-	10:68721011-68792377	-	-	IGV_LINK	1133.6	33.8322	3.28778	64.3766	OK	881.697	23.2612	1.75143	44.771	OK	OK	-0.54047
ENSG0000006	-	-	ENSG0000006049	'OGFR	-	20:62796452-62814000	-	-	IGV_LINK	1828.89	42.6742	11.1478	74.2006	OK	1946.95	43.3455	12.6803	74.0106	OK	OK	0.022517
ENSG0000006	-	-	ENSG0000006055	'GNA15	-	19:3136192-3163769	-	-	IGV_LINK	187.156	5.37995	1.23724	9.52266	OK	210.37	6.0508	1.32794	10.7737	OK	OK	0.169533
ENSG0000006	-	-	ENSG0000006056	'CREB3L3	-	19:4153600-4173054	-	-	IGV_LINK	85.3354	2.0632	0.147339	3.97906	OK	1150.85	27.7353	11.9775	43.493	OK	OK	3.74877
ENSG0000006	-	-	ENSG0000006064	'PIGV	-	1:26787471-26798398	-	-	IGV_LINK	405.343	11.3309	3.31897	19.3428	OK	427.958	11.6545	3.578	19.7309	OK	OK	0.040624
ENSG0000006	-	-	ENSG0000006065	'PTPRU	-	1:29236515-29326813	-	-	IGV_LINK	334.554	4.23843	0.094442	8.38241	OK	976.57	14.3603	0	28.9011	OK	OK	1.76049
ENSG0000006	-	-	ENSG0000006068	'SNRNP40	-	1:31259567-31296782	-	-	IGV_LINK	451.89	22.0988	5.97458	38.223	OK	451.677	20.8236	6.29503	35.3522	OK	OK	-0.08575
ENSG0000006	-	-	ENSG0000006070	'RIMBP2	-	12:130396136-130517917	-	-	IGV_LINK	0.969721	0.144008	0	0.614768	OK	5.15612	0.062416	0	0.358218	OK	NOTEST	-1.20615
ENSG0000006	-	-	ENSG0000006071	'COL11A1	-	1:102876466-103108496	-	-	IGV_LINK	0	0	0	0	OK	2.06245	0.022062	0	0.591927	OK	NOTEST	inf
ENSG0000006	-	-	ENSG0000006074	'QSER1	-	11:32893177-32993316	-	-	IGV_LINK	572.135	4.19058	1.0281	7.35306	OK	475.395	4.13529	0	11.3755	OK	OK	-0.01916
ENSG0000006	-	-	ENSG0000006076	'MPC1	-	6:166364918-166383013	-	-	IGV_LINK	611.894	48.9833	18.4962	79.4704	OK	369.179	29.7456	9.82901	49.6621	OK	OK	-0.71962
ENSG0000006	-	-	ENSG0000006097	'ACAA1	-	3:38039204-38137242	-	-	IGV_LINK	1444.88	60.3509	22.5388	98.163	OK	1437.53	61.8791	23.296	100.462	OK	OK	0.036077
ENSG0000006	-	-	ENSG0000006098	'BCAT1	-	12:24810021-24960158	-	-	IGV_LINK	123.155	1.2732	0	3.06553	OK	177.371	2.70997	0	6.74497	OK	OK	1.08982
ENSG0000006	-	-	ENSG0000006127	'HDAC7	-	12:47706084-47837898	-	-	IGV_LINK	2019.93	34.4834	0	70.4578	OK	1790.21	31.0682	0	63.7267	OK	OK	-0.15046
ENSG0000006	-	-	ENSG0000006133	'LZTS1	-	8:20246164-20303963	-	-	IGV_LINK	678.804	7.0618	2.49902	11.6246	OK	272.243	2.79422	0.197358	5.39109	OK	OK	-1.33759
ENSG0000006	-	-	ENSG0000006145	'PRDM6	-	5:123087247-123194266	-	-	IGV_LINK	75.6382	1.23799	0	4.63383	OK	32.9992	1.41308	0	6.46787	OK	OK	0.190844
ENSG0000006	-	-	ENSG0000006149	'WNT8A	-	5:138083891-138092365	-	-	IGV_LINK	2.90916	0.123677	0	0.383667	OK	1.03122	0.057012	0	0.26659	OK	NOTEST	-1.11725
ENSG0000006	-	-	ENSG0000006165	'SPAG4	-	20:35615891-35621049	-	-	IGV_LINK	350.069	25.7748	4.25697	47.2927	OK	996.163	80.5282	26.2319	134.825	OK	OK	1.64353
ENSG0000006	-	-	ENSG0000006167	'NCKAP1	-	2:182909114-183038858	-	-	IGV_LINK	1637.86	26.1355	1.74731	50.5236	OK	1779.89	28.1898	2.0612	54.3184	OK	OK	0.109164
ENSG0000006	-	-	ENSG0000006179	'MRPS35	-	12:27710772-27756295	-	-	IGV_LINK	613.833	20.2347	8.04225	32.4272	OK	665.14	22.052	8.88993	35.2141	OK	OK	0.124078
ENSG0000006	-	-	ENSG0000006191	'GUCY1B3	-	4:155758991-155807591	-	-	IGV_LINK	1089.97	23.2556	9.82012	36.6911	OK	1164.25	25.2599	10.4318	40.088	OK	OK	0.11927
ENSG0000006	-	-	ENSG0000006193	'SFSWAP	-	12:131711080-131799737	-	-	IGV_LINK	1504.04	28.7005	10.34	47.061	OK	1166.32	23.1877	8.21175	38.1637	OK	OK	-0.30771
ENSG0000006	-	-	ENSG0000006193	'TNK2	-	3:195863363-195911945	-	-	IGV_LINK	1387.67	35.6014	7.49776	63.7051	OK	1235.41	27.292	5.26391	49.3201	OK	OK	-0.38346
ENSG0000006	-	-	ENSG0000006198	'MON2	-	12:62466816-62600479	-	-	IGV_LINK	1062.81	14.6004	0	29.5971	OK	884.791	12.1297	1.15858	23.1007	OK	OK	-0.26747
ENSG0000006	-	-	ENSG0000006203	'CDH3	-	16:68636188-68722616	-	-	IGV_LINK	23.2733	0.803875	0	2.40247	OK	50.53	1.00398	0	2.53214	OK	OK	0.320692
ENSG0000006	-	-	ENSG0000006209	'ARSF	-	X:3041470-3112726	-	-	IGV_LINK	0	0	0	0	OK	1.03122	0.031975	0	0.137404	OK	NOTEST	inf
ENSG0000006	-	-	ENSG0000006219	'GPBP1	-	5:57173947-57264679	-	-	IGV_LINK	1556.4	39.154	13.6949	64.613	OK	1376.69	34.2825	14.0212	54.5438	OK	OK	-0.19169
ENSG0000006	-	-	ENSG0000006228	'DGAT2	-	11:75758454-75803415	-	-	IGV_LINK	81.4565	2.1771	0	4.76187	OK	234.088	6.47828	0.718703	12.2379	OK	OK	1.5732
ENSG0000006	-	-	ENSG0000006237	'ZNF112	-	19:44326554-44448578	-	-	IGV_LINK	108.609	1.92661	0	4.58505	OK	84.5604	1.52084	0	4.37809	OK	OK	-0.3412
ENSG0000006	-	-	ENSG0000006248	'CS	-	12:56271698-56334053	-	-	IGV_LINK	2683.38	65.1984	0	141.604	OK	2125.35	54.5944	0	127.562	OK	OK	-0.25608
ENSG0000006	-	-	ENSG0000006252	'LTK	-	15:41503637-41513887	-	-	IGV_LINK	17.455	0.416752	0	0.970171	OK	130.966	2.83769	0.43327	5.24211	OK	OK	2.76746
ENSG0000006	-	-	ENSG0000006258	'MRPS24	-	7:43866557-43926411	-	-	IGV_LINK	676.061	70.8693	11.2914	130.447	OK	787.856	82.6143	0.60449	164.624	OK	OK	0.22123
ENSG0000006	-	-	ENSG0000006259	'ELMO2	-	20:46364550-46496731	-	-	IGV_LINK	855.293	17.999	3.5742	32.4239	OK	672.359	13.9304	3.10564	24.7551	OK	OK	-0.36969
ENSG0000006	-	-	ENSG0000006265	'WAPL	-	10:86435255-86521815	-	-	IGV_LINK	1100.63	12.6577	4.08984	21.2255	OK	748.669	8.7916	2.68383	14.8994	OK	OK	-0.52581
ENSG0000006	-	-	ENSG0000006271	'VMP1	-	17:59618552-59842255	-	-	IGV_LINK	3401.78	108.052	0	247.279	OK	9007.75	266.737	0	623.065	OK	OK	1.3037
ENSG0000006	-	-	ENSG0000006272	'APPBP2	-	17:60443148-60526219	-	-	IGV_LINK	522.679	5.29713	1.54813	9.04613	OK	717.733	9.44052	0	19.4337	OK	OK	0.833655
ENSG0000006	-	-	ENSG0000006282	'POLD1	-	19:50384203-50431313	-	-	IGV_LINK	515.891	12.4229	0	26.828	OK	413.614	9.33265	0.771671	17.8936	OK	OK	-0.41264
ENSG0000006	-	-	ENSG0000006301	'SEZ6	-	17:28905249-29057220	-	-	IGV_LINK	0.969721	0.086133	0	1.72533	OK	4.1249	0.184797	0	3.6601	OK	NOTEST	1.10131
ENSG0000006	-	-	ENSG0000006304	'EIF4B	-	12:53006157-53079420	-	-	IGV_LINK	13921.3	252.468	1.51886	503.418	OK	11099.1	205.173	0	412.189	OK	OK	-0.29926
ENSG0000006	-	-	ENSG0000006312	'SLC6A16	-	19:49289637-49325225	-	-	IGV_LINK	153.216	4.40024	0	9.55114	OK	129.934	4.04156	0	9.27991	OK	OK	-0.12267
ENSG0000006	-	-	ENSG0000006316	'BICRA	-	19:47607523-47743134	-	-	IGV_LINK	236.612	5.18727	0	25.6794	OK	343.398	8.92826	0	50.8743	OK	OK	0.783403
ENSG0000006	-	-	ENSG0000006317	'SPHK2	-	19:48615327-48637438	-	-	IGV_LINK	244.37	7.08095	0	42.3907	OK	224.807	7.09655	0	36.5458	OK	OK	0.003176
ENSG0000006	-	-	ENSG0000006317	'RPL18	-	19:48615327-48637438	-	-	IGV_LINK	9570.17	1331.51	357.098	2305.93	OK	6587.47	917.648	212.907	1622.39	OK	OK	-0.53705
ENSG0000006	-	-	ENSG0000006318	'CA11	-	19:48637941-48682245	-	-	IGV_LINK	395.646	17.6452	0.895518	34.3948	OK	193.87	9.22136	0	23.0601	OK	OK	-0.93622
ENSG0000006	-	-	ENSG0000006324	'ISOC2	-	19:55452984-55462343	-	-	IGV_LINK	785.474	51.3496	19.5073	83.192	OK	1161.16	77.0829	32.4749	121.691	OK	OK	0.586056
ENSG0000006	-	-	ENSG0000006324	'U2AF2	-	19:55654145-55674715	-	-	IGV_LINK	2470.85	97.2705	24.8552	169.686	OK	2581.16	95.9851	25.7292	166.241	OK	OK	-0.01919
ENSG0000006	-	-	ENSG0000006324	'EPN1	-	19:55675225-55738402	-	-	IGV_LINK	2351.57	64.1331	10.7647	117.501	OK	3045.43	86.825	15.7598	157.89	OK	OK	0.437042
ENSG0000006	-	-	ENSG0000006332	'MED29	-	19:39391302-39400637	-	-	IGV_LINK	1185.97	21.8031	7.17168	36.4346	OK	1451.96	24.953	10.3456	39.5604	OK	OK	0.194679
ENSG0000006	-	-	ENSG0000006343	'AHRR	-	5:271620-438291	-	-	IGV_LINK	53.3346	1.25493	0	8.38439	OK	50.53	0.663374	0	3.33228	OK	OK	-0.91972
ENSG0000006	-	-	ENSG0000006351	'GSC2	-	22:19148575-19150283	-	-	IGV_LINK	0	0	0	0	OK	0	0	0	0	OK	NOTEST	0
ENSG0000006	-	-	ENSG0000006358	'ZNF275	-	X:153334154-153360110	-	-	IGV_LINK	490.679	4.55038	1.56212	7.53864	OK	446.52	4.12036	1.46454	6.77619	OK	OK	-0.14322
ENSG0000006	-	-	ENSG0000006360	'MTMR1	-	X:150692970-150765103	-	-	IGV_LINK	793.231	14.5639	0.805675	28.3221	OK	653.797	9.92461	3.38401	16.4652	OK	OK	-0.55331
ENSG0000006	-	-	ENSG0000006366	'GPC1	-	2:240435670-240468078	-	-	IGV_LINK	571.165	11.3114	2.52121	20.1017	OK	1035.35	19.9716	5.22133	34.7218	OK	OK	0.820164
ENSG0000006	-	-	ENSG0000006376	'ADCK1	-	14:77800082-77935012	-	-	IGV_LINK	111.518	2.97308	0	6.09688	OK	118.591	2.88359	0.285776	5.4814	OK	OK	-0.04409
ENSG0000006	-	-	ENSG0000006385	'HAGH	-	16:1795619-1884294	-	-	IGV_LINK	661.349	47.7483	9.0981	86.3984	OK	812.605	59.9027	6.75384	113.052	OK	OK	0.327173
ENSG0000006	-	-	ENSG0000006397	'RNF4	-	4:2418973-2732565	-	-	IGV_LINK	1260.64	29.6694	1.45689	57.8819	OK	1767.83	41.1627	4.18637	78.139	OK	OK	0.472361
ENSG0000006	-	-	ENSG0000006401	'CASP8	-	2:201233442-201287711	-	-	IGV_LINK	933.841	33.7606	6.84344	60.6778	OK	1375.65	49.7672	9.99948	89.535	OK	OK	0.559855
ENSG0000006	-	-	ENSG0000006404	'LIMCH1	-	4:41359606-41700044	-	-	IGV_LINK	699.168	9.56527	0.455794	18.6747	OK	655.859	7.5369	2.76847	12.3053	OK	OK	-0.34383
ENSG0000006	-	-	ENSG0000006410	'INTS13	-	12:26905180-26938326	-	-	IGV_LINK	412.131	13.1897	2.60741	23.7721	OK	434.146	12.2114	1.6991	22.7237	OK	OK	-0.11119
ENSG0000006	-	-	ENSG0000006411	'TM7SF3	-	12:26971585-27014434	-	-	IGV_LINK	2384.54	91.9639	1.44543	182.482	OK	2691.5	111.904	1.74199	222.067	OK	OK	0.283125
ENSG0000006	-	-	ENSG0000006419	'DLX3	-	17:49990004-49995224	-	-	IGV_LINK	3.87888	0.089977	0	0.284674	OK	0	0	0	0	OK	NOTEST	'-inf
ENSG0000006	-	-	ENSG0000006419	'SPA17	-	11:124622835-124697518	-	-	IGV_LINK	151.276	7.68897	0	28.4101	OK	159.84	9.7677	0	34.3306	OK	OK	0.345229
ENSG0000006	-	-	ENSG0000006420	'TSPAN32	-	11:2295644-2318200	-	-	IGV_LINK	212.369	10.2241	0	26.2587	OK	139.215	8.99179	0	23.9914	OK	OK	-0.1853
ENSG0000006	-	-	ENSG0000006420	'WISP2	-	20:44656450-44810338	-	-	IGV_LINK	0.969721	0.089227	0	0.568647	OK	12.3747	0.483303	0	1.23443	OK	NOTEST	2.43738
ENSG0000006	-	-	ENSG0000006421	'DMRT3	-	9:976963-991731	-	-	IGV_LINK	0	0	0	0	OK	3.09367	0.086768	0	0.350486	OK	NOTEST	inf
ENSG0000006	-	-	ENSG0000006422	'ST3GAL6	-	3:98714329-98901689	-	-	IGV_LINK	205.765	8.07839	0	18.8778	OK	139.91	6.63255	0	15.4634	OK	OK	-0.2845
ENSG0000006	-	-	ENSG0000006427	'ATP2C2	-	16:84368526-84467361	-	-	IGV_LINK	21.3339	1.45204	0	4.28617	OK	29.9055	0.997803	0	2.46694	OK	OK	-0.54126
ENSG0000006	-	-	ENSG0000006430	'NGFR	-	17:49494222-49583827	-	-	IGV_LINK	386.918	6.72064	2.13837	11.3029	OK	30.9367	0.537551	0	1.22025	OK	OK	-3.64412
ENSG0000006	-	-	ENSG0000006430	'CDON	-	11:125946055-126063335	-	-	IGV_LINK	662.319	7.87852	0.035775	15.7213	OK	247.494	3.15384	0	6.76292	OK	OK	-1.32082
ENSG0000006	-	-	ENSG0000006431	'TAF2	-	8:119730774-119832863	-	-	IGV_LINK	673.956	12.0185	1.58956	22.4474	OK	815.699	15.4969	1.64368	29.3501	OK	OK	0.366723
ENSG0000006	-	-	ENSG0000006439	'HIPK2	-	7:139561569-140020325	-	-	IGV_LINK	4818.54	19.6646	5.29821	34.0309	OK	7248.48	29.1149	7.20152	51.0283	OK	OK	0.56616
ENSG0000006	-	-	ENSG0000006441	'TNPO3	-	7:128954179-129055173	-	-	IGV_LINK	1036.63	14.7049	6.2758	23.1339	OK	1239.53	17.6749	7.71704	27.6328	OK	OK	0.265411
ENSG0000006	-	-	ENSG0000006448	'BORCS8-MEF2B	-	19:19145566-19203424	-	-	IGV_LINK	104.285	5.16316	0	19.0669	OK	91.9029	4.73402	0	18.3913	OK	OK	-0.12519
ENSG0000006	-	-	ENSG0000006449	'RFXANK	-	19:19145566-19203424	-	-	IGV_LINK	622.561	43.8794	0	89.3835	OK	710.514	48.2075	2.50628	93.9087	OK	OK	0.135714
ENSG0000006	-	-	ENSG0000006454	'TMEM161A	-	19:19119168-19138513	-	-	IGV_LINK	430.556	15.6396	3.85781	27.4213	OK	542.424	20.6581	5.51794	35.7982	OK	OK	0.401504
ENSG0000006	-	-	ENSG0000006454	'LPAR2	-	19:19623667-19628930	-	-	IGV_LINK	391.767	17.7678	2.90409	32.6315	OK	938.415	44.4234	11.5109	77.336	OK	OK	1.32205
ENSG0000006	-	-	ENSG0000006460	'CTSA	-	20:45888624-45912155	-	-	IGV_LINK	2999.35	117.836	26.038	209.634	OK	6501.87	243.476	0	528.368	OK	OK	1.047
ENSG0000006	-	-	ENSG0000006460	'SUGP2	-	19:18990887-19060311	-	-	IGV_LINK	3133.17	38.8963	2.59233	75.2003	OK	3051.39	39.0134	1.28661	76.7402	OK	OK	0.004335
ENSG0000006	-	-	ENSG0000006465	'SLC12A2	-	5:128083765-128189688	-	-	IGV_LINK	143.519	1.97172	0	5.21177	OK	152.621	1.75248	0	4.30084	OK	OK	-0.17005
ENSG0000006	-	-	ENSG0000006465	'SNX24	-	5:122843438-123054667	-	-	IGV_LINK	306.432	14.3162	0	29.15	OK	315.555	20.2459	0	42.8884	OK	OK	0.499978
ENSG0000006	-	-	ENSG0000006465	'EYA2	-	20:46894623-47188844	-	-	IGV_LINK	13.5761	0.372951	0	1.6643	OK	36.0929	0.974956	0	2.62782	OK	OK	1.38635
ENSG0000006	-	-	ENSG0000006466	'CNN2	-	19:1026580-1039068	-	-	IGV_LINK	863.051	29.7571	9.12134	50.3928	OK	802.293	27.5924	6.57147	48.6133	OK	OK	-0.10896
ENSG0000006	-	-	ENSG0000006468	'ABCA7	-	19:1040100-1065572	-	-	IGV_LINK	728.26	15.3127	0	30.6879	OK	748.669	14.0645	0	30.0026	OK	OK	-0.12267
ENSG0000006	-	-	ENSG0000006469	'SNCAIP	-	5:122311296-122479087	-	-	IGV_LINK	22.3036	0.610841	0	1.85977	OK	29.9055	0.658845	0	1.67595	OK	OK	0.109142
ENSG0000006	-	-	ENSG0000006470	'DDX20	-	1:111483347-111768016	-	-	IGV_LINK	267.643	3.79269	0	10.9541	OK	258.837	3.86925	0	13.3628	OK	OK	0.028834
ENSG0000006	-	-	ENSG0000006472	'BTBD1	-	15:82836945-83103443	-	-	IGV_LINK	800.989	17.525	0.176617	34.8734	OK	874.479	17.8706	1.04212	34.6991	OK	OK	0.028173
ENSG0000006	-	-	ENSG0000006476	'FAR2	-	12:29149102-29381189	-	-	IGV_LINK	351.039	9.94827	0	22.8453	OK	271.212	7.91614	0	19.429	OK	OK	-0.32965
ENSG0000006	-	-	ENSG0000006478	'BCAS1	-	20:53936776-54070594	-	-	IGV_LINK	1.93944	0.038382	0	0.196038	OK	72.1857	1.75488	0	3.90131	OK	OK	5.51479
ENSG0000006	-	-	ENSG0000006483	'POU1F1	-	3:87259403-87276587	-	-	IGV_LINK	0	0	0	0	OK	0	0	0	0	OK	NOTEST	0
ENSG0000006	-	-	ENSG0000006488	'CHI3L2	-	1:111187173-111243440	-	-	IGV_LINK	206.55	8.96072	0	24.4103	OK	397.022	16.8322	0	44.2331	OK	OK	0.909539
ENSG0000006	-	-	ENSG0000006493	'SBNO2	-	19:1107635-1174283	-	-	IGV_LINK	1932.65	29.7712	1.98365	57.5587	OK	2650.25	39.2254	6.42178	72.0291	OK	OK	0.397874
ENSG0000006	-	-	ENSG0000006493	'PMS1	-	2:189770322-189877629	-	-	IGV_LINK	190.065	4.37063	0	11.6914	OK	160.871	4.15764	0	11.9131	OK	OK	-0.07208
ENSG0000006	-	-	ENSG0000006496	'HMG20B	-	19:3506272-3579088	-	-	IGV_LINK	1350.82	55.0076	1.96445	108.051	OK	1166.32	46.4725	0	93.6185	OK	OK	-0.24326
ENSG0000006	-	-	ENSG0000006498	'CALCRL	-	2:187001875-187565760	-	-	IGV_LINK	520.74	10.2724	0	27.1473	OK	714.639	16.4116	0	53.347	OK	OK	0.675946
ENSG0000006	-	-	ENSG0000006499	'TAF11	-	6:34792014-34888089	-	-	IGV_LINK	553.71	22.2019	4.30461	40.0992	OK	372.272	14.918	1.67348	28.1625	OK	OK	-0.57363
ENSG0000006	-	-	ENSG0000006499	'ANKS1A	-	6:34889264-35091413	-	-	IGV_LINK	772.867	7.38042	2.51904	12.2418	OK	678.546	6.53913	2.05933	11.0189	OK	OK	-0.1746
ENSG0000006	-	-	ENSG0000006500	'AP3D1	-	19:2100987-2248679	-	-	IGV_LINK	2338.97	33.2294	0	68.5798	OK	2776.06	37.2906	0.115722	74.4655	OK	OK	0.166353
ENSG0000006	-	-	ENSG0000006502	'ZNF76	-	6:35258908-35295985	-	-	IGV_LINK	894.082	28.1372	7.35301	48.9214	OK	582.642	19.565	4.30996	34.8201	OK	OK	-0.5242
ENSG0000006	-	-	ENSG0000006505	'SLC9A3R2	-	16:2025355-2039026	-	-	IGV_LINK	960.993	51.1838	20.9578	81.4098	OK	836.323	46.0536	18.4731	73.6342	OK	OK	-0.15237
ENSG0000006	-	-	ENSG0000006505	'NTHL1	-	16:2039814-2135898	-	-	IGV_LINK	115.397	7.77007	0	32.2893	OK	82.498	5.8191	0	27.0334	OK	OK	-0.41713
ENSG0000006	-	-	ENSG0000006506	'UHRF1BP1	-	6:34792014-34888089	-	-	IGV_LINK	573.105	3.49989	0.693993	6.30579	OK	491.894	2.95018	0.703713	5.19665	OK	OK	-0.24651
ENSG0000006	-	-	ENSG0000006513	'GNAI3	-	1:109548610-109632051	-	-	IGV_LINK	1011.42	2.11095	0.208272	4.01363	OK	1273.56	2.65819	0.021019	5.29536	OK	OK	0.332552
ENSG0000006	-	-	ENSG0000006515	'IPO5	-	13:97953657-98024297	-	-	IGV_LINK	2486.36	36.46	10.1248	62.7952	OK	2036.67	29.8602	9.95943	49.761	OK	OK	-0.28809
ENSG0000006	-	-	ENSG0000006515	'OAT	-	10:124397302-124418976	-	-	IGV_LINK	731.169	23.2743	8.32913	38.2194	OK	565.111	18.2238	6.10388	30.3438	OK	OK	-0.35291
ENSG0000006	-	-	ENSG0000006518	'WDR3	-	1:117929719-118185223	-	-	IGV_LINK	601.227	9.91558	0	28.6707	OK	666.171	10.9913	0	27.238	OK	OK	0.148596
ENSG0000006	-	-	ENSG0000006524	'PKN2	-	1:88537512-88836255	-	-	IGV_LINK	783.534	16.3863	0	40.6127	OK	691.952	10.6851	0	24.8603	OK	OK	-0.61689
ENSG0000006	-	-	ENSG0000006526	'WDR18	-	19:984270-998438	-	-	IGV_LINK	472.254	21.1838	7.02238	35.3452	OK	432.083	19.2651	6.25047	32.2797	OK	OK	-0.13697
ENSG0000006	-	-	ENSG0000006530	'TRAM2	-	6:52361420-52576915	-	-	IGV_LINK	461.587	3.86207	0	8.31731	OK	708.452	5.92859	0.47821	11.379	OK	OK	0.618313
ENSG0000006	-	-	ENSG0000006532	'NTN1	-	17:9021541-9244000	-	-	IGV_LINK	24.243	0.236311	0	0.57268	OK	43.3114	0.422266	0	0.901551	OK	NOTEST	0.837467

ENSG0000006	-	-	ENSG0000006532	'GLP2R	-	17:9822205-9892102	-	-	IGV_LINK	1.93944	0.032069	0	0.266114	OK	139.215	2.47368	0	6.06205	OK	OK	6.26932
ENSG0000006	-	-	ENSG0000006532	'MCM10	-	10:13161553-13211104	-	-	IGV_LINK	36.8494	0.890846	0	2.67432	OK	28.8743	0.726759	0	2.23367	OK	OK	-0.2937
ENSG0000006	-	-	ENSG0000006535	'DGKA	-	12:55901412-55954027	-	-	IGV_LINK	877.597	30.8912	2.40211	59.3804	OK	715.67	28.2876	0	56.9861	OK	OK	-0.12703
ENSG0000006	-	-	ENSG0000006536	'ERBB3	-	12:56079856-56113907	-	-	IGV_LINK	3142.86	51.2397	0	147.019	OK	7174.23	94.0288	0	222.306	OK	OK	0.875842
ENSG0000006	-	-	ENSG0000006537	'ROPN1	-	3:123968520-123992178	-	-	IGV_LINK	0	0	0	0	OK	1.03122	0.040349	0	0.474547	OK	NOTEST	inf
ENSG0000006	-	-	ENSG0000006541	'ANKRD44	-	2:196967016-197311173	-	-	IGV_LINK	802.929	15.6347	0	37.8035	OK	384.647	7.538	0	21.5333	OK	OK	-1.0525
ENSG0000006	-	-	ENSG0000006542	'KARS	-	16:75627473-75761872	-	-	IGV_LINK	2707.46	87.1032	18.8905	155.316	OK	2191.35	69.4607	15.8971	123.024	OK	OK	-0.32653
ENSG0000006	-	-	ENSG0000006545	'ADAT1	-	16:75528531-75623300	-	-	IGV_LINK	286.068	18.2194	0	54.1583	OK	359.898	22.3456	0	64.0736	OK	OK	0.294516
ENSG0000006	-	-	ENSG0000006548	'PDIA5	-	3:123067061-123274130	-	-	IGV_LINK	836.579	32.3206	5.72503	58.9161	OK	1325.12	47.9369	13.973	81.9008	OK	OK	0.568685
ENSG0000006	-	-	ENSG0000006549	'TBC1D22B	-	6:37212179-37332970	-	-	IGV_LINK	479.042	8.20696	2.90142	13.5125	OK	376.397	6.4507	2.10243	10.799	OK	OK	-0.34739
ENSG0000006	-	-	ENSG0000006551	'NDUFB4	-	3:120596308-120602500	-	-	IGV_LINK	1551.55	167.071	70.5509	263.591	OK	1424.12	154.889	67.5179	242.26	OK	OK	-0.10922
ENSG0000006	-	-	ENSG0000006552	'SPEN	-	1:15834473-15940460	-	-	IGV_LINK	2241.99	16.3803	0	41.8742	OK	1625.21	13.8122	0	36.5925	OK	OK	-0.24602
ENSG0000006	-	-	ENSG0000006553	'MYLK	-	3:123585541-123884331	-	-	IGV_LINK	1734.83	22.9031	1.54521	44.2609	OK	1435.47	22.0747	0	49.8606	OK	OK	-0.05314
ENSG0000006	-	-	ENSG0000006554	'ZC3H15	-	2:186486155-186509363	-	-	IGV_LINK	726.321	25.1764	7.89037	42.4624	OK	908.509	33.4882	9.99237	56.984	OK	OK	0.411582
ENSG0000006	-	-	ENSG0000006555	'MAP2K4	-	17:11998352-12143830	-	-	IGV_LINK	559.529	8.73682	3.29896	14.1747	OK	605.329	9.59075	3.29109	15.8904	OK	OK	0.134535
ENSG0000006	-	-	ENSG0000006560	'TMEM206	-	1:212363930-212414901	-	-	IGV_LINK	139.64	5.04941	0	10.184	OK	174.277	6.27429	0.254402	12.2942	OK	OK	0.313337
ENSG0000006	-	-	ENSG0000006560	'SNAP91	-	6:83552879-83709691	-	-	IGV_LINK	20.3641	0.527806	0	1.63053	OK	0	0	0	0	OK	NOTEST	'-inf
ENSG0000006	-	-	ENSG0000006561	'SLK	-	10:103967200-104029233	-	-	IGV_LINK	1119.06	10.4626	0	21.5253	OK	674.421	6.44319	0	13.7266	OK	OK	-0.69939
ENSG0000006	-	-	ENSG0000006561	'CYB5R4	-	6:83859642-83967424	-	-	IGV_LINK	467.405	13.2521	0	27.0839	OK	443.427	13.846	0	29.5181	OK	OK	0.063255
ENSG0000006	-	-	ENSG0000006561	'COL17A1	-	10:104031285-104086002	-	-	IGV_LINK	0	0	0	0	OK	5.15612	0.052259	0	0.503451	OK	NOTEST	inf
ENSG0000006	-	-	ENSG0000006562	'GSTO2	-	10:104268872-104304945	-	-	IGV_LINK	104.73	3.20834	0	7.20541	OK	11.3435	0.407141	0	1.26342	OK	OK	-2.97823
ENSG0000006	-	-	ENSG0000006566	'SEC61A2	-	10:12129636-12250589	-	-	IGV_LINK	145.458	4.91041	0	16.8743	OK	102.091	3.03055	0	9.79174	OK	OK	-0.69626
ENSG0000006	-	-	ENSG0000006567	'PRKCQ	-	10:6427142-6580301	-	-	IGV_LINK	189.095	3.48179	0.753026	6.21055	OK	106.216	1.94311	0.262619	3.62359	OK	OK	-0.84147
ENSG0000006	-	-	ENSG0000006571	'TLE2	-	19:2997637-3047635	-	-	IGV_LINK	128.003	3.80153	0	7.87967	OK	109.31	3.48022	0	7.64363	OK	OK	-0.1274
ENSG0000006	-	-	ENSG0000006580	'ASB1	-	2:238426741-238452250	-	-	IGV_LINK	560.498	10.0995	0	22.3522	OK	530.05	9.4055	0	20.1949	OK	OK	-0.10272
ENSG0000006	-	-	ENSG0000006580	'FAM107B	-	10:14518556-14774897	-	-	IGV_LINK	1846.35	51.082	8.72638	93.4376	OK	2350.16	61.6112	12.9904	110.232	OK	OK	0.270377
ENSG0000006	-	-	ENSG0000006583	'ME1	-	6:83210388-83431071	-	-	IGV_LINK	145.458	2.56289	0.424113	4.70167	OK	242.338	4.2714	1.2289	7.31389	OK	OK	0.736935
ENSG0000006	-	-	ENSG0000006588	'TBC1D1	-	4:37891086-38139175	-	-	IGV_LINK	1468.16	38.2311	10.3244	66.1378	OK	1741.74	54.6173	13.1138	96.1208	OK	OK	0.514612
ENSG0000006	-	-	ENSG0000006588	'CDK13	-	7:39950036-40097134	-	-	IGV_LINK	917.356	14.3232	2.76071	25.8857	OK	954.914	13.4356	3.35187	23.5194	OK	OK	-0.09229
ENSG0000006	-	-	ENSG0000006591	'MTHFD2	-	2:74198561-74421662	-	-	IGV_LINK	800.989	30.6823	0	76.0416	OK	969.351	36.6887	0	87.7777	OK	OK	0.257926
ENSG0000006	-	-	ENSG0000006592	'SLC9A7	-	X:46599251-46759172	-	-	IGV_LINK	516.861	4.74973	0	12.2446	OK	257.806	2.85036	0	8.32885	OK	OK	-0.7367
ENSG0000006	-	-	ENSG0000006597	'FOXJ2	-	12:8032702-8055503	-	-	IGV_LINK	766.079	8.52566	3.25422	13.7971	OK	682.671	7.55645	2.52172	12.5912	OK	OK	-0.1741
ENSG0000006	-	-	ENSG0000006597	'YBX1	-	1:42682426-42702349	-	-	IGV_LINK	5613.71	238.47	94.5717	382.369	OK	4900.38	209.114	84.363	333.864	OK	OK	-0.18952
ENSG0000006	-	-	ENSG0000006598	'PDE4A	-	19:10391133-10469631	-	-	IGV_LINK	488.739	7.65008	0	20.2237	OK	430.021	6.70639	0	19.0664	OK	OK	-0.18994
ENSG0000006	-	-	ENSG0000006602	'PPP2R5A	-	1:212285536-212361863	-	-	IGV_LINK	848.505	16.2865	6.64882	25.9242	OK	763.106	14.6884	5.97863	23.3982	OK	OK	-0.149
ENSG0000006	-	-	ENSG0000006603	'CTNNA2	-	2:79157002-80648861	-	-	IGV_LINK	1.93944	0.041724	0	0.768137	OK	10.3122	0.275939	0	1.32055	OK	NOTEST	2.72541
ENSG0000006	-	-	ENSG0000006604	'ELAVL1	-	19:7958578-8005659	-	-	IGV_LINK	781.595	20.5142	5.50715	35.5213	OK	888.916	22.9255	5.89865	39.9524	OK	OK	0.16033
ENSG0000006	-	-	ENSG0000006605	'TIE1	-	1:43300992-43323108	-	-	IGV_LINK	560.498	10.6076	0.752874	20.4623	OK	685.765	12.2951	0.146246	24.444	OK	OK	0.21299
ENSG0000006	-	-	ENSG0000006608	'DIP2B	-	12:50504984-50748667	-	-	IGV_LINK	955.175	6.57645	2.55738	10.5955	OK	837.355	5.71456	2.34621	9.08291	OK	OK	-0.20267
ENSG0000006	-	-	ENSG0000006611	'SMARCD1	-	12:50084971-50100712	-	-	IGV_LINK	1066.69	38.6973	4.95709	72.4375	OK	1015.76	34.2682	4.48097	64.0553	OK	OK	-0.17536
ENSG0000006	-	-	ENSG0000006613	'KDM4A	-	1:43650157-43931165	-	-	IGV_LINK	765.109	11.6491	1.58745	21.7108	OK	988.945	14.7596	2.31427	27.2048	OK	OK	0.34143
ENSG0000006	-	-	ENSG0000006613	'NFYC	-	1:40690379-40771603	-	-	IGV_LINK	1021.12	40.0634	9.9349	70.1918	OK	838.386	34.8721	10.9699	58.7744	OK	OK	-0.20021
ENSG0000006	-	-	ENSG0000006618	'ZMYND12	-	1:42430328-42654664	-	-	IGV_LINK	124.124	6.82297	0	23.7995	OK	96.9351	4.45394	0	17.0654	OK	OK	-0.61532
ENSG0000006	-	-	ENSG0000006623	'SLC9A3	-	5:473235-524332	-	-	IGV_LINK	178.429	17.7285	0	58.9402	OK	46.4051	4.41647	0	14.9792	OK	OK	-2.00511
ENSG0000006	-	-	ENSG0000006624	'NGEF	-	2:232697298-233015885	-	-	IGV_LINK	153.216	3.42782	0	13.0519	OK	443.427	10.5949	0	30.4039	OK	OK	1.62801
ENSG0000006	-	-	ENSG0000006627	'ASPM	-	1:197084127-197146694	-	-	IGV_LINK	185.217	1.2724	0.16995	2.37484	OK	126.841	1.05756	0	2.26648	OK	OK	-0.2668
ENSG0000006	-	-	ENSG0000006629	'CD84	-	1:160537072-160579516	-	-	IGV_LINK	1499.19	15.4549	0.418967	30.4909	OK	1300.37	11.0972	2.00835	20.186	OK	OK	-0.47788
ENSG0000006	-	-	ENSG0000006632	'ELOVL1	-	1:43363396-43368074	-	-	IGV_LINK	1229.61	59.5235	24.2261	94.8209	OK	1163.22	54.8444	23.0706	86.6182	OK	OK	-0.11812
ENSG0000006	-	-	ENSG0000006633	'SPI1	-	11:47354859-47378576	-	-	IGV_LINK	1407.06	68.3528	27.8235	108.882	OK	1457.12	71.7188	31.253	112.185	OK	OK	0.069352
ENSG0000006	-	-	ENSG0000006637	'ZNRD1	-	6:30001010-30064909	-	-	IGV_LINK	399.525	33.2114	0	72.7787	OK	230.994	19.1507	0	44.0153	OK	OK	-0.79428
ENSG0000006	-	-	ENSG0000006638	'MPPED2	-	11:30384492-30630508	-	-	IGV_LINK	32.9705	1.25296	0	2.81546	OK	32.9992	0.909712	0	2.13402	OK	OK	-0.46186
ENSG0000006	-	-	ENSG0000006640	'CLDN18	-	3:137998734-138033655	-	-	IGV_LINK	25.2127	0.424463	0	0.885697	OK	25.7806	0.434173	0	0.906209	OK	NOTEST	0.032632
ENSG0000006	-	-	ENSG0000006642	'ZBTB11	-	3:101648888-101679217	-	-	IGV_LINK	448.981	4.83184	0.720301	8.94337	OK	364.022	3.69525	0.369134	7.02137	OK	OK	-0.3869
ENSG0000006	-	-	ENSG0000006642	'ATXN3	-	14:91965990-92106621	-	-	IGV_LINK	442.168	13.1722	0	35.0225	OK	408.59	11.3583	0	30.2838	OK	OK	-0.21375
ENSG0000006	-	-	ENSG0000006645	'GOLGA5	-	14:92794230-92839963	-	-	IGV_LINK	698.199	14.0703	5.78698	22.3536	OK	797.137	16.2141	6.5559	25.8723	OK	OK	0.204594
ENSG0000006	-	-	ENSG0000006646	'FGFR2	-	10:121478333-121598458	-	-	IGV_LINK	1222.82	22.5702	5.73709	39.4034	OK	1782.99	32.0107	7.78375	56.2376	OK	OK	0.504131
ENSG0000006	-	-	ENSG0000006655	'LRRC40	-	1:69568397-70205620	-	-	IGV_LINK	201.702	4.19218	0.909092	7.47526	OK	300.086	6.23965	2.00567	10.4736	OK	OK	0.573766
ENSG0000006	-	-	ENSG0000006658	'ISOC1	-	5:129094750-129114028	-	-	IGV_LINK	318.068	10.1415	3.2876	16.9955	OK	542.424	17.8852	6.4421	29.3283	OK	OK	0.818492
ENSG0000006	-	-	ENSG0000006662	'EML1	-	14:99737692-99942060	-	-	IGV_LINK	552.741	11.5702	0.425395	22.715	OK	361.96	7.09334	0	14.46	OK	OK	-0.70587
ENSG0000006	-	-	ENSG0000006665	'TRMT11	-	6:125986429-126039276	-	-	IGV_LINK	258.915	11.9025	2.64005	21.1649	OK	213.464	10.9655	0	22.2876	OK	OK	-0.11829
ENSG0000006	-	-	ENSG0000006665	'THUMPD1	-	16:20610242-20925006	-	-	IGV_LINK	841.717	16.9056	0	38.4865	OK	718.764	13.2216	0	34.8181	OK	OK	-0.35461
ENSG0000006	-	-	ENSG0000006669	'MSANTD3	-	9:100427155-100577636	-	-	IGV_LINK	312.25	13.1045	3.41477	22.7942	OK	291.837	12.5675	3.24436	21.8907	OK	OK	-0.06036
ENSG0000006	-	-	ENSG0000006673	'KIF26A	-	14:104138722-104180894	-	-	IGV_LINK	145.458	1.25314	0.197217	2.30906	OK	135.09	1.20199	0.221549	2.18244	OK	OK	-0.06012
ENSG0000006	-	-	ENSG0000006673	'ATG2B	-	14:96279201-96387288	-	-	IGV_LINK	712.745	6.16727	0	17.0746	OK	497.05	4.09113	0	10.9679	OK	OK	-0.59213
ENSG0000006	-	-	ENSG0000006677	'ARFGEF1	-	8:66964098-67343677	-	-	IGV_LINK	1050.21	14.3807	0	34.9604	OK	1154.97	19.6463	0	45.1329	OK	OK	0.450124
ENSG0000006	-	-	ENSG0000006681	'ACSM2B	-	16:20536225-20576427	-	-	IGV_LINK	3591.84	131.883	24.7542	239.012	OK	6055.35	207.161	40.4157	373.905	OK	OK	0.65149
ENSG0000006	-	-	ENSG0000006682	'ZFAT	-	8:134477787-134713049	-	-	IGV_LINK	254.067	5.40924	0.079989	10.7385	OK	483.645	11.4375	0.784299	22.0906	OK	OK	1.08027
ENSG0000006	-	-	ENSG0000006685	'MTFR1	-	8:65644733-65842322	-	-	IGV_LINK	236.612	10.5646	0	32.1814	OK	400.115	16.037	0	48.057	OK	OK	0.602171
ENSG0000006	-	-	ENSG0000006692	'STAG3	-	7:100177562-100272218	-	-	IGV_LINK	470.847	16.7086	0	37.0901	OK	354.795	11.3548	0	25.4675	OK	OK	-0.55729
ENSG0000006	-	-	ENSG0000006692	'FECH	-	18:57548282-57586772	-	-	IGV_LINK	328.735	10.6549	0.2553	21.0545	OK	478.488	16.7037	1.48574	31.9217	OK	OK	0.648653
ENSG0000006	-	-	ENSG0000006693	'MYO9A	-	15:71818395-72155459	-	-	IGV_LINK	1977.26	12.2835	0.914969	23.652	OK	1291.09	7.9812	0	16.934	OK	OK	-0.62204
ENSG0000006	-	-	ENSG0000006704	'DDX3Y	-	Y:12904107-12920478	-	-	IGV_LINK	623.53	16.0256	0	32.8979	OK	487.769	9.15335	0	18.5772	OK	OK	-0.80801
ENSG0000006	-	-	ENSG0000006705	'PFKP	-	10:3066332-3172841	-	-	IGV_LINK	7939.1	202.653	37.2396	368.067	OK	4243.49	105.552	24.6202	186.484	OK	OK	-0.94105
ENSG0000006	-	-	ENSG0000006706	'IDI1	-	10:971145-1049170	-	-	IGV_LINK	314.189	10.9283	0	23.1775	OK	255.744	8.51959	0	18.5386	OK	OK	-0.35921
ENSG0000006	-	-	ENSG0000006706	'SP100	-	2:230415941-230580006	-	-	IGV_LINK	1718.09	63.4953	14.7642	112.226	OK	2166.47	79.5503	18.2789	140.822	OK	OK	0.325218
ENSG0000006	-	-	ENSG0000006708	'KLF6	-	10:3775995-3785281	-	-	IGV_LINK	3176.8	58.8253	3.91347	113.737	OK	2382.13	44.249	4.18855	84.3095	OK	OK	-0.41079
ENSG0000006	-	-	ENSG0000006711	'PLPP1	-	5:55307759-55535050	-	-	IGV_LINK	1569.01	63.3672	17.3954	109.339	OK	1565.4	62.8427	16.7105	108.975	OK	OK	-0.01199
ENSG0000006	-	-	ENSG0000006714	'NEO1	-	15:73051709-73305206	-	-	IGV_LINK	1632.04	21.9491	5.0996	38.7985	OK	505.3	9.25031	0	20.94	OK	OK	-1.24659
ENSG0000006	-	-	ENSG0000006716	'TRAM1	-	8:70573441-70608387	-	-	IGV_LINK	2518.36	72.269	13.901	130.637	OK	3706.22	102.6	18.2144	186.985	OK	OK	0.505576
ENSG0000006	-	-	ENSG0000006717	'PHKA1	-	X:72578813-72714319	-	-	IGV_LINK	544.983	5.74969	2.20071	9.29868	OK	558.924	6.05603	2.30791	9.80414	OK	OK	0.074886
ENSG0000006	-	-	ENSG0000006718	'TNFRSF1A	-	12:6328756-6342114	-	-	IGV_LINK	1845.38	70.4957	19.765	121.226	OK	2912.18	114.535	33.6795	195.391	OK	OK	0.700187
ENSG0000006	-	-	ENSG0000006719	'CACNB1	-	17:39173289-39197703	-	-	IGV_LINK	225.945	5.56227	0	12.8302	OK	122.716	4.00295	0	10.5399	OK	OK	-0.47461
ENSG0000006	-	-	ENSG0000006720	'EVI5	-	1:92508695-92792404	-	-	IGV_LINK	261.825	3.85234	0	9.42463	OK	218.62	2.7926	0	6.48434	OK	OK	-0.46413
ENSG0000006	-	-	ENSG0000006722	'STOML1	-	15:73978922-73994622	-	-	IGV_LINK	295.765	10.5968	1.63329	19.5603	OK	410.428	13.7091	3.92034	23.4978	OK	OK	0.371499
ENSG0000006	-	-	ENSG0000006722	'PKM	-	15:72199028-72231822	-	-	IGV_LINK	28015.2	763.524	76.4865	1450.56	OK	39006.1	1077.26	0	2165.96	OK	OK	0.496623
ENSG0000006	-	-	ENSG0000006724	'DHX29	-	5:55233933-55307722	-	-	IGV_LINK	451.89	6.20553	1.75449	10.6566	OK	431.052	5.7186	1.85064	9.58657	OK	OK	-0.11789
ENSG0000006	-	-	ENSG0000006733	'DNTTIP2	-	1:93866282-93879918	-	-	IGV_LINK	794.201	20.1539	6.48244	33.8254	OK	814.668	21.5267	6.61604	36.4373	OK	OK	0.095064
ENSG0000006	-	-	ENSG0000006736	'METTL22	-	16:8621682-8649654	-	-	IGV_LINK	293.825	15.8592	0	32.8999	OK	381.553	23.1365	0.479903	45.793	OK	OK	0.544845
ENSG0000006	-	-	ENSG0000006736	'TP53BP1	-	15:43358171-43510728	-	-	IGV_LINK	1914.23	22.5352	0	56.6683	OK	1859.3	22.8375	0	58.3603	OK	OK	0.019226
ENSG0000006	-	-	ENSG0000006744	'TRO	-	X:54920461-54931431	-	-	IGV_LINK	658.525	20.9898	3.0937	38.8859	OK	582.642	18.872	2.71766	35.0263	OK	OK	-0.15344
ENSG0000006	-	-	ENSG0000006753	'RRP15	-	1:218285286-218337983	-	-	IGV_LINK	189.095	4.9064	0	13.1972	OK	211.401	7.13434	0	17.8942	OK	OK	0.540116
ENSG0000006	-	-	ENSG0000006756	'RHOA	-	3:49359144-49416475	-	-	IGV_LINK	4507.26	140.828	48.8786	232.778	OK	4427.05	141.268	44.9918	237.545	OK	OK	0.004501
ENSG0000006	-	-	ENSG0000006759	'DHX8	-	17:43483864-43610338	-	-	IGV_LINK	861.112	14.6662	0	30.6066	OK	963.164	13.795	0.555809	27.0343	OK	OK	-0.08835
ENSG0000006	-	-	ENSG0000006760	'PMS2P4	-	7:67295607-67321526	-	-	IGV_LINK	71.7593	13.8892	0	33.5891	OK	97.9664	19.1728	0	46.9082	OK	OK	0.465096
ENSG0000006	-	-	ENSG0000006760	'PRKCZ	-	1:2050469-2220738	-	-	IGV_LINK	242.43	7.29229	0	18.0535	OK	268.118	8.74059	0	20.881	OK	OK	0.261359
ENSG0000006	-	-	ENSG0000006764	'ZFY	-	Y:2935280-3002626	-	-	IGV_LINK	99.8812	2.95451	0	6.7465	OK	82.498	1.47708	0	3.07136	OK	OK	-1.00017
ENSG0000006	-	-	ENSG0000006770	'IARS2	-	1:220094101-220148041	-	-	IGV_LINK	1700.89	28.6193	12.0495	45.1891	OK	2026.36	33.7278	14.9171	52.5386	OK	OK	0.236951
ENSG0000006	-	-	ENSG0000006771	'SYT1	-	12:78863992-79503396	-	-	IGV_LINK	25.2127	0.30957	0	1.12391	OK	22.6869	0.307661	0	1.09618	OK	NOTEST	-0.00893
ENSG0000006	-	-	ENSG0000006779	'NAV3	-	12:77324640-78213008	-	-	IGV_LINK	18.4247	1.12527	0	3.15524	OK	71.1545	3.14816	0	9.14432	OK	OK	1.48424
ENSG0000006	-	-	ENSG0000006782	'IDH3G	-	X:153785765-153798505	-	-	IGV_LINK	798.08	39.9335	0	88.1479	OK	632.141	32.1633	0	72.1659	OK	OK	-0.31219
ENSG0000006	-	-	ENSG0000006783	'ROGDI	-	16:4796967-4802950	-	-	IGV_LINK	358.797	13.8915	2.38319	25.3998	OK	283.587	12.5747	2.66455	22.4849	OK	OK	-0.14367
ENSG0000006	-	-	ENSG0000006784	'PDZD4	-	X:153802165-153830565	-	-	IGV_LINK	56.2438	1.37304	0	3.35587	OK	26.8118	0.558369	0	1.44423	OK	OK	-1.29809
ENSG0000006	-	-	ENSG0000006784	'ATP2B3	-	X:153517675-153582939	-	-	IGV_LINK	0.969721	0.014043	0	0.241874	OK	2.06245	0.018606	0	0.247101	OK	NOTEST	0.405898
ENSG0000006	-	-	ENSG0000006790	'ROCK1	-	18:20946905-21111851	-	-	IGV_LINK	1303.3	14.5666	0	29.882	OK	1198.28	12.4803	0.833402	24.1272	OK	OK	-0.22301
ENSG0000006	-	-	ENSG0000006795	'CBFB	-	16:67029115-67101058	-	-	IGV_LINK	382.07	17.524	0.897106	34.1509	OK	375.366	18.6504	0	42.1664	OK	OK	0.089876
ENSG0000006	-	-	ENSG0000006799	'PDK3	-	X:24465220-24539837	-	-	IGV_LINK	157.095	5.13567	0.930578	9.34075	OK	211.401	6.93409	1.85606	12.0121	OK	OK	0.433155
ENSG0000006	-	-	ENSG0000006800	'HYAL2	-	3:50317789-50328251	-	-	IGV_LINK	1036.63	31.1869	10.2465	52.1274	OK	1242.63	37.0787	12.8232	61.3343	OK	OK	0.249649
ENSG0000006	-	-	ENSG0000006802	'HDAC4	-	2:239048167-239402364	-	-	IGV_LINK	256.976	4.53271	0	14.4928	OK	229.963	3.61644	0	10.912	OK	OK	-0.3258
ENSG0000006	-	-	ENSG0000006802	'RASSF1	-	3:50329781-50340980	-	-	IGV_LINK	628.379	22.4039	8.62983	36.1779	OK	526.956	18.8545	6.30555	31.4034	OK	OK	-0.24884
ENSG0000006	-	-	ENSG0000006807	'FGFR3	-	4:1793306-1808872	-	-	IGV_LINK	510.073	8.37908	1.53859	15.2196	OK	302.149	5.18119	0.675801	9.68657	OK	OK	-0.69351
ENSG0000006	-	-	ENSG0000006807	'IFI35	-	17:43006724-43014456	-	-	IGV_LINK	1008.51	60.1116	21.9153	98.308	OK	988.945	57.5375	21.9939	93.0812	OK	OK	-0.06314
ENSG0000006	-	-	ENSG0000006809	'HEATR6	-	17:60043193-60078931	-	-	IGV_LINK	363.645	7.65235	0.146942	15.1578	OK	387.741	9.36804	0	18.8048	OK	OK	0.291845
ENSG0000006	-	-	ENSG0000006812	'COASY	-	17:42536171-42566277	-	-	IGV_LINK	1130.69	34.2918	1.80598	66.7776	OK	1350.9	41.5708	1.1413	82.0002	OK	OK	0.277705
ENSG0000006	-	-	ENSG0000006813	'PLEKHH3	-	17:42667913-42676994	-	-	IGV_LINK	654.561	14.1565	3.31368	24.9994	OK	397.022	10.686	0.821315	20.5506	OK	OK	-0.40575
ENSG0000006	-	-	ENSG0000006830	'MEF2A	-	15:99565416-99733561	-	-	IGV_LINK	2056.78	30.4704	1.68233	59.2584	OK	2184.13	34.3177	1.83246	66.8029	OK	OK	0.171544
ENSG0000006	-	-	ENSG0000006830	'OTUD5	-	X:48922027-48958386	-	-	IGV_LINK	1125.85	30.9967	10.9283	51.0652	OK	1245.72	35.4227	10.4117	60.4338	OK	OK	0.192559
ENSG0000006	-	-	ENSG0000006832	'TFE3	-	X:49028725-49043486	-	-	IGV_LINK	1216.03	24.1166	5.80331	42.4298	OK	1438.56	28.0134	7.25475	48.772	OK	OK	0.216093
ENSG0000006	-	-	ENSG0000006835	'TBC1D25	-	X:48539456-48562609	-	-	IGV_LINK	439.283	8.24377	0.472068	16.0155	OK	414.552	7.41165	1.26848	13.5548	OK	OK	-0.15351
ENSG0000006	-	-	ENSG0000006836	'ACSL4	-	X:109623699-109733403	-	-	IGV_LINK	892.143	12.9108	0.747938	25.0736	OK	1402.47	26.1805	0.715371	51.6456	OK	OK	1.01992
ENSG0000006	-	-	ENSG0000006838	'INPP5A	-	10:132537819-132786052	-	-	IGV_LINK	263.764	8.05401	0	16.5686	OK	167.058	5.08748	0	10.3263	OK	OK	-0.66275
ENSG0000006	-	-	ENSG0000006839	'GPKOW	-	X:49113388-49123801	-	-	IGV_LINK	402.434	14.4503	5.24906	23.6515	OK	452.708	16.2674	6.10563	26.4291	OK	OK	0.170886
ENSG0000006	-	-	ENSG0000006840	'GRIPAP1	-	X:48973719-49002264	-	-	IGV_LINK	1543.8	38.9532	9.9479	67.9586	OK	1649.96	44.4566	10.2658	78.6473	OK	OK	0.190654
ENSG0000006	-	-	ENSG0000006843	'FTSJ1	-	X:48476020-48486364	-	-	IGV_LINK	378.191	17.8509	4.20243	31.4993	OK	425.896	18.5912	4.61303	32.5693	OK	OK	0.058625
ENSG0000006	-	-	ENSG0000006848	'PRR11	-	17:59155498-59215247	-	-	IGV_LINK	106.669	3.00337	0	7.38045	OK	100.029	4.2546	0	24.4401	OK	OK	0.502441
ENSG0000006	-	-	ENSG0000006861	'REEP1	-	2:86213992-86338083	-	-	IGV_LINK	22.3036	0.34126	0	0.977152	OK	166.027	2.89855	0.20544	5.59167	OK	OK	3.08639
ENSG0000006	-	-	ENSG0000006865	'ATP11A	-	13:112690328-112887168	-	-	IGV_LINK	5903.66	64.6922	0	147.747	OK	2633.75	25.9957	0	55.2038	OK	OK	-1.31532
ENSG0000006	-	-	ENSG0000006865	'POLR1A	-	2:86020215-86106155	-	-	IGV_LINK	577.953	4.05005	0	8.89737	OK	751.763	5.60153	0	11.909	OK	OK	0.467881
ENSG0000006	-	-	ENSG0000006869	'LAPTM4A	-	2:20032649-20052028	-	-	IGV_LINK	4415.14	160.231	62.3149	258.147	OK	5701.64	203.866	77.3037	330.429	OK	OK	0.347472
ENSG0000006	-	-	ENSG0000006872	'TTC7A	-	2:46899274-47176601	-	-	IGV_LINK	918.325	15.0985	0	53.8724	OK	1018.85	16.6646	0	57.2247	OK	OK	0.142386
ENSG0000006	-	-	ENSG0000006874	'IP6K2	-	3:48688002-48740353	-	-	IGV_LINK	1131.66	53.3418	19.0444	87.6392	OK	1346.78	60.5763	23.7629	97.3896	OK	OK	0.183486
ENSG0000006	-	-	ENSG0000006878	'STON1-GTF2A1L	-	2:48529382-48776517	-	-	IGV_LINK	5.81832	2.26706	0	15.3899	OK	9.92E-27	1.66E-28	0	0.478748	OK	OK	-93.4606
ENSG0000006	-	-	ENSG0000006878	'SRBD1	-	2:45388679-45612165	-	-	IGV_LINK	287.037	5.72966	0.054584	11.4047	OK	284.618	5.14015	0.688698	9.59161	OK	OK	-0.15664

ENSG0000006	-	-	ENSG0000006879	'KIF2A	-	5:62306161-62628582	-	-	IGV_LINK	429.586	9.34323	0.584389	18.1021	OK	1293.16	30.184	2.98271	57.3853	OK	OK	1.69179
ENSG0000006	-	-	ENSG0000006883	'RASGRP2	-	11:64726910-64745456	-	-	IGV_LINK	419.889	14.8269	3.52365	26.1302	OK	400.115	15.256	3.53996	26.9721	OK	OK	0.041159
ENSG0000006	-	-	ENSG0000006887	'PSME4	-	2:53864066-54305300	-	-	IGV_LINK	871.779	14.6193	0	30.7925	OK	973.476	17.2725	1.65862	32.8863	OK	OK	0.240595
ENSG0000006	-	-	ENSG0000006888	'IFT80	-	3:160225495-160485773	-	-	IGV_LINK	1158.08	31.0996	0	68.619	OK	1041.84	29.8515	0	64.5495	OK	OK	-0.05909
ENSG0000006	-	-	ENSG0000006890	'SIRT2	-	19:38878554-38908893	-	-	IGV_LINK	1268.39	41.8887	9.0926	74.6848	OK	1464.34	50.1899	12.0342	88.3457	OK	OK	0.260838
ENSG0000006	-	-	ENSG0000006891	'ERLEC1	-	2:53532671-53860160	-	-	IGV_LINK	819.414	25.1189	3.07096	47.1669	OK	1157.03	37.066	5.38276	68.7492	OK	OK	0.56132
ENSG0000006	-	-	ENSG0000006897	'PPP2R5B	-	11:64917552-64935888	-	-	IGV_LINK	340.372	9.17839	0.26371	18.0931	OK	477.457	12.2874	1.33863	23.2362	OK	OK	0.420868
ENSG0000006	-	-	ENSG0000006897	'PYGM	-	11:64746388-64760297	-	-	IGV_LINK	64.9713	1.58921	0	3.82846	OK	11.3435	0.452248	0	1.43515	OK	OK	-1.81312
ENSG0000006	-	-	ENSG0000006898	'PAGE1	-	X:49687449-49695993	-	-	IGV_LINK	0	0	0	0	OK	0	0	0	0	OK	NOTEST	0
ENSG0000006	-	-	ENSG0000006901	'PITX1	-	5:135027734-135401296	-	-	IGV_LINK	0.969721	0.024468	0	0.823987	OK	43.3114	1.19759	0	6.7109	OK	OK	5.61308
ENSG0000006	-	-	ENSG0000006901	'TRPC7	-	5:136213319-136365537	-	-	IGV_LINK	1.93944	0.114311	0	0.40351	OK	5.15612	0.361936	0	0.943187	OK	NOTEST	1.66277
ENSG0000006	-	-	ENSG0000006902	'MAST4	-	5:66596360-67169595	-	-	IGV_LINK	1377	11.6514	0	24.2881	OK	1859.3	21.6224	0	58.0406	OK	OK	0.892028
ENSG0000006	-	-	ENSG0000006912	'ADGRF5	-	6:46852511-46954943	-	-	IGV_LINK	1633.98	16.7038	6.98247	26.4252	OK	1615.93	16.4147	7.06938	25.7601	OK	OK	-0.02519
ENSG0000006	-	-	ENSG0000006918	'SDK2	-	17:73334383-73644089	-	-	IGV_LINK	30.0613	0.21122	0	0.693353	OK	193.87	1.24019	0.181492	2.29889	OK	OK	2.55374
ENSG0000006	-	-	ENSG0000006920	'ADAM7	-	8:24294039-24912073	-	-	IGV_LINK	0	0	0	0	OK	7.21857	0.127392	0	1.23745	OK	NOTEST	inf
ENSG0000006	-	-	ENSG0000006924	'NUP133	-	1:229440259-229508341	-	-	IGV_LINK	877.597	11.3886	2.44878	20.3285	OK	695.046	9.07911	1.41084	16.7474	OK	OK	-0.32697
ENSG0000006	-	-	ENSG0000006927	'NUCKS1	-	1:205712818-205750276	-	-	IGV_LINK	3259.23	58.5453	0	224.262	OK	2660.56	33.8367	0	121.572	OK	OK	-0.79097
ENSG0000006	-	-	ENSG0000006932	'VPS35	-	16:46656131-46689518	-	-	IGV_LINK	1767.8	29.0398	2.51192	55.5676	OK	1824.24	34.1747	5.42058	62.9289	OK	OK	0.2349
ENSG0000006	-	-	ENSG0000006934	'DNAJA2	-	16:46955361-46973788	-	-	IGV_LINK	1125.85	31.7054	12.9258	50.485	OK	1102.38	31.6676	11.8775	51.4578	OK	OK	-0.00172
ENSG0000006	-	-	ENSG0000006939	'BCL3	-	19:44747704-44760044	-	-	IGV_LINK	631.288	30.9722	4.75584	57.1886	OK	1197.25	52.4721	12.8074	92.1368	OK	OK	0.760576
ENSG0000006	-	-	ENSG0000006942	'KCNAB2	-	1:5862810-6101193	-	-	IGV_LINK	1346.94	25.6677	4.38593	46.9495	OK	1477.75	28.2239	3.18844	53.2594	OK	OK	0.136964
ENSG0000006	-	-	ENSG0000006943	'ABCC9	-	12:21797400-21942529	-	-	IGV_LINK	378.191	3.38756	0.483708	6.29141	OK	197.995	1.42146	0.237071	2.60585	OK	OK	-1.25287
ENSG0000006	-	-	ENSG0000006948	'GAL	-	11:68683778-68691175	-	-	IGV_LINK	0	0	0	0	OK	0	0	0	0	OK	NOTEST	0
ENSG0000006	-	-	ENSG0000006949	'CLEC2D	-	12:9664968-9699555	-	-	IGV_LINK	1077.1	32.1145	0	64.7338	OK	668.637	18.3371	0.04098	36.6333	OK	OK	-0.80846
ENSG0000006	-	-	ENSG0000006950	'FUNDC1	-	X:44523638-44543001	-	-	IGV_LINK	205.581	11.3199	3.0608	19.579	OK	280.493	15.4622	4.94852	25.9758	OK	OK	0.449885
ENSG0000006	-	-	ENSG0000006953	'MAOB	-	X:43766610-43882447	-	-	IGV_LINK	3130.26	73.7991	31.9163	115.682	OK	5304.62	126.641	46.7649	206.518	OK	OK	0.779073
ENSG0000006	-	-	ENSG0000006966	'RORA	-	15:60479177-61229319	-	-	IGV_LINK	591.53	7.30345	0	18.3992	OK	431.052	6.22099	0	16.0606	OK	OK	-0.23143
ENSG0000006	-	-	ENSG0000006969	'DRD4	-	11:637292-640706	-	-	IGV_LINK	20.3641	0.95439	0	2.04955	OK	7.21857	0.338631	0	0.894698	OK	OK	-1.49486
ENSG0000006	-	-	ENSG0000006970	'TGFBR3	-	1:91680342-91906335	-	-	IGV_LINK	504.255	5.68447	0.372936	10.996	OK	624.922	6.22711	1.16486	11.2894	OK	OK	0.131539
ENSG0000006	-	-	ENSG0000006971	'KIAA1107	-	1:92080304-92184725	-	-	IGV_LINK	98.3923	1.3675	0	2.83039	OK	114.461	1.54711	0.098618	2.9956	OK	OK	0.178029
ENSG0000006	-	-	ENSG0000006976	'PLA2G10	-	16:14672544-14694669	-	-	IGV_LINK	0	0	0	0	OK	0	0	0	0	OK	NOTEST	0
ENSG0000006	-	-	ENSG0000006981	'HES2	-	1:6412417-6424670	-	-	IGV_LINK	28.1219	0.720518	0	1.87278	OK	28.8743	0.439239	0	1.12757	OK	OK	-0.71403
ENSG0000006	-	-	ENSG0000006984	'ATP1B3	-	3:141876123-141926514	-	-	IGV_LINK	799.05	35.4669	11.275	59.6588	OK	734.232	33.5032	10.924	56.0823	OK	OK	-0.08218
ENSG0000006	-	-	ENSG0000006986	'NEDD4	-	15:55826921-55993746	-	-	IGV_LINK	782.564	23.4932	0	49.6764	OK	1175.6	26.7251	0	59.3736	OK	OK	0.185951
ENSG0000006	-	-	ENSG0000006994	'PIGB	-	15:55202965-55508234	-	-	IGV_LINK	239.521	11.1677	0	33.6782	OK	237.182	9.46188	0	31.6149	OK	OK	-0.23913
ENSG0000006	-	-	ENSG0000006995	'MAPK6	-	15:51829627-52100523	-	-	IGV_LINK	335.523	3.88881	0	10.7485	OK	753.825	9.96466	0	26.0042	OK	OK	1.35749
ENSG0000006	-	-	ENSG0000006996	'GNB5	-	15:52115104-52295798	-	-	IGV_LINK	349.099	9.01163	0.690641	17.3326	OK	456.833	12.1448	2.30404	21.9856	OK	OK	0.430484
ENSG0000006	-	-	ENSG0000006997	'RAB27A	-	15:55202965-55508234	-	-	IGV_LINK	487.161	19.8439	0	47.5153	OK	366.085	14.8805	0	43.611	OK	OK	-0.41527
ENSG0000006	-	-	ENSG0000006999	'HDHD5	-	22:17137510-17165445	-	-	IGV_LINK	576.014	19.7345	6.74972	32.7193	OK	706.389	24.5333	8.58479	40.4819	OK	OK	0.314023
ENSG0000007	-	-	ENSG0000007001	'UFD1	-	22:19330697-19479215	-	-	IGV_LINK	876.627	34.4642	0	79.6125	OK	812.605	39.5732	0	102.84	OK	OK	0.199425
ENSG0000007	-	-	ENSG0000007001	'LRP6	-	12:12049843-12267012	-	-	IGV_LINK	718.563	5.43465	0	12.2623	OK	555.83	3.98098	0	8.57466	OK	OK	-0.44906
ENSG0000007	-	-	ENSG0000007001	'GUCY2C	-	12:14503660-14757963	-	-	IGV_LINK	3.87888	0.059539	0	0.618671	OK	5.15612	0.079169	0	0.729953	OK	NOTEST	0.411098
ENSG0000007	-	-	ENSG0000007003	'SCT	-	11:626430-627143	-	-	IGV_LINK	0	0	0	0	OK	0	0	0	0	OK	NOTEST	0
ENSG0000007	-	-	ENSG0000007004	'PHRF1	-	11:576485-612222	-	-	IGV_LINK	795.171	8.98094	3.00492	14.957	OK	1198.28	14.2486	4.12748	24.3696	OK	OK	0.665877
ENSG0000007	-	-	ENSG0000007006	'ELP1	-	9:108867516-108934116	-	-	IGV_LINK	1061.84	16.6585	0	33.9599	OK	950.789	12.312	4.97321	19.6509	OK	OK	-0.43619
ENSG0000007	-	-	ENSG0000007008	'NUCB2	-	11:17208152-17349974	-	-	IGV_LINK	825.232	44.1003	12.886	75.3146	OK	745.576	39.5965	11.738	67.4549	OK	OK	-0.15542
ENSG0000007	-	-	ENSG0000007008	'PFN2	-	3:149761099-150225190	-	-	IGV_LINK	148.367	6.85417	0	19.6574	OK	648.641	29.768	0	64.2728	OK	OK	2.11871
ENSG0000007	-	-	ENSG0000007015	'PTPN3	-	9:109375465-109498313	-	-	IGV_LINK	1441	17.6286	0	38.0465	OK	1163.22	13.2922	0	28.0413	OK	OK	-0.40734
ENSG0000007	-	-	ENSG0000007018	'SPTB	-	14:64704101-64879883	-	-	IGV_LINK	84.3657	0.769811	0	2.87157	OK	136.122	1.06348	0	3.59709	OK	OK	0.46622
ENSG0000007	-	-	ENSG0000007019	'DAPP1	-	4:99816832-99870154	-	-	IGV_LINK	417.95	12.0422	3.13669	20.9477	OK	315.555	9.98272	2.56952	17.3959	OK	OK	-0.27059
ENSG0000007	-	-	ENSG0000007019	'FGF10	-	5:44303543-44413989	-	-	IGV_LINK	0	0	0	0	OK	0	0	0	0	OK	NOTEST	0
ENSG0000007	-	-	ENSG0000007021	'SLC44A1	-	9:105244621-105439171	-	-	IGV_LINK	1081.24	12.942	3.09942	22.7846	OK	870.354	9.92289	3.24641	16.5994	OK	OK	-0.38323
ENSG0000007	-	-	ENSG0000007026	'TMEM260	-	14:56488353-56730535	-	-	IGV_LINK	320.008	6.01639	0	12.4653	OK	272.243	4.64332	0	9.35067	OK	OK	-0.37374
ENSG0000007	-	-	ENSG0000007036	'SMG6	-	17:2054153-2381058	-	-	IGV_LINK	711.775	11.7364	0	30.4918	OK	678.546	12.3831	0	34.996	OK	OK	0.077378
ENSG0000007	-	-	ENSG0000007036	'EXOC5	-	14:57200506-57298742	-	-	IGV_LINK	448.981	9.36605	0	21.2395	OK	426.927	8.85675	0	19.8806	OK	OK	-0.08066
ENSG0000007	-	-	ENSG0000007037	'CLTCL1	-	22:19179472-19291716	-	-	IGV_LINK	103.76	2.0787	0	5.1796	OK	91.779	1.25274	0	3.01492	OK	OK	-0.73059
ENSG0000007	-	-	ENSG0000007038	'FGF22	-	19:639878-644371	-	-	IGV_LINK	0.969721	0.048967	0	0.236009	OK	1.03122	0.052127	0	0.240158	OK	NOTEST	0.09023
ENSG0000007	-	-	ENSG0000007040	'FSTL3	-	19:676364-683399	-	-	IGV_LINK	1003.66	28.3391	9.57516	47.103	OK	725.982	20.5292	6.28968	34.7687	OK	OK	-0.46512
ENSG0000007	-	-	ENSG0000007041	'DGCR2	-	22:19036281-19128449	-	-	IGV_LINK	1504.04	21.0119	6.06467	35.9592	OK	1715.96	25.3029	5.34368	45.2622	OK	OK	0.268095
ENSG0000007	-	-	ENSG0000007042	'RNF126	-	19:647525-663277	-	-	IGV_LINK	435.405	19.0278	5.57719	32.4784	OK	605.329	26.8274	8.4151	45.2398	OK	OK	0.4956
ENSG0000007	-	-	ENSG0000007044	'MNT	-	17:2384059-2401118	-	-	IGV_LINK	351.039	7.00403	0	15.245	OK	422.802	9.78228	0	21.5819	OK	OK	0.481986
ENSG0000007	-	-	ENSG0000007047	'ZXDC	-	3:126437600-126475919	-	-	IGV_LINK	893.113	15.4385	5.73059	25.1463	OK	823.949	14.201	5.18346	23.2186	OK	OK	-0.12053
ENSG0000007	-	-	ENSG0000007049	'JMJD6	-	17:76712831-76726799	-	-	IGV_LINK	410.192	16.476	2.74224	30.2098	OK	430.021	18.1515	3.56402	32.739	OK	OK	0.139722
ENSG0000007	-	-	ENSG0000007050	'POLB	-	8:42338453-42371808	-	-	IGV_LINK	334.554	20.9606	6.11058	35.8107	OK	179.433	11.7622	1.89445	21.6299	OK	OK	-0.83353
ENSG0000007	-	-	ENSG0000007052	'ST6GALNAC1	-	17:76624760-76643838	-	-	IGV_LINK	0	0	0	0	OK	8.2498	0.426597	0	1.31246	OK	NOTEST	inf
ENSG0000007	-	-	ENSG0000007054	'WIPI1	-	17:68259181-68763882	-	-	IGV_LINK	450.92	23.1793	0	74.0037	OK	614.61	33.0878	0	116.689	OK	OK	0.51346
ENSG0000007	-	-	ENSG0000007060	'FRMPD1	-	9:37510891-38069211	-	-	IGV_LINK	18.4247	0.372366	0	5.0427	OK	26.8118	0.314965	0	2.0688	OK	NOTEST	-0.24153
ENSG0000007	-	-	ENSG0000007061	'GBA2	-	9:35696947-35749228	-	-	IGV_LINK	2017.99	68.8556	0	203.257	OK	2102.67	70.5297	0	218.65	OK	OK	0.034656
ENSG0000007	-	-	ENSG0000007061	'NDST1	-	5:150475530-150558211	-	-	IGV_LINK	1341.12	12.4503	0	25.7865	OK	1760.3	14.5058	0.552233	28.4594	OK	OK	0.220453
ENSG0000007	-	-	ENSG0000007066	'ASNS	-	7:97851687-97972985	-	-	IGV_LINK	345.769	13.2676	1.07944	25.4557	OK	232.994	9.81907	0	20.3385	OK	OK	-0.43425
ENSG0000007	-	-	ENSG0000007071	'AP3M2	-	8:42152945-42171673	-	-	IGV_LINK	560.498	11.8128	1.88805	21.7375	OK	498.082	11.1607	1.38709	20.9342	OK	OK	-0.08192
ENSG0000007	-	-	ENSG0000007072	'CNGB1	-	16:57882339-57971116	-	-	IGV_LINK	0.969721	0.077795	0	0.661415	OK	0	0	0	0	OK	NOTEST	'-inf
ENSG0000007	-	-	ENSG0000007073	'ST6GALNAC2	-	17:76557765-76586956	-	-	IGV_LINK	124.875	5.68864	0	20.4923	OK	157.141	6.27059	0	21.7001	OK	OK	0.140519
ENSG0000007	-	-	ENSG0000007074	'CHAT	-	10:49609094-49665104	-	-	IGV_LINK	0	0	0	0	OK	0	0	0	0	OK	NOTEST	0
ENSG0000007	-	-	ENSG0000007075	'PABPC1	-	8:100685815-100722809	-	-	IGV_LINK	13963	629.894	48.7248	1211.06	OK	13949.4	636.588	53.4115	1219.76	OK	OK	0.015252
ENSG0000007	-	-	ENSG0000007075	'TESK2	-	1:45329162-45491166	-	-	IGV_LINK	167.762	3.25997	0	7.13049	OK	89.7166	2.00852	0	6.53061	OK	OK	-0.69873
ENSG0000007	-	-	ENSG0000007076	'CFAP20	-	16:58113587-58129450	-	-	IGV_LINK	362.675	19.882	3.02753	36.7364	OK	427.958	25.871	2.35457	49.3874	OK	OK	0.379875
ENSG0000007	-	-	ENSG0000007077	'CSNK2A2	-	16:58129528-58217805	-	-	IGV_LINK	1166.57	24.1121	7.74135	40.4828	OK	1338.53	27.0116	9.33574	44.6875	OK	OK	0.163826
ENSG0000007	-	-	ENSG0000007077	'PTPN21	-	14:88384923-88554733	-	-	IGV_LINK	178.429	2.23533	0	5.39801	OK	185.62	2.19946	0	4.9198	OK	OK	-0.02333
ENSG0000007	-	-	ENSG0000007078	'EIF2B3	-	1:44850521-44986722	-	-	IGV_LINK	268.613	10.7985	3.11173	18.4853	OK	232.026	9.34701	2.52641	16.1676	OK	OK	-0.20826
ENSG0000007	-	-	ENSG0000007080	'CAMK2A	-	5:150189956-150290291	-	-	IGV_LINK	2.90916	0.039898	0	0.427962	OK	0	0	0	0	OK	NOTEST	'-inf
ENSG0000007	-	-	ENSG0000007081	'TCOF1	-	5:150357638-150400308	-	-	IGV_LINK	962.932	14.2363	2.78424	25.6883	OK	960.07	14.1811	2.23742	26.1248	OK	OK	-0.0056
ENSG0000007	-	-	ENSG0000007083	'CDC42	-	1:22052626-22092946	-	-	IGV_LINK	3005.16	93.4958	34.7107	152.281	OK	3299.92	104.491	36.2619	172.72	OK	OK	0.160405
ENSG0000007	-	-	ENSG0000007088	'OSBPL3	-	7:24796538-24981634	-	-	IGV_LINK	1004.63	16.138	0	33.6352	OK	1196.22	20.0772	0	41.5598	OK	OK	0.3151
ENSG0000007	-	-	ENSG0000007088	'EPHA8	-	1:22563563-22603594	-	-	IGV_LINK	0	0	0	0	OK	2.06245	0.03531	0	0.115827	OK	NOTEST	inf
ENSG0000007	-	-	ENSG0000007091	'SLC12A3	-	16:56865206-56915850	-	-	IGV_LINK	23.2733	0.299422	0	0.710048	OK	13.4059	0.158411	0	0.485512	OK	NOTEST	-0.91851
ENSG0000007	-	-	ENSG0000007095	'RAD18	-	3:8619399-8963773	-	-	IGV_LINK	125.094	1.23387	0	2.72713	OK	153.653	2.49619	0	6.59029	OK	OK	1.01653
ENSG0000007	-	-	ENSG0000007096	'ATP2B1	-	12:89561128-89712590	-	-	IGV_LINK	485.83	9.81501	0	20.7997	OK	659.984	11.4172	1.8311	21.0033	OK	OK	0.218148
ENSG0000007	-	-	ENSG0000007098	'TRPM5	-	11:2404514-2423045	-	-	IGV_LINK	0	0	0	0	OK	2.06245	0.03115	0	0.10919	OK	NOTEST	inf
ENSG0000007	-	-	ENSG0000007105	'NCK2	-	2:105744896-105894274	-	-	IGV_LINK	935.78	23.1884	7.29145	39.0853	OK	983.789	24.5769	9.81804	39.3358	OK	OK	0.083901
ENSG0000007	-	-	ENSG0000007105	'MAP4K4	-	2:101696849-101894689	-	-	IGV_LINK	1841.5	23.2087	4.62571	41.7918	OK	2735.84	35.905	7.13975	64.6703	OK	OK	0.629517
ENSG0000007	-	-	ENSG0000007107	'MGAT4A	-	2:98619105-98731126	-	-	IGV_LINK	1861.86	17.2788	3.4839	31.0737	OK	1178.69	12.7055	0	27.6776	OK	OK	-0.44355
ENSG0000007	-	-	ENSG0000007108	'RPL31	-	2:100820151-101270316	-	-	IGV_LINK	12848.8	2409.75	200.81	4618.68	OK	9463.55	1767.24	217.34	3317.13	OK	OK	-0.44739
ENSG0000007	-	-	ENSG0000007112	'WDR1	-	4:10074338-10116949	-	-	IGV_LINK	4216.34	90.7073	28.2206	153.194	OK	5363.4	121.396	30.6704	212.122	OK	OK	0.420434
ENSG0000007	-	-	ENSG0000007118	'SNX13	-	7:17790760-17940501	-	-	IGV_LINK	832.99	12.2934	3.74119	20.8455	OK	781.669	12.1368	1.8987	22.3749	OK	OK	-0.01849
ENSG0000007	-	-	ENSG0000007120	'MS4A12	-	11:60492777-60507430	-	-	IGV_LINK	0	0	0	0	OK	0	0	0	0	OK	NOTEST	0
ENSG0000007	-	-	ENSG0000007120	'ARHGAP10	-	4:147732062-148072780	-	-	IGV_LINK	388.858	8.08433	2.25121	13.9174	OK	557.893	11.7025	4.02466	19.3804	OK	OK	0.533621
ENSG0000007	-	-	ENSG0000007124	'RPS6KA2	-	6:166409363-166957191	-	-	IGV_LINK	649.713	6.65073	0	27.1775	OK	570.267	5.84231	0	20.0161	OK	OK	-0.18697
ENSG0000007	-	-	ENSG0000007124	'ING3	-	7:120950748-120977216	-	-	IGV_LINK	422.798	16.4596	4.59271	28.3265	OK	273.275	10.1634	1.73392	18.5928	OK	OK	-0.69555
ENSG0000007	-	-	ENSG0000007124	'VASH1	-	14:76762188-76826246	-	-	IGV_LINK	2211.42	35.1846	0	79.0678	OK	1310.05	24.5829	0	56.7915	OK	OK	-0.51729
ENSG0000007	-	-	ENSG0000007128	'LMCD1	-	3:7952804-8611924	-	-	IGV_LINK	147.398	7.43393	0	14.8965	OK	830.136	42.2777	12.2959	72.2594	OK	OK	2.5077
ENSG0000007	-	-	ENSG0000007146	'BUD23	-	7:73680968-73719672	-	-	IGV_LINK	864.991	40.358	7.56252	73.1534	OK	878.604	46.4677	7.5345	85.4009	OK	OK	0.203374
ENSG0000007	-	-	ENSG0000007153	'SEL1L	-	14:81471548-81533861	-	-	IGV_LINK	1611.68	17.6681	0	38.9136	OK	1531.37	13.8113	0	28.4972	OK	OK	-0.3553
ENSG0000007	-	-	ENSG0000007153	'TRIP13	-	5:795605-919357	-	-	IGV_LINK	96.0023	2.27121	0	6.79619	OK	129.934	3.47208	0	10.3601	OK	OK	0.61234
ENSG0000007	-	-	ENSG0000007155	'ATP6AP1	-	X:154428631-154436516	-	-	IGV_LINK	3132.2	87.5368	34.7831	140.291	OK	3385.51	94.6638	34.9301	154.398	OK	OK	0.112923
ENSG0000007	-	-	ENSG0000007156	'TCF3	-	19:1609289-1652605	-	-	IGV_LINK	792.262	20.4422	3.80698	37.0774	OK	721.857	19.9184	4.61791	35.2189	OK	OK	-0.03745
ENSG0000007	-	-	ENSG0000007157	'TRIB2	-	2:12716888-12742734	-	-	IGV_LINK	955.175	19.7704	5.34509	34.1957	OK	1058.04	21.4518	5.55394	37.3497	OK	OK	0.117756
ENSG0000007	-	-	ENSG0000007162	'DAZAP1	-	19:1407568-1435687	-	-	IGV_LINK	1925.86	51.5561	14.5978	88.5143	OK	1746.9	48.4726	11.5765	85.3687	OK	OK	-0.08897
ENSG0000007	-	-	ENSG0000007165	'MBD3	-	19:1573595-1605490	-	-	IGV_LINK	985.802	26.0182	0.150836	51.8856	OK	928.251	24.3262	0	50.0849	OK	OK	-0.09701
ENSG0000007	-	-	ENSG0000007167	'PRLH	-	2:237566573-237567175	-	-	IGV_LINK	0	0	0	0	OK	0	0	0	0	OK	NOTEST	0
ENSG0000007	-	-	ENSG0000007179	'HLTF	-	3:149030126-149102823	-	-	IGV_LINK	662.319	9.38802	0.871708	17.9043	OK	485.707	6.95008	0.09174	13.8084	OK	OK	-0.43379
ENSG0000007	-	-	ENSG0000007185	'FAM50A	-	X:154444125-154450654	-	-	IGV_LINK	1016.27	52.5622	19.1736	85.9509	OK	776.512	40.2301	13.3328	67.1274	OK	OK	-0.38575
ENSG0000007	-	-	ENSG0000007188	'FAM3A	-	X:154506158-154516242	-	-	IGV_LINK	658.44	25.2072	7.96209	42.4524	OK	630.078	24.7979	7.70893	41.8869	OK	OK	-0.02362
ENSG0000007	-	-	ENSG0000007189	'CPSF1	-	8:144373100-144409349	-	-	IGV_LINK	2253.63	57.954	0	116.048	OK	2229.51	60.9988	0.393591	121.604	OK	OK	0.073874
ENSG0000007	-	-	ENSG0000007190	'MYO3B	-	2:170178144-170695374	-	-	IGV_LINK	75.6382	0.809767	0.034418	1.58512	OK	190.777	2.02357	0.524781	3.52236	OK	OK	1.32133
ENSG0000007	-	-	ENSG0000007196	'CYBRD1	-	2:171522246-171558133	-	-	IGV_LINK	664.259	9.72024	3.23407	16.2064	OK	427.958	8.01756	1.7012	14.3339	OK	OK	-0.27783
ENSG0000007	-	-	ENSG0000007199	'CDH19	-	18:66501082-66604138	-	-	IGV_LINK	0	0	0	0	OK	0	0	0	0	OK	NOTEST	0
ENSG0000007	-	-	ENSG0000007199	'PDCD2	-	6:170575294-170584692	-	-	IGV_LINK	800.989	38.599	12.3668	64.8312	OK	468.176	23.685	4.77011	42.5999	OK	OK	-0.70459
ENSG0000007	-	-	ENSG0000007204	'SLC6A15	-	12:84859487-84913615	-	-	IGV_LINK	0	0	0	0	OK	0	0	0	0	OK	NOTEST	0
ENSG0000007	-	-	ENSG0000007204	'RDH11	-	14:67619797-67695814	-	-	IGV_LINK	680.744	21.2122	0	43.2098	OK	737.326	21.4194	0	54.2393	OK	OK	0.014025
ENSG0000007	-	-	ENSG0000007206	'PRKACA	-	19:14091687-14118084	-	-	IGV_LINK	873.718	30.65	10.7627	50.5373	OK	964.195	31.4879	11.0157	51.9602	OK	OK	0.038911
ENSG0000007	-	-	ENSG0000007207	'ADGRL1	-	19:14137178-14206187	-	-	IGV_LINK	181.338	3.96157	0	11.1717	OK	203.151	4.0532	0	11.384	OK	OK	0.03299
ENSG0000007	-	-	ENSG0000007208	'SPP2	-	2:234050678-234077134	-	-	IGV_LINK	4.8486	1.30674	0	3.19671	OK	3.09367	0.838675	0	2.19462	OK	OK	-0.63979
ENSG0000007	-	-	ENSG0000007211	'ACTN1	-	14:68874142-68979440	-	-	IGV_LINK	1467.9	29.5066	8.23529	50.7779	OK	1938.71	37.0109	11.8838	62.1381	OK	OK	0.326914
ENSG0000007	-	-	ENSG0000007212	'ZFYVE26	-	14:67701885-67816590	-	-	IGV_LINK	1018.21	7.29325	1.4781	13.1084	OK	959.039	6.83939	1.80564	11.8731	OK	OK	-0.09269
ENSG0000007	-	-	ENSG0000007213	'RPS6KA6	-	X:84058345-84187907	-	-	IGV_LINK	32.9705	0.58636	0	1.71811	OK	11.3435	0.171204	0	0.616029	OK	OK	-1.77607
ENSG0000007	-	-	ENSG0000007213	'EPN2	-	17:19215614-19336715	-	-	IGV_LINK	544.013	15.6363	0	39.3255	OK	467.145	10.7418	0	22.1417	OK	OK	-0.54166
ENSG0000007	-	-	ENSG0000007213	'PTPN18	-	2:130356006-130375409	-	-	IGV_LINK	2122.72	66.4178	11.9703	120.865	OK	2051.11	62.9699	12.6221	113.318	OK	OK	-0.07691
ENSG0000007	-	-	ENSG0000007216	'LIMS2	-	2:127638380-127681786	-	-	IGV_LINK	563.408	18.9731	6.29797	31.6483	OK	466.114	14.7181	4.18008	25.2561	OK	OK	-0.36637
ENSG0000007	-	-	ENSG0000007218	'ASIC4	-	2:219434845-219538772	-	-	IGV_LINK	3.87888	0.124534	0	1.66438	OK	4.1249	0.092602	0	1.00281	OK	NOTEST	-0.42743
ENSG0000007	-	-	ENSG0000007219	'SPEG	-	2:219434845-219538772	-	-	IGV_LINK	18.4247	1.04679	0	8.1469	OK	57.7486	1.77348	0	10.2207	OK	OK	0.760612
ENSG0000007	-	-	ENSG0000007220	'LNX1	-	4:53377642-54298247	-	-	IGV_LINK	127.033	2.80128	0	8.52032	OK	140.247	3.14293	0	9.91282	OK	OK	0.166021

ENSG0000007	-	-	ENSG0000007221	'ALDH3A2	-	17:19648135-19677598	-	-	IGV_LINK	2433.03	54.8662	13.7387	95.9936	OK	2300.66	52.9042	14.3203	91.4881	OK	OK	-0.05253
ENSG0000007	-	-	ENSG0000007227	'TFRC	-	3:196027182-196082189	-	-	IGV_LINK	943.538	17.8509	0	38.4621	OK	1213.75	17.8485	0.520332	35.1768	OK	OK	-0.00019
ENSG0000007	-	-	ENSG0000007231	'SREBF1	-	17:17681472-17837002	-	-	IGV_LINK	1316.88	37.4864	0	79.7974	OK	2757.5	85.0303	0	176.948	OK	OK	1.18161
ENSG0000007	-	-	ENSG0000007231	'TRPC5	-	X:111774314-112082776	-	-	IGV_LINK	0.969721	0.009644	0	0.044096	OK	2.06245	0.020516	0	0.097568	OK	NOTEST	1.08901
ENSG0000007	-	-	ENSG0000007236	'AFF4	-	5:132875378-132963634	-	-	IGV_LINK	2299.21	14.8659	5.16908	24.5627	OK	1954.17	12.1607	4.80761	19.5138	OK	OK	-0.28978
ENSG0000007	-	-	ENSG0000007240	'UBE2D1	-	10:58334974-58370753	-	-	IGV_LINK	128.003	3.30872	0.24923	6.36822	OK	122.716	3.21587	0.181546	6.25018	OK	OK	-0.04107
ENSG0000007	-	-	ENSG0000007241	'MPP5	-	14:67241108-67386516	-	-	IGV_LINK	315.159	3.66752	0	9.61846	OK	361.96	4.68443	0	13.4957	OK	OK	0.353067
ENSG0000007	-	-	ENSG0000007242	'RHOBTB1	-	10:60869437-61001440	-	-	IGV_LINK	1231.55	21.8145	0	45.2965	OK	1492.18	25.324	0.219261	50.4288	OK	OK	0.215221
ENSG0000007	-	-	ENSG0000007250	'SMC1A	-	X:53374148-53431120	-	-	IGV_LINK	1614.58	14.0959	0	30.0086	OK	1525.18	14.43	0	31.2044	OK	OK	0.033803
ENSG0000007	-	-	ENSG0000007250	'HSD17B10	-	X:53431257-53434373	-	-	IGV_LINK	560.498	40.4478	15.9734	64.9222	OK	662.046	47.5717	19.0158	76.1276	OK	OK	0.234043
ENSG0000007	-	-	ENSG0000007251	'MARK2	-	11:63838927-63911019	-	-	IGV_LINK	789.352	13.4621	3.88028	23.044	OK	844.573	15.2069	3.60338	26.8104	OK	OK	0.175821
ENSG0000007	-	-	ENSG0000007257	'HMMR	-	5:163460202-163494058	-	-	IGV_LINK	61.0924	1.39935	0	3.07888	OK	103.122	2.70463	0	6.14994	OK	OK	0.950671
ENSG0000007	-	-	ENSG0000007260	'CHFR	-	12:132768908-132956306	-	-	IGV_LINK	692.38	18.112	0	45.2944	OK	665.142	20.0203	0	52.4271	OK	OK	0.144517
ENSG0000007	-	-	ENSG0000007265	'TRHDE	-	12:71938845-72670757	-	-	IGV_LINK	248.248	3.00799	0	9.68445	OK	217.588	3.31901	0	11.5939	OK	OK	0.141952
ENSG0000007	-	-	ENSG0000007268	'P4HA2	-	5:132184875-132395614	-	-	IGV_LINK	891.173	26.7842	0	63.8684	OK	1724.21	52.6081	0	106.148	OK	OK	0.973904
ENSG0000007	-	-	ENSG0000007269	'FCGR2B	-	1:161663146-161678654	-	-	IGV_LINK	594.439	15.1499	3.33583	26.9641	OK	900.259	23.573	6.8879	40.2581	OK	OK	0.637822
ENSG0000007	-	-	ENSG0000007273	'NFATC3	-	16:67987393-68238102	-	-	IGV_LINK	530.299	9.66721	0	20.8511	OK	540.876	10.2951	0	24.8084	OK	OK	0.090793
ENSG0000007	-	-	ENSG0000007275	'TRNT1	-	3:3126915-3179727	-	-	IGV_LINK	171.641	8.87164	0	25.8589	OK	161.902	5.9348	0	15.478	OK	OK	-0.58
ENSG0000007	-	-	ENSG0000007277	'ACADVL	-	17:7189889-7225273	-	-	IGV_LINK	5941.48	315.618	77.1138	554.123	OK	8585.98	452.258	94.3569	810.159	OK	OK	0.518966
ENSG0000007	-	-	ENSG0000007278	'STK10	-	5:172042072-172188386	-	-	IGV_LINK	1566.1	25.6989	0	53.1096	OK	1631.4	23.2965	0	46.8535	OK	OK	-0.1416
ENSG0000007	-	-	ENSG0000007280	'FBXW11	-	5:171861548-172006873	-	-	IGV_LINK	1246.09	25.6918	3.25858	48.125	OK	1203.44	21.9986	4.09048	39.9067	OK	OK	-0.2239
ENSG0000007	-	-	ENSG0000007281	'ACAP1	-	17:7336528-7351478	-	-	IGV_LINK	945.477	33.3588	7.10709	59.6105	OK	765.169	27.0717	3.22239	50.921	OK	OK	-0.30128
ENSG0000007	-	-	ENSG0000007283	'CRMP1	-	4:5711196-5893058	-	-	IGV_LINK	548.862	13.6307	0	30.8667	OK	271.212	6.50536	0	14.8082	OK	OK	-1.06715
ENSG0000007	-	-	ENSG0000007284	'EVC	-	4:5711196-5893058	-	-	IGV_LINK	2014.11	18.56	5.02023	32.0997	OK	1139.5	10.6203	2.95831	18.2823	OK	OK	-0.80537
ENSG0000007	-	-	ENSG0000007284	'DERL2	-	17:5471250-5490814	-	-	IGV_LINK	371.403	23.8476	0.554302	47.1408	OK	393.928	24.2429	0.586981	47.8989	OK	OK	0.023723
ENSG0000007	-	-	ENSG0000007285	'SIDT1	-	3:113532295-113629578	-	-	IGV_LINK	101.821	2.04962	0	5.45372	OK	112.404	4.22549	0	10.5978	OK	OK	1.04376
ENSG0000007	-	-	ENSG0000007286	'NDE1	-	16:15643266-15857033	-	-	IGV_LINK	376.252	14.2643	0	45.139	OK	334.117	12.777	0	29.6103	OK	OK	-0.15885
ENSG0000007	-	-	ENSG0000007295	'MRVI1	-	11:10541271-10693988	-	-	IGV_LINK	386.918	4.5137	0	10.028	OK	266.056	2.79678	0	5.97147	OK	OK	-0.69054
ENSG0000007	-	-	ENSG0000007295	'TMEM38A	-	19:16661126-16690029	-	-	IGV_LINK	69.8199	2.40408	0	5.79671	OK	31.968	0.956445	0	2.30953	OK	OK	-1.32973
ENSG0000007	-	-	ENSG0000007295	'AP1M1	-	19:16197577-16245907	-	-	IGV_LINK	971.66	32.7179	8.15159	57.2841	OK	1476.71	49.4663	15.7797	83.153	OK	OK	0.596368
ENSG0000007	-	-	ENSG0000007300	'PVR	-	19:44537057-44718759	-	-	IGV_LINK	331.547	6.28254	1.25313	11.3119	OK	614.61	11.8555	3.39313	20.3179	OK	OK	0.916139
ENSG0000007	-	-	ENSG0000007305	'XRCC1	-	19:43543039-43596135	-	-	IGV_LINK	632.121	22.9829	3.83099	42.1347	OK	556.861	20.9909	1.83346	40.1484	OK	OK	-0.13079
ENSG0000007	-	-	ENSG0000007306	'SCARB1	-	12:124776855-124882668	-	-	IGV_LINK	5226.79	113.578	30.3312	196.826	OK	5107.66	115.862	36.2698	195.453	OK	OK	0.028713
ENSG0000007	-	-	ENSG0000007306	'CYP2W1	-	7:983198-989640	-	-	IGV_LINK	0.969721	0.025629	0	0.356287	OK	1.03122	0.026569	0	0.356299	OK	NOTEST	0.051962
ENSG0000007	-	-	ENSG0000007311	'MCM2	-	3:127573068-127622436	-	-	IGV_LINK	541.104	9.26941	1.45885	17.08	OK	520.769	9.05133	1.21548	16.8872	OK	OK	-0.03435
ENSG0000007	-	-	ENSG0000007314	'MOV10L1	-	22:50089878-50161690	-	-	IGV_LINK	3.87888	0.119394	0	0.478355	OK	8.2498	0.282539	0	0.759626	OK	NOTEST	1.24271
ENSG0000007	-	-	ENSG0000007315	'PANX2	-	22:50170730-50180294	-	-	IGV_LINK	4.8486	0.094927	0	0.244707	OK	221.713	4.64751	1.10697	8.18804	OK	OK	5.61349
ENSG0000007	-	-	ENSG0000007316	'SELENOO	-	22:50200978-50217616	-	-	IGV_LINK	463.526	16.5087	5.64834	27.3691	OK	419.709	14.9283	4.88356	24.973	OK	OK	-0.14518
ENSG0000007	-	-	ENSG0000007328	'TP63	-	3:189631415-189897279	-	-	IGV_LINK	9.69721	0.172057	0	0.592406	OK	3.09367	0.052872	0	0.394568	OK	NOTEST	-1.70231
ENSG0000007	-	-	ENSG0000007333	'ALPK1	-	4:112274541-112442620	-	-	IGV_LINK	1157.85	18.0064	0	38.0718	OK	1167.35	17.6968	1.68743	33.7061	OK	OK	-0.02502
ENSG0000007	-	-	ENSG0000007335	'LLGL2	-	17:75525079-75575208	-	-	IGV_LINK	1032.75	31.3299	0	63.0441	OK	456.833	15.1353	1.22033	29.0502	OK	OK	-1.04963
ENSG0000007	-	-	ENSG0000007341	'PDE8A	-	15:84980439-85139145	-	-	IGV_LINK	996.873	21.4307	4.70493	38.1565	OK	1261.19	25.1462	5.50062	44.7919	OK	OK	0.230662
ENSG0000007	-	-	ENSG0000007346	'CLCN4	-	X:10156944-10237660	-	-	IGV_LINK	339.402	3.83052	0	8.69845	OK	553.768	5.41645	0.042924	10.79	OK	OK	0.499808
ENSG0000007	-	-	ENSG0000007353	'NLE1	-	17:35121578-35142315	-	-	IGV_LINK	415.04	10.4	1.87556	18.9245	OK	486.738	10.782	2.73373	18.8302	OK	OK	0.052034
ENSG0000007	-	-	ENSG0000007357	'SDHA	-	5:218240-256700	-	-	IGV_LINK	3552.09	109.607	25.5374	193.677	OK	3493.79	98.9027	31.177	166.628	OK	OK	-0.14826
ENSG0000007	-	-	ENSG0000007358	'SMARCE1	-	17:40624961-40665181	-	-	IGV_LINK	1601.15	62.0029	17.0925	106.913	OK	1440.6	61.0636	16.4154	105.712	OK	OK	-0.02202
ENSG0000007	-	-	ENSG0000007359	'FNDC8	-	17:35121578-35142315	-	-	IGV_LINK	0.969721	0.044961	0	0.638722	OK	0	0	0	0	OK	NOTEST	'-inf
ENSG0000007	-	-	ENSG0000007360	'GSDMB	-	17:39904594-39919854	-	-	IGV_LINK	574.075	15.8858	3.36408	28.4075	OK	467.145	14.6838	2.10397	27.2637	OK	OK	-0.11351
ENSG0000007	-	-	ENSG0000007361	'KDM5A	-	12:280128-442645	-	-	IGV_LINK	1114.21	9.68595	0	26.5128	OK	1029.16	8.64623	0	24.3068	OK	OK	-0.16382
ENSG0000007	-	-	ENSG0000007367	'ADAM11	-	17:44759030-44781846	-	-	IGV_LINK	30.0613	0.804818	0	2.23275	OK	35.0616	0.44935	0	0.981907	OK	OK	-0.84082
ENSG0000007	-	-	ENSG0000007371	'PPP2R3A	-	3:135965672-136147891	-	-	IGV_LINK	802.929	9.36422	2.50809	16.2204	OK	1358.12	16.229	4.33993	28.118	OK	OK	0.793341
ENSG0000007	-	-	ENSG0000007371	'FERMT2	-	14:52857267-52952435	-	-	IGV_LINK	480.012	9.54738	2.59081	16.504	OK	550.674	11.035	3.76297	18.307	OK	OK	0.208907
ENSG0000007	-	-	ENSG0000007373	'ABCB11	-	2:168922937-169031322	-	-	IGV_LINK	0	0	0	0	OK	0	0	0	0	OK	NOTEST	0
ENSG0000007	-	-	ENSG0000007373	'DHRS9	-	2:169064788-169096167	-	-	IGV_LINK	112.488	4.77367	0.009005	9.53833	OK	58.7798	2.43241	0	4.87557	OK	OK	-0.97271
ENSG0000007	-	-	ENSG0000007375	'CD5L	-	1:157830913-157898256	-	-	IGV_LINK	2.90916	0.081223	0	0.343375	OK	3.09367	0.086424	0	0.348363	OK	NOTEST	0.089541
ENSG0000007	-	-	ENSG0000007375	'PTGS2	-	1:186671790-186680427	-	-	IGV_LINK	117.336	2.64218	0	5.44719	OK	261.931	5.79346	0.80779	10.7791	OK	OK	1.13269
ENSG0000007	-	-	ENSG0000007379	'IGF2BP2	-	3:185643738-185825056	-	-	IGV_LINK	39.7585	1.22258	0	3.79826	OK	35.0616	0.69857	0	2.18864	OK	OK	-0.80745
ENSG0000007	-	-	ENSG0000007380	'MAP3K13	-	3:185282940-185499057	-	-	IGV_LINK	682.683	13.775	0	27.8209	OK	835.292	15.4823	0	32.8	OK	OK	0.168571
ENSG0000007	-	-	ENSG0000007384	'ST6GAL1	-	3:186930484-187078553	-	-	IGV_LINK	2311.81	37.6769	4.9463	70.4075	OK	6800.93	137.967	0	282.251	OK	OK	1.87258
ENSG0000007	-	-	ENSG0000007386	'TBX21	-	17:47733243-47746119	-	-	IGV_LINK	132.852	3.12023	0.568813	5.67165	OK	79.4043	2.00871	0	4.05502	OK	OK	-0.63538
ENSG0000007	-	-	ENSG0000007390	'VDAC1P1	-	X:80929499-80930347	-	-	IGV_LINK	6.78804	0.566334	0	1.33099	OK	7.21857	0.603281	0	1.40489	OK	OK	0.091177
ENSG0000007	-	-	ENSG0000007391	'FRY	-	13:32025313-32299122	-	-	IGV_LINK	634.197	12.2461	0	29.5216	OK	313.492	9.33669	0	21.1127	OK	OK	-0.39133
ENSG0000007	-	-	ENSG0000007392	'PICALM	-	11:85957683-86069882	-	-	IGV_LINK	4499.5	134.037	12.7127	255.36	OK	5378.87	155.651	16.5271	294.774	OK	OK	0.215684
ENSG0000007	-	-	ENSG0000007396	'NSF	-	17:46590668-46757464	-	-	IGV_LINK	522.679	9.85273	0.955189	18.7503	OK	684.733	13.9083	0.611607	27.205	OK	OK	0.49735
ENSG0000007	-	-	ENSG0000007404	'GLI2	-	2:120735622-120992653	-	-	IGV_LINK	123.155	1.29829	0	2.80718	OK	10.3122	0.110539	0	0.626757	OK	OK	-3.55399
ENSG0000007	-	-	ENSG0000007405	'CLASP1	-	2:121337775-121649587	-	-	IGV_LINK	1533.13	15.9203	0	35.8939	OK	2325.41	23.6111	0	53.5767	OK	OK	0.568594
ENSG0000007	-	-	ENSG0000007407	'MRPS34	-	16:1771889-1773155	-	-	IGV_LINK	974.569	75.2511	28.749	121.753	OK	1077.63	81.9691	34.0494	129.889	OK	OK	0.123367
ENSG0000007	-	-	ENSG0000007418	'NOTCH3	-	19:15159037-15200981	-	-	IGV_LINK	2924.68	23.6782	0	49.4112	OK	1732.46	13.2022	0.130524	26.2738	OK	OK	-0.84278
ENSG0000007	-	-	ENSG0000007420	'CLNS1A	-	11:77514935-77637805	-	-	IGV_LINK	991.054	66.802	24.9018	108.702	OK	1101.35	71.9469	27.4846	116.409	OK	OK	0.107042
ENSG0000007	-	-	ENSG0000007421	'PPP2R2C	-	4:6320577-6563600	-	-	IGV_LINK	1.93944	0.151266	0	0.697604	OK	65.9984	1.32817	0	2.79604	OK	OK	3.13428
ENSG0000007	-	-	ENSG0000007421	'TEAD2	-	19:49331616-49388081	-	-	IGV_LINK	138.67	4.36193	0	13.4169	OK	118.591	3.25494	0	9.13311	OK	OK	-0.42233
ENSG0000007	-	-	ENSG0000007426	'EED	-	11:86244543-86278813	-	-	IGV_LINK	329.705	11.8732	3.57735	20.169	OK	318.649	11.5353	2.18527	20.8854	OK	OK	-0.04165
ENSG0000007	-	-	ENSG0000007427	'CDHR2	-	5:176542510-176610133	-	-	IGV_LINK	15.5155	0.785056	0	3.35373	OK	18.562	0.498164	0	2.73922	OK	OK	-0.65618
ENSG0000007	-	-	ENSG0000007431	'SNCB	-	5:176620083-176630556	-	-	IGV_LINK	0	0	0	0	OK	0	0	0	0	OK	NOTEST	0
ENSG0000007	-	-	ENSG0000007431	'TSG101	-	11:18455823-18527232	-	-	IGV_LINK	957.114	54.1001	10.5223	97.678	OK	1096.19	64.0045	18.1028	109.906	OK	OK	0.242542
ENSG0000007	-	-	ENSG0000007435	'NCBP3	-	17:3802164-3846251	-	-	IGV_LINK	1044.39	12.4622	0	25.7515	OK	889.947	12.4636	0	34.7748	OK	OK	0.000163
ENSG0000007	-	-	ENSG0000007437	'ATP2A3	-	17:3923869-3964464	-	-	IGV_LINK	870.809	16.6726	1.84003	31.5052	OK	1160.13	26.0822	2.55062	49.6139	OK	OK	0.645586
ENSG0000007	-	-	ENSG0000007441	'CA12	-	15:63321377-63382161	-	-	IGV_LINK	10409	142.317	30.9818	253.652	OK	31201.8	419.958	0	914.573	OK	OK	1.56114
ENSG0000007	-	-	ENSG0000007441	'MGLL	-	3:127689061-127823250	-	-	IGV_LINK	3376.57	46.7353	16.1733	77.2974	OK	8518.95	119.942	30.2442	209.64	OK	OK	1.35975
ENSG0000007	-	-	ENSG0000007452	'NTN4	-	12:95551581-95791152	-	-	IGV_LINK	2849.04	55.9004	12.4529	99.3478	OK	504.269	9.85186	3.12655	16.5772	OK	OK	-2.50439
ENSG0000007	-	-	ENSG0000007458	'BCS1L	-	2:218637915-218663443	-	-	IGV_LINK	627.409	48.0851	5.8775	90.2927	OK	653.797	46.1969	5.79709	86.5968	OK	OK	-0.05779
ENSG0000007	-	-	ENSG0000007459	'NUAK1	-	12:106063339-106140033	-	-	IGV_LINK	199.762	1.69153	0.347987	3.03507	OK	253.681	2.1499	0.499752	3.80006	OK	OK	0.345944
ENSG0000007	-	-	ENSG0000007460	'DPP8	-	15:65442462-65517704	-	-	IGV_LINK	735.048	8.96694	0	18.5095	OK	748.669	11.6424	0	24.9436	OK	OK	0.376698
ENSG0000007	-	-	ENSG0000007462	'SLC24A1	-	15:65611365-65891991	-	-	IGV_LINK	333.584	5.87432	0	20.7651	OK	375.366	6.15293	0	21.8379	OK	OK	0.066852
ENSG0000007	-	-	ENSG0000007465	'ZNF532	-	18:58862599-58986480	-	-	IGV_LINK	2928.56	46.505	0	94.9337	OK	2038.73	31.4646	0	63.7052	OK	OK	-0.56366
ENSG0000007	-	-	ENSG0000007466	'SCARF1	-	17:1633857-1645747	-	-	IGV_LINK	627.409	12.5957	4.17567	21.0156	OK	600.173	11.6401	3.93539	19.3449	OK	OK	-0.11382
ENSG0000007	-	-	ENSG0000007469	'LMAN1	-	18:59327822-59359962	-	-	IGV_LINK	3054.62	35.2455	6.8012	63.6897	OK	3957.84	43.4599	14.6717	72.2481	OK	OK	0.302247
ENSG0000007	-	-	ENSG0000007469	'HACD3	-	15:65530417-65578352	-	-	IGV_LINK	490.679	9.14336	3.42281	14.8639	OK	722.889	13.7562	4.79154	22.7209	OK	OK	0.58929
ENSG0000007	-	-	ENSG0000007470	'IPCEF1	-	6:154010495-154356792	-	-	IGV_LINK	270.552	4.91383	0	12.1122	OK	137.153	2.20802	0	5.47874	OK	OK	-1.15409
ENSG0000007	-	-	ENSG0000007475	'ZZEF1	-	17:4004444-4143020	-	-	IGV_LINK	1396.4	11.9272	0	24.0397	OK	1266.34	10.1657	0	20.3729	OK	OK	-0.23054
ENSG0000007	-	-	ENSG0000007477	'NOX3	-	6:155395369-155455903	-	-	IGV_LINK	0	0	0	0	OK	0	0	0	0	OK	NOTEST	0
ENSG0000007	-	-	ENSG0000007480	'ENO1	-	1:8861001-8879894	-	-	IGV_LINK	35697.4	1269.02	14.2005	2523.83	OK	46462.9	1647.65	0	3319.69	OK	OK	0.376697
ENSG0000007	-	-	ENSG0000007480	'SLC12A1	-	15:48189036-48304078	-	-	IGV_LINK	9.69721	0.157454	0	0.737598	OK	3.09367	0.020656	0	0.400187	OK	NOTEST	-2.93029
ENSG0000007	-	-	ENSG0000007484	'MYDGF	-	19:4639517-4670370	-	-	IGV_LINK	922.204	61.9767	17.3309	106.622	OK	1703.58	113.708	40.7317	186.684	OK	OK	0.875534
ENSG0000007	-	-	ENSG0000007485	'ANO8	-	19:17292130-17334829	-	-	IGV_LINK	273.461	3.82823	0	8.86813	OK	319.68	4.49864	0	11.6684	OK	OK	0.232811
ENSG0000007	-	-	ENSG0000007493	'TUBE1	-	6:112054071-112087529	-	-	IGV_LINK	444.132	14.9371	4.06153	25.8128	OK	175.308	6.69919	0.39648	13.0019	OK	OK	-1.15685
ENSG0000007	-	-	ENSG0000007496	'ARHGEF10L	-	1:17539834-17697874	-	-	IGV_LINK	2943.1	66.8188	2.09734	131.54	OK	3164.83	67.4798	3.35786	131.602	OK	OK	0.014202
ENSG0000007	-	-	ENSG0000007496	'TXK	-	4:48066392-48134256	-	-	IGV_LINK	107.639	3.31346	0	7.08216	OK	94.8727	3.16893	0	6.95454	OK	OK	-0.06435
ENSG0000007	-	-	ENSG0000007503	'WSCD2	-	12:108129470-108250537	-	-	IGV_LINK	1.93944	0.166947	0	0.977524	OK	1.03122	0.01349	0	0.697264	OK	NOTEST	-3.62944
ENSG0000007	-	-	ENSG0000007504	'KCNQ2	-	20:63400209-63472677	-	-	IGV_LINK	0	0	0	0	OK	0	0	0	0	OK	NOTEST	0
ENSG0000007	-	-	ENSG0000007507	'TACR2	-	10:69403902-69416867	-	-	IGV_LINK	8.72748	0.313779	0	0.737047	OK	9.28102	0.333926	0	0.77843	OK	NOTEST	0.089777
ENSG0000007	-	-	ENSG0000007508	'ACTR6	-	12:100156356-100267079	-	-	IGV_LINK	298.674	12.6598	0	35.1839	OK	277.4	14.3288	0	37.1445	OK	OK	0.178666
ENSG0000007	-	-	ENSG0000007513	'TIPIN	-	15:66336205-66386746	-	-	IGV_LINK	79.3556	4.86041	0	10.3689	OK	73.217	4.18458	0	8.8376	OK	OK	-0.21599
ENSG0000007	-	-	ENSG0000007514	'SRI	-	7:88205117-88306891	-	-	IGV_LINK	1013.36	85.7779	29.7793	141.776	OK	1424.12	127.587	36.0689	219.105	OK	OK	0.572801
ENSG0000007	-	-	ENSG0000007515	'EIF4G3	-	1:20806291-21176888	-	-	IGV_LINK	1683.43	20.2398	5.90751	34.572	OK	1987.17	25.3815	4.66706	46.0959	OK	OK	0.326584
ENSG0000007	-	-	ENSG0000007518	'NUP37	-	12:102063354-102120124	-	-	IGV_LINK	223.036	13.2194	3.30376	23.135	OK	256.775	15.1091	3.77851	26.4397	OK	OK	0.192763
ENSG0000007	-	-	ENSG0000007521	'SEMA3A	-	7:83955776-84492724	-	-	IGV_LINK	158.064	2.55073	0	5.14404	OK	125.809	2.07843	0	4.23287	OK	OK	-0.29541
ENSG0000007	-	-	ENSG0000007521	'GTSE1	-	22:46296740-46330810	-	-	IGV_LINK	98.9115	2.70416	0	5.81348	OK	106.216	2.80304	0	5.88292	OK	OK	0.051811
ENSG0000007	-	-	ENSG0000007522	'SEMA3C	-	7:80742537-80922359	-	-	IGV_LINK	88.2446	2.73012	0	6.81941	OK	432.083	10.6805	0.717069	20.6439	OK	OK	1.96794
ENSG0000007	-	-	ENSG0000007523	'TTC38	-	22:46267960-46294008	-	-	IGV_LINK	876.627	31.2635	5.70199	56.8249	OK	1514.87	51.1237	11.338	90.9094	OK	OK	0.709514
ENSG0000007	-	-	ENSG0000007523	'ACAT1	-	11:108008732-108147776	-	-	IGV_LINK	1319.79	52.6671	12.1544	93.1798	OK	1294.19	47.341	12.8595	81.8225	OK	OK	-0.15381
ENSG0000007	-	-	ENSG0000007524	'GRAMD4	-	22:46576011-46679790	-	-	IGV_LINK	1686.34	30.6999	0	70.2145	OK	1743.8	30.4272	0	68.8526	OK	OK	-0.01287
ENSG0000007	-	-	ENSG0000007527	'CELSR1	-	22:46360833-46537170	-	-	IGV_LINK	1041.48	7.53417	1.35038	13.718	OK	1270.47	8.39776	2.22875	14.5668	OK	OK	0.156556
ENSG0000007	-	-	ENSG0000007529	'WNT8B	-	10:100463040-100483744	-	-	IGV_LINK	5.81832	0.169063	0	0.40152	OK	6.18735	0.179893	0	0.426596	OK	NOTEST	0.089574
ENSG0000007	-	-	ENSG0000007529	'ZNF638	-	2:71276560-71435069	-	-	IGV_LINK	2355.45	57.362	3.31129	111.413	OK	2170.73	58.9127	3.0017	114.824	OK	OK	0.038483
ENSG0000007	-	-	ENSG0000007530	'SLC25A40	-	7:87833567-87909541	-	-	IGV_LINK	292.856	8.94465	0	18.813	OK	296.993	7.11292	0	15.1822	OK	OK	-0.33058
ENSG0000007	-	-	ENSG0000007533	'TIMM21	-	18:74148510-74160530	-	-	IGV_LINK	298.674	23.6854	3.75137	43.6195	OK	395.99	31.7504	6.63436	56.8664	OK	OK	0.422773
ENSG0000007	-	-	ENSG0000007534	'ADD2	-	2:70607617-70768225	-	-	IGV_LINK	326.796	3.40177	0	8.12215	OK	365.054	3.81423	0	9.18848	OK	OK	0.165107
ENSG0000007	-	-	ENSG0000007538	'FGF4	-	11:69771015-69775403	-	-	IGV_LINK	0	0	0	0	OK	0	0	0	0	OK	NOTEST	0
ENSG0000007	-	-	ENSG0000007539	'RASAL2	-	1:178094140-178499634	-	-	IGV_LINK	761.231	6.69195	0	15.0343	OK	382.584	2.33738	0.307564	4.3672	OK	OK	-1.51753
ENSG0000007	-	-	ENSG0000007539	'VPS9D1	-	16:89707133-89816657	-	-	IGV_LINK	339.402	9.07757	0	25.3533	OK	419.709	11.0767	0	27.5577	OK	OK	0.287155
ENSG0000007	-	-	ENSG0000007540	'ZNF37A	-	10:38094333-38378505	-	-	IGV_LINK	742.029	8.53732	0	20.5274	OK	390.914	3.84444	0	8.91992	OK	OK	-1.15101
ENSG0000007	-	-	ENSG0000007541	'MARK3	-	14:103385391-103503831	-	-	IGV_LINK	960.023	26.182	7.5632	44.8007	OK	984.82	24.6506	7.33034	41.9708	OK	OK	-0.08695
ENSG0000007	-	-	ENSG0000007541	'SLC25A3	-	12:98593590-98606379	-	-	IGV_LINK	4197.92	192.682	72.4985	312.865	OK	4781.79	220.485	84.0983	356.872	OK	OK	0.194462
ENSG0000007	-	-	ENSG0000007542	'FNDC3B	-	3:172039627-172401665	-	-	IGV_LINK	1691.19	20.6534	5.41969	35.8871	OK	2693.56	36.4464	0	75.116	OK	OK	0.819396
ENSG0000007	-	-	ENSG0000007542	'FOSL2	-	2:28384408-28417312	-	-	IGV_LINK	2426.24	24.2819	6.26201	42.3017	OK	5533.55	58.0934	8.79162	107.395	OK	OK	1.25849
ENSG0000007	-	-	ENSG0000007542	'CACNG5	-	17:66835116-66885486	-	-	IGV_LINK	0	0	0	0	OK	4.1249	0.308989	0	0.794499	OK	NOTEST	inf
ENSG0000007	-	-	ENSG0000007546	'CACNG4	-	17:66964909-67033398	-	-	IGV_LINK	0.969721	0.017038	0	0.066734	OK	1.03122	0.018125	0	0.067839	OK	NOTEST	0.089236
ENSG0000007	-	-	ENSG0000007553	'FRYL	-	4:48497360-48780322	-	-	IGV_LINK	1148.15	14.2492	0	29.5046	OK	904.384	12.1553	1.40677	22.9039	OK	OK	-0.22929
ENSG0000007	-	-	ENSG0000007556	'TMEM131	-	2:97756332-97995891	-	-	IGV_LINK	1223.79	13.3309	0	27.2758	OK	1363.28	13.9022	0.65091	27.1534	OK	OK	0.060534
ENSG0000007	-	-	ENSG0000007561	'FSCN1	-	7:5592822-5606655	-	-	IGV_LINK	541.104	11.9152	4.5507	19.2796	OK	529.018	11.7087	4.49829	18.919	OK	OK	-0.02522
ENSG0000007	-	-	ENSG0000007562	'ACTB	-	7:5527150-5563784	-	-	IGV_LINK	27317	891.557	111.185	1671.93	OK	24090.4	821.865	66.6071	1577.12	OK	OK	-0.11743

ENSG0000007	-	-	ENSG0000007564	'MOCOS	-	18:36187518-36272157	-	-	IGV_LINK	83.396	1.3829	0	4.56115	OK	826.011	10.3667	0	23.9556	OK	OK	2.90619
ENSG0000007	-	-	ENSG0000007565	'PLD1	-	3:171600404-171810950	-	-	IGV_LINK	629.349	8.64789	0	18.3796	OK	793.012	11.5661	0	23.7384	OK	OK	0.41948
ENSG0000007	-	-	ENSG0000007567	'ATP12A	-	13:24680410-24712493	-	-	IGV_LINK	0	0	0	0	OK	0	0	0	0	OK	NOTEST	0
ENSG0000007	-	-	ENSG0000007570	'WDR62	-	19:36054880-36105106	-	-	IGV_LINK	101.821	1.4169	0	2.98438	OK	71.1545	0.841361	0	1.71849	OK	OK	-0.75194
ENSG0000007	-	-	ENSG0000007571	'DLG1	-	3:197042559-197303747	-	-	IGV_LINK	938.689	19.3319	2.34392	36.32	OK	918.821	19.8094	0.92811	38.6908	OK	OK	0.035201
ENSG0000007	-	-	ENSG0000007578	'RAB7A	-	3:128726121-128814796	-	-	IGV_LINK	3132.2	93.303	28.774	157.832	OK	3260.73	109.979	11.1294	208.828	OK	OK	0.237228
ENSG0000007	-	-	ENSG0000007579	'BCAP29	-	7:107579556-107629550	-	-	IGV_LINK	440.438	15.2242	0.66308	29.7854	OK	545.764	21.2907	1.84368	40.7378	OK	OK	0.483858
ENSG0000007	-	-	ENSG0000007582	'SEC31B	-	10:100486641-100559998	-	-	IGV_LINK	1023.52	16.6572	0	39.411	OK	724.482	12.2923	0	30.3785	OK	OK	-0.43839
ENSG0000007	-	-	ENSG0000007585	'SART3	-	12:108515184-108561400	-	-	IGV_LINK	981.357	22.9049	2.39537	43.4144	OK	1110.63	23.4866	3.70512	43.2682	OK	OK	0.036186
ENSG0000007	-	-	ENSG0000007588	'ARHGAP15	-	2:143091361-143776073	-	-	IGV_LINK	612.863	25.441	4.75909	46.123	OK	369.179	15.6971	1.97129	29.4229	OK	OK	-0.69666
ENSG0000007	-	-	ENSG0000007588	'TUBA3D	-	2:131464899-131492743	-	-	IGV_LINK	10.6669	0.53805	0	3.75509	OK	56.7174	2.56256	0	10.3095	OK	OK	2.25178
ENSG0000007	-	-	ENSG0000007589	'PAX2	-	10:100735602-100829941	-	-	IGV_LINK	2923.71	69.9833	19.6457	120.321	OK	1598.4	37.4879	11.2642	63.7116	OK	OK	-0.90059
ENSG0000007	-	-	ENSG0000007591	'EXOSC7	-	3:44915256-45036071	-	-	IGV_LINK	236.612	13.5764	0	30.9084	OK	248.525	14.2415	0	33.6174	OK	OK	0.068997
ENSG0000007	-	-	ENSG0000007594	'KIFAP3	-	1:169921325-170085208	-	-	IGV_LINK	689.471	14.0293	5.73087	22.3277	OK	1072.47	21.7686	9.46083	34.0765	OK	OK	0.633809
ENSG0000007	-	-	ENSG0000007597	'MKRN2	-	3:12484431-12664226	-	-	IGV_LINK	303.523	7.46351	0	20.2125	OK	259.869	6.09726	0	16.83	OK	OK	-0.29169
ENSG0000007	-	-	ENSG0000007600	'MCM6	-	2:135839625-135876426	-	-	IGV_LINK	518.8	8.36063	2.89404	13.8272	OK	523.862	11.0174	1.43972	20.595	OK	OK	0.398095
ENSG0000007	-	-	ENSG0000007604	'REXO2	-	11:114391442-114450279	-	-	IGV_LINK	743.891	49.3842	8.35891	90.4096	OK	1009.76	63.3592	16.5159	110.203	OK	OK	0.359504
ENSG0000007	-	-	ENSG0000007605	'RBM7	-	11:114391442-114450279	-	-	IGV_LINK	528.624	20.4682	0	46.1342	OK	431.617	17.8438	0	44.3594	OK	OK	-0.19796
ENSG0000007	-	-	ENSG0000007606	'RBMS2	-	12:56521928-56636816	-	-	IGV_LINK	1436.16	12.3962	0	43.4488	OK	1330.28	10.657	0	28.0042	OK	OK	-0.2181
ENSG0000007	-	-	ENSG0000007610	'BAZ2A	-	12:56521928-56636816	-	-	IGV_LINK	4517.93	62.8765	0	153.763	OK	3969.18	55.2527	0	131.958	OK	OK	-0.18648
ENSG0000007	-	-	ENSG0000007620	'PTPN23	-	3:47381010-47413441	-	-	IGV_LINK	685.592	8.68199	1.74121	15.6228	OK	817.761	9.8934	2.54404	17.2428	OK	OK	0.18844
ENSG0000007	-	-	ENSG0000007624	'MLH1	-	3:36993331-37050918	-	-	IGV_LINK	471.284	15.238	2.78009	27.6959	OK	472.301	13.5556	2.81985	24.2913	OK	OK	-0.16879
ENSG0000007	-	-	ENSG0000007624	'UNG	-	12:109097573-109110992	-	-	IGV_LINK	497.467	14.3767	5.22852	23.525	OK	626.985	18.2204	7.11716	29.3236	OK	OK	0.341817
ENSG0000007	-	-	ENSG0000007625	'FMO4	-	1:171314207-171342084	-	-	IGV_LINK	218.187	7.68928	0.407006	14.9715	OK	375.366	11.3418	2.59619	20.0873	OK	OK	0.560724
ENSG0000007	-	-	ENSG0000007632	'KLHL20	-	1:173714940-173786702	-	-	IGV_LINK	302.553	7.67212	0	15.7454	OK	504.269	12.1377	0.332745	23.9427	OK	OK	0.661802
ENSG0000007	-	-	ENSG0000007634	'RGS11	-	16:234545-283010	-	-	IGV_LINK	20.3641	0.500423	0	3.36567	OK	3.09367	0.084538	0	1.20967	OK	NOTEST	-2.56547
ENSG0000007	-	-	ENSG0000007635	'SLC46A1	-	17:28357580-28407197	-	-	IGV_LINK	1462.34	22.6853	0	55.2137	OK	1807.74	27.6693	0	65.8201	OK	OK	0.286529
ENSG0000007	-	-	ENSG0000007635	'PLXNA2	-	1:208022241-208244320	-	-	IGV_LINK	649.713	5.9329	0	16.9923	OK	670.296	5.38546	0	14.5277	OK	OK	-0.13967
ENSG0000007	-	-	ENSG0000007638	'SPAG5	-	17:28455751-28645454	-	-	IGV_LINK	338.432	10.22	0	52.3608	OK	496.019	12.7451	0	67.122	OK	OK	0.318543
ENSG0000007	-	-	ENSG0000007651	'ANKRD13A	-	12:109999185-110073686	-	-	IGV_LINK	5470.19	115.078	15.0578	215.097	OK	3760.88	75.7124	13.2807	138.144	OK	OK	-0.60401
ENSG0000007	-	-	ENSG0000007655	'TPD52	-	8:79918716-80231232	-	-	IGV_LINK	775.334	45.42	0	92.7824	OK	720.203	52.2353	0	108.1	OK	OK	0.201697
ENSG0000007	-	-	ENSG0000007655	'ACACB	-	12:109111217-109268226	-	-	IGV_LINK	1327.55	13.6077	1.08566	26.1298	OK	1431.34	13.581	1.21915	25.9428	OK	OK	-0.00284
ENSG0000007	-	-	ENSG0000007660	'TRAF4	-	17:28743983-28750958	-	-	IGV_LINK	312.25	14.6115	0.426728	28.7963	OK	888.916	40.0131	7.46397	72.5623	OK	OK	1.45337
ENSG0000007	-	-	ENSG0000007664	'PAG1	-	8:80967809-81112068	-	-	IGV_LINK	1414.82	10.5321	0	21.9483	OK	894.072	6.93535	0	14.9527	OK	OK	-0.60275
ENSG0000007	-	-	ENSG0000007665	'GPATCH1	-	19:33080879-33130542	-	-	IGV_LINK	338.432	7.33146	0.277799	14.3851	OK	265.025	6.10228	0	12.2593	OK	OK	-0.26475
ENSG0000007	-	-	ENSG0000007666	'ICAM3	-	19:10304802-10339823	-	-	IGV_LINK	555.65	18.3859	0	37.02	OK	719.795	21.7298	1.86915	41.5904	OK	OK	0.241076
ENSG0000007	-	-	ENSG0000007668	'NT5C2	-	10:102918292-103193306	-	-	IGV_LINK	1001.72	27.9497	0.554856	55.3446	OK	976.57	26.5885	1.77277	51.4042	OK	OK	-0.07203
ENSG0000007	-	-	ENSG0000007670	'MCAM	-	11:119206275-119321521	-	-	IGV_LINK	3971.98	78.9263	0	174.562	OK	4008.37	77.3894	15.0292	139.75	OK	OK	-0.02837
ENSG0000007	-	-	ENSG0000007671	'GPC4	-	X:133300102-133415490	-	-	IGV_LINK	738.927	8.69822	3.56093	13.8355	OK	1064.22	12.5304	5.45553	19.6053	OK	OK	0.526645
ENSG0000007	-	-	ENSG0000007677	'MBNL3	-	X:132203023-132489968	-	-	IGV_LINK	812.626	7.72918	0	18.4694	OK	834.261	5.7677	0	12.4227	OK	OK	-0.42232
ENSG0000007	-	-	ENSG0000007682	'CAMSAP3	-	19:7595901-7618304	-	-	IGV_LINK	323.887	5.8183	0	12.2906	OK	601.204	10.6072	0	21.7802	OK	OK	0.866371
ENSG0000007	-	-	ENSG0000007686	'RAP1GAP	-	1:21596214-21669363	-	-	IGV_LINK	691.411	12.4272	4.37701	20.4774	OK	741.451	14.9472	1.06326	28.8312	OK	OK	0.266375
ENSG0000007	-	-	ENSG0000007692	'XAB2	-	19:7619524-7629565	-	-	IGV_LINK	1326.58	30.9549	12.1821	49.7277	OK	1229.22	28.3334	11.0238	45.643	OK	OK	-0.12766
ENSG0000007	-	-	ENSG0000007692	'ARHGEF1	-	19:41883160-41930150	-	-	IGV_LINK	3602.51	96.2787	11.495	181.062	OK	3179.27	82.5694	11.871	153.268	OK	OK	-0.22161
ENSG0000007	-	-	ENSG0000007694	'STXBP2	-	19:7629736-7647873	-	-	IGV_LINK	1640.77	80.0492	13.7091	146.389	OK	2157.32	109.044	19.77	198.318	OK	OK	0.445951
ENSG0000007	-	-	ENSG0000007698	'MAP2K7	-	19:7903842-7914478	-	-	IGV_LINK	783.534	17.9386	4.05826	31.8189	OK	878.604	20.9378	5.91736	35.9583	OK	OK	0.223045
ENSG0000007	-	-	ENSG0000007700	'NMRK2	-	19:3933102-3942416	-	-	IGV_LINK	0	0	0	0	OK	1.03122	0.06098	0	0.437519	OK	NOTEST	inf
ENSG0000007	-	-	ENSG0000007704	'DGKD	-	2:233354506-233472104	-	-	IGV_LINK	835.899	13.1372	0	35.6374	OK	1348.84	19.9602	0	50.0236	OK	OK	0.60347
ENSG0000007	-	-	ENSG0000007706	'CTTNBP2	-	7:117465783-117874139	-	-	IGV_LINK	615.773	10.2931	0	21.1155	OK	236.151	3.48031	0	8.44151	OK	OK	-1.56439
ENSG0000007	-	-	ENSG0000007708	'ACTL6B	-	7:100643096-100656461	-	-	IGV_LINK	0	0	0	0	OK	0	0	0	0	OK	NOTEST	0
ENSG0000007	-	-	ENSG0000007709	'RARB	-	3:25174331-25664939	-	-	IGV_LINK	87.2748	2.15926	0	7.3798	OK	108.279	3.30186	0	9.86285	OK	OK	0.612742
ENSG0000007	-	-	ENSG0000007709	'TOP2B	-	3:25174331-25664939	-	-	IGV_LINK	1223.79	21.3921	6.29443	36.4897	OK	939.446	15.2848	4.9664	25.6032	OK	OK	-0.48498
ENSG0000007	-	-	ENSG0000007714	'TM9SF3	-	10:96518108-96587452	-	-	IGV_LINK	1706.71	17.5478	3.96269	31.133	OK	1441.65	15.0991	4.2787	25.9195	OK	OK	-0.21683
ENSG0000007	-	-	ENSG0000007715	'NFKB2	-	10:102394109-102402529	-	-	IGV_LINK	2298.24	56.4763	12.4155	100.537	OK	1525.18	37.901	7.89704	67.9049	OK	OK	-0.57541
ENSG0000007	-	-	ENSG0000007715	'UBE2T	-	1:202331656-202341980	-	-	IGV_LINK	52.3649	3.94493	0.005057	7.88481	OK	70.1233	5.29801	0.071055	10.525	OK	OK	0.425449
ENSG0000007	-	-	ENSG0000007715	'PPP1R12B	-	1:202348698-202710417	-	-	IGV_LINK	790.322	5.23083	0	15.7414	OK	667.203	3.40448	0	9.52564	OK	OK	-0.61961
ENSG0000007	-	-	ENSG0000007723	'DNAJC10	-	2:182716040-182794464	-	-	IGV_LINK	1551.55	30.996	1.80217	60.1898	OK	1551.99	25.959	3.17416	48.7438	OK	OK	-0.25585
ENSG0000007	-	-	ENSG0000007723	'GTF3C1	-	16:27459554-27549913	-	-	IGV_LINK	1319.79	24.6879	0	53.1054	OK	1688.12	26.0777	0	56.8942	OK	OK	0.079012
ENSG0000007	-	-	ENSG0000007723	'IL4R	-	16:27313386-27364778	-	-	IGV_LINK	1964.65	42.2627	6.89922	77.6262	OK	1935.61	40.7434	7.55586	73.931	OK	OK	-0.05282
ENSG0000007	-	-	ENSG0000007725	'USP33	-	1:77695986-77759852	-	-	IGV_LINK	988.145	18.9166	1.51309	36.3201	OK	1007.51	19.5943	2.27462	36.914	OK	OK	0.050783
ENSG0000007	-	-	ENSG0000007726	'PAK3	-	X:110944284-111227361	-	-	IGV_LINK	102.79	1.22769	0	3.31139	OK	24.7494	0.403883	0	1.24822	OK	OK	-1.60393
ENSG0000007	-	-	ENSG0000007727	'CAPN6	-	X:111245102-111270523	-	-	IGV_LINK	0	0	0	0	OK	28.8743	0.479395	0	0.988428	OK	NOTEST	inf
ENSG0000007	-	-	ENSG0000007727	'DCX	-	X:111293778-111412429	-	-	IGV_LINK	0	0	0	0	OK	9.28102	0.058176	0	0.377359	OK	NOTEST	inf
ENSG0000007	-	-	ENSG0000007731	'SNRPA	-	19:40691528-40765389	-	-	IGV_LINK	926.083	39.5378	0	82.4947	OK	814.668	37.6951	0	89.743	OK	OK	-0.06886
ENSG0000007	-	-	ENSG0000007732	'SPAG6	-	10:22345444-22454224	-	-	IGV_LINK	2.90916	0.078156	0	0.503507	OK	2.06245	0.151136	0	0.730068	OK	NOTEST	0.951425
ENSG0000007	-	-	ENSG0000007734	'EXOSC5	-	19:41262495-41425005	-	-	IGV_LINK	543.043	34.1484	0	135.816	OK	529.018	32.5472	0	121.276	OK	OK	-0.06928
ENSG0000007	-	-	ENSG0000007738	'DYNC1I2	-	2:171687408-171748420	-	-	IGV_LINK	1664.04	49.1201	15.4659	82.7743	OK	1548.9	42.9829	14.406	71.5598	OK	OK	-0.19255
ENSG0000007	-	-	ENSG0000007742	'APBB1IP	-	10:26438202-26567803	-	-	IGV_LINK	1345.97	30.4745	12.378	48.571	OK	1424.12	32.3879	13.3295	51.4462	OK	OK	0.08785
ENSG0000007	-	-	ENSG0000007745	'LRCH4	-	7:100572227-100608175	-	-	IGV_LINK	1170.91	44.5143	2.83526	86.1933	OK	1051.39	40.0611	0.661781	79.4604	OK	OK	-0.15207
ENSG0000007	-	-	ENSG0000007745	'FAM76B	-	11:95768941-95832693	-	-	IGV_LINK	352.009	18.3858	0	44.3793	OK	238.213	8.83804	0	20.4856	OK	OK	-1.05679
ENSG0000007	-	-	ENSG0000007746	'SIRT6	-	19:4174108-4182604	-	-	IGV_LINK	339.402	17.8875	1.80847	33.9665	OK	404.24	21.0912	4.08634	38.0961	OK	OK	0.237693
ENSG0000007	-	-	ENSG0000007749	'TYR	-	11:89177451-89295759	-	-	IGV_LINK	0.969721	0.042913	0	0.295	OK	1.03122	0.025144	0	0.263581	OK	NOTEST	-0.77122
ENSG0000007	-	-	ENSG0000007751	'POLD3	-	11:74491711-74669117	-	-	IGV_LINK	490.679	11.0303	1.15308	20.9075	OK	530.05	12.6505	0.939384	24.3615	OK	OK	0.19772
ENSG0000007	-	-	ENSG0000007752	'ACTN2	-	1:236686453-236764631	-	-	IGV_LINK	137.7	5.75709	0	13.8064	OK	159.84	3.62035	0	8.7478	OK	OK	-0.66921
ENSG0000007	-	-	ENSG0000007754	'CAPZB	-	1:19338775-19485539	-	-	IGV_LINK	2832.55	157.561	62.2347	252.888	OK	3132.86	177.991	65.8889	290.093	OK	OK	0.175891
ENSG0000007	-	-	ENSG0000007758	'GPR137B	-	1:236142504-236281985	-	-	IGV_LINK	636.137	22.0101	5.87612	38.1441	OK	1092.07	38.4805	10.9787	65.9823	OK	OK	0.80596
ENSG0000007	-	-	ENSG0000007761	'NAALAD2	-	11:90131514-90192894	-	-	IGV_LINK	28.1219	0.48727	0	1.15116	OK	7.21857	0.142173	0	0.536162	OK	NOTEST	-1.77707
ENSG0000007	-	-	ENSG0000007768	'JADE1	-	4:128809622-129116640	-	-	IGV_LINK	705.957	8.90288	1.31667	16.4891	OK	645.547	8.75319	0.358318	17.1481	OK	OK	-0.02446
ENSG0000007	-	-	ENSG0000007771	'SLC25A43	-	X:119399059-119454478	-	-	IGV_LINK	565.347	13.7929	4.46667	23.1192	OK	728.045	17.0359	5.28444	28.7873	OK	OK	0.304647
ENSG0000007	-	-	ENSG0000007772	'UBE2A	-	X:119574466-119605895	-	-	IGV_LINK	663.289	27.0883	6.41723	47.7593	OK	742.482	33.6547	9.60806	57.7014	OK	OK	0.31314
ENSG0000007	-	-	ENSG0000007778	'FGFR1	-	8:38411137-38468834	-	-	IGV_LINK	1073.48	24.3887	1.5617	47.2156	OK	1553.02	34.8311	3.62806	66.0342	OK	OK	0.514167
ENSG0000007	-	-	ENSG0000007780	'FKBP6	-	7:73328163-73358637	-	-	IGV_LINK	2.90916	0.093145	0	0.442467	OK	0	0	0	0	OK	NOTEST	'-inf
ENSG0000007	-	-	ENSG0000007793	'SMC1B	-	22:45344062-45413619	-	-	IGV_LINK	12.6064	0.176439	0	0.407521	OK	27.8431	0.40363	0	0.834089	OK	NOTEST	1.19387
ENSG0000007	-	-	ENSG0000007794	'FBLN1	-	22:45502237-45601135	-	-	IGV_LINK	103.76	2.83352	0	6.19364	OK	275.337	7.0375	1.33849	12.7365	OK	OK	1.31247
ENSG0000007	-	-	ENSG0000007794	'ITGA8	-	10:15513948-15720125	-	-	IGV_LINK	514.922	4.71244	1.09718	8.32771	OK	163.965	1.5665	0	3.22702	OK	OK	-1.58893
ENSG0000007	-	-	ENSG0000007798	'CST7	-	20:24949229-24959928	-	-	IGV_LINK	397.585	29.5801	10.7067	48.4534	OK	257.806	19.2098	5.91394	32.5056	OK	OK	-0.62279
ENSG0000007	-	-	ENSG0000007801	'MAP2	-	2:209424057-209734118	-	-	IGV_LINK	208.49	3.21762	0	6.52448	OK	224.807	3.61575	0	7.4809	OK	OK	0.168304
ENSG0000007	-	-	ENSG0000007804	'PIAS2	-	18:46803223-47102243	-	-	IGV_LINK	509.103	6.88602	0	15.0057	OK	473.332	8.38169	0	18.2254	OK	OK	0.283571
ENSG0000007	-	-	ENSG0000007805	'AMPH	-	7:38383703-38631567	-	-	IGV_LINK	12.6064	0.258154	0	0.809838	OK	1.03122	0.019557	0	0.482523	OK	NOTEST	-3.72251
ENSG0000007	-	-	ENSG0000007806	'ARAF	-	X:47561099-47630305	-	-	IGV_LINK	1072.51	33.0994	0	86.2226	OK	1209.63	37.0069	0	96.7186	OK	OK	0.160991
ENSG0000007	-	-	ENSG0000007807	'MCCC1	-	3:183015217-183116075	-	-	IGV_LINK	793.231	24.7279	6.48104	42.9748	OK	694.014	21.9881	5.54797	38.4281	OK	OK	-0.16942
ENSG0000007	-	-	ENSG0000007808	'LAMP3	-	3:183122212-183163839	-	-	IGV_LINK	224.975	5.12539	0.633353	9.61742	OK	228.932	4.814	1.05983	8.56817	OK	OK	-0.09043
ENSG0000007	-	-	ENSG0000007809	'FAP	-	2:162159761-162245151	-	-	IGV_LINK	10.6669	0.679007	0	1.91352	OK	62.9047	1.81235	0	4.62062	OK	OK	1.41636
ENSG0000007	-	-	ENSG0000007811	'NEBL	-	10:20779972-21175048	-	-	IGV_LINK	860.142	10.8335	0.358077	21.3088	OK	214.495	3.01849	0	7.24403	OK	OK	-1.84359
ENSG0000007	-	-	ENSG0000007812	'ACER3	-	11:76860866-77026797	-	-	IGV_LINK	420.859	5.77923	0	13.1154	OK	433.114	6.73011	0	14.7863	OK	OK	0.219752
ENSG0000007	-	-	ENSG0000007814	'UBE2K	-	4:39698043-39782792	-	-	IGV_LINK	767.049	23.2438	0.479445	46.0081	OK	859.01	30.1503	3.39902	56.9015	OK	OK	0.375325
ENSG0000007	-	-	ENSG0000007814	'PIK3C3	-	18:41955205-42087830	-	-	IGV_LINK	901.84	22.6219	0	46.16	OK	766.2	20.4549	0.645019	40.2649	OK	OK	-0.14527
ENSG0000007	-	-	ENSG0000007817	'N4BP2	-	4:40042916-40158252	-	-	IGV_LINK	173.58	1.07204	0.220992	1.92308	OK	157.777	1.03173	0.188211	1.87525	OK	OK	-0.05529
ENSG0000007	-	-	ENSG0000007823	'TIGAR	-	12:4307762-4354593	-	-	IGV_LINK	146.428	5.44748	0	12.229	OK	154.684	5.41879	0	11.9989	OK	OK	-0.00762
ENSG0000007	-	-	ENSG0000007824	'TULP3	-	12:1946053-2941140	-	-	IGV_LINK	998.812	21.3442	0	51.7467	OK	982.757	20.4427	0	50.2213	OK	OK	-0.06226
ENSG0000007	-	-	ENSG0000007826	'SYNJ2	-	6:157981886-158099176	-	-	IGV_LINK	217.217	2.0698	0	4.25249	OK	191.808	1.61266	0.055068	3.17025	OK	OK	-0.36005
ENSG0000007	-	-	ENSG0000007829	'ADCY2	-	5:7396207-7830081	-	-	IGV_LINK	20.3641	1.06709	0	3.11306	OK	3.09367	0.359349	0	1.18284	OK	OK	-1.57022
ENSG0000007	-	-	ENSG0000007830	'PPP2R5C	-	14:101761797-101927989	-	-	IGV_LINK	1075.42	35.5291	5.75976	65.2984	OK	964.195	33.2872	8.26363	58.3108	OK	OK	-0.09403
ENSG0000007	-	-	ENSG0000007831	'PMS2P1	-	7:100307701-100400099	-	-	IGV_LINK	128.003	6.83453	0	30.235	OK	163.965	8.7642	0	35.7962	OK	OK	0.35878
ENSG0000007	-	-	ENSG0000007832	'RBFOX1	-	16:5239801-7713338	-	-	IGV_LINK	5.81832	0.105917	0	0.864354	OK	14.4371	0.491476	0	1.51803	OK	NOTEST	2.21419
ENSG0000007	-	-	ENSG0000007836	'GNB1	-	1:1785284-1891117	-	-	IGV_LINK	5574.92	124.909	16.0294	233.789	OK	5914.08	130.438	23.5967	237.279	OK	OK	0.062484
ENSG0000007	-	-	ENSG0000007839	'HOXA9	-	7:27162434-27180261	-	-	IGV_LINK	119.276	6.99237	0	16.2986	OK	149.528	8.94457	0	19.5065	OK	OK	0.35523
ENSG0000007	-	-	ENSG0000007840	'EDN1	-	6:12290362-12297194	-	-	IGV_LINK	1489.49	45.118	19.5662	70.6698	OK	420.74	12.7525	4.6917	20.8132	OK	OK	-1.82293
ENSG0000007	-	-	ENSG0000007840	'MLLT10	-	10:21513477-21743630	-	-	IGV_LINK	795.171	16.8342	0	67.1253	OK	460.958	7.71622	0.379635	15.0528	OK	OK	-1.12543
ENSG0000007	-	-	ENSG0000007848	'ZCWPW1	-	7:100400825-100434126	-	-	IGV_LINK	181.338	6.121	0	15.3058	OK	129.934	5.13795	0	15.597	OK	OK	-0.25257
ENSG0000007	-	-	ENSG0000007854	'ADCYAP1R1	-	7:31052460-31111479	-	-	IGV_LINK	0	0	0	0	OK	1.03122	0.041116	0	0.424507	OK	NOTEST	inf
ENSG0000007	-	-	ENSG0000007857	'FGF20	-	8:16992168-17002181	-	-	IGV_LINK	30.0613	2.41517	0	5.94251	OK	5.15612	0.198357	0	0.715781	OK	OK	-3.60595
ENSG0000007	-	-	ENSG0000007858	'P2RY10	-	X:78945331-78961954	-	-	IGV_LINK	112.488	4.73186	0.414804	9.04891	OK	59.811	2.62374	0	5.46724	OK	OK	-0.85078
ENSG0000007	-	-	ENSG0000007859	'ITM2A	-	X:79360383-79367667	-	-	IGV_LINK	689.471	43.9606	15.5249	72.3962	OK	118.591	7.97804	0	16.8217	OK	OK	-2.4621
ENSG0000007	-	-	ENSG0000007861	'NRDC	-	1:51561865-51878937	-	-	IGV_LINK	2488.3	53.097	0	117.791	OK	2724.5	53.0254	0	112.612	OK	OK	-0.00195
ENSG0000007	-	-	ENSG0000007866	'VDAC3	-	8:42391623-42405897	-	-	IGV_LINK	1406.09	75.0458	30.3952	119.696	OK	1437.53	77.5806	30.8475	124.314	OK	OK	0.047924
ENSG0000007	-	-	ENSG0000007867	'PCM1	-	8:17922839-18029944	-	-	IGV_LINK	2466.97	45.6147	0.829315	90.4001	OK	1863.42	32.5827	4.78614	60.3793	OK	OK	-0.48539
ENSG0000007	-	-	ENSG0000007868	'TNRC6C	-	17:77959239-78142968	-	-	IGV_LINK	320.008	3.76234	0	17.8132	OK	237.182	2.86752	0	15.9511	OK	OK	-0.39182
ENSG0000007	-	-	ENSG0000007869	'CBFA2T2	-	20:33490074-33650036	-	-	IGV_LINK	894.082	8.07488	1.57806	14.5717	OK	630.078	6.52938	1.08535	11.9734	OK	OK	-0.3065
ENSG0000007	-	-	ENSG0000007872	'BRINP1	-	9:119153457-119369467	-	-	IGV_LINK	1.93944	0.036078	0	0.255215	OK	0	0	0	0	OK	NOTEST	'-inf
ENSG0000007	-	-	ENSG0000007874	'ITCH	-	20:34363234-34511393	-	-	IGV_LINK	1057.97	13.3099	3.00138	23.6185	OK	943.571	11.8717	1.87712	21.8662	OK	OK	-0.16498
ENSG0000007	-	-	ENSG0000007879	'PKD2L2	-	5:137814332-138051961	-	-	IGV_LINK	7.75776	0.218757	0	1.65716	OK	6.18735	0.174868	0	1.34768	OK	NOTEST	-0.32306
ENSG0000007	-	-	ENSG0000007880	'TP53INP2	-	20:34560541-34825649	-	-	IGV_LINK	1038.57	15.1346	2.68291	27.5863	OK	1622.12	23.6881	6.49938	40.8769	OK	OK	0.646316
ENSG0000007	-	-	ENSG0000007880	'SDF4	-	1:1216907-1232031	-	-	IGV_LINK	2818.98	85.3616	35.9147	134.808	OK	3714.47	116.093	46.5605	185.626	OK	OK	0.443622
ENSG0000007	-	-	ENSG0000007881	'MYH7B	-	20:34975402-35092871	-	-	IGV_LINK	71.7593	0.886415	0	3.97159	OK	72.1857	1.55623	0	9.39485	OK	OK	0.812002
ENSG0000007	-	-	ENSG0000007889	'BPIFB2	-	20:33007599-33023709	-	-	IGV_LINK	0	0	0	0	OK	0	0	0	0	OK	NOTEST	0
ENSG0000007	-	-	ENSG0000007890	'TP73	-	1:3630766-3771645	-	-	IGV_LINK	21.3339	0.330578	0	2.17494	OK	34.0304	0.458988	0	2.2989	OK	NOTEST	0.473465
ENSG0000007	-	-	ENSG0000007890	'TOLLIP	-	11:1274370-1309654	-	-	IGV_LINK	1212.15	25.0969	6.4801	43.7136	OK	1343.69	28.1673	8.69608	47.6385	OK	OK	0.166514
ENSG0000007	-	-	ENSG0000007896	'UBE2D4	-	7:43926437-44019175	-	-	IGV_LINK	185.217	4.57086	0	12.2126	OK	187.683	5.4963	0	14.5109	OK	OK	0.265997
ENSG0000007	-	-	ENSG0000007910	'CLUL1	-	18:596987-712676	-	-	IGV_LINK	10.6669	0.652482	0	9.49586	OK	6.18735	0.278702	0	5.24558	OK	OK	-1.22721
ENSG0000007	-	-	ENSG0000007910	'RUNX1T1	-	8:91954966-92103286	-	-	IGV_LINK	68.8502	1.6706	0	4.99766	OK	24.7494	0.291528	0	1.61622	OK	OK	-2.51866
ENSG0000007	-	-	ENSG0000007911	'CDH17	-	8:94127170-94217303	-	-	IGV_LINK	1.93944	0.031047	0	0.313412	OK	6.18735	0.148326	0	0.490007	OK	NOTEST	2.25626

ENSG0000007	-	-	ENSG0000007913	'THOC1	-	18:158382-268050	-	-	IGV_LINK	672.986	28.3172	0	57.7409	OK	551.705	22.7356	0	49.1434	OK	OK	-0.31673
ENSG0000007	-	-	ENSG0000007915	'FKBP7	-	2:178463663-178478600	-	-	IGV_LINK	128.003	7.64237	0	15.5711	OK	90.7478	4.57377	0	10.0289	OK	OK	-0.74064
ENSG0000007	-	-	ENSG0000007915	'OSBPL6	-	2:178194480-178402891	-	-	IGV_LINK	23.2733	0.264869	0	0.750385	OK	12.3747	0.157496	0	0.636036	OK	NOTEST	-0.74997
ENSG0000007	-	-	ENSG0000007921	'SLC1A3	-	5:36606354-36725195	-	-	IGV_LINK	641.955	14.2863	0	29.079	OK	468.176	11.0694	0	22.6042	OK	OK	-0.36806
ENSG0000007	-	-	ENSG0000007924	'XRCC5	-	2:216107463-216206303	-	-	IGV_LINK	3575.36	65.9136	21.8182	110.009	OK	3573.19	66.0663	21.7901	110.343	OK	OK	0.003339
ENSG0000007	-	-	ENSG0000007925	'LXN	-	3:158644277-158692575	-	-	IGV_LINK	378.191	22.6425	2.24886	43.0361	OK	490.863	29.356	4.84661	53.8653	OK	OK	0.374622
ENSG0000007	-	-	ENSG0000007926	'SP140	-	2:230121369-230313215	-	-	IGV_LINK	314.189	9.00661	0	23.0568	OK	192.839	6.14162	0	18.8181	OK	OK	-0.55237
ENSG0000007	-	-	ENSG0000007927	'MKNK1	-	1:46538695-46616891	-	-	IGV_LINK	867.9	28.2399	0	57.9332	OK	1173.53	43.602	0	87.7128	OK	OK	0.62666
ENSG0000007	-	-	ENSG0000007930	'TNS1	-	2:217799788-218002995	-	-	IGV_LINK	16590	122.437	0	287.097	OK	16205.7	127.148	0	319.557	OK	OK	0.054464
ENSG0000007	-	-	ENSG0000007931	'REXO1	-	19:1815245-1848463	-	-	IGV_LINK	644.864	16.8734	0	34.9951	OK	806.418	22.3932	0	49.6408	OK	OK	0.40831
ENSG0000007	-	-	ENSG0000007933	'SAR1A	-	10:70147288-70170523	-	-	IGV_LINK	1259.67	27.8144	7.80565	47.8231	OK	972.445	20.7095	5.69677	35.7223	OK	OK	-0.42554
ENSG0000007	-	-	ENSG0000007933	'CDC14A	-	1:100344476-100520277	-	-	IGV_LINK	127.033	1.89086	0.18375	3.59797	OK	130.966	2.0742	0.300658	3.84775	OK	OK	0.133517
ENSG0000007	-	-	ENSG0000007933	'RAPGEF3	-	12:47706084-47837898	-	-	IGV_LINK	400.495	8.78845	0	27.5919	OK	385.678	8.38771	0	25.1173	OK	OK	-0.06733
ENSG0000007	-	-	ENSG0000007938	'CEACAM1	-	19:42397127-42652355	-	-	IGV_LINK	215.278	4.86545	0	11.4572	OK	301.118	6.6533	0	16.2573	OK	OK	0.451497
ENSG0000007	-	-	ENSG0000007938	'SENP1	-	12:48042897-48146404	-	-	IGV_LINK	338.432	4.90675	0	11.7682	OK	311.43	4.36149	0	11.2554	OK	OK	-0.16995
ENSG0000007	-	-	ENSG0000007939	'DUSP13	-	10:75094431-75182123	-	-	IGV_LINK	0	0	0	0	OK	0	0	0	0	OK	NOTEST	0
ENSG0000007	-	-	ENSG0000007943	'CIC	-	19:42268536-42295797	-	-	IGV_LINK	1714.47	21.7696	0.264302	43.2749	OK	1892.3	24.5525	0	49.7772	OK	OK	0.173552
ENSG0000007	-	-	ENSG0000007943	'LIPE	-	19:42397127-42652355	-	-	IGV_LINK	29.0916	1.34729	0	4.88918	OK	51.5612	2.21565	0	7.19365	OK	OK	0.717674
ENSG0000007	-	-	ENSG0000007945	'FDFT1	-	8:11795572-11839309	-	-	IGV_LINK	1316.88	52.1143	18.5362	85.6924	OK	1435.47	56.9416	21.9192	91.964	OK	OK	0.127804
ENSG0000007	-	-	ENSG0000007946	'PAFAH1B3	-	19:42297032-42310821	-	-	IGV_LINK	132.852	10.9288	0.624367	21.2332	OK	118.591	12.656	0.856156	24.4557	OK	OK	0.211686
ENSG0000007	-	-	ENSG0000007948	'OPHN1	-	X:68042343-68433913	-	-	IGV_LINK	275.401	2.01393	0.310607	3.71725	OK	226.869	1.65928	0.104988	3.21358	OK	OK	-0.27945
ENSG0000007	-	-	ENSG0000007955	'AFM	-	4:73481682-73504001	-	-	IGV_LINK	6.78804	0.206826	0	0.749467	OK	4.1249	0.1233	0	0.455014	OK	NOTEST	-0.74625
ENSG0000007	-	-	ENSG0000007961	'KIF22	-	16:29790718-29805385	-	-	IGV_LINK	598.318	18.2597	6.38862	30.1307	OK	511.488	15.916	5.00321	26.8289	OK	OK	-0.19818
ENSG0000007	-	-	ENSG0000007968	'SCGN	-	6:25652200-25701783	-	-	IGV_LINK	326.796	14.6525	4.93263	24.3723	OK	10.3122	0.456405	0	1.09768	OK	OK	-5.00469
ENSG0000007	-	-	ENSG0000007969	'CARMIL1	-	6:25279077-25620530	-	-	IGV_LINK	247.279	2.7542	0.653881	4.85452	OK	199.026	2.52361	0.265299	4.78191	OK	OK	-0.12615
ENSG0000007	-	-	ENSG0000007973	'PGM1	-	1:63440769-63660245	-	-	IGV_LINK	1089	27.4837	7.16251	47.8049	OK	3285.48	81.0447	29.4455	132.644	OK	OK	1.56014
ENSG0000007	-	-	ENSG0000007978	'DDX1	-	2:15591177-15631111	-	-	IGV_LINK	1465.25	38.4597	12.7062	64.2132	OK	1459.18	37.6153	13.8083	61.4222	OK	OK	-0.03203
ENSG0000007	-	-	ENSG0000007980	'DNM2	-	19:10718078-10836318	-	-	IGV_LINK	6466.1	137.683	0	278.794	OK	5594.4	125.933	0	254.348	OK	OK	-0.12869
ENSG0000007	-	-	ENSG0000007981	'EPB41L2	-	6:130839346-131063322	-	-	IGV_LINK	1830.83	34.6718	2.97284	66.3707	OK	1258.09	20.2572	6.3128	34.2016	OK	OK	-0.77533
ENSG0000007	-	-	ENSG0000007984	'RIMS1	-	6:71886702-72403143	-	-	IGV_LINK	0	0	0	0	OK	0	0	0	0	OK	NOTEST	0
ENSG0000007	-	-	ENSG0000007993	'MOXD1	-	6:132296054-132401545	-	-	IGV_LINK	80.4868	2.3642	0	5.67906	OK	130.966	3.42088	0	7.48695	OK	OK	0.533015
ENSG0000007	-	-	ENSG0000007995	'STX7	-	6:132445866-132513198	-	-	IGV_LINK	1877.38	52.5312	0	105.225	OK	1107.54	29.4445	0	59.4605	OK	OK	-0.83518
ENSG0000007	-	-	ENSG0000007997	'RABL2B	-	22:50756947-50801309	-	-	IGV_LINK	866.93	29.0976	0.537757	57.6575	OK	732.17	24.0619	1.64627	46.4776	OK	OK	-0.27415
ENSG0000007	-	-	ENSG0000007999	'KEAP1	-	19:10486119-10503741	-	-	IGV_LINK	1077.36	32.7425	6.82994	58.6551	OK	1614.9	43.4747	11.6276	75.3218	OK	OK	0.409011
ENSG0000008	-	-	ENSG0000008000	'DDX43	-	6:73368554-73452276	-	-	IGV_LINK	17.455	0.575927	0	2.37529	OK	15.4684	0.60858	0	2.74376	OK	OK	0.07956
ENSG0000008	-	-	ENSG0000008003	'PTPRH	-	19:55181247-55209506	-	-	IGV_LINK	0.969721	0.022183	0	0.40492	OK	75.2794	1.43062	0	2.9272	OK	OK	6.01104
ENSG0000008	-	-	ENSG0000008016	'DCT	-	13:94436807-94479682	-	-	IGV_LINK	66.9107	0.971095	0	2.07006	OK	10.3122	0.118844	0	0.365277	OK	OK	-3.03054
ENSG0000008	-	-	ENSG0000008018	'SLC35C2	-	20:46345979-46364458	-	-	IGV_LINK	1064.75	33.1323	10.4756	55.7891	OK	1197.25	37.477	13.1703	61.7837	OK	OK	0.177766
ENSG0000008	-	-	ENSG0000008020	'CRYBG3	-	3:97822039-97972457	-	-	IGV_LINK	219.157	3.24464	0	11.0028	OK	274.306	3.80391	0	10.5639	OK	OK	0.229425
ENSG0000008	-	-	ENSG0000008022	'EPHA6	-	3:96814580-97752460	-	-	IGV_LINK	131.882	3.93077	0.306159	7.55539	OK	79.4043	2.41301	0	4.84416	OK	OK	-0.70398
ENSG0000008	-	-	ENSG0000008029	'SCTR	-	2:119439842-119525301	-	-	IGV_LINK	87.2748	5.83046	0	16.9598	OK	53.6237	4.01747	0	12.5707	OK	OK	-0.53732
ENSG0000008	-	-	ENSG0000008029	'RFX3	-	9:3218296-3526004	-	-	IGV_LINK	308.371	2.03336	0.23604	3.83067	OK	213.464	1.74853	0	4.08769	OK	OK	-0.21772
ENSG0000008	-	-	ENSG0000008034	'RIF1	-	2:151409882-151734487	-	-	IGV_LINK	768.019	5.80495	0	15.1507	OK	543.456	5.39458	0	14.1148	OK	OK	-0.10577
ENSG0000008	-	-	ENSG0000008037	'RAB21	-	12:71754873-71800285	-	-	IGV_LINK	1004.63	24.4443	0	54.6365	OK	1069.38	29.6375	0	67.6126	OK	OK	0.277927
ENSG0000008	-	-	ENSG0000008049	'SLC4A4	-	4:71186756-71572087	-	-	IGV_LINK	1783.32	14.9313	6.08694	23.7756	OK	4235.24	34.4218	12.7119	56.1318	OK	OK	1.20499
ENSG0000008	-	-	ENSG0000008050	'SMARCA2	-	9:1980289-2193624	-	-	IGV_LINK	1979.2	48.568	3.64782	93.4882	OK	2303.76	56.1868	0	115.225	OK	OK	0.210225
ENSG0000008	-	-	ENSG0000008051	'RDH8	-	19:10013248-10022279	-	-	IGV_LINK	0	0	0	0	OK	0	0	0	0	OK	NOTEST	0
ENSG0000008	-	-	ENSG0000008054	'SESN1	-	6:108986436-109094819	-	-	IGV_LINK	453.829	11.4217	2.69715	20.1463	OK	275.337	6.57409	0.975777	12.1724	OK	OK	-0.79692
ENSG0000008	-	-	ENSG0000008056	'MID2	-	X:107825754-107935980	-	-	IGV_LINK	81.4565	2.34263	0	4.79726	OK	79.4043	2.04599	0	4.2	OK	OK	-0.19533
ENSG0000008	-	-	ENSG0000008057	'PIH1D3	-	X:107206631-107244243	-	-	IGV_LINK	0	0	0	0	OK	0	0	0	0	OK	NOTEST	0
ENSG0000008	-	-	ENSG0000008057	'COL5A3	-	19:9959560-10010471	-	-	IGV_LINK	998.812	10.0583	2.96826	17.1483	OK	648.641	6.2568	2.09505	10.4186	OK	OK	-0.68489
ENSG0000008	-	-	ENSG0000008060	'SRCAP	-	16:30697706-30776307	-	-	IGV_LINK	1739.49	16.5295	0	51.0413	OK	2263.38	19.6417	0	58.858	OK	OK	0.248871
ENSG0000008	-	-	ENSG0000008060	'PUM3	-	9:2717501-2844241	-	-	IGV_LINK	446.071	14.4865	1.63064	27.3425	OK	781.669	21.485	7.15895	35.811	OK	OK	0.568614
ENSG0000008	-	-	ENSG0000008061	'CPB2	-	13:46052805-46113332	-	-	IGV_LINK	0.969721	0.036855	0	0.206487	OK	1.03122	0.039222	0	0.167633	OK	NOTEST	0.089821
ENSG0000008	-	-	ENSG0000008064	'CHRNA3	-	15:78565519-78621295	-	-	IGV_LINK	2.90916	0.054117	0	0.610737	OK	1.03122	0.033879	0	0.581754	OK	NOTEST	-0.6757
ENSG0000008	-	-	ENSG0000008070	'KCNN2	-	5:114360944-114668410	-	-	IGV_LINK	9.69721	0.256257	0	0.764147	OK	8.2498	0.191927	0	0.628655	OK	NOTEST	-0.41703
ENSG0000008	-	-	ENSG0000008080	'CNOT4	-	7:135361794-135510127	-	-	IGV_LINK	375.282	6.86721	2.07043	11.664	OK	424.865	9.49817	1.96327	17.0331	OK	OK	0.467926
ENSG0000008	-	-	ENSG0000008081	'PSEN1	-	14:73136417-73223691	-	-	IGV_LINK	907.658	15.6868	2.33083	29.0428	OK	1163.22	19.7309	5.48706	33.9748	OK	OK	0.330909
ENSG0000008	-	-	ENSG0000008081	'CPOX	-	3:98497603-98593723	-	-	IGV_LINK	285.098	8.07985	0	21.7132	OK	404.24	10.3416	0	24.5869	OK	OK	0.356057
ENSG0000008	-	-	ENSG0000008082	'CLDND1	-	3:98497603-98593723	-	-	IGV_LINK	1030.81	58.2564	12.8183	103.695	OK	1051.85	57.8146	10.5946	105.035	OK	OK	-0.01098
ENSG0000008	-	-	ENSG0000008082	'MOK	-	14:102080737-102305200	-	-	IGV_LINK	410.192	15.7644	0	66.6818	OK	403.209	15.9143	0	73.1464	OK	OK	0.013656
ENSG0000008	-	-	ENSG0000008082	'HSP90AA1	-	14:102080737-102305200	-	-	IGV_LINK	5687.41	104.584	25.3404	183.827	OK	6131.66	111.894	33.0811	190.706	OK	OK	0.097466
ENSG0000008	-	-	ENSG0000008083	'RBL1	-	20:36996348-37097178	-	-	IGV_LINK	214.308	2.36453	0.568217	4.16085	OK	122.716	1.30253	0.152453	2.4526	OK	OK	-0.86024
ENSG0000008	-	-	ENSG0000008084	'DLGAP4	-	20:36306335-36573391	-	-	IGV_LINK	1281	30.7802	4.0974	57.4631	OK	1413.81	34.1493	3.61504	64.6836	OK	OK	0.149852
ENSG0000008	-	-	ENSG0000008085	'IGSF9B	-	11:133908563-133956985	-	-	IGV_LINK	124.124	5.39036	0	13.2804	OK	186.652	7.67348	0	17.4624	OK	OK	0.509498
ENSG0000008	-	-	ENSG0000008091	'CFHR2	-	1:196819744-196959226	-	-	IGV_LINK	0	0	0	0	OK	0	0	0	0	OK	NOTEST	0
ENSG0000008	-	-	ENSG0000008094	'CROCCP3	-	1:16460947-16499257	-	-	IGV_LINK	285.098	8.11319	0	25.1401	OK	173.246	5.44648	0	18.0097	OK	OK	-0.57494
ENSG0000008	-	-	ENSG0000008098	'NDC80	-	18:2571510-2616635	-	-	IGV_LINK	108.609	3.41182	0	7.32025	OK	90.7478	2.55921	0.197781	4.92064	OK	OK	-0.41484
ENSG0000008	-	-	ENSG0000008101	'AP4E1	-	15:50908671-51005900	-	-	IGV_LINK	195.884	1.69021	0.371941	3.00848	OK	234.088	2.00357	0.560198	3.44694	OK	OK	0.245368
ENSG0000008	-	-	ENSG0000008101	'RSBN1	-	1:113761831-113914086	-	-	IGV_LINK	392.737	6.42228	0	17.4654	OK	266.056	4.44162	0	12.3855	OK	OK	-0.532
ENSG0000008	-	-	ENSG0000008102	'MAGI3	-	1:113390748-113685923	-	-	IGV_LINK	67.8804	0.645623	0	1.30218	OK	141.278	1.35874	0.04271	2.67478	OK	OK	1.07351
ENSG0000008	-	-	ENSG0000008104	'CXCL2	-	4:74097034-74099293	-	-	IGV_LINK	29.0916	2.20758	0	4.86067	OK	400.115	29.0035	3.48471	54.5223	OK	OK	3.71569
ENSG0000008	-	-	ENSG0000008105	'AFP	-	4:73431137-73456174	-	-	IGV_LINK	0	0	0	0	OK	17.5308	1.83413	0	4.22555	OK	OK	inf
ENSG0000008	-	-	ENSG0000008105	'COL4A4	-	2:227002710-227164113	-	-	IGV_LINK	384.009	2.22587	0.761855	3.68989	OK	101.06	0.585854	0.07377	1.09794	OK	OK	-1.92576
ENSG0000008	-	-	ENSG0000008105	'TCF7	-	5:134114710-134371047	-	-	IGV_LINK	320.977	6.44474	0	29.5618	OK	286.681	6.91619	0	40.5929	OK	OK	0.101855
ENSG0000008	-	-	ENSG0000008108	'OSTM1	-	6:108041408-108165854	-	-	IGV_LINK	481.951	13.974	0	28.5171	OK	470.239	10.3402	0.317365	20.3631	OK	OK	-0.43447
ENSG0000008	-	-	ENSG0000008113	'CDH7	-	18:65750251-65890341	-	-	IGV_LINK	0	0	0	0	OK	0	0	0	0	OK	NOTEST	0
ENSG0000008	-	-	ENSG0000008114	'IMPG2	-	3:101222545-101320560	-	-	IGV_LINK	30.0613	0.207499	0	0.426646	OK	22.6869	0.156619	0	0.34505	OK	NOTEST	-0.40584
ENSG0000008	-	-	ENSG0000008115	'PCNP	-	3:101574094-101594437	-	-	IGV_LINK	1470.1	73.9078	6.46715	141.348	OK	1236.44	67.6087	16.0542	119.163	OK	OK	-0.12852
ENSG0000008	-	-	ENSG0000008117	'EXD2	-	14:69183019-69359308	-	-	IGV_LINK	346.19	6.83097	0	13.7669	OK	300.086	5.6752	0.698654	10.6517	OK	OK	-0.26742
ENSG0000008	-	-	ENSG0000008118	'ARG2	-	14:67619797-67695814	-	-	IGV_LINK	40.7283	3.19049	0	16.0732	OK	1510.74	122.406	0	253.507	OK	OK	5.26175
ENSG0000008	-	-	ENSG0000008118	'MEF2C	-	5:88507545-89466398	-	-	IGV_LINK	1251.91	23.8553	5.23287	42.4777	OK	962.133	17.0543	2.37638	31.7321	OK	OK	-0.48418
ENSG0000008	-	-	ENSG0000008123	'PTPRC	-	1:198638670-198757283	-	-	IGV_LINK	4946.54	75.2458	0	163.239	OK	3674.25	53.7731	0	112.832	OK	OK	-0.48473
ENSG0000008	-	-	ENSG0000008124	'CACNA1S	-	1:201031135-201112566	-	-	IGV_LINK	0	0	0	0	OK	3.09367	0.029941	0	0.080702	OK	NOTEST	inf
ENSG0000008	-	-	ENSG0000008127	'PKP1	-	1:201283451-201332993	-	-	IGV_LINK	133.821	1.88195	0	4.45	OK	5.15612	0.05576	0	0.297456	OK	OK	-5.07686
ENSG0000008	-	-	ENSG0000008130	'UBA5	-	3:132558137-132874223	-	-	IGV_LINK	2326.36	51.6782	0	125.266	OK	1357.09	34.5447	0	89.0917	OK	OK	-0.58109
ENSG0000008	-	-	ENSG0000008132	'STK17B	-	2:196133565-196176503	-	-	IGV_LINK	473.224	5.91788	1.68096	10.1548	OK	516.644	6.51207	1.41717	11.607	OK	OK	0.138035
ENSG0000008	-	-	ENSG0000008137	'CDC14B	-	9:96450200-96619830	-	-	IGV_LINK	716.623	9.28621	0	18.6526	OK	683.702	8.17322	2.06105	14.2854	OK	OK	-0.18419
ENSG0000008	-	-	ENSG0000008138	'ZNF510	-	9:96755864-96778129	-	-	IGV_LINK	298.674	4.28008	0	8.8273	OK	223.776	2.98608	0	6.43598	OK	OK	-0.51939
ENSG0000008	-	-	ENSG0000008147	'LRP2	-	2:169127108-169362685	-	-	IGV_LINK	7473.64	75.0442	0	221.284	OK	7686.75	108.98	0	302.646	OK	OK	0.538244
ENSG0000008	-	-	ENSG0000008166	'ZNF506	-	19:19757365-19836073	-	-	IGV_LINK	357.827	9.55601	0	20.8609	OK	352.679	10.5732	0	21.2523	OK	OK	0.145931
ENSG0000008	-	-	ENSG0000008169	'JMJD4	-	1:227728538-227781231	-	-	IGV_LINK	341.342	10.8259	0	24.4275	OK	508.394	14.7163	0.064679	29.3678	OK	OK	0.442928
ENSG0000008	-	-	ENSG0000008172	'DUSP12	-	1:161749757-161757238	-	-	IGV_LINK	226.915	17.9922	1.26784	34.7165	OK	218.62	15.6505	2.02881	29.2723	OK	OK	-0.20116
ENSG0000008	-	-	ENSG0000008176	'AACS	-	12:125065378-125143333	-	-	IGV_LINK	272.491	5.21279	0.565712	9.85986	OK	345.46	6.31173	1.54463	11.0788	OK	OK	0.27598
ENSG0000008	-	-	ENSG0000008179	'KIAA0141	-	5:141923807-141942047	-	-	IGV_LINK	1949.14	65.3792	17.1144	113.644	OK	2750.28	87.2992	21.5239	153.075	OK	OK	0.417137
ENSG0000008	-	-	ENSG0000008180	'SLC13A1	-	7:123113530-123199986	-	-	IGV_LINK	75.6382	1.54925	0.07288	3.02561	OK	6.18735	0.142708	0	0.348554	OK	OK	-3.44043
ENSG0000008	-	-	ENSG0000008180	'CADPS2	-	7:122318424-122886759	-	-	IGV_LINK	308.371	4.98824	1.44192	8.53455	OK	248.525	4.15068	0.571949	7.72941	OK	OK	-0.26518
ENSG0000008	-	-	ENSG0000008181	'PCDHB4	-	5:141100241-141249365	-	-	IGV_LINK	30.0613	0.843425	0	4.82581	OK	13.4059	0.221871	0	1.60241	OK	OK	-1.92654
ENSG0000008	-	-	ENSG0000008184	'PCDHA6	-	5:140786135-141012347	-	-	IGV_LINK	16.3211	0.220927	0	1.17949	OK	14.337	0.165425	0	0.973602	OK	NOTEST	-0.41739
ENSG0000008	-	-	ENSG0000008185	'PCDHGA2	-	5:141330570-141512981	-	-	IGV_LINK	18.4344	0.2377	0	2.36428	OK	42.9515	0.55021	0	3.88203	OK	NOTEST	1.21084
ENSG0000008	-	-	ENSG0000008187	'HSPB11	-	1:53916573-53945929	-	-	IGV_LINK	314.189	25.2834	8.30065	42.2661	OK	294.93	23.103	7.48912	38.7169	OK	OK	-0.13011
ENSG0000008	-	-	ENSG0000008191	'PHLPP1	-	18:62715449-62980433	-	-	IGV_LINK	125.094	1.13386	0	2.26778	OK	180.464	1.63605	0.222358	3.04974	OK	OK	0.528979
ENSG0000008	-	-	ENSG0000008192	'ATP8B1	-	18:57630301-57803101	-	-	IGV_LINK	145.458	2.05623	0	6.02809	OK	173.246	3.13488	0	8.98214	OK	OK	0.60841
ENSG0000008	-	-	ENSG0000008198	'IL12RB2	-	1:67307363-67396900	-	-	IGV_LINK	197.823	3.19255	0.266237	6.11887	OK	35.0616	0.705241	0	1.83284	OK	OK	-2.17852
ENSG0000008	-	-	ENSG0000008201	'SMARCD3	-	7:151232488-151277896	-	-	IGV_LINK	201.702	11.4148	0	29.2459	OK	246.463	13.982	0	37.2721	OK	OK	0.292664
ENSG0000008	-	-	ENSG0000008206	'WDR70	-	5:37379211-37753435	-	-	IGV_LINK	644.864	18.8998	2.77317	35.0264	OK	565.111	22.6751	2.14667	43.2036	OK	OK	0.26274
ENSG0000008	-	-	ENSG0000008207	'FYB1	-	5:39105235-39274528	-	-	IGV_LINK	2089.75	37.9925	9.69438	66.2906	OK	1858.27	34.3166	9.45292	59.1802	OK	OK	-0.14681
ENSG0000008	-	-	ENSG0000008212	'MPP4	-	2:201644869-201698694	-	-	IGV_LINK	0.969721	0.02909	0	0.316402	OK	2.06245	0.061927	0	0.366668	OK	NOTEST	1.09004
ENSG0000008	-	-	ENSG0000008214	'STRADB	-	2:201377206-201480846	-	-	IGV_LINK	505.224	17.0331	0	37.2163	OK	713.608	25.5415	0	53.8741	OK	OK	0.584503
ENSG0000008	-	-	ENSG0000008215	'BZW1	-	2:200678232-200827338	-	-	IGV_LINK	1413.85	47.079	6.8494	87.3087	OK	1913.95	65.2364	11.1337	119.339	OK	OK	0.470592
ENSG0000008	-	-	ENSG0000008217	'PGR	-	11:101029623-101209591	-	-	IGV_LINK	32.9705	0.136908	0	0.55033	OK	3.09367	0.026922	0	0.34924	OK	NOTEST	-2.34637
ENSG0000008	-	-	ENSG0000008219	'C1QTNF3	-	5:33986177-34124528	-	-	IGV_LINK	233.62	11.3483	0	31.8102	OK	126.087	5.81839	0	29.7292	OK	OK	-0.96379
ENSG0000008	-	-	ENSG0000008221	'ME2	-	18:50878733-50954257	-	-	IGV_LINK	850.445	20.9403	8.32099	33.5596	OK	821.886	20.4422	8.16663	32.7177	OK	OK	-0.03473
ENSG0000008	-	-	ENSG0000008221	'C5orf22	-	5:31532265-31555058	-	-	IGV_LINK	246.309	5.89414	0	11.8737	OK	255.744	6.66552	0	13.3832	OK	OK	0.177436
ENSG0000008	-	-	ENSG0000008225	'CCNT2	-	2:134735463-134959342	-	-	IGV_LINK	1018.21	15.0221	0.259506	29.7846	OK	905.416	12.184	0	27.5411	OK	OK	-0.3021
ENSG0000008	-	-	ENSG0000008226	'FAM135A	-	6:70412827-70561174	-	-	IGV_LINK	465.466	7.04877	0	14.1909	OK	196.964	2.86078	0	6.24664	OK	OK	-1.30096
ENSG0000008	-	-	ENSG0000008229	'COL19A1	-	6:69866570-70209976	-	-	IGV_LINK	12.6064	0.27568	0	0.97564	OK	4.1249	0.0721	0	0.61509	OK	NOTEST	-1.93493
ENSG0000008	-	-	ENSG0000008239	'EPB41L3	-	18:5392380-5630700	-	-	IGV_LINK	895.052	18.8633	2.38582	35.3407	OK	2169.7	47.2336	6.50691	87.9603	OK	OK	1.32423
ENSG0000008	-	-	ENSG0000008243	'COBLL1	-	2:164653623-165392310	-	-	IGV_LINK	661.349	10.3511	0	22.3347	OK	296.993	5.01447	0	12.413	OK	OK	-1.04562
ENSG0000008	-	-	ENSG0000008245	'DLG3	-	X:70444860-70505490	-	-	IGV_LINK	755.412	15.4912	1.56171	29.4207	OK	484.676	8.48313	0.667045	16.2992	OK	OK	-0.86878
ENSG0000008	-	-	ENSG0000008248	'KCNK2	-	1:215005774-215237093	-	-	IGV_LINK	41.698	1.31205	0	3.26143	OK	22.6869	0.659884	0	1.67832	OK	OK	-0.99154
ENSG0000008	-	-	ENSG0000008249	'SERTAD4	-	1:210231455-210246631	-	-	IGV_LINK	61.0924	0.824784	0	2.14671	OK	19.5933	0.256151	0	0.800606	OK	OK	-1.68702
ENSG0000008	-	-	ENSG0000008251	'TRAF5	-	1:211326614-211374946	-	-	IGV_LINK	908.628	16.2276	6.2645	26.1906	OK	419.709	6.86016	2.10487	11.6155	OK	OK	-1.24213
ENSG0000008	-	-	ENSG0000008251	'MRPL22	-	5:154941069-154969411	-	-	IGV_LINK	419.889	35.8099	11.2283	60.3915	OK	454.77	38.5522	11.0709	66.0335	OK	OK	0.106455
ENSG0000008	-	-	ENSG0000008251	'GEMIN5	-	5:154887415-154938209	-	-	IGV_LINK	537.225	5.79498	2.22686	9.36309	OK	641.422	6.92045	2.78333	11.0576	OK	OK	0.256063
ENSG0000008	-	-	ENSG0000008255	'OPRK1	-	8:53177570-53251697	-	-	IGV_LINK	0	0	0	0	OK	0	0	0	0	OK	NOTEST	0
ENSG0000008	-	-	ENSG0000008264	'NFE2L1	-	17:48048328-48061487	-	-	IGV_LINK	11404.9	163.347	20.8962	305.797	OK	14897.1	225.726	0	458.035	OK	OK	0.466637
ENSG0000008	-	-	ENSG0000008268	'SEMA5B	-	3:122909192-123028605	-	-	IGV_LINK	3305.78	66.3198	17.7169	114.923	OK	2619.31	52.658	8.78957	96.5264	OK	OK	-0.33279
ENSG0000008	-	-	ENSG0000008270	'GSK3B	-	3:119821322-120094417	-	-	IGV_LINK	698.199	9.22477	0	19.4128	OK	746.607	8.96182	0	18.2924	OK	OK	-0.04172
ENSG0000008	-	-	ENSG0000008278	'ITGB5	-	3:124761947-124901418	-	-	IGV_LINK	1352.76	27.3895	5.09563	49.6833	OK	2045.95	53.2744	7.95771	98.5912	OK	OK	0.959821
ENSG0000008	-	-	ENSG0000008280	'ERC1	-	12:912076-1495933	-	-	IGV_LINK	965.842	6.06357	1.72032	10.4068	OK	1096.19	7.02838	2.2664	11.7904	OK	OK	0.213025
ENSG0000008	-	-	ENSG0000008289	'XPO1	-	2:61471187-61538626	-	-	IGV_LINK	2159.57	43.1663	0	91.6095	OK	1806.71	29.7706	2.53979	57.0013	OK	OK	-0.53602

ENSG0000008	-	-	ENSG0000008292	'LINC01587	-	4:5524568-5527801	-	-	IGV_LINK	252.127	21.026	6.20908	35.843	OK	614.61	50.6005	19.9583	81.2427	OK	OK	1.26697
ENSG0000008	-	-	ENSG0000008299	'RNF13	-	3:149761099-150225190	-	-	IGV_LINK	1152.03	46.87	9.76918	83.9708	OK	1403.5	55.9971	8.8747	103.12	OK	OK	0.256689
ENSG0000008	-	-	ENSG0000008306	'TRPM3	-	9:70529062-71446904	-	-	IGV_LINK	621.591	4.2322	0	9.00749	OK	354.741	2.77598	0	5.92133	OK	OK	-0.60841
ENSG0000008	-	-	ENSG0000008309	'PALB2	-	16:23603159-23641310	-	-	IGV_LINK	241.46	4.4972	0	10.8661	OK	250.588	3.89582	0.619114	7.17253	OK	OK	-0.2071
ENSG0000008	-	-	ENSG0000008309	'DOPEY1	-	6:83067665-83198932	-	-	IGV_LINK	700.138	7.24239	0	19.9151	OK	315.555	2.88454	0	7.02283	OK	OK	-1.32813
ENSG0000008	-	-	ENSG0000008309	'LYRM2	-	6:89433169-89638753	-	-	IGV_LINK	607.045	13.6632	0	30.5714	OK	443.427	8.81243	0	19.6579	OK	OK	-0.63268
ENSG0000008	-	-	ENSG0000008312	'BCKDHB	-	6:80106646-80346270	-	-	IGV_LINK	253.097	6.04535	0.764635	11.3261	OK	163.965	4.5233	0.574899	8.4717	OK	OK	-0.41845
ENSG0000008	-	-	ENSG0000008316	'KAT6A	-	8:41929478-42051990	-	-	IGV_LINK	1313	9.53265	0.531946	18.5334	OK	1078.66	6.78677	2.88252	10.691	OK	OK	-0.49015
ENSG0000008	-	-	ENSG0000008322	'ZCCHC6	-	9:86287732-86354454	-	-	IGV_LINK	729.23	10.6162	0.837139	20.3953	OK	824.98	10.8875	2.3668	19.4082	OK	OK	0.036407
ENSG0000008	-	-	ENSG0000008329	'ULK2	-	17:19770828-19867936	-	-	IGV_LINK	617.712	8.49596	1.31302	15.6789	OK	417.646	7.00292	0	14.7005	OK	OK	-0.27882
ENSG0000008	-	-	ENSG0000008330	'GRHL2	-	8:101461176-101669726	-	-	IGV_LINK	0	0	0	0	OK	1.03122	0.011473	0	0.668771	OK	NOTEST	inf
ENSG0000008	-	-	ENSG0000008331	'TNPO1	-	5:72794404-72916733	-	-	IGV_LINK	1039.54	13.2006	1.63232	24.7688	OK	1048.76	15.4333	0.573006	30.2936	OK	OK	0.225448
ENSG0000008	-	-	ENSG0000008344	'PLOD1	-	1:11934204-11975538	-	-	IGV_LINK	3068.2	70.1178	22.7983	117.437	OK	4583.8	104.561	33.2738	175.848	OK	OK	0.576492
ENSG0000008	-	-	ENSG0000008345	'P2RX5	-	17:3662895-3696404	-	-	IGV_LINK	62.4762	2.90554	0	9.78203	OK	55.4401	2.32689	0	9.14362	OK	OK	-0.3204
ENSG0000008	-	-	ENSG0000008345	'ITGAE	-	17:3714627-3801243	-	-	IGV_LINK	547.892	20.7635	2.18816	39.3389	OK	284.618	17.8825	2.45548	33.3095	OK	OK	-0.21551
ENSG0000008	-	-	ENSG0000008352	'DIS3	-	13:72727748-73016461	-	-	IGV_LINK	790.322	10.3887	2.2854	18.4919	OK	846.636	11.4357	2.55348	20.318	OK	OK	0.13854
ENSG0000008	-	-	ENSG0000008353	'PIBF1	-	13:72727748-73016461	-	-	IGV_LINK	253.097	5.03305	0	11.8934	OK	274.306	5.41778	0	12.7354	OK	OK	0.106268
ENSG0000008	-	-	ENSG0000008354	'TDRD3	-	13:60396456-60573878	-	-	IGV_LINK	520.74	12.5634	4.54474	20.582	OK	283.587	6.70632	1.64288	11.7698	OK	OK	-0.90563
ENSG0000008	-	-	ENSG0000008362	'AC000061.1	-	7:117465783-117874139	-	-	IGV_LINK	0	0	0	0	OK	0	0	0	0	OK	NOTEST	0
ENSG0000008	-	-	ENSG0000008363	'NUFIP1	-	13:44939248-45083125	-	-	IGV_LINK	84.3657	1.43229	0	4.2212	OK	122.716	2.08409	0	4.82002	OK	OK	0.541092
ENSG0000008	-	-	ENSG0000008364	'PDS5B	-	13:32586426-32778019	-	-	IGV_LINK	490.679	7.74612	0.050803	15.4414	OK	343.398	5.71738	0	11.7951	OK	OK	-0.43812
ENSG0000008	-	-	ENSG0000008372	'OXCT1	-	5:41730064-41872241	-	-	IGV_LINK	406.313	9.74686	0	20.7436	OK	162.934	3.23579	0	7.23482	OK	OK	-1.59082
ENSG0000008	-	-	ENSG0000008375	'RRAGB	-	X:55717738-55758774	-	-	IGV_LINK	279.28	15.0652	0	30.2421	OK	183.558	9.84965	0	20.0904	OK	OK	-0.61308
ENSG0000008	-	-	ENSG0000008378	'EPYC	-	12:90963678-91005026	-	-	IGV_LINK	1.93944	0.17701	0	0.60872	OK	0	0	0	0	OK	NOTEST	'-inf
ENSG0000008	-	-	ENSG0000008379	'CYLD	-	16:50693602-50803338	-	-	IGV_LINK	1840.53	24.1709	0	49.8903	OK	1153.94	15.5732	0	33.9283	OK	OK	-0.63421
ENSG0000008	-	-	ENSG0000008380	'SLC27A5	-	19:58467044-58512413	-	-	IGV_LINK	111.518	5.05172	0	14.184	OK	84.5604	3.19646	0	10.0485	OK	OK	-0.6603
ENSG0000008	-	-	ENSG0000008381	'ZNF324	-	19:58467044-58512413	-	-	IGV_LINK	151.481	3.71867	0	9.19561	OK	128.796	2.40198	0	6.34846	OK	OK	-0.63057
ENSG0000008	-	-	ENSG0000008381	'ZNF671	-	19:57681968-57758159	-	-	IGV_LINK	158.064	4.04689	0	10.7269	OK	111.372	2.98823	0	7.81617	OK	OK	-0.43752
ENSG0000008	-	-	ENSG0000008381	'ZNF416	-	19:57571565-57593777	-	-	IGV_LINK	71.7593	1.69314	0	3.62733	OK	64.9672	1.53362	0	3.32819	OK	OK	-0.14276
ENSG0000008	-	-	ENSG0000008382	'ZNF586	-	19:57769654-57916610	-	-	IGV_LINK	91.2115	3.7179	0	15.6817	OK	139.261	6.01758	0	22.3895	OK	OK	0.694695
ENSG0000008	-	-	ENSG0000008383	'ZNF446	-	19:58467044-58512413	-	-	IGV_LINK	199.558	5.89899	0	12.4721	OK	196.04	7.1029	0	15.5014	OK	OK	0.267939
ENSG0000008	-	-	ENSG0000008384	'ZNF264	-	19:57191499-57222846	-	-	IGV_LINK	524.619	5.37461	0	11.7476	OK	428.99	4.25466	0	10.5887	OK	OK	-0.33712
ENSG0000008	-	-	ENSG0000008384	'RPS5	-	19:58386399-58394806	-	-	IGV_LINK	9098.89	902.833	312.674	1492.99	OK	6370.91	633.657	246.715	1020.6	OK	OK	-0.51076
ENSG0000008	-	-	ENSG0000008385	'FAT1	-	4:186587782-186726722	-	-	IGV_LINK	3335.84	43.4642	0	96.4246	OK	4684.86	92.7214	0	208.237	OK	OK	1.09307
ENSG0000008	-	-	ENSG0000008389	'YTHDC1	-	4:68310386-68350089	-	-	IGV_LINK	1754.22	49.5676	8.10626	91.0288	OK	1323.06	33.7953	6.37295	61.2177	OK	OK	-0.55257
ENSG0000008	-	-	ENSG0000008393	'CHMP2B	-	3:87227270-87255548	-	-	IGV_LINK	354.918	13.8951	1.80193	25.9882	OK	493.957	19.9599	4.84354	35.0763	OK	OK	0.522531
ENSG0000008	-	-	ENSG0000008407	'SMAP2	-	1:40344849-40423326	-	-	IGV_LINK	1449.73	34.618	8.00168	61.2344	OK	1784.02	43.2073	9.70074	76.7138	OK	OK	0.319749
ENSG0000008	-	-	ENSG0000008407	'PPIE	-	1:39692181-39788861	-	-	IGV_LINK	887.294	47.6367	16.3542	78.9191	OK	859.01	44.9762	17.017	72.9353	OK	OK	-0.08291
ENSG0000008	-	-	ENSG0000008407	'ZMPSTE24	-	1:40258106-40294184	-	-	IGV_LINK	638.076	12.9336	3.86047	22.0067	OK	870.354	16.8941	6.78635	27.0019	OK	OK	0.385399
ENSG0000008	-	-	ENSG0000008409	'STARD7	-	2:96184858-96242621	-	-	IGV_LINK	1876.41	36.9896	1.85952	72.1197	OK	1947.98	35.9615	13.5985	58.3245	OK	OK	-0.04067
ENSG0000008	-	-	ENSG0000008409	'NOA1	-	4:56907875-57205510	-	-	IGV_LINK	513.952	8.93267	0	44.0627	OK	485.707	8.44476	0	49.9699	OK	OK	-0.08104
ENSG0000008	-	-	ENSG0000008409	'REST	-	4:56907875-57205510	-	-	IGV_LINK	520.74	4.44752	0	25.1769	OK	510.456	4.56336	0	31.56	OK	OK	0.037094
ENSG0000008	-	-	ENSG0000008411	'HAL	-	12:95972661-95996365	-	-	IGV_LINK	11.6366	0.300218	0	1.07062	OK	59.811	1.39073	0	3.74676	OK	OK	2.21176
ENSG0000008	-	-	ENSG0000008411	'SSH1	-	12:108778143-108857590	-	-	IGV_LINK	1409.97	11.7121	0	28.7965	OK	1607.68	12.2015	0	27.617	OK	OK	0.059058
ENSG0000008	-	-	ENSG0000008420	'GSTP1	-	11:67583594-67586660	-	-	IGV_LINK	5217.1	370.992	149.118	592.865	OK	6917.46	494.443	187.54	801.346	OK	OK	0.414418
ENSG0000008	-	-	ENSG0000008423	'APLP2	-	11:130069836-130144811	-	-	IGV_LINK	10450.7	211.139	20.344	401.934	OK	14604.2	305.362	0	618.943	OK	OK	0.532329
ENSG0000008	-	-	ENSG0000008444	'FAM234B	-	12:13044283-13142521	-	-	IGV_LINK	150.307	2.35498	0	4.84485	OK	260.9	3.42944	0.97467	5.88421	OK	OK	0.542257
ENSG0000008	-	-	ENSG0000008445	'SLCO1A2	-	12:21264599-21419594	-	-	IGV_LINK	4.8486	0.177686	0	1.0364	OK	6.18735	0.188356	0	1.01894	OK	NOTEST	0.084133
ENSG0000008	-	-	ENSG0000008446	'WBP11	-	12:14784578-14803540	-	-	IGV_LINK	1150.09	19.1525	3.14504	35.16	OK	1115.79	21.4413	3.02262	39.86	OK	OK	0.162859
ENSG0000008	-	-	ENSG0000008462	'EIF3I	-	1:32209093-32231604	-	-	IGV_LINK	1976.29	88.8811	27.6457	150.116	OK	2428.53	108.415	33.7337	183.097	OK	OK	0.286618
ENSG0000008	-	-	ENSG0000008462	'NKAIN1	-	1:31179744-31239554	-	-	IGV_LINK	17.455	0.407077	0	1.05563	OK	175.308	4.09037	0.927414	7.25333	OK	OK	3.32886
ENSG0000008	-	-	ENSG0000008463	'COL16A1	-	1:31617685-31704319	-	-	IGV_LINK	175.519	4.31508	0	12.7543	OK	147.465	4.81216	0	16.4605	OK	OK	0.157298
ENSG0000008	-	-	ENSG0000008465	'TXLNA	-	1:32179685-32198285	-	-	IGV_LINK	1295.55	15.4435	6.81346	24.0734	OK	1935.61	23.0787	10.1637	35.9937	OK	OK	0.579568
ENSG0000008	-	-	ENSG0000008467	'APOB	-	2:21001428-21044073	-	-	IGV_LINK	16.4852	0.066605	0	0.151275	OK	3051.39	12.3294	5.35369	19.3051	OK	OK	7.53227
ENSG0000008	-	-	ENSG0000008467	'NCOA1	-	2:24491913-24770702	-	-	IGV_LINK	1163.66	10.756	2.42972	19.0823	OK	910.572	8.27904	1.95719	14.6009	OK	OK	-0.37761
ENSG0000008	-	-	ENSG0000008469	'AGBL5	-	2:27042363-27071773	-	-	IGV_LINK	1002.69	25.4703	0	55.4633	OK	1267.38	33.1397	0	69.5965	OK	OK	0.379743
ENSG0000008	-	-	ENSG0000008471	'EFR3B	-	2:25042129-25159137	-	-	IGV_LINK	46.5466	0.376358	0	0.768093	OK	56.7174	0.446522	0	0.893697	OK	NOTEST	0.246626
ENSG0000008	-	-	ENSG0000008473	'KIF3C	-	2:25926595-25982749	-	-	IGV_LINK	177.459	2.43111	0	7.15767	OK	204.183	3.51855	0	9.76406	OK	OK	0.533366
ENSG0000008	-	-	ENSG0000008473	'RAB10	-	2:26034106-26137454	-	-	IGV_LINK	1470.1	56.9775	0	119.331	OK	1860.33	74.4864	11.6375	137.335	OK	OK	0.386586
ENSG0000008	-	-	ENSG0000008473	'GCKR	-	2:27496841-27523684	-	-	IGV_LINK	0	0	0	0	OK	3.09367	0.227285	0	0.756449	OK	NOTEST	inf
ENSG0000008	-	-	ENSG0000008475	'HADHA	-	2:26190634-26290468	-	-	IGV_LINK	4328.83	96.3997	4.71051	188.089	OK	5879.01	128.523	6.29045	250.755	OK	OK	0.414925
ENSG0000008	-	-	ENSG0000008476	'MAPRE3	-	2:26847746-27027196	-	-	IGV_LINK	354.918	14.3394	3.84336	24.8354	OK	526.956	22.8266	7.36065	38.2925	OK	OK	0.670734
ENSG0000008	-	-	ENSG0000008477	'CAD	-	2:27217389-27243943	-	-	IGV_LINK	825.232	9.75523	0	20.3498	OK	853.854	12.8368	0	27.5613	OK	OK	0.396035
ENSG0000008	-	-	ENSG0000008506	'CD59	-	11:33698260-33736445	-	-	IGV_LINK	4862.18	211.829	56.8108	366.848	OK	5519.12	252.097	63.2119	440.982	OK	OK	0.251078
ENSG0000008	-	-	ENSG0000008511	'CD82	-	11:44564426-44620363	-	-	IGV_LINK	623.53	34.9949	7.70673	62.2831	OK	1035.35	53.5019	15.333	91.6707	OK	OK	0.612445
ENSG0000008	-	-	ENSG0000008518	'BCORL1	-	X:129981106-130058083	-	-	IGV_LINK	307.401	3.37598	0.204156	6.54781	OK	375.366	4.39498	0.306402	8.48356	OK	OK	0.380549
ENSG0000008	-	-	ENSG0000008522	'ATRX	-	X:77504877-77786269	-	-	IGV_LINK	1138.45	14.7568	0	35.5716	OK	750.732	12.0212	0	28.8599	OK	OK	-0.2958
ENSG0000008	-	-	ENSG0000008523	'AK6	-	5:69350983-69414801	-	-	IGV_LINK	225.719	24.9101	0	60.0845	OK	257.867	28.4986	0	67.1024	OK	OK	0.194165
ENSG0000008	-	-	ENSG0000008526	'FCN1	-	9:134905889-134917963	-	-	IGV_LINK	336.493	12.6513	3.43655	21.866	OK	513.55	19.6028	6.26949	32.936	OK	OK	0.631774
ENSG0000008	-	-	ENSG0000008527	'MYNN	-	3:169772830-169789716	-	-	IGV_LINK	233.703	7.28734	0	14.7031	OK	272.243	8.28731	0.458742	16.1159	OK	OK	0.185512
ENSG0000008	-	-	ENSG0000008527	'MECOM	-	3:169083498-169663618	-	-	IGV_LINK	250.188	3.53796	0	7.83721	OK	481.582	6.53785	1.1935	11.8822	OK	OK	0.885901
ENSG0000008	-	-	ENSG0000008536	'SCAMP1	-	5:78360582-78479071	-	-	IGV_LINK	480.012	12.5629	2.00145	23.1244	OK	579.548	14.4779	3.47964	25.4761	OK	OK	0.204677
ENSG0000008	-	-	ENSG0000008537	'PREP	-	6:105277564-105403084	-	-	IGV_LINK	390.797	8.30647	2.52351	14.0894	OK	301.118	6.63763	1.816	11.4593	OK	OK	-0.32357
ENSG0000008	-	-	ENSG0000008538	'HACE1	-	6:104728092-104859919	-	-	IGV_LINK	250.188	4.79014	0.85531	8.72498	OK	142.309	3.06567	0.160467	5.97088	OK	OK	-0.64387
ENSG0000008	-	-	ENSG0000008541	'SEH1L	-	18:12947132-12991173	-	-	IGV_LINK	396.616	11.3835	1.39165	21.3753	OK	394.959	11.4018	1.697	21.1066	OK	OK	0.002323
ENSG0000008	-	-	ENSG0000008543	'WDR47	-	1:108970213-109042113	-	-	IGV_LINK	287.037	4.02706	1.13027	6.92386	OK	280.493	3.93348	1.12342	6.74354	OK	OK	-0.03392
ENSG0000008	-	-	ENSG0000008544	'WDFY1	-	2:223855715-223945387	-	-	IGV_LINK	822.323	17.5081	0	39.6871	OK	886.853	16.1721	0	34.9566	OK	OK	-0.11451
ENSG0000008	-	-	ENSG0000008546	'OVGP1	-	1:111414313-111427777	-	-	IGV_LINK	77.5776	2.58879	0	5.65229	OK	119.622	4.0226	0	8.2368	OK	OK	0.635852
ENSG0000008	-	-	ENSG0000008549	'SLC25A24	-	1:108134035-108202743	-	-	IGV_LINK	501.345	8.51967	2.88884	14.1505	OK	641.422	10.9091	4.06213	17.7561	OK	OK	0.356664
ENSG0000008	-	-	ENSG0000008551	'MAP3K4	-	6:160990317-161117385	-	-	IGV_LINK	567.286	10.1853	0.150757	20.2198	OK	308.336	4.82705	0	11.8457	OK	OK	-1.07727
ENSG0000008	-	-	ENSG0000008551	'PILRA	-	7:100307701-100400099	-	-	IGV_LINK	589.59	33.5996	0	88.6514	OK	986.882	55.3999	0	137.865	OK	OK	0.721438
ENSG0000008	-	-	ENSG0000008555	'IGSF9	-	1:159927038-159945604	-	-	IGV_LINK	16.4852	0.24297	0	0.528099	OK	16.4996	0.248393	0	0.543532	OK	NOTEST	0.031846
ENSG0000008	-	-	ENSG0000008556	'ABCB1	-	7:87503632-87832296	-	-	IGV_LINK	566.317	11.8213	0	24.3785	OK	1001.32	22.3359	2.00241	42.6695	OK	OK	0.917978
ENSG0000008	-	-	ENSG0000008564	'ZNF213	-	16:3110459-3142805	-	-	IGV_LINK	296.734	6.30412	0	15.557	OK	240.275	5.09212	0	13.34	OK	OK	-0.30803
ENSG0000008	-	-	ENSG0000008566	'AKR1B1	-	7:134442349-134459284	-	-	IGV_LINK	2175.08	102.424	40.4607	164.388	OK	2548.16	118.616	48.8509	188.381	OK	OK	0.211737
ENSG0000008	-	-	ENSG0000008571	'CPNE3	-	8:86514426-86765023	-	-	IGV_LINK	1681.5	28.1498	2.98407	53.3155	OK	1642.74	32.6759	0	123.498	OK	OK	0.215102
ENSG0000008	-	-	ENSG0000008572	'RRN3	-	16:14974590-15154564	-	-	IGV_LINK	613.833	10.9503	0	34.2648	OK	998.226	16.7589	0	52.2083	OK	OK	0.613954
ENSG0000008	-	-	ENSG0000008573	'CTTN	-	11:70270699-70436584	-	-	IGV_LINK	3809.06	108.173	10.4504	205.896	OK	5376.81	141.559	15.5359	267.583	OK	OK	0.388062
ENSG0000008	-	-	ENSG0000008574	'WNT11	-	11:76186324-76210736	-	-	IGV_LINK	2.90916	0.372044	0	1.09619	OK	3.09367	0.099461	0	0.529718	OK	NOTEST	-1.90327
ENSG0000008	-	-	ENSG0000008576	'MTIF2	-	2:55236594-55269347	-	-	IGV_LINK	785.474	21.5811	7.0148	36.1473	OK	809.512	22.1449	7.71748	36.5723	OK	OK	0.037209
ENSG0000008	-	-	ENSG0000008578	'DDHD2	-	8:38225217-38382272	-	-	IGV_LINK	1081.24	35.3294	0	87.3981	OK	871.385	32.3472	0	81.9798	OK	OK	-0.12723
ENSG0000008	-	-	ENSG0000008583	'TTC39A	-	1:51287257-51345116	-	-	IGV_LINK	1364.4	43.692	15.1961	72.1878	OK	1600.46	49.8082	17.7631	81.8533	OK	OK	0.189016
ENSG0000008	-	-	ENSG0000008583	'EPS15	-	1:51354262-51561629	-	-	IGV_LINK	1399.31	16.0797	6.08352	26.0759	OK	1356.06	15.4095	6.20482	24.6141	OK	OK	-0.06143
ENSG0000008	-	-	ENSG0000008584	'ORC1	-	1:52372828-52404459	-	-	IGV_LINK	58.1832	1.12776	0	2.2888	OK	76.3106	1.44152	0.102949	2.78009	OK	OK	0.354131
ENSG0000008	-	-	ENSG0000008587	'MGST2	-	4:139665767-140154184	-	-	IGV_LINK	568.256	51.6377	13.8773	89.3981	OK	947.696	87.0002	28.3925	145.608	OK	OK	0.752594
ENSG0000008	-	-	ENSG0000008587	'CHERP	-	19:16479056-16660442	-	-	IGV_LINK	688.502	15.8331	0	53.8208	OK	808.463	19.9718	0	68.9265	OK	OK	0.335025
ENSG0000008	-	-	ENSG0000008597	'ATG16L1	-	2:233210050-233295674	-	-	IGV_LINK	527.528	10.1901	2.25874	18.1214	OK	2281.07	43.4513	15.0189	71.8836	OK	OK	2.09223
ENSG0000008	-	-	ENSG0000008598	'USP40	-	2:233475519-233566782	-	-	IGV_LINK	1678.59	30.0964	0	76.2869	OK	1614.9	28.13	0	73.997	OK	OK	-0.09748
ENSG0000008	-	-	ENSG0000008599	'POMGNT1	-	1:46188681-46221261	-	-	IGV_LINK	1920.05	49.3416	11.0144	87.6689	OK	2382.13	62.3547	14.0986	110.611	OK	OK	0.337692
ENSG0000008	-	-	ENSG0000008599	'RAD54L	-	1:46247687-46303608	-	-	IGV_LINK	64.9713	2.28864	0	16.7326	OK	52.5925	2.52575	0	16.7521	OK	OK	0.142221
ENSG0000008	-	-	ENSG0000008601	'MAST2	-	1:45786986-46036124	-	-	IGV_LINK	497.467	6.62415	0	14.8239	OK	652.765	9.00783	0	19.6799	OK	OK	0.443445
ENSG0000008	-	-	ENSG0000008606	'DNAJA1	-	9:33025210-33039907	-	-	IGV_LINK	2310.84	61.2485	23.536	98.961	OK	2723.47	70.17	29.8004	110.539	OK	OK	0.196179
ENSG0000008	-	-	ENSG0000008606	'B4GALT1	-	9:33104081-33179983	-	-	IGV_LINK	1323.67	18.5171	8.17598	28.8583	OK	4967.41	69.5102	27.2328	111.788	OK	OK	1.90836
ENSG0000008	-	-	ENSG0000008606	'CHMP5	-	9:33264878-33282069	-	-	IGV_LINK	1425.49	62.2619	27.0297	97.4942	OK	1983.05	86.9486	37.522	136.375	OK	OK	0.481813
ENSG0000008	-	-	ENSG0000008610	'NFX1	-	9:33290510-33371157	-	-	IGV_LINK	1489.49	23.5028	8.40317	38.6025	OK	1582.93	25.2969	8.38567	42.2081	OK	OK	0.106126
ENSG0000008	-	-	ENSG0000008615	'AQP6	-	12:49967193-50033136	-	-	IGV_LINK	3.87888	0.162353	0	1.40917	OK	1.03122	0.028057	0	0.4284	OK	NOTEST	-2.53271
ENSG0000008	-	-	ENSG0000008618	'DIMT1	-	5:62306161-62628582	-	-	IGV_LINK	224.005	6.99472	0	17.1391	OK	436.208	13.0153	0	32.4577	OK	OK	0.895866
ENSG0000008	-	-	ENSG0000008620	'IPO11	-	5:62306161-62628582	-	-	IGV_LINK	335.511	12.2964	0	29.5845	OK	379.269	10.0276	0	28.4069	OK	OK	-0.29425
ENSG0000008	-	-	ENSG0000008620	'FOLH1	-	11:49146634-49208670	-	-	IGV_LINK	314.189	8.82046	0.844697	16.7962	OK	1211.69	37.6524	7.85735	67.4475	OK	OK	2.09382
ENSG0000008	-	-	ENSG0000008623	'EIF2AK1	-	7:6009244-6059230	-	-	IGV_LINK	1678.59	28.2126	4.90988	51.5153	OK	2017.08	34.4786	5.43528	63.522	OK	OK	0.289365
ENSG0000008	-	-	ENSG0000008628	'NME8	-	7:37683795-38025695	-	-	IGV_LINK	51.3952	4.85454	0	18.3606	OK	20.6245	1.51289	0	12.8464	OK	OK	-1.68203
ENSG0000008	-	-	ENSG0000008628	'EPDR1	-	7:37683795-38025695	-	-	IGV_LINK	436.374	12.2957	2.12538	22.4659	OK	1384.94	41.9453	10.484	73.4066	OK	OK	1.77036
ENSG0000008	-	-	ENSG0000008630	'SNX10	-	7:26291894-26376701	-	-	IGV_LINK	1521.49	55.6392	13.1006	98.1779	OK	2043.89	74.5694	22.4145	126.724	OK	OK	0.42248
ENSG0000008	-	-	ENSG0000008647	'SEPHS1	-	10:13317423-13348298	-	-	IGV_LINK	556.62	19.0998	0.459137	37.7404	OK	379.491	14.3874	0	31.3007	OK	OK	-0.40875
ENSG0000008	-	-	ENSG0000008650	'MRPL28	-	16:367383-370527	-	-	IGV_LINK	802.929	48.0081	19.415	76.6012	OK	854.886	51.544	20.7749	82.3131	OK	OK	0.102526
ENSG0000008	-	-	ENSG0000008650	'HBQ1	-	16:180452-181181	-	-	IGV_LINK	0	0	0	0	OK	0	0	0	0	OK	NOTEST	0
ENSG0000008	-	-	ENSG0000008654	'ITPKC	-	19:40691528-40765389	-	-	IGV_LINK	503.285	8.82896	0	20.5388	OK	890.978	15.6357	0	33.2793	OK	OK	0.824532
ENSG0000008	-	-	ENSG0000008654	'CEACAM6	-	19:41708584-41786893	-	-	IGV_LINK	0	0	0	0	OK	0	0	0	0	OK	NOTEST	0
ENSG0000008	-	-	ENSG0000008657	'FAT2	-	5:151504092-151568944	-	-	IGV_LINK	35.8797	0.187695	0	0.524172	OK	61.8735	0.411153	0	0.97013	OK	NOTEST	1.13128
ENSG0000008	-	-	ENSG0000008658	'RBM22	-	5:150690793-150701107	-	-	IGV_LINK	1067.66	32.6877	11.734	53.6413	OK	1033.29	30.9198	11.0287	50.8109	OK	OK	-0.08022
ENSG0000008	-	-	ENSG0000008659	'TMED2	-	12:123575890-123598577	-	-	IGV_LINK	2053.87	73.7051	4.15805	143.252	OK	2855.46	95.3801	7.33994	183.42	OK	OK	0.371923
ENSG0000008	-	-	ENSG0000008661	'ERO1B	-	1:236142504-236281985	-	-	IGV_LINK	314.189	4.1151	0	8.33973	OK	284.618	4.00174	0	9.89775	OK	OK	-0.0403
ENSG0000008	-	-	ENSG0000008666	'ZFAND6	-	15:80059567-80138393	-	-	IGV_LINK	689.471	44.24	13.2683	75.2117	OK	823.949	51.1825	19.2597	83.1053	OK	OK	0.210298
ENSG0000008	-	-	ENSG0000008669	'HSD17B2	-	16:82035003-82139631	-	-	IGV_LINK	15.5155	0.566339	0	1.41265	OK	71.1545	2.89745	0	5.95734	OK	OK	2.35504
ENSG0000008	-	-	ENSG0000008671	'TXLNG	-	X:16786426-16870414	-	-	IGV_LINK	450.92	6.00983	0	14.6001	OK	414.552	5.59126	0	12.8905	OK	OK	-0.10415
ENSG0000008	-	-	ENSG0000008671	'PPEF1	-	X:18675908-18827921	-	-	IGV_LINK	5.81832	0.141432	0	0.670239	OK	24.7494	0.573589	0	1.50771	OK	OK	2.01991
ENSG0000008	-	-	ENSG0000008673	'LAT2	-	7:74199651-74229834	-	-	IGV_LINK	968.751	37.0452	10.1173	63.9732	OK	843.542	30.3462	9.90351	50.7889	OK	OK	-0.28777
ENSG0000008	-	-	ENSG0000008675	'HUWE1	-	X:53532095-53686729	-	-	IGV_LINK	4805.93	48.7112	0	115.027	OK	5875.92	64.7238	0	148.839	OK	OK	0.410041
ENSG0000008	-	-	ENSG0000008682	'ZW10	-	11:113733186-113773811	-	-	IGV_LINK	244.37	4.88444	1.30786	8.46102	OK	327.93	6.69509	2.16337	11.2268	OK	OK	0.454912
ENSG0000008	-	-	ENSG0000008684	'ALG9	-	11:111782194-111885975	-	-	IGV_LINK	470.314	12.3495	0	26.0055	OK	509.02	13.5917	0	27.2722	OK	OK	0.138281
ENSG0000008	-	-	ENSG0000008696	'MYBPC2	-	19:50432902-50466321	-	-	IGV_LINK	256.006	5.06068	0	10.8755	OK	128.903	2.55875	0	6.02701	OK	OK	-0.98389
ENSG0000008	-	-	ENSG0000008699	'NOX4	-	11:89324355-89498187	-	-	IGV_LINK	128.003	3.73831	0.159096	7.31753	OK	254.713	7.14592	1.48449	12.8073	OK	OK	0.934732

ENSG0000008	-	-	ENSG0000008700	'ACOX3	-	4:8366281-8512610	-	-	IGV_LINK	269.582	7.67862	0	16.3171	OK	542.424	16.3963	1.17071	31.6218	OK	OK	1.09445
ENSG0000008	-	-	ENSG0000008705	'MTMR2	-	11:95832881-95925315	-	-	IGV_LINK	615.773	11.9478	2.51742	21.3781	OK	351.648	6.56673	1.63839	11.4951	OK	OK	-0.8635
ENSG0000008	-	-	ENSG0000008707	'PPP1R15A	-	19:48872391-48876057	-	-	IGV_LINK	1198.57	31.8286	13.4366	50.2206	OK	1460.21	39.0459	16.9373	61.1544	OK	OK	0.294846
ENSG0000008	-	-	ENSG0000008707	'HSD17B14	-	19:48813016-48836678	-	-	IGV_LINK	803.898	48.2819	18.9615	77.6024	OK	681.64	41.9496	14.7355	69.1637	OK	OK	-0.20283
ENSG0000008	-	-	ENSG0000008707	'TRIP6	-	7:100867137-100873454	-	-	IGV_LINK	1527.31	67.0348	24.2836	109.786	OK	1928.39	82.962	30.9077	135.016	OK	OK	0.307541
ENSG0000008	-	-	ENSG0000008708	'ACHE	-	7:100889993-100896974	-	-	IGV_LINK	300.613	8.79497	2.37199	15.218	OK	288.743	8.57299	2.21302	14.933	OK	OK	-0.03688
ENSG0000008	-	-	ENSG0000008708	'FTL	-	19:48965300-48966878	-	-	IGV_LINK	52564.7	4199.03	0	8785.95	OK	81731.8	6539.66	0	14971.7	OK	OK	0.639158
ENSG0000008	-	-	ENSG0000008708	'SRRT	-	7:100875110-100888664	-	-	IGV_LINK	1794.95	61.799	10.7072	112.891	OK	1746.9	60.4368	9.49629	111.377	OK	OK	-0.03216
ENSG0000008	-	-	ENSG0000008708	'BAX	-	19:48954814-48961798	-	-	IGV_LINK	907.658	75.848	21.531	130.165	OK	1093.1	86.5242	26.6717	146.377	OK	OK	0.189993
ENSG0000008	-	-	ENSG0000008709	'NLK	-	17:28041736-28196381	-	-	IGV_LINK	512.982	11.0996	2.80085	19.3984	OK	486.738	10.8924	2.47419	19.3105	OK	OK	-0.0272
ENSG0000008	-	-	ENSG0000008711	'PIGS	-	17:28455751-28645454	-	-	IGV_LINK	1058.93	33.3428	0	110.078	OK	1691.21	50.2615	0	161.978	OK	OK	0.59208
ENSG0000008	-	-	ENSG0000008711	'ADAMTS2	-	5:179110850-179345430	-	-	IGV_LINK	50.4255	0.915965	0	3.20861	OK	110.341	1.61947	0	5.70936	OK	OK	0.822154
ENSG0000008	-	-	ENSG0000008712	'TMPRSS11E	-	4:68447448-68497604	-	-	IGV_LINK	0	0	0	0	OK	0	0	0	0	OK	NOTEST	0
ENSG0000008	-	-	ENSG0000008715	'ATXN7L3	-	17:44191804-44223710	-	-	IGV_LINK	986.206	20.5479	0	49.2389	OK	1043.6	21.6321	0	50.4322	OK	OK	0.074183
ENSG0000008	-	-	ENSG0000008715	'PGS1	-	17:78378639-78577394	-	-	IGV_LINK	848.505	28.2639	7.57135	48.9566	OK	1227.16	44.2794	7.53204	81.0267	OK	OK	0.647672
ENSG0000008	-	-	ENSG0000008719	'PSMC5	-	17:63819432-63832026	-	-	IGV_LINK	1702.83	77.3445	19.3165	135.372	OK	1858.27	85.8015	19.2704	152.333	OK	OK	0.149704
ENSG0000008	-	-	ENSG0000008720	'UIMC1	-	5:176905004-177022633	-	-	IGV_LINK	500.376	17.7821	4.39998	31.1641	OK	493.957	17.5441	2.94613	32.1421	OK	OK	-0.01944
ENSG0000008	-	-	ENSG0000008723	'CETP	-	16:56961849-56983845	-	-	IGV_LINK	55.2741	2.08418	0	4.18983	OK	276.368	10.3112	2.368	18.2545	OK	OK	2.30666
ENSG0000008	-	-	ENSG0000008724	'MMP2	-	16:55389699-55506691	-	-	IGV_LINK	434.417	11.9501	0	24.8793	OK	637.283	15.1935	3.22484	27.1621	OK	OK	0.346424
ENSG0000008	-	-	ENSG0000008725	'MT3	-	16:56589073-56591088	-	-	IGV_LINK	32.9705	4.35008	0	9.61247	OK	74.2482	11.2332	0	22.8136	OK	OK	1.36865
ENSG0000008	-	-	ENSG0000008725	'LPCAT2	-	16:55508997-55586670	-	-	IGV_LINK	427.647	12.6542	0	27.9318	OK	295.962	5.41804	0	12.4936	OK	OK	-1.22378
ENSG0000008	-	-	ENSG0000008725	'GNAO1	-	16:56191346-56357457	-	-	IGV_LINK	38.7888	0.478468	0	1.47699	OK	26.8118	0.688709	0	2.86794	OK	OK	0.525472
ENSG0000008	-	-	ENSG0000008726	'OGFOD1	-	16:56429132-56520283	-	-	IGV_LINK	382.07	10.7007	0	30.9041	OK	450.645	12.7991	0	33.731	OK	OK	0.258341
ENSG0000008	-	-	ENSG0000008726	'SH3BP2	-	4:2793022-2841098	-	-	IGV_LINK	4333.68	70.8752	0	143.085	OK	5989.35	103.928	0	213.943	OK	OK	0.552232
ENSG0000008	-	-	ENSG0000008726	'NOP14	-	4:2934898-2963385	-	-	IGV_LINK	580.863	10.7505	2.83023	18.6708	OK	707.42	13.5147	3.14192	23.8874	OK	OK	0.330122
ENSG0000008	-	-	ENSG0000008727	'ADD1	-	4:2843856-2930076	-	-	IGV_LINK	4298.77	88.5989	1.82401	175.374	OK	4697.23	91.432	10.1065	172.757	OK	OK	0.04541
ENSG0000008	-	-	ENSG0000008729	'L2HGDH	-	14:50237562-50416461	-	-	IGV_LINK	188.126	2.48924	0	7.26625	OK	134.059	1.91758	0	4.91525	OK	OK	-0.37642
ENSG0000008	-	-	ENSG0000008730	'TXNDC16	-	14:52430589-52552522	-	-	IGV_LINK	224.005	2.87465	0.752484	4.99681	OK	145.403	1.86643	0.204275	3.52859	OK	OK	-0.6231
ENSG0000008	-	-	ENSG0000008730	'C14orf166	-	14:51989474-52069228	-	-	IGV_LINK	889.234	65.8447	11.859	119.831	OK	849.729	62.1371	13.0524	111.222	OK	OK	-0.08361
ENSG0000008	-	-	ENSG0000008730	'NID2	-	14:51989474-52069228	-	-	IGV_LINK	314.189	7.54645	0	19.7487	OK	300.086	8.94059	0	23.2904	OK	OK	0.244571
ENSG0000008	-	-	ENSG0000008733	'GMCL1	-	2:69829641-69881396	-	-	IGV_LINK	363.645	5.59347	1.16675	10.0202	OK	680.608	11.668	1.10676	22.2293	OK	OK	1.06075
ENSG0000008	-	-	ENSG0000008736	'SF3B2	-	11:66050728-66069619	-	-	IGV_LINK	3408.57	94.287	26.7881	161.786	OK	3322.61	89.5259	24.2329	154.819	OK	OK	-0.07475
ENSG0000008	-	-	ENSG0000008744	'KLHL42	-	12:27779820-27803040	-	-	IGV_LINK	352.009	4.55532	0	9.6366	OK	286.681	3.00115	0	6.06734	OK	OK	-0.60204
ENSG0000008	-	-	ENSG0000008746	'GNAS	-	20:58818918-58911192	-	-	IGV_LINK	9496.47	478.561	91.7656	865.357	OK	8067.27	422.332	93.3081	751.356	OK	OK	-0.18033
ENSG0000008	-	-	ENSG0000008747	'DNM1L	-	12:32679199-32755902	-	-	IGV_LINK	856.263	22.9022	0	47.0309	OK	706.389	19.163	0	39.9335	OK	OK	-0.25717
ENSG0000008	-	-	ENSG0000008749	'PTHLH	-	12:27958083-27972705	-	-	IGV_LINK	21.3339	1.3157	0	3.04098	OK	50.53	3.08901	0	6.35165	OK	OK	1.23132
ENSG0000008	-	-	ENSG0000008749	'PHACTR3	-	20:59577508-59847711	-	-	IGV_LINK	0	0	0	0	OK	6.18735	0.164028	0	0.498863	OK	NOTEST	inf
ENSG0000008	-	-	ENSG0000008750	'ERGIC2	-	12:29149102-29381189	-	-	IGV_LINK	399.525	23.9852	0	50.7687	OK	457.864	29.2022	0.064737	58.3396	OK	OK	0.283932
ENSG0000008	-	-	ENSG0000008751	'TFAP2C	-	20:56629301-56639283	-	-	IGV_LINK	0	0	0	0	OK	1.03122	0.021586	0	0.26298	OK	NOTEST	inf
ENSG0000008	-	-	ENSG0000008758	'AURKA	-	20:56369388-56392337	-	-	IGV_LINK	108.609	3.56716	0	7.263	OK	176.339	5.22608	0.933877	9.51828	OK	OK	0.550952
ENSG0000008	-	-	ENSG0000008758	'CASS4	-	20:56412111-56460387	-	-	IGV_LINK	140.609	3.09862	0.329535	5.8677	OK	275.337	5.98222	1.68137	10.2831	OK	OK	0.949056
ENSG0000008	-	-	ENSG0000008784	'PIR	-	X:15384798-15556529	-	-	IGV_LINK	131.882	6.72302	0.765407	12.6806	OK	252.65	13.473	3.18802	23.7579	OK	OK	1.00288
ENSG0000008	-	-	ENSG0000008788	'AAMDC	-	11:77821108-77994678	-	-	IGV_LINK	276.37	40.3983	0	90.6164	OK	272.243	40.2514	0	81.1144	OK	OK	-0.00526
ENSG0000008	-	-	ENSG0000008790	'RFX2	-	19:5978402-6205121	-	-	IGV_LINK	178.429	3.57158	0	7.56424	OK	115.497	2.79528	0	6.89594	OK	OK	-0.35357
ENSG0000008	-	-	ENSG0000008799	'METTL2A	-	17:62423866-62450822	-	-	IGV_LINK	146.428	5.1047	0.315808	9.89359	OK	200.058	6.47302	0.712251	12.2338	OK	OK	0.342613
ENSG0000008	-	-	ENSG0000008800	'SULT2B1	-	19:48552074-48599425	-	-	IGV_LINK	10.6669	0.715816	0	1.68965	OK	36.0929	1.81396	0	3.70928	OK	OK	1.34148
ENSG0000008	-	-	ENSG0000008803	'ALG6	-	1:63367589-63438562	-	-	IGV_LINK	128.003	3.95316	0.123776	7.78255	OK	154.684	4.75545	0.508735	9.00216	OK	OK	0.266574
ENSG0000008	-	-	ENSG0000008803	'CNOT3	-	19:54137727-54159882	-	-	IGV_LINK	912.507	20.682	6.81984	34.5441	OK	843.542	21.5461	6.12157	36.9707	OK	OK	0.059054
ENSG0000008	-	-	ENSG0000008805	'GP6	-	19:55006192-55071291	-	-	IGV_LINK	6.78804	0.316472	0	2.9462	OK	21.6557	0.864287	0	5.3557	OK	OK	1.44943
ENSG0000008	-	-	ENSG0000008817	'PTPN4	-	2:119759630-119983818	-	-	IGV_LINK	337.463	11.0275	0	25.1284	OK	308.336	9.00902	0	21.5087	OK	OK	-0.29166
ENSG0000008	-	-	ENSG0000008820	'DDX18	-	2:117814649-117832379	-	-	IGV_LINK	1103.54	28.7858	4.26879	53.3028	OK	1259.13	28.873	5.65684	52.0891	OK	OK	0.004363
ENSG0000008	-	-	ENSG0000008824	'KHSRP	-	19:6413347-6424794	-	-	IGV_LINK	1794.95	82.2682	24.3768	140.16	OK	1680.9	77.8035	23.9058	131.701	OK	OK	-0.0805
ENSG0000008	-	-	ENSG0000008825	'GNA11	-	19:3094409-3124004	-	-	IGV_LINK	736.018	13.0919	2.54653	23.6372	OK	887.885	19.8923	0.433684	39.3509	OK	OK	0.60354
ENSG0000008	-	-	ENSG0000008828	'ASAP3	-	1:23428562-23484568	-	-	IGV_LINK	284.128	6.05438	0.370895	11.7379	OK	670.296	12.1572	3.49094	20.8234	OK	OK	1.00575
ENSG0000008	-	-	ENSG0000008829	'EDEM2	-	20:35115356-35147364	-	-	IGV_LINK	541.104	17.6682	6.88155	28.4549	OK	842.511	27.2042	11.4401	42.9683	OK	OK	0.622673
ENSG0000008	-	-	ENSG0000008830	'DNMT3B	-	20:32762384-32809356	-	-	IGV_LINK	96.9721	1.38193	0.130306	2.63356	OK	183.558	2.61266	0.656523	4.56879	OK	OK	0.918831
ENSG0000008	-	-	ENSG0000008832	'REM1	-	20:31465505-31484905	-	-	IGV_LINK	32.0008	1.20834	0	2.47653	OK	11.3435	0.428657	0	0.983408	OK	OK	-1.49513
ENSG0000008	-	-	ENSG0000008832	'TPX2	-	20:31739270-31801805	-	-	IGV_LINK	278.31	4.71781	1.50495	7.93067	OK	311.43	5.27354	1.76204	8.78505	OK	OK	0.160655
ENSG0000008	-	-	ENSG0000008834	'FER1L4	-	20:35558736-35607562	-	-	IGV_LINK	458.678	11.6502	0.342541	22.9578	OK	1288	32.2794	5.44269	59.116	OK	OK	1.47026
ENSG0000008	-	-	ENSG0000008835	'PDRG1	-	20:31847636-31952092	-	-	IGV_LINK	318.068	9.81929	2.4011	17.2375	OK	298.024	9.20629	2.1794	16.2332	OK	OK	-0.093
ENSG0000008	-	-	ENSG0000008836	'EPB41L1	-	20:36091503-36232799	-	-	IGV_LINK	1448.76	31.8094	4.14225	59.4766	OK	860.042	19.8895	2.82805	36.951	OK	OK	-0.67745
ENSG0000008	-	-	ENSG0000008838	'SLC15A1	-	13:98683800-98752654	-	-	IGV_LINK	5.81832	0.130876	0	0.337866	OK	351.648	7.31083	2.40007	12.2216	OK	OK	5.80377
ENSG0000008	-	-	ENSG0000008838	'DOCK9	-	13:98793428-99086625	-	-	IGV_LINK	772.867	17.8321	0	35.8028	OK	992.038	23.3881	0	48.1069	OK	OK	0.391293
ENSG0000008	-	-	ENSG0000008844	'ANKRD10	-	13:110878539-110915069	-	-	IGV_LINK	2259.45	100.231	19.6018	180.86	OK	1852.08	91.4594	0	398.359	OK	OK	-0.13212
ENSG0000008	-	-	ENSG0000008845	'TGDS	-	13:94574050-94596257	-	-	IGV_LINK	210.429	7.08656	1.78993	12.3832	OK	241.307	8.45105	2.18177	14.7203	OK	OK	0.254045
ENSG0000008	-	-	ENSG0000008853	'DOCK3	-	3:50675240-51384198	-	-	IGV_LINK	31.0311	0.20376	0	0.418751	OK	18.562	0.121901	0	0.257764	OK	NOTEST	-0.74116
ENSG0000008	-	-	ENSG0000008854	'C3orf18	-	3:50558024-50596168	-	-	IGV_LINK	122.185	4.29136	0	13.251	OK	91.779	3.04659	0	9.87121	OK	OK	-0.49424
ENSG0000008	-	-	ENSG0000008868	'COQ9	-	16:57428168-57487327	-	-	IGV_LINK	1260.64	59.5225	0	130.089	OK	1424.12	63.4469	0	163.395	OK	OK	0.092116
ENSG0000008	-	-	ENSG0000008872	'TMEM40	-	3:12733524-12769457	-	-	IGV_LINK	1.93944	0.07952	0	0.55272	OK	0	0	0	0	OK	NOTEST	'-inf
ENSG0000008	-	-	ENSG0000008872	'KIF9	-	3:47164496-47346816	-	-	IGV_LINK	113.457	9.19159	0	21.7712	OK	82.498	5.99253	0	16.1075	OK	OK	-0.61715
ENSG0000008	-	-	ENSG0000008875	'ARHGAP28	-	18:6727052-6915716	-	-	IGV_LINK	127.033	1.98048	0	4.25787	OK	71.1545	1.06168	0	2.65546	OK	OK	-0.89949
ENSG0000008	-	-	ENSG0000008876	'CRLS1	-	20:6006089-6040053	-	-	IGV_LINK	572.135	25.8623	5.38198	46.3427	OK	883.76	39.9371	10.0651	69.8091	OK	OK	0.626878
ENSG0000008	-	-	ENSG0000008878	'DEFB127	-	20:157469-159163	-	-	IGV_LINK	0	0	0	0	OK	0	0	0	0	OK	NOTEST	0
ENSG0000008	-	-	ENSG0000008880	'PPP1R13B	-	14:103733751-103847590	-	-	IGV_LINK	378.191	9.92493	0	21.6515	OK	333.086	5.99707	0	12.0329	OK	OK	-0.7268
ENSG0000008	-	-	ENSG0000008881	'ATRN	-	20:3471039-3651122	-	-	IGV_LINK	992.024	8.01101	2.96255	13.0595	OK	1162.19	9.95731	3.87782	16.0368	OK	OK	0.313773
ENSG0000008	-	-	ENSG0000008882	'SMOX	-	20:4120979-4187747	-	-	IGV_LINK	226.915	6.63422	1.13825	12.1302	OK	230.994	6.38447	1.28787	11.4811	OK	OK	-0.05536
ENSG0000008	-	-	ENSG0000008882	'SIGLEC1	-	20:3686969-3707128	-	-	IGV_LINK	1008.51	9.46759	3.85375	15.0814	OK	2818.34	26.073	10.3531	41.793	OK	OK	1.46149
ENSG0000008	-	-	ENSG0000008883	'FKBP1A	-	20:1309908-1393172	-	-	IGV_LINK	2652.03	136.513	42.3659	230.66	OK	2873.9	165.125	54.6365	275.613	OK	OK	0.274519
ENSG0000008	-	-	ENSG0000008883	'NSFL1C	-	20:1442161-1491587	-	-	IGV_LINK	1273.23	49.4594	13.1095	85.8093	OK	1356.44	50.2688	8.33751	92.2	OK	OK	0.023417
ENSG0000008	-	-	ENSG0000008883	'SLC4A11	-	20:3227416-3239190	-	-	IGV_LINK	67.8804	1.32405	0	2.76876	OK	14.4371	0.316283	0	0.846935	OK	OK	-2.06566
ENSG0000008	-	-	ENSG0000008885	'C20orf194	-	20:3249304-3407625	-	-	IGV_LINK	1097.72	9.48365	3.981	14.9863	OK	790.95	6.72731	2.75773	10.6969	OK	OK	-0.49541
ENSG0000008	-	-	ENSG0000008887	'ZNF343	-	20:2461633-2524702	-	-	IGV_LINK	372.561	7.13898	0	18.8119	OK	371.072	6.65719	0	18.0941	OK	OK	-0.10081
ENSG0000008	-	-	ENSG0000008888	'EBF4	-	20:2692877-2760108	-	-	IGV_LINK	228.854	6.4669	1.20347	11.7303	OK	130.966	3.84464	0.244881	7.44441	OK	OK	-0.75023
ENSG0000008	-	-	ENSG0000008888	'CPXM1	-	20:2794068-2800637	-	-	IGV_LINK	32.9705	0.837542	0	1.71311	OK	124.778	3.17136	0.546245	5.79647	OK	OK	1.92087
ENSG0000008	-	-	ENSG0000008888	'MAVS	-	20:3846798-3876123	-	-	IGV_LINK	2383.57	11.643	4.95962	18.3264	OK	3110.17	15.1944	6.58404	23.8048	OK	OK	0.384073
ENSG0000008	-	-	ENSG0000008889	'LZTS3	-	20:3162616-3173592	-	-	IGV_LINK	394.676	5.22433	1.87677	8.5719	OK	171.183	2.34778	0.556186	4.13937	OK	OK	-1.15395
ENSG0000008	-	-	ENSG0000008892	'F11	-	4:186265944-186555567	-	-	IGV_LINK	4.8486	0.157949	0	0.676828	OK	16.4996	0.802108	0	2.34524	OK	OK	2.34434
ENSG0000008	-	-	ENSG0000008893	'XRN2	-	20:21302730-21389827	-	-	IGV_LINK	1485.61	25.6465	11.2802	40.0127	OK	1855.17	32.0376	14.0374	50.0378	OK	OK	0.321006
ENSG0000008	-	-	ENSG0000008897	'KIZ	-	20:21125982-21246622	-	-	IGV_LINK	588.62	14.7162	4.07827	25.3541	OK	608.423	13.8329	4.31441	23.3514	OK	OK	-0.0893
ENSG0000008	-	-	ENSG0000008898	'DYNLL1	-	12:120469849-120498493	-	-	IGV_LINK	1825.98	190.61	74.5934	306.627	OK	2096.48	219.41	85.907	352.913	OK	OK	0.203005
ENSG0000008	-	-	ENSG0000008899	'TESC	-	12:117038922-117099479	-	-	IGV_LINK	171.641	12.1753	1.85931	22.4913	OK	37.1241	2.40566	0	5.10211	OK	OK	-2.33945
ENSG0000008	-	-	ENSG0000008900	'SNX5	-	20:17941596-18059188	-	-	IGV_LINK	2739.46	91.0412	21.6434	160.439	OK	3248.36	114.268	28.6681	199.868	OK	OK	0.327833
ENSG0000008	-	-	ENSG0000008900	'RPL6	-	12:112405189-112509913	-	-	IGV_LINK	13375.4	968.652	189.17	1748.13	OK	11807.5	856.216	164.075	1548.36	OK	OK	-0.17801
ENSG0000008	-	-	ENSG0000008901	'SIRPG	-	20:1629151-1657779	-	-	IGV_LINK	229.824	9.775	1.75297	17.797	OK	98.9976	4.25796	0	9.1675	OK	OK	-1.19893
ENSG0000008	-	-	ENSG0000008902	'MAPKAPK5	-	12:111839763-111902238	-	-	IGV_LINK	769.561	25.6359	0.684604	50.5872	OK	673.119	22.7868	1.12444	44.4492	OK	OK	-0.16997
ENSG0000008	-	-	ENSG0000008904	'P2RX7	-	12:121132818-121188032	-	-	IGV_LINK	1021.12	14.2593	4.41296	24.1056	OK	469.207	6.51856	1.92597	11.1111	OK	OK	-1.12928
ENSG0000008	-	-	ENSG0000008904	'ESF1	-	20:13714321-13784886	-	-	IGV_LINK	326.796	8.1528	2.04709	14.2585	OK	416.615	9.5268	1.99293	17.0607	OK	OK	0.224696
ENSG0000008	-	-	ENSG0000008905	'RBBP9	-	20:18486539-18497243	-	-	IGV_LINK	523.649	8.41001	2.79024	14.0298	OK	822.918	13.0066	4.87698	21.1362	OK	OK	0.629061
ENSG0000008	-	-	ENSG0000008905	'ANAPC5	-	12:121308244-121399896	-	-	IGV_LINK	2557.15	61.5309	17.4283	105.633	OK	2552.28	66.1929	18.6628	113.723	OK	OK	0.105366
ENSG0000008	-	-	ENSG0000008905	'SLC23A2	-	20:4852355-5010293	-	-	IGV_LINK	1210.21	10.5058	3.44864	17.563	OK	1080.72	9.2135	3.41947	15.0075	OK	OK	-0.18937
ENSG0000008	-	-	ENSG0000008906	'SLC8B1	-	12:113298758-113391625	-	-	IGV_LINK	947.417	21.4617	1.49067	41.4327	OK	1243.66	27.2829	1.08042	53.4854	OK	OK	0.346234
ENSG0000008	-	-	ENSG0000008906	'TMEM230	-	20:5068231-5113103	-	-	IGV_LINK	1435.19	60.4922	26.0643	94.9201	OK	1836.61	77.0318	31.5649	122.499	OK	OK	0.348704
ENSG0000008	-	-	ENSG0000008909	'DZANK1	-	20:18383366-18484643	-	-	IGV_LINK	180.368	4.40791	0.012	8.80382	OK	182.527	4.1671	0	8.59404	OK	OK	-0.08105
ENSG0000008	-	-	ENSG0000008909	'KDM2B	-	12:121400040-121593504	-	-	IGV_LINK	770.928	12.5002	0	25.3403	OK	799.199	13.1515	0.572686	25.7303	OK	OK	0.073273
ENSG0000008	-	-	ENSG0000008910	'CFAP61	-	20:20034367-20360702	-	-	IGV_LINK	13.5761	1.06526	0	6.4935	OK	16.4996	1.09456	0	6.56442	OK	OK	0.039149
ENSG0000008	-	-	ENSG0000008911	'LHX5	-	12:113462033-113480624	-	-	IGV_LINK	0	0	0	0	OK	0	0	0	0	OK	NOTEST	0
ENSG0000008	-	-	ENSG0000008912	'TASP1	-	20:13389391-13638940	-	-	IGV_LINK	179.398	5.36276	0.764652	9.96086	OK	203.151	5.65351	1.23295	10.0741	OK	OK	0.076171
ENSG0000008	-	-	ENSG0000008912	'OAS1	-	12:112906776-113017751	-	-	IGV_LINK	1715.44	75.3369	0	165.286	OK	2435.75	105.119	0	237.456	OK	OK	0.480592
ENSG0000008	-	-	ENSG0000008915	'GCN1	-	12:120127202-120194710	-	-	IGV_LINK	2053.87	17.9631	0	43.2677	OK	2439.88	19.3028	0	44.76	OK	OK	0.10377
ENSG0000008	-	-	ENSG0000008915	'RPLP0	-	12:120196685-120201235	-	-	IGV_LINK	26764.3	1493.36	235.283	2751.44	OK	25113.4	1398.26	187.419	2609.11	OK	OK	-0.09493
ENSG0000008	-	-	ENSG0000008915	'PXN	-	12:120201290-120265771	-	-	IGV_LINK	2519.33	42.0206	8.83014	75.2111	OK	3020.46	49.6608	12.112	87.2095	OK	OK	0.241009
ENSG0000008	-	-	ENSG0000008916	'SIRT4	-	12:120302315-120313341	-	-	IGV_LINK	32.9705	2.21947	0	4.93626	OK	23.7182	1.30974	0	2.7728	OK	OK	-0.76094
ENSG0000008	-	-	ENSG0000008916	'RPH3A	-	12:112570379-112898881	-	-	IGV_LINK	7.75776	0.393616	0	1.53757	OK	3.09367	0.078732	0	0.99985	OK	NOTEST	-2.32176
ENSG0000008	-	-	ENSG0000008917	'KIF16B	-	20:16272103-16573434	-	-	IGV_LINK	476.133	6.79057	0.845882	12.7353	OK	698.139	11.3329	1.40535	21.2604	OK	OK	0.738908
ENSG0000008	-	-	ENSG0000008919	'TRMT6	-	20:5937234-5950558	-	-	IGV_LINK	148.367	3.63336	0.729472	6.53725	OK	213.464	5.56662	1.35255	9.7807	OK	OK	0.615497
ENSG0000008	-	-	ENSG0000008919	'CHGB	-	20:5911429-5925361	-	-	IGV_LINK	5.81832	0.144352	0	0.392768	OK	7.21857	0.185654	0	0.469509	OK	NOTEST	0.363024
ENSG0000008	-	-	ENSG0000008922	'PEBP1	-	12:118063592-118145584	-	-	IGV_LINK	7175.93	278.606	87.7505	469.461	OK	5636.68	226.053	67.1338	384.973	OK	OK	-0.30156
ENSG0000008	-	-	ENSG0000008922	'TBX5	-	12:114353930-114412831	-	-	IGV_LINK	0	0	0	0	OK	0	0	0	0	OK	NOTEST	0
ENSG0000008	-	-	ENSG0000008923	'BRAP	-	12:111642145-111685986	-	-	IGV_LINK	385.949	7.42319	1.85996	12.9864	OK	407.334	8.0322	2.46489	13.5995	OK	OK	0.113756
ENSG0000008	-	-	ENSG0000008924	'ERP29	-	12:112013315-112023451	-	-	IGV_LINK	2101.38	83.4164	34.5567	132.276	OK	2260.45	86.5642	33.4308	139.698	OK	OK	0.053438
ENSG0000008	-	-	ENSG0000008925	'NOS1	-	12:117208141-117452170	-	-	IGV_LINK	0.969721	0.004555	0	0.330056	OK	1.03122	0.004844	0	0.331206	OK	NOTEST	0.088915
ENSG0000008	-	-	ENSG0000008928	'FUS	-	16:31180109-31194871	-	-	IGV_LINK	7652.06	197.857	8.73943	386.974	OK	6110.01	162.003	19.4904	304.516	OK	OK	-0.28844
ENSG0000008	-	-	ENSG0000008928	'IGBP1	-	X:70133448-70166324	-	-	IGV_LINK	1729.98	65.2433	28.6888	101.798	OK	1530.34	57.753	25.4343	90.0717	OK	OK	-0.17593
ENSG0000008	-	-	ENSG0000008932	'FXYD5	-	19:35154729-35169883	-	-	IGV_LINK	1122.94	89.0377	37.9051	140.17	OK	1286.97	102.39	44.3063	160.474	OK	OK	0.201589
ENSG0000008	-	-	ENSG0000008933	'ZNF302	-	19:34677638-34686397	-	-	IGV_LINK	995.903	25.7687	4.5308	47.0067	OK	962.133	25.1779	5.96015	44.3956	OK	OK	-0.03347
ENSG0000008	-	-	ENSG0000008935	'GRAMD1A	-	19:34994783-35026471	-	-	IGV_LINK	1470.1	52.1839	12.8504	91.5173	OK	1496.31	55.9808	13.6398	98.3218	OK	OK	0.101328
ENSG0000008	-	-	ENSG0000008935	'FXYD3	-	19:35115878-35124324	-	-	IGV_LINK	0.969721	0.047583	0	0.631795	OK	42.2802	5.24982	0	11.0822	OK	OK	6.78567
ENSG0000008	-	-	ENSG0000008947	'HEPH	-	X:66162548-66268867	-	-	IGV_LINK	129.943	2.11345	0	4.35089	OK	209.339	3.68932	0.069769	7.30887	OK	OK	0.803757
ENSG0000008	-	-	ENSG0000008948	'CDIP1	-	16:4510674-4538828	-	-	IGV_LINK	461.587	12.7054	3.54637	21.8644	OK	881.697	24.5352	9.44655	39.6239	OK	OK	0.949413
ENSG0000008	-	-	ENSG0000008950	'CMTM1	-	16:66552562-66579137	-	-	IGV_LINK	28.1219	2.5603	0	9.0708	OK	29.9055	2.45473	0	8.09298	OK	OK	-0.06075
ENSG0000008	-	-	ENSG0000008955	'KCNH4	-	17:42156890-42181278	-	-	IGV_LINK	21.3339	0.320828	0	0.674195	OK	25.7806	0.409338	0	0.850673	OK	NOTEST	0.351493
ENSG0000008	-	-	ENSG0000008959	'GANAB	-	11:62624825-62646726	-	-	IGV_LINK	7012.05	130.154	23.8631	236.444	OK	10116.3	181.011	30.0972	331.925	OK	OK	0.47586

ENSG0000008	-	-	ENSG0000008963	'GMIP	-	19:19629475-19643667	-	-	IGV_LINK	963.902	20.2308	5.84094	34.6206	OK	1418.97	30.1838	8.28486	52.0827	OK	OK	0.577224
ENSG0000008	-	-	ENSG0000008968	'RBM41	-	X:107064419-107118823	-	-	IGV_LINK	288.007	9.97746	1.86757	18.0873	OK	233.057	6.91547	1.22753	12.6034	OK	OK	-0.52884
ENSG0000008	-	-	ENSG0000008968	'BIRC5	-	17:78214185-78225636	-	-	IGV_LINK	123.155	3.60653	0	7.60471	OK	158.809	4.2407	0.213983	8.26742	OK	OK	0.233694
ENSG0000008	-	-	ENSG0000008969	'LAG3	-	12:6772511-6778455	-	-	IGV_LINK	313.22	10.6453	2.63378	18.6569	OK	193.87	6.15264	0.995474	11.3098	OK	OK	-0.79095
ENSG0000008	-	-	ENSG0000008969	'MLF2	-	12:6747995-6770952	-	-	IGV_LINK	2212.9	98.9441	18.8834	179.005	OK	2609	119.041	24.2136	213.869	OK	OK	0.266776
ENSG0000008	-	-	ENSG0000008972	'OTUB2	-	14:94026328-94081245	-	-	IGV_LINK	27.1522	0.408103	0	2.44271	OK	23.7182	0.449858	0	3.55602	OK	NOTEST	0.140538
ENSG0000008	-	-	ENSG0000008973	'DDX24	-	14:94026328-94081245	-	-	IGV_LINK	2120.78	59.9337	14.3892	105.478	OK	1854.14	53.2661	12.2119	94.3203	OK	OK	-0.17015
ENSG0000008	-	-	ENSG0000008977	'ZBTB25	-	14:64388030-64546173	-	-	IGV_LINK	395.646	6.46312	0	20.5734	OK	356.804	4.78239	0	15.8579	OK	OK	-0.4345
ENSG0000008	-	-	ENSG0000008981	'NECAP1	-	12:8076938-8221115	-	-	IGV_LINK	824.043	21.8572	2.13716	41.5773	OK	926.089	31.107	3.77259	58.4414	OK	OK	0.50913
ENSG0000008	-	-	ENSG0000008982	'ARHGAP4	-	X:153861513-153935223	-	-	IGV_LINK	2533.28	83.921	10.2329	157.609	OK	2179.09	61.2515	7.53515	114.968	OK	OK	-0.45429
ENSG0000008	-	-	ENSG0000008984	'ANKRD24	-	19:4183353-4224814	-	-	IGV_LINK	177.459	3.61677	0.225111	7.00843	OK	126.841	2.21431	0.10725	4.32137	OK	OK	-0.70785
ENSG0000008	-	-	ENSG0000008987	'DHX32	-	10:125823545-125896436	-	-	IGV_LINK	456.738	9.86444	0	20.3253	OK	359.898	7.45554	0	15.0288	OK	OK	-0.40393
ENSG0000008	-	-	ENSG0000008990	'RCOR1	-	14:102592660-102730576	-	-	IGV_LINK	464.496	5.98057	0	14.4593	OK	445.489	5.01187	0	10.7643	OK	OK	-0.25493
ENSG0000008	-	-	ENSG0000008991	'GPATCH2L	-	14:76131706-76263474	-	-	IGV_LINK	680.744	7.28188	0	18.3875	OK	588.829	7.44713	0	16.8345	OK	OK	0.032375
ENSG0000009	-	-	ENSG0000009000	'LTBP4	-	19:40592882-40629818	-	-	IGV_LINK	521.71	18.1699	0	39.2853	OK	670.296	18.2296	1.33769	35.1215	OK	OK	0.004737
ENSG0000009	-	-	ENSG0000009001	'BLVRB	-	19:40447788-40465840	-	-	IGV_LINK	1072.51	86.3602	37.5959	135.125	OK	1770.61	142.981	63.0756	222.887	OK	OK	0.72739
ENSG0000009	-	-	ENSG0000009002	'SLC9A1	-	1:27098814-27166981	-	-	IGV_LINK	445.102	6.7155	1.23685	12.1942	OK	563.049	7.44835	2.90847	11.9882	OK	OK	0.149427
ENSG0000009	-	-	ENSG0000009005	'SPTLC1	-	9:92031998-92115384	-	-	IGV_LINK	961.963	34.2284	13.05	55.4067	OK	1113.72	39.4212	16.2114	62.631	OK	OK	0.203779
ENSG0000009	-	-	ENSG0000009006	'PAPOLA	-	14:96500809-96567111	-	-	IGV_LINK	1639.8	30.7798	6.42467	55.1349	OK	1484.96	28.3665	5.91246	50.8205	OK	OK	-0.1178
ENSG0000009	-	-	ENSG0000009006	'CCNK	-	14:99481168-99604026	-	-	IGV_LINK	185.217	9.17	0	21.6515	OK	155.715	7.93327	0	18.9469	OK	OK	-0.20901
ENSG0000009	-	-	ENSG0000009009	'PCBP4	-	3:51955313-51989202	-	-	IGV_LINK	199.762	6.70636	0	21.2126	OK	173.246	6.70895	0	27.9882	OK	OK	0.000557
ENSG0000009	-	-	ENSG0000009010	'RGS1	-	1:192575726-192580031	-	-	IGV_LINK	2936.31	131.514	40.4437	222.584	OK	1872.7	84.3007	30.6487	137.953	OK	OK	-0.6416
ENSG0000009	-	-	ENSG0000009023	'YPEL3	-	16:30092313-30104116	-	-	IGV_LINK	1914.23	110.121	35.5147	184.727	OK	1815.99	111.067	32.4813	189.653	OK	OK	0.012343
ENSG0000009	-	-	ENSG0000009026	'MRPS33	-	7:141002609-141015228	-	-	IGV_LINK	572.135	54.0926	17.8791	90.306	OK	680.608	74.1857	25.3456	123.026	OK	OK	0.455711
ENSG0000009	-	-	ENSG0000009026	'NDUFB2	-	7:140518419-140924764	-	-	IGV_LINK	1013.36	168.264	6.22982	330.298	OK	1232.31	205.551	30.1121	380.99	OK	OK	0.28877
ENSG0000009	-	-	ENSG0000009027	'NUDC	-	1:26890487-26946862	-	-	IGV_LINK	1366.34	48.4342	18.1706	78.6978	OK	1241.59	44.7646	16.1691	73.36	OK	OK	-0.11367
ENSG0000009	-	-	ENSG0000009031	'MAEA	-	4:1289850-1340147	-	-	IGV_LINK	1083.18	30.0813	9.53672	50.626	OK	1285.94	35.2231	12.7564	57.6899	OK	OK	0.227654
ENSG0000009	-	-	ENSG0000009033	'ICAM1	-	19:10251900-10289019	-	-	IGV_LINK	2300.18	68.0927	0	138.951	OK	3697.97	103.433	0	207.095	OK	OK	0.603129
ENSG0000009	-	-	ENSG0000009037	'STRN4	-	19:46719506-46789043	-	-	IGV_LINK	1162.69	29.1095	0	59.5039	OK	1242.63	35.659	0	76.1888	OK	OK	0.292775
ENSG0000009	-	-	ENSG0000009037	'IRAK3	-	12:66188878-66257434	-	-	IGV_LINK	1062.81	7.21909	2.91833	11.5198	OK	944.602	6.53296	2.48413	10.5818	OK	OK	-0.14408
ENSG0000009	-	-	ENSG0000009038	'LYZ	-	12:69348340-69354234	-	-	IGV_LINK	3097.29	219.637	60.2876	378.987	OK	3868.12	280.648	81.8886	479.408	OK	OK	0.35364
ENSG0000009	-	-	ENSG0000009040	'SI	-	3:164978897-165078495	-	-	IGV_LINK	0	0	0	0	OK	0	0	0	0	OK	NOTEST	0
ENSG0000009	-	-	ENSG0000009043	'MUL1	-	1:20499447-20508161	-	-	IGV_LINK	647.773	16.1138	6.55392	25.6738	OK	895.103	22.2777	9.40579	35.1496	OK	OK	0.467298
ENSG0000009	-	-	ENSG0000009044	'TFAP4	-	16:4257185-4273075	-	-	IGV_LINK	254.067	6.55775	0.850806	12.2647	OK	152.621	4.62546	0	10.0531	OK	OK	-0.5036
ENSG0000009	-	-	ENSG0000009047	'PDCD7	-	15:65117378-65133836	-	-	IGV_LINK	403.404	8.98937	2.39577	15.583	OK	430.021	9.3192	2.91104	15.7274	OK	OK	0.051986
ENSG0000009	-	-	ENSG0000009048	'SPG21	-	15:64963020-64990310	-	-	IGV_LINK	1452.64	54.3728	20.2203	88.5252	OK	1273.56	47.9998	19.1869	76.8128	OK	OK	-0.17986
ENSG0000009	-	-	ENSG0000009051	'FETUB	-	3:186538440-186773476	-	-	IGV_LINK	0	0	0	0	OK	1.03122	0.06706	0	1.3839	OK	NOTEST	inf
ENSG0000009	-	-	ENSG0000009052	'DNAJB11	-	3:186538440-186773476	-	-	IGV_LINK	723.29	25.0169	6.85814	43.1757	OK	785.522	27.849	8.60611	47.0918	OK	OK	0.154718
ENSG0000009	-	-	ENSG0000009053	'P3H2	-	3:189956727-190144846	-	-	IGV_LINK	3583.12	70.7142	21.8867	119.542	OK	7019.55	141.513	34.6612	248.364	OK	OK	1.00086
ENSG0000009	-	-	ENSG0000009053	'THPO	-	3:184371934-184378144	-	-	IGV_LINK	3.87888	0.227828	0	0.810514	OK	74.2482	3.43225	0	7.68515	OK	OK	3.91314
ENSG0000009	-	-	ENSG0000009053	'CHRD	-	3:184380072-184390736	-	-	IGV_LINK	191.035	4.50906	0	9.91433	OK	345.46	7.97678	0.048922	15.9046	OK	OK	0.822978
ENSG0000009	-	-	ENSG0000009055	'FLT3LG	-	19:49446297-49492308	-	-	IGV_LINK	201.223	14.6838	0	186.213	OK	145.825	10.1755	0	112.247	OK	OK	-0.52913
ENSG0000009	-	-	ENSG0000009056	'RAB11FIP3	-	16:425618-523011	-	-	IGV_LINK	1537.98	36.8533	2.20944	71.4972	OK	3369.01	87.6026	11.5221	163.683	OK	OK	1.24918
ENSG0000009	-	-	ENSG0000009058	'GNPTG	-	16:1351922-1414751	-	-	IGV_LINK	1577.74	80.726	11.5039	149.948	OK	1674.71	80.7292	16.5435	144.915	OK	OK	5.80E-05
ENSG0000009	-	-	ENSG0000009061	'ZNF268	-	12:133130574-133214831	-	-	IGV_LINK	400.495	5.60753	0	12.8917	OK	530.05	6.5991	0	13.4915	OK	OK	0.234905
ENSG0000009	-	-	ENSG0000009061	'GOLGA3	-	12:132768908-132956306	-	-	IGV_LINK	1275.18	9.91602	0	32.1945	OK	1654.08	11.5805	0.224186	22.9369	OK	OK	0.22387
ENSG0000009	-	-	ENSG0000009062	'PABPC4	-	1:39560815-39576790	-	-	IGV_LINK	2663.82	83.6871	4.17952	163.195	OK	2459.47	75.9702	15.807	136.133	OK	OK	-0.13957
ENSG0000009	-	-	ENSG0000009065	'CD209	-	19:7739993-7747564	-	-	IGV_LINK	66.9107	1.23609	0	3.1087	OK	468.176	7.81601	1.39618	14.2358	OK	OK	2.66064
ENSG0000009	-	-	ENSG0000009066	'CERS4	-	19:8206735-8262421	-	-	IGV_LINK	697.229	31.8387	8.80756	54.8699	OK	835.292	38.4394	11.6783	65.2005	OK	OK	0.271805
ENSG0000009	-	-	ENSG0000009067	'MCOLN1	-	19:7522625-7561764	-	-	IGV_LINK	439.283	16.9273	0	38.5132	OK	673.39	24.1573	0	52.998	OK	OK	0.51311
ENSG0000009	-	-	ENSG0000009068	'USP48	-	1:21678297-21783606	-	-	IGV_LINK	1227.67	23.9272	5.3497	42.5046	OK	1064.22	21.8388	4.20897	39.4686	OK	OK	-0.13176
ENSG0000009	-	-	ENSG0000009077	'EFNB1	-	X:68828996-68842147	-	-	IGV_LINK	583.772	10.4724	4.17299	16.7717	OK	711.545	12.7692	5.21815	20.3202	OK	OK	0.286079
ENSG0000009	-	-	ENSG0000009085	'PDPR	-	16:70113625-70187361	-	-	IGV_LINK	1751.32	19.0778	0.192062	37.9635	OK	1556.12	16.5775	1.36704	31.7879	OK	OK	-0.20267
ENSG0000009	-	-	ENSG0000009086	'AARS	-	16:70252294-70289543	-	-	IGV_LINK	2220.66	42.5968	14.3013	70.8923	OK	1818.05	34.0303	11.9537	56.1068	OK	OK	-0.32393
ENSG0000009	-	-	ENSG0000009086	'GLG1	-	16:74447426-74607144	-	-	IGV_LINK	3950.64	45.8081	11.1367	80.4794	OK	4367.24	52.9594	14.9391	90.9796	OK	OK	0.209284
ENSG0000009	-	-	ENSG0000009088	'KIF4A	-	X:70286594-70420832	-	-	IGV_LINK	105.7	1.53759	0	4.23252	OK	173.246	2.3389	0	5.66114	OK	OK	0.605163
ENSG0000009	-	-	ENSG0000009090	'TNRC6A	-	16:24610208-24827632	-	-	IGV_LINK	2461.15	31.6816	0	65.5072	OK	2046.98	25.4744	1.80523	49.1436	OK	OK	-0.3146
ENSG0000009	-	-	ENSG0000009092	'PLEKHG2	-	19:39412584-39428415	-	-	IGV_LINK	1262.58	25.8951	0	71.7723	OK	1228.19	22.3928	0	45.4511	OK	OK	-0.20964
ENSG0000009	-	-	ENSG0000009093	'DLL3	-	19:39498894-39508481	-	-	IGV_LINK	0.969721	0.029028	0	0.409717	OK	0	0	0	0	OK	NOTEST	'-inf
ENSG0000009	-	-	ENSG0000009097	'NAT14	-	19:55485003-55487568	-	-	IGV_LINK	230.793	14.0953	2.14392	26.0468	OK	325.867	17.8411	4.11649	31.5656	OK	OK	0.339983
ENSG0000009	-	-	ENSG0000009097	'PITPNM2	-	12:122983479-123150015	-	-	IGV_LINK	682.683	6.90004	0.397721	13.4024	OK	1058.04	12.0441	2.09341	21.9947	OK	OK	0.803648
ENSG0000009	-	-	ENSG0000009098	'EXOC1	-	4:55853615-55905034	-	-	IGV_LINK	841.717	20.623	2.51581	38.7302	OK	936.352	22.1605	3.15824	41.1628	OK	OK	0.103736
ENSG0000009	-	-	ENSG0000009100	'RBM27	-	5:146203549-146376963	-	-	IGV_LINK	394.627	3.54217	0.999185	6.08515	OK	390.857	3.50897	1.0665	5.95144	OK	OK	-0.01358
ENSG0000009	-	-	ENSG0000009101	'POU4F3	-	5:146203549-146376963	-	-	IGV_LINK	0	0	0	0	OK	2.06245	0.115271	0	1.20477	OK	NOTEST	inf
ENSG0000009	-	-	ENSG0000009103	'OSBPL8	-	12:76351796-76559809	-	-	IGV_LINK	773.837	11.3452	0	24.4602	OK	533.143	6.51335	1.08496	11.9417	OK	OK	-0.80061
ENSG0000009	-	-	ENSG0000009107	'DTX2	-	7:76461675-76505995	-	-	IGV_LINK	308.371	14.0393	2.14362	25.9349	OK	471.27	19.0136	4.49596	33.5312	OK	OK	0.437562
ENSG0000009	-	-	ENSG0000009110	'NLRC4	-	2:32224452-32265854	-	-	IGV_LINK	173.58	3.07376	0.732789	5.41473	OK	188.714	3.34193	0.505654	6.17821	OK	OK	0.120678
ENSG0000009	-	-	ENSG0000009112	'PUS7	-	7:105439660-105522267	-	-	IGV_LINK	158.064	4.12902	0	11.1822	OK	170.152	3.50196	0.363201	6.64071	OK	OK	-0.23764
ENSG0000009	-	-	ENSG0000009112	'LAMB4	-	7:108023547-108130356	-	-	IGV_LINK	7.75776	0.264721	0	0.794773	OK	19.5933	0.638567	0	1.86999	OK	OK	1.27037
ENSG0000009	-	-	ENSG0000009112	'NRCAM	-	7:108147622-108456717	-	-	IGV_LINK	929.962	11.6736	0.014451	23.3328	OK	1731.43	22.0223	0.384359	43.6603	OK	OK	0.915718
ENSG0000009	-	-	ENSG0000009113	'LAMB1	-	7:107890969-108003255	-	-	IGV_LINK	2221.63	29.3423	0	60.5739	OK	2806.99	39.2295	0	85.8463	OK	OK	0.418959
ENSG0000009	-	-	ENSG0000009113	'SLC26A4	-	7:107653967-107717809	-	-	IGV_LINK	12.6064	0.149349	0	0.697878	OK	19.5933	0.787096	0	2.25896	OK	OK	2.39785
ENSG0000009	-	-	ENSG0000009113	'SLC26A3	-	7:107765466-107803225	-	-	IGV_LINK	0	0	0	0	OK	0	0	0	0	OK	NOTEST	0
ENSG0000009	-	-	ENSG0000009114	'DLD	-	7:107890969-108003255	-	-	IGV_LINK	953.235	39.0941	0	100.231	OK	1195.19	43.8501	0	107.551	OK	OK	0.165628
ENSG0000009	-	-	ENSG0000009115	'WDR7	-	18:56597207-57038845	-	-	IGV_LINK	480.012	6.18085	0	18.4823	OK	443.427	5.19229	0	15.6453	OK	OK	-0.25143
ENSG0000009	-	-	ENSG0000009116	'TXNL1	-	18:56597207-57038845	-	-	IGV_LINK	781.595	27.911	4.67789	51.1441	OK	909.54	34.9753	9.20289	60.7478	OK	OK	0.325505
ENSG0000009	-	-	ENSG0000009118	'IL5RA	-	3:2098812-3126613	-	-	IGV_LINK	0.969721	0.010218	0	0.347805	OK	1.03122	0.0946	0	0.651701	OK	NOTEST	3.21078
ENSG0000009	-	-	ENSG0000009126	'ABCC6	-	16:16148927-16224261	-	-	IGV_LINK	592.499	11.0547	0.941011	21.1683	OK	1482.9	26.7826	4.69888	48.8663	OK	OK	1.27664
ENSG0000009	-	-	ENSG0000009131	'CMTM6	-	3:32391670-32503408	-	-	IGV_LINK	984.266	15.0302	5.47265	24.5878	OK	1392.15	21.5491	7.40008	35.6982	OK	OK	0.519763
ENSG0000009	-	-	ENSG0000009140	'ITGA6	-	2:172427353-172608669	-	-	IGV_LINK	1563.19	18.3746	0	40.405	OK	1496.31	18.2667	0	40.0164	OK	OK	-0.0085
ENSG0000009	-	-	ENSG0000009142	'RAPGEF4	-	2:172677140-173052893	-	-	IGV_LINK	328.735	6.2863	0.104244	12.4684	OK	229.963	4.64736	0	9.87258	OK	OK	-0.4358
ENSG0000009	-	-	ENSG0000009143	'MAP3K20	-	2:173075434-173282036	-	-	IGV_LINK	539.165	6.07576	1.61694	10.5346	OK	727.014	9.18832	1.94431	16.4323	OK	OK	0.596735
ENSG0000009	-	-	ENSG0000009148	'SMPX	-	X:21705971-21758163	-	-	IGV_LINK	0	0	0	0	OK	0	0	0	0	OK	NOTEST	0
ENSG0000009	-	-	ENSG0000009148	'FH	-	1:241497602-241519761	-	-	IGV_LINK	739.897	25.6681	10.5455	40.7907	OK	827.042	29.6177	10.7248	48.5105	OK	OK	0.206481
ENSG0000009	-	-	ENSG0000009149	'SEL1L3	-	4:25747426-25929874	-	-	IGV_LINK	3267.96	48.6662	13.4685	83.8639	OK	4603.39	74.6179	15.9258	133.31	OK	OK	0.616603
ENSG0000009	-	-	ENSG0000009151	'TF	-	3:133661997-133779006	-	-	IGV_LINK	89.2143	3.33703	0	7.86591	OK	27.8431	1.15158	0	3.25795	OK	OK	-1.53495
ENSG0000009	-	-	ENSG0000009152	'CDV3	-	3:133573729-133590261	-	-	IGV_LINK	2256.54	51.4933	12.5079	90.4788	OK	2929.71	67.4078	14.83	119.986	OK	OK	0.38853
ENSG0000009	-	-	ENSG0000009153	'MYO15A	-	17:18087885-18209954	-	-	IGV_LINK	59.153	2.00575	0	11.4253	OK	71.1545	2.63579	0	15.8226	OK	OK	0.394094
ENSG0000009	-	-	ENSG0000009154	'ALKBH5	-	17:18087885-18209954	-	-	IGV_LINK	1435.19	32.3382	8.49086	56.1856	OK	1905.7	42.5349	13.697	71.3728	OK	OK	0.395407
ENSG0000009	-	-	ENSG0000009158	'APOH	-	17:66212032-66256525	-	-	IGV_LINK	0.969721	0.102248	0	0.554615	OK	9.28102	0.521825	0	1.30722	OK	NOTEST	2.35149
ENSG0000009	-	-	ENSG0000009159	'NLRP1	-	17:5499426-5619424	-	-	IGV_LINK	1881.26	25.5823	7.39471	43.7698	OK	1500.43	19.5763	6.75363	32.399	OK	OK	-0.38603
ENSG0000009	-	-	ENSG0000009162	'PITPNM3	-	17:6444414-6556494	-	-	IGV_LINK	83.396	0.711763	0	1.46121	OK	78.3731	0.781217	0	2.11639	OK	OK	0.134326
ENSG0000009	-	-	ENSG0000009164	'SPAG7	-	17:4959225-4967872	-	-	IGV_LINK	610.924	40.1391	14.2345	66.0436	OK	543.456	36.7428	13.3037	60.1819	OK	OK	-0.12755
ENSG0000009	-	-	ENSG0000009165	'ORC6	-	16:46689642-46698394	-	-	IGV_LINK	91.1537	3.4409	0.092583	6.78922	OK	88.6853	3.46802	0.172055	6.76398	OK	OK	0.011325
ENSG0000009	-	-	ENSG0000009165	'ZFHX4	-	8:76403997-76867285	-	-	IGV_LINK	37.8191	0.362291	0	1.63549	OK	112.404	0.457522	0	1.31006	OK	NOTEST	0.336694
ENSG0000009	-	-	ENSG0000009166	'SLC17A6	-	11:22283729-22379503	-	-	IGV_LINK	0	0	0	0	OK	0	0	0	0	OK	NOTEST	0
ENSG0000009	-	-	ENSG0000009170	'CPA1	-	7:130380338-130388114	-	-	IGV_LINK	0	0	0	0	OK	1.03122	0.051457	0	0.488359	OK	NOTEST	inf
ENSG0000009	-	-	ENSG0000009173	'ZC3HC1	-	7:129953233-130051451	-	-	IGV_LINK	437.344	15.1784	4.70839	25.6485	OK	698.139	22.7908	9.15141	36.4302	OK	OK	0.586426
ENSG0000009	-	-	ENSG0000009183	'ESR1	-	6:151656690-152637801	-	-	IGV_LINK	125.094	1.25555	0	5.60732	OK	86.6229	0.85298	0	3.37134	OK	OK	-0.55774
ENSG0000009	-	-	ENSG0000009184	'RGS17	-	6:153004458-153131249	-	-	IGV_LINK	39.7585	0.294798	0	0.606477	OK	22.6869	0.25462	0	0.634702	OK	NOTEST	-0.21138
ENSG0000009	-	-	ENSG0000009187	'ANGPT2	-	8:6403550-6708209	-	-	IGV_LINK	832.99	16.4981	3.69696	29.2993	OK	449.614	8.64992	0	17.3707	OK	OK	-0.93155
ENSG0000009	-	-	ENSG0000009194	'TMEM101	-	17:44011187-44023946	-	-	IGV_LINK	545.953	23.434	8.36257	38.5055	OK	564.08	24.0175	8.78993	39.2451	OK	OK	0.03548
ENSG0000009	-	-	ENSG0000009197	'CD200	-	3:112302477-112362812	-	-	IGV_LINK	818.444	28.9098	11.1322	46.6873	OK	341.335	12.6325	3.38527	21.8797	OK	OK	-1.19442
ENSG0000009	-	-	ENSG0000009198	'CCDC80	-	3:112596793-112649530	-	-	IGV_LINK	268.613	4.68679	0	10.0425	OK	167.058	3.95866	0	9.33972	OK	OK	-0.24359
ENSG0000009	-	-	ENSG0000009200	'CMA1	-	14:24501593-24508688	-	-	IGV_LINK	0	0	0	0	OK	0	0	0	0	OK	NOTEST	0
ENSG0000009	-	-	ENSG0000009201	'PSME1	-	14:24136157-24141588	-	-	IGV_LINK	3659.73	248.99	98.8807	399.098	OK	2380.07	162.098	68.0065	256.189	OK	OK	-0.61922
ENSG0000009	-	-	ENSG0000009202	'PPP2R3C	-	14:35044906-35317493	-	-	IGV_LINK	220.127	10.6427	0	35.5261	OK	208.307	9.21052	0	30.15	OK	OK	-0.20852
ENSG0000009	-	-	ENSG0000009203	'HAUS4	-	14:22929608-22982642	-	-	IGV_LINK	703.169	35.4289	5.08089	65.7768	OK	678.026	32.4003	4.77125	60.0293	OK	OK	-0.12892
ENSG0000009	-	-	ENSG0000009205	'JPH4	-	14:23511759-23578800	-	-	IGV_LINK	23.2733	0.460003	0	3.76905	OK	7.21857	0.249268	0	3.52933	OK	NOTEST	-0.88395
ENSG0000009	-	-	ENSG0000009205	'MYH7	-	14:23412737-23435718	-	-	IGV_LINK	1.93944	0.01848	0	0.056593	OK	0	0	0	0	OK	NOTEST	'-inf
ENSG0000009	-	-	ENSG0000009206	'CEBPE	-	14:23117303-23119616	-	-	IGV_LINK	8.72748	0.356026	0	0.810285	OK	4.1249	0.16841	0	0.426627	OK	NOTEST	-1.08001
ENSG0000009	-	-	ENSG0000009206	'SLC7A8	-	14:23125294-23183674	-	-	IGV_LINK	37.8191	0.826736	0	2.08972	OK	579.548	13.5123	0.946695	26.078	OK	OK	4.03071
ENSG0000009	-	-	ENSG0000009209	'OSGEP	-	14:20446410-20455105	-	-	IGV_LINK	915.416	43.206	11.7441	74.6679	OK	690.921	34.3586	8.15652	60.5608	OK	OK	-0.33056
ENSG0000009	-	-	ENSG0000009209	'SLC22A17	-	14:23346305-23352912	-	-	IGV_LINK	655.531	20.4544	6.88102	34.0278	OK	447.552	14.7255	3.90625	25.5448	OK	OK	-0.47409
ENSG0000009	-	-	ENSG0000009209	'RNF31	-	14:24143361-24167402	-	-	IGV_LINK	703.474	28.2084	0	106.965	OK	687.276	21.4834	0	66.7301	OK	OK	-0.3929
ENSG0000009	-	-	ENSG0000009210	'SCFD1	-	14:30622111-30822702	-	-	IGV_LINK	431.526	17.2552	4.0559	30.4546	OK	485.707	16.6504	4.935	28.3657	OK	OK	-0.05148
ENSG0000009	-	-	ENSG0000009214	'G2E3	-	14:30437991-30620063	-	-	IGV_LINK	115.397	3.66644	0	10.1014	OK	110.341	3.70326	0	9.47959	OK	OK	0.014417
ENSG0000009	-	-	ENSG0000009214	'HECTD1	-	14:31100111-31207804	-	-	IGV_LINK	1387.67	23.903	0	49.2585	OK	1495.28	28.3728	0.297897	56.4476	OK	OK	0.247318
ENSG0000009	-	-	ENSG0000009219	'HNRNPC	-	14:21209135-21269494	-	-	IGV_LINK	5991.9	265.523	96.3859	434.661	OK	6237.88	286.25	96.4629	476.037	OK	OK	0.108437
ENSG0000009	-	-	ENSG0000009220	'RPGRIP1	-	14:21287938-21351301	-	-	IGV_LINK	31.0311	0.776846	0	1.90513	OK	49.4988	1.83587	0	5.12657	OK	OK	1.24076
ENSG0000009	-	-	ENSG0000009220	'SUPT16H	-	14:21351471-21384266	-	-	IGV_LINK	1378.94	20.1952	7.00415	33.3863	OK	1415.87	21.8994	6.20646	37.5924	OK	OK	0.116879
ENSG0000009	-	-	ENSG0000009220	'TOX4	-	14:21459019-21511375	-	-	IGV_LINK	1011.42	27.3541	0	64.3089	OK	1063.19	30.4682	0	65.4615	OK	OK	0.155543
ENSG0000009	-	-	ENSG0000009220	'GEMIN2	-	14:39114222-39136973	-	-	IGV_LINK	69.8199	5.59867	0.025784	11.1716	OK	70.1233	5.14228	0	10.2875	OK	OK	-0.12268
ENSG0000009	-	-	ENSG0000009229	'TGM1	-	14:24249113-24264432	-	-	IGV_LINK	31.0311	1.22432	0	3.42912	OK	17.5308	1.02526	0	2.92155	OK	OK	-0.25599
ENSG0000009	-	-	ENSG0000009233	'TINF2	-	14:24239642-24242674	-	-	IGV_LINK	777.716	25.3352	7.91083	42.7595	OK	769.294	24.0909	7.66983	40.5121	OK	OK	-0.07265
ENSG0000009	-	-	ENSG0000009234	'DAZL	-	3:16586791-16670306	-	-	IGV_LINK	0	0	0	0	OK	0	0	0	0	OK	NOTEST	0
ENSG0000009	-	-	ENSG0000009237	'TBL1Y	-	Y:6910685-7091683	-	-	IGV_LINK	0	0	0	0	OK	2.06245	0.054347	0	0.181893	OK	NOTEST	inf
ENSG0000009	-	-	ENSG0000009242	'SEMA6A	-	5:116443615-116591398	-	-	IGV_LINK	2259.45	29.8275	1.55064	58.1043	OK	767.231	11.1121	1.80083	20.4234	OK	OK	-1.42451
ENSG0000009	-	-	ENSG0000009243	'TRPM7	-	15:50552472-50686815	-	-	IGV_LINK	1127.78	12.4564	1.53096	23.3819	OK	954.914	12.4547	0.961267	23.9482	OK	OK	-0.0002
ENSG0000009	-	-	ENSG0000009244	'TYRO3	-	15:41557674-41583586	-	-	IGV_LINK	68.8502	1.7891	0	4.43996	OK	158.809	3.11205	0	6.47586	OK	OK	0.798634
ENSG0000009	-	-	ENSG0000009247	'WDR76	-	15:43826962-43868419	-	-	IGV_LINK	150.307	4.19324	0.071314	8.31516	OK	94.8727	2.04771	0.183572	3.91186	OK	OK	-1.03405
ENSG0000009	-	-	ENSG0000009252	'CAPN3	-	15:42208412-42412318	-	-	IGV_LINK	531.958	22.5281	0	57.9587	OK	523.372	26.7715	0	69.7977	OK	OK	0.248975

ENSG0000009	-	-	ENSG0000009253	'SNAP23	-	15:42491232-42548802	-	-	IGV_LINK	823.293	50.4297	8.12278	92.7366	OK	846.636	57.0276	7.55934	106.496	OK	OK	0.177387
ENSG0000009	-	-	ENSG0000009260	'TBX15	-	1:118883045-118989556	-	-	IGV_LINK	9.69721	0.164582	0	0.378414	OK	50.53	0.943191	0	1.90733	OK	OK	2.51875
ENSG0000009	-	-	ENSG0000009262	'PHGDH	-	1:119648410-119744226	-	-	IGV_LINK	203.641	8.53796	0.490016	16.5859	OK	42.2802	1.90633	0	4.47362	OK	OK	-2.16309
ENSG0000009	-	-	ENSG0000009275	'COL9A3	-	20:62816243-62861763	-	-	IGV_LINK	17.455	1.70508	0	5.29996	OK	13.4059	0.828111	0	2.71987	OK	OK	-1.04195
ENSG0000009	-	-	ENSG0000009282	'EZR	-	6:158765740-158820593	-	-	IGV_LINK	7639.46	148.216	48.9468	247.486	OK	4922.04	95.7437	38.3566	153.131	OK	OK	-0.63045
ENSG0000009	-	-	ENSG0000009284	'MYL6	-	12:56150795-56190284	-	-	IGV_LINK	12683	1416.07	179.531	2652.61	OK	12219	1395.18	131.606	2658.75	OK	OK	-0.02145
ENSG0000009	-	-	ENSG0000009284	'AGO1	-	1:35869807-35930528	-	-	IGV_LINK	965.842	5.1493	1.44749	8.85112	OK	982.757	4.89974	1.67257	8.12691	OK	OK	-0.07167
ENSG0000009	-	-	ENSG0000009285	'TEKT2	-	1:36084074-36088275	-	-	IGV_LINK	68.8502	2.81035	0	6.07195	OK	23.7182	0.978566	0	2.10123	OK	OK	-1.52201
ENSG0000009	-	-	ENSG0000009285	'CLSPN	-	1:35720217-35769967	-	-	IGV_LINK	87.2748	1.45517	0	3.31847	OK	57.7486	0.796104	0	1.7114	OK	OK	-0.87015
ENSG0000009	-	-	ENSG0000009287	'RFFL	-	17:34927858-35121522	-	-	IGV_LINK	957.114	18.1018	0	51.5342	OK	1536.53	27.444	0	67.238	OK	OK	0.600355
ENSG0000009	-	-	ENSG0000009292	'UNC13D	-	17:75827224-75844717	-	-	IGV_LINK	715.654	17.6881	0	38.0136	OK	741.451	18.9525	0	48.7386	OK	OK	0.099611
ENSG0000009	-	-	ENSG0000009293	'MFSD11	-	17:76726829-76781449	-	-	IGV_LINK	631.288	24.5474	0	80.0691	OK	699.171	18.6938	0	49.148	OK	OK	-0.39301
ENSG0000009	-	-	ENSG0000009296	'DPYSL2	-	8:26379258-26658178	-	-	IGV_LINK	2118.84	27.3503	0	57.6527	OK	2879.18	38.3871	0	100.606	OK	OK	0.489062
ENSG0000009	-	-	ENSG0000009296	'TGFB2	-	1:218346234-218444619	-	-	IGV_LINK	39.5593	1.16032	0	3.69153	OK	32.3132	1.06216	0	3.51072	OK	OK	-0.12751
ENSG0000009	-	-	ENSG0000009297	'GPATCH2	-	1:217426991-217631082	-	-	IGV_LINK	288.007	6.29068	0	15.9511	OK	251.619	6.74849	0	17.4096	OK	OK	0.10135
ENSG0000009	-	-	ENSG0000009300	'NUP50	-	22:45163840-45188015	-	-	IGV_LINK	1142.33	16.224	1.47679	30.9712	OK	1483.93	19.2324	4.02606	34.4388	OK	OK	0.245412
ENSG0000009	-	-	ENSG0000009300	'CDC45	-	22:19479458-19520612	-	-	IGV_LINK	59.153	2.16791	0	4.6204	OK	54.6549	2.69621	0	5.8775	OK	OK	0.314625
ENSG0000009	-	-	ENSG0000009301	'COMT	-	22:19875516-20016808	-	-	IGV_LINK	1145.24	80.6358	17.0666	144.205	OK	1645.84	120.582	27.2245	213.939	OK	OK	0.58052
ENSG0000009	-	-	ENSG0000009307	'ADA2	-	22:17178789-17221989	-	-	IGV_LINK	3102.14	53.7173	11.4217	96.0128	OK	2487.31	42.897	6.56175	79.2322	OK	OK	-0.32451
ENSG0000009	-	-	ENSG0000009310	'AC016026.1	-	22:17787648-18024559	-	-	IGV_LINK	680.351	9.22673	0	21.9755	OK	459.631	6.2351	0	14.4835	OK	OK	-0.56541
ENSG0000009	-	-	ENSG0000009313	'VNN3	-	6:132722786-132734765	-	-	IGV_LINK	14.5458	0.706151	0	1.8061	OK	91.779	3.73148	0.085612	7.37736	OK	OK	2.4017
ENSG0000009	-	-	ENSG0000009314	'ECHDC1	-	6:127288709-127343609	-	-	IGV_LINK	683.653	31.4825	10.477	52.488	OK	494.988	23.4082	7.78866	39.0277	OK	OK	-0.42754
ENSG0000009	-	-	ENSG0000009316	'LRRFIP2	-	3:37052655-37183689	-	-	IGV_LINK	837.839	22.7869	5.02238	40.5514	OK	845.604	22.4879	5.89188	39.084	OK	OK	-0.01905
ENSG0000009	-	-	ENSG0000009318	'SEC22C	-	3:42547968-42665854	-	-	IGV_LINK	408.252	9.55487	0	30.3486	OK	460.958	11.9584	0	32.6821	OK	OK	0.323717
ENSG0000009	-	-	ENSG0000009321	'XYLB	-	3:38346759-38421348	-	-	IGV_LINK	111.518	2.84745	0	7.29603	OK	103.122	1.86527	0	4.06687	OK	OK	-0.61028
ENSG0000009	-	-	ENSG0000009463	'HDAC6	-	X:48801376-48824982	-	-	IGV_LINK	2166.36	61.9617	0	124.081	OK	2928.68	87.067	0	174.992	OK	OK	0.490748
ENSG0000009	-	-	ENSG0000009466	'OR1I1	-	19:15082210-15092970	-	-	IGV_LINK	0	0	0	0	OK	0	0	0	0	OK	NOTEST	0
ENSG0000009	-	-	ENSG0000009475	'GABRP	-	5:170747046-170814047	-	-	IGV_LINK	44.6071	1.13885	0	3.18217	OK	31.968	0.866042	0	2.74861	OK	OK	-0.39506
ENSG0000009	-	-	ENSG0000009479	'KRT31	-	17:41393723-41397592	-	-	IGV_LINK	1.93944	0.076783	0	0.235143	OK	0	0	0	0	OK	NOTEST	'-inf
ENSG0000009	-	-	ENSG0000009480	'CDC6	-	17:40287632-40304657	-	-	IGV_LINK	66.9107	1.80504	0	4.86702	OK	78.3731	1.69562	0	4.42007	OK	OK	-0.09021
ENSG0000009	-	-	ENSG0000009484	'UPRT	-	X:75274084-75304600	-	-	IGV_LINK	136.731	4.85365	0.547977	9.15933	OK	158.809	5.21791	0.846867	9.58896	OK	OK	0.104401
ENSG0000009	-	-	ENSG0000009488	'CDC23	-	5:138139765-138213343	-	-	IGV_LINK	764.14	17.7914	0	48.9568	OK	733.201	16.9497	0	46.9371	OK	OK	-0.06992
ENSG0000009	-	-	ENSG0000009491	'AAAS	-	12:53307455-53324864	-	-	IGV_LINK	1068.63	44.8552	15.6012	74.1093	OK	1216.85	54.9647	19.3995	90.5298	OK	OK	0.293229
ENSG0000009	-	-	ENSG0000009491	'CBX5	-	12:54230939-54351849	-	-	IGV_LINK	2627.94	15.3529	0	60.5261	OK	2440.91	13.5161	0	47.0153	OK	OK	-0.18383
ENSG0000009	-	-	ENSG0000009496	'FMO2	-	1:171185207-171227788	-	-	IGV_LINK	842.687	20.1677	2.23162	38.1039	OK	9.28102	0.231437	0	0.859813	OK	OK	-6.44529
ENSG0000009	-	-	ENSG0000009497	'SUCO	-	1:172532348-172611833	-	-	IGV_LINK	312.25	3.95784	0.084733	7.83095	OK	380.522	4.78087	0.153069	9.40867	OK	OK	0.272559
ENSG0000009	-	-	ENSG0000009500	'MSH2	-	2:47402968-47570939	-	-	IGV_LINK	373.342	7.44362	1.26681	13.6204	OK	333.086	6.13169	1.80444	10.4589	OK	OK	-0.27972
ENSG0000009	-	-	ENSG0000009501	'MAP3K1	-	5:56815573-56896152	-	-	IGV_LINK	1037.6	8.85414	3.73947	13.9688	OK	625.954	5.41617	2.11398	8.71836	OK	OK	-0.70908
ENSG0000009	-	-	ENSG0000009505	'DHPS	-	19:12643274-12681902	-	-	IGV_LINK	1168.51	72.733	0	179.06	OK	1093.1	69.0186	0	185.009	OK	OK	-0.07562
ENSG0000009	-	-	ENSG0000009506	'HOOK2	-	19:12763002-12874951	-	-	IGV_LINK	901.84	45.91	0	132.155	OK	1195.19	64.0693	0	194.743	OK	OK	0.480824
ENSG0000009	-	-	ENSG0000009511	'NXPE1	-	11:114521714-114559895	-	-	IGV_LINK	0.969721	0.031796	0	0.368486	OK	0	0	0	0	OK	NOTEST	'-inf
ENSG0000009	-	-	ENSG0000009513	'ARCN1	-	11:118436489-118603033	-	-	IGV_LINK	2196.42	40.4072	1.20719	79.6073	OK	2602.81	49.4938	7.19199	91.7956	OK	OK	0.292634
ENSG0000009	-	-	ENSG0000009520	'EPB41L4B	-	9:109171974-109320964	-	-	IGV_LINK	18.4247	0.257073	0	0.546742	OK	8.2498	0.121316	0	0.280796	OK	NOTEST	-1.08342
ENSG0000009	-	-	ENSG0000009520	'TMEM38B	-	9:105694543-105776612	-	-	IGV_LINK	786.443	30.9033	3.47657	58.3301	OK	472.301	17.3747	1.80836	32.9411	OK	OK	-0.83077
ENSG0000009	-	-	ENSG0000009526	'PSMD5	-	9:120815495-120854385	-	-	IGV_LINK	640.016	13.1137	0	29.0636	OK	766.2	14.5359	0	29.6732	OK	OK	0.148538
ENSG0000009	-	-	ENSG0000009530	'PTGS1	-	9:122370529-122395703	-	-	IGV_LINK	4679.87	66.7321	24.0403	109.424	OK	2940.02	43.8159	14.7777	72.8541	OK	OK	-0.60693
ENSG0000009	-	-	ENSG0000009531	'NUP188	-	9:128920894-129028303	-	-	IGV_LINK	826.202	16.3728	0	59.789	OK	809.512	14.3721	0	47.1883	OK	OK	-0.18803
ENSG0000009	-	-	ENSG0000009532	'CRAT	-	9:129094809-129148946	-	-	IGV_LINK	848.505	22.6266	0	58.2646	OK	1351.94	33.7683	0	89.014	OK	OK	0.577651
ENSG0000009	-	-	ENSG0000009537	'SH2D3C	-	9:127738316-127778741	-	-	IGV_LINK	680.744	13.9857	5.01587	22.9556	OK	615.641	14.3308	4.00502	24.6565	OK	OK	0.035158
ENSG0000009	-	-	ENSG0000009538	'NANS	-	9:98056738-98119212	-	-	IGV_LINK	356.857	19.7173	0	45.0185	OK	439.302	23.893	0	64.6824	OK	OK	0.277131
ENSG0000009	-	-	ENSG0000009538	'TBC1D2	-	9:98198998-98255721	-	-	IGV_LINK	203.641	3.90057	0.92183	6.87931	OK	1029.16	20.0405	7.4458	32.6351	OK	OK	2.36116
ENSG0000009	-	-	ENSG0000009539	'WHRN	-	9:114402079-114505450	-	-	IGV_LINK	632.258	12.7587	4.34425	21.1732	OK	372.272	7.09853	1.93214	12.2649	OK	OK	-0.84589
ENSG0000009	-	-	ENSG0000009546	'PDE6C	-	10:93612587-93666010	-	-	IGV_LINK	5.81832	0.104609	0	0.384421	OK	4.1249	0.190186	0	0.727096	OK	NOTEST	0.862405
ENSG0000009	-	-	ENSG0000009548	'CWF19L1	-	10:100229666-100267680	-	-	IGV_LINK	404.373	11.9776	3.49201	20.4633	OK	274.306	8.62456	1.30135	15.9478	OK	OK	-0.47382
ENSG0000009	-	-	ENSG0000009553	'SEMA4G	-	10:100969457-100994401	-	-	IGV_LINK	481.951	13.334	0	34.7471	OK	248.525	7.07993	0	20.6359	OK	OK	-0.91331
ENSG0000009	-	-	ENSG0000009556	'BTAF1	-	10:91923768-92030325	-	-	IGV_LINK	1929.74	19.4684	6.61405	32.3227	OK	837.355	8.07516	2.64283	13.5075	OK	OK	-1.26957
ENSG0000009	-	-	ENSG0000009557	'IKZF5	-	10:122930902-123008817	-	-	IGV_LINK	335.523	5.86979	0	13.2765	OK	183.558	3.76015	0	9.33984	OK	OK	-0.64252
ENSG0000009	-	-	ENSG0000009558	'BLNK	-	10:96129714-96271587	-	-	IGV_LINK	547.892	23.5548	1.06856	46.041	OK	345.46	13.836	0.350211	27.3217	OK	OK	-0.7676
ENSG0000009	-	-	ENSG0000009558	'TLL2	-	10:96364605-96513918	-	-	IGV_LINK	2.90916	0.032074	0	0.138458	OK	7.21857	0.078952	0	0.23727	OK	NOTEST	1.29959
ENSG0000009	-	-	ENSG0000009559	'CYP26A1	-	10:93073474-93077890	-	-	IGV_LINK	0	0	0	0	OK	2.06245	0.173913	0	0.623882	OK	NOTEST	inf
ENSG0000009	-	-	ENSG0000009562	'TDRD1	-	10:114179269-114232304	-	-	IGV_LINK	2.90916	0.046411	0	0.133842	OK	2.06245	0.026804	0	0.093587	OK	NOTEST	-0.79201
ENSG0000009	-	-	ENSG0000009563	'SORBS1	-	10:95311770-95561414	-	-	IGV_LINK	1095.78	15.4722	0	31.0344	OK	4334.24	57.4331	0	115.243	OK	OK	1.8922
ENSG0000009	-	-	ENSG0000009571	'CRTAC1	-	10:97850238-98030828	-	-	IGV_LINK	1.93944	0.054473	0	0.594465	OK	3239.08	76.2602	26.562	125.958	OK	OK	10.4512
ENSG0000009	-	-	ENSG0000009573	'BAMBI	-	10:28677341-28682939	-	-	IGV_LINK	204.611	6.76343	1.74315	11.7837	OK	31.968	1.05768	0	2.16859	OK	OK	-2.67686
ENSG0000009	-	-	ENSG0000009575	'IL11	-	19:55364388-55370463	-	-	IGV_LINK	0.969721	0.025919	0	0.266569	OK	1.03122	0.068022	0	0.352449	OK	NOTEST	1.392
ENSG0000009	-	-	ENSG0000009577	'MYO3A	-	10:25934266-26212527	-	-	IGV_LINK	470.314	5.42065	0.845933	9.99537	OK	142.309	1.84956	0	4.56083	OK	OK	-1.55128
ENSG0000009	-	-	ENSG0000009578	'WAC	-	10:28522651-28623112	-	-	IGV_LINK	2891.71	65.6326	13.1525	118.113	OK	1602.52	35.7479	9.32253	62.1733	OK	OK	-0.87656
ENSG0000009	-	-	ENSG0000009579	'CREM	-	10:35098005-35212958	-	-	IGV_LINK	289.946	20.9412	5.55082	36.3317	OK	397.022	31.0273	8.32045	53.7342	OK	OK	0.567193
ENSG0000009	-	-	ENSG0000009590	'NUBP2	-	16:1773206-1794971	-	-	IGV_LINK	716.623	33.6233	0	72.6615	OK	675.452	31.1856	0	70.9386	OK	OK	-0.10858
ENSG0000009	-	-	ENSG0000009591	'TPSD1	-	16:1256058-1258998	-	-	IGV_LINK	11.6366	0.593803	0	1.38012	OK	3.09367	0.13155	0	0.395698	OK	OK	-2.17437
ENSG0000009	-	-	ENSG0000009593	'SMIM24	-	19:3473985-3480542	-	-	IGV_LINK	499.406	38.8246	12.685	64.9642	OK	698.139	50.9485	15.3084	86.5887	OK	OK	0.39207
ENSG0000009	-	-	ENSG0000009595	'HIVEP1	-	6:12007669-12164999	-	-	IGV_LINK	453.829	6.88803	0	14.3914	OK	301.118	4.65269	0.527062	8.77832	OK	OK	-0.56603
ENSG0000009	-	-	ENSG0000009597	'TREM2	-	6:41158505-41163186	-	-	IGV_LINK	965.842	63.5504	26.2179	100.883	OK	667.203	43.6809	17.5726	69.7892	OK	OK	-0.5409
ENSG0000009	-	-	ENSG0000009598	'KCNK16	-	6:39314697-39322968	-	-	IGV_LINK	0	0	0	0	OK	0	0	0	0	OK	NOTEST	0
ENSG0000009	-	-	ENSG0000009600	'CRISP3	-	6:49727383-49744437	-	-	IGV_LINK	0	0	0	0	OK	0	0	0	0	OK	NOTEST	0
ENSG0000009	-	-	ENSG0000009606	'FKBP5	-	6:35573584-35728583	-	-	IGV_LINK	1220.88	18.4761	7.95518	28.997	OK	5245.84	81.5295	32.8161	130.243	OK	OK	2.14166
ENSG0000009	-	-	ENSG0000009606	'SRPK1	-	6:35805292-35921342	-	-	IGV_LINK	565.347	10.3087	0.511826	20.1056	OK	401.147	7.7561	0	15.973	OK	OK	-0.41046
ENSG0000009	-	-	ENSG0000009607	'BRPF3	-	6:36146697-36232790	-	-	IGV_LINK	940.629	10.5911	3.02252	18.1597	OK	715.67	7.675	2.70923	12.6408	OK	OK	-0.46461
ENSG0000009	-	-	ENSG0000009608	'MRPS18A	-	6:43645045-43687791	-	-	IGV_LINK	579.893	37.3093	13.6272	60.9914	OK	350.616	22.757	6.77927	38.7347	OK	OK	-0.71322
ENSG0000009	-	-	ENSG0000009608	'PGC	-	6:41736710-41754109	-	-	IGV_LINK	0.969721	0.045599	0	0.449625	OK	0	0	0	0	OK	NOTEST	'-inf
ENSG0000009	-	-	ENSG0000009609	'TMEM14A	-	6:52671108-52686588	-	-	IGV_LINK	363.645	25.008	8.6694	41.3466	OK	266.056	18.3225	5.65889	30.9861	OK	OK	-0.44878
ENSG0000009	-	-	ENSG0000009609	'EFHC1	-	6:52361420-52576915	-	-	IGV_LINK	1146.21	30.1514	0	66.6182	OK	637.376	17.7707	0	42.5022	OK	OK	-0.76273
ENSG0000009	-	-	ENSG0000009626	'NCR2	-	6:41335654-41350887	-	-	IGV_LINK	0.969721	0.071012	0	0.342028	OK	1.03122	0.075627	0	0.347915	OK	NOTEST	0.090839
ENSG0000009	-	-	ENSG0000009638	'HSP90AB1	-	6:44246165-44253888	-	-	IGV_LINK	17029.3	399.8	104.043	695.557	OK	9494.49	223.058	76.2722	369.843	OK	OK	-0.84186
ENSG0000009	-	-	ENSG0000009639	'MLN	-	6:33794672-33804011	-	-	IGV_LINK	0	0	0	0	OK	0	0	0	0	OK	NOTEST	0
ENSG0000009	-	-	ENSG0000009640	'CDC5L	-	6:44387524-44450426	-	-	IGV_LINK	814.565	7.34426	3.10083	11.5877	OK	590.892	5.32856	2.09738	8.55975	OK	OK	-0.46287
ENSG0000009	-	-	ENSG0000009643	'ITPR3	-	6:33620364-33711727	-	-	IGV_LINK	2177.02	13.828	5.14808	22.5079	OK	2255.29	14.3066	5.49198	23.1212	OK	OK	0.049088
ENSG0000009	-	-	ENSG0000009665	'ZNF184	-	6:27450742-27473118	-	-	IGV_LINK	172.61	3.51297	0.582061	6.44388	OK	93.8415	1.90493	0.209111	3.60075	OK	OK	-0.88295
ENSG0000009	-	-	ENSG0000009669	'DSP	-	6:7541574-7586717	-	-	IGV_LINK	1946.23	11.5667	5.09228	18.0411	OK	1263.25	7.84131	3.40113	12.2815	OK	OK	-0.56081
ENSG0000009	-	-	ENSG0000009671	'SIRT1	-	10:67884668-67918390	-	-	IGV_LINK	451.89	7.19899	2.25003	12.148	OK	272.243	4.09809	1.11744	7.07874	OK	OK	-0.81284
ENSG0000009	-	-	ENSG0000009674	'HNRNPH3	-	10:68282659-68407294	-	-	IGV_LINK	2177.99	97.2783	0	197.045	OK	1424.12	67.2099	0	137.91	OK	OK	-0.53345
ENSG0000009	-	-	ENSG0000009687	'IFT74	-	9:26904082-27062930	-	-	IGV_LINK	320.977	10.2713	0	21.0859	OK	389.803	12.7754	0	26.7943	OK	OK	0.314754
ENSG0000009	-	-	ENSG0000009696	'JAK2	-	9:4984389-5128183	-	-	IGV_LINK	897.961	10.5763	3.38365	17.769	OK	421.771	5.25283	0.918937	9.58673	OK	OK	-1.00967
ENSG0000009	-	-	ENSG0000009699	'IL12RB1	-	19:18058994-18151692	-	-	IGV_LINK	350.069	9.89504	0	22.6641	OK	201.089	5.59293	0	14.1044	OK	OK	-0.8231
ENSG0000009	-	-	ENSG0000009700	'ABL1	-	9:130713945-130887675	-	-	IGV_LINK	1131.66	15.4697	0	31.3026	OK	1365.34	17.0982	1.48013	32.7163	OK	OK	0.1444
ENSG0000009	-	-	ENSG0000009702	'ACOT7	-	1:6264268-6394391	-	-	IGV_LINK	486.8	20.6127	7.75221	33.4733	OK	1540.65	66.7846	27.7402	105.829	OK	OK	1.69598
ENSG0000009	-	-	ENSG0000009703	'SH3GLB1	-	1:86704569-86748184	-	-	IGV_LINK	1658.22	32.6414	2.16894	63.1138	OK	2054.2	48.4258	10.9194	85.9322	OK	OK	0.569075
ENSG0000009	-	-	ENSG0000009704	'CDC7	-	1:91500892-91525764	-	-	IGV_LINK	118.306	2.29486	0.020573	4.56915	OK	135.09	2.78106	0.141575	5.42054	OK	OK	0.277227
ENSG0000009	-	-	ENSG0000009709	'SYDE2	-	1:85156872-85201046	-	-	IGV_LINK	92.1234	1.04359	0.090609	1.99657	OK	48.4676	0.555336	0	1.12772	OK	OK	-0.91012
ENSG0000009	-	-	ENSG0000009913	'PCSK5	-	9:75890643-76362339	-	-	IGV_LINK	105.7	0.976201	0.054431	1.89797	OK	367.116	2.82919	0.787667	4.87072	OK	OK	1.53514
ENSG0000009	-	-	ENSG0000009919	'SCD	-	10:100347123-100364834	-	-	IGV_LINK	8378.39	90.9855	32.5128	149.458	OK	7802.25	84.7477	31.4205	138.075	OK	OK	-0.10246
ENSG0000009	-	-	ENSG0000009920	'TMED1	-	19:10718078-10836318	-	-	IGV_LINK	605.106	30.277	0	96.1739	OK	921.915	46.404	0	126.652	OK	OK	0.616026
ENSG0000009	-	-	ENSG0000009920	'ABLIM1	-	10:114431112-114685003	-	-	IGV_LINK	2498	21.8504	2.7878	40.913	OK	2115.04	17.9415	3.67678	32.2062	OK	OK	-0.28436
ENSG0000009	-	-	ENSG0000009921	'ERMP1	-	9:5629024-5833117	-	-	IGV_LINK	542.074	6.34697	0.583766	12.1102	OK	512.519	6.43294	0	13.2175	OK	OK	0.01941
ENSG0000009	-	-	ENSG0000009924	'RAB18	-	10:27504173-27542237	-	-	IGV_LINK	667.168	31.1264	5.10783	57.1451	OK	569.236	25.371	0	51.5949	OK	OK	-0.29496
ENSG0000009	-	-	ENSG0000009925	'NRP1	-	10:33177491-33336262	-	-	IGV_LINK	4857.33	80.8772	0	176.96	OK	5215.94	87.3465	0	191.881	OK	OK	0.111017
ENSG0000009	-	-	ENSG0000009925	'HSD17B7P2	-	10:38094333-38378505	-	-	IGV_LINK	52.5655	2.41141	0	8.15085	OK	53.1204	2.43915	0	7.0924	OK	OK	0.016501
ENSG0000009	-	-	ENSG0000009925	'PRTFDC1	-	10:24848606-24952604	-	-	IGV_LINK	171.641	7.86275	0.690266	15.0352	OK	55.6861	3.08343	0	6.5784	OK	OK	-1.3505
ENSG0000009	-	-	ENSG0000009926	'PALMD	-	1:99645942-99694541	-	-	IGV_LINK	143.519	3.62336	0.483886	6.76283	OK	315.555	7.9533	1.89255	14.0141	OK	OK	1.13423
ENSG0000009	-	-	ENSG0000009928	'TSPAN15	-	10:69451472-69507669	-	-	IGV_LINK	436.374	18.0644	5.68685	30.4419	OK	342.367	13.9846	4.12365	23.8455	OK	OK	-0.36931
ENSG0000009	-	-	ENSG0000009928	'H2AFY2	-	10:70052795-70132934	-	-	IGV_LINK	91.1537	2.84209	0	6.90467	OK	141.278	4.40772	0	10.7253	OK	OK	0.63308
ENSG0000009	-	-	ENSG0000009929	'WASHC2A	-	10:50067887-50133506	-	-	IGV_LINK	1310.09	24.0739	4.00212	44.1458	OK	744.544	13.0483	1.82262	24.2739	OK	OK	-0.88361
ENSG0000009	-	-	ENSG0000009930	'MAST3	-	19:18058994-18151692	-	-	IGV_LINK	638.076	6.49007	1.31074	11.6694	OK	851.792	9.08001	1.18701	16.973	OK	OK	0.48446
ENSG0000009	-	-	ENSG0000009932	'MZF1	-	19:58559128-58599801	-	-	IGV_LINK	929.962	22.4765	4.69018	40.2628	OK	795.074	18.4552	3.90418	33.0062	OK	OK	-0.28439
ENSG0000009	-	-	ENSG0000009933	'OCEL1	-	19:17226203-17229219	-	-	IGV_LINK	387.888	28.0654	6.04811	50.0827	OK	504.269	33.3067	10.2139	56.3995	OK	OK	0.247021
ENSG0000009	-	-	ENSG0000009933	'MYO9B	-	19:17075780-17214537	-	-	IGV_LINK	3066.26	57.9001	0	117.094	OK	3609.29	65.7327	0	137.767	OK	OK	0.183047
ENSG0000009	-	-	ENSG0000009933	'KCNK6	-	19:38319843-38332076	-	-	IGV_LINK	297.704	3.1928	0.257694	6.12791	OK	448.583	5.46173	0	11.7341	OK	OK	0.774536
ENSG0000009	-	-	ENSG0000009933	'CATSPERG	-	19:38335774-38370943	-	-	IGV_LINK	73.6988	4.308	0	9.98866	OK	78.3731	5.31772	0	11.5264	OK	OK	0.30379
ENSG0000009	-	-	ENSG0000009934	'PSMD8	-	19:38374535-38383824	-	-	IGV_LINK	2209.99	124.832	51.8654	197.799	OK	2411	138.241	56.0701	220.413	OK	OK	0.147201
ENSG0000009	-	-	ENSG0000009936	'FBXL19	-	16:30919318-30956511	-	-	IGV_LINK	378.696	7.59572	0	17.6013	OK	438.271	9.09881	0	21.7874	OK	OK	0.260492
ENSG0000009	-	-	ENSG0000009936	'STX1B	-	16:30989255-31010661	-	-	IGV_LINK	211.399	3.49611	0	7.37473	OK	227.901	4.32947	0	9.34838	OK	OK	0.30844
ENSG0000009	-	-	ENSG0000009937	'HSD3B7	-	16:30957293-30989152	-	-	IGV_LINK	1703.8	50.7745	14.5813	86.9676	OK	4110.46	122.738	36.2569	209.22	OK	OK	1.27341
ENSG0000009	-	-	ENSG0000009938	'SETD1A	-	16:30957293-30989152	-	-	IGV_LINK	620.621	6.05878	0	15.065	OK	755.888	9.22244	0	34.8711	OK	OK	0.60612
ENSG0000009	-	-	ENSG0000009938	'BCL7C	-	16:30833625-30895220	-	-	IGV_LINK	496.497	18.0109	6.71641	29.3055	OK	426.927	15.62	5.29832	25.9418	OK	OK	-0.20548
ENSG0000009	-	-	ENSG0000009939	'MAGEB2	-	X:30215559-30220089	-	-	IGV_LINK	0	0	0	0	OK	0	0	0	0	OK	NOTEST	0
ENSG0000009	-	-	ENSG0000009961	'EFNA2	-	19:1286153-1300237	-	-	IGV_LINK	0	0	0	0	OK	0	0	0	0	OK	NOTEST	0
ENSG0000009	-	-	ENSG0000009962	'CIRBP	-	19:1259383-1274880	-	-	IGV_LINK	7001.38	378.269	106.729	649.808	OK	4679.7	257.841	93.2548	422.427	OK	OK	-0.55293
ENSG0000009	-	-	ENSG0000009962	'ATP5D	-	19:1241745-1244826	-	-	IGV_LINK	1062.81	122.975	41.7927	204.157	OK	820.855	86.475	33.1274	139.823	OK	OK	-0.50801
ENSG0000009	-	-	ENSG0000009962	'CBARP	-	19:1177557-1238027	-	-	IGV_LINK	9.69721	0.28872	0	2.78114	OK	10.3122	0.293659	0	2.78427	OK	NOTEST	0.024469
ENSG0000009	-	-	ENSG0000009971	'PCDH11Y	-	Y:5000225-5742224	-	-	IGV_LINK	4.8486	0.03931	0	0.449651	OK	6.18735	0.076861	0	0.49728	OK	NOTEST	0.967354
ENSG0000009	-	-	ENSG0000009972	'AMELY	-	Y:6865917-6874027	-	-	IGV_LINK	0	0	0	0	OK	0	0	0	0	OK	NOTEST	0
ENSG0000009	-	-	ENSG0000009972	'PRKY	-	Y:7273971-7381548	-	-	IGV_LINK	310.311	3.33179	0	8.46832	OK	281.524	2.4939	0	6.7376	OK	OK	-0.4179

ENSG0000009	-	ENSG0000009976	IGFALS	-	16:1773206-1794971	-	-	IGV_LINK	7.75776	0.222662	0	2.54281	OK	2.06245	0.208926	0	4.70355	OK	NOTEST	-0.09187
ENSG0000009	-	ENSG0000009978	HNRNPM	-	19:8444569-8489114	-	-	IGV_LINK	2221.63	73.2533	25.2079	121.299	OK	2117.1	70.7494	20.4075	121.091	OK	OK	-0.05018
ENSG0000009	-	ENSG0000009978	MARCH2	-	19:8413269-8439017	-	-	IGV_LINK	626.439	32.4965	10.9556	54.0375	OK	653.797	34.1727	11.0603	57.285	OK	OK	0.072558
ENSG0000009	-	ENSG0000009979	NDUFB7	-	19:14566077-14572062	-	-	IGV_LINK	1144.27	179.893	78.2981	281.487	OK	1085.88	171.256	74.585	267.927	OK	OK	-0.07098
ENSG0000009	-	ENSG0000009979	TECR	-	19:14514769-14565980	-	-	IGV_LINK	1069.6	64.9596	9.05537	120.864	OK	1355.03	79.9631	10.622	149.304	OK	OK	0.299792
ENSG0000009	-	ENSG0000009980	TIMM13	-	19:2389770-2456996	-	-	IGV_LINK	737.957	42.8882	4.23379	81.5426	OK	815.18	43.2218	5.34172	81.1018	OK	OK	0.011178
ENSG0000009	-	ENSG0000009980	CDC34	-	19:531711-542092	-	-	IGV_LINK	639.046	30.5698	11.7747	49.365	OK	602.235	30.5608	7.94273	53.1788	OK	OK	-0.00043
ENSG0000009	-	ENSG0000009981	MTAP	-	9:21802542-22121097	-	-	IGV_LINK	592.555	9.26222	0.112492	18.4119	OK	606.834	11.7968	0	30.3967	OK	OK	0.348968
ENSG0000009	-	ENSG0000009981	MISP	-	19:751125-764318	-	-	IGV_LINK	3.87888	0.151252	0	0.442954	OK	176.339	3.68428	0.878675	6.48988	OK	OK	4.60636
ENSG0000009	-	ENSG0000009981	CEP170B	-	14:104865279-104896770	-	-	IGV_LINK	469.345	5.20702	0.077673	10.3364	OK	514.581	5.436	0.38231	10.4897	OK	OK	0.062087
ENSG0000009	-	ENSG0000009981	POLR2E	-	19:1065922-1095380	-	-	IGV_LINK	2283.69	119.365	15.786	222.945	OK	2466.69	132.946	12.8862	253.006	OK	OK	0.155454
ENSG0000009	-	ENSG0000009982	POLRMT	-	19:617223-633604	-	-	IGV_LINK	1863.8	55.4221	0.987392	109.857	OK	1721.11	47.952	1.55531	94.3486	OK	OK	-0.20887
ENSG0000009	-	ENSG0000009982	HCN2	-	19:589892-617159	-	-	IGV_LINK	2.90916	0.050672	0	0.136002	OK	5.15612	0.089841	0	0.220232	OK	NOTEST	0.826197
ENSG0000009	-	ENSG0000009983	CDHR5	-	11:616564-626078	-	-	IGV_LINK	259.885	6.65384	0	13.7491	OK	1962.42	56.3409	5.7981	106.884	OK	OK	3.08192
ENSG0000009	-	ENSG0000009984	IZUMO4	-	19:2071037-2099593	-	-	IGV_LINK	88.2446	6.10064	0	20.455	OK	113.435	7.33626	0	22.828	OK	OK	0.266085
ENSG0000009	-	ENSG0000009984	RASSF7	-	11:537526-564021	-	-	IGV_LINK	963.902	39.5274	3.79067	75.2642	OK	616.673	24.6598	2.31823	47.0014	OK	OK	-0.68069
ENSG0000009	-	ENSG0000009986	GADD45B	-	19:2476121-2478259	-	-	IGV_LINK	647.773	32.6003	10.1456	55.055	OK	947.696	45.1602	17.8522	72.4682	OK	OK	0.470166
ENSG0000009	-	ENSG0000009986	PALM	-	19:708938-748329	-	-	IGV_LINK	151.276	3.46241	0.476512	6.4483	OK	90.7478	2.40961	0	4.99541	OK	OK	-0.52298
ENSG0000009	-	ENSG0000009986	MADCAM1	-	19:489175-519654	-	-	IGV_LINK	14.5458	0.528699	0	2.00217	OK	10.3122	0.472954	0	2.23221	OK	NOTEST	-0.16075
ENSG0000009	-	ENSG0000009986	IGF2-AS	-	11:2129111-2161341	-	-	IGV_LINK	0	0	0	0	OK	0	0	0	0	OK	NOTEST	0
ENSG0000009	-	ENSG0000009987	MKNK2	-	19:2037464-2051244	-	-	IGV_LINK	1577.74	35.8416	5.64378	66.0394	OK	1683.99	42.2204	2.44268	81.9981	OK	OK	0.236304
ENSG0000009	-	ENSG0000009988	ARVCF	-	22:19875516-20016808	-	-	IGV_LINK	266.673	7.29621	0	20.1168	OK	262.962	7.39086	0	23.9691	OK	OK	0.018596
ENSG0000009	-	ENSG0000009989	TRMT2A	-	22:20080231-20127357	-	-	IGV_LINK	920.265	48.0574	0	104.443	OK	976.57	50.3576	0	106.29	OK	OK	0.067452
ENSG0000009	-	ENSG0000009990	RANBP1	-	22:20080231-20127357	-	-	IGV_LINK	755.412	54.5434	0	114.03	OK	754.857	57.6033	0	117.646	OK	OK	0.078747
ENSG0000009	-	ENSG0000009990	ZDHHC8	-	22:20129455-20148007	-	-	IGV_LINK	758.321	22.2858	2.77533	41.7962	OK	819.824	27.345	4.1555	50.5345	OK	OK	0.295153
ENSG0000009	-	ENSG0000009991	KLHL22	-	22:20424814-20495883	-	-	IGV_LINK	432.495	12.8804	1.75439	24.0064	OK	359.898	11.8596	1.14778	22.5715	OK	OK	-0.11912
ENSG0000009	-	ENSG0000009991	MED15	-	22:20495912-20592218	-	-	IGV_LINK	2060.66	44.1647	7.70009	80.6294	OK	1818.05	38.6373	5.57794	71.6966	OK	OK	-0.1929
ENSG0000009	-	ENSG0000009993	SERPIND1	-	22:20707690-20891218	-	-	IGV_LINK	3.87888	0.102321	0	1.74797	OK	4.1249	0.102707	0	1.66399	OK	NOTEST	0.00544
ENSG0000009	-	ENSG0000009994	SNAP29	-	22:20707690-20891218	-	-	IGV_LINK	642.925	17.2026	0	50.8453	OK	748.669	20.5173	0	57.6506	OK	OK	0.254214
ENSG0000009	-	ENSG0000009994	CRKL	-	22:20917425-20953749	-	-	IGV_LINK	1346.94	14.7322	6.51553	22.9489	OK	1612.84	17.6444	7.75954	27.5292	OK	OK	0.260234
ENSG0000009	-	ENSG0000009994	LZTR1	-	22:20965107-20999038	-	-	IGV_LINK	1483.67	35.7522	3.2775	68.2269	OK	1606.65	35.7406	2.8968	68.5844	OK	OK	-0.00047
ENSG0000009	-	ENSG0000009995	MMP11	-	22:23763020-23784316	-	-	IGV_LINK	184.247	6.29746	0	17.2749	OK	161.902	5.45774	0	12.8705	OK	OK	-0.20647
ENSG0000009	-	ENSG0000009995	CECR2	-	22:17359948-17558149	-	-	IGV_LINK	45.5769	0.29649	0	0.667998	OK	60.8423	0.514329	0	1.52194	OK	NOTEST	0.794709
ENSG0000009	-	ENSG0000009995	SMARCB1	-	22:23786962-23839128	-	-	IGV_LINK	904.749	35.3544	10.9816	59.7272	OK	778.575	30.9912	9.04177	52.9406	OK	OK	-0.19003
ENSG0000009	-	ENSG0000009995	P2RX6	-	22:20999103-21045017	-	-	IGV_LINK	4.8486	0.123551	0	1.12402	OK	1.03122	0.035002	0	0.738196	OK	NOTEST	-1.81959
ENSG0000009	-	ENSG0000009995	DERL3	-	22:23786962-23839128	-	-	IGV_LINK	332.614	12.3479	0	29.4846	OK	375.366	15.6012	0	35.2927	OK	OK	0.337398
ENSG0000009	-	ENSG0000009996	SLC7A4	-	22:20999103-21045017	-	-	IGV_LINK	0	0	0	0	OK	2.06245	0.054419	0	0.621547	OK	NOTEST	inf
ENSG0000009	-	ENSG0000009996	BCL2L13	-	22:17628854-17730855	-	-	IGV_LINK	1494.34	34.2271	12.2641	56.1901	OK	1897.45	44.2631	16.2543	72.272	OK	OK	0.370966
ENSG0000009	-	ENSG0000009997	DDTL	-	22:23966900-23983915	-	-	IGV_LINK	82.4262	3.38452	0	9.87834	OK	88.6853	3.64458	0	10.0471	OK	OK	0.106802
ENSG0000009	-	ENSG0000009997	DDT	-	22:23966900-23983915	-	-	IGV_LINK	475.163	50.2177	15.4394	84.996	OK	593.986	64.2395	21.7397	106.739	OK	OK	0.355264
ENSG0000009	-	ENSG0000009998	GSTT2	-	22:23966900-23983915	-	-	IGV_LINK	1.93944	0.142972	0	1.64062	OK	0	0	0	0	OK	NOTEST	'-inf
ENSG0000009	-	ENSG0000009998	OSM	-	22:30262828-30266840	-	-	IGV_LINK	34.9099	1.29091	0	2.84893	OK	78.3731	2.62851	0.163964	5.09306	OK	OK	1.02586
ENSG0000009	-	ENSG0000009999	CABIN1	-	22:24011191-24178628	-	-	IGV_LINK	1491.43	26.4639	0.085725	52.8421	OK	1424.12	22.4747	0	45.527	OK	OK	-0.23573
ENSG0000009	-	ENSG0000009999	TBC1D10A	-	22:30285116-30327046	-	-	IGV_LINK	502.875	14.3838	2.52953	26.2381	OK	781.281	24.8908	3.2353	46.5463	OK	OK	0.791167
ENSG0000009	-	ENSG0000009999	SUSD2	-	22:24181258-24189110	-	-	IGV_LINK	116.366	1.95742	0.302311	3.61253	OK	225.838	3.80872	1.04815	6.56928	OK	OK	0.960351
ENSG0000009	-	ENSG0000009999	SF3A1	-	22:30331987-30429053	-	-	IGV_LINK	1808.53	31.0155	0	74.6736	OK	2001.61	46.4051	0	119.681	OK	OK	0.581292
ENSG0000009	-	ENSG0000009999	GGT5	-	22:24219653-24245142	-	-	IGV_LINK	213.339	5.78657	0.962106	10.611	OK	159.84	4.34526	0.482967	8.20755	OK	OK	-0.41327
ENSG0000009	-	ENSG0000009999	RNF215	-	22:30331987-30429053	-	-	IGV_LINK	399.525	13.462	0	42.8635	OK	618.735	21.8562	0	69.2423	OK	OK	0.69915
ENSG0000010	-	ENSG0000010000	SEC14L2	-	22:30331987-30429053	-	-	IGV_LINK	214.648	8.27411	0	32.484	OK	395.037	16.9373	0	59.4104	OK	OK	1.03353
ENSG0000010	-	ENSG0000010001	SEC14L3	-	22:30447958-30472049	-	-	IGV_LINK	0	0	0	0	OK	4.1249	0.114964	0	0.419941	OK	NOTEST	inf
ENSG0000010	-	ENSG0000010001	SPECC1L	-	22:24270816-24528390	-	-	IGV_LINK	1506.13	14.9095	0	31.5198	OK	1601.29	15.2522	0	31.7063	OK	OK	0.032777
ENSG0000010	-	ENSG0000010002	PPIL2	-	22:21652269-21735834	-	-	IGV_LINK	1051.07	23.3602	2.41628	44.3041	OK	1211.33	27.0445	3.91876	50.1703	OK	OK	0.211285
ENSG0000010	-	ENSG0000010002	UPB1	-	22:24270816-24528390	-	-	IGV_LINK	607.045	17.2578	0	43.8895	OK	1010.6	29.677	0	70.1052	OK	OK	0.782095
ENSG0000010	-	ENSG0000010002	YPEL1	-	22:21652269-21735834	-	-	IGV_LINK	256.976	3.53825	0	8.445	OK	279.462	3.79012	0	8.87102	OK	OK	0.099207
ENSG0000010	-	ENSG0000010002	SNRPD3	-	22:24540422-24629005	-	-	IGV_LINK	872.9	67.7865	0	207.56	OK	984.107	77.0114	0	259.881	OK	OK	0.184075
ENSG0000010	-	ENSG0000010002	PES1	-	22:30576624-30627278	-	-	IGV_LINK	1680.53	53.5781	0	127.831	OK	1968.61	61.1795	0	182.738	OK	OK	0.191404
ENSG0000010	-	ENSG0000010003	MAPK1	-	22:21754499-21867680	-	-	IGV_LINK	2734.61	45.0328	0	101.491	OK	3050.36	50.9574	0	114.89	OK	OK	0.178315
ENSG0000010	-	ENSG0000010003	GGT1	-	22:24540422-24629005	-	-	IGV_LINK	3236.78	123.608	0	272.029	OK	4259.67	169.349	0	388.532	OK	OK	0.454235
ENSG0000010	-	ENSG0000010003	PRODH	-	22:18906027-18947741	-	-	IGV_LINK	268.613	7.09423	0	16.2076	OK	209.339	6.7073	0	15.6356	OK	OK	-0.08091
ENSG0000010	-	ENSG0000010003	PPM1F	-	22:21919419-21982816	-	-	IGV_LINK	944.508	12.0305	0	24.2294	OK	985.851	13.84	0	29.4488	OK	OK	0.202142
ENSG0000010	-	ENSG0000010003	SLC35E4	-	22:30635651-30669016	-	-	IGV_LINK	47.5163	1.08297	0	2.943	OK	47.4363	1.05234	0	2.96748	OK	OK	-0.04139
ENSG0000010	-	ENSG0000010003	TOP3B	-	22:21919419-21982816	-	-	IGV_LINK	552.741	17.0723	0	46.1764	OK	493.957	14.4585	0	38.7194	OK	OK	-0.23974
ENSG0000010	-	ENSG0000010005	CRYBB3	-	22:25199849-25207363	-	-	IGV_LINK	12.6064	1.02299	0	2.34926	OK	19.5933	1.5926	0	3.37422	OK	OK	0.638598
ENSG0000010	-	ENSG0000010005	CYTH4	-	22:37282026-37315345	-	-	IGV_LINK	1482.7	30.2214	9.81598	50.6269	OK	1433.4	31.3848	4.50999	58.2595	OK	OK	0.054492
ENSG0000010	-	ENSG0000010005	ESS2	-	22:19130278-19144684	-	-	IGV_LINK	384.979	11.7849	3.43638	20.1334	OK	384.647	11.9559	3.56425	20.3476	OK	OK	0.020786
ENSG0000010	-	ENSG0000010005	CRYBB2P1	-	22:25448104-25520854	-	-	IGV_LINK	326.796	25.4375	0.37797	50.497	OK	314.524	27.9945	3.26576	52.7233	OK	OK	0.138188
ENSG0000010	-	ENSG0000010006	MFNG	-	22:37469062-37486401	-	-	IGV_LINK	448.011	15.1287	4.11831	26.139	OK	435.177	16.6171	2.61985	30.6144	OK	OK	0.135384
ENSG0000010	-	ENSG0000010006	CARD10	-	22:37490361-37519542	-	-	IGV_LINK	599.287	12.1499	1.42127	22.8785	OK	622.86	14.9026	1.23658	28.5687	OK	OK	0.294622
ENSG0000010	-	ENSG0000010006	LRP5L	-	22:25351417-25405377	-	-	IGV_LINK	154.186	4.03514	0	8.09854	OK	77.3419	2.16403	0	4.57129	OK	OK	-0.8989
ENSG0000010	-	ENSG0000010007	SLC25A1	-	22:19175574-19178830	-	-	IGV_LINK	920.265	37.3111	14.2218	60.4003	OK	1494.24	60.3395	25.1396	95.5395	OK	OK	0.693501
ENSG0000010	-	ENSG0000010007	GRK3	-	22:25564848-25729294	-	-	IGV_LINK	738.927	5.79725	1.75207	9.84242	OK	653.797	5.51772	1.12296	9.91249	OK	OK	-0.07129
ENSG0000010	-	ENSG0000010007	PLA2G3	-	22:31134808-31140607	-	-	IGV_LINK	0	0	0	0	OK	0	0	0	0	OK	NOTEST	0
ENSG0000010	-	ENSG0000010007	LGALS2	-	22:37570245-37582616	-	-	IGV_LINK	859.172	115.704	48.505	182.902	OK	672.359	91.2739	37.2383	145.309	OK	OK	-0.34216
ENSG0000010	-	ENSG0000010008	GGA1	-	22:37608474-37633564	-	-	IGV_LINK	1323.67	39.7353	9.63976	69.8309	OK	1494.24	42.6412	10.5057	74.7766	OK	OK	0.101825
ENSG0000010	-	ENSG0000010008	HIRA	-	22:19330697-19479215	-	-	IGV_LINK	522.101	7.58238	0	18.5889	OK	571.299	8.29932	0	17.9801	OK	OK	0.130343
ENSG0000010	-	ENSG0000010009	SH3BP1	-	22:37634653-37666934	-	-	IGV_LINK	680.866	20.1472	0	41.2894	OK	515.134	13.8143	0.400941	27.2276	OK	OK	-0.54442
ENSG0000010	-	ENSG0000010009	SEZ6L	-	22:26169473-26383597	-	-	IGV_LINK	4.8486	0.08016	0	0.403881	OK	4.1249	0.043085	0	0.355535	OK	NOTEST	-0.89569
ENSG0000010	-	ENSG0000010009	LGALS1	-	22:37675607-37679806	-	-	IGV_LINK	2965.41	431.779	183.042	680.515	OK	4772.51	696.57	279.561	1113.58	OK	OK	0.689977
ENSG0000010	-	ENSG0000010009	HPS4	-	22:26429272-26483837	-	-	IGV_LINK	1727.07	28.3088	5.51867	51.0989	OK	1991.3	35.096	4.14541	66.0466	OK	OK	0.310056
ENSG0000010	-	ENSG0000010010	PIK3IP1	-	22:31281592-31346232	-	-	IGV_LINK	1298.46	31.9788	10.5466	53.4111	OK	1878.89	46.5728	15.1933	77.9523	OK	OK	0.54237
ENSG0000010	-	ENSG0000010010	Z83844.1	-	22:37681672-37776556	-	-	IGV_LINK	2.68028	0.031924	0	0.524171	OK	5.9848	0.071301	0	0.770459	OK	NOTEST	1.15927
ENSG0000010	-	ENSG0000010010	SRRD	-	22:26483876-26512505	-	-	IGV_LINK	249.218	11.4965	0	31.7389	OK	285.649	14.7027	0	43.2294	OK	OK	0.354884
ENSG0000010	-	ENSG0000010010	PATZ1	-	22:31281592-31346232	-	-	IGV_LINK	557.589	11.0449	0	23.1053	OK	708.452	14.4936	0	30.8741	OK	OK	0.392031
ENSG0000010	-	ENSG0000010010	TRIOBP	-	22:37681672-37776556	-	-	IGV_LINK	1683.98	35.4521	10.2153	60.6889	OK	1439.51	30.3621	8.58445	52.1397	OK	OK	-0.2236
ENSG0000010	-	ENSG0000010010	TFIP11	-	22:26483876-26512505	-	-	IGV_LINK	833.96	23.8247	2.61201	45.0373	OK	1349.87	35.3623	5.1677	65.5569	OK	OK	0.569757
ENSG0000010	-	ENSG0000010011	GCAT	-	22:37807904-37817176	-	-	IGV_LINK	38.7888	1.69895	0	3.59269	OK	177.371	7.69345	1.50357	13.8833	OK	OK	2.17898
ENSG0000010	-	ENSG0000010012	GGTLC2	-	22:22644474-22647903	-	-	IGV_LINK	0	0	0	0	OK	0	0	0	0	OK	NOTEST	0
ENSG0000010	-	ENSG0000010012	CRYBB1	-	22:26599277-26618088	-	-	IGV_LINK	9.69721	0.587957	0	1.3261	OK	26.8118	1.62766	0	3.37027	OK	OK	1.46902
ENSG0000010	-	ENSG0000010012	ANKRD54	-	22:37830854-37895563	-	-	IGV_LINK	345.22	10.9131	0	43.3513	OK	317.617	10.5604	0	39.5985	OK	OK	-0.04739
ENSG0000010	-	ENSG0000010012	EIF3L	-	22:37830854-37895563	-	-	IGV_LINK	5651.75	267.06	33.1436	500.976	OK	4058.1	170.203	32.2467	308.159	OK	OK	-0.64991
ENSG0000010	-	ENSG0000010013	SNU13	-	22:41673929-41698136	-	-	IGV_LINK	1133.6	62.7344	11.809	113.66	OK	1060.1	60.2563	5.87528	114.637	OK	OK	-0.05815
ENSG0000010	-	ENSG0000010013	MICALL1	-	22:37905656-37942822	-	-	IGV_LINK	340.372	5.75214	0.929061	10.5752	OK	412.49	7.41818	0.960367	13.876	OK	OK	0.366966
ENSG0000010	-	ENSG0000010014	POLR2F	-	22:37943049-38041915	-	-	IGV_LINK	389.338	43.8205	9.48351	78.1574	OK	451.929	55.0465	12.7462	97.3469	OK	OK	0.329048
ENSG0000010	-	ENSG0000010014	SOX10	-	22:37943049-38041915	-	-	IGV_LINK	0	0	0	0	OK	3.09367	0.064872	0	1.13691	OK	NOTEST	inf
ENSG0000010	-	ENSG0000010014	CCDC134	-	22:41800678-41826299	-	-	IGV_LINK	93.0932	4.78035	0.506627	9.05407	OK	58.7798	3.02153	0	6.13112	OK	OK	-0.66184
ENSG0000010	-	ENSG0000010015	DEPDC5	-	22:31753950-31907034	-	-	IGV_LINK	434.435	10.0038	0	33.9395	OK	412.49	8.73096	0	28.0901	OK	OK	-0.19634
ENSG0000010	-	ENSG0000010015	PICK1	-	22:38056310-38075701	-	-	IGV_LINK	738.927	29.0935	6.03746	52.1495	OK	732.17	29.7556	6.17679	53.3343	OK	OK	0.032464
ENSG0000010	-	ENSG0000010015	TTC28	-	22:27851668-28679865	-	-	IGV_LINK	1061.84	9.5905	0	33.8038	OK	948.727	8.79444	0	32.6152	OK	OK	-0.12501
ENSG0000010	-	ENSG0000010015	SLC16A8	-	22:38078133-38084093	-	-	IGV_LINK	23.2733	1.60535	0	3.73441	OK	30.9367	1.5082	0	3.4108	OK	OK	-0.09005
ENSG0000010	-	ENSG0000010016	CENPM	-	22:41938720-41947164	-	-	IGV_LINK	64.9713	4.99167	0	10.3468	OK	63.9359	4.6287	0.020866	9.23654	OK	OK	-0.10892
ENSG0000010	-	ENSG0000010016	SEPT3	-	22:41976271-41998221	-	-	IGV_LINK	49.4557	0.702887	0	1.52526	OK	31.968	0.406953	0	0.902823	OK	OK	-0.78843
ENSG0000010	-	ENSG0000010017	SLC5A1	-	22:32043031-32113029	-	-	IGV_LINK	74.6685	2.01982	0	5.31526	OK	6.18735	0.108961	0	0.448662	OK	OK	-4.21234
ENSG0000010	-	ENSG0000010018	TPTEP1	-	22:16601886-16704477	-	-	IGV_LINK	117.896	3.31572	0	6.88326	OK	84.2517	2.51969	0	5.70321	OK	OK	-0.39607
ENSG0000010	-	ENSG0000010019	SLC5A4	-	22:32205114-32273110	-	-	IGV_LINK	29.0916	0.882634	0	1.93653	OK	40.2178	1.22096	0	2.65167	OK	OK	0.468123
ENSG0000010	-	ENSG0000010019	KDELR3	-	22:38468061-38507660	-	-	IGV_LINK	128.003	5.67947	0	76.7324	OK	355.773	16.6901	0	118.487	OK	OK	1.55516
ENSG0000010	-	ENSG0000010019	CYP2D6	-	22:42085524-42139927	-	-	IGV_LINK	64.0016	1.85932	0	6.19939	OK	62.9047	1.74653	0	5.64435	OK	OK	-0.09028
ENSG0000010	-	ENSG0000010020	DDX17	-	22:38468061-38507660	-	-	IGV_LINK	26030.2	309.361	0	668.549	OK	19536.6	241.076	0	498.782	OK	OK	-0.3598
ENSG0000010	-	ENSG0000010020	DMC1	-	22:38518948-38570286	-	-	IGV_LINK	22.3036	1.66007	0	5.75401	OK	23.7182	3.3251	0	9.78601	OK	OK	1.00215
ENSG0000010	-	ENSG0000010020	TCF20	-	22:42160012-42343616	-	-	IGV_LINK	996.873	9.52448	1.99369	17.0553	OK	988.945	9.36036	2.40979	16.3109	OK	OK	-0.02508
ENSG0000010	-	ENSG0000010020	HSCB	-	22:28687742-28757515	-	-	IGV_LINK	149.337	10.2716	0.162153	20.381	OK	192.839	13.6628	0	27.3469	OK	OK	0.411596
ENSG0000010	-	ENSG0000010021	CBY1	-	22:38656635-38681820	-	-	IGV_LINK	396.616	23.8827	7.15338	40.612	OK	326.898	19.6684	5.0763	34.2606	OK	OK	-0.28008
ENSG0000010	-	ENSG0000010021	TOMM22	-	22:38681947-38685421	-	-	IGV_LINK	807.777	33.8325	11.0677	56.5973	OK	822.918	35.3007	10.7796	59.8219	OK	OK	0.061289
ENSG0000010	-	ENSG0000010021	RSPH14	-	22:23059414-23145021	-	-	IGV_LINK	114.427	8.01453	0	17.1884	OK	133.028	9.40725	0	20.9582	OK	OK	0.231154
ENSG0000010	-	ENSG0000010021	XBP1	-	22:28794554-28800597	-	-	IGV_LINK	1558.34	58.7979	22.9597	94.636	OK	2506.91	93.9696	34.0539	153.885	OK	OK	0.676429
ENSG0000010	-	ENSG0000010022	RTCB	-	22:32387581-32412255	-	-	IGV_LINK	1403.19	46.4047	17.4101	75.3994	OK	2407.91	75.9006	30.9308	120.87	OK	OK	0.70984
ENSG0000010	-	ENSG0000010022	JOSD1	-	22:38685542-38701556	-	-	IGV_LINK	1039.54	19.3195	3.69208	34.947	OK	1165.28	21.5193	4.35936	38.6793	OK	OK	0.155572
ENSG0000010	-	ENSG0000010022	FBXO7	-	22:32474675-32498829	-	-	IGV_LINK	2648.31	72.2549	28.72	115.79	OK	2468.75	69.73	26.044	113.416	OK	OK	-0.05132
ENSG0000010	-	ENSG0000010022	GTPBP1	-	22:38705722-38794198	-	-	IGV_LINK	1178.21	21.1477	0	66.1289	OK	1146.72	23.9799	0	89.488	OK	OK	0.181324
ENSG0000010	-	ENSG0000010022	POLDIP3	-	22:42583720-42614962	-	-	IGV_LINK	1985.02	36.5256	14.7308	58.3204	OK	1960.36	36.4531	14.3167	58.5895	OK	OK	-0.00287
ENSG0000010	-	ENSG0000010022	RAB36	-	22:23145325-23164350	-	-	IGV_LINK	576.984	9.30946	2.75498	15.8639	OK	826.011	13.6725	3.83679	23.5083	OK	OK	0.554513
ENSG0000010	-	ENSG0000010023	TIMP3	-	22:32512551-33058372	-	-	IGV_LINK	4013.67	51.0537	21.0855	81.0218	OK	2501.75	31.8304	13.8954	49.7653	OK	OK	-0.68161
ENSG0000010	-	ENSG0000010023	PPP6R2	-	22:50343303-50475024	-	-	IGV_LINK	1728.04	42.1077	0	91.7147	OK	1770.61	44.1014	0	94.2508	OK	OK	0.06674
ENSG0000010	-	ENSG0000010024	SBF1	-	22:50343303-50475024	-	-	IGV_LINK	2700.67	37.9648	0	92.0873	OK	2343.97	28.7337	0	65.964	OK	OK	-0.40192
ENSG0000010	-	ENSG0000010024	SUN2	-	22:38705722-38794198	-	-	IGV_LINK	3298.99	69.0753	0	140.991	OK	4013.53	80.6979	4.68333	156.712	OK	OK	0.224361
ENSG0000010	-	ENSG0000010024	CYB5R3	-	22:42617839-42649568	-	-	IGV_LINK	6032.63	190.96	72.0413	309.879	OK	12352	411.652	111.687	711.617	OK	OK	1.10815
ENSG0000010	-	ENSG0000010024	DNAL4	-	22:38705722-38794198	-	-	IGV_LINK	472.254	20.5393	0	60.8153	OK	536.237	23.8205	0	72.6558	OK	OK	0.213818
ENSG0000010	-	ENSG0000010024	C22orf31	-	22:29058671-29061844	-	-	IGV_LINK	0.969721	0.066445	0	0.260247	OK	0	0	0	0	OK	NOTEST	'-inf
ENSG0000010	-	ENSG0000010025	MIOX	-	22:50486783-50490648	-	-	IGV_LINK	517.831	30.3603	11.5396	49.181	OK	321.742	18.996	5.59483	32.3971	OK	OK	-0.67649
ENSG0000010	-	ENSG0000010025	LMF2	-	22:50502948-50507691	-	-	IGV_LINK	2231.33	59.3804	21.4663	97.2946	OK	2004.7	53.0009	18.9838	87.018	OK	OK	-0.16397
ENSG0000010	-	ENSG0000010026	RHBDD3	-	22:29259851-29300525	-	-	IGV_LINK	254.067	12.4836	0	50.5856	OK	309.367	16.4434	0	57.8045	OK	OK	0.397479
ENSG0000010	-	ENSG0000010026	PACSIN2	-	22:42796501-43015145	-	-	IGV_LINK	1300.4	26.1672	5.55716	46.7772	OK	1305.71	27.5261	3.93893	51.1133	OK	OK	0.073041
ENSG0000010	-	ENSG0000010027	TTLL1	-	22:43038584-43089428	-	-	IGV_LINK	94.0629	3.60003	0.328925	6.87114	OK	101.06	3.86267	0.481347	7.24399	OK	OK	0.101588

ENSG0000010	-	-	ENSG0000010027	'RASL10A	-	22:29312932-29319679	-	-	IGV_LINK	15.5155	1.03799	0	2.68258	OK	38.1553	2.58308	0	6.13066	OK	OK	1.3153
ENSG0000010	-	-	ENSG0000010028	'AP1B1	-	22:29327679-29423193	-	-	IGV_LINK	2227.45	34.1024	11.4338	56.7709	OK	3559.79	54.6509	16.6651	92.6368	OK	OK	0.680374
ENSG0000010	-	-	ENSG0000010028	'HMGXB4	-	22:35257451-35295807	-	-	IGV_LINK	623.53	10.7591	2.78192	18.7363	OK	571.299	9.70986	2.50085	16.9189	OK	OK	-0.14804
ENSG0000010	-	-	ENSG0000010028	'TOM1	-	22:35298837-35347994	-	-	IGV_LINK	1165.6	34.4778	10.7271	58.2285	OK	1772.68	51.5483	15.3151	87.7816	OK	OK	0.580259
ENSG0000010	-	-	ENSG0000010028	'NEFH	-	22:29480219-29491390	-	-	IGV_LINK	14.5458	0.227043	0	0.522012	OK	21.6557	0.338129	0	0.710249	OK	NOTEST	0.574605
ENSG0000010	-	-	ENSG0000010028	'CHKB	-	22:50568860-50613981	-	-	IGV_LINK	1827.51	89.9089	4.17815	175.64	OK	1403.42	68.5244	3.25636	133.792	OK	OK	-0.39185
ENSG0000010	-	-	ENSG0000010029	'BIK	-	22:43110747-43129712	-	-	IGV_LINK	0.969721	0.07002	0	0.274252	OK	3.09367	0.223714	0	0.577975	OK	NOTEST	1.67581
ENSG0000010	-	-	ENSG0000010029	'HMOX1	-	22:35380360-35394214	-	-	IGV_LINK	3683.97	128.632	52.1586	205.105	OK	6915.39	240.96	84.7273	397.193	OK	OK	0.905546
ENSG0000010	-	-	ENSG0000010029	'MCAT	-	22:43132205-43143394	-	-	IGV_LINK	270.552	12.2311	2.60998	21.8522	OK	345.46	14.3092	3.74207	24.8764	OK	OK	0.226393
ENSG0000010	-	-	ENSG0000010029	'THOC5	-	22:29505878-29581337	-	-	IGV_LINK	525.641	15.6692	0	41.2116	OK	656.89	17.5727	0	46.3961	OK	OK	0.165406
ENSG0000010	-	-	ENSG0000010029	'MCM5	-	22:35400062-35425430	-	-	IGV_LINK	1483.67	42.0304	8.62307	75.4376	OK	1362.25	44.2291	7.89247	80.5658	OK	OK	0.073565
ENSG0000010	-	-	ENSG0000010029	'APOBEC3H	-	22:39097223-39104067	-	-	IGV_LINK	119.276	8.74127	0.045013	17.4375	OK	76.3106	5.46843	0.161662	10.7752	OK	OK	-0.67672
ENSG0000010	-	-	ENSG0000010029	'ARSA	-	22:50622753-50628173	-	-	IGV_LINK	963.902	35.858	11.9543	59.7618	OK	1433.4	51.2955	17.3651	85.226	OK	OK	0.516536
ENSG0000010	-	-	ENSG0000010030	'TSPO	-	22:43151513-43163242	-	-	IGV_LINK	1937.5	156.935	69.0662	244.804	OK	1898.49	154.198	67.1732	241.222	OK	OK	-0.02539
ENSG0000010	-	-	ENSG0000010030	'RASD2	-	22:35540867-35554001	-	-	IGV_LINK	242.43	4.21742	1.25819	7.17666	OK	166.027	2.88931	0.666129	5.11249	OK	OK	-0.54564
ENSG0000010	-	-	ENSG0000010030	'TTLL12	-	22:43166621-43187133	-	-	IGV_LINK	521.71	9.90891	3.0958	16.722	OK	499.113	10.1216	2.91715	17.326	OK	OK	0.030636
ENSG0000010	-	-	ENSG0000010030	'CBX7	-	22:39120166-39152674	-	-	IGV_LINK	1984.05	35.0626	4.88821	65.2369	OK	1186.94	21.578	2.19235	40.9636	OK	OK	-0.70037
ENSG0000010	-	-	ENSG0000010031	'PDGFB	-	22:39223358-39279897	-	-	IGV_LINK	566.317	10.1196	2.91605	17.3231	OK	374.335	7.64264	1.93697	13.3483	OK	OK	-0.405
ENSG0000010	-	-	ENSG0000010031	'ACR	-	22:50738195-50745334	-	-	IGV_LINK	4.8486	0.21678	0	0.734312	OK	23.7182	1.62066	0	3.96432	OK	OK	2.90228
ENSG0000010	-	-	ENSG0000010031	'CABP7	-	22:29720083-29767011	-	-	IGV_LINK	9.69721	0.177059	0	0.87854	OK	4.1249	0.075344	0	0.48703	OK	NOTEST	-1.23267
ENSG0000010	-	-	ENSG0000010031	'RPL3	-	22:39312881-39320389	-	-	IGV_LINK	36403.3	1719.42	0	3711.39	OK	27788.4	1310.66	142.309	2479	OK	OK	-0.39163
ENSG0000010	-	-	ENSG0000010031	'ZMAT5	-	22:29720083-29767011	-	-	IGV_LINK	351.039	26.1883	8.74182	43.6348	OK	251.619	18.999	5.59105	32.407	OK	OK	-0.463
ENSG0000010	-	-	ENSG0000010032	'RBFOX2	-	22:35738735-36028425	-	-	IGV_LINK	1600.04	19.1212	4.15413	34.0883	OK	1346.78	17.1288	3.4798	30.7778	OK	OK	-0.15875
ENSG0000010	-	-	ENSG0000010032	'SYNGR1	-	22:39349924-39385588	-	-	IGV_LINK	496.497	13.4172	2.90027	23.934	OK	327.93	7.48219	1.60772	13.3567	OK	OK	-0.84255
ENSG0000010	-	-	ENSG0000010032	'TAB1	-	22:39399740-39437060	-	-	IGV_LINK	750.564	15.0096	4.53329	25.486	OK	774.45	15.5736	4.47909	26.6681	OK	OK	0.053215
ENSG0000010	-	-	ENSG0000010032	'ASCC2	-	22:29788607-29838304	-	-	IGV_LINK	1231.55	35.7569	0	73.5494	OK	1395.25	41.8487	0	99.5704	OK	OK	0.22696
ENSG0000010	-	-	ENSG0000010033	'MTMR3	-	22:29883154-30177075	-	-	IGV_LINK	517.831	26.9423	0	70.5348	OK	453.739	19.8253	0	55.2342	OK	OK	-0.44253
ENSG0000010	-	-	ENSG0000010033	'MIEF1	-	22:39499431-39518132	-	-	IGV_LINK	858.203	10.7573	2.5227	18.9919	OK	1262.22	16.11	4.66307	27.557	OK	OK	0.582639
ENSG0000010	-	-	ENSG0000010033	'APOL4	-	22:36189123-36204840	-	-	IGV_LINK	153.216	3.18228	0	6.50772	OK	42.2802	1.21945	0	3.18158	OK	OK	-1.38384
ENSG0000010	-	-	ENSG0000010034	'PNPLA5	-	22:43879677-43892013	-	-	IGV_LINK	0	0	0	0	OK	0	0	0	0	OK	NOTEST	0
ENSG0000010	-	-	ENSG0000010034	'APOL1	-	22:36253009-36267530	-	-	IGV_LINK	2494.12	62.9697	22.1282	103.811	OK	14327.8	374.239	17.9117	730.566	OK	OK	2.57123
ENSG0000010	-	-	ENSG0000010034	'PNPLA3	-	22:43923738-44172949	-	-	IGV_LINK	3.87888	0.277764	0	4.43678	OK	54.6976	1.48953	0	6.45981	OK	OK	2.42293
ENSG0000010	-	-	ENSG0000010034	'MYH9	-	22:36281280-36388018	-	-	IGV_LINK	14108.5	116.927	3.65643	230.197	OK	16497.5	134.188	1.37591	267	OK	OK	0.198648
ENSG0000010	-	-	ENSG0000010034	'CACNA1I	-	22:39570752-39689737	-	-	IGV_LINK	2.90916	0.018914	0	0.690785	OK	29.9055	0.173418	0	0.866164	OK	NOTEST	3.19672
ENSG0000010	-	-	ENSG0000010034	'SAMM50	-	22:43923738-44172949	-	-	IGV_LINK	828.141	29.5908	6.02684	53.1547	OK	945.591	34.2362	2.95688	65.5155	OK	OK	0.210377
ENSG0000010	-	-	ENSG0000010034	'TXN2	-	22:36467035-36482030	-	-	IGV_LINK	1289.73	65.1365	26.6809	103.592	OK	1309.66	73.0671	28.6265	117.508	OK	OK	0.165755
ENSG0000010	-	-	ENSG0000010035	'FOXRED2	-	22:36487189-36507101	-	-	IGV_LINK	105.7	1.56699	0	3.67837	OK	232.026	3.58514	0.300055	6.87023	OK	OK	1.19403
ENSG0000010	-	-	ENSG0000010035	'GRAP2	-	22:39901081-39973721	-	-	IGV_LINK	205.581	5.75659	0	12.7919	OK	126.841	2.80898	0	6.23696	OK	OK	-1.03516
ENSG0000010	-	-	ENSG0000010035	'EIF3D	-	22:36510849-36529436	-	-	IGV_LINK	3898.28	132.549	49.0099	216.088	OK	3395.82	115.943	42.1342	189.752	OK	OK	-0.19311
ENSG0000010	-	-	ENSG0000010035	'TNRC6B	-	22:40044816-40335808	-	-	IGV_LINK	876.627	3.81168	0	11.5315	OK	769.294	4.18013	0	12.885	OK	OK	0.133123
ENSG0000010	-	-	ENSG0000010035	'SGSM3	-	22:40346499-40636702	-	-	IGV_LINK	1223.43	44.122	0	103.075	OK	1236.5	38.7455	0	83.9112	OK	OK	-0.18747
ENSG0000010	-	-	ENSG0000010036	'IFT27	-	22:36758201-36776256	-	-	IGV_LINK	495.527	19.0192	0	38.4033	OK	447.552	19.6254	0.82282	38.428	OK	OK	0.045269
ENSG0000010	-	-	ENSG0000010036	'PVALB	-	22:36800683-36819736	-	-	IGV_LINK	0	0	0	0	OK	11.3435	1.61707	0	3.85553	OK	OK	inf
ENSG0000010	-	-	ENSG0000010036	'KIAA0930	-	22:45190337-45240769	-	-	IGV_LINK	1315.91	26.1225	8.11533	44.1297	OK	1737.61	33.1122	12.8718	53.3527	OK	OK	0.34207
ENSG0000010	-	-	ENSG0000010036	'NCF4	-	22:36847371-36878015	-	-	IGV_LINK	360.736	14.8502	4.77513	24.9253	OK	803.324	33.5305	13.5873	53.4737	OK	OK	1.17499
ENSG0000010	-	-	ENSG0000010036	'CSF2RB	-	22:36913627-36940449	-	-	IGV_LINK	367.524	4.8419	1.5486	8.13519	OK	813.637	10.9053	3.56531	18.2453	OK	OK	1.17138
ENSG0000010	-	-	ENSG0000010037	'SLC25A17	-	22:40769629-40819399	-	-	IGV_LINK	322.917	12.4371	2.60623	22.2679	OK	438.271	16.7999	4.49231	29.1075	OK	OK	0.433804
ENSG0000010	-	-	ENSG0000010037	'UPK3A	-	22:45284981-45295874	-	-	IGV_LINK	0.969721	0.062209	0	0.317817	OK	5.15612	0.331213	0	0.870966	OK	NOTEST	2.41256
ENSG0000010	-	-	ENSG0000010037	'FAM118A	-	22:45308967-45341955	-	-	IGV_LINK	254.067	8.79359	0.060475	17.5267	OK	253.681	8.04377	0.288832	15.7987	OK	OK	-0.12858
ENSG0000010	-	-	ENSG0000010037	'KCTD17	-	22:37051735-37063390	-	-	IGV_LINK	220.127	10.4097	2.19117	18.6282	OK	325.867	16.2949	3.91624	28.6735	OK	OK	0.64649
ENSG0000010	-	-	ENSG0000010038	'ST13	-	22:40824534-40857022	-	-	IGV_LINK	5614.68	244.383	57.558	431.208	OK	7568.16	335.829	70.0561	601.601	OK	OK	0.458581
ENSG0000010	-	-	ENSG0000010038	'IL2RB	-	22:37125837-37199385	-	-	IGV_LINK	1113.24	16.0789	4.7193	27.4386	OK	787.856	11.2717	2.72442	19.8189	OK	OK	-0.51247
ENSG0000010	-	-	ENSG0000010038	'RBX1	-	22:40951346-40973309	-	-	IGV_LINK	719.533	39.6877	15.984	63.3915	OK	658.953	36.1253	14.4829	57.7676	OK	OK	-0.13568
ENSG0000010	-	-	ENSG0000010039	'EP300	-	22:41091785-41197456	-	-	IGV_LINK	1974.35	21.5469	0	53.3989	OK	2125.35	19.9168	0	49.6258	OK	OK	-0.11349
ENSG0000010	-	-	ENSG0000010039	'L3MBTL2	-	22:41205204-41240934	-	-	IGV_LINK	800.019	14.7069	5.05394	24.3599	OK	922.946	17.3666	5.70324	29.0299	OK	OK	0.239818
ENSG0000010	-	-	ENSG0000010039	'CHADL	-	22:41205204-41240934	-	-	IGV_LINK	18.4247	1.02591	0	5.79662	OK	19.5933	0.81051	0	4.84451	OK	OK	-0.34
ENSG0000010	-	-	ENSG0000010040	'RANGAP1	-	22:41245610-41286251	-	-	IGV_LINK	911.537	18.141	6.51849	29.7634	OK	865.198	16.8621	6.49353	27.2307	OK	OK	-0.10547
ENSG0000010	-	-	ENSG0000010040	'ZC3H7B	-	22:41301521-41360147	-	-	IGV_LINK	2189.63	21.5904	9.55616	33.6246	OK	2119.17	20.9117	9.26265	32.5607	OK	OK	-0.04608
ENSG0000010	-	-	ENSG0000010041	'PHF5A	-	22:41459716-41468725	-	-	IGV_LINK	338.432	20.8791	6.80551	34.9527	OK	374.335	23.1232	8.1285	38.1178	OK	OK	0.147279
ENSG0000010	-	-	ENSG0000010041	'ACO2	-	22:41469124-41544606	-	-	IGV_LINK	2307.93	54.4049	10.109	98.7008	OK	3131.83	72.6774	16.5584	128.796	OK	OK	0.41777
ENSG0000010	-	-	ENSG0000010041	'POLR3H	-	22:41469124-41544606	-	-	IGV_LINK	943.538	15.8901	0	37.3018	OK	1407.62	26.9851	0	64.5721	OK	OK	0.764039
ENSG0000010	-	-	ENSG0000010041	'TRMU	-	22:46330874-46357340	-	-	IGV_LINK	800.019	28.8605	7.58336	50.1376	OK	756.919	28.3135	6.2423	50.3848	OK	OK	-0.0276
ENSG0000010	-	-	ENSG0000010041	'PMM1	-	22:41560762-41589890	-	-	IGV_LINK	1100.63	53.1098	22.7233	83.4962	OK	1341.62	66.5939	28.9014	104.286	OK	OK	0.326412
ENSG0000010	-	-	ENSG0000010041	'DESI1	-	22:41598027-41621096	-	-	IGV_LINK	927.053	18.402	2.27355	34.5305	OK	1049.79	20.8389	2.21668	39.4611	OK	OK	0.179412
ENSG0000010	-	-	ENSG0000010042	'CERK	-	22:46684410-46738261	-	-	IGV_LINK	927.053	12.802	3.26466	22.3394	OK	853.854	12.5096	1.3569	23.6622	OK	OK	-0.03334
ENSG0000010	-	-	ENSG0000010042	'BRD1	-	22:49773282-49827512	-	-	IGV_LINK	625.47	11.2778	0	24.6022	OK	568.205	10.9333	0	24.3941	OK	OK	-0.04475
ENSG0000010	-	-	ENSG0000010042	'ZBED4	-	22:49853841-49890078	-	-	IGV_LINK	314.189	2.63467	0.882901	4.38644	OK	337.211	2.8282	0.97165	4.68476	OK	OK	0.102262
ENSG0000010	-	-	ENSG0000010042	'MLC1	-	22:50059390-50085902	-	-	IGV_LINK	34.9099	0.586516	0	2.31522	OK	11.3435	0.190834	0	0.800614	OK	OK	-1.61985
ENSG0000010	-	-	ENSG0000010042	'HDAC10	-	22:50245182-50261825	-	-	IGV_LINK	883.297	38.094	9.19395	66.9941	OK	1044.33	45.4269	9.58954	81.2642	OK	OK	0.253981
ENSG0000010	-	-	ENSG0000010043	'KCNK10	-	14:88175390-88326907	-	-	IGV_LINK	16.4852	0.294471	0	0.776469	OK	4.1249	0.031731	0	0.252227	OK	NOTEST	-3.21418
ENSG0000010	-	-	ENSG0000010043	'ABHD4	-	14:22595807-22613215	-	-	IGV_LINK	571.165	12.1779	2.51435	21.8414	OK	910.572	18.6103	6.13559	31.085	OK	OK	0.61184
ENSG0000010	-	-	ENSG0000010044	'KHNYN	-	14:24426531-24442905	-	-	IGV_LINK	1313.97	26.908	0	59.0698	OK	1371.53	26.8371	0	55.7433	OK	OK	-0.00381
ENSG0000010	-	-	ENSG0000010044	'FKBP3	-	14:45084098-45135319	-	-	IGV_LINK	420.859	21.8757	2.71673	41.0347	OK	365.054	18.281	1.88742	34.6746	OK	OK	-0.25898
ENSG0000010	-	-	ENSG0000010044	'SDR39U1	-	14:24426531-24442905	-	-	IGV_LINK	886.325	48.644	0	102.967	OK	805.387	45.6513	0	97.7431	OK	OK	-0.09161
ENSG0000010	-	-	ENSG0000010044	'CTSG	-	14:24573521-24576260	-	-	IGV_LINK	0	0	0	0	OK	0	0	0	0	OK	NOTEST	0
ENSG0000010	-	-	ENSG0000010045	'GZMH	-	14:24595722-24657774	-	-	IGV_LINK	192.005	16.1242	2.04754	30.2009	OK	95.9039	8.13123	0	17.9166	OK	OK	-0.98768
ENSG0000010	-	-	ENSG0000010045	'GZMB	-	14:24595722-24657774	-	-	IGV_LINK	106.669	7.80101	0	17.809	OK	107.247	8.25797	0	17.5976	OK	OK	0.082126
ENSG0000010	-	-	ENSG0000010046	'RBM23	-	14:22893205-22929585	-	-	IGV_LINK	1109.36	33.3435	0	68.9495	OK	1087.94	33.4239	0	68.3778	OK	OK	0.003474
ENSG0000010	-	-	ENSG0000010046	'PRMT5	-	14:22893205-22929585	-	-	IGV_LINK	635.167	37.4065	0	91.6213	OK	654.828	25.9346	0	64.4977	OK	OK	-0.52841
ENSG0000010	-	-	ENSG0000010047	'COCH	-	14:30874513-31096450	-	-	IGV_LINK	4.8486	0.12889	0	1.04034	OK	11.3435	0.751278	0	4.52449	OK	OK	2.54321
ENSG0000010	-	-	ENSG0000010047	'AP4S1	-	14:30874513-31096450	-	-	IGV_LINK	99.8812	2.50534	0	6.7851	OK	126.841	3.48958	0	9.39428	OK	OK	0.478043
ENSG0000010	-	-	ENSG0000010047	'POLE2	-	14:49643554-49688422	-	-	IGV_LINK	28.1219	1.98462	0	5.67883	OK	27.8431	2.10886	0	5.57128	OK	OK	0.087604
ENSG0000010	-	-	ENSG0000010048	'VCPKMT	-	14:50108631-50116600	-	-	IGV_LINK	71.7593	2.75516	0	6.02566	OK	79.4043	2.94949	0.100971	5.798	OK	OK	0.09833
ENSG0000010	-	-	ENSG0000010048	'SOS2	-	14:50117119-50231558	-	-	IGV_LINK	523.649	6.53623	2.29846	10.774	OK	500.144	6.05429	2.03642	10.0722	OK	OK	-0.1105
ENSG0000010	-	-	ENSG0000010049	'CDKL1	-	14:50237562-50416461	-	-	IGV_LINK	130.912	8.47673	0	37.7592	OK	137.153	10.5043	0	47.702	OK	OK	0.309402
ENSG0000010	-	-	ENSG0000010050	'NIN	-	14:50719762-50831121	-	-	IGV_LINK	1000.75	9.23961	0	19.0887	OK	699.171	5.82846	0	12.031	OK	OK	-0.66472
ENSG0000010	-	-	ENSG0000010050	'PYGL	-	14:50848121-50944736	-	-	IGV_LINK	693.35	17.5285	4.42813	30.6288	OK	1442.68	34.7038	11.6836	57.724	OK	OK	0.985395
ENSG0000010	-	-	ENSG0000010050	'TRIM9	-	14:50975261-51096061	-	-	IGV_LINK	204.611	4.23762	0	10.2281	OK	325.867	6.37644	0	14.339	OK	OK	0.589497
ENSG0000010	-	-	ENSG0000010051	'PSMC6	-	14:52707171-52728587	-	-	IGV_LINK	871.779	41.0176	14.8352	67.1999	OK	746.607	35.1923	12.6166	57.7679	OK	OK	-0.22098
ENSG0000010	-	-	ENSG0000010052	'GNPNAT1	-	14:52775193-52791668	-	-	IGV_LINK	121.215	3.31757	0	7.62383	OK	142.309	4.02766	0	9.08629	OK	OK	0.279814
ENSG0000010	-	-	ENSG0000010052	'DDHD1	-	14:53036744-53153282	-	-	IGV_LINK	524.619	2.82802	0	6.09836	OK	346.492	2.02472	0	4.94253	OK	OK	-0.48207
ENSG0000010	-	-	ENSG0000010052	'CDKN3	-	14:54396848-54420218	-	-	IGV_LINK	42.6677	3.56985	0	7.66519	OK	30.9367	3.07275	0	6.79867	OK	OK	-0.21633
ENSG0000010	-	-	ENSG0000010052	'CNIH1	-	14:54423559-54441431	-	-	IGV_LINK	531.407	39.0777	9.16242	68.993	OK	545.518	41.8365	5.76769	77.9054	OK	OK	0.098417
ENSG0000010	-	-	ENSG0000010053	'CGRRF1	-	14:54509811-54539309	-	-	IGV_LINK	96.0023	5.68762	0	11.8945	OK	82.498	3.44031	0	7.35613	OK	OK	-0.72529
ENSG0000010	-	-	ENSG0000010055	'ATP6V1D	-	14:67241108-67386516	-	-	IGV_LINK	727.29	33.9189	0.927069	66.9107	OK	817.761	38.2532	2.67824	73.8282	OK	OK	0.173493
ENSG0000010	-	-	ENSG0000010055	'C14orf105	-	14:57469300-57493867	-	-	IGV_LINK	1026.93	51.7977	1.55731	102.038	OK	625.954	26.5171	0.998789	52.0354	OK	OK	-0.96597
ENSG0000010	-	-	ENSG0000010055	'PLEK2	-	14:67386982-67412200	-	-	IGV_LINK	184.247	8.0589	1.60502	14.5128	OK	178.402	8.24102	1.51648	14.9656	OK	OK	0.032239
ENSG0000010	-	-	ENSG0000010056	'PIGH	-	14:67447083-67600287	-	-	IGV_LINK	199.762	12.0711	0	28.2966	OK	168.09	13.5399	0	31.2504	OK	OK	0.165663
ENSG0000010	-	-	ENSG0000010056	'LRRC74A	-	14:76826371-76870302	-	-	IGV_LINK	0	0	0	0	OK	0	0	0	0	OK	NOTEST	0
ENSG0000010	-	-	ENSG0000010056	'PSMA3	-	14:57999734-58298139	-	-	IGV_LINK	704.017	54.4994	0	121.141	OK	614.61	49.891	0	104.289	OK	OK	-0.12746
ENSG0000010	-	-	ENSG0000010056	'VTI1B	-	14:67619797-67695814	-	-	IGV_LINK	1054.09	34.7841	0	72.9677	OK	2055.23	35.0874	0	85.0635	OK	OK	0.012524
ENSG0000010	-	-	ENSG0000010057	'TIMM9	-	14:58395915-58551289	-	-	IGV_LINK	231.763	22.5776	0.616224	44.5391	OK	243.369	23.9346	1.49177	46.3775	OK	OK	0.084204
ENSG0000010	-	-	ENSG0000010057	'GSTZ1	-	14:77274955-77331597	-	-	IGV_LINK	119.276	6.71676	0	15.9842	OK	203.151	11.9278	0	25.3942	OK	OK	0.828494
ENSG0000010	-	-	ENSG0000010057	'KIAA0586	-	14:58395915-58551289	-	-	IGV_LINK	219.157	3.76478	0	10.3701	OK	184.589	4.78495	0	13.1936	OK	OK	0.345935
ENSG0000010	-	-	ENSG0000010058	'TMED8	-	14:77335020-77391497	-	-	IGV_LINK	609.954	4.5161	1.62845	7.40375	OK	506.331	3.74944	1.20794	6.29094	OK	OK	-0.2684
ENSG0000010	-	-	ENSG0000010058	'SAMD15	-	14:77335020-77391497	-	-	IGV_LINK	68.8502	1.62247	0	4.6694	OK	93.8415	2.21257	0	6.65466	OK	OK	0.447538
ENSG0000010	-	-	ENSG0000010059	'AHSA1	-	14:77426674-77469474	-	-	IGV_LINK	658.44	39.38	4.28473	74.4753	OK	682.671	44.8605	4.46482	85.2562	OK	OK	0.187983
ENSG0000010	-	-	ENSG0000010059	'DAAM1	-	14:59188645-59371405	-	-	IGV_LINK	138.67	3.70742	0	8.42122	OK	104.154	3.02201	0	7.84308	OK	OK	-0.29491
ENSG0000010	-	-	ENSG0000010059	'ISM2	-	14:77474393-77498850	-	-	IGV_LINK	0	0	0	0	OK	0	0	0	0	OK	NOTEST	0
ENSG0000010	-	-	ENSG0000010059	'SPTLC2	-	14:77505996-77616773	-	-	IGV_LINK	951.296	6.70346	2.8122	10.5947	OK	830.136	5.85064	2.39951	9.30176	OK	OK	-0.19631
ENSG0000010	-	-	ENSG0000010059	'RIN3	-	14:92513773-92688994	-	-	IGV_LINK	765.109	13.0512	3.72129	22.381	OK	720.826	12.3269	1.82268	22.8311	OK	OK	-0.08237
ENSG0000010	-	-	ENSG0000010060	'LGMN	-	14:92703806-92748702	-	-	IGV_LINK	3789.67	136.582	41.6459	231.517	OK	7416.57	253.702	67.3623	440.041	OK	OK	0.893368
ENSG0000010	-	-	ENSG0000010060	'ALKBH1	-	14:77672403-77708020	-	-	IGV_LINK	160.974	4.9691	0.599609	9.33858	OK	145.403	4.5297	0.771267	8.28814	OK	OK	-0.13357
ENSG0000010	-	-	ENSG0000010060	'SNW1	-	14:77708070-77769742	-	-	IGV_LINK	781.595	24.1168	7.03507	41.1985	OK	838.386	25.1563	8.33518	41.9774	OK	OK	0.060883
ENSG0000010	-	-	ENSG0000010060	'CHGA	-	14:92923079-92935293	-	-	IGV_LINK	0.969721	0.076204	0	0.439464	OK	0	0	0	0	OK	NOTEST	'-inf
ENSG0000010	-	-	ENSG0000010060	'ITPK1	-	14:92936913-93116320	-	-	IGV_LINK	919.295	20.972	4.83066	37.1134	OK	942.54	25.0224	4.09902	45.9458	OK	OK	0.254752
ENSG0000010	-	-	ENSG0000010061	'DHRS7	-	14:60091910-60169856	-	-	IGV_LINK	629.349	31.2214	5.33969	57.1031	OK	885.822	44.5165	10.8524	78.1806	OK	OK	0.511804
ENSG0000010	-	-	ENSG0000010061	'PPM1A	-	14:60240120-60299087	-	-	IGV_LINK	580.863	14.1894	3.52942	24.8494	OK	647.609	18.1351	5.41005	30.8601	OK	OK	0.353968
ENSG0000010	-	-	ENSG0000010062	'SIX4	-	14:60709527-60724348	-	-	IGV_LINK	0.969721	0.008941	0	0.106197	OK	4.1249	0.038043	0	0.150389	OK	NOTEST	2.08918
ENSG0000010	-	-	ENSG0000010062	'GALNT16	-	14:69183019-69359308	-	-	IGV_LINK	107.639	1.4526	0	3.7103	OK	30.9367	0.649108	0	2.79698	OK	OK	-1.1621
ENSG0000010	-	-	ENSG0000010062	'ASB2	-	14:93934152-93976791	-	-	IGV_LINK	199.762	5.86103	0	11.7474	OK	79.4043	2.26037	0	5.36241	OK	OK	-1.3746
ENSG0000010	-	-	ENSG0000010062	'CEP128	-	14:80476982-81170414	-	-	IGV_LINK	95.0326	1.78179	0	4.2294	OK	89.7166	2.51363	0	7.00118	OK	OK	0.496448
ENSG0000010	-	-	ENSG0000010063	'ERH	-	14:69380122-69462388	-	-	IGV_LINK	535.286	32.4848	0	65.0841	OK	483.645	28.6809	0	57.7066	OK	OK	-0.17968
ENSG0000010	-	-	ENSG0000010064	'HIF1A	-	14:61681040-61796428	-	-	IGV_LINK	1278.09	30.392	1.82941	58.9547	OK	2683.25	69.7191	4.83037	134.608	OK	OK	1.19786
ENSG0000010	-	-	ENSG0000010064	'SUSD6	-	14:69611595-69715142	-	-	IGV_LINK	1993.75	23.6701	4.56208	42.7782	OK	2257.35	26.9013	6.07362	47.7289	OK	OK	0.184606
ENSG0000010	-	-	ENSG0000010065	'SRSF5	-	14:69726899-69772005	-	-	IGV_LINK	4918.42	239.629	55.0646	424.194	OK	4090.87	193.938	48.7929	339.084	OK	OK	-0.30521
ENSG0000010	-	-	ENSG0000010065	'SLC10A1	-	14:69775416-69797289	-	-	IGV_LINK	0.969721	0.029123	0	0.114068	OK	0	0	0	0	OK	NOTEST	'-inf
ENSG0000010	-	-	ENSG0000010066	'EIF5	-	14:103333543-103345025	-	-	IGV_LINK	2160.54	59.7752	4.09132	115.459	OK	2295.51	68.2474	4.3199	132.175	OK	OK	0.191227
ENSG0000010	-	-	ENSG0000010066	'SERPINA4	-	14:94561090-94624646	-	-	IGV_LINK	1.93944	0.067825	0	0.460904	OK	4.1249	0.144356	0	1.2341	OK	NOTEST	1.08975
ENSG0000010	-	-	ENSG0000010067	'SLC8A3	-	14:70044216-70230187	-	-	IGV_LINK	2.90916	0.044623	0	0.298759	OK	3.09367	0.055622	0	0.314056	OK	NOTEST	0.31787
ENSG0000010	-	-	ENSG0000010069	'DICER1	-	14:95086227-95179933	-	-	IGV_LINK	848.505	9.42992	0	22.3712	OK	551.705	7.02105	0	16.6082	OK	OK	-0.42556
ENSG0000010	-	-	ENSG0000010071	'ZFYVE21	-	14:103715729-103733668	-	-	IGV_LINK	411.161	17.6354	5.18165	30.0892	OK	394.959	17.0309	5.24697	28.8149	OK	OK	-0.05032

ENSG0000010	-	-	ENSG0000010071	MTHFD1	-	14:64388030-64546173	-	-	IGV_LINK	1164.63	28.8429	0	62.6009	OK	1135.38	26.7353	0	55.9087	OK	OK	-0.10947
ENSG0000010	-	-	ENSG0000010072	TCL1A	-	14:95709966-95757656	-	-	IGV_LINK	12.6064	0.619217	0	1.54449	OK	8.2498	0.618914	0	1.74465	OK	OK	-0.00071
ENSG0000010	-	-	ENSG0000010072	ZC3H14	-	14:88562908-88792752	-	-	IGV_LINK	574.075	15.2872	2.54249	28.0319	OK	688.858	20.2945	3.19763	37.3913	OK	OK	0.408764
ENSG0000010	-	-	ENSG0000010072	TELO2	-	16:1493343-1612110	-	-	IGV_LINK	821.353	30.1997	0	68.9219	OK	781.669	26.8594	0	61.135	OK	OK	-0.16911
ENSG0000010	-	-	ENSG0000010073	PCNX1	-	14:70907404-71115382	-	-	IGV_LINK	778.686	8.79732	0	23.5312	OK	900.259	8.65617	0	19.4507	OK	OK	-0.02334
ENSG0000010	-	-	ENSG0000010073	BDKRB1	-	14:96204678-96268967	-	-	IGV_LINK	1.93944	0.080753	0	0.523776	OK	20.0937	1.13466	0	4.56571	OK	OK	3.81261
ENSG0000010	-	-	ENSG0000010074	GSKIP	-	14:96279201-96387288	-	-	IGV_LINK	108.609	5.49209	0	18.8768	OK	148.496	7.13309	0	19.8779	OK	OK	0.377171
ENSG0000010	-	-	ENSG0000010074	VRK1	-	14:96797303-96945394	-	-	IGV_LINK	109.578	3.92732	0.216496	7.63815	OK	84.5604	3.33489	0	6.94323	OK	OK	-0.23591
ENSG0000010	-	-	ENSG0000010076	PSMC1	-	14:90256494-90332137	-	-	IGV_LINK	744.745	59.3217	4.13753	114.506	OK	783.731	66.2257	6.6352	125.816	OK	OK	0.158831
ENSG0000010	-	-	ENSG0000010076	PAPLN	-	14:73237496-73274640	-	-	IGV_LINK	1853.14	26.6671	6.66702	46.6672	OK	1897.45	28.8739	7.90704	49.8408	OK	OK	0.114705
ENSG0000010	-	-	ENSG0000010078	RPS6KA5	-	14:90847861-91225632	-	-	IGV_LINK	213.339	2.70872	0	7.59394	OK	176.339	2.76191	0	14.2239	OK	OK	0.028055
ENSG0000010	-	-	ENSG0000010079	PPP4R3A	-	14:91457610-91510554	-	-	IGV_LINK	986.206	38.4486	6.20641	70.6907	OK	996.163	35.6716	4.4838	66.8593	OK	OK	-0.10816
ENSG0000010	-	-	ENSG0000010080	C14orf93	-	14:22982697-23010166	-	-	IGV_LINK	213.339	6.32146	1.1428	11.5001	OK	155.715	4.90819	0.266965	9.54942	OK	OK	-0.36507
ENSG0000010	-	-	ENSG0000010080	PSMB5	-	14:23016542-23035230	-	-	IGV_LINK	754.443	43.5285	16.8045	70.2526	OK	1048.76	59.8163	24.4261	95.2065	OK	OK	0.458577
ENSG0000010	-	-	ENSG0000010081	YY1	-	14:100207406-100306547	-	-	IGV_LINK	1394.46	21.8179	0	55.6792	OK	1212.72	20.629	0	44.7715	OK	OK	-0.08084
ENSG0000010	-	-	ENSG0000010081	ACIN1	-	14:23058563-23104992	-	-	IGV_LINK	3194.26	85.3386	4.77979	165.897	OK	3270.01	88.954	4.86115	173.047	OK	OK	0.059861
ENSG0000010	-	-	ENSG0000010081	CCNB1IP1	-	14:20311367-20333312	-	-	IGV_LINK	578.923	28.0245	8.87811	47.1708	OK	286.681	12.5111	3.96185	21.0603	OK	OK	-1.16348
ENSG0000010	-	-	ENSG0000010081	TRIP11	-	14:91965990-92106621	-	-	IGV_LINK	416.035	3.05962	0	6.23751	OK	478.264	4.25497	0	9.83469	OK	OK	0.475796
ENSG0000010	-	-	ENSG0000010082	APEX1	-	14:20455190-20461612	-	-	IGV_LINK	2068.41	92.6632	27.4891	157.837	OK	1819.08	80.623	26.0437	135.202	OK	OK	-0.2008
ENSG0000010	-	-	ENSG0000010083	PABPN1	-	14:23185315-23326185	-	-	IGV_LINK	2139.13	71.3	1.97362	140.626	OK	1977.99	63.7138	1.46439	125.963	OK	OK	-0.1623
ENSG0000010	-	-	ENSG0000010084	EFS	-	14:23356401-23365752	-	-	IGV_LINK	380.13	7.41392	2.61503	12.2128	OK	379.491	7.52862	2.28042	12.7768	OK	OK	0.022147
ENSG0000010	-	-	ENSG0000010085	ARHGAP5	-	14:32074945-32159728	-	-	IGV_LINK	576.984	8.19263	0	16.8375	OK	487.769	9.08965	0	19.4331	OK	OK	0.149899
ENSG0000010	-	-	ENSG0000010086	CINP	-	14:102317376-102362916	-	-	IGV_LINK	357.827	15.3816	0.958374	29.8049	OK	365.054	16.9533	2.30866	31.5979	OK	OK	0.140355
ENSG0000010	-	-	ENSG0000010086	DHRS2	-	14:23630114-23645639	-	-	IGV_LINK	0	0	0	0	OK	4.1249	0.151804	0	0.551599	OK	NOTEST	inf
ENSG0000010	-	-	ENSG0000010088	SRP54	-	14:34920857-35029567	-	-	IGV_LINK	533.346	20.8128	0	66.9446	OK	681.64	20.8883	5.20967	36.5669	OK	OK	0.005223
ENSG0000010	-	-	ENSG0000010088	CPNE6	-	14:24070836-24078100	-	-	IGV_LINK	0.969721	0.026509	0	0.756865	OK	1.03122	0.028206	0	0.748295	OK	NOTEST	0.089531
ENSG0000010	-	-	ENSG0000010088	CHD8	-	14:21385193-21456126	-	-	IGV_LINK	1095.78	12.3405	0	24.8922	OK	1012.66	12.6182	0	29.961	OK	OK	0.032108
ENSG0000010	-	-	ENSG0000010088	PCK2	-	14:24080106-24132849	-	-	IGV_LINK	512.982	17.6615	0	41.8893	OK	443.427	14.5003	0	33.615	OK	OK	-0.28453
ENSG0000010	-	-	ENSG0000010089	KIAA0391	-	14:35044906-35317493	-	-	IGV_LINK	462.682	13.5866	0	36.0751	OK	480.555	14.4053	0	37.2728	OK	OK	0.084415
ENSG0000010	-	-	ENSG0000010089	DCAF11	-	14:24080106-24132849	-	-	IGV_LINK	1341.12	32.6844	3.72301	61.6458	OK	1069.61	26.7782	0.567936	52.9884	OK	OK	-0.28755
ENSG0000010	-	-	ENSG0000010090	PSMA6	-	14:35044906-35317493	-	-	IGV_LINK	1278.65	82.7819	17.023	148.541	OK	1159.71	73.4502	7.89699	139.003	OK	OK	-0.17255
ENSG0000010	-	-	ENSG0000010090	NFKBIA	-	14:35401510-35404749	-	-	IGV_LINK	1947.2	91.5678	34.0455	149.09	OK	1571.59	79.1499	25.0728	133.227	OK	OK	-0.21025
ENSG0000010	-	-	ENSG0000010090	EMC9	-	14:24136157-24141588	-	-	IGV_LINK	134.791	13.456	0	56.8194	OK	117.56	10.5183	0	38.4237	OK	OK	-0.35535
ENSG0000010	-	-	ENSG0000010091	PSME2	-	14:24143361-24167402	-	-	IGV_LINK	2889.77	261.812	25.1856	498.438	OK	2001.61	184.995	1.30504	368.684	OK	OK	-0.50105
ENSG0000010	-	-	ENSG0000010091	BRMS1L	-	14:35819223-36067190	-	-	IGV_LINK	52.3649	1.63371	0	3.85876	OK	57.7486	2.12485	0	4.88926	OK	OK	0.379213
ENSG0000010	-	-	ENSG0000010091	REC8	-	14:24171852-24239242	-	-	IGV_LINK	347.16	15.6675	0	64.4074	OK	307.305	16.5966	0	76.0853	OK	OK	0.083114
ENSG0000010	-	-	ENSG0000010092	TM9SF1	-	14:24171852-24239242	-	-	IGV_LINK	691.725	21.8876	0	58.9597	OK	973.223	32.6918	0	84.5123	OK	OK	0.578811
ENSG0000010	-	-	ENSG0000010093	SEC23A	-	14:39031918-39109646	-	-	IGV_LINK	462.557	12.5215	0.873833	24.1692	OK	745.576	20.1051	2.08431	38.1259	OK	OK	0.683151
ENSG0000010	-	-	ENSG0000010093	GMPR2	-	14:24171852-24239242	-	-	IGV_LINK	1010.45	51.2334	0	156.776	OK	1274.59	63.6049	0	173.853	OK	OK	0.312051
ENSG0000010	-	-	ENSG0000010094	PNN	-	14:39174884-39183218	-	-	IGV_LINK	2262.36	41.4684	12.2329	70.7039	OK	1905.7	34.1421	11.3851	56.899	OK	OK	-0.28046
ENSG0000010	-	-	ENSG0000010094	RABGGTA	-	14:24265537-24271739	-	-	IGV_LINK	481.951	23.1718	5.56725	40.7763	OK	385.678	18.7761	3.5557	33.9965	OK	OK	-0.30347
ENSG0000010	-	-	ENSG0000010096	NFATC4	-	14:24365672-24379604	-	-	IGV_LINK	565.347	15.6343	0.088744	31.1799	OK	238.213	8.1783	0	17.4408	OK	OK	-0.93484
ENSG0000010	-	-	ENSG0000010097	PLTP	-	20:45888624-45912155	-	-	IGV_LINK	564.377	20.6285	0	50.4783	OK	5762.49	206.804	0	415.113	OK	OK	3.32555
ENSG0000010	-	-	ENSG0000010098	PCIF1	-	20:45934627-45948023	-	-	IGV_LINK	734.078	17.5553	5.6231	29.4875	OK	678.546	15.8535	5.56238	26.1447	OK	OK	-0.1471
ENSG0000010	-	-	ENSG0000010098	GSS	-	20:34928429-34955817	-	-	IGV_LINK	776.746	25.0211	10.4609	39.5814	OK	909.54	29.3181	12.4841	46.1521	OK	OK	0.228643
ENSG0000010	-	-	ENSG0000010098	MMP9	-	20:46008907-46060152	-	-	IGV_LINK	150.307	3.91527	0.69035	7.14019	OK	211.401	5.50962	1.26713	9.75212	OK	OK	0.492841
ENSG0000010	-	-	ENSG0000010098	VSX1	-	20:25070884-25082365	-	-	IGV_LINK	42.6677	1.44131	0	3.13641	OK	35.0616	1.62724	0	3.78904	OK	OK	0.175047
ENSG0000010	-	-	ENSG0000010099	TRPC4AP	-	20:34975402-35092871	-	-	IGV_LINK	1676.65	31.3882	13.0446	49.7318	OK	1973.76	36.8483	15.0792	58.6174	OK	OK	0.231376
ENSG0000010	-	-	ENSG0000010099	PYGB	-	20:25248068-25390983	-	-	IGV_LINK	1256.76	18.8767	1.51367	36.2398	OK	1902.61	28.301	4.87719	51.7247	OK	OK	0.584242
ENSG0000010	-	-	ENSG0000010099	ABHD12	-	20:25248068-25390983	-	-	IGV_LINK	1174.33	40.5217	1.93116	79.1123	OK	1316.87	45.359	0	93.4708	OK	OK	0.162693
ENSG0000010	-	-	ENSG0000010100	PROCR	-	20:35172072-35284985	-	-	IGV_LINK	325.826	16.2457	0	46.6716	OK	332.054	16.1022	0	48.8258	OK	OK	-0.0128
ENSG0000010	-	-	ENSG0000010100	GINS1	-	20:25407726-25452628	-	-	IGV_LINK	87.2748	1.59872	0.068398	3.12903	OK	88.6853	1.62516	0.04161	3.20871	OK	OK	0.023671
ENSG0000010	-	-	ENSG0000010100	NINL	-	20:25452704-25585517	-	-	IGV_LINK	516.861	9.18431	0	19.6038	OK	407.334	8.39462	0	18.4635	OK	OK	-0.12971
ENSG0000010	-	-	ENSG0000010101	CD40	-	20:46118271-46129863	-	-	IGV_LINK	1529.25	64.0583	25.3502	102.766	OK	1074.54	43.8667	17.4333	70.3	OK	OK	-0.54626
ENSG0000010	-	-	ENSG0000010101	UQCC1	-	20:35302565-35412141	-	-	IGV_LINK	930.932	27.7949	6.05035	49.5394	OK	1271.5	44.2681	9.3426	79.1936	OK	OK	0.671448
ENSG0000010	-	-	ENSG0000010104	ZMYND8	-	20:47209213-47356889	-	-	IGV_LINK	1481.73	17.4707	6.70633	28.235	OK	2613.12	31.5887	10.2582	52.9191	OK	OK	0.854471
ENSG0000010	-	-	ENSG0000010104	SGK2	-	20:43507679-43588237	-	-	IGV_LINK	155.155	5.00414	0	13.1595	OK	488.801	17.4181	0	34.9938	OK	OK	1.7994
ENSG0000010	-	-	ENSG0000010105	IFT52	-	20:43590930-43647296	-	-	IGV_LINK	500.376	20.5601	6.10193	35.0184	OK	521.8	22.1302	6.23349	38.0269	OK	OK	0.106165
ENSG0000010	-	-	ENSG0000010105	MYBL2	-	20:43667018-43716496	-	-	IGV_LINK	114.427	2.60809	0.380553	4.83563	OK	161.902	3.70096	0.833784	6.56813	OK	OK	0.504903
ENSG0000010	-	-	ENSG0000010107	R3HDML	-	20:44336985-44355185	-	-	IGV_LINK	0	0	0	0	OK	0	0	0	0	OK	NOTEST	0
ENSG0000010	-	-	ENSG0000010107	HNF4A	-	20:44355699-44434596	-	-	IGV_LINK	846.566	13.6749	4.897	22.4528	OK	3609.29	56.504	21.7982	91.2098	OK	OK	2.04682
ENSG0000010	-	-	ENSG0000010107	NDRG3	-	20:36651765-36746078	-	-	IGV_LINK	627.409	13.1993	5.1991	21.1995	OK	581.611	11.7958	4.68537	18.9062	OK	OK	-0.16219
ENSG0000010	-	-	ENSG0000010108	SLA2	-	20:36573487-36646216	-	-	IGV_LINK	240.491	5.32911	0	12.4102	OK	157.777	3.52529	0	9.87053	OK	OK	-0.59615
ENSG0000010	-	-	ENSG0000010108	C20orf24	-	20:36573487-36646216	-	-	IGV_LINK	488.855	31.1364	0	62.9995	OK	1036.68	71.4699	12.2295	130.71	OK	OK	1.19873
ENSG0000010	-	-	ENSG0000010109	NFATC2	-	20:51386956-51562831	-	-	IGV_LINK	825.232	6.70618	1.53144	11.8809	OK	483.645	3.97406	0.52915	7.41897	OK	OK	-0.75488
ENSG0000010	-	-	ENSG0000010109	RIMS4	-	20:44656450-44810338	-	-	IGV_LINK	0	0	0	0	OK	2.06245	0.023111	0	0.156713	OK	NOTEST	inf
ENSG0000010	-	-	ENSG0000010110	PABPC1L	-	20:44910061-44960486	-	-	IGV_LINK	1473.01	58.1054	14.3563	101.855	OK	1001.32	38.7706	8.7995	68.7417	OK	OK	-0.58371
ENSG0000010	-	-	ENSG0000010110	STK4	-	20:44966473-45079959	-	-	IGV_LINK	1069.6	10.5518	3.3599	17.7438	OK	1061.13	10.1042	3.9428	16.2657	OK	OK	-0.06254
ENSG0000010	-	-	ENSG0000010111	SALL4	-	20:51782330-51802520	-	-	IGV_LINK	44.6071	0.622316	0	1.48665	OK	25.7806	0.670626	0	1.80435	OK	OK	0.107861
ENSG0000010	-	-	ENSG0000010112	ADNP	-	20:50888918-50958555	-	-	IGV_LINK	1655.31	18.2075	6.19361	30.2214	OK	1441.65	16.0927	5.53891	26.6464	OK	OK	-0.17813
ENSG0000010	-	-	ENSG0000010113	PFDN4	-	20:54207846-54228052	-	-	IGV_LINK	202.672	23.1021	4.12459	42.0796	OK	186.652	22.3251	3.91157	40.7387	OK	OK	-0.04936
ENSG0000010	-	-	ENSG0000010113	DOK5	-	20:54475596-54651171	-	-	IGV_LINK	36.8494	1.66227	0	3.69345	OK	90.7478	3.34382	0	6.75514	OK	OK	1.00834
ENSG0000010	-	-	ENSG0000010113	CSTF1	-	20:56392370-56406369	-	-	IGV_LINK	371.403	11.7526	2.76588	20.7393	OK	444.458	16.3323	3.08237	29.5822	OK	OK	0.474747
ENSG0000010	-	-	ENSG0000010114	BMP7	-	20:57168747-57266629	-	-	IGV_LINK	0	0	0	0	OK	0	0	0	0	OK	NOTEST	0
ENSG0000010	-	-	ENSG0000010114	RAE1	-	20:57351009-57379211	-	-	IGV_LINK	482.921	15.1279	4.22983	26.0259	OK	547.58	20.714	3.21655	38.2114	OK	OK	0.453395
ENSG0000010	-	-	ENSG0000010115	TPD52L2	-	20:63865227-63891545	-	-	IGV_LINK	1759.07	44.9039	18.4855	71.3223	OK	2420.29	61.0601	24.8994	97.2209	OK	OK	0.44339
ENSG0000010	-	-	ENSG0000010115	DNAJC5	-	20:63895181-63936031	-	-	IGV_LINK	1126.82	12.5365	5.02049	20.0525	OK	1653.05	18.4149	7.67233	29.1574	OK	OK	0.554738
ENSG0000010	-	-	ENSG0000010115	NELFCD	-	20:58981207-58995133	-	-	IGV_LINK	1658.22	50.9625	16.1292	85.7959	OK	1277.69	41.815	12.7836	70.8465	OK	OK	-0.28542
ENSG0000010	-	-	ENSG0000010116	CTSZ	-	20:58995184-59007247	-	-	IGV_LINK	6649.37	288.298	101.502	475.093	OK	8484.92	365.648	130.246	601.05	OK	OK	0.342896
ENSG0000010	-	-	ENSG0000010116	PRPF6	-	20:63981134-64033100	-	-	IGV_LINK	1857.98	36.4647	16.1931	56.7363	OK	1784.02	35.0271	15.2879	54.7662	OK	OK	-0.05803
ENSG0000010	-	-	ENSG0000010116	TUBB1	-	20:59019253-59032382	-	-	IGV_LINK	5.81832	0.09866	0	1.31625	OK	9.28102	0.157431	0	1.64512	OK	NOTEST	0.67418
ENSG0000010	-	-	ENSG0000010116	PRELID3B	-	20:59033144-59042909	-	-	IGV_LINK	547.892	24.468	5.37048	43.5654	OK	704.327	29.2	6.93818	51.4618	OK	OK	0.255074
ENSG0000010	-	-	ENSG0000010118	HRH3	-	20:62214969-62220267	-	-	IGV_LINK	0	0	0	0	OK	0	0	0	0	OK	NOTEST	0
ENSG0000010	-	-	ENSG0000010118	MTG2	-	20:62183028-62203568	-	-	IGV_LINK	381.1	15.9434	1.66837	30.2184	OK	457.864	20.0858	3.05782	37.1137	OK	OK	0.333217
ENSG0000010	-	-	ENSG0000010118	PSMA7	-	20:62136734-62143440	-	-	IGV_LINK	1615.55	115.615	49.4207	181.809	OK	1681.93	119.47	51.5481	187.392	OK	OK	0.047325
ENSG0000010	-	-	ENSG0000010118	SLCO4A1	-	20:62640718-62685785	-	-	IGV_LINK	315.159	9.26381	0.48115	18.0465	OK	45.3739	1.80784	0	5.03519	OK	OK	-2.35734
ENSG0000010	-	-	ENSG0000010118	NTSR1	-	20:62708836-62762771	-	-	IGV_LINK	34.9099	0.669016	0	1.40626	OK	29.9055	0.523206	0	1.15606	OK	OK	-0.35466
ENSG0000010	-	-	ENSG0000010118	MRGBP	-	20:62796452-62814000	-	-	IGV_LINK	264.734	5.7195	0	16.7696	OK	278.431	6.01807	0	17.1304	OK	OK	0.073413
ENSG0000010	-	-	ENSG0000010119	TCFL5	-	20:62816243-62861763	-	-	IGV_LINK	65.941	1.62801	0	3.30767	OK	49.4988	1.22249	0	2.5603	OK	OK	-0.41329
ENSG0000010	-	-	ENSG0000010119	DIDO1	-	20:62877737-62937952	-	-	IGV_LINK	2333.15	26.1311	8.9231	43.3392	OK	2252.2	25.7476	8.13635	43.3589	OK	OK	-0.02133
ENSG0000010	-	-	ENSG0000010119	GID8	-	20:62938118-62948475	-	-	IGV_LINK	806.807	11.4411	0.99148	21.8908	OK	729.076	10.4733	0.28975	20.6568	OK	OK	-0.12752
ENSG0000010	-	-	ENSG0000010119	SLC17A9	-	20:62952646-62969585	-	-	IGV_LINK	272.491	7.96819	0	16.7178	OK	244.4	6.41763	0	13.361	OK	OK	-0.31221
ENSG0000010	-	-	ENSG0000010119	BIRC7	-	20:63235882-63240507	-	-	IGV_LINK	7.75776	0.365706	0	0.932338	OK	23.7182	1.16742	0	2.46383	OK	OK	1.67456
ENSG0000010	-	-	ENSG0000010119	NKAIN4	-	20:63240783-63272694	-	-	IGV_LINK	93.0932	8.41518	0	17.4041	OK	721.857	49.7764	13.0795	86.4733	OK	OK	2.5644
ENSG0000010	-	-	ENSG0000010119	ARFGAP1	-	20:63272784-63289793	-	-	IGV_LINK	1508.89	41.0459	13.3206	68.7713	OK	1787.11	49.1724	16.4513	81.8934	OK	OK	0.260608
ENSG0000010	-	-	ENSG0000010120	AVP	-	20:3082555-3084724	-	-	IGV_LINK	0	0	0	0	OK	3.09367	0.391504	0	1.01147	OK	NOTEST	inf
ENSG0000010	-	-	ENSG0000010120	COL20A1	-	20:63293185-63334851	-	-	IGV_LINK	1.93944	0.111801	0	0.469566	OK	1.03122	0.014533	0	0.278529	OK	NOTEST	-2.94349
ENSG0000010	-	-	ENSG0000010120	CHRNA4	-	20:63343222-63378401	-	-	IGV_LINK	0	0	0	0	OK	0	0	0	0	OK	NOTEST	0
ENSG0000010	-	-	ENSG0000010121	EEF1A2	-	20:63488012-63499315	-	-	IGV_LINK	18.4247	0.625086	0	1.34401	OK	2.06245	0.073826	0	0.22921	OK	OK	-3.08185
ENSG0000010	-	-	ENSG0000010121	PTK6	-	20:63528000-63537370	-	-	IGV_LINK	30.0613	0.614175	0	1.26544	OK	29.9055	0.644814	0	1.32977	OK	OK	0.070234
ENSG0000010	-	-	ENSG0000010121	GMEB2	-	20:63587601-63627041	-	-	IGV_LINK	336.493	4.71484	1.37788	8.0518	OK	334.117	4.62536	1.57858	7.67213	OK	OK	-0.02764
ENSG0000010	-	-	ENSG0000010122	C20orf27	-	20:3753507-3768387	-	-	IGV_LINK	450.92	20.1804	6.88229	33.4786	OK	357.835	15.9537	4.96742	26.94	OK	OK	-0.33907
ENSG0000010	-	-	ENSG0000010122	SPEF1	-	20:3777503-3781448	-	-	IGV_LINK	96.9721	3.69973	0.440163	6.9593	OK	94.8727	3.80182	0.356249	7.24739	OK	OK	0.03927
ENSG0000010	-	-	ENSG0000010122	CDC25B	-	20:3786771-3806121	-	-	IGV_LINK	1286.82	28.7442	3.27558	54.2128	OK	1222	26.268	5.35789	47.1781	OK	OK	-0.12996
ENSG0000010	-	-	ENSG0000010123	ISM1	-	20:13221770-13300651	-	-	IGV_LINK	55.2741	1.28693	0	2.59359	OK	40.2178	0.936827	0	1.91406	OK	OK	-0.45808
ENSG0000010	-	-	ENSG0000010123	RNF24	-	20:3888238-4015582	-	-	IGV_LINK	574.075	5.43851	0	11.3544	OK	551.705	4.33022	0.736367	7.92407	OK	OK	-0.32877
ENSG0000010	-	-	ENSG0000010124	ARFRP1	-	20:63657809-63743505	-	-	IGV_LINK	836.869	30.1654	0	76.5653	OK	824.98	28.1063	0	68.1549	OK	OK	-0.102
ENSG0000010	-	-	ENSG0000010124	NDUFAF5	-	20:13784949-13821582	-	-	IGV_LINK	185.217	10.7183	0	21.6325	OK	182.527	12.5042	0.110621	24.8977	OK	OK	0.222331
ENSG0000010	-	-	ENSG0000010125	SEL1L2	-	20:13849246-16053197	-	-	IGV_LINK	0	0	0	0	OK	0	0	0	0	OK	NOTEST	0
ENSG0000010	-	-	ENSG0000010125	TRIB3	-	20:362834-397559	-	-	IGV_LINK	343.281	8.31547	2.65228	13.9787	OK	890.978	22.4221	9.09047	35.7538	OK	OK	1.43105
ENSG0000010	-	-	ENSG0000010126	RASSF2	-	20:4780022-4823645	-	-	IGV_LINK	1474.94	16.0051	7.03263	24.9775	OK	1598.4	17.3253	7.45602	27.1946	OK	OK	0.114354
ENSG0000010	-	-	ENSG0000010126	CSNK2A1	-	20:473590-543821	-	-	IGV_LINK	1799.8	36.0501	10.0556	62.0446	OK	1874.77	43.9573	8.20401	79.7106	OK	OK	0.2861
ENSG0000010	-	-	ENSG0000010127	SLC52A3	-	20:760079-776015	-	-	IGV_LINK	0.969721	0.022008	0	0.275546	OK	89.7166	2.55641	0	5.71772	OK	OK	6.85993
ENSG0000010	-	-	ENSG0000010127	RPS10P5	-	20:833714-857463	-	-	IGV_LINK	6.78804	1.06709	0	5.73043	OK	5.15612	0.81317	0	4.75275	OK	OK	-0.39205
ENSG0000010	-	-	ENSG0000010128	ANGPT4	-	20:869898-916317	-	-	IGV_LINK	3.87888	0.048539	0	0.123622	OK	3.09367	0.038723	0	0.100044	OK	NOTEST	-0.32595
ENSG0000010	-	-	ENSG0000010128	RSPO4	-	20:958451-1002264	-	-	IGV_LINK	29.0916	0.75269	0	1.70676	OK	37.1241	0.825849	0	1.70135	OK	OK	0.133822
ENSG0000010	-	-	ENSG0000010129	CDS2	-	20:5126785-5197887	-	-	IGV_LINK	1589.37	16.0787	1.43967	30.7177	OK	1440.62	19.3762	2.16399	36.5883	OK	OK	0.269135
ENSG0000010	-	-	ENSG0000010129	PROKR2	-	20:5302039-5314369	-	-	IGV_LINK	3.87888	0.222496	0	0.56666	OK	1.03122	0.059221	0	0.221652	OK	NOTEST	-1.90959
ENSG0000010	-	-	ENSG0000010129	HM13	-	20:31514427-31577923	-	-	IGV_LINK	2050.75	85.3564	29.0521	141.661	OK	3198.32	130.886	46.4013	215.371	OK	OK	0.616742
ENSG0000010	-	-	ENSG0000010129	SNPH	-	20:1226055-1309328	-	-	IGV_LINK	65.941	0.770584	0	1.57349	OK	61.8735	0.687351	0.013671	1.36103	OK	OK	-0.1649
ENSG0000010	-	-	ENSG0000010130	MYLK2	-	20:31819307-31834689	-	-	IGV_LINK	6.78804	0.188894	0	0.459691	OK	3.09367	0.079815	0	0.232498	OK	NOTEST	-1.24285
ENSG0000010	-	-	ENSG0000010130	SIRPB1	-	20:1516758-1620061	-	-	IGV_LINK	260.556	8.93713	0.847355	17.0269	OK	782.647	26.7812	6.51646	47.046	OK	OK	1.58334
ENSG0000010	-	-	ENSG0000010131	SEC23B	-	20:18507492-18561415	-	-	IGV_LINK	1111.3	25.7797	7.61916	43.9403	OK	1543.74	34.2715	10.3186	58.2244	OK	OK	0.410771
ENSG0000010	-	-	ENSG0000010131	FERMT1	-	20:6074844-6123544	-	-	IGV_LINK	0.969721	0.017352	0	0.228243	OK	18.562	0.255979	0	0.621583	OK	NOTEST	3.8829
ENSG0000010	-	-	ENSG0000010132	HAO1	-	20:7882980-7940474	-	-	IGV_LINK	0	0	0	0	OK	0	0	0	0	OK	NOTEST	0
ENSG0000010	-	-	ENSG0000010132	PDYN	-	20:1947245-2007517	-	-	IGV_LINK	0	0	0	0	OK	0	0	0	0	OK	NOTEST	0
ENSG0000010	-	-	ENSG0000010133	CCM2L	-	20:32010449-32032180	-	-	IGV_LINK	104.73	3.0032	0.089102	5.91729	OK	102.091	2.87387	0	5.7786	OK	OK	-0.06351
ENSG0000010	-	-	ENSG0000010133	PLCB4	-	20:9068762-9481242	-	-	IGV_LINK	369.464	5.66137	0	12.6117	OK	598.11	9.30943	0	19.457	OK	OK	0.717541
ENSG0000010	-	-	ENSG0000010133	MYL9	-	20:36306335-36573391	-	-	IGV_LINK	886.325	42.7257	1.3329	84.1184	OK	840.448	39.9079	0	86.9602	OK	OK	-0.09843
ENSG0000010	-	-	ENSG0000010133	HCK	-	20:32052187-32101856	-	-	IGV_LINK	1029.84	30.9565	13.2809	48.6321	OK	1017.82	30.4262	13.0734	47.779	OK	OK	-0.02493
ENSG0000010	-	-	ENSG0000010133	TM9SF4	-	20:32109505-32167258	-	-	IGV_LINK	1798.83	27.7755	10.3086	45.2424	OK	2298.6	36.0844	12.174	59.9948	OK	OK	0.377562
ENSG0000010	-	-	ENSG0000010134	TLDC2	-	20:36876120-36951843	-	-	IGV_LINK	266.673	11.7941	0	38.2878	OK	170.152	7.43353	0	27.1611	OK	OK	-0.66595

ENSG0000010	-	-	ENSG0000010134	'CRNKL1	-	20:20034367-20360702	-	-	IGV_LINK	558.559	14.1907	0.760613	27.6207	OK	578.517	13.5314	0.966397	26.0964	OK	OK	-0.06863
ENSG0000010	-	-	ENSG0000010134	'POFUT1	-	20:32207879-32238667	-	-	IGV_LINK	1151.06	15.2102	3.7922	26.6283	OK	1446.81	18.6659	5.37674	31.9551	OK	OK	0.295365
ENSG0000010	-	-	ENSG0000010134	'SAMHD1	-	20:36876120-36951843	-	-	IGV_LINK	3761.55	48.99	10.6147	87.3652	OK	3461.82	43.7595	13.3905	74.1284	OK	OK	-0.16289
ENSG0000010	-	-	ENSG0000010134	'PAK5	-	20:9537388-9839041	-	-	IGV_LINK	0	0	0	0	OK	0	0	0	0	OK	NOTEST	0
ENSG0000010	-	-	ENSG0000010135	'KIF3B	-	20:32277663-32335011	-	-	IGV_LINK	1834.71	17.4347	7.73936	27.1299	OK	2045.95	19.4458	8.46636	30.4252	OK	OK	0.157498
ENSG0000010	-	-	ENSG0000010135	'MROH8	-	20:37101225-37241623	-	-	IGV_LINK	19.3944	0.599158	0	6.15786	OK	25.7806	0.725051	0	7.44191	OK	OK	0.275146
ENSG0000010	-	-	ENSG0000010136	'NOP56	-	20:2652144-2658393	-	-	IGV_LINK	1486.58	79.4771	27.1006	131.854	OK	1707.71	82.5241	27.3	137.748	OK	OK	0.054277
ENSG0000010	-	-	ENSG0000010136	'MANBAL	-	20:37289637-37317260	-	-	IGV_LINK	614.803	29.6174	10.8239	48.4109	OK	699.171	33.7671	13.4647	54.0694	OK	OK	0.189171
ENSG0000010	-	-	ENSG0000010136	'IDH3B	-	20:2658394-2664219	-	-	IGV_LINK	1669.86	87.417	31.3852	143.449	OK	1614.9	82.9425	30.108	135.777	OK	OK	-0.0758
ENSG0000010	-	-	ENSG0000010136	'MAPRE1	-	20:32819892-32854257	-	-	IGV_LINK	1230.58	28.3005	12.3388	44.2621	OK	1462.28	33.6449	14.9237	52.366	OK	OK	0.249561
ENSG0000010	-	-	ENSG0000010138	'JAG1	-	20:10637683-10994924	-	-	IGV_LINK	3477.42	61.3197	0	125.291	OK	2239.82	34.2414	0	68.7476	OK	OK	-0.84061
ENSG0000010	-	-	ENSG0000010139	'CDK5RAP1	-	20:33358838-33401561	-	-	IGV_LINK	458.678	19.9355	5.39222	34.4788	OK	478.488	21.3481	5.54328	37.1529	OK	OK	0.098767
ENSG0000010	-	-	ENSG0000010140	'SNTA1	-	20:33407954-33443892	-	-	IGV_LINK	759.291	19.7237	7.98939	31.4581	OK	372.272	9.67546	3.33289	16.018	OK	OK	-1.02753
ENSG0000010	-	-	ENSG0000010140	'OXT	-	20:3071619-3072517	-	-	IGV_LINK	4.8486	0.802469	0	1.94912	OK	37.1241	6.1652	0	12.5281	OK	OK	2.94163
ENSG0000010	-	-	ENSG0000010140	'TTI1	-	20:37983006-38033468	-	-	IGV_LINK	574.075	11.0356	0.800393	21.2708	OK	756.919	14.1764	1.3592	26.9936	OK	OK	0.361326
ENSG0000010	-	-	ENSG0000010141	'E2F1	-	20:33675682-33686404	-	-	IGV_LINK	123.155	2.97811	0.504928	5.4513	OK	114.466	2.76938	0.272962	5.26579	OK	OK	-0.10484
ENSG0000010	-	-	ENSG0000010141	'RPRD1B	-	20:38033545-38166578	-	-	IGV_LINK	653.592	15.6192	0	79.8791	OK	774.45	16.439	0	93.7358	OK	OK	0.073796
ENSG0000010	-	-	ENSG0000010141	'PXMP4	-	20:33702753-33720319	-	-	IGV_LINK	187.156	3.79074	0	8.03717	OK	207.276	3.55943	0	7.43603	OK	OK	-0.09083
ENSG0000010	-	-	ENSG0000010142	'CHMP4B	-	20:33811303-33854366	-	-	IGV_LINK	1651.43	63.1618	27.6653	98.6582	OK	2283.13	87.3903	38.3507	136.43	OK	OK	0.468422
ENSG0000010	-	-	ENSG0000010142	'BPI	-	20:38210487-38337505	-	-	IGV_LINK	1.93944	0.089096	0	0.91916	OK	4.1249	0.186772	0	1.21576	OK	NOTEST	1.06785
ENSG0000010	-	-	ENSG0000010143	'CST9L	-	20:23564731-23568749	-	-	IGV_LINK	0	0	0	0	OK	0	0	0	0	OK	NOTEST	0
ENSG0000010	-	-	ENSG0000010143	'SLC32A1	-	20:38724461-38729372	-	-	IGV_LINK	0	0	0	0	OK	0	0	0	0	OK	NOTEST	0
ENSG0000010	-	-	ENSG0000010143	'CST3	-	20:23626705-23638473	-	-	IGV_LINK	5065.82	400.669	144.148	657.191	OK	8646.82	671.507	197.72	1145.29	OK	OK	0.74499
ENSG0000010	-	-	ENSG0000010144	'ASIP	-	20:34194568-34311802	-	-	IGV_LINK	8.72748	1.18953	0	10.7218	OK	13.4059	1.83229	0	14.131	OK	OK	0.623257
ENSG0000010	-	-	ENSG0000010144	'CST4	-	20:23685639-23689040	-	-	IGV_LINK	0	0	0	0	OK	0	0	0	0	OK	NOTEST	0
ENSG0000010	-	-	ENSG0000010144	'ACTR5	-	20:38748441-38772520	-	-	IGV_LINK	253.097	5.96177	1.66776	10.2558	OK	230.994	5.44375	1.57711	9.3104	OK	OK	-0.13114
ENSG0000010	-	-	ENSG0000010144	'WFDC2	-	20:45469705-45481532	-	-	IGV_LINK	625.47	77.5634	30.2324	124.894	OK	2246.01	279.897	122.976	436.818	OK	OK	1.85145
ENSG0000010	-	-	ENSG0000010144	'AHCY	-	20:34194568-34311802	-	-	IGV_LINK	1786.21	53.4953	21.2932	85.6974	OK	1933.55	56.2458	24.2822	88.2094	OK	OK	0.072333
ENSG0000010	-	-	ENSG0000010144	'PPP1R16B	-	20:38805704-38923024	-	-	IGV_LINK	451.89	4.18961	1.27271	7.10651	OK	365.054	3.38518	0.769131	6.00124	OK	OK	-0.30758
ENSG0000010	-	-	ENSG0000010144	'SPINT3	-	20:45512460-45515624	-	-	IGV_LINK	0	0	0	0	OK	0	0	0	0	OK	NOTEST	0
ENSG0000010	-	-	ENSG0000010144	'FAM83D	-	20:38926311-38953106	-	-	IGV_LINK	66.9107	1.69776	0.08393	3.31159	OK	157.777	4.03829	0.893186	7.18339	OK	OK	1.25011
ENSG0000010	-	-	ENSG0000010144	'EPPIN	-	20:45534195-45547752	-	-	IGV_LINK	0.969721	0.066203	0	0.29091	OK	0	0	0	0	OK	NOTEST	'-inf
ENSG0000010	-	-	ENSG0000010145	'DHX35	-	20:38962298-39039723	-	-	IGV_LINK	293.825	5.7478	1.07096	10.4246	OK	241.307	4.38316	1.27432	7.492	OK	OK	-0.39104
ENSG0000010	-	-	ENSG0000010145	'DNTTIP1	-	20:45791936-45811427	-	-	IGV_LINK	354.918	18.8614	6.08553	31.6374	OK	518.706	27.6165	9.95013	45.2829	OK	OK	0.550092
ENSG0000010	-	-	ENSG0000010146	'MAP1LC3A	-	20:34546853-34560345	-	-	IGV_LINK	227.884	20.3654	4.38825	36.3426	OK	128.903	10.4046	0.019921	20.7892	OK	OK	-0.96891
ENSG0000010	-	-	ENSG0000010146	'SYNDIG1	-	20:24469198-24666616	-	-	IGV_LINK	83.396	2.00037	0.156153	3.84458	OK	503.238	12.2906	4.20684	20.3744	OK	OK	2.61922
ENSG0000010	-	-	ENSG0000010146	'PIGU	-	20:34560541-34825649	-	-	IGV_LINK	283.158	11.1648	0	31.1474	OK	273.275	10.747	0	29.8432	OK	OK	-0.05503
ENSG0000010	-	-	ENSG0000010147	'TNNC2	-	20:45823213-45833745	-	-	IGV_LINK	5.81832	0.61006	0	1.48214	OK	3.09367	0.287547	0	0.790792	OK	OK	-1.08515
ENSG0000010	-	-	ENSG0000010147	'ACOT8	-	20:45833809-45857406	-	-	IGV_LINK	425.707	16.9293	3.2984	30.5603	OK	460.958	19.9562	2.07911	37.8333	OK	OK	0.237312
ENSG0000010	-	-	ENSG0000010147	'APMAP	-	20:24962924-24992979	-	-	IGV_LINK	1599.07	41.6621	17.5365	65.7876	OK	1909.83	49.07	21.6765	76.4635	OK	OK	0.236107
ENSG0000010	-	-	ENSG0000010148	'CELF4	-	18:37243046-37566037	-	-	IGV_LINK	12.6064	0.299824	0	1.2652	OK	11.3435	0.316658	0	1.30616	OK	NOTEST	0.078812
ENSG0000010	-	-	ENSG0000010149	'ZNF516	-	18:76358189-76495190	-	-	IGV_LINK	619.651	6.95151	0	16.3259	OK	620.797	8.26388	0	21.5554	OK	OK	0.249493
ENSG0000010	-	-	ENSG0000010154	'CDH20	-	18:61333581-61555773	-	-	IGV_LINK	1.93944	0.043534	0	0.280625	OK	5.15612	0.092875	0	0.353685	OK	NOTEST	1.09316
ENSG0000010	-	-	ENSG0000010154	'ADNP2	-	18:80109030-80147523	-	-	IGV_LINK	476.133	5.13988	1.80499	8.47478	OK	537.268	5.80113	2.13128	9.47098	OK	OK	0.174597
ENSG0000010	-	-	ENSG0000010154	'RBFA	-	18:80034345-80097088	-	-	IGV_LINK	395.882	18.232	3.35732	33.1067	OK	413.152	19.1962	5.96422	32.4281	OK	OK	0.074342
ENSG0000010	-	-	ENSG0000010155	'USP14	-	18:158382-268050	-	-	IGV_LINK	705.957	13.7932	0.90001	26.6863	OK	813.637	14.1912	2.49008	25.8923	OK	OK	0.041042
ENSG0000010	-	-	ENSG0000010155	'VAPA	-	18:9914001-9960021	-	-	IGV_LINK	1435.19	81.6431	21.7404	141.546	OK	2020.17	124.815	41.8955	207.735	OK	OK	0.61239
ENSG0000010	-	-	ENSG0000010157	'METTL4	-	18:2537524-2571509	-	-	IGV_LINK	203.641	4.62171	0.397489	8.84593	OK	144.371	2.98791	0.415536	5.56027	OK	OK	-0.62929
ENSG0000010	-	-	ENSG0000010157	'LPIN2	-	18:2916993-3013315	-	-	IGV_LINK	1535.07	17.3762	1.26121	33.4912	OK	2460.5	28.5114	1.43603	55.5867	OK	OK	0.714426
ENSG0000010	-	-	ENSG0000010159	'SMCHD1	-	18:2655737-2833065	-	-	IGV_LINK	1046.33	12.0109	1.86194	22.1598	OK	893.041	11.1322	0.567396	21.697	OK	OK	-0.1096
ENSG0000010	-	-	ENSG0000010160	'MYOM1	-	18:3066806-3247277	-	-	IGV_LINK	66.9107	1.83841	0	5.37261	OK	69.0921	1.04849	0	3.21748	OK	OK	-0.81015
ENSG0000010	-	-	ENSG0000010160	'MYL12A	-	18:3247480-3261850	-	-	IGV_LINK	3307.72	213.506	87.4727	339.54	OK	3871.22	248.965	100.509	397.421	OK	OK	0.221664
ENSG0000010	-	-	ENSG0000010162	'CEP76	-	18:12438889-12725740	-	-	IGV_LINK	77.5776	2.00116	0	11.4564	OK	88.6853	2.96502	0	16.8233	OK	OK	0.567204
ENSG0000010	-	-	ENSG0000010163	'ST8SIA5	-	18:46667820-46764408	-	-	IGV_LINK	22.3036	0.342174	0	1.1268	OK	38.1553	0.356249	0	1.27014	OK	NOTEST	0.058159
ENSG0000010	-	-	ENSG0000010163	'CEP192	-	18:12991361-13125052	-	-	IGV_LINK	465.466	5.85768	0	11.894	OK	434.146	6.401	0.641089	12.1609	OK	OK	0.127967
ENSG0000010	-	-	ENSG0000010165	'RNMT	-	18:13663346-13764558	-	-	IGV_LINK	912.507	11.153	2.90476	19.4013	OK	1007.51	12.675	2.84286	22.5072	OK	OK	0.184552
ENSG0000010	-	-	ENSG0000010166	'SMAD7	-	18:48919852-48950711	-	-	IGV_LINK	679.774	19.4915	5.50277	33.4801	OK	392.897	13.5243	1.15098	25.8976	OK	OK	-0.52729
ENSG0000010	-	-	ENSG0000010167	'LIPG	-	18:49560698-49599182	-	-	IGV_LINK	101.821	2.46232	0	5.84114	OK	26.8118	0.796252	0	2.18775	OK	OK	-1.62872
ENSG0000010	-	-	ENSG0000010168	'LAMA1	-	18:6941743-7117814	-	-	IGV_LINK	1485.61	15.586	0	31.8123	OK	734.232	9.16048	0	19.2437	OK	OK	-0.76676
ENSG0000010	-	-	ENSG0000010169	'RNF125	-	18:32018371-32470484	-	-	IGV_LINK	192.243	2.38176	0	8.83534	OK	163.913	1.58003	0	4.41049	OK	OK	-0.59207
ENSG0000010	-	-	ENSG0000010174	'ANKRD12	-	18:9102629-9285985	-	-	IGV_LINK	991.054	6.90281	0	17.3682	OK	736.295	4.41863	0	9.9361	OK	OK	-0.64359
ENSG0000010	-	-	ENSG0000010174	'NOL4	-	18:33851099-34224952	-	-	IGV_LINK	0	0	0	0	OK	0	0	0	0	OK	NOTEST	0
ENSG0000010	-	-	ENSG0000010175	'POLI	-	18:54268345-54321266	-	-	IGV_LINK	717.593	16.3406	4.22625	28.455	OK	477.457	12.9623	1.14468	24.7799	OK	OK	-0.33414
ENSG0000010	-	-	ENSG0000010175	'MIB1	-	18:21704956-21870957	-	-	IGV_LINK	420.859	2.78573	0.860789	4.71067	OK	314.524	2.08434	0	4.54422	OK	OK	-0.41846
ENSG0000010	-	-	ENSG0000010177	'RBBP8	-	18:22699480-23026488	-	-	IGV_LINK	559.529	11.5917	3.25918	19.9242	OK	847.667	17.6984	6.3363	29.0604	OK	OK	0.610523
ENSG0000010	-	-	ENSG0000010178	'RIOK3	-	18:23452822-23486603	-	-	IGV_LINK	1173.36	28.732	8.33911	49.1249	OK	1999.55	49.1887	16.3015	82.0758	OK	OK	0.775667
ENSG0000010	-	-	ENSG0000010181	'CSTF2	-	X:100820358-100840932	-	-	IGV_LINK	190.065	5.95736	1.37063	10.5441	OK	229.963	7.28578	2.05738	12.5142	OK	OK	0.29041
ENSG0000010	-	-	ENSG0000010181	'H2BFM	-	X:104039948-104042454	-	-	IGV_LINK	4.8486	0.159842	0	0.560354	OK	0	0	0	0	OK	NOTEST	'-inf
ENSG0000010	-	-	ENSG0000010182	'MXRA5	-	X:3308564-3346641	-	-	IGV_LINK	113.457	0.664616	0.067523	1.26171	OK	58.7798	0.344365	0.005206	0.683524	OK	OK	-0.94858
ENSG0000010	-	-	ENSG0000010184	'VSIG1	-	X:108044969-108079184	-	-	IGV_LINK	30.0613	0.9926	0	2.671	OK	91.779	2.52812	0	6.6183	OK	OK	1.34878
ENSG0000010	-	-	ENSG0000010184	'PSMD10	-	X:108084206-108091618	-	-	IGV_LINK	400.495	22.1282	5.48256	38.7738	OK	400.115	22.4502	5.5964	39.3041	OK	OK	0.020845
ENSG0000010	-	-	ENSG0000010184	'ATG4A	-	X:108091667-108154671	-	-	IGV_LINK	232.733	8.22266	0.677413	15.7679	OK	263.994	8.82189	0.925184	16.7186	OK	OK	0.101483
ENSG0000010	-	-	ENSG0000010184	'STS	-	X:7219455-7354810	-	-	IGV_LINK	324.856	2.88375	0.978107	4.78939	OK	507.363	4.50467	1.69178	7.31756	OK	OK	0.643477
ENSG0000010	-	-	ENSG0000010184	'TBL1X	-	X:9463294-9719743	-	-	IGV_LINK	1611.68	19.8802	0.618051	39.1423	OK	1091.04	11.3916	4.68146	18.1017	OK	OK	-0.80336
ENSG0000010	-	-	ENSG0000010185	'GPR143	-	X:9725345-9786297	-	-	IGV_LINK	60.1227	2.58114	0	5.30934	OK	38.1553	1.49121	0	3.22164	OK	OK	-0.79153
ENSG0000010	-	-	ENSG0000010185	'PGRMC1	-	X:119236244-119244466	-	-	IGV_LINK	3357.17	108.843	45.0639	172.623	OK	3560.82	115.506	47.9571	183.055	OK	OK	0.085714
ENSG0000010	-	-	ENSG0000010186	'POLA1	-	X:24693918-24996986	-	-	IGV_LINK	396.616	4.29476	1.20643	7.38308	OK	294.93	3.14664	0.909611	5.38366	OK	OK	-0.44877
ENSG0000010	-	-	ENSG0000010187	'MID1	-	X:10445309-10833654	-	-	IGV_LINK	651.652	8.58748	0.95562	16.2193	OK	470.239	6.53267	0.238791	12.8266	OK	OK	-0.39456
ENSG0000010	-	-	ENSG0000010188	'NKAP	-	X:119895836-119992810	-	-	IGV_LINK	242.43	4.88228	0.110283	9.65427	OK	253.681	4.88425	0.601925	9.16657	OK	OK	0.000583
ENSG0000010	-	-	ENSG0000010188	'RHOXF1	-	X:120036235-120245267	-	-	IGV_LINK	22.3036	2.10727	0	6.16446	OK	2.06245	0.195239	0	1.17901	OK	OK	-3.43206
ENSG0000010	-	-	ENSG0000010188	'NXT2	-	X:109535780-109544690	-	-	IGV_LINK	160.974	6.09904	0.532339	11.6657	OK	144.371	5.15621	0.491591	9.82084	OK	OK	-0.24227
ENSG0000010	-	-	ENSG0000010189	'GUCY2F	-	X:109372905-109482072	-	-	IGV_LINK	0	0	0	0	OK	1.03122	0.016415	0	0.061439	OK	NOTEST	inf
ENSG0000010	-	-	ENSG0000010189	'ATP1B4	-	X:120362084-120383249	-	-	IGV_LINK	0	0	0	0	OK	0	0	0	0	OK	NOTEST	0
ENSG0000010	-	-	ENSG0000010189	'MCTS2P	-	20:31514427-31577923	-	-	IGV_LINK	20.5692	3.10369	0	20.4791	OK	14.979	2.26719	0	21.1622	OK	OK	-0.45308
ENSG0000010	-	-	ENSG0000010190	'ALG13	-	X:111665810-111760649	-	-	IGV_LINK	843.657	38.3502	5.04431	71.6561	OK	837.355	41.9176	7.5181	76.3171	OK	OK	0.128323
ENSG0000010	-	-	ENSG0000010191	'PRPS2	-	X:12791354-12824222	-	-	IGV_LINK	450.92	15.2272	2.89937	27.5549	OK	413.521	11.98	2.55433	21.4056	OK	OK	-0.34602
ENSG0000010	-	-	ENSG0000010191	'TLR8	-	X:12902816-12923169	-	-	IGV_LINK	351.039	4.93385	1.65473	8.21296	OK	344.429	4.82609	1.62368	8.0285	OK	OK	-0.03186
ENSG0000010	-	-	ENSG0000010192	'MOSPD1	-	X:134887625-134915267	-	-	IGV_LINK	109.578	4.21917	0	8.70081	OK	119.622	3.23527	0.273014	6.19752	OK	OK	-0.38308
ENSG0000010	-	-	ENSG0000010193	'AMMECR1	-	X:110194185-110456334	-	-	IGV_LINK	163.883	2.48601	0	6.64154	OK	232.026	3.16272	0	7.24886	OK	OK	0.347334
ENSG0000010	-	-	ENSG0000010193	'CHRDL1	-	X:110673855-110795819	-	-	IGV_LINK	0.969721	0.01482	0	0.150231	OK	41.249	0.70867	0	1.53063	OK	OK	5.57948
ENSG0000010	-	-	ENSG0000010194	'WDR13	-	X:48590041-48608867	-	-	IGV_LINK	1915.2	114.161	21.0629	207.259	OK	2429.57	150.279	26.2126	274.345	OK	OK	0.396571
ENSG0000010	-	-	ENSG0000010194	'SUV39H1	-	X:48695553-48737163	-	-	IGV_LINK	120.245	3.05586	0	6.31717	OK	119.622	3.10062	0	6.44939	OK	OK	0.020975
ENSG0000010	-	-	ENSG0000010195	'PAGE4	-	X:49829259-49833973	-	-	IGV_LINK	0	0	0	0	OK	0	0	0	0	OK	NOTEST	0
ENSG0000010	-	-	ENSG0000010195	'SRPX	-	X:37349274-38688920	-	-	IGV_LINK	13.5761	0.737873	0	9.57808	OK	26.8118	0.88865	0	7.81634	OK	OK	0.268242
ENSG0000010	-	-	ENSG0000010195	'GLRA2	-	X:14529297-14731812	-	-	IGV_LINK	0	0	0	0	OK	0	0	0	0	OK	NOTEST	0
ENSG0000010	-	-	ENSG0000010196	'XIAP	-	X:123859723-123913979	-	-	IGV_LINK	621.591	7.05431	0	17.1481	OK	700.202	10.9402	0	26.4475	OK	OK	0.633068
ENSG0000010	-	-	ENSG0000010197	'STAG2	-	X:123959232-124963817	-	-	IGV_LINK	1517.61	21.6901	0	56.7	OK	1261.19	17.204	0	38.7975	OK	OK	-0.3343
ENSG0000010	-	-	ENSG0000010197	'ATP11C	-	X:139726345-139945276	-	-	IGV_LINK	526.558	5.03739	1.0416	9.03319	OK	516.644	5.43735	1.0273	9.8474	OK	OK	0.110226
ENSG0000010	-	-	ENSG0000010197	'MCF2	-	X:139581769-139708227	-	-	IGV_LINK	14.5458	0.288443	0	0.707964	OK	5.15612	0.099867	0	0.346615	OK	NOTEST	-1.53021
ENSG0000010	-	-	ENSG0000010198	'F9	-	X:139530757-139563458	-	-	IGV_LINK	0	0	0	0	OK	0	0	0	0	OK	NOTEST	0
ENSG0000010	-	-	ENSG0000010198	'ABCD1	-	X:153724867-153785732	-	-	IGV_LINK	429.586	7.57754	2.01609	13.139	OK	643.484	10.9448	3.49696	18.3927	OK	OK	0.530448
ENSG0000010	-	-	ENSG0000010199	'CCDC22	-	X:49235466-49264826	-	-	IGV_LINK	413.101	10.8459	2.46556	19.2263	OK	386.709	10.3138	2.19875	18.4288	OK	OK	-0.07258
ENSG0000010	-	-	ENSG0000010200	'CACNA1F	-	X:49205062-49233371	-	-	IGV_LINK	26.1825	0.292674	0	0.90871	OK	23.7182	0.467411	0	1.54873	OK	NOTEST	0.675398
ENSG0000010	-	-	ENSG0000010200	'SYP	-	X:49187803-49202454	-	-	IGV_LINK	32.9705	0.850389	0	1.85185	OK	19.5933	0.51197	0	1.32941	OK	OK	-0.73206
ENSG0000010	-	-	ENSG0000010200	'PLP2	-	X:49171925-49175239	-	-	IGV_LINK	660.38	41.9542	16.3033	67.605	OK	783.731	49.4112	20.33	78.4925	OK	OK	0.236026
ENSG0000010	-	-	ENSG0000010201	'BMX	-	X:15384798-15556529	-	-	IGV_LINK	3.87888	0.147958	0	1.24901	OK	18.562	0.443755	0	1.68523	OK	NOTEST	1.58458
ENSG0000010	-	-	ENSG0000010202	'LUZP4	-	X:115289726-115307556	-	-	IGV_LINK	0	0	0	0	OK	1.03122	0.040568	0	0.174117	OK	NOTEST	inf
ENSG0000010	-	-	ENSG0000010202	'PLS3	-	X:115518181-115650861	-	-	IGV_LINK	1066.69	25.4144	0	61.8379	OK	1508.68	40.2048	0	112.682	OK	OK	0.661725
ENSG0000010	-	-	ENSG0000010203	'NAA10	-	X:153861513-153935223	-	-	IGV_LINK	588.246	45.2846	0	107.25	OK	543.34	43.4334	0	101.176	OK	OK	-0.06022
ENSG0000010	-	-	ENSG0000010203	'RENBP	-	X:153935262-153944691	-	-	IGV_LINK	314.189	15.7891	4.09697	27.4813	OK	347.523	17.8058	5.25227	30.3594	OK	OK	0.17342
ENSG0000010	-	-	ENSG0000010203	'ELF4	-	X:130064873-130110716	-	-	IGV_LINK	748.624	11.0668	4.51487	17.6187	OK	923.978	13.5904	5.27962	21.9013	OK	OK	0.296356
ENSG0000010	-	-	ENSG0000010203	'SMARCA1	-	X:129446500-129523500	-	-	IGV_LINK	569.226	9.14258	3.39196	14.8932	OK	685.765	11.018	4.10354	17.9324	OK	OK	0.269188
ENSG0000010	-	-	ENSG0000010204	'MTMR8	-	X:64268080-64395431	-	-	IGV_LINK	34.9099	2.15449	0	5.21725	OK	18.562	0.778631	0	2.16362	OK	OK	-1.46833
ENSG0000010	-	-	ENSG0000010204	'ASB9	-	X:15235287-15270467	-	-	IGV_LINK	45.5769	1.89167	0	4.18377	OK	48.4676	1.87198	0	4.25254	OK	OK	-0.0151
ENSG0000010	-	-	ENSG0000010205	'ZC3H12B	-	X:65366637-65507887	-	-	IGV_LINK	74.6685	1.02519	0	2.29863	OK	46.4051	0.511642	0	1.18499	OK	OK	-1.00268
ENSG0000010	-	-	ENSG0000010205	'RBBP7	-	X:16786426-16870414	-	-	IGV_LINK	1628.16	57.0655	15.3683	98.7627	OK	1833.52	61.7398	18.8835	104.596	OK	OK	0.113581
ENSG0000010	-	-	ENSG0000010205	'PPP1R2P9	-	X:42777367-42778247	-	-	IGV_LINK	0	0	0	0	OK	0	0	0	0	OK	NOTEST	0
ENSG0000010	-	-	ENSG0000010205	'KCND1	-	X:48961377-48971569	-	-	IGV_LINK	54.3043	0.717636	0	1.52225	OK	63.9359	0.88186	0	1.87494	OK	OK	0.297297
ENSG0000010	-	-	ENSG0000010207	'OPN1LW	-	X:154144223-154159032	-	-	IGV_LINK	0	0	0	0	OK	0	0	0	0	OK	NOTEST	0
ENSG0000010	-	-	ENSG0000010207	'SLC25A14	-	X:130339887-130373361	-	-	IGV_LINK	224.975	15.3953	2.5113	28.2794	OK	237.182	15.7452	3.86643	27.6239	OK	OK	0.032415
ENSG0000010	-	-	ENSG0000010208	'FMR1	-	X:147911950-147951125	-	-	IGV_LINK	699.168	16.464	2.97621	29.9517	OK	747.638	21.3825	1.44197	41.323	OK	OK	0.377117
ENSG0000010	-	-	ENSG0000010209	'PIM2	-	X:48913181-48919024	-	-	IGV_LINK	905.719	32.6055	11.6989	53.5121	OK	1157.03	41.5914	15.005	68.1778	OK	OK	0.351169
ENSG0000010	-	-	ENSG0000010209	'SCML2	-	X:18239313-18354727	-	-	IGV_LINK	12.6064	0.176397	0	0.465383	OK	43.3114	0.65846	0	1.43437	OK	OK	1.90027
ENSG0000010	-	-	ENSG0000010210	'SLC35A2	-	X:48903179-48911958	-	-	IGV_LINK	437.344	16.356	4.94018	27.7718	OK	642.453	24.6195	8.84798	40.3909	OK	OK	0.589982
ENSG0000010	-	-	ENSG0000010210	'PQBP1	-	X:48893446-48903143	-	-	IGV_LINK	846.566	57.4729	15.5309	99.4148	OK	654.828	44.2937	8.488	80.0994	OK	OK	-0.37578
ENSG0000010	-	-	ENSG0000010210	'RS1	-	X:18425582-18672109	-	-	IGV_LINK	0.969721	0.02072	0	0.302426	OK	1.03122	0.022044	0	0.26894	OK	NOTEST	0.089348
ENSG0000010	-	-	ENSG0000010210	'PCSK1N	-	X:48831093-48835633	-	-	IGV_LINK	109.578	7.03764	1.01005	13.0652	OK	160.871	10.3455	2.31126	18.3796	OK	OK	0.555834
ENSG0000010	-	-	ENSG0000010211	'EMD	-	X:154379196-154381523	-	-	IGV_LINK	638.076	39.9869	15.2404	64.7334	OK	556.861	35.2914	11.5383	59.0444	OK	OK	-0.18021
ENSG0000010	-	-	ENSG0000010212	'TAZ	-	X:154389954-154421726	-	-	IGV_LINK	841.717	41.2961	0	316.054	OK	755.888	38.3301	0	263.347	OK	OK	-0.10753
ENSG0000010	-	-	ENSG0000010212	'RAB40AL	-	X:102937271-102940561	-	-	IGV_LINK	0	0	0	0	OK	0	0	0	0	OK	NOTEST	0
ENSG0000010	-	-	ENSG0000010214	'PGK1	-	X:78065187-78129296	-	-	IGV_LINK	12152.5	423.345	0	974.877	OK	19606.7	703.162	0	1628.81	OK	OK	0.732023
ENSG0000010	-	-	ENSG0000010214	'GATA1	-	X:48786553-48794311	-	-	IGV_LINK	2.90916	0.123783	0	0.353237	OK	1.03122	0.043916	0	0.199983	OK	NOTEST	-1.49499

ENSG0000010	-	-	ENSG0000010215	'MAGT1	-	X:77826363-77895593	-	-	IGV_LINK	939.659	15.7708	5.70546	25.8361	OK	952.852	16.6531	6.502	26.8042	OK	OK	0.078536
ENSG0000010	-	-	ENSG0000010217	'SMS	-	X:21940572-21994835	-	-	IGV_LINK	1270.33	49.3932	18.2803	80.5062	OK	1710.8	67.7047	28.3694	107.04	OK	OK	0.454942
ENSG0000010	-	-	ENSG0000010217	'PHEX	-	X:22032440-22251310	-	-	IGV_LINK	30.0613	0.282378	0	0.645747	OK	55.6861	0.523181	0	1.09274	OK	NOTEST	0.889686
ENSG0000010	-	-	ENSG0000010217	'UBL4A	-	X:154483716-154486670	-	-	IGV_LINK	633.227	17.469	5.80712	29.1308	OK	520.769	14.4245	4.87028	23.9787	OK	OK	-0.27627
ENSG0000010	-	-	ENSG0000010218	'CD99L2	-	X:150766336-150898816	-	-	IGV_LINK	1129.72	19.7249	6.0239	33.4259	OK	1084.85	19.0161	6.51907	31.5132	OK	OK	-0.05279
ENSG0000010	-	-	ENSG0000010218	'EEA1	-	12:92721461-92929331	-	-	IGV_LINK	388.858	3.219	0	8.21704	OK	403.209	2.34226	0.772199	3.91231	OK	OK	-0.45871
ENSG0000010	-	-	ENSG0000010219	'GPR50	-	X:151175191-151181465	-	-	IGV_LINK	0	0	0	0	OK	0	0	0	0	OK	NOTEST	0
ENSG0000010	-	-	ENSG0000010221	'RP2	-	X:46836939-46882358	-	-	IGV_LINK	377.221	5.85555	2.01784	9.69327	OK	415.584	6.45309	2.36589	10.5403	OK	OK	0.140185
ENSG0000010	-	-	ENSG0000010222	'JADE3	-	X:46912275-47061242	-	-	IGV_LINK	193.944	2.39503	0.572246	4.21781	OK	245.432	3.32793	0.204889	6.45096	OK	OK	0.474581
ENSG0000010	-	-	ENSG0000010222	'CDK16	-	X:47217859-47229997	-	-	IGV_LINK	1372.15	45.7985	11.976	79.621	OK	1398.34	46.7528	11.331	82.1746	OK	OK	0.029754
ENSG0000010	-	-	ENSG0000010222	'USP11	-	X:47232689-47248328	-	-	IGV_LINK	777.716	21.398	1.07124	41.7249	OK	968.32	24.5517	1.26704	47.8364	OK	OK	0.198344
ENSG0000010	-	-	ENSG0000010223	'PCYT1B	-	X:24558086-24672677	-	-	IGV_LINK	0.969721	0.010644	0	0.186574	OK	2.06245	0.104216	0	0.375425	OK	NOTEST	3.29153
ENSG0000010	-	-	ENSG0000010223	'BRS3	-	X:136487886-136493780	-	-	IGV_LINK	0	0	0	0	OK	0	0	0	0	OK	NOTEST	0
ENSG0000010	-	-	ENSG0000010224	'HTATSF1	-	X:136497078-136512346	-	-	IGV_LINK	907.658	19.7732	8.07314	31.4733	OK	935.321	20.2722	8.60793	31.9364	OK	OK	0.035953
ENSG0000010	-	-	ENSG0000010224	'VGLL1	-	X:136532151-136556807	-	-	IGV_LINK	0	0	0	0	OK	0	0	0	0	OK	NOTEST	0
ENSG0000010	-	-	ENSG0000010224	'CD40LG	-	X:136648192-136660390	-	-	IGV_LINK	49.4557	1.69483	0	3.40169	OK	49.4988	1.69749	0	3.4055	OK	OK	0.002265
ENSG0000010	-	-	ENSG0000010226	'TIMP1	-	X:47561099-47630305	-	-	IGV_LINK	3967.13	313.33	94.3018	532.358	OK	4138.31	325.608	99.9276	551.288	OK	OK	0.055454
ENSG0000010	-	-	ENSG0000010227	'KLHL4	-	X:87517748-87670050	-	-	IGV_LINK	2.90916	0.072145	0	0.195505	OK	11.3435	0.153966	0	0.37136	OK	NOTEST	1.09365
ENSG0000010	-	-	ENSG0000010228	'GABRE	-	X:151953123-151974680	-	-	IGV_LINK	2279.81	41.3496	0	105.392	OK	2734.81	47.3152	0	110.951	OK	OK	0.19443
ENSG0000010	-	-	ENSG0000010229	'PCDH11X	-	X:91779260-92623230	-	-	IGV_LINK	0	0	0	0	OK	1.03122	0.006465	0	0.075247	OK	NOTEST	inf
ENSG0000010	-	-	ENSG0000010230	'FGD1	-	X:54440400-54496166	-	-	IGV_LINK	91.1537	1.25217	0	3.54743	OK	128.903	1.77123	0	4.24182	OK	OK	0.500318
ENSG0000010	-	-	ENSG0000010230	'PIN4	-	X:72181352-72307187	-	-	IGV_LINK	305.462	15.823	0	96.2923	OK	378.46	20.1385	0	103.719	OK	OK	0.347933
ENSG0000010	-	-	ENSG0000010231	'PORCN	-	X:48508961-48520814	-	-	IGV_LINK	116.366	5.94751	0	12.2923	OK	89.7166	3.35678	0.028748	6.68481	OK	OK	-0.82521
ENSG0000010	-	-	ENSG0000010231	'ITIH6	-	X:54748898-54798240	-	-	IGV_LINK	0	0	0	0	OK	0	0	0	0	OK	NOTEST	0
ENSG0000010	-	-	ENSG0000010231	'MAGED2	-	X:54807598-54816012	-	-	IGV_LINK	3594.75	120.872	28.4527	213.291	OK	4259.99	144.704	34.7975	254.61	OK	OK	0.259629
ENSG0000010	-	-	ENSG0000010231	'RBM3	-	X:48568013-48579066	-	-	IGV_LINK	2728.79	130.284	43.0432	217.525	OK	3362.82	158.021	43.44	272.601	OK	OK	0.278455
ENSG0000010	-	-	ENSG0000010234	'KLF8	-	X:56232420-56287889	-	-	IGV_LINK	1277.12	21.5428	6.10557	36.98	OK	549.643	10.5897	2.18753	18.9919	OK	OK	-1.02454
ENSG0000010	-	-	ENSG0000010235	'SRPX2	-	X:100644165-100732123	-	-	IGV_LINK	8.72748	0.539699	0	3.76584	OK	614.61	23.9703	0.319672	47.6209	OK	OK	5.47295
ENSG0000010	-	-	ENSG0000010236	'SYTL4	-	X:100644165-100732123	-	-	IGV_LINK	331.644	6.65708	1.16387	12.1503	OK	339.273	9.47174	0	33.002	OK	OK	0.508739
ENSG0000010	-	-	ENSG0000010238	'ZDHHC15	-	X:75368426-75523502	-	-	IGV_LINK	54.3043	0.544208	0	1.13226	OK	25.7806	0.254591	0	0.572929	OK	NOTEST	-1.09598
ENSG0000010	-	-	ENSG0000010238	'CENPI	-	X:101098217-101163681	-	-	IGV_LINK	24.243	0.442231	0	1.02748	OK	27.8431	0.75395	0	1.89256	OK	OK	0.769667
ENSG0000010	-	-	ENSG0000010238	'DRP2	-	X:101219768-101264497	-	-	IGV_LINK	9.69721	0.081986	0	0.193342	OK	4.1249	0.034864	0	0.105282	OK	NOTEST	-1.23366
ENSG0000010	-	-	ENSG0000010238	'TAF7L	-	X:101268252-101293057	-	-	IGV_LINK	3.87888	0.100809	0	0.285755	OK	1.03122	0.026814	0	0.144655	OK	NOTEST	-1.91055
ENSG0000010	-	-	ENSG0000010239	'PBDC1	-	X:76172935-76178204	-	-	IGV_LINK	325.826	18.2728	6.16544	30.3801	OK	378.46	21.4861	7.67117	35.3011	OK	OK	0.233709
ENSG0000010	-	-	ENSG0000010239	'GLA	-	X:101390823-101414133	-	-	IGV_LINK	426.677	25.402	0	109.333	OK	838.386	51.7709	0	163.056	OK	OK	1.0272
ENSG0000010	-	-	ENSG0000010240	'ARMCX3	-	X:101622796-101627843	-	-	IGV_LINK	925.113	26.744	6.11559	47.3724	OK	1196.22	34.3312	7.62066	61.0417	OK	OK	0.360304
ENSG0000010	-	-	ENSG0000010240	'BEX4	-	X:103215091-103217246	-	-	IGV_LINK	291.886	14.3721	4.66942	24.0748	OK	276.368	13.4926	4.28847	22.6968	OK	OK	-0.0911
ENSG0000010	-	-	ENSG0000010244	'RUBCNL	-	13:46341999-46438190	-	-	IGV_LINK	224.975	6.84454	1.33786	12.3512	OK	301.118	9.15525	1.97702	16.3335	OK	OK	0.419647
ENSG0000010	-	-	ENSG0000010245	'NALCN	-	13:100708324-101416492	-	-	IGV_LINK	3.87888	0.057437	0	0.367513	OK	3.09367	0.084853	0	0.413586	OK	NOTEST	0.562973
ENSG0000010	-	-	ENSG0000010246	'FGF14	-	13:101452592-102402457	-	-	IGV_LINK	307.401	1.36305	0.078	2.64811	OK	162.934	0.722532	0	1.5713	OK	OK	-0.91571
ENSG0000010	-	-	ENSG0000010246	'HTR2A	-	13:46831549-46897076	-	-	IGV_LINK	0	0	0	0	OK	0	0	0	0	OK	NOTEST	0
ENSG0000010	-	-	ENSG0000010247	'NDFIP2	-	13:79477363-79556075	-	-	IGV_LINK	517.831	14.032	1.43301	26.6309	OK	1264.28	41.1962	0	83.0702	OK	OK	1.55379
ENSG0000010	-	-	ENSG0000010252	'TNFSF13B	-	13:108251239-108308484	-	-	IGV_LINK	562.438	26.501	2.49669	50.5053	OK	569.236	24.0675	1.70324	46.4318	OK	OK	-0.13896
ENSG0000010	-	-	ENSG0000010253	'FNDC3A	-	13:48974966-49209779	-	-	IGV_LINK	1382.82	13.5299	5.80103	21.2588	OK	1248.81	12.3198	5.30685	19.3327	OK	OK	-0.13518
ENSG0000010	-	-	ENSG0000010253	'MLNR	-	13:49220337-49222377	-	-	IGV_LINK	2.90916	0.153728	0	0.412604	OK	0	0	0	0	OK	NOTEST	'-inf
ENSG0000010	-	-	ENSG0000010254	'CDADC1	-	13:49247924-49293485	-	-	IGV_LINK	206.55	6.11584	0	12.7189	OK	168.09	7.12295	0	14.8668	OK	OK	0.219924
ENSG0000010	-	-	ENSG0000010254	'CAB39L	-	13:49308649-49444126	-	-	IGV_LINK	172.61	4.61415	0.430168	8.79813	OK	143.34	3.80405	0.238425	7.36966	OK	OK	-0.27853
ENSG0000010	-	-	ENSG0000010255	'KLF5	-	13:73054975-73077542	-	-	IGV_LINK	33.9402	0.936323	0	2.5418	OK	91.779	3.15708	0	7.09054	OK	OK	1.75351
ENSG0000010	-	-	ENSG0000010257	'STK24	-	13:98142561-98578830	-	-	IGV_LINK	2242.96	44.2709	0	102.59	OK	2280.04	47.0341	3.80837	90.2598	OK	OK	0.087346
ENSG0000010	-	-	ENSG0000010257	'ACP5	-	19:11559373-11619135	-	-	IGV_LINK	831.05	43.6308	2.84731	84.4143	OK	1469.5	73.2517	14.9022	131.601	OK	OK	0.747516
ENSG0000010	-	-	ENSG0000010258	'DNAJC3	-	13:95677138-95794989	-	-	IGV_LINK	869.839	9.04687	3.78394	14.3098	OK	930.165	9.67633	3.99378	15.3589	OK	OK	0.097043
ENSG0000010	-	-	ENSG0000010259	'UGGT2	-	13:95801579-96053482	-	-	IGV_LINK	276.37	4.15104	0.070024	8.23205	OK	286.681	3.57188	1.00125	6.14252	OK	OK	-0.21679
ENSG0000010	-	-	ENSG0000010260	'ARHGEF7	-	13:111113811-111305737	-	-	IGV_LINK	1813.38	34.4594	1.0921	67.8266	OK	1652.02	30.9992	0.594306	61.4041	OK	OK	-0.15267
ENSG0000010	-	-	ENSG0000010267	'FGF9	-	13:21671382-21704498	-	-	IGV_LINK	0	0	0	0	OK	0	0	0	0	OK	NOTEST	0
ENSG0000010	-	-	ENSG0000010268	'SGCG	-	13:23180951-23325165	-	-	IGV_LINK	8.72748	0.338856	0	0.798255	OK	2.06245	0.080141	0	0.235569	OK	NOTEST	-2.08006
ENSG0000010	-	-	ENSG0000010269	'PARP4	-	13:24408156-24597676	-	-	IGV_LINK	2532.91	28.6359	9.50608	47.7658	OK	3671.16	39.709	15.5334	63.8847	OK	OK	0.471641
ENSG0000010	-	-	ENSG0000010271	'SUPT20H	-	13:36949774-37059713	-	-	IGV_LINK	941.599	25.8218	0	53.6397	OK	912.634	26.3421	0	54.3108	OK	OK	0.028782
ENSG0000010	-	-	ENSG0000010273	'MRPS31	-	13:40729134-40771173	-	-	IGV_LINK	315.159	16.1822	4.33206	28.0323	OK	286.681	15.0726	3.56969	26.5755	OK	OK	-0.10248
ENSG0000010	-	-	ENSG0000010274	'SLC25A15	-	13:40789411-40810111	-	-	IGV_LINK	32.9705	1.43468	0	3.04441	OK	28.8743	1.40136	0	3.28083	OK	OK	-0.0339
ENSG0000010	-	-	ENSG0000010275	'KPNA3	-	13:49699306-49793307	-	-	IGV_LINK	349.099	5.02598	0.545359	9.50661	OK	326.898	5.05765	0.104387	10.0109	OK	OK	0.00906
ENSG0000010	-	-	ENSG0000010275	'FLT1	-	13:28300343-28495145	-	-	IGV_LINK	5503.16	47.0054	18.8468	75.1639	OK	3933.09	33.3789	12.812	53.9458	OK	OK	-0.49389
ENSG0000010	-	-	ENSG0000010276	'RGCC	-	13:41457558-41470882	-	-	IGV_LINK	782.564	56.941	21.2774	92.6047	OK	550.674	39.4666	15.4745	63.4586	OK	OK	-0.52884
ENSG0000010	-	-	ENSG0000010276	'VWA8	-	13:41566836-41981565	-	-	IGV_LINK	517.831	4.23291	1.56254	6.90327	OK	339.273	3.08685	0	6.71406	OK	OK	-0.45551
ENSG0000010	-	-	ENSG0000010278	'DGKH	-	13:42040035-42256578	-	-	IGV_LINK	589.59	2.08394	0.196759	3.97113	OK	433.114	1.79239	0	4.40351	OK	OK	-0.21743
ENSG0000010	-	-	ENSG0000010278	'KATNAL1	-	13:30202629-30307484	-	-	IGV_LINK	399.525	5.09584	0	10.8993	OK	446.52	5.44021	0.566271	10.3141	OK	OK	0.094343
ENSG0000010	-	-	ENSG0000010278	'INTS6	-	13:51335772-51552364	-	-	IGV_LINK	537.225	10.0406	0	20.2634	OK	473.332	9.45665	0	19.3786	OK	OK	-0.08644
ENSG0000010	-	-	ENSG0000010279	'ACOD1	-	13:76948496-76958642	-	-	IGV_LINK	0.969721	0.028389	0	0.129136	OK	1.03122	0.030207	0	0.130933	OK	NOTEST	0.089542
ENSG0000010	-	-	ENSG0000010279	'DHRS12	-	13:51767992-51845175	-	-	IGV_LINK	270.552	16.6925	2.45509	30.9299	OK	257.806	16.9837	1.25424	32.7132	OK	OK	0.024955
ENSG0000010	-	-	ENSG0000010280	'MEDAG	-	13:30881932-30977623	-	-	IGV_LINK	44.6071	1.1419	0	2.37392	OK	79.4043	2.03373	0.08625	3.98121	OK	OK	0.832697
ENSG0000010	-	-	ENSG0000010280	'TSC22D1	-	13:44432142-44580432	-	-	IGV_LINK	2956.68	60.3939	17.5844	103.203	OK	7123.7	153.148	25.783	280.512	OK	OK	1.34245
ENSG0000010	-	-	ENSG0000010280	'CLN5	-	13:76990659-77327050	-	-	IGV_LINK	676.429	17.0658	0	49.404	OK	711.413	17.7701	0	47.0822	OK	OK	0.05834
ENSG0000010	-	-	ENSG0000010283	'OLFM4	-	13:53028758-53052057	-	-	IGV_LINK	0	0	0	0	OK	3.09367	0.064019	0	0.165397	OK	NOTEST	inf
ENSG0000010	-	-	ENSG0000010285	'MSLN	-	16:760761-768865	-	-	IGV_LINK	3.87888	0.258286	0	0.772688	OK	2.06245	0.12768	0	0.513253	OK	NOTEST	-1.01644
ENSG0000010	-	-	ENSG0000010285	'MGRN1	-	16:4616492-4690974	-	-	IGV_LINK	2160.54	38.0951	5.5327	70.6575	OK	2627.56	45.7631	7.85177	83.6745	OK	OK	0.264581
ENSG0000010	-	-	ENSG0000010287	'ZNF629	-	16:30778448-30787202	-	-	IGV_LINK	508.134	4.84169	1.82243	7.86095	OK	632.141	6.02445	2.439	9.60989	OK	OK	0.315319
ENSG0000010	-	-	ENSG0000010287	'TRADD	-	16:67154179-67169945	-	-	IGV_LINK	903.78	33.2933	0	91.4915	OK	1088.97	38.445	0	82.5316	OK	OK	0.207565
ENSG0000010	-	-	ENSG0000010287	'HSF4	-	16:67154179-67169945	-	-	IGV_LINK	4461.12	317.271	0	657.793	OK	2301.21	151.309	0	323.724	OK	OK	-1.06821
ENSG0000010	-	-	ENSG0000010287	'CORO1A	-	16:30182826-30189076	-	-	IGV_LINK	4013.67	141.906	42.5545	241.258	OK	2599.72	90.0084	30.1868	149.83	OK	OK	-0.65681
ENSG0000010	-	-	ENSG0000010288	'MAPK3	-	16:30114104-30123506	-	-	IGV_LINK	1588.4	84.8114	29.1183	140.505	OK	1511.78	79.9784	26.6958	133.261	OK	OK	-0.08465
ENSG0000010	-	-	ENSG0000010288	'GDPD3	-	16:30104809-30113856	-	-	IGV_LINK	131.882	6.28998	0.423353	12.1566	OK	74.2482	4.36069	0	12.5062	OK	OK	-0.5285
ENSG0000010	-	-	ENSG0000010289	'ELMO3	-	16:67199110-67204029	-	-	IGV_LINK	818.444	20.6948	8.29124	33.0983	OK	922.946	23.3094	9.22279	37.3961	OK	OK	0.171646
ENSG0000010	-	-	ENSG0000010289	'MT4	-	16:56565048-56568957	-	-	IGV_LINK	0	0	0	0	OK	0	0	0	0	OK	NOTEST	0
ENSG0000010	-	-	ENSG0000010289	'PHKB	-	16:47144322-47701523	-	-	IGV_LINK	1198.57	20.7858	0	43.5634	OK	1480.84	26.1963	0	56.4755	OK	OK	0.333763
ENSG0000010	-	-	ENSG0000010289	'LYRM1	-	16:20610242-20925006	-	-	IGV_LINK	360.736	23.0109	0	64.9987	OK	1142.6	61.4677	0	136.473	OK	OK	1.41751
ENSG0000010	-	-	ENSG0000010289	'NUTF2	-	16:67806764-67872567	-	-	IGV_LINK	615.773	34.9395	0	86.711	OK	678.546	39.6923	0	85.8901	OK	OK	0.183997
ENSG0000010	-	-	ENSG0000010290	'NUP93	-	16:56730104-56850286	-	-	IGV_LINK	513.952	17.2068	0.825331	33.5884	OK	433.114	16.1649	0.943423	31.3863	OK	OK	-0.09012
ENSG0000010	-	-	ENSG0000010290	'CENPT	-	16:67806764-67872567	-	-	IGV_LINK	1135.54	34.6424	0.793383	68.4914	OK	982.757	29.0496	0	58.8925	OK	OK	-0.25402
ENSG0000010	-	-	ENSG0000010290	'TSNAXIP1	-	16:67806764-67872567	-	-	IGV_LINK	147.398	4.40042	0	15.1878	OK	130.966	4.57555	0	18.3967	OK	OK	0.056304
ENSG0000010	-	-	ENSG0000010290	'NFAT5	-	16:69565093-69704666	-	-	IGV_LINK	1216.2	6.85569	0	15.867	OK	680.035	3.01821	0.726956	5.30946	OK	OK	-1.18361
ENSG0000010	-	-	ENSG0000010291	'LONP2	-	16:48164951-48448402	-	-	IGV_LINK	1618.46	20.4149	0	41.1551	OK	1650.99	21.5278	0.343503	42.7121	OK	OK	0.076578
ENSG0000010	-	-	ENSG0000010292	'N4BP1	-	16:48538725-48620148	-	-	IGV_LINK	1032.75	15.8509	0	34.8716	OK	1047.72	15.3141	0	33.5137	OK	OK	-0.04971
ENSG0000010	-	-	ENSG0000010292	'CBLN1	-	16:49277916-49281831	-	-	IGV_LINK	0	0	0	0	OK	30.9367	1.34179	0	3.09964	OK	OK	inf
ENSG0000010	-	-	ENSG0000010293	'ARL2BP	-	16:57245097-57284687	-	-	IGV_LINK	209.46	9.58601	0	28.8991	OK	272.243	9.50463	0	24.7551	OK	OK	-0.0123
ENSG0000010	-	-	ENSG0000010293	'PLLP	-	16:57245097-57284687	-	-	IGV_LINK	1057.97	38.875	0	87.3643	OK	1101.35	47.0319	0	108.979	OK	OK	0.274798
ENSG0000010	-	-	ENSG0000010293	'ZNF423	-	16:49487523-49857919	-	-	IGV_LINK	36.8494	0.397766	0	1.01438	OK	61.8735	1.0984	0	3.23313	OK	OK	1.46541
ENSG0000010	-	-	ENSG0000010296	'CCL22	-	16:57358771-57366190	-	-	IGV_LINK	62.0621	1.26884	0.018911	2.51878	OK	57.7486	1.18115	0.018504	2.34379	OK	OK	-0.10333
ENSG0000010	-	-	ENSG0000010296	'DHODH	-	16:72008587-72027664	-	-	IGV_LINK	265.703	6.76541	0.568322	12.9625	OK	489.832	15.926	1.74457	30.1074	OK	OK	1.23513
ENSG0000010	-	-	ENSG0000010297	'CCL17	-	16:57404766-57416062	-	-	IGV_LINK	15.5155	2.31634	0	4.98141	OK	2.06245	0.308842	0	0.965346	OK	OK	-2.90691
ENSG0000010	-	-	ENSG0000010297	'CTCF	-	16:67549213-67639183	-	-	IGV_LINK	804.868	12.0427	4.95249	19.133	OK	806.418	12.0696	4.9396	19.1996	OK	OK	0.003216
ENSG0000010	-	-	ENSG0000010297	'ACD	-	16:67644918-67660815	-	-	IGV_LINK	488.739	21.1864	3.62789	38.7448	OK	384.647	15.7177	2.51014	28.9252	OK	OK	-0.43075
ENSG0000010	-	-	ENSG0000010297	'POLR2C	-	16:57428168-57487327	-	-	IGV_LINK	1520.52	54.7875	0	120.4	OK	1729.36	65.7415	0	157.415	OK	OK	0.262958
ENSG0000010	-	-	ENSG0000010298	'PARD6A	-	16:67660945-67662778	-	-	IGV_LINK	39.7585	2.08582	0	4.23118	OK	48.4676	2.56794	0.001918	5.13396	OK	OK	0.299993
ENSG0000010	-	-	ENSG0000010298	'ZNF821	-	16:71845990-71999978	-	-	IGV_LINK	256.006	10.5118	0	40.1523	OK	181.496	7.18166	0	31.7144	OK	OK	-0.54962
ENSG0000010	-	-	ENSG0000010299	'MMP15	-	16:58025565-58046901	-	-	IGV_LINK	221.096	3.0558	0.766541	5.34507	OK	415.584	5.74546	2.04471	9.44621	OK	OK	0.910871
ENSG0000010	-	-	ENSG0000010300	'USB1	-	16:57994667-58021618	-	-	IGV_LINK	588.62	15.4081	2.71111	28.105	OK	751.763	20.9895	3.21257	38.7664	OK	OK	0.445983
ENSG0000010	-	-	ENSG0000010301	'CYB5B	-	16:69424524-69466266	-	-	IGV_LINK	1004.63	45.1619	10.5545	79.7692	OK	1105.47	51.0259	11.2041	90.8477	OK	OK	0.176125
ENSG0000010	-	-	ENSG0000010302	'CCDC113	-	16:58231156-58295047	-	-	IGV_LINK	70.7896	1.43864	0	3.60985	OK	110.341	1.93597	0	4.57552	OK	OK	0.428346
ENSG0000010	-	-	ENSG0000010302	'PRSS54	-	16:58231156-58295047	-	-	IGV_LINK	7.75776	0.316239	0	1.30269	OK	17.5308	0.715225	0	2.34106	OK	OK	1.17738
ENSG0000010	-	-	ENSG0000010302	'NME3	-	16:1706182-1771730	-	-	IGV_LINK	860.142	64.4772	0	167.604	OK	727.014	51.3819	0	126.198	OK	OK	-0.32753
ENSG0000010	-	-	ENSG0000010303	'NDRG4	-	16:58462845-58513628	-	-	IGV_LINK	32.0008	1.57331	0	4.39906	OK	44.3427	2.21483	0	5.87133	OK	OK	0.493389
ENSG0000010	-	-	ENSG0000010303	'PSMD7	-	16:74296774-74369826	-	-	IGV_LINK	599.287	39.2043	0	81.3197	OK	755.888	46.5437	5.17851	87.9088	OK	OK	0.247574
ENSG0000010	-	-	ENSG0000010303	'SETD6	-	16:58515478-58629886	-	-	IGV_LINK	238.551	11.0193	0	43.0601	OK	284.618	13.1538	0	48.5673	OK	OK	0.255442
ENSG0000010	-	-	ENSG0000010304	'SLC38A7	-	16:58665108-58685104	-	-	IGV_LINK	414.071	9.02778	0	18.3534	OK	592.954	11.0385	1.55146	20.5256	OK	OK	0.290105
ENSG0000010	-	-	ENSG0000010304	'VAC14	-	16:70687438-70801161	-	-	IGV_LINK	863.051	18.404	4.32164	32.4864	OK	1079.69	22.1366	6.93642	37.3368	OK	OK	0.266416
ENSG0000010	-	-	ENSG0000010304	'HAS3	-	16:69105563-69408571	-	-	IGV_LINK	32.9705	0.655727	0	10.4559	OK	80.4355	1.19487	0	8.20522	OK	OK	0.865681
ENSG0000010	-	-	ENSG0000010304	'TANGO6	-	16:68843603-69085180	-	-	IGV_LINK	256.976	3.37376	0	6.74765	OK	287.712	3.61129	0.667607	6.55496	OK	OK	0.098155
ENSG0000010	-	-	ENSG0000010305	'COG4	-	16:70480567-70523565	-	-	IGV_LINK	1734.83	37.7739	12.9953	62.5525	OK	1627.27	36.0622	13.5306	58.5937	OK	OK	-0.0669
ENSG0000010	-	-	ENSG0000010305	'SMPD3	-	16:68310973-68448688	-	-	IGV_LINK	92.2945	1.22219	0	3.79044	OK	75.2794	0.959736	0	2.94648	OK	OK	-0.34876
ENSG0000010	-	-	ENSG0000010306	'SLC7A6OS	-	16:68264515-68310946	-	-	IGV_LINK	1094.81	18.0656	0	36.5192	OK	1003.38	16.3294	0.341695	32.317	OK	OK	-0.14578
ENSG0000010	-	-	ENSG0000010306	'SLC7A6	-	16:68264515-68310946	-	-	IGV_LINK	358.797	22.5775	0	58.7245	OK	343.398	20.981	0	53.5916	OK	OK	-0.1058
ENSG0000010	-	-	ENSG0000010306	'PLA2G15	-	16:68245303-68261062	-	-	IGV_LINK	647.773	16.9604	5.21268	28.7081	OK	862.104	22.8078	7.54801	38.0675	OK	OK	0.427355
ENSG0000010	-	-	ENSG0000010306	'ESRP2	-	16:67987393-68238102	-	-	IGV_LINK	151.276	3.60961	0	13.2914	OK	232.026	5.59043	0	19.5391	OK	OK	0.631118
ENSG0000010	-	-	ENSG0000010308	'FA2H	-	16:74712954-74774831	-	-	IGV_LINK	21.3339	1.1146	0	3.74263	OK	14.4371	0.426495	0	1.18532	OK	OK	-1.38592
ENSG0000010	-	-	ENSG0000010309	'WDR59	-	16:74871366-75110994	-	-	IGV_LINK	1296.52	53.256	0	107.433	OK	1245.72	43.4383	0	88.472	OK	OK	-0.29398
ENSG0000010	-	-	ENSG0000010311	'MON1B	-	16:77190834-77444336	-	-	IGV_LINK	938.689	19.0362	3.08244	34.99	OK	1133.32	23.5504	2.70033	44.4005	OK	OK	0.307002
ENSG0000010	-	-	ENSG0000010312	'CMC2	-	16:80966447-81096425	-	-	IGV_LINK	316.835	31.8953	0	80.0559	OK	269.15	27.0767	0	71.4669	OK	OK	-0.23629
ENSG0000010	-	-	ENSG0000010312	'AXIN1	-	16:287439-352673	-	-	IGV_LINK	607.045	9.11587	3.38633	14.8454	OK	619.766	9.84233	1.20518	18.4795	OK	OK	0.110619
ENSG0000010	-	-	ENSG0000010314	'HCFC1R1	-	16:3022619-3039133	-	-	IGV_LINK	575.044	65.4945	7.15554	123.833	OK	1005.44	116.734	27.1775	206.29	OK	OK	0.833776
ENSG0000010	-	-	ENSG0000010314	'NPRL3	-	16:77006-138860	-	-	IGV_LINK	746.685	18.7385	2.61782	34.8591	OK	742.482	19.4916	1.20299	37.7801	OK	OK	0.056845
ENSG0000010	-	-	ENSG0000010315	'MLYCD	-	16:83899125-83966332	-	-	IGV_LINK	542.169	3.77482	0.502717	7.04692	OK	726.064	5.31782	0.510828	10.1248	OK	OK	0.494428
ENSG0000010	-	-	ENSG0000010315	'MPG	-	16:77006-138860	-	-	IGV_LINK	311.28	21.1208	0	44.3952	OK	386.709	26.0903	0	55.2396	OK	OK	0.304849
ENSG0000010	-	-	ENSG0000010315	'NECAB2	-	16:83968631-84002776	-	-	IGV_LINK	243.4	16.7734	0.624149	32.9227	OK	120.653	10.5971	0	21.784	OK	OK	-0.66251

ENSG0000010	-	-	ENSG0000010316	'HSDL1	-	16:84122145-84145192	-	-	IGV_LINK	321.947	8.77001	0	18.0564	OK	261.931	5.82008	0	11.9121	OK	OK	-0.59154
ENSG0000010	-	-	ENSG0000010316	'TAF1C	-	16:84145286-84187070	-	-	IGV_LINK	1479.79	37.5448	0	75.1248	OK	1538.59	43.6616	0	89.3847	OK	OK	0.217749
ENSG0000010	-	-	ENSG0000010317	'NAGPA	-	16:4882506-5097808	-	-	IGV_LINK	329.705	10.1588	0	27.5674	OK	471.27	13.1674	0	36.3136	OK	OK	0.374239
ENSG0000010	-	-	ENSG0000010317	'WFDC1	-	16:84294645-84329851	-	-	IGV_LINK	5.81832	0.210331	0	0.612719	OK	20.6245	0.831519	0	1.81777	OK	OK	1.98309
ENSG0000010	-	-	ENSG0000010318	'SEC14L5	-	16:4882506-5097808	-	-	IGV_LINK	11.6366	0.120627	0	1.33713	OK	46.4051	0.424893	0	2.65446	OK	NOTEST	1.81654
ENSG0000010	-	-	ENSG0000010318	'COTL1	-	16:84565593-84618077	-	-	IGV_LINK	3561.78	120.185	48.0216	192.349	OK	1979.95	66.4925	27.7738	105.211	OK	OK	-0.854
ENSG0000010	-	-	ENSG0000010319	'USP10	-	16:84699977-84779922	-	-	IGV_LINK	1133.6	24.6459	7.67939	41.6123	OK	1132.29	23.7398	8.37291	39.1067	OK	OK	-0.05404
ENSG0000010	-	-	ENSG0000010319	'CRISPLD2	-	16:84819983-84920768	-	-	IGV_LINK	675.895	9.43088	2.75113	16.1106	OK	252.65	3.30709	0.871352	5.74282	OK	OK	-1.51183
ENSG0000010	-	-	ENSG0000010319	'TSC2	-	16:2039814-2135898	-	-	IGV_LINK	1737.74	35.9587	0	80.5782	OK	1754.11	37.5157	0	85.3198	OK	OK	0.061155
ENSG0000010	-	-	ENSG0000010319	'ZNF500	-	16:4696509-4767624	-	-	IGV_LINK	377.221	8.84186	0	18.7976	OK	349.585	9.67643	0	21.7999	OK	OK	0.130125
ENSG0000010	-	-	ENSG0000010320	'AC069335.1	-	7:140435315-140435787	-	-	IGV_LINK	0.969721	0.182428	0	0.714526	OK	0	0	0	0	OK	NOTEST	'-inf
ENSG0000010	-	-	ENSG0000010320	'NME4	-	16:396724-412487	-	-	IGV_LINK	1222.25	76.7815	22.2545	131.309	OK	1649.52	104.967	34.4696	175.464	OK	OK	0.451098
ENSG0000010	-	-	ENSG0000010322	'ABCC1	-	16:15949576-16143074	-	-	IGV_LINK	1975.32	20.913	4.69019	37.1358	OK	1896.42	19.2884	4.90336	33.6734	OK	OK	-0.11667
ENSG0000010	-	-	ENSG0000010322	'NOMO3	-	16:16232494-16294814	-	-	IGV_LINK	133.821	6.68425	0	14.1644	OK	208.307	8.3388	0	17.9422	OK	OK	0.319075
ENSG0000010	-	-	ENSG0000010322	'LMF1	-	16:853633-981596	-	-	IGV_LINK	291.886	12.2135	1.88156	22.5455	OK	377.428	13.6762	2.55232	24.8002	OK	OK	0.16319
ENSG0000010	-	-	ENSG0000010324	'FOXF1	-	16:86510526-86515418	-	-	IGV_LINK	76.6079	1.29125	0	2.59809	OK	46.4051	0.782443	0	1.5697	OK	OK	-0.72271
ENSG0000010	-	-	ENSG0000010324	'NARFL	-	16:720580-741329	-	-	IGV_LINK	683.653	23.3654	2.66015	44.0707	OK	664.109	22.1373	4.8562	39.4185	OK	OK	-0.07789
ENSG0000010	-	-	ENSG0000010324	'MTHFSD	-	16:86530175-86555235	-	-	IGV_LINK	416.01	17.7442	2.09515	33.3932	OK	406.303	14.5275	2.80626	26.2488	OK	OK	-0.28856
ENSG0000010	-	-	ENSG0000010324	'CLCN7	-	16:1444933-1475580	-	-	IGV_LINK	1853.14	48.2835	5.59328	90.9738	OK	2107.82	59.1679	5.35818	112.978	OK	OK	0.293284
ENSG0000010	-	-	ENSG0000010325	'HAGHL	-	16:720580-741329	-	-	IGV_LINK	191.035	13.2308	0	34.8719	OK	94.8727	6.6293	0	20.6205	OK	OK	-0.99697
ENSG0000010	-	-	ENSG0000010325	'FAM173A	-	16:720580-741329	-	-	IGV_LINK	124.124	12.7553	0	37.1781	OK	124.778	10.4573	0	29.283	OK	OK	-0.28658
ENSG0000010	-	-	ENSG0000010325	'SLC7A5	-	16:87830022-87869488	-	-	IGV_LINK	337.463	4.61882	1.41056	7.82708	OK	822.918	11.7978	4.85606	18.7396	OK	OK	1.35292
ENSG0000010	-	-	ENSG0000010326	'METRN	-	16:715114-719655	-	-	IGV_LINK	113.457	5.76446	0	12.4734	OK	53.6237	2.26371	0	5.47884	OK	OK	-1.3485
ENSG0000010	-	-	ENSG0000010326	'FBXO31	-	16:87083561-87404779	-	-	IGV_LINK	660.163	14.9245	0	37.0929	OK	751.282	20.4561	0	55.4399	OK	OK	0.454851
ENSG0000010	-	-	ENSG0000010326	'STUB1	-	16:680223-684528	-	-	IGV_LINK	842.687	50.7389	0	103.275	OK	763.106	44.047	0.350198	87.7437	OK	OK	-0.20405
ENSG0000010	-	-	ENSG0000010326	'RHBDL1	-	16:675665-678268	-	-	IGV_LINK	32.0008	1.31974	0	2.93723	OK	35.0616	1.44302	0	3.0775	OK	OK	0.128831
ENSG0000010	-	-	ENSG0000010327	'NUBP1	-	16:10743785-10818794	-	-	IGV_LINK	410.192	24.7407	4.81673	44.6646	OK	413.521	24.4234	6.44683	42.3999	OK	OK	-0.01862
ENSG0000010	-	-	ENSG0000010327	'UBE2I	-	16:1305546-1327018	-	-	IGV_LINK	1433.25	58.636	12.8718	104.4	OK	1350.9	54.2242	15.3643	93.084	OK	OK	-0.11285
ENSG0000010	-	-	ENSG0000010331	'ZP2	-	16:21194846-21214510	-	-	IGV_LINK	0	0	0	0	OK	0	0	0	0	OK	NOTEST	0
ENSG0000010	-	-	ENSG0000010331	'MEFV	-	16:3242027-3256627	-	-	IGV_LINK	64.0016	1.33696	0	2.8173	OK	174.277	3.72704	0.231255	7.22283	OK	OK	1.47908
ENSG0000010	-	-	ENSG0000010331	'CRYM	-	16:21233664-21339037	-	-	IGV_LINK	56.2438	2.57664	0	6.35945	OK	86.6229	4.2564	0	10.4705	OK	OK	0.72414
ENSG0000010	-	-	ENSG0000010331	'EEF2K	-	16:22206281-22288738	-	-	IGV_LINK	528.498	4.6951	0.732055	8.65815	OK	612.548	6.61249	0	13.8303	OK	OK	0.494036
ENSG0000010	-	-	ENSG0000010332	'CAPN15	-	16:527716-554636	-	-	IGV_LINK	1014.33	17.7763	5.61115	29.9415	OK	1126.1	19.9745	4.69694	35.2521	OK	OK	0.168204
ENSG0000010	-	-	ENSG0000010333	'PIEZO1	-	16:88706462-88785211	-	-	IGV_LINK	2856.8	51.353	0	105.196	OK	2551.25	46.3817	0	102.004	OK	OK	-0.14689
ENSG0000010	-	-	ENSG0000010334	'GSPT1	-	16:11851648-11916082	-	-	IGV_LINK	1177.24	24.7652	8.3388	41.1915	OK	1457.12	34.1271	12.5226	55.7316	OK	OK	0.462605
ENSG0000010	-	-	ENSG0000010334	'ZNF174	-	16:3365098-3577400	-	-	IGV_LINK	200.732	6.81178	0	20.6531	OK	153.653	5.42934	0	18.9034	OK	OK	-0.32726
ENSG0000010	-	-	ENSG0000010335	'CLUAP1	-	16:3365098-3577400	-	-	IGV_LINK	470.813	18.2377	0	48.3925	OK	629.936	27.8364	0	69.6415	OK	OK	0.610049
ENSG0000010	-	-	ENSG0000010335	'UBFD1	-	16:23522013-23574389	-	-	IGV_LINK	1156.88	19.7555	0	40.4666	OK	1396.28	25.2399	0	52.4382	OK	OK	0.353454
ENSG0000010	-	-	ENSG0000010335	'PRSS33	-	16:2783952-2787948	-	-	IGV_LINK	0	0	0	0	OK	0	0	0	0	OK	NOTEST	0
ENSG0000010	-	-	ENSG0000010335	'EARS2	-	16:23522013-23574389	-	-	IGV_LINK	540.134	11.752	0	30.4842	OK	614.61	15.4416	0	41.0516	OK	OK	0.393914
ENSG0000010	-	-	ENSG0000010336	'ELOB	-	16:2737075-2777297	-	-	IGV_LINK	1768.77	242.133	0	1243.1	OK	1678.83	227.724	0	1105.59	OK	OK	-0.08851
ENSG0000010	-	-	ENSG0000010336	'GGA2	-	16:23463541-23521995	-	-	IGV_LINK	3769.3	82.8014	0	168.095	OK	2826.59	64.8373	0	129.815	OK	OK	-0.35283
ENSG0000010	-	-	ENSG0000010337	'AQP8	-	16:25215730-25228940	-	-	IGV_LINK	0.969721	0.049518	0	0.221967	OK	1.03122	0.052714	0	0.225441	OK	NOTEST	0.090233
ENSG0000010	-	-	ENSG0000010338	'CPPED1	-	16:12659798-12804017	-	-	IGV_LINK	642.925	12.5818	1.5547	23.609	OK	1405.56	29.9826	3.47877	56.4864	OK	OK	1.25278
ENSG0000010	-	-	ENSG0000010340	'USP31	-	16:23061405-23149270	-	-	IGV_LINK	487.768	3.22208	0.53865	5.90551	OK	397.021	2.92015	0.217516	5.62278	OK	OK	-0.14195
ENSG0000010	-	-	ENSG0000010341	'HMOX2	-	16:4461679-4510347	-	-	IGV_LINK	607.045	30.7112	0.000464	61.4219	OK	715.67	34.921	2.70946	67.1325	OK	OK	0.185328
ENSG0000010	-	-	ENSG0000010342	'DNAJA3	-	16:4425804-4456775	-	-	IGV_LINK	932.871	24.551	6.50782	42.5942	OK	1219.94	31.9391	8.36303	55.5151	OK	OK	0.379539
ENSG0000010	-	-	ENSG0000010342	'CORO7-PAM16	-	16:4314760-4425705	-	-	IGV_LINK	15.4024	0.278967	0	2.60076	OK	32.2751	0.532181	0	3.97423	OK	NOTEST	0.931821
ENSG0000010	-	-	ENSG0000010342	'BFAR	-	16:14632814-14669236	-	-	IGV_LINK	1121.97	32.6395	10.6436	54.6354	OK	1398.34	41.5505	13.6749	69.4262	OK	OK	0.348248
ENSG0000010	-	-	ENSG0000010344	'SALL1	-	16:51135974-51151367	-	-	IGV_LINK	681.714	11.692	0	23.5071	OK	1300.37	31.2214	0	62.8535	OK	OK	1.41702
ENSG0000010	-	-	ENSG0000010346	'TOX3	-	16:52438004-52547802	-	-	IGV_LINK	13.5761	0.400876	0	1.18005	OK	53.6237	1.22032	0	2.94681	OK	OK	1.60604
ENSG0000010	-	-	ENSG0000010347	'RRN3P2	-	16:29053731-29372110	-	-	IGV_LINK	78.2049	2.3226	0	5.52664	OK	84.2861	2.50686	0	5.35126	OK	OK	0.110138
ENSG0000010	-	-	ENSG0000010347	'RBL2	-	16:53433976-53504411	-	-	IGV_LINK	2946.98	54.2193	0.033966	108.405	OK	2913.21	50.6722	0.733528	100.611	OK	OK	-0.09761
ENSG0000010	-	-	ENSG0000010348	'QPRT	-	16:29662978-29698699	-	-	IGV_LINK	265.411	7.69903	0	17.6066	OK	63.9359	2.12436	0	6.80484	OK	OK	-1.85765
ENSG0000010	-	-	ENSG0000010348	'XYLT1	-	16:17101768-17470881	-	-	IGV_LINK	182.307	1.13354	0	2.38381	OK	166.027	0.962889	0.092937	1.83284	OK	OK	-0.23539
ENSG0000010	-	-	ENSG0000010349	'PYCARD	-	16:31201484-31203452	-	-	IGV_LINK	490.679	38.672	13.871	63.4731	OK	389.803	31.6512	10.2543	53.0482	OK	OK	-0.28903
ENSG0000010	-	-	ENSG0000010349	'RPGRIP1L	-	16:53597682-54158512	-	-	IGV_LINK	450.92	5.57447	0	15.8367	OK	272.243	4.0094	0	11.9616	OK	OK	-0.47545
ENSG0000010	-	-	ENSG0000010349	'MAZ	-	16:29806105-29848039	-	-	IGV_LINK	859.727	56.1117	0	196.375	OK	673.947	46.755	0	184.492	OK	OK	-0.26318
ENSG0000010	-	-	ENSG0000010349	'STX4	-	16:31032888-31042975	-	-	IGV_LINK	1510.82	74.9583	27.3024	122.614	OK	1678.83	81.7011	31.4814	131.921	OK	OK	0.124265
ENSG0000010	-	-	ENSG0000010350	'CDIPT	-	16:29858356-29868053	-	-	IGV_LINK	1723.19	69.0771	28.5635	109.591	OK	2149.07	88.1172	36.9785	139.256	OK	OK	0.351216
ENSG0000010	-	-	ENSG0000010350	'BCKDK	-	16:31106106-31112791	-	-	IGV_LINK	1480.76	47.3924	15.0019	79.7829	OK	2300.66	73.4845	25.1949	121.774	OK	OK	0.632783
ENSG0000010	-	-	ENSG0000010351	'KAT8	-	16:31115753-31131393	-	-	IGV_LINK	768.988	36.1583	10.7206	61.5959	OK	807.449	37.1707	12.5378	61.8036	OK	OK	0.039842
ENSG0000010	-	-	ENSG0000010351	'NOMO1	-	16:14833680-14896160	-	-	IGV_LINK	789.352	19.3328	1.2594	37.4062	OK	1514.87	40.147	0	83.3374	OK	OK	1.05424
ENSG0000010	-	-	ENSG0000010352	'IL21R	-	16:27402161-27453393	-	-	IGV_LINK	224.005	4.84175	0	11.9192	OK	179.433	3.2657	0	7.94787	OK	OK	-0.56814
ENSG0000010	-	-	ENSG0000010352	'SYT17	-	16:19113931-19268334	-	-	IGV_LINK	133.821	3.057	0	9.37893	OK	75.9706	1.33453	0	4.50373	OK	OK	-1.19579
ENSG0000010	-	-	ENSG0000010353	'TMC5	-	16:19410495-19499113	-	-	IGV_LINK	1.93944	0.031099	0	0.543097	OK	6.18735	0.256807	0	0.92267	OK	NOTEST	3.04575
ENSG0000010	-	-	ENSG0000010354	'CCP110	-	16:19523810-19553408	-	-	IGV_LINK	488.739	7.59184	1.24713	13.9366	OK	312.461	4.34924	0.510895	8.18759	OK	OK	-0.80369
ENSG0000010	-	-	ENSG0000010354	'C16orf62	-	16:19555239-19886167	-	-	IGV_LINK	779.655	18.4029	0	46.2938	OK	677.515	15.9322	0	36.8656	OK	OK	-0.20799
ENSG0000010	-	-	ENSG0000010354	'SLC6A2	-	16:55655603-55706422	-	-	IGV_LINK	0	0	0	0	OK	0	0	0	0	OK	NOTEST	0
ENSG0000010	-	-	ENSG0000010354	'RNF40	-	16:30697706-30776307	-	-	IGV_LINK	1564.16	42.7294	0	113.007	OK	2195.48	69.2819	0	179.292	OK	OK	0.697249
ENSG0000010	-	-	ENSG0000010355	'KNOP1	-	16:19555239-19886167	-	-	IGV_LINK	503.285	14.9917	0	45.2782	OK	472.301	14.5349	0	36.0973	OK	OK	-0.04464
ENSG0000010	-	-	ENSG0000010356	'AQP9	-	15:57953423-58569843	-	-	IGV_LINK	81.4565	1.62218	0	6.14344	OK	5259.25	104.618	25.545	183.692	OK	OK	6.01106
ENSG0000010	-	-	ENSG0000010359	'AAGAB	-	15:67201032-67530143	-	-	IGV_LINK	562.438	14.4029	4.00177	24.804	OK	568.205	14.8644	3.87195	25.8569	OK	OK	0.045504
ENSG0000010	-	-	ENSG0000010359	'IQCH	-	15:67201032-67530143	-	-	IGV_LINK	54.3043	1.99683	0	8.62804	OK	30.9367	1.38717	0	7.66102	OK	OK	-0.52557
ENSG0000010	-	-	ENSG0000010364	'LACTB	-	15:63121799-63158021	-	-	IGV_LINK	405.343	13.1074	0	33.6073	OK	438.271	14.8299	0	37.9751	OK	OK	0.178126
ENSG0000010	-	-	ENSG0000010364	'CORO2B	-	15:68578968-68727806	-	-	IGV_LINK	12.6064	0.224857	0	0.527287	OK	35.0616	0.625618	0	1.27851	OK	OK	1.47627
ENSG0000010	-	-	ENSG0000010365	'CSK	-	15:74782056-74803198	-	-	IGV_LINK	1616.52	58.1122	19.4418	96.7826	OK	1205.5	43.0973	12.4396	73.755	OK	OK	-0.43124
ENSG0000010	-	-	ENSG0000010365	'HERC1	-	15:63608617-63833942	-	-	IGV_LINK	1629.13	28.4397	0	71.6531	OK	1178.69	26.1154	0	62.392	OK	OK	-0.12301
ENSG0000010	-	-	ENSG0000010367	'TRIP4	-	15:64387747-64455303	-	-	IGV_LINK	513.952	16.6667	4.94441	28.3889	OK	563.049	18.8711	6.35776	31.3845	OK	OK	0.179214
ENSG0000010	-	-	ENSG0000010370	'MTFMT	-	15:65001511-65029639	-	-	IGV_LINK	115.397	4.42024	0.290521	8.54995	OK	123.747	4.44441	0.225952	8.66287	OK	OK	0.007868
ENSG0000010	-	-	ENSG0000010371	'RASL12	-	15:65045369-65076690	-	-	IGV_LINK	351.039	8.14013	2.68077	13.5995	OK	128.903	2.94546	0.475877	5.41505	OK	OK	-1.46656
ENSG0000010	-	-	ENSG0000010372	'AP3B2	-	15:82536752-82709914	-	-	IGV_LINK	25.2127	1.00544	0	20.3201	OK	90.7478	2.79484	0	34.5686	OK	OK	1.47494
ENSG0000010	-	-	ENSG0000010374	'ACSBG1	-	15:78077807-78245688	-	-	IGV_LINK	288.977	2.63327	0.154923	5.11161	OK	320.711	2.92299	0.264786	5.58119	OK	OK	0.150589
ENSG0000010	-	-	ENSG0000010374	'IGDCC4	-	15:65381463-65423072	-	-	IGV_LINK	32.0008	0.635381	0	2.06525	OK	68.0608	0.711366	0	1.63513	OK	OK	0.16297
ENSG0000010	-	-	ENSG0000010376	'RAB11A	-	15:65611365-65891991	-	-	IGV_LINK	1379.91	92.6827	4.32747	181.038	OK	1738.65	130.904	4.57746	257.23	OK	OK	0.498136
ENSG0000010	-	-	ENSG0000010381	'CTSH	-	15:78921057-78949574	-	-	IGV_LINK	4987.27	349.793	112.587	587	OK	3528.85	244.901	86.5847	403.218	OK	OK	-0.5143
ENSG0000010	-	-	ENSG0000010383	'GOLGA8UP	-	15:30791572-30801859	-	-	IGV_LINK	1.93944	0.069068	0	0.211517	OK	3.09367	0.110253	0	0.285556	OK	NOTEST	0.67473
ENSG0000010	-	-	ENSG0000010385	'TTC23	-	15:99136322-99251223	-	-	IGV_LINK	319.038	6.40965	0.730475	12.0888	OK	365.054	10.458	0	21.8906	OK	OK	0.706283
ENSG0000010	-	-	ENSG0000010385	'CD276	-	15:73683965-73714518	-	-	IGV_LINK	819.414	21.5383	6.54565	36.5309	OK	829.105	20.8106	5.4255	36.1956	OK	OK	-0.04959
ENSG0000010	-	-	ENSG0000010387	'FAH	-	15:80152489-80186946	-	-	IGV_LINK	569.226	25.4621	5.96115	44.963	OK	1327.19	56.3178	19.3358	93.2999	OK	OK	1.14524
ENSG0000010	-	-	ENSG0000010388	'CEMIP	-	15:80779342-80989878	-	-	IGV_LINK	23.2733	1.55342	0	8.63459	OK	9.28102	0.632547	0	6.07802	OK	OK	-1.29621
ENSG0000010	-	-	ENSG0000010393	'RPAP1	-	15:41517175-41544269	-	-	IGV_LINK	540.134	9.71657	0.046519	19.3866	OK	646.578	11.3365	0	22.6863	OK	OK	0.22246
ENSG0000010	-	-	ENSG0000010394	'HOMER2	-	15:82836945-83103443	-	-	IGV_LINK	45.5769	2.53328	0	23.3384	OK	51.5612	3.33929	0	33.5293	OK	OK	0.398533
ENSG0000010	-	-	ENSG0000010396	'EHD4	-	15:41828084-41972578	-	-	IGV_LINK	967.781	8.80016	0	17.803	OK	1120.94	10.1563	1.98239	18.3302	OK	OK	0.206774
ENSG0000010	-	-	ENSG0000010397	'TMEM87A	-	15:42208412-42412318	-	-	IGV_LINK	1003.66	22.905	0	50.2157	OK	1229.22	27.0062	0	57.4649	OK	OK	0.237626
ENSG0000010	-	-	ENSG0000010399	'ZNF106	-	15:42412822-42491123	-	-	IGV_LINK	1545.73	10.8543	1.67318	20.0355	OK	992.038	6.41483	1.11613	11.7135	OK	OK	-0.75879
ENSG0000010	-	-	ENSG0000010399	'CEP152	-	15:48712927-48811909	-	-	IGV_LINK	306.432	3.73202	0	9.92465	OK	155.715	1.66867	0	3.65243	OK	OK	-1.16126
ENSG0000010	-	-	ENSG0000010404	'ATP8B4	-	15:49858237-50236395	-	-	IGV_LINK	218.187	3.72982	0	9.08766	OK	308.336	4.59869	0.442055	8.75533	OK	OK	0.302119
ENSG0000010	-	-	ENSG0000010404	'OCA2	-	15:27754874-28099358	-	-	IGV_LINK	68.8502	1.28758	0	2.67773	OK	25.7806	0.482315	0	1.05498	OK	OK	-1.41661
ENSG0000010	-	-	ENSG0000010404	'DTWD1	-	15:49620979-49656232	-	-	IGV_LINK	306.432	7.30268	0	16.0579	OK	238.213	6.36021	0	13.8489	OK	OK	-0.19935
ENSG0000010	-	-	ENSG0000010405	'TGM5	-	15:43232594-43266857	-	-	IGV_LINK	1.93944	0.073826	0	0.459097	OK	2.06245	0.049747	0	0.421259	OK	NOTEST	-0.56953
ENSG0000010	-	-	ENSG0000010405	'FAM189A1	-	15:29120253-29570723	-	-	IGV_LINK	283.158	3.52071	0.573283	6.46813	OK	177.371	2.3859	0	4.94005	OK	OK	-0.56133
ENSG0000010	-	-	ENSG0000010406	'GABPB1	-	15:50275391-50372202	-	-	IGV_LINK	597.348	9.55888	0	24.0952	OK	452.708	8.81181	0	23.2039	OK	OK	-0.1174
ENSG0000010	-	-	ENSG0000010406	'TJP1	-	15:29699366-29968865	-	-	IGV_LINK	1181.12	16.5763	0.353606	32.799	OK	927.071	13.1379	1.05243	25.2235	OK	OK	-0.33538
ENSG0000010	-	-	ENSG0000010408	'BMF	-	15:40087889-40108892	-	-	IGV_LINK	776.746	13.4661	0	27.7521	OK	655.859	10.7503	0	21.9939	OK	OK	-0.32496
ENSG0000010	-	-	ENSG0000010409	'DMXL2	-	15:51414096-51622833	-	-	IGV_LINK	1023.06	17.5282	0	41.5598	OK	982.757	20.3512	0	45.8502	OK	OK	0.21544
ENSG0000010	-	-	ENSG0000010411	'SCG3	-	15:51681352-51721031	-	-	IGV_LINK	1.93944	0.034783	0	0.190421	OK	2.06245	0.037002	0	0.19289	OK	NOTEST	0.089241
ENSG0000010	-	-	ENSG0000010412	'DNAJC17	-	15:40764019-40815084	-	-	IGV_LINK	270.552	27.0154	0	68.1218	OK	262.962	25.6444	0	61.8374	OK	OK	-0.07514
ENSG0000010	-	-	ENSG0000010413	'EIF3J	-	15:44537056-44663678	-	-	IGV_LINK	515.891	22.3289	0	58.7527	OK	634.203	29.6275	0	72.5296	OK	OK	0.408019
ENSG0000010	-	-	ENSG0000010413	'SPG11	-	15:44537056-44663678	-	-	IGV_LINK	2027.69	35.5668	0	80.9222	OK	1789.18	41.2523	0	96.5629	OK	OK	0.213944
ENSG0000010	-	-	ENSG0000010414	'RHOV	-	15:40872213-40874289	-	-	IGV_LINK	435.405	16.0116	5.94706	26.0762	OK	282.556	10.3985	3.1352	17.6619	OK	OK	-0.62274
ENSG0000010	-	-	ENSG0000010414	'VPS18	-	15:40894429-40903975	-	-	IGV_LINK	412.131	6.3458	2.2087	10.4829	OK	688.858	10.7325	4.14076	17.3242	OK	OK	0.758107
ENSG0000010	-	-	ENSG0000010414	'OIP5	-	15:41283989-41332621	-	-	IGV_LINK	30.0613	1.59301	0	8.95569	OK	59.811	3.17293	0	12.9019	OK	OK	0.994064
ENSG0000010	-	-	ENSG0000010415	'SLC30A4	-	15:45430528-45586304	-	-	IGV_LINK	127.033	1.21651	0	2.74551	OK	112.404	1.0998	0	2.52148	OK	OK	-0.1455
ENSG0000010	-	-	ENSG0000010416	'BLOC1S6	-	15:45587122-45691294	-	-	IGV_LINK	1152.03	25.7201	0	60.6221	OK	1157.03	24.9897	0	60.0487	OK	OK	-0.04156
ENSG0000010	-	-	ENSG0000010417	'MYEF2	-	15:48120971-48178517	-	-	IGV_LINK	1190.82	35.531	6.80427	64.2577	OK	646.578	20.4021	3.72277	37.0813	OK	OK	-0.80036
ENSG0000010	-	-	ENSG0000010420	'SGK3	-	8:66667595-66862022	-	-	IGV_LINK	264.85	5.48451	0	12.4575	OK	300.986	6.12005	0	13.7171	OK	OK	0.15818
ENSG0000010	-	-	ENSG0000010421	'PDGFRL	-	8:17576432-17800917	-	-	IGV_LINK	18.4247	0.600576	0	2.53193	OK	275.337	8.98296	0	18.0025	OK	OK	3.90277
ENSG0000010	-	-	ENSG0000010421	'CSPP1	-	8:66964098-67343677	-	-	IGV_LINK	317.099	6.90659	0	23.686	OK	274.306	4.77944	0	17.3088	OK	OK	-0.53113
ENSG0000010	-	-	ENSG0000010421	'ZDHHC2	-	8:17156028-17224799	-	-	IGV_LINK	507.164	14.4621	0	33.6416	OK	545.518	15.6158	0	36.0534	OK	OK	0.11073
ENSG0000010	-	-	ENSG0000010422	'BRF2	-	8:37784190-37849904	-	-	IGV_LINK	433.465	10.1361	1.01836	19.2538	OK	405.271	9.82648	1.57845	18.0745	OK	OK	-0.04475
ENSG0000010	-	-	ENSG0000010422	'TRIM35	-	8:27284886-27311319	-	-	IGV_LINK	352.009	5.20014	1.43128	8.96901	OK	513.55	8.75794	0	17.8984	OK	OK	0.752041
ENSG0000010	-	-	ENSG0000010423	'ZFAND1	-	8:81701333-81759515	-	-	IGV_LINK	823.293	49.9291	10.7491	89.1091	OK	737.326	46.6771	3.56249	89.7917	OK	OK	-0.09717
ENSG0000010	-	-	ENSG0000010423	'RP1	-	8:54554360-54871720	-	-	IGV_LINK	25.2127	3.15033	0	11.1324	OK	43.3114	4.22908	0	14.9779	OK	OK	0.424838
ENSG0000010	-	-	ENSG0000010426	'CA2	-	8:85327607-85481493	-	-	IGV_LINK	1936.53	79.2219	30.6245	127.819	OK	3652.6	157.093	56.0841	258.103	OK	OK	0.987651
ENSG0000010	-	-	ENSG0000010429	'FZD3	-	8:28494204-28574268	-	-	IGV_LINK	108.609	0.798541	0	2.88228	OK	65.9984	0.990411	0	3.52019	OK	OK	0.310661
ENSG0000010	-	-	ENSG0000010429	'INTS9	-	8:28767660-28890242	-	-	IGV_LINK	315.159	9.16414	1.28547	17.0428	OK	405.271	12.1732	2.1158	22.2306	OK	OK	0.409636
ENSG0000010	-	-	ENSG0000010431	'RIPK2	-	8:89757746-89791063	-	-	IGV_LINK	388.858	10.5712	3.42655	17.7159	OK	354.741	10.1737	2.97237	17.3751	OK	OK	-0.0553
ENSG0000010	-	-	ENSG0000010431	'EYA1	-	8:71155456-71362232	-	-	IGV_LINK	121.215	2.55618	0	5.43186	OK	319.68	5.36614	0.845336	9.88695	OK	OK	1.0699
ENSG0000010	-	-	ENSG0000010432	'NBN	-	8:89933335-90052092	-	-	IGV_LINK	1376.03	20.137	0	44.2752	OK	1692.24	22.9046	0.139204	45.6699	OK	OK	0.185784
ENSG0000010	-	-	ENSG0000010432	'TRPA1	-	8:71828166-72118393	-	-	IGV_LINK	170.671	10.7373	0	33.9918	OK	230.994	21.2158	0	95.5097	OK	OK	0.982513
ENSG0000010	-	-	ENSG0000010432	'CPQ	-	8:96645226-97149654	-	-	IGV_LINK	1782.35	57.5291	24.8117	90.2464	OK	1623.15	52.9081	22.1612	83.655	OK	OK	-0.1208

ENSG0000010	-	-	ENSG0000010432	'DECR1	-	8:89933335-90052092	-	-	IGV_LINK	1189.85	78.1925	9.50928	146.876	OK	1233.35	78.1911	3.19289	153.189	OK	OK	-2.71E-05
ENSG0000010	-	-	ENSG0000010432	'CALB1	-	8:90058607-90095475	-	-	IGV_LINK	23.2733	0.540689	0	1.77357	OK	16.4996	0.421239	0	1.4757	OK	NOTEST	-0.36016
ENSG0000010	-	-	ENSG0000010433	'IMPAD1	-	8:56957932-56993844	-	-	IGV_LINK	766.079	6.7001	0.866275	12.5339	OK	1303.47	10.9374	3.02298	18.8518	OK	OK	0.707011
ENSG0000010	-	-	ENSG0000010433	'SFRP1	-	8:41261957-41309497	-	-	IGV_LINK	1.93944	0.029535	0	0.096027	OK	4.1249	0.053832	0	0.141879	OK	NOTEST	0.866029
ENSG0000010	-	-	ENSG0000010434	'LAPTM4B	-	8:97775056-97853013	-	-	IGV_LINK	1169.48	32.1587	10.3885	53.9288	OK	1704.61	44.6919	16.1022	73.2817	OK	OK	0.474807
ENSG0000010	-	-	ENSG0000010434	'UBE2W	-	8:73420003-73878910	-	-	IGV_LINK	465.676	7.02667	0	15.8488	OK	447.587	6.6802	0	14.1128	OK	OK	-0.07295
ENSG0000010	-	-	ENSG0000010435	'POP1	-	8:98117296-98159834	-	-	IGV_LINK	189.095	3.51903	0	7.72135	OK	384.647	6.57453	0.407842	12.7412	OK	OK	0.901711
ENSG0000010	-	-	ENSG0000010436	'NIPAL2	-	8:98189832-98294393	-	-	IGV_LINK	968.751	15.1568	0	30.4343	OK	1077.63	16.1219	2.79696	29.4468	OK	OK	0.089056
ENSG0000010	-	-	ENSG0000010436	'IKBKB	-	8:42271301-42332653	-	-	IGV_LINK	1991.81	43.8275	0	89.4078	OK	1823.21	39.8978	1.59668	78.199	OK	OK	-0.13553
ENSG0000010	-	-	ENSG0000010436	'PLAT	-	8:42175232-42207724	-	-	IGV_LINK	533.346	13.2946	3.67132	22.918	OK	199.026	4.59979	1.02833	8.17126	OK	OK	-1.5312
ENSG0000010	-	-	ENSG0000010436	'JPH1	-	8:74234699-74488872	-	-	IGV_LINK	16.4852	0.29593	0	1.08241	OK	14.4371	0.418898	0	1.92527	OK	NOTEST	0.501346
ENSG0000010	-	-	ENSG0000010437	'DKK4	-	8:42374067-42377232	-	-	IGV_LINK	0	0	0	0	OK	0	0	0	0	OK	NOTEST	0
ENSG0000010	-	-	ENSG0000010437	'STK3	-	8:98401402-98942827	-	-	IGV_LINK	509.103	11.9218	3.23458	20.6089	OK	491.894	12.9049	2.25777	23.552	OK	OK	0.114319
ENSG0000010	-	-	ENSG0000010438	'GDAP1	-	8:74234699-74488872	-	-	IGV_LINK	86.3051	2.30945	0	6.89003	OK	147.465	4.051	0	9.86972	OK	OK	0.810729
ENSG0000010	-	-	ENSG0000010438	'RAB2A	-	8:60516856-60623627	-	-	IGV_LINK	986.206	55.8013	11.9228	99.6798	OK	1306.56	77.2411	17.7384	136.744	OK	OK	0.46907
ENSG0000010	-	-	ENSG0000010440	'EIF3E	-	8:108201215-108435333	-	-	IGV_LINK	6424.4	277.161	103.899	450.422	OK	5290.18	227.377	87.2594	367.494	OK	OK	-0.28564
ENSG0000010	-	-	ENSG0000010441	'EMC2	-	8:108443600-108486918	-	-	IGV_LINK	1024.02	57.5566	23.4315	91.6818	OK	946.665	50.2996	20.5548	80.0444	OK	OK	-0.19444
ENSG0000010	-	-	ENSG0000010441	'ESRP1	-	8:94641073-94707466	-	-	IGV_LINK	0	0	0	0	OK	0	0	0	0	OK	NOTEST	0
ENSG0000010	-	-	ENSG0000010441	'WISP1	-	8:133191038-133230344	-	-	IGV_LINK	99.8446	1.53257	0.081199	2.98393	OK	37.346	0.573426	0	1.28775	OK	OK	-1.41827
ENSG0000010	-	-	ENSG0000010441	'NDRG1	-	8:133237170-133302022	-	-	IGV_LINK	59033.7	1475.37	0	3784.94	OK	63343	1636.83	0	4282.3	OK	OK	0.149833
ENSG0000010	-	-	ENSG0000010442	'ZC2HC1A	-	8:78666046-78805523	-	-	IGV_LINK	302.553	17.2146	0	35.8302	OK	186.652	10.3225	0	23.8457	OK	OK	-0.73784
ENSG0000010	-	-	ENSG0000010443	'IL7	-	8:78666046-78805523	-	-	IGV_LINK	119.276	4.93754	0	11.6005	OK	176.339	7.58547	0	16.2683	OK	OK	0.619448
ENSG0000010	-	-	ENSG0000010443	'STMN2	-	8:79610813-79666175	-	-	IGV_LINK	0	0	0	0	OK	0	0	0	0	OK	NOTEST	0
ENSG0000010	-	-	ENSG0000010444	'ARMC1	-	8:65602455-65634217	-	-	IGV_LINK	306.432	8.89503	1.57187	16.2182	OK	373.303	10.6076	1.87757	19.3377	OK	OK	0.254032
ENSG0000010	-	-	ENSG0000010444	'TRPS1	-	8:115408495-115809673	-	-	IGV_LINK	601.227	7.73606	0	16.4251	OK	544.487	5.4425	0	11.9021	OK	OK	-0.50733
ENSG0000010	-	-	ENSG0000010445	'SPAG1	-	8:100157905-100336218	-	-	IGV_LINK	327.766	6.71271	0	20.7995	OK	398.053	7.64168	0	26.6526	OK	OK	0.186993
ENSG0000010	-	-	ENSG0000010447	'CHRAC1	-	8:140511297-140517137	-	-	IGV_LINK	388.858	16.7485	0	34.5741	OK	498.082	21.2287	0	42.5252	OK	OK	0.341983
ENSG0000010	-	-	ENSG0000010449	'NCALD	-	8:101686542-102124907	-	-	IGV_LINK	251.158	5.91908	0	12.5771	OK	788.887	19.2863	1.44731	37.1252	OK	OK	1.70413
ENSG0000010	-	-	ENSG0000010449	'SNX16	-	8:81791822-81842866	-	-	IGV_LINK	112.488	3.89116	0.013361	7.76896	OK	129.934	4.54048	0	9.10561	OK	OK	0.222644
ENSG0000010	-	-	ENSG0000010449	'GML	-	8:142834137-142917843	-	-	IGV_LINK	0	0	0	0	OK	0	0	0	0	OK	NOTEST	0
ENSG0000010	-	-	ENSG0000010451	'UBR5	-	8:102239393-102412841	-	-	IGV_LINK	2209.02	33.1751	0	71.1833	OK	1546.84	34.1405	0	68.8896	OK	OK	0.041381
ENSG0000010	-	-	ENSG0000010451	'GSDMD	-	8:143553206-143563062	-	-	IGV_LINK	2175.08	80.726	26.0551	135.397	OK	2430.6	84.8386	31.3084	138.369	OK	OK	0.071688
ENSG0000010	-	-	ENSG0000010452	'TSTA3	-	8:143612617-143618048	-	-	IGV_LINK	723.411	37.0152	14.5721	59.4582	OK	674.421	34.6316	13.1261	56.1371	OK	OK	-0.09603
ENSG0000010	-	-	ENSG0000010452	'PYCR3	-	8:143603912-143609773	-	-	IGV_LINK	149.337	3.96601	0.725862	7.20617	OK	254.713	6.57942	1.8706	11.2882	OK	OK	0.730271
ENSG0000010	-	-	ENSG0000010452	'EEF1D	-	8:143573489-143603224	-	-	IGV_LINK	9343.26	657.348	100.021	1214.68	OK	6908.18	474.143	90.6851	857.6	OK	OK	-0.47134
ENSG0000010	-	-	ENSG0000010453	'ANXA13	-	8:123680793-123737407	-	-	IGV_LINK	448.011	19.0123	5.83165	32.1929	OK	179.433	7.35948	1.67382	13.0451	OK	OK	-1.36925
ENSG0000010	-	-	ENSG0000010454	'SQLE	-	8:124998496-125022283	-	-	IGV_LINK	255.036	5.90803	1.20926	10.6068	OK	506.331	10.4751	3.88345	17.0667	OK	OK	0.826213
ENSG0000010	-	-	ENSG0000010461	'SH2D4A	-	8:19313616-19396218	-	-	IGV_LINK	287.037	8.17585	0	16.6748	OK	426.927	13.2836	0.106063	26.4611	OK	OK	0.700206
ENSG0000010	-	-	ENSG0000010461	'INTS10	-	8:19817139-19852083	-	-	IGV_LINK	1030.81	44.0118	0	94.2783	OK	969.351	34.066	7.83089	60.3011	OK	OK	-0.36956
ENSG0000010	-	-	ENSG0000010462	'ERI1	-	8:9002146-9116746	-	-	IGV_LINK	162.913	4.08571	0	8.41115	OK	225.838	4.56658	0.588512	8.54464	OK	OK	0.160526
ENSG0000010	-	-	ENSG0000010463	'SLC39A14	-	8:22367248-22434129	-	-	IGV_LINK	4460.71	64.3762	13.6785	115.074	OK	18109.3	259.119	0	525.207	OK	OK	2.00902
ENSG0000010	-	-	ENSG0000010464	'MTMR9	-	8:11283480-11328146	-	-	IGV_LINK	223.036	2.0967	0.205654	3.98774	OK	207.276	1.72575	0.309957	3.14154	OK	OK	-0.2809
ENSG0000010	-	-	ENSG0000010466	'LEPROTL1	-	8:30095397-30183640	-	-	IGV_LINK	1782.35	71.4094	0.93867	141.88	OK	952.852	45.5362	0	94.9254	OK	OK	-0.6491
ENSG0000010	-	-	ENSG0000010467	'DCTN6	-	8:30095397-30183640	-	-	IGV_LINK	746.685	45.2968	0	95.7841	OK	585.736	34.8692	0	69.8342	OK	OK	-0.37746
ENSG0000010	-	-	ENSG0000010467	'R3HCC1	-	8:23270119-23296279	-	-	IGV_LINK	702.078	28.7461	9.91628	47.5759	OK	773.419	30.7078	11.6225	49.7931	OK	OK	0.095238
ENSG0000010	-	-	ENSG0000010468	'GSR	-	8:30678060-30727926	-	-	IGV_LINK	807.777	19.5471	6.71653	32.3776	OK	2521.35	59.2329	19.9094	98.5563	OK	OK	1.59944
ENSG0000010	-	-	ENSG0000010468	'TNFRSF10A	-	8:23189278-23230926	-	-	IGV_LINK	219.157	6.22376	0.712683	11.7348	OK	367.116	11.1387	1.42651	20.851	OK	OK	0.839726
ENSG0000010	-	-	ENSG0000010469	'UBXN8	-	8:30732246-30767006	-	-	IGV_LINK	164.852	10.9976	1.35463	20.6405	OK	251.619	15.2005	2.20479	28.1961	OK	OK	0.466928
ENSG0000010	-	-	ENSG0000010469	'PPP2CB	-	8:30774456-30814314	-	-	IGV_LINK	1114.21	51.533	9.82941	93.2367	OK	2039.76	95.956	6.31036	185.602	OK	OK	0.896875
ENSG0000010	-	-	ENSG0000010471	'ERICH1	-	8:614745-1708474	-	-	IGV_LINK	595.408	24.5491	4.87769	44.2206	OK	634.203	26.7552	5.73261	47.7778	OK	OK	0.124147
ENSG0000010	-	-	ENSG0000010472	'NEFM	-	8:24912164-24919098	-	-	IGV_LINK	73.6988	1.78151	0	4.53909	OK	364.022	6.06705	1.78824	10.3459	OK	OK	1.7679
ENSG0000010	-	-	ENSG0000010472	'TUSC3	-	8:15417214-15766649	-	-	IGV_LINK	976.509	49.0335	17.3587	80.7083	OK	1456.09	73.7275	28.5467	118.908	OK	OK	0.588436
ENSG0000010	-	-	ENSG0000010472	'ARHGEF10	-	8:1755777-1958641	-	-	IGV_LINK	891.006	15.4899	0	35.0105	OK	676.85	11.2958	0	26.8702	OK	OK	-0.45554
ENSG0000010	-	-	ENSG0000010473	'KLHDC4	-	16:87600525-87765992	-	-	IGV_LINK	755.412	24.0592	3.81797	44.3004	OK	820.855	28.3863	4.02192	52.7507	OK	OK	0.238608
ENSG0000010	-	-	ENSG0000010473	'MCM4	-	8:47960184-47978160	-	-	IGV_LINK	499.406	17.0961	0	38.6585	OK	598.11	17.9815	0	41.9002	OK	OK	0.072847
ENSG0000010	-	-	ENSG0000010475	'ADAM2	-	8:39743734-39838289	-	-	IGV_LINK	0	0	0	0	OK	0	0	0	0	OK	NOTEST	0
ENSG0000010	-	-	ENSG0000010475	'KCTD9	-	8:25427846-25458476	-	-	IGV_LINK	880.506	20.9072	0.026597	41.7877	OK	1142.6	27.7551	0	56.3985	OK	OK	0.408756
ENSG0000010	-	-	ENSG0000010476	'FGL1	-	8:17864379-17910365	-	-	IGV_LINK	0.969721	0.030454	0	0.53231	OK	1.03122	0.032406	0	0.532722	OK	NOTEST	0.089643
ENSG0000010	-	-	ENSG0000010476	'ASAH1	-	8:18055991-18096394	-	-	IGV_LINK	6087.91	205.833	52.852	358.815	OK	7300.04	253.146	74.6689	431.624	OK	OK	0.298495
ENSG0000010	-	-	ENSG0000010476	'BNIP3L	-	8:26379258-26658178	-	-	IGV_LINK	2186.72	81.009	0	183.536	OK	4240.4	163.14	0	390.906	OK	OK	1.00995
ENSG0000010	-	-	ENSG0000010477	'MAN2B1	-	19:12643274-12681902	-	-	IGV_LINK	3154.27	79.8789	0	180.046	OK	4097.38	86.7613	0	185.691	OK	OK	0.119237
ENSG0000010	-	-	ENSG0000010478	'KCNN4	-	19:43766532-43781257	-	-	IGV_LINK	134.791	4.64415	0.523074	8.76522	OK	93.8415	3.78987	0	8.01482	OK	OK	-0.29326
ENSG0000010	-	-	ENSG0000010480	'TULP2	-	19:48880964-48898733	-	-	IGV_LINK	1.93944	0.069112	0	0.639323	OK	2.06245	0.073548	0	0.644006	OK	NOTEST	0.089768
ENSG0000010	-	-	ENSG0000010480	'NUCB1	-	19:48900049-48923372	-	-	IGV_LINK	6182.94	243.102	72.9336	413.269	OK	7285.6	301.696	87.1823	516.209	OK	OK	0.311536
ENSG0000010	-	-	ENSG0000010480	'DHDH	-	19:48933681-48944969	-	-	IGV_LINK	9.69721	0.855452	0	2.03505	OK	9.28102	0.63677	0	1.51359	OK	OK	-0.42591
ENSG0000010	-	-	ENSG0000010481	'GYS1	-	19:48968124-48993310	-	-	IGV_LINK	1344.03	28.9267	2.72379	55.1295	OK	3214.33	63.64	9.56295	117.717	OK	OK	1.13753
ENSG0000010	-	-	ENSG0000010481	'MAP4K1	-	19:38587640-38618882	-	-	IGV_LINK	618.682	19.8881	0	42.7613	OK	338.242	12.866	0	26.5724	OK	OK	-0.62834
ENSG0000010	-	-	ENSG0000010481	'CGB2	-	19:49022868-49036895	-	-	IGV_LINK	0	0	0	0	OK	0	0	0	0	OK	NOTEST	0
ENSG0000010	-	-	ENSG0000010482	'ECH1	-	19:38815421-38852347	-	-	IGV_LINK	2709.4	167.05	0	341.674	OK	3884.62	238.616	0	481.737	OK	OK	0.514408
ENSG0000010	-	-	ENSG0000010482	'HNRNPL	-	19:38815421-38852347	-	-	IGV_LINK	3244.68	118.389	11.4313	225.347	OK	3367.98	127.966	1.13583	254.796	OK	OK	0.112227
ENSG0000010	-	-	ENSG0000010482	'NFKBIB	-	19:38878554-38908893	-	-	IGV_LINK	408.252	15.676	0	35.2241	OK	447.552	16.055	0	35.4947	OK	OK	0.034467
ENSG0000010	-	-	ENSG0000010482	'LHB	-	19:48993447-49017081	-	-	IGV_LINK	0	0	0	0	OK	0	0	0	0	OK	NOTEST	0
ENSG0000010	-	-	ENSG0000010482	'CGB3	-	19:49022868-49036895	-	-	IGV_LINK	0	0	0	0	OK	0	0	0	0	OK	NOTEST	0
ENSG0000010	-	-	ENSG0000010483	'TUBB4A	-	19:6494318-6502848	-	-	IGV_LINK	90.184	2.61339	0	5.83263	OK	5.15612	0.375587	0	1.44825	OK	OK	-2.7987
ENSG0000010	-	-	ENSG0000010483	'SARS2	-	19:38915265-38975910	-	-	IGV_LINK	240.548	12.8031	0	52.1044	OK	350.612	17.7484	0	69.5086	OK	OK	0.471202
ENSG0000010	-	-	ENSG0000010484	'KCNA7	-	19:49067417-49072941	-	-	IGV_LINK	0.969721	0.013013	0	0.05097	OK	3.09367	0.041527	0	0.107288	OK	NOTEST	1.67408
ENSG0000010	-	-	ENSG0000010485	'SNRNP70	-	19:49085418-49108605	-	-	IGV_LINK	6594.1	174.104	24.16	324.049	OK	5808.89	152.951	41.4077	264.494	OK	OK	-0.18689
ENSG0000010	-	-	ENSG0000010485	'CLPTM1	-	19:44954584-44993341	-	-	IGV_LINK	2066.47	57.8758	20.7025	95.0491	OK	2518.25	72.4074	24.5353	120.28	OK	OK	0.323177
ENSG0000010	-	-	ENSG0000010485	'RELB	-	19:45001429-45038198	-	-	IGV_LINK	738.927	20.9817	7.71042	34.2529	OK	608.423	18.839	5.833	31.8449	OK	OK	-0.15541
ENSG0000010	-	-	ENSG0000010485	'CLASRP	-	19:45039039-45070956	-	-	IGV_LINK	999.782	52.478	5.8199	99.136	OK	931.196	54.1118	2.19194	106.032	OK	OK	0.044232
ENSG0000010	-	-	ENSG0000010486	'LIN7B	-	19:49114323-49119140	-	-	IGV_LINK	21.3339	2.47936	0	5.64039	OK	26.8118	3.46819	0	7.62131	OK	OK	0.484219
ENSG0000010	-	-	ENSG0000010486	'PPP1R37	-	19:45071499-45305283	-	-	IGV_LINK	1161.73	23.4426	0	51.4592	OK	1027.1	20.737	0	47.1906	OK	OK	-0.17692
ENSG0000010	-	-	ENSG0000010487	'FCGRT	-	19:49506815-49526333	-	-	IGV_LINK	3596.69	147.023	54.7659	239.28	OK	4929.26	200.049	71.966	328.132	OK	OK	0.444311
ENSG0000010	-	-	ENSG0000010487	'PIH1D1	-	19:49446297-49492308	-	-	IGV_LINK	993.964	68.3921	0	446.351	OK	895.103	60.5671	0	321.533	OK	OK	-0.1753
ENSG0000010	-	-	ENSG0000010487	'CKM	-	19:45306413-45322977	-	-	IGV_LINK	0.969721	0.036814	0	0.14419	OK	4.1249	0.156716	0	0.397003	OK	NOTEST	2.08984
ENSG0000010	-	-	ENSG0000010488	'ARHGEF18	-	19:7348942-7472477	-	-	IGV_LINK	746.542	9.27909	1.80344	16.7547	OK	1040.8	13.1684	2.59254	23.7443	OK	OK	0.505028
ENSG0000010	-	-	ENSG0000010488	'PPP1R13L	-	19:45379633-45478828	-	-	IGV_LINK	1577.74	64.4769	0	138.948	OK	876.541	40.7121	0	101.555	OK	OK	-0.66333
ENSG0000010	-	-	ENSG0000010488	'PEX11G	-	19:7476874-7521026	-	-	IGV_LINK	65.941	4.13482	0	13.182	OK	112.404	7.08833	0	19.2759	OK	OK	0.777621
ENSG0000010	-	-	ENSG0000010488	'ERCC2	-	19:45333433-45370918	-	-	IGV_LINK	332.614	10.1106	1.85131	18.3699	OK	355.773	10.3736	2.19274	18.5546	OK	OK	0.037051
ENSG0000010	-	-	ENSG0000010488	'DOT1L	-	19:2100987-2248679	-	-	IGV_LINK	588.62	5.54748	0	19.2097	OK	551.705	5.04262	0	15.4087	OK	OK	-0.13766
ENSG0000010	-	-	ENSG0000010488	'PLEKHJ1	-	19:2100987-2248679	-	-	IGV_LINK	686.562	39.3487	0	105.375	OK	621.829	32.9976	0	90.2691	OK	OK	-0.25396
ENSG0000010	-	-	ENSG0000010488	'SLC17A7	-	19:49429400-49442360	-	-	IGV_LINK	8.72748	0.179119	0	0.493026	OK	4.1249	0.083042	0	0.341973	OK	NOTEST	-1.10901
ENSG0000010	-	-	ENSG0000010488	'RNASEH2A	-	19:12763002-12874951	-	-	IGV_LINK	171.641	9.91991	0	41.354	OK	313.492	17.7959	0	68.0154	OK	OK	0.843143
ENSG0000010	-	-	ENSG0000010489	'KLC3	-	19:45333433-45370918	-	-	IGV_LINK	1.93944	0.091721	0	0.965287	OK	3.09367	0.146359	0	1.25869	OK	NOTEST	0.674193
ENSG0000010	-	-	ENSG0000010489	'CD37	-	19:49331616-49388081	-	-	IGV_LINK	1351.79	76.2934	10.0058	142.581	OK	1046.69	56.5271	10.0473	103.007	OK	OK	-0.43262
ENSG0000010	-	-	ENSG0000010489	'SF3A2	-	19:2100987-2248679	-	-	IGV_LINK	647.773	29.288	0	81.0376	OK	598.11	26.9502	0	77.7638	OK	OK	-0.12001
ENSG0000010	-	-	ENSG0000010489	'AMH	-	19:2249308-2252073	-	-	IGV_LINK	22.3036	1.08077	0	2.59148	OK	22.6869	1.34152	0	3.29134	OK	OK	0.311819
ENSG0000010	-	-	ENSG0000010490	'DKKL1	-	19:49331616-49388081	-	-	IGV_LINK	0	0	0	0	OK	0	0	0	0	OK	NOTEST	0
ENSG0000010	-	-	ENSG0000010490	'LYL1	-	19:13099032-13103161	-	-	IGV_LINK	236.612	10.843	2.91781	18.7681	OK	171.183	7.79518	1.43972	14.1506	OK	OK	-0.4761
ENSG0000010	-	-	ENSG0000010490	'OAZ1	-	19:2252251-2354806	-	-	IGV_LINK	5878.68	429.524	67.656	791.393	OK	7580.09	557.052	96.4741	1017.63	OK	OK	0.375073
ENSG0000010	-	-	ENSG0000010490	'TRMT1	-	19:13104901-13117567	-	-	IGV_LINK	929.962	28.327	10.0996	46.5544	OK	904.384	30.0613	9.39376	50.7288	OK	OK	0.08573
ENSG0000010	-	-	ENSG0000010491	'STX10	-	19:13144057-13150383	-	-	IGV_LINK	684.623	39.6896	14.2958	65.0834	OK	629.047	37.2087	14.6499	59.7676	OK	OK	-0.09312
ENSG0000010	-	-	ENSG0000010491	'RETN	-	19:7669043-7670454	-	-	IGV_LINK	28.1219	4.56064	0	9.63003	OK	32.9992	5.37765	0	11.1762	OK	OK	0.237741
ENSG0000010	-	-	ENSG0000010492	'FCER2	-	19:7688757-7702146	-	-	IGV_LINK	3.87888	0.128641	0	0.431778	OK	16.4996	0.634909	0	1.38989	OK	OK	2.3032
ENSG0000010	-	-	ENSG0000010493	'DMPK	-	19:45764784-45792802	-	-	IGV_LINK	645.834	18.1126	0	39.9551	OK	516.644	14.5282	0	33.352	OK	OK	-0.31813
ENSG0000010	-	-	ENSG0000010493	'CLEC4M	-	19:7763148-7769605	-	-	IGV_LINK	0	0	0	0	OK	0	0	0	0	OK	NOTEST	0
ENSG0000010	-	-	ENSG0000010494	'RSPH6A	-	19:45795709-45815319	-	-	IGV_LINK	0	0	0	0	OK	1.03122	0.027251	0	0.138586	OK	NOTEST	inf
ENSG0000010	-	-	ENSG0000010494	'TBC1D17	-	19:49818278-49888749	-	-	IGV_LINK	1412.88	39.6823	0	94.7636	OK	1158.07	33.8075	0	85.8808	OK	OK	-0.23115
ENSG0000010	-	-	ENSG0000010495	'IL4I1	-	19:49889653-49961172	-	-	IGV_LINK	551.819	18.9841	0	45.3362	OK	567.207	19.0003	0	53.5818	OK	OK	0.001233
ENSG0000010	-	-	ENSG0000010495	'TLE6	-	19:2977445-2995184	-	-	IGV_LINK	8.72748	0.557095	0	1.50163	OK	9.28102	0.420901	0	1.30639	OK	NOTEST	-0.40444
ENSG0000010	-	-	ENSG0000010495	'CCDC130	-	19:13731759-13763296	-	-	IGV_LINK	793.231	40.9313	13.4342	68.4283	OK	874.479	44.9423	15.952	73.9326	OK	OK	0.134872
ENSG0000010	-	-	ENSG0000010496	'PTOV1	-	19:49818278-49888749	-	-	IGV_LINK	1404.16	64.4538	0	155.868	OK	1196.22	57.0348	0	142.028	OK	OK	-0.17642
ENSG0000010	-	-	ENSG0000010496	'AES	-	19:3052909-3063107	-	-	IGV_LINK	4445.2	213.605	68.4534	358.756	OK	3362.82	162.754	56.7858	268.722	OK	OK	-0.39225
ENSG0000010	-	-	ENSG0000010496	'NOVA2	-	19:45933733-45973546	-	-	IGV_LINK	144.488	1.86101	0	4.98505	OK	131.997	2.23362	0	5.91234	OK	OK	0.263295
ENSG0000010	-	-	ENSG0000010496	'SGTA	-	19:2754713-2783371	-	-	IGV_LINK	1014.33	27.0046	11.2513	42.7578	OK	1051.85	28.4507	11.6414	45.26	OK	OK	0.075261
ENSG0000010	-	-	ENSG0000010497	'KIR3DX1	-	19:54532691-54545771	-	-	IGV_LINK	9.69721	0.212284	0	0.600879	OK	6.18735	0.253261	0	0.713001	OK	NOTEST	0.254633
ENSG0000010	-	-	ENSG0000010497	'LILRB1	-	19:54617157-54638892	-	-	IGV_LINK	821.353	22.1635	5.24006	39.0868	OK	789.918	22.176	5.16686	39.1852	OK	OK	0.000819
ENSG0000010	-	-	ENSG0000010497	'MED25	-	19:49818278-49888749	-	-	IGV_LINK	722.442	20.6441	0	60.7172	OK	908.509	25.7896	0	68.8033	OK	OK	0.321057
ENSG0000010	-	-	ENSG0000010497	'LILRA1	-	19:54593581-54602090	-	-	IGV_LINK	228.854	6.14481	1.69013	10.5995	OK	140.247	3.79709	0.593618	7.00056	OK	OK	-0.69447
ENSG0000010	-	-	ENSG0000010497	'SNAPC2	-	19:7920315-7923250	-	-	IGV_LINK	225.945	10.2297	1.83147	18.628	OK	225.838	10.0852	2.15229	18.0181	OK	OK	-0.02053
ENSG0000010	-	-	ENSG0000010497	'C19orf53	-	19:13764531-13778462	-	-	IGV_LINK	762.2	73.3789	5.26787	141.49	OK	905.416	90.7158	14.4023	167.029	OK	OK	0.305988
ENSG0000010	-	-	ENSG0000010498	'TIMM44	-	19:7924484-7943920	-	-	IGV_LINK	720.502	37.0904	11.0268	63.1539	OK	761.044	35.5632	10.4911	60.6354	OK	OK	-0.06066
ENSG0000010	-	-	ENSG0000010498	'CCDC61	-	19:45995460-46023065	-	-	IGV_LINK	255.036	13.2497	2.22883	24.2706	OK	234.088	12.7274	1.25937	24.1953	OK	OK	-0.05803
ENSG0000010	-	-	ENSG0000010499	'IL27RA	-	19:14031747-14053216	-	-	IGV_LINK	517.831	10.4617	3.91124	17.0121	OK	894.072	18.0703	7.63565	28.5049	OK	OK	0.788505
ENSG0000010	-	-	ENSG0000010501	'ASF1B	-	19:14119105-14136956	-	-	IGV_LINK	140.609	4.36618	0.70106	8.0313	OK	182.527	6.0498	1.08917	11.0104	OK	OK	0.470516
ENSG0000010	-	-	ENSG0000010504	'TNNT1	-	19:55132793-55149354	-	-	IGV_LINK	0	0	0	0	OK	224.807	17.9327	4.54994	31.3155	OK	OK	inf
ENSG0000010	-	-	ENSG0000010505	'VRK3	-	19:49976466-50066793	-	-	IGV_LINK	879.536	39.1329	8.34953	69.9162	OK	1278.72	52.2991	14.1303	90.4679	OK	OK	0.418405
ENSG0000010	-	-	ENSG0000010505	'FAM32A	-	19:16185379-16192046	-	-	IGV_LINK	1240.27	56.2732	24.1166	88.4299	OK	1468.46	66.0447	28.6786	103.411	OK	OK	0.230994
ENSG0000010	-	-	ENSG0000010506	'PPP6R1	-	19:55226638-55258995	-	-	IGV_LINK	1750.86	31.4974	9.59446	53.4004	OK	1947.9	34.6605	11.3488	57.9722	OK	OK	0.138059
ENSG0000010	-	-	ENSG0000010507	'C19orf44	-	19:16479056-16660442	-	-	IGV_LINK	352.978	6.61994	0	20.4419	OK	365.054	6.62533	0	22.3317	OK	OK	0.001175
ENSG0000010	-	-	ENSG0000010508	'MED26	-	19:16479056-16660442	-	-	IGV_LINK	162.63	3.11854	0	13.9876	OK	229.777	4.439	0	17.4851	OK	OK	0.509362
ENSG0000010	-	-	ENSG0000010508	'OLFM2	-	19:9853717-9936552	-	-	IGV_LINK	37.8191	1.67352	0	3.63824	OK	41.249	1.70834	0	3.68159	OK	OK	0.029712

ENSG0000010	-	-	ENSG0000010512	'RASAL3	-	19:15451623-15464571	-	-	IGV_LINK	1107.42	25.212	0	58.1911	OK	774.45	19.1045	0	44.7818	OK	OK	-0.4002
ENSG0000010	-	-	ENSG0000010512	'AKAP8	-	19:15353384-15419141	-	-	IGV_LINK	1456.52	33.2061	0	72.8704	OK	1655.12	35.3345	0	71.9196	OK	OK	0.089628
ENSG0000010	-	-	ENSG0000010513	'EPHX3	-	19:15226918-15233435	-	-	IGV_LINK	65.941	2.25298	0	4.59257	OK	55.6861	1.93628	0.039365	3.83319	OK	OK	-0.21855
ENSG0000010	-	-	ENSG0000010513	'ILVBL	-	19:15107402-15126174	-	-	IGV_LINK	1316.88	43.0724	8.30122	77.8435	OK	1275.63	41.0777	8.28619	73.8691	OK	OK	-0.06841
ENSG0000010	-	-	ENSG0000010513	'ZNF419	-	19:57487710-57518404	-	-	IGV_LINK	233.844	6.114	0	12.4816	OK	235.675	7.05773	0.346512	13.769	OK	OK	0.207089
ENSG0000010	-	-	ENSG0000010513	'SYDE1	-	19:15107402-15126174	-	-	IGV_LINK	571.165	10.4633	0	21.2246	OK	549.643	10.0285	0	21.1393	OK	OK	-0.06124
ENSG0000010	-	-	ENSG0000010514	'CASP14	-	19:15049383-15058293	-	-	IGV_LINK	0	0	0	0	OK	0	0	0	0	OK	NOTEST	0
ENSG0000010	-	-	ENSG0000010514	'SLC1A6	-	19:14950033-15023269	-	-	IGV_LINK	1.93944	0.048631	0	0.564129	OK	0	0	0	0	OK	NOTEST	'-inf
ENSG0000010	-	-	ENSG0000010514	'AURKC	-	19:57230801-57235548	-	-	IGV_LINK	18.4247	0.891451	0	2.06271	OK	14.4371	1.14153	0	2.62834	OK	OK	0.356745
ENSG0000010	-	-	ENSG0000010517	'POP4	-	19:29604016-29617237	-	-	IGV_LINK	548.862	54.5202	15.676	93.3643	OK	723.92	78.9933	19.958	138.029	OK	OK	0.534939
ENSG0000010	-	-	ENSG0000010517	'CCNE1	-	19:29811897-29824308	-	-	IGV_LINK	39.7585	1.49808	0	3.25439	OK	59.811	2.51935	0	5.1101	OK	OK	0.749936
ENSG0000010	-	-	ENSG0000010517	'URI1	-	19:29923643-30016608	-	-	IGV_LINK	837.839	26.7728	9.54431	44.0014	OK	824.98	26.0355	9.78267	42.2883	OK	OK	-0.04029
ENSG0000010	-	-	ENSG0000010518	'PDCD5	-	19:32581067-32587452	-	-	IGV_LINK	724.381	64.0175	21.4331	106.602	OK	808.48	73.6366	22.7985	124.475	OK	OK	0.201958
ENSG0000010	-	-	ENSG0000010518	'ANKRD27	-	19:32597006-32678300	-	-	IGV_LINK	513.952	7.47818	0.899553	14.0568	OK	480.551	7.25069	0.512023	13.9894	OK	OK	-0.04457
ENSG0000010	-	-	ENSG0000010519	'RPS16	-	19:39433206-39435948	-	-	IGV_LINK	7892.56	1124.5	388.026	1860.98	OK	6302.85	890.05	339.117	1440.98	OK	OK	-0.33733
ENSG0000010	-	-	ENSG0000010519	'TIMM50	-	19:39480411-39493785	-	-	IGV_LINK	676.865	32.8664	11.6664	54.0663	OK	644.516	31.3631	11.049	51.6772	OK	OK	-0.06754
ENSG0000010	-	-	ENSG0000010519	'LGALS13	-	19:39602500-39607476	-	-	IGV_LINK	0	0	0	0	OK	0	0	0	0	OK	NOTEST	0
ENSG0000010	-	-	ENSG0000010520	'FBL	-	19:39834457-39846414	-	-	IGV_LINK	1718.34	146.367	57.8477	234.886	OK	1033.29	88.1513	31.1715	145.131	OK	OK	-0.73153
ENSG0000010	-	-	ENSG0000010520	'DYRK1B	-	19:39825349-39834201	-	-	IGV_LINK	447.041	14.2241	1.94498	26.5032	OK	547.58	14.7881	5.60136	23.9749	OK	OK	0.056103
ENSG0000010	-	-	ENSG0000010520	'CLC	-	19:39731249-39738028	-	-	IGV_LINK	3.87888	0.472668	0	1.20381	OK	3.09367	0.377925	0	0.976388	OK	NOTEST	-0.32273
ENSG0000010	-	-	ENSG0000010521	'CNTD2	-	19:40222207-40226690	-	-	IGV_LINK	7.75776	0.575694	0	1.47736	OK	3.09367	0.108334	0	0.46625	OK	OK	-2.40981
ENSG0000010	-	-	ENSG0000010522	'GPI	-	19:34359479-34428273	-	-	IGV_LINK	6336.75	195.133	61.5548	328.711	OK	6894.31	207.538	61.75	353.326	OK	OK	0.088918
ENSG0000010	-	-	ENSG0000010522	'AKT2	-	19:40230316-40285536	-	-	IGV_LINK	2405.88	67.2708	11.6757	122.866	OK	2175.88	68.6422	12.4158	124.869	OK	OK	0.029115
ENSG0000010	-	-	ENSG0000010522	'PLD3	-	19:40319535-40390187	-	-	IGV_LINK	3077.89	125.841	17.3689	234.313	OK	5304.62	195.494	37.3343	353.655	OK	OK	0.635528
ENSG0000010	-	-	ENSG0000010522	'PRX	-	19:40393767-40413366	-	-	IGV_LINK	101.821	1.21452	0.132806	2.29622	OK	62.9047	0.746273	0	1.5079	OK	OK	-0.70261
ENSG0000010	-	-	ENSG0000010522	'PIAS4	-	19:4007645-4039386	-	-	IGV_LINK	336.493	10.0879	0	25.2965	OK	315.555	10.832	0	21.6692	OK	OK	0.102685
ENSG0000010	-	-	ENSG0000010524	'NUMBL	-	19:40665904-40690972	-	-	IGV_LINK	187.156	5.9138	0.470594	11.357	OK	205.214	5.18225	0.567291	9.79721	OK	OK	-0.19051
ENSG0000010	-	-	ENSG0000010524	'EBI3	-	19:4229497-4237531	-	-	IGV_LINK	254.067	14.1277	3.84447	24.4109	OK	179.433	9.98894	2.34705	17.6308	OK	OK	-0.50012
ENSG0000010	-	-	ENSG0000010524	'CCDC94	-	19:4246338-4269090	-	-	IGV_LINK	324.856	14.6259	4.79952	24.4523	OK	310.399	13.8795	4.45773	23.3013	OK	OK	-0.07557
ENSG0000010	-	-	ENSG0000010525	'SHD	-	19:4278600-4290724	-	-	IGV_LINK	2.90916	0.070166	0	0.630805	OK	1.03122	0.489699	0	1.90387	OK	NOTEST	2.80305
ENSG0000010	-	-	ENSG0000010525	'TBCB	-	19:36111150-36125947	-	-	IGV_LINK	915.404	76.3818	24.5253	128.238	OK	910.126	76.2447	24.4839	128.006	OK	OK	-0.00259
ENSG0000010	-	-	ENSG0000010525	'FSD1	-	19:4304599-4323843	-	-	IGV_LINK	13.5761	0.673189	0	1.90715	OK	16.4996	0.70568	0	1.76024	OK	OK	0.068002
ENSG0000010	-	-	ENSG0000010525	'POLR2I	-	19:36111150-36125947	-	-	IGV_LINK	293.825	52.8934	0	118.694	OK	276.368	49.7802	0	111.553	OK	OK	-0.08752
ENSG0000010	-	-	ENSG0000010526	'OVOL3	-	19:36111150-36125947	-	-	IGV_LINK	0	0	0	0	OK	0	0	0	0	OK	NOTEST	0
ENSG0000010	-	-	ENSG0000010527	'CLIP3	-	19:36014507-36054762	-	-	IGV_LINK	405.343	7.22395	1.88954	12.5584	OK	379.491	6.69092	1.90221	11.4796	OK	OK	-0.11058
ENSG0000010	-	-	ENSG0000010527	'ZFR2	-	19:3804023-3869032	-	-	IGV_LINK	0	0	0	0	OK	2.06245	0.166918	0	0.832215	OK	NOTEST	inf
ENSG0000010	-	-	ENSG0000010528	'SLC1A5	-	19:46719506-46789043	-	-	IGV_LINK	614.803	19.3386	0	39.9052	OK	951.821	29.755	0	64.137	OK	OK	0.621649
ENSG0000010	-	-	ENSG0000010528	'PRKD2	-	19:46647611-46717127	-	-	IGV_LINK	1201.48	28.906	9.03262	48.7795	OK	1142.6	24.6218	9.59635	39.6472	OK	OK	-0.23144
ENSG0000010	-	-	ENSG0000010528	'TJP3	-	19:3708108-3750813	-	-	IGV_LINK	11.6366	0.464055	0	1.37341	OK	92.8102	2.93272	0	6.57023	OK	OK	2.65987
ENSG0000010	-	-	ENSG0000010529	'APLP1	-	19:35825963-35879791	-	-	IGV_LINK	8.72748	0.963266	0	2.65254	OK	43.3114	1.38869	0	3.71739	OK	OK	0.527718
ENSG0000010	-	-	ENSG0000010529	'CACTIN	-	19:3607246-3626815	-	-	IGV_LINK	496.497	10.6712	2.75849	18.5839	OK	574.392	12.5821	2.22826	22.936	OK	OK	0.237655
ENSG0000010	-	-	ENSG0000010532	'CCDC9	-	19:47255979-47271953	-	-	IGV_LINK	438.314	17.5541	2.50611	32.602	OK	412.49	17.7658	0.588844	34.9427	OK	OK	0.017297
ENSG0000010	-	-	ENSG0000010532	'HNRNPUL1	-	19:41262495-41425005	-	-	IGV_LINK	3779	77.6476	0	186.246	OK	3374.17	70.1509	0	162.713	OK	OK	-0.14648
ENSG0000010	-	-	ENSG0000010532	'FZR1	-	19:3506272-3579088	-	-	IGV_LINK	816.505	21.3132	0	57.5906	OK	781.669	21.2826	0	55.1547	OK	OK	-0.00207
ENSG0000010	-	-	ENSG0000010532	'BBC3	-	19:47220821-47232766	-	-	IGV_LINK	300.613	14.0607	3.69635	24.425	OK	257.806	11.1585	2.70006	19.617	OK	OK	-0.33352
ENSG0000010	-	-	ENSG0000010532	'TGFB1	-	19:41262495-41425005	-	-	IGV_LINK	1872.53	48.4404	0	166.925	OK	1472.59	38.7475	0	136.606	OK	OK	-0.32211
ENSG0000010	-	-	ENSG0000010533	'DENND3	-	8:141117277-141195808	-	-	IGV_LINK	1947.2	42.8742	3.09144	82.6569	OK	2223.32	46.9624	2.83338	91.0914	OK	OK	0.131396
ENSG0000010	-	-	ENSG0000010534	'DMAC2	-	19:41431317-41440717	-	-	IGV_LINK	665.228	25.5992	8.57676	42.6216	OK	755.888	31.4372	9.83032	53.044	OK	OK	0.296374
ENSG0000010	-	-	ENSG0000010535	'CEACAM4	-	19:41618970-41627074	-	-	IGV_LINK	22.3036	1.2174	0	2.58408	OK	64.9672	4.63503	0	9.67871	OK	OK	1.92878
ENSG0000010	-	-	ENSG0000010535	'PLIN3	-	19:4838331-4867768	-	-	IGV_LINK	732.139	20.0605	7.71544	32.4056	OK	2037.7	59.8536	21.2268	98.4803	OK	OK	1.57708
ENSG0000010	-	-	ENSG0000010535	'MYH14	-	19:50188185-50310545	-	-	IGV_LINK	101.821	1.06505	0.030979	2.09911	OK	889.947	9.77089	3.66091	15.8809	OK	OK	3.19757
ENSG0000010	-	-	ENSG0000010536	'MRPL4	-	19:10251900-10289019	-	-	IGV_LINK	531.407	27.8413	0	70.3427	OK	643.484	32.21	0	89.2542	OK	OK	0.210278
ENSG0000010	-	-	ENSG0000010536	'SIGLEC8	-	19:51450846-51458456	-	-	IGV_LINK	301.583	6.49891	1.97081	11.027	OK	193.87	5.02784	0.121894	9.93379	OK	OK	-0.37026
ENSG0000010	-	-	ENSG0000010536	'CD79A	-	19:41877119-41881372	-	-	IGV_LINK	102.79	5.75229	0.398737	11.1058	OK	151.59	8.86742	1.38248	16.3524	OK	OK	0.624377
ENSG0000010	-	-	ENSG0000010537	'LIM2	-	19:51379908-51387960	-	-	IGV_LINK	0	0	0	0	OK	0	0	0	0	OK	NOTEST	0
ENSG0000010	-	-	ENSG0000010537	'ICAM4	-	19:10251900-10289019	-	-	IGV_LINK	7.75776	0.422683	0	5.58725	OK	8.2498	0.448193	0	7.26585	OK	NOTEST	0.084545
ENSG0000010	-	-	ENSG0000010537	'RPS19	-	19:41859917-41872926	-	-	IGV_LINK	14132.7	2485.21	575.539	4394.89	OK	14531	2535.16	566.101	4504.21	OK	OK	0.028705
ENSG0000010	-	-	ENSG0000010537	'NOP53	-	19:47745521-47768840	-	-	IGV_LINK	6227.55	273.514	96.793	450.234	OK	4409.52	198.837	74.7017	322.973	OK	OK	-0.46002
ENSG0000010	-	-	ENSG0000010537	'NKG7	-	19:51371605-51372715	-	-	IGV_LINK	756.382	68.8274	24.0237	113.631	OK	331.023	29.8734	8.60761	51.1392	OK	OK	-1.20412
ENSG0000010	-	-	ENSG0000010537	'ICAM5	-	19:10289980-10296778	-	-	IGV_LINK	4.8486	0.112609	0	0.453522	OK	8.2498	0.296761	0	0.898447	OK	NOTEST	1.39798
ENSG0000010	-	-	ENSG0000010537	'ETFB	-	19:51345168-51369003	-	-	IGV_LINK	941.053	68.4091	27.6312	109.187	OK	1294.65	94.5658	39.8399	149.292	OK	OK	0.46713
ENSG0000010	-	-	ENSG0000010538	'CD33	-	19:51225063-51243860	-	-	IGV_LINK	363.645	12.9217	2.81355	23.0299	OK	354.741	10.7349	2.48003	18.9899	OK	OK	-0.26749
ENSG0000010	-	-	ENSG0000010538	'CEACAM5	-	19:41708584-41786893	-	-	IGV_LINK	0	0	0	0	OK	0	0	0	0	OK	NOTEST	0
ENSG0000010	-	-	ENSG0000010539	'CRX	-	19:47819778-47843330	-	-	IGV_LINK	4.8486	0.067961	0	0.846614	OK	3.09367	0.043375	0	0.815984	OK	NOTEST	-0.64786
ENSG0000010	-	-	ENSG0000010539	'BABAM1	-	19:17231882-17287646	-	-	IGV_LINK	1098.67	59.6896	11.0172	108.362	OK	1357.03	76.7516	10.8594	142.644	OK	OK	0.362718
ENSG0000010	-	-	ENSG0000010539	'TYK2	-	19:10350528-10380676	-	-	IGV_LINK	3205.9	147.214	10.8128	283.615	OK	3688.69	148.012	8.29917	287.725	OK	OK	0.0078
ENSG0000010	-	-	ENSG0000010539	'SULT2A1	-	19:47870465-47886397	-	-	IGV_LINK	0	0	0	0	OK	0	0	0	0	OK	NOTEST	0
ENSG0000010	-	-	ENSG0000010540	'CDC37	-	19:10391133-10469631	-	-	IGV_LINK	2918.86	127.498	44.2893	210.706	OK	3379.32	144.612	54.2056	235.019	OK	OK	0.181719
ENSG0000010	-	-	ENSG0000010540	'NAPA	-	19:47484281-47515240	-	-	IGV_LINK	2141.53	103.165	25.566	180.764	OK	2400.26	121.757	29.0326	214.482	OK	OK	0.239056
ENSG0000010	-	-	ENSG0000010540	'RABAC1	-	19:41956680-41959390	-	-	IGV_LINK	728.26	64.4276	26.521	102.334	OK	754.857	66.6754	27.6491	105.702	OK	OK	0.049476
ENSG0000010	-	-	ENSG0000010540	'ATP1A3	-	19:41966581-41997497	-	-	IGV_LINK	31.0311	0.534553	0	2.73141	OK	22.6869	0.367656	0	2.52907	OK	NOTEST	-0.53998
ENSG0000010	-	-	ENSG0000010541	'MEIS3	-	19:47403123-47419523	-	-	IGV_LINK	85.3354	3.1624	0.055536	6.26927	OK	304.211	12.0447	3.328	20.7613	OK	OK	1.9293
ENSG0000010	-	-	ENSG0000010542	'PTPRS	-	19:5158494-5340803	-	-	IGV_LINK	1345	24.7735	0	50.9299	OK	823.949	14.2003	0	28.853	OK	OK	-0.80288
ENSG0000010	-	-	ENSG0000010542	'CNFN	-	19:42387018-42390287	-	-	IGV_LINK	9.69721	1.55918	0	3.61938	OK	17.5308	2.82806	0	6.02321	OK	OK	0.859022
ENSG0000010	-	-	ENSG0000010542	'ZNRF4	-	19:5455430-5456856	-	-	IGV_LINK	0	0	0	0	OK	0	0	0	0	OK	NOTEST	0
ENSG0000010	-	-	ENSG0000010542	'MEGF8	-	19:42325608-42378769	-	-	IGV_LINK	1048.27	7.66489	0.098863	15.2309	OK	1440.62	9.94641	1.26268	18.6301	OK	OK	0.37591
ENSG0000010	-	-	ENSG0000010543	'KDELR1	-	19:48382569-48391553	-	-	IGV_LINK	2379.69	99.488	41.7863	157.19	OK	3096.77	126.718	54.4664	198.969	OK	OK	0.349022
ENSG0000010	-	-	ENSG0000010544	'CYTH2	-	19:48445772-48482314	-	-	IGV_LINK	1605.86	40.5222	7.73326	73.3111	OK	1171.47	30.8367	5.22206	56.4513	OK	OK	-0.39406
ENSG0000010	-	-	ENSG0000010544	'GRWD1	-	19:48445772-48482314	-	-	IGV_LINK	504.203	10.7917	0	30.8348	OK	702.226	15.7172	0	38.103	OK	OK	0.542428
ENSG0000010	-	-	ENSG0000010546	'GRIN2D	-	19:48394874-48444931	-	-	IGV_LINK	22.3036	0.255448	0	0.565016	OK	19.5933	0.224459	0	0.496694	OK	NOTEST	-0.18658
ENSG0000010	-	-	ENSG0000010546	'SYNGR4	-	19:48364394-48376376	-	-	IGV_LINK	0.969721	0.066043	0	0.508055	OK	1.03122	0.07033	0	0.512582	OK	NOTEST	0.090725
ENSG0000010	-	-	ENSG0000010547	'CLEC11A	-	19:50723328-50725718	-	-	IGV_LINK	142.549	6.44491	1.05158	11.8382	OK	112.404	5.08667	0.761642	9.41171	OK	OK	-0.34144
ENSG0000010	-	-	ENSG0000010547	'CCDC114	-	19:48296456-48330553	-	-	IGV_LINK	14.5458	0.345371	0	2.26051	OK	11.3435	0.284455	0	1.8348	OK	NOTEST	-0.27995
ENSG0000010	-	-	ENSG0000010548	'CARD8	-	19:48170691-48258199	-	-	IGV_LINK	1363.43	36.5393	1.27318	71.8054	OK	1367.4	38.1373	0	77.4788	OK	OK	0.061752
ENSG0000010	-	-	ENSG0000010548	'LIG1	-	19:48115444-48170603	-	-	IGV_LINK	913.477	25.217	2.78164	47.6524	OK	830.136	23.6003	2.99905	44.2015	OK	OK	-0.09559
ENSG0000010	-	-	ENSG0000010549	'SIGLEC6	-	19:51519524-51531856	-	-	IGV_LINK	3.87888	0.281045	0	0.895633	OK	8.2498	0.437564	0	1.17886	OK	NOTEST	0.638696
ENSG0000010	-	-	ENSG0000010549	'ZNF175	-	19:51571297-51592508	-	-	IGV_LINK	298.674	4.33561	0	8.89223	OK	261.931	3.76142	0	7.78286	OK	OK	-0.20496
ENSG0000010	-	-	ENSG0000010549	'PLA2G4C	-	19:48047842-48110817	-	-	IGV_LINK	587.651	27.1175	5.80535	48.4296	OK	685.765	31.6396	5.73856	57.5407	OK	OK	0.222511
ENSG0000010	-	-	ENSG0000010550	'SIGLEC5	-	19:51611926-51646889	-	-	IGV_LINK	20.2942	0.522834	0	1.24419	OK	8.71328	0.224596	0	0.832393	OK	NOTEST	-1.21902
ENSG0000010	-	-	ENSG0000010550	'CABP5	-	19:48029952-48044053	-	-	IGV_LINK	0	0	0	0	OK	1.03122	0.053645	0	0.461089	OK	NOTEST	inf
ENSG0000010	-	-	ENSG0000010550	'HAS1	-	19:51713111-51723994	-	-	IGV_LINK	0	0	0	0	OK	1.03122	0.099118	0	0.440762	OK	NOTEST	inf
ENSG0000010	-	-	ENSG0000010551	'RAB3D	-	19:11296138-11354944	-	-	IGV_LINK	419.281	7.53736	0	23.7282	OK	796.856	15.7429	0	43.3718	OK	OK	1.06257
ENSG0000010	-	-	ENSG0000010551	'DBP	-	19:48615327-48637438	-	-	IGV_LINK	457.708	21.1956	0	105.55	OK	319.68	14.1869	0	67.117	OK	OK	-0.5792
ENSG0000010	-	-	ENSG0000010551	'TMEM205	-	19:11296138-11354944	-	-	IGV_LINK	2003.08	167.336	39.7085	294.964	OK	2377.25	205.881	49.6484	362.114	OK	OK	0.299063
ENSG0000010	-	-	ENSG0000010551	'CAPS	-	19:5904840-5915877	-	-	IGV_LINK	395.646	8.47633	0.473811	16.4788	OK	421.087	11.284	0	23.7957	OK	OK	0.412772
ENSG0000010	-	-	ENSG0000010552	'PLPPR2	-	19:11355385-11365698	-	-	IGV_LINK	409.222	9.71716	2.26061	17.1737	OK	556.861	13.6897	4.00518	23.3743	OK	OK	0.494488
ENSG0000010	-	-	ENSG0000010552	'FAM83E	-	19:48600809-48614854	-	-	IGV_LINK	0	0	0	0	OK	0	0	0	0	OK	NOTEST	0
ENSG0000010	-	-	ENSG0000010553	'RASIP1	-	19:48720586-48740721	-	-	IGV_LINK	212.369	4.58671	0.955597	8.21782	OK	189.745	3.94104	0.737201	7.14487	OK	OK	-0.21888
ENSG0000010	-	-	ENSG0000010554	'THEG	-	19:361746-376670	-	-	IGV_LINK	4.8486	0.276152	0	0.732501	OK	1.03122	0.058802	0	0.334942	OK	NOTEST	-2.23153
ENSG0000010	-	-	ENSG0000010555	'FGF21	-	19:48755558-48758330	-	-	IGV_LINK	0	0	0	0	OK	0	0	0	0	OK	NOTEST	0
ENSG0000010	-	-	ENSG0000010555	'BCAT2	-	19:48795061-48811029	-	-	IGV_LINK	479.042	20.9276	6.45549	35.3998	OK	619.766	27.56	8.67772	46.4423	OK	OK	0.397167
ENSG0000010	-	-	ENSG0000010555	'MIER2	-	19:301443-344815	-	-	IGV_LINK	270.552	10.0961	0	21.6878	OK	316.586	11.8669	0	25.6192	OK	OK	0.233143
ENSG0000010	-	-	ENSG0000010555	'PLEKHA4	-	19:48837096-48868632	-	-	IGV_LINK	705.957	19.7022	3.05467	36.3497	OK	468.176	12.1953	1.78123	22.6093	OK	OK	-0.69204
ENSG0000010	-	-	ENSG0000010556	'PPP2R1A	-	19:52190038-52231294	-	-	IGV_LINK	3639.36	94.8549	26.1239	163.586	OK	4003.22	108.096	26.2106	189.981	OK	OK	0.188513
ENSG0000010	-	-	ENSG0000010557	'TNPO2	-	19:12699193-12724011	-	-	IGV_LINK	1543.8	27.3143	0	54.975	OK	1488.06	25.6477	2.2108	49.0846	OK	OK	-0.09083
ENSG0000010	-	-	ENSG0000010558	'WDR83OS	-	19:12643274-12681902	-	-	IGV_LINK	1196.64	120.272	0	297.01	OK	1409.68	134.172	0	340.428	OK	OK	0.157778
ENSG0000010	-	-	ENSG0000010560	'CACNG7	-	19:53909334-53943941	-	-	IGV_LINK	0	0	0	0	OK	0	0	0	0	OK	NOTEST	0
ENSG0000010	-	-	ENSG0000010560	'GCDH	-	19:12891025-12919276	-	-	IGV_LINK	238.551	9.76498	1.17826	18.3517	OK	272.243	10.9636	1.23095	20.6962	OK	OK	0.167031
ENSG0000010	-	-	ENSG0000010560	'LILRB5	-	19:54249430-54257301	-	-	IGV_LINK	82.4262	1.45556	0	2.92239	OK	335.148	6.07167	1.41521	10.7281	OK	OK	2.06051
ENSG0000010	-	-	ENSG0000010561	'KLF1	-	19:12884422-12887181	-	-	IGV_LINK	1.93944	0.076999	0	0.235805	OK	1.03122	0.040975	0	0.153359	OK	NOTEST	-0.91011
ENSG0000010	-	-	ENSG0000010561	'DNASE2	-	19:12875210-12884088	-	-	IGV_LINK	1483.67	55.3371	20.453	90.2213	OK	2044.92	75.703	28.2683	123.138	OK	OK	0.452102
ENSG0000010	-	-	ENSG0000010561	'MAST1	-	19:12763002-12874951	-	-	IGV_LINK	3.87888	0.237116	0	5.36243	OK	10.3122	0.559609	0	10.0454	OK	NOTEST	1.23883
ENSG0000010	-	-	ENSG0000010561	'LENG1	-	19:54137727-54159882	-	-	IGV_LINK	203.641	8.38007	0	19.4585	OK	126.841	5.22403	0	15.0978	OK	OK	-0.6818
ENSG0000010	-	-	ENSG0000010561	'PRPF31	-	19:54102727-54131719	-	-	IGV_LINK	892.143	28.2974	4.99969	51.5951	OK	946.665	29.9172	3.34612	56.4883	OK	OK	0.080305
ENSG0000010	-	-	ENSG0000010561	'TFPT	-	19:54102727-54131719	-	-	IGV_LINK	195.884	14.4154	0	38.855	OK	236.151	16.976	0	44.1426	OK	OK	0.23589
ENSG0000010	-	-	ENSG0000010563	'JAK3	-	19:17824779-17848071	-	-	IGV_LINK	1432.28	18.8821	6.30754	31.4567	OK	1579.84	20.5687	7.22342	33.914	OK	OK	0.123429
ENSG0000010	-	-	ENSG0000010564	'RPL18A	-	19:17859875-17864153	-	-	IGV_LINK	10315.9	1206.24	356.76	2055.71	OK	7525.88	879.037	325.955	1432.12	OK	OK	-0.45652
ENSG0000010	-	-	ENSG0000010564	'SLC5A5	-	19:17871972-17895174	-	-	IGV_LINK	3.87888	0.064227	0	0.285236	OK	3.09367	0.299333	0	0.867187	OK	NOTEST	2.2205
ENSG0000010	-	-	ENSG0000010564	'KCNN1	-	19:17951292-18000080	-	-	IGV_LINK	23.2733	0.575487	0	1.4908	OK	4.1249	0.077532	0	0.612713	OK	OK	-2.89192
ENSG0000010	-	-	ENSG0000010564	'ARRDC2	-	19:18001131-18014102	-	-	IGV_LINK	1897.74	55.3201	12.2179	98.4223	OK	1711.83	50.6608	10.4199	90.9016	OK	OK	-0.12694
ENSG0000010	-	-	ENSG0000010564	'PIK3R2	-	19:18153117-18178117	-	-	IGV_LINK	644.564	11.393	0	39.227	OK	695.286	11.4023	0	37.8729	OK	OK	0.001177
ENSG0000010	-	-	ENSG0000010564	'RAB3A	-	19:18193181-18204074	-	-	IGV_LINK	41.698	2.11619	0	6.20903	OK	41.249	2.28692	0	6.70353	OK	OK	0.111937
ENSG0000010	-	-	ENSG0000010565	'PDE4C	-	19:18204729-18255419	-	-	IGV_LINK	80.4868	2.09942	0	5.40972	OK	55.6861	1.51314	0	4.64759	OK	OK	-0.47245
ENSG0000010	-	-	ENSG0000010565	'ISYNA1	-	19:18418863-18438301	-	-	IGV_LINK	934.811	31.5777	0.983876	62.1715	OK	185.62	6.21377	0	14.7753	OK	OK	-2.34536
ENSG0000010	-	-	ENSG0000010565	'ELL	-	19:18441418-18522127	-	-	IGV_LINK	428.616	6.46419	1.80903	11.1193	OK	446.52	9.01858	0.355246	17.6819	OK	OK	0.480432
ENSG0000010	-	-	ENSG0000010566	'CRTC1	-	19:18683676-18782333	-	-	IGV_LINK	376.252	6.52401	1.47848	11.5695	OK	351.648	6.20093	1.72479	10.6771	OK	OK	-0.07328
ENSG0000010	-	-	ENSG0000010566	'COMP	-	19:18782772-18791314	-	-	IGV_LINK	0	0	0	0	OK	0	0	0	0	OK	NOTEST	0
ENSG0000010	-	-	ENSG0000010566	'UPK1A	-	19:35666515-35678483	-	-	IGV_LINK	2.90916	0.156057	0	0.481396	OK	0	0	0	0	OK	NOTEST	'-inf
ENSG0000010	-	-	ENSG0000010566	'COPE	-	19:18899513-18946831	-	-	IGV_LINK	1576.77	95.488	12.858	178.118	OK	1863.42	114.005	15.2393	212.771	OK	OK	0.255707
ENSG0000010	-	-	ENSG0000010567	'DDX49	-	19:18899513-18946831	-	-	IGV_LINK	845.596	35.7661	0	83.3151	OK	897.166	34.169	0	75.4168	OK	OK	-0.0659
ENSG0000010	-	-	ENSG0000010567	'ETV2	-	19:35641744-35644871	-	-	IGV_LINK	4.8486	0.433694	0	1.21947	OK	5.15612	0.46216	0	1.25324	OK	NOTEST	0.091713
ENSG0000010	-	-	ENSG0000010567	'ATP4A	-	19:35550042-35581954	-	-	IGV_LINK	0.969721	0.114455	0	0.940968	OK	1.03122	0.016438	0	0.343225	OK	NOTEST	-2.79963

ENSG000001	-	-	ENSG0000010567	'ARMC6	-	19:18990887-19060311	-	-	IGV_LINK	334.554	9.39649	0	27.6942	OK	415.584	11.1448	0	30.1938	OK	OK	0.246175
ENSG000001	-	-	ENSG0000010567	'TMEM147	-	19:35533411-35547526	-	-	IGV_LINK	749.594	56.8438	18.0728	95.6148	OK	864.167	65.9684	22.9361	109.001	OK	OK	0.214771
ENSG000001	-	-	ENSG0000010567	'GAPDHS	-	19:35533411-35547526	-	-	IGV_LINK	0	0	0	0	OK	0	0	0	0	OK	NOTEST	0
ENSG000001	-	-	ENSG0000010569	'ELOCP28	-	19:2511218-2702709	-	-	IGV_LINK	0	0	0	0	OK	0	0	0	0	OK	NOTEST	0
ENSG000001	-	-	ENSG0000010569	'MAG	-	19:35292124-35313804	-	-	IGV_LINK	4.8486	0.463908	0	1.50998	OK	4.1249	0.103209	0	0.590172	OK	NOTEST	-2.16827
ENSG000001	-	-	ENSG0000010569	'TMEM59L	-	19:18571729-18621039	-	-	IGV_LINK	0.969721	0.039473	0	2.23827	OK	1.03122	0.040377	0	1.967	OK	NOTEST	0.032665
ENSG000001	-	-	ENSG0000010569	'HAMP	-	19:35280715-35285143	-	-	IGV_LINK	48.486	5.60586	0	11.7387	OK	32.9992	3.56738	0	7.93551	OK	OK	-0.65207
ENSG000001	-	-	ENSG0000010569	'USF2	-	19:35268977-35279821	-	-	IGV_LINK	2099.44	113.066	30.9325	195.2	OK	1804.64	89.4247	27.707	151.142	OK	OK	-0.33843
ENSG000001	-	-	ENSG0000010569	'LSR	-	19:35248329-35267964	-	-	IGV_LINK	803.898	28.3637	5.87526	50.8521	OK	873.448	33.7411	8.04312	59.4391	OK	OK	0.250463
ENSG000001	-	-	ENSG0000010570	'KXD1	-	19:18557761-18569387	-	-	IGV_LINK	1110.33	57.5501	12.5686	102.532	OK	1134.35	64.483	17.5468	111.419	OK	OK	0.164099
ENSG000001	-	-	ENSG0000010570	'FKBP8	-	19:18531612-18544077	-	-	IGV_LINK	3845.91	138.927	53.9474	223.906	OK	3718.6	135.884	51.4427	220.326	OK	OK	-0.03195
ENSG000001	-	-	ENSG0000010570	'SUGP1	-	19:19276017-19358755	-	-	IGV_LINK	637.106	31.4977	0	72.3451	OK	563.049	24.7632	0	59.2546	OK	OK	-0.34704
ENSG000001	-	-	ENSG0000010570	'HPN	-	19:35040505-35107318	-	-	IGV_LINK	3544.33	121.758	44.1212	199.395	OK	3880.5	133.844	43.2166	224.472	OK	OK	0.136534
ENSG000001	-	-	ENSG0000010570	'ZNF14	-	19:19710470-19733097	-	-	IGV_LINK	142.549	2.89338	0.578402	5.20836	OK	118.591	2.40809	0.21676	4.59942	OK	OK	-0.26487
ENSG000001	-	-	ENSG0000010571	'SCN1B	-	19:35030465-35040449	-	-	IGV_LINK	230.793	7.63735	1.43911	13.8356	OK	175.308	4.91237	0.907605	8.91713	OK	OK	-0.63665
ENSG000001	-	-	ENSG0000010571	'PBX4	-	19:19561706-19618916	-	-	IGV_LINK	117.336	8.37234	0	16.8596	OK	262.962	19.4264	5.11852	33.7342	OK	OK	1.21431
ENSG000001	-	-	ENSG0000010572	'ERF	-	19:42230185-42255157	-	-	IGV_LINK	615.772	21.1619	0	54.6724	OK	794.043	25.6552	0	56.6724	OK	OK	0.277784
ENSG000001	-	-	ENSG0000010572	'GSK3A	-	19:42230185-42255157	-	-	IGV_LINK	1143.3	39.8645	6.80974	72.9193	OK	1251.91	41.0991	7.80602	74.3921	OK	OK	0.044001
ENSG000001	-	-	ENSG0000010572	'ATP13A1	-	19:19645197-19663693	-	-	IGV_LINK	1478.82	37.3064	7.16112	67.4516	OK	1373.59	31.4076	7.38475	55.4305	OK	OK	-0.24831
ENSG000001	-	-	ENSG0000010573	'ZNF574	-	19:41998320-42081549	-	-	IGV_LINK	237.582	4.55487	1.13687	7.97287	OK	259.869	4.94125	1.31911	8.56339	OK	OK	0.117466
ENSG000001	-	-	ENSG0000010573	'GRIK5	-	19:41998320-42081549	-	-	IGV_LINK	23.2733	0.45565	0	1.65447	OK	12.3747	0.217263	0	0.976393	OK	NOTEST	-1.06848
ENSG000001	-	-	ENSG0000010573	'SIPA1L3	-	19:37907227-38208372	-	-	IGV_LINK	740.866	10.8199	0	26.4776	OK	915.728	13.3522	0	31.0111	OK	OK	0.30339
ENSG000001	-	-	ENSG0000010575	'ZNF85	-	19:20923221-20950697	-	-	IGV_LINK	58.1832	3.76469	0	8.79168	OK	61.8735	3.10591	0	7.21672	OK	OK	-0.27752
ENSG000001	-	-	ENSG0000010575	'ETHE1	-	19:43506718-43536130	-	-	IGV_LINK	318.068	25.0983	7.11431	43.0824	OK	430.021	32.6537	10.9977	54.3097	OK	OK	0.379653
ENSG000001	-	-	ENSG0000010576	'CADM4	-	19:43622367-43639839	-	-	IGV_LINK	59.153	1.66229	0	3.56233	OK	56.7174	1.59472	0	3.28493	OK	OK	-0.05987
ENSG000001	-	-	ENSG0000010577	'SMG9	-	19:43727991-43754990	-	-	IGV_LINK	576.984	28.7126	0.737972	56.6872	OK	501.175	23.5156	1.71383	45.3173	OK	OK	-0.28807
ENSG000001	-	-	ENSG0000010577	'AVL9	-	7:32495425-32762924	-	-	IGV_LINK	783.534	11.0653	0	26.2653	OK	828.074	13.2941	0	31.1219	OK	OK	0.264744
ENSG000001	-	-	ENSG0000010578	'RUNDC3B	-	7:87503632-87832296	-	-	IGV_LINK	39.7585	0.866256	0	3.08549	OK	52.5925	1.20407	0	4.69805	OK	OK	0.475053
ENSG000001	-	-	ENSG0000010579	'CFAP69	-	7:90245173-90311063	-	-	IGV_LINK	207.52	7.20199	0	15.9071	OK	141.278	6.21522	0	14.2761	OK	OK	-0.21259
ENSG000001	-	-	ENSG0000010579	'GTPBP10	-	7:90335222-91210590	-	-	IGV_LINK	244.37	7.37404	0	23.7584	OK	234.088	9.01743	0	28.9943	OK	OK	0.290261
ENSG000001	-	-	ENSG0000010580	'RASA4	-	7:102482444-102679295	-	-	IGV_LINK	683.653	23.3241	0	67.0698	OK	491.894	22.7038	0	63.598	OK	OK	-0.03889
ENSG000001	-	-	ENSG0000010581	'CDK6	-	7:92604920-92917187	-	-	IGV_LINK	868.87	4.39096	1.54762	7.2343	OK	547.58	2.7771	0.811508	4.7427	OK	OK	-0.66096
ENSG000001	-	-	ENSG0000010581	'PMPCB	-	7:103297421-103989516	-	-	IGV_LINK	1051.18	41.822	0	84.5943	OK	1059.07	42.821	0.393921	85.248	OK	OK	0.034056
ENSG000001	-	-	ENSG0000010582	'DNAJC2	-	7:103297421-103989516	-	-	IGV_LINK	450.92	13.3505	0	34.2103	OK	477.457	14.1448	0	37.3321	OK	OK	0.083385
ENSG000001	-	-	ENSG0000010582	'TFPI2	-	7:93591572-93911265	-	-	IGV_LINK	218.187	6.12796	0.956027	11.2999	OK	787.856	31.5068	6.53382	56.4797	OK	OK	2.36218
ENSG000001	-	-	ENSG0000010582	'BET1	-	7:93962761-94011113	-	-	IGV_LINK	346.19	19.3841	0.026527	38.7417	OK	540.362	30.1283	4.9427	55.3138	OK	OK	0.636244
ENSG000001	-	-	ENSG0000010583	'NAMPT	-	7:106248284-106286326	-	-	IGV_LINK	1570.95	46.3197	0	96.8029	OK	4807.57	135.309	6.76687	263.851	OK	OK	1.54656
ENSG000001	-	-	ENSG0000010584	'TWISTNB	-	7:19695461-19709087	-	-	IGV_LINK	148.367	2.21817	0.284644	4.1517	OK	176.339	5.21053	0	13.3623	OK	OK	1.23206
ENSG000001	-	-	ENSG0000010585	'PIK3CG	-	7:106865277-106907145	-	-	IGV_LINK	315.159	4.08264	1.26408	6.9012	OK	234.088	3.32001	0.468711	6.1713	OK	OK	-0.29832
ENSG000001	-	-	ENSG0000010585	'PON3	-	7:95359943-95396368	-	-	IGV_LINK	42.6677	2.28573	0	5.30993	OK	10.3122	0.487419	0	1.28949	OK	OK	-2.22942
ENSG000001	-	-	ENSG0000010585	'PON2	-	7:95404862-95435329	-	-	IGV_LINK	1135.54	52.0357	18.1922	85.8791	OK	2388.32	101.982	38.656	165.308	OK	OK	0.970746
ENSG000001	-	-	ENSG0000010585	'ITGB8	-	7:20328298-20415754	-	-	IGV_LINK	1250.94	9.17203	3.56984	14.7742	OK	310.399	2.17193	0.628963	3.71489	OK	OK	-2.07826
ENSG000001	-	-	ENSG0000010585	'HBP1	-	7:107168960-107578464	-	-	IGV_LINK	1394.46	54.6879	0	120.945	OK	1396.28	54.9892	0	122.098	OK	OK	0.007927
ENSG000001	-	-	ENSG0000010586	'DUS4L	-	7:107168960-107578464	-	-	IGV_LINK	157.095	5.94698	0	21.034	OK	166.027	6.22553	0	21.7409	OK	OK	0.06604
ENSG000001	-	-	ENSG0000010586	'SP4	-	7:21428033-21514822	-	-	IGV_LINK	171.641	1.62474	0	3.29422	OK	139.215	1.31806	0	2.86806	OK	OK	-0.30179
ENSG000001	-	-	ENSG0000010587	'WDR91	-	7:135183838-135211534	-	-	IGV_LINK	1635.92	32.1785	0.902649	63.4544	OK	1852.08	44.2414	0	89.5067	OK	OK	0.459298
ENSG000001	-	-	ENSG0000010587	'DNAH11	-	7:21543214-21946084	-	-	IGV_LINK	1907.44	26.5608	0	67.6256	OK	1753.08	31.7958	0	79.6078	OK	OK	0.259538
ENSG000001	-	-	ENSG0000010587	'CBLL1	-	7:107742816-107761667	-	-	IGV_LINK	316.129	9.21552	0.343282	18.0878	OK	352.679	8.72321	1.04982	16.3966	OK	OK	-0.07921
ENSG000001	-	-	ENSG0000010588	'DLX5	-	7:97020391-97025097	-	-	IGV_LINK	20.3641	1.07545	0	2.5072	OK	14.4371	0.810186	0	1.89852	OK	OK	-0.40861
ENSG000001	-	-	ENSG0000010588	'MTPN	-	7:135926454-135977353	-	-	IGV_LINK	1884.17	38.9016	10.9537	66.8495	OK	2060.39	42.7903	11.7872	73.7933	OK	OK	0.137453
ENSG000001	-	-	ENSG0000010588	'STEAP1B	-	7:22419443-22665533	-	-	IGV_LINK	7.75776	0.406969	0	1.93414	OK	2.06245	0.186466	0	1.43558	OK	NOTEST	-1.12601
ENSG000001	-	-	ENSG0000010589	'PTN	-	7:137227340-137343865	-	-	IGV_LINK	595.408	23.2794	9.0467	37.5121	OK	52.5925	2.05778	0	4.18779	OK	OK	-3.49989
ENSG000001	-	-	ENSG0000010592	'MPP6	-	7:24573267-24694193	-	-	IGV_LINK	190.065	3.87936	0	8.06956	OK	253.681	12.4944	0	118.789	OK	OK	1.68739
ENSG000001	-	-	ENSG0000010592	'DFNA5	-	7:24698352-24758113	-	-	IGV_LINK	383.04	12.5644	2.50036	22.6285	OK	1236.44	40.4548	13.7029	67.2067	OK	OK	1.68697
ENSG000001	-	-	ENSG0000010592	'ATP6V0A4	-	7:138706294-138981318	-	-	IGV_LINK	1.93944	0.043089	0	0.676165	OK	0	0	0	0	OK	NOTEST	'-inf
ENSG000001	-	-	ENSG0000010593	'ZC3HAV1	-	7:139043519-139109719	-	-	IGV_LINK	805.838	8.10065	2.38684	13.8145	OK	894.072	9.33971	2.59318	16.0862	OK	OK	0.205339
ENSG000001	-	-	ENSG0000010594	'TTC26	-	7:139133743-139191986	-	-	IGV_LINK	187.156	4.01941	0	8.04834	OK	266.056	5.84946	0.916322	10.7826	OK	OK	0.541321
ENSG000001	-	-	ENSG0000010595	'OGDH	-	7:44606571-44709066	-	-	IGV_LINK	3032.32	46.6855	15.8913	77.4797	OK	2782.24	42.6886	15.2792	70.0981	OK	OK	-0.12912
ENSG000001	-	-	ENSG0000010595	'NPVF	-	7:25224569-25228486	-	-	IGV_LINK	0	0	0	0	OK	0	0	0	0	OK	NOTEST	0
ENSG000001	-	-	ENSG0000010596	'ADAP1	-	7:897900-975599	-	-	IGV_LINK	157.627	6.40592	0	15.7291	OK	371.332	12.5999	0	25.4612	OK	OK	0.97594
ENSG000001	-	-	ENSG0000010596	'TFEC	-	7:115935147-116159896	-	-	IGV_LINK	1017.24	20.6251	3.15888	38.0914	OK	842.511	15.7158	1.41361	30.018	OK	OK	-0.39219
ENSG000001	-	-	ENSG0000010596	'H2AFV	-	7:44826790-44848083	-	-	IGV_LINK	1380.88	57.6068	8.90905	106.305	OK	1186.94	53.2199	12.9975	93.4423	OK	OK	-0.11427
ENSG000001	-	-	ENSG0000010597	'CAV2	-	7:116209233-116508541	-	-	IGV_LINK	818.444	32.8712	0	68.966	OK	921.915	44.8544	0	105.344	OK	OK	0.448426
ENSG000001	-	-	ENSG0000010597	'CAV1	-	7:116524784-116561184	-	-	IGV_LINK	1816.29	53.8405	12.4133	95.2677	OK	2765.75	90.2462	19.2761	161.216	OK	OK	0.745175
ENSG000001	-	-	ENSG0000010597	'MET	-	7:116672389-116798386	-	-	IGV_LINK	3707.24	34.2233	9.95416	58.4925	OK	5596.46	51.1504	14.2085	88.0923	OK	OK	0.579766
ENSG000001	-	-	ENSG0000010598	'RNF32	-	7:156472195-156893230	-	-	IGV_LINK	45.5769	2.37165	0	10.0042	OK	61.8735	3.69645	0	13.9105	OK	OK	0.640247
ENSG000001	-	-	ENSG0000010598	'LMBR1	-	7:156472195-156893230	-	-	IGV_LINK	479.042	11.4882	0	25.2855	OK	515.612	12.6306	0	27.6358	OK	OK	0.136779
ENSG000001	-	-	ENSG0000010598	'NHP2P1	-	10:92046691-92291087	-	-	IGV_LINK	0	0	0	0	OK	0	0	0	0	OK	NOTEST	0
ENSG000001	-	-	ENSG0000010598	'WNT2	-	7:117276630-117323289	-	-	IGV_LINK	0	0	0	0	OK	0	0	0	0	OK	NOTEST	0
ENSG000001	-	-	ENSG0000010599	'HOXA1	-	7:27092992-27100265	-	-	IGV_LINK	9.69721	0.296445	0	1.16892	OK	42.2802	1.15939	0	2.93796	OK	OK	1.96753
ENSG000001	-	-	ENSG0000010599	'DNAJB6	-	7:157335380-157417439	-	-	IGV_LINK	1374.09	45.358	11.6457	79.0702	OK	1357.09	48.6449	14.1361	83.1538	OK	OK	0.100933
ENSG000001	-	-	ENSG0000010599	'HOXA2	-	7:27100353-27102811	-	-	IGV_LINK	16.4852	0.546719	0	1.19029	OK	6.18735	0.202384	0	0.51505	OK	NOTEST	-1.4337
ENSG000001	-	-	ENSG0000010599	'HOXA3	-	7:27106183-27157936	-	-	IGV_LINK	137.7	3.51069	0	11.0187	OK	95.9039	2.35764	0	7.30438	OK	OK	-0.57441
ENSG000001	-	-	ENSG0000010600	'LFNG	-	7:2512528-2529177	-	-	IGV_LINK	246.309	7.90848	1.89991	13.9171	OK	287.712	9.28316	2.4604	16.1059	OK	OK	0.231215
ENSG000001	-	-	ENSG0000010600	'HOXA5	-	7:27106183-27157936	-	-	IGV_LINK	72.729	4.66217	0	19.5123	OK	104.154	4.97658	0	15.856	OK	OK	0.094152
ENSG000001	-	-	ENSG0000010600	'HOXA6	-	7:27106183-27157936	-	-	IGV_LINK	19.3944	1.42842	0	6.68021	OK	7.21857	0.498951	0	3.21699	OK	OK	-1.51745
ENSG000001	-	-	ENSG0000010600	'BRAT1	-	7:2537876-2555727	-	-	IGV_LINK	978.448	17.1279	6.35887	27.8969	OK	897.166	16.4804	4.74806	28.2127	OK	OK	-0.0556
ENSG000001	-	-	ENSG0000010601	'IQCE	-	7:2558971-2614734	-	-	IGV_LINK	567.286	6.11164	0.573651	11.6496	OK	620.797	6.9063	0.921119	12.8915	OK	OK	0.176354
ENSG000001	-	-	ENSG0000010601	'ANKRD7	-	7:118214668-118496171	-	-	IGV_LINK	4.8486	0.346395	0	1.00102	OK	5.15612	0.433577	0	1.18424	OK	NOTEST	0.323871
ENSG000001	-	-	ENSG0000010601	'VIPR2	-	7:159006521-159144957	-	-	IGV_LINK	20.3641	0.327286	0	0.823306	OK	15.4684	0.231211	0	0.569307	OK	NOTEST	-0.50134
ENSG000001	-	-	ENSG0000010602	'TSPAN12	-	7:120787319-120858402	-	-	IGV_LINK	4766.18	190.594	51.2793	329.909	OK	1802.58	72.4154	22.1092	122.722	OK	OK	-1.39614
ENSG000001	-	-	ENSG0000010602	'SSBP1	-	7:141704337-141788640	-	-	IGV_LINK	847.536	90.3624	34.689	146.036	OK	964.195	104.478	41.337	167.619	OK	OK	0.209403
ENSG000001	-	-	ENSG0000010603	'HOXA13	-	7:27193502-27207259	-	-	IGV_LINK	0.969721	0.011251	0	0.144536	OK	27.8431	0.323107	0	0.707663	OK	NOTEST	4.84395
ENSG000001	-	-	ENSG0000010603	'CPED1	-	7:120988676-121297444	-	-	IGV_LINK	187.882	2.33679	0.038136	4.63544	OK	224.222	3.1764	0	6.65897	OK	OK	0.442865
ENSG000001	-	-	ENSG0000010603	'EVX1	-	7:27241428-27250493	-	-	IGV_LINK	0	0	0	0	OK	82.498	1.90527	0	3.98334	OK	OK	inf
ENSG000001	-	-	ENSG0000010604	'HIBADH	-	7:27525441-27662995	-	-	IGV_LINK	1582.58	53.0022	21.7174	84.2871	OK	2303.76	78.5855	30.8658	126.305	OK	OK	0.56821
ENSG000001	-	-	ENSG0000010605	'TAX1BP1	-	7:27733063-28243917	-	-	IGV_LINK	1891.92	43.9818	15.7333	72.2304	OK	2184.13	50.065	19.5833	80.5466	OK	OK	0.186893
ENSG000001	-	-	ENSG0000010606	'CPVL	-	7:28979966-29563670	-	-	IGV_LINK	2323.45	82.9192	29.5441	136.294	OK	3989.81	137.186	47.6691	226.703	OK	OK	0.726358
ENSG000001	-	-	ENSG0000010606	'CHN2	-	7:28979966-29563670	-	-	IGV_LINK	160.004	6.72405	0	29.041	OK	211.401	11.7652	0	54.4999	OK	OK	0.807123
ENSG000001	-	-	ENSG0000010607	'GRB10	-	7:50590062-50793462	-	-	IGV_LINK	1609.74	30.4405	1.21391	59.667	OK	1836.61	29.0756	5.16656	52.9846	OK	OK	-0.06618
ENSG000001	-	-	ENSG0000010607	'ABHD11	-	7:73735037-73738867	-	-	IGV_LINK	596.378	40.0068	9.76508	70.2485	OK	389.803	24.5295	5.02322	44.0358	OK	OK	-0.70573
ENSG000001	-	-	ENSG0000010607	'COBL	-	7:50866746-51316818	-	-	IGV_LINK	124.124	1.46468	0.049292	2.88006	OK	176.339	1.99528	0.326209	3.66436	OK	OK	0.44601
ENSG000001	-	-	ENSG0000010608	'FKBP14	-	7:29920102-30026684	-	-	IGV_LINK	124.124	1.66002	0	5.83888	OK	179.433	3.01586	0	9.55427	OK	OK	0.861366
ENSG000001	-	-	ENSG0000010608	'PLEKHA8	-	7:30027403-30130483	-	-	IGV_LINK	205.581	2.40209	0.086755	4.71743	OK	176.339	1.88659	0.202412	3.57078	OK	OK	-0.34851
ENSG000001	-	-	ENSG0000010608	'STX1A	-	7:73680968-73719672	-	-	IGV_LINK	43.6374	2.10542	0	9.57488	OK	40.2178	2.23102	0	10.7297	OK	OK	0.083595
ENSG000001	-	-	ENSG0000010610	'NOD1	-	7:30424526-30478784	-	-	IGV_LINK	270.552	7.23813	0	14.9132	OK	244.4	5.74249	0	12.3118	OK	OK	-0.33394
ENSG000001	-	-	ENSG0000010610	'GARS	-	7:30496620-30634033	-	-	IGV_LINK	820.384	23.0014	0	50.9911	OK	1151.88	31.0501	0.328869	61.7714	OK	OK	0.432876
ENSG000001	-	-	ENSG0000010611	'CRHR2	-	7:30651942-30925516	-	-	IGV_LINK	4.8486	0.292343	0	7.72273	OK	6.18735	0.392395	0	7.22932	OK	NOTEST	0.424641
ENSG000001	-	-	ENSG0000010612	'EPHB6	-	7:142855060-142871094	-	-	IGV_LINK	476.133	8.31543	1.48422	15.1466	OK	302.149	7.49291	0	15.257	OK	OK	-0.15027
ENSG000001	-	-	ENSG0000010612	'MINDY4	-	7:30651942-30925516	-	-	IGV_LINK	229.044	5.16982	0	18.4651	OK	209.946	4.72759	0	15.1524	OK	OK	-0.12901
ENSG000001	-	-	ENSG0000010612	'GHRHR	-	7:30938668-30993254	-	-	IGV_LINK	0	0	0	0	OK	0	0	0	0	OK	NOTEST	0
ENSG000001	-	-	ENSG0000010613	'NSUN5P2	-	7:72879364-72954790	-	-	IGV_LINK	461.587	17.9968	0	38.0282	OK	477.457	18.6007	0	39.175	OK	OK	0.047615
ENSG000001	-	-	ENSG0000010614	'CASP2	-	7:143288214-143307696	-	-	IGV_LINK	731.169	10.6773	4.13653	17.218	OK	588.829	8.14605	2.96054	13.3316	OK	OK	-0.39037
ENSG000001	-	-	ENSG0000010615	'CHCHD2	-	7:56101568-56106576	-	-	IGV_LINK	2978.01	230.832	94.3577	367.306	OK	3108.11	241.355	99.7435	382.966	OK	OK	0.064313
ENSG000001	-	-	ENSG0000010617	'CCL24	-	7:75811664-75823356	-	-	IGV_LINK	0.969721	0.13314	0	0.519701	OK	63.9359	8.82422	0	17.8738	OK	OK	6.05045
ENSG000001	-	-	ENSG0000010621	'HSPB1	-	7:76302543-76304295	-	-	IGV_LINK	3577.3	270.939	114.358	427.52	OK	5094.25	383.592	155.175	612.009	OK	OK	0.501605
ENSG000001	-	-	ENSG0000010623	'NPTX2	-	7:98617296-98629868	-	-	IGV_LINK	1328.52	30.1149	12.9751	47.2548	OK	4123.87	92.8987	38.3627	147.435	OK	OK	1.62518
ENSG000001	-	-	ENSG0000010624	'PDAP1	-	7:99325878-99466331	-	-	IGV_LINK	1325.61	61.4467	0	187.608	OK	1270.47	61.4945	0	194.636	OK	OK	0.001122
ENSG000001	-	-	ENSG0000010624	'BUD31	-	7:99325878-99466331	-	-	IGV_LINK	537.225	49.7318	0	194.467	OK	642.453	58.2852	0	222.048	OK	OK	0.228961
ENSG000001	-	-	ENSG0000010624	'PTCD1	-	7:99325878-99466331	-	-	IGV_LINK	323.368	3.63274	0	18.0119	OK	347.698	3.8603	0	20.636	OK	OK	0.087652
ENSG000001	-	-	ENSG0000010625	'CYP3A5	-	7:99648193-99679998	-	-	IGV_LINK	514.922	18.5646	4.10794	33.0213	OK	1104.44	41.5864	11.515	71.6578	OK	OK	1.16356
ENSG000001	-	-	ENSG0000010626	'ZKSCAN1	-	7:100015571-100041689	-	-	IGV_LINK	1585.49	10.9813	3.09853	18.8641	OK	1231.28	9.49064	0.487523	18.4938	OK	OK	-0.21048
ENSG000001	-	-	ENSG0000010626	'EIF3B	-	7:2251769-2380745	-	-	IGV_LINK	3030.38	66.6039	18.5834	114.624	OK	3226.7	80.3655	13.4925	147.238	OK	OK	0.270969
ENSG000001	-	-	ENSG0000010626	'SNX8	-	7:2251769-2380745	-	-	IGV_LINK	527.528	21.4056	0	73.756	OK	595.017	24.3438	0	83.6722	OK	OK	0.185561
ENSG000001	-	-	ENSG0000010626	'NUDT1	-	7:2242221-2251146	-	-	IGV_LINK	146.428	16.4578	2.20977	30.7058	OK	170.152	18.7228	4.29773	33.1478	OK	OK	0.186023
ENSG000001	-	-	ENSG0000010627	'PTPRZ1	-	7:121873088-122062036	-	-	IGV_LINK	0	0	0	0	OK	6.18735	0.126145	0	0.668232	OK	NOTEST	inf
ENSG000001	-	-	ENSG0000010629	'TAF6	-	7:100092727-100128498	-	-	IGV_LINK	736.988	24.606	0	66.9708	OK	718.764	23.8371	0	62.0121	OK	OK	-0.0458
ENSG000001	-	-	ENSG0000010629	'WASL	-	7:123681934-123749067	-	-	IGV_LINK	768.988	10.167	4.2567	16.0773	OK	779.606	10.3102	4.20793	16.4124	OK	OK	0.020173
ENSG000001	-	-	ENSG0000010630	'HYAL4	-	7:123814138-123877478	-	-	IGV_LINK	0	0	0	0	OK	3.09367	0.081773	0	0.526418	OK	NOTEST	inf
ENSG000001	-	-	ENSG0000010630	'SPAM1	-	7:123925236-123971414	-	-	IGV_LINK	0	0	0	0	OK	0	0	0	0	OK	NOTEST	0
ENSG000001	-	-	ENSG0000010630	'AIMP2	-	7:6009244-6059230	-	-	IGV_LINK	110.548	6.0928	0	19.926	OK	113.435	6.21567	0	21.3607	OK	OK	0.028804
ENSG000001	-	-	ENSG0000010632	'TFR2	-	7:100620415-100642779	-	-	IGV_LINK	35.8797	0.983478	0	2.12818	OK	37.1241	1.32739	0	3.14641	OK	OK	0.432626
ENSG000001	-	-	ENSG0000010632	'FSCN3	-	7:127580617-127602144	-	-	IGV_LINK	5.94094	0.247902	0	3.30975	OK	2.06245	0.056304	0	1.01772	OK	NOTEST	-2.13845
ENSG000001	-	-	ENSG0000010633	'MOSPD3	-	7:100612101-100615384	-	-	IGV_LINK	229.824	13.9756	3.36531	24.5859	OK	250.588	15.0604	4.25398	25.8668	OK	OK	0.107847
ENSG000001	-	-	ENSG0000010633	'PAX4	-	7:127610291-127618114	-	-	IGV_LINK	1.93944	0.10287	0	0.431716	OK	0	0	0	0	OK	NOTEST	'-inf
ENSG000001	-	-	ENSG0000010633	'PCOLCE	-	7:100572227-100608175	-	-	IGV_LINK	203.641	15.7576	0	47.101	OK	328.961	17.8411	0	46.5866	OK	OK	0.17915
ENSG000001	-	-	ENSG0000010633	'FBXO24	-	7:100572227-100608175	-	-	IGV_LINK	12.6064	0.714077	0	5.90519	OK	5.15612	0.304342	0	4.6155	OK	OK	-1.23039
ENSG000001	-	-	ENSG0000010634	'PPP1R17	-	7:31686714-31708455	-	-	IGV_LINK	0.969721	0.02608	0	0.324398	OK	0	0	0	0	OK	NOTEST	'-inf
ENSG000001	-	-	ENSG0000010634	'RBM28	-	7:128297684-128343908	-	-	IGV_LINK	706.926	15.4662	0	31.4854	OK	574.392	15.8592	0	33.4555	OK	OK	0.036201
ENSG000001	-	-	ENSG0000010634	'USP42	-	7:6104883-6161564	-	-	IGV_LINK	251.158	3.0039	0.811616	5.19619	OK	242.338	2.85702	0.626583	5.08747	OK	OK	-0.07233
ENSG000001	-	-	ENSG0000010634	'IMPDH1	-	7:128392276-128410252	-	-	IGV_LINK	481.951	14.6132	3.31977	25.9065	OK	639.359	21.1758	5.05084	37.3008	OK	OK	0.535152
ENSG000001	-	-	ENSG0000010635	'AGFG2	-	7:100539210-100568219	-	-	IGV_LINK	288.977	11.327	2.25227	20.4017	OK	145.403	5.18339	0.504366	9.8624	OK	OK	-1.1278

ENSG0000010	-	-	ENSG0000010635	'LSM5	-	7:32485331-32495283	-	-	IGV_LINK	287.037	26.5266	5.17689	47.8763	OK	322.773	29.5712	5.4363	53.7061	OK	OK	0.156753
ENSG0000010	-	-	ENSG0000010636	'SERPINE1	-	7:101127088-101139266	-	-	IGV_LINK	525.589	9.81607	3.66614	15.966	OK	3201.95	59.8236	25.8734	93.7737	OK	OK	2.6075
ENSG0000010	-	-	ENSG0000010636	'AP1S1	-	7:101154396-101161596	-	-	IGV_LINK	750.564	47.3239	17.5472	77.1006	OK	1250.88	83.0168	32.7212	133.312	OK	OK	0.810835
ENSG0000010	-	-	ENSG0000010638	'MOGAT3	-	7:101195006-101201021	-	-	IGV_LINK	9.69721	0.278897	0	0.686987	OK	37.1241	1.19111	0	2.54433	OK	OK	2.09451
ENSG0000010	-	-	ENSG0000010639	'C1GALT1	-	7:7156933-7248651	-	-	IGV_LINK	260.855	9.41501	0	19.7758	OK	439.302	16.1396	1.56718	30.7119	OK	OK	0.777567
ENSG0000010	-	-	ENSG0000010639	'PLOD3	-	7:101205976-101224190	-	-	IGV_LINK	1555.43	45.8446	0	92.413	OK	1829.39	51.0497	0	103.03	OK	OK	0.155151
ENSG0000010	-	-	ENSG0000010639	'RPA3	-	7:7636517-8094272	-	-	IGV_LINK	349.099	28.6509	0	59.5863	OK	416.615	35.536	0.627199	70.4447	OK	OK	0.310702
ENSG0000010	-	-	ENSG0000010640	'ZNHIT1	-	7:101205976-101224190	-	-	IGV_LINK	1091.91	48.8606	0	98.5896	OK	1360.19	63.7392	0	132.068	OK	OK	0.383509
ENSG0000010	-	-	ENSG0000010640	'CLDN15	-	7:101232091-101238820	-	-	IGV_LINK	240.491	8.55383	2.16693	14.9407	OK	246.463	9.43911	2.03845	16.8398	OK	OK	0.142082
ENSG0000010	-	-	ENSG0000010641	'NOBOX	-	7:144397239-144410227	-	-	IGV_LINK	0	0	0	0	OK	0	0	0	0	OK	NOTEST	0
ENSG0000010	-	-	ENSG0000010641	'GLCCI1	-	7:7636517-8094272	-	-	IGV_LINK	411.584	9.75999	0	26.4293	OK	315.124	8.56997	0	25.427	OK	OK	-0.18759
ENSG0000010	-	-	ENSG0000010643	'MYL10	-	7:101613324-101629296	-	-	IGV_LINK	0	0	0	0	OK	0	0	0	0	OK	NOTEST	0
ENSG0000010	-	-	ENSG0000010644	'PHF14	-	7:10973871-11169630	-	-	IGV_LINK	544.983	11.4241	2.73659	20.1117	OK	577.486	11.3629	2.85247	19.8733	OK	OK	-0.00776
ENSG0000010	-	-	ENSG0000010645	'NRF1	-	7:129611713-129757082	-	-	IGV_LINK	311.28	6.99107	0.318681	13.6634	OK	256.775	5.60717	0	11.2972	OK	OK	-0.31824
ENSG0000010	-	-	ENSG0000010646	'TMEM106B	-	7:12211240-12243367	-	-	IGV_LINK	498.436	11.381	0	24.2035	OK	597.079	12.7589	0	25.5559	OK	OK	0.16488
ENSG0000010	-	-	ENSG0000010646	'EZH2	-	7:148807382-148884321	-	-	IGV_LINK	399.525	9.43249	0.999995	17.865	OK	159.84	4.13986	0	9.21367	OK	OK	-1.18806
ENSG0000010	-	-	ENSG0000010647	'CEP41	-	7:130393770-130442433	-	-	IGV_LINK	120.245	3.73473	0	8.45059	OK	135.09	4.42006	0	9.53276	OK	OK	0.243063
ENSG0000010	-	-	ENSG0000010647	'ZNF862	-	7:149838366-149867479	-	-	IGV_LINK	735.048	12.9778	0	30.6894	OK	413.521	6.79228	0	16.4673	OK	OK	-0.93408
ENSG0000010	-	-	ENSG0000010648	'SFRP4	-	7:37683795-38025695	-	-	IGV_LINK	35.8797	1.31207	0	6.00695	OK	212.432	5.58113	0	18.0539	OK	OK	2.08871
ENSG0000010	-	-	ENSG0000010648	'MEST	-	7:130486041-130687432	-	-	IGV_LINK	69.8199	2.38188	0	8.23483	OK	220.682	9.01738	0	22.7206	OK	OK	1.92061
ENSG0000010	-	-	ENSG0000010651	'MEOX2	-	7:15611211-15686812	-	-	IGV_LINK	10.6669	0.258279	0	0.585083	OK	160.871	3.89711	0.864816	6.92941	OK	OK	3.9154
ENSG0000010	-	-	ENSG0000010652	'ANKMY2	-	7:16599775-16645817	-	-	IGV_LINK	489.709	12.4353	0	26.502	OK	548.612	15.801	0	39.8099	OK	OK	0.345569
ENSG0000010	-	-	ENSG0000010652	'ACTR3C	-	7:150243915-150338150	-	-	IGV_LINK	108.609	5.20036	0	17.3864	OK	145.403	7.97493	0	27.192	OK	OK	0.616862
ENSG0000010	-	-	ENSG0000010653	'POU6F2	-	7:38977997-39493095	-	-	IGV_LINK	0.969721	0.038883	0	0.474996	OK	1.03122	0.026069	0	0.455761	OK	NOTEST	-0.57683
ENSG0000010	-	-	ENSG0000010653	'TSPAN13	-	7:16753534-16784536	-	-	IGV_LINK	93.0932	2.732	0.284842	5.17916	OK	164.996	4.84737	1.10266	8.59207	OK	OK	0.827243
ENSG0000010	-	-	ENSG0000010653	'RARRES2	-	7:150338316-150341674	-	-	IGV_LINK	4586.78	425.559	165.827	685.291	OK	7970.34	761.47	271.763	1251.18	OK	OK	0.839429
ENSG0000010	-	-	ENSG0000010654	'AC004837.1	-	7:39566375-39610320	-	-	IGV_LINK	0.969721	0.139596	0	1.55006	OK	2.06237	0.297765	0	2.33961	OK	NOTEST	1.09291
ENSG0000010	-	-	ENSG0000010654	'AGR2	-	7:16791810-16833433	-	-	IGV_LINK	0	0	0	0	OK	0	0	0	0	OK	NOTEST	0
ENSG0000010	-	-	ENSG0000010654	'AHR	-	7:17279833-17558909	-	-	IGV_LINK	592.019	5.91922	0	14.5007	OK	1198.52	12.4795	0	30.161	OK	OK	1.07608
ENSG0000010	-	-	ENSG0000010655	'CHCHD3	-	7:132784867-133082088	-	-	IGV_LINK	800.989	34.5754	10.7822	58.3685	OK	802.293	39.4499	10.6177	68.2821	OK	OK	0.190279
ENSG0000010	-	-	ENSG0000010656	'GIMAP2	-	7:150685696-150693641	-	-	IGV_LINK	580.863	26.6009	9.38568	43.8162	OK	372.272	18.8588	2.92859	34.7889	OK	OK	-0.49624
ENSG0000010	-	-	ENSG0000010656	'TMEM176B	-	7:150791284-150805120	-	-	IGV_LINK	11695.8	683.105	0	1382.86	OK	20427.5	1195.82	0	2720.42	OK	OK	0.807825
ENSG0000010	-	-	ENSG0000010657	'GLI3	-	7:41960949-42237870	-	-	IGV_LINK	87.2748	0.927832	0	3.09762	OK	65.9984	0.585709	0	1.79363	OK	OK	-0.66368
ENSG0000010	-	-	ENSG0000010658	'PSMA2	-	7:42908725-42948958	-	-	IGV_LINK	1556.24	94.0438	18.257	169.831	OK	1415.51	83.088	0	171.318	OK	OK	-0.17869
ENSG0000010	-	-	ENSG0000010659	'MRPL32	-	7:42908725-42948958	-	-	IGV_LINK	604.136	34.3763	0	76.7911	OK	573.361	33.4191	0	75.3605	OK	OK	-0.04074
ENSG0000010	-	-	ENSG0000010660	'COA1	-	7:43582757-43729717	-	-	IGV_LINK	817.474	40.4651	2.37001	78.5603	OK	789.918	42.8607	7.41482	78.3066	OK	OK	0.082976
ENSG0000010	-	-	ENSG0000010660	'BLVRA	-	7:43758679-43807342	-	-	IGV_LINK	832.99	52.6904	21.7658	83.6151	OK	570.267	35.7921	12.6972	58.887	OK	OK	-0.5579
ENSG0000010	-	-	ENSG0000010660	'URGCP	-	7:43866557-43926411	-	-	IGV_LINK	831.932	15.4043	0	34.0301	OK	1242.63	23.0134	0	47.252	OK	OK	0.579139
ENSG0000010	-	-	ENSG0000010660	'TMEM248	-	7:66921224-66958551	-	-	IGV_LINK	2022.84	39.6075	9.76697	69.448	OK	2298.6	48.4712	11.0166	85.9257	OK	OK	0.291354
ENSG0000010	-	-	ENSG0000010661	'STAG3L4	-	7:67295607-67321526	-	-	IGV_LINK	248.248	13.1291	0	26.5663	OK	213.464	10.6698	0	21.6173	OK	OK	-0.29924
ENSG0000010	-	-	ENSG0000010661	'RHEB	-	7:151466011-151520120	-	-	IGV_LINK	787.413	42.5369	14.7388	70.3351	OK	1136.41	63.6411	23.316	103.966	OK	OK	0.581243
ENSG0000010	-	-	ENSG0000010661	'PRKAG2	-	7:151556110-151879223	-	-	IGV_LINK	608.015	16.995	0	34.1177	OK	633.172	20.1664	0.004445	40.3284	OK	OK	0.246846
ENSG0000010	-	-	ENSG0000010662	'AEBP1	-	7:44104360-44114562	-	-	IGV_LINK	3153.53	57.5675	13.9646	101.17	OK	478.488	9.27078	1.04547	17.4961	OK	OK	-2.63449
ENSG0000010	-	-	ENSG0000010662	'POLD2	-	7:44114680-44124358	-	-	IGV_LINK	1183.06	53.1737	13.8283	92.5191	OK	1404.53	62.6861	21.4865	103.886	OK	OK	0.237433
ENSG0000010	-	-	ENSG0000010663	'MYL7	-	7:44138863-44141332	-	-	IGV_LINK	0	0	0	0	OK	0	0	0	0	OK	NOTEST	0
ENSG0000010	-	-	ENSG0000010663	'GCK	-	7:44144270-44198170	-	-	IGV_LINK	0.969721	0.037215	0	0.435195	OK	4.1249	0.272024	0	0.916721	OK	NOTEST	2.86978
ENSG0000010	-	-	ENSG0000010663	'BCL7B	-	7:73536355-73558002	-	-	IGV_LINK	810.686	36.1512	13.2841	59.0183	OK	921.915	40.9368	14.6807	67.1929	OK	OK	0.179356
ENSG0000010	-	-	ENSG0000010663	'YKT6	-	7:44200967-44214294	-	-	IGV_LINK	967.781	28.4374	7.18906	49.6858	OK	1121.97	33.372	9.99967	56.7444	OK	OK	0.230849
ENSG0000010	-	-	ENSG0000010663	'TBL2	-	7:73568931-73578791	-	-	IGV_LINK	495.527	14.5603	3.90986	25.2108	OK	659.984	17.9883	5.60318	30.3733	OK	OK	0.305013
ENSG0000010	-	-	ENSG0000010664	'GALNTL5	-	7:151956378-152019934	-	-	IGV_LINK	0	0	0	0	OK	0	0	0	0	OK	NOTEST	0
ENSG0000010	-	-	ENSG0000010666	'CLIP2	-	7:74289474-74405943	-	-	IGV_LINK	1104.51	12.845	2.63532	23.0547	OK	623.891	7.78579	1.17443	14.3971	OK	OK	-0.7223
ENSG0000010	-	-	ENSG0000010668	'EIF4H	-	7:74174244-74197101	-	-	IGV_LINK	5870.69	143.968	52.2081	235.728	OK	5901.7	144.546	53.1387	235.954	OK	OK	0.005782
ENSG0000010	-	-	ENSG0000010668	'LIMK1	-	7:74082932-74122525	-	-	IGV_LINK	724.381	15.2456	5.95717	24.5341	OK	648.641	13.5303	4.68819	22.3725	OK	OK	-0.1722
ENSG0000010	-	-	ENSG0000010668	'SPATA6L	-	9:4490443-4666674	-	-	IGV_LINK	232.733	8.75221	0	24.1107	OK	249.556	9.57855	0	28.5662	OK	OK	0.130159
ENSG0000010	-	-	ENSG0000010668	'SLC1A1	-	9:4490443-4666674	-	-	IGV_LINK	980.387	15.7462	4.70392	26.7885	OK	1269.44	21.4693	5.34063	37.5979	OK	OK	0.447268
ENSG0000010	-	-	ENSG0000010668	'LHX2	-	9:124001669-124033301	-	-	IGV_LINK	0	0	0	0	OK	2.06245	0.054331	0	0.346156	OK	NOTEST	inf
ENSG0000010	-	-	ENSG0000010669	'FKTN	-	9:105558129-105641118	-	-	IGV_LINK	553.71	4.34657	1.58137	7.11177	OK	575.424	5.54193	0	12.8235	OK	OK	0.35051
ENSG0000010	-	-	ENSG0000010670	'FSD1L	-	9:105447795-105552433	-	-	IGV_LINK	84.3657	1.1152	0	2.48521	OK	83.5292	1.103	0	3.04255	OK	OK	-0.01588
ENSG0000010	-	-	ENSG0000010671	'CNTNAP3	-	9:39072766-39288315	-	-	IGV_LINK	84.3657	1.75432	0	4.37181	OK	202.12	3.61433	0	7.70095	OK	OK	1.04281
ENSG0000010	-	-	ENSG0000010672	'SPIN1	-	9:88388418-88478694	-	-	IGV_LINK	1613.61	26.3712	4.94356	47.7989	OK	1331.31	19.5786	5.76682	33.3904	OK	OK	-0.42969
ENSG0000010	-	-	ENSG0000010673	'NMRK1	-	9:75060572-75088217	-	-	IGV_LINK	720.502	38.5847	12.6709	64.4986	OK	818.793	42.7	15.3368	70.0632	OK	OK	0.146206
ENSG0000010	-	-	ENSG0000010677	'TMEM245	-	9:109015151-109119945	-	-	IGV_LINK	1677.62	14.4498	5.0114	23.8883	OK	1029.16	8.14018	3.06931	13.211	OK	OK	-0.82792
ENSG0000010	-	-	ENSG0000010677	'PRUNE2	-	9:76611375-76906087	-	-	IGV_LINK	4524.72	42.8316	11.1583	74.5048	OK	7062.86	91.9346	19.4731	164.396	OK	OK	1.10193
ENSG0000010	-	-	ENSG0000010678	'MEGF9	-	9:120600812-120714470	-	-	IGV_LINK	643.894	5.92393	2.39649	9.45137	OK	578.517	5.32345	2.11728	8.52962	OK	OK	-0.15419
ENSG0000010	-	-	ENSG0000010678	'TRIM14	-	9:98056738-98119212	-	-	IGV_LINK	1469.13	25.077	3.94199	46.2121	OK	2262.51	34.5139	7.86505	61.1627	OK	OK	0.46081
ENSG0000010	-	-	ENSG0000010678	'CORO2A	-	9:98120974-98192640	-	-	IGV_LINK	68.8502	0.733959	0	1.49974	OK	278.431	2.9688	0.947298	4.99029	OK	OK	2.01611
ENSG0000010	-	-	ENSG0000010679	'TGFBR1	-	9:99104037-99154192	-	-	IGV_LINK	1156.88	14.1078	1.21639	26.9992	OK	1051.85	13.4648	1.06291	25.8667	OK	OK	-0.0673
ENSG0000010	-	-	ENSG0000010680	'SEC61B	-	9:99222063-99230615	-	-	IGV_LINK	718.563	108.088	42.7636	173.413	OK	780.637	117.81	49.0514	186.569	OK	OK	0.124257
ENSG0000010	-	-	ENSG0000010680	'C5	-	9:120952334-121050276	-	-	IGV_LINK	123.155	1.46407	0	3.40633	OK	109.31	1.16421	0	2.4615	OK	OK	-0.33063
ENSG0000010	-	-	ENSG0000010680	'OGN	-	9:92297357-92670265	-	-	IGV_LINK	0.969721	0.02126	0	0.550313	OK	1.03122	0.022619	0	0.616603	OK	NOTEST	0.089364
ENSG0000010	-	-	ENSG0000010681	'ASPN	-	9:92297357-92670265	-	-	IGV_LINK	23.2733	0.570877	0	3.03509	OK	51.5612	1.26539	0	5.43019	OK	OK	1.14833
ENSG0000010	-	-	ENSG0000010682	'ECM2	-	9:92297357-92670265	-	-	IGV_LINK	124.124	3.06302	0	10.5233	OK	422.802	12.3367	0	31.9157	OK	OK	2.00993
ENSG0000010	-	-	ENSG0000010682	'TLE4	-	9:79571772-79726882	-	-	IGV_LINK	190.473	4.90269	0	10.8622	OK	267.795	7.52781	0	15.1772	OK	OK	0.618658
ENSG0000010	-	-	ENSG0000010685	'LHX6	-	9:122202576-122229626	-	-	IGV_LINK	21.3339	0.374396	0	1.01054	OK	38.1553	0.653808	0	1.55545	OK	OK	0.804303
ENSG0000010	-	-	ENSG0000010685	'PTGR1	-	9:111525158-111599855	-	-	IGV_LINK	1321.73	65.1762	26.2592	104.093	OK	9348.05	448.575	150.999	746.152	OK	OK	2.78293
ENSG0000010	-	-	ENSG0000010686	'SUSD1	-	9:112040784-112175408	-	-	IGV_LINK	141.579	3.23489	0	6.50844	OK	281.524	5.71919	1.78499	9.65338	OK	OK	0.822092
ENSG0000010	-	-	ENSG0000010692	'AMBP	-	9:114060126-114078472	-	-	IGV_LINK	12.6064	0.56875	0	1.31864	OK	5.15612	0.23664	0	0.648232	OK	OK	-1.2651
ENSG0000010	-	-	ENSG0000010694	'AKNA	-	9:114334155-114394405	-	-	IGV_LINK	2200.3	28.0143	8.40529	47.6234	OK	1894.36	24.171	5.27885	43.0631	OK	OK	-0.21289
ENSG0000010	-	-	ENSG0000010695	'TNFSF8	-	9:114893342-114930595	-	-	IGV_LINK	211.399	7.1439	1.94071	12.3471	OK	218.62	8.50209	1.11297	15.8912	OK	OK	0.251107
ENSG0000010	-	-	ENSG0000010697	'DNM1	-	9:128166063-128255248	-	-	IGV_LINK	641.955	14.2496	0	35.9247	OK	123.747	3.47985	0	19.6529	OK	OK	-2.03382
ENSG0000010	-	-	ENSG0000010699	'ENG	-	9:127815011-127854756	-	-	IGV_LINK	2211.93	45.9108	16.1637	75.6579	OK	3077.18	62.7991	21.6569	103.941	OK	OK	0.451908
ENSG0000010	-	-	ENSG0000010699	'AK1	-	9:127866479-127905408	-	-	IGV_LINK	164.205	10.5449	0	34.1281	OK	172.625	11.9604	0	35.6291	OK	OK	0.181716
ENSG0000010	-	-	ENSG0000010699	'CDC37L1	-	9:4679558-4708398	-	-	IGV_LINK	133.821	4.33247	0	8.88634	OK	295.962	10.2965	2.22519	18.3678	OK	OK	1.24889
ENSG0000010	-	-	ENSG0000010701	'RLN2	-	9:5299867-5304969	-	-	IGV_LINK	7.75776	0.780042	0	1.81146	OK	3.09367	0.311708	0	0.819073	OK	OK	-1.32336
ENSG0000010	-	-	ENSG0000010701	'RLN1	-	9:5334968-5339873	-	-	IGV_LINK	1.93944	0.184787	0	0.591494	OK	1.03122	0.098443	0	0.403462	OK	NOTEST	-0.9085
ENSG0000010	-	-	ENSG0000010702	'PLGRKT	-	9:5357972-5437878	-	-	IGV_LINK	192.974	14.9734	3.72955	26.2172	OK	188.714	14.1499	3.253	25.0468	OK	OK	-0.08161
ENSG0000010	-	-	ENSG0000010702	'TBC1D13	-	9:128787203-128810432	-	-	IGV_LINK	815.535	13.244	3.35394	23.1342	OK	1016.79	15.3528	6.64422	24.0613	OK	OK	0.213155
ENSG0000010	-	-	ENSG0000010703	'RIC1	-	9:5629024-5833117	-	-	IGV_LINK	850.445	11.315	0	23.085	OK	761.044	10.6946	0	22.0635	OK	OK	-0.08135
ENSG0000010	-	-	ENSG0000010707	'KDM4C	-	9:6716494-7175648	-	-	IGV_LINK	750.564	14.1402	3.10428	25.1762	OK	828.074	15.7098	2.15314	29.2665	OK	OK	0.15186
ENSG0000010	-	-	ENSG0000010709	'DOCK8	-	9:212823-746106	-	-	IGV_LINK	3458.02	33.3945	0	81.3732	OK	3174.11	30.9514	0	78.3669	OK	OK	-0.10961
ENSG0000010	-	-	ENSG0000010710	'KANK1	-	9:212823-746106	-	-	IGV_LINK	1388.64	18.0959	0	55.0907	OK	2748.21	35.9038	0	80.7596	OK	OK	0.988472
ENSG0000010	-	-	ENSG0000010710	'ELAVL2	-	9:23690103-23826337	-	-	IGV_LINK	2.90916	0.248121	0	0.924734	OK	6.18735	0.564403	0	1.73197	OK	OK	1.18568
ENSG0000010	-	-	ENSG0000010713	'NCS1	-	9:130172577-130237304	-	-	IGV_LINK	189.095	4.30014	0	9.58813	OK	224.807	4.42665	0.080509	8.77279	OK	OK	0.041832
ENSG0000010	-	-	ENSG0000010714	'TESK1	-	9:35605304-35647099	-	-	IGV_LINK	504.255	18.6181	0	38.2627	OK	512.519	20.0807	0	40.6375	OK	OK	0.109101
ENSG0000010	-	-	ENSG0000010714	'KCNT1	-	9:135702184-135795508	-	-	IGV_LINK	6.78804	0.099973	0	3.80297	OK	17.5308	0.236223	0	3.90804	OK	NOTEST	1.24055
ENSG0000010	-	-	ENSG0000010715	'CA9	-	9:35658289-35681159	-	-	IGV_LINK	2624.06	130.225	29.6784	230.772	OK	3737.16	183.529	48.5031	318.555	OK	OK	0.495001
ENSG0000010	-	-	ENSG0000010716	'FUBP3	-	9:130578964-130638352	-	-	IGV_LINK	890.203	26.2763	0	56.4452	OK	906.447	23.4485	0	49.4719	OK	OK	-0.16426
ENSG0000010	-	-	ENSG0000010716	'TYRP1	-	9:12685438-12822131	-	-	IGV_LINK	0	0	0	0	OK	1.03122	0.094447	0	1.16321	OK	NOTEST	inf
ENSG0000010	-	-	ENSG0000010717	'CREB3	-	9:35696947-35749228	-	-	IGV_LINK	833.96	29.7715	0	99.33	OK	1094.13	38.3294	0	123.05	OK	OK	0.364522
ENSG0000010	-	-	ENSG0000010718	'RGP1	-	9:35749286-35758575	-	-	IGV_LINK	890.203	8.16658	2.54679	13.7864	OK	1067.32	9.9017	3.20184	16.6016	OK	OK	0.277944
ENSG0000010	-	-	ENSG0000010718	'MPDZ	-	9:13105703-13279590	-	-	IGV_LINK	632.258	8.10276	1.85731	14.3482	OK	1381.84	22.1651	7.55701	36.7732	OK	OK	1.4518
ENSG0000010	-	-	ENSG0000010718	'LHX3	-	9:136196249-136205109	-	-	IGV_LINK	0	0	0	0	OK	0	0	0	0	OK	NOTEST	0
ENSG0000010	-	-	ENSG0000010720	'DDX58	-	9:32455704-32526324	-	-	IGV_LINK	908.628	12.4181	5.14853	19.6877	OK	1540.65	20.9215	8.80562	33.0373	OK	OK	0.75254
ENSG0000010	-	-	ENSG0000010722	'EDF1	-	9:136862118-136866286	-	-	IGV_LINK	3234.02	377.749	162.48	593.019	OK	2973.02	349.242	150.192	548.292	OK	OK	-0.1132
ENSG0000010	-	-	ENSG0000010724	'PIP5K1B	-	9:68705413-69009176	-	-	IGV_LINK	102.79	2.40181	0	5.77034	OK	28.8743	0.655725	0	2.21785	OK	OK	-1.87296
ENSG0000010	-	-	ENSG0000010724	'GLIS3	-	9:3824126-4348392	-	-	IGV_LINK	773.837	12.4676	1.16387	23.7713	OK	545.518	7.59332	0.341504	14.8451	OK	OK	-0.71538
ENSG0000010	-	-	ENSG0000010726	'BAG1	-	9:33218364-33264720	-	-	IGV_LINK	1042.45	55.408	22.7473	88.0687	OK	742.482	39.3362	13.0028	65.6697	OK	OK	-0.49424
ENSG0000010	-	-	ENSG0000010726	'RAPGEF1	-	9:131576769-131740074	-	-	IGV_LINK	2528.06	30.0486	0.691864	59.4052	OK	2521.35	27.7337	3.01993	52.4474	OK	OK	-0.11566
ENSG0000010	-	-	ENSG0000010728	'NPDC1	-	9:137007226-137046203	-	-	IGV_LINK	890.106	45.1025	0	118.629	OK	1331.31	63.6147	0	152.767	OK	OK	0.496154
ENSG0000010	-	-	ENSG0000010728	'APBA1	-	9:69427529-69672306	-	-	IGV_LINK	90.5224	0.80811	0	1.73683	OK	240.62	2.45513	0	6.15663	OK	OK	1.60318
ENSG0000010	-	-	ENSG0000010729	'SETX	-	9:132261355-132354985	-	-	IGV_LINK	1858.95	18.6442	1.60429	35.6841	OK	1722.15	18.477	3.89746	33.0566	OK	OK	-0.013
ENSG0000010	-	-	ENSG0000010729	'SH3GL2	-	9:17579081-17797129	-	-	IGV_LINK	6.78804	0.159894	0	0.611736	OK	11.3435	0.267327	0	0.795383	OK	NOTEST	0.741489
ENSG0000010	-	-	ENSG0000010731	'PTGDS	-	9:136975091-136986410	-	-	IGV_LINK	74.6685	6.89436	0	14.0518	OK	53.6237	4.73883	0	9.57833	OK	OK	-0.54089
ENSG0000010	-	-	ENSG0000010733	'ABCA2	-	9:137007226-137046203	-	-	IGV_LINK	1950.11	27.1662	0	68.513	OK	2170.73	26.3342	0	65.2018	OK	OK	-0.04487
ENSG0000010	-	-	ENSG0000010733	'SHB	-	9:37510891-38069211	-	-	IGV_LINK	493.513	10.654	0	25.9983	OK	398.053	8.59702	0	20.9157	OK	OK	-0.30949
ENSG0000010	-	-	ENSG0000010734	'UBE2R2	-	9:33750465-33920404	-	-	IGV_LINK	1058.93	15.292	6.64807	23.9359	OK	1744.83	25.2044	11.0172	39.3917	OK	OK	0.7209
ENSG0000010	-	-	ENSG0000010736	'ABHD17B	-	9:71862451-71910931	-	-	IGV_LINK	222.066	6.96946	1.20737	12.7315	OK	257.806	7.58829	1.84802	13.3286	OK	OK	0.122729
ENSG0000010	-	-	ENSG0000010737	'EXOSC3	-	9:37510891-38069211	-	-	IGV_LINK	224.942	16.3499	0	50.588	OK	277.4	25.9495	0	76.5088	OK	OK	0.66642
ENSG0000010	-	-	ENSG0000010737	'ZFAND5	-	9:72351424-72365235	-	-	IGV_LINK	2070.35	76.4351	10.7748	142.095	OK	3745.41	144.209	17.7642	270.654	OK	OK	0.915855
ENSG0000010	-	-	ENSG0000010740	'DVL1	-	1:1331313-1349350	-	-	IGV_LINK	905.719	26.5221	6.26177	46.7824	OK	721.857	19.5786	3.97976	35.1775	OK	OK	-0.43792
ENSG0000010	-	-	ENSG0000010743	'PDLIM1	-	10:95237571-95291024	-	-	IGV_LINK	1842.47	86.3459	34.3773	138.315	OK	1763.39	82.3588	32.5607	132.157	OK	OK	-0.06821
ENSG0000010	-	-	ENSG0000010744	'CCNJ	-	10:95711778-96090238	-	-	IGV_LINK	111.518	1.71519	0	5.86982	OK	97.9664	1.76515	0	5.89749	OK	OK	0.041421
ENSG0000010	-	-	ENSG0000010744	'DNTT	-	10:96292684-96338564	-	-	IGV_LINK	0	0	0	0	OK	0	0	0	0	OK	NOTEST	0
ENSG0000010	-	-	ENSG0000010748	'GATA3	-	10:8053603-8075198	-	-	IGV_LINK	95.0326	1.89111	0.09194	3.69028	OK	48.4676	0.98525	0	2.06901	OK	OK	-0.94067
ENSG0000010	-	-	ENSG0000010751	'ATRNL1	-	10:115093364-115948992	-	-	IGV_LINK	4.8486	0.032895	0	0.56919	OK	3.09367	0.123706	0	0.694668	OK	NOTEST	1.91096
ENSG0000010	-	-	ENSG0000010752	'HPS1	-	10:98416197-98446947	-	-	IGV_LINK	1990.84	53.4526	13.908	92.9972	OK	1370.5	36.7973	8.99998	64.5947	OK	OK	-0.53866
ENSG0000010	-	-	ENSG0000010753	'PHYH	-	10:13277795-13302412	-	-	IGV_LINK	373.342	15.1965	5.04148	25.3515	OK	203.151	9.02331	1.80159	16.245	OK	OK	-0.75201
ENSG0000010	-	-	ENSG0000010755	'RASSF4	-	10:44811023-44995891	-	-	IGV_LINK	8950.52	161.732	0	419.904	OK	5227.28	97.95	0	248.547	OK	OK	-0.72349
ENSG0000010	-	-	ENSG0000010755	'DNMBP	-	10:99875576-100009919	-	-	IGV_LINK	498.436	5.69672	1.5398	9.85365	OK	317.617	3.77607	0.686261	6.86588	OK	OK	-0.59325
ENSG0000010	-	-	ENSG0000010756	'RAB11FIP2	-	10:118004915-118210153	-	-	IGV_LINK	400.495	5.57376	0	19.0396	OK	266.056	3.27415	0	7.37186	OK	OK	-0.76753
ENSG0000010	-	-	ENSG0000010756	'CXCL12	-	10:44370164-44386493	-	-	IGV_LINK	714.684	18.9916	5.50781	32.4755	OK	1051.85	28.5003	9.02501	47.9756	OK	OK	0.585613
ENSG0000010	-	-	ENSG0000010756	'ERLIN1	-	10:100150093-100229619	-	-	IGV_LINK	682.683	11.7437	0.315908	23.1714	OK	613.579	11.1665	0	24.2269	OK	OK	-0.0727

ENSG0000010	-	-	ENSG0000010758	EIF3A	-	10:119029715-119080823	-	-	IGV_LINK	4329.8	52.0251	20.0377	84.0126	OK	2723.47	32.4132	13.1093	51.7172	OK	OK	-0.68263
ENSG0000010	-	-	ENSG0000010759	PKD2L1	-	10:100288153-100330486	-	-	IGV_LINK	18.4247	2.98915	0	7.30861	OK	56.7174	6.01393	0	16.3801	OK	OK	1.00857
ENSG0000010	-	-	ENSG0000010761	CUBN	-	10:16823963-17129831	-	-	IGV_LINK	2983.83	14.4695	5.03707	23.9019	OK	2208.88	10.5715	4.66474	16.4782	OK	OK	-0.45284
ENSG0000010	-	-	ENSG0000010761	TRDMT1	-	10:17142253-17202054	-	-	IGV_LINK	117.336	1.87946	0	4.4255	OK	54.6549	1.24397	0	3.56163	OK	OK	-0.59536
ENSG0000010	-	-	ENSG0000010762	DDX50	-	10:68901277-68946847	-	-	IGV_LINK	745.715	20.1103	6.94557	33.275	OK	368.147	10.3813	2.43834	18.3242	OK	OK	-0.95395
ENSG0000010	-	-	ENSG0000010764	MAPK8	-	10:48306638-48439360	-	-	IGV_LINK	466.436	15.1187	0	30.8181	OK	309.367	11.4945	0	24.2155	OK	OK	-0.39538
ENSG0000010	-	-	ENSG0000010765	SEC23IP	-	10:119892710-119944658	-	-	IGV_LINK	772.867	15.1573	0	34.1461	OK	665.14	13.2282	0	28.1425	OK	OK	-0.1964
ENSG0000010	-	-	ENSG0000010766	ATE1	-	10:121740420-121951965	-	-	IGV_LINK	429.586	6.64297	0	15.6209	OK	290.805	4.35739	0	8.90314	OK	OK	-0.60837
ENSG0000010	-	-	ENSG0000010767	NSMCE4A	-	10:121956781-121975217	-	-	IGV_LINK	583.772	31.5602	10.3825	52.7379	OK	310.399	16.9999	4.49239	29.5074	OK	OK	-0.89258
ENSG0000010	-	-	ENSG0000010767	PLEKHA1	-	10:122374695-122442602	-	-	IGV_LINK	1665.01	27.8285	2.30324	53.3538	OK	1264.28	20.3445	2.38188	38.3071	OK	OK	-0.45193
ENSG0000010	-	-	ENSG0000010771	PALD1	-	10:70478820-70568449	-	-	IGV_LINK	1138.45	14.6396	6.36743	22.9119	OK	570.267	7.33514	2.90091	11.7694	OK	OK	-0.99698
ENSG0000010	-	-	ENSG0000010773	UNC5B	-	10:71212569-71302864	-	-	IGV_LINK	1357.61	12.0433	5.18512	18.9015	OK	1138.47	10.0961	4.24528	15.947	OK	OK	-0.25443
ENSG0000010	-	-	ENSG0000010773	CDH23	-	10:71396933-71815947	-	-	IGV_LINK	158.064	2.21917	0	8.66239	OK	126.841	1.18566	0	4.31336	OK	OK	-0.90433
ENSG0000010	-	-	ENSG0000010773	VSIR	-	10:71396933-71815947	-	-	IGV_LINK	1714.47	48.7303	4.81642	92.6441	OK	1914.98	59.1634	7.47495	110.852	OK	OK	0.279888
ENSG0000010	-	-	ENSG0000010774	SPOCK2	-	10:72059034-72089032	-	-	IGV_LINK	1598.1	20.4495	5.04287	35.8561	OK	1940.77	27.4333	4.55103	50.3155	OK	OK	0.423862
ENSG0000010	-	-	ENSG0000010774	MICU1	-	10:72367326-72626191	-	-	IGV_LINK	966.811	25.9236	10.5847	41.2625	OK	681.64	17.5585	7.0867	28.0302	OK	OK	-0.5621
ENSG0000010	-	-	ENSG0000010775	PPP3CB	-	10:73436427-73625953	-	-	IGV_LINK	616.742	12.8419	2.05827	23.6256	OK	398.053	8.38448	0.495418	16.2735	OK	OK	-0.61507
ENSG0000010	-	-	ENSG0000010777	CCSER2	-	10:84328585-84518521	-	-	IGV_LINK	635.167	9.61215	0	20.3857	OK	391.865	7.60885	0	17.0516	OK	OK	-0.33718
ENSG0000010	-	-	ENSG0000010777	BMPR1A	-	10:86756600-86932838	-	-	IGV_LINK	404.373	7.12065	0	15.1487	OK	186.652	2.96908	0	6.41114	OK	OK	-1.26199
ENSG0000010	-	-	ENSG0000010778	MINPP1	-	10:87504874-87553460	-	-	IGV_LINK	260.855	7.11734	1.62815	12.6065	OK	392.897	9.88945	3.56821	16.2107	OK	OK	0.474551
ENSG0000010	-	-	ENSG0000010779	ACTA2	-	10:88879733-89015785	-	-	IGV_LINK	985.236	49.2721	2.08654	96.4577	OK	1355.03	66.0418	15.3897	116.694	OK	OK	0.422608
ENSG0000010	-	-	ENSG0000010779	LIPA	-	10:89213568-89414557	-	-	IGV_LINK	2341.88	65.654	0	151.104	OK	2762.65	77.7543	0	169.844	OK	OK	0.244039
ENSG0000010	-	-	ENSG0000010780	TLX1	-	10:101089320-101137789	-	-	IGV_LINK	0	0	0	0	OK	2.06245	0.231649	0	1.06358	OK	NOTEST	inf
ENSG0000010	-	-	ENSG0000010781	TWNK	-	10:100969457-100994401	-	-	IGV_LINK	160.004	4.75532	0	15.506	OK	101.06	2.61182	0	8.17029	OK	OK	-0.86449
ENSG0000010	-	-	ENSG0000010781	LZTS2	-	10:100996617-101031157	-	-	IGV_LINK	1144.27	24.5869	6.32781	42.846	OK	736.295	15.4383	4.0655	26.8112	OK	OK	-0.67137
ENSG0000010	-	-	ENSG0000010781	SFXN3	-	10:101031233-101041244	-	-	IGV_LINK	1863.8	48.2059	10.316	86.0959	OK	1370.5	34.6321	8.10743	61.1567	OK	OK	-0.4771
ENSG0000010	-	-	ENSG0000010782	KAZALD1	-	10:101061840-101068131	-	-	IGV_LINK	19.3944	1.03827	0	2.35141	OK	13.4059	0.739623	0	1.86276	OK	OK	-0.48932
ENSG0000010	-	-	ENSG0000010782	FBXW4	-	10:101610663-101695295	-	-	IGV_LINK	1120.03	41.0879	7.67608	74.4998	OK	648.641	21.7552	4.53982	38.9707	OK	OK	-0.91735
ENSG0000010	-	-	ENSG0000010783	FGF8	-	10:101770129-101780369	-	-	IGV_LINK	0.969721	0.123223	0	0.701528	OK	0	0	0	0	OK	NOTEST	'-inf
ENSG0000010	-	-	ENSG0000010783	NPM3	-	10:101781324-101783413	-	-	IGV_LINK	82.4262	7.33459	0.040171	14.629	OK	52.5925	5.47052	0	11.9928	OK	OK	-0.42304
ENSG0000010	-	-	ENSG0000010785	TNKS2	-	10:91798311-91865276	-	-	IGV_LINK	1045.36	9.84406	4.27453	15.4136	OK	697.108	6.56588	2.69924	10.4325	OK	OK	-0.58427
ENSG0000010	-	-	ENSG0000010785	PITX3	-	10:102230185-102241474	-	-	IGV_LINK	0	0	0	0	OK	0	0	0	0	OK	NOTEST	0
ENSG0000010	-	-	ENSG0000010786	GBF1	-	10:102245531-102382899	-	-	IGV_LINK	1611.68	15.8647	2.34688	29.3825	OK	1404.53	12.8845	4.87922	20.8897	OK	OK	-0.30019
ENSG0000010	-	-	ENSG0000010786	ARHGAP21	-	10:24583608-24723668	-	-	IGV_LINK	1163.66	15.6877	0	31.5024	OK	803.324	14.6671	0	39.2592	OK	OK	-0.09705
ENSG0000010	-	-	ENSG0000010786	CPEB3	-	10:92046691-92291087	-	-	IGV_LINK	160.004	1.6927	0.314763	3.07064	OK	74.2482	0.696309	0	1.40392	OK	OK	-1.28153
ENSG0000010	-	-	ENSG0000010787	FBXL15	-	10:102402616-102423136	-	-	IGV_LINK	239.521	8.39938	1.07341	15.7253	OK	178.402	5.92783	0.581013	11.2746	OK	OK	-0.50278
ENSG0000010	-	-	ENSG0000010787	CUEDC2	-	10:102423244-102432661	-	-	IGV_LINK	830.081	47.2184	19.7308	74.706	OK	518.706	29.3369	10.8009	47.8729	OK	OK	-0.68664
ENSG0000010	-	-	ENSG0000010788	SUFU	-	10:102503986-102633535	-	-	IGV_LINK	746.685	9.04175	3.66921	14.4143	OK	696.077	8.47034	3.35468	13.586	OK	OK	-0.09418
ENSG0000010	-	-	ENSG0000010789	ANKRD26	-	10:26991913-27100498	-	-	IGV_LINK	173.58	1.51893	0.23876	2.7991	OK	95.9039	1.62088	0	4.27163	OK	OK	0.093723
ENSG0000010	-	-	ENSG0000010789	ACBD5	-	10:27195213-27242130	-	-	IGV_LINK	911.537	17.4927	6.18647	28.799	OK	643.484	13.1527	3.38232	22.9231	OK	OK	-0.4114
ENSG0000010	-	-	ENSG0000010790	LHPP	-	10:124461833-124791870	-	-	IGV_LINK	256.006	10.6238	0	25.7645	OK	155.715	7.08144	0	21.1359	OK	OK	-0.58519
ENSG0000010	-	-	ENSG0000010792	LARP4B	-	10:806913-931705	-	-	IGV_LINK	1308.15	18.4989	1.55109	35.4468	OK	914.697	12.9721	1.30394	24.6402	OK	OK	-0.51203
ENSG0000010	-	-	ENSG0000010793	GTPBP4	-	10:971145-1049170	-	-	IGV_LINK	494.557	16.3655	0	39.2927	OK	409.396	10.3546	0	24.4757	OK	OK	-0.66039
ENSG0000010	-	-	ENSG0000010793	EDRF1	-	10:125683228-125764143	-	-	IGV_LINK	444.132	11.087	0.413415	21.7606	OK	262.962	6.56151	0.092536	13.0305	OK	OK	-0.75677
ENSG0000010	-	-	ENSG0000010794	BCCIP	-	10:125823545-125896436	-	-	IGV_LINK	871.779	32.8259	8.6703	56.9816	OK	486.738	18.9986	3.88487	34.1124	OK	OK	-0.78894
ENSG0000010	-	-	ENSG0000010795	MTPAP	-	10:30309800-30374448	-	-	IGV_LINK	259.885	4.79815	0	10.2414	OK	179.433	3.89531	0	8.63243	OK	OK	-0.30074
ENSG0000010	-	-	ENSG0000010795	NEURL1	-	10:103479602-103592552	-	-	IGV_LINK	19.3944	0.678138	0	2.02241	OK	10.3122	0.275892	0	0.930914	OK	OK	-1.29747
ENSG0000010	-	-	ENSG0000010795	SH3PXD2A	-	10:103594026-103855543	-	-	IGV_LINK	1553.49	7.78994	3.19541	12.3845	OK	1185.91	5.9354	2.33754	9.53326	OK	OK	-0.39227
ENSG0000010	-	-	ENSG0000010795	PITRM1	-	10:3066332-3172841	-	-	IGV_LINK	1337.24	32.0935	0	113.691	OK	1168.38	25.9397	0	72.7243	OK	OK	-0.30712
ENSG0000010	-	-	ENSG0000010796	STN1	-	10:103882541-103918205	-	-	IGV_LINK	342.311	16.1542	5.27116	27.0373	OK	216.557	9.9772	2.75018	17.2042	OK	OK	-0.6952
ENSG0000010	-	-	ENSG0000010796	MAP3K8	-	10:30433936-30461833	-	-	IGV_LINK	318.068	10.6194	2.05491	19.184	OK	320.711	10.0977	2.02587	18.1696	OK	OK	-0.07267
ENSG0000010	-	-	ENSG0000010798	DKK1	-	10:52314295-52318042	-	-	IGV_LINK	7.75776	0.608459	0	1.72912	OK	0	0	0	0	OK	OK	'-inf
ENSG0000010	-	-	ENSG0000010800	EBF3	-	10:129835282-129963841	-	-	IGV_LINK	18.4247	0.247076	0	0.577735	OK	20.6245	0.306598	0	0.693932	OK	NOTEST	0.311393
ENSG0000010	-	-	ENSG0000010801	GLRX3	-	10:130136398-130184521	-	-	IGV_LINK	714.684	36.4032	5.87947	66.9269	OK	380.522	19.5432	2.67128	36.4151	OK	OK	-0.8974
ENSG0000010	-	-	ENSG0000010801	SORCS1	-	10:106573662-107164534	-	-	IGV_LINK	2.90916	0.025109	0	0.379912	OK	1.03122	0.0089	0	0.360874	OK	NOTEST	-1.4964
ENSG0000010	-	-	ENSG0000010802	FAM208B	-	10:5684837-5763740	-	-	IGV_LINK	1300.4	13.6509	0	31.4997	OK	782.7	6.7501	0	15.0028	OK	OK	-1.01602
ENSG0000010	-	-	ENSG0000010803	XPNPEP1	-	10:109864765-109923553	-	-	IGV_LINK	1382.82	39.6219	9.29279	69.951	OK	994.101	29.307	5.91214	52.7019	OK	OK	-0.43505
ENSG0000010	-	-	ENSG0000010805	SMC3	-	10:110567690-110604636	-	-	IGV_LINK	1016.27	14.5306	6.29325	22.768	OK	616.673	8.81978	3.42936	14.2102	OK	OK	-0.72028
ENSG0000010	-	-	ENSG0000010806	SHOC2	-	10:110919546-111013667	-	-	IGV_LINK	567.286	11.6892	1.68106	21.6972	OK	449.614	10.401	1.13259	19.6695	OK	OK	-0.16845
ENSG0000010	-	-	ENSG0000010806	TFAM	-	10:58385021-58399221	-	-	IGV_LINK	376.252	8.52661	0.732243	16.321	OK	284.618	8.59416	0.267324	16.921	OK	OK	0.011383
ENSG0000010	-	-	ENSG0000010809	CCDC6	-	10:59788762-59906656	-	-	IGV_LINK	1020.15	10.3909	4.29516	16.4867	OK	680.608	6.82811	2.78883	10.8674	OK	OK	-0.60577
ENSG0000010	-	-	ENSG0000010809	CUL2	-	10:35008550-35090642	-	-	IGV_LINK	434.435	9.42578	1.55594	17.2956	OK	263.994	5.30287	1.11805	9.4877	OK	OK	-0.82984
ENSG0000010	-	-	ENSG0000010810	CCNY	-	10:35247024-35572669	-	-	IGV_LINK	1329.49	37.7607	0	77.4605	OK	753.825	21.1183	0	43.7622	OK	OK	-0.83839
ENSG0000010	-	-	ENSG0000010810	UBE2S	-	19:55385344-55407777	-	-	IGV_LINK	176.489	7.53291	0	58.6292	OK	276.368	11.4336	0	55.1844	OK	OK	0.602006
ENSG0000010	-	-	ENSG0000010810	RPL28	-	19:55385344-55407777	-	-	IGV_LINK	5990.93	722.614	133.646	1311.58	OK	5241.72	619.354	126.484	1112.23	OK	OK	-0.22246
ENSG0000010	-	-	ENSG0000010817	ZMIZ1	-	10:79069034-79316528	-	-	IGV_LINK	2187.69	19.7639	2.8657	36.6622	OK	1457.12	14.9277	0.375244	29.4802	OK	OK	-0.40488
ENSG0000010	-	-	ENSG0000010817	DNAJC12	-	10:67796664-67838166	-	-	IGV_LINK	22.3036	1.41121	0	3.0621	OK	23.7182	1.3011	0	2.77244	OK	OK	-0.1172
ENSG0000010	-	-	ENSG0000010817	PPIF	-	10:79347468-79355337	-	-	IGV_LINK	1101.6	35.937	12.8263	59.0477	OK	1110.63	36.277	13.3706	59.1835	OK	OK	0.013587
ENSG0000010	-	-	ENSG0000010818	PBLD	-	10:68282659-68407294	-	-	IGV_LINK	1750.35	48.6494	0	101.575	OK	1358.12	37.112	0.518432	73.7057	OK	OK	-0.39054
ENSG0000010	-	-	ENSG0000010821	TSPAN14	-	10:80454165-80535942	-	-	IGV_LINK	2091.69	41.2501	10.6906	71.8097	OK	1686.05	34.4408	9.35067	59.5309	OK	OK	-0.26028
ENSG0000010	-	-	ENSG0000010823	LGI1	-	10:93757808-93806272	-	-	IGV_LINK	0	0	0	0	OK	0	0	0	0	OK	NOTEST	0
ENSG0000010	-	-	ENSG0000010823	TBC1D12	-	10:94402503-94613905	-	-	IGV_LINK	370.433	4.44105	0.912742	7.96936	OK	281.524	3.44121	0.609423	6.27299	OK	OK	-0.36799
ENSG0000010	-	-	ENSG0000010824	CYP2C18	-	10:94683620-94853260	-	-	IGV_LINK	0.969706	0.042086	0	0.174654	OK	9.28102	0.354284	0	0.831027	OK	NOTEST	3.07348
ENSG0000010	-	-	ENSG0000010824	KRT23	-	17:40921429-40975926	-	-	IGV_LINK	1.93944	0.083271	0	0.528167	OK	11.3435	0.417724	0	1.09456	OK	NOTEST	2.32667
ENSG0000010	-	-	ENSG0000010825	CRYBA1	-	17:29246862-29254494	-	-	IGV_LINK	0	0	0	0	OK	0	0	0	0	OK	NOTEST	0
ENSG0000010	-	-	ENSG0000010825	NUFIP2	-	17:29255835-29294118	-	-	IGV_LINK	1524.4	8.36782	1.77187	14.9638	OK	1381.84	7.60679	1.53714	13.6764	OK	OK	-0.13756
ENSG0000010	-	-	ENSG0000010826	GIT1	-	17:29560546-30186475	-	-	IGV_LINK	1255.79	25.0462	0	66.623	OK	1205.5	24.3512	0	76.7989	OK	OK	-0.0406
ENSG0000010	-	-	ENSG0000010829	RPL19	-	17:39200282-39204727	-	-	IGV_LINK	19266.4	1952.76	456.884	3448.63	OK	14988.9	1515.38	387.716	2643.04	OK	OK	-0.36584
ENSG0000010	-	-	ENSG0000010830	FBXL20	-	17:39252643-39451286	-	-	IGV_LINK	502.315	3.26012	0	7.24394	OK	580.58	3.42509	0	7.67986	OK	OK	0.071214
ENSG0000010	-	-	ENSG0000010830	RUNDC3A	-	17:44299573-44318671	-	-	IGV_LINK	21.3339	1.07424	0	2.50942	OK	291.837	11.7482	1.82832	21.668	OK	OK	3.45105
ENSG0000010	-	-	ENSG0000010831	UBTF	-	17:44191804-44223710	-	-	IGV_LINK	1385.73	27.9347	0.466175	55.4032	OK	1299.34	26.5044	0	55.0559	OK	OK	-0.07582
ENSG0000010	-	-	ENSG0000010834	CSF3	-	17:40015360-40017813	-	-	IGV_LINK	0.969721	0.048984	0	0.481233	OK	0	0	0	0	OK	NOTEST	'-inf
ENSG0000010	-	-	ENSG0000010834	PSMD3	-	17:39980796-39997960	-	-	IGV_LINK	1470.1	46.0722	15.0638	77.0807	OK	1850.02	57.1118	19.1264	95.0972	OK	OK	0.309892
ENSG0000010	-	-	ENSG0000010834	CASC3	-	17:40140317-40172183	-	-	IGV_LINK	1633.98	31.8807	4.30888	59.4524	OK	1739.68	31.0418	5.90812	56.1755	OK	OK	-0.03847
ENSG0000010	-	-	ENSG0000010835	RAPGEFL1	-	17:40177009-40195656	-	-	IGV_LINK	95.0326	2.93358	0	6.71093	OK	157.777	4.98865	0	11.0648	OK	OK	0.76599
ENSG0000010	-	-	ENSG0000010837	RGS9	-	17:65100811-65227703	-	-	IGV_LINK	482.921	15.2057	3.98548	26.426	OK	155.715	5.34055	0.518861	10.1622	OK	OK	-1.50956
ENSG0000010	-	-	ENSG0000010837	RNF43	-	17:58301227-58417595	-	-	IGV_LINK	129.188	1.79197	0	14.9886	OK	100.065	1.43262	0	9.23775	OK	OK	-0.32289
ENSG0000010	-	-	ENSG0000010837	WNT3	-	17:46762505-46833154	-	-	IGV_LINK	70.7896	1.25354	0.028591	2.4785	OK	131.997	2.33825	0.388429	4.28807	OK	OK	0.899415
ENSG0000010	-	-	ENSG0000010838	ASPA	-	17:3439922-3557995	-	-	IGV_LINK	992.024	16.5167	1.83749	31.1958	OK	1399.37	24.7701	2.97086	46.5694	OK	OK	0.584679
ENSG0000010	-	-	ENSG0000010838	RAD51C	-	17:58692572-58735611	-	-	IGV_LINK	184.247	14.5599	2.13059	26.9892	OK	185.62	13.082	2.60612	23.5579	OK	OK	-0.15441
ENSG0000010	-	-	ENSG0000010838	SEPT4	-	17:58519836-58692055	-	-	IGV_LINK	518.8	21.1009	6.26262	35.9392	OK	434.146	16.2602	4.86322	27.6571	OK	OK	-0.37597
ENSG0000010	-	-	ENSG0000010838	MTMR4	-	17:58489528-58517905	-	-	IGV_LINK	983.297	14.6667	0	29.7649	OK	747.638	10.5257	0	21.5997	OK	OK	-0.47863
ENSG0000010	-	-	ENSG0000010839	TRIM37	-	17:58755868-59155269	-	-	IGV_LINK	315.159	5.06496	0	11.1748	OK	320.711	5.34693	0	11.8417	OK	OK	0.078161
ENSG0000010	-	-	ENSG0000010840	P2RX1	-	17:3896591-3916500	-	-	IGV_LINK	100.851	2.37128	0.164523	4.57804	OK	128.903	3.26709	0.245993	6.28818	OK	OK	0.462337
ENSG0000010	-	-	ENSG0000010840	DHX40	-	17:59565524-59608345	-	-	IGV_LINK	1429.37	25.3525	9.36119	41.3438	OK	1498.37	26.5304	10.6101	42.4507	OK	OK	0.065518
ENSG0000010	-	-	ENSG0000010841	KRT37	-	17:41402415-41425049	-	-	IGV_LINK	0	0	0	0	OK	0	0	0	0	OK	NOTEST	0
ENSG0000010	-	-	ENSG0000010842	TUBD1	-	17:59859481-59892945	-	-	IGV_LINK	115.397	4.02491	0.028567	8.02125	OK	136.122	5.12276	0.10282	10.1427	OK	OK	0.347965
ENSG0000010	-	-	ENSG0000010842	KPNB1	-	17:47649475-47685505	-	-	IGV_LINK	3343.6	79.8401	1.61861	158.062	OK	3471.1	96.8418	5.53427	188.149	OK	OK	0.278515
ENSG0000010	-	-	ENSG0000010843	GOSR2	-	17:46923074-47100323	-	-	IGV_LINK	978.857	20.4159	4.82472	36.0071	OK	1808.72	40.8471	11.5498	70.1444	OK	OK	1.00054
ENSG0000010	-	-	ENSG0000010843	PNPO	-	17:47941505-47996196	-	-	IGV_LINK	602.54	14.4559	0	51.6886	OK	828.074	18.6384	0	52.3915	OK	OK	0.366626
ENSG0000010	-	-	ENSG0000010844	TVP23BP2	-	2:74557882-74648338	-	-	IGV_LINK	0	0	0	0	OK	0	0	0	0	OK	NOTEST	0
ENSG0000010	-	-	ENSG0000010844	RPS6KB1	-	17:59893045-60018975	-	-	IGV_LINK	579.835	17.2864	0	34.9671	OK	599.127	16.9886	0	34.0808	OK	OK	-0.02508
ENSG0000010	-	-	ENSG0000010844	TRIM16L	-	17:18697997-18736118	-	-	IGV_LINK	141.579	5.61063	0.419842	10.8014	OK	126.841	4.71462	0	9.55336	OK	OK	-0.25102
ENSG0000010	-	-	ENSG0000010845	ZNF29P	-	17:15565482-15684311	-	-	IGV_LINK	0	0	0	0	OK	0	0	0	0	OK	NOTEST	0
ENSG0000010	-	-	ENSG0000010846	CDK5RAP3	-	17:47941505-47996196	-	-	IGV_LINK	4630.76	165.087	26.4788	303.694	OK	4754.98	168.414	21.4869	315.341	OK	OK	0.028789
ENSG0000010	-	-	ENSG0000010846	CBX1	-	17:48070051-48101521	-	-	IGV_LINK	786.443	21.4731	8.9032	34.0431	OK	738.357	20.9286	7.6513	34.2058	OK	OK	-0.03706
ENSG0000010	-	-	ENSG0000010846	RECQL5	-	17:75626844-75708062	-	-	IGV_LINK	777.716	25.9218	0	72.6054	OK	794.043	23.4855	0	64.7875	OK	OK	-0.1424
ENSG0000010	-	-	ENSG0000010847	PIGL	-	17:15976559-16353656	-	-	IGV_LINK	312.25	23.1413	0	78.9984	OK	315.555	21.8348	0	68.8998	OK	OK	-0.08384
ENSG0000010	-	-	ENSG0000010847	GALK1	-	17:75721327-75765711	-	-	IGV_LINK	416.98	23.6836	1.21003	46.1572	OK	465.082	27.3936	3.00831	51.7789	OK	OK	0.20995
ENSG0000010	-	-	ENSG0000010850	INTS2	-	17:61865366-61928016	-	-	IGV_LINK	285.098	3.51084	0.186878	6.8348	OK	273.275	3.02337	0.656623	5.39012	OK	OK	-0.21566
ENSG0000010	-	-	ENSG0000010850	CAMTA2	-	17:4967991-4987652	-	-	IGV_LINK	903.78	18.6395	0	38.1232	OK	952.852	24.0137	0	48.9113	OK	OK	0.365495
ENSG0000010	-	-	ENSG0000010851	MED13	-	17:61942604-62065282	-	-	IGV_LINK	1008.51	5.52199	2.27692	8.76707	OK	816.73	4.4957	1.45354	7.53786	OK	OK	-0.29664
ENSG0000010	-	-	ENSG0000010851	HOXB6	-	17:48540893-48606414	-	-	IGV_LINK	264.642	10.8802	0	33.4967	OK	182.527	6.76662	0	20.1029	OK	OK	-0.68519
ENSG0000010	-	-	ENSG0000010851	ENO3	-	17:4945651-4957131	-	-	IGV_LINK	145.458	11.3267	0	66.6203	OK	90.7478	8.60226	0	55.5122	OK	OK	-0.39694
ENSG0000010	-	-	ENSG0000010851	AC003958.1	-	17:41334631-41335733	-	-	IGV_LINK	0	0	0	0	OK	0	0	0	0	OK	NOTEST	0
ENSG0000010	-	-	ENSG0000010851	PFN1	-	17:4945651-4957131	-	-	IGV_LINK	6110.21	333.478	112.954	554.003	OK	4996.28	282.272	99.8764	464.667	OK	OK	-0.24051
ENSG0000010	-	-	ENSG0000010852	RNF167	-	17:4937129-4945222	-	-	IGV_LINK	1140.39	60.1451	7.43173	112.858	OK	1292.12	62.9419	7.40155	118.482	OK	OK	0.065573
ENSG0000010	-	-	ENSG0000010852	SLC25A11	-	17:4937129-4945222	-	-	IGV_LINK	700.138	28.2222	0	58.7773	OK	720.826	30.2794	0	65.607	OK	OK	0.101506
ENSG0000010	-	-	ENSG0000010855	RASD1	-	17:17494436-17496395	-	-	IGV_LINK	128.003	4.58455	0.812474	8.35663	OK	124.778	4.55689	0.552685	8.56109	OK	OK	-0.00873
ENSG0000010	-	-	ENSG0000010855	CHRNE	-	17:4833387-4903074	-	-	IGV_LINK	66.9107	1.65206	0	7.14663	OK	50.53	1.24824	0	5.98888	OK	OK	-0.40437
ENSG0000010	-	-	ENSG0000010855	RAI1	-	17:17681472-17837002	-	-	IGV_LINK	873.718	10.0595	0	30.0134	OK	1003.38	10.9311	0	35.4818	OK	OK	0.11988
ENSG0000010	-	-	ENSG0000010855	NUP88	-	17:5282264-5469060	-	-	IGV_LINK	525.589	27.095	0	77.3808	OK	522.831	23.199	0	63.841	OK	OK	-0.22397
ENSG0000010	-	-	ENSG0000010856	C1QBP	-	17:5282264-5469060	-	-	IGV_LINK	440.253	30.448	0	75.088	OK	568.205	38.6911	0	87.5572	OK	OK	0.345656
ENSG0000010	-	-	ENSG0000010857	SLC6A4	-	17:30194318-30236002	-	-	IGV_LINK	6.78804	0.06004	0	0.305299	OK	6.18735	0.064214	0	0.319382	OK	NOTEST	0.096947
ENSG0000010	-	-	ENSG0000010857	BLMH	-	17:30238740-30292056	-	-	IGV_LINK	725.351	21.334	4.93695	37.731	OK	691.952	20.6827	3.04728	38.3181	OK	OK	-0.04473
ENSG0000010	-	-	ENSG0000010858	CPD	-	17:30378904-30469989	-	-	IGV_LINK	1622.34	14.0973	1.22849	26.9661	OK	5500.55	47.5612	0	104.817	OK	OK	1.75437
ENSG0000010	-	-	ENSG0000010858	GOSR1	-	17:30477361-30527592	-	-	IGV_LINK	1003.66	20.108	2.06202	38.1539	OK	1255	26.5962	2.9196	50.2729	OK	OK	0.403456
ENSG0000010	-	-	ENSG0000010858	CCDC47	-	17:63622414-63819317	-	-	IGV_LINK	1787.19	51.7513	0	124.491	OK	2750.28	83.9989	0	180.054	OK	OK	0.698776
ENSG0000010	-	-	ENSG0000010859	MED31	-	17:6578147-6651634	-	-	IGV_LINK	77.5776	5.68533	0	17.8594	OK	95.9039	6.80795	0	20.2092	OK	OK	0.259977
ENSG0000010	-	-	ENSG0000010859	DRG2	-	17:18087885-18209954	-	-	IGV_LINK	583.772	18.511	0	42.3146	OK	551.705	19.539	0	51.1494	OK	OK	0.077977
ENSG0000010	-	-	ENSG0000010859	FTSJ3	-	17:63819432-63832026	-	-	IGV_LINK	939.659	26.4198	0	66.977	OK	1026.07	26.1705	0	65.6914	OK	OK	-0.01368
ENSG0000010	-	-	ENSG0000010859	AKAP10	-	17:19904301-19978343	-	-	IGV_LINK	698.199	12.2652	2.34717	22.1833	OK	883.76	18.4659	1.50953	35.4222	OK	OK	0.59029

ENSG0000010	-	-	ENSG0000010860	'ALDH3A1	-	17:19737681-19748943	-	-	IGV_LINK	30.0613	1.05928	0	2.62769	OK	175.308	6.6266	0.577262	12.6759	OK	OK	2.64518
ENSG0000010	-	-	ENSG0000010860	'SMARCD2	-	17:63832080-63843065	-	-	IGV_LINK	923.174	28.1176	6.66443	49.5709	OK	975.539	30.0216	7.26927	52.774	OK	OK	0.094527
ENSG0000010	-	-	ENSG0000010862	'ICAM2	-	17:63996070-64020634	-	-	IGV_LINK	508.134	32.4756	11.1025	53.8487	OK	408.365	24.9212	7.41406	42.4283	OK	OK	-0.38198
ENSG0000010	-	-	ENSG0000010863	'SYNGR2	-	17:78168557-78173527	-	-	IGV_LINK	3670.39	162.622	59.2343	266.011	OK	3854.72	172.565	63.7698	281.36	OK	OK	0.085612
ENSG0000010	-	-	ENSG0000010864	'B9D1	-	17:19337553-19387240	-	-	IGV_LINK	440.253	33.3675	6.45446	60.2806	OK	555.83	43.2567	8.10242	78.411	OK	OK	0.37448
ENSG0000010	-	-	ENSG0000010865	'UTP6	-	17:31860898-31901765	-	-	IGV_LINK	1063.78	59.9823	10.229	109.736	OK	894.072	44.5838	5.74512	83.4224	OK	OK	-0.42802
ENSG0000010	-	-	ENSG0000010865	'DDX5	-	17:64499615-64662068	-	-	IGV_LINK	15672.6	382.07	0	779.084	OK	14392.8	388.592	0	822.02	OK	OK	0.024419
ENSG0000010	-	-	ENSG0000010866	'C17orf75	-	17:32266175-32381886	-	-	IGV_LINK	261.825	11.7413	0	42.7539	OK	257.806	12.7584	0	42.6632	OK	OK	0.119854
ENSG0000010	-	-	ENSG0000010866	'CYTH1	-	17:78674047-78782297	-	-	IGV_LINK	2009.26	37.5502	11.7468	63.3537	OK	1993.36	39	11.3886	66.6113	OK	OK	0.054652
ENSG0000010	-	-	ENSG0000010867	'PSMD11	-	17:32423970-32483318	-	-	IGV_LINK	1139.42	35.6748	4.13894	67.2106	OK	1384.94	49.2701	5.51746	93.0227	OK	OK	0.465807
ENSG0000010	-	-	ENSG0000010867	'LGALS3BP	-	17:78971237-78980109	-	-	IGV_LINK	8311.47	242.266	76.168	408.365	OK	11732.2	344.63	94.1582	595.102	OK	OK	0.508453
ENSG0000010	-	-	ENSG0000010868	'ASIC2	-	17:33013086-34195937	-	-	IGV_LINK	0.969721	0.018175	0	0.354256	OK	3.09367	0.053328	0	0.407376	OK	NOTEST	1.55293
ENSG0000010	-	-	ENSG0000010868	'CCL7	-	17:34270220-34272242	-	-	IGV_LINK	0.969721	0.085728	0	0.429841	OK	0	0	0	0	OK	NOTEST	'-inf
ENSG0000010	-	-	ENSG0000010869	'CCL2	-	17:34255217-34257203	-	-	IGV_LINK	1758.1	171.688	75.5463	267.83	OK	819.824	79.9358	33.0841	126.787	OK	OK	-1.10288
ENSG0000010	-	-	ENSG0000010870	'CCL8	-	17:34319035-34321402	-	-	IGV_LINK	45.5769	2.35973	0	4.73911	OK	93.8415	4.86376	0.548503	9.17901	OK	OK	1.04345
ENSG0000010	-	-	ENSG0000010870	'CCL1	-	17:34360327-34363231	-	-	IGV_LINK	0	0	0	0	OK	0	0	0	0	OK	NOTEST	0
ENSG0000010	-	-	ENSG0000010873	'PEX12	-	17:35568108-35868891	-	-	IGV_LINK	163.883	4.6558	0	22.7048	OK	218.62	5.64529	0	24.3042	OK	OK	0.278018
ENSG0000010	-	-	ENSG0000010875	'KRT32	-	17:41459810-41467429	-	-	IGV_LINK	0.969721	0.034643	0	0.135689	OK	0	0	0	0	OK	NOTEST	'-inf
ENSG0000010	-	-	ENSG0000010877	'DHX58	-	17:42101403-42112733	-	-	IGV_LINK	715.654	23.7724	7.15054	40.3942	OK	832.199	26.7882	8.90139	44.675	OK	OK	0.172312
ENSG0000010	-	-	ENSG0000010877	'KAT2A	-	17:42113107-42155972	-	-	IGV_LINK	2193.84	46.3388	2.84882	89.8289	OK	2251.59	48.5719	0	104.644	OK	OK	0.0679
ENSG0000010	-	-	ENSG0000010877	'RAB5C	-	17:42113107-42155972	-	-	IGV_LINK	2240.77	90.3678	9.13105	171.605	OK	2837.88	114.493	14.4932	214.493	OK	OK	0.341377
ENSG0000010	-	-	ENSG0000010878	'NAGLU	-	17:42536171-42566277	-	-	IGV_LINK	808.747	24.6031	0	53.008	OK	917.79	28.9739	0	64.6391	OK	OK	0.235912
ENSG0000010	-	-	ENSG0000010878	'HSD17B1P1	-	17:42536171-42566277	-	-	IGV_LINK	7.75776	0.558731	0	5.8981	OK	4.1249	0.297522	0	4.49089	OK	NOTEST	-0.90916
ENSG0000010	-	-	ENSG0000010878	'HSD17B1	-	17:42536171-42566277	-	-	IGV_LINK	189.095	7.44454	0	24.7547	OK	189.745	8.67384	0	29.241	OK	OK	0.220488
ENSG0000010	-	-	ENSG0000010878	'MLX	-	17:42567067-42577831	-	-	IGV_LINK	920.265	30.3215	6.71644	53.9265	OK	995.132	34.291	6.9424	61.6395	OK	OK	0.177488
ENSG0000010	-	-	ENSG0000010879	'CNTNAP1	-	17:42678888-42699814	-	-	IGV_LINK	387.888	6.8263	0	13.8329	OK	868.291	12.3807	1.69338	23.068	OK	OK	0.858916
ENSG0000010	-	-	ENSG0000010879	'ABI3	-	17:49202790-49223225	-	-	IGV_LINK	574.075	18.089	5.92932	30.2487	OK	577.486	18.5591	6.25668	30.8616	OK	OK	0.037013
ENSG0000010	-	-	ENSG0000010879	'EZH1	-	17:42700274-42745049	-	-	IGV_LINK	1824.04	34.0756	2.72689	65.4244	OK	1807.74	36.7506	1.11641	72.3849	OK	OK	0.109029
ENSG0000010	-	-	ENSG0000010881	'DLX4	-	17:49968969-49974959	-	-	IGV_LINK	33.9402	1.44846	0	3.12698	OK	80.4355	3.3743	0	7.12218	OK	OK	1.22007
ENSG0000010	-	-	ENSG0000010881	'PPP1R9B	-	17:50133734-50150630	-	-	IGV_LINK	1861.86	30.4986	7.53092	53.4662	OK	2010.89	32.3591	8.4646	56.2536	OK	OK	0.08543
ENSG0000010	-	-	ENSG0000010882	'COL1A1	-	17:50183288-50201632	-	-	IGV_LINK	1284.88	13.3579	0.961967	25.7538	OK	1604.59	16.8298	1.41782	32.2418	OK	OK	0.33333
ENSG0000010	-	-	ENSG0000010882	'SGCA	-	17:50163522-50181178	-	-	IGV_LINK	14.5458	0.839338	0	2.23377	OK	17.5308	1.24227	0	3.00009	OK	OK	0.56565
ENSG0000010	-	-	ENSG0000010882	'PTGES3L-AARSD1	-	17:42950525-42980528	-	-	IGV_LINK	2.18437	0.299169	0	3.36629	OK	10.3051	0.792643	0	4.9487	OK	OK	1.40571
ENSG0000010	-	-	ENSG0000010882	'MRPL27	-	17:50367856-50373214	-	-	IGV_LINK	611.894	57.2175	20.1353	94.2998	OK	678.546	62.7852	23.8369	101.733	OK	OK	0.133967
ENSG0000010	-	-	ENSG0000010882	'VAT1	-	17:43014604-43025123	-	-	IGV_LINK	4547.02	102.699	39.1599	166.238	OK	3678.38	82.8476	32.9005	132.795	OK	OK	-0.30989
ENSG0000010	-	-	ENSG0000010882	'LRRC59	-	17:50373219-50397888	-	-	IGV_LINK	819.414	16.8892	6.59697	27.1815	OK	1024.01	21.1329	7.93334	34.3324	OK	OK	0.323384
ENSG0000010	-	-	ENSG0000010883	'RND2	-	17:43025240-43032036	-	-	IGV_LINK	14.5458	0.20625	0	0.558258	OK	43.3114	0.614305	0	1.27986	OK	OK	1.57456
ENSG0000010	-	-	ENSG0000010883	'ALOX12	-	17:6853860-7019659	-	-	IGV_LINK	9.69721	0.466766	0	4.90518	OK	13.4059	1.1318	0	10.6942	OK	OK	1.27785
ENSG0000010	-	-	ENSG0000010884	'HDAC5	-	17:44076745-44123702	-	-	IGV_LINK	1224.76	22.571	4.75526	40.3867	OK	1078.66	21.5758	3.48293	39.6688	OK	OK	-0.06505
ENSG0000010	-	-	ENSG0000010884	'ABCC3	-	17:50634776-50692252	-	-	IGV_LINK	3780.94	68.7987	5.50949	132.088	OK	14472.2	241.854	0	515.849	OK	OK	1.81368
ENSG0000010	-	-	ENSG0000010884	'LUC7L3	-	17:50719543-50756213	-	-	IGV_LINK	4890.3	129.381	21.1849	237.576	OK	3393.76	90.106	19.8735	160.338	OK	OK	-0.52193
ENSG0000010	-	-	ENSG0000010884	'PPY	-	17:43940803-43942468	-	-	IGV_LINK	0	0	0	0	OK	1.03122	0.225651	0	1.15895	OK	NOTEST	inf
ENSG0000010	-	-	ENSG0000010885	'MPP2	-	17:43875356-43909711	-	-	IGV_LINK	8.72748	0.168997	0	0.816264	OK	10.3122	0.300012	0	1.14573	OK	NOTEST	0.828023
ENSG0000010	-	-	ENSG0000010885	'SMURF2	-	17:64499615-64662068	-	-	IGV_LINK	649.713	11.4844	0	61.2649	OK	583.673	12.4953	0	73.7925	OK	OK	0.121707
ENSG0000010	-	-	ENSG0000010886	'DUSP3	-	17:43766120-43778988	-	-	IGV_LINK	1695.07	25.657	9.59928	41.7146	OK	2383.16	36.1279	12.9855	59.2703	OK	OK	0.493765
ENSG0000010	-	-	ENSG0000010887	'CACNG1	-	17:67044589-67056797	-	-	IGV_LINK	0	0	0	0	OK	1.03122	0.053203	0	0.199128	OK	NOTEST	inf
ENSG0000010	-	-	ENSG0000010888	'EFTUD2	-	17:44846352-44916937	-	-	IGV_LINK	1639.8	38.8163	2.04845	75.5841	OK	2076.89	46.2256	3.85937	88.5919	OK	OK	0.25203
ENSG0000010	-	-	ENSG0000010892	'HLF	-	17:55265011-55325065	-	-	IGV_LINK	512.982	7.15765	1.23285	13.0824	OK	524.894	8.78874	0	17.6133	OK	OK	0.29617
ENSG0000010	-	-	ENSG0000010893	'SLC16A6	-	17:68259181-68763882	-	-	IGV_LINK	50.4255	0.959482	0	6.78797	OK	77.3419	1.86941	0	17.8065	OK	OK	0.962254
ENSG0000010	-	-	ENSG0000010894	'PRKAR1A	-	17:68259181-68763882	-	-	IGV_LINK	3809.06	117.563	14.0202	221.106	OK	4408.17	138.459	0	292.861	OK	OK	0.236024
ENSG0000010	-	-	ENSG0000010894	'EFNB3	-	17:7705201-7711378	-	-	IGV_LINK	5.81832	0.107525	0	0.255367	OK	8.2498	0.152517	0	0.348168	OK	NOTEST	0.5043
ENSG0000010	-	-	ENSG0000010895	'FAM20A	-	17:68259181-68763882	-	-	IGV_LINK	333.584	8.03843	0	27.3233	OK	2504.85	67.249	0	158.075	OK	OK	3.06453
ENSG0000010	-	-	ENSG0000010895	'YWHAE	-	17:1344271-1400378	-	-	IGV_LINK	3619.97	184.153	42.2575	326.048	OK	3615.47	191.635	45.4481	337.821	OK	OK	0.057457
ENSG0000010	-	-	ENSG0000010895	'AC130689.1	-	17:1779484-1900082	-	-	IGV_LINK	0	0	0	0	OK	1.03122	0.247678	0	5.72458	OK	NOTEST	inf
ENSG0000010	-	-	ENSG0000010896	'MMD	-	17:55392612-55421992	-	-	IGV_LINK	171.641	4.12331	0.368297	7.87833	OK	249.556	6.14045	1.05791	11.223	OK	OK	0.57454
ENSG0000010	-	-	ENSG0000010896	'RANGRF	-	17:8287762-8295343	-	-	IGV_LINK	208.49	13.652	2.06079	25.2432	OK	150.559	10.1494	0	20.4883	OK	OK	-0.42772
ENSG0000010	-	-	ENSG0000010896	'DPH1	-	17:2030109-2043430	-	-	IGV_LINK	729.458	28.8135	4.76658	52.8604	OK	816.16	32.9575	6.71128	59.2037	OK	OK	0.193861
ENSG0000010	-	-	ENSG0000010898	'MAP2K6	-	17:69414697-69543331	-	-	IGV_LINK	117.336	4.19179	0.199137	8.18444	OK	61.8735	2.90474	0	6.07037	OK	OK	-0.52916
ENSG0000010	-	-	ENSG0000010901	'DHRS7B	-	17:21123363-21193265	-	-	IGV_LINK	347.16	20.4725	4.94651	35.9984	OK	414.552	25.5715	6.41866	44.7243	OK	OK	0.320851
ENSG0000010	-	-	ENSG0000010904	'WSB1	-	17:27294075-27315926	-	-	IGV_LINK	6872.41	189.91	25.5626	354.257	OK	5684.11	157.047	21.9017	292.192	OK	OK	-0.27412
ENSG0000010	-	-	ENSG0000010904	'RCVRN	-	17:9896319-9905621	-	-	IGV_LINK	2.90916	0.098212	0	0.330166	OK	2.06245	0.10752	0	0.41598	OK	NOTEST	0.130627
ENSG0000010	-	-	ENSG0000010906	'MYH1	-	17:10383131-10623886	-	-	IGV_LINK	0	0	0	0	OK	0	0	0	0	OK	NOTEST	0
ENSG0000010	-	-	ENSG0000010906	'SLC9A3R1	-	17:74670577-74769353	-	-	IGV_LINK	3119.59	112.819	36.8808	188.758	OK	6183.22	224.393	31.061	417.724	OK	OK	0.992009
ENSG0000010	-	-	ENSG0000010906	'MYH3	-	17:10628525-10657309	-	-	IGV_LINK	162.913	2.06308	0	5.05403	OK	96.9351	2.81406	0	7.67185	OK	OK	0.447854
ENSG0000010	-	-	ENSG0000010906	'NAT9	-	17:74770546-74776367	-	-	IGV_LINK	521.71	23.6337	5.98917	41.2782	OK	579.548	24.369	6.35035	42.3876	OK	OK	0.044201
ENSG0000010	-	-	ENSG0000010906	'TMEM104	-	17:74776482-74839779	-	-	IGV_LINK	254.067	4.13219	0	8.35116	OK	317.617	4.70466	0	9.97175	OK	OK	0.187183
ENSG0000010	-	-	ENSG0000010907	'VTN	-	17:28357580-28407197	-	-	IGV_LINK	3.87888	0.155879	0	3.07202	OK	9.28102	1.05788	0	12.9711	OK	OK	2.76268
ENSG0000010	-	-	ENSG0000010907	'TNFAIP1	-	17:28335601-28347009	-	-	IGV_LINK	1005.6	17.3487	4.92851	29.7689	OK	1282.84	21.9856	7.13235	36.8388	OK	OK	0.34173
ENSG0000010	-	-	ENSG0000010908	'IFT20	-	17:28319094-28335489	-	-	IGV_LINK	462.557	32.3963	6.49064	58.302	OK	447.552	30.6513	7.87751	53.4251	OK	OK	-0.07988
ENSG0000010	-	-	ENSG0000010908	'TMEM97	-	17:28319094-28335489	-	-	IGV_LINK	151.276	4.40111	0	10.3001	OK	146.434	5.84189	0	15.2249	OK	OK	0.408566
ENSG0000010	-	-	ENSG0000010908	'CDR2L	-	17:74987631-75005800	-	-	IGV_LINK	102.79	1.72226	0.207371	3.23716	OK	109.31	1.83212	0.200584	3.46366	OK	OK	0.089212
ENSG0000010	-	-	ENSG0000010909	'PMP22	-	17:15229776-15265707	-	-	IGV_LINK	1228.64	42.1048	18.2807	65.9289	OK	1182.82	40.565	17.5312	63.5988	OK	OK	-0.05375
ENSG0000010	-	-	ENSG0000010910	'FOXN1	-	17:28455751-28645454	-	-	IGV_LINK	0	0	0	0	OK	2.06245	0.035628	0	1.63009	OK	NOTEST	inf
ENSG0000010	-	-	ENSG0000010910	'UNC119	-	17:28455751-28645454	-	-	IGV_LINK	495.527	16.4683	0	61.7371	OK	530.05	20.0567	0	88.0718	OK	OK	0.284394
ENSG0000010	-	-	ENSG0000010910	'ALDOC	-	17:28455751-28645454	-	-	IGV_LINK	1648.52	63.3122	0	155.827	OK	2207.85	86.3886	0	212.259	OK	OK	0.448359
ENSG0000010	-	-	ENSG0000010911	'SUPT6H	-	17:28648355-28702684	-	-	IGV_LINK	2838.37	33.1485	0	69.6267	OK	3328.79	37.6882	0	76.1431	OK	OK	0.185171
ENSG0000010	-	-	ENSG0000010911	'RAB34	-	17:28714280-28718429	-	-	IGV_LINK	1195.67	63.0976	23.4285	102.767	OK	1713.9	93.2024	37.8993	148.506	OK	OK	0.562782
ENSG0000010	-	-	ENSG0000010911	'PHF12	-	17:28905249-29057220	-	-	IGV_LINK	966.811	20.3622	0.636317	40.088	OK	1091.04	19.7033	0	45.4581	OK	OK	-0.04746
ENSG0000010	-	-	ENSG0000010913	'PHOX2B	-	4:41744081-41824119	-	-	IGV_LINK	0	0	0	0	OK	0	0	0	0	OK	NOTEST	0
ENSG0000010	-	-	ENSG0000010913	'TMEM33	-	4:41935119-41960572	-	-	IGV_LINK	375.282	6.68561	0	13.8692	OK	550.674	12.2424	0.931422	23.5534	OK	OK	0.872755
ENSG0000010	-	-	ENSG0000010915	'GABRA4	-	4:46918899-47426444	-	-	IGV_LINK	0	0	0	0	OK	1.03122	0.040098	0	0.339176	OK	NOTEST	inf
ENSG0000010	-	-	ENSG0000010916	'GNRHR	-	4:67701279-68129880	-	-	IGV_LINK	7.75776	0.238655	0	1.2939	OK	6.18735	0.175369	0	1.05022	OK	NOTEST	-0.44454
ENSG0000010	-	-	ENSG0000010917	'SLAIN2	-	4:48341321-48426212	-	-	IGV_LINK	610.924	16.2532	0	39.3129	OK	555.83	17.6967	0	41.9192	OK	OK	0.122756
ENSG0000010	-	-	ENSG0000010918	'OCIAD1	-	4:48805211-48861817	-	-	IGV_LINK	1745.5	89.8897	33.4386	146.341	OK	2145.98	113.142	41.6208	184.663	OK	OK	0.331906
ENSG0000010	-	-	ENSG0000010918	'UGT2B10	-	4:68815992-68831196	-	-	IGV_LINK	2.90916	0.093254	0	0.27332	OK	10.3122	0.4648	0	1.05806	OK	NOTEST	2.31737
ENSG0000010	-	-	ENSG0000010918	'CWH43	-	4:48986246-49062081	-	-	IGV_LINK	0	0	0	0	OK	0	0	0	0	OK	NOTEST	0
ENSG0000010	-	-	ENSG0000010918	'DCUN1D4	-	4:51842999-51916837	-	-	IGV_LINK	815.535	24.2798	1.96244	46.5971	OK	643.484	28.1619	2.38518	53.9386	OK	OK	0.213991
ENSG0000010	-	-	ENSG0000010918	'USP46	-	4:52590971-52659335	-	-	IGV_LINK	138.67	1.91318	0	4.18069	OK	204.183	2.60243	0.228793	4.97608	OK	OK	0.443892
ENSG0000010	-	-	ENSG0000010919	'SULT1E1	-	4:69791871-69860152	-	-	IGV_LINK	0.969721	0.033997	0	0.191099	OK	2.06245	0.157212	0	0.469808	OK	NOTEST	2.20924
ENSG0000010	-	-	ENSG0000010920	'ODAM	-	4:70196495-70204576	-	-	IGV_LINK	0	0	0	0	OK	0	0	0	0	OK	NOTEST	0
ENSG0000010	-	-	ENSG0000010920	'SMR3A	-	4:70360760-70367158	-	-	IGV_LINK	0	0	0	0	OK	0	0	0	0	OK	NOTEST	0
ENSG0000010	-	-	ENSG0000010922	'CHIC2	-	4:53377642-54298247	-	-	IGV_LINK	154.186	9.1262	0	22.1974	OK	189.745	11.9368	0	28.4626	OK	OK	0.387335
ENSG0000010	-	-	ENSG0000010925	'NMU	-	4:55595228-55636698	-	-	IGV_LINK	0.969721	0.478365	0	1.94453	OK	11.3435	0.995826	0	2.68949	OK	OK	1.05778
ENSG0000010	-	-	ENSG0000010926	'KIAA1211	-	4:56049072-56328625	-	-	IGV_LINK	2.90916	0.140042	0	0.769373	OK	9.28102	0.192915	0	0.798143	OK	NOTEST	0.462101
ENSG0000010	-	-	ENSG0000010927	'LAMTOR3	-	4:99878335-99894490	-	-	IGV_LINK	684.623	29.7306	8.37572	51.0855	OK	713.608	31.9132	9.75244	54.0739	OK	OK	0.102202
ENSG0000010	-	-	ENSG0000010927	'PF4V1	-	4:73853188-73854155	-	-	IGV_LINK	32.0008	5.16002	0	10.5756	OK	263.994	42.7094	13.1733	72.2455	OK	OK	3.04911
ENSG0000010	-	-	ENSG0000010932	'NFKB1	-	4:102501328-102617302	-	-	IGV_LINK	1491.43	23.2101	9.36508	37.0551	OK	1296.25	20.8374	8.25786	33.4169	OK	OK	-0.15558
ENSG0000010	-	-	ENSG0000010932	'AREG	-	4:74445133-74455009	-	-	IGV_LINK	21.3339	1.12629	0	2.38518	OK	23.7182	1.3064	0	2.89596	OK	OK	0.214015
ENSG0000010	-	-	ENSG0000010932	'MANBA	-	4:102631487-102760994	-	-	IGV_LINK	1288.76	22.2707	5.20969	39.3317	OK	1302.44	23.5469	6.33563	40.7581	OK	OK	0.080389
ENSG0000010	-	-	ENSG0000010933	'UBE2D3	-	4:102794382-102868896	-	-	IGV_LINK	3315.47	134.245	36.3749	232.115	OK	3845.44	167.589	38.6645	296.513	OK	OK	0.320062
ENSG0000010	-	-	ENSG0000010933	'MAPK10	-	4:85475113-86815171	-	-	IGV_LINK	772.867	17.9521	0	47.5027	OK	812.605	18.6276	0	42.3425	OK	OK	0.053284
ENSG0000010	-	-	ENSG0000010938	'ELF2	-	4:139015788-139177218	-	-	IGV_LINK	773.837	21.1659	5.31323	37.0185	OK	806.418	20.3451	4.29909	36.391	OK	OK	-0.05706
ENSG0000010	-	-	ENSG0000010939	'NDUFC1	-	4:139266162-139476609	-	-	IGV_LINK	1250.16	129.53	29.8356	229.225	OK	1321	144.584	39.0423	250.126	OK	OK	0.158619
ENSG0000010	-	-	ENSG0000010942	'UCP1	-	4:140559433-140568805	-	-	IGV_LINK	0	0	0	0	OK	0	0	0	0	OK	NOTEST	0
ENSG0000010	-	-	ENSG0000010943	'TBC1D9	-	4:140620764-140756120	-	-	IGV_LINK	704.987	8.17149	1.96625	14.3767	OK	771.356	8.46987	3.50708	13.4327	OK	OK	0.051741
ENSG0000010	-	-	ENSG0000010944	'ZNF330	-	4:141220886-141234697	-	-	IGV_LINK	516.861	23.91	6.83226	40.9878	OK	645.547	27.4042	9.64423	45.1642	OK	OK	0.196781
ENSG0000010	-	-	ENSG0000010945	'INPP4B	-	4:142023159-142847432	-	-	IGV_LINK	225.945	3.01789	0	6.48126	OK	158.809	1.73579	0	4.21328	OK	OK	-0.79795
ENSG0000010	-	-	ENSG0000010945	'GAB1	-	4:143336761-143474568	-	-	IGV_LINK	415.04	6.441	0	12.9831	OK	562.018	8.63998	0.502629	16.7773	OK	OK	0.423745
ENSG0000010	-	-	ENSG0000010946	'KLHL2	-	4:165070607-165323156	-	-	IGV_LINK	323.887	8.33067	0	18.7354	OK	459.926	12.2787	0	26.0497	OK	OK	0.559656
ENSG0000010	-	-	ENSG0000010947	'IL2	-	4:122451469-122456725	-	-	IGV_LINK	1.93944	0.264339	0	0.826482	OK	0	0	0	0	OK	NOTEST	'-inf
ENSG0000010	-	-	ENSG0000010947	'CPE	-	4:165361193-165498320	-	-	IGV_LINK	1235.42	43.6657	9.95415	77.3772	OK	1889.2	68.9281	15.8669	121.989	OK	OK	0.658593
ENSG0000010	-	-	ENSG0000010947	'RPL34	-	4:108620565-108630412	-	-	IGV_LINK	9092.1	1870.41	653.554	3087.28	OK	6245.1	1277.89	488.067	2067.71	OK	OK	-0.54959
ENSG0000010	-	-	ENSG0000010950	'WFS1	-	4:6269848-6303265	-	-	IGV_LINK	190.065	3.7984	0	7.90209	OK	214.495	3.65821	0.588075	6.72834	OK	OK	-0.05426
ENSG0000010	-	-	ENSG0000010951	'ANXA10	-	4:167946673-168187690	-	-	IGV_LINK	0	0	0	0	OK	0	0	0	0	OK	NOTEST	0
ENSG0000010	-	-	ENSG0000010951	'GRPEL1	-	4:7058905-7068197	-	-	IGV_LINK	475.163	20.5622	5.29409	35.8303	OK	985.851	47.2623	13.3803	81.1443	OK	OK	1.20069
ENSG0000010	-	-	ENSG0000010953	'GAR1	-	4:109815509-109824740	-	-	IGV_LINK	96.9721	6.21438	0.279032	12.1497	OK	120.653	7.07378	0.678769	13.4688	OK	OK	0.186871
ENSG0000010	-	-	ENSG0000010953	'FRG1	-	4:189940787-189963204	-	-	IGV_LINK	437.344	32.9597	10.688	55.2314	OK	369.179	27.7277	6.91985	48.5356	OK	OK	-0.24938
ENSG0000010	-	-	ENSG0000010957	'CLCN3	-	4:169612632-169723673	-	-	IGV_LINK	1869.62	26.1559	7.90508	44.4067	OK	2765.75	38.5282	9.1808	67.8756	OK	OK	0.558778
ENSG0000010	-	-	ENSG0000010957	'AADAT	-	4:170060221-170091699	-	-	IGV_LINK	45.5769	1.59651	0	3.64373	OK	73.217	2.26689	0	4.65797	OK	OK	0.50579
ENSG0000010	-	-	ENSG0000010958	'GALNT7	-	4:173131927-173329694	-	-	IGV_LINK	212.369	6.9923	0	14.5345	OK	412.49	12.6962	0.376603	25.0158	OK	OK	0.860558
ENSG0000010	-	-	ENSG0000010960	'DHX15	-	4:24517440-24584550	-	-	IGV_LINK	2268.18	52.3081	17.8226	86.7936	OK	1884.05	42.4377	13.8103	71.065	OK	OK	-0.30169
ENSG0000010	-	-	ENSG0000010961	'SOD3	-	4:24789911-24800842	-	-	IGV_LINK	464.496	19.3405	6.75565	31.9254	OK	533.143	22.2177	8.62604	35.8093	OK	OK	0.200084
ENSG0000010	-	-	ENSG0000010961	'SEPSECS	-	4:25120013-25160442	-	-	IGV_LINK	774.807	11.5323	1.53964	21.525	OK	889.947	13.1416	2.43743	23.8458	OK	OK	0.188462
ENSG0000010	-	-	ENSG0000010962	'CPZ	-	4:8558724-8619761	-	-	IGV_LINK	180.241	5.08267	0.831411	9.33392	OK	4.1249	0.118587	0	0.580958	OK	OK	-5.42156
ENSG0000010	-	-	ENSG0000010965	'TRIM2	-	4:153152341-153339320	-	-	IGV_LINK	464.496	6.08514	0	12.722	OK	555.83	6.87616	0	14.6165	OK	OK	0.176313
ENSG0000010	-	-	ENSG0000010966	'SLC2A9	-	4:9771152-10054936	-	-	IGV_LINK	409.222	18.3515	4.26037	32.4427	OK	1022.98	44.2216	14.5609	73.8824	OK	OK	1.26885
ENSG0000010	-	-	ENSG0000010967	'FBXW7	-	4:152321258-152536101	-	-	IGV_LINK	465.466	9.72046	1.25976	18.1812	OK	428.99	9.25417	0	18.8425	OK	OK	-0.07092
ENSG0000010	-	-	ENSG0000010967	'NEIL3	-	4:177309835-177362943	-	-	IGV_LINK	29.0916	0.733388	0	1.51539	OK	38.1553	0.962375	0	1.9683	OK	OK	0.392024
ENSG0000010	-	-	ENSG0000010968	'TBC1D19	-	4:26576436-26755351	-	-	IGV_LINK	163.883	6.34799	0.553537	12.1424	OK	150.559	5.03256	0.341511	9.7236	OK	OK	-0.33501
ENSG0000010	-	-	ENSG0000010968	'CLNK	-	4:10486394-10684865	-	-	IGV_LINK	45.5769	0.789949	0	1.82945	OK	30.9367	0.348717	0	0.749475	OK	OK	-1.1797
ENSG0000010	-	-	ENSG0000010968	'NSD2	-	4:1871423-1982207	-	-	IGV_LINK	1241.24	12.8525	0.844814	24.8602	OK	934.29	10.4489	0	20.918	OK	OK	-0.2987
ENSG0000010	-	-	ENSG0000010968	'SH3D19	-	4:151099572-151325632	-	-	IGV_LINK	1759.07	218.716	0	855.765	OK	1600.46	164.334	0	595.904	OK	OK	-0.41243

ENSG0000010	-	-	ENSG0000010968	'STIM2	-	4:26857677-27025381	-	-	IGV_LINK	581.832	20.0723	0	40.502	OK		504.269	20.7471	0.369773	41.1244	OK		OK	0.047704
ENSG0000010	-	-	ENSG0000010970	'NKX3-2	-	4:13540829-13545050	-	-	IGV_LINK	0	0	0	0	OK		0	0	0	0	OK		NOTEST	0
ENSG0000010	-	-	ENSG0000010973	'MFSD10	-	4:2930560-2934859	-	-	IGV_LINK	1709.62	74.8317	28.0632	121.6	OK		1780.93	74.5894	29.0888	120.09	OK		OK	-0.00468
ENSG0000010	-	-	ENSG0000010973	'GLRB	-	4:157076056-157172090	-	-	IGV_LINK	16.4852	0.592612	0	1.45339	OK		18.562	0.683485	0	1.64062	OK		OK	0.205822
ENSG0000010	-	-	ENSG0000010974	'BST1	-	4:15604538-15738313	-	-	IGV_LINK	101.821	4.29661	0	19.9792	OK		163.965	7.51991	0	30.2846	OK		OK	0.807514
ENSG0000010	-	-	ENSG0000010975	'RAPGEF2	-	4:159104177-159360169	-	-	IGV_LINK	1168.51	23.9577	0	48.2983	OK		1100.32	22.6159	0	46.3933	OK		OK	-0.08315
ENSG0000010	-	-	ENSG0000010975	'HGFAC	-	4:3441886-3449495	-	-	IGV_LINK	7.75776	0.246315	0	0.65532	OK		0	0	0	0	OK		NOTEST	'-inf
ENSG0000010	-	-	ENSG0000010976	'SNX25	-	4:185159664-185395899	-	-	IGV_LINK	372.373	7.01265	0	14.7536	OK		422.802	7.82739	0.172843	15.4819	OK		OK	0.158573
ENSG0000010	-	-	ENSG0000010977	'LRP2BP	-	4:185159664-185395899	-	-	IGV_LINK	161.943	3.81468	0	12.7288	OK		71.1545	1.39588	0	5.89743	OK		OK	-1.45039
ENSG0000010	-	-	ENSG0000010977	'UFSP2	-	4:185396020-185425985	-	-	IGV_LINK	773.837	30.1648	7.56127	52.7684	OK		593.986	24.1184	2.46032	45.7765	OK		OK	-0.32273
ENSG0000010	-	-	ENSG0000010978	'KLF3	-	4:38612700-38701042	-	-	IGV_LINK	633.227	8.12251	2.38888	13.8561	OK		854.886	10.3445	3.89634	16.7927	OK		OK	0.348868
ENSG0000010	-	-	ENSG0000010979	'KLHL5	-	4:39045038-39126857	-	-	IGV_LINK	578.923	14.321	3.82208	24.8199	OK		502.207	12.5495	3.48836	21.6106	OK		OK	-0.1905
ENSG0000010	-	-	ENSG0000010979	'FAM149A	-	4:186104418-186172667	-	-	IGV_LINK	1423.61	59.7778	15.4228	104.133	OK		921.1	42.0403	9.8195	74.261	OK		OK	-0.50784
ENSG0000010	-	-	ENSG0000010980	'NCAPG	-	4:17810901-18021876	-	-	IGV_LINK	99.8812	1.83288	0	4.35185	OK		114.466	1.8603	0	3.83203	OK		OK	0.021428
ENSG0000010	-	-	ENSG0000010981	'UGDH	-	4:39498754-39639090	-	-	IGV_LINK	427.647	13.1571	0	30.465	OK		1020.91	29.7258	0	66.0772	OK		OK	1.17588
ENSG0000010	-	-	ENSG0000010981	'PPARGC1A	-	4:23560922-23904089	-	-	IGV_LINK	425.707	5.09256	0.765686	9.41943	OK		211.401	3.14877	0	7.80947	OK		OK	-0.6936
ENSG0000010	-	-	ENSG0000010983	'DDX25	-	11:125903246-125943702	-	-	IGV_LINK	4.8486	0.146926	0	0.714538	OK		0	0	0	0	OK		NOTEST	'-inf
ENSG0000010	-	-	ENSG0000010984	'CRYAB	-	11:111908564-111926872	-	-	IGV_LINK	5292.73	423.737	135.074	712.399	OK		14093.8	1142.61	274.663	2010.56	OK		OK	1.43109
ENSG0000010	-	-	ENSG0000010985	'DBX1	-	11:20156154-20160613	-	-	IGV_LINK	2.90916	0.135777	0	0.364423	OK		4.1249	0.192702	0	0.488164	OK		NOTEST	0.505131
ENSG0000010	-	-	ENSG0000010985	'HTATIP2	-	11:20363684-20383783	-	-	IGV_LINK	933.841	55.7422	22.184	89.3004	OK		1918.08	113.23	43.4376	183.022	OK		OK	1.02241
ENSG0000010	-	-	ENSG0000010986	'CTSC	-	11:88293591-88337787	-	-	IGV_LINK	4041.8	147.769	31.1146	264.423	OK		6363.69	228.029	53.1291	402.93	OK		OK	0.625879
ENSG0000010	-	-	ENSG0000010988	'CCDC34	-	11:27330826-27363868	-	-	IGV_LINK	160.974	6.78298	0.477257	13.0887	OK		147.465	5.31095	0.169321	10.4526	OK		OK	-0.35295
ENSG0000010	-	-	ENSG0000010990	'ZBTB16	-	11:114059592-114356571	-	-	IGV_LINK	171.641	5.19766	0	27.7296	OK		565.111	16.2334	0	147.656	OK		OK	1.64303
ENSG0000010	-	-	ENSG0000010991	'ELP4	-	11:31509699-32105755	-	-	IGV_LINK	295.765	14.7997	0	47.1406	OK		340.304	16.673	0	60.2853	OK		OK	0.171945
ENSG0000010	-	-	ENSG0000010991	'ZPR1	-	11:116773388-116788050	-	-	IGV_LINK	747.655	27.7542	4.28406	51.2243	OK		949.758	38.697	4.00201	73.392	OK		OK	0.479518
ENSG0000010	-	-	ENSG0000010991	'MTCH2	-	11:47617314-47642623	-	-	IGV_LINK	896.022	43.1067	10.758	75.4554	OK		1178.69	63.1256	15.3105	110.941	OK		OK	0.550311
ENSG0000010	-	-	ENSG0000010992	'FNBP4	-	11:47716516-47767443	-	-	IGV_LINK	2471.82	68.3425	2.01224	134.673	OK		1749.99	46.1084	2.96093	89.2559	OK		OK	-0.56775
ENSG0000010	-	-	ENSG0000010992	'TECTA	-	11:121101172-121191493	-	-	IGV_LINK	53.3346	0.417429	0	0.862533	OK		41.249	0.320537	0	0.672897	OK		NOTEST	-0.38104
ENSG0000010	-	-	ENSG0000010992	'SC5D	-	11:121292452-121308694	-	-	IGV_LINK	205.581	6.84364	0.220895	13.4664	OK		283.587	8.27127	1.98989	14.5527	OK		OK	0.273345
ENSG0000010	-	-	ENSG0000010994	'CRTAM	-	11:122838499-122872639	-	-	IGV_LINK	97.9418	3.05065	0	6.57035	OK		38.1553	1.36607	0	3.21431	OK		OK	-1.15908
ENSG0000010	-	-	ENSG0000010994	'C11orf63	-	11:122882527-122959798	-	-	IGV_LINK	158.064	3.56722	0.691932	6.4425	OK		77.3419	1.72039	0.080643	3.36013	OK		OK	-1.05207
ENSG0000010	-	-	ENSG0000010995	'B3GAT1	-	11:134378503-134411918	-	-	IGV_LINK	53.3346	0.755581	0	1.91293	OK		56.7174	0.655604	0	1.41449	OK		OK	-0.20476
ENSG0000010	-	-	ENSG0000010997	'HSPA8	-	11:123057488-123063230	-	-	IGV_LINK	12011.9	436.395	47.8452	824.945	OK		11856	446.37	42.7644	849.977	OK		OK	0.032607
ENSG0000010	-	-	ENSG0000010999	'P2RX3	-	11:57338373-57370600	-	-	IGV_LINK	0.969721	0.04653	0	0.362595	OK		11.3435	0.445182	0	1.06514	OK		NOTEST	3.25817
ENSG0000011	-	-	ENSG0000011000	'VWA5A	-	11:124115361-124147721	-	-	IGV_LINK	543.043	10.9377	3.92917	17.9463	OK		1240.56	23.8858	10.0447	37.727	OK		OK	1.12684
ENSG0000011	-	-	ENSG0000011001	'DNAJC4	-	11:64206677-64234352	-	-	IGV_LINK	669.279	46.851	0	115.376	OK		538.851	36.8315	0	92.742	OK		OK	-0.34714
ENSG0000011	-	-	ENSG0000011001	'SIAE	-	11:124622835-124697518	-	-	IGV_LINK	1437.13	25.9887	5.03647	46.9409	OK		1495.28	28.289	7.01813	49.56	OK		OK	0.12236
ENSG0000011	-	-	ENSG0000011002	'SNX15	-	11:65014112-65040572	-	-	IGV_LINK	362.675	10.526	0	22.0089	OK		365.052	10.6517	0.512202	20.7912	OK		OK	0.017127
ENSG0000011	-	-	ENSG0000011003	'LPXN	-	11:58526870-58578220	-	-	IGV_LINK	835.899	33.1263	10.1213	56.1313	OK		714.639	26.1377	8.85738	43.418	OK		OK	-0.34185
ENSG0000011	-	-	ENSG0000011004	'DTX4	-	11:59171429-59212951	-	-	IGV_LINK	384.979	4.39905	0	14.8126	OK		1472.59	17.2295	0	48.6923	OK		OK	1.96961
ENSG0000011	-	-	ENSG0000011004	'ATG2A	-	11:64894545-64917248	-	-	IGV_LINK	687.532	7.42702	0.71223	14.1418	OK		1085.88	13.6453	0.10416	27.1865	OK		OK	0.877553
ENSG0000011	-	-	ENSG0000011004	'EHD1	-	11:64851641-64888296	-	-	IGV_LINK	1163.66	27.3328	6.96417	47.7015	OK		1216.85	31.1928	6.41687	55.9687	OK		OK	0.190578
ENSG0000011	-	-	ENSG0000011004	'OSBP	-	11:59574397-59616144	-	-	IGV_LINK	1323.67	15.779	6.08008	25.478	OK		1761.33	21.7428	7.62022	35.8653	OK		OK	0.462527
ENSG0000011	-	-	ENSG0000011005	'UNC93B1	-	11:67991103-68004982	-	-	IGV_LINK	1074.45	33.6537	11.487	55.8204	OK		1199.31	39.3271	12.3976	66.2565	OK		OK	0.224758
ENSG0000011	-	-	ENSG0000011006	'PUS3	-	11:125883613-125903221	-	-	IGV_LINK	142.549	5.68069	0.117331	11.2441	OK		137.153	5.08399	0.576862	9.59112	OK		OK	-0.16011
ENSG0000011	-	-	ENSG0000011006	'DCPS	-	11:126283064-126355587	-	-	IGV_LINK	216.248	8.78396	0.753763	16.8142	OK		305.243	12.7439	1.58111	23.9066	OK		OK	0.536861
ENSG0000011	-	-	ENSG0000011006	'KMT5B	-	11:68154862-68213828	-	-	IGV_LINK	799.05	12.6603	2.88616	22.4345	OK		641.422	10.2365	2.17428	18.2987	OK		OK	-0.30659
ENSG0000011	-	-	ENSG0000011007	'FOXRED1	-	11:126202093-126278131	-	-	IGV_LINK	574.075	22.7782	0	64.805	OK		692.983	27.519	0	81.1932	OK		OK	0.272778
ENSG0000011	-	-	ENSG0000011007	'PPP6R3	-	11:68460730-68616711	-	-	IGV_LINK	1259.67	25.0248	2.2893	47.7602	OK		1242.63	21.1528	4.01428	38.2914	OK		OK	-0.24251
ENSG0000011	-	-	ENSG0000011007	'NRXN2	-	11:64606173-64723188	-	-	IGV_LINK	309.341	7.46832	0	15.0974	OK		304.211	8.77258	0	18.1522	OK		OK	0.232217
ENSG0000011	-	-	ENSG0000011007	'MS4A6A	-	11:60172013-60184666	-	-	IGV_LINK	3522.02	264.432	83.3708	445.494	OK		4310.52	342.739	100.712	584.767	OK		OK	0.374214
ENSG0000011	-	-	ENSG0000011007	'MS4A4A	-	11:60280540-60308972	-	-	IGV_LINK	437.344	24.5016	6.10089	42.9023	OK		1355.03	72.2319	23.5459	120.918	OK		OK	1.55976
ENSG0000011	-	-	ENSG0000011008	'ST3GAL4	-	11:126355639-127006058	-	-	IGV_LINK	245.339	9.81922	2.34803	17.2904	OK		229.963	10.0818	1.48889	18.6746	OK		OK	0.038068
ENSG0000011	-	-	ENSG0000011009	'CPT1A	-	11:68754619-68844410	-	-	IGV_LINK	1796.89	20.7893	8.58392	32.9946	OK		1743.8	21.9665	8.57064	35.3623	OK		OK	0.079464
ENSG0000011	-	-	ENSG0000011009	'CCND1	-	11:69641086-69675416	-	-	IGV_LINK	7942.98	141.316	0	302.152	OK		6363.69	128.79	0	276.863	OK		OK	-0.1339
ENSG0000011	-	-	ENSG0000011010	'CCDC86	-	11:60835995-60855971	-	-	IGV_LINK	400.495	13.1185	4.70406	21.533	OK		339.273	11.1206	3.7386	18.5026	OK		OK	-0.23837
ENSG0000011	-	-	ENSG0000011010	'PRPF19	-	11:60890729-60906588	-	-	IGV_LINK	1325.61	38.797	16.2231	61.371	OK		1457.12	42.9764	17.1864	68.7665	OK		OK	0.147598
ENSG0000011	-	-	ENSG0000011010	'TMEM109	-	11:60913165-60952530	-	-	IGV_LINK	2060.66	63.7088	0	130.242	OK		1652.02	47.8831	0	101.463	OK		OK	-0.41198
ENSG0000011	-	-	ENSG0000011014	'CCKBR	-	11:6259735-6272127	-	-	IGV_LINK	0	0	0	0	OK		1.03122	0.029801	0	0.574802	OK		NOTEST	inf
ENSG0000011	-	-	ENSG0000011016	'HPX	-	11:6431048-6442617	-	-	IGV_LINK	24.243	2.02207	0	4.77438	OK		38.1553	2.33933	0	5.43275	OK		OK	0.210261
ENSG0000011	-	-	ENSG0000011017	'TRIM3	-	11:6448612-6474459	-	-	IGV_LINK	386.918	15.094	2.20275	27.9852	OK		380.522	17.1196	0	35.6633	OK		OK	0.181678
ENSG0000011	-	-	ENSG0000011017	'CHORDC1	-	11:90201159-90226538	-	-	IGV_LINK	310.311	10.1406	0.434218	19.8469	OK		214.495	7.26834	0.074193	14.4625	OK		OK	-0.48044
ENSG0000011	-	-	ENSG0000011019	'FOLR1	-	11:72163321-72209241	-	-	IGV_LINK	867.9	53.0892	21.7603	84.4181	OK		502.207	30.6167	11.5924	49.641	OK		OK	-0.7941
ENSG0000011	-	-	ENSG0000011020	'ANAPC15	-	11:71998540-72112780	-	-	IGV_LINK	210.429	18.131	0	111.014	OK		196.964	17.3438	0	111.781	OK		OK	-0.06404
ENSG0000011	-	-	ENSG0000011020	'FOLR3	-	11:72114868-72139892	-	-	IGV_LINK	9.69721	0.686352	0	1.85215	OK		34.0304	2.76391	0	6.09086	OK		OK	2.00969
ENSG0000011	-	-	ENSG0000011021	'PANX1	-	11:94128927-94181972	-	-	IGV_LINK	247.279	5.36855	1.6191	9.11801	OK		379.491	8.24321	2.8788	13.6076	OK		OK	0.618673
ENSG0000011	-	-	ENSG0000011023	'ARHGEF17	-	11:73307234-73369091	-	-	IGV_LINK	3479.36	47.8707	0	108.458	OK		1725.24	24.527	0	55.8519	OK		OK	-0.96477
ENSG0000011	-	-	ENSG0000011024	'APOA5	-	11:116789366-116792420	-	-	IGV_LINK	0.969721	0.031672	0	0.274701	OK		0	0	0	0	OK		NOTEST	'-inf
ENSG0000011	-	-	ENSG0000011024	'APOA4	-	11:116820699-116823306	-	-	IGV_LINK	0	0	0	0	OK		0	0	0	0	OK		NOTEST	0
ENSG0000011	-	-	ENSG0000011024	'APOC3	-	11:116829705-116833072	-	-	IGV_LINK	0	0	0	0	OK		1.03122	0.15812	0	0.961762	OK		NOTEST	inf
ENSG0000011	-	-	ENSG0000011027	'CEP164	-	11:117232624-117413268	-	-	IGV_LINK	719.533	15.2945	0	37.8692	OK		688.858	12.2549	0	28.5679	OK		OK	-0.31965
ENSG0000011	-	-	ENSG0000011031	'RNF141	-	11:10511677-10541230	-	-	IGV_LINK	544.983	12.2355	1.4095	23.0615	OK		513.55	12.9731	0.964556	24.9816	OK		OK	0.084447
ENSG0000011	-	-	ENSG0000011031	'CEP126	-	11:101890673-102001058	-	-	IGV_LINK	273.461	3.96714	0	9.09742	OK		266.056	4.32208	0	10.1173	OK		OK	0.123626
ENSG0000011	-	-	ENSG0000011032	'EIF4G2	-	11:10783312-10809110	-	-	IGV_LINK	9319.01	197.561	21.153	373.969	OK		10821.7	224.454	34.7246	414.183	OK		OK	0.184123
ENSG0000011	-	-	ENSG0000011032	'IL10RA	-	11:117986347-118003037	-	-	IGV_LINK	2826.74	48.5236	11.5532	85.4941	OK		3460.79	60.2732	14.6978	105.849	OK		OK	0.31283
ENSG0000011	-	-	ENSG0000011032	'GALNT18	-	11:11270875-11622005	-	-	IGV_LINK	643.894	15.5639	6.1039	25.024	OK		604.298	14.614	5.54736	23.6807	OK		OK	-0.09085
ENSG0000011	-	-	ENSG0000011033	'BIRC2	-	11:102347210-102378670	-	-	IGV_LINK	1054.09	26.3955	6.49246	46.2985	OK		1338.53	35.5753	10.7558	60.3948	OK		OK	0.430584
ENSG0000011	-	-	ENSG0000011034	'UBE4A	-	11:118359584-118431496	-	-	IGV_LINK	1033.72	15.4629	0	34.7287	OK		788.887	11.8279	0	26.4614	OK		OK	-0.38661
ENSG0000011	-	-	ENSG0000011036	'DDX6	-	11:118704606-118791149	-	-	IGV_LINK	1985.02	21.0185	7.59095	34.4461	OK		1923.23	19.6611	6.68089	32.6413	OK		OK	-0.09632
ENSG0000011	-	-	ENSG0000011037	'UPK2	-	11:118883765-118958559	-	-	IGV_LINK	4.8486	0.367511	0	4.05886	OK		3.09367	0.234851	0	3.5157	OK		NOTEST	-0.64604
ENSG0000011	-	-	ENSG0000011039	'CBL	-	11:119206275-119321521	-	-	IGV_LINK	1052.15	5.36187	0	12.4308	OK		857.979	4.37282	0	10.5506	OK		OK	-0.29417
ENSG0000011	-	-	ENSG0000011040	'NECTIN1	-	11:119608422-119729084	-	-	IGV_LINK	200.732	2.4296	0	5.18776	OK		196.964	2.62555	0	6.08925	OK		OK	0.111899
ENSG0000011	-	-	ENSG0000011042	'HIPK3	-	11:33256671-33357023	-	-	IGV_LINK	1127.78	12.8239	2.89174	22.756	OK		1039.47	11.0452	4.11484	17.9756	OK		OK	-0.21541
ENSG0000011	-	-	ENSG0000011042	'KIAA1549L	-	11:33542071-33696701	-	-	IGV_LINK	4.8486	0.042409	0	0.135952	OK		5.15612	0.025371	0	0.095031	OK		NOTEST	-0.74118
ENSG0000011	-	-	ENSG0000011042	'FBXO3	-	11:33740938-33774543	-	-	IGV_LINK	885.355	24.0328	5.52027	42.5454	OK		908.509	26.9159	5.73913	48.0927	OK		OK	0.163452
ENSG0000011	-	-	ENSG0000011043	'PDHX	-	11:34853093-35020591	-	-	IGV_LINK	315.159	9.58771	0	19.2217	OK		379.491	15.5644	0	32.2777	OK		OK	0.698993
ENSG0000011	-	-	ENSG0000011043	'SLC1A2	-	11:35251205-35421002	-	-	IGV_LINK	18.4247	0.116902	0	0.622145	OK		4.1249	0.033318	0	0.410599	OK		NOTEST	-1.81092
ENSG0000011	-	-	ENSG0000011044	'COMMD9	-	11:36269283-36289449	-	-	IGV_LINK	781.595	61.4011	9.01276	113.79	OK		814.668	63.5637	11.8956	115.232	OK		OK	0.049938
ENSG0000011	-	-	ENSG0000011044	'SLC15A3	-	11:60913165-60952530	-	-	IGV_LINK	1628.16	90.8228	0	204.369	OK		1478.78	84.5335	0	189.048	OK		OK	-0.10353
ENSG0000011	-	-	ENSG0000011044	'CD5	-	11:61102394-61127852	-	-	IGV_LINK	330.675	7.30893	0.537707	14.0802	OK		216.557	4.62377	0.122153	9.12539	OK		OK	-0.66059
ENSG0000011	-	-	ENSG0000011045	'ACCS	-	11:44065924-44084222	-	-	IGV_LINK	1212.15	39.7938	8.71022	70.8774	OK		1314.81	46.9949	10.34	83.6498	OK		OK	0.239961
ENSG0000011	-	-	ENSG0000011048	'SCGB2A2	-	11:62270154-62273156	-	-	IGV_LINK	0	0	0	0	OK		0	0	0	0	OK		NOTEST	0
ENSG0000011	-	-	ENSG0000011049	'MDK	-	11:46380755-46383837	-	-	IGV_LINK	576.014	46.9525	17.4942	76.4108	OK		516.644	42.3742	16.17	68.5783	OK		OK	-0.14802
ENSG0000011	-	-	ENSG0000011049	'AMBRA1	-	11:46396413-46594125	-	-	IGV_LINK	617.712	7.74391	1.82753	13.6603	OK		753.825	9.95048	1.21175	18.6892	OK		OK	0.361705
ENSG0000011	-	-	ENSG0000011051	'MADD	-	11:47269160-47330031	-	-	IGV_LINK	1561.25	27.1934	2.55606	51.8307	OK		1412.78	25.8764	1.60464	50.1482	OK		OK	-0.07162
ENSG0000011	-	-	ENSG0000011053	'PTPMT1	-	11:47465932-47584562	-	-	IGV_LINK	263.856	21.1459	0	71.1033	OK		288.703	23.7858	0	77.0096	OK		OK	0.169722
ENSG0000011	-	-	ENSG0000011058	'NAA40	-	11:63938958-63957328	-	-	IGV_LINK	766.079	14.4465	1.16731	27.7257	OK		702.264	14.2868	0.360424	28.2132	OK		OK	-0.01604
ENSG0000011	-	-	ENSG0000011061	'CARS	-	11:3000921-3057613	-	-	IGV_LINK	1054.09	26.9859	8.80812	45.1636	OK		1183.85	29.4297	4.05886	54.8005	OK		OK	0.125067
ENSG0000011	-	-	ENSG0000011062	'SLC22A18	-	11:2887779-2925246	-	-	IGV_LINK	588.62	28.8878	6.77987	50.9958	OK		1673.68	73.3259	22.297	124.355	OK		OK	1.34386
ENSG0000011	-	-	ENSG0000011065	'CD81	-	11:2328748-2397419	-	-	IGV_LINK	13301.7	688.422	159.864	1216.98	OK		14065.9	753.492	163.737	1343.25	OK		OK	0.1303
ENSG0000011	-	-	ENSG0000011066	'SLC35F2	-	11:107790990-107928293	-	-	IGV_LINK	246.309	5.62609	1.09788	10.1543	OK		98.9976	2.46959	0	5.12853	OK		OK	-1.18786
ENSG0000011	-	-	ENSG0000011066	'C11orf21	-	11:2295644-2318200	-	-	IGV_LINK	473.224	12.3929	0.668914	24.1168	OK		275.337	7.07003	0.448709	13.6914	OK		OK	-0.80972
ENSG0000011	-	-	ENSG0000011067	'ELMOD1	-	11:107591090-107666779	-	-	IGV_LINK	27.1522	0.795518	0	1.72358	OK		19.5933	0.541242	0	1.34572	OK		OK	-0.55562
ENSG0000011	-	-	ENSG0000011068	'CALCA	-	11:14904996-15082342	-	-	IGV_LINK	0	0	0	0	OK		1.03122	0.034939	0	0.386933	OK		NOTEST	inf
ENSG0000011	-	-	ENSG0000011069	'SOX6	-	11:15966448-16756881	-	-	IGV_LINK	455.769	4.1149	0	14.8585	OK		378.46	3.28013	0	13.2438	OK		OK	-0.3271
ENSG0000011	-	-	ENSG0000011069	'C11orf58	-	11:15966448-16756881	-	-	IGV_LINK	2017.02	104.773	8.16894	201.378	OK		2091.32	116.383	2.11146	230.655	OK		OK	0.151613
ENSG0000011	-	-	ENSG0000011069	'PITPNM1	-	11:67491767-67506263	-	-	IGV_LINK	850.445	13.5605	2.72783	24.3932	OK		1034.32	18.5927	0	38.1879	OK		OK	0.455321
ENSG0000011	-	-	ENSG0000011070	'RPS13	-	11:17074388-17207983	-	-	IGV_LINK	7081.87	868.758	280.563	1456.95	OK		5704.74	705.272	222.052	1188.49	OK		OK	-0.30077
ENSG0000011	-	-	ENSG0000011071	'AIP	-	11:67483040-67491103	-	-	IGV_LINK	1156.88	63.4423	26.0811	100.804	OK		1013.69	55.8555	23.2783	88.4327	OK		OK	-0.18375
ENSG0000011	-	-	ENSG0000011071	'NUP98	-	11:3665586-3826371	-	-	IGV_LINK	1521.49	20.0522	0	50.1389	OK		1741.74	24.8589	0	57.2231	OK		OK	0.310006
ENSG0000011	-	-	ENSG0000011071	'NDUFS8	-	11:68030616-68036644	-	-	IGV_LINK	581.832	47.94	17.4588	78.4212	OK		565.111	48.3042	16.8729	79.7355	OK		OK	0.01092
ENSG0000011	-	-	ENSG0000011071	'TCIRG1	-	11:68039015-68121444	-	-	IGV_LINK	2770.19	107.876	25.7289	190.022	OK		2655.01	99.5078	24.2013	174.814	OK		OK	-0.11649
ENSG0000011	-	-	ENSG0000011072	'CHKA	-	11:68039015-68121444	-	-	IGV_LINK	477.102	18.6386	0	55.6628	OK		326.898	13.6023	0	44.3009	OK		OK	-0.45445
ENSG0000011	-	-	ENSG0000011072	'EXPH5	-	11:108505430-108593738	-	-	IGV_LINK	147.398	0.829464	0.062604	1.59632	OK		91.779	0.516536	0.004189	1.02888	OK		OK	-0.68331
ENSG0000011	-	-	ENSG0000011075	'HPS5	-	11:18278667-18322198	-	-	IGV_LINK	501.345	9.81343	1.27055	18.3563	OK		633.172	9.5477	2.54095	16.5545	OK		OK	-0.0396
ENSG0000011	-	-	ENSG0000011076	'GTF2H1	-	11:18322294-18367044	-	-	IGV_LINK	767.049	22.2716	4.89197	39.6513	OK		754.857	21.5676	5.43118	37.7041	OK		OK	-0.04634
ENSG0000011	-	-	ENSG0000011077	'POU2AF1	-	11:111352251-111512354	-	-	IGV_LINK	176.489	4.02855	0	8.50931	OK		211.401	5.21341	0	11.0438	OK		OK	0.371969
ENSG0000011	-	-	ENSG0000011078	'PTPN5	-	11:18693121-18792721	-	-	IGV_LINK	2.90916	0.051211	0	0.478505	OK		9.28102	0.246097	0	1.42864	OK		NOTEST	2.2647
ENSG0000011	-	-	ENSG0000011079	'VWF	-	12:5948873-6124770	-	-	IGV_LINK	6434.1	54.6613	0	130.479	OK		11444.5	86.6288	0	201.153	OK		OK	0.664326
ENSG0000011	-	-	ENSG0000011080	'PSMD9	-	12:121888730-122003927	-	-	IGV_LINK	590.528	38.6378	4.79519	72.4805	OK		711.014	47.8261	7.76987	87.8823	OK		OK	0.307784
ENSG0000011	-	-	ENSG0000011081	'P3H3	-	12:6821544-6839851	-	-	IGV_LINK	54.3043	2.43577	0	7.28141	OK		26.8118	0.772758	0	2.29859	OK		OK	-1.65629
ENSG0000011	-	-	ENSG0000011084	'PPFIBP1	-	12:27523430-27695564	-	-	IGV_LINK	2305.03	48.3189	4.75758	91.8802	OK		1170.44	22.3547	2.42639	42.283	OK		OK	-1.11201
ENSG0000011	-	-	ENSG0000011084	'PRPF40B	-	12:49568217-49764934	-	-	IGV_LINK	639.046	34.7327	0	229.685	OK		444.458	22.7009	0	229.721	OK		OK	-0.61354
ENSG0000011	-	-	ENSG0000011084	'CD69	-	12:9752485-9760901	-	-	IGV_LINK	175.519	7.85686	1.44701	14.2667	OK		131.997	6.01018	0.750218	11.2701	OK		OK	-0.38655
ENSG0000011	-	-	ENSG0000011085	'PRDM4	-	12:107732865-107761272	-	-	IGV_LINK	734.078	10.6962	3.35395	18.0385	OK		1012.66	15.3955	4.30293	26.4881	OK		OK	0.525408
ENSG0000011	-	-	ENSG0000011085	'CLEC2B	-	12:9852983-9870136	-	-	IGV_LINK	413.101	24.6795	7.00547	42.3536	OK		705.358	36.2126	12.1425	60.2827	OK		OK	0.553176
ENSG0000011	-	-	ENSG0000011087	'COQ5	-	12:120503273-120577594	-	-	IGV_LINK	519.77	22.8297	0	56.2914	OK		623.891	26.872	0	63.7651	OK		OK	0.23519
ENSG0000011	-	-	ENSG0000011087	'SELPLG	-	12:108622276-108641318	-	-	IGV_LINK	954.205	27.0728	11.3797	42.766	OK		1041.54	30.1592	12.6788	47.6396	OK		OK	0.155751
ENSG0000011	-	-	ENSG0000011088	'CORO1C	-	12:108645108-108731596	-	-	IGV_LINK	1408.03	28.7198	7.26238	50.1773	OK		2079.98	46.2687	11.5749	80.9624	OK		OK	0.687989
ENSG0000011	-	-	ENSG0000011088	'ASIC1	-	12:50057547-50083611	-	-	IGV_LINK	25.2127	0.503373	0	1.28792	OK		41.249	0.643583	0	1.46223	OK		OK	0.3545
ENSG0000011	-	-	ENSG0000011088	'DAO	-	12:108858931-108901043	-	-	IGV_LINK	5.81832	0.389561	0	1.10472	OK		176.339	7.68519	0.90923	14.4612	OK		OK	4.30216
ENSG0000011	-	-	ENSG0000011088	'CAPRIN2	-	12:30709551-30754951	-	-	IGV_LINK	747.655	15.4023	0.016416	30.7881	OK		511.488	9.57478	0.039583	19.11	OK		OK	-0.68583

ENSG0000011	-	-	ENSG0000011090	'TSPAN11	-	12:30926427-31006010	-	-	IGV_LINK	14.5458	0.258724	0	0.714998	OK	344.429	3.69781	0.823474	6.57214	OK	OK	3.83719
ENSG0000011	-	-	ENSG0000011090	'KCTD10	-	12:109347902-109536705	-	-	IGV_LINK	1070.57	19.0281	0.420275	37.6358	OK	1328.22	23.2651	0.930544	45.5997	OK	OK	0.290039
ENSG0000011	-	-	ENSG0000011091	'SLC11A2	-	12:50979400-51028566	-	-	IGV_LINK	944.508	22.9882	2.35543	43.621	OK	1200.35	27.3904	4.76777	50.0129	OK	OK	0.252773
ENSG0000011	-	-	ENSG0000011091	'MLEC	-	12:120686868-120701864	-	-	IGV_LINK	5584.62	57.2368	0	118.213	OK	8801.51	93.0886	0	203.555	OK	OK	0.701661
ENSG0000011	-	-	ENSG0000011092	'MVK	-	12:109553736-109598117	-	-	IGV_LINK	415.04	15.653	0	33.0043	OK	1069.38	38.4543	6.00356	70.905	OK	OK	1.2967
ENSG0000011	-	-	ENSG0000011092	'CSRNP2	-	12:51061204-51083664	-	-	IGV_LINK	531.407	7.81807	1.50776	14.1284	OK	464.051	6.69571	1.27775	12.1137	OK	OK	-0.22357
ENSG0000011	-	-	ENSG0000011093	'CAMKK2	-	12:121237690-121298308	-	-	IGV_LINK	1081.24	16.5106	6.49292	26.5282	OK	1215.81	18.8279	6.85343	30.8023	OK	OK	0.189479
ENSG0000011	-	-	ENSG0000011093	'BIN2	-	12:51281037-51324668	-	-	IGV_LINK	959.054	26.1431	9.19979	43.0864	OK	809.512	23.1181	6.91667	39.3195	OK	OK	-0.17741
ENSG0000011	-	-	ENSG0000011094	'IL23A	-	12:56334173-56340410	-	-	IGV_LINK	18.4247	1.18875	0	2.54882	OK	20.6245	1.33243	0	2.82966	OK	OK	0.164604
ENSG0000011	-	-	ENSG0000011095	'ATP5B	-	12:56638174-56646068	-	-	IGV_LINK	8627.6	329.912	81.8245	577.999	OK	8536.48	322.074	78.4941	565.654	OK	OK	-0.03469
ENSG0000011	-	-	ENSG0000011095	'PTGES3	-	12:56663340-56688408	-	-	IGV_LINK	1990.84	67.2747	28.4741	106.075	OK	2005.73	67.3296	28.3192	106.34	OK	OK	0.001176
ENSG0000011	-	-	ENSG0000011097	'SYT10	-	12:33374237-33439819	-	-	IGV_LINK	6.78804	0.09731	0	0.347809	OK	1.03122	0.013555	0	0.228903	OK	NOTEST	-2.84376
ENSG0000011	-	-	ENSG0000011098	'BCL7A	-	12:122007433-122062044	-	-	IGV_LINK	371.403	5.83794	1.47009	10.2058	OK	410.428	6.42197	1.61742	11.2265	OK	OK	0.137555
ENSG0000011	-	-	ENSG0000011101	'RSRC2	-	12:122503457-122527000	-	-	IGV_LINK	1470.1	58.272	19.336	97.208	OK	1276.66	54.3284	15.9404	92.7163	OK	OK	-0.1011
ENSG0000011	-	-	ENSG0000011101	'CYP27B1	-	12:57762333-57818704	-	-	IGV_LINK	14.5458	0.591912	0	4.4111	OK	310.399	8.71809	0	25.3959	OK	OK	3.88056
ENSG0000011	-	-	ENSG0000011104	'MYF6	-	12:80707497-80709474	-	-	IGV_LINK	0	0	0	0	OK	0	0	0	0	OK	NOTEST	0
ENSG0000011	-	-	ENSG0000011104	'MYF5	-	12:80716911-80719673	-	-	IGV_LINK	0	0	0	0	OK	0	0	0	0	OK	NOTEST	0
ENSG0000011	-	-	ENSG0000011105	'LIN7A	-	12:80792519-81759553	-	-	IGV_LINK	561.468	9.75612	0	23.929	OK	56.7174	0.734071	0	3.44143	OK	OK	-3.73232
ENSG0000011	-	-	ENSG0000011105	'KRT18	-	12:52897186-52952901	-	-	IGV_LINK	2711.34	118.371	0	248.116	OK	2404.82	106.677	1.15981	212.194	OK	OK	-0.15007
ENSG0000011	-	-	ENSG0000011105	'ACSS3	-	12:80792519-81759553	-	-	IGV_LINK	656.501	10.8988	0.326098	21.4714	OK	1036.38	16.7083	5.09145	28.3252	OK	OK	0.616404
ENSG0000011	-	-	ENSG0000011107	'TNS2	-	12:53006157-53079420	-	-	IGV_LINK	2833.52	59.2137	0	247.428	OK	3010.15	65.1473	0	241.9	OK	OK	0.137775
ENSG0000011	-	-	ENSG0000011108	'GLI1	-	12:57460134-57520517	-	-	IGV_LINK	60.1227	2.36082	0	23.556	OK	27.8431	1.37742	0	16.8519	OK	OK	-0.77732
ENSG0000011	-	-	ENSG0000011111	'PPM1H	-	12:62643981-62935037	-	-	IGV_LINK	68.8502	0.627798	0	1.50281	OK	232.026	2.2279	0.294658	4.16114	OK	OK	1.82731
ENSG0000011	-	-	ENSG0000011114	'METAP2	-	12:95473519-95515839	-	-	IGV_LINK	909.598	34.7689	8.82404	60.7137	OK	865.198	33.2615	8.65338	57.8697	OK	OK	-0.06394
ENSG0000011	-	-	ENSG0000011114	'LTA4H	-	12:95996520-96043520	-	-	IGV_LINK	2213.87	60.9669	25.475	96.4588	OK	1800.52	51.3695	20.2908	82.4482	OK	OK	-0.24712
ENSG0000011	-	-	ENSG0000011114	'ELK3	-	12:96194381-96269835	-	-	IGV_LINK	668.137	10.5978	2.14133	19.0543	OK	749.701	13.2229	1.87615	24.5697	OK	OK	0.319273
ENSG0000011	-	-	ENSG0000011118	'SLC6A12	-	12:190076-214570	-	-	IGV_LINK	551.771	17.7439	1.03439	34.4535	OK	877.572	23.1898	2.61029	43.7693	OK	OK	0.386164
ENSG0000011	-	-	ENSG0000011118	'WNT5B	-	12:1529890-1647243	-	-	IGV_LINK	158.064	5.1854	0	10.9985	OK	70.1233	2.33395	0	6.08174	OK	OK	-1.15168
ENSG0000011	-	-	ENSG0000011119	'MAGOHB	-	12:10604189-10613623	-	-	IGV_LINK	157.095	11.7125	0.926377	22.4985	OK	139.215	12.4344	0	25.2598	OK	OK	0.086294
ENSG0000011	-	-	ENSG0000011119	'TRPV4	-	12:109783084-109833436	-	-	IGV_LINK	141.579	3.25128	0	7.04693	OK	262.962	5.15279	1.55685	8.74873	OK	OK	0.664344
ENSG0000011	-	-	ENSG0000011120	'ITFG2	-	12:1946053-2941140	-	-	IGV_LINK	606.075	33.9448	0	122.652	OK	592.954	29.3641	0	101.086	OK	OK	-0.20914
ENSG0000011	-	-	ENSG0000011120	'FOXM1	-	12:1946053-2941140	-	-	IGV_LINK	264.734	6.37793	0	26.042	OK	323.805	6.46147	0	21.7175	OK	OK	0.018775
ENSG0000011	-	-	ENSG0000011121	'PRR4	-	12:10824959-11176016	-	-	IGV_LINK	99.6744	10.4915	0	27.9563	OK	66.7852	5.24161	0	14.7881	OK	OK	-1.00114
ENSG0000011	-	-	ENSG0000011121	'PRMT8	-	12:3381348-3593973	-	-	IGV_LINK	0	0	0	0	OK	1.03122	0.056404	0	0.225702	OK	NOTEST	inf
ENSG0000011	-	-	ENSG0000011122	'PARP11	-	12:3791046-3910338	-	-	IGV_LINK	224.005	4.80693	0	10.5043	OK	199.026	3.84554	0	8.35645	OK	OK	-0.32193
ENSG0000011	-	-	ENSG0000011122	'ARPC3	-	12:110372899-110450422	-	-	IGV_LINK	3554.97	244.193	78.5575	409.828	OK	4019.7	272.65	87.2342	458.065	OK	OK	0.159028
ENSG0000011	-	-	ENSG0000011123	'GPN3	-	12:110452483-110490385	-	-	IGV_LINK	244.37	17.6934	0	38.0092	OK	253.681	16.8323	0	34.1355	OK	OK	-0.07198
ENSG0000011	-	-	ENSG0000011123	'VPS29	-	12:110491096-110532086	-	-	IGV_LINK	940.629	90.6927	34.1294	147.256	OK	1088.97	107.492	40.8812	174.103	OK	OK	0.245171
ENSG0000011	-	-	ENSG0000011124	'FGF6	-	12:4428154-4445614	-	-	IGV_LINK	0	0	0	0	OK	0	0	0	0	OK	NOTEST	0
ENSG0000011	-	-	ENSG0000011124	'MYL2	-	12:110910818-110920722	-	-	IGV_LINK	2.90916	0.25841	0	0.917017	OK	0	0	0	0	OK	NOTEST	'-inf
ENSG0000011	-	-	ENSG0000011124	'RAD51AP1	-	12:4538797-4560048	-	-	IGV_LINK	70.7896	2.82802	0	6.0335	OK	69.0921	3.50273	0	7.50572	OK	OK	0.308687
ENSG0000011	-	-	ENSG0000011124	'CUX2	-	12:111034023-111350554	-	-	IGV_LINK	2.90916	0.024574	0	0.266703	OK	2.06245	0.017425	0	0.258642	OK	NOTEST	-0.496
ENSG0000011	-	-	ENSG0000011125	'SH2B3	-	12:111405947-111451623	-	-	IGV_LINK	1655.31	17.8248	7.83585	27.8138	OK	1918.08	21.1841	8.58752	33.7807	OK	OK	0.249093
ENSG0000011	-	-	ENSG0000011125	'AKAP3	-	12:4562203-4851927	-	-	IGV_LINK	26.1825	0.916389	0	6.59274	OK	14.4371	0.512106	0	4.65025	OK	OK	-0.83952
ENSG0000011	-	-	ENSG0000011126	'MANSC1	-	12:12326055-12350541	-	-	IGV_LINK	241.46	7.40971	0.513921	14.3055	OK	383.616	12.439	1.60934	23.2687	OK	OK	0.747382
ENSG0000011	-	-	ENSG0000011126	'KCNA1	-	12:4909866-5026012	-	-	IGV_LINK	0	0	0	0	OK	0	0	0	0	OK	NOTEST	0
ENSG0000011	-	-	ENSG0000011126	'DUSP16	-	12:12474209-12562383	-	-	IGV_LINK	864.021	10.4053	2.71715	18.0934	OK	762.075	9.15124	2.15995	16.1425	OK	OK	-0.18528
ENSG0000011	-	-	ENSG0000011126	'CREBL2	-	12:12611826-12645108	-	-	IGV_LINK	2187.69	35.2277	14.2835	56.1718	OK	1926.33	32.0223	11.5531	52.4915	OK	OK	-0.13763
ENSG0000011	-	-	ENSG0000011127	'ACAD10	-	12:111686055-111817529	-	-	IGV_LINK	929.584	19.9321	0	54.0802	OK	799.199	15.3327	0	40.6614	OK	OK	-0.37848
ENSG0000011	-	-	ENSG0000011127	'ALDH2	-	12:111686055-111817529	-	-	IGV_LINK	3409.51	107.553	19.3036	195.803	OK	2221.28	76.99	12.1902	141.79	OK	OK	-0.48231
ENSG0000011	-	-	ENSG0000011127	'CDKN1B	-	12:12715057-12722371	-	-	IGV_LINK	1189.85	27.9484	10.2541	45.6427	OK	1066.29	24.9048	9.18738	40.6221	OK	OK	-0.16635
ENSG0000011	-	-	ENSG0000011129	'GPRC5D	-	12:12927725-13000273	-	-	IGV_LINK	0	0	0	0	OK	1.03122	0.079678	0	1.33689	OK	NOTEST	inf
ENSG0000011	-	-	ENSG0000011130	'NAA25	-	12:112026688-112109022	-	-	IGV_LINK	644.864	6.84187	2.50953	11.1742	OK	516.644	5.5495	1.78253	9.31646	OK	OK	-0.30204
ENSG0000011	-	-	ENSG0000011130	'GSG1	-	12:13044283-13142521	-	-	IGV_LINK	2.90916	0.073934	0	0.598614	OK	0	0	0	0	OK	NOTEST	'-inf
ENSG0000011	-	-	ENSG0000011131	'SCNN1A	-	12:6346842-6391571	-	-	IGV_LINK	63.0318	1.59947	0	8.78915	OK	620.797	20.1274	0	63.4057	OK	OK	3.6535
ENSG0000011	-	-	ENSG0000011132	'LTBR	-	12:6346842-6391571	-	-	IGV_LINK	1761.98	63.242	14.628	111.856	OK	2703.87	97.1979	14.373	180.023	OK	OK	0.620042
ENSG0000011	-	-	ENSG0000011132	'OGFOD2	-	12:122920950-122982913	-	-	IGV_LINK	477.514	19.0337	0	49.4028	OK	609.395	25.4195	0	70.0705	OK	OK	0.417376
ENSG0000011	-	-	ENSG0000011132	'CDK2AP1	-	12:123152319-123272334	-	-	IGV_LINK	480.518	26.0204	0	55.3221	OK	506.308	27.4195	0.34785	54.4911	OK	OK	0.075558
ENSG0000011	-	-	ENSG0000011133	'OAS3	-	12:112906776-113017751	-	-	IGV_LINK	2006.35	18.8305	0	41.366	OK	3085.43	30.3251	0	77.2771	OK	OK	0.687444
ENSG0000011	-	-	ENSG0000011133	'OAS2	-	12:112906776-113017751	-	-	IGV_LINK	1828.89	31.1069	0	83.0683	OK	2508.97	44.1717	0	131.771	OK	OK	0.505888
ENSG0000011	-	-	ENSG0000011133	'ART4	-	12:14803571-14906586	-	-	IGV_LINK	10.6669	0.227643	0	3.20057	OK	7.21857	0.157466	0	1.83268	OK	NOTEST	-0.53173
ENSG0000011	-	-	ENSG0000011134	'MGP	-	12:14803571-14906586	-	-	IGV_LINK	2313.75	173.368	54.199	292.538	OK	827.042	62.0685	15.8454	108.292	OK	OK	-1.48191
ENSG0000011	-	-	ENSG0000011134	'RASAL1	-	12:113098818-113136239	-	-	IGV_LINK	412.131	13.8111	0.882376	26.7399	OK	205.214	6.25082	0.384402	12.1172	OK	OK	-1.14371
ENSG0000011	-	-	ENSG0000011134	'ARHGDIB	-	12:14942016-14961728	-	-	IGV_LINK	4736.11	250.183	97.3741	402.992	OK	3821.72	203	83.3727	322.627	OK	OK	-0.30151
ENSG0000011	-	-	ENSG0000011135	'GTF2H3	-	12:123602076-123662606	-	-	IGV_LINK	280.249	9.42378	0	28.4427	OK	333.086	12.4322	0	37.1865	OK	OK	0.399704
ENSG0000011	-	-	ENSG0000011136	'EIF2B1	-	12:123602076-123662606	-	-	IGV_LINK	993.964	29.6408	4.08751	55.194	OK	1096.19	32.6578	5.37368	59.942	OK	OK	0.139847
ENSG0000011	-	-	ENSG0000011136	'DDX55	-	12:123602076-123662606	-	-	IGV_LINK	511.043	16.0264	0	38.158	OK	531.081	17.5068	0	40.6885	OK	OK	0.127467
ENSG0000011	-	-	ENSG0000011137	'SLC38A1	-	12:46183062-46270017	-	-	IGV_LINK	4082.52	31.47	9.44056	53.4995	OK	4430.14	33.4132	10.5212	56.3052	OK	OK	0.086438
ENSG0000011	-	-	ENSG0000011140	'RERGL	-	12:18080868-18648416	-	-	IGV_LINK	10.6669	0.79799	0	1.89617	OK	6.18735	0.426994	0	1.21469	OK	OK	-0.90215
ENSG0000011	-	-	ENSG0000011140	'ENDOU	-	12:47706084-47837898	-	-	IGV_LINK	0	0	0	0	OK	2.06245	0.123886	0	2.93588	OK	NOTEST	inf
ENSG0000011	-	-	ENSG0000011141	'C12orf49	-	12:116710184-116738070	-	-	IGV_LINK	902.81	15.3239	0	34.415	OK	442.396	8.70335	0	19.7991	OK	OK	-0.81614
ENSG0000011	-	-	ENSG0000011142	'VDR	-	12:47841536-47943048	-	-	IGV_LINK	320.008	4.86093	0	9.87724	OK	346.492	4.72607	1.02352	8.42862	OK	OK	-0.04059
ENSG0000011	-	-	ENSG0000011143	'FZD10	-	12:130162458-130165740	-	-	IGV_LINK	1.93944	0.035156	0	0.107291	OK	13.4059	0.243095	0	0.603556	OK	NOTEST	2.78969
ENSG0000011	-	-	ENSG0000011144	'RFC5	-	12:118013587-118062430	-	-	IGV_LINK	318.068	11.7136	0	27.3528	OK	254.713	8.95105	0	22.9288	OK	OK	-0.38805
ENSG0000011	-	-	ENSG0000011145	'STX2	-	12:130789599-130839266	-	-	IGV_LINK	649.713	13.2797	3.21035	23.349	OK	471.27	9.79621	1.5873	18.0051	OK	OK	-0.43892
ENSG0000011	-	-	ENSG0000011145	'ADGRD1	-	12:130953906-131141469	-	-	IGV_LINK	32.9705	0.614383	0	1.83381	OK	38.1553	0.986389	0	2.80999	OK	OK	0.683019
ENSG0000011	-	-	ENSG0000011148	'COPZ1	-	12:54230939-54351849	-	-	IGV_LINK	2609.52	119.337	0	415.758	OK	3304.04	153.481	0	459.361	OK	OK	0.363021
ENSG0000011	-	-	ENSG0000011149	'TBC1D30	-	12:64781192-64881032	-	-	IGV_LINK	96.9721	1.91021	0	5.17858	OK	93.8415	2.35255	0	6.37765	OK	OK	0.300496
ENSG0000011	-	-	ENSG0000011153	'CAND1	-	12:67269280-67319951	-	-	IGV_LINK	1467.19	22.5717	0.010173	45.1332	OK	1375.65	22.7067	0	45.4357	OK	OK	0.008606
ENSG0000011	-	-	ENSG0000011153	'IL26	-	12:67989444-68234686	-	-	IGV_LINK	0.969721	0.060555	0	0.604942	OK	4.1249	0.25791	0	0.844449	OK	NOTEST	2.09056
ENSG0000011	-	-	ENSG0000011153	'IFNG	-	12:67989444-68234686	-	-	IGV_LINK	69.8199	3.76358	0	7.68935	OK	2.06245	0.111297	0	0.469251	OK	OK	-5.07962
ENSG0000011	-	-	ENSG0000011154	'RAB5B	-	12:55973912-55996683	-	-	IGV_LINK	2864.55	52.6648	18.7857	86.5439	OK	2827.62	53.2375	18.0269	88.4481	OK	OK	0.015605
ENSG0000011	-	-	ENSG0000011155	'MDM1	-	12:68272442-68332381	-	-	IGV_LINK	390.797	12.1827	1.67963	22.6857	OK	365.054	13.1233	1.58445	24.6621	OK	OK	0.107299
ENSG0000011	-	-	ENSG0000011158	'NUP107	-	12:68674370-68745809	-	-	IGV_LINK	774.807	22.9188	3.58729	42.2504	OK	727.014	20.1285	4.19326	36.0637	OK	OK	-0.1873
ENSG0000011	-	-	ENSG0000011159	'CNOT2	-	12:69738680-70354993	-	-	IGV_LINK	1052.15	31.2835	0	72.2545	OK	1000.29	30.2366	4.36817	56.105	OK	OK	-0.04911
ENSG0000011	-	-	ENSG0000011160	'TIMELESS	-	12:56416372-56449403	-	-	IGV_LINK	384.979	5.50264	1.24933	9.75595	OK	468.176	6.63153	1.98556	11.2775	OK	OK	0.269219
ENSG0000011	-	-	ENSG0000011160	'CPSF6	-	12:69239536-69274358	-	-	IGV_LINK	899.901	16.669	3.18668	30.1512	OK	655.859	14.6725	0	31.8237	OK	OK	-0.18405
ENSG0000011	-	-	ENSG0000011161	'KRR1	-	12:75480679-75511636	-	-	IGV_LINK	675.895	18.7309	0	45.7722	OK	667.203	20.0869	0	48.8457	OK	OK	0.100837
ENSG0000011	-	-	ENSG0000011163	'MRPL51	-	12:6491885-6531955	-	-	IGV_LINK	1184.03	119.273	8.47589	230.07	OK	1542.71	140.888	0	283.837	OK	OK	0.240282
ENSG0000011	-	-	ENSG0000011164	'GAPDH	-	12:6533926-6538374	-	-	IGV_LINK	36503.2	1807.34	0	3771.95	OK	57949.7	2867.73	0	7075.5	OK	OK	0.666045
ENSG0000011	-	-	ENSG0000011164	'NOP2	-	12:6556862-6568691	-	-	IGV_LINK	586.681	17.3221	3.60437	31.0399	OK	718.764	22.3641	5.14162	39.5866	OK	OK	0.36857
ENSG0000011	-	-	ENSG0000011164	'CHD4	-	12:6570082-6607476	-	-	IGV_LINK	4234.77	51.4007	0	147.293	OK	4571.42	60.791	0	187.54	OK	OK	0.24207
ENSG0000011	-	-	ENSG0000011164	'ACRBP	-	12:6638074-6647460	-	-	IGV_LINK	119.276	6.06124	0.257299	11.8652	OK	130.966	6.07541	0.36102	11.7898	OK	OK	0.003369
ENSG0000011	-	-	ENSG0000011164	'UHRF1BP1L	-	12:100028454-100142848	-	-	IGV_LINK	469.345	6.65142	0.533063	12.7698	OK	485.707	7.77453	0	15.8579	OK	OK	0.225092
ENSG0000011	-	-	ENSG0000011165	'COPS7A	-	12:6723740-6731875	-	-	IGV_LINK	1053.12	38.0561	14.2932	61.819	OK	1072.47	40.4567	14.8174	66.096	OK	OK	0.088252
ENSG0000011	-	-	ENSG0000011165	'ING4	-	12:6650279-6663148	-	-	IGV_LINK	892.143	52.5144	18.7708	86.258	OK	863.135	53.5815	19.6027	87.5604	OK	OK	0.029023
ENSG0000011	-	-	ENSG0000011166	'GNB3	-	12:6839953-6852066	-	-	IGV_LINK	32.0008	1.10025	0	3.32112	OK	7.21857	0.509376	0	2.36123	OK	OK	-1.11103
ENSG0000011	-	-	ENSG0000011166	'CDCA3	-	12:6839953-6852066	-	-	IGV_LINK	107.639	4.77329	0	10.2907	OK	128.903	5.64831	0	11.7121	OK	OK	0.242835
ENSG0000011	-	-	ENSG0000011166	'CHPT1	-	12:101696946-101744140	-	-	IGV_LINK	383.04	14.3188	3.11198	25.5255	OK	461.989	18.0251	5.56754	30.4826	OK	OK	0.332098
ENSG0000011	-	-	ENSG0000011166	'USP5	-	12:6852127-6866632	-	-	IGV_LINK	1084.15	22.8349	6.26574	39.4041	OK	1391.12	29.4794	8.44662	50.5122	OK	OK	0.368465
ENSG0000011	-	-	ENSG0000011166	'TPI1	-	12:6867118-6923698	-	-	IGV_LINK	17209.6	926.007	89.0806	1762.93	OK	21604.2	1156.7	133.093	2180.31	OK	OK	0.320921
ENSG0000011	-	-	ENSG0000011167	'GNPTAB	-	12:101745496-101830938	-	-	IGV_LINK	987.175	12.1617	0.846638	23.4768	OK	1140.53	14.1138	0.857829	27.3698	OK	OK	0.21476
ENSG0000011	-	-	ENSG0000011167	'SPSB2	-	12:6867118-6923698	-	-	IGV_LINK	104.73	5.3516	0	66.0168	OK	100.029	4.99474	0	69.5211	OK	OK	-0.09956
ENSG0000011	-	-	ENSG0000011167	'ENO2	-	12:6867118-6923698	-	-	IGV_LINK	3772.21	102.351	0	318.06	OK	2450.19	71.1295	0	291.057	OK	OK	-0.525
ENSG0000011	-	-	ENSG0000011167	'ATN1	-	12:6924462-6942321	-	-	IGV_LINK	2839.34	40.5074	14.7192	66.2956	OK	2370.79	33.0893	12.5275	53.6512	OK	OK	-0.29182
ENSG0000011	-	-	ENSG0000011167	'C12orf57	-	12:6942977-6946003	-	-	IGV_LINK	1193.73	165.074	66.3489	263.8	OK	1115.79	153.749	62.7248	244.773	OK	OK	-0.10254
ENSG0000011	-	-	ENSG0000011167	'PTPN6	-	12:6946467-6961316	-	-	IGV_LINK	1823.07	55.5839	18.3824	92.7854	OK	1633.46	50.1283	18.3377	81.9188	OK	OK	-0.14904
ENSG0000011	-	-	ENSG0000011168	'LPCAT3	-	12:6970892-7071032	-	-	IGV_LINK	1466.22	45.6149	0	157.476	OK	3706.22	109.717	0	558.126	OK	OK	1.26621
ENSG0000011	-	-	ENSG0000011169	'NT5DC3	-	12:103770452-103841197	-	-	IGV_LINK	103.76	0.987605	0	2.40867	OK	292.868	2.94697	0.35459	5.53935	OK	OK	1.57723
ENSG0000011	-	-	ENSG0000011170	'SLCO1B3	-	12:20810701-21239246	-	-	IGV_LINK	0	0	0	0	OK	0	0	0	0	OK	NOTEST	0
ENSG0000011	-	-	ENSG0000011170	'APOBEC1	-	12:7649399-7665903	-	-	IGV_LINK	0	0	0	0	OK	1.03122	0.084522	0	0.366855	OK	NOTEST	inf
ENSG0000011	-	-	ENSG0000011170	'NANOG	-	12:7787793-7799141	-	-	IGV_LINK	1.93944	0.021814	0	0.30411	OK	2.06245	0.150516	0	0.607687	OK	NOTEST	2.7866
ENSG0000011	-	-	ENSG0000011170	'SUDS3	-	12:118376379-118418031	-	-	IGV_LINK	927.053	12.5541	2.3884	22.7197	OK	867.26	13.1162	0.76758	25.4648	OK	OK	0.063194
ENSG0000011	-	-	ENSG0000011171	'GOLT1B	-	12:21501780-21518408	-	-	IGV_LINK	295.765	15.643	0	41.4816	OK	367.116	16.8436	0	38.9976	OK	OK	0.106687
ENSG0000011	-	-	ENSG0000011171	'GYS2	-	12:21526306-21604847	-	-	IGV_LINK	21.3339	0.406247	0	1.64151	OK	7.21857	0.137512	0	0.791893	OK	NOTEST	-1.5628
ENSG0000011	-	-	ENSG0000011171	'LDHB	-	12:21635341-21775581	-	-	IGV_LINK	6046.21	301.441	109.168	493.714	OK	5939.86	297.806	106.716	488.897	OK	OK	-0.0175
ENSG0000011	-	-	ENSG0000011172	'PRKAB1	-	12:119334711-119681630	-	-	IGV_LINK	1657.25	42.7549	10.7771	74.7326	OK	1336.47	38.3539	8.29024	68.4176	OK	OK	-0.15672
ENSG0000011	-	-	ENSG0000011172	'CMAS	-	12:22046173-22437041	-	-	IGV_LINK	459.648	17.8764	4.00046	31.7523	OK	666.171	24.3691	8.81401	39.9243	OK	OK	0.447001
ENSG0000011	-	-	ENSG0000011172	'HCFC2	-	12:104064456-104106524	-	-	IGV_LINK	291.886	6.49018	0.962846	12.0175	OK	287.712	5.74874	0.871027	10.6265	OK	OK	-0.17501
ENSG0000011	-	-	ENSG0000011172	'ST8SIA1	-	12:22046173-22437041	-	-	IGV_LINK	160.004	6.20899	0	16.7565	OK	27.8431	1.02531	0	5.94662	OK	OK	-2.5983
ENSG0000011	-	-	ENSG0000011172	'CLEC4A	-	12:8076938-8221115	-	-	IGV_LINK	215.278	16.1238	0	38.6305	OK	204.183	14.7121	0	36.7686	OK	OK	-0.13219
ENSG0000011	-	-	ENSG0000011173	'C2CD5	-	12:22448582-22544546	-	-	IGV_LINK	928.023	16.5349	1.82258	31.2472	OK	806.418	13.6818	1.20392	26.1597	OK	OK	-0.27326
ENSG0000011	-	-	ENSG0000011173	'AICDA	-	12:8602165-8612871	-	-	IGV_LINK	0.969721	0.02065	0	0.651054	OK	8.2498	0.548673	0	1.68171	OK	NOTEST	4.73175
ENSG0000011	-	-	ENSG0000011173	'RAB35	-	12:120095094-120119000	-	-	IGV_LINK	965.842	20.5009	7.87575	33.1261	OK	1154.97	24.5605	8.97229	40.1488	OK	OK	0.260654
ENSG0000011	-	-	ENSG0000011175	'PHC1	-	12:8681599-8949955	-	-	IGV_LINK	1224.76	26.4044	0	78.7373	OK	753.825	14.3866	0	47.587	OK	OK	-0.87605
ENSG0000011	-	-	ENSG0000011177	'COX6A1	-	12:120438089-120469793	-	-	IGV_LINK	2223.57	313.091	57.5884	568.594	OK	2827.62	399.77	72.9343	726.605	OK	OK	0.352587
ENSG0000011	-	-	ENSG0000011178	'AL021546.1	-	12:120438089-120469793	-	-	IGV_LINK	1.88E-09	2.79E-10	0	0.297012	OK	5.86E-08	8.72E-09	0	0.297917	OK	NOTEST	4.96634
ENSG0000011	-	-	ENSG0000011178	'RFX4	-	12:106357657-106889316	-	-	IGV_LINK	0	0	0	0	OK	0	0	0	0	OK	NOTEST	0
ENSG0000011	-	-	ENSG0000011178	'RIC8B	-	12:106357657-106889316	-	-	IGV_LINK	355.887	7.19913	0	15.1278	OK	332.054	5.81265	1.21381	10.4115	OK	OK	-0.30863
ENSG0000011	-	-	ENSG0000011178	'SRSF9	-	12:120438089-120469793	-	-	IGV_LINK	1569.01	79.5271	0	162.16	OK	1885.08	98.0335	0	208.761	OK	OK	0.301828
ENSG0000011	-	-	ENSG0000011178	'AC009533.1	-	12:9277234-9313241	-	-	IGV_LINK	160.974	3.03862	0.682154	5.39509	OK	139.215	2.62891	0.460923	4.79691	OK	OK	-0.20895
ENSG0000011	-	-	ENSG0000011179	'FGFR1OP2	-	12:26938382-26966650	-	-	IGV_LINK	729.23	25.2511	7.15879	43.3434	OK	549.643	18.335	4.66001	32.01	OK	OK	-0.46175

ENSG0000011	-	-	ENSG0000011179	'KLRB1	-	12:9448294-9658412	-	-	IGV_LINK	115.397	5.09868	0	10.8954	OK	175.308	7.75279	0.10311	15.4025	OK	OK	0.604593
ENSG0000011	-	-	ENSG0000011179	'COL12A1	-	6:75084325-75206051	-	-	IGV_LINK	925.113	7.36245	2.14119	12.5837	OK	214.495	1.90136	0	4.06379	OK	OK	-1.95316
ENSG0000011	-	-	ENSG0000011180	'BTN3A3	-	6:26440471-26453415	-	-	IGV_LINK	2934.37	73.958	8.74468	139.171	OK	1176.63	27.7787	4.89761	50.6599	OK	OK	-1.41273
ENSG0000011	-	-	ENSG0000011180	'TDP2	-	6:24649976-24667033	-	-	IGV_LINK	580.863	20.1592	6.41772	33.9007	OK	607.392	19.1176	6.99887	31.2363	OK	OK	-0.07654
ENSG0000011	-	-	ENSG0000011181	'FRK	-	6:115931148-116060758	-	-	IGV_LINK	490.679	2.11774	0.811328	3.42415	OK	458.895	1.98074	0.700356	3.26112	OK	OK	-0.09649
ENSG0000011	-	-	ENSG0000011181	'DSE	-	6:116254172-116444860	-	-	IGV_LINK	1088.03	16.419	2.67679	30.1613	OK	1225.1	17.6403	4.27606	31.0046	OK	OK	0.103507
ENSG0000011	-	-	ENSG0000011183	'RWDD1	-	6:116571366-116597675	-	-	IGV_LINK	530.437	21.1588	5.748	36.5696	OK	306.274	11.472	2.82625	20.1178	OK	OK	-0.88313
ENSG0000011	-	-	ENSG0000011183	'RSPH4A	-	6:116616478-116632985	-	-	IGV_LINK	28.1219	0.59749	0	1.24235	OK	18.562	0.413413	0	0.885659	OK	OK	-0.53133
ENSG0000011	-	-	ENSG0000011183	'MAK	-	6:10747758-10979320	-	-	IGV_LINK	9.69721	0.15203	0	0.937679	OK	4.1249	0.075885	0	0.621283	OK	NOTEST	-1.00247
ENSG0000011	-	-	ENSG0000011184	'TMEM14C	-	6:10722914-10731129	-	-	IGV_LINK	1565.13	95.1568	40.9395	149.374	OK	964.195	59.7992	24.6615	94.937	OK	OK	-0.67018
ENSG0000011	-	-	ENSG0000011184	'PAK1IP1	-	6:10671417-10709782	-	-	IGV_LINK	175.519	6.0778	1.44185	10.7138	OK	111.372	3.85927	0.175794	7.54275	OK	OK	-0.65522
ENSG0000011	-	-	ENSG0000011184	'GCNT2	-	6:10492222-10629368	-	-	IGV_LINK	384.009	5.61624	0	11.9854	OK	433.114	6.46854	0.996403	11.9407	OK	OK	0.203837
ENSG0000011	-	-	ENSG0000011185	'SMIM8	-	6:87282977-87399749	-	-	IGV_LINK	120.245	6.61787	0.088953	13.1468	OK	59.811	3.2258	0	7.02075	OK	OK	-1.03671
ENSG0000011	-	-	ENSG0000011185	'NEDD9	-	6:11173451-11382348	-	-	IGV_LINK	1185.97	18.3055	3.85107	32.76	OK	1036.38	15.7991	3.6627	27.9356	OK	OK	-0.21243
ENSG0000011	-	-	ENSG0000011186	'CEP85L	-	6:118460771-118710075	-	-	IGV_LINK	275.401	3.58809	0	8.8103	OK	139.215	1.25615	0	2.60651	OK	OK	-1.5142
ENSG0000011	-	-	ENSG0000011186	'ADTRP	-	6:11712053-11807046	-	-	IGV_LINK	58.1832	2.92075	0	6.03046	OK	51.5612	2.52255	0	5.48439	OK	OK	-0.21146
ENSG0000011	-	-	ENSG0000011187	'ASF1A	-	6:118813441-119149387	-	-	IGV_LINK	121.215	4.14058	0	11.6301	OK	98.9976	2.62581	0	6.47209	OK	OK	-0.65707
ENSG0000011	-	-	ENSG0000011187	'MCM9	-	6:118813441-119149387	-	-	IGV_LINK	352.978	7.9751	0	19.0049	OK	218.62	4.63707	0	11.3896	OK	OK	-0.78229
ENSG0000011	-	-	ENSG0000011187	'FAM184A	-	6:118813441-119149387	-	-	IGV_LINK	156.125	3.34454	0	8.17974	OK	31.968	0.566293	0	1.95421	OK	OK	-2.56219
ENSG0000011	-	-	ENSG0000011188	'RNGTT	-	6:88610271-88963721	-	-	IGV_LINK	301.583	4.39569	1.15995	7.63143	OK	161.902	2.54414	0.358656	4.72963	OK	OK	-0.78891
ENSG0000011	-	-	ENSG0000011188	'MAN1A1	-	6:119177208-119349761	-	-	IGV_LINK	771.898	8.98498	3.76138	14.2086	OK	1518.99	17.6855	7.79736	27.5736	OK	OK	0.976978
ENSG0000011	-	-	ENSG0000011188	'GABRR2	-	6:89257207-89315299	-	-	IGV_LINK	40.7283	1.51156	0	3.08465	OK	32.9992	1.22563	0	2.52777	OK	OK	-0.30251
ENSG0000011	-	-	ENSG0000011189	'SERINC1	-	6:122443353-122471822	-	-	IGV_LINK	3770.27	71.6738	30.0021	113.345	OK	2946.21	56.0299	24.2643	87.7955	OK	OK	-0.35525
ENSG0000011	-	-	ENSG0000011190	'HDDC2	-	6:125119048-125302078	-	-	IGV_LINK	745.715	59.7691	17.7657	101.772	OK	480.551	37.8114	9.62743	65.9954	OK	OK	-0.66058
ENSG0000011	-	-	ENSG0000011190	'TPD52L1	-	6:125119048-125302078	-	-	IGV_LINK	272.491	20.1396	0	44.4399	OK	130.966	8.78709	0	21.0441	OK	OK	-1.19658
ENSG0000011	-	-	ENSG0000011191	'HINT3	-	6:125956780-125980244	-	-	IGV_LINK	355.887	6.38432	2.2246	10.544	OK	237.182	4.2564	1.24521	7.26758	OK	OK	-0.5849
ENSG0000011	-	-	ENSG0000011191	'NCOA7	-	6:125781160-125932030	-	-	IGV_LINK	684.623	13.4479	1.40896	25.4868	OK	420.74	10.1787	0.391898	19.9655	OK	OK	-0.40183
ENSG0000011	-	-	ENSG0000011191	'RIPOR2	-	6:24797320-25057073	-	-	IGV_LINK	458.798	6.24847	1.43674	11.0602	OK	386.686	5.55207	1.06703	10.0371	OK	OK	-0.17048
ENSG0000011	-	-	ENSG0000011196	'SASH1	-	6:148237584-148552050	-	-	IGV_LINK	901.84	7.59297	0.673678	14.5123	OK	2458.44	19.2871	6.46363	32.1106	OK	OK	1.3449
ENSG0000011	-	-	ENSG0000011196	'UST	-	6:148747327-149076990	-	-	IGV_LINK	222.066	4.45212	0	10.2492	OK	123.747	2.90094	0	7.54589	OK	OK	-0.61797
ENSG0000011	-	-	ENSG0000011198	'ULBP1	-	6:149964006-149973710	-	-	IGV_LINK	0.969721	0.018404	0	0.072082	OK	0	0	0	0	OK	NOTEST	'-inf
ENSG0000011	-	-	ENSG0000011202	'FBXO5	-	6:152970518-153002685	-	-	IGV_LINK	125.094	3.50672	0	8.39928	OK	73.217	2.08025	0	5.00828	OK	OK	-0.75337
ENSG0000011	-	-	ENSG0000011203	'MTRF1L	-	6:152970518-153002685	-	-	IGV_LINK	359.766	8.20441	0	18.3686	OK	259.869	5.09251	0.085784	10.0992	OK	OK	-0.68802
ENSG0000011	-	-	ENSG0000011203	'PPARD	-	6:35342557-35428191	-	-	IGV_LINK	1275.18	20.2365	8.89101	31.5819	OK	925.009	14.8099	6.29348	23.3263	OK	OK	-0.4504
ENSG0000011	-	-	ENSG0000011203	'OPRM1	-	6:154010495-154356792	-	-	IGV_LINK	0	0	0	0	OK	0	0	0	0	OK	NOTEST	0
ENSG0000011	-	-	ENSG0000011203	'FANCE	-	6:35452360-35467103	-	-	IGV_LINK	66.9107	1.58338	0.012848	3.15391	OK	34.0304	0.805688	0	1.64608	OK	OK	-0.97472
ENSG0000011	-	-	ENSG0000011204	'TULP1	-	6:35497873-35512938	-	-	IGV_LINK	1.93944	0.331513	0	1.11418	OK	0	0	0	0	OK	NOTEST	'-inf
ENSG0000011	-	-	ENSG0000011205	'SLC26A8	-	6:35943513-36024868	-	-	IGV_LINK	9.69721	0.206594	0	0.618687	OK	17.5308	0.335387	0	0.87993	OK	NOTEST	0.699033
ENSG0000011	-	-	ENSG0000011206	'MAPK14	-	6:36027676-36111236	-	-	IGV_LINK	1124.88	19.6476	6.4255	32.8697	OK	1173.53	20.5417	7.18187	33.9016	OK	OK	0.064206
ENSG0000011	-	-	ENSG0000011207	'RHAG	-	6:49605157-49636839	-	-	IGV_LINK	0	0	0	0	OK	0	0	0	0	OK	NOTEST	0
ENSG0000011	-	-	ENSG0000011207	'KCTD20	-	6:36354090-36491143	-	-	IGV_LINK	1281	14.795	0.400555	29.1895	OK	1087.94	11.736	3.77607	19.6959	OK	OK	-0.33417
ENSG0000011	-	-	ENSG0000011207	'STK38	-	6:36493891-36547470	-	-	IGV_LINK	980.387	16.2334	7.01161	25.4553	OK	838.386	13.8868	5.80925	21.9644	OK	OK	-0.22525
ENSG0000011	-	-	ENSG0000011208	'SRSF3	-	6:36594352-36605600	-	-	IGV_LINK	2411.69	142.904	32.8821	252.926	OK	1692.24	104.078	24.0952	184.062	OK	OK	-0.45738
ENSG0000011	-	-	ENSG0000011209	'SOD2	-	6:159669056-159789749	-	-	IGV_LINK	26610.1	717.89	0	1781.75	OK	56713.2	1584.74	0	4394.35	OK	OK	1.14241
ENSG0000011	-	-	ENSG0000011211	'MRPL18	-	6:159789811-159798436	-	-	IGV_LINK	597.348	42.5259	16.7648	68.2871	OK	544.487	38.3756	14.9633	61.788	OK	OK	-0.14815
ENSG0000011	-	-	ENSG0000011211	'IL17A	-	6:52186386-52190638	-	-	IGV_LINK	0	0	0	0	OK	1.03122	0.034483	0	0.129063	OK	NOTEST	inf
ENSG0000011	-	-	ENSG0000011211	'IL17F	-	6:52236680-52244537	-	-	IGV_LINK	0	0	0	0	OK	1.03122	0.077248	0	0.32754	OK	NOTEST	inf
ENSG0000011	-	-	ENSG0000011211	'MCM3	-	6:52264008-52284881	-	-	IGV_LINK	1167.54	23.2276	9.03688	37.4183	OK	763.106	15.2299	5.85979	24.6001	OK	OK	-0.60893
ENSG0000011	-	-	ENSG0000011213	'RNF8	-	6:37353971-37394738	-	-	IGV_LINK	252.127	7.55132	2.0305	13.0721	OK	170.152	5.74898	1.01692	10.481	OK	OK	-0.39342
ENSG0000011	-	-	ENSG0000011213	'PHACTR1	-	6:12716804-13328583	-	-	IGV_LINK	331.644	7.37743	0	17.0634	OK	298.024	6.38248	0	14.5127	OK	OK	-0.209
ENSG0000011	-	-	ENSG0000011213	'MDGA1	-	6:37630678-37699306	-	-	IGV_LINK	64.9713	0.595118	0	1.69323	OK	30.9367	0.422794	0	1.61929	OK	OK	-0.49322
ENSG0000011	-	-	ENSG0000011214	'ICK	-	6:53001278-53100873	-	-	IGV_LINK	499.406	4.72262	0	9.72487	OK	296.993	2.80092	0	5.89999	OK	OK	-0.75369
ENSG0000011	-	-	ENSG0000011214	'FBXO9	-	6:53001278-53100873	-	-	IGV_LINK	1310	47.1216	3.58332	90.66	OK	813.07	30.5285	4.52077	56.5362	OK	OK	-0.62623
ENSG0000011	-	-	ENSG0000011214	'CD83	-	6:14117255-14136918	-	-	IGV_LINK	552.741	14.3628	5.62633	23.0993	OK	332.054	8.47239	2.742	14.2028	OK	OK	-0.7615
ENSG0000011	-	-	ENSG0000011215	'MDN1	-	6:89642498-89819723	-	-	IGV_LINK	1138.45	9.2738	0	24.7061	OK	597.079	6.44812	0	17.1025	OK	OK	-0.52428
ENSG0000011	-	-	ENSG0000011216	'GLP1R	-	6:39048797-39087743	-	-	IGV_LINK	0	0	0	0	OK	5.15612	0.099128	0	0.242996	OK	NOTEST	inf
ENSG0000011	-	-	ENSG0000011216	'SAYSD1	-	6:39104063-39115189	-	-	IGV_LINK	172.61	5.68854	0.437033	10.9401	OK	139.215	4.57485	0.409013	8.74068	OK	OK	-0.31434
ENSG0000011	-	-	ENSG0000011217	'BMP5	-	6:55753644-55875564	-	-	IGV_LINK	5.81832	0.086756	0	0.206044	OK	0	0	0	0	OK	NOTEST	'-inf
ENSG0000011	-	-	ENSG0000011218	'BACH2	-	6:89926528-90296908	-	-	IGV_LINK	84.3657	0.53179	0	1.57314	OK	40.2178	0.253613	0	0.884058	OK	NOTEST	-1.06823
ENSG0000011	-	-	ENSG0000011218	'RBM24	-	6:17281345-17293875	-	-	IGV_LINK	4.8486	0.17587	0	0.608869	OK	21.6557	0.922079	0	2.27488	OK	OK	2.39038
ENSG0000011	-	-	ENSG0000011218	'CAP2	-	6:17393215-17557792	-	-	IGV_LINK	252.127	8.34207	1.26074	15.4234	OK	78.3731	2.37749	0	4.77777	OK	OK	-1.81097
ENSG0000011	-	-	ENSG0000011219	'TREML2	-	6:41190276-41201194	-	-	IGV_LINK	16.4852	0.296865	0	0.661165	OK	22.6869	0.408695	0	0.900403	OK	NOTEST	0.461217
ENSG0000011	-	-	ENSG0000011220	'ZNF451	-	6:57086843-57222314	-	-	IGV_LINK	534.316	9.00985	0	21.625	OK	361.96	7.86932	0	20.6595	OK	OK	-0.19527
ENSG0000011	-	-	ENSG0000011220	'BAG2	-	6:57086843-57222314	-	-	IGV_LINK	49.4557	0.430209	0	1.37771	OK	46.4051	0.403743	0	1.21899	OK	NOTEST	-0.0916
ENSG0000011	-	-	ENSG0000011221	'RAB23	-	6:57086843-57222314	-	-	IGV_LINK	69.8199	1.24566	0	3.67225	OK	65.9984	1.20052	0	3.3376	OK	OK	-0.05325
ENSG0000011	-	-	ENSG0000011221	'TSPO2	-	6:41026910-41099976	-	-	IGV_LINK	4.8486	0.500933	0	5.87955	OK	0	0	0	0	OK	NOTEST	'-inf
ENSG0000011	-	-	ENSG0000011221	'FHL5	-	6:96562547-96616636	-	-	IGV_LINK	20.3641	0.643669	0	1.44204	OK	19.5933	0.681966	0	1.55992	OK	OK	0.083383
ENSG0000011	-	-	ENSG0000011221	'GPR63	-	6:96785136-96837463	-	-	IGV_LINK	6.78804	0.06619	0	0.155559	OK	6.18735	0.060345	0	0.143605	OK	NOTEST	-0.13339
ENSG0000011	-	-	ENSG0000011223	'KHDRBS2	-	6:61630232-62286227	-	-	IGV_LINK	0.969721	0.025307	0	0.09912	OK	10.3122	0.26926	0	0.613162	OK	NOTEST	3.41141
ENSG0000011	-	-	ENSG0000011223	'FBXL4	-	6:98868537-98948006	-	-	IGV_LINK	530.437	7.32582	2.3124	12.3392	OK	298.024	4.35581	0.8007	7.91092	OK	OK	-0.75005
ENSG0000011	-	-	ENSG0000011223	'CCNC	-	6:99542379-99569096	-	-	IGV_LINK	473.224	21.9759	0.97698	42.9749	OK	336.179	16.7983	0	37.6412	OK	OK	-0.38761
ENSG0000011	-	-	ENSG0000011223	'PRDM13	-	6:99606729-99615578	-	-	IGV_LINK	0	0	0	0	OK	0	0	0	0	OK	NOTEST	0
ENSG0000011	-	-	ENSG0000011224	'E2F3	-	6:20401905-20493715	-	-	IGV_LINK	250.188	3.26173	0.891332	5.63213	OK	362.991	4.71312	1.59706	7.82919	OK	OK	0.531046
ENSG0000011	-	-	ENSG0000011224	'PTP4A1	-	6:63521747-63583587	-	-	IGV_LINK	829.111	16.3808	0	37.9737	OK	752.794	14.4955	0	34.9216	OK	OK	-0.1764
ENSG0000011	-	-	ENSG0000011224	'SIM1	-	6:100385014-100464929	-	-	IGV_LINK	1.93944	0.013236	0	0.227061	OK	0	0	0	0	OK	NOTEST	'-inf
ENSG0000011	-	-	ENSG0000011224	'ASCC3	-	6:100508193-100881372	-	-	IGV_LINK	627.409	8.0026	0	17.7516	OK	411.459	4.24825	0	9.45854	OK	OK	-0.9136
ENSG0000011	-	-	ENSG0000011227	'HDGFL1	-	6:22569492-22571666	-	-	IGV_LINK	0.969721	0.027304	0	0.106944	OK	0	0	0	0	OK	NOTEST	'-inf
ENSG0000011	-	-	ENSG0000011227	'BVES	-	6:105096821-105179995	-	-	IGV_LINK	35.8797	0.374846	0	0.844402	OK	39.1865	0.409464	0	0.842167	OK	NOTEST	0.127439
ENSG0000011	-	-	ENSG0000011228	'COL9A1	-	6:70215060-70303083	-	-	IGV_LINK	2.90916	0.112524	0	0.760235	OK	1.03122	0.112555	0	0.756196	OK	NOTEST	0.000402
ENSG0000011	-	-	ENSG0000011228	'MED23	-	6:131573143-131628229	-	-	IGV_LINK	1054.09	16.881	2.68589	31.0762	OK	537.268	7.78221	1.06953	14.4949	OK	OK	-1.11715
ENSG0000011	-	-	ENSG0000011229	'WASF1	-	6:110099818-110180004	-	-	IGV_LINK	152.246	4.75687	0.17791	9.33584	OK	115.497	2.75968	0.147895	5.37147	OK	OK	-0.78551
ENSG0000011	-	-	ENSG0000011229	'GPLD1	-	6:24402907-24537207	-	-	IGV_LINK	82.4262	3.40202	0	12.8383	OK	29.9055	1.8198	0	7.48152	OK	OK	-0.90261
ENSG0000011	-	-	ENSG0000011229	'ALDH5A1	-	6:24402907-24537207	-	-	IGV_LINK	586.681	8.43529	0	19.8598	OK	311.43	3.75693	0.290492	7.22338	OK	OK	-1.16688
ENSG0000011	-	-	ENSG0000011229	'CRYBG1	-	6:106360807-106629487	-	-	IGV_LINK	1803.68	14.8829	4.08466	25.6811	OK	1238.5	9.54116	3.7398	15.3425	OK	OK	-0.64142
ENSG0000011	-	-	ENSG0000011229	'VNN1	-	6:132681589-132714049	-	-	IGV_LINK	650.682	12.5003	5.08843	19.9123	OK	897.166	17.2423	7.27223	27.2124	OK	OK	0.463987
ENSG0000011	-	-	ENSG0000011230	'VNN2	-	6:132743869-132763459	-	-	IGV_LINK	330.675	12.2443	2.44043	22.0482	OK	490.863	16.1562	5.18855	27.1238	OK	OK	0.399975
ENSG0000011	-	-	ENSG0000011230	'ACOT13	-	6:24667034-24720836	-	-	IGV_LINK	361.706	6.93501	0	23.5931	OK	239.244	4.41038	0	15.5165	OK	OK	-0.653
ENSG0000011	-	-	ENSG0000011230	'SMAP1	-	6:70667775-70957038	-	-	IGV_LINK	164.852	4.07691	0	8.2091	OK	142.309	4.22121	0	10.0966	OK	OK	0.050179
ENSG0000011	-	-	ENSG0000011230	'RPS12	-	6:132814440-132817564	-	-	IGV_LINK	13516.9	1659.3	456.174	2862.42	OK	6636.96	817.15	315.931	1318.37	OK	OK	-1.0219
ENSG0000011	-	-	ENSG0000011230	'C6orf62	-	6:24667034-24720836	-	-	IGV_LINK	2735.58	81.464	7.10943	155.818	OK	2183.1	63.2169	6.19199	120.242	OK	OK	-0.36585
ENSG0000011	-	-	ENSG0000011230	'B3GAT2	-	6:70667775-70957038	-	-	IGV_LINK	149.337	1.59597	0.002702	3.18924	OK	110.341	1.13188	0	2.34769	OK	OK	-0.49572
ENSG0000011	-	-	ENSG0000011231	'GMNN	-	6:24774930-24786099	-	-	IGV_LINK	198.793	13.3723	2.14985	24.5948	OK	139.215	8.14236	0.940947	15.3438	OK	OK	-0.71573
ENSG0000011	-	-	ENSG0000011231	'EYA4	-	6:133240597-133895553	-	-	IGV_LINK	36.8494	0.594924	0	1.5642	OK	0	0	0	0	OK	OK	'-inf
ENSG0000011	-	-	ENSG0000011232	'SOBP	-	6:107489957-107660167	-	-	IGV_LINK	31.0311	0.344764	0	0.820367	OK	16.4996	0.257239	0	0.665598	OK	NOTEST	-0.4225
ENSG0000011	-	-	ENSG0000011233	'NR2E1	-	6:108166057-108188809	-	-	IGV_LINK	1.93944	0.044803	0	0.37469	OK	0	0	0	0	OK	NOTEST	'-inf
ENSG0000011	-	-	ENSG0000011233	'SNX3	-	6:108211221-108261260	-	-	IGV_LINK	1943.32	83.4414	30.6852	136.198	OK	1453	60.9473	21.5704	100.324	OK	OK	-0.4532
ENSG0000011	-	-	ENSG0000011233	'SLC17A2	-	6:25912753-25930726	-	-	IGV_LINK	14.5458	0.466529	0	1.04168	OK	1.03122	0.02772	0	0.146852	OK	NOTEST	-4.07296
ENSG0000011	-	-	ENSG0000011233	'HBS1L	-	6:134960377-135103056	-	-	IGV_LINK	542.074	23.5635	4.24936	42.8777	OK	390.834	18.8104	2.93388	34.687	OK	OK	-0.32502
ENSG0000011	-	-	ENSG0000011234	'TRIM38	-	6:25962801-26001775	-	-	IGV_LINK	2461.15	15.0099	5.78271	24.2372	OK	2184.13	13.3221	5.54404	21.1002	OK	OK	-0.17209
ENSG0000011	-	-	ENSG0000011235	'PEX7	-	6:136822563-136913937	-	-	IGV_LINK	128.003	5.58563	0.983683	10.1876	OK	102.091	5.2914	0.195241	10.3876	OK	OK	-0.07807
ENSG0000011	-	-	ENSG0000011236	'ZBTB24	-	6:109444061-109483237	-	-	IGV_LINK	300.661	3.16916	0	8.93038	OK	198.114	2.0887	0	6.29621	OK	OK	-0.6015
ENSG0000011	-	-	ENSG0000011236	'FIG4	-	6:109691311-109825428	-	-	IGV_LINK	497.467	10.4008	3.4429	17.3588	OK	469.207	9.57144	3.13367	16.0092	OK	OK	-0.11989
ENSG0000011	-	-	ENSG0000011237	'PERP	-	6:138088504-138107511	-	-	IGV_LINK	1266.45	17.3338	7.5544	27.1132	OK	1120.94	15.3465	6.67573	24.0172	OK	OK	-0.17568
ENSG0000011	-	-	ENSG0000011237	'ARFGEF3	-	6:138161920-138344663	-	-	IGV_LINK	184.247	0.706132	0.166156	1.24611	OK	242.338	0.92884	0.267231	1.59045	OK	OK	0.395492
ENSG0000011	-	-	ENSG0000011239	'SLC16A10	-	6:111087502-111259034	-	-	IGV_LINK	51.3952	1.1143	0	3.00624	OK	160.871	3.16194	0	6.85367	OK	OK	1.50467
ENSG0000011	-	-	ENSG0000011240	'HECA	-	6:139135111-139292139	-	-	IGV_LINK	589.59	6.10617	1.95922	10.2531	OK	493.957	5.11681	1.58163	8.65198	OK	OK	-0.25502
ENSG0000011	-	-	ENSG0000011241	'ADGRG6	-	6:142301853-142446266	-	-	IGV_LINK	1155.91	11.3833	0.431247	22.3354	OK	269.15	2.57107	0	5.35656	OK	OK	-2.14648
ENSG0000011	-	-	ENSG0000011241	'PHACTR2	-	6:143536844-143938471	-	-	IGV_LINK	427.647	6.25826	0	17.761	OK	306.274	4.99016	0	14.5383	OK	OK	-0.32668
ENSG0000011	-	-	ENSG0000011242	'EPM2A	-	6:145382534-145737218	-	-	IGV_LINK	153.216	3.61031	0	8.29812	OK	76.3106	2.08645	0	5.08824	OK	OK	-0.79107
ENSG0000011	-	-	ENSG0000011246	'OR12D3	-	6:29353748-29457071	-	-	IGV_LINK	0	0	0	0	OK	0	0	0	0	OK	NOTEST	0
ENSG0000011	-	-	ENSG0000011247	'SLC39A7	-	6:33193587-33204439	-	-	IGV_LINK	1662.1	48.5406	8.51653	88.5646	OK	1429.28	40.8254	10.0248	71.626	OK	OK	-0.24972
ENSG0000011	-	-	ENSG0000011248	'CCR6	-	6:166999181-167139696	-	-	IGV_LINK	114.824	1.88696	0	6.98524	OK	148.168	2.80592	0	6.70164	OK	OK	0.572414
ENSG0000011	-	-	ENSG0000011249	'UNC93A	-	6:167271168-167316019	-	-	IGV_LINK	0	0	0	0	OK	41.249	1.49104	0	3.31347	OK	OK	inf
ENSG0000011	-	-	ENSG0000011249	'SLC22A2	-	6:160171060-160277638	-	-	IGV_LINK	2081.99	51.6958	20.2323	83.1594	OK	2188.26	54.9594	22.5767	87.342	OK	OK	0.088317
ENSG0000011	-	-	ENSG0000011251	'PHF1	-	6:33410398-33418317	-	-	IGV_LINK	1696.04	66.8267	4.32401	129.329	OK	716.701	29.5357	1.33183	57.7395	OK	OK	-1.17797
ENSG0000011	-	-	ENSG0000011251	'CUTA	-	6:33410398-33418317	-	-	IGV_LINK	1471.07	138.205	18.1744	258.236	OK	729.076	70.1768	13.4636	126.89	OK	OK	-0.97774
ENSG0000011	-	-	ENSG0000011253	'PACRG	-	6:161347419-163324530	-	-	IGV_LINK	12.6064	1.27974	0	7.98043	OK	9.28102	0.818718	0	4.11829	OK	OK	-0.64441
ENSG0000011	-	-	ENSG0000011253	'QKI	-	6:163413999-163578596	-	-	IGV_LINK	1750.35	49.9737	6.95527	92.9921	OK	1478.78	42.3975	6.8221	77.9729	OK	OK	-0.23719
ENSG0000011	-	-	ENSG0000011253	'C6orf118	-	6:165279663-165309607	-	-	IGV_LINK	0	0	0	0	OK	5.15612	0.177361	0	0.538829	OK	NOTEST	inf
ENSG0000011	-	-	ENSG0000011254	'PDE10A	-	6:165327286-165989615	-	-	IGV_LINK	163.883	1.1666	0.225944	2.10725	OK	1031.22	7.82579	3.03777	12.6138	OK	OK	2.74593
ENSG0000011	-	-	ENSG0000011255	'MDFI	-	6:41636881-41654246	-	-	IGV_LINK	27.1522	1.22698	0	2.89953	OK	10.3122	0.48309	0	1.54264	OK	OK	-1.34474
ENSG0000011	-	-	ENSG0000011256	'TFEB	-	6:41683977-41736259	-	-	IGV_LINK	706.926	25.0826	4.84383	45.3215	OK	425.896	15.4571	1.74353	29.1707	OK	OK	-0.69842
ENSG0000011	-	-	ENSG0000011256	'SMOC2	-	6:168441150-168673445	-	-	IGV_LINK	134.791	2.87786	0.26274	5.49297	OK	269.15	5.54226	1.50222	9.5823	OK	OK	0.94548
ENSG0000011	-	-	ENSG0000011257	'CCND3	-	6:41934932-42050357	-	-	IGV_LINK	1754.22	66.0941	21.1654	111.023	OK	728.045	27.3856	9.31018	45.4609	OK	OK	-1.27111
ENSG0000011	-	-	ENSG0000011257	'BYSL	-	6:41921187-41933046	-	-	IGV_LINK	159.034	5.64063	1.07857	10.2027	OK	164.996	5.61558	0.768667	10.4625	OK	OK	-0.00642
ENSG0000011	-	-	ENSG0000011258	'FAM120B	-	6:170282205-170407065	-	-	IGV_LINK	714.684	12.0998	1.8584	22.3411	OK	551.705	10.2679	1.9565	18.5794	OK	OK	-0.23683
ENSG0000011	-	-	ENSG0000011259	'TBP	-	6:170554301-170572870	-	-	IGV_LINK	295.765	11.112	3.15354	19.0705	OK	177.371	7.1502	1.33199	12.9684	OK	OK	-0.63607
ENSG0000011	-	-	ENSG0000011259	'GUCA1B	-	6:42184400-42194916	-	-	IGV_LINK	14.5458	0.87162	0	1.94176	OK	9.28102	0.556822	0	1.25649	OK	OK	-0.64648
ENSG0000011	-	-	ENSG0000011261	'PRPH2	-	6:42696599-42722574	-	-	IGV_LINK	6.78804	0.136453	0	0.320691	OK	10.3122	0.207382	0	0.46599	OK	NOTEST	0.603883
ENSG0000011	-	-	ENSG0000011262	'BICRAL	-	6:42746957-42868560	-	-	IGV_LINK	663.289	5.88178	2.29682	9.46674	OK	358.866	3.18175	1.11167	5.25184	OK	OK	-0.88643
ENSG0000011	-	-	ENSG0000011264	'PPP2R5D	-	6:42984498-43013968	-	-	IGV_LINK	832.99	20.9165	0.324204	41.5088	OK	483.645	11.1971	0	22.7388	OK	OK	-0.90151
ENSG0000011	-	-	ENSG0000011265	'MRPL2	-	6:43037616-43075099	-	-	IGV_LINK	428.616	36.4272	0	100.429	OK	282.556	24.6461	0	69.5804	OK	OK	-0.56366

ENSG0000011	-	-	ENSG0000011265	'PTK7	-	6:43076267-43161719	-	-	IGV_LINK	379.161	7.35223	0.81777	13.8867	OK	85.5917	1.38416	0	2.9549	OK	OK	-2.40917
ENSG0000011	-	-	ENSG0000011265	'SRF	-	6:43171298-43181507	-	-	IGV_LINK	613.833	8.58494	3.40997	13.7599	OK	428.99	6.00147	2.21202	9.79092	OK	OK	-0.51649
ENSG0000011	-	-	ENSG0000011265	'CUL9	-	6:43182174-43224587	-	-	IGV_LINK	1060.87	23.8787	0	48.6726	OK	791.981	20.1527	0	41.8143	OK	OK	-0.24475
ENSG0000011	-	-	ENSG0000011266	'DNPH1	-	6:43225628-43229484	-	-	IGV_LINK	988.145	113.213	48.6792	177.747	OK	550.674	63.2296	24.1241	102.335	OK	OK	-0.84037
ENSG0000011	-	-	ENSG0000011267	'DUSP22	-	6:291629-351355	-	-	IGV_LINK	927.053	24.5929	5.02553	44.1602	OK	694.014	18.9871	3.49414	34.48	OK	OK	-0.37322
ENSG0000011	-	-	ENSG0000011268	'EXOC2	-	6:485132-693111	-	-	IGV_LINK	480.012	6.86063	1.54855	12.1727	OK	352.679	5.08924	0.593248	9.58524	OK	OK	-0.43089
ENSG0000011	-	-	ENSG0000011269	'COX7A2	-	6:75237674-75250323	-	-	IGV_LINK	1124.88	149.925	54.1749	245.675	OK	808.48	108.201	35.0885	181.314	OK	OK	-0.47052
ENSG0000011	-	-	ENSG0000011269	'TMEM30A	-	6:75252923-75284968	-	-	IGV_LINK	1729.01	22.2578	9.75434	34.7612	OK	1449.9	18.5349	8.20542	28.8643	OK	OK	-0.26407
ENSG0000011	-	-	ENSG0000011269	'GMDS	-	6:1623805-2245692	-	-	IGV_LINK	254.067	13.5263	2.06955	24.983	OK	140.247	7.91494	0	16.0276	OK	OK	-0.77312
ENSG0000011	-	-	ENSG0000011270	'SENP6	-	6:75601508-75718278	-	-	IGV_LINK	1418.7	28.0933	8.25908	47.9276	OK	906.447	18.1107	4.74022	31.4812	OK	OK	-0.63339
ENSG0000011	-	-	ENSG0000011270	'IMPG1	-	6:75921114-76072678	-	-	IGV_LINK	10.6669	0.190746	0	0.511924	OK	7.21857	0.172977	0	0.535137	OK	NOTEST	-0.14107
ENSG0000011	-	-	ENSG0000011271	'VEGFA	-	6:43770183-43786487	-	-	IGV_LINK	76777.6	2022.15	0	5879.76	OK	23201.5	617.544	0	1423.36	OK	OK	-1.71128
ENSG0000011	-	-	ENSG0000011273	'PRPF4B	-	6:4021266-4157385	-	-	IGV_LINK	1614.58	25.3832	0	52.0424	OK	915.728	14.1306	0	30.8265	OK	OK	-0.84505
ENSG0000011	-	-	ENSG0000011274	'TTK	-	6:80003886-80042527	-	-	IGV_LINK	47.5163	0.917725	0	2.19998	OK	25.7806	0.48943	0	1.54477	OK	OK	-0.90696
ENSG0000011	-	-	ENSG0000011275	'SLC29A1	-	6:44219504-44234151	-	-	IGV_LINK	1290.7	38.8473	16.0756	61.619	OK	755.888	23.2181	8.55706	37.8792	OK	OK	-0.74256
ENSG0000011	-	-	ENSG0000011276	'WISP3	-	6:112054071-112087529	-	-	IGV_LINK	1.93944	0.070192	0	0.942804	OK	1.03122	0.024853	0	0.478442	OK	NOTEST	-1.49787
ENSG0000011	-	-	ENSG0000011276	'BTN2A1	-	6:26457903-26476621	-	-	IGV_LINK	1814.35	37.8213	14.4399	61.2027	OK	1138.47	23.4667	8.73776	38.1956	OK	OK	-0.68858
ENSG0000011	-	-	ENSG0000011276	'LAMA4	-	6:112108759-112351296	-	-	IGV_LINK	2449.51	31.4806	0	64.8302	OK	3095.74	42.3017	0	87.7364	OK	OK	0.426254
ENSG0000011	-	-	ENSG0000011277	'FAM46A	-	6:81491438-81752774	-	-	IGV_LINK	497.467	5.31777	1.18421	9.45132	OK	353.71	3.69454	1.1713	6.21778	OK	OK	-0.52543
ENSG0000011	-	-	ENSG0000011278	'CLIC5	-	6:45898450-46080395	-	-	IGV_LINK	87.2748	1.49266	0	3.79419	OK	100.029	1.63711	0	4.00041	OK	OK	0.133267
ENSG0000011	-	-	ENSG0000011278	'FBRSL1	-	12:132489550-132585188	-	-	IGV_LINK	717.593	18.2396	0	54.7184	OK	683.702	18.1038	0	52.327	OK	OK	-0.01078
ENSG0000011	-	-	ENSG0000011279	'ENPP5	-	6:46159186-46170971	-	-	IGV_LINK	270.552	5.86674	1.57712	10.1564	OK	191.808	3.98459	0.885356	7.08381	OK	OK	-0.55813
ENSG0000011	-	-	ENSG0000011279	'LY86	-	6:6346464-6654983	-	-	IGV_LINK	384.979	24.3125	7.35733	41.2676	OK	293.899	20.2917	5.0159	35.5675	OK	OK	-0.26081
ENSG0000011	-	-	ENSG0000011281	'PRSS16	-	6:27247700-27256624	-	-	IGV_LINK	321.947	13.2753	2.67001	23.8806	OK	143.34	6.59674	0.148272	13.0452	OK	OK	-1.00892
ENSG0000011	-	-	ENSG0000011281	'MEP1A	-	6:46793389-46839782	-	-	IGV_LINK	1.93944	0.040184	0	0.132487	OK	6.18735	0.128267	0	0.309423	OK	NOTEST	1.67446
ENSG0000011	-	-	ENSG0000011283	'TBX18	-	6:84687350-84764519	-	-	IGV_LINK	1.93944	0.017999	0	0.391342	OK	8.2498	0.076576	0	0.463027	OK	NOTEST	2.08899
ENSG0000011	-	-	ENSG0000011285	'ERBIN	-	5:65926474-66082549	-	-	IGV_LINK	1217	15.0106	0	32.1733	OK	950.789	12.2969	0	25.7549	OK	OK	-0.28768
ENSG0000011	-	-	ENSG0000011285	'PCDHB2	-	5:141046259-141098703	-	-	IGV_LINK	21.3339	0.792176	0	2.57882	OK	24.7494	1.01221	0	3.16737	OK	OK	0.353622
ENSG0000011	-	-	ENSG0000011285	'HARS2	-	5:140664675-140706676	-	-	IGV_LINK	1055.1	32.1974	0	90.5334	OK	1205.05	34.6615	0	98.4034	OK	OK	0.106389
ENSG0000011	-	-	ENSG0000011287	'NUDT12	-	5:103548854-103562793	-	-	IGV_LINK	279.28	7.25348	1.68931	12.8177	OK	317.617	8.07102	1.8337	14.3083	OK	OK	0.154077
ENSG0000011	-	-	ENSG0000011287	'CEP72	-	5:612271-693395	-	-	IGV_LINK	80.4868	2.51729	0	6.57964	OK	82.498	2.27544	0	5.03341	OK	OK	-0.14573
ENSG0000011	-	-	ENSG0000011289	'MAN2A1	-	5:109689365-109869625	-	-	IGV_LINK	1165.6	14.9067	4.03753	25.7759	OK	870.354	10.4503	4.04469	16.8559	OK	OK	-0.51241
ENSG0000011	-	-	ENSG0000011290	'SEMA5A	-	5:9001773-9546075	-	-	IGV_LINK	517.831	2.51802	0.919232	4.11681	OK	218.62	1.06318	0.193181	1.93317	OK	OK	-1.24391
ENSG0000011	-	-	ENSG0000011293	'C7	-	5:40909251-40982939	-	-	IGV_LINK	9.69721	0.149297	0	0.800284	OK	57.7486	1.66554	0	6.69836	OK	OK	3.47974
ENSG0000011	-	-	ENSG0000011294	'PAPD7	-	5:6713006-6757048	-	-	IGV_LINK	844.627	15.5567	0	31.1166	OK	715.67	13.6861	0	27.6026	OK	OK	-0.18482
ENSG0000011	-	-	ENSG0000011296	'GHR	-	5:42423776-42721878	-	-	IGV_LINK	98.9115	1.47573	0	2.95373	OK	164.996	2.82252	0	5.73858	OK	OK	0.935562
ENSG0000011	-	-	ENSG0000011297	'HMGCS1	-	5:43287600-43313512	-	-	IGV_LINK	667.168	15.2673	2.39836	28.1363	OK	632.141	14.3006	2.48285	26.1183	OK	OK	-0.09437
ENSG0000011	-	-	ENSG0000011297	'DAP	-	5:10679229-10770294	-	-	IGV_LINK	1952.05	51.2785	22.3576	80.1993	OK	1407.62	38.0546	11.4383	64.6709	OK	OK	-0.43028
ENSG0000011	-	-	ENSG0000011298	'NME5	-	5:138115171-138139443	-	-	IGV_LINK	263.764	16.8382	4.28144	29.395	OK	337.211	19.5286	6.24765	32.8095	OK	OK	0.213844
ENSG0000011	-	-	ENSG0000011298	'BRD8	-	5:138139765-138213343	-	-	IGV_LINK	2494.12	73.0515	4.30269	141.8	OK	2287.26	61.1219	1.7744	120.469	OK	OK	-0.25722
ENSG0000011	-	-	ENSG0000011298	'KIF20A	-	5:138139765-138213343	-	-	IGV_LINK	122.185	3.53098	0	19.274	OK	254.713	5.68683	0	20.3329	OK	OK	0.687556
ENSG0000011	-	-	ENSG0000011299	'NNT	-	5:43571593-43707405	-	-	IGV_LINK	974.569	15.2974	0.204472	30.3904	OK	823.949	14.7418	0	30.151	OK	OK	-0.05337
ENSG0000011	-	-	ENSG0000011299	'MRPS30	-	5:44808924-44820428	-	-	IGV_LINK	341.342	12.2309	1.84375	22.618	OK	358.866	12.8439	3.46429	22.2235	OK	OK	0.070556
ENSG0000011	-	-	ENSG0000011301	'HSPA9	-	5:138554881-138575444	-	-	IGV_LINK	5216.13	125.032	6.18126	243.882	OK	7757.91	177.737	0	369.367	OK	OK	0.507448
ENSG0000011	-	-	ENSG0000011304	'MRPS27	-	5:72219408-72368395	-	-	IGV_LINK	1027.9	24.616	4.21272	45.0194	OK	1158.07	28.2561	5.88976	50.6224	OK	OK	0.198962
ENSG0000011	-	-	ENSG0000011306	'PFDN1	-	5:140174641-140303121	-	-	IGV_LINK	1068.63	53.3388	0	109.807	OK	1170.44	59.348	7.29657	111.399	OK	OK	0.154013
ENSG0000011	-	-	ENSG0000011307	'HBEGF	-	5:140332842-140346631	-	-	IGV_LINK	141.579	3.7407	0.668244	6.81316	OK	283.587	8.30864	1.95508	14.6622	OK	OK	1.1513
ENSG0000011	-	-	ENSG0000011307	'SLC4A9	-	5:140360201-140401367	-	-	IGV_LINK	3.87888	0.07503	0	0.378898	OK	0	0	0	0	OK	NOTEST	'-inf
ENSG0000011	-	-	ENSG0000011308	'LOX	-	5:121961960-122078360	-	-	IGV_LINK	493.588	10.167	1.63643	18.6977	OK	8845.85	184.345	41.1545	327.535	OK	OK	4.18043
ENSG0000011	-	-	ENSG0000011308	'GZMK	-	5:54977863-55042844	-	-	IGV_LINK	625.47	28.0342	0	72.7923	OK	169.121	7.59195	0	23.887	OK	OK	-1.88464
ENSG0000011	-	-	ENSG0000011310	'CDH9	-	5:26880599-27121150	-	-	IGV_LINK	15.5155	0.330545	0	0.82098	OK	9.28102	0.180156	0	0.618015	OK	NOTEST	-0.8756
ENSG0000011	-	-	ENSG0000011310	'APBB3	-	5:140558267-140594570	-	-	IGV_LINK	967.781	42.1917	0	95.597	OK	1095.16	47.8079	0	108.499	OK	OK	0.18029
ENSG0000011	-	-	ENSG0000011311	'TMCO6	-	5:140638739-140662479	-	-	IGV_LINK	471.284	23.8716	0	83.4791	OK	422.802	19.0176	0	63.3407	OK	OK	-0.32796
ENSG0000011	-	-	ENSG0000011314	'SPARC	-	5:151661095-151724782	-	-	IGV_LINK	15941.2	700.23	0	1419.68	OK	17047.2	814.654	0	1651.63	OK	OK	0.218359
ENSG0000011	-	-	ENSG0000011314	'IK	-	5:140638739-140662479	-	-	IGV_LINK	2906.25	108.496	13.3276	203.665	OK	2849.27	106.264	11.7924	200.736	OK	OK	-0.02999
ENSG0000011	-	-	ENSG0000011316	'HMGCR	-	5:75320154-75362104	-	-	IGV_LINK	432.495	6.33498	0.474329	12.1956	OK	380.522	6.58856	0	13.8789	OK	OK	0.056622
ENSG0000011	-	-	ENSG0000011316	'COL4A3BP	-	5:75368485-75601144	-	-	IGV_LINK	799.05	13.2783	1.12597	25.4307	OK	791.981	13.2642	1.47363	25.0549	OK	OK	-0.00153
ENSG0000011	-	-	ENSG0000011319	'FAF2	-	5:176447627-176510074	-	-	IGV_LINK	1308.15	18.597	5.39405	31.8	OK	2024.29	27.83	8.20869	47.4512	OK	OK	0.581568
ENSG0000011	-	-	ENSG0000011319	'HAND1	-	5:154474971-154478264	-	-	IGV_LINK	0	0	0	0	OK	0	0	0	0	OK	NOTEST	0
ENSG0000011	-	-	ENSG0000011320	'PCDHB3	-	5:141100241-141249365	-	-	IGV_LINK	9.69721	0.171718	0	1.55711	OK	21.6557	0.383619	0	2.26393	OK	NOTEST	1.15963
ENSG0000011	-	-	ENSG0000011320	'PCDHB5	-	5:141100241-141249365	-	-	IGV_LINK	221.096	3.85105	0	11.5702	OK	196.964	3.43193	0	10.068	OK	OK	-0.16623
ENSG0000011	-	-	ENSG0000011321	'PCDHB6	-	5:141100241-141249365	-	-	IGV_LINK	219.157	4.19986	0	11.1345	OK	240.275	4.60651	0	12.3092	OK	OK	0.133334
ENSG0000011	-	-	ENSG0000011321	'PCDHB7	-	5:141100241-141249365	-	-	IGV_LINK	12.6064	0.197757	0	1.40474	OK	14.4371	0.226549	0	1.48974	OK	NOTEST	0.196095
ENSG0000011	-	-	ENSG0000011323	'PDE8B	-	5:77210448-77620611	-	-	IGV_LINK	91.1537	0.951515	0	2.74661	OK	107.247	1.24133	0	4.12446	OK	OK	0.383586
ENSG0000011	-	-	ENSG0000011324	'CLK4	-	5:178602663-178630615	-	-	IGV_LINK	1975.8	47.2145	17.0676	77.3615	OK	1076.52	27.2603	9.09031	45.4303	OK	OK	-0.79243
ENSG0000011	-	-	ENSG0000011324	'PCDHB15	-	5:141100241-141249365	-	-	IGV_LINK	65.941	1.33856	0	7.16188	OK	61.2125	1.26216	0	6.87478	OK	OK	-0.08479
ENSG0000011	-	-	ENSG0000011324	'HAVCR1	-	5:157029412-157059119	-	-	IGV_LINK	1017.24	48.7926	14.2257	83.3596	OK	797.137	40.326	12.9014	67.7506	OK	OK	-0.27495
ENSG0000011	-	-	ENSG0000011326	'GRM6	-	5:178969389-178996206	-	-	IGV_LINK	4.8486	0.045766	0	0.468888	OK	14.4371	0.136299	0	0.580871	OK	NOTEST	1.57442
ENSG0000011	-	-	ENSG0000011326	'ITK	-	5:157085831-157255191	-	-	IGV_LINK	566.317	7.45729	0	17.9811	OK	289.774	4.0067	0	10.8955	OK	OK	-0.89624
ENSG0000011	-	-	ENSG0000011326	'RNF130	-	5:179911650-180072118	-	-	IGV_LINK	2825.77	94.7144	37.095	152.334	OK	2842.06	95.1512	36.1768	154.126	OK	OK	0.006639
ENSG0000011	-	-	ENSG0000011327	'THG1L	-	5:157731196-157741448	-	-	IGV_LINK	299.644	10.3893	1.51133	19.2674	OK	445.489	17.4505	3.35042	31.5506	OK	OK	0.748165
ENSG0000011	-	-	ENSG0000011327	'ARSB	-	5:78777208-78986087	-	-	IGV_LINK	530.437	7.35106	0.785613	13.9165	OK	896.135	13.3539	2.42799	24.2797	OK	OK	0.861232
ENSG0000011	-	-	ENSG0000011328	'CLINT1	-	5:157785742-157859175	-	-	IGV_LINK	1585.49	36.6029	13.5713	59.6346	OK	3344.26	81.9935	21.4865	142.5	OK	OK	1.16355
ENSG0000011	-	-	ENSG0000011329	'THBS4	-	5:79991310-80083654	-	-	IGV_LINK	34.9099	0.67341	0	1.62523	OK	107.247	2.71342	0	6.25256	OK	OK	2.01055
ENSG0000011	-	-	ENSG0000011330	'CNOT6	-	5:180494411-180578405	-	-	IGV_LINK	548.862	6.63536	0	13.5629	OK	577.486	6.90506	0	14.2944	OK	OK	0.057479
ENSG0000011	-	-	ENSG0000011330	'IL12B	-	5:159310744-159972544	-	-	IGV_LINK	58.1832	1.26675	0	3.0289	OK	11.3435	0.247076	0	0.950852	OK	OK	-2.3581
ENSG0000011	-	-	ENSG0000011330	'BTNL8	-	5:180899076-180950906	-	-	IGV_LINK	12.6064	0.662321	0	1.50058	OK	18.562	0.776386	0	1.73505	OK	OK	0.229243
ENSG0000011	-	-	ENSG0000011331	'TTC1	-	5:160009112-160119423	-	-	IGV_LINK	1079.3	47.1492	14.5933	79.7051	OK	1450.93	63.9818	18.9294	109.034	OK	OK	0.440428
ENSG0000011	-	-	ENSG0000011331	'MSH3	-	5:80626227-80876460	-	-	IGV_LINK	239.521	3.5824	0	8.30566	OK	322.773	4.64229	0	9.87451	OK	OK	0.373911
ENSG0000011	-	-	ENSG0000011331	'RASGRF2	-	5:80947696-81301560	-	-	IGV_LINK	203.641	2.12539	0	4.26592	OK	244.4	2.60241	0	5.21203	OK	OK	0.29212
ENSG0000011	-	-	ENSG0000011332	'GABRG2	-	5:161910251-162162977	-	-	IGV_LINK	23.2733	1.10529	0	2.72226	OK	17.5308	0.680788	0	1.5857	OK	OK	-0.69914
ENSG0000011	-	-	ENSG0000011332	'CCNG1	-	5:163437568-163446151	-	-	IGV_LINK	2104.29	86.6423	25.2025	148.082	OK	1634.49	65.9693	16.8283	115.11	OK	OK	-0.39328
ENSG0000011	-	-	ENSG0000011335	'POLR3G	-	5:90458202-90514553	-	-	IGV_LINK	13.5761	0.72714	0	3.39633	OK	23.7182	1.30334	0	4.44995	OK	OK	0.841907
ENSG0000011	-	-	ENSG0000011336	'DROSHA	-	5:31400496-31532196	-	-	IGV_LINK	944.508	21.5778	0	44.9207	OK	800.231	19.6145	0	41.6589	OK	OK	-0.13762
ENSG0000011	-	-	ENSG0000011336	'CDH6	-	5:31093976-31329146	-	-	IGV_LINK	2248.56	30.7209	4.35727	57.0845	OK	11758	175.725	0	361.883	OK	OK	2.51603
ENSG0000011	-	-	ENSG0000011336	'LMNB1	-	5:126776622-126837020	-	-	IGV_LINK	523.649	14.6664	4.29764	25.0352	OK	380.522	11.0075	2.72953	19.2854	OK	OK	-0.41404
ENSG0000011	-	-	ENSG0000011336	'ARRDC3	-	5:91368723-91439085	-	-	IGV_LINK	4193.07	79.1133	4.23474	153.992	OK	4317.74	76.9087	7.767	146.05	OK	OK	-0.04077
ENSG0000011	-	-	ENSG0000011338	'GOLPH3	-	5:32124703-32174350	-	-	IGV_LINK	1007.54	22.7121	9.31795	36.1063	OK	1277.69	28.4635	12.337	44.59	OK	OK	0.32565
ENSG0000011	-	-	ENSG0000011338	'SUB1	-	5:32531632-32604079	-	-	IGV_LINK	1610.71	128.346	37.0713	219.62	OK	1239.53	99.668	29.9728	169.363	OK	OK	-0.36483
ENSG0000011	-	-	ENSG0000011338	'NPR3	-	5:32689069-32791724	-	-	IGV_LINK	1452.64	16.7246	4.81495	28.6342	OK	131.997	1.68738	0	3.38253	OK	OK	-3.30912
ENSG0000011	-	-	ENSG0000011339	'FAM172A	-	5:93618068-94111699	-	-	IGV_LINK	418.919	12.9516	0	30.6392	OK	359.898	9.92877	0	26.6003	OK	OK	-0.38345
ENSG0000011	-	-	ENSG0000011339	'SLC27A6	-	5:128257908-129033642	-	-	IGV_LINK	0	0	0	0	OK	0	0	0	0	OK	NOTEST	0
ENSG0000011	-	-	ENSG0000011340	'TARS	-	5:33440695-33469539	-	-	IGV_LINK	1178.21	34.0003	9.43553	58.5651	OK	1108.57	28.5514	9.3588	47.744	OK	OK	-0.25199
ENSG0000011	-	-	ENSG0000011343	'IRX4	-	5:1877412-1887236	-	-	IGV_LINK	0	0	0	0	OK	0	0	0	0	OK	NOTEST	0
ENSG0000011	-	-	ENSG0000011344	'LNPEP	-	5:96814027-97037515	-	-	IGV_LINK	1359.55	6.4764	0	14.1495	OK	1057.01	5.22872	0	10.8137	OK	OK	-0.30873
ENSG0000011	-	-	ENSG0000011344	'PDE4D	-	5:58969037-60547657	-	-	IGV_LINK	530.437	7.39833	0	20.1138	OK	431.052	5.88706	0	14.8363	OK	OK	-0.32965
ENSG0000011	-	-	ENSG0000011345	'RAD1	-	5:34905263-34925996	-	-	IGV_LINK	330.675	8.6387	0	19.591	OK	277.4	7.34559	0	16.1691	OK	OK	-0.23394
ENSG0000011	-	-	ENSG0000011346	'BRIX1	-	5:34905263-34925996	-	-	IGV_LINK	298.674	12.3646	0.142437	24.5867	OK	298.024	11.9171	0.241207	23.5929	OK	OK	-0.05318
ENSG0000011	-	-	ENSG0000011349	'AGXT2	-	5:34998100-35048135	-	-	IGV_LINK	591.53	16.1046	6.02725	26.1819	OK	2288.29	62.2894	25.756	98.8229	OK	OK	1.95152
ENSG0000011	-	-	ENSG0000011349	'PRLR	-	5:35048755-35230589	-	-	IGV_LINK	35.8797	0.450127	0	1.7382	OK	96.9351	0.524849	0	1.48023	OK	NOTEST	0.221572
ENSG0000011	-	-	ENSG0000011350	'SLC12A7	-	5:1050375-1112035	-	-	IGV_LINK	3156.44	42.4696	0.557582	84.3817	OK	3734.07	49.9375	1.47873	98.3963	OK	OK	0.233692
ENSG0000011	-	-	ENSG0000011352	'IL4	-	5:132673985-132682676	-	-	IGV_LINK	1.93944	0.349436	0	1.42572	OK	0	0	0	0	OK	NOTEST	'-inf
ENSG0000011	-	-	ENSG0000011352	'RAD50	-	5:132410635-132664272	-	-	IGV_LINK	1133.05	12.5149	0	42.0687	OK	1437.17	14.6527	0	34.4132	OK	OK	0.227521
ENSG0000011	-	-	ENSG0000011352	'IL5	-	5:132410635-132664272	-	-	IGV_LINK	0	0	0	0	OK	0	0	0	0	OK	NOTEST	0
ENSG0000011	-	-	ENSG0000011353	'ST8SIA4	-	5:100806934-100903266	-	-	IGV_LINK	499.406	5.11419	0.386824	9.84156	OK	521.8	6.44962	0	13.2644	OK	OK	0.334707
ENSG0000011	-	-	ENSG0000011355	'GNPDA1	-	5:141991748-142013041	-	-	IGV_LINK	1212.15	37.7962	10.0426	65.5499	OK	1912.92	58.7093	18.8045	98.614	OK	OK	0.635347
ENSG0000011	-	-	ENSG0000011355	'PCDH12	-	5:141943584-141990291	-	-	IGV_LINK	700.138	6.38425	0	13.5999	OK	839.417	8.46445	0	20.8073	OK	OK	0.406899
ENSG0000011	-	-	ENSG0000011355	'SKP1	-	5:134114710-134371047	-	-	IGV_LINK	5132.73	509.835	63.568	956.102	OK	5733.61	590.959	89.4248	1092.49	OK	OK	0.213026
ENSG0000011	-	-	ENSG0000011356	'NUP155	-	5:37288136-37371181	-	-	IGV_LINK	422.798	5.12543	0.577931	9.67292	OK	441.364	5.12354	1.13352	9.11356	OK	OK	-0.00053
ENSG0000011	-	-	ENSG0000011357	'PPP2CA	-	5:134114710-134371047	-	-	IGV_LINK	1512.97	65.4118	0	207.519	OK	1914.55	74.6268	0	236.669	OK	OK	0.190143
ENSG0000011	-	-	ENSG0000011357	'FGF1	-	5:142310426-142698070	-	-	IGV_LINK	24.243	0.379812	0	2.11196	OK	19.5933	0.316715	0	1.8031	OK	NOTEST	-0.2621
ENSG0000011	-	-	ENSG0000011358	'NR3C1	-	5:143277930-143435512	-	-	IGV_LINK	2253.63	26.5164	2.81439	50.2184	OK	4455.92	50.6735	2.92188	98.4252	OK	OK	0.934347
ENSG0000011	-	-	ENSG0000011358	'C5orf15	-	5:133955501-133968787	-	-	IGV_LINK	2069.38	87.776	22.658	152.894	OK	2889.49	120.498	30.3947	210.601	OK	OK	0.457107
ENSG0000011	-	-	ENSG0000011359	'PPWD1	-	5:65563235-65587549	-	-	IGV_LINK	907.658	37.3018	11.3725	63.2311	OK	552.737	20.5959	5.91866	35.2731	OK	OK	-0.85689
ENSG0000011	-	-	ENSG0000011359	'LIFR	-	5:38474962-38671216	-	-	IGV_LINK	1464.28	9.71383	0	20.2162	OK	538.299	3.27083	0.447318	6.09434	OK	OK	-1.57039
ENSG0000011	-	-	ENSG0000011359	'TRIM23	-	5:65589679-65871725	-	-	IGV_LINK	328.735	7.3318	0	16.9147	OK	248.525	5.61407	0	13.9826	OK	OK	-0.38512
ENSG0000011	-	-	ENSG0000011359	'TRAPPC13	-	5:65589679-65871725	-	-	IGV_LINK	318.755	10.2727	0	24.9505	OK	298.076	10.2786	0	25.5557	OK	OK	0.000825
ENSG0000011	-	-	ENSG0000011360	'C9	-	5:39284261-39462300	-	-	IGV_LINK	13.5761	0.695412	0	11.7546	OK	142.309	3.94049	0	38.6842	OK	OK	2.50243
ENSG0000011	-	-	ENSG0000011361	'SEC24A	-	5:134601143-134727823	-	-	IGV_LINK	562.438	5.3762	0.557497	10.1949	OK	832.199	8.27185	0.117887	16.4258	OK	OK	0.621622
ENSG0000011	-	-	ENSG0000011362	'TXNDC15	-	5:134873802-134901525	-	-	IGV_LINK	1010.45	47.4932	8.35762	86.6288	OK	1107.54	57.8894	10.0092	105.77	OK	OK	0.28558
ENSG0000011	-	-	ENSG0000011363	'TTC33	-	5:40512332-40755975	-	-	IGV_LINK	177.459	8.29425	0	19.2904	OK	141.278	6.68309	0	15.3449	OK	OK	-0.3116
ENSG0000011	-	-	ENSG0000011364	'RARS	-	5:168486444-168519299	-	-	IGV_LINK	1231.55	37.8757	14.4853	61.2661	OK	1415.87	42.7814	16.6738	68.8889	OK	OK	0.17571
ENSG0000011	-	-	ENSG0000011364	'WWC1	-	5:168291650-168472303	-	-	IGV_LINK	2192.54	38.3893	8.1473	68.6313	OK	2478.03	43.3192	8.58378	78.0546	OK	OK	0.174303
ENSG0000011	-	-	ENSG0000011364	'H2AFY	-	5:135027734-135401296	-	-	IGV_LINK	2466	79.3573	27.37	131.345	OK	2282.1	79.329	27.0309	131.627	OK	OK	-0.00051
ENSG0000011	-	-	ENSG0000011364	'TCERG1	-	5:146447310-146511961	-	-	IGV_LINK	1476.88	37.421	6.37041	68.4715	OK	1143.63	33.7096	4.13844	63.2807	OK	OK	-0.15069
ENSG0000011	-	-	ENSG0000011365	'DPYSL3	-	5:147390810-147510056	-	-	IGV_LINK	595.408	6.65566	2.10643	11.2049	OK	1010.6	12.3264	2.62592	22.0269	OK	OK	0.889103
ENSG0000011	-	-	ENSG0000011365	'SMAD5	-	5:136129506-136188747	-	-	IGV_LINK	766.079	10.7731	0	23.3774	OK	639.359	11.3818	0	25.2742	OK	OK	0.079285
ENSG0000011	-	-	ENSG0000011371	'CSNK1A1	-	5:149492196-149551552	-	-	IGV_LINK	1930.25	59.3393	6.40194	112.277	OK	2922.76	90.1534	11.0754	169.231	OK	OK	0.603395
ENSG0000011	-	-	ENSG0000011371	'HMGXB3	-	5:149960736-150053142	-	-	IGV_LINK	2042.23	31.2881	0.315793	62.2605	OK	2240.85	33.1176	1.85713	64.3781	OK	OK	0.081983
ENSG0000011	-	-	ENSG0000011371	'ERGIC1	-	5:172834274-172952685	-	-	IGV_LINK	8449.17	182.256	46.4612	318.05	OK	10615.4	231.386	47.6596	415.113	OK	OK	0.344338
ENSG0000011	-	-	ENSG0000011372	'PDGFRB	-	5:150113836-150155872	-	-	IGV_LINK	3444.45	44.6186	0	95.6436	OK	1578.81	20.6264	0	45.0583	OK	OK	-1.11315
ENSG0000011	-	-	ENSG0000011372	'CDX1	-	5:150166794-150184558	-	-	IGV_LINK	0.969721	0.046689	0	0.201082	OK	2.06245	0.099398	0	0.305891	OK	NOTEST	1.09016

ENSG0000011	-	-	ENSG0000011373	'ATP6V0E1	-	5:172983756-173035445	-	-	IGV_LINK	3015.83	233.884	85.2412	382.526	OK	5235.53	386.503	135.04	637.966	OK	OK	0.724689
ENSG0000011	-	-	ENSG0000011373	'BNIP1	-	5:173144161-173164387	-	-	IGV_LINK	181.338	10.0571	2.47675	17.6374	OK	238.213	13.2524	3.71304	22.7919	OK	OK	0.398044
ENSG0000011	-	-	ENSG0000011373	'STC2	-	5:173314712-173329503	-	-	IGV_LINK	1120.03	15.4399	0.492237	30.3876	OK	1429.28	25.9955	0	53.5477	OK	OK	0.751596
ENSG0000011	-	-	ENSG0000011374	'CPEB4	-	5:173888279-173961976	-	-	IGV_LINK	1685.37	29.3913	0	59.4509	OK	1517.96	26.7066	0	58.3412	OK	OK	-0.1382
ENSG0000011	-	-	ENSG0000011374	'HRH2	-	5:175658029-175710756	-	-	IGV_LINK	747.655	11.2998	4.61722	17.9823	OK	2753.37	42.0853	17.5328	66.6378	OK	OK	1.89702
ENSG0000011	-	-	ENSG0000011375	'DBN1	-	5:177456607-177474401	-	-	IGV_LINK	416.98	10.645	2.38072	18.9094	OK	496.019	13.2195	0.759947	25.6791	OK	OK	0.312487
ENSG0000011	-	-	ENSG0000011376	'ZNF346	-	5:177022695-177081189	-	-	IGV_LINK	320.008	9.81892	0.100895	19.537	OK	288.743	8.31928	0.725214	15.9134	OK	OK	-0.23911
ENSG0000011	-	-	ENSG0000011376	'UNC5A	-	5:176810476-176899332	-	-	IGV_LINK	1.93944	0.030031	0	0.279243	OK	244.4	4.11703	0	8.53253	OK	OK	7.099
ENSG0000011	-	-	ENSG0000011379	'EHHADH	-	3:185162870-185281990	-	-	IGV_LINK	1219.91	20.7045	8.03156	33.3774	OK	2820.4	46.0468	18.6577	73.4359	OK	OK	1.15316
ENSG0000011	-	-	ENSG0000011380	'CNTN3	-	3:74262567-74521140	-	-	IGV_LINK	0.969721	0.141037	0	0.553338	OK	7.21857	0.342353	0	1.06396	OK	NOTEST	1.27941
ENSG0000011	-	-	ENSG0000011381	'SMC4	-	3:160225495-160485773	-	-	IGV_LINK	680.744	16.9014	0	46.3289	OK	485.707	12.3385	0	34.278	OK	OK	-0.45397
ENSG0000011	-	-	ENSG0000011381	'SELENOK	-	3:53884409-53891988	-	-	IGV_LINK	458.678	39.7473	14.113	65.3815	OK	412.49	35.8427	11.5406	60.1449	OK	OK	-0.14918
ENSG0000011	-	-	ENSG0000011381	'ACTR8	-	3:53867065-53882202	-	-	IGV_LINK	313.22	9.33766	0.457264	18.2181	OK	300.086	10.8932	0.047856	21.7385	OK	OK	0.222291
ENSG0000011	-	-	ENSG0000011383	'TBCCD1	-	3:186538440-186773476	-	-	IGV_LINK	167.288	4.07313	0	11.6215	OK	173.399	4.84237	0	15.5237	OK	OK	0.249575
ENSG0000011	-	-	ENSG0000011384	'TIMMDC1	-	3:119498531-119559602	-	-	IGV_LINK	832.02	51.6593	14.0778	89.2409	OK	1015.76	57.6061	16.5132	98.6989	OK	OK	0.157192
ENSG0000011	-	-	ENSG0000011385	'CRBN	-	3:3126915-3179727	-	-	IGV_LINK	671.047	25.2633	1.45106	49.0755	OK	499.113	15.4677	0.689121	30.2462	OK	OK	-0.70779
ENSG0000011	-	-	ENSG0000011388	'KNG1	-	3:186538440-186773476	-	-	IGV_LINK	0.969721	0.016751	0	0.34901	OK	0	0	0	0	OK	NOTEST	'-inf
ENSG0000011	-	-	ENSG0000011390	'HRG	-	3:186538440-186773476	-	-	IGV_LINK	0	0	0	0	OK	0	0	0	0	OK	NOTEST	0
ENSG0000011	-	-	ENSG0000011391	'BCL6	-	3:187698258-187746028	-	-	IGV_LINK	1887.08	45.9309	11.71	80.1518	OK	2298.6	56.1107	16.7613	95.46	OK	OK	0.28881
ENSG0000011	-	-	ENSG0000011392	'HGD	-	3:120628172-120682571	-	-	IGV_LINK	862.082	36.8268	9.04853	64.605	OK	1418.97	61.2459	16.8045	105.687	OK	OK	0.733857
ENSG0000011	-	-	ENSG0000011394	'CLDN16	-	3:190322540-190412143	-	-	IGV_LINK	9.69721	0.362536	0	1.12018	OK	12.3747	0.225014	0	0.697338	OK	NOTEST	-0.68811
ENSG0000011	-	-	ENSG0000011396	'ARL6	-	3:97764520-97821884	-	-	IGV_LINK	157.095	6.95526	0	15.5759	OK	127.872	3.68813	0	8.00513	OK	OK	-0.91522
ENSG0000011	-	-	ENSG0000011397	'NPHP3	-	3:132558137-132874223	-	-	IGV_LINK	1010.62	18.4881	0	58.0075	OK	584.001	9.99477	0	30.4942	OK	OK	-0.88735
ENSG0000011	-	-	ENSG0000011401	'CD86	-	3:122055365-122121139	-	-	IGV_LINK	837.839	22.3557	7.21109	37.5004	OK	870.354	23.9814	8.5429	39.42	OK	OK	0.101273
ENSG0000011	-	-	ENSG0000011401	'AMOTL2	-	3:134351851-134375479	-	-	IGV_LINK	1693.13	24.9604	9.00578	40.9149	OK	2112.98	30.8607	11.6193	50.1021	OK	OK	0.306133
ENSG0000011	-	-	ENSG0000011402	'NIT2	-	3:100334700-100361635	-	-	IGV_LINK	1986.96	89.0226	28.1398	149.905	OK	2432.66	117.537	37.0272	198.048	OK	OK	0.400877
ENSG0000011	-	-	ENSG0000011402	'FAM162A	-	3:122384175-122416051	-	-	IGV_LINK	928.023	60.6094	16.6586	104.56	OK	1086.91	75.2681	20.4164	130.12	OK	OK	0.312497
ENSG0000011	-	-	ENSG0000011402	'OGG1	-	3:9749943-9855138	-	-	IGV_LINK	664.259	17.1925	0	55.8852	OK	710.514	20.8502	0	74.6851	OK	OK	0.278287
ENSG0000011	-	-	ENSG0000011403	'KPNA1	-	3:122416206-122514945	-	-	IGV_LINK	816.505	13.3744	1.94397	24.8047	OK	850.761	15.0742	2.96712	27.1813	OK	OK	0.172612
ENSG0000011	-	-	ENSG0000011405	'PCCB	-	3:136250305-136752403	-	-	IGV_LINK	352.009	10.9284	0	25.0255	OK	533.143	18.5839	1.86896	35.2989	OK	OK	0.765977
ENSG0000011	-	-	ENSG0000011406	'UBE3A	-	15:24823636-25439056	-	-	IGV_LINK	1298.46	25.9808	0	80.6074	OK	1153.94	25.6882	0	72.6321	OK	OK	-0.01634
ENSG0000011	-	-	ENSG0000011409	'ARMC8	-	3:138187247-138329886	-	-	IGV_LINK	612.863	14.3053	1.56039	27.0501	OK	590.892	12.3054	2.98603	21.6248	OK	OK	-0.21725
ENSG0000011	-	-	ENSG0000011410	'CEP70	-	3:138494343-138594538	-	-	IGV_LINK	223.036	7.46031	0.567562	14.3531	OK	465.082	16.0967	3.03181	29.1617	OK	OK	1.10946
ENSG0000011	-	-	ENSG0000011411	'RBP2	-	3:139389814-139678017	-	-	IGV_LINK	0	0	0	0	OK	1.03122	0.111691	0	1.47946	OK	NOTEST	inf
ENSG0000011	-	-	ENSG0000011411	'RBP1	-	3:139389814-139678017	-	-	IGV_LINK	119.276	13.9073	0	33.8322	OK	51.5612	6.49512	0	16.9471	OK	OK	-1.09841
ENSG0000011	-	-	ENSG0000011412	'SLC25A36	-	3:140941829-140979933	-	-	IGV_LINK	1046.33	26.6498	2.06376	51.2358	OK	886.853	21.732	1.99677	41.4672	OK	OK	-0.2943
ENSG0000011	-	-	ENSG0000011412	'GRK7	-	3:141778147-141818490	-	-	IGV_LINK	1.93944	0.035643	0	0.109154	OK	1.03122	0.018959	0	0.070959	OK	NOTEST	-0.91074
ENSG0000011	-	-	ENSG0000011412	'RNF7	-	3:141738203-141747560	-	-	IGV_LINK	786.443	53.0062	17.0165	88.9959	OK	893.041	60.5349	19.5695	101.5	OK	OK	0.191607
ENSG0000011	-	-	ENSG0000011412	'TFDP2	-	3:141944427-142149544	-	-	IGV_LINK	963.902	17.8026	0	41.1512	OK	977.601	14.3158	0	28.9937	OK	OK	-0.31448
ENSG0000011	-	-	ENSG0000011412	'XRN1	-	3:142306606-142448062	-	-	IGV_LINK	992.024	8.02074	0	17.1941	OK	622.86	4.44662	0	9.67652	OK	OK	-0.85103
ENSG0000011	-	-	ENSG0000011416	'KAT2B	-	3:20040022-20154404	-	-	IGV_LINK	864.021	16.065	0	36.8135	OK	720.826	11.2509	0	23.9496	OK	OK	-0.51389
ENSG0000011	-	-	ENSG0000011420	'BCHE	-	3:165772903-165837472	-	-	IGV_LINK	0	0	0	0	OK	0	0	0	0	OK	NOTEST	0
ENSG0000011	-	-	ENSG0000011420	'SERPINI2	-	3:167441788-167653983	-	-	IGV_LINK	1.93944	0.094347	0	0.575519	OK	0	0	0	0	OK	NOTEST	'-inf
ENSG0000011	-	-	ENSG0000011420	'PDCD10	-	3:167683297-167734939	-	-	IGV_LINK	398.555	24.952	4.6042	45.2998	OK	390.834	25.123	7.85605	42.39	OK	OK	0.009857
ENSG0000011	-	-	ENSG0000011424	'LRRC31	-	3:169839178-169869930	-	-	IGV_LINK	2.90916	0.075116	0	0.245914	OK	106.216	3.31017	0.412774	6.20757	OK	OK	5.46163
ENSG0000011	-	-	ENSG0000011425	'WNT5A	-	3:55465714-55490539	-	-	IGV_LINK	35.8797	0.439872	0	1.18855	OK	67.0296	1.04931	0	2.97435	OK	OK	1.25429
ENSG0000011	-	-	ENSG0000011426	'PFKFB4	-	3:48517683-48563773	-	-	IGV_LINK	580.863	12.2009	1.65767	22.7441	OK	1098.25	22.0478	4.7836	39.312	OK	OK	0.853645
ENSG0000011	-	-	ENSG0000011427	'COL7A1	-	3:48564072-48595267	-	-	IGV_LINK	294.795	2.78499	0	8.01671	OK	237.182	2.43239	0	5.79981	OK	OK	-0.1953
ENSG0000011	-	-	ENSG0000011427	'FGF12	-	3:192139394-192767764	-	-	IGV_LINK	18.4247	1.14826	0	2.98556	OK	15.4684	0.988956	0	2.61464	OK	OK	-0.21548
ENSG0000011	-	-	ENSG0000011430	'PRKAR2A	-	3:48744596-48851981	-	-	IGV_LINK	457.708	5.00537	1.19159	8.81914	OK	597.079	6.50306	1.04213	11.964	OK	OK	0.377644
ENSG0000011	-	-	ENSG0000011431	'HES1	-	3:194136144-194138732	-	-	IGV_LINK	434.435	18.8453	6.7918	30.8989	OK	488.801	22.2192	7.69994	36.7384	OK	OK	0.237599
ENSG0000011	-	-	ENSG0000011431	'USP4	-	3:49268601-49340712	-	-	IGV_LINK	987.175	25.8119	3.76508	47.8588	OK	985.851	26.4046	4.00728	48.8019	OK	OK	0.032751
ENSG0000011	-	-	ENSG0000011433	'ACAP2	-	3:195274735-195443078	-	-	IGV_LINK	640.985	6.2254	0.452474	11.9983	OK	634.203	6.15617	0.169896	12.1425	OK	OK	-0.01613
ENSG0000011	-	-	ENSG0000011434	'ECT2	-	3:172750681-172821474	-	-	IGV_LINK	214.308	4.96694	0	9.99822	OK	353.71	8.04057	0.467513	15.6136	OK	OK	0.694942
ENSG0000011	-	-	ENSG0000011434	'GNAT1	-	3:50191611-50196516	-	-	IGV_LINK	0	0	0	0	OK	0	0	0	0	OK	NOTEST	0
ENSG0000011	-	-	ENSG0000011435	'GNAI2	-	3:50226291-50259355	-	-	IGV_LINK	4808.84	145.616	51.1075	240.124	OK	5189.12	159.55	41.6489	277.452	OK	OK	0.131845
ENSG0000011	-	-	ENSG0000011435	'TFG	-	3:100709330-100748966	-	-	IGV_LINK	1095.78	35.6148	14.8853	56.3444	OK	1721.11	58.3929	23.7427	93.0431	OK	OK	0.713315
ENSG0000011	-	-	ENSG0000011437	'USP9Y	-	Y:12701230-12860839	-	-	IGV_LINK	347.16	5.67124	0	12.036	OK	224.807	4.16849	0	9.00363	OK	OK	-0.44414
ENSG0000011	-	-	ENSG0000011437	'HYAL1	-	3:50299888-50312381	-	-	IGV_LINK	421.828	12.2149	4.1302	20.2997	OK	1128.16	35.2935	12.9008	57.6862	OK	OK	1.53076
ENSG0000011	-	-	ENSG0000011438	'TUSC2	-	3:50317789-50328251	-	-	IGV_LINK	321.947	12.2117	0	26.4077	OK	368.147	14.817	0	37.0416	OK	OK	0.278992
ENSG0000011	-	-	ENSG0000011438	'NPRL2	-	3:50347329-50504244	-	-	IGV_LINK	408.252	16.0447	0	33.8955	OK	339.273	13.9327	0	31.7043	OK	OK	-0.20363
ENSG0000011	-	-	ENSG0000011439	'RPL24	-	3:101681090-101686782	-	-	IGV_LINK	7236.05	899.858	337.947	1461.77	OK	5860.45	729.577	271.708	1187.45	OK	OK	-0.30264
ENSG0000011	-	-	ENSG0000011439	'CYB561D2	-	3:50347329-50504244	-	-	IGV_LINK	258.915	12.9082	0	29.4777	OK	312.461	16.2837	0	37.2283	OK	OK	0.335136
ENSG0000011	-	-	ENSG0000011440	'C3orf14	-	3:61561568-62369330	-	-	IGV_LINK	15.5155	1.57497	0	8.79764	OK	17.5308	2.08878	0	10.5149	OK	OK	0.407337
ENSG0000011	-	-	ENSG0000011441	'FXR1	-	3:180602129-180982753	-	-	IGV_LINK	1599.07	50.6031	3.05982	98.1463	OK	1788.14	54.9176	8.46733	101.368	OK	OK	0.118043
ENSG0000011	-	-	ENSG0000011442	'CBLB	-	3:105655460-105869552	-	-	IGV_LINK	676.865	12.6998	1.89762	23.502	OK	669.265	12.955	1.48454	24.4255	OK	OK	0.028705
ENSG0000011	-	-	ENSG0000011443	'BBX	-	3:107522935-107811324	-	-	IGV_LINK	917.356	8.50411	0	19.4455	OK	760.013	7.54237	0	18.2956	OK	OK	-0.17314
ENSG0000011	-	-	ENSG0000011444	'IFT57	-	3:108160811-108222570	-	-	IGV_LINK	828.141	31.9034	6.14384	57.6629	OK	849.729	33.0319	5.9959	60.068	OK	OK	0.050152
ENSG0000011	-	-	ENSG0000011445	'GNB4	-	3:179396088-179451590	-	-	IGV_LINK	575.044	10.9916	0	24.0871	OK	550.674	11.4345	0	22.9534	OK	OK	0.056995
ENSG0000011	-	-	ENSG0000011445	'HHLA2	-	3:108296489-108378285	-	-	IGV_LINK	792.262	21.8625	6.51412	37.2109	OK	1165.28	31.7657	9.87481	53.6566	OK	OK	0.539013
ENSG0000011	-	-	ENSG0000011447	'IQCG	-	3:197889074-197960142	-	-	IGV_LINK	247.279	9.53766	0	52.6171	OK	346.492	13.5208	0	56.0377	OK	OK	0.503473
ENSG0000011	-	-	ENSG0000011448	'GBE1	-	3:81489698-81762161	-	-	IGV_LINK	581.832	12.6882	4.40303	20.9733	OK	1562.31	31.9704	12.5017	51.4391	OK	OK	1.33325
ENSG0000011	-	-	ENSG0000011448	'MORC1	-	3:108958238-109118142	-	-	IGV_LINK	0	0	0	0	OK	0	0	0	0	OK	NOTEST	0
ENSG0000011	-	-	ENSG0000011449	'UMPS	-	3:124730365-124749273	-	-	IGV_LINK	413.101	12.5471	0.419087	24.6751	OK	546.549	20.9594	2.40528	39.5135	OK	OK	0.740244
ENSG0000011	-	-	ENSG0000011450	'NCBP2	-	3:196935401-196942597	-	-	IGV_LINK	780.625	25.1491	8.78272	41.5155	OK	943.571	31.3854	10.6474	52.1234	OK	OK	0.319587
ENSG0000011	-	-	ENSG0000011452	'SNX4	-	3:125446643-125520197	-	-	IGV_LINK	566.317	28.5237	6.82248	50.2249	OK	629.047	30.6961	9.13636	52.2558	OK	OK	0.105894
ENSG0000011	-	-	ENSG0000011452	'C3orf52	-	3:112086334-112133305	-	-	IGV_LINK	23.2733	0.471273	0	1.38282	OK	22.6869	0.5513	0	1.62404	OK	NOTEST	0.226274
ENSG0000011	-	-	ENSG0000011454	'FRMD4B	-	3:69169989-69542583	-	-	IGV_LINK	833.96	21.889	0	43.8612	OK	1134.35	25.4352	0.679097	50.1913	OK	OK	0.216618
ENSG0000011	-	-	ENSG0000011454	'SLC41A3	-	3:126006354-126101561	-	-	IGV_LINK	605.106	16.8047	5.9537	27.6556	OK	449.614	12.3277	3.38334	21.272	OK	OK	-0.44696
ENSG0000011	-	-	ENSG0000011454	'ROPN1B	-	3:125969143-125983454	-	-	IGV_LINK	0.969721	0.089666	0	0.581344	OK	3.09367	0.166605	0	0.757309	OK	NOTEST	0.893802
ENSG0000011	-	-	ENSG0000011455	'PLXNA1	-	3:126988593-127037392	-	-	IGV_LINK	1240.27	9.07232	0	18.6437	OK	1732.46	14.4312	0	33.6197	OK	OK	0.669649
ENSG0000011	-	-	ENSG0000011457	'ATP6V1A	-	3:113746871-113812056	-	-	IGV_LINK	1689.25	27.8621	6.78293	48.9414	OK	2128.45	32.3168	7.71971	56.9138	OK	OK	0.213977
ENSG0000011	-	-	ENSG0000011462	'ABTB1	-	3:127672934-127680920	-	-	IGV_LINK	1348.88	35.4008	9.26947	61.5322	OK	1322.03	35.7732	10.3173	61.2291	OK	OK	0.015097
ENSG0000011	-	-	ENSG0000011463	'PODXL2	-	3:127629180-127672809	-	-	IGV_LINK	11.6366	0.3257	0	0.728185	OK	11.3435	0.317675	0	0.711867	OK	NOTEST	-0.03599
ENSG0000011	-	-	ENSG0000011463	'UPK1B	-	3:119146149-119205153	-	-	IGV_LINK	0	0	0	0	OK	4.1249	0.131827	0	0.537679	OK	NOTEST	inf
ENSG0000011	-	-	ENSG0000011464	'CSPG5	-	3:47562238-47580792	-	-	IGV_LINK	3.87888	0.175549	0	0.581398	OK	2.06245	0.169149	0	0.575316	OK	NOTEST	-0.05358
ENSG0000011	-	-	ENSG0000011464	'KLHL18	-	3:47164496-47346816	-	-	IGV_LINK	246.309	4.69576	0	13.2015	OK	281.524	3.89668	0	8.44834	OK	OK	-0.26911
ENSG0000011	-	-	ENSG0000011465	'SCAP	-	3:47413693-47477126	-	-	IGV_LINK	740.866	14.9359	1.68329	28.1886	OK	866.229	17.9657	1.91687	34.0145	OK	OK	0.266455
ENSG0000011	-	-	ENSG0000011465	'EFCC1	-	3:128879595-129040742	-	-	IGV_LINK	27.1522	0.671732	0	2.92791	OK	14.4371	0.389468	0	2.29391	OK	OK	-0.78638
ENSG0000011	-	-	ENSG0000011465	'KIAA1257	-	3:128879595-129040742	-	-	IGV_LINK	10.8094	0.193199	0	1.34139	OK	17.5123	0.335692	0	1.93915	OK	NOTEST	0.797054
ENSG0000011	-	-	ENSG0000011467	'NEK11	-	3:130850594-131381475	-	-	IGV_LINK	393.707	9.91586	0	28.6646	OK	309.367	7.8063	0	22.6414	OK	OK	-0.3451
ENSG0000011	-	-	ENSG0000011468	'MRPL3	-	3:131462211-131502983	-	-	IGV_LINK	805.838	32.3494	12.6477	52.051	OK	933.259	38.5739	14.8025	62.3453	OK	OK	0.253888
ENSG0000011	-	-	ENSG0000011469	'PLSCR4	-	3:146192338-146251179	-	-	IGV_LINK	512.012	15.985	4.28025	27.6898	OK	376.397	12.8021	3.06382	22.5403	OK	OK	-0.32034
ENSG0000011	-	-	ENSG0000011473	'HEMK1	-	3:50558024-50596168	-	-	IGV_LINK	860.142	13.8318	0.953742	26.7098	OK	708.452	12.9274	0.490389	25.3645	OK	OK	-0.09755
ENSG0000011	-	-	ENSG0000011473	'CISH	-	3:50606489-50649297	-	-	IGV_LINK	407.283	9.75489	0	21.9213	OK	338.242	8.58041	0	17.7534	OK	OK	-0.18508
ENSG0000011	-	-	ENSG0000011473	'MAPKAPK3	-	3:50606489-50649297	-	-	IGV_LINK	896.022	23.0897	5.53067	40.6488	OK	767.231	21.2213	4.78835	37.6543	OK	OK	-0.12174
ENSG0000011	-	-	ENSG0000011473	'ACVR2B	-	3:38451026-38493142	-	-	IGV_LINK	284.128	1.39063	0.390853	2.39041	OK	157.777	0.772862	0.129753	1.41597	OK	OK	-0.84746
ENSG0000011	-	-	ENSG0000011474	'WDR48	-	3:39051997-39138903	-	-	IGV_LINK	716.623	12.388	0	26.8146	OK	579.548	10.2298	0	23.9958	OK	OK	-0.27617
ENSG0000011	-	-	ENSG0000011474	'COMMD2	-	3:149738469-149752499	-	-	IGV_LINK	459.648	17.128	0.144702	34.1112	OK	357.835	13.8657	0	30.1164	OK	OK	-0.30483
ENSG0000011	-	-	ENSG0000011474	'GORASP1	-	3:39051997-39138903	-	-	IGV_LINK	702.078	25.4369	0	60.5936	OK	773.419	28.5779	0	67.2923	OK	OK	0.167979
ENSG0000011	-	-	ENSG0000011475	'PEX5L	-	3:179794957-180037053	-	-	IGV_LINK	7.75776	0.052248	0	0.639438	OK	14.4371	0.437386	0	1.59695	OK	NOTEST	3.06546
ENSG0000011	-	-	ENSG0000011476	'RRP9	-	3:51933429-51941941	-	-	IGV_LINK	171.641	6.88228	1.63156	12.133	OK	146.434	5.87638	1.17125	10.5815	OK	OK	-0.22796
ENSG0000011	-	-	ENSG0000011477	'ABCC5	-	3:183919933-184018015	-	-	IGV_LINK	1554.46	18.8175	6.35514	31.2798	OK	2043.89	25.6635	6.94393	44.383	OK	OK	0.447642
ENSG0000011	-	-	ENSG0000011477	'AADAC	-	3:151733915-151928175	-	-	IGV_LINK	0	0	0	0	OK	2.06245	0.079158	0	0.501796	OK	NOTEST	inf
ENSG0000011	-	-	ENSG0000011477	'ABHD14B	-	3:51955313-51989202	-	-	IGV_LINK	1025.96	37.7169	7.18237	68.2514	OK	1226.13	44.8708	3.66979	86.0719	OK	OK	0.250567
ENSG0000011	-	-	ENSG0000011478	'EIF1B	-	3:39808913-40312424	-	-	IGV_LINK	350.069	24.7565	7.45859	42.0544	OK	342.367	23.703	7.1426	40.2635	OK	OK	-0.06273
ENSG0000011	-	-	ENSG0000011478	'ABHD14A-ACY1	-	3:51955313-51989202	-	-	IGV_LINK	73.4167	2.6173	0	14.004	OK	95.0207	5.16845	0	27.8737	OK	OK	0.981652
ENSG0000011	-	-	ENSG0000011479	'ARHGEF26	-	3:154024400-154257827	-	-	IGV_LINK	9.69721	0.194926	0	0.47222	OK	4.1249	0.094869	0	0.27514	OK	NOTEST	-1.03892
ENSG0000011	-	-	ENSG0000011479	'KLHL24	-	3:183635567-183684477	-	-	IGV_LINK	1119.06	18.232	0	37.095	OK	777.544	11.4126	0	23.0987	OK	OK	-0.67585
ENSG0000011	-	-	ENSG0000011480	'PLCH1	-	3:155375579-155745067	-	-	IGV_LINK	253.097	2.36721	0.636178	4.09824	OK	158.809	1.49207	0.312507	2.67164	OK	OK	-0.66587
ENSG0000011	-	-	ENSG0000011481	'VIPR1	-	3:42489298-42537573	-	-	IGV_LINK	32.9705	0.729175	0	1.89397	OK	28.8743	0.596594	0	1.58535	OK	OK	-0.28952
ENSG0000011	-	-	ENSG0000011484	'DNAH1	-	3:52316318-52400491	-	-	IGV_LINK	800.989	6.52817	0	13.8603	OK	949.758	8.7531	0	18.4757	OK	OK	0.423114
ENSG0000011	-	-	ENSG0000011485	'SSR3	-	3:156540139-156555184	-	-	IGV_LINK	1344.03	59.7037	13.1121	106.295	OK	1771.64	82.61	20.0503	145.17	OK	OK	0.468496
ENSG0000011	-	-	ENSG0000011485	'ZBTB47	-	3:42547968-42665854	-	-	IGV_LINK	133.821	2.4151	0	9.84916	OK	160.871	2.92635	0	9.77808	OK	OK	0.27702
ENSG0000011	-	-	ENSG0000011485	'TNNC1	-	3:52451101-52454070	-	-	IGV_LINK	6.78804	0.699893	0	1.71094	OK	3.09367	0.562959	0	1.63205	OK	OK	-0.31411
ENSG0000011	-	-	ENSG0000011485	'NKTR	-	3:42547968-42665854	-	-	IGV_LINK	2982.86	32.3341	0	70.3486	OK	1911.89	18.5587	0	40.4335	OK	OK	-0.80096
ENSG0000011	-	-	ENSG0000011485	'CLCN2	-	3:184335925-184361651	-	-	IGV_LINK	180.368	3.59158	0	7.76447	OK	197.995	5.03214	0	12.103	OK	OK	0.486554
ENSG0000011	-	-	ENSG0000011486	'FOXP1	-	3:70954692-71628558	-	-	IGV_LINK	266.673	5.51453	0	11.8765	OK	318.649	4.8307	0	10.5063	OK	OK	-0.19101
ENSG0000011	-	-	ENSG0000011486	'EIF4G1	-	3:184314494-184335358	-	-	IGV_LINK	4073.8	61.2468	9.93977	112.554	OK	6868.99	98.9621	9.19068	188.734	OK	OK	0.692241
ENSG0000011	-	-	ENSG0000011490	'SPCS1	-	3:52495337-52770949	-	-	IGV_LINK	626.439	51.9122	0	146.136	OK	688.858	57.7801	0	256.785	OK	OK	0.1545
ENSG0000011	-	-	ENSG0000011490	'NEK4	-	3:52495337-52770949	-	-	IGV_LINK	304.492	7.22518	0	40.7911	OK	294.93	7.307	0	82.2408	OK	OK	0.016245
ENSG0000011	-	-	ENSG0000011492	'SLC4A3	-	2:219627326-219641980	-	-	IGV_LINK	24.243	0.731432	0	2.15114	OK	42.2802	0.862024	0	2.16356	OK	OK	0.237005
ENSG0000011	-	-	ENSG0000011493	'INO80D	-	2:205989584-206086564	-	-	IGV_LINK	551.771	2.2269	0.802037	3.65176	OK	463.02	1.86886	0.640934	3.09679	OK	OK	-0.25288
ENSG0000011	-	-	ENSG0000011494	'EEF1B2	-	2:206114816-206162928	-	-	IGV_LINK	6873.79	551.666	166.716	936.615	OK	5138.59	414.057	108.807	719.307	OK	OK	-0.41397
ENSG0000011	-	-	ENSG0000011494	'ADAM23	-	2:206443538-206621130	-	-	IGV_LINK	7.75776	0.181723	0	0.755215	OK	8.2498	0.206277	0	0.897869	OK	NOTEST	0.182839
ENSG0000011	-	-	ENSG0000011495	'DGUOK	-	2:73926825-73981441	-	-	IGV_LINK	480.981	40.4636	13.1997	67.7274	OK	513.55	42.2141	14.4052	70.0231	OK	OK	0.061101
ENSG0000011	-	-	ENSG0000011497	'MOB1A	-	2:74152527-74178898	-	-	IGV_LINK	1614.58	27.6735	2.73589	52.6112	OK	1835.58	33.1945	3.26752	63.1215	OK	OK	0.262438
ENSG0000011	-	-	ENSG0000011498	'KANSL3	-	2:96593169-96704887	-	-	IGV_LINK	1645.62	21.1465	3.67377	38.6193	OK	1941.8	24.7874	5.20515	44.3697	OK	OK	0.229188
ENSG0000011	-	-	ENSG0000011498	'LMAN2L	-	2:96705928-96740064	-	-	IGV_LINK	642.925	17.2755	5.68843	28.8627	OK	785.793	20.6401	7.80887	33.4713	OK	OK	0.256718
ENSG0000011	-	-	ENSG0000011499	'RTKN	-	2:74425835-74442422	-	-	IGV_LINK	1082.21	45.5475	13.8006	77.2944	OK	1325.12	55.288	17.2493	93.3267	OK	OK	0.279593
ENSG0000011	-	-	ENSG0000011499	'TTL	-	2:112482153-112541739	-	-	IGV_LINK	520.74	7.98662	0.937978	15.0353	OK	467.145	6.32226	1.22846	11.4161	OK	OK	-0.33714
ENSG0000011	-	-	ENSG0000011500	'IL1A	-	2:112773914-112784590	-	-	IGV_LINK	2.90916	0.084012	0	0.225486	OK	1.03122	0.029798	0	0.111526	OK	NOTEST	-1.49539

ENSG0000011	-	-	ENSG0000011500	'CCL20	-	2:227813841-227817564	-	-	IGV_LINK	196.853	17.4423	3.40363	31.481	OK	2504.85	221.277	93.6353	348.918	OK	OK	3.66519
ENSG0000011	-	-	ENSG0000011502	'PIKFYVE	-	2:208266266-208358751	-	-	IGV_LINK	746.685	4.44105	1.70041	7.18168	OK	546.549	3.29235	1.12407	5.46063	OK	OK	-0.43178
ENSG0000011	-	-	ENSG0000011504	'KCNIP3	-	2:95297303-95386083	-	-	IGV_LINK	368.494	9.48122	2.15538	16.8071	OK	373.303	16.8811	1.60355	32.1587	OK	OK	0.832266
ENSG0000011	-	-	ENSG0000011504	'FAHD2A	-	2:95402720-95416616	-	-	IGV_LINK	363.645	12.9378	1.18444	24.6912	OK	312.461	12.0258	1.23104	22.8205	OK	OK	-0.10546
ENSG0000011	-	-	ENSG0000011505	'NCL	-	2:231453530-231483641	-	-	IGV_LINK	5680.62	204.007	23.3288	384.684	OK	5936.76	193.162	16.1078	370.215	OK	OK	-0.07881
ENSG0000011	-	-	ENSG0000011507	'ACTR1B	-	2:97655962-97664107	-	-	IGV_LINK	1305.24	40.861	14.891	66.8311	OK	1147.75	36.8163	11.4554	62.1772	OK	OK	-0.15038
ENSG0000011	-	-	ENSG0000011508	'SLC35F5	-	2:113705010-113756823	-	-	IGV_LINK	795.171	27.6496	6.58538	48.7139	OK	835.292	30.0281	7.12399	52.9321	OK	OK	0.119051
ENSG0000011	-	-	ENSG0000011508	'ZAP70	-	2:97713559-97739862	-	-	IGV_LINK	648.743	16.9878	4.09073	29.8849	OK	587.798	18.7489	3.6389	33.8588	OK	OK	0.142305
ENSG0000011	-	-	ENSG0000011509	'ACTR3	-	2:113889959-113962596	-	-	IGV_LINK	2996.44	104.418	27.9146	180.921	OK	2820.4	102.082	26.1543	178.01	OK	OK	-0.03263
ENSG0000011	-	-	ENSG0000011510	'STEAP3	-	2:119223830-119265652	-	-	IGV_LINK	473.224	6.92662	2.37062	11.4826	OK	8173.49	118.599	35.6471	201.55	OK	OK	4.09779
ENSG0000011	-	-	ENSG0000011510	'EPB41L5	-	2:120013004-120217279	-	-	IGV_LINK	519.77	5.56466	0.630328	10.499	OK	325.867	4.22071	0.392689	8.04873	OK	OK	-0.39881
ENSG0000011	-	-	ENSG0000011511	'TFCP2L1	-	2:121216586-121285207	-	-	IGV_LINK	48.486	0.400944	0	1.28005	OK	177.371	1.90119	0	5.36093	OK	OK	2.24543
ENSG0000011	-	-	ENSG0000011512	'SF3B6	-	2:24067583-24076443	-	-	IGV_LINK	892.143	84.234	35.5766	132.891	OK	901.291	85.2381	35.994	134.482	OK	OK	0.017097
ENSG0000011	-	-	ENSG0000011512	'TP53I3	-	2:24076525-24201698	-	-	IGV_LINK	352.009	17.6105	1.71786	33.5032	OK	711.545	36.245	9.9777	62.5124	OK	OK	1.04134
ENSG0000011	-	-	ENSG0000011513	'DNAJC27	-	2:24943635-25039694	-	-	IGV_LINK	234.672	3.22337	0.102806	6.34394	OK	120.653	2.01564	0	5.04398	OK	OK	-0.67734
ENSG0000011	-	-	ENSG0000011513	'POMC	-	2:25160852-25168903	-	-	IGV_LINK	16.4852	0.902317	0	1.96872	OK	8.2498	0.451312	0	1.07657	OK	OK	-0.99951
ENSG0000011	-	-	ENSG0000011514	'STAM2	-	2:152116800-152175992	-	-	IGV_LINK	528.498	10.6357	2.08655	19.1848	OK	481.582	10.4422	0.687715	20.1966	OK	OK	-0.02649
ENSG0000011	-	-	ENSG0000011515	'OTOF	-	2:26457202-26558698	-	-	IGV_LINK	29.0916	0.35769	0	0.813974	OK	28.8743	0.314169	0	0.809628	OK	NOTEST	-0.18717
ENSG0000011	-	-	ENSG0000011515	'GPD2	-	2:156435289-156613735	-	-	IGV_LINK	517.831	9.93008	0	21.6426	OK	352.679	6.19884	0	14.5129	OK	OK	-0.67981
ENSG0000011	-	-	ENSG0000011516	'CENPA	-	2:26764283-26801067	-	-	IGV_LINK	42.6677	2.24676	0	8.41389	OK	29.9055	1.53092	0	7.3884	OK	OK	-0.55345
ENSG0000011	-	-	ENSG0000011516	'CYTIP	-	2:157414618-157488961	-	-	IGV_LINK	422.798	13.0397	3.74879	22.3306	OK	407.334	11.928	3.39173	20.4642	OK	OK	-0.12856
ENSG0000011	-	-	ENSG0000011517	'ACVR1	-	2:157736443-157875862	-	-	IGV_LINK	367.524	8.1164	2.57391	13.6589	OK	402.178	9.01709	2.848	15.1862	OK	OK	0.151823
ENSG0000011	-	-	ENSG0000011518	'TANC1	-	2:158968670-159232659	-	-	IGV_LINK	740.866	6.13427	2.33729	9.93125	OK	626.985	5.22193	1.83257	8.6113	OK	OK	-0.23231
ENSG0000011	-	-	ENSG0000011519	'SLC30A3	-	2:27253683-27281499	-	-	IGV_LINK	6.78804	0.138595	0	0.618813	OK	0	0	0	0	OK	NOTEST	'-inf
ENSG0000011	-	-	ENSG0000011520	'MPV17	-	2:27309491-27325680	-	-	IGV_LINK	1120.03	83.8459	32.5641	135.128	OK	1255	90.5756	36.7205	144.431	OK	OK	0.111384
ENSG0000011	-	-	ENSG0000011520	'GTF3C2	-	2:27325848-27370486	-	-	IGV_LINK	985.236	28.2091	0	66.697	OK	1336.47	36.4475	0	85.0282	OK	OK	0.369657
ENSG0000011	-	-	ENSG0000011521	'EIF2B4	-	2:27325848-27370486	-	-	IGV_LINK	677.835	26.259	0	58.0324	OK	771.356	28.9809	0	64.1633	OK	OK	0.142291
ENSG0000011	-	-	ENSG0000011521	'NRBP1	-	2:27427789-27442259	-	-	IGV_LINK	1775.56	51.5508	19.0739	84.0277	OK	1831.46	51.9653	19.7582	84.1725	OK	OK	0.011554
ENSG0000011	-	-	ENSG0000011522	'ITGB6	-	2:160099665-160271888	-	-	IGV_LINK	53.3346	2.70119	0	7.83062	OK	129.934	7.81301	0	18.7567	OK	OK	1.53228
ENSG0000011	-	-	ENSG0000011522	'FNDC4	-	2:27491882-27495245	-	-	IGV_LINK	4.8486	0.242068	0	0.675081	OK	17.5308	0.667613	0	1.46691	OK	OK	1.4636
ENSG0000011	-	-	ENSG0000011523	'ITGA4	-	2:181457201-181536187	-	-	IGV_LINK	1117.12	20.4272	4.47036	36.384	OK	861.073	14.9232	4.2999	25.5466	OK	OK	-0.45293
ENSG0000011	-	-	ENSG0000011523	'PSMD14	-	2:161096230-161411717	-	-	IGV_LINK	573.105	18.129	2.09938	34.1586	OK	749.701	25.1135	1.75141	48.4756	OK	OK	0.470164
ENSG0000011	-	-	ENSG0000011523	'SNX17	-	2:27370495-27380790	-	-	IGV_LINK	1841.5	58.1617	20.0686	96.2547	OK	1878.89	61.5756	17.6142	105.537	OK	OK	0.082291
ENSG0000011	-	-	ENSG0000011523	'ASB3	-	2:53532671-53860160	-	-	IGV_LINK	247.279	7.83728	0	17.7361	OK	279.431	9.41485	0	23.669	OK	OK	0.264585
ENSG0000011	-	-	ENSG0000011524	'PPM1G	-	2:27381193-27409687	-	-	IGV_LINK	1039.54	27.1369	11.6226	42.6512	OK	1270.47	33.1776	14.5786	51.7765	OK	OK	0.289951
ENSG0000011	-	-	ENSG0000011525	'PDE1A	-	2:182140035-182523192	-	-	IGV_LINK	103.76	2.3453	0.047694	4.64291	OK	45.3739	0.960287	0	2.19272	OK	OK	-1.28824
ENSG0000011	-	-	ENSG0000011525	'REEP6	-	19:1481427-1497927	-	-	IGV_LINK	22.3036	0.775175	0	2.32432	OK	26.8118	0.932525	0	2.67298	OK	OK	0.26662
ENSG0000011	-	-	ENSG0000011525	'PCSK4	-	19:1481427-1497927	-	-	IGV_LINK	96.9721	3.943	0	9.16439	OK	109.31	4.46954	0	10.3298	OK	OK	0.18083
ENSG0000011	-	-	ENSG0000011526	'GCG	-	2:162142872-162152404	-	-	IGV_LINK	0	0	0	0	OK	0	0	0	0	OK	NOTEST	0
ENSG0000011	-	-	ENSG0000011526	'APC2	-	19:1446301-1479556	-	-	IGV_LINK	13.5761	0.108867	0	0.726415	OK	20.6245	0.19073	0	1.11377	OK	NOTEST	0.808966
ENSG0000011	-	-	ENSG0000011526	'IFIH1	-	2:162267078-162318703	-	-	IGV_LINK	827.172	13.7416	5.75666	21.7266	OK	897.166	15.1254	5.96059	24.2902	OK	OK	0.138422
ENSG0000011	-	-	ENSG0000011526	'RPS15	-	19:1438357-1440494	-	-	IGV_LINK	4947.51	819.24	335.379	1303.1	OK	5122.09	851.501	342.891	1360.11	OK	OK	0.055722
ENSG0000011	-	-	ENSG0000011527	'GCA	-	2:162318839-162838730	-	-	IGV_LINK	317.099	20.8294	3.22206	38.4367	OK	408.365	27.1429	5.95811	48.3276	OK	OK	0.381954
ENSG0000011	-	-	ENSG0000011527	'INO80B	-	2:74455022-74460891	-	-	IGV_LINK	293.067	17.3559	0	41.5295	OK	282.137	17.2992	0	40.3057	OK	OK	-0.00472
ENSG0000011	-	-	ENSG0000011527	'MOGS	-	2:74461056-74465410	-	-	IGV_LINK	1426.46	33.3017	12.6268	53.9766	OK	1525.18	38.4318	14.1045	62.7592	OK	OK	0.206705
ENSG0000011	-	-	ENSG0000011528	'TTC31	-	2:74471957-74494886	-	-	IGV_LINK	978.448	23.9493	0.748988	47.1497	OK	958.008	24.2544	0	50.3416	OK	OK	0.018259
ENSG0000011	-	-	ENSG0000011528	'NDUFS7	-	19:1383526-1396467	-	-	IGV_LINK	790.322	72.1154	24.0872	120.144	OK	828.074	77.2236	24.4195	130.028	OK	OK	0.098736
ENSG0000011	-	-	ENSG0000011528	'PCGF1	-	2:74505042-74508580	-	-	IGV_LINK	306.432	23.6745	1.238	46.111	OK	251.619	20.5137	0	43.8606	OK	OK	-0.20675
ENSG0000011	-	-	ENSG0000011529	'GRB14	-	2:164492811-164621848	-	-	IGV_LINK	173.58	5.17282	0.896858	9.44879	OK	38.1553	1.00947	0	2.2935	OK	OK	-2.35735
ENSG0000011	-	-	ENSG0000011529	'CLIP4	-	2:29097704-29189643	-	-	IGV_LINK	237.582	5.07584	0	11.318	OK	283.587	6.47661	0	14.9447	OK	OK	0.351594
ENSG0000011	-	-	ENSG0000011529	'TLX2	-	2:74513462-74517147	-	-	IGV_LINK	0.969721	0.027833	0	0.33148	OK	1.03122	0.186042	0	0.727069	OK	NOTEST	2.74077
ENSG0000011	-	-	ENSG0000011530	'SPTBN1	-	2:54456284-54680045	-	-	IGV_LINK	8665.42	65.7856	20.368	111.203	OK	5975.95	45.4277	15.5725	75.283	OK	OK	-0.5342
ENSG0000011	-	-	ENSG0000011530	'AUP1	-	2:74526644-74557554	-	-	IGV_LINK	2125.63	95.6031	16.3723	174.834	OK	1974.8	87.4579	18.958	155.958	OK	OK	-0.12847
ENSG0000011	-	-	ENSG0000011531	'RTN4	-	2:54972186-55112621	-	-	IGV_LINK	3952.58	129.829	40.7309	218.927	OK	4411.58	147.801	45.4345	250.168	OK	OK	0.187045
ENSG0000011	-	-	ENSG0000011531	'HTRA2	-	2:74526644-74557554	-	-	IGV_LINK	515.891	25.5113	0	68.6108	OK	334.117	15.271	0	44.3374	OK	OK	-0.74035
ENSG0000011	-	-	ENSG0000011531	'LOXL3	-	2:74526644-74557554	-	-	IGV_LINK	425.707	9.06173	0	24.0555	OK	380.522	7.45675	0	19.59	OK	OK	-0.28124
ENSG0000011	-	-	ENSG0000011532	'DOK1	-	2:74526644-74557554	-	-	IGV_LINK	464.496	19.0457	0	55.5226	OK	412.49	16.6056	0	46.2002	OK	OK	-0.19779
ENSG0000011	-	-	ENSG0000011533	'GALNT3	-	2:165747590-165794682	-	-	IGV_LINK	164.852	4.68922	0	9.96461	OK	102.091	3.06353	0	6.93303	OK	OK	-0.61415
ENSG0000011	-	-	ENSG0000011535	'POLE4	-	2:74958491-74970128	-	-	IGV_LINK	275.401	38.3824	8.6715	68.0933	OK	476.426	68.0205	19.7664	116.275	OK	OK	0.825525
ENSG0000011	-	-	ENSG0000011535	'TACR1	-	2:75046462-75199700	-	-	IGV_LINK	2.90916	0.399691	0	1.85368	OK	19.5933	0.384905	0	1.87924	OK	NOTEST	-0.05438
ENSG0000011	-	-	ENSG0000011535	'CCDC88A	-	2:55282318-55419921	-	-	IGV_LINK	1045.36	12.2371	2.28645	22.1876	OK	710.514	9.0557	1.51328	16.5981	OK	OK	-0.43436
ENSG0000011	-	-	ENSG0000011536	'ACADL	-	2:210171517-210233976	-	-	IGV_LINK	925.113	27.7547	9.68458	45.8248	OK	478.488	12.7375	4.23359	21.2415	OK	OK	-1.12365
ENSG0000011	-	-	ENSG0000011536	'EVA1A	-	2:75469301-75569722	-	-	IGV_LINK	224.975	12.3863	1.20385	23.5687	OK	366.085	20.5979	3.43714	37.7586	OK	OK	0.733755
ENSG0000011	-	-	ENSG0000011536	'MRPL19	-	2:75646782-75710989	-	-	IGV_LINK	290.916	12.4755	0	26.8502	OK	357.835	15.4399	0	32.819	OK	OK	0.307568
ENSG0000011	-	-	ENSG0000011536	'LANCL1	-	2:210324758-210477652	-	-	IGV_LINK	899.901	16.6593	4.15455	29.164	OK	1127.13	21.7502	5.93767	37.5626	OK	OK	0.384698
ENSG0000011	-	-	ENSG0000011536	'WDR75	-	2:189441432-189475565	-	-	IGV_LINK	794.201	17.3617	6.65824	28.0651	OK	588.829	13.4359	4.54652	22.3254	OK	OK	-0.36981
ENSG0000011	-	-	ENSG0000011538	'EFEMP1	-	2:55865966-55924139	-	-	IGV_LINK	1594.22	51.2508	19.0297	83.4719	OK	998.226	31.6384	12.2182	51.0587	OK	OK	-0.6959
ENSG0000011	-	-	ENSG0000011538	'REG1A	-	2:79120361-79123419	-	-	IGV_LINK	583.772	51.4876	20.5319	82.4432	OK	15032.2	1334.95	341.666	2328.24	OK	OK	4.69642
ENSG0000011	-	-	ENSG0000011539	'FANCL	-	2:57907650-58241686	-	-	IGV_LINK	417.95	18.9825	2.41075	35.5542	OK	334.117	14.8105	1.69876	27.9221	OK	OK	-0.35805
ENSG0000011	-	-	ENSG0000011541	'FN1	-	2:215360439-215436172	-	-	IGV_LINK	15500	190.714	0	442.827	OK	15350.8	189.88	0	428.67	OK	OK	-0.00633
ENSG0000011	-	-	ENSG0000011541	'STAT1	-	2:190880796-191020960	-	-	IGV_LINK	10273.2	215.706	0	447.578	OK	5646.99	136.168	0	300.832	OK	OK	-0.66368
ENSG0000011	-	-	ENSG0000011541	'GLS	-	2:190880796-191020960	-	-	IGV_LINK	2471.82	62.992	0	218.658	OK	3358.7	80.5718	0	221.721	OK	OK	0.355108
ENSG0000011	-	-	ENSG0000011542	'PAPOLG	-	2:60756229-60802085	-	-	IGV_LINK	257.946	5.1539	0	11.6078	OK	250.588	3.16202	0.721948	5.60208	OK	OK	-0.70482
ENSG0000011	-	-	ENSG0000011542	'DNAH6	-	2:84516454-84819589	-	-	IGV_LINK	232.733	1.03916	0.233741	1.84457	OK	95.9039	0.428221	0	0.927306	OK	OK	-1.27899
ENSG0000011	-	-	ENSG0000011542	'PECR	-	2:215942804-216102783	-	-	IGV_LINK	488.933	23.3256	3.44678	43.2043	OK	569.485	24.419	5.90044	42.9375	OK	OK	0.066091
ENSG0000011	-	-	ENSG0000011544	'UNC50	-	2:98608578-98618515	-	-	IGV_LINK	436.374	23.0438	6.70258	39.385	OK	537.268	28.0531	9.77493	46.3312	OK	OK	0.28378
ENSG0000011	-	-	ENSG0000011545	'IGFBP2	-	2:216632827-216664436	-	-	IGV_LINK	18.4247	1.0554	0	2.44272	OK	84.5604	5.38854	0	10.9598	OK	OK	2.3521
ENSG0000011	-	-	ENSG0000011545	'ELMOD3	-	2:85342087-85391752	-	-	IGV_LINK	808.747	25.8696	0	61.2023	OK	680.608	21.2323	0	58.3097	OK	OK	-0.285
ENSG0000011	-	-	ENSG0000011546	'IGFBP5	-	2:216672104-216994079	-	-	IGV_LINK	7638.49	71.5805	25.0877	118.073	OK	2510	23.5306	9.92496	37.1362	OK	OK	-1.60503
ENSG0000011	-	-	ENSG0000011546	'USP34	-	2:61177417-61470769	-	-	IGV_LINK	2846.13	63.4568	0	153.946	OK	2105.76	50.0684	0	118.332	OK	OK	-0.34188
ENSG0000011	-	-	ENSG0000011546	'EFHD1	-	2:232547967-232682781	-	-	IGV_LINK	386.918	14.7734	0	32.9162	OK	82.498	3.26924	0	11.4029	OK	OK	-2.17597
ENSG0000011	-	-	ENSG0000011547	'KCNJ13	-	2:232697298-233015885	-	-	IGV_LINK	0	0	0	0	OK	1.03122	0.062805	0	1.66644	OK	NOTEST	inf
ENSG0000011	-	-	ENSG0000011548	'CCT4	-	2:61868088-62147247	-	-	IGV_LINK	2112.05	53.3721	13.9611	92.7831	OK	1989.23	49.784	12.7309	86.8372	OK	OK	-0.1004
ENSG0000011	-	-	ENSG0000011548	'GGCX	-	2:85539164-85561547	-	-	IGV_LINK	1972.41	26.5487	0	83.3169	OK	2488.35	39.0178	0	116.531	OK	OK	0.555493
ENSG0000011	-	-	ENSG0000011548	'NEU2	-	2:233032671-233035057	-	-	IGV_LINK	0	0	0	0	OK	0	0	0	0	OK	NOTEST	0
ENSG0000011	-	-	ENSG0000011550	'EHBP1	-	2:62673850-63048640	-	-	IGV_LINK	786.443	15.0763	0	30.2045	OK	1066.29	22.2335	1.30564	43.1613	OK	OK	0.560448
ENSG0000011	-	-	ENSG0000011550	'OTX1	-	2:63050056-63057836	-	-	IGV_LINK	0	0	0	0	OK	2.06245	0.076451	0	0.400451	OK	NOTEST	inf
ENSG0000011	-	-	ENSG0000011551	'TXNDC9	-	2:98997260-99490035	-	-	IGV_LINK	281.219	18.5272	0	68.3457	OK	349.585	24.5387	0	80.7609	OK	OK	0.405415
ENSG0000011	-	-	ENSG0000011552	'COQ10B	-	2:197453422-197475308	-	-	IGV_LINK	210.429	6.75361	1.2723	12.2349	OK	290.805	8.45609	2.43624	14.4759	OK	OK	0.324332
ENSG0000011	-	-	ENSG0000011552	'GNLY	-	2:85685174-85698854	-	-	IGV_LINK	568.256	38.2469	12.0642	64.4296	OK	353.71	22.9667	6.5135	39.4198	OK	OK	-0.7358
ENSG0000011	-	-	ENSG0000011552	'SF3B1	-	2:197389783-197435091	-	-	IGV_LINK	8703.24	184.261	0	402.117	OK	6323.47	128.064	0	273.73	OK	OK	-0.52489
ENSG0000011	-	-	ENSG0000011552	'ST3GAL5	-	2:85837119-85905199	-	-	IGV_LINK	587.651	15.3069	2.70326	27.9106	OK	395.99	10.7333	2.25478	19.2118	OK	OK	-0.5121
ENSG0000011	-	-	ENSG0000011552	'CHST10	-	2:100391859-100417656	-	-	IGV_LINK	126.064	2.66135	0.310849	5.01184	OK	172.215	4.49249	0	9.1275	OK	OK	0.755359
ENSG0000011	-	-	ENSG0000011553	'PDCL3	-	2:100562689-100576735	-	-	IGV_LINK	231.763	11.8528	3.15826	20.5474	OK	242.338	11.8933	3.31792	20.4688	OK	OK	0.004923
ENSG0000011	-	-	ENSG0000011554	'MOB4	-	2:197486580-197553699	-	-	IGV_LINK	251.668	5.81498	0	27.2383	OK	286.494	8.96078	0	46.8569	OK	OK	0.62385
ENSG0000011	-	-	ENSG0000011554	'HSPE1	-	2:197486580-197553699	-	-	IGV_LINK	979.747	95.4198	0	242.393	OK	1060.89	106.741	0	288.174	OK	OK	0.161752
ENSG0000011	-	-	ENSG0000011554	'KDM3A	-	2:86440646-86492716	-	-	IGV_LINK	2716.19	39.5925	0.013724	79.1712	OK	2338.82	34.7209	0.064449	69.3773	OK	OK	-0.18942
ENSG0000011	-	-	ENSG0000011555	'PLCD4	-	2:218607764-218637184	-	-	IGV_LINK	72.729	2.17104	0	5.72827	OK	154.684	2.44427	0.29274	4.59579	OK	OK	0.171015
ENSG0000011	-	-	ENSG0000011556	'CHMP3	-	2:86503430-86778041	-	-	IGV_LINK	1546.95	60.8301	0	125.93	OK	1646.37	61.4102	0	127.165	OK	OK	0.013693
ENSG0000011	-	-	ENSG0000011556	'ZNF142	-	2:218637915-218663443	-	-	IGV_LINK	507.164	7.40414	0	17.4165	OK	511.488	7.2901	0	18.2897	OK	OK	-0.02239
ENSG0000011	-	-	ENSG0000011559	'IL1R2	-	2:101991843-102028544	-	-	IGV_LINK	35.8797	1.74443	0	3.79705	OK	1597.37	74.316	31.8827	116.749	OK	OK	5.41285
ENSG0000011	-	-	ENSG0000011559	'PRKAG3	-	2:218822382-218832086	-	-	IGV_LINK	0	0	0	0	OK	0	0	0	0	OK	NOTEST	0
ENSG0000011	-	-	ENSG0000011559	'SMYD1	-	2:88067779-88113387	-	-	IGV_LINK	0	0	0	0	OK	0	0	0	0	OK	NOTEST	0
ENSG0000011	-	-	ENSG0000011559	'IL1R1	-	2:102064543-102182108	-	-	IGV_LINK	722.442	9.71429	1.53672	17.8919	OK	1406.59	20.5608	4.1538	36.9678	OK	OK	1.08172
ENSG0000011	-	-	ENSG0000011559	'WNT6	-	2:218859820-218874233	-	-	IGV_LINK	38.7888	1.42829	0	2.9717	OK	5.15612	0.190003	0	0.579414	OK	OK	-2.9102
ENSG0000011	-	-	ENSG0000011559	'IL1RL2	-	2:102186972-102240002	-	-	IGV_LINK	91.1537	2.50877	0	5.27175	OK	59.811	1.64523	0	3.37548	OK	OK	-0.60869
ENSG0000011	-	-	ENSG0000011560	'IL1RL1	-	2:102311501-102398775	-	-	IGV_LINK	8.72748	0.175683	0	0.980295	OK	10.3122	0.300584	0	2.00693	OK	NOTEST	0.774796
ENSG0000011	-	-	ENSG0000011560	'IL18R1	-	2:102311501-102398775	-	-	IGV_LINK	256.006	4.98289	0	10.2069	OK	459.926	8.66829	1.11708	16.2195	OK	OK	0.798765
ENSG0000011	-	-	ENSG0000011560	'IL18RAP	-	2:102418688-102452565	-	-	IGV_LINK	92.1234	3.03168	0	6.91861	OK	224.807	6.91678	0.418108	13.4154	OK	OK	1.18998
ENSG0000011	-	-	ENSG0000011561	'SLC9A2	-	2:102619706-102711318	-	-	IGV_LINK	0	0	0	0	OK	0	0	0	0	OK	NOTEST	0
ENSG0000011	-	-	ENSG0000011564	'FHL2	-	2:105357711-105438513	-	-	IGV_LINK	76.6079	2.85376	0	6.13817	OK	1984.08	83.1922	35.787	130.597	OK	OK	4.86551
ENSG0000011	-	-	ENSG0000011564	'MLPH	-	2:237485427-237555318	-	-	IGV_LINK	101.821	5.06672	0	10.8917	OK	14.4371	1.324	0	3.20619	OK	OK	-1.93615
ENSG0000011	-	-	ENSG0000011564	'CNPPD1	-	2:219171896-219185479	-	-	IGV_LINK	2163.45	66.4784	0	134.585	OK	1982.01	61.0363	0	122.532	OK	OK	-0.12322
ENSG0000011	-	-	ENSG0000011565	'UXS1	-	2:106093302-106194339	-	-	IGV_LINK	541.104	19.0488	3.87558	34.2219	OK	498.082	17.2389	4.79061	29.6871	OK	OK	-0.14403
ENSG0000011	-	-	ENSG0000011565	'ABCB6	-	2:219209767-219229717	-	-	IGV_LINK	823.202	23.5786	0	48.9303	OK	1345.33	36.4158	0.000789	72.8307	OK	OK	0.627083
ENSG0000011	-	-	ENSG0000011566	'STK16	-	2:219229756-219278170	-	-	IGV_LINK	672.986	36.6167	0	105.646	OK	786.825	45.282	0	144.845	OK	OK	0.306435
ENSG0000011	-	-	ENSG0000011566	'SLC5A7	-	2:107986522-108013994	-	-	IGV_LINK	0	0	0	0	OK	0	0	0	0	OK	NOTEST	0
ENSG0000011	-	-	ENSG0000011567	'HDLBP	-	2:241227263-241354027	-	-	IGV_LINK	6936.41	118.721	0	292.97	OK	9095.4	159.603	0	393.162	OK	OK	0.426912
ENSG0000011	-	-	ENSG0000011568	'PPP1R7	-	2:241106098-241183652	-	-	IGV_LINK	1050.21	54.9388	16.0123	93.8654	OK	1092.07	59.1642	16.8304	101.498	OK	OK	0.106899
ENSG0000011	-	-	ENSG0000011568	'PASK	-	2:241106098-241183652	-	-	IGV_LINK	371.403	7.27851	0	19.8502	OK	341.335	6.69269	0	18.81	OK	OK	-0.12106
ENSG0000011	-	-	ENSG0000011569	'STK25	-	2:241356242-241509730	-	-	IGV_LINK	1839.56	78.1225	10.4793	145.766	OK	1709.77	80.0944	14.334	145.855	OK	OK	0.035964
ENSG0000011	-	-	ENSG0000011570	'TPO	-	2:1374222-1543711	-	-	IGV_LINK	0.969721	0.055568	0	0.572288	OK	0	0	0	0	OK	NOTEST	'-inf
ENSG0000011	-	-	ENSG0000011571	'PROC	-	2:127418426-127429246	-	-	IGV_LINK	37.8191	2.33439	0	5.25729	OK	4.1249	0.345712	0	1.16323	OK	OK	-2.7554
ENSG0000011	-	-	ENSG0000011573	'ID2	-	2:8666635-8684453	-	-	IGV_LINK	1239.3	89.007	31.4905	146.524	OK	2034.61	150.119	54.7779	245.461	OK	OK	0.754118
ENSG0000011	-	-	ENSG0000011575	'TAF1B	-	2:9843353-9934416	-	-	IGV_LINK	216.248	7.63448	1.02331	14.2457	OK	230.994	6.57818	1.18832	11.968	OK	OK	-0.21484
ENSG0000011	-	-	ENSG0000011575	'HPCAL1	-	2:10302888-10427617	-	-	IGV_LINK	2313.75	81.4817	29.9657	132.998	OK	2866.81	104.621	37.943	171.299	OK	OK	0.360622
ENSG0000011	-	-	ENSG0000011575	'ODC1	-	2:10439967-10448504	-	-	IGV_LINK	1022.09	29.5365	12.0926	46.9804	OK	915.728	26.4674	10.5883	42.3466	OK	OK	-0.15828
ENSG0000011	-	-	ENSG0000011576	'BIRC6	-	2:32357027-32618899	-	-	IGV_LINK	1665.01	19.0767	0	39.9353	OK	1429.28	20.5321	0	42.282	OK	OK	0.106068
ENSG0000011	-	-	ENSG0000011576	'NOL10	-	2:10570765-10689987	-	-	IGV_LINK	427.647	9.88498	3.04498	16.725	OK	395.99	9.45332	2.8591	16.0475	OK	OK	-0.06442
ENSG0000011	-	-	ENSG0000011576	'PLEKHB2	-	2:131104846-131355769	-	-	IGV_LINK	2081.02	30.4241	12.0001	48.8482	OK	3138.02	45.4761	19.0922	71.86	OK	OK	0.579892
ENSG0000011	-	-	ENSG0000011580	'GORASP2	-	2:170928463-170967129	-	-	IGV_LINK	877.597	23.3398	8.9069	37.7726	OK	1355.03	40.4245	14.1812	66.6678	OK	OK	0.79244
ENSG0000011	-	-	ENSG0000011580	'STRN	-	2:36843639-36966472	-	-	IGV_LINK	458.678	4.72669	0.429847	9.02353	OK	373.303	3.24301	0.751475	5.73454	OK	OK	-0.5435

ENSG0000011	-	-	ENSG0000011581	'CEBPZ	-	2:37167819-37324808	-	-	IGV_LINK	704.987	13.1982	0	28.933	OK	713.608	13.3334	0	29.1017	OK	OK	0.014704
ENSG0000011	-	-	ENSG0000011582	'PRKD3	-	2:37167819-37324808	-	-	IGV_LINK	675.895	9.31307	0	27.2226	OK	487.769	5.55867	0	15.2197	OK	OK	-0.74452
ENSG0000011	-	-	ENSG0000011582	'DCAF17	-	2:171317404-171485052	-	-	IGV_LINK	296.734	4.54398	0	10.5625	OK	237.182	3.37163	0	7.62487	OK	OK	-0.43051
ENSG0000011	-	-	ENSG0000011582	'QPCT	-	2:37344573-37373322	-	-	IGV_LINK	257.946	10.3616	2.6521	18.0712	OK	195.933	8.66437	0.41004	16.9187	OK	OK	-0.25809
ENSG0000011	-	-	ENSG0000011583	'RAB3GAP1	-	2:135052264-135531236	-	-	IGV_LINK	1209.24	16.4724	5.8152	27.1296	OK	1249.84	17.3613	6.20546	28.5171	OK	OK	0.075822
ENSG0000011	-	-	ENSG0000011584	'SLC25A12	-	2:171784369-172082430	-	-	IGV_LINK	470.314	11.6258	0	24.2012	OK	383.616	9.35157	0	20.1634	OK	OK	-0.31405
ENSG0000011	-	-	ENSG0000011584	'RMDN2	-	2:37923186-38239590	-	-	IGV_LINK	101.821	3.90012	0	8.85343	OK	124.778	5.52977	0	17.6669	OK	OK	0.503701
ENSG0000011	-	-	ENSG0000011584	'DLX2	-	2:172099438-172102900	-	-	IGV_LINK	4.8486	0.136287	0	0.343169	OK	0	0	0	0	OK	NOTEST	'-inf
ENSG0000011	-	-	ENSG0000011585	'LCT	-	2:135787839-135837180	-	-	IGV_LINK	0.969721	0.008949	0	0.05432	OK	0	0	0	0	OK	NOTEST	'-inf
ENSG0000011	-	-	ENSG0000011586	'DARS	-	2:135906676-136022593	-	-	IGV_LINK	2681.28	110.875	27.5974	194.152	OK	2148.04	91.2519	22.4277	160.076	OK	OK	-0.28101
ENSG0000011	-	-	ENSG0000011587	'SRSF7	-	2:38743598-38751494	-	-	IGV_LINK	2084.9	79.2837	29.5832	128.984	OK	1610.77	64.4487	20.0528	108.845	OK	OK	-0.29887
ENSG0000011	-	-	ENSG0000011588	'SDC1	-	2:20200796-20225433	-	-	IGV_LINK	642.925	13.9725	3.36015	24.5849	OK	3562.88	75.4165	21.9605	128.872	OK	OK	2.43229
ENSG0000011	-	-	ENSG0000011589	'PLCL1	-	2:197804701-198772356	-	-	IGV_LINK	150.307	1.93536	0.233867	3.63685	OK	108.279	1.2016	0.00324	2.39996	OK	OK	-0.68764
ENSG0000011	-	-	ENSG0000011590	'SLC1A4	-	2:64988476-65023865	-	-	IGV_LINK	501.345	6.71798	2.16302	11.2729	OK	404.24	5.43837	1.44678	9.42996	OK	OK	-0.30485
ENSG0000011	-	-	ENSG0000011590	'SOS1	-	2:38981395-39124345	-	-	IGV_LINK	1009.48	12.708	0	31.6845	OK	847.667	10.8869	0	27.4506	OK	OK	-0.22313
ENSG0000011	-	-	ENSG0000011591	'KYNU	-	2:142877497-143055832	-	-	IGV_LINK	257.946	8.51881	1.1065	15.9311	OK	571.299	20.5924	6.2795	34.9053	OK	OK	1.27339
ENSG0000011	-	-	ENSG0000011593	'AC087235.1	-	12:22699858-23251499	-	-	IGV_LINK	0	0	0	0	OK	0	0	0	0	OK	NOTEST	0
ENSG0000011	-	-	ENSG0000011593	'WIPF1	-	2:174547140-174787935	-	-	IGV_LINK	2754.98	60.9416	6.91705	114.966	OK	2546.09	54.0948	7.5547	100.635	OK	OK	-0.17194
ENSG0000011	-	-	ENSG0000011594	'ORC2	-	2:200908972-201085750	-	-	IGV_LINK	511.043	11.2757	0	26.2151	OK	433.114	9.9103	0	25.3038	OK	OK	-0.18621
ENSG0000011	-	-	ENSG0000011594	'COX7A2L	-	2:42333545-42425088	-	-	IGV_LINK	1230.58	73.0474	29.9458	116.149	OK	934.29	55.9972	23.7126	88.2818	OK	OK	-0.38348
ENSG0000011	-	-	ENSG0000011594	'PNO1	-	2:68122935-68261230	-	-	IGV_LINK	224.975	6.47215	0	14.788	OK	322.773	8.96414	0	20.2144	OK	OK	0.46992
ENSG0000011	-	-	ENSG0000011594	'ORC4	-	2:147844516-148516971	-	-	IGV_LINK	448.011	12.5703	0	33.5756	OK	428.99	13.1934	0	35.6221	OK	OK	0.0698
ENSG0000011	-	-	ENSG0000011595	'PLEK	-	2:68361213-68397453	-	-	IGV_LINK	1734.83	40.5294	13.6972	67.3616	OK	1714.93	37.9129	14.732	61.0937	OK	OK	-0.09628
ENSG0000011	-	-	ENSG0000011596	'RND3	-	2:150468194-150539011	-	-	IGV_LINK	183.277	5.05059	0.559074	9.5421	OK	285.649	7.27963	0.725236	13.834	OK	OK	0.527415
ENSG0000011	-	-	ENSG0000011596	'ATF2	-	2:175072249-175168522	-	-	IGV_LINK	731.169	18.2922	5.1801	31.4043	OK	747.638	18.633	5.64414	31.6218	OK	OK	0.026629
ENSG0000011	-	-	ENSG0000011597	'THADA	-	2:43229572-43596046	-	-	IGV_LINK	887.294	13.3959	1.73881	25.0531	OK	822.918	12.8211	0.409975	25.2322	OK	OK	-0.06328
ENSG0000011	-	-	ENSG0000011597	'AAK1	-	2:69457996-69827100	-	-	IGV_LINK	835.899	8.53534	0	44.6083	OK	1095.16	10.7474	0	58.4042	OK	OK	0.332471
ENSG0000011	-	-	ENSG0000011599	'TRAK2	-	2:201377206-201480846	-	-	IGV_LINK	987.175	8.77836	2.4086	15.1481	OK	877.572	7.80514	1.88484	13.7254	OK	OK	-0.16953
ENSG0000011	-	-	ENSG0000011599	'C2orf42	-	2:70149879-70248660	-	-	IGV_LINK	135.761	4.87611	0	19.3131	OK	141.278	4.47593	0	14.6675	OK	OK	-0.12354
ENSG0000011	-	-	ENSG0000011600	'TIA1	-	2:70149879-70248660	-	-	IGV_LINK	1697.98	80.7977	9.23049	152.365	OK	1163.22	51.6034	3.59724	99.6095	OK	OK	-0.64685
ENSG0000011	-	-	ENSG0000011600	'PCYOX1	-	2:70257385-70281191	-	-	IGV_LINK	1389.61	18.7225	3.54374	33.9012	OK	1556.12	19.0003	5.20689	32.7937	OK	OK	0.021253
ENSG0000011	-	-	ENSG0000011601	'KISS1R	-	19:917286-921015	-	-	IGV_LINK	20.3641	1.03748	0	2.50238	OK	47.4363	2.37574	0	5.1993	OK	OK	1.1953
ENSG0000011	-	-	ENSG0000011601	'EPAS1	-	2:46293666-46386703	-	-	IGV_LINK	5572.98	75.2756	6.61579	143.936	OK	5196.34	69.787	7.66599	131.908	OK	OK	-0.10923
ENSG0000011	-	-	ENSG0000011601	'ARID3A	-	19:925780-975934	-	-	IGV_LINK	156.125	3.01261	0	8.17037	OK	210.37	3.95904	0	9.85554	OK	OK	0.394137
ENSG0000011	-	-	ENSG0000011603	'SUMO1	-	2:202206179-202238608	-	-	IGV_LINK	994.933	48.7873	19.3097	78.2649	OK	1167.35	56.5522	20.8211	92.2833	OK	OK	0.213079
ENSG0000011	-	-	ENSG0000011603	'CD207	-	2:70830213-70835822	-	-	IGV_LINK	86.3051	2.86698	0.274186	5.45978	OK	47.4363	1.57686	0	3.15855	OK	OK	-0.86248
ENSG0000011	-	-	ENSG0000011603	'GRIN3B	-	19:999795-1021179	-	-	IGV_LINK	8.72748	0.158226	0	1.84173	OK	5.15612	0.093513	0	1.53687	OK	NOTEST	-0.75875
ENSG0000011	-	-	ENSG0000011603	'VAX2	-	2:70900589-70933446	-	-	IGV_LINK	0.969721	0.056081	0	0.219653	OK	3.09367	0.179124	0	0.462775	OK	NOTEST	1.67538
ENSG0000011	-	-	ENSG0000011603	'ATP6V1B1	-	2:70935881-71079805	-	-	IGV_LINK	2.90916	0.397696	0	10.1054	OK	6.18735	0.50243	0	10.6156	OK	NOTEST	0.337258
ENSG0000011	-	-	ENSG0000011604	'NFE2L2	-	2:177227594-177559299	-	-	IGV_LINK	1830.38	60.2899	3.59652	116.983	OK	2751.5	87.0625	7.42099	166.704	OK	OK	0.530137
ENSG0000011	-	-	ENSG0000011606	'MSH6	-	2:47695529-47907810	-	-	IGV_LINK	694.32	10.1984	0	21.5542	OK	703.295	10.1658	0.782078	19.5495	OK	OK	-0.00461
ENSG0000011	-	-	ENSG0000011609	'PLEKHA3	-	2:178480467-178516462	-	-	IGV_LINK	262.794	7.91175	0	18.8491	OK	303.18	10.682	0	24.0249	OK	OK	0.43311
ENSG0000011	-	-	ENSG0000011609	'SPR	-	2:72887359-72892158	-	-	IGV_LINK	589.59	25.6479	10.2148	41.0809	OK	729.076	31.4527	13.0037	49.9017	OK	OK	0.294343
ENSG0000011	-	-	ENSG0000011610	'EPHA4	-	2:221418026-221574454	-	-	IGV_LINK	75.6382	0.815068	0	2.27533	OK	128.903	1.65216	0	3.80976	OK	OK	1.01936
ENSG0000011	-	-	ENSG0000011611	'PARD3B	-	2:204545792-205620162	-	-	IGV_LINK	265.703	2.03885	0.438301	3.6394	OK	114.466	0.812777	0	1.78387	OK	OK	-1.32683
ENSG0000011	-	-	ENSG0000011612	'FARSB	-	2:222570535-222656337	-	-	IGV_LINK	365.585	6.44543	2.22537	10.6655	OK	352.679	6.22014	2.16904	10.2712	OK	OK	-0.05133
ENSG0000011	-	-	ENSG0000011612	'ALMS1	-	2:73385757-73610793	-	-	IGV_LINK	537.225	6.18412	0	15.3063	OK	332.054	7.84667	0	24.5913	OK	OK	0.343512
ENSG0000011	-	-	ENSG0000011612	'BCL9	-	1:147541411-147626216	-	-	IGV_LINK	420.859	4.78963	0	10.7813	OK	303.18	3.25476	0	6.6214	OK	OK	-0.55737
ENSG0000011	-	-	ENSG0000011613	'PRRX1	-	1:170662727-170739419	-	-	IGV_LINK	18.4247	0.292494	0	1.0448	OK	73.217	1.40043	0	3.59347	OK	OK	2.25939
ENSG0000011	-	-	ENSG0000011613	'DHCR24	-	1:54849632-54888850	-	-	IGV_LINK	905.719	13.0338	5.26653	20.8011	OK	5896.54	85.5368	30.2501	140.824	OK	OK	2.71429
ENSG0000011	-	-	ENSG0000011613	'DNAJC16	-	1:15526812-15603626	-	-	IGV_LINK	723.411	14.282	0	36.5676	OK	716.701	13.9586	0	39.923	OK	OK	-0.03305
ENSG0000011	-	-	ENSG0000011614	'MARK1	-	1:220528182-220664461	-	-	IGV_LINK	48.486	0.694086	0	1.61509	OK	51.5612	0.826913	0	2.25985	OK	OK	0.252622
ENSG0000011	-	-	ENSG0000011614	'TNR	-	1:175307217-175743770	-	-	IGV_LINK	0	0	0	0	OK	4.1249	0.158299	0	0.596084	OK	NOTEST	inf
ENSG0000011	-	-	ENSG0000011615	'MORN1	-	1:2321252-2391707	-	-	IGV_LINK	343.281	11.7694	2.13793	21.4009	OK	379.491	12.7343	2.47284	22.9957	OK	OK	0.113674
ENSG0000011	-	-	ENSG0000011615	'GPX7	-	1:52602371-52609051	-	-	IGV_LINK	155.155	8.4192	1.47593	15.3625	OK	92.8102	4.99585	0.548233	9.44347	OK	OK	-0.75295
ENSG0000011	-	-	ENSG0000011616	'CACYBP	-	1:174999162-175023425	-	-	IGV_LINK	674.925	42.3733	0	91.0541	OK	608.423	37.6801	0	86.1684	OK	OK	-0.16935
ENSG0000011	-	-	ENSG0000011617	'SCP2	-	1:52927228-53051703	-	-	IGV_LINK	1853.07	92.9828	32.3598	153.606	OK	2824.2	152.808	52.4506	253.165	OK	OK	0.716683
ENSG0000011	-	-	ENSG0000011617	'TPSG1	-	16:1153120-1225257	-	-	IGV_LINK	1.93944	0.121025	0	1.36166	OK	2.06245	0.1244	0	1.14368	OK	NOTEST	0.039692
ENSG0000011	-	-	ENSG0000011618	'PAPPA2	-	1:176463170-176845605	-	-	IGV_LINK	10.6669	0.063155	0	0.539833	OK	4.1249	0.024425	0	0.496922	OK	NOTEST	-1.37054
ENSG0000011	-	-	ENSG0000011619	'RALGPS2	-	1:178724305-178921841	-	-	IGV_LINK	232.733	3.95902	0	10.957	OK	204.183	2.75224	0	7.62284	OK	OK	-0.52454
ENSG0000011	-	-	ENSG0000011619	'ANGPTL1	-	1:178724305-178921841	-	-	IGV_LINK	3.87888	0.103283	0	0.717168	OK	4.1249	0.155411	0	1.28855	OK	NOTEST	0.589484
ENSG0000011	-	-	ENSG0000011619	'CEP104	-	1:3812080-3857214	-	-	IGV_LINK	638.076	14.2135	0	29.8472	OK	735.263	14.0432	0	29.767	OK	OK	-0.0174
ENSG0000011	-	-	ENSG0000011619	'FAM20B	-	1:179025803-179076562	-	-	IGV_LINK	670.077	7.10283	1.22811	12.9776	OK	643.484	6.24136	2.37112	10.1116	OK	OK	-0.18653
ENSG0000011	-	-	ENSG0000011620	'TCEANC2	-	1:54053586-54112519	-	-	IGV_LINK	521.71	3.29177	0.907829	5.67572	OK	415.584	2.91164	0.170711	5.65257	OK	OK	-0.17703
ENSG0000011	-	-	ENSG0000011620	'TMEM59	-	1:54026680-54053504	-	-	IGV_LINK	4906.79	313.353	73.1671	553.538	OK	5929.54	397.509	81.6968	713.322	OK	OK	0.343202
ENSG0000011	-	-	ENSG0000011621	'LRRC42	-	1:53946076-53968168	-	-	IGV_LINK	329.705	12.1149	3.7121	20.5178	OK	348.554	13.2045	4.13749	22.2716	OK	OK	0.124247
ENSG0000011	-	-	ENSG0000011621	'WRAP73	-	1:3630766-3771645	-	-	IGV_LINK	502.315	15.5538	0	37.7548	OK	550.674	16.6024	0	36.9451	OK	OK	0.094131
ENSG0000011	-	-	ENSG0000011621	'NPHS2	-	1:179365719-179575952	-	-	IGV_LINK	0.969721	0.036494	0	0.201434	OK	0	0	0	0	OK	NOTEST	'-inf
ENSG0000011	-	-	ENSG0000011622	'MRPL37	-	1:54132685-54413479	-	-	IGV_LINK	1140.39	49.8442	9.70718	89.9812	OK	1394.22	60.1176	15.3406	104.895	OK	OK	0.270361
ENSG0000011	-	-	ENSG0000011623	'ICMT	-	1:6181268-6235972	-	-	IGV_LINK	826.202	12.056	0	39.237	OK	964.195	14.8881	0	43.4222	OK	OK	0.304411
ENSG0000011	-	-	ENSG0000011625	'RPL22	-	1:6181268-6235972	-	-	IGV_LINK	7651.09	485.912	0	1114.01	OK	5865.61	375.778	0	762.322	OK	OK	-0.37081
ENSG0000011	-	-	ENSG0000011625	'CHD5	-	1:6101792-6180123	-	-	IGV_LINK	5.81832	0.034612	0	0.317311	OK	3.09367	0.123184	0	0.522024	OK	NOTEST	1.83147
ENSG0000011	-	-	ENSG0000011626	'QSOX1	-	1:180154833-180204030	-	-	IGV_LINK	1337.24	29.2955	7.30641	51.2847	OK	3382.42	68.6768	19.2602	118.093	OK	OK	1.22914
ENSG0000011	-	-	ENSG0000011626	'STXBP3	-	1:108746673-108809526	-	-	IGV_LINK	709.835	21.022	1.28236	40.7617	OK	756.919	24.0483	1.13437	46.9622	OK	OK	0.194032
ENSG0000011	-	-	ENSG0000011627	'PHF13	-	1:6590723-6624033	-	-	IGV_LINK	450.92	7.24297	0	15.1863	OK	406.303	6.52843	0	14.1399	OK	OK	-0.14984
ENSG0000011	-	-	ENSG0000011628	'ERRFI1	-	1:7991133-8026308	-	-	IGV_LINK	1112.27	24.7972	8.51767	41.0767	OK	3025.61	67.1485	27.0173	107.28	OK	OK	1.43718
ENSG0000011	-	-	ENSG0000011628	'PARK7	-	1:7954290-7985505	-	-	IGV_LINK	2613.4	212.536	77.4664	347.606	OK	2810.09	235.636	93.891	377.381	OK	OK	0.148854
ENSG0000011	-	-	ENSG0000011629	'KIAA1324	-	1:109105950-109206781	-	-	IGV_LINK	124.124	1.95714	0.065653	3.84863	OK	48.4676	0.886838	0	2.55843	OK	OK	-1.14201
ENSG0000011	-	-	ENSG0000011632	'OPRD1	-	1:28812141-28877336	-	-	IGV_LINK	12.6064	0.077453	0	0.212649	OK	8.2498	0.050693	0	0.170816	OK	NOTEST	-0.61154
ENSG0000011	-	-	ENSG0000011633	'AMPD2	-	1:109548610-109632051	-	-	IGV_LINK	1569.01	48.5202	0	102.946	OK	2135.67	68.8109	0	146.609	OK	OK	0.504052
ENSG0000011	-	-	ENSG0000011635	'SRSF4	-	1:29147742-29181987	-	-	IGV_LINK	2060.66	59.8588	0	129.015	OK	2336.53	71.0543	0	159.145	OK	OK	0.24736
ENSG0000011	-	-	ENSG0000011635	'MECR	-	1:29192872-29230942	-	-	IGV_LINK	288.977	16.7069	3.10379	30.3101	OK	350.616	18.2012	3.54794	32.8546	OK	OK	0.12359
ENSG0000011	-	-	ENSG0000011639	'KCNC4	-	1:110211342-110283100	-	-	IGV_LINK	908.628	8.1051	0	22.2912	OK	445.489	4.02698	0	8.65198	OK	OK	-1.00913
ENSG0000011	-	-	ENSG0000011640	'EDEM3	-	1:184690230-184754913	-	-	IGV_LINK	641.955	5.57615	2.03658	9.11572	OK	629.047	7.26177	0	16.198	OK	OK	0.381051
ENSG0000011	-	-	ENSG0000011645	'WDR77	-	1:111438637-111462773	-	-	IGV_LINK	379.398	10.5473	0	31.6572	OK	447.964	13.8583	0	41.5753	OK	OK	0.393878
ENSG0000011	-	-	ENSG0000011645	'ATP5F1	-	1:111438637-111462773	-	-	IGV_LINK	2184.78	192.229	62.199	322.258	OK	2263.54	203.964	62.4874	345.44	OK	OK	0.085488
ENSG0000011	-	-	ENSG0000011647	'RAP1A	-	1:111483347-111768016	-	-	IGV_LINK	1036.63	45.2005	3.60017	86.8009	OK	887.885	41.3925	5.98998	76.7951	OK	OK	-0.12697
ENSG0000011	-	-	ENSG0000011647	'HDAC1	-	1:32292085-32333635	-	-	IGV_LINK	2030.59	67.6061	24.7352	110.477	OK	1763.39	58.6976	21.017	96.3781	OK	OK	-0.20385
ENSG0000011	-	-	ENSG0000011648	'CAPZA1	-	1:112466540-112671619	-	-	IGV_LINK	1716.41	49.1408	6.89572	91.3858	OK	2272.82	75.9931	2.74371	149.243	OK	OK	0.628949
ENSG0000011	-	-	ENSG0000011649	'S100PBP	-	1:32775236-32858879	-	-	IGV_LINK	838.808	19.9784	0	50.4555	OK	666.171	17.5687	0	46.9156	OK	OK	-0.18543
ENSG0000011	-	-	ENSG0000011651	'RNF19B	-	1:32936444-32964685	-	-	IGV_LINK	1124.88	28.0665	11.9914	44.1417	OK	1297.28	32.5678	14.0884	51.0472	OK	OK	0.214596
ENSG0000011	-	-	ENSG0000011652	'SCAMP3	-	1:155255978-155262430	-	-	IGV_LINK	1055.06	50.582	20.1552	81.0089	OK	1141.57	53.6765	21.4184	85.9347	OK	OK	0.085667
ENSG0000011	-	-	ENSG0000011652	'TRIM62	-	1:33145401-33182059	-	-	IGV_LINK	122.185	2.20538	0.047159	4.3636	OK	161.902	2.79595	0.286539	5.30536	OK	OK	0.34231
ENSG0000011	-	-	ENSG0000011653	'ASH1L	-	1:155335267-155563944	-	-	IGV_LINK	1691.19	10.4279	0	22.4761	OK	1164.25	8.70201	0	20.6013	OK	OK	-0.26102
ENSG0000011	-	-	ENSG0000011654	'DLGAP3	-	1:34865435-34929585	-	-	IGV_LINK	24.243	0.430434	0	1.20035	OK	19.5933	0.580634	0	1.81943	OK	OK	0.431838
ENSG0000011	-	-	ENSG0000011656	'SFPQ	-	1:35176377-35193148	-	-	IGV_LINK	4572.23	160.462	29.1541	291.769	OK	3468.01	127.369	24.0603	230.678	OK	OK	-0.33321
ENSG0000011	-	-	ENSG0000011657	'RHOU	-	1:228735076-228746669	-	-	IGV_LINK	268.613	3.60468	1.10593	6.10342	OK	333.086	4.47111	1.5296	7.41262	OK	OK	0.310762
ENSG0000011	-	-	ENSG0000011658	'GON4L	-	1:155745828-155859400	-	-	IGV_LINK	1763.92	22.2342	0.317324	44.151	OK	1571.59	20.9944	0.341503	41.6473	OK	OK	-0.08277
ENSG0000011	-	-	ENSG0000011658	'ARHGEF2	-	1:155946850-156007070	-	-	IGV_LINK	1926.83	39.541	9.60076	69.4813	OK	1819.08	39.7928	6.08209	73.5036	OK	OK	0.009158
ENSG0000011	-	-	ENSG0000011658	'LAMTOR2	-	1:156054751-156058510	-	-	IGV_LINK	516.861	59.8988	23.062	96.7357	OK	702.264	82.9745	32.8517	133.097	OK	OK	0.47014
ENSG0000011	-	-	ENSG0000011660	'MEF2D	-	1:156463726-156500828	-	-	IGV_LINK	1276.15	18.2625	2.72828	33.7968	OK	1310.69	18.9368	3.61877	34.2548	OK	OK	0.052302
ENSG0000011	-	-	ENSG0000011664	'DOCK7	-	1:62454297-62688368	-	-	IGV_LINK	804.868	9.64239	0.355961	18.9288	OK	515.612	7.30297	0	15.5568	OK	OK	-0.40091
ENSG0000011	-	-	ENSG0000011664	'SRM	-	1:11054583-11060024	-	-	IGV_LINK	373.342	20.8171	6.18549	35.4486	OK	472.301	26.7503	8.32631	45.1743	OK	OK	0.361788
ENSG0000011	-	-	ENSG0000011665	'DLEU2L	-	1:63440769-63660245	-	-	IGV_LINK	43.7929	0.729633	0	3.18824	OK	17.4888	0.29148	0	2.72983	OK	OK	-1.32377
ENSG0000011	-	-	ENSG0000011666	'FBXO2	-	1:11623557-11663327	-	-	IGV_LINK	74.6685	2.99997	0	9.59089	OK	86.6229	3.52131	0	10.305	OK	OK	0.23116
ENSG0000011	-	-	ENSG0000011666	'FBXO6	-	1:11664123-11674354	-	-	IGV_LINK	379.161	16.4658	5.58066	27.3509	OK	301.118	13.1544	4.19684	22.1119	OK	OK	-0.32393
ENSG0000011	-	-	ENSG0000011666	'C1orf21	-	1:184387057-184629020	-	-	IGV_LINK	262.794	5.87673	0	14.2495	OK	582.642	27.0879	1.89218	52.2836	OK	OK	2.20456
ENSG0000011	-	-	ENSG0000011666	'SWT1	-	1:185157079-185291765	-	-	IGV_LINK	149.337	2.32288	0.445447	4.20032	OK	110.341	1.7007	0.097827	3.30357	OK	OK	-0.44979
ENSG0000011	-	-	ENSG0000011667	'MAD2L2	-	1:11674479-11691650	-	-	IGV_LINK	330.675	19.7923	6.72328	32.8613	OK	412.49	25.0939	8.76191	41.4259	OK	OK	0.3424
ENSG0000011	-	-	ENSG0000011667	'DNAJC6	-	1:65248218-65416006	-	-	IGV_LINK	98.9115	1.17548	0	2.55383	OK	347.523	5.3124	0	12.3581	OK	OK	2.17612
ENSG0000011	-	-	ENSG0000011667	'LEPR	-	1:65420586-65641559	-	-	IGV_LINK	343.364	4.72188	0	12.3873	OK	368.207	4.62287	0	12.7784	OK	OK	-0.03057
ENSG0000011	-	-	ENSG0000011667	'IVNS1ABP	-	1:185296387-185317329	-	-	IGV_LINK	5649.59	120.423	26.0175	214.828	OK	4257.93	96.0379	24.0364	168.039	OK	OK	-0.32643
ENSG0000011	-	-	ENSG0000011668	'KIAA2013	-	1:11919590-11926428	-	-	IGV_LINK	1063.78	23.3829	10.162	36.6039	OK	1339.56	29.0635	12.7815	45.3454	OK	OK	0.31375
ENSG0000011	-	-	ENSG0000011668	'MFN2	-	1:11980180-12013514	-	-	IGV_LINK	1818.23	24.4503	8.21914	40.6814	OK	2107.82	27.9259	10.5653	45.2865	OK	OK	0.191751
ENSG0000011	-	-	ENSG0000011669	'PRG4	-	1:186296278-186375693	-	-	IGV_LINK	1.93944	0.023796	0	0.585168	OK	6.18735	0.075942	0	0.907903	OK	NOTEST	1.67418
ENSG0000011	-	-	ENSG0000011669	'MIIP	-	1:12019465-12032045	-	-	IGV_LINK	333.584	14.1362	4.12736	24.145	OK	253.681	11.6149	2.82759	20.4021	OK	OK	-0.28342
ENSG0000011	-	-	ENSG0000011669	'SMG7	-	1:183460873-183598246	-	-	IGV_LINK	2054.84	25.4825	0	66.9172	OK	1735.55	19.6328	0.585848	38.6797	OK	OK	-0.37624
ENSG0000011	-	-	ENSG0000011670	'NCF2	-	1:183460873-183598246	-	-	IGV_LINK	772.867	21.6248	0	45.5462	OK	946.665	27.5264	0	55.5197	OK	OK	0.348134
ENSG0000011	-	-	ENSG0000011670	'PDC	-	1:186435160-186470291	-	-	IGV_LINK	0	0	0	0	OK	1.03122	0.058089	0	0.25128	OK	NOTEST	inf
ENSG0000011	-	-	ENSG0000011670	'SLC35D1	-	1:66999331-67054099	-	-	IGV_LINK	494.557	4.65332	1.76635	7.5403	OK	1062.16	9.99587	4.35126	15.6405	OK	OK	1.10307
ENSG0000011	-	-	ENSG0000011671	'PLA2G4A	-	1:186828952-186988981	-	-	IGV_LINK	150.307	3.1344	0.52548	5.74331	OK	104.154	2.25736	0.117633	4.39708	OK	OK	-0.47355
ENSG0000011	-	-	ENSG0000011671	'GADD45A	-	1:67685060-67688338	-	-	IGV_LINK	439.283	19.5141	7.01397	32.0143	OK	1824.24	81.1709	34.7471	127.595	OK	OK	2.05644
ENSG0000011	-	-	ENSG0000011672	'PRAMEF1	-	1:12791396-12796628	-	-	IGV_LINK	0	0	0	0	OK	0	0	0	0	OK	NOTEST	0
ENSG0000011	-	-	ENSG0000011672	'PRAMEF12	-	1:12774840-12777906	-	-	IGV_LINK	0	0	0	0	OK	0	0	0	0	OK	NOTEST	0
ENSG0000011	-	-	ENSG0000011672	'WLS	-	1:67701465-68233120	-	-	IGV_LINK	1368.28	41.0849	0	84.7421	OK	872.416	27.1982	0	65.4113	OK	OK	-0.5951
ENSG0000011	-	-	ENSG0000011673	'PRDM2	-	1:13700197-13825079	-	-	IGV_LINK	815.535	9.2015	1.25889	17.1441	OK	1339.56	17.0634	0.894799	33.2321	OK	OK	0.890967
ENSG0000011	-	-	ENSG0000011674	'RGS2	-	1:192809038-192812283	-	-	IGV_LINK	685.592	39.524	13.8947	65.1533	OK	929.134	50.0247	19.5786	80.4708	OK	OK	0.339913
ENSG0000011	-	-	ENSG0000011674	'RPE65	-	1:68428821-68449959	-	-	IGV_LINK	0	0	0	0	OK	2.06245	0.047707	0	0.140232	OK	NOTEST	inf
ENSG0000011	-	-	ENSG0000011674	'TROVE2	-	1:193012249-193091777	-	-	IGV_LINK	305.462	5.20397	0	12.0613	OK	259.869	6.29068	0	17.4854	OK	OK	0.273601
ENSG0000011	-	-	ENSG0000011674	'AMPD1	-	1:114673089-114695618	-	-	IGV_LINK	6.78804	0.354256	0	1.11002	OK	10.3122	0.261986	0	0.84974	OK	NOTEST	-0.4353
ENSG0000011	-	-	ENSG0000011675	'UCHL5	-	1:193012249-193091777	-	-	IGV_LINK	288.977	10.6543	0	22.6892	OK	284.618	12.0636	0	25.0724	OK	OK	0.179218
ENSG0000011	-	-	ENSG0000011675	'BCAS2	-	1:114567556-114581639	-	-	IGV_LINK	320.977	17.7296	4.45557	31.0037	OK	440.333	22.0524	7.85339	36.2514	OK	OK	0.314774

ENSG000001	-	-	ENSG0000011675	SRSF11	-	1:70205681-70253052	-	-	IGV_LINK	3989.43	165.093	34.9707	295.216	OK	3858.84	155.973	30.8098	281.137	OK	OK	-0.08198
ENSG000001	-	-	ENSG0000011676	CTH	-	1:70411217-70439851	-	-	IGV_LINK	43.6374	1.92552	0	4.44476	OK	111.372	4.48478	0.181542	8.78801	OK	OK	1.21978
ENSG000001	-	-	ENSG0000011677	AGMAT	-	1:15526812-15603626	-	-	IGV_LINK	538.195	13.0314	1.52757	24.5352	OK	1160.13	28.1042	8.12392	48.0845	OK	OK	1.1088
ENSG000001	-	-	ENSG0000011677	OLFML3	-	1:113979390-114035572	-	-	IGV_LINK	648.743	22.5587	9.2285	35.8888	OK	337.211	11.7653	4.01229	19.5183	OK	OK	-0.93914
ENSG000001	-	-	ENSG0000011678	TNNI3K	-	1:74198211-74544428	-	-	IGV_LINK	43.9315	1.77132	0	6.6019	OK	43.4727	1.67477	0	6.58901	OK	OK	-0.08086
ENSG000001	-	-	ENSG0000011678	CFHR3	-	1:196774794-196795406	-	-	IGV_LINK	1.93944	0.131606	0	0.469174	OK	39.1865	2.35905	0	4.92261	OK	OK	4.16391
ENSG000001	-	-	ENSG0000011678	PLEKHM2	-	1:15684331-15749896	-	-	IGV_LINK	1660.16	27.6445	8.54347	46.7456	OK	2223.32	35.6395	11.457	59.822	OK	OK	0.366484
ENSG000001	-	-	ENSG0000011679	CRYZ	-	1:74705481-74766678	-	-	IGV_LINK	2196.42	106.976	24.6885	189.263	OK	4480.67	216.455	44.5047	388.405	OK	OK	1.01678
ENSG000001	-	-	ENSG0000011679	PHTF1	-	1:113696830-113759489	-	-	IGV_LINK	427.647	10.0502	2.07997	18.0205	OK	500.144	12.5124	2.81704	22.2078	OK	OK	0.316133
ENSG000001	-	-	ENSG0000011680	ZBTB17	-	1:15941868-15976132	-	-	IGV_LINK	596.378	23.2233	3.77359	42.6731	OK	492.926	18.4448	2.90985	33.9797	OK	OK	-0.33236
ENSG000001	-	-	ENSG0000011681	CD58	-	1:116514534-116571039	-	-	IGV_LINK	278.31	15.3247	4.34462	26.3049	OK	284.618	16.0801	5.05043	27.1098	OK	OK	0.069414
ENSG000001	-	-	ENSG0000011681	TFAP2E	-	1:35569812-35595328	-	-	IGV_LINK	28.1219	0.785936	0	1.87619	OK	21.6557	0.605568	0	1.47559	OK	OK	-0.37612
ENSG000001	-	-	ENSG0000011682	CD2	-	1:116754384-116769228	-	-	IGV_LINK	800.989	31.4243	13.163	49.6855	OK	402.178	15.7908	5.72484	25.8568	OK	OK	-0.9928
ENSG000001	-	-	ENSG0000011683	TTF2	-	1:117060302-117107453	-	-	IGV_LINK	364.615	7.54142	0	16.6186	OK	278.431	4.60255	0	10.7411	OK	OK	-0.7124
ENSG000001	-	-	ENSG0000011683	NR5A2	-	1:200027601-200177424	-	-	IGV_LINK	49.4557	0.629165	0	1.47637	OK	64.9672	0.770297	0	1.67274	OK	OK	0.291978
ENSG000001	-	-	ENSG0000011685	KIF21B	-	1:200969389-201023700	-	-	IGV_LINK	485.83	3.22028	1.10496	5.3356	OK	349.585	2.33799	0.60865	4.06732	OK	OK	-0.46192
ENSG000001	-	-	ENSG0000011685	TMEM9	-	1:201134771-201171574	-	-	IGV_LINK	1292.64	55.4109	22.6757	88.1461	OK	1848.99	79.9203	32.5756	127.265	OK	OK	0.528392
ENSG000001	-	-	ENSG0000011686	ADPRHL2	-	1:36088874-36093932	-	-	IGV_LINK	660.38	24.8857	10.1575	39.6139	OK	846.636	31.9292	13.5122	50.3461	OK	OK	0.359556
ENSG000001	-	-	ENSG0000011687	MAP7D1	-	1:36136569-36180849	-	-	IGV_LINK	1276.15	31.4038	3.56117	59.2464	OK	1317.91	33.8381	0	68.2105	OK	OK	0.107709
ENSG000001	-	-	ENSG0000011687	WARS2	-	1:119031215-119327303	-	-	IGV_LINK	278.31	6.62794	0.348411	12.9075	OK	304.211	7.49336	0.568463	14.4182	OK	OK	0.177052
ENSG000001	-	-	ENSG0000011688	HAO2	-	1:119368778-119394130	-	-	IGV_LINK	54.3043	2.38105	0	5.03893	OK	93.8415	4.10269	0.012523	8.19287	OK	OK	0.784975
ENSG000001	-	-	ENSG0000011688	AL591845.1	-	1:36306386-36329221	-	-	IGV_LINK	108.047	3.65603	0	10.039	OK	153.507	5.19786	0	14.236	OK	OK	0.50764
ENSG000001	-	-	ENSG0000011688	OSCP1	-	1:36415826-36450451	-	-	IGV_LINK	262.794	16.1029	0.437521	31.7684	OK	266.056	17.4805	0.97955	33.9815	OK	OK	0.118424
ENSG000001	-	-	ENSG0000011689	MRPS15	-	1:36455717-36464437	-	-	IGV_LINK	648.743	42.9192	16.3471	69.4913	OK	882.729	57.6688	23.1543	92.1834	OK	OK	0.426169
ENSG000001	-	-	ENSG0000011690	EXOC8	-	1:231332752-231355023	-	-	IGV_LINK	302.553	3.46029	0.596579	6.32399	OK	253.681	2.90202	0.55927	5.24477	OK	OK	-0.25383
ENSG000001	-	-	ENSG0000011690	GNPAT	-	1:231241206-231277973	-	-	IGV_LINK	1117.12	29.2165	9.53966	48.8932	OK	994.101	26.873	7.94435	45.8016	OK	OK	-0.12063
ENSG000001	-	-	ENSG0000011691	TSNAX	-	1:231528652-232041272	-	-	IGV_LINK	521.354	16.3288	0	34.977	OK	566.468	22.2849	0	45.8111	OK	OK	0.448648
ENSG000001	-	-	ENSG0000011692	C1orf109	-	1:37681569-37692249	-	-	IGV_LINK	341.342	14.8085	2.79972	26.8173	OK	282.556	11.7419	1.71857	21.7653	OK	OK	-0.33476
ENSG000001	-	-	ENSG0000011695	RRAGC	-	1:38838197-38941799	-	-	IGV_LINK	590.56	13.0295	3.73717	22.3218	OK	571.28	12.6085	2.51876	22.6982	OK	OK	-0.04739
ENSG000001	-	-	ENSG0000011695	TBCE	-	1:235367359-235448968	-	-	IGV_LINK	320.977	12.6489	1.17023	24.1276	OK	288.743	10.3038	1.42989	19.1777	OK	OK	-0.29584
ENSG000001	-	-	ENSG0000011696	NID1	-	1:235975829-236065162	-	-	IGV_LINK	2039.32	20.1928	8.95161	31.434	OK	2846.18	28.1878	12.2279	44.1477	OK	OK	0.48123
ENSG000001	-	-	ENSG0000011697	LGALS8	-	1:236517999-236604504	-	-	IGV_LINK	1253.85	40.3744	0	86.1003	OK	1440.62	45.9236	0	96.5301	OK	OK	0.185793
ENSG000001	-	-	ENSG0000011698	NT5C1A	-	1:39659120-39672038	-	-	IGV_LINK	1.93944	0.116962	0	0.358191	OK	0	0	0	0	OK	NOTEST	'-inf
ENSG000001	-	-	ENSG0000011698	HPCAL4	-	1:39678647-39691689	-	-	IGV_LINK	4.8486	0.064088	0	0.162697	OK	7.21857	0.088733	0	0.211708	OK	NOTEST	0.469406
ENSG000001	-	-	ENSG0000011698	MTR	-	1:236795280-236903981	-	-	IGV_LINK	2349.63	14.1245	5.59542	22.6536	OK	3634.04	22.2107	8.51985	35.9016	OK	OK	0.653055
ENSG000001	-	-	ENSG0000011698	BMP8B	-	1:39692181-39788861	-	-	IGV_LINK	127.033	1.53975	0	4.4087	OK	96.9351	1.17522	0	3.27806	OK	OK	-0.38976
ENSG000001	-	-	ENSG0000011699	MYCL	-	1:39895425-39902256	-	-	IGV_LINK	265.703	5.54802	1.5353	9.56074	OK	119.622	2.20443	0.295223	4.11363	OK	OK	-1.33157
ENSG000001	-	-	ENSG0000011699	SIPA1L2	-	1:232397964-232561558	-	-	IGV_LINK	765.109	9.61319	0	20.0284	OK	1071.44	14.7202	0	29.8108	OK	OK	0.614715
ENSG000001	-	-	ENSG0000011699	ZP4	-	1:237862174-237928321	-	-	IGV_LINK	0	0	0	0	OK	0	0	0	0	OK	NOTEST	0
ENSG000001	-	-	ENSG0000011700	RLF	-	1:40161372-40240921	-	-	IGV_LINK	575.044	5.33579	2.10639	8.5652	OK	496.019	4.6034	1.76054	7.44626	OK	OK	-0.213
ENSG000001	-	-	ENSG0000011700	KMO	-	1:241532133-241802133	-	-	IGV_LINK	317.099	9.88463	0	25.3027	OK	2457.41	87.6713	1.56505	173.777	OK	OK	3.14885
ENSG000001	-	-	ENSG0000011701	ZNF684	-	1:40531560-40548169	-	-	IGV_LINK	48.486	3.05301	0	7.03712	OK	36.0929	2.28398	0	5.25228	OK	OK	-0.41868
ENSG000001	-	-	ENSG0000011701	KCNQ4	-	1:40784011-40840452	-	-	IGV_LINK	290.916	4.7181	1.49598	7.94021	OK	16.4996	0.257699	0	0.571455	OK	OK	-4.19445
ENSG000001	-	-	ENSG0000011701	RIMS3	-	1:40620678-40665657	-	-	IGV_LINK	66.9107	0.533928	0	1.09357	OK	86.6229	0.691329	0.05952	1.32314	OK	OK	0.372728
ENSG000001	-	-	ENSG0000011702	AKT3	-	1:243256033-243851079	-	-	IGV_LINK	1484.64	15.4772	0	32.2042	OK	1024.01	11.8913	0	26.5119	OK	OK	-0.38024
ENSG000001	-	-	ENSG0000011703	ETV3	-	1:157121190-157138474	-	-	IGV_LINK	485.83	5.76709	1.87578	9.6584	OK	442.396	5.44873	1.48392	9.41355	OK	OK	-0.08192
ENSG000001	-	-	ENSG0000011705	ACADM	-	1:75724346-75795079	-	-	IGV_LINK	1302.33	62.7008	5.48879	119.913	OK	1460.21	69.4123	10.2833	128.541	OK	OK	0.146709
ENSG000001	-	-	ENSG0000011706	ST6GALNAC5	-	1:76867440-77065711	-	-	IGV_LINK	8.72748	0.232353	0	0.791313	OK	0	0	0	0	OK	NOTEST	'-inf
ENSG000001	-	-	ENSG0000011709	SLAMF1	-	1:160608099-160647295	-	-	IGV_LINK	165.822	3.17752	0	6.55484	OK	139.215	2.04721	0.328314	3.7661	OK	OK	-0.63424
ENSG000001	-	-	ENSG0000011709	CD48	-	1:160670147-160711851	-	-	IGV_LINK	1175.3	67.5432	28.4739	106.613	OK	685.765	38.9417	15.4825	62.401	OK	OK	-0.79449
ENSG000001	-	-	ENSG0000011711	ADGRL2	-	1:81306159-81992436	-	-	IGV_LINK	1370.22	23.7005	2.35508	45.046	OK	1861.36	31.6031	3.48855	59.7176	OK	OK	0.415147
ENSG000001	-	-	ENSG0000011711	PADI2	-	1:17066760-17119435	-	-	IGV_LINK	503.285	6.95146	2.69555	11.2074	OK	522.831	8.06376	2.86174	13.2658	OK	OK	0.214138
ENSG000001	-	-	ENSG0000011711	SDHB	-	1:17018721-17054170	-	-	IGV_LINK	1217	75.5463	30.873	120.22	OK	1469.5	93.7994	39.4723	148.126	OK	OK	0.312218
ENSG000001	-	-	ENSG0000011712	MFAP2	-	1:16974501-17011928	-	-	IGV_LINK	7.75776	0.45428	0	3.11989	OK	21.6557	1.04944	0	4.96994	OK	OK	1.20797
ENSG000001	-	-	ENSG0000011713	RPF1	-	1:84479258-84497790	-	-	IGV_LINK	307.401	14.3833	4.73816	24.0285	OK	338.242	15.9485	5.27129	26.6257	OK	OK	0.149023
ENSG000001	-	-	ENSG0000011713	KDM5B	-	1:202724490-202809470	-	-	IGV_LINK	779.655	9.98427	0.624216	19.3443	OK	773.419	9.99317	1.22347	18.7629	OK	OK	0.001285
ENSG000001	-	-	ENSG0000011714	UAP1	-	1:162561505-162599842	-	-	IGV_LINK	492.618	14.4049	4.99177	23.8181	OK	849.729	24.851	10.2276	39.4743	OK	OK	0.786741
ENSG000001	-	-	ENSG0000011714	ACTL8	-	1:17755312-17827063	-	-	IGV_LINK	0	0	0	0	OK	0	0	0	0	OK	NOTEST	0
ENSG000001	-	-	ENSG0000011715	CTBS	-	1:84498324-84574480	-	-	IGV_LINK	474.193	12.6492	0	25.8466	OK	683.702	18.5846	0.143544	37.0257	OK	OK	0.555065
ENSG000001	-	-	ENSG0000011715	RGS4	-	1:163068774-163076802	-	-	IGV_LINK	46.5466	1.4044	0	3.36774	OK	23.7182	0.821166	0	2.21529	OK	OK	-0.77421
ENSG000001	-	-	ENSG0000011715	KLHL12	-	1:202891099-202928636	-	-	IGV_LINK	897.961	16.1415	6.80462	25.4784	OK	1206.53	21.6535	9.14403	34.163	OK	OK	0.423825
ENSG000001	-	-	ENSG0000011715	IGSF21	-	1:18107745-18378483	-	-	IGV_LINK	38.7888	1.4524	0	3.26657	OK	70.1233	2.84597	0	5.94848	OK	OK	0.970479
ENSG000001	-	-	ENSG0000011715	SSX2IP	-	1:84643706-84690803	-	-	IGV_LINK	300.613	5.76804	0.952695	10.5834	OK	623.891	11.9381	3.66648	20.2096	OK	OK	1.04942
ENSG000001	-	-	ENSG0000011717	ZNHIT6	-	1:85649422-85708433	-	-	IGV_LINK	356.857	6.96468	2.27972	11.6496	OK	357.835	7.10671	2.31234	11.9011	OK	OK	0.029125
ENSG000001	-	-	ENSG0000011721	PLA2G2D	-	1:20111938-20119566	-	-	IGV_LINK	253.097	7.13956	1.97823	12.3009	OK	121.685	3.4221	0.500241	6.34396	OK	OK	-1.06095
ENSG000001	-	-	ENSG0000011722	RBBP5	-	1:205086141-205122015	-	-	IGV_LINK	324.856	4.35067	1.1452	7.55614	OK	349.585	4.69006	1.52309	7.85704	OK	OK	0.108371
ENSG000001	-	-	ENSG0000011722	GBP3	-	1:89006665-89022894	-	-	IGV_LINK	1908.41	47.311	14.7748	79.8472	OK	2610.03	64.3499	17.8127	110.887	OK	OK	0.44376
ENSG000001	-	-	ENSG0000011722	GBP1	-	1:89052318-89065360	-	-	IGV_LINK	4087.37	124.979	21.7606	228.197	OK	1825.27	51.6291	11.0841	92.1741	OK	OK	-1.27543
ENSG000001	-	-	ENSG0000011724	PINK1-AS	-	1:20633454-20661544	-	-	IGV_LINK	118.767	1.56731	0	7.98119	OK	150.631	1.98834	0	11.2283	OK	OK	0.343272
ENSG000001	-	-	ENSG0000011724	KIF17	-	1:20664013-20718017	-	-	IGV_LINK	95.0326	2.79354	0	7.4764	OK	107.247	2.34584	0	4.81621	OK	OK	-0.25199
ENSG000001	-	-	ENSG0000011726	GPR89A	-	1:145607989-145670648	-	-	IGV_LINK	93.0932	3.35277	0	7.03358	OK	89.7166	4.01275	0	8.92532	OK	OK	0.259237
ENSG000001	-	-	ENSG0000011726	CDK18	-	1:205504594-205532793	-	-	IGV_LINK	8486.99	214.081	32.8411	395.321	OK	8063.15	197.314	35.611	359.016	OK	OK	-0.11767
ENSG000001	-	-	ENSG0000011728	RAB29	-	1:205767985-205775460	-	-	IGV_LINK	916.386	20.6403	7.11903	34.1617	OK	1289.03	28.9992	10.8822	47.1161	OK	OK	0.490544
ENSG000001	-	-	ENSG0000011728	CD160	-	1:145719470-145824077	-	-	IGV_LINK	46.5466	1.59393	0	7.06024	OK	58.7798	3.32963	0	18.8243	OK	OK	1.06277
ENSG000001	-	-	ENSG0000011729	ECE1	-	1:21217246-21345504	-	-	IGV_LINK	1464.28	20.1216	3.90442	36.3387	OK	1729.36	24.9979	5.1961	44.7997	OK	OK	0.313063
ENSG000001	-	-	ENSG0000011730	HMGCL	-	1:23801884-23838620	-	-	IGV_LINK	1122.94	46.0692	19.0102	73.1283	OK	1586.02	64.9476	27.6579	102.237	OK	OK	0.495472
ENSG000001	-	-	ENSG0000011730	GALE	-	1:23795598-23800804	-	-	IGV_LINK	728.26	32.8334	11.3314	54.3355	OK	1293.16	63.0255	23.0767	102.974	OK	OK	0.940769
ENSG000001	-	-	ENSG0000011731	ID3	-	1:23557917-23559794	-	-	IGV_LINK	1065.72	70.4091	23.2943	117.524	OK	905.416	60.6575	15.3298	105.985	OK	OK	-0.21508
ENSG000001	-	-	ENSG0000011732	CR2	-	1:207454229-207489895	-	-	IGV_LINK	8.72748	0.120921	0	1.03248	OK	12.3747	0.179361	0	1.10519	OK	NOTEST	0.568795
ENSG000001	-	-	ENSG0000011733	CD46	-	1:207752056-207795513	-	-	IGV_LINK	4799.15	90.8144	26.2814	155.347	OK	3824.81	80.7026	10.1321	151.273	OK	OK	-0.17031
ENSG000001	-	-	ENSG0000011736	PRPF3	-	1:150321475-150353195	-	-	IGV_LINK	1607.8	60.0097	21.2055	98.8139	OK	1715.96	63.7142	22.3505	105.078	OK	OK	0.086418
ENSG000001	-	-	ENSG0000011736	APH1A	-	1:150265398-150280916	-	-	IGV_LINK	2778.25	89.4799	30.0778	148.882	OK	2970.96	97.7429	30.8264	164.659	OK	OK	0.127429
ENSG000001	-	-	ENSG0000011738	P3H1	-	1:42746334-42767084	-	-	IGV_LINK	734.078	23.4345	4.75657	42.1125	OK	954.914	26.2989	7.05221	45.5455	OK	OK	0.166365
ENSG000001	-	-	ENSG0000011739	SLC2A1	-	1:42925374-42983358	-	-	IGV_LINK	2492.18	58.587	17.5162	99.6578	OK	5194.28	119.871	33.4229	206.32	OK	OK	1.03283
ENSG000001	-	-	ENSG0000011739	EBNA1BP2	-	1:43164174-43274002	-	-	IGV_LINK	707.896	39.2251	9.17678	69.2735	OK	850.761	44.9134	15.0692	74.7576	OK	OK	0.195369
ENSG000001	-	-	ENSG0000011739	CDC20	-	1:43358954-43363203	-	-	IGV_LINK	99.8812	4.27504	0.224084	8.326	OK	153.653	5.86969	1.00041	10.739	OK	OK	0.457346
ENSG000001	-	-	ENSG0000011740	MPL	-	1:43337848-43352772	-	-	IGV_LINK	1.93944	0.066644	0	0.219049	OK	5.15612	0.169571	0	0.423522	OK	NOTEST	1.34735
ENSG000001	-	-	ENSG0000011740	ARTN	-	1:43933319-43937241	-	-	IGV_LINK	8.72748	0.435074	0	1.28163	OK	6.18735	0.451235	0	1.36029	OK	NOTEST	0.052616
ENSG000001	-	-	ENSG0000011740	IPO13	-	1:43946938-43968022	-	-	IGV_LINK	531.407	8.9486	2.68607	15.2111	OK	614.61	13.0965	0	27.24	OK	OK	0.54945
ENSG000001	-	-	ENSG0000011741	ATP6V0B	-	1:43974486-43978295	-	-	IGV_LINK	1912.29	118.415	41.1197	195.711	OK	2092.36	127.367	50.7546	203.98	OK	OK	0.105143
ENSG000001	-	-	ENSG0000011741	B4GALT2	-	1:43978942-43991170	-	-	IGV_LINK	441.223	14.6129	4.44037	24.7855	OK	460.958	14.8972	5.24665	24.5477	OK	OK	0.027794
ENSG000001	-	-	ENSG0000011741	ERI3	-	1:44221069-44355260	-	-	IGV_LINK	698.199	28.8074	8.46948	49.1453	OK	1094.13	44.855	15.0826	74.6274	OK	OK	0.63883
ENSG000001	-	-	ENSG0000011742	PTCH2	-	1:44819843-44843063	-	-	IGV_LINK	75.6382	1.09666	0.012554	2.18076	OK	43.3114	0.772109	0	1.73887	OK	OK	-0.50624
ENSG000001	-	-	ENSG0000011744	AKR1A1	-	1:45550542-45570049	-	-	IGV_LINK	3132.2	194.984	75.607	314.36	OK	4100.15	260.251	94.9477	425.555	OK	OK	0.416553
ENSG000001	-	-	ENSG0000011745	PRDX1	-	1:45493865-45523047	-	-	IGV_LINK	3930.28	261.082	99.114	423.05	OK	6582.31	447.289	168.871	725.707	OK	OK	0.776705
ENSG000001	-	-	ENSG0000011746	PIK3R3	-	1:46040139-46185958	-	-	IGV_LINK	580.863	6.47634	0	14.0944	OK	685.765	7.3213	0	20.0747	OK	OK	0.176922
ENSG000001	-	-	ENSG0000011747	TSPAN1	-	1:46040139-46185958	-	-	IGV_LINK	1509.85	67.5521	18.303	116.801	OK	5067.44	232.438	68.1692	396.707	OK	OK	1.78277
ENSG000001	-	-	ENSG0000011747	BLZF1	-	1:169367969-169460669	-	-	IGV_LINK	385.949	11.1317	2.78068	19.4827	OK	448.583	13.0473	3.55183	22.5428	OK	OK	0.22908
ENSG000001	-	-	ENSG0000011747	CCDC181	-	1:169367969-169460669	-	-	IGV_LINK	1.93944	0.06476	0	0.728163	OK	0	0	0	0	OK	NOTEST	'-inf
ENSG000001	-	-	ENSG0000011747	SLC19A2	-	1:169463908-169486003	-	-	IGV_LINK	53.3346	0.861177	0.003965	1.71839	OK	71.1545	1.14658	0.066651	2.22652	OK	OK	0.41296
ENSG000001	-	-	ENSG0000011748	FAAH	-	1:46394264-46413848	-	-	IGV_LINK	709.835	29.6526	6.38172	52.9235	OK	678.546	25.3241	6.91505	43.7331	OK	OK	-0.22765
ENSG000001	-	-	ENSG0000011748	NSUN4	-	1:46340176-46365152	-	-	IGV_LINK	560.498	14.3354	3.24276	25.4281	OK	664.109	17.4728	4.36282	30.5828	OK	OK	0.285529
ENSG000001	-	-	ENSG0000011750	TMED5	-	1:93149741-93369498	-	-	IGV_LINK	823.293	12.619	0	32.081	OK	1589.12	23.3301	0	51.3472	OK	OK	0.886595
ENSG000001	-	-	ENSG0000011750	MROH9	-	1:170935470-171064765	-	-	IGV_LINK	0	0	0	0	OK	0	0	0	0	OK	NOTEST	0
ENSG000001	-	-	ENSG0000011750	DR1	-	1:93149741-93369498	-	-	IGV_LINK	830.081	17.5163	0	47.4542	OK	743.513	16.6682	0	42.6335	OK	OK	-0.0716
ENSG000001	-	-	ENSG0000011750	FMO6P	-	1:171137739-171161568	-	-	IGV_LINK	0	0	0	0	OK	1.03122	0.029706	0	0.35472	OK	NOTEST	inf
ENSG000001	-	-	ENSG0000011751	CNN3	-	1:94896948-94927278	-	-	IGV_LINK	3370.75	138.474	38.2545	238.694	OK	3259.7	126.874	38.1706	215.577	OK	OK	-0.12622
ENSG000001	-	-	ENSG0000011752	PRRC2C	-	1:171485550-171593511	-	-	IGV_LINK	3908.94	35.0282	0	85.5233	OK	3208.14	39.5064	0	94.2925	OK	OK	0.17357
ENSG000001	-	-	ENSG0000011752	F3	-	1:94529224-94541800	-	-	IGV_LINK	8.72748	0.256102	0	0.776401	OK	16.4996	0.506426	0	1.22431	OK	NOTEST	0.983635
ENSG000001	-	-	ENSG0000011752	ABCD3	-	1:94418454-94518666	-	-	IGV_LINK	600.257	10.7762	2.98286	18.5695	OK	1057.01	18.0332	7.03051	29.0358	OK	OK	0.742811
ENSG000001	-	-	ENSG0000011753	VAMP4	-	1:171700159-171742247	-	-	IGV_LINK	269.582	6.33992	0	13.8947	OK	219.651	4.00114	0	8.10329	OK	OK	-0.66405
ENSG000001	-	-	ENSG0000011754	DPH5	-	1:100989622-101087268	-	-	IGV_LINK	320.008	17.2619	4.32792	30.1958	OK	218.62	12.9556	1.73626	24.1749	OK	OK	-0.41401
ENSG000001	-	-	ENSG0000011756	FASLG	-	1:172659017-172666874	-	-	IGV_LINK	144.488	4.92126	0.847345	8.99518	OK	87.6541	2.88148	0.279418	5.48354	OK	OK	-0.77222
ENSG000001	-	-	ENSG0000011756	PTBP2	-	1:96721664-96823738	-	-	IGV_LINK	310.311	11.5678	0	26.3114	OK	242.338	7.12117	0.370944	13.8714	OK	OK	-0.69993
ENSG000001	-	-	ENSG0000011758	TNFSF4	-	1:173183733-173207313	-	-	IGV_LINK	103.76	2.33594	0	5.53547	OK	45.3739	1.16755	0	3.19024	OK	OK	-1.00052
ENSG000001	-	-	ENSG0000011759	PRDX6	-	1:173477265-173488807	-	-	IGV_LINK	2597.88	134.992	51.6034	218.38	OK	3130.8	164.112	63.1619	265.062	OK	OK	0.281812
ENSG000001	-	-	ENSG0000011759	DARS2	-	1:173799549-173858546	-	-	IGV_LINK	375.282	8.19071	0	19.8793	OK	420.74	7.11606	2.29321	11.9389	OK	OK	-0.20291
ENSG000001	-	-	ENSG0000011759	HSD11B1	-	1:209661363-209734950	-	-	IGV_LINK	27.1522	1.83364	0	5.68854	OK	4.1249	0.349113	0	3.65625	OK	OK	-2.39294
ENSG000001	-	-	ENSG0000011759	IRF6	-	1:209785622-209806175	-	-	IGV_LINK	415.04	6.90581	0.897473	12.9142	OK	123.747	1.84503	0	3.82605	OK	OK	-1.90417
ENSG000001	-	-	ENSG0000011759	DIEXF	-	1:209828006-209857565	-	-	IGV_LINK	566.317	4.00823	1.51063	6.50583	OK	673.39	4.69091	1.85963	7.52218	OK	OK	0.2269
ENSG000001	-	-	ENSG0000011759	PLPPR5	-	1:98890244-99148852	-	-	IGV_LINK	5.81832	0.105246	0	0.390979	OK	27.8431	0.463478	0	1.04232	OK	NOTEST	2.13873
ENSG000001	-	-	ENSG0000011760	PLPPR4	-	1:99263952-99309590	-	-	IGV_LINK	0.969721	0.012612	0	0.061855	OK	32.9992	0.410948	0	0.879773	OK	NOTEST	5.02606
ENSG000001	-	-	ENSG0000011760	SERPINC1	-	1:173903803-173917378	-	-	IGV_LINK	7.75776	0.356841	0	0.938847	OK	8.2498	0.373312	0	0.965115	OK	NOTEST	0.065103
ENSG000001	-	-	ENSG0000011760	RCAN3	-	1:24502350-24556024	-	-	IGV_LINK	295.765	3.92266	0.668548	7.17678	OK	266.056	3.45114	0.470009	6.43227	OK	OK	-0.18476
ENSG000001	-	-	ENSG0000011761	SYF2	-	1:25222678-25232502	-	-	IGV_LINK	1307.18	63.4822	27.0816	99.6479	OK	1225.1	59.138	24.2367	94.0392	OK	OK	-0.10227
ENSG000001	-	-	ENSG0000011761	RSRP1	-	1:25242236-25430192	-	-	IGV_LINK	3314.5	123.665	14.0935	233.236	OK	2982.3	107.106	3.0032	211.208	OK	OK	-0.2074
ENSG000001	-	-	ENSG0000011762	SLC35A3	-	1:99968382-100132955	-	-	IGV_LINK	416.726	6.7493	0	15.0148	OK	487.633	7.49528	0	15.727	OK	OK	0.151246
ENSG000001	-	-	ENSG0000011762	RCOR3	-	1:211258376-211316385	-	-	IGV_LINK	1029.84	36.8469	4.7843	68.9096	OK	727.014	27.8394	4.06037	51.6185	OK	OK	-0.40441
ENSG000001	-	-	ENSG0000011763	STMN1	-	1:25884180-25906991	-	-	IGV_LINK	960.993	60.3578	21.9404	98.7752	OK	777.544	48.9189	16.5459	81.292	OK	OK	-0.30315
ENSG000001	-	-	ENSG0000011764	MTFR1L	-	1:25800175-25859458	-	-	IGV_LINK	1343.06	48.5009	4.30539	92.6964	OK	1127.57	39.0296	0	80.7978	OK	OK	-0.31344
ENSG000001	-	-	ENSG0000011764	MAN1C1	-	1:25617467-25786207	-	-	IGV_LINK	135.761	3.63813	0	7.44249	OK	184.589	4.36687	0.329419	8.40433	OK	OK	0.263404
ENSG000001	-	-	ENSG0000011765	NEK2	-	1:211658656-211675630	-	-	IGV_LINK	42.6677	1.68134	0	3.57538	OK	78.3731	2.58571	0.038526	5.13289	OK	OK	0.620945

ENSG000001	-	-	ENSG0000011767	'RPS6KA1	-	1:26529760-26575030	-	-	IGV_LINK	1434.22	31.0343	9.81409	52.2546	OK	1595.31	36.2271	9.38975	63.0644	OK	OK	0.223203
ENSG000001	-	-	ENSG0000011768	'DHDDS	-	1:26432281-26471294	-	-	IGV_LINK	622.561	22.0215	3.51111	40.532	OK	1165.28	41.334	7.93309	74.7349	OK	OK	0.908413
ENSG000001	-	-	ENSG0000011769	'NENF	-	1:212432886-212446379	-	-	IGV_LINK	820.384	60.4881	25.5381	95.4381	OK	683.702	50.3128	20.5725	80.0531	OK	OK	-0.26572
ENSG000001	-	-	ENSG0000011769	'NSL1	-	1:212726152-212791782	-	-	IGV_LINK	352.978	16.6509	1.25221	32.0495	OK	365.054	17.0172	2.48197	31.5523	OK	OK	0.031393
ENSG000001	-	-	ENSG0000011770	'PROX1	-	1:213817750-214041502	-	-	IGV_LINK	34.9099	0.305176	0	1.08081	OK	13.4059	0.100444	0	0.446701	OK	NOTEST	-1.60324
ENSG000001	-	-	ENSG0000011771	'ARID1A	-	1:26692131-26782104	-	-	IGV_LINK	1607.8	17.2618	5.13028	29.3933	OK	1949.02	20.7782	5.8813	35.6752	OK	OK	0.267492
ENSG000001	-	-	ENSG0000011772	'CENPF	-	1:214603194-214664588	-	-	IGV_LINK	365.585	6.85188	0	16.6118	OK	262.962	5.32325	0	12.6889	OK	OK	-0.36419
ENSG000001	-	-	ENSG0000011774	'RPA2	-	1:27891523-27914746	-	-	IGV_LINK	798.08	31.9749	11.1503	52.7995	OK	1108.57	43.1328	17.4958	68.7698	OK	OK	0.431845
ENSG000001	-	-	ENSG0000011775	'PPP1R8	-	1:27830777-27851676	-	-	IGV_LINK	589.59	21.1178	5.6518	36.5839	OK	639.359	24.1192	7.94992	40.2885	OK	OK	0.191721
ENSG000001	-	-	ENSG0000011775	'STX12	-	1:27773182-27824452	-	-	IGV_LINK	899.901	23.6506	1.30707	45.9941	OK	1024.01	24.083	2.95495	45.211	OK	OK	0.026138
ENSG000001	-	-	ENSG0000011779	'MARC2	-	1:220748224-220784815	-	-	IGV_LINK	631.288	34.0167	8.8874	59.1461	OK	1255	69.435	19.7458	119.124	OK	OK	1.02942
ENSG000001	-	-	ENSG0000011783	'SLC5A9	-	1:48222684-48248644	-	-	IGV_LINK	363.645	6.78414	2.01291	11.5554	OK	1458.15	28.8309	9.24189	48.42	OK	OK	2.08738
ENSG000001	-	-	ENSG0000011785	'OSBPL9	-	1:51561865-51878937	-	-	IGV_LINK	1876.54	41.8955	0	86.3555	OK	2010.98	45.4309	0	91.1624	OK	OK	0.116879
ENSG000001	-	-	ENSG0000011786	'TXNDC12	-	1:52020130-52090716	-	-	IGV_LINK	1069.79	33.8313	0.476621	67.1859	OK	1737.94	46.4677	11.4687	81.4666	OK	OK	0.45787
ENSG000001	-	-	ENSG0000011786	'ESYT2	-	7:158730994-158830253	-	-	IGV_LINK	2407.82	28.9166	11.5399	46.2933	OK	2817.31	35.7659	11.6882	59.8436	OK	OK	0.306685
ENSG000001	-	-	ENSG0000011787	'CD3EAP	-	19:45379633-45478828	-	-	IGV_LINK	50.4255	1.32991	0	8.4979	OK	57.7486	1.37104	0	7.84078	OK	OK	0.043943
ENSG000001	-	-	ENSG0000011789	'MESD	-	15:80779342-80989878	-	-	IGV_LINK	838.63	13.7421	4.99221	22.4921	OK	1311.72	20.2369	7.51493	32.959	OK	OK	0.558385
ENSG000001	-	-	ENSG0000011790	'RCN2	-	15:76931618-76954392	-	-	IGV_LINK	471.284	16.7767	4.45496	29.0984	OK	459.926	13.9428	3.8239	24.0617	OK	OK	-0.26694
ENSG000001	-	-	ENSG0000011797	'CHRNB4	-	15:78624118-78735495	-	-	IGV_LINK	14.5458	0.731349	0	1.9931	OK	17.5308	1.19682	0	2.95829	OK	OK	0.710576
ENSG000001	-	-	ENSG0000011798	'MUC5B	-	11:1223065-1262172	-	-	IGV_LINK	5.81832	0.042481	0	0.487542	OK	2.06245	0.01509	0	0.457277	OK	NOTEST	-1.49327
ENSG000001	-	-	ENSG0000011798	'CTSD	-	11:1732409-1764573	-	-	IGV_LINK	23941.2	830.171	101.441	1558.9	OK	29132.1	1025.82	33.0621	2018.58	OK	OK	0.3053
ENSG000001	-	-	ENSG0000011800	'COLEC11	-	2:3594831-3644644	-	-	IGV_LINK	108.609	5.8011	0.454465	11.1477	OK	78.3731	3.42197	0.060362	6.78358	OK	OK	-0.7615
ENSG000001	-	-	ENSG0000011800	'STAG1	-	3:136250305-136752403	-	-	IGV_LINK	923.174	9.75233	2.18606	17.3186	OK	752.794	7.9873	1.30147	14.6731	OK	OK	-0.28804
ENSG000001	-	-	ENSG0000011801	'A4GNT	-	3:138123717-138132387	-	-	IGV_LINK	34.9099	1.23078	0	2.51168	OK	20.6245	0.727656	0	1.60747	OK	OK	-0.75824
ENSG000001	-	-	ENSG0000011804	'STK11	-	19:1177557-1238027	-	-	IGV_LINK	1260.64	30.7879	8.99519	52.5806	OK	1157.03	28.5053	8.66936	48.3413	OK	OK	-0.11113
ENSG000001	-	-	ENSG0000011805	'KMT2A	-	11:118436489-118603033	-	-	IGV_LINK	2310.84	15.4104	0	49.0294	OK	1328.22	10.2302	0	37.7656	OK	OK	-0.59108
ENSG000001	-	-	ENSG0000011809	'TREH	-	11:118606439-118679690	-	-	IGV_LINK	286.068	6.2042	0	17.7415	OK	275.337	6.11258	0	18.0262	OK	OK	-0.02146
ENSG000001	-	-	ENSG0000011809	'IFT46	-	11:118436489-118603033	-	-	IGV_LINK	442.193	17.035	0	54.0022	OK	618.735	24.6573	0	68.4819	OK	OK	0.533512
ENSG000001	-	-	ENSG0000011811	'MMP8	-	11:102711794-102727050	-	-	IGV_LINK	0	0	0	0	OK	0	0	0	0	OK	NOTEST	0
ENSG000001	-	-	ENSG0000011813	'APOA1	-	11:116835750-117098437	-	-	IGV_LINK	2.90916	0.215865	0	2.51501	OK	1.03122	0.077074	0	1.32983	OK	NOTEST	-1.48581
ENSG000001	-	-	ENSG0000011815	'ZNF541	-	19:47520684-47555856	-	-	IGV_LINK	7.75776	0.137156	0	0.491954	OK	35.0616	0.823529	0	1.72818	OK	OK	2.58601
ENSG000001	-	-	ENSG0000011816	'SLC8A2	-	19:47428016-47472168	-	-	IGV_LINK	0	0	0	0	OK	10.3122	0.234695	0	0.823717	OK	NOTEST	inf
ENSG000001	-	-	ENSG0000011816	'KPTN	-	19:47475143-47484268	-	-	IGV_LINK	82.4262	3.58199	0	7.35967	OK	81.4668	4.52891	0	9.65837	OK	OK	0.338402
ENSG000001	-	-	ENSG0000011818	'RPS25	-	11:118998141-119030906	-	-	IGV_LINK	12355.2	1066.53	121.953	2011.1	OK	8750.98	757.81	29.0548	1486.57	OK	OK	-0.49301
ENSG000001	-	-	ENSG0000011819	'KIF14	-	1:200551496-200620734	-	-	IGV_LINK	68.8502	0.57381	0	1.15198	OK	60.8423	0.514498	0	1.04379	OK	OK	-0.15741
ENSG000001	-	-	ENSG0000011819	'TNNT2	-	1:201359007-201401190	-	-	IGV_LINK	3.87888	0.351768	0	2.85397	OK	7.21857	0.361201	0	1.86186	OK	NOTEST	0.038177
ENSG000001	-	-	ENSG0000011819	'DDX59	-	1:200623895-200694250	-	-	IGV_LINK	441.223	13.7661	3.43313	24.099	OK	367.116	14.5076	2.43043	26.5847	OK	OK	0.075687
ENSG000001	-	-	ENSG0000011820	'CAMSAP2	-	1:200739557-200860704	-	-	IGV_LINK	490.679	5.1482	0.729414	9.56699	OK	487.769	5.16687	0.069283	10.2645	OK	OK	0.005221
ENSG000001	-	-	ENSG0000011821	'ATF6	-	1:161766293-161964070	-	-	IGV_LINK	1251.91	9.63367	4.20888	15.0585	OK	1339.56	10.3098	4.5462	16.0733	OK	OK	0.097855
ENSG000001	-	-	ENSG0000011823	'CRYGD	-	2:208121606-208124501	-	-	IGV_LINK	0	0	0	0	OK	0	0	0	0	OK	NOTEST	0
ENSG000001	-	-	ENSG0000011824	'MREG	-	2:215942804-216102783	-	-	IGV_LINK	247.085	7.56254	0	16.4447	OK	136.904	4.91292	0	13.068	OK	OK	-0.62229
ENSG000001	-	-	ENSG0000011824	'TNP1	-	2:216672104-216994079	-	-	IGV_LINK	0	0	0	0	OK	0	0	0	0	OK	NOTEST	0
ENSG000001	-	-	ENSG0000011824	'FASTKD2	-	2:206737762-206792509	-	-	IGV_LINK	455.769	9.41173	2.29733	16.5261	OK	555.83	12.1956	3.45685	20.9343	OK	OK	0.373828
ENSG000001	-	-	ENSG0000011825	'NRP2	-	2:205681989-205798133	-	-	IGV_LINK	1083.18	14.1723	0.304164	28.0404	OK	3794.91	52.4673	2.02847	102.906	OK	OK	1.88835
ENSG000001	-	-	ENSG0000011826	'CREB1	-	2:207239649-207625928	-	-	IGV_LINK	644.864	14.8771	0	34.2494	OK	572.33	12.2845	0	28.4883	OK	OK	-0.27625
ENSG000001	-	-	ENSG0000011826	'KLF7	-	2:207074136-207167267	-	-	IGV_LINK	851.415	9.25006	0	20.3037	OK	668.234	5.95691	0.341302	11.5725	OK	OK	-0.6349
ENSG000001	-	-	ENSG0000011827	'TTR	-	18:31591725-31599021	-	-	IGV_LINK	0.969721	0.142505	0	0.664392	OK	0	0	0	0	OK	NOTEST	'-inf
ENSG000001	-	-	ENSG0000011827	'B4GALT6	-	18:31622246-31686823	-	-	IGV_LINK	43.6374	1.0987	0	2.60821	OK	31.968	0.75322	0	1.77952	OK	OK	-0.54466
ENSG000001	-	-	ENSG0000011829	'C1orf54	-	1:150265398-150280916	-	-	IGV_LINK	277.34	41.4406	0	126.244	OK	200.058	30.1612	0	105.799	OK	OK	-0.45835
ENSG000001	-	-	ENSG0000011829	'CA14	-	1:150255094-150265078	-	-	IGV_LINK	2.90916	0.131106	0	0.452714	OK	3.09367	0.112786	0	0.411022	OK	NOTEST	-0.21714
ENSG000001	-	-	ENSG0000011830	'CASC1	-	12:25108419-25195162	-	-	IGV_LINK	95.0326	2.54222	0.098799	4.98564	OK	72.1857	1.86617	0	3.88792	OK	OK	-0.44601
ENSG000001	-	-	ENSG0000011830	'LRMP	-	12:24993423-25108334	-	-	IGV_LINK	427.647	14.5787	1.49617	27.6612	OK	564.08	19.7446	3.08974	36.3995	OK	OK	0.437603
ENSG000001	-	-	ENSG0000011832	'ATP10B	-	5:160563119-160852214	-	-	IGV_LINK	0.969721	0.02353	0	0.565004	OK	2.06245	0.029074	0	0.57217	OK	NOTEST	0.305257
ENSG000001	-	-	ENSG0000011836	'SPCS2	-	11:74949246-74979031	-	-	IGV_LINK	1006.57	77.656	18.7241	136.588	OK	1208.6	94.8216	29.3428	160.3	OK	OK	0.288118
ENSG000001	-	-	ENSG0000011836	'USP35	-	11:78139770-78214711	-	-	IGV_LINK	246.309	4.65627	0.149662	9.16288	OK	359.898	5.77022	0.644853	10.8956	OK	OK	0.309451
ENSG000001	-	-	ENSG0000011840	'ELOVL4	-	6:79914811-79947580	-	-	IGV_LINK	3.87888	0.07618	0	0.194017	OK	4.1249	0.081044	0	0.205306	OK	NOTEST	0.089297
ENSG000001	-	-	ENSG0000011840	'FILIP1	-	6:75285013-75493738	-	-	IGV_LINK	87.2748	0.760612	0.004366	1.51686	OK	133.028	1.25752	0.154553	2.36049	OK	OK	0.725348
ENSG000001	-	-	ENSG0000011841	'CASP8AP2	-	6:89829893-89874436	-	-	IGV_LINK	359.766	4.11809	0.712515	7.52366	OK	202.12	2.00537	0.297245	3.7135	OK	OK	-1.0381
ENSG000001	-	-	ENSG0000011841	'HMGN3	-	6:79201244-79236797	-	-	IGV_LINK	775.776	63.9347	22.911	104.958	OK	520.769	43.0333	15.9955	70.0712	OK	OK	-0.57114
ENSG000001	-	-	ENSG0000011842	'UBE3D	-	6:82892397-83065841	-	-	IGV_LINK	77.5776	3.29537	0	7.03446	OK	44.3427	2.9384	0	6.49489	OK	OK	-0.16541
ENSG000001	-	-	ENSG0000011843	'CNR1	-	6:88139863-88166359	-	-	IGV_LINK	3.87888	0.048786	0	0.317801	OK	21.6557	0.273814	0	0.744993	OK	NOTEST	2.48868
ENSG000001	-	-	ENSG0000011843	'SPACA1	-	6:88047788-88066832	-	-	IGV_LINK	0	0	0	0	OK	0	0	0	0	OK	NOTEST	0
ENSG000001	-	-	ENSG0000011845	'ANKRD13C	-	1:70260587-70354734	-	-	IGV_LINK	364.615	6.99824	0.534699	13.4618	OK	383.616	7.04301	0.954654	13.1314	OK	OK	0.009199
ENSG000001	-	-	ENSG0000011847	'SGIP1	-	1:66533382-66748299	-	-	IGV_LINK	94.0629	2.38147	0	5.61061	OK	22.6869	0.946254	0	2.73397	OK	OK	-1.33155
ENSG000001	-	-	ENSG0000011848	'PHF3	-	6:63635819-65707225	-	-	IGV_LINK	1820.17	17.0109	4.99687	29.0249	OK	1177.66	11.2887	2.91275	19.6647	OK	OK	-0.59158
ENSG000001	-	-	ENSG0000011849	'ZC2HC1B	-	6:143536844-143938471	-	-	IGV_LINK	0	0	0	0	OK	0	0	0	0	OK	NOTEST	0
ENSG000001	-	-	ENSG0000011849	'ADGB	-	6:146598964-146815462	-	-	IGV_LINK	3.87888	0.073575	0	0.765325	OK	4.1249	0.089641	0	0.792329	OK	NOTEST	0.284943
ENSG000001	-	-	ENSG0000011849	'PLAGL1	-	6:143940299-144064599	-	-	IGV_LINK	1058.93	25.8628	3.69795	48.0276	OK	1008.54	24.3799	3.76084	44.999	OK	OK	-0.08519
ENSG000001	-	-	ENSG0000011849	'FBXO30	-	6:145793501-145964423	-	-	IGV_LINK	358.797	2.28825	0.247588	4.32892	OK	180.464	1.15108	0.006013	2.29614	OK	OK	-0.99126
ENSG000001	-	-	ENSG0000011850	'TNFAIP3	-	6:137823672-137883312	-	-	IGV_LINK	2302.12	43.287	0	86.6099	OK	2241.88	38.9875	4.34658	73.6284	OK	OK	-0.15092
ENSG000001	-	-	ENSG0000011850	'AKAP7	-	6:131135665-131283535	-	-	IGV_LINK	244.37	6.48071	1.42777	11.5337	OK	123.747	3.1585	0	6.37981	OK	OK	-1.03691
ENSG000001	-	-	ENSG0000011850	'RAB32	-	6:146543692-146554965	-	-	IGV_LINK	797.11	43.0087	18.03	67.9874	OK	633.172	34.201	13.8508	54.5512	OK	OK	-0.33059
ENSG000001	-	-	ENSG0000011851	'MYB	-	6:135181314-135219173	-	-	IGV_LINK	28.1219	0.661544	0	1.73363	OK	9.28102	0.208505	0	0.810921	OK	OK	-1.66575
ENSG000001	-	-	ENSG0000011851	'ALDH8A1	-	6:134917389-134950122	-	-	IGV_LINK	351.039	8.43175	2.61059	14.2529	OK	1302.44	32.7751	11.9967	53.5536	OK	OK	1.9587
ENSG000001	-	-	ENSG0000011851	'SGK1	-	6:134169245-134318112	-	-	IGV_LINK	2326.36	55.1832	7.95248	102.414	OK	1934.58	54.6955	10.6333	98.7577	OK	OK	-0.01281
ENSG000001	-	-	ENSG0000011851	'RNF146	-	6:127266609-127288567	-	-	IGV_LINK	622.561	20.1416	6.35624	33.927	OK	475.395	14.957	4.20288	25.7111	OK	OK	-0.42936
ENSG000001	-	-	ENSG0000011852	'ARG1	-	6:131573143-131628229	-	-	IGV_LINK	0	0	0	0	OK	1.03122	0.111906	0	1.75386	OK	NOTEST	inf
ENSG000001	-	-	ENSG0000011852	'CTGF	-	6:131948175-132077393	-	-	IGV_LINK	165.822	4.31345	0.993619	7.63328	OK	351.648	9.15209	3.17714	15.127	OK	OK	1.08526
ENSG000001	-	-	ENSG0000011852	'TCF21	-	6:133240597-133895553	-	-	IGV_LINK	36.8494	0.520152	0	1.22554	OK	8.2498	0.106211	0	0.256439	OK	NOTEST	-2.292
ENSG000001	-	-	ENSG0000011855	'PMFBP1	-	16:72044288-72176878	-	-	IGV_LINK	163.883	10.5883	0	30.8859	OK	167.058	10.8496	0	169.012	OK	OK	0.035168
ENSG000001	-	-	ENSG0000011856	'FBXL5	-	4:15604538-15738313	-	-	IGV_LINK	3566.63	85.0664	25.312	144.821	OK	3577.32	86.2615	23.4307	149.092	OK	OK	0.020127
ENSG000001	-	-	ENSG0000011857	'MED28	-	4:17614630-17781512	-	-	IGV_LINK	1569.98	30.9774	0	84.7103	OK	1418.97	37.4189	0	95.7926	OK	OK	0.27255
ENSG000001	-	-	ENSG0000011859	'SLC16A7	-	12:59596066-59789855	-	-	IGV_LINK	149.337	1.73918	0	5.16618	OK	142.309	0.987345	0	3.19366	OK	OK	-0.81678
ENSG000001	-	-	ENSG0000011860	'TMEM5	-	12:63558912-63823895	-	-	IGV_LINK	287.037	10.5249	0	22.956	OK	324.836	11.2788	1.67249	20.8851	OK	OK	0.09981
ENSG000001	-	-	ENSG0000011862	'ZNF430	-	19:21020619-21060050	-	-	IGV_LINK	122.185	2.54063	0	5.91585	OK	120.653	3.54868	0	8.87944	OK	OK	0.4821
ENSG000001	-	-	ENSG0000011864	'VAMP8	-	2:85561561-85582031	-	-	IGV_LINK	2433.03	228.933	99.7528	358.113	OK	2484.22	233.763	97.8473	369.679	OK	OK	0.030124
ENSG000001	-	-	ENSG0000011865	'DCLRE1B	-	1:113761831-113914086	-	-	IGV_LINK	206.55	3.12089	0	7.5133	OK	180.464	2.70028	0	8.11406	OK	OK	-0.20885
ENSG000001	-	-	ENSG0000011868	'MYL12B	-	18:3261908-3278284	-	-	IGV_LINK	4417.08	239.927	100.039	379.815	OK	5013.82	273.266	109.584	436.948	OK	OK	0.187711
ENSG000001	-	-	ENSG0000011868	'FOXO3	-	6:108559834-108684774	-	-	IGV_LINK	1401.25	11.0539	4.89318	17.2146	OK	1048.76	8.26597	3.41856	13.1134	OK	OK	-0.4193
ENSG000001	-	-	ENSG0000011869	'ARMC2	-	6:108848415-108974472	-	-	IGV_LINK	97.9418	3.82964	0	8.83956	OK	58.7798	2.72557	0	6.60857	OK	OK	-0.49065
ENSG000001	-	-	ENSG0000011870	'GHRH	-	20:37251081-37261835	-	-	IGV_LINK	0	0	0	0	OK	0	0	0	0	OK	NOTEST	0
ENSG000001	-	-	ENSG0000011870	'RPN2	-	20:37101225-37241623	-	-	IGV_LINK	3685.91	101.083	27.6358	174.53	OK	5889.33	152.807	44.1609	261.452	OK	OK	0.596165
ENSG000001	-	-	ENSG0000011870	'TGIF2	-	20:36573487-36646216	-	-	IGV_LINK	562.801	10.0399	0.758791	19.321	OK	349.631	6.09701	0	14.5984	OK	OK	-0.71957
ENSG000001	-	-	ENSG0000011872	'CASQ2	-	1:115700006-115768781	-	-	IGV_LINK	42.6677	0.961255	0	2.05898	OK	116.528	2.62646	0.34943	4.90349	OK	OK	1.45013
ENSG000001	-	-	ENSG0000011873	'OLFM3	-	1:101802573-101997030	-	-	IGV_LINK	0	0	0	0	OK	0	0	0	0	OK	NOTEST	0
ENSG000001	-	-	ENSG0000011876	'PKD2	-	4:88007667-88077777	-	-	IGV_LINK	1933.62	42.6786	10.1939	75.1634	OK	2337.79	52.9895	12.705	93.2741	OK	OK	0.312193
ENSG000001	-	-	ENSG0000011877	'ABCG2	-	4:88090263-88231322	-	-	IGV_LINK	77.5776	1.91323	0	5.70138	OK	153.653	3.09881	0	8.04628	OK	OK	0.695704
ENSG000001	-	-	ENSG0000011878	'SPP1	-	4:87975649-87983426	-	-	IGV_LINK	40779.7	1951.87	0	3908.65	OK	72561.1	3490.03	0	8779.24	OK	OK	0.838382
ENSG000001	-	-	ENSG0000011880	'STBD1	-	4:76158732-76311599	-	-	IGV_LINK	174.761	3.03274	0	11.7284	OK	502.423	8.72193	0	26.8301	OK	OK	1.52403
ENSG000001	-	-	ENSG0000011881	'CCNI	-	4:77047157-77076005	-	-	IGV_LINK	4817.57	149.618	26.9999	272.237	OK	2942.08	86.627	20.05	153.204	OK	OK	-0.7884
ENSG000001	-	-	ENSG0000011884	'RARRES1	-	3:158696891-158829719	-	-	IGV_LINK	116.366	6.49217	0	29.5656	OK	2656.44	141.606	0	337.73	OK	OK	4.44704
ENSG000001	-	-	ENSG0000011885	'MFSD1	-	3:158696891-158829719	-	-	IGV_LINK	1495.31	51.1165	19.0018	83.2312	OK	2184.75	84.187	0	171.989	OK	OK	0.719809
ENSG000001	-	-	ENSG0000011887	'RAB3GAP2	-	1:220148292-220272454	-	-	IGV_LINK	848.505	10.2234	0	20.8405	OK	747.638	9.18599	0	18.7607	OK	OK	-0.15437
ENSG000001	-	-	ENSG0000011889	'EEF2KMT	-	16:4882506-5097808	-	-	IGV_LINK	134.791	4.48123	0	14.3209	OK	176.339	4.9478	0	16.7622	OK	OK	0.142893
ENSG000001	-	-	ENSG0000011889	'PPL	-	16:4882506-5097808	-	-	IGV_LINK	493.588	4.95305	0	11.0774	OK	1770.61	18.3941	0	37.6798	OK	OK	1.89285
ENSG000001	-	-	ENSG0000011890	'UBN1	-	16:4803202-4882360	-	-	IGV_LINK	856.263	10.0576	0	27.3046	OK	1092.07	12.1806	0	31.8649	OK	OK	0.2763
ENSG000001	-	-	ENSG0000011890	'BTF3P11	-	13:76928065-76928836	-	-	IGV_LINK	0	0	0	0	OK	0	0	0	0	OK	NOTEST	0
ENSG000001	-	-	ENSG0000011892	'KLF12	-	13:73686088-73995056	-	-	IGV_LINK	685.592	3.9331	1.01574	6.85045	OK	458.895	2.47217	0.874597	4.06974	OK	OK	-0.66989
ENSG000001	-	-	ENSG0000011893	'UCHL3	-	13:75549479-75859870	-	-	IGV_LINK	197.082	16.9883	0	47.1778	OK	208.253	17.9313	0	41.4454	OK	OK	0.077934
ENSG000001	-	-	ENSG0000011894	'PCDH17	-	13:57631809-57729311	-	-	IGV_LINK	320.008	3.51139	0.189834	6.83294	OK	348.554	3.2385	0.366468	6.11052	OK	OK	-0.11672
ENSG000001	-	-	ENSG0000011896	'HS1BP3	-	2:20560447-20651089	-	-	IGV_LINK	1205.36	31.3788	12.6342	50.1235	OK	1124.04	28.966	11.617	46.315	OK	OK	-0.11543
ENSG000001	-	-	ENSG0000011896	'LDAH	-	2:20684013-20823130	-	-	IGV_LINK	292.856	5.72434	1.7014	9.74727	OK	292.868	7.53229	0	15.2401	OK	OK	0.39598
ENSG000001	-	-	ENSG0000011896	'WDR35	-	2:19910259-19990131	-	-	IGV_LINK	847.536	8.1729	2.17972	14.1661	OK	604.298	5.9685	2.05657	9.88042	OK	OK	-0.45348
ENSG000001	-	-	ENSG0000011897	'CCND2	-	12:4248764-4305350	-	-	IGV_LINK	1225.73	11.4951	1.31268	21.6775	OK	691.952	6.53395	1.41528	11.6526	OK	OK	-0.81499
ENSG000001	-	-	ENSG0000011897	'FGF23	-	12:4368226-4379728	-	-	IGV_LINK	0	0	0	0	OK	0	0	0	0	OK	NOTEST	0
ENSG000001	-	-	ENSG0000011897	'OTUD4P1	-	12:3791046-3910338	-	-	IGV_LINK	0.969721	0.01888	0	0.224931	OK	1.03122	0.020086	0	0.226219	OK	NOTEST	0.089292
ENSG000001	-	-	ENSG0000011898	'ELL2	-	5:95885097-96808100	-	-	IGV_LINK	498.436	6.99299	0	37.8046	OK	1219.94	20.1327	0	85.4537	OK	OK	1.52556
ENSG000001	-	-	ENSG0000011899	'GLRXP3	-	5:161751447-161751768	-	-	IGV_LINK	0	0	0	0	OK	0	0	0	0	OK	NOTEST	0
ENSG000001	-	-	ENSG0000011899	'DNAH7	-	2:195737702-196068812	-	-	IGV_LINK	238.551	3.97449	0	8.6337	OK	111.372	2.20461	0	5.29312	OK	OK	-0.85025
ENSG000001	-	-	ENSG0000011900	'CYP20A1	-	2:203238939-203305611	-	-	IGV_LINK	123.155	4.31465	0.230969	8.39832	OK	124.778	4.4898	0	9.01865	OK	OK	0.05741
ENSG000001	-	-	ENSG0000011901	'NDUFB3	-	2:200908972-201085750	-	-	IGV_LINK	548.862	56.1098	1.32696	110.893	OK	671.327	72.074	11.4638	132.684	OK	OK	0.361226
ENSG000001	-	-	ENSG0000011904	'GTF3C3	-	2:196639553-196799725	-	-	IGV_LINK	602.196	13.0229	0	28.8294	OK	592.954	10.8351	0	21.9822	OK	OK	-0.26534
ENSG000001	-	-	ENSG0000011904	'SATB2	-	2:199269499-199476935	-	-	IGV_LINK	187.156	2.43093	0.279081	4.58278	OK	48.4676	0.687113	0	1.60289	OK	OK	-1.82289
ENSG000001	-	-	ENSG0000011904	'UBE2B	-	5:134371178-134391992	-	-	IGV_LINK	773.837	56.2849	12.3635	100.206	OK	637.297	43.8232	11.3929	76.2535	OK	OK	-0.36105
ENSG000001	-	-	ENSG0000011912	'TRPM6	-	9:74722494-74888094	-	-	IGV_LINK	4.8486	0.03374	0	0.246709	OK	23.7182	0.165602	0	0.443545	OK	NOTEST	2.29518
ENSG000001	-	-	ENSG0000011912	'GDA	-	9:72114594-72257193	-	-	IGV_LINK	119.276	1.33659	0.149267	2.52391	OK	2938.99	36.4524	13.0784	59.8265	OK	OK	4.76939
ENSG000001	-	-	ENSG0000011913	'KLF9	-	9:70384596-70414624	-	-	IGV_LINK	992.024	11.1756	4.81655	17.5347	OK	1021.94	11.5154	4.99468	18.036	OK	OK	0.043201
ENSG000001	-	-	ENSG0000011913	'TJP2	-	9:69121263-69255208	-	-	IGV_LINK	1856.05	26.1022	9.06566	43.1387	OK	1902.61	26.3452	8.90286	43.7875	OK	OK	0.01337
ENSG000001	-	-	ENSG0000011914	'C2orf40	-	2:106063245-106078159	-	-	IGV_LINK	23.2733	2.3296	0	4.92472	OK	4.1249	0.392293	0	1.15935	OK	OK	-2.57008
ENSG000001	-	-	ENSG0000011918	'ITGB1BP1	-	2:9206764-9423547	-	-	IGV_LINK	841.717	43.7267	6.37373	81.0796	OK	864.167	48.6039	7.37336	89.8345	OK	OK	0.15256
ENSG000001	-	-	ENSG0000011920	'CPSF3	-	2:9423567-9473101	-	-	IGV_LINK	443.162	13.7461	2.87887	24.6133	OK	530.05	14.9232	4.45194	25.3944	OK	OK	0.118536
ENSG000001	-	-	ENSG0000011922	'PIGZ	-	3:196946342-196969060	-	-	IGV_LINK	60.1227	1.49316	0	3.11823	OK	120.653	3.74641	0	7.53738	OK	OK	1.32714

ENSG0000011	-	-	ENSG0000011923	'SENP5	-	3:196867855-196934714	-	-	IGV_LINK	596.378	7.84583	0	15.8808	OK	592.954	9.01582	0	19.2188	OK	OK	0.200532
ENSG0000011	-	-	ENSG0000011924	'CCDC92	-	12:123762187-124316024	-	-	IGV_LINK	668.137	17.1199	0	40.2457	OK	578.517	16.1368	0	38.181	OK	OK	-0.08532
ENSG0000011	-	-	ENSG0000011928	'C1orf198	-	1:230837118-230869589	-	-	IGV_LINK	357.827	5.7487	1.3819	10.1155	OK	234.088	3.58696	1.00112	6.17279	OK	OK	-0.68048
ENSG0000011	-	-	ENSG0000011928	'TRIM67	-	1:231162111-231221556	-	-	IGV_LINK	2.90916	0.017925	0	0.08482	OK	4.1249	0.025416	0	0.09656	OK	NOTEST	0.503743
ENSG0000011	-	-	ENSG0000011928	'HEATR1	-	1:236517999-236604504	-	-	IGV_LINK	1143.15	7.78571	1.38484	14.1866	OK	1238.27	8.43471	1.40385	15.4656	OK	OK	0.115509
ENSG0000011	-	-	ENSG0000011931	'PTBP3	-	9:112217715-112333667	-	-	IGV_LINK	1172.39	11.3958	0	23.3962	OK	1105.47	10.587	1.50039	19.6736	OK	OK	-0.1062
ENSG0000011	-	-	ENSG0000011931	'RAD23B	-	9:107283136-107332194	-	-	IGV_LINK	1356.64	21.3272	6.70891	35.9454	OK	1950.05	33.9609	8.03351	59.8883	OK	OK	0.671182
ENSG0000011	-	-	ENSG0000011932	'FKBP15	-	9:113150941-113221361	-	-	IGV_LINK	2529.03	24.8738	9.21544	40.5323	OK	2960.65	26.216	8.8149	43.6171	OK	OK	0.075817
ENSG0000011	-	-	ENSG0000011932	'CTNNAL1	-	9:108934180-109013529	-	-	IGV_LINK	140.609	3.41946	0	7.34168	OK	187.683	4.72675	0	9.83927	OK	OK	0.46708
ENSG0000011	-	-	ENSG0000011932	'FAM206A	-	9:108934180-109013529	-	-	IGV_LINK	338.432	10.7967	1.40355	20.1898	OK	365.054	11.0916	1.87845	20.3048	OK	OK	0.038886
ENSG0000011	-	-	ENSG0000011933	'WDR34	-	9:128552557-128656787	-	-	IGV_LINK	1037.6	41.6472	0	100.24	OK	951.821	36.587	0	87.965	OK	OK	-0.18689
ENSG0000011	-	-	ENSG0000011933	'SET	-	9:128683423-128696400	-	-	IGV_LINK	3463.84	136.266	14.4322	258.1	OK	2934.87	115.42	28.3904	202.45	OK	OK	-0.23953
ENSG0000011	-	-	ENSG0000011938	'PTPA	-	9:129094809-129148946	-	-	IGV_LINK	3783.85	146.156	33.7152	258.596	OK	5453.12	221.464	40.3596	402.567	OK	OK	0.599562
ENSG0000011	-	-	ENSG0000011939	'GLE1	-	9:128504699-128552410	-	-	IGV_LINK	839.778	15.1904	6.32526	24.0555	OK	864.167	15.628	6.5708	24.6852	OK	OK	0.040974
ENSG0000011	-	-	ENSG0000011939	'RAB14	-	9:121178136-121332843	-	-	IGV_LINK	1151.06	19.7443	0	66.3617	OK	1154.97	19.222	0	55.6241	OK	OK	-0.03867
ENSG0000011	-	-	ENSG0000011939	'CNTRL	-	9:121074862-121177610	-	-	IGV_LINK	724.381	10.2489	2.93103	17.5667	OK	480.551	5.98916	1.59749	10.3808	OK	OK	-0.77504
ENSG0000011	-	-	ENSG0000011940	'TRIM32	-	9:116425224-117415070	-	-	IGV_LINK	261.825	4.19722	0.505227	7.88921	OK	312.461	4.99587	0	10.2408	OK	OK	0.251302
ENSG0000011	-	-	ENSG0000011940	'FBXW2	-	9:120751977-120793412	-	-	IGV_LINK	1379.91	18.0638	0.616486	35.5112	OK	1321	18.3893	0	37.1349	OK	OK	0.025759
ENSG0000011	-	-	ENSG0000011940	'PHF19	-	9:120855651-120894896	-	-	IGV_LINK	516.861	17.2854	0.704921	33.8659	OK	322.773	10.2478	0.167176	20.3284	OK	OK	-0.75424
ENSG0000011	-	-	ENSG0000011940	'NEK6	-	9:124257605-124353307	-	-	IGV_LINK	2846.13	66.3175	18.5275	114.107	OK	3781.5	101.171	24.0546	178.287	OK	OK	0.60933
ENSG0000011	-	-	ENSG0000011941	'BSPRY	-	9:113349540-113371233	-	-	IGV_LINK	46.5466	1.22343	0	2.53741	OK	6.18735	0.161035	0	0.392147	OK	OK	-2.92549
ENSG0000011	-	-	ENSG0000011941	'PPP6C	-	9:125146572-125189939	-	-	IGV_LINK	784.504	13.3581	1.48666	25.2296	OK	777.544	12.3425	2.53231	22.1527	OK	OK	-0.11408
ENSG0000011	-	-	ENSG0000011942	'NDUFA8	-	9:122144057-122159819	-	-	IGV_LINK	611.894	51.355	20.5911	82.119	OK	754.857	63.4624	25.672	101.253	OK	OK	0.305395
ENSG0000011	-	-	ENSG0000011943	'HDHD3	-	9:113373418-113376999	-	-	IGV_LINK	583.772	23.8647	8.38301	39.3464	OK	857.979	38.7239	15.3193	62.1285	OK	OK	0.698347
ENSG0000011	-	-	ENSG0000011944	'LCN1P1	-	9:133224904-133228591	-	-	IGV_LINK	0	0	0	0	OK	0	0	0	0	OK	NOTEST	0
ENSG0000011	-	-	ENSG0000011944	'RBM18	-	9:122237623-122331343	-	-	IGV_LINK	464.496	10.1174	0	25.2649	OK	516.644	11.562	0	27.8323	OK	OK	0.192559
ENSG0000011	-	-	ENSG0000011945	'SLC46A2	-	9:112750837-112890913	-	-	IGV_LINK	44.6071	1.13203	0	4.61081	OK	57.7486	1.44679	0	5.70398	OK	OK	0.353938
ENSG0000011	-	-	ENSG0000011947	'HSDL2	-	9:112379936-112669397	-	-	IGV_LINK	771.898	15.6575	2.47948	28.8356	OK	1664.4	31.6455	9.92366	53.3674	OK	OK	1.01514
ENSG0000011	-	-	ENSG0000011948	'MAPKAP1	-	9:125437392-125707234	-	-	IGV_LINK	1257.73	42.5207	6.708	78.3334	OK	1252.94	43.5242	6.43397	80.6145	OK	OK	0.033652
ENSG0000011	-	-	ENSG0000011950	'NR4A3	-	9:99821854-99866891	-	-	IGV_LINK	80.4868	1.06783	0	2.13602	OK	93.8415	1.33835	0.042588	2.63412	OK	OK	0.325783
ENSG0000011	-	-	ENSG0000011950	'INVS	-	9:100099255-100301000	-	-	IGV_LINK	464.496	9.80863	1.7639	17.8534	OK	558.924	11.7739	2.66874	20.879	OK	OK	0.263464
ENSG0000011	-	-	ENSG0000011951	'GALNT12	-	9:98807698-98872415	-	-	IGV_LINK	144.488	4.34626	0	9.29393	OK	184.589	5.11685	0	10.3494	OK	OK	0.235483
ENSG0000011	-	-	ENSG0000011952	'DENND1A	-	9:123356169-123930152	-	-	IGV_LINK	708.866	9.4944	0.741362	18.2474	OK	698.139	10.8882	1.43289	20.3434	OK	OK	0.197612
ENSG0000011	-	-	ENSG0000011952	'ALG2	-	9:99216425-99221956	-	-	IGV_LINK	490.679	15.9827	5.74624	26.2192	OK	667.203	21.9656	8.25602	35.6752	OK	OK	0.458734
ENSG0000011	-	-	ENSG0000011953	'CSF3R	-	1:36466042-36483278	-	-	IGV_LINK	1881.26	50.4756	12.3123	88.6389	OK	4065.09	113.295	21.4451	205.144	OK	OK	1.16642
ENSG0000011	-	-	ENSG0000011953	'KDSR	-	18:63327725-63381629	-	-	IGV_LINK	1066.69	27.9127	4.83681	50.9885	OK	1144.66	31.9492	4.4147	59.4837	OK	OK	0.19486
ENSG0000011	-	-	ENSG0000011954	'VPS4B	-	18:63389189-63422483	-	-	IGV_LINK	677.835	15.5341	3.10319	27.965	OK	794.043	15.813	4.74499	26.881	OK	OK	0.025674
ENSG0000011	-	-	ENSG0000011954	'ONECUT2	-	18:57435684-57491297	-	-	IGV_LINK	2.90916	0.01028	0	0.268537	OK	145.403	0.51383	0.008413	1.01925	OK	NOTEST	5.64341
ENSG0000011	-	-	ENSG0000011955	'C19orf25	-	19:1446301-1479556	-	-	IGV_LINK	523.649	23.2254	6.8166	39.6342	OK	511.488	25.976	5.29546	46.6566	OK	OK	0.161478
ENSG0000011	-	-	ENSG0000011957	'ZBTB45	-	19:58513529-58538911	-	-	IGV_LINK	154.186	4.16662	0.630201	7.70304	OK	168.09	4.5702	0.551088	8.58932	OK	OK	0.13338
ENSG0000011	-	-	ENSG0000011959	'YLPM1	-	14:74763365-74871940	-	-	IGV_LINK	965.842	13.2345	0	27.0686	OK	912.634	10.8957	0.139809	21.6517	OK	OK	-0.28054
ENSG0000011	-	-	ENSG0000011959	'DCAF4	-	14:72926331-72959703	-	-	IGV_LINK	144.488	5.42511	0	11.5206	OK	119.622	4.55147	0	9.82435	OK	OK	-0.25332
ENSG0000011	-	-	ENSG0000011960	'PROX2	-	14:74763365-74871940	-	-	IGV_LINK	24.243	0.532428	0	3.53802	OK	11.3435	0.200691	0	1.25667	OK	NOTEST	-1.40761
ENSG0000011	-	-	ENSG0000011961	'VSX2	-	14:74239471-74262738	-	-	IGV_LINK	0	0	0	0	OK	0	0	0	0	OK	NOTEST	0
ENSG0000011	-	-	ENSG0000011961	'FCF1	-	14:74713143-74738620	-	-	IGV_LINK	343.281	16.4051	2.69234	30.118	OK	366.085	17.0118	1.27622	32.7473	OK	OK	0.052385
ENSG0000011	-	-	ENSG0000011963	'PGF	-	14:74941833-74955784	-	-	IGV_LINK	748.624	25.4535	9.2577	41.6494	OK	273.275	9.49064	2.58689	16.3944	OK	OK	-1.42329
ENSG0000011	-	-	ENSG0000011963	'IFI27L2	-	14:94127778-94130253	-	-	IGV_LINK	581.832	90.6567	35.0277	146.286	OK	511.488	81.7391	30.6931	132.785	OK	OK	-0.14939
ENSG0000011	-	-	ENSG0000011963	'BBOF1	-	14:73851843-74084493	-	-	IGV_LINK	138.67	5.51805	0	22.5651	OK	136.122	4.19185	0	18.3068	OK	OK	-0.39657
ENSG0000011	-	-	ENSG0000011963	'NEK9	-	14:75053220-75176631	-	-	IGV_LINK	1810.47	28.8966	0	66.0117	OK	1385.97	22.2478	0	56.7031	OK	OK	-0.37724
ENSG0000011	-	-	ENSG0000011964	'ACYP1	-	14:75053220-75176631	-	-	IGV_LINK	79.5171	9.92122	0	50.78	OK	65.9984	8.15017	0	44.8536	OK	OK	-0.28369
ENSG0000011	-	-	ENSG0000011965	'IFT43	-	14:75574887-76084585	-	-	IGV_LINK	332.614	26.2324	0	56.5256	OK	362.991	29.6179	0	67.6057	OK	OK	0.175122
ENSG0000011	-	-	ENSG0000011965	'NPC2	-	14:74476191-74497106	-	-	IGV_LINK	3679.12	245.213	92.9175	397.509	OK	3626.82	264.394	93.4448	435.344	OK	OK	0.108654
ENSG0000011	-	-	ENSG0000011966	'DPPA5P4	-	14:75292130-75292495	-	-	IGV_LINK	0	0	0	0	OK	0	0	0	0	OK	NOTEST	0
ENSG0000011	-	-	ENSG0000011966	'DNAL1	-	14:73644874-73703732	-	-	IGV_LINK	310.311	5.7886	0	14.6184	OK	247.494	5.03348	0	12.7141	OK	OK	-0.20166
ENSG0000011	-	-	ENSG0000011966	'IRF2BPL	-	14:77024542-77031572	-	-	IGV_LINK	308.371	4.36152	1.0196	7.70344	OK	364.022	5.15012	1.4722	8.82804	OK	OK	0.239776
ENSG0000011	-	-	ENSG0000011967	'ACOT2	-	14:73567619-73575658	-	-	IGV_LINK	147.398	5.28899	0.790022	9.78795	OK	173.246	6.83561	0.289796	13.3814	OK	OK	0.370079
ENSG0000011	-	-	ENSG0000011968	'LTBP2	-	14:74498169-74612378	-	-	IGV_LINK	99.8812	0.79258	0	1.94514	OK	416.615	3.21421	0.18902	6.23939	OK	OK	2.01983
ENSG0000011	-	-	ENSG0000011968	'AREL1	-	14:74653436-74713115	-	-	IGV_LINK	535.286	6.96655	0.450411	13.4827	OK	514.581	6.4846	0.579411	12.3898	OK	OK	-0.10343
ENSG0000011	-	-	ENSG0000011968	'MLH3	-	14:75013763-75051532	-	-	IGV_LINK	537.225	11.7442	0.116049	23.3723	OK	492.926	13.72	0.589933	26.85	OK	OK	0.224333
ENSG0000011	-	-	ENSG0000011968	'TTLL5	-	14:75574887-76084585	-	-	IGV_LINK	335.523	6.20869	0	16.9815	OK	359.898	9.32447	0	30.2987	OK	OK	0.586733
ENSG0000011	-	-	ENSG0000011968	'FLVCR2	-	14:75574887-76084585	-	-	IGV_LINK	265.703	7.13925	0	20.8361	OK	732.17	18.7367	0	43.0487	OK	OK	1.39202
ENSG0000011	-	-	ENSG0000011968	'ABCD4	-	14:74285422-74303056	-	-	IGV_LINK	1109.08	45.3687	13.6532	77.0842	OK	1330.68	54.6744	16.4769	92.8718	OK	OK	0.269168
ENSG0000011	-	-	ENSG0000011968	'DLST	-	14:74881890-74903745	-	-	IGV_LINK	1313.97	32.0643	10.6057	53.523	OK	1346.78	32.0544	10.9206	53.1881	OK	OK	-0.00045
ENSG0000011	-	-	ENSG0000011969	'PPP4R4	-	14:94146127-94279735	-	-	IGV_LINK	0.969721	0.014788	0	0.423164	OK	2.06245	0.031462	0	0.441931	OK	NOTEST	1.08917
ENSG0000011	-	-	ENSG0000011969	'TGFB3	-	14:75574887-76084585	-	-	IGV_LINK	178.429	5.55048	0	16.0337	OK	101.06	2.96218	0	11.6754	OK	OK	-0.90595
ENSG0000011	-	-	ENSG0000011970	'ZC2HC1C	-	14:75053220-75176631	-	-	IGV_LINK	93.0932	3.32088	0	18.531	OK	84.5604	3.87609	0	23.2651	OK	OK	0.223037
ENSG0000011	-	-	ENSG0000011970	'SLIRP	-	14:77708070-77769742	-	-	IGV_LINK	298.674	69.8967	0	146.139	OK	318.649	73.6996	0	167.997	OK	OK	0.076433
ENSG0000011	-	-	ENSG0000011970	'RBM25	-	14:73057924-73123898	-	-	IGV_LINK	2125.63	30.8087	9.0239	52.5935	OK	1752.05	27.9842	9.63988	46.3286	OK	OK	-0.13872
ENSG0000011	-	-	ENSG0000011971	'ALDH6A1	-	14:73851843-74084493	-	-	IGV_LINK	346.19	6.81767	0	17.9263	OK	308.336	6.33311	0	17.6284	OK	OK	-0.10637
ENSG0000011	-	-	ENSG0000011971	'GPR68	-	14:91232531-91253925	-	-	IGV_LINK	79.5171	1.85577	0	4.39058	OK	79.4043	1.66641	0	3.34511	OK	OK	-0.15527
ENSG0000011	-	-	ENSG0000011971	'ESRRB	-	14:76310613-76761388	-	-	IGV_LINK	3.87888	0.130628	0	0.588913	OK	14.4371	0.428048	0	1.27518	OK	NOTEST	1.71231
ENSG0000011	-	-	ENSG0000011971	'EIF2B2	-	14:75002910-75012851	-	-	IGV_LINK	358.797	20.4692	4.27718	36.6612	OK	409.396	22.7518	5.24013	40.2634	OK	OK	0.152524
ENSG0000011	-	-	ENSG0000011972	'NRDE2	-	14:90256494-90332137	-	-	IGV_LINK	630.318	5.81008	0	15.7381	OK	650.703	7.20957	0	17.4269	OK	OK	0.311356
ENSG0000011	-	-	ENSG0000011972	'COQ6	-	14:73851843-74084493	-	-	IGV_LINK	309.341	14.0184	0	39.0751	OK	312.461	14.0814	0	40.9774	OK	OK	0.006477
ENSG0000011	-	-	ENSG0000011972	'ZNF410	-	14:73851843-74084493	-	-	IGV_LINK	302.431	14.7947	0	40.8677	OK	360.655	16.5385	0	45.8031	OK	OK	0.160747
ENSG0000011	-	-	ENSG0000011972	'RHOQ	-	2:46490749-46625742	-	-	IGV_LINK	798.08	24.3614	0	50.8704	OK	973.476	28.4916	0	61.2179	OK	OK	0.22594
ENSG0000011	-	-	ENSG0000011973	'GPR75	-	2:53532671-53860160	-	-	IGV_LINK	21.3339	0.618941	0	3.12677	OK	25.7806	0.748395	0	3.91246	OK	OK	0.273997
ENSG0000011	-	-	ENSG0000011976	'SUPT7L	-	2:27582968-27694976	-	-	IGV_LINK	1059.9	21.2549	0	45.348	OK	1346.78	28.2276	0	57.7664	OK	OK	0.40931
ENSG0000011	-	-	ENSG0000011977	'KLHL29	-	2:23385216-23708611	-	-	IGV_LINK	244.37	2.69287	0.574232	4.8115	OK	143.34	1.56138	0.223082	2.89967	OK	OK	-0.78632
ENSG0000011	-	-	ENSG0000011977	'DNMT3A	-	2:25227854-25342590	-	-	IGV_LINK	773.837	9.06432	0	18.7174	OK	581.611	7.19503	0	14.5061	OK	OK	-0.3332
ENSG0000011	-	-	ENSG0000011977	'TMEM214	-	2:27032909-27041695	-	-	IGV_LINK	1614.58	40.6974	12.4408	68.954	OK	2750.28	70.8548	19.3591	122.35	OK	OK	0.799928
ENSG0000011	-	-	ENSG0000011977	'ATAD2B	-	2:23748663-23927114	-	-	IGV_LINK	336.493	3.02824	0	6.07122	OK	232.026	1.8776	0	3.891	OK	OK	-0.68959
ENSG0000011	-	-	ENSG0000011978	'FKBP1B	-	2:24010080-24063681	-	-	IGV_LINK	31.0311	2.101	0	6.26631	OK	18.562	1.17674	0	4.17652	OK	OK	-0.83628
ENSG0000011	-	-	ENSG0000011978	'ATL2	-	2:38294879-38377285	-	-	IGV_LINK	665.228	21.7095	4.30768	39.1112	OK	634.203	22.209	3.78842	40.6297	OK	OK	0.032823
ENSG0000011	-	-	ENSG0000011980	'YPEL5	-	2:30146940-30160533	-	-	IGV_LINK	1200.51	43.6043	13.9036	73.3051	OK	1470.53	59.5781	16.9571	102.199	OK	OK	0.45031
ENSG0000011	-	-	ENSG0000011981	'FAM98A	-	2:33583657-33599382	-	-	IGV_LINK	368.494	11.6132	0	25.7114	OK	517.675	24.4085	0	62.8271	OK	OK	1.07162
ENSG0000011	-	-	ENSG0000011982	'YIPF4	-	2:32277909-32316594	-	-	IGV_LINK	830.081	17.6622	0	45.461	OK	882.729	20.9497	0	45.9423	OK	OK	0.246263
ENSG0000011	-	-	ENSG0000011984	'AFTPH	-	2:64524304-64593005	-	-	IGV_LINK	1140.39	24.5753	8.18726	40.9634	OK	1125.07	23.6394	8.70506	38.5737	OK	OK	-0.05602
ENSG0000011	-	-	ENSG0000011986	'LGALSL	-	2:64453968-64461381	-	-	IGV_LINK	235.642	3.87939	0.377452	7.38132	OK	131.997	2.20936	0.022409	4.3963	OK	OK	-0.8122
ENSG0000011	-	-	ENSG0000011986	'CNRIP1	-	2:68284170-68320051	-	-	IGV_LINK	224.975	8.24928	1.80216	14.6964	OK	194.902	7.04231	1.65017	12.4344	OK	OK	-0.22822
ENSG0000011	-	-	ENSG0000011986	'BCL11A	-	2:60451166-60553567	-	-	IGV_LINK	59.153	2.07959	0	5.08265	OK	30.9367	1.46699	0	4.04885	OK	OK	-0.50344
ENSG0000011	-	-	ENSG0000011987	'CRIPT	-	2:46490749-46625742	-	-	IGV_LINK	172.61	5.54187	0	14.7427	OK	228.932	7.25082	0	21.3027	OK	OK	0.38777
ENSG0000011	-	-	ENSG0000011988	'EPCAM	-	2:47345157-47387601	-	-	IGV_LINK	424.738	19.3525	4.81225	33.8927	OK	301.118	14.4243	2.13879	26.7098	OK	OK	-0.42402
ENSG0000011	-	-	ENSG0000011989	'SLC17A5	-	6:73593378-73654155	-	-	IGV_LINK	529.467	9.0902	3.52264	14.6578	OK	430.021	7.38555	2.69191	12.0792	OK	OK	-0.29961
ENSG0000011	-	-	ENSG0000011990	'OGFRL1	-	6:71221456-71420769	-	-	IGV_LINK	1384.76	10.2833	1.50681	19.0598	OK	912.634	6.42298	1.70556	11.1404	OK	OK	-0.67899
ENSG0000011	-	-	ENSG0000011990	'SLF2	-	10:100911102-100965136	-	-	IGV_LINK	940.629	12.2965	0	25.9822	OK	480.551	5.88178	0	12.757	OK	OK	-1.06392
ENSG0000011	-	-	ENSG0000011991	'IDE	-	10:92451683-92574076	-	-	IGV_LINK	395.646	6.49086	0	13.3458	OK	355.773	5.24297	0	10.8907	OK	OK	-0.30803
ENSG0000011	-	-	ENSG0000011991	'TECTB	-	10:112283734-112305035	-	-	IGV_LINK	0.969721	0.021511	0	0.348703	OK	0	0	0	0	OK	NOTEST	'-inf
ENSG0000011	-	-	ENSG0000011991	'ELOVL3	-	10:102226327-102229589	-	-	IGV_LINK	0	0	0	0	OK	5.15612	0.242688	0	0.594911	OK	NOTEST	inf
ENSG0000011	-	-	ENSG0000011991	'IFIT3	-	10:89213568-89414557	-	-	IGV_LINK	1859.92	45.5235	0	101.846	OK	1441.65	35.2639	0	84.2794	OK	OK	-0.36842
ENSG0000011	-	-	ENSG0000011991	'NKX2-3	-	10:99532932-99536524	-	-	IGV_LINK	0	0	0	0	OK	0	0	0	0	OK	NOTEST	0
ENSG0000011	-	-	ENSG0000011992	'IFIT2	-	10:89213568-89414557	-	-	IGV_LINK	1389.94	27.0161	0	64.2008	OK	1491.58	28.9844	0	68.747	OK	OK	0.101452
ENSG0000011	-	-	ENSG0000011992	'GPAM	-	10:112149863-112215377	-	-	IGV_LINK	186.186	1.72796	0.243208	3.21271	OK	102.091	0.936652	0.031166	1.84214	OK	OK	-0.88349
ENSG0000011	-	-	ENSG0000011992	'CUTC	-	10:99659505-99756134	-	-	IGV_LINK	318.068	18.0705	0	39.498	OK	156.746	8.63553	0	22.9393	OK	OK	-1.06528
ENSG0000011	-	-	ENSG0000011993	'PPP1R3C	-	10:91628441-91633054	-	-	IGV_LINK	544.013	13.0378	5.11699	20.9586	OK	2661.59	63.8189	27.9391	99.6986	OK	OK	2.29128
ENSG0000011	-	-	ENSG0000011994	'PYROXD2	-	10:98383564-98415184	-	-	IGV_LINK	279.28	11.9866	0	26.4422	OK	180.464	6.02227	0.106744	11.9378	OK	OK	-0.99305
ENSG0000011	-	-	ENSG0000011994	'CNNM1	-	10:99329098-99394330	-	-	IGV_LINK	91.1537	0.88776	0.072842	1.70268	OK	67.0296	0.652941	0	1.35405	OK	OK	-0.44322
ENSG0000011	-	-	ENSG0000011995	'MXI1	-	10:110207604-110287365	-	-	IGV_LINK	2811.22	87.5122	28.6555	146.369	OK	1338.53	44.4761	11.3623	77.59	OK	OK	-0.97645
ENSG0000011	-	-	ENSG0000011995	'SMNDC1	-	10:110290729-110304951	-	-	IGV_LINK	343.281	15.8135	3.39232	28.2347	OK	238.213	10.2467	1.47588	19.0176	OK	OK	-0.62599
ENSG0000011	-	-	ENSG0000011996	'C10orf88	-	10:122930902-123008817	-	-	IGV_LINK	158.064	6.66658	0	16.7152	OK	97.9664	2.25185	0	5.44639	OK	OK	-1.56583
ENSG0000011	-	-	ENSG0000011996	'HELLS	-	10:94402503-94613905	-	-	IGV_LINK	201.702	3.52205	0	9.60322	OK	129.934	1.99611	0	4.76644	OK	OK	-0.81923
ENSG0000011	-	-	ENSG0000011997	'PRLHR	-	10:118589988-118595699	-	-	IGV_LINK	0	0	0	0	OK	0	0	0	0	OK	NOTEST	0
ENSG0000011	-	-	ENSG0000011997	'TCTN3	-	10:95663395-95694143	-	-	IGV_LINK	969.721	23.9166	9.82018	38.0131	OK	1040.51	25.1714	10.742	39.6007	OK	OK	0.073769
ENSG0000011	-	-	ENSG0000011997	'FAM45A	-	10:119104085-119137984	-	-	IGV_LINK	363.645	16.323	3.75429	28.8916	OK	349.585	17.6354	1.76293	33.5078	OK	OK	0.111569
ENSG0000011	-	-	ENSG0000011998	'AVPI1	-	10:97677423-97687323	-	-	IGV_LINK	60.1227	2.63784	0.032327	5.24336	OK	101.06	4.43791	0.55882	8.31701	OK	OK	0.750524
ENSG0000012	-	-	ENSG0000012000	'WDR11	-	10:120761811-120909524	-	-	IGV_LINK	1987.93	35.1891	6.19293	64.1853	OK	1243.66	21.0654	4.84268	37.2881	OK	OK	-0.74025
ENSG0000012	-	-	ENSG0000012002	'C10orf76	-	10:101845598-102056193	-	-	IGV_LINK	732.139	11.5759	3.13916	20.0127	OK	536.237	7.95034	2.48525	13.4154	OK	OK	-0.54204
ENSG0000012	-	-	ENSG0000012004	'KCNIP2	-	10:101819077-101843920	-	-	IGV_LINK	82.4262	3.14311	0	7.31513	OK	28.8743	0.944022	0	2.28286	OK	OK	-1.7353
ENSG0000012	-	-	ENSG0000012005	'CFAP58	-	10:104353763-104455090	-	-	IGV_LINK	46.5466	0.835272	0	1.72174	OK	30.9367	0.55536	0	1.25795	OK	OK	-0.58882
ENSG0000012	-	-	ENSG0000012005	'GOT1	-	10:99396869-99430624	-	-	IGV_LINK	1671.8	64.1374	22.829	105.446	OK	611.516	23.7309	7.29914	40.1627	OK	OK	-1.4344
ENSG0000012	-	-	ENSG0000012005	'CPN1	-	10:100042192-100081877	-	-	IGV_LINK	0	0	0	0	OK	0	0	0	0	OK	NOTEST	0
ENSG0000012	-	-	ENSG0000012005	'C10orf95	-	10:102449816-102461106	-	-	IGV_LINK	4.8486	0.21076	0	1.3996	OK	7.21857	0.314057	0	1.63081	OK	NOTEST	0.575426
ENSG0000012	-	-	ENSG0000012005	'SFRP5	-	10:97766750-97771952	-	-	IGV_LINK	0	0	0	0	OK	0	0	0	0	OK	NOTEST	0
ENSG0000012	-	-	ENSG0000012006	'GNA13	-	17:65010714-65056839	-	-	IGV_LINK	1091.91	14.3232	5.30692	23.3395	OK	1145.69	14.5141	5.64173	23.3864	OK	OK	0.019097
ENSG0000012	-	-	ENSG0000012006	'HOXB8	-	17:48607226-48634932	-	-	IGV_LINK	197.823	8.42325	0	18.1438	OK	43.3114	1.6466	0	4.7397	OK	OK	-2.35489
ENSG0000012	-	-	ENSG0000012007	'KANSL1	-	17:46029915-46225389	-	-	IGV_LINK	1315.91	22.7988	3.80086	41.7967	OK	1178.69	19.5975	3.73598	35.459	OK	OK	-0.21829
ENSG0000012	-	-	ENSG0000012007	'HOXB5	-	17:48540893-48606414	-	-	IGV_LINK	95.0326	2.8663	0	10.5833	OK	56.7174	1.71172	0	6.86742	OK	OK	-0.74374
ENSG0000012	-	-	ENSG0000012008	'CRHR1	-	17:45620327-46028334	-	-	IGV_LINK	0.969721	0.090408	0	1.62143	OK	0	0	0	0	OK	NOTEST	'-inf
ENSG0000012	-	-	ENSG0000012009	'HOXB3	-	17:48540893-48606414	-	-	IGV_LINK	931.024	24.9218	0	59.4204	OK	601.204	14.199	0	33.0332	OK	OK	-0.81162
ENSG0000012	-	-	ENSG0000012009	'HOXB1	-	17:48528525-48530997	-	-	IGV_LINK	0.969721	0.02958	0	0.147132	OK	0	0	0	0	OK	NOTEST	'-inf
ENSG0000012	-	-	ENSG0000012012	'DUSP1	-	5:172758225-172777774	-	-	IGV_LINK	5101.7	155.707	60.5948	250.818	OK	11152.7	340.599	111.458	569.739	OK	OK	1.12924
ENSG0000012	-	-	ENSG0000012013	'PANK3	-	5:168548494-168579600	-	-	IGV_LINK	869.275	4.74094	1.71929	7.76258	OK	609.936	4.15292	0	10.1818	OK	OK	-0.19105

ENSG0000012	-	-	ENSG0000012014	'MSX2	-	5:174724532-174730893	-	-	IGV_LINK	7.75776	0.211363	0	0.511642	OK	11.3435	0.309226	0	0.721442	OK	NOTEST	0.54894
ENSG0000012	-	-	ENSG0000012015	'TEK	-	9:27109140-27230175	-	-	IGV_LINK	213.339	2.78169	0.776532	4.78684	OK	352.679	4.67475	1.49657	7.85293	OK	OK	0.74893
ENSG0000012	-	-	ENSG0000012015	'RCL1	-	9:4792868-4885917	-	-	IGV_LINK	274.431	8.34814	2.39488	14.3014	OK	604.298	24.016	6.36068	41.6713	OK	OK	1.52447
ENSG0000012	-	-	ENSG0000012015	'CAAP1	-	9:26840684-26892804	-	-	IGV_LINK	198.793	9.80436	0.779064	18.8297	OK	366.085	17.0033	2.70944	31.2973	OK	OK	0.794323
ENSG0000012	-	-	ENSG0000012016	'EQTN	-	9:27245679-27297139	-	-	IGV_LINK	4.72741	0.377102	0	1.36909	OK	2.06245	0.135258	0	0.655668	OK	NOTEST	-1.47924
ENSG0000012	-	-	ENSG0000012016	'MOB3B	-	9:27325208-27529781	-	-	IGV_LINK	1029.84	9.23942	3.99406	14.4848	OK	1137.44	10.3002	4.26107	16.3394	OK	OK	0.156804
ENSG0000012	-	-	ENSG0000012021	'INSL6	-	9:5131978-5185668	-	-	IGV_LINK	0.969721	0.20192	0	0.809564	OK	0	0	0	0	OK	NOTEST	'-inf
ENSG0000012	-	-	ENSG0000012021	'INSL4	-	9:5231418-5235304	-	-	IGV_LINK	0	0	0	0	OK	0	0	0	0	OK	NOTEST	0
ENSG0000012	-	-	ENSG0000012021	'MLANA	-	9:5881595-6007904	-	-	IGV_LINK	3.87888	0.139153	0	1.40494	OK	3.09367	0.090975	0	0.99492	OK	NOTEST	-0.61312
ENSG0000012	-	-	ENSG0000012021	'CD274	-	9:5450502-5470566	-	-	IGV_LINK	205.581	5.19435	0	12.0766	OK	140.247	2.93575	0	6.44519	OK	OK	-0.82321
ENSG0000012	-	-	ENSG0000012023	'IFNA6	-	9:21349834-21351378	-	-	IGV_LINK	0	0	0	0	OK	0	0	0	0	OK	NOTEST	0
ENSG0000012	-	-	ENSG0000012024	'IFNA8	-	9:21409146-21410185	-	-	IGV_LINK	1.93944	0.126141	0	0.386301	OK	0	0	0	0	OK	NOTEST	'-inf
ENSG0000012	-	-	ENSG0000012025	'GRIA2	-	4:157204181-157366075	-	-	IGV_LINK	0	0	0	0	OK	0	0	0	0	OK	NOTEST	0
ENSG0000012	-	-	ENSG0000012025	'NUP43	-	6:149724314-149923121	-	-	IGV_LINK	603.166	11.7059	0	24.5948	OK	393.928	7.15278	0	15.4013	OK	OK	-0.71067
ENSG0000012	-	-	ENSG0000012025	'MTHFD1L	-	6:150865548-151228447	-	-	IGV_LINK	364.615	8.66027	2.05573	15.2648	OK	188.714	5.1907	0	10.9297	OK	OK	-0.73848
ENSG0000012	-	-	ENSG0000012025	'LRP11	-	6:149724314-149923121	-	-	IGV_LINK	149.337	2.77921	0	7.72111	OK	145.403	2.65501	0	7.90365	OK	OK	-0.06596
ENSG0000012	-	-	ENSG0000012026	'CCDC170	-	6:151494029-151621193	-	-	IGV_LINK	40.7283	1.15417	0	3.41654	OK	91.779	1.01142	0.026373	1.99646	OK	OK	-0.19048
ENSG0000012	-	-	ENSG0000012026	'PCMT1	-	6:149724314-149923121	-	-	IGV_LINK	638.076	25.9251	3.43794	48.4123	OK	421.771	17.427	1.55765	33.2964	OK	OK	-0.57302
ENSG0000012	-	-	ENSG0000012027	'PLEKHG1	-	6:150599882-150843665	-	-	IGV_LINK	410.192	4.46459	0	10.4359	OK	439.302	5.50996	0	13.9077	OK	OK	0.303514
ENSG0000012	-	-	ENSG0000012027	'MYCT1	-	6:152697894-152724567	-	-	IGV_LINK	167.762	3.70642	0.83674	6.5761	OK	185.62	4.04422	0.700537	7.3879	OK	OK	0.125835
ENSG0000012	-	-	ENSG0000012028	'CXorf21	-	X:30558823-30577844	-	-	IGV_LINK	233.703	7.828	2.28917	13.3668	OK	199.026	6.67106	1.70359	11.6385	OK	OK	-0.23073
ENSG0000012	-	-	ENSG0000012028	'MAGEB4	-	X:30242051-30252038	-	-	IGV_LINK	0	0	0	0	OK	0	0	0	0	OK	NOTEST	0
ENSG0000012	-	-	ENSG0000012030	'CYSTM1	-	5:140174641-140303121	-	-	IGV_LINK	2063.57	152.634	24.4263	280.842	OK	1335.44	96.0467	12.1894	179.904	OK	OK	-0.66827
ENSG0000012	-	-	ENSG0000012031	'WDR55	-	5:140664675-140706676	-	-	IGV_LINK	832.99	13.4875	0	33.8763	OK	1091.04	17.8372	0	44.4863	OK	OK	0.403272
ENSG0000012	-	-	ENSG0000012031	'ARAP3	-	5:141653400-141682221	-	-	IGV_LINK	586.681	8.60875	3.13329	14.0842	OK	751.763	11.0151	4.13805	17.8922	OK	OK	0.35561
ENSG0000012	-	-	ENSG0000012032	'PCDHB8	-	5:141100241-141249365	-	-	IGV_LINK	1.93944	0.04257	0	0.705438	OK	10.6737	0.234389	0	1.75452	OK	NOTEST	2.46099
ENSG0000012	-	-	ENSG0000012032	'PCDHB10	-	5:141100241-141249365	-	-	IGV_LINK	85.3354	1.54263	0	5.16025	OK	62.9047	1.13756	0	4.17609	OK	OK	-0.43944
ENSG0000012	-	-	ENSG0000012032	'PCDHB14	-	5:141100241-141249365	-	-	IGV_LINK	311.28	5.76577	0	13.3831	OK	321.555	5.9325	0	13.6503	OK	OK	0.041128
ENSG0000012	-	-	ENSG0000012032	'PCDHB12	-	5:141100241-141249365	-	-	IGV_LINK	32.9705	0.593145	0	2.95233	OK	23.7182	0.453553	0	2.55163	OK	OK	-0.38712
ENSG0000012	-	-	ENSG0000012032	'SLC25A2	-	5:141259234-141320821	-	-	IGV_LINK	1.93944	0.087827	0	1.72058	OK	8.2498	0.373934	0	3.97845	OK	NOTEST	2.09005
ENSG0000012	-	-	ENSG0000012033	'TNN	-	1:175067857-175148066	-	-	IGV_LINK	3.87888	0.053822	0	0.145061	OK	10.3122	0.128873	0	0.29352	OK	NOTEST	1.25969
ENSG0000012	-	-	ENSG0000012033	'MRPS14	-	1:174999162-175023425	-	-	IGV_LINK	435.405	17.2348	0	35.2565	OK	389.803	17.4887	0	37.5412	OK	OK	0.021096
ENSG0000012	-	-	ENSG0000012033	'CENPL	-	1:173799549-173858546	-	-	IGV_LINK	67.8804	2.0982	0	5.5649	OK	80.4355	2.85517	0	7.69287	OK	OK	0.444424
ENSG0000012	-	-	ENSG0000012033	'TNFSF18	-	1:172775904-173064015	-	-	IGV_LINK	8.72748	0.267964	0	0.621366	OK	16.4996	0.506912	0	1.13319	OK	NOTEST	0.919698
ENSG0000012	-	-	ENSG0000012034	'SEC16B	-	1:177923955-178038007	-	-	IGV_LINK	556.083	11.6672	0	30.105	OK	548.745	8.51697	0	17.5469	OK	OK	-0.45404
ENSG0000012	-	-	ENSG0000012037	'GORAB	-	1:170460452-170553446	-	-	IGV_LINK	211.399	9.29897	0	19.2391	OK	221.713	7.99264	0	16.65	OK	OK	-0.2184
ENSG0000012	-	-	ENSG0000012043	'GPR31	-	6:167146413-167196913	-	-	IGV_LINK	10.6669	0.318657	0	0.737863	OK	4.1249	0.1233	0	0.337645	OK	NOTEST	-1.36983
ENSG0000012	-	-	ENSG0000012043	'ACAT2	-	6:159669056-159789749	-	-	IGV_LINK	350.069	11.0478	0	124.222	OK	325.867	10.9203	0	214.964	OK	OK	-0.01675
ENSG0000012	-	-	ENSG0000012043	'TCP1	-	6:159669056-159789749	-	-	IGV_LINK	2426.77	91.5349	0	434.004	OK	2114.21	75.3508	0	600.52	OK	OK	-0.2807
ENSG0000012	-	-	ENSG0000012044	'TTLL2	-	6:167325085-167384510	-	-	IGV_LINK	0.969721	0.028725	0	0.259681	OK	27.8431	0.794644	0	1.68527	OK	OK	4.78995
ENSG0000012	-	-	ENSG0000012045	'SNX19	-	11:130875435-130916509	-	-	IGV_LINK	1086.09	14.2403	0.755245	27.7254	OK	1146.72	16.7767	2.05225	31.5011	OK	OK	0.236474
ENSG0000012	-	-	ENSG0000012045	'KCNJ5	-	11:128891355-128921035	-	-	IGV_LINK	278.31	3.08799	0.172398	6.00358	OK	388.772	5.01168	0	10.0633	OK	OK	0.698626
ENSG0000012	-	-	ENSG0000012045	'MSANTD2	-	11:124766497-124834487	-	-	IGV_LINK	466.436	10.3765	1.99658	18.7563	OK	246.463	6.14996	0.183729	12.1162	OK	OK	-0.75467
ENSG0000012	-	-	ENSG0000012047	'TP53AIP1	-	11:128934730-128943399	-	-	IGV_LINK	1.93944	0.080044	0	0.489523	OK	14.4371	0.405096	0	1.11498	OK	NOTEST	2.3394
ENSG0000012	-	-	ENSG0000012049	'TEX11	-	X:70528939-70908731	-	-	IGV_LINK	75.6382	2.07783	0.144265	4.0114	OK	446.52	12.2731	4.31616	20.23	OK	OK	2.56235
ENSG0000012	-	-	ENSG0000012050	'ARR3	-	X:70268304-70281840	-	-	IGV_LINK	0.969721	0.216235	0	0.896647	OK	0	0	0	0	OK	NOTEST	'-inf
ENSG0000012	-	-	ENSG0000012050	'PDZD11	-	X:70286594-70420832	-	-	IGV_LINK	505.224	34.7734	11.3092	58.2376	OK	562.018	40.1125	12.5092	67.7157	OK	OK	0.206066
ENSG0000012	-	-	ENSG0000012051	'SLC10A7	-	4:146253974-146521964	-	-	IGV_LINK	148.367	3.52761	0.228629	6.82659	OK	173.246	4.4989	0.335322	8.66248	OK	OK	0.350881
ENSG0000012	-	-	ENSG0000012052	'NUDCD1	-	8:109240918-109345953	-	-	IGV_LINK	298.674	6.70031	0	14.9727	OK	241.307	6.23601	0	14.8453	OK	OK	-0.1036
ENSG0000012	-	-	ENSG0000012053	'ENY2	-	8:109240918-109345953	-	-	IGV_LINK	1012.39	77.958	10.7192	145.197	OK	1183.85	118.73	30.5605	206.899	OK	OK	0.606914
ENSG0000012	-	-	ENSG0000012053	'MASTL	-	10:27110111-27186924	-	-	IGV_LINK	258.915	4.73123	0	12.4623	OK	159.84	2.88654	0	8.06866	OK	OK	-0.71287
ENSG0000012	-	-	ENSG0000012054	'KIAA1217	-	10:23694745-24547848	-	-	IGV_LINK	1197.6	13.642	2.4593	24.8248	OK	879.635	10.1606	1.6726	18.6486	OK	OK	-0.42508
ENSG0000012	-	-	ENSG0000012055	'SEPT7P9	-	10:38383068-38402916	-	-	IGV_LINK	3.87888	0.383184	0	1.10028	OK	2.06245	0.204155	0	0.778241	OK	NOTEST	-0.90837
ENSG0000012	-	-	ENSG0000012056	'LYZL1	-	10:29289060-29318328	-	-	IGV_LINK	0	0	0	0	OK	0	0	0	0	OK	NOTEST	0
ENSG0000012	-	-	ENSG0000012059	'PLXDC2	-	10:19816238-20289856	-	-	IGV_LINK	2978.01	27.6172	0	56.555	OK	1623.15	15.1434	1.12135	29.1654	OK	OK	-0.86689
ENSG0000012	-	-	ENSG0000012061	'EPC1	-	10:32266288-32378798	-	-	IGV_LINK	771.898	20.0908	0.190512	39.9912	OK	502.207	12.3196	0	24.8817	OK	OK	-0.70558
ENSG0000012	-	-	ENSG0000012064	'IQSEC3	-	12:66764-182399	-	-	IGV_LINK	40.7283	0.940398	0	4.48726	OK	20.6245	0.168029	0	0.734703	OK	OK	-2.48456
ENSG0000012	-	-	ENSG0000012064	'CCDC77	-	12:280128-442645	-	-	IGV_LINK	151.276	6.96679	0	23.9018	OK	175.308	6.74506	0	22.3304	OK	OK	-0.04666
ENSG0000012	-	-	ENSG0000012065	'TAF12	-	1:28589322-28643085	-	-	IGV_LINK	281.219	13.7534	2.74105	24.7657	OK	317.617	15.4682	0	35.9677	OK	OK	0.169515
ENSG0000012	-	-	ENSG0000012065	'ENOX1	-	13:43213517-43786908	-	-	IGV_LINK	9.69721	0.194517	0	0.568447	OK	14.4371	0.289728	0	0.890133	OK	NOTEST	0.574804
ENSG0000012	-	-	ENSG0000012065	'TNFSF11	-	13:42562735-42608013	-	-	IGV_LINK	3.87888	0.107871	0	0.420335	OK	3.09367	0.089244	0	0.376635	OK	NOTEST	-0.27347
ENSG0000012	-	-	ENSG0000012066	'MTRF1	-	13:41132938-41263577	-	-	IGV_LINK	233.703	10.5219	0	21.9565	OK	265.025	9.51359	0.885668	18.1415	OK	OK	-0.14533
ENSG0000012	-	-	ENSG0000012066	'SPG20-AS1	-	13:36301637-36370180	-	-	IGV_LINK	26.1825	3.81695	0	21.4258	OK	8.2498	1.33976	0	11.5712	OK	OK	-1.51045
ENSG0000012	-	-	ENSG0000012066	'SOHLH2	-	13:36168207-36297842	-	-	IGV_LINK	16.4852	0.459808	0	1.14042	OK	14.8609	0.414739	0	1.17765	OK	NOTEST	-0.14883
ENSG0000012	-	-	ENSG0000012067	'DNAJC15	-	13:43023202-43114224	-	-	IGV_LINK	687.532	13.7797	0.294363	27.2651	OK	1043.6	23.9471	2.30119	45.5931	OK	OK	0.797303
ENSG0000012	-	-	ENSG0000012068	'PROSER1	-	13:39009865-39038076	-	-	IGV_LINK	649.713	10.4041	0	22.6115	OK	534.175	8.03969	0	17.6615	OK	OK	-0.37193
ENSG0000012	-	-	ENSG0000012068	'UFM1	-	13:38349848-38363619	-	-	IGV_LINK	1270.33	30.4592	8.29234	52.626	OK	1415.87	31.84	9.90164	53.7783	OK	OK	0.063961
ENSG0000012	-	-	ENSG0000012068	'WBP4	-	13:40932027-41084006	-	-	IGV_LINK	344.251	7.91699	0	19.2468	OK	378.46	8.7078	0	21.2439	OK	OK	0.137357
ENSG0000012	-	-	ENSG0000012069	'ELF1	-	13:40932027-41084006	-	-	IGV_LINK	1202.45	27.9626	8.24586	47.6794	OK	1369.47	32.5636	10.2178	54.9095	OK	OK	0.219761
ENSG0000012	-	-	ENSG0000012069	'SMAD9	-	13:36844830-36920765	-	-	IGV_LINK	237.582	2.54753	0.613141	4.48191	OK	489.832	5.9406	0.198836	11.6824	OK	OK	1.22151
ENSG0000012	-	-	ENSG0000012069	'HSPH1	-	13:31134973-31162388	-	-	IGV_LINK	512.012	9.86213	0.198338	19.5259	OK	684.733	11.5106	4.29308	18.7282	OK	OK	0.222994
ENSG0000012	-	-	ENSG0000012069	'KBTBD7	-	13:41132938-41263577	-	-	IGV_LINK	175.519	2.16848	0.021614	4.31534	OK	86.6229	1.07046	0	2.49815	OK	OK	-1.01845
ENSG0000012	-	-	ENSG0000012069	'ALG5	-	13:36949774-37059713	-	-	IGV_LINK	550.801	32.0123	0	66.7733	OK	611.516	35.4084	0.333379	70.4833	OK	OK	0.145463
ENSG0000012	-	-	ENSG0000012069	'EXOSC8	-	13:36949774-37059713	-	-	IGV_LINK	547.892	26.9183	0	61.0604	OK	402.178	18.8921	0	44.8727	OK	OK	-0.5108
ENSG0000012	-	-	ENSG0000012070	'ETF1	-	5:138506094-138543300	-	-	IGV_LINK	1457.49	26.7099	9.39341	44.0264	OK	1992.33	38.9566	11.4639	66.4494	OK	OK	0.544495
ENSG0000012	-	-	ENSG0000012070	'TGFBI	-	5:136028894-136063818	-	-	IGV_LINK	10117.1	259.396	63.6806	455.111	OK	133129	3091.52	0	8527.88	OK	OK	3.57509
ENSG0000012	-	-	ENSG0000012070	'FAM53C	-	5:138285264-138349729	-	-	IGV_LINK	893.113	13.4883	4.01172	22.9649	OK	1134.35	16.3593	5.88216	26.8365	OK	OK	0.278405
ENSG0000012	-	-	ENSG0000012072	'SIL1	-	5:138946719-139331677	-	-	IGV_LINK	929.962	32.3955	0	85.8741	OK	1266.34	53.8912	0	151.145	OK	OK	0.734256
ENSG0000012	-	-	ENSG0000012072	'PAIP2	-	5:139341586-139384553	-	-	IGV_LINK	1393.49	94.6322	32.0334	157.231	OK	1538.59	106.09	34.9068	177.274	OK	OK	0.164888
ENSG0000012	-	-	ENSG0000012072	'MYOT	-	5:137814332-138051961	-	-	IGV_LINK	97.9418	3.86792	0	12.7014	OK	51.5612	1.506	0	5.04956	OK	OK	-1.36084
ENSG0000012	-	-	ENSG0000012073	'KDM3B	-	5:138352595-138437028	-	-	IGV_LINK	2700.67	32.3405	7.23078	57.4503	OK	2592.5	31.0213	8.1459	53.8967	OK	OK	-0.06008
ENSG0000012	-	-	ENSG0000012073	'EGR1	-	5:138465489-138469315	-	-	IGV_LINK	608.015	11.5546	4.64254	18.4667	OK	1022.98	19.448	8.43618	30.4598	OK	OK	0.751153
ENSG0000012	-	-	ENSG0000012074	'SERP1	-	3:150541993-150630445	-	-	IGV_LINK	1422.58	42.4095	0	99.4821	OK	1246.75	36.1479	0	87.3992	OK	OK	-0.23048
ENSG0000012	-	-	ENSG0000012075	'PLS1	-	3:142596386-142713664	-	-	IGV_LINK	365.585	9.82382	1.59678	18.0509	OK	371.241	9.27635	2.34607	16.2066	OK	OK	-0.08273
ENSG0000012	-	-	ENSG0000012078	'ZFP30	-	19:37497158-37719790	-	-	IGV_LINK	182.699	4.60056	0	15.237	OK	136.122	2.3783	0	7.99053	OK	OK	-0.95188
ENSG0000012	-	-	ENSG0000012079	'NR2C1	-	12:95020228-95073703	-	-	IGV_LINK	364.615	15.8277	1.74586	29.9094	OK	397.022	16.765	2.94094	30.5891	OK	OK	0.083007
ENSG0000012	-	-	ENSG0000012080	'UTP20	-	12:101280108-101386616	-	-	IGV_LINK	551.771	3.55212	1.30237	5.80188	OK	430.021	2.73789	0.939671	4.53611	OK	OK	-0.37562
ENSG0000012	-	-	ENSG0000012080	'TMPO	-	12:98512972-98550379	-	-	IGV_LINK	1068.63	24.9298	3.90747	45.9521	OK	737.326	15.4829	2.0133	28.9524	OK	OK	-0.6872
ENSG0000012	-	-	ENSG0000012080	'ARL1	-	12:101393119-101407820	-	-	IGV_LINK	1580.64	64.3018	14.911	113.693	OK	1479.81	72.5408	17.9935	127.088	OK	OK	0.173935
ENSG0000012	-	-	ENSG0000012082	'GLT8D2	-	12:103988983-104064183	-	-	IGV_LINK	8.72748	0.759851	0	1.98911	OK	23.7182	0.789184	0	1.81368	OK	OK	0.054647
ENSG0000012	-	-	ENSG0000012083	'MTERF2	-	12:106955718-106987166	-	-	IGV_LINK	441.223	14.0462	0	29.6699	OK	251.619	7.1746	0	17.4378	OK	OK	-0.96921
ENSG0000012	-	-	ENSG0000012083	'SOCS2	-	12:93542462-93583487	-	-	IGV_LINK	125.094	3.665	0.003211	7.32679	OK	160.871	5.52363	0	11.1285	OK	OK	0.591802
ENSG0000012	-	-	ENSG0000012083	'NFYB	-	12:104117076-104138289	-	-	IGV_LINK	380.13	14.6694	2.45152	26.8872	OK	412.49	15.8462	2.76724	28.9252	OK	OK	0.111333
ENSG0000012	-	-	ENSG0000012086	'WASHC3	-	12:102012926-102062149	-	-	IGV_LINK	515.891	45.5691	12.1425	78.9956	OK	545.518	45.5587	17.0194	74.0981	OK	OK	-0.00033
ENSG0000012	-	-	ENSG0000012086	'APAF1	-	12:98645140-99984654	-	-	IGV_LINK	597.348	5.83358	0.576261	11.0909	OK	611.516	5.5745	1.37554	9.77346	OK	OK	-0.06554
ENSG0000012	-	-	ENSG0000012087	'DUSP4	-	8:29333063-29350668	-	-	IGV_LINK	437.344	4.82294	1.73406	7.91183	OK	149.528	1.67906	0.321178	3.03695	OK	OK	-1.52226
ENSG0000012	-	-	ENSG0000012088	'CLU	-	8:27596916-27615031	-	-	IGV_LINK	42125.6	3035.67	0	6995.37	OK	119175	9095.22	0	26969.1	OK	OK	1.58309
ENSG0000012	-	-	ENSG0000012088	'TNFRSF10B	-	8:22687658-23083619	-	-	IGV_LINK	2762.73	55.016	11.8546	98.1774	OK	4273.4	84.1824	15.026	153.339	OK	OK	0.613667
ENSG0000012	-	-	ENSG0000012089	'SORBS3	-	8:22440818-22575788	-	-	IGV_LINK	1263.55	47.2275	9.26256	85.1925	OK	1739.68	64.5175	16.1678	112.867	OK	OK	0.450062
ENSG0000012	-	-	ENSG0000012089	'PTK2B	-	8:27311481-27459391	-	-	IGV_LINK	1336.27	28.9498	2.08959	55.8099	OK	2159.39	42.7231	3.81519	81.631	OK	OK	0.561465
ENSG0000012	-	-	ENSG0000012090	'CHRNA2	-	8:27459760-27479883	-	-	IGV_LINK	0	0	0	0	OK	1.03122	0.037221	0	1.29242	OK	NOTEST	inf
ENSG0000012	-	-	ENSG0000012090	'ADRA1A	-	8:26748149-26867273	-	-	IGV_LINK	2.90916	0.094094	0	0.438015	OK	5.15612	0.156766	0	0.559242	OK	NOTEST	0.736429
ENSG0000012	-	-	ENSG0000012091	'PPP3CC	-	8:22440818-22575788	-	-	IGV_LINK	392.737	13.0529	0	33.2531	OK	338.242	11.7863	0	32.6853	OK	OK	-0.14726
ENSG0000012	-	-	ENSG0000012091	'PDLIM2	-	8:22578278-22604150	-	-	IGV_LINK	450.92	26.2661	1.64259	50.8897	OK	683.702	41.19	4.68492	77.6951	OK	OK	0.64909
ENSG0000012	-	-	ENSG0000012091	'EPHX2	-	8:27490778-27545564	-	-	IGV_LINK	2172.17	85.611	23.7326	147.489	OK	2770.9	108.235	30.83	185.641	OK	OK	0.338305
ENSG0000012	-	-	ENSG0000012092	'RNF170	-	8:42849636-43085788	-	-	IGV_LINK	466.436	9.25081	0	26.8742	OK	479.52	9.72729	0	24.3261	OK	OK	0.072459
ENSG0000012	-	-	ENSG0000012093	'NPPB	-	1:11857463-11858931	-	-	IGV_LINK	0.969721	0.104797	0	0.410465	OK	0	0	0	0	OK	NOTEST	'-inf
ENSG0000012	-	-	ENSG0000012094	'UBIAD1	-	1:11273205-11296049	-	-	IGV_LINK	298.674	5.14645	1.50124	8.79166	OK	350.616	6.11059	1.77027	10.4509	OK	OK	0.247735
ENSG0000012	-	-	ENSG0000012094	'TARDBP	-	1:11012343-11047233	-	-	IGV_LINK	2820.84	92.5953	22.1949	162.996	OK	2618.08	94.3241	33.4449	155.203	OK	OK	0.026687
ENSG0000012	-	-	ENSG0000012094	'TNFRSF8	-	1:12063376-12144207	-	-	IGV_LINK	55.2741	1.21324	0	2.42882	OK	49.4988	1.13511	0	2.36649	OK	OK	-0.09604
ENSG0000012	-	-	ENSG0000012095	'PRAMEF2	-	1:12857085-12861909	-	-	IGV_LINK	0	0	0	0	OK	0	0	0	0	OK	NOTEST	0
ENSG0000012	-	-	ENSG0000012096	'ZNF706	-	8:101177877-101206193	-	-	IGV_LINK	1044.39	75.2658	22.2462	128.285	OK	1180.75	82.6764	19.672	145.681	OK	OK	0.135481
ENSG0000012	-	-	ENSG0000012099	'LYPLA1	-	8:54046366-54102017	-	-	IGV_LINK	519.77	33.8561	3.89476	63.8174	OK	620.797	37.4461	2.71944	72.1727	OK	OK	0.1454
ENSG0000012	-	-	ENSG0000012100	'CRISPLD1	-	8:74984514-75034558	-	-	IGV_LINK	9.69721	0.274733	0	0.860725	OK	55.6861	2.15718	0	5.38481	OK	OK	2.97304
ENSG0000012	-	-	ENSG0000012102	'COPS5	-	8:66964098-67343677	-	-	IGV_LINK	1056.03	38.2444	0	77.2571	OK	1105.47	41.9152	1.89622	81.9341	OK	OK	0.132223
ENSG0000012	-	-	ENSG0000012103	'RDH10	-	8:73290266-73356461	-	-	IGV_LINK	366.554	14.1169	0	153.423	OK	617.704	23.6252	0	188.605	OK	OK	0.742906
ENSG0000012	-	-	ENSG0000012105	'EPX	-	17:58192736-58205174	-	-	IGV_LINK	2.90916	0.064661	0	0.201873	OK	2.06245	0.045862	0	0.152385	OK	NOTEST	-0.49559
ENSG0000012	-	-	ENSG0000012105	'AKAP1	-	17:57085091-57121349	-	-	IGV_LINK	1035.66	20.6197	1.77302	39.4663	OK	973.476	16.5017	4.67884	28.3246	OK	OK	-0.3214
ENSG0000012	-	-	ENSG0000012105	'COIL	-	17:56938186-56961054	-	-	IGV_LINK	271.522	6.17633	1.94439	10.4083	OK	314.524	7.15783	2.31884	11.9968	OK	OK	0.212771
ENSG0000012	-	-	ENSG0000012106	'TRIM25	-	17:56887908-56914038	-	-	IGV_LINK	1306.21	23.9906	0	52.9725	OK	1356.06	24.3038	0	52.769	OK	OK	0.018714
ENSG0000012	-	-	ENSG0000012106	'SCPEP1	-	17:56978104-57006768	-	-	IGV_LINK	1292.64	56.1752	18.9219	93.4285	OK	1291.09	54.2174	18.3552	90.0797	OK	OK	-0.05118
ENSG0000012	-	-	ENSG0000012106	'SPOP	-	17:49598883-49678234	-	-	IGV_LINK	840.748	28.8527	6.35801	51.3473	OK	1141.57	37.3536	10.0817	64.6255	OK	OK	0.372544
ENSG0000012	-	-	ENSG0000012106	'TBX2	-	17:60677452-61411555	-	-	IGV_LINK	2205.14	30.6573	9.5981	51.7166	OK	1109.6	15.5863	4.62524	26.5473	OK	OK	-0.97596
ENSG0000012	-	-	ENSG0000012107	'SLC35B1	-	17:49700942-49836454	-	-	IGV_LINK	663.289	41.1314	0	92.8669	OK	691.952	38.8342	0	82.1631	OK	OK	-0.08292
ENSG0000012	-	-	ENSG0000012107	'TBX4	-	17:61452403-61485110	-	-	IGV_LINK	2.90916	0.198859	0	0.786084	OK	1.03122	0.025307	0	0.417152	OK	NOTEST	-2.97412
ENSG0000012	-	-	ENSG0000012108	'NACA3P	-	4:164943289-164943937	-	-	IGV_LINK	0	0	0	0	OK	0	0	0	0	OK	NOTEST	0
ENSG0000012	-	-	ENSG0000012110	'TEX14	-	17:58519836-58692055	-	-	IGV_LINK	6.78804	0.099423	0	0.923031	OK	3.09367	0.113655	0	1.46274	OK	NOTEST	0.193012
ENSG0000012	-	-	ENSG0000012110	'FAM117A	-	17:49700942-49836454	-	-	IGV_LINK	326.796	9.81178	0	25.4685	OK	312.461	8.36061	0	20.3897	OK	OK	-0.23091
ENSG0000012	-	-	ENSG0000012115	'NCAPH	-	2:96335786-96373845	-	-	IGV_LINK	96.9721	3.35544	0	6.90432	OK	75.2794	2.2688	0	4.88544	OK	OK	-0.56457
ENSG0000012	-	-	ENSG0000012120	'LRAT	-	4:154626944-154753118	-	-	IGV_LINK	90.184	4.29166	0	10.1327	OK	135.09	4.82287	0	11.1554	OK	OK	0.168354
ENSG0000012	-	-	ENSG0000012121	'TMEM131L	-	4:153466345-153636711	-	-	IGV_LINK	391.767	5.60007	0.406157	10.794	OK	326.898	4.28284	0.52913	8.03655	OK	OK	-0.38688
ENSG0000012	-	-	ENSG0000012121	'MND1	-	4:153344648-153415118	-	-	IGV_LINK	11.6366	0.990804	0	2.40679	OK	19.5933	1.87294	0	4.09481	OK	OK	0.918631

ENSG0000012	-	-	ENSG0000012123	'TRIM6	-	11:5253189-5645789	-	-	IGV_LINK	484.2	11.7828	1.14936	22.4163	OK	676.326	15.911	1.85999	29.9619	OK	OK	0.433337
ENSG0000012	-	-	ENSG0000012127	'ABCC11	-	16:48164951-48448402	-	-	IGV_LINK	1.93944	0.028673	0	0.667842	OK	3.09367	0.052245	0	0.950396	OK	NOTEST	0.865621
ENSG0000012	-	-	ENSG0000012127	'PAPD5	-	16:50152917-50235310	-	-	IGV_LINK	240.491	3.97447	0.827829	7.1211	OK	158.809	2.74397	0.395473	5.09247	OK	OK	-0.5345
ENSG0000012	-	-	ENSG0000012128	'ADCY7	-	16:50246136-50371480	-	-	IGV_LINK	848.505	20.1935	0	59.0784	OK	736.295	16.8204	0	48.0058	OK	OK	-0.26368
ENSG0000012	-	-	ENSG0000012128	'CEP89	-	19:32875924-32971991	-	-	IGV_LINK	615.773	12.0525	3.87438	20.2305	OK	517.675	11.3511	2.9737	19.7284	OK	OK	-0.0865
ENSG0000012	-	-	ENSG0000012129	'TSHZ3	-	19:31274944-31417794	-	-	IGV_LINK	58.1832	0.655353	0	1.3882	OK	128.903	1.45253	0.220268	2.6848	OK	OK	1.14823
ENSG0000012	-	-	ENSG0000012131	'ECHDC2	-	1:52895909-52927212	-	-	IGV_LINK	4091.25	221.109	53.2843	388.933	OK	3073.05	167.354	47.2501	287.458	OK	OK	-0.40185
ENSG0000012	-	-	ENSG0000012131	'TAS2R8	-	12:10806050-10807293	-	-	IGV_LINK	0	0	0	0	OK	0	0	0	0	OK	NOTEST	0
ENSG0000012	-	-	ENSG0000012131	'PLBD1	-	12:14503660-14757963	-	-	IGV_LINK	1023.06	29.662	10.4094	48.9146	OK	1057.01	31.2161	10.1664	52.2658	OK	OK	0.073674
ENSG0000012	-	-	ENSG0000012131	'TAS2R10	-	12:10824959-11176016	-	-	IGV_LINK	1.93944	0.125706	0	1.41552	OK	3.09367	0.200784	0	1.80636	OK	NOTEST	0.675591
ENSG0000012	-	-	ENSG0000012133	'PRB2	-	12:11351822-11501041	-	-	IGV_LINK	0	0	0	0	OK	0	0	0	0	OK	NOTEST	0
ENSG0000012	-	-	ENSG0000012135	'PYROXD1	-	12:21437614-21501669	-	-	IGV_LINK	286.068	8.30986	0	20.8122	OK	354.741	10.508	0	27.036	OK	OK	0.338598
ENSG0000012	-	-	ENSG0000012135	'IAPP	-	12:21264599-21419594	-	-	IGV_LINK	0	0	0	0	OK	0	0	0	0	OK	NOTEST	0
ENSG0000012	-	-	ENSG0000012136	'KCNJ8	-	12:21635341-21775581	-	-	IGV_LINK	505.224	12.8457	0	41.1798	OK	186.652	4.8081	0	22.0928	OK	OK	-1.41774
ENSG0000012	-	-	ENSG0000012137	'TAS2R7	-	12:10801531-10802627	-	-	IGV_LINK	0	0	0	0	OK	0	0	0	0	OK	NOTEST	0
ENSG0000012	-	-	ENSG0000012138	'BCL2L14	-	12:12049843-12267012	-	-	IGV_LINK	28.1219	1.19034	0	5.62938	OK	9.28102	0.425898	0	3.08762	OK	OK	-1.4828
ENSG0000012	-	-	ENSG0000012138	'TAS2R9	-	12:10809093-10810168	-	-	IGV_LINK	0	0	0	0	OK	0	0	0	0	OK	NOTEST	0
ENSG0000012	-	-	ENSG0000012138	'AL139327.1	-	13:19185224-19187349	-	-	IGV_LINK	0	0	0	0	OK	0	0	0	0	OK	NOTEST	0
ENSG0000012	-	-	ENSG0000012139	'PSPC1	-	13:19674623-19783019	-	-	IGV_LINK	552.741	18.9001	5.31196	32.4882	OK	531.081	17.4108	6.10003	28.7216	OK	OK	-0.11841
ENSG0000012	-	-	ENSG0000012140	'ZNF549	-	19:57527324-57559863	-	-	IGV_LINK	112.488	1.6079	0	3.75737	OK	79.4043	1.14756	0	2.72068	OK	OK	-0.48661
ENSG0000012	-	-	ENSG0000012141	'A1BG	-	19:58345177-58366591	-	-	IGV_LINK	29.4295	4.50683	0	13.9876	OK	14.4371	2.12151	0	7.95949	OK	OK	-1.08702
ENSG0000012	-	-	ENSG0000012141	'ZSCAN18	-	19:58059238-58118427	-	-	IGV_LINK	1020.15	27.1727	6.03998	48.3055	OK	445.489	12.2592	1.8514	22.6671	OK	OK	-1.14829
ENSG0000012	-	-	ENSG0000012141	'ZNF211	-	19:57630394-57642779	-	-	IGV_LINK	353.948	11.728	1.78239	21.6737	OK	299.055	9.97853	1.25596	18.7011	OK	OK	-0.23306
ENSG0000012	-	-	ENSG0000012144	'PDZRN3	-	3:73382432-73627626	-	-	IGV_LINK	25.2127	0.487341	0	1.34091	OK	13.4059	0.213764	0	0.779982	OK	NOTEST	-1.18892
ENSG0000012	-	-	ENSG0000012144	'RGSL1	-	1:182407620-182560599	-	-	IGV_LINK	0.969721	0.029201	0	0.84147	OK	2.06245	0.033151	0	0.851691	OK	NOTEST	0.183036
ENSG0000012	-	-	ENSG0000012145	'LHX4	-	1:180230285-180502954	-	-	IGV_LINK	91.1537	0.90961	0	2.55033	OK	75.2794	0.86021	0	3.12753	OK	OK	-0.08056
ENSG0000012	-	-	ENSG0000012148	'RNF2	-	1:185045363-185102608	-	-	IGV_LINK	207.52	9.05702	0.324268	17.7898	OK	204.183	8.10336	1.09487	15.1118	OK	OK	-0.16052
ENSG0000012	-	-	ENSG0000012148	'TRMT1L	-	1:185118097-185157072	-	-	IGV_LINK	370.433	9.53476	0	19.4065	OK	382.584	10.2162	0	20.9289	OK	OK	0.099585
ENSG0000012	-	-	ENSG0000012154	'SEC22A	-	3:123067061-123274130	-	-	IGV_LINK	283.448	12.0217	0	30.5787	OK	346.492	17.3495	0	44.9059	OK	OK	0.529257
ENSG0000012	-	-	ENSG0000012155	'CSTA	-	3:122325243-122341972	-	-	IGV_LINK	188.126	18.3149	4.40295	32.2268	OK	116.528	11.3749	1.79273	20.9571	OK	OK	-0.68716
ENSG0000012	-	-	ENSG0000012157	'DPPA4	-	3:109326140-109337572	-	-	IGV_LINK	2.90916	0.120854	0	0.649027	OK	2.06245	0.124526	0	0.683455	OK	NOTEST	0.043177
ENSG0000012	-	-	ENSG0000012157	'POPDC2	-	3:119636456-119677454	-	-	IGV_LINK	115.397	5.48818	0	14.6057	OK	87.4793	4.68767	0	13.3775	OK	OK	-0.22746
ENSG0000012	-	-	ENSG0000012157	'B4GALT4	-	3:119211731-119290666	-	-	IGV_LINK	473.224	10.9641	1.68917	20.239	OK	822.918	22.8691	5.48335	40.2549	OK	OK	1.06062
ENSG0000012	-	-	ENSG0000012157	'NAA50	-	3:113716459-113746300	-	-	IGV_LINK	881.476	24.0617	2.52194	45.6015	OK	1101.35	32.1005	7.83108	56.37	OK	OK	0.415858
ENSG0000012	-	-	ENSG0000012159	'CD80	-	3:119498531-119559602	-	-	IGV_LINK	99.8812	2.82268	0	8.0158	OK	64.9672	1.90195	0	7.26774	OK	OK	-0.56959
ENSG0000012	-	-	ENSG0000012162	'KIF18A	-	11:28020619-28527041	-	-	IGV_LINK	53.3346	0.885205	0	2.61544	OK	70.1233	1.16424	0	3.59308	OK	OK	0.39531
ENSG0000012	-	-	ENSG0000012163	'GJA8	-	1:147907955-147909257	-	-	IGV_LINK	0	0	0	0	OK	0	0	0	0	OK	NOTEST	0
ENSG0000012	-	-	ENSG0000012164	'DESI2	-	1:244652934-244709033	-	-	IGV_LINK	401.464	5.83191	1.89314	9.77067	OK	502.207	7.26031	2.55267	11.968	OK	OK	0.316063
ENSG0000012	-	-	ENSG0000012165	'MAPK8IP1	-	11:45885650-45906465	-	-	IGV_LINK	232.733	5.25964	0.024585	10.4947	OK	332.054	6.50589	2.09281	10.919	OK	OK	0.306783
ENSG0000012	-	-	ENSG0000012167	'CRY2	-	11:45847117-45883248	-	-	IGV_LINK	1732.89	28.3946	8.16809	48.6211	OK	1393.18	22.5807	5.60992	39.5516	OK	OK	-0.33053
ENSG0000012	-	-	ENSG0000012168	'PEX16	-	11:45909668-45918812	-	-	IGV_LINK	320.977	16.5328	3.50155	29.564	OK	440.333	22.5474	6.15372	38.9411	OK	OK	0.447631
ENSG0000012	-	-	ENSG0000012169	'DEPDC7	-	11:33015863-33033582	-	-	IGV_LINK	159.034	6.46029	1.28122	11.6394	OK	316.586	12.6009	3.95772	21.2441	OK	OK	0.96386
ENSG0000012	-	-	ENSG0000012169	'CAT	-	11:34438924-34472062	-	-	IGV_LINK	2363.21	69.7802	21.1965	118.364	OK	3690.75	105.646	35.9697	175.322	OK	OK	0.598348
ENSG0000012	-	-	ENSG0000012171	'PILRB	-	7:100307701-100400099	-	-	IGV_LINK	1378.71	49.3501	0	109.518	OK	1310.23	48.4967	0	110.303	OK	OK	-0.02517
ENSG0000012	-	-	ENSG0000012174	'ZMYM2	-	13:19958669-20091829	-	-	IGV_LINK	1507.92	21.7429	7.63448	35.8512	OK	1527.24	29.1256	0	65.0172	OK	OK	0.421744
ENSG0000012	-	-	ENSG0000012174	'GJB6	-	13:20221970-20232395	-	-	IGV_LINK	0	0	0	0	OK	0	0	0	0	OK	NOTEST	0
ENSG0000012	-	-	ENSG0000012174	'GJA3	-	13:20138254-20161049	-	-	IGV_LINK	0.969721	0.010846	0	0.042482	OK	0	0	0	0	OK	NOTEST	'-inf
ENSG0000012	-	-	ENSG0000012174	'TBC1D15	-	12:71839706-71927248	-	-	IGV_LINK	748.624	20.8343	6.85942	34.8092	OK	871.385	26.6503	8.01943	45.2811	OK	OK	0.355189
ENSG0000012	-	-	ENSG0000012175	'ADGRB2	-	1:31727116-31764893	-	-	IGV_LINK	32.0008	0.918785	0	2.44824	OK	306.274	4.81498	0.457104	9.17285	OK	OK	2.38973
ENSG0000012	-	-	ENSG0000012176	'HCRTR1	-	1:31617685-31704319	-	-	IGV_LINK	0	0	0	0	OK	0	0	0	0	OK	NOTEST	0
ENSG0000012	-	-	ENSG0000012176	'ZCCHC17	-	1:31296981-31364953	-	-	IGV_LINK	441.223	17.9143	6.38326	29.4453	OK	491.894	20.0793	7.41567	32.7428	OK	OK	0.164594
ENSG0000012	-	-	ENSG0000012176	'FABP3	-	1:31365624-31376850	-	-	IGV_LINK	835.899	85.9409	36.4445	135.437	OK	2881.24	298.497	129.289	467.705	OK	OK	1.7963
ENSG0000012	-	-	ENSG0000012177	'KHDRBS1	-	1:32013828-32102866	-	-	IGV_LINK	2116.9	50.3217	17.2748	83.3687	OK	1839.71	43.8531	14.3476	73.3587	OK	OK	-0.1985
ENSG0000012	-	-	ENSG0000012177	'TMEM39B	-	1:32013828-32102866	-	-	IGV_LINK	205.581	7.68287	0	22.4234	OK	301.118	11.1168	0	27.8702	OK	OK	0.53302
ENSG0000012	-	-	ENSG0000012179	'CCRL2	-	3:46407162-46412997	-	-	IGV_LINK	162.913	6.23965	1.07695	11.4023	OK	157.777	6.2537	0.786918	11.7205	OK	OK	0.003245
ENSG0000012	-	-	ENSG0000012180	'CCR2	-	3:46353733-46360928	-	-	IGV_LINK	764.14	20.644	7.7999	33.488	OK	349.585	9.23324	2.99149	15.475	OK	OK	-1.16081
ENSG0000012	-	-	ENSG0000012185	'POLR3GL	-	1:145911349-145978848	-	-	IGV_LINK	596.378	34.7861	0	81.7529	OK	598.11	35.0246	0	80.5578	OK	OK	0.009855
ENSG0000012	-	-	ENSG0000012185	'GHSR	-	3:172445132-172448456	-	-	IGV_LINK	0	0	0	0	OK	0	0	0	0	OK	NOTEST	0
ENSG0000012	-	-	ENSG0000012185	'TNFSF10	-	3:172505507-172523507	-	-	IGV_LINK	5765.96	213.918	61.8986	365.938	OK	4300.21	161.601	49.0358	274.165	OK	OK	-0.40463
ENSG0000012	-	-	ENSG0000012186	'ZNF639	-	3:179322990-179338583	-	-	IGV_LINK	320.977	12.8388	2.68308	22.9945	OK	274.306	11.2752	2.15158	20.3989	OK	OK	-0.18735
ENSG0000012	-	-	ENSG0000012187	'SLITRK3	-	3:165186719-165197109	-	-	IGV_LINK	0	0	0	0	OK	0	0	0	0	OK	NOTEST	0
ENSG0000012	-	-	ENSG0000012187	'PIK3CA	-	3:179148113-179267002	-	-	IGV_LINK	432.495	7.74689	0	18.9514	OK	343.398	5.27643	0	14.0387	OK	OK	-0.55406
ENSG0000012	-	-	ENSG0000012189	'PDS5A	-	4:39822862-39977956	-	-	IGV_LINK	1634.95	14.512	0	29.3397	OK	1449.9	12.071	5.10738	19.0347	OK	OK	-0.2657
ENSG0000012	-	-	ENSG0000012189	'TMEM156	-	4:38966743-39032922	-	-	IGV_LINK	88.2446	4.31121	0	9.58133	OK	64.9672	3.8412	0	8.78334	OK	OK	-0.16654
ENSG0000012	-	-	ENSG0000012189	'LIAS	-	4:39454123-39485109	-	-	IGV_LINK	415.04	15.3297	0	113.558	OK	347.523	12.8669	0	84.0778	OK	OK	-0.25267
ENSG0000012	-	-	ENSG0000012189	'CPXM2	-	10:123706206-123940267	-	-	IGV_LINK	2017.99	33.728	14.9473	52.5087	OK	480.551	8.02387	2.98404	13.0637	OK	OK	-2.07158
ENSG0000012	-	-	ENSG0000012190	'TMEM54	-	1:32885993-32901438	-	-	IGV_LINK	554.68	39.4718	14.7252	64.2184	OK	950.789	67.58	28.1493	107.011	OK	OK	0.775773
ENSG0000012	-	-	ENSG0000012190	'ZSCAN20	-	1:33472644-33496507	-	-	IGV_LINK	101.821	1.46405	0.1561	2.77201	OK	106.216	1.48502	0.156143	2.81389	OK	OK	0.020511
ENSG0000012	-	-	ENSG0000012190	'CSMD2	-	1:33513998-34165842	-	-	IGV_LINK	27.1522	0.291239	0	1.04443	OK	16.4996	0.546855	0	1.59901	OK	NOTEST	0.908953
ENSG0000012	-	-	ENSG0000012190	'HPCA	-	1:32885993-32901438	-	-	IGV_LINK	40.7283	3.15784	0	11.8564	OK	32.9992	2.86443	0	13.4012	OK	OK	-0.14069
ENSG0000012	-	-	ENSG0000012193	'LRIF1	-	1:110943466-110964649	-	-	IGV_LINK	190.065	3.71763	0.727402	6.70787	OK	244.4	4.88495	1.103	8.66689	OK	OK	0.393958
ENSG0000012	-	-	ENSG0000012193	'TMIGD3	-	1:111483347-111768016	-	-	IGV_LINK	35.4236	2.50526	0	12.5035	OK	48.4676	3.5809	0	15.1223	OK	OK	0.515362
ENSG0000012	-	-	ENSG0000012194	'CLCC1	-	1:108815897-108963484	-	-	IGV_LINK	700.138	16.4202	0	34.7013	OK	871.385	20.582	4.16948	36.9945	OK	OK	0.325912
ENSG0000012	-	-	ENSG0000012195	'GPSM2	-	1:108815897-108963484	-	-	IGV_LINK	209.46	5.28022	0	15.8756	OK	218.62	4.51028	0	14.5762	OK	OK	-0.22738
ENSG0000012	-	-	ENSG0000012196	'GTDC1	-	2:143937072-144332568	-	-	IGV_LINK	334.554	9.71912	0.516431	18.9218	OK	453.739	12.2692	1.33098	23.2073	OK	OK	0.336139
ENSG0000012	-	-	ENSG0000012196	'CXCR4	-	2:136114348-136118165	-	-	IGV_LINK	3715	137.444	58.549	216.338	OK	4122.84	153.153	63.057	243.248	OK	OK	0.156131
ENSG0000012	-	-	ENSG0000012198	'ZRANB3	-	2:135052264-135531236	-	-	IGV_LINK	67.8804	1.10523	0	4.69954	OK	84.5604	1.17275	0	4.47157	OK	OK	0.085548
ENSG0000012	-	-	ENSG0000012198	'ACVR2A	-	2:147844516-148516971	-	-	IGV_LINK	146.428	2.8597	0	8.44678	OK	167.058	3.01234	0	8.50902	OK	OK	0.075022
ENSG0000012	-	-	ENSG0000012200	'POLK	-	5:75368485-75601144	-	-	IGV_LINK	437.344	9.23512	0	19.9486	OK	395.99	8.11374	0	17.6753	OK	OK	-0.18676
ENSG0000012	-	-	ENSG0000012201	'SV2C	-	5:76081945-76353939	-	-	IGV_LINK	4.8486	0.025155	0	0.290455	OK	6.18735	0.032104	0	0.299436	OK	NOTEST	0.351905
ENSG0000012	-	-	ENSG0000012202	'FLT3	-	13:28003273-28100592	-	-	IGV_LINK	79.5171	1.23498	0.036192	2.43377	OK	44.3427	0.705093	0	1.56419	OK	OK	-0.8086
ENSG0000012	-	-	ENSG0000012202	'RPL21	-	13:27251308-27256691	-	-	IGV_LINK	15366.2	1573.36	247.334	2899.39	OK	10369	1052.71	233.901	1871.52	OK	OK	-0.57974
ENSG0000012	-	-	ENSG0000012203	'MTIF3	-	13:27424543-27450591	-	-	IGV_LINK	639.046	52.4143	0	115.041	OK	578.517	48.4279	0	114.55	OK	OK	-0.11412
ENSG0000012	-	-	ENSG0000012203	'GTF3A	-	13:27424543-27450591	-	-	IGV_LINK	1183.06	62.1493	12.6188	111.68	OK	1596.34	83.6705	24.1687	143.172	OK	OK	0.428982
ENSG0000012	-	-	ENSG0000012203	'RASL11A	-	13:27270326-27273690	-	-	IGV_LINK	167.762	13.1495	0	27.1309	OK	465.082	31.1023	5.34752	56.857	OK	OK	1.24201
ENSG0000012	-	-	ENSG0000012204	'UBL3	-	13:29764370-29850684	-	-	IGV_LINK	871.779	11.6656	4.97037	18.3608	OK	784.762	10.5041	4.29899	16.7092	OK	OK	-0.15131
ENSG0000012	-	-	ENSG0000012204	'LINC00544	-	13:29935904-29950488	-	-	IGV_LINK	4.8486	0.606786	0	1.56926	OK	0	0	0	0	OK	OK	'-inf
ENSG0000012	-	-	ENSG0000012206	'FYTTD1	-	3:197671392-197787596	-	-	IGV_LINK	795.171	21.3453	0	46.1345	OK	628.016	15.4827	0	33.9286	OK	OK	-0.46326
ENSG0000012	-	-	ENSG0000012208	'MTERF4	-	2:240998837-241102332	-	-	IGV_LINK	1338.21	49.6333	5.57391	93.6927	OK	941.508	40.4572	8.08879	72.8255	OK	OK	-0.29491
ENSG0000012	-	-	ENSG0000012212	'XPNPEP2	-	X:129738973-129769538	-	-	IGV_LINK	1.93944	0.035286	0	0.179343	OK	0	0	0	0	OK	NOTEST	'-inf
ENSG0000012	-	-	ENSG0000012212	'SASH3	-	X:129779978-129796963	-	-	IGV_LINK	1358.52	35.3889	5.50114	65.2766	OK	1151.68	28.985	5.91606	52.054	OK	OK	-0.28799
ENSG0000012	-	-	ENSG0000012212	'OCRL	-	X:129539848-129592561	-	-	IGV_LINK	556.62	6.42303	2.32281	10.5232	OK	745.576	8.72897	3.17715	14.2808	OK	OK	0.442558
ENSG0000012	-	-	ENSG0000012213	'PAEP	-	9:135561755-135566955	-	-	IGV_LINK	0	0	0	0	OK	10.3122	0.728748	0	1.84008	OK	OK	inf
ENSG0000012	-	-	ENSG0000012213	'OBP2A	-	9:135546138-135549969	-	-	IGV_LINK	0.969721	0.147895	0	0.829164	OK	0	0	0	0	OK	NOTEST	'-inf
ENSG0000012	-	-	ENSG0000012214	'MRPS2	-	9:135495180-135506447	-	-	IGV_LINK	646.804	28.4598	9.37317	47.5464	OK	711.545	31.3934	10.0732	52.7137	OK	OK	0.141537
ENSG0000012	-	-	ENSG0000012214	'TBX22	-	X:80014755-80031769	-	-	IGV_LINK	0	0	0	0	OK	0	0	0	0	OK	NOTEST	0
ENSG0000012	-	-	ENSG0000012217	'FMOD	-	1:203340627-203351489	-	-	IGV_LINK	884.385	15.8854	6.61996	25.1507	OK	2324.38	44.0069	16.5955	71.4184	OK	OK	1.47003
ENSG0000012	-	-	ENSG0000012218	'MYOG	-	1:203083131-203086036	-	-	IGV_LINK	0.969721	0.040317	0	0.157912	OK	0	0	0	0	OK	NOTEST	'-inf
ENSG0000012	-	-	ENSG0000012218	'LAX1	-	1:203765175-203776233	-	-	IGV_LINK	128.003	3.33626	0.256974	6.41556	OK	121.685	2.69824	0.261328	5.13515	OK	OK	-0.30622
ENSG0000012	-	-	ENSG0000012219	'PLG	-	6:160702237-160753315	-	-	IGV_LINK	12.6064	0.348688	0	1.19646	OK	4.1249	0.289234	0	1.0704	OK	NOTEST	-0.2697
ENSG0000012	-	-	ENSG0000012220	'KIAA1191	-	5:176346060-176361968	-	-	IGV_LINK	2945.04	74.2128	21.0094	127.416	OK	5286.06	133.339	36.4119	230.266	OK	OK	0.845361
ENSG0000012	-	-	ENSG0000012221	'COPA	-	1:160289272-160358952	-	-	IGV_LINK	3590.88	51.9713	0	109.185	OK	5106.63	76.0138	0	163.789	OK	OK	0.548546
ENSG0000012	-	-	ENSG0000012222	'CD244	-	1:160830159-160862855	-	-	IGV_LINK	215.278	5.68407	0.91854	10.4496	OK	141.278	3.75712	0.189709	7.32453	OK	OK	-0.5973
ENSG0000012	-	-	ENSG0000012222	'LY9	-	1:160796073-160828261	-	-	IGV_LINK	126.064	4.25504	0.470297	8.03979	OK	148.496	5.18592	0.223674	10.1482	OK	OK	0.285427
ENSG0000012	-	-	ENSG0000012225	'HS3ST2	-	16:22814176-22916338	-	-	IGV_LINK	4.8486	0.130522	0	0.325666	OK	196.964	5.22651	1.21515	9.23787	OK	OK	5.32348
ENSG0000012	-	-	ENSG0000012225	'RBBP6	-	16:24537692-24572863	-	-	IGV_LINK	1231.55	22.7233	0	47.9139	OK	1052.88	20.5943	0	43.3165	OK	OK	-0.14193
ENSG0000012	-	-	ENSG0000012229	'ZC3H7A	-	16:11750585-11797267	-	-	IGV_LINK	1803.68	43.0635	4.71515	81.4118	OK	1121.97	22.7644	6.884	38.6448	OK	OK	-0.91969
ENSG0000012	-	-	ENSG0000012230	'PRM2	-	16:11249618-11351762	-	-	IGV_LINK	0	0	0	0	OK	0	0	0	0	OK	NOTEST	0
ENSG0000012	-	-	ENSG0000012233	'SERAC1	-	6:158109514-158168270	-	-	IGV_LINK	96.9721	1.5873	0	3.71748	OK	89.7166	1.80454	0	4.66192	OK	OK	0.185049
ENSG0000012	-	-	ENSG0000012235	'ANXA11	-	10:80150888-80205572	-	-	IGV_LINK	3111.83	85.808	1.18568	170.43	OK	2141.85	57.4383	7.79642	107.08	OK	OK	-0.5791
ENSG0000012	-	-	ENSG0000012236	'LDB3	-	10:86668448-86736068	-	-	IGV_LINK	19.3944	0.378279	0	0.93864	OK	17.5308	0.557896	0	1.85265	OK	NOTEST	0.560545
ENSG0000012	-	-	ENSG0000012237	'OPN4	-	10:86654556-86666848	-	-	IGV_LINK	0	0	0	0	OK	1.03122	0.027223	0	0.14011	OK	NOTEST	inf
ENSG0000012	-	-	ENSG0000012237	'FAM35A	-	10:87050485-87191468	-	-	IGV_LINK	844.627	21.8225	0	57.2638	OK	467.145	13.1953	0	34.446	OK	OK	-0.7258
ENSG0000012	-	-	ENSG0000012237	'FAM213A	-	10:80407828-80437115	-	-	IGV_LINK	1983.08	59.2025	19.1545	99.2506	OK	856.948	31.4841	7.89707	55.0711	OK	OK	-0.91104
ENSG0000012	-	-	ENSG0000012238	'ZNF205	-	16:3110459-3142805	-	-	IGV_LINK	343.281	10.2904	0	20.5941	OK	312.461	9.53694	0	19.4467	OK	OK	-0.1097
ENSG0000012	-	-	ENSG0000012239	'NAA60	-	16:3365098-3577400	-	-	IGV_LINK	769.958	28.5037	0	73.5288	OK	966.137	38.5388	0	112.953	OK	OK	0.435164
ENSG0000012	-	-	ENSG0000012240	'RPL5	-	1:92832024-92961522	-	-	IGV_LINK	14840.6	960.83	233.529	1688.13	OK	10737.1	696.254	227.509	1165	OK	OK	-0.46467
ENSG0000012	-	-	ENSG0000012241	'ODF2L	-	1:86346823-86396342	-	-	IGV_LINK	651.652	18.7791	0	41.0075	OK	517.675	14.655	0	31.6289	OK	OK	-0.35774
ENSG0000012	-	-	ENSG0000012242	'PTGFR	-	1:78229598-78539749	-	-	IGV_LINK	4.8486	0.065613	0	0.198602	OK	2.06245	0.027904	0	0.125381	OK	NOTEST	-1.23354
ENSG0000012	-	-	ENSG0000012243	'SPATA1	-	1:84498324-84574480	-	-	IGV_LINK	13.5761	2.31869	0	16.084	OK	15.4684	2.82413	0	20.0544	OK	OK	0.284499
ENSG0000012	-	-	ENSG0000012243	'TRMT13	-	1:100133149-100178273	-	-	IGV_LINK	240.491	8.25772	0	18.4271	OK	184.589	4.92837	0	10.9872	OK	OK	-0.74463
ENSG0000012	-	-	ENSG0000012247	'LRRC39	-	1:100133149-100178273	-	-	IGV_LINK	73.6988	2.6304	0	6.94472	OK	48.4676	1.59984	0	4.77285	OK	OK	-0.71735
ENSG0000012	-	-	ENSG0000012248	'RWDD3	-	1:95117337-95278940	-	-	IGV_LINK	161.31	9.07077	0.761618	17.3799	OK	174.663	10.2717	1.35027	19.1931	OK	OK	0.179374
ENSG0000012	-	-	ENSG0000012248	'ZNF644	-	1:90915297-91022272	-	-	IGV_LINK	521.71	9.85927	0	20.7982	OK	478.488	8.76938	0	17.7716	OK	OK	-0.16901
ENSG0000012	-	-	ENSG0000012248	'CCDC18	-	1:93149741-93369498	-	-	IGV_LINK	180.368	7.15162	0	33.6705	OK	312.461	12.3665	0	53.5321	OK	OK	0.790093
ENSG0000012	-	-	ENSG0000012248	'RPAP2	-	1:92246401-92402056	-	-	IGV_LINK	421.828	4.47039	0	11.379	OK	380.522	4.73349	0	11.9468	OK	OK	0.082504
ENSG0000012	-	-	ENSG0000012249	'PQLC1	-	18:79902419-79951664	-	-	IGV_LINK	480.012	12.4756	4.22958	20.7217	OK	645.547	17.529	5.33881	29.7192	OK	OK	0.490632
ENSG0000012	-	-	ENSG0000012250	'BBS9	-	7:33129243-33606068	-	-	IGV_LINK	395.646	9.16106	0.454869	17.8672	OK	369.179	8.23522	0	16.8867	OK	OK	-0.15371
ENSG0000012	-	-	ENSG0000012251	'PMS2	-	7:5973238-6009125	-	-	IGV_LINK	326.796	8.11982	2.29087	13.9488	OK	310.399	7.43081	2.10134	12.7603	OK	OK	-0.12793
ENSG0000012	-	-	ENSG0000012251	'ZMIZ2	-	7:44748580-44769881	-	-	IGV_LINK	4406.41	107.629	14.8533	200.404	OK	3103.99	71.685	11.3872	131.983	OK	OK	-0.58632
ENSG0000012	-	-	ENSG0000012254	'OCM	-	7:5879826-5886362	-	-	IGV_LINK	3.87888	0.418388	0	1.1143	OK	3.09367	0.334431	0	0.922495	OK	NOTEST	-0.32313
ENSG0000012	-	-	ENSG0000012254	'SEPT7	-	7:35800931-35907105	-	-	IGV_LINK	2076.17	98.6967	29.6506	167.743	OK	1871.67	89.7105	28.5842	150.837	OK	OK	-0.13773

ENSG0000012	-	-	ENSG0000012254	'EEPD1	-	7:36153148-36301543	-	-	IGV_LINK	145.458	6.86164	0	15.0149	OK	376.397	16.8126	0.256266	33.3689	OK	OK	1.29291
ENSG0000012	-	-	ENSG0000012254	'KIAA0087	-	7:26533120-26541048	-	-	IGV_LINK	25.2127	0.345671	0	0.977825	OK	10.3122	0.141422	0	0.403054	OK	NOTEST	-1.28939
ENSG0000012	-	-	ENSG0000012255	'KLHL7	-	7:23105757-23177914	-	-	IGV_LINK	233.703	9.47277	0	21.9445	OK	184.589	6.08032	0	13.656	OK	OK	-0.63964
ENSG0000012	-	-	ENSG0000012255	'HERPUD2	-	7:35632658-35699413	-	-	IGV_LINK	655.531	16.4976	3.257	29.7381	OK	672.359	15.6624	4.41073	26.9141	OK	OK	-0.07495
ENSG0000012	-	-	ENSG0000012256	'CBX3	-	7:26189926-26213356	-	-	IGV_LINK	1844.41	72.3071	0	221.559	OK	1908.8	72.136	0	208.436	OK	OK	-0.00342
ENSG0000012	-	-	ENSG0000012256	'HNRNPA2B1	-	7:26189926-26213356	-	-	IGV_LINK	12038.1	390.556	53.1957	727.917	OK	10761.9	356.719	34.3849	679.054	OK	OK	-0.13074
ENSG0000012	-	-	ENSG0000012257	'WIPF3	-	7:29806485-29917066	-	-	IGV_LINK	96.9721	1.43846	0.172258	2.70466	OK	135.09	1.97788	0.363191	3.59257	OK	OK	0.459432
ENSG0000012	-	-	ENSG0000012258	'NXPH1	-	7:8433954-8752963	-	-	IGV_LINK	0	0	0	0	OK	0	0	0	0	OK	NOTEST	0
ENSG0000012	-	-	ENSG0000012258	'NPY	-	7:24284162-24291865	-	-	IGV_LINK	0	0	0	0	OK	0	0	0	0	OK	NOTEST	0
ENSG0000012	-	-	ENSG0000012259	'FAM126A	-	7:22889370-23014130	-	-	IGV_LINK	211.399	2.70638	0	5.91916	OK	235.119	3.16411	0	6.74064	OK	OK	0.225433
ENSG0000012	-	-	ENSG0000012259	'HOXA7	-	7:27106183-27157936	-	-	IGV_LINK	45.5769	1.39178	0	4.73502	OK	32.9992	1.48063	0	6.71338	OK	OK	0.089278
ENSG0000012	-	-	ENSG0000012264	'INHBA	-	7:41667167-41779388	-	-	IGV_LINK	18.4247	0.30071	0	0.849843	OK	22.6869	0.289784	0	0.792566	OK	NOTEST	-0.05339
ENSG0000012	-	-	ENSG0000012264	'FKBP9	-	7:32957403-33006931	-	-	IGV_LINK	1229.61	28.1964	6.10701	50.2858	OK	1495.28	39.4252	7.38202	71.4685	OK	OK	0.483609
ENSG0000012	-	-	ENSG0000012264	'NT5C3A	-	7:33014113-33062797	-	-	IGV_LINK	512.982	24.851	2.24982	47.4521	OK	643.484	29.5151	1.81947	57.2107	OK	OK	0.24815
ENSG0000012	-	-	ENSG0000012264	'ARL4A	-	7:12686855-12690934	-	-	IGV_LINK	111.518	4.87353	0	9.97221	OK	241.307	12.1038	3.10384	21.1038	OK	OK	1.31242
ENSG0000012	-	-	ENSG0000012267	'CCZ1	-	7:5898724-5970683	-	-	IGV_LINK	168.731	5.40539	0.197526	10.6133	OK	151.59	4.77018	0.407605	9.13275	OK	OK	-0.18036
ENSG0000012	-	-	ENSG0000012267	'POLM	-	7:44072246-44082540	-	-	IGV_LINK	670.077	18.0581	5.99033	30.1259	OK	672.359	21.8947	4.86795	38.9215	OK	OK	0.277937
ENSG0000012	-	-	ENSG0000012267	'RAMP3	-	7:45157790-45186302	-	-	IGV_LINK	513.952	26.0487	9.5655	42.5318	OK	743.513	37.0206	14.9425	59.0987	OK	OK	0.50712
ENSG0000012	-	-	ENSG0000012268	'MRM2	-	7:2234230-2242198	-	-	IGV_LINK	422.798	14.6007	4.9843	24.2171	OK	443.427	16.6415	4.21097	29.072	OK	OK	0.188747
ENSG0000012	-	-	ENSG0000012269	'TWIST1	-	7:19020990-19117672	-	-	IGV_LINK	1.93944	0.127454	0	0.495162	OK	2.06245	0.077885	0	0.400534	OK	NOTEST	-0.71055
ENSG0000012	-	-	ENSG0000012269	'SMU1	-	9:33041763-33076659	-	-	IGV_LINK	865.96	7.02233	2.98944	11.0552	OK	975.539	7.91224	3.41573	12.4088	OK	OK	0.172137
ENSG0000012	-	-	ENSG0000012269	'GLIPR2	-	9:36136535-36163913	-	-	IGV_LINK	367.524	11.517	3.87511	19.1588	OK	317.617	10.6299	3.31088	17.949	OK	OK	-0.11563
ENSG0000012	-	-	ENSG0000012269	'SLC25A51	-	9:37510891-38069211	-	-	IGV_LINK	111.454	4.88828	0	17.5711	OK	124.778	5.292	0	19.3304	OK	OK	0.114488
ENSG0000012	-	-	ENSG0000012270	'CLTA	-	9:36190855-36304924	-	-	IGV_LINK	1846.35	115.156	39.788	190.524	OK	1803.61	112.746	35.0862	190.407	OK	OK	-0.03051
ENSG0000012	-	-	ENSG0000012270	'RECK	-	9:36036432-36124451	-	-	IGV_LINK	206.55	2.8434	0.492758	5.19404	OK	177.371	2.64204	0.291839	4.99224	OK	OK	-0.10597
ENSG0000012	-	-	ENSG0000012271	'SPINK4	-	9:33218364-33264720	-	-	IGV_LINK	0	0	0	0	OK	0	0	0	0	OK	NOTEST	0
ENSG0000012	-	-	ENSG0000012272	'TAF1L	-	9:32629453-32648685	-	-	IGV_LINK	0	0	0	0	OK	0	0	0	0	OK	NOTEST	0
ENSG0000012	-	-	ENSG0000012272	'ACO1	-	9:32384602-32454769	-	-	IGV_LINK	1072.51	16.5807	6.93351	26.2279	OK	1745.86	27.1507	11.7562	42.5452	OK	OK	0.711481
ENSG0000012	-	-	ENSG0000012273	'PHF24	-	9:34957607-34982544	-	-	IGV_LINK	18.4247	0.371757	0	1.79385	OK	15.4684	0.592991	0	2.35253	OK	OK	0.673652
ENSG0000012	-	-	ENSG0000012273	'DNAI1	-	9:34398183-34520989	-	-	IGV_LINK	145.458	8.20371	0	30.0603	OK	122.716	5.49088	0	17.8663	OK	OK	-0.57924
ENSG0000012	-	-	ENSG0000012274	'DCAF10	-	9:37510891-38069211	-	-	IGV_LINK	415.04	6.39285	0	22.8679	OK	434.146	9.61343	0	36.6589	OK	OK	0.588591
ENSG0000012	-	-	ENSG0000012275	'CNTFR	-	9:34551431-34590140	-	-	IGV_LINK	0	0	0	0	OK	2.06245	0.155528	0	0.477612	OK	NOTEST	inf
ENSG0000012	-	-	ENSG0000012277	'KIAA1549	-	7:138706294-138981318	-	-	IGV_LINK	552.741	3.32316	1.1789	5.46743	OK	301.118	1.74595	0.504972	2.98693	OK	OK	-0.92855
ENSG0000012	-	-	ENSG0000012277	'TRIM24	-	7:138460333-138589993	-	-	IGV_LINK	287.037	3.68428	1.01245	6.3561	OK	224.807	3.44781	0.24765	6.64797	OK	OK	-0.0957
ENSG0000012	-	-	ENSG0000012278	'C7orf49	-	7:134986507-135170795	-	-	IGV_LINK	1062.81	59.5229	0	136.251	OK	1267.38	70.479	0	168.787	OK	OK	0.243749
ENSG0000012	-	-	ENSG0000012278	'CALD1	-	7:134744251-134970728	-	-	IGV_LINK	4520.84	82.9495	24.3971	141.502	OK	4855.01	94.1458	0	188.723	OK	OK	0.182664
ENSG0000012	-	-	ENSG0000012278	'AKR1D1	-	7:138002323-138117986	-	-	IGV_LINK	0	0	0	0	OK	0	0	0	0	OK	NOTEST	0
ENSG0000012	-	-	ENSG0000012282	'NUDT10	-	X:51332230-51337525	-	-	IGV_LINK	5.81832	0.187955	0	0.460957	OK	2.06245	0.066665	0	0.210563	OK	NOTEST	-1.49539
ENSG0000012	-	-	ENSG0000012285	'SFTPA1	-	10:79610938-79615455	-	-	IGV_LINK	0	0	0	0	OK	0	0	0	0	OK	NOTEST	0
ENSG0000012	-	-	ENSG0000012285	'NEUROG3	-	10:69571697-69573238	-	-	IGV_LINK	0.969721	0.045409	0	0.177857	OK	0	0	0	0	OK	NOTEST	'-inf
ENSG0000012	-	-	ENSG0000012286	'PLAU	-	10:73909176-73922777	-	-	IGV_LINK	438.314	11.5048	3.26314	19.7465	OK	1079.69	28.0135	10.9694	45.0575	OK	OK	1.28388
ENSG0000012	-	-	ENSG0000012286	'SRGN	-	10:69088105-69104811	-	-	IGV_LINK	1841.5	101.173	44.7598	157.587	OK	3129.77	172.67	72.462	272.878	OK	OK	0.771191
ENSG0000012	-	-	ENSG0000012286	'CHST3	-	10:71964364-72013564	-	-	IGV_LINK	212.369	1.76067	0.431744	3.0896	OK	157.777	1.3083	0.245754	2.37084	OK	OK	-0.42844
ENSG0000012	-	-	ENSG0000012287	'BICC1	-	10:58513139-58831437	-	-	IGV_LINK	1366.34	15.507	6.19715	24.8168	OK	744.544	8.18963	3.12006	13.2592	OK	OK	-0.92105
ENSG0000012	-	-	ENSG0000012287	'ARL4AP1	-	10:60026297-60733490	-	-	IGV_LINK	0	0	0	0	OK	0	0	0	0	OK	NOTEST	0
ENSG0000012	-	-	ENSG0000012287	'CISD1	-	10:58269057-58289586	-	-	IGV_LINK	654.561	56.4086	16.4619	96.3553	OK	630.078	51.2346	12.9274	89.5418	OK	OK	-0.1388
ENSG0000012	-	-	ENSG0000012287	'EGR2	-	10:62811995-62919900	-	-	IGV_LINK	144.488	3.54715	0	7.40176	OK	90.7478	2.14618	0	4.70999	OK	OK	-0.72489
ENSG0000012	-	-	ENSG0000012288	'ECD	-	10:73110374-73358859	-	-	IGV_LINK	593.469	15.3429	0	36.158	OK	464.051	12.3835	0	26.8663	OK	OK	-0.30916
ENSG0000012	-	-	ENSG0000012288	'P4HA1	-	10:73007216-73096974	-	-	IGV_LINK	2122.72	47.1055	19.5729	74.638	OK	1192.1	26.5939	10.4375	42.7502	OK	OK	-0.8248
ENSG0000012	-	-	ENSG0000012291	'SLC25A16	-	10:68477998-68527474	-	-	IGV_LINK	468.375	13.5717	0	29.8616	OK	318.649	7.17263	0	14.5586	OK	OK	-0.92003
ENSG0000012	-	-	ENSG0000012295	'ZWINT	-	10:56357227-56361275	-	-	IGV_LINK	268.613	12.629	1.81309	23.4449	OK	237.182	12.4327	1.01994	23.8454	OK	OK	-0.0226
ENSG0000012	-	-	ENSG0000012295	'VPS26A	-	10:69123511-69172861	-	-	IGV_LINK	647.773	15.6864	5.07318	26.2996	OK	438.271	11.3981	3.10761	19.6885	OK	OK	-0.46072
ENSG0000012	-	-	ENSG0000012296	'RBM19	-	12:113816737-113966371	-	-	IGV_LINK	1059.9	17.1956	6.11582	28.2753	OK	1094.13	18.5345	6.33749	30.7316	OK	OK	0.108177
ENSG0000012	-	-	ENSG0000012296	'CIT	-	12:119685789-119877291	-	-	IGV_LINK	2516.42	33.5943	0	81.0057	OK	547.58	6.98939	0	17.6948	OK	OK	-2.26498
ENSG0000012	-	-	ENSG0000012297	'IFT81	-	12:110124334-110218797	-	-	IGV_LINK	482.921	10.5949	3.27293	17.9168	OK	501.175	12.4885	2.51687	22.4601	OK	OK	0.237232
ENSG0000012	-	-	ENSG0000012297	'ACADS	-	12:120725734-120740008	-	-	IGV_LINK	1004.63	33.0821	13.9072	52.2571	OK	1569.52	51.5594	22.2521	80.8667	OK	OK	0.640183
ENSG0000012	-	-	ENSG0000012298	'HVCN1	-	12:110614026-110704950	-	-	IGV_LINK	334.554	13.5178	0	31.6398	OK	201.089	8.26613	0	22.025	OK	OK	-0.70957
ENSG0000012	-	-	ENSG0000012300	'NME2P1	-	12:120282302-120282716	-	-	IGV_LINK	0	0	0	0	OK	2.06245	0.483081	0	1.41999	OK	NOTEST	inf
ENSG0000012	-	-	ENSG0000012306	'DDX54	-	12:113149857-113185479	-	-	IGV_LINK	1059.9	27.3602	3.11393	51.6066	OK	1166.32	27.743	2.11699	53.369	OK	OK	0.020042
ENSG0000012	-	-	ENSG0000012306	'MED13L	-	12:115953871-116277338	-	-	IGV_LINK	3179.71	22.6215	0	61.438	OK	1681.93	11.1469	0	28.7668	OK	OK	-1.02105
ENSG0000012	-	-	ENSG0000012308	'CDKN2C	-	1:50960744-50974633	-	-	IGV_LINK	503.285	20.4058	7.16003	33.6517	OK	319.68	12.6924	3.71645	21.6683	OK	OK	-0.68502
ENSG0000012	-	-	ENSG0000012309	'RNF11	-	1:51236270-51273455	-	-	IGV_LINK	953.235	50.15	5.29943	95.0007	OK	1157.03	65.2303	14.5763	115.884	OK	OK	0.379291
ENSG0000012	-	-	ENSG0000012309	'RASSF8	-	12:25939328-26079892	-	-	IGV_LINK	369.464	8.55577	0	19.7612	OK	354.741	6.51698	0	15.2203	OK	OK	-0.3927
ENSG0000012	-	-	ENSG0000012309	'BHLHE41	-	12:26120025-26833198	-	-	IGV_LINK	3470.63	75.6795	0	192.793	OK	3085.43	72.3867	0	185.795	OK	OK	-0.06418
ENSG0000012	-	-	ENSG0000012309	'SSPN	-	12:26120025-26833198	-	-	IGV_LINK	681.714	20.5652	0	85.0806	OK	430.021	13.9569	0	62.5798	OK	OK	-0.55923
ENSG0000012	-	-	ENSG0000012310	'ITPR2	-	12:26120025-26833198	-	-	IGV_LINK	832.02	4.7925	0	19.1911	OK	776.512	4.80181	0	15.8783	OK	OK	0.0028
ENSG0000012	-	-	ENSG0000012310	'CCDC91	-	12:28133248-28581511	-	-	IGV_LINK	328.735	12.682	1.61731	23.7468	OK	368.147	17.7785	0	57.9449	OK	OK	0.487344
ENSG0000012	-	-	ENSG0000012311	'NECAB1	-	8:90621994-90985257	-	-	IGV_LINK	271.522	7.47501	0	22.2316	OK	45.3739	1.58507	0	8.32297	OK	OK	-2.23753
ENSG0000012	-	-	ENSG0000012312	'WWP1	-	8:86333273-86514357	-	-	IGV_LINK	1220.88	27.6282	0	57.9538	OK	1694.3	34.622	0	71.013	OK	OK	0.325551
ENSG0000012	-	-	ENSG0000012313	'ACOT9	-	X:23702252-23766475	-	-	IGV_LINK	564.377	21.5623	5.57442	37.5502	OK	565.111	20.356	4.43017	36.2819	OK	OK	-0.08305
ENSG0000012	-	-	ENSG0000012313	'PRDX4	-	X:23664261-23686399	-	-	IGV_LINK	1335.31	90.4448	39.9793	140.91	OK	1757.21	119.121	52.5179	185.725	OK	OK	0.397323
ENSG0000012	-	-	ENSG0000012313	'DDX39A	-	19:14408818-14419383	-	-	IGV_LINK	950.326	43.5828	16.1305	71.035	OK	801.262	37.5591	13.8613	61.2568	OK	OK	-0.2146
ENSG0000012	-	-	ENSG0000012314	'PKN1	-	19:14433052-14471867	-	-	IGV_LINK	3000.32	67.6495	19.7367	115.562	OK	2866.81	63.0071	20.1747	105.84	OK	OK	-0.10256
ENSG0000012	-	-	ENSG0000012314	'TRIR	-	19:12730639-12734775	-	-	IGV_LINK	1898.71	135.144	60.3342	209.954	OK	1685.02	119.949	51.9702	187.928	OK	OK	-0.17208
ENSG0000012	-	-	ENSG0000012314	'ADGRE5	-	19:14380500-14408725	-	-	IGV_LINK	1951.08	46.6774	11.4133	81.9414	OK	2167.63	53.0604	12.3416	93.7791	OK	OK	0.184913
ENSG0000012	-	-	ENSG0000012315	'WDR83	-	19:12643274-12681902	-	-	IGV_LINK	259.885	16.8987	0	67.8594	OK	211.401	13.1259	0	62.0886	OK	OK	-0.3645
ENSG0000012	-	-	ENSG0000012315	'GIPC1	-	19:14477759-14496149	-	-	IGV_LINK	650.682	26.418	9.14354	43.6925	OK	750.732	29.0181	11.0907	46.9455	OK	OK	0.13543
ENSG0000012	-	-	ENSG0000012316	'ACTRT1	-	X:128050961-128052398	-	-	IGV_LINK	0	0	0	0	OK	0	0	0	0	OK	NOTEST	0
ENSG0000012	-	-	ENSG0000012317	'CCDC70	-	13:51861980-51866232	-	-	IGV_LINK	0	0	0	0	OK	0	0	0	0	OK	NOTEST	0
ENSG0000012	-	-	ENSG0000012317	'SPRYD7	-	13:49912705-49936490	-	-	IGV_LINK	159.034	3.18381	0.387271	5.98035	OK	184.589	3.70149	0.633922	6.76905	OK	OK	0.217351
ENSG0000012	-	-	ENSG0000012317	'EBPL	-	13:49660722-49691475	-	-	IGV_LINK	213.339	18.5487	4.91537	32.182	OK	236.151	19.0351	4.92531	33.1448	OK	OK	0.037341
ENSG0000012	-	-	ENSG0000012319	'ATP7B	-	13:51930435-52012125	-	-	IGV_LINK	341.342	3.88067	0	8.98183	OK	210.37	2.49084	0	6.03934	OK	OK	-0.63967
ENSG0000012	-	-	ENSG0000012320	'ZC3H13	-	13:45954464-46052759	-	-	IGV_LINK	1110.33	10.1409	0.490334	19.7914	OK	1068.35	8.65819	2.34673	14.9697	OK	OK	-0.22804
ENSG0000012	-	-	ENSG0000012320	'GUCY1B2	-	13:50994510-51080862	-	-	IGV_LINK	0.969721	0.020252	0	0.418754	OK	1.03122	0.021546	0	0.41886	OK	NOTEST	0.089333
ENSG0000012	-	-	ENSG0000012321	'NLN	-	5:65589679-65871725	-	-	IGV_LINK	233.703	3.53447	0	10.1517	OK	326.898	5.38494	0	13.5283	OK	OK	0.607436
ENSG0000012	-	-	ENSG0000012321	'CENPK	-	5:65517765-65563171	-	-	IGV_LINK	59.153	4.21835	0	9.27131	OK	64.9672	3.87582	0	8.67179	OK	OK	-0.12218
ENSG0000012	-	-	ENSG0000012324	'OPTN	-	10:12896624-13138308	-	-	IGV_LINK	3451.24	95.0927	20.3691	169.816	OK	2082.04	59.2738	9.78388	108.764	OK	OK	-0.68194
ENSG0000012	-	-	ENSG0000012324	'ITIH5	-	10:7559269-7666998	-	-	IGV_LINK	946.447	13.2487	3.61639	22.8811	OK	469.207	6.58058	1.47196	11.6892	OK	OK	-1.00957
ENSG0000012	-	-	ENSG0000012326	'ATF1	-	12:50763709-50821122	-	-	IGV_LINK	228.854	15.8623	0	32.772	OK	209.339	17.5672	2.21189	32.9224	OK	OK	0.147282
ENSG0000012	-	-	ENSG0000012329	'TSFM	-	12:57762333-57818704	-	-	IGV_LINK	240.491	15.033	0	35.7012	OK	383.341	23.8582	0	60.0447	OK	OK	0.666357
ENSG0000012	-	-	ENSG0000012330	'NEUROD4	-	12:55019944-55030014	-	-	IGV_LINK	0	0	0	0	OK	3.09367	0.046131	0	0.119182	OK	NOTEST	inf
ENSG0000012	-	-	ENSG0000012332	'ARHGAP9	-	12:57460134-57520517	-	-	IGV_LINK	1154.94	42.7619	0	95.8346	OK	1141.57	51.936	0	111.843	OK	OK	0.280411
ENSG0000012	-	-	ENSG0000012333	'NCKAP1L	-	12:54497710-54548238	-	-	IGV_LINK	2423.33	34.0605	5.1389	62.9822	OK	2133.6	30.0214	5.73345	54.3094	OK	OK	-0.18211
ENSG0000012	-	-	ENSG0000012334	'MMP19	-	12:55743279-55842966	-	-	IGV_LINK	155.155	2.41429	0	8.08995	OK	546.549	10.9993	0	29.74	OK	OK	2.18774
ENSG0000012	-	-	ENSG0000012334	'PFDN5	-	12:53295290-53307177	-	-	IGV_LINK	6452.52	879.004	277.73	1480.28	OK	4962.25	673.225	237.474	1108.98	OK	OK	-0.38478
ENSG0000012	-	-	ENSG0000012335	'SPATS2	-	12:49366583-49527424	-	-	IGV_LINK	521.71	11.483	0.156845	22.8092	OK	504.269	11.2469	1.68856	20.8052	OK	OK	-0.02998
ENSG0000012	-	-	ENSG0000012335	'ORMDL2	-	12:55743279-55842966	-	-	IGV_LINK	313.22	25.927	0	72.2752	OK	476.426	42.6619	0	117.637	OK	OK	0.718493
ENSG0000012	-	-	ENSG0000012335	'NR4A1	-	12:52022831-52059507	-	-	IGV_LINK	517.831	22.5765	2.41413	42.739	OK	1558.18	53.4212	5.54877	101.294	OK	OK	1.24259
ENSG0000012	-	-	ENSG0000012336	'PDE1B	-	12:54549349-54588659	-	-	IGV_LINK	388.858	7.67318	2.05494	13.2914	OK	289.774	6.19922	0	17.5585	OK	OK	-0.30774
ENSG0000012	-	-	ENSG0000012336	'HOXC13	-	12:53935327-53946544	-	-	IGV_LINK	0	0	0	0	OK	0	0	0	0	OK	NOTEST	0
ENSG0000012	-	-	ENSG0000012337	'CDK2	-	12:55954104-55973317	-	-	IGV_LINK	470.314	22.1956	0	44.442	OK	486.738	19.0792	0	38.4589	OK	OK	-0.21827
ENSG0000012	-	-	ENSG0000012338	'LRP1	-	12:57088893-57213351	-	-	IGV_LINK	4607.14	28.0174	0	86.6207	OK	7583.63	38.265	0	115.318	OK	OK	0.449701
ENSG0000012	-	-	ENSG0000012338	'HOXC11	-	12:53962307-53977643	-	-	IGV_LINK	105.7	2.95687	0.166464	5.74727	OK	76.3106	2.21127	0	4.67402	OK	OK	-0.41919
ENSG0000012	-	-	ENSG0000012339	'ATG101	-	12:52069245-52108261	-	-	IGV_LINK	215.278	15.9434	0	35.7839	OK	226.869	16.4324	0	35.4049	OK	OK	0.043582
ENSG0000012	-	-	ENSG0000012340	'NFE2	-	12:54230939-54351849	-	-	IGV_LINK	14.5458	0.526685	0	14.7723	OK	47.4363	1.76232	0	25.5604	OK	OK	1.74247
ENSG0000012	-	-	ENSG0000012340	'HOXC12	-	12:53954833-53958956	-	-	IGV_LINK	0	0	0	0	OK	0	0	0	0	OK	NOTEST	0
ENSG0000012	-	-	ENSG0000012341	'IKZF4	-	12:56007658-56041806	-	-	IGV_LINK	425.707	6.59058	0	13.6436	OK	235.119	3.29068	0	6.77045	OK	OK	-1.00202
ENSG0000012	-	-	ENSG0000012341	'SMUG1	-	12:54058253-54189008	-	-	IGV_LINK	513.704	28.7407	2.20802	55.2734	OK	600.173	39.1853	6.34586	72.0248	OK	OK	0.447218
ENSG0000012	-	-	ENSG0000012341	'TUBA1B	-	12:49127781-49147869	-	-	IGV_LINK	4783.63	245.387	0	494.654	OK	7082.45	375.2	0	788.469	OK	OK	0.6126
ENSG0000012	-	-	ENSG0000012342	'EEF1AKMT3	-	12:57762333-57818704	-	-	IGV_LINK	455.769	10.8667	0.666821	21.0666	OK	978.633	23.857	0	49.5169	OK	OK	1.13449
ENSG0000012	-	-	ENSG0000012344	'KBTBD4	-	11:47465932-47584562	-	-	IGV_LINK	409.222	10.1966	0	29.1828	OK	377.428	9.52608	0	27.8608	OK	OK	-0.09813
ENSG0000012	-	-	ENSG0000012344	'TYRL	-	11:49405090-49415789	-	-	IGV_LINK	0	0	0	0	OK	0	0	0	0	OK	NOTEST	0
ENSG0000012	-	-	ENSG0000012345	'SARDH	-	9:133663559-133739955	-	-	IGV_LINK	280.249	7.04405	1.13836	12.9497	OK	296.993	7.07499	1.24199	12.908	OK	OK	0.006323
ENSG0000012	-	-	ENSG0000012345	'DBH	-	9:133636359-133659344	-	-	IGV_LINK	82.4262	1.79317	0	4.20003	OK	21.6557	0.471326	0	1.31653	OK	OK	-1.92771
ENSG0000012	-	-	ENSG0000012347	'ATPAF1	-	1:46632736-46673867	-	-	IGV_LINK	928.992	31.1217	9.74407	52.4993	OK	964.195	31.463	11.3079	51.618	OK	OK	0.015735
ENSG0000012	-	-	ENSG0000012347	'STIL	-	1:47250138-47314147	-	-	IGV_LINK	74.6685	0.871959	0	1.76812	OK	141.278	1.73926	0.224483	3.25405	OK	OK	0.996146
ENSG0000012	-	-	ENSG0000012348	'HJURP	-	2:233775678-233854566	-	-	IGV_LINK	106.669	2.65876	0.094177	5.22335	OK	155.715	4.33418	0	10.5011	OK	OK	0.705005
ENSG0000012	-	-	ENSG0000012349	'IL13RA2	-	X:115003974-115019977	-	-	IGV_LINK	4.8486	0.270599	0	0.766181	OK	19.5933	0.922209	0	1.98188	OK	OK	1.76894
ENSG0000012	-	-	ENSG0000012350	'COL10A1	-	6:116100848-116249497	-	-	IGV_LINK	1.93944	0.035068	0	0.575585	OK	1.03122	0.018652	0	0.497858	OK	NOTEST	-0.91086
ENSG0000012	-	-	ENSG0000012350	'AMD1	-	6:110874769-110895713	-	-	IGV_LINK	948.387	18.8717	6.09868	31.6447	OK	907.478	19.0886	6.92868	31.2485	OK	OK	0.016487
ENSG0000012	-	-	ENSG0000012354	'NDUFAF4	-	6:96889312-96897881	-	-	IGV_LINK	171.641	10.2596	0	20.6688	OK	78.3731	3.47483	0	7.92778	OK	OK	-1.56195
ENSG0000012	-	-	ENSG0000012355	'USP45	-	6:99432378-99531918	-	-	IGV_LINK	163.883	2.81865	0	6.13345	OK	96.9351	1.62073	0	4.11268	OK	OK	-0.79836
ENSG0000012	-	-	ENSG0000012356	'PLP1	-	X:103773717-103792619	-	-	IGV_LINK	1.93944	0.176009	0	1.11863	OK	0	0	0	0	OK	NOTEST	'-inf
ENSG0000012	-	-	ENSG0000012356	'SERPINA7	-	X:106032441-106038738	-	-	IGV_LINK	0	0	0	0	OK	2.06245	0.047089	0	0.35832	OK	NOTEST	inf
ENSG0000012	-	-	ENSG0000012356	'MORF4L2	-	X:103675495-103691772	-	-	IGV_LINK	2165.39	143.283	30.5292	256.037	OK	2875.06	188.595	57.3572	319.833	OK	OK	0.396426
ENSG0000012	-	-	ENSG0000012356	'H2BFWT	-	X:104011146-104013687	-	-	IGV_LINK	0	0	0	0	OK	0	0	0	0	OK	NOTEST	0
ENSG0000012	-	-	ENSG0000012357	'RAB9B	-	X:103822321-103832228	-	-	IGV_LINK	60.1227	0.947632	0.011613	1.88365	OK	25.7806	0.406476	0	0.843416	OK	OK	-1.22116
ENSG0000012	-	-	ENSG0000012357	'NRK	-	X:105822542-105958610	-	-	IGV_LINK	1.93944	0.013854	0	0.158417	OK	1.03122	0.01156	0	0.154818	OK	NOTEST	-0.26116
ENSG0000012	-	-	ENSG0000012357	'FAM199X	-	X:104166619-104195902	-	-	IGV_LINK	626.439	5.1739	1.84987	8.49794	OK	1083.82	9.31758	3.50516	15.13	OK	OK	0.848702
ENSG0000012	-	-	ENSG0000012357	'ESX1	-	X:104250037-104254933	-	-	IGV_LINK	0	0	0	0	OK	0	0	0	0	OK	NOTEST	0
ENSG0000012	-	-	ENSG0000012358	'MAGEA9	-	X:149781929-149787737	-	-	IGV_LINK	0	0	0	0	OK	0	0	0	0	OK	NOTEST	0
ENSG0000012	-	-	ENSG0000012359	'ATXN3L	-	X:13310651-13320399	-	-	IGV_LINK	0	0	0	0	OK	0	0	0	0	OK	NOTEST	0

ENSG0000012	-	-	ENSG0000012359	'RAB9A	-	X:13689124-13710506	-	-	IGV_LINK	487.769	25.6975	5.95521	45.4397	OK	795.074	41.6964	10.4479	72.9449	OK	OK	0.698297
ENSG0000012	-	-	ENSG0000012360	'METTL8	-	2:171317404-171485052	-	-	IGV_LINK	211.399	5.60447	0	14.6415	OK	184.589	4.01434	0	10.464	OK	OK	-0.48142
ENSG0000012	-	-	ENSG0000012360	'TTC21B	-	2:165857474-165953843	-	-	IGV_LINK	613.833	14.5551	0	31.0619	OK	310.399	6.70422	0	13.7837	OK	OK	-1.11838
ENSG0000012	-	-	ENSG0000012360	'NMI	-	2:151270464-151290057	-	-	IGV_LINK	644.864	25.0807	9.30654	40.8548	OK	470.239	18.6044	6.57191	30.6368	OK	OK	-0.43093
ENSG0000012	-	-	ENSG0000012361	'TNFAIP6	-	2:151357591-151380048	-	-	IGV_LINK	428.616	20.5485	6.54949	34.5475	OK	209.339	9.91405	2.2612	17.5669	OK	OK	-1.05149
ENSG0000012	-	-	ENSG0000012361	'ACVR1C	-	2:157526766-157629005	-	-	IGV_LINK	4.8486	0.034588	0	0.17863	OK	4.1249	0.026355	0	0.167215	OK	NOTEST	-0.39217
ENSG0000012	-	-	ENSG0000012363	'BAZ2B	-	2:159318978-159617082	-	-	IGV_LINK	473.224	10.2639	0	23.7353	OK	477.457	9.16864	0	21.8988	OK	OK	-0.1628
ENSG0000012	-	-	ENSG0000012364	'SLC36A1	-	5:151437045-151492381	-	-	IGV_LINK	690.441	7.24881	2.3949	12.1027	OK	1021.94	10.7412	3.33745	18.1449	OK	OK	0.567338
ENSG0000012	-	-	ENSG0000012368	'LPGAT1	-	1:211743456-211853703	-	-	IGV_LINK	1570.95	11.8321	3.8018	19.8624	OK	2020.17	16.7251	0	37.6074	OK	OK	0.499312
ENSG0000012	-	-	ENSG0000012368	'BATF3	-	1:212686417-212699985	-	-	IGV_LINK	123.155	7.8541	0.120057	15.5881	OK	121.685	7.80303	0	15.892	OK	OK	-0.00941
ENSG0000012	-	-	ENSG0000012368	'G0S2	-	1:209661363-209734950	-	-	IGV_LINK	132.852	10.7963	1.03913	20.5534	OK	1096.19	89.2306	36.4692	141.992	OK	OK	3.04701
ENSG0000012	-	-	ENSG0000012370	'KCNJ2	-	17:70166960-70180048	-	-	IGV_LINK	296.734	3.37559	0.877561	5.87362	OK	339.273	4.03924	1.06155	7.01692	OK	OK	0.258943
ENSG0000012	-	-	ENSG0000012372	'RAP2C	-	X:132203023-132489968	-	-	IGV_LINK	400.495	15.7313	0	36.2868	OK	410.428	16.7157	0	46.9913	OK	OK	0.087564
ENSG0000012	-	-	ENSG0000012373	'EXOSC9	-	4:121801316-121823933	-	-	IGV_LINK	587.651	21.7713	5.56828	37.9743	OK	471.27	16.7229	4.77702	28.6688	OK	OK	-0.3806
ENSG0000012	-	-	ENSG0000012373	'PLA2G12A	-	4:109709988-109730077	-	-	IGV_LINK	620.621	23.1187	0	65.553	OK	783.731	33.1204	0	89.3967	OK	OK	0.518658
ENSG0000012	-	-	ENSG0000012381	'B9D2	-	19:41262495-41425005	-	-	IGV_LINK	200.732	13.9643	0	82.9283	OK	164.996	11.1253	0	65.2083	OK	OK	-0.3279
ENSG0000012	-	-	ENSG0000012381	'COQ8B	-	19:40691528-40765389	-	-	IGV_LINK	860.142	35.0086	0	80.7939	OK	1236.44	51.3552	0	121.497	OK	OK	0.552799
ENSG0000012	-	-	ENSG0000012383	'PFKFB2	-	1:207034365-207081024	-	-	IGV_LINK	402.434	4.63418	0.311483	8.95687	OK	290.805	3.51269	0	7.78477	OK	OK	-0.39974
ENSG0000012	-	-	ENSG0000012383	'C4BPA	-	1:207104261-207144972	-	-	IGV_LINK	16.4852	0.498378	0	1.24444	OK	545.518	15.8153	5.5078	26.1227	OK	OK	4.98793
ENSG0000012	-	-	ENSG0000012384	'C4BPB	-	1:207088841-207099993	-	-	IGV_LINK	5.81832	0.2528	0	0.87395	OK	7.21857	0.314768	0	0.934213	OK	NOTEST	0.316296
ENSG0000012	-	-	ENSG0000012387	'ZNF137P	-	19:52588504-52690496	-	-	IGV_LINK	106.669	5.99484	0	45.2745	OK	84.5604	5.68048	0	42.6656	OK	OK	-0.07771
ENSG0000012	-	-	ENSG0000012389	'RAB38	-	11:88098590-88175467	-	-	IGV_LINK	200.732	8.96017	2.20692	15.7134	OK	205.214	9.30905	2.46066	16.1574	OK	OK	0.055108
ENSG0000012	-	-	ENSG0000012390	'GPR83	-	11:94377310-94401419	-	-	IGV_LINK	15.5155	0.314891	0	0.715951	OK	10.3122	0.168915	0	0.396673	OK	NOTEST	-0.89856
ENSG0000012	-	-	ENSG0000012390	'AGO2	-	8:140520155-140635619	-	-	IGV_LINK	1083.18	5.931	0	21.4559	OK	653.797	2.67779	0	5.49128	OK	OK	-1.14723
ENSG0000012	-	-	ENSG0000012393	'MXD4	-	4:2247431-2262294	-	-	IGV_LINK	1919.08	35.5301	4.06898	66.9911	OK	2028.42	37.453	3.65184	71.2541	OK	OK	0.07604
ENSG0000012	-	-	ENSG0000012396	'PMS2P5	-	7:74894115-74897835	-	-	IGV_LINK	14.5458	2.3864	0	5.47113	OK	8.2498	1.35805	0	3.14185	OK	OK	-0.8133
ENSG0000012	-	-	ENSG0000012397	'CKS2	-	9:89311197-89316703	-	-	IGV_LINK	132.852	16.9975	3.1512	30.8437	OK	214.495	27.515	7.65051	47.3795	OK	OK	0.694899
ENSG0000012	-	-	ENSG0000012397	'DAW1	-	2:227871053-227924344	-	-	IGV_LINK	1.93944	0.13918	0	0.573775	OK	8.2498	0.275597	0	0.783976	OK	NOTEST	0.985605
ENSG0000012	-	-	ENSG0000012398	'ACSL3	-	2:222860933-222944639	-	-	IGV_LINK	468.375	13.9188	0	28.9939	OK	705.358	19.6925	0.182003	39.203	OK	OK	0.500616
ENSG0000012	-	-	ENSG0000012398	'CHPF	-	2:219538946-219550595	-	-	IGV_LINK	660.38	14.0403	5.01748	23.0631	OK	1245.72	26.4172	9.8352	42.9991	OK	OK	0.911902
ENSG0000012	-	-	ENSG0000012399	'DNPEP	-	2:219373545-219403633	-	-	IGV_LINK	1357.61	57.359	14.685	100.033	OK	1328.22	53.196	17.6106	88.7813	OK	OK	-0.10871
ENSG0000012	-	-	ENSG0000012399	'INHA	-	2:219550728-219575713	-	-	IGV_LINK	12.6064	0.660453	0	4.87586	OK	20.6245	1.16987	0	6.81773	OK	OK	0.824826
ENSG0000012	-	-	ENSG0000012400	'MOGAT1	-	2:222671785-222709930	-	-	IGV_LINK	0	0	0	0	OK	1.03122	0.066469	0	0.255011	OK	NOTEST	inf
ENSG0000012	-	-	ENSG0000012400	'OBSL1	-	2:219550728-219575713	-	-	IGV_LINK	1322.7	35.7636	7.87883	63.6483	OK	1165.28	29.2345	7.41388	51.0552	OK	OK	-0.29082
ENSG0000012	-	-	ENSG0000012401	'FAM124B	-	2:224378697-224402085	-	-	IGV_LINK	9.69721	0.236832	0	0.541929	OK	20.6245	0.502169	0	1.09072	OK	NOTEST	1.08431
ENSG0000012	-	-	ENSG0000012406	'SLC12A4	-	16:67939749-67969601	-	-	IGV_LINK	1541.78	29.5383	0	61.4302	OK	1277.04	26.7261	0	56.1237	OK	OK	-0.14434
ENSG0000012	-	-	ENSG0000012407	'ENKD1	-	16:67662944-67668758	-	-	IGV_LINK	589.59	26.6966	5.84328	47.55	OK	507.363	23.8095	4.84386	42.775	OK	OK	-0.16512
ENSG0000012	-	-	ENSG0000012408	'MC3R	-	20:56248731-56249815	-	-	IGV_LINK	0	0	0	0	OK	0	0	0	0	OK	NOTEST	0
ENSG0000012	-	-	ENSG0000012409	'GCNT7	-	20:56468584-56526142	-	-	IGV_LINK	2.90916	0.091623	0	1.42379	OK	2.06245	0.061487	0	1.15603	OK	NOTEST	-0.57543
ENSG0000012	-	-	ENSG0000012409	'CTCFL	-	20:57495965-57525652	-	-	IGV_LINK	0	0	0	0	OK	0	0	0	0	OK	NOTEST	0
ENSG0000012	-	-	ENSG0000012409	'HMGB1P1	-	20:57488391-57489027	-	-	IGV_LINK	0.969721	0.117912	0	0.461832	OK	1.03122	0.125703	0	0.470478	OK	NOTEST	0.092303
ENSG0000012	-	-	ENSG0000012409	'FAM210B	-	20:56358914-56368663	-	-	IGV_LINK	518.8	10.5939	3.46895	17.7189	OK	624.922	12.4846	4.47709	20.492	OK	OK	0.236906
ENSG0000012	-	-	ENSG0000012410	'PI3	-	20:45174875-45176544	-	-	IGV_LINK	0.969721	0.135111	0	0.529193	OK	29.9055	4.17862	0	8.59719	OK	OK	4.95081
ENSG0000012	-	-	ENSG0000012410	'FAM209A	-	20:56468584-56526142	-	-	IGV_LINK	10.9602	0.913211	0	7.75621	OK	7.22697	0.554344	0	5.8147	OK	OK	-0.72017
ENSG0000012	-	-	ENSG0000012410	'SNX21	-	20:45833809-45857406	-	-	IGV_LINK	187.156	7.58921	0	17.1661	OK	355.773	14.6329	0	29.7715	OK	OK	0.94719
ENSG0000012	-	-	ENSG0000012410	'SLPI	-	20:45252238-45254564	-	-	IGV_LINK	28.1219	3.74243	0	7.72591	OK	1935.61	258.291	114.629	401.953	OK	OK	6.10888
ENSG0000012	-	-	ENSG0000012411	'WFDC3	-	20:45747943-45791932	-	-	IGV_LINK	0.969721	0.239786	0	1.23049	OK	3.09367	0.47708	0	1.62065	OK	NOTEST	0.992486
ENSG0000012	-	-	ENSG0000012412	'TTPAL	-	20:44475885-44494603	-	-	IGV_LINK	267.643	2.6204	0.753859	4.48694	OK	637.297	6.537	1.97596	11.098	OK	OK	1.31884
ENSG0000012	-	-	ENSG0000012412	'PREX1	-	20:48624251-48849458	-	-	IGV_LINK	2664.79	25.3061	4.86569	45.7466	OK	3007.05	27.6081	8.10438	47.1118	OK	OK	0.125604
ENSG0000012	-	-	ENSG0000012413	'KCNS1	-	20:45092309-45101112	-	-	IGV_LINK	1.93944	0.025037	0	0.098425	OK	0	0	0	0	OK	NOTEST	'-inf
ENSG0000012	-	-	ENSG0000012414	'SLC12A5	-	20:46008907-46060152	-	-	IGV_LINK	1.93944	0.262798	0	3.7069	OK	10.3122	0.538028	0	4.4308	OK	NOTEST	1.03373
ENSG0000012	-	-	ENSG0000012414	'ARHGAP40	-	20:38601933-38651035	-	-	IGV_LINK	19.3944	0.460597	0	1.11171	OK	21.6557	0.526533	0	1.21442	OK	NOTEST	0.193017
ENSG0000012	-	-	ENSG0000012414	'SDC4	-	20:45325287-45348424	-	-	IGV_LINK	2190.6	50.5853	22.3517	78.8188	OK	3198.86	73.9028	30.1314	117.674	OK	OK	0.546913
ENSG0000012	-	-	ENSG0000012415	'NCOA3	-	20:47501901-47786616	-	-	IGV_LINK	1014.33	7.51577	0.900324	14.1312	OK	794.043	6.29463	0	20.9239	OK	OK	-0.2558
ENSG0000012	-	-	ENSG0000012415	'PIGT	-	20:45416066-45456934	-	-	IGV_LINK	1575.8	47.2834	17.8299	76.7368	OK	1836.61	52.3779	21.3433	83.4125	OK	OK	0.147625
ENSG0000012	-	-	ENSG0000012415	'SEMG2	-	20:45221299-45224458	-	-	IGV_LINK	0	0	0	0	OK	0	0	0	0	OK	NOTEST	0
ENSG0000012	-	-	ENSG0000012415	'MATN4	-	20:45293444-45317824	-	-	IGV_LINK	0.969721	0.036813	0	0.195581	OK	2.06245	0.061056	0	0.22915	OK	NOTEST	0.729935
ENSG0000012	-	-	ENSG0000012416	'NCOA5	-	20:46060984-46089952	-	-	IGV_LINK	786.443	16.5067	4.56444	28.449	OK	744.544	17.5834	4.08041	31.0863	OK	OK	0.091157
ENSG0000012	-	-	ENSG0000012416	'VAPB	-	20:58389121-58451101	-	-	IGV_LINK	850.445	14.4823	2.87826	26.0863	OK	1073.51	19.6351	3.43267	35.8376	OK	OK	0.43915
ENSG0000012	-	-	ENSG0000012417	'PARD6B	-	20:50731543-50756795	-	-	IGV_LINK	204.611	2.59561	0.669554	4.52167	OK	205.214	2.60415	0.671218	4.53708	OK	OK	0.004737
ENSG0000012	-	-	ENSG0000012417	'ATP5E	-	20:59019253-59032382	-	-	IGV_LINK	2789.89	59.2868	13.4919	105.082	OK	2610.03	58.4228	11.9139	104.932	OK	OK	-0.02118
ENSG0000012	-	-	ENSG0000012417	'CHD6	-	20:41402100-41618494	-	-	IGV_LINK	1114.21	8.13268	0.456792	15.8086	OK	766.2	5.92435	0.45328	11.3954	OK	OK	-0.45708
ENSG0000012	-	-	ENSG0000012418	'PLCG1	-	20:41028817-41317672	-	-	IGV_LINK	1886.11	57.8376	0	147.11	OK	1628.3	48.9703	0	133.011	OK	OK	-0.2401
ENSG0000012	-	-	ENSG0000012419	'TOX2	-	20:43914863-44069616	-	-	IGV_LINK	197.823	6.53679	0.747434	12.3261	OK	92.8102	3.9942	0.360203	7.62821	OK	OK	-0.71067
ENSG0000012	-	-	ENSG0000012419	'SRSF6	-	20:43457927-43464247	-	-	IGV_LINK	3419.23	94.652	35.5066	153.797	OK	2989.52	81.8003	31.0336	132.567	OK	OK	-0.21053
ENSG0000012	-	-	ENSG0000012419	'GDAP1L1	-	20:44247098-44280917	-	-	IGV_LINK	0	0	0	0	OK	1.03122	0.024091	0	0.36898	OK	NOTEST	inf
ENSG0000012	-	-	ENSG0000012419	'GTSF1L	-	20:43726163-43726998	-	-	IGV_LINK	2.90916	0.247486	0	0.824377	OK	0	0	0	0	OK	NOTEST	'-inf
ENSG0000012	-	-	ENSG0000012419	'ARFGEF2	-	20:48921889-49036693	-	-	IGV_LINK	1062.81	6.90079	2.7451	11.0565	OK	974.508	6.32826	2.69833	9.95819	OK	OK	-0.12495
ENSG0000012	-	-	ENSG0000012420	'ZNFX1	-	20:49219294-49484297	-	-	IGV_LINK	1666.95	13.3351	0	29.226	OK	1920.14	15.2591	0.984535	29.5337	OK	OK	0.194439
ENSG0000012	-	-	ENSG0000012420	'ZNF831	-	20:59123380-59259113	-	-	IGV_LINK	192.005	1.07218	0.224815	1.91954	OK	115.497	0.728993	0	1.64241	OK	OK	-0.55657
ENSG0000012	-	-	ENSG0000012420	'EDN3	-	20:59300426-59325992	-	-	IGV_LINK	0.969721	0.025465	0	0.17448	OK	31.968	0.730074	0	1.51033	OK	OK	4.84145
ENSG0000012	-	-	ENSG0000012420	'CSE1L	-	20:49046245-49096960	-	-	IGV_LINK	819.414	14.2958	5.78238	22.8092	OK	855.917	15.1119	6.03017	24.1935	OK	OK	0.080089
ENSG0000012	-	-	ENSG0000012420	'TMEM189-UBE2V1	-	20:50081123-50153734	-	-	IGV_LINK	15.1777	0.330954	0	2.32421	OK	84.1028	1.83471	0	6.86222	OK	OK	2.47085
ENSG0000012	-	-	ENSG0000012420	'RAB22A	-	20:58309695-58367507	-	-	IGV_LINK	564.377	4.18451	0.763928	7.6051	OK	736.295	5.26064	1.38075	9.14052	OK	OK	0.330178
ENSG0000012	-	-	ENSG0000012421	'PTGIS	-	20:49503873-49568146	-	-	IGV_LINK	6.78804	0.139099	0	0.389183	OK	7.21857	0.075262	0	0.215994	OK	NOTEST	-0.88611
ENSG0000012	-	-	ENSG0000012421	'STAU1	-	20:49113338-49188367	-	-	IGV_LINK	1980.17	37.4776	15.1922	59.7631	OK	3192.67	60.2925	22.5456	98.0394	OK	OK	0.685949
ENSG0000012	-	-	ENSG0000012421	'CDH26	-	20:59958426-60034011	-	-	IGV_LINK	30.0613	0.693846	0	1.60914	OK	25.7806	0.888151	0	2.06608	OK	OK	0.35619
ENSG0000012	-	-	ENSG0000012421	'SNAI1	-	20:49982998-49988886	-	-	IGV_LINK	39.7585	1.48044	0	2.99754	OK	68.0608	2.53623	0.001254	5.0712	OK	OK	0.776657
ENSG0000012	-	-	ENSG0000012421	'MOCS3	-	20:50958825-50963931	-	-	IGV_LINK	109.578	1.25172	0.138432	2.36501	OK	138.184	1.57885	0.305308	2.8524	OK	OK	0.334965
ENSG0000012	-	-	ENSG0000012422	'STX16	-	20:58651252-58719238	-	-	IGV_LINK	3373.7	53.6161	0	114.231	OK	4624.49	75.3406	0	163.529	OK	OK	0.490762
ENSG0000012	-	-	ENSG0000012422	'PPP4R1L	-	20:58228939-58309439	-	-	IGV_LINK	364.615	7.66163	0.255084	15.0682	OK	337.211	7.67104	0.506933	14.8352	OK	OK	0.001771
ENSG0000012	-	-	ENSG0000012422	'PMEPA1	-	20:57648391-57712780	-	-	IGV_LINK	928.023	15.4754	2.17269	28.7782	OK	803.324	15.4965	1.53353	29.4594	OK	OK	0.001961
ENSG0000012	-	-	ENSG0000012422	'RNF114	-	20:49936335-49953892	-	-	IGV_LINK	1710.59	47.1033	15.6573	78.5493	OK	1919.11	52.1859	18.4072	85.9647	OK	OK	0.147832
ENSG0000012	-	-	ENSG0000012422	'ANKRD60	-	20:58218494-58228653	-	-	IGV_LINK	0	0	0	0	OK	0	0	0	0	OK	NOTEST	0
ENSG0000012	-	-	ENSG0000012422	'DDX27	-	20:49219294-49484297	-	-	IGV_LINK	1084.15	23.6606	0	59.6789	OK	1106.5	23.8058	0	57.704	OK	OK	0.008826
ENSG0000012	-	-	ENSG0000012423	'RBPJL	-	20:45293444-45317824	-	-	IGV_LINK	0	0	0	0	OK	0	0	0	0	OK	NOTEST	0
ENSG0000012	-	-	ENSG0000012423	'SEMG1	-	20:45206996-45209772	-	-	IGV_LINK	0	0	0	0	OK	0	0	0	0	OK	NOTEST	0
ENSG0000012	-	-	ENSG0000012423	'C20orf85	-	20:58150903-58161150	-	-	IGV_LINK	0	0	0	0	OK	0	0	0	0	OK	NOTEST	0
ENSG0000012	-	-	ENSG0000012424	'BCAS4	-	20:50794893-50882676	-	-	IGV_LINK	172.61	5.55514	0	13.9086	OK	137.153	3.94715	0	9.94207	OK	OK	-0.49301
ENSG0000012	-	-	ENSG0000012424	'KCNK15	-	20:44656450-44810338	-	-	IGV_LINK	4.8486	0.11261	0	0.353973	OK	2.06245	0.047924	0	0.208909	OK	NOTEST	-1.23253
ENSG0000012	-	-	ENSG0000012425	'TP53TG5	-	20:45361936-45410610	-	-	IGV_LINK	16.4852	0.658866	0	5.96322	OK	23.7182	0.607477	0	3.42343	OK	OK	-0.11716
ENSG0000012	-	-	ENSG0000012425	'PCK1	-	20:57561079-57568112	-	-	IGV_LINK	320.977	6.25769	0	13.1209	OK	1000.29	17.3431	2.41656	32.2696	OK	OK	1.47066
ENSG0000012	-	-	ENSG0000012425	'ZBP1	-	20:57603845-57620576	-	-	IGV_LINK	155.155	6.57795	0	14.3014	OK	225.838	9.39484	0	19.4087	OK	OK	0.51423
ENSG0000012	-	-	ENSG0000012425	'NEURL2	-	20:45888624-45912155	-	-	IGV_LINK	15.5155	1.8759	0	20.3147	OK	16.4996	1.90791	0	42.8137	OK	OK	0.02441
ENSG0000012	-	-	ENSG0000012426	'MAGEA10	-	X:152114598-152138578	-	-	IGV_LINK	0.969721	0.022251	0	0.361766	OK	0	0	0	0	OK	NOTEST	'-inf
ENSG0000012	-	-	ENSG0000012427	'MTRR	-	5:7830377-7906025	-	-	IGV_LINK	1027.9	37.0937	0.260752	73.9266	OK	1236.44	43.5646	0.673568	86.4557	OK	OK	0.231984
ENSG0000012	-	-	ENSG0000012427	'FASTKD3	-	5:7830377-7906025	-	-	IGV_LINK	168.731	11.5274	0	34.7873	OK	182.527	11.7913	0	36.4051	OK	OK	0.032656
ENSG0000012	-	-	ENSG0000012429	'PEPD	-	19:33386949-33521794	-	-	IGV_LINK	891.173	29.197	12.0894	46.3046	OK	1728.33	56.1771	24.7369	87.6172	OK	OK	0.94416
ENSG0000012	-	-	ENSG0000012430	'CHST8	-	19:33621954-33773509	-	-	IGV_LINK	3.87888	0.111496	0	0.433681	OK	1.03122	0.029654	0	0.261031	OK	NOTEST	-1.91069
ENSG0000012	-	-	ENSG0000012431	'IQSEC2	-	X:53225827-53321328	-	-	IGV_LINK	608.015	16.195	0.566221	31.8237	OK	706.389	21.0774	1.94706	40.2078	OK	OK	0.380152
ENSG0000012	-	-	ENSG0000012433	'VAMP7	-	X:155881292-155943769	-	-	IGV_LINK	487.769	16.3394	2.97783	29.7009	OK	510.456	14.5866	3.22931	25.9439	OK	OK	-0.16371
ENSG0000012	-	-	ENSG0000012433	'IL9R	-	X:155997580-156027877	-	-	IGV_LINK	91.1537	5.57777	0	27.3825	OK	24.7494	0.750548	0	3.4646	OK	OK	-2.89367
ENSG0000012	-	-	ENSG0000012434	'XG	-	X:2752049-2815927	-	-	IGV_LINK	0.969721	0.285571	0	1.21879	OK	2.06245	0.05428	0	0.608248	OK	NOTEST	-2.39537
ENSG0000012	-	-	ENSG0000012435	'STAMBP	-	2:73828915-73873659	-	-	IGV_LINK	934.811	25.929	6.5314	45.3267	OK	1078.66	29.2237	6.67358	51.7738	OK	OK	0.172568
ENSG0000012	-	-	ENSG0000012435	'NAGK	-	2:70935881-71079805	-	-	IGV_LINK	2481.51	103.246	0	215.858	OK	2337.79	105.034	0	223.945	OK	OK	0.024769
ENSG0000012	-	-	ENSG0000012437	'MCEE	-	2:71109683-71130239	-	-	IGV_LINK	166.792	15.0626	2.20083	27.9244	OK	177.371	15.3052	3.15272	27.4577	OK	OK	0.02305
ENSG0000012	-	-	ENSG0000012437	'PAIP2B	-	2:71182738-71227083	-	-	IGV_LINK	258.915	2.38947	0.737238	4.04171	OK	142.309	1.31359	0.262117	2.36506	OK	OK	-0.86318
ENSG0000012	-	-	ENSG0000012438	'SNRNP27	-	2:69893559-69942945	-	-	IGV_LINK	433.465	36.1014	3.58155	68.6213	OK	532.112	44.2517	0	89.0185	OK	OK	0.293677
ENSG0000012	-	-	ENSG0000012438	'MPHOSPH10	-	2:71130313-71150101	-	-	IGV_LINK	402.434	11.6718	2.00132	21.3422	OK	411.459	11.0104	2.7788	19.2419	OK	OK	-0.08416
ENSG0000012	-	-	ENSG0000012439	'IL17C	-	16:88638590-88640473	-	-	IGV_LINK	2.90916	0.191645	0	0.537681	OK	3.09367	0.200323	0	0.545149	OK	NOTEST	0.06389
ENSG0000012	-	-	ENSG0000012439	'NDUFB4P12	-	4:43898961-43899351	-	-	IGV_LINK	0	0	0	0	OK	0	0	0	0	OK	NOTEST	0
ENSG0000012	-	-	ENSG0000012440	'ATP8A1	-	4:42408372-42657105	-	-	IGV_LINK	584.741	5.79416	0	14.4527	OK	246.463	3.00027	0	9.34012	OK	OK	-0.94951
ENSG0000012	-	-	ENSG0000012442	'USP22	-	17:20814619-21043760	-	-	IGV_LINK	2236.18	32.1666	0	68.9927	OK	2546.09	32.0866	4.47117	59.7021	OK	OK	-0.00359
ENSG0000012	-	-	ENSG0000012442	'POF1B	-	X:85277395-85379743	-	-	IGV_LINK	1.93944	0.060739	0	0.189825	OK	2.06245	0.032514	0	0.118866	OK	NOTEST	-0.90154
ENSG0000012	-	-	ENSG0000012444	'HIF3A	-	19:46297045-46343433	-	-	IGV_LINK	107.639	1.8179	0	5.38401	OK	250.588	6.61527	0	17.0031	OK	OK	1.86353
ENSG0000012	-	-	ENSG0000012444	'ZNF576	-	19:43596391-43619874	-	-	IGV_LINK	182.307	8.17246	0	18.5192	OK	227.488	11.4157	0	23.6586	OK	OK	0.482177
ENSG0000012	-	-	ENSG0000012444	'IRGC	-	19:43716009-43720021	-	-	IGV_LINK	0	0	0	0	OK	1.03122	0.077247	0	0.302972	OK	NOTEST	inf
ENSG0000012	-	-	ENSG0000012445	'ZNF45	-	19:43902000-43935278	-	-	IGV_LINK	286.068	6.39826	0	12.8573	OK	337.211	8.34266	0.07154	16.6138	OK	OK	0.382827
ENSG0000012	-	-	ENSG0000012446	'LYPD3	-	19:43460786-43465660	-	-	IGV_LINK	41.698	1.63219	0	3.40954	OK	18.562	0.828266	0	1.9126	OK	OK	-0.97864
ENSG0000012	-	-	ENSG0000012446	'PSG8	-	19:42752685-42855718	-	-	IGV_LINK	0	0	0	0	OK	0	0	0	0	OK	NOTEST	0
ENSG0000012	-	-	ENSG0000012446	'CEACAM8	-	19:42397127-42652355	-	-	IGV_LINK	0	0	0	0	OK	0	0	0	0	OK	NOTEST	0
ENSG0000012	-	-	ENSG0000012447	'NDP	-	X:43948775-43973504	-	-	IGV_LINK	0	0	0	0	OK	0	0	0	0	OK	NOTEST	0
ENSG0000012	-	-	ENSG0000012448	'USP9X	-	X:41085634-41236579	-	-	IGV_LINK	1977.26	13.4713	1.27416	25.6684	OK	2408.94	16.5567	1.21316	31.9003	OK	OK	0.297531
ENSG0000012	-	-	ENSG0000012449	'CRISP2	-	6:49692357-49713590	-	-	IGV_LINK	0	0	0	0	OK	0	0	0	0	OK	NOTEST	0
ENSG0000012	-	-	ENSG0000012449	'F13A1	-	6:6144084-6321013	-	-	IGV_LINK	1476.88	21.7512	9.62148	33.881	OK	3556.69	52.5848	21.257	83.9127	OK	OK	1.27355
ENSG0000012	-	-	ENSG0000012449	'GRM4	-	6:34018644-34155622	-	-	IGV_LINK	2.90916	0.039395	0	0.373794	OK	1.03122	0.02421	0	0.357059	OK	NOTEST	-0.7024
ENSG0000012	-	-	ENSG0000012449	'TRERF1	-	6:42224930-42452051	-	-	IGV_LINK	478.072	4.40258	1.55156	7.25359	OK	205.214	1.76256	0.444353	3.08078	OK	OK	-1.32067
ENSG0000012	-	-	ENSG0000012450	'PACSIN1	-	6:34466060-34535231	-	-	IGV_LINK	31.0311	0.458626	0	1.13436	OK	25.7806	0.411353	0	1.09542	OK	NOTEST	-0.15694
ENSG0000012	-	-	ENSG0000012450	'BTN2A2	-	6:26383095-26394874	-	-	IGV_LINK	1636.89	45.1181	10.9495	79.2866	OK	740.42	20.804	4.46301	37.1449	OK	OK	-1.11685
ENSG0000012	-	-	ENSG0000012452	'SIRT5	-	6:13574528-13615158	-	-	IGV_LINK	437.344	8.04046	2.78688	13.294	OK	269.15	5.16111	0.963764	9.35845	OK	OK	-0.6396
ENSG0000012	-	-	ENSG0000012453	'MRS2	-	6:24402907-24537207	-	-	IGV_LINK	523.649	13.7549	0	30.2489	OK	327.93	9.20909	0	18.8941	OK	OK	-0.57881
ENSG0000012	-	-	ENSG0000012453	'WRNIP1	-	6:2765413-2786952	-	-	IGV_LINK	629.349	21.1163	7.86373	34.3688	OK	345.46	11.2579	3.54573	18.9702	OK	OK	-0.90741

ENSG0000012	-	-	ENSG0000012454	RRP36	-	6:43021644-43034405	-	-	IGV_LINK	288.007	17.6164	5.09661	30.1362	OK	299.055	17.7024	4.94599	30.4589	OK	OK	0.007029
ENSG0000012	-	-	ENSG0000012454	BTN2A3P	-	6:26421390-26432383	-	-	IGV_LINK	208.49	5.59962	0.706888	10.4923	OK	114.466	3.28542	0	6.5855	OK	OK	-0.76925
ENSG0000012	-	-	ENSG0000012455	BTN1A1	-	6:26501220-26510422	-	-	IGV_LINK	1.93944	0.040147	0	0.138372	OK	4.1249	0.085505	0	0.227271	OK	NOTEST	1.09073
ENSG0000012	-	-	ENSG0000012456	SNRPC	-	6:34757405-34773794	-	-	IGV_LINK	875.658	74.26	31.5305	116.99	OK	671.327	57.8443	23.4029	92.2857	OK	OK	-0.36041
ENSG0000012	-	-	ENSG0000012456	SLC17A3	-	6:25833065-25882395	-	-	IGV_LINK	6533.01	319.258	98.5895	539.927	OK	521.8	25.4341	7.78203	43.0862	OK	OK	-3.64989
ENSG0000012	-	-	ENSG0000012456	SLC17A1	-	6:25782896-25832059	-	-	IGV_LINK	1174.33	43.5377	18.87	68.2054	OK	85.5917	3.18282	0.246947	6.1187	OK	OK	-3.77389
ENSG0000012	-	-	ENSG0000012457	SERPINB6	-	6:2948158-2972165	-	-	IGV_LINK	2582.37	109.381	44.2298	174.533	OK	1553.02	65.4043	27.6921	103.116	OK	OK	-0.74191
ENSG0000012	-	-	ENSG0000012457	XPO5	-	6:43506968-43576075	-	-	IGV_LINK	697.229	14.1479	0	66.7746	OK	539.331	8.85839	0	26.3953	OK	OK	-0.67547
ENSG0000012	-	-	ENSG0000012457	ABCC10	-	6:43427365-43456632	-	-	IGV_LINK	455.769	10.6826	0	24.0321	OK	435.177	9.68136	0	24.2374	OK	OK	-0.14198
ENSG0000012	-	-	ENSG0000012457	HIST1H1D	-	6:26234267-26234933	-	-	IGV_LINK	8.72748	0.996694	0	2.26839	OK	16.4996	1.88869	0	4.21072	OK	OK	0.92216
ENSG0000012	-	-	ENSG0000012458	PEX6	-	6:42960757-42979220	-	-	IGV_LINK	563.408	9.68728	3.76724	15.6073	OK	321.742	5.53269	1.85986	9.20552	OK	OK	-0.80811
ENSG0000012	-	-	ENSG0000012458	NQO2	-	6:2987966-3028869	-	-	IGV_LINK	612.863	25.043	2.2869	47.7991	OK	532.112	21.8256	2.48395	41.1672	OK	OK	-0.19839
ENSG0000012	-	-	ENSG0000012459	AL365205.1	-	6:41770175-41895361	-	-	IGV_LINK	481.225	9.26489	0	21.6801	OK	390.62	7.50385	0	15.5111	OK	OK	-0.30414
ENSG0000012	-	-	ENSG0000012459	OARD1	-	6:41026910-41099976	-	-	IGV_LINK	663.183	36.6925	0.129809	73.2551	OK	392.252	20.3088	0	42.439	OK	OK	-0.85338
ENSG0000012	-	-	ENSG0000012460	UNC5CL	-	6:41026910-41099976	-	-	IGV_LINK	580.968	12.004	0	25.8118	OK	386.323	8.18067	0	17.753	OK	OK	-0.55323
ENSG0000012	-	-	ENSG0000012460	AARS2	-	6:44270465-44378957	-	-	IGV_LINK	553.71	8.56365	3.19252	13.9348	OK	479.52	7.52756	2.38101	12.6741	OK	OK	-0.18604
ENSG0000012	-	-	ENSG0000012461	HIST1H1A	-	6:26017084-26017732	-	-	IGV_LINK	0	0	0	0	OK	0	0	0	0	OK	NOTEST	0
ENSG0000012	-	-	ENSG0000012461	ZNF391	-	6:27374614-27403904	-	-	IGV_LINK	102.79	2.25745	0	5.32346	OK	27.8431	0.401793	0	1.01386	OK	OK	-2.49017
ENSG0000012	-	-	ENSG0000012461	RPS10	-	6:34246379-34426125	-	-	IGV_LINK	12145.1	1451.63	272.617	2630.65	OK	5959.11	713.981	198.884	1229.08	OK	OK	-1.02372
ENSG0000012	-	-	ENSG0000012461	MOCS1	-	6:39792297-39934551	-	-	IGV_LINK	903.78	16.2143	1.6322	30.7965	OK	1342.65	22.64	0	45.8495	OK	OK	0.481603
ENSG0000012	-	-	ENSG0000012463	HIST1H2BJ	-	6:27125896-27132750	-	-	IGV_LINK	6.78804	1.33337	0	3.25672	OK	22.6869	3.42692	0	7.87942	OK	OK	1.36184
ENSG0000012	-	-	ENSG0000012464	MED20	-	6:41905353-41921139	-	-	IGV_LINK	190.065	5.48017	0.860441	10.0999	OK	164.996	4.99432	0.727477	9.26115	OK	OK	-0.13393
ENSG0000012	-	-	ENSG0000012465	OR2B6	-	6:27957240-27958182	-	-	IGV_LINK	0	0	0	0	OK	4.1249	0.302556	0	0.766453	OK	NOTEST	inf
ENSG0000012	-	-	ENSG0000012465	TBCC	-	6:42744480-42746096	-	-	IGV_LINK	334.554	13.0615	4.47518	21.6478	OK	251.619	9.83142	2.99024	16.6726	OK	OK	-0.40985
ENSG0000012	-	-	ENSG0000012466	SPDEF	-	6:34537801-34556333	-	-	IGV_LINK	3.87888	0.129062	0	0.339629	OK	2.06245	0.067524	0	0.213952	OK	NOTEST	-0.9346
ENSG0000012	-	-	ENSG0000012467	TCP11	-	6:35118070-35148610	-	-	IGV_LINK	5.81832	0.235319	0	1.11373	OK	3.09367	0.114976	0	0.929354	OK	NOTEST	-1.03329
ENSG0000012	-	-	ENSG0000012468	MAD2L1BP	-	6:43629539-43640952	-	-	IGV_LINK	315.159	15.7302	5.14022	26.3202	OK	288.743	14.5309	4.49472	24.567	OK	OK	-0.11442
ENSG0000012	-	-	ENSG0000012470	APOBEC2	-	6:41026910-41099976	-	-	IGV_LINK	8.72748	0.502662	0	4.52114	OK	1.03122	0.059464	0	1.14745	OK	NOTEST	-3.07951
ENSG0000012	-	-	ENSG0000012470	KLHDC3	-	6:43014102-43021298	-	-	IGV_LINK	1150.09	37.503	16.1069	58.8992	OK	719.795	23.377	9.64111	37.1129	OK	OK	-0.68192
ENSG0000012	-	-	ENSG0000012471	GNMT	-	6:42960757-42979220	-	-	IGV_LINK	14.5458	0.91135	0	4.37265	OK	3.09367	0.194079	0	1.51	OK	OK	-2.23136
ENSG0000012	-	-	ENSG0000012472	DNAH8	-	6:38715340-39030529	-	-	IGV_LINK	7.75776	0.031931	0	0.337758	OK	4.1249	0.016979	0	0.321319	OK	NOTEST	-0.91121
ENSG0000012	-	-	ENSG0000012473	TREM1	-	6:41267925-41286719	-	-	IGV_LINK	32.9705	2.7358	0	6.22514	OK	131.997	6.37857	0	12.9366	OK	OK	1.22127
ENSG0000012	-	-	ENSG0000012473	MEA1	-	6:42984498-43013968	-	-	IGV_LINK	876.627	53.9052	9.48709	98.3234	OK	578.517	35.6187	8.36485	62.8725	OK	OK	-0.59779
ENSG0000012	-	-	ENSG0000012474	KLHL31	-	6:53647900-53665708	-	-	IGV_LINK	36.8494	0.411397	0	0.874398	OK	19.5933	0.198262	0	0.426605	OK	NOTEST	-1.05312
ENSG0000012	-	-	ENSG0000012474	COL21A1	-	6:56056589-56394094	-	-	IGV_LINK	17.455	0.256715	0	0.974071	OK	3.09367	0.287737	0	1.0736	OK	NOTEST	0.164586
ENSG0000012	-	-	ENSG0000012476	CDKN1A	-	6:36676459-36687339	-	-	IGV_LINK	957.114	27.1899	11.2404	43.1395	OK	1654.08	49.6138	18.6683	80.5592	OK	OK	0.867669
ENSG0000012	-	-	ENSG0000012476	SOX4	-	6:21528738-21598619	-	-	IGV_LINK	428.616	4.253	1.54848	6.95753	OK	317.617	3.15224	1.04098	5.2635	OK	OK	-0.43211
ENSG0000012	-	-	ENSG0000012476	GLO1	-	6:38675924-38703141	-	-	IGV_LINK	1080.27	33.1005	14.4233	51.7778	OK	763.106	23.3937	9.78224	37.0051	OK	OK	-0.50074
ENSG0000012	-	-	ENSG0000012477	CPNE5	-	6:36740774-36840002	-	-	IGV_LINK	113.457	3.14702	0.02948	6.26457	OK	149.528	4.7982	0.103518	9.49289	OK	OK	0.608506
ENSG0000012	-	-	ENSG0000012478	KCNK17	-	6:39299000-39314553	-	-	IGV_LINK	3.87888	0.147144	0	0.414327	OK	22.6869	0.893599	0	1.90376	OK	OK	2.6024
ENSG0000012	-	-	ENSG0000012478	RREB1	-	6:7107596-7251980	-	-	IGV_LINK	804.868	6.76062	1.58673	11.9345	OK	959.039	7.70814	2.4145	13.0018	OK	OK	0.189227
ENSG0000012	-	-	ENSG0000012478	SSR1	-	6:7268305-7418037	-	-	IGV_LINK	1792.04	46.3121	4.50914	88.115	OK	1494.24	43.5317	8.70661	78.3567	OK	OK	-0.08932
ENSG0000012	-	-	ENSG0000012478	RIOK1	-	6:7268305-7418037	-	-	IGV_LINK	277.34	6.92496	0	22.0248	OK	209.339	6.79216	0	24.0223	OK	OK	-0.02794
ENSG0000012	-	-	ENSG0000012478	NRN1	-	6:5997998-6007605	-	-	IGV_LINK	77.5776	3.08473	0.146847	6.02261	OK	144.371	5.60987	0.934507	10.2852	OK	OK	0.862825
ENSG0000012	-	-	ENSG0000012478	SLC35B3	-	6:8413067-8435483	-	-	IGV_LINK	285.098	8.17868	2.45855	13.8988	OK	214.495	6.15687	1.51691	10.7968	OK	OK	-0.40967
ENSG0000012	-	-	ENSG0000012478	RPP40	-	6:4994731-5004063	-	-	IGV_LINK	31.0311	2.65974	0	5.71851	OK	27.8431	1.83846	0	4.1514	OK	OK	-0.53278
ENSG0000012	-	-	ENSG0000012478	ATXN1	-	6:16299111-16762652	-	-	IGV_LINK	1238.33	12.0437	0	25.5521	OK	807.449	7.47391	0	16.0607	OK	OK	-0.68835
ENSG0000012	-	-	ENSG0000012478	NUP153	-	6:17615034-17707344	-	-	IGV_LINK	1208.27	12.7893	5.35329	20.2253	OK	723.92	7.6782	3.05108	12.3053	OK	OK	-0.7361
ENSG0000012	-	-	ENSG0000012479	DEK	-	6:18223867-18264823	-	-	IGV_LINK	1501.13	48.0515	12.6786	83.4243	OK	1025.04	33.3106	9.23367	57.3876	OK	OK	-0.5286
ENSG0000012	-	-	ENSG0000012480	EEF1E1	-	6:7881516-8102578	-	-	IGV_LINK	326.796	30.6861	0	103.093	OK	178.402	15.8912	0	63.4466	OK	OK	-0.94936
ENSG0000012	-	-	ENSG0000012481	CRISP1	-	6:49834256-49877096	-	-	IGV_LINK	0	0	0	0	OK	0	0	0	0	OK	NOTEST	0
ENSG0000012	-	-	ENSG0000012481	RUNX2	-	6:44809316-45664349	-	-	IGV_LINK	323.887	4.14323	0.544941	7.74152	OK	295.962	3.53503	0.754635	6.31543	OK	OK	-0.22903
ENSG0000012	-	-	ENSG0000012481	OPN5	-	6:47781981-47832780	-	-	IGV_LINK	0	0	0	0	OK	0	0	0	0	OK	NOTEST	0
ENSG0000012	-	-	ENSG0000012482	GCM2	-	6:10747758-10979320	-	-	IGV_LINK	0	0	0	0	OK	0	0	0	0	OK	NOTEST	0
ENSG0000012	-	-	ENSG0000012483	LRRFIP1	-	2:237627575-237842808	-	-	IGV_LINK	1963.68	37.5104	12.0596	62.9611	OK	1999.55	39.9435	12.0588	67.8281	OK	OK	0.090669
ENSG0000012	-	-	ENSG0000012483	AC105760.1	-	2:237048598-237056167	-	-	IGV_LINK	0	0	0	0	OK	0	0	0	0	OK	NOTEST	0
ENSG0000012	-	-	ENSG0000012483	RAB17	-	2:237574321-237601614	-	-	IGV_LINK	1254.82	43.6925	13.6019	73.7831	OK	1797.43	62.1031	20.9525	103.254	OK	OK	0.50728
ENSG0000012	-	-	ENSG0000012487	CXCL6	-	4:73836496-73849064	-	-	IGV_LINK	180.368	9.3283	0.539482	18.1171	OK	2398.63	98.8875	35.7393	162.036	OK	OK	3.4061
ENSG0000012	-	-	ENSG0000012488	EREG	-	4:74365142-74388751	-	-	IGV_LINK	0	0	0	0	OK	25.7806	0.371955	0	0.865633	OK	NOTEST	inf
ENSG0000012	-	-	ENSG0000012490	TRIM51	-	11:55883296-55891810	-	-	IGV_LINK	0	0	0	0	OK	0	0	0	0	OK	NOTEST	0
ENSG0000012	-	-	ENSG0000012491	DKFZP434K028	-	11:61680432-61867354	-	-	IGV_LINK	3.87888	0.095312	0	1.6289	OK	11.3435	0.278872	0	3.42672	OK	NOTEST	1.54887
ENSG0000012	-	-	ENSG0000012492	MYRF	-	11:61680432-61867354	-	-	IGV_LINK	166.792	2.00473	0	7.69416	OK	210.37	2.50654	0	9.70831	OK	OK	0.322288
ENSG0000012	-	-	ENSG0000012493	SCGB1D2	-	11:62208667-62260549	-	-	IGV_LINK	34.9099	6.39692	0	13.1941	OK	6.18735	1.13808	0	2.78327	OK	OK	-2.49077
ENSG0000012	-	-	ENSG0000012493	SCGB2A1	-	11:62208667-62260549	-	-	IGV_LINK	1.93944	0.313625	0	1.75999	OK	1.03122	0.167314	0	0.816304	OK	NOTEST	-0.90648
ENSG0000012	-	-	ENSG0000012494	AHNAK	-	11:62433541-62556235	-	-	IGV_LINK	27530.4	89.0987	0	211.961	OK	20620.4	72.4642	0	188.022	OK	OK	-0.29814
ENSG0000012	-	-	ENSG0000012503	EMC3	-	3:9958716-10011209	-	-	IGV_LINK	733.109	45.7655	7.15523	84.3759	OK	901.291	59.3931	7.51888	111.267	OK	OK	0.376033
ENSG0000012	-	-	ENSG0000012504	SSUH2	-	3:8619399-8963773	-	-	IGV_LINK	1.93944	0.146915	0	1.66535	OK	3.09367	0.158419	0	1.49485	OK	NOTEST	0.108767
ENSG0000012	-	-	ENSG0000012508	WNT1	-	12:48978452-48981676	-	-	IGV_LINK	0	0	0	0	OK	1.03122	0.057457	0	0.24592	OK	NOTEST	inf
ENSG0000012	-	-	ENSG0000012508	SH3TC1	-	4:8182071-8241803	-	-	IGV_LINK	975.539	21.2264	2.16919	40.2837	OK	1211.69	29.5393	2.52033	56.5583	OK	OK	0.476774
ENSG0000012	-	-	ENSG0000012510	CNOT1	-	16:58515478-58629886	-	-	IGV_LINK	4008.82	57.5971	0	138.624	OK	3940.31	69.8471	0	160.318	OK	OK	0.278205
ENSG0000012	-	-	ENSG0000012512	LRRC29	-	16:67207138-67227048	-	-	IGV_LINK	91.1537	5.02863	0	10.1376	OK	162.934	9.16322	1.10717	17.2193	OK	OK	0.865689
ENSG0000012	-	-	ENSG0000012512	BBS2	-	16:56429132-56520283	-	-	IGV_LINK	949.356	29.7516	0	73.5344	OK	1166.32	33.2928	0	75.0679	OK	OK	0.162247
ENSG0000012	-	-	ENSG0000012514	MT1G	-	16:56666730-56668065	-	-	IGV_LINK	187.156	45.0184	11.0112	79.0256	OK	2672.94	647.223	270.674	1023.77	OK	OK	3.84568
ENSG0000012	-	-	ENSG0000012514	MT2A	-	16:56608198-56609497	-	-	IGV_LINK	869.839	80.5613	34.1396	126.983	OK	4290.93	396.663	164.223	629.104	OK	OK	2.29976
ENSG0000012	-	-	ENSG0000012514	C16orf70	-	16:67109957-67151214	-	-	IGV_LINK	482.921	18.0506	0	39.0825	OK	507.363	16.488	0	34.0787	OK	OK	-0.13063
ENSG0000012	-	-	ENSG0000012516	GOT2	-	16:58707130-59108974	-	-	IGV_LINK	787.413	19.8782	7.98308	31.7733	OK	894.072	23.1563	9.12126	37.1913	OK	OK	0.220218
ENSG0000012	-	-	ENSG0000012517	DOK4	-	16:57428168-57487327	-	-	IGV_LINK	1220.88	30.9873	0	77.4156	OK	2491.44	76.6294	0	183.91	OK	OK	1.30622
ENSG0000012	-	-	ENSG0000012520	PIWIL1	-	12:130337886-130372637	-	-	IGV_LINK	1.93944	0.029346	0	0.563507	OK	2.06245	0.154955	0	0.794946	OK	NOTEST	2.40062
ENSG0000012	-	-	ENSG0000012524	GPR18	-	13:99200773-99386434	-	-	IGV_LINK	55.2741	2.36401	0	9.3089	OK	23.7182	1.0327	0	6.17948	OK	OK	-1.19482
ENSG0000012	-	-	ENSG0000012524	CLYBL	-	13:99606668-99897134	-	-	IGV_LINK	291.886	12.9193	3.13655	22.7021	OK	435.177	21.7098	7.15483	36.2647	OK	OK	0.748815
ENSG0000012	-	-	ENSG0000012524	TMTC4	-	13:100603926-100675093	-	-	IGV_LINK	175.519	4.61275	0.123431	9.10207	OK	263.994	6.28839	0	12.7269	OK	OK	0.447063
ENSG0000012	-	-	ENSG0000012524	RAP2A	-	13:97434221-97469128	-	-	IGV_LINK	496.497	7.09623	0.710687	13.4818	OK	457.864	6.57994	0.588157	12.5717	OK	OK	-0.10898
ENSG0000012	-	-	ENSG0000012525	SLC10A2	-	13:103043997-103066846	-	-	IGV_LINK	239.521	3.74279	1.10943	6.37616	OK	103.122	1.61192	0.186988	3.03686	OK	OK	-1.21533
ENSG0000012	-	-	ENSG0000012525	ABCC4	-	13:95019828-95301446	-	-	IGV_LINK	1245.12	13.1629	4.11164	22.2142	OK	731.139	7.5371	2.48777	12.5864	OK	OK	-0.8044
ENSG0000012	-	-	ENSG0000012526	EFNB2	-	13:106489730-106535114	-	-	IGV_LINK	877.597	11.5326	4.76133	18.3039	OK	896.135	11.7794	4.8754	18.6833	OK	OK	0.030544
ENSG0000012	-	-	ENSG0000012528	SOX21	-	13:94709621-94712399	-	-	IGV_LINK	0	0	0	0	OK	2.06245	0.04463	0	0.131186	OK	NOTEST	inf
ENSG0000012	-	-	ENSG0000012530	TM9SF2	-	13:99501416-99564006	-	-	IGV_LINK	2572.67	44.7144	18.9254	70.5034	OK	3465.95	60.4135	23.7985	97.0285	OK	OK	0.434132
ENSG0000012	-	-	ENSG0000012531	C17orf53	-	17:44141905-44162476	-	-	IGV_LINK	42.6677	0.952449	0	2.02798	OK	39.1865	1.29725	0	3.13799	OK	OK	0.445748
ENSG0000012	-	-	ENSG0000012533	KIF25	-	6:167992821-168045089	-	-	IGV_LINK	8.72748	0.404588	0	1.078	OK	3.09367	0.486595	0	1.40787	OK	NOTEST	0.266268
ENSG0000012	-	-	ENSG0000012534	IRF1	-	5:132410635-132664272	-	-	IGV_LINK	4003.98	119.31	0	250.952	OK	1605.62	47.4236	0	101.481	OK	OK	-1.33104
ENSG0000012	-	-	ENSG0000012535	UPF3B	-	X:119805310-119852998	-	-	IGV_LINK	270.552	7.45546	1.82091	13.09	OK	284.618	7.81917	2.21915	13.4192	OK	OK	0.068719
ENSG0000012	-	-	ENSG0000012535	RNF113A	-	X:119870474-119876662	-	-	IGV_LINK	234.672	11.2032	0	30.7318	OK	201.089	9.60928	0	26.2003	OK	OK	-0.22141
ENSG0000012	-	-	ENSG0000012535	SEPT6	-	X:119615723-119693370	-	-	IGV_LINK	1063.78	25.9263	7.06375	44.7889	OK	819.824	18.0083	5.12702	30.8895	OK	OK	-0.52576
ENSG0000012	-	-	ENSG0000012535	TMEM255A	-	X:120258649-120311556	-	-	IGV_LINK	464.496	10.6402	0.072232	21.2081	OK	377.428	9.18767	0	18.7292	OK	OK	-0.21175
ENSG0000012	-	-	ENSG0000012535	NDUFA1	-	X:119870474-119876662	-	-	IGV_LINK	1742.59	165.474	64.6191	266.329	OK	1616.96	153.843	63.009	244.676	OK	OK	-0.10515
ENSG0000012	-	-	ENSG0000012536	AMELX	-	X:11137542-11665701	-	-	IGV_LINK	0	0	0	0	OK	0	0	0	0	OK	NOTEST	0
ENSG0000012	-	-	ENSG0000012537	ATP5S	-	14:50237562-50416461	-	-	IGV_LINK	347.16	7.90123	0	21.4695	OK	277.4	7.32371	0	19.2661	OK	OK	-0.1095
ENSG0000012	-	-	ENSG0000012537	BMP4	-	14:53949735-53958761	-	-	IGV_LINK	46.5466	1.94863	0	4.33152	OK	57.7486	2.14648	0	4.45147	OK	OK	0.139513
ENSG0000012	-	-	ENSG0000012538	PTGER2	-	14:52314304-52328606	-	-	IGV_LINK	43.6374	1.11252	0	2.26659	OK	194.902	5.74097	0.6791	10.8028	OK	OK	2.36746
ENSG0000012	-	-	ENSG0000012538	AL161781.1	-	9:37046834-37047237	-	-	IGV_LINK	0	0	0	0	OK	0	0	0	0	OK	NOTEST	0
ENSG0000012	-	-	ENSG0000012538	FAM193A	-	4:2418973-2732565	-	-	IGV_LINK	751.532	9.53017	0.774679	18.2857	OK	694.733	8.7693	0	18.1667	OK	OK	-0.12004
ENSG0000012	-	-	ENSG0000012538	GRK4	-	4:2963607-3040747	-	-	IGV_LINK	51.3952	2.82479	0	7.22294	OK	41.249	1.94837	0	4.93994	OK	OK	-0.53588
ENSG0000012	-	-	ENSG0000012539	SOX9	-	17:72021850-72237203	-	-	IGV_LINK	420.859	6.30373	1.98573	10.6217	OK	493.957	7.40088	2.44221	12.3595	OK	OK	0.23149
ENSG0000012	-	-	ENSG0000012540	TEKT3	-	17:15303810-15341641	-	-	IGV_LINK	7.75776	0.300249	0	1.09452	OK	3.09367	0.434643	0	1.31474	OK	NOTEST	0.533669
ENSG0000012	-	-	ENSG0000012541	MYH2	-	17:10383131-10623886	-	-	IGV_LINK	0	0	0	0	OK	0	0	0	0	OK	NOTEST	0
ENSG0000012	-	-	ENSG0000012543	HS3ST3B1	-	17:14301082-14349404	-	-	IGV_LINK	47.5163	0.547696	0	1.18871	OK	40.2178	0.436479	0	0.901925	OK	NOTEST	-0.32746
ENSG0000012	-	-	ENSG0000012543	SLC25A35	-	17:8287762-8295343	-	-	IGV_LINK	112.488	3.55246	0	7.67191	OK	117.56	3.72922	0	8.53108	OK	OK	0.070057
ENSG0000012	-	-	ENSG0000012544	MRPS7	-	17:75236598-75271227	-	-	IGV_LINK	869.839	47.6173	0	98.9191	OK	995.132	56.5665	1.20991	111.923	OK	OK	0.248463
ENSG0000012	-	-	ENSG0000012544	GGA3	-	17:75236598-75271227	-	-	IGV_LINK	1136.51	25.6562	0	63.9692	OK	1339.56	27.2038	0	66.6459	OK	OK	0.0845
ENSG0000012	-	-	ENSG0000012544	ARMC7	-	17:75109951-75133043	-	-	IGV_LINK	173.58	5.68789	0	13.0325	OK	194.902	6.46264	0	14.697	OK	OK	0.184231
ENSG0000012	-	-	ENSG0000012545	NUP85	-	17:75205658-75235758	-	-	IGV_LINK	832.99	37.5345	3.46449	71.6046	OK	816.73	32.7535	5.65888	59.8481	OK	OK	-0.19657
ENSG0000012	-	-	ENSG0000012545	SLC25A19	-	17:75271368-75289510	-	-	IGV_LINK	215.278	11.4633	0.503961	22.4227	OK	271.212	12.2173	1.25748	23.1771	OK	OK	0.091896
ENSG0000012	-	-	ENSG0000012545	MIF4GD	-	17:75236598-75271227	-	-	IGV_LINK	445.102	24.4021	0	58.918	OK	332.054	17.5662	0	46.9072	OK	OK	-0.4742
ENSG0000012	-	-	ENSG0000012545	NT5C	-	17:75109951-75133043	-	-	IGV_LINK	459.648	37.526	7.35455	67.6974	OK	467.145	40.1521	6.56997	73.7343	OK	OK	0.097587
ENSG0000012	-	-	ENSG0000012545	MSTO1	-	1:155609775-155739010	-	-	IGV_LINK	371.403	24.0404	0	71.6196	OK	359.898	23.5551	0	69.8865	OK	OK	-0.02942
ENSG0000012	-	-	ENSG0000012546	C1orf61	-	1:156404249-156430701	-	-	IGV_LINK	4.8486	0.196764	0	1.35896	OK	4.1249	0.645664	0	2.08333	OK	OK	1.71432
ENSG0000012	-	-	ENSG0000012548	TTF1	-	9:132375547-132406851	-	-	IGV_LINK	306.432	7.1911	0.947663	13.4345	OK	352.679	7.78307	2.36648	13.1997	OK	OK	0.114126
ENSG0000012	-	-	ENSG0000012548	GTF3C4	-	9:132592996-132694955	-	-	IGV_LINK	587.651	3.80224	0.781172	6.82332	OK	494.988	3.14513	0.556008	5.73426	OK	OK	-0.27373
ENSG0000012	-	-	ENSG0000012548	DDX31	-	9:132592996-132694955	-	-	IGV_LINK	454.799	10.296	1.32728	19.2646	OK	428.99	9.72483	1.37346	18.0762	OK	OK	-0.08233
ENSG0000012	-	-	ENSG0000012549	BARHL1	-	9:132582184-132590266	-	-	IGV_LINK	0	0	0	0	OK	1.03122	0.026814	0	0.146471	OK	NOTEST	inf
ENSG0000012	-	-	ENSG0000012549	KIR2DL1	-	19:54724478-54798285	-	-	IGV_LINK	7.75776	0.307131	0	0.938304	OK	7.21857	0.286015	0	0.868476	OK	NOTEST	-0.10276
ENSG0000012	-	-	ENSG0000012550	PPP1R12C	-	19:55090912-55117559	-	-	IGV_LINK	2062.6	75.0763	19.6104	130.542	OK	2267.66	84.1011	22.8004	145.402	OK	OK	0.163768
ENSG0000012	-	-	ENSG0000012550	MBOAT7	-	19:54173411-54189882	-	-	IGV_LINK	872.748	24.0093	6.62652	41.3921	OK	1319.97	34.5996	11.4887	57.7105	OK	OK	0.52716
ENSG0000012	-	-	ENSG0000012550	SRMS	-	20:63540809-63547504	-	-	IGV_LINK	0	0	0	0	OK	3.09367	0.129883	0	0.3364	OK	NOTEST	inf
ENSG0000012	-	-	ENSG0000012551	OPRL1	-	20:64080172-64100643	-	-	IGV_LINK	91.1537	1.71412	0.146764	3.28148	OK	70.1233	1.32909	0	2.70429	OK	OK	-0.36703
ENSG0000012	-	-	ENSG0000012551	LINC00029	-	20:63009382-63085071	-	-	IGV_LINK	0	0	0	0	OK	0	0	0	0	OK	NOTEST	0
ENSG0000012	-	-	ENSG0000012552	SLC2A4RG	-	20:63657809-63743505	-	-	IGV_LINK	1087.06	56.4206	0	138.116	OK	791.981	42.1826	0	116.137	OK	OK	-0.41957
ENSG0000012	-	-	ENSG0000012552	NPBWR2	-	20:64105819-64107171	-	-	IGV_LINK	0	0	0	0	OK	0	0	0	0	OK	NOTEST	0
ENSG0000012	-	-	ENSG0000012553	FNDC11	-	20:63547890-63556708	-	-	IGV_LINK	5.81832	0.118317	0	0.392121	OK	4.1249	0.089366	0	0.365882	OK	NOTEST	-0.40487
ENSG0000012	-	-	ENSG0000012553	BHLHE23	-	20:63005926-63007035	-	-	IGV_LINK	0	0	0	0	OK	0	0	0	0	OK	NOTEST	0
ENSG0000012	-	-	ENSG0000012553	PPDPF	-	20:63520723-63522206	-	-	IGV_LINK	1959.81	164.939	68.5619	261.317	OK	1814.96	152.228	65.406	239.051	OK	OK	-0.1157
ENSG0000012	-	-	ENSG0000012553	IL1B	-	2:112829750-112836903	-	-	IGV_LINK	342.311	15.7986	4.23101	27.3662	OK	219.651	9.84934	2.04296	17.6557	OK	OK	-0.6817

ENSG0000012	-	-	ENSG0000012555	'PLGLB2	-	2:87748086-87825952	-	-	IGV_LINK	3.87888	0.11478	0	0.472703	OK	0	0	0	0	OK	NOTEST	'-inf
ENSG0000012	-	-	ENSG0000012557	'IL37	-	2:112912970-112918882	-	-	IGV_LINK	0	0	0	0	OK	0	0	0	0	OK	NOTEST	0
ENSG0000012	-	-	ENSG0000012561	'CHCHD5	-	2:112584239-112614431	-	-	IGV_LINK	162.241	17.2446	3.58449	30.9047	OK	221.713	24.4553	6.40302	42.5077	OK	OK	0.504004
ENSG0000012	-	-	ENSG0000012561	'PAX8	-	2:113211521-113278950	-	-	IGV_LINK	2323.45	64.7053	0	131.837	OK	5605.74	164.963	0	349.644	OK	OK	1.35019
ENSG0000012	-	-	ENSG0000012562	'INSIG2	-	2:118088451-118110997	-	-	IGV_LINK	819.414	27.3654	4.39395	50.3368	OK	1226.13	46.6269	7.562	85.6917	OK	OK	0.768809
ENSG0000012	-	-	ENSG0000012563	'POLR1B	-	2:112541914-112577150	-	-	IGV_LINK	411.161	6.73219	0.495511	12.9689	OK	383.616	6.88802	0.256905	13.5191	OK	OK	0.033014
ENSG0000012	-	-	ENSG0000012563	'HTR5BP	-	2:117859426-118055033	-	-	IGV_LINK	1.93944	0.109233	0	1.86168	OK	2.06245	0.114027	0	1.77148	OK	NOTEST	0.061963
ENSG0000012	-	-	ENSG0000012563	'CCDC93	-	2:117859426-118055033	-	-	IGV_LINK	1530.22	20.3596	0	51.0718	OK	1302.44	16.407	0	39.6028	OK	OK	-0.3114
ENSG0000012	-	-	ENSG0000012563	'PSD4	-	2:113157324-113209396	-	-	IGV_LINK	1413.85	19.3562	2.85097	35.8613	OK	1312.75	17.6879	4.52017	30.8556	OK	OK	-0.13003
ENSG0000012	-	-	ENSG0000012564	'SLC25A23	-	19:6436078-6482557	-	-	IGV_LINK	2040.29	39.0981	5.63682	72.5594	OK	1283.88	25.1693	4.19262	46.146	OK	OK	-0.63543
ENSG0000012	-	-	ENSG0000012565	'PSPN	-	19:6372432-6379058	-	-	IGV_LINK	35.8797	0.540865	0	1.86808	OK	41.249	0.622005	0	1.98558	OK	OK	0.201656
ENSG0000012	-	-	ENSG0000012565	'GTF2F1	-	19:6379568-6412404	-	-	IGV_LINK	1653.37	47.0322	13.2819	80.7824	OK	1694.3	45.4575	12.8313	78.0836	OK	OK	-0.04913
ENSG0000012	-	-	ENSG0000012565	'ALKBH7	-	19:6372432-6379058	-	-	IGV_LINK	541.104	47.3542	13.4026	81.3057	OK	431.052	33.0828	8.43505	57.7305	OK	OK	-0.51741
ENSG0000012	-	-	ENSG0000012565	'CLPP	-	19:6343760-6368908	-	-	IGV_LINK	509.103	34.0975	11.6042	56.5907	OK	746.607	51.1701	20.0923	82.2479	OK	OK	0.585636
ENSG0000012	-	-	ENSG0000012565	'TNFSF9	-	19:6530998-6535928	-	-	IGV_LINK	94.0629	3.55179	0.401623	6.70195	OK	87.6541	3.31235	0.327268	6.29743	OK	OK	-0.10069
ENSG0000012	-	-	ENSG0000012567	'GRIA3	-	X:123184152-123490915	-	-	IGV_LINK	6.78804	0.128248	0	0.606426	OK	97.9664	4.24098	0	9.74183	OK	OK	5.04739
ENSG0000012	-	-	ENSG0000012567	'THOC2	-	X:123600560-123733056	-	-	IGV_LINK	1030.81	25.6278	1.8044	49.4512	OK	856.948	21.5882	1.79664	41.3797	OK	OK	-0.24747
ENSG0000012	-	-	ENSG0000012568	'MED1	-	17:39252643-39451286	-	-	IGV_LINK	864.021	11.338	1.19262	21.4833	OK	767.231	9.96664	0	20.6079	OK	OK	-0.18598
ENSG0000012	-	-	ENSG0000012569	'RPL23	-	17:38847864-38853843	-	-	IGV_LINK	13428.7	1658.57	488.278	2828.87	OK	10340.1	1274.61	420.865	2128.35	OK	OK	-0.37989
ENSG0000012	-	-	ENSG0000012569	'AC046185.1	-	17:63622414-63819317	-	-	IGV_LINK	4.35E-21	1.25E-22	0	0.057639	OK	7.49E-60	2.16E-61	0	0.057673	OK	NOTEST	-128.772
ENSG0000012	-	-	ENSG0000012570	'ATG4C	-	1:62784134-62865513	-	-	IGV_LINK	211.399	5.84947	0	11.7362	OK	237.182	6.72683	0.197729	13.2559	OK	OK	0.201621
ENSG0000012	-	-	ENSG0000012572	'CD70	-	19:6583182-6604103	-	-	IGV_LINK	268.613	20.6638	6.34499	34.9826	OK	284.618	21.8671	7.01554	36.7187	OK	OK	0.081657
ENSG0000012	-	-	ENSG0000012573	'C3	-	19:6677703-6737603	-	-	IGV_LINK	54562.3	998.059	0	3528.94	OK	72470.4	961.084	0	3278.87	OK	OK	-0.05446
ENSG0000012	-	-	ENSG0000012573	'SH2D3A	-	19:6752159-6767588	-	-	IGV_LINK	207.52	7.7104	1.06534	14.3555	OK	283.587	9.60544	1.97604	17.2348	OK	OK	0.317046
ENSG0000012	-	-	ENSG0000012573	'TRIP10	-	19:6737924-6751526	-	-	IGV_LINK	1629.13	67.555	15.6559	119.454	OK	1030.19	40.8202	10.1699	71.4705	OK	OK	-0.72678
ENSG0000012	-	-	ENSG0000012573	'GPR108	-	19:6677703-6737603	-	-	IGV_LINK	1514.7	67.2413	0	626.428	OK	1463.31	68.9754	0	827.213	OK	OK	0.036733
ENSG0000012	-	-	ENSG0000012573	'TNFSF14	-	19:6661252-6670588	-	-	IGV_LINK	238.551	4.49334	0.598325	8.38835	OK	623.891	11.0464	2.69425	19.3985	OK	OK	1.29771
ENSG0000012	-	-	ENSG0000012574	'FOSB	-	19:45379633-45478828	-	-	IGV_LINK	21.3339	0.578456	0	6.93701	OK	661.015	12.1555	0	26.9733	OK	OK	4.39326
ENSG0000012	-	-	ENSG0000012574	'OPA3	-	19:45527426-45602212	-	-	IGV_LINK	489.709	4.55962	0	9.68086	OK	857.979	6.87399	1.6483	12.0997	OK	OK	0.592234
ENSG0000012	-	-	ENSG0000012574	'SNRPD2	-	19:45687453-45703989	-	-	IGV_LINK	1640.77	198	69.4101	326.589	OK	1482.9	174.051	62.9518	285.149	OK	OK	-0.18599
ENSG0000012	-	-	ENSG0000012574	'RTN2	-	19:45485288-45502510	-	-	IGV_LINK	85.3354	5.05556	0	12.7099	OK	164.996	6.53628	0	14.7024	OK	OK	0.370599
ENSG0000012	-	-	ENSG0000012574	'EML2	-	19:45606993-45645629	-	-	IGV_LINK	665.228	29.0791	6.79721	51.3609	OK	649.672	27.5872	5.89901	49.2755	OK	OK	-0.07598
ENSG0000012	-	-	ENSG0000012575	'VASP	-	19:45506578-45526983	-	-	IGV_LINK	1225.73	36.5231	9.3774	63.6687	OK	1483.93	47.0711	7.005	87.1371	OK	OK	0.366033
ENSG0000012	-	-	ENSG0000012575	'SYMPK	-	19:45815409-45863290	-	-	IGV_LINK	1967.56	30.0797	6.45681	53.7025	OK	2011.92	32.8432	5.97604	59.7103	OK	OK	0.126805
ENSG0000012	-	-	ENSG0000012577	'GPCPD1	-	20:5544403-5611026	-	-	IGV_LINK	1176.27	14.9676	1.78561	28.1496	OK	893.041	14.0408	0.364301	27.7172	OK	OK	-0.09222
ENSG0000012	-	-	ENSG0000012577	'SDCBP2	-	20:1309908-1393172	-	-	IGV_LINK	86.8963	4.80252	0	22.3973	OK	34.4846	1.63765	0	10.4086	OK	OK	-1.55216
ENSG0000012	-	-	ENSG0000012577	'PANK2	-	20:3888238-4015582	-	-	IGV_LINK	567.286	22.8917	0	47.8775	OK	609.454	26.7692	0.101839	53.4365	OK	OK	0.225749
ENSG0000012	-	-	ENSG0000012578	'TGM3	-	20:2296000-2341078	-	-	IGV_LINK	6.78804	0.155043	0	0.544662	OK	2.06245	0.047089	0	0.282367	OK	NOTEST	-1.71921
ENSG0000012	-	-	ENSG0000012578	'GNRH2	-	20:3043621-3045747	-	-	IGV_LINK	0	0	0	0	OK	3.09367	0.429948	0	1.29076	OK	NOTEST	inf
ENSG0000012	-	-	ENSG0000012578	'DEFB126	-	20:142368-145751	-	-	IGV_LINK	0	0	0	0	OK	0	0	0	0	OK	NOTEST	0
ENSG0000012	-	-	ENSG0000012579	'FOXA2	-	20:22547670-22585455	-	-	IGV_LINK	0	0	0	0	OK	0	0	0	0	OK	NOTEST	0
ENSG0000012	-	-	ENSG0000012580	'FAM182A	-	20:26054654-26086917	-	-	IGV_LINK	39.7585	0.457502	0	1.1069	OK	44.3427	0.510372	0	1.19827	OK	NOTEST	0.15777
ENSG0000012	-	-	ENSG0000012581	'CD93	-	20:23079348-23086340	-	-	IGV_LINK	2111.08	18.2038	7.92603	28.4816	OK	3550.51	30.6214	13.1102	48.1325	OK	OK	0.750297
ENSG0000012	-	-	ENSG0000012581	'GZF1	-	20:23362181-23373063	-	-	IGV_LINK	227.884	3.90567	0	9.37002	OK	298.024	8.29706	0	21.4296	OK	OK	1.08703
ENSG0000012	-	-	ENSG0000012581	'PAX1	-	20:21705658-21718486	-	-	IGV_LINK	0	0	0	0	OK	0	0	0	0	OK	NOTEST	0
ENSG0000012	-	-	ENSG0000012581	'NAPB	-	20:23374518-23421519	-	-	IGV_LINK	360.736	6.12755	1.65839	10.5967	OK	337.211	5.61712	1.25512	9.97913	OK	OK	-0.12548
ENSG0000012	-	-	ENSG0000012581	'CST8	-	20:23481644-23519054	-	-	IGV_LINK	0	0	0	0	OK	0	0	0	0	OK	NOTEST	0
ENSG0000012	-	-	ENSG0000012581	'NKX2-4	-	20:21395366-21400391	-	-	IGV_LINK	0	0	0	0	OK	0	0	0	0	OK	NOTEST	0
ENSG0000012	-	-	ENSG0000012581	'CENPB	-	20:3783850-3786690	-	-	IGV_LINK	1193.73	25.2197	10.9801	39.4593	OK	1237.47	26.1551	11.4302	40.8801	OK	OK	0.052544
ENSG0000012	-	-	ENSG0000012581	'PSMF1	-	20:1113262-1208274	-	-	IGV_LINK	2263.33	77.2215	12.3159	142.127	OK	2198.57	83.6508	13.6042	153.697	OK	OK	0.115376
ENSG0000012	-	-	ENSG0000012582	'NKX2-2	-	20:21511009-21514026	-	-	IGV_LINK	0	0	0	0	OK	0	0	0	0	OK	NOTEST	0
ENSG0000012	-	-	ENSG0000012582	'DTD1	-	20:18567346-18763917	-	-	IGV_LINK	626.885	30.0198	5.48018	54.5595	OK	773.564	38.0238	9.14703	66.9006	OK	OK	0.340988
ENSG0000012	-	-	ENSG0000012582	'CSTL1	-	20:23439684-23444930	-	-	IGV_LINK	0	0	0	0	OK	0	0	0	0	OK	NOTEST	0
ENSG0000012	-	-	ENSG0000012582	'RBCK1	-	20:407497-430966	-	-	IGV_LINK	2459.21	93.1051	22.374	163.836	OK	2602.81	95.8259	25.4687	166.183	OK	OK	0.041557
ENSG0000012	-	-	ENSG0000012582	'TMX4	-	20:7977347-8043512	-	-	IGV_LINK	1362.46	13.4326	4.6016	22.2635	OK	1550.96	17.2183	2.00011	32.4364	OK	OK	0.358205
ENSG0000012	-	-	ENSG0000012583	'CST11	-	20:23450402-23452857	-	-	IGV_LINK	0	0	0	0	OK	0	0	0	0	OK	NOTEST	0
ENSG0000012	-	-	ENSG0000012583	'STK35	-	20:2101610-2177038	-	-	IGV_LINK	578.923	5.00968	1.96891	8.05046	OK	691.952	5.98883	2.45754	9.52012	OK	OK	0.257555
ENSG0000012	-	-	ENSG0000012583	'SNRPB	-	20:2461633-2524702	-	-	IGV_LINK	1472.5	87.284	30.9077	143.66	OK	1448.42	86.1103	29.2862	142.934	OK	OK	-0.01953
ENSG0000012	-	-	ENSG0000012584	'NRSN2	-	20:320312-359660	-	-	IGV_LINK	544.983	19.7382	0.972459	38.504	OK	698.139	23.0866	3.43527	42.7379	OK	OK	0.226065
ENSG0000012	-	-	ENSG0000012584	'AP5S1	-	20:3808356-3828837	-	-	IGV_LINK	179.504	4.05036	0.021062	8.07965	OK	227.958	6.3177	0.958701	11.6767	OK	OK	0.64135
ENSG0000012	-	-	ENSG0000012584	'RRBP1	-	20:17613677-17682295	-	-	IGV_LINK	2580.43	37.2482	12.5881	61.9083	OK	4005.28	56.7961	18.3062	95.286	OK	OK	0.608622
ENSG0000012	-	-	ENSG0000012584	'BMP2	-	20:6767663-6780280	-	-	IGV_LINK	115.397	1.89683	0.182843	3.61081	OK	127.872	2.10259	0.373031	3.83215	OK	OK	0.148581
ENSG0000012	-	-	ENSG0000012584	'ZNF133	-	20:18288282-18316996	-	-	IGV_LINK	406.313	11.4131	3.08657	19.7397	OK	346.492	11.294	1.61179	20.9763	OK	OK	-0.01513
ENSG0000012	-	-	ENSG0000012584	'FLRT3	-	20:13849246-16053197	-	-	IGV_LINK	25.2127	0.374064	0	1.14572	OK	12.3747	0.168294	0	0.548878	OK	NOTEST	-1.1523
ENSG0000012	-	-	ENSG0000012585	'OVOL2	-	20:17941596-18059188	-	-	IGV_LINK	0	0	0	0	OK	2.06245	0.142383	0	3.73452	OK	NOTEST	inf
ENSG0000012	-	-	ENSG0000012585	'PCSK2	-	20:17226106-17484578	-	-	IGV_LINK	0	0	0	0	OK	0	0	0	0	OK	NOTEST	0
ENSG0000012	-	-	ENSG0000012586	'GFRA4	-	20:3659291-3663399	-	-	IGV_LINK	0	0	0	0	OK	0	0	0	0	OK	NOTEST	0
ENSG0000012	-	-	ENSG0000012586	'MKKS	-	20:10401008-10434222	-	-	IGV_LINK	553.71	34.3902	0	119.276	OK	510.456	52.9986	0	164.257	OK	OK	0.623959
ENSG0000012	-	-	ENSG0000012586	'BFSP1	-	20:17493904-17569220	-	-	IGV_LINK	7.75776	0.304937	0	0.864165	OK	12.3747	0.369069	0	0.906422	OK	NOTEST	0.275375
ENSG0000012	-	-	ENSG0000012586	'DSTN	-	20:17569862-17609919	-	-	IGV_LINK	2533.88	106.985	38.2628	175.708	OK	2503.81	109.822	38.0395	181.604	OK	OK	0.037749
ENSG0000012	-	-	ENSG0000012586	'LAMP5	-	20:9505179-9530524	-	-	IGV_LINK	3.87888	0.117117	0	0.31578	OK	17.5308	0.529644	0	1.13232	OK	NOTEST	2.17707
ENSG0000012	-	-	ENSG0000012587	'SNRPB2	-	20:16714843-16742563	-	-	IGV_LINK	553.71	31.6612	3.22083	60.1016	OK	564.08	32.147	2.96944	61.3245	OK	OK	0.021966
ENSG0000012	-	-	ENSG0000012587	'MGME1	-	20:17941596-18059188	-	-	IGV_LINK	470.314	15.3019	0	41.5522	OK	578.517	18.759	0	53.6272	OK	OK	0.293875
ENSG0000012	-	-	ENSG0000012587	'LRRN4	-	20:6040777-6054049	-	-	IGV_LINK	70.7896	1.58342	0.001864	3.16497	OK	94.8727	2.12307	0.243843	4.0023	OK	OK	0.423114
ENSG0000012	-	-	ENSG0000012587	'TBC1D20	-	20:435479-462553	-	-	IGV_LINK	971.66	14.0519	5.26911	22.8347	OK	1053.91	15.2397	5.89025	24.5892	OK	OK	0.117073
ENSG0000012	-	-	ENSG0000012587	'ITPA	-	20:3208867-3223870	-	-	IGV_LINK	788.383	53.0116	16.5958	89.4273	OK	767.231	48.8111	19.459	78.1633	OK	OK	-0.1191
ENSG0000012	-	-	ENSG0000012587	'TCF15	-	20:603796-610398	-	-	IGV_LINK	3.87888	0.158119	0	0.402704	OK	19.5933	0.799369	0	1.76889	OK	OK	2.33785
ENSG0000012	-	-	ENSG0000012587	'OTOR	-	20:16748357-16770062	-	-	IGV_LINK	0	0	0	0	OK	0	0	0	0	OK	NOTEST	0
ENSG0000012	-	-	ENSG0000012588	'MCM8	-	20:5950651-6005821	-	-	IGV_LINK	192.005	2.39037	0.365141	4.4156	OK	180.464	2.06668	0.351081	3.78227	OK	OK	-0.20992
ENSG0000012	-	-	ENSG0000012588	'BANF2	-	20:17693671-17735871	-	-	IGV_LINK	0	0	0	0	OK	0	0	0	0	OK	NOTEST	0
ENSG0000012	-	-	ENSG0000012589	'TMEM74B	-	20:1113262-1208274	-	-	IGV_LINK	142.549	6.23025	0	21.993	OK	145.403	7.02257	0	24.9716	OK	OK	0.172709
ENSG0000012	-	-	ENSG0000012589	'FAM110A	-	20:833714-857463	-	-	IGV_LINK	272.491	9.63902	2.97208	16.306	OK	241.307	8.55517	2.52413	14.5862	OK	OK	-0.17209
ENSG0000012	-	-	ENSG0000012589	'C20orf187	-	20:11027759-11029366	-	-	IGV_LINK	0	0	0	0	OK	0	0	0	0	OK	NOTEST	0
ENSG0000012	-	-	ENSG0000012590	'SIRPD	-	20:1516758-1620061	-	-	IGV_LINK	7.59641	0.381877	0	2.21405	OK	9.55937	0.465286	0	3.31261	OK	NOTEST	0.285008
ENSG0000012	-	-	ENSG0000012590	'MRPS26	-	20:3045944-3048254	-	-	IGV_LINK	352.978	20.8411	7.26979	34.4123	OK	346.492	20.4827	7.10139	33.8641	OK	OK	-0.02502
ENSG0000012	-	-	ENSG0000012590	'DEFB129	-	20:227257-229886	-	-	IGV_LINK	0	0	0	0	OK	0	0	0	0	OK	NOTEST	0
ENSG0000012	-	-	ENSG0000012591	'S1PR4	-	19:3172345-3180332	-	-	IGV_LINK	96.0023	3.88535	0.376565	7.39414	OK	97.9664	3.96812	0.364124	7.57211	OK	OK	0.03041
ENSG0000012	-	-	ENSG0000012591	'NCLN	-	19:3185562-3209575	-	-	IGV_LINK	721.472	14.7429	4.79619	24.6896	OK	776.512	15.378	5.50307	25.2529	OK	OK	0.060846
ENSG0000012	-	-	ENSG0000012593	'CITED1	-	X:72181352-72307187	-	-	IGV_LINK	0.969721	0.084404	0	7.45339	OK	1.03122	0.089917	0	6.80089	OK	NOTEST	0.091283
ENSG0000012	-	-	ENSG0000012594	'HNRNPR	-	1:23303770-23344336	-	-	IGV_LINK	1463.31	50.0157	13.2193	86.8121	OK	1616.96	48.7224	15.1739	82.2709	OK	OK	-0.0378
ENSG0000012	-	-	ENSG0000012594	'ZNF436	-	1:23359447-23371839	-	-	IGV_LINK	952.266	14.1742	4.61051	23.7378	OK	1119.91	16.5393	5.33689	27.7417	OK	OK	0.222635
ENSG0000012	-	-	ENSG0000012595	'MAX	-	14:64914360-65102695	-	-	IGV_LINK	1063.78	33.1922	0	72.7848	OK	1097.22	39.5176	0	84.7524	OK	OK	0.251654
ENSG0000012	-	-	ENSG0000012595	'CHURC1-FNTB	-	14:64914360-65102695	-	-	IGV_LINK	3.78994	0.164869	0	2.57003	OK	8.21209	0.276573	0	3.1121	OK	NOTEST	0.746339
ENSG0000012	-	-	ENSG0000012596	'ARMCX5	-	X:102599167-102717733	-	-	IGV_LINK	257.188	5.17686	0	11.9257	OK	277.425	7.28164	0	16.134	OK	OK	0.492185
ENSG0000012	-	-	ENSG0000012596	'GDF5	-	20:35433028-35454746	-	-	IGV_LINK	0	0	0	0	OK	1.03122	0.026301	0	0.112572	OK	NOTEST	inf
ENSG0000012	-	-	ENSG0000012596	'MMP24	-	20:35172072-35284985	-	-	IGV_LINK	302.553	4.01994	0	11.3957	OK	295.962	3.93343	0	12.2577	OK	OK	-0.03139
ENSG0000012	-	-	ENSG0000012596	'NECAB3	-	20:33657086-33674463	-	-	IGV_LINK	201.702	9.64771	0.293367	19.0021	OK	209.339	10.754	0.461385	21.0466	OK	OK	0.156612
ENSG0000012	-	-	ENSG0000012596	'ID1	-	20:31605282-31606515	-	-	IGV_LINK	179.398	12.1953	2.9907	21.3999	OK	285.649	19.1475	6.0771	32.2178	OK	OK	0.650829
ENSG0000012	-	-	ENSG0000012597	'RALY	-	20:33983051-34112332	-	-	IGV_LINK	2237.15	92.5514	17.3895	167.713	OK	2039.76	85.6488	12.1985	159.099	OK	OK	-0.11182
ENSG0000012	-	-	ENSG0000012597	'DYNLRB1	-	20:34516408-34540958	-	-	IGV_LINK	1327.55	147.684	65.121	230.248	OK	1408.65	157.025	69.3919	244.658	OK	OK	0.088476
ENSG0000012	-	-	ENSG0000012597	'C20orf173	-	20:35520851-35529652	-	-	IGV_LINK	0	0	0	0	OK	1.03122	0.06755	0	0.449341	OK	NOTEST	inf
ENSG0000012	-	-	ENSG0000012597	'EIF2S2	-	20:33983051-34112332	-	-	IGV_LINK	1231.55	27.9464	0.647569	55.2452	OK	1435.47	32.5889	2.95934	62.2184	OK	OK	0.221717
ENSG0000012	-	-	ENSG0000012599	'ERGIC3	-	20:35542020-35557634	-	-	IGV_LINK	3176.8	165.107	61.5298	268.684	OK	3584.54	183.686	68.5966	298.776	OK	OK	0.15384
ENSG0000012	-	-	ENSG0000012599	'ROMO1	-	20:35626030-35700984	-	-	IGV_LINK	491.648	104.679	0	270.211	OK	633.172	136.533	0	340.111	OK	OK	0.383271
ENSG0000012	-	-	ENSG0000012599	'BPIFB9P	-	20:33347719-33354444	-	-	IGV_LINK	0	0	0	0	OK	1.03122	0.156504	0	0.601293	OK	NOTEST	inf
ENSG0000012	-	-	ENSG0000012599	'FAM83C	-	20:35285250-35292401	-	-	IGV_LINK	0	0	0	0	OK	0	0	0	0	OK	NOTEST	0
ENSG0000012	-	-	ENSG0000012599	'BPIFB1	-	20:33273479-33309878	-	-	IGV_LINK	0	0	0	0	OK	0	0	0	0	OK	NOTEST	0
ENSG0000012	-	-	ENSG0000012600	'CEP250	-	20:35455163-35519280	-	-	IGV_LINK	914.446	17.7743	0	41.9888	OK	988.945	17.3672	0	39.9043	OK	OK	-0.03342
ENSG0000012	-	-	ENSG0000012600	'PLAGL2	-	20:32192502-32207791	-	-	IGV_LINK	395.646	4.03016	1.4566	6.60372	OK	369.179	3.76134	1.29816	6.22451	OK	OK	-0.09959
ENSG0000012	-	-	ENSG0000012600	'MMP24-AS1	-	20:35172072-35284985	-	-	IGV_LINK	895.177	54.1888	0	118.436	OK	899.097	52.3732	0	128.239	OK	OK	-0.04917
ENSG0000012	-	-	ENSG0000012601	'GRPR	-	X:16123555-16171464	-	-	IGV_LINK	1.93944	0.062226	0	0.232	OK	5.15612	0.16554	0	0.53995	OK	NOTEST	1.41159
ENSG0000012	-	-	ENSG0000012601	'KDM5C	-	X:53191320-53225422	-	-	IGV_LINK	1587.43	22.2758	0	50.4524	OK	1736.58	23.3842	1.73993	45.0286	OK	OK	0.070058
ENSG0000012	-	-	ENSG0000012601	'AMOT	-	X:112774502-112840815	-	-	IGV_LINK	400.495	3.38853	0.33115	6.44592	OK	456.833	4.6308	0	10.348	OK	OK	0.450601
ENSG0000012	-	-	ENSG0000012606	'TMEM115	-	3:50347329-50504244	-	-	IGV_LINK	607.045	16.8983	2.8783	30.9184	OK	741.451	20.6515	3.49508	37.808	OK	OK	0.289367
ENSG0000012	-	-	ENSG0000012606	'PSMB2	-	1:35599543-35641844	-	-	IGV_LINK	1219.91	35.639	11.1328	60.1451	OK	1603.55	47.8139	13.7794	81.8484	OK	OK	0.423976
ENSG0000012	-	-	ENSG0000012607	'AGO3	-	1:35930717-36072500	-	-	IGV_LINK	577.953	2.64133	0	6.45665	OK	444.458	3.22287	0	8.1488	OK	OK	0.28708
ENSG0000012	-	-	ENSG0000012608	'UROD	-	1:45012146-45015575	-	-	IGV_LINK	1651.43	97.3796	39.4319	155.327	OK	1741.74	99.7115	40.3662	159.057	OK	OK	0.03414
ENSG0000012	-	-	ENSG0000012609	'ST3GAL3	-	1:43650157-43931165	-	-	IGV_LINK	228.854	9.02741	0	22.6072	OK	196.964	6.86734	0	19.3212	OK	OK	-0.39456
ENSG0000012	-	-	ENSG0000012610	'TMEM53	-	1:44405193-44674555	-	-	IGV_LINK	124.124	5.33173	0	15.4865	OK	184.589	9.10199	0	27.8633	OK	OK	0.771579
ENSG0000012	-	-	ENSG0000012610	'HECTD3	-	1:45002539-45011329	-	-	IGV_LINK	1216.03	29.4489	2.2726	56.6251	OK	1610.77	33.0402	10.0403	56.0401	OK	OK	0.16601
ENSG0000012	-	-	ENSG0000012621	'KLC1	-	14:103553420-103715504	-	-	IGV_LINK	1162.61	42.5632	0	90.0298	OK	908.503	34.1778	0	75.1661	OK	OK	-0.31655
ENSG0000012	-	-	ENSG0000012621	'XRCC3	-	14:103553420-103715504	-	-	IGV_LINK	269.582	10.2676	0	40.7298	OK	263.994	9.81606	0	38.9523	OK	OK	-0.06488
ENSG0000012	-	-	ENSG0000012621	'TUBGCP3	-	13:112485004-112588167	-	-	IGV_LINK	496.497	12.758	0.618927	24.897	OK	502.207	10.5875	1.24197	19.9331	OK	OK	-0.26903
ENSG0000012	-	-	ENSG0000012621	'MCF2L	-	13:112894377-113099739	-	-	IGV_LINK	1545.73	30.2013	5.5396	54.8631	OK	1026.07	21.6498	4.16637	39.1333	OK	OK	-0.48026
ENSG0000012	-	-	ENSG0000012621	'F10	-	13:113122813-113154002	-	-	IGV_LINK	33.9402	1.49563	0	3.59298	OK	10.3122	0.432622	0	1.12667	OK	OK	-1.78957
ENSG0000012	-	-	ENSG0000012622	'PCID2	-	13:113177538-113267108	-	-	IGV_LINK	829.111	27.08	0	57.7673	OK	586.767	19.1139	0	42.0104	OK	OK	-0.50261
ENSG0000012	-	-	ENSG0000012623	'PROZ	-	13:113158653-113172383	-	-	IGV_LINK	3.87888	0.166164	0	0.553297	OK	1.03122	0.044207	0	0.356027	OK	NOTEST	-1.91026
ENSG0000012	-	-	ENSG0000012623	'SLURP1	-	8:142740943-142742411	-	-	IGV_LINK	0	0	0	0	OK	0	0	0	0	OK	NOTEST	0
ENSG0000012	-	-	ENSG0000012624	'LRFN3	-	19:35935357-35945767	-	-	IGV_LINK	39.7585	0.959887	0	2.53892	OK	146.434	4.90009	0	11.0091	OK	OK	2.35187
ENSG0000012	-	-	ENSG0000012624	'IGFLR1	-	19:35718018-35754519	-	-	IGV_LINK	454.295	32.9653	0	101.003	OK	544.452	44.3706	0	140.819	OK	OK	0.428656
ENSG0000012	-	-	ENSG0000012624	'CAPNS1	-	19:36139574-36150353	-	-	IGV_LINK	4113.55	399.352	138.518	660.186	OK	4410.55	423.535	142.326	704.744	OK	OK	0.084819
ENSG0000012	-	-	ENSG0000012624	'PDCD2L	-	19:34359479-34428273	-	-	IGV_LINK	33.2313	1.86248	0	17.6229	OK	41.9036	2.37471	0	21.3162	OK	OK	0.350527

ENSG0000012	-	-	ENSG0000012625	'GPR42	-	19:35370928-35372962	-	-	IGV_LINK	0	0	0	0	OK	0	0	0	0	OK	NOTEST	0
ENSG0000012	-	-	ENSG0000012625	'RBM42	-	19:35629029-35637686	-	-	IGV_LINK	857.233	33.6261	14.021	53.2313	OK	888.916	34.7574	13.1007	56.4141	OK	OK	0.047739
ENSG0000012	-	-	ENSG0000012625	'KIRREL2	-	19:35825963-35879791	-	-	IGV_LINK	0	0	0	0	OK	0	0	0	0	OK	NOTEST	0
ENSG0000012	-	-	ENSG0000012626	'UBA2	-	19:34428351-34471251	-	-	IGV_LINK	1462.34	71.6244	19.6652	123.584	OK	1088.97	54.0876	14.1336	94.0416	OK	OK	-0.40515
ENSG0000012	-	-	ENSG0000012626	'FFAR2	-	19:35443906-35451767	-	-	IGV_LINK	10.6669	0.38544	0	1.23226	OK	86.6229	2.49154	0.102421	4.88066	OK	OK	2.69246
ENSG0000012	-	-	ENSG0000012626	'HCST	-	19:35887652-35904377	-	-	IGV_LINK	306.432	48.0999	4.38987	91.8099	OK	210.37	32.9769	0	69.0532	OK	OK	-0.54458
ENSG0000012	-	-	ENSG0000012626	'FFAR1	-	19:35351551-35353862	-	-	IGV_LINK	0	0	0	0	OK	0	0	0	0	OK	NOTEST	0
ENSG0000012	-	-	ENSG0000012626	'COX6B1	-	19:35648222-35658861	-	-	IGV_LINK	2255.57	257.556	113.05	402.063	OK	2028.42	234.599	101.473	367.726	OK	OK	-0.13469
ENSG0000012	-	-	ENSG0000012633	'KRT36	-	17:41486135-41492546	-	-	IGV_LINK	1.93944	0.074269	0	0.244009	OK	6.18735	0.227409	0	0.553171	OK	NOTEST	1.61445
ENSG0000012	-	-	ENSG0000012635	'THRA	-	17:40019096-40100725	-	-	IGV_LINK	1029.84	19.3698	0	39.5534	OK	816.73	15.5153	0	33.1521	OK	OK	-0.32012
ENSG0000012	-	-	ENSG0000012635	'CCR7	-	17:40553768-40565472	-	-	IGV_LINK	99.8812	2.81376	0.23354	5.39397	OK	81.4668	2.32925	0.064917	4.59359	OK	OK	-0.27263
ENSG0000012	-	-	ENSG0000012636	'NR1D1	-	17:40019096-40100725	-	-	IGV_LINK	549.832	11.9201	0	28.4236	OK	492.926	10.6911	0	24.616	OK	OK	-0.15698
ENSG0000012	-	-	ENSG0000012639	'FRMD8	-	11:65386598-65413525	-	-	IGV_LINK	688.502	11.9495	2.43445	21.4646	OK	837.355	14.944	2.06372	27.8243	OK	OK	0.322616
ENSG0000012	-	-	ENSG0000012643	'PRDX5	-	11:64318087-64321811	-	-	IGV_LINK	2777.28	219.449	94.9519	343.947	OK	2879.18	228.316	94.276	362.356	OK	OK	0.057143
ENSG0000012	-	-	ENSG0000012645	'BCL2L12	-	19:49659568-49673916	-	-	IGV_LINK	212.369	9.29015	0	28.5269	OK	225.838	10.0156	0	33.0781	OK	OK	0.108477
ENSG0000012	-	-	ENSG0000012645	'IRF3	-	19:49659568-49673916	-	-	IGV_LINK	1551.55	73.7807	14.919	132.642	OK	1597.37	72.7819	16.6201	128.944	OK	OK	-0.01966
ENSG0000012	-	-	ENSG0000012645	'PRMT1	-	19:49675785-49690575	-	-	IGV_LINK	1420.25	76.8312	28.4302	125.232	OK	1112.19	62.272	22.9587	101.585	OK	OK	-0.30311
ENSG0000012	-	-	ENSG0000012645	'RRAS	-	19:49635291-49640201	-	-	IGV_LINK	770.928	50.177	20.932	79.4221	OK	946.665	61.556	25.2418	97.8702	OK	OK	0.294872
ENSG0000012	-	-	ENSG0000012646	'PRRG2	-	19:49555710-49591015	-	-	IGV_LINK	3.87888	0.160076	0	1.46934	OK	16.4996	0.772313	0	3.70712	OK	OK	2.27043
ENSG0000012	-	-	ENSG0000012646	'SCAF1	-	19:49642124-49658642	-	-	IGV_LINK	784.504	11.8844	2.30735	21.4614	OK	998.226	13.9135	4.86737	22.9597	OK	OK	0.227423
ENSG0000012	-	-	ENSG0000012646	'PRR12	-	19:49591642-49626439	-	-	IGV_LINK	622.561	7.0447	0	19.9793	OK	708.452	8.37643	0	24.9417	OK	OK	0.249797
ENSG0000012	-	-	ENSG0000012646	'TSKS	-	19:49739752-49763330	-	-	IGV_LINK	6.78804	0.223651	0	0.591856	OK	9.28102	0.378142	0	0.921482	OK	NOTEST	0.757678
ENSG0000012	-	-	ENSG0000012650	'FLRT1	-	11:63974606-64166106	-	-	IGV_LINK	5.81832	0.086825	0	1.33142	OK	6.18735	0.09236	0	1.38372	OK	NOTEST	0.089158
ENSG0000012	-	-	ENSG0000012652	'ASL	-	7:66075797-66165011	-	-	IGV_LINK	932.987	39.8987	11.056	68.7413	OK	1723.64	73.0256	21.6768	124.375	OK	OK	0.872062
ENSG0000012	-	-	ENSG0000012652	'SBDS	-	7:66987676-67239519	-	-	IGV_LINK	1131.66	45.8431	14.3306	77.3556	OK	1179.72	45.6263	13.0322	78.2204	OK	OK	-0.00684
ENSG0000012	-	-	ENSG0000012654	'CSN1S1	-	4:69931080-69946574	-	-	IGV_LINK	0	0	0	0	OK	1.03122	0.083314	0	0.878368	OK	NOTEST	inf
ENSG0000012	-	-	ENSG0000012654	'STATH	-	4:69995929-70002570	-	-	IGV_LINK	0	0	0	0	OK	0	0	0	0	OK	NOTEST	0
ENSG0000012	-	-	ENSG0000012655	'HTN1	-	4:70050401-70058845	-	-	IGV_LINK	0	0	0	0	OK	0	0	0	0	OK	NOTEST	0
ENSG0000012	-	-	ENSG0000012656	'STAT5A	-	17:42287546-42311943	-	-	IGV_LINK	1398.34	27.9977	5.71489	50.2805	OK	1245.72	25.6416	5.55673	45.7264	OK	OK	-0.12682
ENSG0000012	-	-	ENSG0000012656	'WNK4	-	17:42780677-42798704	-	-	IGV_LINK	460.617	9.17274	0	22.8783	OK	716.701	13.1218	0	32.7275	OK	OK	0.51654
ENSG0000012	-	-	ENSG0000012658	'BECN1	-	17:42798791-42843758	-	-	IGV_LINK	1278.09	48.4129	0.193387	96.6325	OK	1974.8	81.3496	0	163.003	OK	OK	0.748743
ENSG0000012	-	-	ENSG0000012658	'PRKCG	-	19:53879189-53907652	-	-	IGV_LINK	0	0	0	0	OK	3.09367	0.058914	0	0.395026	OK	NOTEST	inf
ENSG0000012	-	-	ENSG0000012660	'TRAP1	-	16:3611727-3717597	-	-	IGV_LINK	2235.21	80.8158	15.0319	146.6	OK	1980.98	71.3773	14.9161	127.839	OK	OK	-0.17917
ENSG0000012	-	-	ENSG0000012660	'GLIS2	-	16:4314760-4425705	-	-	IGV_LINK	571.165	9.11209	0	21.5067	OK	743.513	11.767	0	27.5559	OK	OK	0.368887
ENSG0000012	-	-	ENSG0000012665	'NSRP1	-	17:29560546-30186475	-	-	IGV_LINK	694.32	21.4112	0	63.5369	OK	813.637	24.7229	0	73.8432	OK	OK	0.207482
ENSG0000012	-	-	ENSG0000012669	'DNAJC8	-	1:28199455-28233025	-	-	IGV_LINK	1535.07	60.6013	23.2183	97.9842	OK	1376.69	54.9493	20.9142	88.9843	OK	OK	-0.14125
ENSG0000012	-	-	ENSG0000012670	'AHDC1	-	1:27534034-27604431	-	-	IGV_LINK	727.29	7.23775	0.487477	13.988	OK	778.575	7.37721	1.69559	13.0588	OK	OK	0.027534
ENSG0000012	-	-	ENSG0000012670	'IFI6	-	1:27666060-27703063	-	-	IGV_LINK	1374.09	116.211	49.5792	182.842	OK	3181.33	269.692	115.973	423.411	OK	OK	1.21457
ENSG0000012	-	-	ENSG0000012673	'DACH2	-	X:86148457-86832604	-	-	IGV_LINK	8.72748	0.14017	0	0.406316	OK	0	0	0	0	OK	NOTEST	'-inf
ENSG0000012	-	-	ENSG0000012674	'ZNF384	-	12:6663259-6689572	-	-	IGV_LINK	1519.55	52.8203	14.2594	91.3811	OK	1568.49	53.8739	15.8003	91.9475	OK	OK	0.028495
ENSG0000012	-	-	ENSG0000012674	'EMG1	-	12:6970892-7071032	-	-	IGV_LINK	272.638	12.386	0	97.648	OK	348.968	14.5138	0	257.904	OK	OK	0.228713
ENSG0000012	-	-	ENSG0000012675	'SSX1	-	X:48255316-48267444	-	-	IGV_LINK	0	0	0	0	OK	0	0	0	0	OK	NOTEST	0
ENSG0000012	-	-	ENSG0000012675	'UXT	-	X:47651797-47660377	-	-	IGV_LINK	753.473	105.246	41.7057	168.786	OK	667.203	93.0096	36.6623	149.357	OK	OK	-0.17832
ENSG0000012	-	-	ENSG0000012675	'CFP	-	X:47561099-47630305	-	-	IGV_LINK	74.6685	3.15037	0	23.0375	OK	110.341	4.14852	0	24.5246	OK	OK	0.397077
ENSG0000012	-	-	ENSG0000012676	'ELK1	-	X:47635520-47650604	-	-	IGV_LINK	450.92	12.2557	3.3147	21.1967	OK	475.395	11.822	3.22906	20.4149	OK	OK	-0.05199
ENSG0000012	-	-	ENSG0000012676	'TIMM17B	-	X:48893446-48903143	-	-	IGV_LINK	510.073	36.6138	0	73.7999	OK	524.894	37.6671	3.09412	72.2401	OK	OK	0.040917
ENSG0000012	-	-	ENSG0000012677	'PCNX4	-	14:60091910-60169856	-	-	IGV_LINK	692.38	13.3116	0	31.3776	OK	624.922	13.6556	0	34.2806	OK	OK	0.036802
ENSG0000012	-	-	ENSG0000012677	'ATG14	-	14:55366391-55411858	-	-	IGV_LINK	505.224	6.24882	2.22851	10.2691	OK	404.24	4.99742	1.52309	8.47175	OK	OK	-0.3224
ENSG0000012	-	-	ENSG0000012677	'KTN1	-	14:55499277-55701526	-	-	IGV_LINK	1910.35	31.6809	10.0516	53.3103	OK	1803.61	30.9799	8.70418	53.2556	OK	OK	-0.03228
ENSG0000012	-	-	ENSG0000012677	'SIX1	-	14:60643414-60658259	-	-	IGV_LINK	6.78804	0.116146	0	0.427577	OK	14.4371	0.292487	0	0.751633	OK	NOTEST	1.33243
ENSG0000012	-	-	ENSG0000012678	'RHOJ	-	14:63204113-63293219	-	-	IGV_LINK	176.489	3.61245	0.011854	7.21304	OK	214.495	5.47262	0	11.695	OK	OK	0.599255
ENSG0000012	-	-	ENSG0000012678	'DLGAP5	-	14:55148111-55191678	-	-	IGV_LINK	98.9115	2.08348	0.091959	4.075	OK	40.2178	1.03915	0	2.39756	OK	OK	-1.00358
ENSG0000012	-	-	ENSG0000012679	'L3HYPDH	-	14:59429021-59576831	-	-	IGV_LINK	248.854	17.4956	0	39.4467	OK	216.557	13.8666	0	31.6075	OK	OK	-0.33538
ENSG0000012	-	-	ENSG0000012680	'HSPA2	-	14:64388030-64546173	-	-	IGV_LINK	44.6071	1.13668	0	7.88207	OK	187.683	4.361	0	15.3831	OK	OK	1.93984
ENSG0000012	-	-	ENSG0000012680	'ZBTB1	-	14:64388030-64546173	-	-	IGV_LINK	671.047	15.6675	0	40.1086	OK	596.048	13.2366	0	33.1698	OK	OK	-0.24324
ENSG0000012	-	-	ENSG0000012681	'TRMT5	-	14:60734741-61083733	-	-	IGV_LINK	160.963	2.81264	0	8.87351	OK	151.59	3.84201	0	11.9895	OK	OK	0.449936
ENSG0000012	-	-	ENSG0000012682	'SGPP1	-	14:63684213-63728039	-	-	IGV_LINK	214.308	3.84696	0.93956	6.75435	OK	195.933	3.51839	0.919649	6.11714	OK	OK	-0.1288
ENSG0000012	-	-	ENSG0000012682	'PLEKHG3	-	14:64704101-64879883	-	-	IGV_LINK	860.142	17.5085	0.339597	34.6775	OK	1117.85	23.8961	0.801913	46.9902	OK	OK	0.448714
ENSG0000012	-	-	ENSG0000012683	'PZP	-	12:9148839-9208370	-	-	IGV_LINK	7.75776	0.098523	0	0.494329	OK	10.3122	0.4863	0	1.58036	OK	NOTEST	2.30331
ENSG0000012	-	-	ENSG0000012685	'PRDM7	-	16:90056565-90092072	-	-	IGV_LINK	0.969721	0.02408	0	0.322736	OK	0	0	0	0	OK	NOTEST	'-inf
ENSG0000012	-	-	ENSG0000012685	'RHOT1	-	17:32141225-32253374	-	-	IGV_LINK	602.196	13.9213	1.69914	26.1435	OK	513.55	11.9707	1.46654	22.4749	OK	OK	-0.21779
ENSG0000012	-	-	ENSG0000012686	'EVI2A	-	17:31008496-31382116	-	-	IGV_LINK	528.498	18.0525	0	51.9219	OK	490.513	16.5794	0	38.1063	OK	OK	-0.12281
ENSG0000012	-	-	ENSG0000012686	'OMG	-	17:31008496-31382116	-	-	IGV_LINK	20.3641	0.410972	0	3.34822	OK	13.4059	0.296746	0	2.68561	OK	NOTEST	-0.46981
ENSG0000012	-	-	ENSG0000012687	'WDR60	-	7:158856577-158956747	-	-	IGV_LINK	1630.1	27.2099	4.04654	50.3733	OK	1049.79	16.8591	5.21712	28.5011	OK	OK	-0.6906
ENSG0000012	-	-	ENSG0000012687	'AIF1L	-	9:131096475-131123152	-	-	IGV_LINK	287.037	6.01896	0.500765	11.5371	OK	221.713	5.82743	0	11.8708	OK	OK	-0.04665
ENSG0000012	-	-	ENSG0000012688	'FAM78A	-	9:131258075-131276547	-	-	IGV_LINK	499.406	7.32724	2.81937	11.8351	OK	410.428	6.61257	1.6639	11.5612	OK	OK	-0.14806
ENSG0000012	-	-	ENSG0000012688	'NUP214	-	9:131125560-131234670	-	-	IGV_LINK	1622.34	22.0115	0	46.9116	OK	1871.67	26.3486	0	55.6825	OK	OK	0.259469
ENSG0000012	-	-	ENSG0000012689	'CTAG2	-	X:154651971-154653579	-	-	IGV_LINK	0	0	0	0	OK	0	0	0	0	OK	NOTEST	0
ENSG0000012	-	-	ENSG0000012689	'AVPR2	-	X:153861513-153935223	-	-	IGV_LINK	18.4247	0.775378	0	6.66401	OK	17.5308	0.683669	0	5.5086	OK	OK	-0.1816
ENSG0000012	-	-	ENSG0000012690	'SLC10A3	-	X:154487305-154490690	-	-	IGV_LINK	410.192	13.0276	4.39544	21.6597	OK	527.987	17.1731	6.44547	27.9008	OK	OK	0.398585
ENSG0000012	-	-	ENSG0000012693	'MAP2K2	-	19:4090320-4124129	-	-	IGV_LINK	1583.55	68.9415	20.9775	116.905	OK	1389.06	56.5191	22.8645	90.1737	OK	OK	-0.28663
ENSG0000012	-	-	ENSG0000012694	'HNRNPH2	-	X:101390823-101414133	-	-	IGV_LINK	1127.37	30.9848	0	89.2491	OK	1200.35	33.0089	0	92.2224	OK	OK	0.091293
ENSG0000012	-	-	ENSG0000012694	'ARMCX1	-	X:101550530-101554700	-	-	IGV_LINK	369.464	10.5774	3.63683	17.5179	OK	282.556	8.09401	2.44037	13.7476	OK	OK	-0.38605
ENSG0000012	-	-	ENSG0000012695	'TMEM35A	-	X:101009345-101096364	-	-	IGV_LINK	0	0	0	0	OK	0	0	0	0	OK	NOTEST	0
ENSG0000012	-	-	ENSG0000012695	'NXF5	-	X:101832111-101857577	-	-	IGV_LINK	14.5458	0.488231	0	1.14226	OK	11.3435	0.393813	0	0.905941	OK	NOTEST	-0.31005
ENSG0000012	-	-	ENSG0000012695	'TIMM8A	-	X:101345660-101349196	-	-	IGV_LINK	94.0629	3.76516	0.221454	7.30886	OK	123.747	4.91131	0.664079	9.15855	OK	OK	0.383399
ENSG0000012	-	-	ENSG0000012697	'ZC4H2	-	X:64915801-65034713	-	-	IGV_LINK	228.854	7.385	0.212273	14.5577	OK	177.371	5.10153	0.600313	9.60276	OK	OK	-0.53367
ENSG0000012	-	-	ENSG0000012702	'CANX	-	5:179678582-179730925	-	-	IGV_LINK	16075.1	378.398	0	860.635	OK	14746.5	328.879	0	713.858	OK	OK	-0.20235
ENSG0000012	-	-	ENSG0000012705	'INTS11	-	1:1292375-1324691	-	-	IGV_LINK	2103.32	86.0717	4.40247	167.741	OK	1936.64	78.6169	7.11182	150.122	OK	OK	-0.1307
ENSG0000012	-	-	ENSG0000012707	'RGS13	-	1:192636137-192660306	-	-	IGV_LINK	61.0924	2.14253	0	4.9286	OK	6.18735	0.236762	0	0.6375	OK	OK	-3.1778
ENSG0000012	-	-	ENSG0000012708	'IPPK	-	9:92297357-92670265	-	-	IGV_LINK	245.339	5.58867	0	15.2279	OK	273.275	5.3633	0	14.1992	OK	OK	-0.05938
ENSG0000012	-	-	ENSG0000012708	'ZNF484	-	9:92809387-92899608	-	-	IGV_LINK	107.639	2.13137	0	4.32518	OK	134.059	2.63864	0	5.29625	OK	OK	0.308018
ENSG0000012	-	-	ENSG0000012708	'OMD	-	9:92297357-92670265	-	-	IGV_LINK	0	0	0	0	OK	0	0	0	0	OK	NOTEST	0
ENSG0000012	-	-	ENSG0000012708	'FGD3	-	9:92947450-93036236	-	-	IGV_LINK	672.986	11.4407	2.60642	20.2749	OK	742.482	11.9876	3.04531	20.9298	OK	OK	0.06737
ENSG0000012	-	-	ENSG0000012712	'HIVEP3	-	1:41506364-42035925	-	-	IGV_LINK	391.767	3.76765	0	8.09346	OK	391.865	2.92635	0	6.06535	OK	OK	-0.36456
ENSG0000012	-	-	ENSG0000012712	'PPCS	-	1:42430328-42654664	-	-	IGV_LINK	814.892	50.5503	14.2192	86.8814	OK	1107.88	67.2126	22.4757	111.95	OK	OK	0.411013
ENSG0000012	-	-	ENSG0000012712	'EDN2	-	1:41478774-41484673	-	-	IGV_LINK	27.1522	1.5693	0	3.48851	OK	16.4996	1.26906	0	3.37995	OK	OK	-0.30636
ENSG0000012	-	-	ENSG0000012715	'BCL11B	-	14:99169286-99271524	-	-	IGV_LINK	234.672	1.81977	0.434177	3.20536	OK	123.747	0.941999	0.119904	1.76409	OK	OK	-0.94996
ENSG0000012	-	-	ENSG0000012718	'COX7C	-	5:86617903-86620962	-	-	IGV_LINK	3729.55	506.791	173.591	839.991	OK	3275.17	443.823	138.135	749.511	OK	OK	-0.19141
ENSG0000012	-	-	ENSG0000012719	'TRAF2	-	9:136881911-136926607	-	-	IGV_LINK	645.834	19.0719	6.34859	31.7951	OK	476.426	13.1032	4.67032	21.5362	OK	OK	-0.54152
ENSG0000012	-	-	ENSG0000012722	'ABHD8	-	19:17292130-17334829	-	-	IGV_LINK	219.157	6.44122	0	19.7046	OK	204.183	6.00461	0	17.5014	OK	OK	-0.10126
ENSG0000012	-	-	ENSG0000012724	'MASP1	-	3:187217284-187297933	-	-	IGV_LINK	1388.64	22.4085	2.3473	42.4697	OK	1256.03	22.314	1.7858	42.8421	OK	OK	-0.0061
ENSG0000012	-	-	ENSG0000012724	'ATP13A4	-	3:193402076-193593111	-	-	IGV_LINK	57.2135	1.20001	0	2.68166	OK	28.8743	0.70293	0	1.78588	OK	OK	-0.77159
ENSG0000012	-	-	ENSG0000012725	'HRASLS	-	3:193241124-193378843	-	-	IGV_LINK	6.78804	0.475324	0	1.29684	OK	29.9055	2.02546	0	4.29337	OK	OK	2.09127
ENSG0000012	-	-	ENSG0000012731	'HELB	-	12:66302544-66804186	-	-	IGV_LINK	262.794	1.3308	0.14535	2.51626	OK	230.994	1.20612	0	2.84121	OK	OK	-0.14193
ENSG0000012	-	-	ENSG0000012731	'RAP1B	-	12:68610838-68671901	-	-	IGV_LINK	1729.01	89.6001	29.5614	149.639	OK	2482.16	140.401	48.2055	232.596	OK	OK	0.647977
ENSG0000012	-	-	ENSG0000012731	'IL22	-	12:68248241-68253607	-	-	IGV_LINK	0	0	0	0	OK	0	0	0	0	OK	NOTEST	0
ENSG0000012	-	-	ENSG0000012732	'TSPAN8	-	12:71125084-71586310	-	-	IGV_LINK	0.969721	0.054893	0	0.680594	OK	3.09367	0.209569	0	0.94636	OK	NOTEST	1.93272
ENSG0000012	-	-	ENSG0000012732	'BEST3	-	12:69643359-69699476	-	-	IGV_LINK	19.3944	0.422922	0	1.17886	OK	36.0929	1.68868	0	4.83167	OK	OK	1.99743
ENSG0000012	-	-	ENSG0000012732	'RAB3IP	-	12:69738680-70354993	-	-	IGV_LINK	1487.55	39.5313	0	95.8591	OK	149.528	4.97346	0	17.7843	OK	OK	-2.99068
ENSG0000012	-	-	ENSG0000012732	'PTPRB	-	12:70468079-70637440	-	-	IGV_LINK	1059.9	7.78556	1.29858	14.2725	OK	1450.93	9.85316	3.27133	16.435	OK	OK	0.339785
ENSG0000012	-	-	ENSG0000012733	'DYRK2	-	12:67648337-67670919	-	-	IGV_LINK	682.683	9.46678	0.857282	18.0763	OK	551.705	7.33932	0.755368	13.9233	OK	OK	-0.36723
ENSG0000012	-	-	ENSG0000012733	'YEATS4	-	12:69359702-69390796	-	-	IGV_LINK	109.578	5.80411	0	11.6823	OK	102.091	5.09965	0.322041	9.87725	OK	OK	-0.18668
ENSG0000012	-	-	ENSG0000012736	'TAS2R3	-	7:141704337-141788640	-	-	IGV_LINK	0	0	0	0	OK	0	0	0	0	OK	NOTEST	0
ENSG0000012	-	-	ENSG0000012736	'TAS2R4	-	7:141704337-141788640	-	-	IGV_LINK	34.9099	0.892036	0	3.54409	OK	25.7806	0.659103	0	3.03274	OK	OK	-0.4366
ENSG0000012	-	-	ENSG0000012736	'TAS2R5	-	7:141790216-141791367	-	-	IGV_LINK	53.3346	3.07182	0.039444	6.1042	OK	39.1865	2.25962	0	4.57764	OK	OK	-0.44302
ENSG0000012	-	-	ENSG0000012737	'CRYGN	-	7:151427682-151440813	-	-	IGV_LINK	0	0	0	0	OK	0	0	0	0	OK	NOTEST	0
ENSG0000012	-	-	ENSG0000012739	'LRRC61	-	7:150243915-150338150	-	-	IGV_LINK	502.315	19.4483	1.04065	37.8559	OK	806.418	33.138	3.06742	63.2087	OK	OK	0.768847
ENSG0000012	-	-	ENSG0000012741	'TRPV5	-	7:142908100-142933808	-	-	IGV_LINK	0.969721	0.028061	0	0.134746	OK	0	0	0	0	OK	NOTEST	'-inf
ENSG0000012	-	-	ENSG0000012741	'IDUA	-	4:958886-1004506	-	-	IGV_LINK	444.132	18.765	0	48.5647	OK	355.773	14.0814	0	31.4605	OK	OK	-0.41425
ENSG0000012	-	-	ENSG0000012741	'FGFRL1	-	4:1009935-1026897	-	-	IGV_LINK	534.316	10.1629	3.75537	16.5705	OK	732.17	15.2758	1.20284	29.3487	OK	OK	0.587929
ENSG0000012	-	-	ENSG0000012741	'TMEM175	-	4:932386-958656	-	-	IGV_LINK	445.102	19.4236	3.37292	35.4742	OK	457.864	20.7797	4.88479	36.6747	OK	OK	0.097369
ENSG0000012	-	-	ENSG0000012742	'AUNIP	-	1:25800175-25859458	-	-	IGV_LINK	30.0486	1.31007	0	7.97494	OK	35.0941	1.51256	0	9.13115	OK	OK	0.207345
ENSG0000012	-	-	ENSG0000012744	'PIN1	-	19:9835256-9849682	-	-	IGV_LINK	802.929	48.8867	16.6	81.1735	OK	842.511	53.7363	20.6162	86.8565	OK	OK	0.136455
ENSG0000012	-	-	ENSG0000012745	'FBXL12	-	19:9810266-9827816	-	-	IGV_LINK	306.432	12.5298	2.76137	22.2983	OK	376.397	15.5518	3.56534	27.5383	OK	OK	0.311713
ENSG0000012	-	-	ENSG0000012746	'EMC1	-	1:19210500-19260128	-	-	IGV_LINK	1017.24	19.7865	0	44.6468	OK	1373.59	21.3468	0	44.5648	OK	OK	0.109509
ENSG0000012	-	-	ENSG0000012747	'PLA2G5	-	1:20028178-20091190	-	-	IGV_LINK	0	0	0	0	OK	12.3747	0.605431	0	1.45751	OK	OK	inf
ENSG0000012	-	-	ENSG0000012748	'UBR4	-	1:19072109-19210276	-	-	IGV_LINK	5664.42	145.497	0	298.659	OK	5829.84	202.126	0	405.187	OK	OK	0.474264
ENSG0000012	-	-	ENSG0000012748	'HP1BP3	-	1:20742660-20787323	-	-	IGV_LINK	4593.57	165.806	23.7051	307.906	OK	3350.45	113.428	15.3791	211.478	OK	OK	-0.54771
ENSG0000012	-	-	ENSG0000012750	'ADGRE2	-	19:14619116-14778541	-	-	IGV_LINK	815.535	10.4484	0.031171	20.8657	OK	1032.26	15.0788	0	30.5362	OK	OK	0.529231
ENSG0000012	-	-	ENSG0000012751	'SIN3B	-	19:16829399-16880353	-	-	IGV_LINK	1919.08	37.7967	3.8813	71.7122	OK	2056.26	40.5205	4.26504	76.776	OK	OK	0.100391
ENSG0000012	-	-	ENSG0000012751	'OR7A10	-	19:14840465-14848922	-	-	IGV_LINK	0	0	0	0	OK	0	0	0	0	OK	NOTEST	0
ENSG0000012	-	-	ENSG0000012752	'SLC35E1	-	19:16479056-16660442	-	-	IGV_LINK	1401.25	20.3136	0	49.0914	OK	1778.85	27.2056	0	65.3943	OK	OK	0.421459
ENSG0000012	-	-	ENSG0000012752	'EPS15L1	-	19:16355238-16472085	-	-	IGV_LINK	596.378	14.2131	1.70282	26.7234	OK	573.361	13.1041	1.90574	24.3025	OK	OK	-0.1172
ENSG0000012	-	-	ENSG0000012752	'KLF2	-	19:16324816-16327874	-	-	IGV_LINK	159.034	5.85101	0.18633	11.5157	OK	275.337	8.98796	2.20065	15.7753	OK	OK	0.619309
ENSG0000012	-	-	ENSG0000012752	'OR7C2	-	19:14941488-14942448	-	-	IGV_LINK	0	0	0	0	OK	0	0	0	0	OK	NOTEST	0
ENSG0000012	-	-	ENSG0000012753	'OR7C1	-	19:14789259-14835376	-	-	IGV_LINK	14.5458	0.245984	0	0.655437	OK	5.15612	0.079787	0	0.268757	OK	NOTEST	-1.62434
ENSG0000012	-	-	ENSG0000012753	'F2RL3	-	19:16888859-16892606	-	-	IGV_LINK	133.821	2.59225	0	5.31791	OK	62.9047	1.41846	0	3.44408	OK	OK	-0.86988
ENSG0000012	-	-	ENSG0000012754	'UQCR11	-	19:1573595-1605490	-	-	IGV_LINK	1153.29	124.238	0	284.551	OK	1346.17	149.291	4.65857	293.923	OK	OK	0.265019
ENSG0000012	-	-	ENSG0000012755	'GFER	-	16:1984206-1994275	-	-	IGV_LINK	256.006	18.4988	0	41.426	OK	312.461	23.0887	0.499636	45.6778	OK	OK	0.319754
ENSG0000012	-	-	ENSG0000012756	'SYNGR3	-	16:1984206-1994275	-	-	IGV_LINK	10.6669	0.336664	0	1.63919	OK	11.3435	0.384128	0	1.9174	OK	NOTEST	0.190279
ENSG0000012	-	-	ENSG0000012756	'PKMYT1	-	16:2963943-2980539	-	-	IGV_LINK	115.397	9.71187	0	20.4002	OK	116.528	10.1484	0	21.7167	OK	OK	0.063433

ENSG0000012	-	-	ENSG0000012757	'WFIKKN1	-	16:589356-634116	-	-	IGV_LINK	32.9705	0.394873	0	1.35541	OK	23.7182	0.349938	0	1.60438	OK	NOTEST	-0.17429
ENSG0000012	-	-	ENSG0000012758	'WDR24	-	16:684621-705829	-	-	IGV_LINK	134.791	2.55508	0	5.19451	OK	119.622	2.77032	0	6.22556	OK	OK	0.116685
ENSG0000012	-	-	ENSG0000012758	'FBXL16	-	16:684621-705829	-	-	IGV_LINK	24.243	0.463977	0	1.73435	OK	20.6245	0.451795	0	1.73266	OK	NOTEST	-0.03839
ENSG0000012	-	-	ENSG0000012758	'CHTF18	-	16:784973-800737	-	-	IGV_LINK	362.675	7.7836	0	16.9251	OK	306.274	10.4129	0	23.7119	OK	OK	0.419862
ENSG0000012	-	-	ENSG0000012758	'GNG13	-	16:784973-800737	-	-	IGV_LINK	0	0	0	0	OK	0	0	0	0	OK	NOTEST	0
ENSG0000012	-	-	ENSG0000012758	'TUBBP1	-	8:30351872-30353518	-	-	IGV_LINK	5.81832	0.282064	0	0.68023	OK	17.5308	0.704455	0	1.49929	OK	OK	1.32049
ENSG0000012	-	-	ENSG0000012760	'MACF1	-	1:39081315-39487177	-	-	IGV_LINK	4861.21	59.2318	0	123.297	OK	3343.23	43.5834	3.86631	83.3005	OK	OK	-0.44259
ENSG0000012	-	-	ENSG0000012761	'SMARCA4	-	19:10961000-11065395	-	-	IGV_LINK	1675.68	32.8074	0.969782	64.6451	OK	1687.08	32.5008	0.984542	64.0171	OK	OK	-0.01355
ENSG0000012	-	-	ENSG0000012766	'KDM4B	-	19:4969112-5153595	-	-	IGV_LINK	1008.51	12.5061	1.85682	23.1554	OK	1295.22	15.9213	2.50283	29.3399	OK	OK	0.348327
ENSG0000012	-	-	ENSG0000012766	'TICAM1	-	19:4815931-4831704	-	-	IGV_LINK	595.408	13.4033	5.21707	21.5895	OK	921.915	20.7629	8.51234	33.0134	OK	OK	0.631418
ENSG0000012	-	-	ENSG0000012772	'METTL25	-	12:82223680-82479236	-	-	IGV_LINK	94.0629	3.77912	0	10.5607	OK	70.1233	4.06355	0	11.0909	OK	OK	0.104689
ENSG0000012	-	-	ENSG0000012774	'IL17B	-	5:149371323-149404202	-	-	IGV_LINK	6.78804	0.838041	0	2.65053	OK	3.09367	0.382896	0	1.12444	OK	OK	-1.13007
ENSG0000012	-	-	ENSG0000012777	'EMC6	-	17:3662895-3696404	-	-	IGV_LINK	181.338	20.126	0	52.9774	OK	216.557	24.5531	0	68.284	OK	OK	0.286845
ENSG0000012	-	-	ENSG0000012778	'OR1E2	-	17:3432869-3433841	-	-	IGV_LINK	0	0	0	0	OK	0	0	0	0	OK	NOTEST	0
ENSG0000012	-	-	ENSG0000012780	'METTL16	-	17:2405561-2511891	-	-	IGV_LINK	408.252	5.43091	0.165299	10.6965	OK	406.303	4.54633	0.792928	8.29972	OK	OK	-0.25649
ENSG0000012	-	-	ENSG0000012782	'TUBA4A	-	2:219229756-219278170	-	-	IGV_LINK	815.535	39.7766	0	109.994	OK	1070.41	51.4174	0	145.879	OK	OK	0.370337
ENSG0000012	-	-	ENSG0000012783	'VIL1	-	2:218419091-218568361	-	-	IGV_LINK	37.8191	1.20333	0	4.72998	OK	3.09367	0.064789	0	0.626887	OK	OK	-4.21515
ENSG0000012	-	-	ENSG0000012783	'AAMP	-	2:218264122-218270257	-	-	IGV_LINK	2017.99	69.1067	28.8622	109.351	OK	2214.04	76.3547	31.4434	121.266	OK	OK	0.143892
ENSG0000012	-	-	ENSG0000012783	'PNKD	-	2:218270391-218368099	-	-	IGV_LINK	836.869	45.079	0	118.656	OK	1517.96	57.6505	0	163.965	OK	OK	0.354877
ENSG0000012	-	-	ENSG0000012786	'TNFRSF19	-	13:23570369-23676104	-	-	IGV_LINK	278.31	4.10089	1.0519	7.14989	OK	261.931	3.76302	1.00212	6.52393	OK	OK	-0.12405
ENSG0000012	-	-	ENSG0000012787	'RNF6	-	13:26094723-26222654	-	-	IGV_LINK	583.772	14.0674	0	30.1366	OK	665.14	14.9478	0	30.4935	OK	OK	0.087573
ENSG0000012	-	-	ENSG0000012788	'ECHS1	-	10:133362479-133373689	-	-	IGV_LINK	1701.86	66.3972	29.1948	103.6	OK	1050.82	41.0298	17.8432	64.2164	OK	OK	-0.69445
ENSG0000012	-	-	ENSG0000012790	'ZNF835	-	19:56661980-56671783	-	-	IGV_LINK	34.9099	0.699608	0	1.78629	OK	38.1553	1.33643	0	3.74093	OK	OK	0.933769
ENSG0000012	-	-	ENSG0000012791	'AKAP9	-	7:91940866-92110673	-	-	IGV_LINK	1664.04	20.5045	3.29212	37.7169	OK	1120.94	17.3337	2.82257	31.8449	OK	OK	-0.24236
ENSG0000012	-	-	ENSG0000012792	'GNG11	-	7:93921698-93928610	-	-	IGV_LINK	905.719	17.7077	7.53564	27.8798	OK	1055.97	20.6536	8.98594	32.3213	OK	OK	0.222015
ENSG0000012	-	-	ENSG0000012792	'SEM1	-	7:96481625-96709891	-	-	IGV_LINK	881.476	117.913	25.8949	209.932	OK	1165.28	179.992	46.8439	313.139	OK	OK	0.610203
ENSG0000012	-	-	ENSG0000012792	'GNGT1	-	7:93591572-93911265	-	-	IGV_LINK	0	0	0	0	OK	0	0	0	0	OK	NOTEST	0
ENSG0000012	-	-	ENSG0000012794	'HIP1	-	7:75533299-75738962	-	-	IGV_LINK	1227.67	10.7447	0	22.1743	OK	1152.91	10.1243	0	21.1082	OK	OK	-0.0858
ENSG0000012	-	-	ENSG0000012794	'PTPN12	-	7:77537274-77640071	-	-	IGV_LINK	1669.86	45.0746	11.3464	78.8028	OK	1546.84	41.3967	10.6606	72.1328	OK	OK	-0.1228
ENSG0000012	-	-	ENSG0000012794	'POR	-	7:75899199-75994659	-	-	IGV_LINK	798.08	23.9638	0.032363	47.8953	OK	2046.98	59.7866	7.84722	111.726	OK	OK	1.31896
ENSG0000012	-	-	ENSG0000012795	'FGL2	-	7:76959834-77416400	-	-	IGV_LINK	2909.16	54.6998	0	134.794	OK	2030.48	40.1379	0	85.9928	OK	OK	-0.44657
ENSG0000012	-	-	ENSG0000012795	'STYXL1	-	7:75996337-76048004	-	-	IGV_LINK	339.402	27.9681	7.38546	48.5507	OK	298.024	24.0123	4.29028	43.7344	OK	OK	-0.22001
ENSG0000012	-	-	ENSG0000012795	'STEAP4	-	7:88205117-88306891	-	-	IGV_LINK	75.6382	0.722359	0	2.73784	OK	302.149	3.03606	0	8.59842	OK	OK	2.07141
ENSG0000012	-	-	ENSG0000012795	'GNAI1	-	7:80133954-80219402	-	-	IGV_LINK	335.523	11.3697	0.85107	21.8882	OK	346.492	13.8111	0.617997	27.0041	OK	OK	0.280637
ENSG0000012	-	-	ENSG0000012795	'PMS2P3	-	7:75493624-75528148	-	-	IGV_LINK	176.489	13.2974	1.4619	25.1329	OK	176.339	13.2232	1.21282	25.2336	OK	OK	-0.00807
ENSG0000012	-	-	ENSG0000012798	'PEX1	-	7:92412549-92528531	-	-	IGV_LINK	623.53	14.3406	0	37.6932	OK	567.174	9.71348	0	23.9192	OK	OK	-0.56204
ENSG0000012	-	-	ENSG0000012798	'MTERF1	-	7:91692007-91880720	-	-	IGV_LINK	125.094	4.43594	0	10.0346	OK	138.184	5.0852	0	10.3178	OK	OK	0.197062
ENSG0000012	-	-	ENSG0000012799	'SGCE	-	7:94585229-94656209	-	-	IGV_LINK	639.046	26.4516	7.07947	45.8238	OK	222.745	8.63953	2.1666	15.1125	OK	OK	-1.61433
ENSG0000012	-	-	ENSG0000012799	'RBM48	-	7:92528772-92538005	-	-	IGV_LINK	210.429	6.20445	1.20817	11.2007	OK	197.995	6.49748	0.991799	12.0032	OK	OK	0.066577
ENSG0000012	-	-	ENSG0000012799	'CASD1	-	7:94509218-94557019	-	-	IGV_LINK	299.644	5.12834	1.41064	8.84604	OK	309.367	6.24106	0	14.3797	OK	OK	0.2833
ENSG0000012	-	-	ENSG0000012800	'ZNF780B	-	19:40028259-40094406	-	-	IGV_LINK	363.645	5.06915	0	15.085	OK	238.213	4.17935	0	14.0255	OK	OK	-0.27846
ENSG0000012	-	-	ENSG0000012801	'LRFN1	-	19:39306567-39320858	-	-	IGV_LINK	47.1781	0.828662	0	1.69272	OK	44.1381	0.775543	0	1.58837	OK	OK	-0.09558
ENSG0000012	-	-	ENSG0000012801	'ZFP36	-	19:39406812-39409412	-	-	IGV_LINK	2512.55	98.4048	37.8043	159.005	OK	3969.18	158.554	57.2761	259.832	OK	OK	0.688172
ENSG0000012	-	-	ENSG0000012803	'SRD5A3	-	4:55346108-55395847	-	-	IGV_LINK	265.703	9.49242	0	19.3112	OK	395.99	14.8262	0	30.8016	OK	OK	0.643305
ENSG0000012	-	-	ENSG0000012804	'SPINK2	-	4:56809859-56821742	-	-	IGV_LINK	9.69721	1.26779	0	3.15358	OK	76.3106	10.0178	0.285671	19.75	OK	OK	2.98218
ENSG0000012	-	-	ENSG0000012804	'RASL11B	-	4:52862289-52866835	-	-	IGV_LINK	2.90916	0.105061	0	0.358347	OK	0	0	0	0	OK	NOTEST	'-inf
ENSG0000012	-	-	ENSG0000012805	'PAICS	-	4:56435740-56464579	-	-	IGV_LINK	1330.46	33.1301	11.0877	55.1725	OK	2072.76	53.8646	19.5379	88.1913	OK	OK	0.701195
ENSG0000012	-	-	ENSG0000012805	'KDR	-	4:55053059-55125589	-	-	IGV_LINK	2084.9	21.2057	8.2479	34.1635	OK	2508.97	25.1358	10.613	39.6586	OK	OK	0.245291
ENSG0000012	-	-	ENSG0000012805	'PPAT	-	4:56393361-56435615	-	-	IGV_LINK	207.52	3.55883	0.321907	6.79576	OK	235.119	4.07728	0.381805	7.77276	OK	OK	0.196204
ENSG0000012	-	-	ENSG0000012815	'TUBGCP6	-	22:50217688-50244992	-	-	IGV_LINK	2431.09	32.3558	9.74932	54.9623	OK	2172.79	29.7139	8.17466	51.2531	OK	OK	-0.12289
ENSG0000012	-	-	ENSG0000012816	'ADM2	-	22:50481555-50486440	-	-	IGV_LINK	486.8	7.20313	1.41986	12.9864	OK	491.894	6.7574	2.58065	10.9342	OK	OK	-0.09215
ENSG0000012	-	-	ENSG0000012818	'DGCR6L	-	22:20314275-20320080	-	-	IGV_LINK	769.958	44.4297	17.5494	71.31	OK	601.204	34.6579	13.5207	55.7952	OK	OK	-0.35834
ENSG0000012	-	-	ENSG0000012819	'DGCR8	-	22:20080231-20127357	-	-	IGV_LINK	655.531	13.7062	0	38.5225	OK	653.797	16.3147	0	46.3304	OK	OK	0.251344
ENSG0000012	-	-	ENSG0000012820	'ASPHD2	-	22:26429272-26483837	-	-	IGV_LINK	90.184	1.59598	0	5.58544	OK	65.9984	1.16839	0	4.80063	OK	OK	-0.44992
ENSG0000012	-	-	ENSG0000012821	'VPREB3	-	22:23752742-23754468	-	-	IGV_LINK	1.93944	0.249841	0	0.870626	OK	6.18735	0.799161	0	1.9545	OK	OK	1.67747
ENSG0000012	-	-	ENSG0000012822	'SDF2L1	-	22:21642260-21644298	-	-	IGV_LINK	134.791	11.1149	2.03959	20.1902	OK	118.591	9.66732	1.54544	17.7892	OK	OK	-0.20131
ENSG0000012	-	-	ENSG0000012824	'GAL3ST1	-	22:30554634-30574587	-	-	IGV_LINK	1172.39	54.4515	20.5683	88.3347	OK	3282.39	159.097	56.1247	262.068	OK	OK	1.54686
ENSG0000012	-	-	ENSG0000012824	'YWHAH	-	22:31933520-31957603	-	-	IGV_LINK	2658.97	112.91	31.6928	194.127	OK	2241.88	95.5339	27.7086	163.359	OK	OK	-0.24109
ENSG0000012	-	-	ENSG0000012825	'RFPL1	-	22:29436533-29478175	-	-	IGV_LINK	1.93944	0.082584	0	0.323461	OK	9.28102	0.395543	0	1.21862	OK	NOTEST	2.2599
ENSG0000012	-	-	ENSG0000012825	'RFPL2	-	22:32190434-32203477	-	-	IGV_LINK	2.90916	0.16787	0	0.514582	OK	2.06245	0.119143	0	0.426943	OK	NOTEST	-0.49465
ENSG0000012	-	-	ENSG0000012825	'C22orf24	-	22:31933520-31957603	-	-	IGV_LINK	3.87888	0.336057	0	5.29605	OK	3.09367	0.348902	0	5.97206	OK	NOTEST	0.054116
ENSG0000012	-	-	ENSG0000012826	'POM121L9P	-	22:24251827-24265525	-	-	IGV_LINK	21.3339	0.248124	0	0.594748	OK	25.7806	0.470832	0	1.31033	OK	NOTEST	0.924149
ENSG0000012	-	-	ENSG0000012826	'GNAZ	-	22:23059414-23145021	-	-	IGV_LINK	60.1227	1.07209	0	2.58763	OK	81.4668	1.45319	0	3.19525	OK	OK	0.438801
ENSG0000012	-	-	ENSG0000012826	'MGAT3	-	22:39457343-39492194	-	-	IGV_LINK	275.401	3.31855	0.529803	6.10729	OK	804.355	9.64519	2.90522	16.3852	OK	OK	1.53926
ENSG0000012	-	-	ENSG0000012827	'ADORA2A	-	22:24270816-24528390	-	-	IGV_LINK	315.894	8.25375	0	22.7143	OK	275.715	7.06737	0	21.6741	OK	OK	-0.22388
ENSG0000012	-	-	ENSG0000012827	'ATF4	-	22:39519694-39522685	-	-	IGV_LINK	5115.28	227.604	91.7659	363.442	OK	4615.76	204.072	83.996	324.148	OK	OK	-0.15745
ENSG0000012	-	-	ENSG0000012827	'A4GALT	-	22:42692120-42721298	-	-	IGV_LINK	587.651	19.2254	6.34725	32.1035	OK	1063.19	34.1942	13.1078	55.2806	OK	OK	0.830741
ENSG0000012	-	-	ENSG0000012827	'RFPL3	-	22:32354884-32385631	-	-	IGV_LINK	0	0	0	0	OK	1.03122	0.055863	0	0.391599	OK	NOTEST	inf
ENSG0000012	-	-	ENSG0000012828	'CDC42EP1	-	22:37560446-37569405	-	-	IGV_LINK	660.38	19.0829	7.2165	30.9493	OK	616.673	17.313	6.74441	27.8816	OK	OK	-0.14042
ENSG0000012	-	-	ENSG0000012828	'APOL3	-	22:36140329-36166177	-	-	IGV_LINK	1942.35	67.7619	13.9618	121.562	OK	1264.28	48.1056	11.1068	85.1044	OK	OK	-0.49427
ENSG0000012	-	-	ENSG0000012828	'MCHR1	-	22:40678749-40682814	-	-	IGV_LINK	112.488	3.44796	0.124519	6.77139	OK	7.21857	0.42497	0	1.12532	OK	OK	-3.02031
ENSG0000012	-	-	ENSG0000012829	'TPST2	-	22:26521982-26596717	-	-	IGV_LINK	404.373	12.3126	2.41265	22.2125	OK	1159.1	33.3895	11.9835	54.7954	OK	OK	1.43926
ENSG0000012	-	-	ENSG0000012829	'BAIAP2L2	-	22:38084888-38110670	-	-	IGV_LINK	452.859	13.418	4.25619	22.5798	OK	548.612	16.6019	5.37737	27.8264	OK	OK	0.307178
ENSG0000012	-	-	ENSG0000012830	'MPST	-	22:37010858-37029822	-	-	IGV_LINK	747.655	27.101	6.11906	48.083	OK	1094.13	38.4551	7.09253	69.8176	OK	OK	0.504825
ENSG0000012	-	-	ENSG0000012831	'GALR3	-	22:37823381-37825495	-	-	IGV_LINK	0	0	0	0	OK	0	0	0	0	OK	NOTEST	0
ENSG0000012	-	-	ENSG0000012831	'TST	-	22:37010858-37029822	-	-	IGV_LINK	272.491	15.4234	0	35.3225	OK	713.608	40.5875	4.34913	76.8258	OK	OK	1.39591
ENSG0000012	-	-	ENSG0000012831	'APOL5	-	22:35717871-35729483	-	-	IGV_LINK	0	0	0	0	OK	8.2498	0.367723	0	0.839445	OK	NOTEST	inf
ENSG0000012	-	-	ENSG0000012832	'IGLL1	-	22:23567063-23580308	-	-	IGV_LINK	2.90916	0.301663	0	0.863101	OK	0	0	0	0	OK	NOTEST	'-inf
ENSG0000012	-	-	ENSG0000012833	'APOL2	-	22:36226202-36239954	-	-	IGV_LINK	1482.7	50.9568	14.967	86.9466	OK	2557.44	92.3297	27.04	157.619	OK	OK	0.85752
ENSG0000012	-	-	ENSG0000012834	'RAC2	-	22:37225260-37244448	-	-	IGV_LINK	1591.31	71.6236	28.3203	114.927	OK	1411.75	64.4738	26.7306	102.217	OK	OK	-0.15172
ENSG0000012	-	-	ENSG0000012834	'LIF	-	22:30239193-30246998	-	-	IGV_LINK	336.493	7.60197	0	15.9125	OK	1151.88	23.004	2.37837	43.6297	OK	OK	1.59744
ENSG0000012	-	-	ENSG0000012834	'C22orf23	-	22:37943049-38041915	-	-	IGV_LINK	45.5769	1.62701	0	5.12157	OK	35.0616	1.52792	0	6.02098	OK	OK	-0.09065
ENSG0000012	-	-	ENSG0000012838	'APOBEC3A	-	22:38952740-38998209	-	-	IGV_LINK	23.2733	2.13032	0	7.22093	OK	101.06	7.42158	0	17.3387	OK	OK	1.80066
ENSG0000012	-	-	ENSG0000012839	'APOBEC3F	-	22:39040603-39053910	-	-	IGV_LINK	173.58	6.4704	0	13.395	OK	151.59	5.31912	0	11.1567	OK	OK	-0.28267
ENSG0000012	-	-	ENSG0000012840	'RIBC2	-	22:45413690-45432496	-	-	IGV_LINK	26.1825	1.98509	0	4.58318	OK	29.9055	2.06082	0	4.57397	OK	OK	0.054012
ENSG0000012	-	-	ENSG0000012842	'KRT17	-	17:41619436-41624842	-	-	IGV_LINK	0	0	0	0	OK	2.06245	0.091779	0	0.496039	OK	NOTEST	inf
ENSG0000012	-	-	ENSG0000012843	'TBC1D27	-	17:16922914-16972118	-	-	IGV_LINK	21.3339	0.315829	0	0.86561	OK	32.9992	0.541841	0	1.45953	OK	NOTEST	0.778723
ENSG0000012	-	-	ENSG0000012846	'EMC4	-	15:34224998-34338060	-	-	IGV_LINK	940.629	62.3053	11.7004	112.91	OK	1030.19	70.971	10.7672	131.175	OK	OK	0.187875
ENSG0000012	-	-	ENSG0000012848	'RNF112	-	17:19411124-19417276	-	-	IGV_LINK	18.4247	0.341619	0	1.0323	OK	22.6869	0.420806	0	1.18781	OK	NOTEST	0.300767
ENSG0000012	-	-	ENSG0000012848	'SPECC1	-	17:20009343-20403557	-	-	IGV_LINK	308.371	6.29453	0	13.532	OK	338.242	7.40834	0	16.0313	OK	OK	0.235051
ENSG0000012	-	-	ENSG0000012851	'CPA4	-	7:130293133-130324180	-	-	IGV_LINK	0.969721	0.064031	0	0.632657	OK	23.7182	0.580035	0	1.62644	OK	OK	3.17929
ENSG0000012	-	-	ENSG0000012851	'DOCK4	-	7:111726109-112206411	-	-	IGV_LINK	1296.52	16.7754	0.948215	32.6025	OK	951.821	14.162	0.092449	28.2316	OK	OK	-0.24432
ENSG0000012	-	-	ENSG0000012851	'POT1	-	7:124822385-125379321	-	-	IGV_LINK	461.587	14.7606	1.39077	28.1305	OK	447.552	16.0447	0.703057	31.3864	OK	OK	0.120348
ENSG0000012	-	-	ENSG0000012851	'TAS2R16	-	7:122994703-122995700	-	-	IGV_LINK	0	0	0	0	OK	0	0	0	0	OK	NOTEST	0
ENSG0000012	-	-	ENSG0000012852	'ATP6V1F	-	7:128830376-128910719	-	-	IGV_LINK	1459.43	154.725	44.078	265.372	OK	1653.05	175.641	17.8141	333.469	OK	OK	0.182926
ENSG0000012	-	-	ENSG0000012853	'LSM8	-	7:118184031-118192824	-	-	IGV_LINK	405.343	19.6794	0	42.1468	OK	411.459	19.7376	0	42.7924	OK	OK	0.00426
ENSG0000012	-	-	ENSG0000012853	'CDHR3	-	7:105876795-106033773	-	-	IGV_LINK	120.245	7.33417	0	15.3287	OK	133.028	7.59079	0.126821	15.0548	OK	OK	0.049616
ENSG0000012	-	-	ENSG0000012856	'PRKRIP1	-	7:102363871-102426676	-	-	IGV_LINK	969.646	24.9502	8.3312	41.5693	OK	1060.86	28.7871	9.83246	47.7418	OK	OK	0.206371
ENSG0000012	-	-	ENSG0000012856	'VGF	-	7:101162508-101165593	-	-	IGV_LINK	0.969721	0.023709	0	0.132523	OK	7.21857	0.200457	0	0.498135	OK	NOTEST	3.07982
ENSG0000012	-	-	ENSG0000012856	'PODXL	-	7:131500261-131558217	-	-	IGV_LINK	2663.82	28.6953	9.75062	47.64	OK	3198.86	36.8391	10.7578	62.9204	OK	OK	0.360424
ENSG0000012	-	-	ENSG0000012857	'FOXP2	-	7:114086326-114693772	-	-	IGV_LINK	310.311	6.12595	0	14.7028	OK	217.588	3.58918	0	7.26058	OK	OK	-0.77128
ENSG0000012	-	-	ENSG0000012857	'STRIP2	-	7:129434432-129488399	-	-	IGV_LINK	67.8804	1.21128	0	3.34576	OK	423.833	6.45126	0	13.0465	OK	OK	2.41305
ENSG0000012	-	-	ENSG0000012858	'IFT22	-	7:101313366-101321823	-	-	IGV_LINK	448.981	27.1533	9.2538	45.0528	OK	608.423	35.9155	14.1536	57.6774	OK	OK	0.40348
ENSG0000012	-	-	ENSG0000012858	'MKLN1	-	7:130941759-131497694	-	-	IGV_LINK	716.623	9.38372	0	21.3769	OK	648.641	10.8114	0	26.3367	OK	OK	0.204328
ENSG0000012	-	-	ENSG0000012859	'DNAJB9	-	7:108470421-108574850	-	-	IGV_LINK	582.802	15.099	0	30.7474	OK	632.141	16.6838	0	33.6936	OK	OK	0.144
ENSG0000012	-	-	ENSG0000012859	'FLNC	-	7:128830376-128910719	-	-	IGV_LINK	12.6064	0.080095	0	0.727048	OK	74.2482	0.465643	0	2.30991	OK	NOTEST	2.53943
ENSG0000012	-	-	ENSG0000012859	'LRRC4	-	7:127652179-128092609	-	-	IGV_LINK	32.0008	0.493346	0	3.33533	OK	72.1857	1.37129	0	10.3296	OK	OK	1.47486
ENSG0000012	-	-	ENSG0000012859	'CALU	-	7:128739291-128771807	-	-	IGV_LINK	1585.49	35.1317	7.97938	62.284	OK	2452.25	53.138	14.8296	91.4463	OK	OK	0.596971
ENSG0000012	-	-	ENSG0000012859	'CCDC136	-	7:128790756-128822132	-	-	IGV_LINK	64.9713	2.21137	0	5.66408	OK	47.4363	2.34267	0	5.70455	OK	OK	0.083214
ENSG0000012	-	-	ENSG0000012860	'SMO	-	7:129188871-129213545	-	-	IGV_LINK	713.714	12.3433	3.43177	21.2548	OK	586.767	9.7509	3.13882	16.363	OK	OK	-0.34012
ENSG0000012	-	-	ENSG0000012860	'IRF5	-	7:128937611-128950035	-	-	IGV_LINK	896.022	32.0618	9.64399	54.4797	OK	799.199	28.2915	8.56254	48.0204	OK	OK	-0.18049
ENSG0000012	-	-	ENSG0000012860	'LRRC17	-	7:102813229-103074843	-	-	IGV_LINK	66.9107	2.94833	0	6.93663	OK	37.1241	1.29627	0	3.34283	OK	OK	-1.18553
ENSG0000012	-	-	ENSG0000012860	'KLHDC10	-	7:130070509-130135720	-	-	IGV_LINK	1518.58	15.7998	0	32.8658	OK	2597.66	26.3471	0.116436	52.5778	OK	OK	0.737743
ENSG0000012	-	-	ENSG0000012860	'NDUFA5	-	7:123536996-123558255	-	-	IGV_LINK	938.689	48.7592	12.9403	84.5781	OK	994.101	49.7016	11.4401	87.9631	OK	OK	0.027617
ENSG0000012	-	-	ENSG0000012861	'FEZF1	-	7:122301393-122310691	-	-	IGV_LINK	0	0	0	0	OK	1.03122	0.112994	0	0.65928	OK	NOTEST	inf
ENSG0000012	-	-	ENSG0000012861	'OPN1SW	-	7:128772490-128775790	-	-	IGV_LINK	0	0	0	0	OK	0	0	0	0	OK	NOTEST	0
ENSG0000012	-	-	ENSG0000012862	'MRPS12	-	19:38915265-38975910	-	-	IGV_LINK	430.556	27.6535	0	73.8572	OK	560.986	36.4998	0	95.936	OK	OK	0.400426
ENSG0000012	-	-	ENSG0000012864	'MYO1B	-	2:191229164-191425389	-	-	IGV_LINK	883.415	12.2331	3.36716	21.0991	OK	807.449	10.8714	3.30821	18.4345	OK	OK	-0.17026
ENSG0000012	-	-	ENSG0000012864	'HOXD1	-	2:176173194-176192538	-	-	IGV_LINK	7.75776	0.242551	0	1.18468	OK	18.562	0.580724	0	2.24681	OK	OK	1.25956
ENSG0000012	-	-	ENSG0000012865	'HOXD3	-	2:176121610-176173102	-	-	IGV_LINK	168.416	6.26875	0	16.6068	OK	140.279	5.72287	0	16.8396	OK	OK	-0.13144
ENSG0000012	-	-	ENSG0000012865	'MTX2	-	2:176269394-176338025	-	-	IGV_LINK	459.648	25.0572	6.16993	43.9444	OK	503.238	27.2234	8.46778	45.9791	OK	OK	0.119626
ENSG0000012	-	-	ENSG0000012865	'PDE11A	-	2:177623251-178108339	-	-	IGV_LINK	13.1996	0.11673	0	0.652234	OK	89.7166	0.834234	0	2.48065	OK	OK	2.83728
ENSG0000012	-	-	ENSG0000012865	'CHN1	-	2:174799362-175005369	-	-	IGV_LINK	128.973	4.52487	0	9.43872	OK	111.372	4.21618	0	8.70089	OK	OK	-0.10194
ENSG0000012	-	-	ENSG0000012868	'GAD1	-	2:170813212-170861151	-	-	IGV_LINK	26.1825	1.55282	0	3.80214	OK	29.9055	0.981348	0	2.49916	OK	OK	-0.66206
ENSG0000012	-	-	ENSG0000012869	'EIF2S2P4	-	2:170723085-170770768	-	-	IGV_LINK	3.87888	0.269382	0	0.842067	OK	11.3435	0.788903	0	2.3809	OK	OK	1.5502
ENSG0000012	-	-	ENSG0000012869	'OSGEPL1	-	2:189674289-189765556	-	-	IGV_LINK	213.339	8.12568	0.756865	15.4945	OK	136.122	5.12845	0	10.2719	OK	OK	-0.66397
ENSG0000012	-	-	ENSG0000012869	'ORMDL1	-	2:189770322-189877629	-	-	IGV_LINK	864.991	48.0457	15.4071	80.6842	OK	823.949	47.0228	14.6182	79.4274	OK	OK	-0.03105
ENSG0000012	-	-	ENSG0000012870	'HAT1	-	2:171784369-172082430	-	-	IGV_LINK	595.408	23.0474	1.09549	44.9994	OK	670.296	25.5968	3.44252	47.7511	OK	OK	0.151358
ENSG0000012	-	-	ENSG0000012870	'HOXD9	-	2:176121610-176173102	-	-	IGV_LINK	409.222	13.586	0.082153	27.0898	OK	566.143	18.8084	2.25037	35.3665	OK	OK	0.469259
ENSG0000012	-	-	ENSG0000012871	'HOXD10	-	2:176104215-176119942	-	-	IGV_LINK	188.181	7.10052	1.23585	12.9652	OK	291.414	11.1131	2.06291	20.1632	OK	OK	0.646258
ENSG0000012	-	-	ENSG0000012871	'HOXD11	-	2:176104215-176119942	-	-	IGV_LINK	21.2789	1.05685	0	3.4748	OK	122.107	5.70291	0	13.0054	OK	OK	2.43193
ENSG0000012	-	-	ENSG0000012871	'HOXD13	-	2:176092890-176095938	-	-	IGV_LINK	0.969721	0.026433	0	0.10353	OK	0	0	0	0	OK	NOTEST	'-inf

ENSG0000012	-	-	ENSG0000012873	'HERC2	-	15:28111039-28322152	-	-	IGV_LINK	1155.91	13.5567	0	32.1027	OK	1323.06	18.4318	0	42.1626	OK	OK	0.443186
ENSG0000012	-	-	ENSG0000012873	'SNRPN	-	15:24823636-25439056	-	-	IGV_LINK	1499.61	65.681	0	132.291	OK	1172.77	52.317	2.92102	101.713	OK	OK	-0.3282
ENSG0000012	-	-	ENSG0000012878	'PSMG2	-	18:12438889-12725740	-	-	IGV_LINK	857.233	71.7017	0	169.136	OK	827.042	68.4702	0	158.264	OK	OK	-0.06653
ENSG0000012	-	-	ENSG0000012879	'TWSG1	-	18:9334766-9402420	-	-	IGV_LINK	320.977	6.63706	1.68545	11.5887	OK	372.272	7.49892	2.45326	12.5446	OK	OK	0.176139
ENSG0000012	-	-	ENSG0000012880	'ARHGAP22	-	10:48446033-48656265	-	-	IGV_LINK	403.404	14.0877	1.14122	27.0343	OK	256.775	7.6829	0.497499	14.8683	OK	OK	-0.87472
ENSG0000012	-	-	ENSG0000012881	'WDFY4	-	10:48684875-49115509	-	-	IGV_LINK	1250.94	10.737	0	23.1275	OK	997.195	7.1748	1.17043	13.1792	OK	OK	-0.58159
ENSG0000012	-	-	ENSG0000012882	'EIF2AK4	-	15:39934145-40035591	-	-	IGV_LINK	990.085	11.5169	4.16583	18.868	OK	1063.19	13.5503	4.16875	22.9319	OK	OK	0.234574
ENSG0000012	-	-	ENSG0000012883	'MYO5C	-	15:52115104-52295798	-	-	IGV_LINK	224.975	2.22501	0	5.06644	OK	272.243	2.4456	0	4.99889	OK	OK	0.136379
ENSG0000012	-	-	ENSG0000012884	'CGNL1	-	15:57375966-57550727	-	-	IGV_LINK	2047.08	16.492	7.00354	25.9804	OK	5912.01	48.1595	16.3295	79.9896	OK	OK	1.54606
ENSG0000012	-	-	ENSG0000012887	'TMOD2	-	15:51723010-51816368	-	-	IGV_LINK	429.586	2.83569	0.455688	5.2157	OK	420.74	2.83137	0.314726	5.34802	OK	OK	-0.0022
ENSG0000012	-	-	ENSG0000012888	'TTBK2	-	15:42738733-42920809	-	-	IGV_LINK	581.833	3.82819	0	9.58099	OK	267.087	1.58388	0	4.00332	OK	OK	-1.2732
ENSG0000012	-	-	ENSG0000012888	'ELL3	-	15:43726917-43803043	-	-	IGV_LINK	46.2321	2.39472	0	33.8372	OK	40.4324	2.16097	0	32.9045	OK	OK	-0.14818
ENSG0000012	-	-	ENSG0000012889	'CCDC32	-	15:40488040-40565057	-	-	IGV_LINK	424.738	17.9769	0	37.017	OK	460.958	22.0879	0	45.8465	OK	OK	0.297116
ENSG0000012	-	-	ENSG0000012890	'INO80	-	15:40978879-41116354	-	-	IGV_LINK	1001.72	12.9172	0	28.2209	OK	838.386	12.5557	0	28.2974	OK	OK	-0.04095
ENSG0000012	-	-	ENSG0000012891	'ICE2	-	15:60419608-60479160	-	-	IGV_LINK	677.835	16.6015	0	36.1383	OK	474.363	11.9311	0	27.0753	OK	OK	-0.47659
ENSG0000012	-	-	ENSG0000012891	'DLL4	-	15:40929339-40939072	-	-	IGV_LINK	971.66	18.7684	7.39932	30.1374	OK	1074.54	23.5039	8.77706	38.2308	OK	OK	0.324597
ENSG0000012	-	-	ENSG0000012891	'ALDH1A2	-	15:57953423-58569843	-	-	IGV_LINK	156.125	4.61354	0	18.7386	OK	1288	32.3428	0	100.001	OK	OK	2.8095
ENSG0000012	-	-	ENSG0000012892	'MINDY2	-	15:58771191-59097419	-	-	IGV_LINK	505.224	3.58864	0	9.96431	OK	385.678	2.46257	0	6.96592	OK	OK	-0.54328
ENSG0000012	-	-	ENSG0000012892	'IVD	-	15:40405794-40435947	-	-	IGV_LINK	1228.64	38.7868	5.07802	72.4956	OK	1589.12	44.9507	5.17541	84.726	OK	OK	0.212778
ENSG0000012	-	-	ENSG0000012894	'KNSTRN	-	15:40382720-40394246	-	-	IGV_LINK	137.7	7.64933	0	15.7635	OK	136.122	6.57302	0.56374	12.5823	OK	OK	-0.21878
ENSG0000012	-	-	ENSG0000012895	'DUT	-	15:48312352-48343373	-	-	IGV_LINK	592.499	45.6811	14.8386	76.5236	OK	610.485	46.5208	15.2372	77.8043	OK	OK	0.026278
ENSG0000012	-	-	ENSG0000012896	'CHAC1	-	15:40952961-40956519	-	-	IGV_LINK	18.4247	0.783357	0	1.68932	OK	18.562	0.754569	0	1.62673	OK	OK	-0.05402
ENSG0000012	-	-	ENSG0000012897	'CLN6	-	15:68054178-68257211	-	-	IGV_LINK	752.34	22.342	0	46.6419	OK	956.686	28.1181	0.850222	55.386	OK	OK	0.33174
ENSG0000012	-	-	ENSG0000012898	'ARPP19	-	15:52547044-52569883	-	-	IGV_LINK	1967.56	33.2478	0	68.9481	OK	2327.47	36.9479	1.60985	72.2859	OK	OK	0.152233
ENSG0000012	-	-	ENSG0000012900	'VPS13C	-	15:61852388-62060473	-	-	IGV_LINK	1856.05	21.49	0	47.6775	OK	1204.47	16.0222	0	34.7814	OK	OK	-0.42359
ENSG0000012	-	-	ENSG0000012900	'CALML4	-	15:68054178-68257211	-	-	IGV_LINK	380.896	15.5714	0	44.2013	OK	741.474	39.9536	0	90.7951	OK	OK	1.35943
ENSG0000012	-	-	ENSG0000012900	'ISLR	-	15:74152799-74179226	-	-	IGV_LINK	54.3043	1.41791	0	2.97339	OK	3.09367	0.087112	0	0.397467	OK	OK	-4.02475
ENSG0000012	-	-	ENSG0000012902	'THAP10	-	15:70829129-71053657	-	-	IGV_LINK	54.3043	2.66145	0	10.13	OK	44.3427	1.15834	0	3.52865	OK	OK	-1.20015
ENSG0000012	-	-	ENSG0000012903	'LOXL1	-	15:73908070-73952137	-	-	IGV_LINK	55.2741	3.5051	0	9.06199	OK	84.5604	3.20322	0	7.50467	OK	OK	-0.12994
ENSG0000012	-	-	ENSG0000012904	'ACKR4	-	3:132558137-132874223	-	-	IGV_LINK	67.8804	2.19666	0	15.7473	OK	39.1865	1.26894	0	9.44734	OK	OK	-0.79168
ENSG0000012	-	-	ENSG0000012905	'ANAPC13	-	3:134477705-134575017	-	-	IGV_LINK	649.713	35.5204	7.12223	63.9185	OK	788.887	43.9701	8.1785	79.7618	OK	OK	0.307878
ENSG0000012	-	-	ENSG0000012907	'MBD4	-	3:129430943-129520507	-	-	IGV_LINK	771.898	24.944	0	55.9252	OK	1100.32	36.6224	0	76.0989	OK	OK	0.554035
ENSG0000012	-	-	ENSG0000012908	'COPB1	-	11:14443439-14643635	-	-	IGV_LINK	1920.05	36.621	0.739506	72.5025	OK	2658.17	49.6588	5.37948	93.9382	OK	OK	0.439378
ENSG0000012	-	-	ENSG0000012908	'PSMA1	-	11:14443439-14643635	-	-	IGV_LINK	1421.61	68.0217	3.15517	132.888	OK	1949.57	93.2634	0	187.256	OK	OK	0.455315
ENSG0000012	-	-	ENSG0000012910	'SUMF2	-	7:56064001-56092996	-	-	IGV_LINK	1701.86	78.124	22.1863	134.062	OK	1931.48	84.3975	22.5816	146.213	OK	OK	0.111434
ENSG0000012	-	-	ENSG0000012911	'PALLD	-	4:168356734-169010275	-	-	IGV_LINK	391.767	7.60998	0	20.2013	OK	694.014	12.4591	0	29.2866	OK	OK	0.71123
ENSG0000012	-	-	ENSG0000012912	'SPCS3	-	4:176308267-176332245	-	-	IGV_LINK	1248.03	17.8521	5.65487	30.0494	OK	1443.71	22.3594	4.37582	40.343	OK	OK	0.324786
ENSG0000012	-	-	ENSG0000012915	'BBOX1	-	11:27040724-27220113	-	-	IGV_LINK	1280.03	53.2517	21.9956	84.5079	OK	1591.18	65.1748	27.2554	103.094	OK	OK	0.291486
ENSG0000012	-	-	ENSG0000012915	'MYOD1	-	11:17719567-17722131	-	-	IGV_LINK	1.93944	0.067117	0	0.205542	OK	0	0	0	0	OK	NOTEST	'-inf
ENSG0000012	-	-	ENSG0000012915	'SERGEF	-	11:17734773-18042426	-	-	IGV_LINK	391.767	27.2294	5.10791	49.3509	OK	301.118	19.8361	2.95986	36.7124	OK	OK	-0.45703
ENSG0000012	-	-	ENSG0000012915	'KCNC1	-	11:17734773-18042426	-	-	IGV_LINK	5.81832	0.106751	0	0.874632	OK	1.03122	0.081243	0	1.13651	OK	NOTEST	-0.39393
ENSG0000012	-	-	ENSG0000012916	'TPH1	-	11:17734773-18042426	-	-	IGV_LINK	51.3952	0.725407	0	2.54801	OK	42.2802	0.489379	0	1.56974	OK	OK	-0.56784
ENSG0000012	-	-	ENSG0000012917	'CSRP3	-	11:19182029-19281426	-	-	IGV_LINK	0	0	0	0	OK	0	0	0	0	OK	NOTEST	0
ENSG0000012	-	-	ENSG0000012917	'E2F8	-	11:19182029-19281426	-	-	IGV_LINK	53.3346	1.12851	0	3.00516	OK	51.5612	1.14507	0	2.62477	OK	OK	0.021005
ENSG0000012	-	-	ENSG0000012918	'DCTD	-	4:182890059-182917936	-	-	IGV_LINK	1081.24	41.8918	14.2565	69.527	OK	1083.82	43.9252	14.7342	73.1161	OK	OK	0.068382
ENSG0000012	-	-	ENSG0000012919	'SOX15	-	17:7561874-7633383	-	-	IGV_LINK	9.69721	0.515924	0	14.4948	OK	10.3122	0.475448	0	15.9906	OK	NOTEST	-0.11787
ENSG0000012	-	-	ENSG0000012919	'PIMREG	-	17:6444414-6556494	-	-	IGV_LINK	47.5163	2.29349	0	5.73688	OK	44.3427	2.58303	0	6.4395	OK	OK	0.171519
ENSG0000012	-	-	ENSG0000012919	'RPAIN	-	17:5282264-5469060	-	-	IGV_LINK	675.895	25.0842	0	62.1262	OK	549.643	23.471	0	61.4401	OK	OK	-0.0959
ENSG0000012	-	-	ENSG0000012920	'USP6	-	17:5105540-5175034	-	-	IGV_LINK	31.0311	0.219399	0	0.661663	OK	43.3114	0.317467	0	0.821021	OK	NOTEST	0.533049
ENSG0000012	-	-	ENSG0000012921	'SHBG	-	17:7561874-7633383	-	-	IGV_LINK	3.87888	0.40103	0	19.1276	OK	3.09367	0.313762	0	19.7013	OK	NOTEST	-0.35404
ENSG0000012	-	-	ENSG0000012921	'PLD2	-	17:4807095-4823434	-	-	IGV_LINK	875.658	25.0452	0.196368	49.8941	OK	983.789	30.091	0.323708	59.8582	OK	OK	0.264796
ENSG0000012	-	-	ENSG0000012922	'AIPL1	-	17:6393692-6435199	-	-	IGV_LINK	0	0	0	0	OK	0	0	0	0	OK	NOTEST	0
ENSG0000012	-	-	ENSG0000012922	'CD68	-	17:7561874-7633383	-	-	IGV_LINK	4874.03	227.838	0	567.59	OK	9474.05	468.009	0	1115.48	OK	OK	1.03853
ENSG0000012	-	-	ENSG0000012923	'TXNDC17	-	17:6578147-6651634	-	-	IGV_LINK	290.916	54.7377	0	123.092	OK	359.898	71.074	0	142.901	OK	OK	0.376788
ENSG0000012	-	-	ENSG0000012924	'ATP1B2	-	17:7646626-7657768	-	-	IGV_LINK	162.913	3.08094	0.673635	5.48825	OK	109.31	3.03883	0	6.96526	OK	OK	-0.01986
ENSG0000012	-	-	ENSG0000012924	'FXR2	-	17:7561874-7633383	-	-	IGV_LINK	447.041	11.2544	0	74.3217	OK	422.802	10.6221	0	86.623	OK	OK	-0.08343
ENSG0000012	-	-	ENSG0000012925	'KIF1C	-	17:4997947-5028401	-	-	IGV_LINK	1057	13.4222	0	28.6356	OK	1271.5	17.8208	0	37.679	OK	OK	0.408941
ENSG0000012	-	-	ENSG0000012925	'MPDU1	-	17:7561874-7633383	-	-	IGV_LINK	647.773	34.755	0	159.938	OK	902.322	50.4511	0	249.568	OK	OK	0.537665
ENSG0000012	-	-	ENSG0000012929	'PHF20L1	-	8:132775357-132848807	-	-	IGV_LINK	726.321	13.3198	1.62372	25.0159	OK	648.641	12.0824	0.925694	23.2391	OK	OK	-0.14067
ENSG0000012	-	-	ENSG0000012929	'LRRC6	-	8:132571952-132675617	-	-	IGV_LINK	260.855	15.0379	2.2668	27.8089	OK	236.151	14.2198	1.61936	26.8203	OK	OK	-0.0807
ENSG0000012	-	-	ENSG0000012931	'CCNT1	-	12:48688457-48716998	-	-	IGV_LINK	605.106	5.30918	1.26183	9.35653	OK	637.297	5.60846	1.23067	9.98625	OK	OK	0.079117
ENSG0000012	-	-	ENSG0000012931	'PUS7L	-	12:43718992-43758817	-	-	IGV_LINK	275.401	1.26516	0.122778	2.40754	OK	234.088	1.7262	0	4.81959	OK	OK	0.448275
ENSG0000012	-	-	ENSG0000012934	'KRI1	-	19:10543894-10566037	-	-	IGV_LINK	729.23	15.7263	2.73332	28.7192	OK	795.074	16.6612	4.68678	28.6357	OK	OK	0.08332
ENSG0000012	-	-	ENSG0000012935	'ILF3	-	19:10654260-10692417	-	-	IGV_LINK	5110.43	81.6061	19.3007	143.912	OK	4243.49	66.9381	16.4688	117.407	OK	OK	-0.28585
ENSG0000012	-	-	ENSG0000012935	'SLC44A2	-	19:10602456-10644559	-	-	IGV_LINK	1843.44	37.7469	13.537	61.9567	OK	2396.57	48.4777	18.0187	78.9368	OK	OK	0.360965
ENSG0000012	-	-	ENSG0000012935	'AP1M2	-	19:10572670-10587315	-	-	IGV_LINK	86.3051	8.64571	0	18.762	OK	248.525	22.314	0	44.6898	OK	OK	1.36789
ENSG0000012	-	-	ENSG0000012935	'CDKN2D	-	19:10566461-10569059	-	-	IGV_LINK	109.578	6.23684	0.690992	11.7827	OK	107.247	6.05551	0.400665	11.7104	OK	OK	-0.04257
ENSG0000012	-	-	ENSG0000012942	'MTUS1	-	8:17576432-17800917	-	-	IGV_LINK	479.042	6.55474	1.58974	11.5197	OK	559.955	10.7288	0	22.6045	OK	OK	0.710874
ENSG0000012	-	-	ENSG0000012943	'KLK14	-	19:51077494-51084245	-	-	IGV_LINK	3.87888	0.227593	0	0.602964	OK	1.03122	0.06556	0	0.279611	OK	NOTEST	-1.79556
ENSG0000012	-	-	ENSG0000012945	'SIGLEC9	-	19:51124907-51136651	-	-	IGV_LINK	186.186	6.70159	1.6411	11.7621	OK	287.712	10.3973	3.06833	17.7264	OK	OK	0.633641
ENSG0000012	-	-	ENSG0000012945	'KLK10	-	19:51012738-51020175	-	-	IGV_LINK	0.969721	0.142137	0	0.624431	OK	0	0	0	0	OK	NOTEST	'-inf
ENSG0000012	-	-	ENSG0000012945	'KLK8	-	19:50943302-51012129	-	-	IGV_LINK	0	0	0	0	OK	0	0	0	0	OK	NOTEST	0
ENSG0000012	-	-	ENSG0000012946	'NGDN	-	14:23469687-23509862	-	-	IGV_LINK	380.13	22.4537	5.64665	39.2608	OK	309.367	18.586	4.53686	32.6352	OK	OK	-0.27274
ENSG0000012	-	-	ENSG0000012946	'RIPK3	-	14:24318348-24340045	-	-	IGV_LINK	264.734	10.2776	0	22.2112	OK	299.366	12.9978	0	27.9456	OK	OK	0.338763
ENSG0000012	-	-	ENSG0000012946	'ADCY4	-	14:24318348-24340045	-	-	IGV_LINK	393.707	9.1978	0	18.9025	OK	535.83	14.0916	0	28.3228	OK	OK	0.615474
ENSG0000012	-	-	ENSG0000012947	'RAB2B	-	14:21459019-21511375	-	-	IGV_LINK	342.311	10.1843	0	32.3028	OK	347.523	10.2204	0	32.0302	OK	OK	0.005098
ENSG0000012	-	-	ENSG0000012947	'BCL2L2	-	14:23185315-23326185	-	-	IGV_LINK	736.641	14.3051	0	40.0568	OK	879.433	16.8207	0	46.0745	OK	OK	0.233712
ENSG0000012	-	-	ENSG0000012947	'AJUBA	-	14:22929608-22982642	-	-	IGV_LINK	335.523	6.93334	0	16.5571	OK	404.24	9.83265	0	25.3008	OK	OK	0.504031
ENSG0000012	-	-	ENSG0000012948	'DTD2	-	14:31248627-31457510	-	-	IGV_LINK	79.1313	2.7392	0	7.85853	OK	92.3599	3.5073	0	9.14964	OK	OK	0.356607
ENSG0000012	-	-	ENSG0000012948	'PARP2	-	14:20343047-20357905	-	-	IGV_LINK	289.946	13.0667	1.87975	24.2538	OK	249.556	11.1014	1.5387	20.6642	OK	OK	-0.23515
ENSG0000012	-	-	ENSG0000012949	'HEATR5A	-	14:31248627-31457510	-	-	IGV_LINK	358.797	3.00211	0.565104	5.43911	OK	291.837	3.74264	0	9.83618	OK	OK	0.318083
ENSG0000012	-	-	ENSG0000012951	'FOXA1	-	14:37589983-38202923	-	-	IGV_LINK	0.969721	0.033456	0	0.448078	OK	3.09367	0.165165	0	0.754146	OK	NOTEST	2.30359
ENSG0000012	-	-	ENSG0000012951	'SNX6	-	14:34556773-34630183	-	-	IGV_LINK	772.867	26.7441	5.8349	47.6533	OK	994.101	40.2631	7.76546	72.7608	OK	OK	0.590239
ENSG0000012	-	-	ENSG0000012951	'EAPP	-	14:34515928-34539711	-	-	IGV_LINK	359.766	17.7897	6.20764	29.3718	OK	414.552	20.5605	7.45984	33.6611	OK	OK	0.208827
ENSG0000012	-	-	ENSG0000012952	'EGLN3	-	14:33924230-34462774	-	-	IGV_LINK	4138.77	132.914	35.9846	229.844	OK	4771.48	158.92	23.9286	293.911	OK	OK	0.257803
ENSG0000012	-	-	ENSG0000012953	'MIS18BP1	-	14:45203189-45253540	-	-	IGV_LINK	318.068	10.1118	0	20.4228	OK	271.212	7.01028	0	14.5643	OK	OK	-0.5285
ENSG0000012	-	-	ENSG0000012953	'NRL	-	14:24080106-24132849	-	-	IGV_LINK	14.5458	0.525414	0	4.18632	OK	23.7182	0.895413	0	5.28166	OK	OK	0.769097
ENSG0000012	-	-	ENSG0000012953	'RNASE1	-	14:20801227-20803278	-	-	IGV_LINK	529.467	42.3477	12.2597	72.4356	OK	3495.85	273.802	104.731	442.873	OK	OK	2.69278
ENSG0000012	-	-	ENSG0000012955	'NEDD8	-	14:24171852-24239242	-	-	IGV_LINK	986.537	103.705	0	237.866	OK	1086.91	115.243	0	268.907	OK	OK	0.152185
ENSG0000012	-	-	ENSG0000012956	'DAD1	-	14:22564904-22589269	-	-	IGV_LINK	1014.33	102.98	44.4465	161.514	OK	1169.41	119.095	51.4591	186.73	OK	OK	0.209743
ENSG0000012	-	-	ENSG0000012956	'TEP1	-	14:20365666-20413429	-	-	IGV_LINK	1417.73	21.6183	0	45.751	OK	1358.12	23.8683	0	50.6506	OK	OK	0.142839
ENSG0000012	-	-	ENSG0000012959	'EPB41L4A	-	5:112142440-112419316	-	-	IGV_LINK	924.144	14.4751	0	31.1807	OK	1242.63	18.397	1.50108	35.2929	OK	OK	0.345896
ENSG0000012	-	-	ENSG0000012959	'CDO1	-	5:115804732-115816954	-	-	IGV_LINK	2.90916	0.097312	0	0.632492	OK	5.15612	0.185311	0	0.779529	OK	NOTEST	0.929262
ENSG0000012	-	-	ENSG0000012962	'REEP5	-	5:112707497-112922539	-	-	IGV_LINK	2189.63	66.5056	0	133.236	OK	2474.94	90.0599	3.36093	176.759	OK	OK	0.437409
ENSG0000012	-	-	ENSG0000012963	'ITFG1	-	16:47144322-47701523	-	-	IGV_LINK	1303.3	41.0435	2.02704	80.06	OK	1910.86	63.4732	9.93818	117.008	OK	OK	0.628993
ENSG0000012	-	-	ENSG0000012964	'QRICH2	-	17:76274048-76307998	-	-	IGV_LINK	59.153	1.0802	0	2.40211	OK	42.2802	1.21076	0	2.86418	OK	OK	0.164613
ENSG0000012	-	-	ENSG0000012965	'FOXJ1	-	17:76136332-76240373	-	-	IGV_LINK	5.81832	0.132833	0	0.800782	OK	0	0	0	0	OK	NOTEST	'-inf
ENSG0000012	-	-	ENSG0000012965	'SEC14L1	-	17:77086715-77217101	-	-	IGV_LINK	2735.64	41.8177	2.04964	81.5857	OK	2997.5	47.854	5.76431	89.9436	OK	OK	0.194526
ENSG0000012	-	-	ENSG0000012966	'RHBDF2	-	17:76470890-76501790	-	-	IGV_LINK	1225.73	36.9346	4.96618	68.9029	OK	1273.56	39.3521	5.74516	72.9591	OK	OK	0.091471
ENSG0000012	-	-	ENSG0000012967	'AANAT	-	17:76453350-76470117	-	-	IGV_LINK	10.6669	0.500635	0	1.24847	OK	6.18735	0.515499	0	1.46393	OK	NOTEST	0.042211
ENSG0000012	-	-	ENSG0000012967	'ARHGEF6	-	X:136665546-136782088	-	-	IGV_LINK	1482.7	17.3289	7.62601	27.0318	OK	1412.78	16.5048	7.30494	25.7047	OK	OK	-0.07029
ENSG0000012	-	-	ENSG0000012968	'MAP7D3	-	X:136213219-136256482	-	-	IGV_LINK	626.439	10.3513	2.77009	17.9324	OK	840.448	14.6621	4.52318	24.8011	OK	OK	0.50229
ENSG0000012	-	-	ENSG0000012968	'FGF13	-	X:138614730-139222777	-	-	IGV_LINK	64.0016	1.71994	0	3.87824	OK	14.4371	0.329323	0	1.00159	OK	OK	-2.38478
ENSG0000012	-	-	ENSG0000012969	'ASH2L	-	8:38105241-38213301	-	-	IGV_LINK	848.505	19.297	4.46724	34.1268	OK	1040.51	23.1686	6.11768	40.2195	OK	OK	0.263793
ENSG0000012	-	-	ENSG0000012969	'TTI2	-	8:33370823-33513601	-	-	IGV_LINK	215.278	6.29042	0	12.9005	OK	251.619	7.43773	0.290487	14.585	OK	OK	0.241706
ENSG0000012	-	-	ENSG0000012974	'ART1	-	11:3645127-3664416	-	-	IGV_LINK	0	0	0	0	OK	1.03122	0.051053	0	0.404731	OK	NOTEST	inf
ENSG0000012	-	-	ENSG0000012974	'CHRNA10	-	11:3665586-3826371	-	-	IGV_LINK	31.0311	1.44524	0	8.19057	OK	22.6869	0.921908	0	6.25022	OK	OK	-0.64861
ENSG0000012	-	-	ENSG0000012975	'CDKN1C	-	11:2883212-2885881	-	-	IGV_LINK	274.431	11.9986	3.20305	20.7941	OK	157.777	7.36194	1.39464	13.3292	OK	OK	-0.70471
ENSG0000012	-	-	ENSG0000012981	'SGO1	-	3:20160592-21226305	-	-	IGV_LINK	22.3036	0.947559	0	2.28943	OK	13.4059	0.674443	0	1.70731	OK	OK	-0.49052
ENSG0000012	-	-	ENSG0000012981	'TTTY1B	-	Y:6390430-6424145	-	-	IGV_LINK	0	0	0	0	OK	0	0	0	0	OK	NOTEST	0
ENSG0000012	-	-	ENSG0000012982	'RPS4Y1	-	Y:2841485-2932000	-	-	IGV_LINK	1249	89.7021	36.3487	143.055	OK	862.104	59.9851	22.4275	97.5427	OK	OK	-0.58054
ENSG0000012	-	-	ENSG0000012984	'TTTY1	-	Y:9740583-9774289	-	-	IGV_LINK	0	0	0	0	OK	0	0	0	0	OK	NOTEST	0
ENSG0000012	-	-	ENSG0000012986	'VCY1B	-	Y:14056216-14056958	-	-	IGV_LINK	0	0	0	0	OK	0	0	0	0	OK	NOTEST	0
ENSG0000012	-	-	ENSG0000012986	'VCY	-	Y:13985771-13986513	-	-	IGV_LINK	0	0	0	0	OK	0	0	0	0	OK	NOTEST	0
ENSG0000012	-	-	ENSG0000012987	'CDY2B	-	Y:17877409-17880220	-	-	IGV_LINK	0	0	0	0	OK	0	0	0	0	OK	NOTEST	0
ENSG0000012	-	-	ENSG0000012991	'CDH15	-	16:89171766-89195492	-	-	IGV_LINK	2.90916	0.115906	0	0.335085	OK	1.03122	0.04112	0	0.190549	OK	NOTEST	-1.49507
ENSG0000012	-	-	ENSG0000012991	'KLF16	-	19:1852381-1863568	-	-	IGV_LINK	223.036	4.62537	1.29798	7.95276	OK	257.806	5.7548	0.807317	10.7023	OK	OK	0.315196
ENSG0000012	-	-	ENSG0000012992	'TMEM8A	-	16:370772-392960	-	-	IGV_LINK	663.289	18.0689	3.42391	32.7138	OK	816.73	27.3916	5.32164	49.4616	OK	OK	0.60023
ENSG0000012	-	-	ENSG0000012993	'DOHH	-	19:3490821-3500940	-	-	IGV_LINK	193.944	5.88106	1.52967	10.2325	OK	237.182	7.19663	2.12194	12.2713	OK	OK	0.291245
ENSG0000012	-	-	ENSG0000012993	'MAU2	-	19:19276017-19358755	-	-	IGV_LINK	1716.41	34.1976	0	70.9633	OK	1843.83	38.2189	0	78.5762	OK	OK	0.160389
ENSG0000012	-	-	ENSG0000012994	'SHC2	-	19:416582-460996	-	-	IGV_LINK	524.619	16.4263	5.59422	27.2584	OK	998.226	31.7119	12.2279	51.1959	OK	OK	0.949018
ENSG0000012	-	-	ENSG0000012995	'PLPPR3	-	19:812487-821977	-	-	IGV_LINK	1.93944	0.050698	0	0.418815	OK	0	0	0	0	OK	NOTEST	'-inf
ENSG0000012	-	-	ENSG0000012996	'INS-IGF2	-	11:2129111-2161341	-	-	IGV_LINK	0	0	0	0	OK	0.001838	3.85E-05	0	0.312185	OK	NOTEST	inf
ENSG0000012	-	-	ENSG0000012996	'ABHD17A	-	19:1876809-1885547	-	-	IGV_LINK	1623.31	36.79	12.119	61.4609	OK	1374.62	31.9801	11.4045	52.5557	OK	OK	-0.20214
ENSG0000012	-	-	ENSG0000012998	'LBP	-	20:38346355-38377023	-	-	IGV_LINK	1.93944	0.061112	0	0.187154	OK	6305.94	198.83	77.6141	320.046	OK	OK	11.6678
ENSG0000012	-	-	ENSG0000012999	'SYT5	-	19:55151766-55180441	-	-	IGV_LINK	0.969721	0.345539	0	2.18869	OK	3.09367	0.279479	0	1.79649	OK	NOTEST	-0.30611
ENSG0000012	-	-	ENSG0000012999	'TNNI3	-	19:55151766-55180441	-	-	IGV_LINK	0	0	0	0	OK	3.09367	0.261653	0	1.60297	OK	NOTEST	inf
ENSG0000012	-	-	ENSG0000012999	'CBFA2T3	-	16:88874857-88977204	-	-	IGV_LINK	120.245	3.41243	0	9.74505	OK	102.091	3.13398	0	7.56988	OK	OK	-0.1228
ENSG0000013	-	-	ENSG0000013000	'GAMT	-	19:1397025-1401570	-	-	IGV_LINK	476.133	27.9365	10.3709	45.5021	OK	810.543	46.0634	18.0399	74.0868	OK	OK	0.721468
ENSG0000013	-	-	ENSG0000013002	'PUDP	-	X:7048919-7148190	-	-	IGV_LINK	185.217	5.34975	1.28609	9.41341	OK	253.681	8.9092	1.64313	16.1753	OK	OK	0.735823
ENSG0000013	-	-	ENSG0000013002	'ERMARD	-	6:169751621-169781584	-	-	IGV_LINK	564.377	24.3639	4.99239	43.7353	OK	303.18	14.078	1.58578	26.5702	OK	OK	-0.7913
ENSG0000013	-	-	ENSG0000013002	'PHF10	-	6:169702189-169738992	-	-	IGV_LINK	967.622	22.5211	2.92242	42.1197	OK	509.425	12.3695	0.931814	23.8071	OK	OK	-0.86449

ENSG000001	-	-	ENSG0000013003	'PRRG3	-	X:151695123-151705924	-	-	IGV_LINK	9.69721	0.313799	0	1.36287	OK	5.15612	0.050071	0	0.54189	OK	NOTEST	-2.64781
ENSG000001	-	-	ENSG0000013003	'GALNT8	-	12:4562203-4851927	-	-	IGV_LINK	14.3964	0.376994	0	4.01917	OK	9.28102	0.333582	0	3.61553	OK	NOTEST	-0.1765
ENSG000001	-	-	ENSG0000013003	'KCNA5	-	12:5043988-5046788	-	-	IGV_LINK	14.5458	0.311986	0	0.695028	OK	5.15612	0.11064	0	0.271216	OK	NOTEST	-1.49561
ENSG000001	-	-	ENSG0000013003	'CRACR2A	-	12:3606632-3764819	-	-	IGV_LINK	182.307	4.24383	0	8.77734	OK	200.058	5.03775	0	10.2592	OK	OK	0.247415
ENSG000001	-	-	ENSG0000013004	'NXNL2	-	9:88535100-88584274	-	-	IGV_LINK	158.064	8.36629	1.80318	14.9294	OK	160.871	8.94579	1.73891	16.1527	OK	OK	0.096622
ENSG000001	-	-	ENSG0000013005	'STARD8	-	X:68647665-68725842	-	-	IGV_LINK	547.892	6.92	2.1395	11.7005	OK	524.894	6.7603	1.81013	11.7105	OK	OK	-0.03369
ENSG000001	-	-	ENSG0000013005	'FAM155B	-	X:69505240-69532508	-	-	IGV_LINK	20.3641	0.298824	0	0.661392	OK	29.9055	0.438967	0	0.903141	OK	NOTEST	0.554814
ENSG000001	-	-	ENSG0000013005	'GDPD2	-	X:70423030-70435350	-	-	IGV_LINK	2.90916	0.072368	0	0.265407	OK	2.06245	0.061384	0	0.250084	OK	NOTEST	-0.2375
ENSG000001	-	-	ENSG0000013006	'SAT1	-	X:23783172-23786226	-	-	IGV_LINK	9056.22	617.265	182.499	1052.03	OK	14210.3	956.533	272.5	1640.57	OK	OK	0.631925
ENSG000001	-	-	ENSG0000013011	'GNL3L	-	X:54530210-54561071	-	-	IGV_LINK	230.793	6.44371	1.86929	11.0181	OK	285.649	7.87079	2.28862	13.453	OK	OK	0.288616
ENSG000001	-	-	ENSG0000013014	'SH3BP4	-	2:234951972-235055714	-	-	IGV_LINK	879.536	11.1726	1.94321	20.402	OK	521.8	6.6126	1.14148	12.0837	OK	OK	-0.75668
ENSG000001	-	-	ENSG0000013015	'MOSPD2	-	X:14873440-14922327	-	-	IGV_LINK	435.405	8.36536	1.82195	14.9088	OK	428.99	9.08688	1.75284	16.4209	OK	OK	0.119356
ENSG000001	-	-	ENSG0000013015	'DOCK6	-	19:11199294-11262481	-	-	IGV_LINK	1603.92	28.9024	0	68.9312	OK	1586.02	28.776	0	63.6286	OK	OK	-0.00633
ENSG000001	-	-	ENSG0000013015	'ECSIT	-	19:11451325-11529174	-	-	IGV_LINK	629.349	26.6524	8.36647	44.9383	OK	566.143	25.3806	7.33509	43.4261	OK	OK	-0.07054
ENSG000001	-	-	ENSG0000013016	'LDLR	-	19:11089361-11155808	-	-	IGV_LINK	41.698	1.05057	0	4.00673	OK	47.4363	1.00057	0	2.97988	OK	OK	-0.07035
ENSG000001	-	-	ENSG0000013016	'ELOF1	-	19:11551146-11559236	-	-	IGV_LINK	533.346	36.8931	13.5079	60.2782	OK	632.141	43.6495	16.5546	70.7444	OK	OK	0.242617
ENSG000001	-	-	ENSG0000013016	'TSPAN16	-	19:11296138-11354944	-	-	IGV_LINK	0	0	0	0	OK	1.03122	0.067083	0	2.50709	OK	NOTEST	inf
ENSG000001	-	-	ENSG0000013017	'ANGPTL8	-	19:11199294-11262481	-	-	IGV_LINK	1.93944	0.202379	0	4.27135	OK	90.7478	7.24031	0	24.8104	OK	OK	5.16092
ENSG000001	-	-	ENSG0000013017	'PRKCSH	-	19:11420603-11450968	-	-	IGV_LINK	4722.54	161.799	52.2451	271.352	OK	6530.75	227.125	57.8896	396.36	OK	OK	0.489285
ENSG000001	-	-	ENSG0000013017	'CNN1	-	19:11538716-11550323	-	-	IGV_LINK	38.7888	1.62491	0	3.56876	OK	42.2802	1.63275	0	3.44552	OK	OK	0.006953
ENSG000001	-	-	ENSG0000013017	'CDC16	-	13:114234886-114272723	-	-	IGV_LINK	1275.18	43.3823	16.9881	69.7766	OK	1232.31	38.745	14.548	62.942	OK	OK	-0.1631
ENSG000001	-	-	ENSG0000013018	'ZSCAN10	-	16:3088889-3099317	-	-	IGV_LINK	0	0	0	0	OK	0	0	0	0	OK	NOTEST	0
ENSG000001	-	-	ENSG0000013019	'THEM6	-	8:142727202-142736927	-	-	IGV_LINK	433.465	11.6979	4.22094	19.1749	OK	402.178	10.8583	3.8473	17.8692	OK	OK	-0.10746
ENSG000001	-	-	ENSG0000013020	'NECTIN2	-	19:44846174-44903689	-	-	IGV_LINK	936.75	34.7697	4.82641	64.713	OK	1282.84	42.4308	7.59761	77.264	OK	OK	0.28728
ENSG000001	-	-	ENSG0000013020	'APOE	-	19:44905753-44919349	-	-	IGV_LINK	17653.8	978.038	94.4527	1861.62	OK	15942.7	885.12	108.805	1661.43	OK	OK	-0.14402
ENSG000001	-	-	ENSG0000013020	'TOMM40	-	19:44846174-44903689	-	-	IGV_LINK	560.498	20.0995	0.638359	39.5606	OK	633.172	21.7342	0	44.2348	OK	OK	0.112809
ENSG000001	-	-	ENSG0000013020	'APOC1	-	19:44905753-44919349	-	-	IGV_LINK	4817.56	698.457	0	2061.57	OK	4332.16	647.965	0	1888.45	OK	OK	-0.10826
ENSG000001	-	-	ENSG0000013022	'GADD45G	-	9:89605012-89606555	-	-	IGV_LINK	87.2748	5.52645	0.534723	10.5182	OK	71.1545	4.57558	0.258119	8.89305	OK	OK	-0.2724
ENSG000001	-	-	ENSG0000013022	'LRCH2	-	X:115110615-115234072	-	-	IGV_LINK	15.5155	0.18383	0	0.396361	OK	10.3122	0.123789	0	0.28543	OK	NOTEST	-0.57049
ENSG000001	-	-	ENSG0000013022	'DPP6	-	7:153887096-154894285	-	-	IGV_LINK	0	0	0	0	OK	2.06245	0.032925	0	0.597275	OK	NOTEST	inf
ENSG000001	-	-	ENSG0000013022	'XPO7	-	8:21919670-22006585	-	-	IGV_LINK	1507.92	21.0816	5.0157	37.1474	OK	1564.37	22.3796	6.25526	38.504	OK	OK	0.086205
ENSG000001	-	-	ENSG0000013023	'ACE2	-	X:15561032-15602148	-	-	IGV_LINK	1032.75	18.6902	7.37143	30.0089	OK	178.402	3.12298	0.713925	5.53204	OK	OK	-2.58128
ENSG000001	-	-	ENSG0000013024	'FAM98C	-	19:38403134-38426305	-	-	IGV_LINK	421.828	23.3091	2.05562	44.5625	OK	360.929	17.7333	1.34203	34.1246	OK	OK	-0.39443
ENSG000001	-	-	ENSG0000013025	'SAFB2	-	19:5586998-5668478	-	-	IGV_LINK	1797.86	68.7291	0	151.809	OK	2045.95	78.0641	0	170.862	OK	OK	0.183738
ENSG000001	-	-	ENSG0000013025	'RPL36	-	19:5674946-5720572	-	-	IGV_LINK	7179.06	861.669	201.502	1521.84	OK	5716.08	645.659	135.157	1156.16	OK	OK	-0.41636
ENSG000001	-	-	ENSG0000013027	'ATP8B3	-	19:1782074-1812276	-	-	IGV_LINK	160.004	2.85946	0.128412	5.59051	OK	722.889	10.2752	3.07144	17.4789	OK	OK	1.84535
ENSG000001	-	-	ENSG0000013028	'GDF1	-	19:18868544-18896727	-	-	IGV_LINK	0	0	0	0	OK	0	0	0	0	OK	NOTEST	0
ENSG000001	-	-	ENSG0000013028	'NCAN	-	19:19211972-19252233	-	-	IGV_LINK	0	0	0	0	OK	3.09367	0.028106	0	0.328827	OK	NOTEST	inf
ENSG000001	-	-	ENSG0000013029	'KIF1A	-	2:240713763-240820308	-	-	IGV_LINK	0	0	0	0	OK	1.03122	0.006712	0	0.573524	OK	NOTEST	inf
ENSG000001	-	-	ENSG0000013029	'GTPBP3	-	19:17334919-17342735	-	-	IGV_LINK	352.009	13.5698	1.47517	25.6644	OK	500.144	22.4392	3.95954	40.9188	OK	OK	0.725619
ENSG000001	-	-	ENSG0000013029	'PLVAP	-	19:17351447-17377350	-	-	IGV_LINK	5828.99	158.002	58.4027	257.6	OK	5281.93	146.05	46.3298	245.77	OK	OK	-0.11348
ENSG000001	-	-	ENSG0000013030	'BST2	-	19:17402938-17405648	-	-	IGV_LINK	5600.14	375.832	146.421	605.243	OK	5774.86	387.608	149.366	625.85	OK	OK	0.044511
ENSG000001	-	-	ENSG0000013030	'SLC27A1	-	19:17468768-17521288	-	-	IGV_LINK	802.929	14.0846	2.13032	26.0388	OK	772.388	13.6501	1.28212	26.0181	OK	OK	-0.04521
ENSG000001	-	-	ENSG0000013030	'NSUN5	-	7:73302516-73308867	-	-	IGV_LINK	410.192	15.4414	4.6457	26.2371	OK	415.584	15.4821	4.29421	26.67	OK	OK	0.003799
ENSG000001	-	-	ENSG0000013030	'USHBP1	-	19:17231882-17287646	-	-	IGV_LINK	171.609	4.55091	0	17.1088	OK	155.715	4.03103	0	17.3204	OK	OK	-0.17501
ENSG000001	-	-	ENSG0000013030	'COLGALT1	-	19:17555593-17583162	-	-	IGV_LINK	2212.9	39.7442	14.6918	64.7966	OK	3251.45	63.5023	14.9625	112.042	OK	OK	0.676064
ENSG000001	-	-	ENSG0000013031	'DDA1	-	19:17292130-17334829	-	-	IGV_LINK	545.953	24.0356	0	62.4217	OK	612.548	35.4265	0	85.8956	OK	OK	0.559654
ENSG000001	-	-	ENSG0000013031	'MRPL34	-	19:17292130-17334829	-	-	IGV_LINK	470.314	54.5646	0	111.588	OK	422.802	45.7122	0	97.436	OK	OK	-0.25539
ENSG000001	-	-	ENSG0000013031	'PGLS	-	19:17468768-17521288	-	-	IGV_LINK	778.686	51.0852	9.37539	92.7949	OK	859.01	56.5891	12.3241	100.854	OK	OK	0.147621
ENSG000001	-	-	ENSG0000013033	'LSM7	-	19:2252251-2354806	-	-	IGV_LINK	439.283	66.6097	0	245.43	OK	406.303	62.644	0	270.184	OK	OK	-0.08856
ENSG000001	-	-	ENSG0000013033	'TULP4	-	6:158232235-158511828	-	-	IGV_LINK	355.887	3.78951	0	10.4821	OK	259.869	2.63891	0	7.95766	OK	OK	-0.52207
ENSG000001	-	-	ENSG0000013034	'SNX9	-	6:157700386-157945077	-	-	IGV_LINK	1084.15	15.1777	6.58624	23.7692	OK	745.576	10.6113	3.92749	17.2952	OK	OK	-0.51635
ENSG000001	-	-	ENSG0000013034	'RTN4IP1	-	6:106360807-106629487	-	-	IGV_LINK	99.8812	3.72902	0	12.8135	OK	70.1233	2.62001	0	8.98356	OK	OK	-0.50922
ENSG000001	-	-	ENSG0000013034	'QRSL1	-	6:106629577-106668417	-	-	IGV_LINK	386.918	6.45322	0.807363	12.0991	OK	269.15	4.65024	0.016145	9.28433	OK	OK	-0.47271
ENSG000001	-	-	ENSG0000013034	'C6orf203	-	6:107028212-107051342	-	-	IGV_LINK	174.55	13.0364	2.97912	23.0937	OK	123.747	9.47206	1.21173	17.7324	OK	OK	-0.4608
ENSG000001	-	-	ENSG0000013036	'RSPH3	-	6:158972870-159065273	-	-	IGV_LINK	631.288	10.1654	0	22.0289	OK	444.458	6.44644	0	13.3551	OK	OK	-0.65709
ENSG000001	-	-	ENSG0000013036	'MAS1	-	6:159906689-159916530	-	-	IGV_LINK	0.969721	0.005652	0	0.022139	OK	0	0	0	0	OK	NOTEST	'-inf
ENSG000001	-	-	ENSG0000013037	'ACSBG2	-	19:5978402-6205121	-	-	IGV_LINK	4.8486	0.456482	0	2.81441	OK	1.03122	0.146264	0	1.57295	OK	NOTEST	-1.64198
ENSG000001	-	-	ENSG0000013038	'MLLT1	-	19:6210378-6279948	-	-	IGV_LINK	886.325	15.0011	0.628686	29.3736	OK	1026.07	14.8082	3.07742	26.539	OK	OK	-0.01867
ENSG000001	-	-	ENSG0000013038	'FUT5	-	19:5865825-5904006	-	-	IGV_LINK	0	0	0	0	OK	0	0	0	0	OK	NOTEST	0
ENSG000001	-	-	ENSG0000013038	'BMP15	-	X:50910783-50916607	-	-	IGV_LINK	0	0	0	0	OK	0	0	0	0	OK	NOTEST	0
ENSG000001	-	-	ENSG0000013039	'AFDN	-	6:167826921-167972023	-	-	IGV_LINK	3594.75	67.4339	0	135.878	OK	1200.35	20.3577	0	40.9563	OK	OK	-1.7279
ENSG000001	-	-	ENSG0000013040	'ACTN4	-	19:38647648-38769904	-	-	IGV_LINK	5892.02	200.925	0	427.127	OK	6831.87	243.95	0	534.18	OK	OK	0.279928
ENSG000001	-	-	ENSG0000013041	'STK33	-	11:8391867-8594289	-	-	IGV_LINK	21.3339	0.676833	0	1.84492	OK	101.06	4.45637	0	9.2558	OK	OK	2.719
ENSG000001	-	-	ENSG0000013041	'NDUFA10	-	2:239892449-240025402	-	-	IGV_LINK	1832.77	68.766	19.6014	117.931	OK	1670.58	68.3377	19.6511	117.024	OK	OK	-0.00901
ENSG000001	-	-	ENSG0000013042	'EPO	-	7:100720799-100723700	-	-	IGV_LINK	12.6064	0.613782	0	1.36044	OK	1.03122	0.050259	0	0.188107	OK	OK	-3.61029
ENSG000001	-	-	ENSG0000013042	'ARPC1B	-	7:99325878-99466331	-	-	IGV_LINK	2604.2	126.147	0	282.985	OK	2795.82	139.85	0	336.976	OK	OK	0.14877
ENSG000001	-	-	ENSG0000013043	'CACNG6	-	19:53992287-54012669	-	-	IGV_LINK	0	0	0	0	OK	0	0	0	0	OK	NOTEST	0
ENSG000001	-	-	ENSG0000013044	'ZSWIM6	-	5:61332272-61546170	-	-	IGV_LINK	370.433	3.9178	1.38347	6.45213	OK	397.022	4.19991	1.51721	6.88261	OK	OK	0.100315
ENSG000001	-	-	ENSG0000013047	'FCHO1	-	19:17747717-17788568	-	-	IGV_LINK	307.401	8.27777	0.867353	15.6882	OK	456.833	12.2674	1.92351	22.6113	OK	OK	0.567515
ENSG000001	-	-	ENSG0000013047	'UNC13A	-	19:17601327-17688365	-	-	IGV_LINK	25.2127	0.267956	0	1.04245	OK	83.5292	0.876503	0	2.91998	OK	OK	1.70976
ENSG000001	-	-	ENSG0000013047	'MAP1S	-	19:17719241-17734516	-	-	IGV_LINK	490.679	12.8885	0	25.9176	OK	466.114	9.65914	1.30641	18.0119	OK	OK	-0.41612
ENSG000001	-	-	ENSG0000013048	'KLHDC7B	-	22:50548032-50551023	-	-	IGV_LINK	102.79	2.05529	0.24747	3.86312	OK	60.8423	1.21704	0.019295	2.41478	OK	OK	-0.75597
ENSG000001	-	-	ENSG0000013048	'SCO2	-	22:50523567-50532580	-	-	IGV_LINK	521.726	35.8793	0	133.337	OK	421.331	29.0158	0	99.8659	OK	OK	-0.30631
ENSG000001	-	-	ENSG0000013050	'PXDN	-	2:1631886-1744852	-	-	IGV_LINK	1406.09	14.5174	2.10488	26.93	OK	1802.58	17.3446	3.78826	30.901	OK	OK	0.256703
ENSG000001	-	-	ENSG0000013051	'SSBP4	-	19:18418863-18438301	-	-	IGV_LINK	855.293	57.4861	0	117.978	OK	612.548	41.947	6.21654	77.6774	OK	OK	-0.45465
ENSG000001	-	-	ENSG0000013051	'GDF15	-	19:18374730-18389176	-	-	IGV_LINK	459.648	25.2534	8.98786	41.5189	OK	704.327	38.7623	15.9609	61.5638	OK	OK	0.618179
ENSG000001	-	-	ENSG0000013051	'PGPEP1	-	19:18340586-18369950	-	-	IGV_LINK	1501.13	19.6883	0	45.1943	OK	1846.92	24.5538	0	55.9809	OK	OK	0.318602
ENSG000001	-	-	ENSG0000013051	'KIAA1683	-	19:18257096-18274509	-	-	IGV_LINK	97.9418	1.15016	0.0052	2.29511	OK	86.6229	1.14396	0	2.8599	OK	OK	-0.0078
ENSG000001	-	-	ENSG0000013052	'LSM4	-	19:18306229-18323274	-	-	IGV_LINK	732.139	50.5163	17.9267	83.1059	OK	760.013	51.2068	19.2713	83.1423	OK	OK	0.019585
ENSG000001	-	-	ENSG0000013052	'JUND	-	19:18279759-18281622	-	-	IGV_LINK	991.054	44.4597	11.0104	77.909	OK	1068.35	53.5805	9.36111	97.7999	OK	OK	0.269211
ENSG000001	-	-	ENSG0000013052	'HRC	-	19:49151197-49155424	-	-	IGV_LINK	119.276	3.35486	0	6.9638	OK	73.217	2.20545	0	5.12168	OK	OK	-0.60518
ENSG000001	-	-	ENSG0000013052	'TRPM4	-	19:49157740-49211836	-	-	IGV_LINK	151.276	4.49845	0	10.3243	OK	314.524	7.16196	0	15.1237	OK	OK	0.670924
ENSG000001	-	-	ENSG0000013053	'OR11H1	-	22:15528157-15529139	-	-	IGV_LINK	0	0	0	0	OK	0	0	0	0	OK	NOTEST	0
ENSG000001	-	-	ENSG0000013054	'SULT4A1	-	22:43824508-43862503	-	-	IGV_LINK	2.90916	0.079334	0	0.340476	OK	2.06245	0.056275	0	0.305361	OK	NOTEST	-0.49546
ENSG000001	-	-	ENSG0000013054	'ZNF557	-	19:7069443-7087968	-	-	IGV_LINK	159.034	2.16585	0.198939	4.13276	OK	141.278	2.1919	0.205055	4.17875	OK	OK	0.01725
ENSG000001	-	-	ENSG0000013054	'CRB3	-	19:6436078-6482557	-	-	IGV_LINK	256.006	21.4996	0	67.5089	OK	222.745	17.443	0	49.5516	OK	OK	-0.30166
ENSG000001	-	-	ENSG0000013055	'OLFM1	-	9:135075421-135121179	-	-	IGV_LINK	320.977	10.0259	2.0561	17.9958	OK	239.244	7.91819	0.420805	15.4156	OK	OK	-0.34049
ENSG000001	-	-	ENSG0000013055	'CAMSAP1	-	9:135808486-135907228	-	-	IGV_LINK	805.838	7.03908	2.65107	11.4271	OK	590.892	5.30877	1.7051	8.91243	OK	OK	-0.40701
ENSG000001	-	-	ENSG0000013056	'UBAC1	-	9:135932968-135961380	-	-	IGV_LINK	683.653	24.8976	9.40993	40.3853	OK	715.67	26.5331	9.92214	43.144	OK	OK	0.091783
ENSG000001	-	-	ENSG0000013056	'SAG	-	2:233307815-233347055	-	-	IGV_LINK	0.969721	0.184886	0	1.25466	OK	2.06245	0.024163	0	0.926907	OK	NOTEST	-2.93579
ENSG000001	-	-	ENSG0000013058	'ZBTB46	-	20:63743665-63831244	-	-	IGV_LINK	317.099	3.74642	0.804501	6.68834	OK	232.026	2.67393	0.661115	4.68674	OK	OK	-0.48655
ENSG000001	-	-	ENSG0000013058	'HELZ2	-	20:63558085-63574239	-	-	IGV_LINK	980.387	12.1378	0	24.6341	OK	1346.78	14.8322	1.91835	27.746	OK	OK	0.289221
ENSG000001	-	-	ENSG0000013059	'SAMD10	-	20:63974112-63980008	-	-	IGV_LINK	279.28	9.55919	2.09136	17.027	OK	169.121	6.91863	0.049852	13.7874	OK	OK	-0.4664
ENSG000001	-	-	ENSG0000013059	'LSP1	-	11:1852969-1892267	-	-	IGV_LINK	2118.84	87.5278	33.2753	141.78	OK	1423.09	56.4809	20.6985	92.2633	OK	OK	-0.63198
ENSG000001	-	-	ENSG0000013059	'TNNT3	-	11:1919561-1938706	-	-	IGV_LINK	3.87888	0.543688	0	1.96685	OK	4.1249	0.425494	0	1.39584	OK	NOTEST	-0.35364
ENSG000001	-	-	ENSG0000013059	'TNNI2	-	11:1838988-1841680	-	-	IGV_LINK	35.8797	4.49638	0	9.47919	OK	11.3435	1.48155	0	3.42158	OK	OK	-1.60165
ENSG000001	-	-	ENSG0000013060	'H19	-	11:1995162-2001470	-	-	IGV_LINK	293.825	14.3216	1.37665	27.2665	OK	496.019	19.1784	3.68851	34.6683	OK	OK	0.42129
ENSG000001	-	-	ENSG0000013061	'CYP2G1P	-	19:40771647-40900508	-	-	IGV_LINK	0.969721	0.02337	0	0.613994	OK	0	0	0	0	OK	NOTEST	'-inf
ENSG000001	-	-	ENSG0000013063	'COL5A1	-	9:134641773-134844843	-	-	IGV_LINK	401.464	3.13014	0	6.62492	OK	366.085	2.78276	0	5.77528	OK	OK	-0.16971
ENSG000001	-	-	ENSG0000013063	'ATXN10	-	22:45657018-45845307	-	-	IGV_LINK	1198.2	78.9552	14.2341	143.676	OK	1289.67	87.5088	24.6946	150.323	OK	OK	0.148394
ENSG000001	-	-	ENSG0000013064	'TUBGCP2	-	10:133278629-133352683	-	-	IGV_LINK	1369.25	31.4985	0	64.6231	OK	993.07	22.5658	0	46.4672	OK	OK	-0.48115
ENSG000001	-	-	ENSG0000013064	'CALY	-	10:133278629-133352683	-	-	IGV_LINK	0.969721	0.089519	0	2.35118	OK	2.06245	0.190753	0	2.8942	OK	NOTEST	1.09144
ENSG000001	-	-	ENSG0000013064	'CYP2E1	-	10:133453927-133569835	-	-	IGV_LINK	46.5466	1.3066	0	3.54316	OK	22.6869	0.671759	0	2.26904	OK	OK	-0.95981
ENSG000001	-	-	ENSG0000013065	'PNPLA7	-	9:137459952-137550534	-	-	IGV_LINK	1065.72	14.7168	4.57647	24.8571	OK	1077.63	14.1737	5.2233	23.1241	OK	OK	-0.05425
ENSG000001	-	-	ENSG0000013065	'HBZ	-	16:152686-166768	-	-	IGV_LINK	0	0	0	0	OK	0	0	0	0	OK	NOTEST	0
ENSG000001	-	-	ENSG0000013066	'PAK4	-	19:39125769-39182816	-	-	IGV_LINK	570.196	14.7036	2.69227	26.715	OK	714.639	19.4253	4.11929	34.7313	OK	OK	0.401765
ENSG000001	-	-	ENSG0000013067	'MNX1	-	7:156994050-157010651	-	-	IGV_LINK	3.87888	0.116431	0	1.11542	OK	24.7494	0.950814	0	2.37466	OK	OK	3.02969
ENSG000001	-	-	ENSG0000013068	'ZNF337	-	20:25612934-25696841	-	-	IGV_LINK	930.932	16.5746	5.47748	27.6718	OK	919.853	16.2971	5.65447	26.9396	OK	OK	-0.02436
ENSG000001	-	-	ENSG0000013069	'CEP85	-	1:26234152-26279038	-	-	IGV_LINK	269.582	5.24709	0.130065	10.3641	OK	312.461	6.0013	1.196	10.8066	OK	OK	0.193758
ENSG000001	-	-	ENSG0000013069	'TAF4	-	20:61953468-62065810	-	-	IGV_LINK	295.765	5.79564	0.870843	10.7204	OK	248.525	6.24557	0	17.0148	OK	OK	0.107865
ENSG000001	-	-	ENSG0000013070	'GATA5	-	20:62463496-62475970	-	-	IGV_LINK	9.69721	0.225592	0	0.508809	OK	0	0	0	0	OK	NOTEST	'-inf
ENSG000001	-	-	ENSG0000013070	'RBBP8NL	-	20:62410236-62427533	-	-	IGV_LINK	0	0	0	0	OK	0	0	0	0	OK	NOTEST	0
ENSG000001	-	-	ENSG0000013070	'LAMA5	-	20:62302092-62367312	-	-	IGV_LINK	7266.12	104.204	0	274.73	OK	7134.01	91.4641	0	252.89	OK	OK	-0.18814
ENSG000001	-	-	ENSG0000013070	'OSBPL2	-	20:62231921-62296213	-	-	IGV_LINK	811.656	15.6572	2.74094	28.5735	OK	1030.19	21.4477	3.8519	39.0434	OK	OK	0.453993
ENSG000001	-	-	ENSG0000013070	'ADRM1	-	20:62302092-62367312	-	-	IGV_LINK	767.049	34.4853	0	107.418	OK	927.071	41.7996	0	122.479	OK	OK	0.27751
ENSG000001	-	-	ENSG0000013070	'ASS1	-	9:130444928-130501274	-	-	IGV_LINK	2429.15	98.7106	43.0758	154.345	OK	3891.84	159.469	65.8365	253.102	OK	OK	0.692
ENSG000001	-	-	ENSG0000013071	'PRDM12	-	9:130664593-130682981	-	-	IGV_LINK	0	0	0	0	OK	1.03122	0.025242	0	0.094475	OK	NOTEST	inf
ENSG000001	-	-	ENSG0000013071	'EXOSC2	-	9:130693720-130704894	-	-	IGV_LINK	609.954	20.1588	5.73402	34.5835	OK	639.359	20.5135	5.30114	35.7259	OK	OK	0.025167
ENSG000001	-	-	ENSG0000013071	'POMT1	-	9:131502901-131531268	-	-	IGV_LINK	838.808	21.1146	0	42.2931	OK	755.888	20.5903	0.577785	40.6029	OK	OK	-0.03627
ENSG000001	-	-	ENSG0000013071	'UCK1	-	9:131502901-131531268	-	-	IGV_LINK	842.687	26.5724	3.58638	49.5585	OK	755.888	23.629	2.68	44.5779	OK	OK	-0.16937
ENSG000001	-	-	ENSG0000013072	'FIBCD1	-	9:130902437-130945520	-	-	IGV_LINK	3.87888	0.074256	0	0.51366	OK	3.09367	0.068147	0	0.502421	OK	NOTEST	-0.12386
ENSG000001	-	-	ENSG0000013072	'PRRC2B	-	9:131394092-131500197	-	-	IGV_LINK	2722.98	36.0815	0	87.5274	OK	3600.01	53.8129	0	129.539	OK	OK	0.576694
ENSG000001	-	-	ENSG0000013072	'CHMP2A	-	19:58551565-58555124	-	-	IGV_LINK	1540.89	119.624	49.692	189.557	OK	1725.24	133.266	51.3552	215.176	OK	OK	0.155793
ENSG000001	-	-	ENSG0000013072	'UBE2M	-	19:58555711-58558960	-	-	IGV_LINK	695.29	45.3872	10.3842	80.3902	OK	680.608	45.8258	10.1558	81.4959	OK	OK	0.013876
ENSG000001	-	-	ENSG0000013072	'TRIM28	-	19:58544090-58550722	-	-	IGV_LINK	2181.87	91.0889	24.6957	157.482	OK	2210.95	116.52	36.9877	196.053	OK	OK	0.355234
ENSG000001	-	-	ENSG0000013073	'METTL26	-	16:634426-636366	-	-	IGV_LINK	1256.76	114.4	46.2654	182.535	OK	1274.59	122.994	51.0094	194.98	OK	OK	0.1045
ENSG000001	-	-	ENSG0000013073	'YIPF2	-	19:10871512-10928681	-	-	IGV_LINK	717.593	27.1597	4.57978	49.7396	OK	987.914	38.7639	9.14905	68.3788	OK	OK	0.513248
ENSG000001	-	-	ENSG0000013073	'ATG4D	-	19:10543894-10566037	-	-	IGV_LINK	294.795	16.4706	0	37.9347	OK	285.649	15.792	0	36.7498	OK	OK	-0.06069
ENSG000001	-	-	ENSG0000013074	'EIF2S3	-	X:24054715-24077971	-	-	IGV_LINK	3418.26	81.2174	17.789	144.646	OK	3276.2	78.5977	16.3006	140.895	OK	OK	-0.0473
ENSG000001	-	-	ENSG0000013074	'TMEM160	-	19:47045906-47048630	-	-	IGV_LINK	141.579	16.2675	3.22745	29.3075	OK	83.5292	9.62009	0.858056	18.3821	OK	OK	-0.75787
ENSG000001	-	-	ENSG0000013074	'ZC3H4	-	19:47064186-47210636	-	-	IGV_LINK	643.894	7.97641	0	18.4796	OK	608.423	8.2707	0	20.7818	OK	OK	0.052269

ENSG000001	-	-	ENSG0000013075	NPAS1	-	19:47019819-47045775	-	-	IGV_LINK	16.4852	0.820168	0	1.88519	OK	20.6245	1.42906	0	3.38143	OK	OK	0.801078
ENSG000001	-	-	ENSG0000013075	GMFG	-	19:39328352-39342372	-	-	IGV_LINK	820.384	84.9291	33.1312	136.727	OK	664.109	72.0349	26.7883	117.281	OK	OK	-0.23756
ENSG000001	-	-	ENSG0000013075	MAP3K10	-	19:40191743-40215575	-	-	IGV_LINK	186.186	5.32572	0	11.2581	OK	188.714	5.12565	0	10.3829	OK	OK	-0.05524
ENSG000001	-	-	ENSG0000013076	ARHGEF16	-	1:3454425-3481113	-	-	IGV_LINK	396.616	8.76424	2.57721	14.9513	OK	692.983	15.42	5.48742	25.3526	OK	OK	0.815103
ENSG000001	-	-	ENSG0000013076	LRRC47	-	1:3778557-3796504	-	-	IGV_LINK	806.807	21.9825	0	44.9524	OK	839.417	24.3071	0	49.9366	OK	OK	0.145022
ENSG000001	-	-	ENSG0000013076	SESN2	-	1:28259526-28282491	-	-	IGV_LINK	303.523	5.21422	1.72472	8.70372	OK	598.11	10.2786	4.08602	16.4711	OK	OK	0.979114
ENSG000001	-	-	ENSG0000013076	SMPDL3B	-	1:27934992-27968096	-	-	IGV_LINK	221.096	7.54194	0.553906	14.53	OK	399.084	14.1143	2.84642	25.3822	OK	OK	0.904151
ENSG000001	-	-	ENSG0000013077	ATPIF1	-	1:28236108-28246906	-	-	IGV_LINK	1471.07	173.424	54.4915	292.356	OK	1515.9	199.456	71.7812	327.13	OK	OK	0.201766
ENSG000001	-	-	ENSG0000013077	MED18	-	1:28329001-28335967	-	-	IGV_LINK	163.883	7.6429	0.664661	14.6211	OK	246.463	12.4069	2.03449	22.7794	OK	OK	0.698953
ENSG000001	-	-	ENSG0000013077	THEMIS2	-	1:27872542-27886685	-	-	IGV_LINK	1331.43	34.5111	13.1373	55.8848	OK	2256.32	56.4168	21.9552	90.8785	OK	OK	0.709067
ENSG000001	-	-	ENSG0000013077	CLIP1	-	12:122271431-122422632	-	-	IGV_LINK	1093.84	14.925	3.99893	25.8511	OK	1296.25	17.4392	4.31095	30.5674	OK	OK	0.224599
ENSG000001	-	-	ENSG0000013078	CCDC62	-	12:122774326-122827528	-	-	IGV_LINK	7.75776	0.307113	0	1.32956	OK	6.18735	0.111266	0	0.494326	OK	NOTEST	-1.46475
ENSG000001	-	-	ENSG0000013078	HIP1R	-	12:122834452-122862960	-	-	IGV_LINK	1359.55	25.2529	6.92591	43.5799	OK	999.257	18.3723	4.86848	31.8762	OK	OK	-0.45891
ENSG000001	-	-	ENSG0000013080	ZNF317	-	19:9140379-9163424	-	-	IGV_LINK	726.321	12.9846	1.5476	24.4216	OK	773.419	15.9971	0	32.0203	OK	OK	0.301013
ENSG000001	-	-	ENSG0000013081	PPAN	-	19:10106222-10119918	-	-	IGV_LINK	348.767	17.6876	0	47.9471	OK	458.801	24.517	0	61.4065	OK	OK	0.471044
ENSG000001	-	-	ENSG0000013081	EIF3G	-	19:10106222-10119918	-	-	IGV_LINK	2155.69	135.013	47.2884	222.738	OK	1983.05	126.603	40.3266	212.88	OK	OK	-0.09279
ENSG000001	-	-	ENSG0000013081	ANGPTL6	-	19:10086121-10102796	-	-	IGV_LINK	204.611	8.12024	0	19.3276	OK	243.369	9.39005	0	24.5134	OK	OK	0.20961
ENSG000001	-	-	ENSG0000013081	C19orf66	-	19:10086121-10102796	-	-	IGV_LINK	976.509	40.3981	3.73706	77.0591	OK	1178.69	48.4797	8.57279	88.3867	OK	OK	0.263096
ENSG000001	-	-	ENSG0000013081	DNMT1	-	19:10133344-10231286	-	-	IGV_LINK	1447.79	29.7323	0	61.5614	OK	1177.66	26.8851	0	55.8268	OK	OK	-0.14523
ENSG000001	-	-	ENSG0000013081	ZNF426	-	19:9523223-9538645	-	-	IGV_LINK	376.252	5.3397	0	10.8072	OK	295.962	3.60539	0.034644	7.17614	OK	OK	-0.5666
ENSG000001	-	-	ENSG0000013082	SLC6A8	-	X:153669729-153696593	-	-	IGV_LINK	1237.36	29.4088	0.311367	58.5062	OK	1178.69	28.5843	4.10292	53.0657	OK	OK	-0.04102
ENSG000001	-	-	ENSG0000013082	PNCK	-	X:153669729-153696593	-	-	IGV_LINK	131.882	6.87058	0	21.4541	OK	18.562	1.35274	0	8.49317	OK	OK	-2.34455
ENSG000001	-	-	ENSG0000013082	DKC1	-	X:154762741-154777689	-	-	IGV_LINK	645.834	18.4431	4.6607	32.2255	OK	684.733	20.1546	5.65341	34.6558	OK	OK	0.12803
ENSG000001	-	-	ENSG0000013082	PLXNA3	-	X:154458280-154479257	-	-	IGV_LINK	1279.06	29.5852	0	73.5898	OK	781.669	17.679	0	47.5623	OK	OK	-0.74284
ENSG000001	-	-	ENSG0000013082	DUSP9	-	X:153642491-153651326	-	-	IGV_LINK	1.93944	0.051638	0	0.336183	OK	2.06245	0.054943	0	0.340312	OK	NOTEST	0.089498
ENSG000001	-	-	ENSG0000013083	MPP1	-	X:154778683-154821007	-	-	IGV_LINK	752.503	26.3646	8.02341	44.7058	OK	1004.41	35.537	10.5762	60.4978	OK	OK	0.43072
ENSG000001	-	-	ENSG0000013084	ZNF331	-	19:53520980-53580269	-	-	IGV_LINK	475.163	9.66733	1.69678	17.6379	OK	534.175	10.8773	2.70952	19.0451	OK	OK	0.170133
ENSG000001	-	-	ENSG0000013085	ZNF236	-	18:76822606-76970727	-	-	IGV_LINK	408.252	3.20318	0.887599	5.51875	OK	253.681	1.97351	0.356817	3.5902	OK	OK	-0.69874
ENSG000001	-	-	ENSG0000013087	SLC7A10	-	19:33177602-33225850	-	-	IGV_LINK	0	0	0	0	OK	5.15612	0.176276	0	1.04928	OK	NOTEST	inf
ENSG000001	-	-	ENSG0000013088	LRP3	-	19:33177602-33225850	-	-	IGV_LINK	110.687	1.76177	0.210494	3.31305	OK	204.392	4.53602	0	9.92097	OK	OK	1.3644
ENSG000001	-	-	ENSG0000013092	C12orf65	-	12:123152319-123272334	-	-	IGV_LINK	287.037	9.02836	0	21.1789	OK	348.554	11.4753	0	26.9105	OK	OK	0.345996
ENSG000001	-	-	ENSG0000013093	NOL11	-	17:67717832-67744531	-	-	IGV_LINK	507.164	16.1441	1.44008	30.848	OK	511.488	13.5774	3.07517	24.0797	OK	OK	-0.24979
ENSG000001	-	-	ENSG0000013093	UBE4B	-	1:10032831-10181239	-	-	IGV_LINK	1087.06	15.9943	3.21413	28.7745	OK	1042.57	16.2701	2.55611	29.9841	OK	OK	0.024665
ENSG000001	-	-	ENSG0000013094	CASZ1	-	1:10636603-10796650	-	-	IGV_LINK	97.9418	1.46698	0	4.18054	OK	89.7166	1.82926	0	6.55551	OK	OK	0.318413
ENSG000001	-	-	ENSG0000013094	PKDREJ	-	22:46255662-46263355	-	-	IGV_LINK	19.3944	0.145334	0	0.322122	OK	15.4684	0.115931	0	0.258063	OK	NOTEST	-0.3261
ENSG000001	-	-	ENSG0000013094	HSD17B3	-	9:96235305-96302152	-	-	IGV_LINK	80.4868	4.88287	0	9.98901	OK	122.716	7.74654	0.98784	14.5052	OK	OK	0.665821
ENSG000001	-	-	ENSG0000013095	NUTM2F	-	9:94318195-94328644	-	-	IGV_LINK	0.969721	0.023309	0	0.106666	OK	0	0	0	0	OK	NOTEST	'-inf
ENSG000001	-	-	ENSG0000013095	HABP4	-	9:96450200-96619830	-	-	IGV_LINK	288.007	6.56836	0	13.6066	OK	184.589	4.20701	0	9.69016	OK	OK	-0.64274
ENSG000001	-	-	ENSG0000013095	FBP2	-	9:94555068-94593793	-	-	IGV_LINK	0	0	0	0	OK	2.06245	0.102738	0	0.346775	OK	NOTEST	inf
ENSG000001	-	-	ENSG0000013095	SLC35D2	-	9:96320705-96383710	-	-	IGV_LINK	545.953	25.7417	8.1393	43.3442	OK	643.484	30.7592	10.2782	51.2403	OK	OK	0.256909
ENSG000001	-	-	ENSG0000013096	PRRG1	-	X:37349274-38688920	-	-	IGV_LINK	145.731	3.11975	0	18.7311	OK	148.051	2.89042	0	17.5017	OK	OK	-0.11015
ENSG000001	-	-	ENSG0000013098	UBA1	-	X:47190860-47215128	-	-	IGV_LINK	3999.13	76.1256	27.9837	124.267	OK	4532.23	83.3147	31.1355	135.494	OK	OK	0.13019
ENSG000001	-	-	ENSG0000013098	RGN	-	X:47078354-47093314	-	-	IGV_LINK	278.31	10.8736	3.3505	18.3967	OK	421.771	16.8339	5.96327	27.7045	OK	OK	0.630535
ENSG000001	-	-	ENSG0000013099	POLN	-	4:2071917-2242164	-	-	IGV_LINK	71.0403	2.03785	0	6.54843	OK	89.4696	2.53318	0	7.27416	OK	OK	0.313903
ENSG000001	-	-	ENSG0000013100	TXLNGY	-	Y:19567312-19606274	-	-	IGV_LINK	324.856	6.31502	0.075028	12.555	OK	183.558	3.36469	0	7.47963	OK	OK	-0.90831
ENSG000001	-	-	ENSG0000013100	TTTY9B	-	Y:18490914-18629077	-	-	IGV_LINK	0	0	0	0	OK	0	0	0	0	OK	NOTEST	0
ENSG000001	-	-	ENSG0000013101	PPIL4	-	6:149504732-149546038	-	-	IGV_LINK	407.283	11.4442	4.05972	18.8287	OK	317.617	8.69613	2.85792	14.5343	OK	OK	-0.39617
ENSG000001	-	-	ENSG0000013101	ULBP2	-	6:149941999-149949235	-	-	IGV_LINK	8.72748	0.418064	0	0.951478	OK	8.2498	0.395568	0	0.903011	OK	NOTEST	-0.0798
ENSG000001	-	-	ENSG0000013101	AKAP12	-	6:151239998-151358557	-	-	IGV_LINK	3116.68	26.4738	11.4672	41.4804	OK	3477.29	29.6771	11.2139	48.1404	OK	OK	0.164785
ENSG000001	-	-	ENSG0000013101	SYNE1	-	6:151656690-152637801	-	-	IGV_LINK	3955.49	42.985	6.24077	79.7293	OK	1773.71	19.6018	6.75481	32.4487	OK	OK	-1.13285
ENSG000001	-	-	ENSG0000013101	ULBP3	-	6:150063149-150069095	-	-	IGV_LINK	6.78804	0.496923	0	1.17884	OK	6.18735	0.527691	0	1.37715	OK	NOTEST	0.086671
ENSG000001	-	-	ENSG0000013102	LATS1	-	6:149658152-149718888	-	-	IGV_LINK	532.377	5.14359	1.58953	8.69765	OK	327.93	2.73798	0.801721	4.67424	OK	OK	-0.90966
ENSG000001	-	-	ENSG0000013103	EPS8L1	-	19:55072019-55087923	-	-	IGV_LINK	33.9402	2.69221	0	6.87234	OK	57.7486	5.51685	0	12.1272	OK	OK	1.03506
ENSG000001	-	-	ENSG0000013104	LILRB2	-	19:54273820-54281184	-	-	IGV_LINK	706.926	18.1898	5.69036	30.6891	OK	1048.76	26.5768	7.98568	45.168	OK	OK	0.547044
ENSG000001	-	-	ENSG0000013104	AAR2	-	20:36236458-36270918	-	-	IGV_LINK	706.926	17.5348	7.16634	27.9033	OK	737.326	18.1939	7.42769	28.96	OK	OK	0.05323
ENSG000001	-	-	ENSG0000013104	TTLL9	-	20:31847636-31952092	-	-	IGV_LINK	98.9115	2.94059	0	7.13906	OK	101.06	3.09465	0	7.42406	OK	OK	0.073671
ENSG000001	-	-	ENSG0000013105	BPIFA2	-	20:33161767-33181412	-	-	IGV_LINK	0	0	0	0	OK	2.06245	0.133084	0	0.418417	OK	NOTEST	inf
ENSG000001	-	-	ENSG0000013105	RBM39	-	20:35701346-35742312	-	-	IGV_LINK	5748.5	216.992	47.7338	386.25	OK	4547.7	177.798	44.6737	310.923	OK	OK	-0.2874
ENSG000001	-	-	ENSG0000013105	COX4I2	-	20:31637887-31645006	-	-	IGV_LINK	64.9713	7.38681	0	15.1292	OK	76.3106	8.28112	0.557629	16.0046	OK	OK	0.164874
ENSG000001	-	-	ENSG0000013105	BPIFA3	-	20:33217309-33227758	-	-	IGV_LINK	0	0	0	0	OK	0	0	0	0	OK	NOTEST	0
ENSG000001	-	-	ENSG0000013106	ZNF341	-	20:33728930-33811097	-	-	IGV_LINK	113.457	2.03635	0.147971	3.92472	OK	106.216	1.80506	0.160102	3.45001	OK	OK	-0.17394
ENSG000001	-	-	ENSG0000013106	GGT7	-	20:34844719-34927962	-	-	IGV_LINK	657.471	17.1004	0	35.1522	OK	530.05	13.9788	0	30.0566	OK	OK	-0.29079
ENSG000001	-	-	ENSG0000013106	DEFB118	-	20:31368617-31373923	-	-	IGV_LINK	0	0	0	0	OK	0	0	0	0	OK	NOTEST	0
ENSG000001	-	-	ENSG0000013106	ACSS2	-	20:34844719-34927962	-	-	IGV_LINK	699.168	19.805	0	43.7819	OK	593.986	17.2927	0	38.3456	OK	OK	-0.1957
ENSG000001	-	-	ENSG0000013108	EDA2R	-	X:66595636-66639298	-	-	IGV_LINK	278.31	4.80127	1.30964	8.29289	OK	366.085	6.30667	2.22956	10.3838	OK	OK	0.393464
ENSG000001	-	-	ENSG0000013108	ARHGEF9	-	X:63634966-63809274	-	-	IGV_LINK	573.105	11.2813	0.786128	21.7764	OK	289.774	4.28796	0.087459	8.48846	OK	OK	-1.39556
ENSG000001	-	-	ENSG0000013109	C1QL1	-	17:44959692-44968071	-	-	IGV_LINK	132.852	6.67001	1.23657	12.1035	OK	246.463	12.3867	3.72775	21.0457	OK	OK	0.893032
ENSG000001	-	-	ENSG0000013109	GFAP	-	17:44846352-44916937	-	-	IGV_LINK	182.307	5.46321	0	16.354	OK	299.055	9.03152	0	30.6958	OK	OK	0.72522
ENSG000001	-	-	ENSG0000013109	PYY	-	17:43952737-44004469	-	-	IGV_LINK	1.93944	0.124136	0	0.443706	OK	3.09367	0.198272	0	0.566179	OK	NOTEST	0.675567
ENSG000001	-	-	ENSG0000013109	HIGD1B	-	17:44846352-44916937	-	-	IGV_LINK	53.3346	7.11761	0	31.9959	OK	54.6549	8.59114	0	46.0014	OK	OK	0.271456
ENSG000001	-	-	ENSG0000013110	ATP6V1E1	-	22:17592135-17628818	-	-	IGV_LINK	2221.63	103.581	45.2618	161.9	OK	2694.59	127.576	54.4272	200.725	OK	OK	0.300601
ENSG000001	-	-	ENSG0000013111	ZNF227	-	19:44207546-44305046	-	-	IGV_LINK	289.946	9.61724	0	21.2052	OK	305.243	9.07655	0	20.0893	OK	OK	-0.08348
ENSG000001	-	-	ENSG0000013111	ZNF428	-	19:43596391-43619874	-	-	IGV_LINK	339.402	19.8138	0	39.6623	OK	268.118	15.2959	1.40587	29.186	OK	OK	-0.37335
ENSG000001	-	-	ENSG0000013112	TEX101	-	19:43401495-43418597	-	-	IGV_LINK	0	0	0	0	OK	0	0	0	0	OK	NOTEST	0
ENSG000001	-	-	ENSG0000013112	ZNF141	-	4:337813-384864	-	-	IGV_LINK	354.918	12.9668	2.89984	23.0337	OK	201.089	8.89104	0	17.9654	OK	OK	-0.5444
ENSG000001	-	-	ENSG0000013114	CCL25	-	19:8052766-8062650	-	-	IGV_LINK	0.969721	0.095626	0	0.528358	OK	1.03122	0.101896	0	0.535783	OK	NOTEST	0.091621
ENSG000001	-	-	ENSG0000013114	COX4I1	-	16:85771757-85807044	-	-	IGV_LINK	4075.74	413.588	142.58	684.596	OK	3623.72	368.064	128.795	607.334	OK	OK	-0.16824
ENSG000001	-	-	ENSG0000013114	EMC8	-	16:85771757-85807044	-	-	IGV_LINK	263.764	11.5251	0	46.2463	OK	342.367	15.8284	0	55.1626	OK	OK	0.457735
ENSG000001	-	-	ENSG0000013114	GSE1	-	16:85169524-85690073	-	-	IGV_LINK	1346.94	20.789	0	44.9812	OK	1192.1	17.5628	0	40.9222	OK	OK	-0.2433
ENSG000001	-	-	ENSG0000013115	AC010531.1	-	16:87083561-87404779	-	-	IGV_LINK	0.723155	0.052217	0	1.56684	OK	2.66128	0.192446	0	3.41343	OK	NOTEST	1.88187
ENSG000001	-	-	ENSG0000013115	GINS2	-	16:85169524-85690073	-	-	IGV_LINK	105.7	3.04023	0	11.8182	OK	120.653	2.72052	0	7.76114	OK	OK	-0.1603
ENSG000001	-	-	ENSG0000013116	CHMP1A	-	16:89644430-89671272	-	-	IGV_LINK	1485.61	44.2216	12.4214	76.0218	OK	1699.46	52.1431	13.3181	90.9681	OK	OK	0.237727
ENSG000001	-	-	ENSG0000013117	SH3BGRL	-	X:81201942-81298547	-	-	IGV_LINK	1458.46	72.9605	20.7964	125.125	OK	1350.9	68.1163	24.8393	111.393	OK	OK	-0.09912
ENSG000001	-	-	ENSG0000013117	COX7B	-	X:77899437-77907373	-	-	IGV_LINK	1385.73	92.5607	8.21263	176.909	OK	1160.13	85.5317	22.5061	148.557	OK	OK	-0.11394
ENSG000001	-	-	ENSG0000013118	SLC34A1	-	5:177379234-177398848	-	-	IGV_LINK	0.969721	0.030954	0	0.275686	OK	14.4371	0.342887	0	0.813896	OK	NOTEST	3.46955
ENSG000001	-	-	ENSG0000013118	F12	-	5:177402139-177456286	-	-	IGV_LINK	32.0008	1.00517	0	3.60882	OK	93.8415	4.63541	0	12.8006	OK	OK	2.20525
ENSG000001	-	-	ENSG0000013118	PRR7	-	5:177402139-177456286	-	-	IGV_LINK	28.1219	1.37654	0	5.10058	OK	62.9047	3.06309	0	9.06396	OK	OK	1.15394
ENSG000001	-	-	ENSG0000013119	NFATC1	-	18:79395855-79529325	-	-	IGV_LINK	439.283	7.01671	1.35725	12.6762	OK	331.023	6.74185	0.49014	12.9936	OK	OK	-0.05765
ENSG000001	-	-	ENSG0000013120	IDO1	-	8:39902274-40016391	-	-	IGV_LINK	629.349	26.1198	7.44316	44.7964	OK	446.52	21.0194	4.1777	37.861	OK	OK	-0.31342
ENSG000001	-	-	ENSG0000013123	GJA9	-	1:38838197-38941799	-	-	IGV_LINK	14.4958	0.472623	0	2.62318	OK	9.72761	0.337285	0	2.36707	OK	NOTEST	-0.48672
ENSG000001	-	-	ENSG0000013123	CAP1	-	1:40040232-40097727	-	-	IGV_LINK	3361.05	185.724	0	409.936	OK	3413.35	197.802	0	429.921	OK	OK	0.090898
ENSG000001	-	-	ENSG0000013123	PPT1	-	1:40040232-40097727	-	-	IGV_LINK	6278.94	115.944	17.0209	214.867	OK	5747.02	112.57	0	233.307	OK	OK	-0.04261
ENSG000001	-	-	ENSG0000013124	RAB11FIP4	-	17:31391623-31538217	-	-	IGV_LINK	368.494	3.18452	0.373582	5.99546	OK	228.932	3.71057	0	8.71218	OK	OK	0.220563
ENSG000001	-	-	ENSG0000013126	RLIM	-	X:74585216-74614617	-	-	IGV_LINK	717.593	5.16916	1.96846	8.36987	OK	801.262	5.7434	2.3272	9.1596	OK	OK	0.151973
ENSG000001	-	-	ENSG0000013126	CDX4	-	X:73447253-73455245	-	-	IGV_LINK	0	0	0	0	OK	0	0	0	0	OK	NOTEST	0
ENSG000001	-	-	ENSG0000013126	ABCB7	-	X:75053171-75156732	-	-	IGV_LINK	556.62	17.2762	3.87501	30.6773	OK	585.736	16.1488	4.99497	27.3026	OK	OK	-0.09736
ENSG000001	-	-	ENSG0000013132	TRAF3	-	14:102777475-102911500	-	-	IGV_LINK	632.258	7.16903	0	15.0826	OK	603.267	6.91661	0.62956	13.2037	OK	OK	-0.05171
ENSG000001	-	-	ENSG0000013135	HAUS8	-	19:17049728-17075625	-	-	IGV_LINK	105.7	4.45495	0.335354	8.57454	OK	78.3731	4.04851	0	8.35622	OK	OK	-0.13802
ENSG000001	-	-	ENSG0000013135	ADGRE3	-	19:14619116-14778541	-	-	IGV_LINK	30.0613	0.813203	0	3.79336	OK	101.06	2.81378	0	8.71248	OK	OK	1.79082
ENSG000001	-	-	ENSG0000013136	MRPS25	-	3:14799330-15065335	-	-	IGV_LINK	909.598	27.2592	0	73.6415	OK	814.668	26.6111	0	74.292	OK	OK	-0.03472
ENSG000001	-	-	ENSG0000013137	SH3BP5	-	3:15254183-15341368	-	-	IGV_LINK	576.014	14.0173	3.0172	25.0174	OK	857.979	20.4358	6.29292	34.5787	OK	OK	0.543892
ENSG000001	-	-	ENSG0000013137	HACL1	-	3:15560703-15645822	-	-	IGV_LINK	242.43	7.69593	0.926485	14.4654	OK	234.088	8.03925	0.364371	15.7141	OK	OK	0.062965
ENSG000001	-	-	ENSG0000013137	TBC1D5	-	3:17157161-18920401	-	-	IGV_LINK	1265.49	16.2747	0	39.4721	OK	961.102	10.8915	0	29.5131	OK	OK	-0.57943
ENSG000001	-	-	ENSG0000013137	CAPN7	-	3:15206151-15252918	-	-	IGV_LINK	376.252	7.02363	0.676875	13.3704	OK	334.117	6.08282	0.311108	11.8545	OK	OK	-0.20748
ENSG000001	-	-	ENSG0000013137	RFTN1	-	3:16265159-16514026	-	-	IGV_LINK	1560.28	35.6794	10.5204	60.8384	OK	1272.53	29.9621	8.53913	51.3851	OK	OK	-0.25195
ENSG000001	-	-	ENSG0000013137	C3orf20	-	3:14675098-14773034	-	-	IGV_LINK	0	0	0	0	OK	10.3122	0.226095	0	0.573273	OK	NOTEST	inf
ENSG000001	-	-	ENSG0000013138	RBSN	-	3:15070072-15099163	-	-	IGV_LINK	406.313	5.08977	0	10.8736	OK	410.428	4.28732	0	8.7612	OK	OK	-0.24753
ENSG000001	-	-	ENSG0000013138	GALNT15	-	3:16174648-16231992	-	-	IGV_LINK	57.2135	0.898153	0	2.3651	OK	133.028	1.66788	0.218995	3.11676	OK	OK	0.892978
ENSG000001	-	-	ENSG0000013138	SLC6A6	-	3:14402575-14556075	-	-	IGV_LINK	1798.83	19.8851	3.86314	35.9071	OK	1693.27	18.0435	5.5906	30.4964	OK	OK	-0.14021
ENSG000001	-	-	ENSG0000013139	KCNC3	-	19:50311936-50382982	-	-	IGV_LINK	447.041	8.0703	0	20.6411	OK	477.457	8.65365	0	21.0232	OK	OK	0.100687
ENSG000001	-	-	ENSG0000013140	NAPSA	-	19:50311936-50382982	-	-	IGV_LINK	233.703	8.00428	0	27.1886	OK	74.2482	1.88994	0	8.48805	OK	OK	-2.08243
ENSG000001	-	-	ENSG0000013140	NAPSB	-	19:50311936-50382982	-	-	IGV_LINK	1023.06	58.2541	0	132.887	OK	329.992	17.5593	0	45.2939	OK	OK	-1.73012
ENSG000001	-	-	ENSG0000013140	NR1H2	-	19:50311936-50382982	-	-	IGV_LINK	1618.46	51.2732	8.15998	94.3865	OK	1532.4	48.2712	12.7377	83.8046	OK	OK	-0.08704
ENSG000001	-	-	ENSG0000013140	LRRC4B	-	19:50516891-50568045	-	-	IGV_LINK	32.0008	0.647427	0	1.34592	OK	54.6549	1.33062	0	2.90762	OK	OK	1.03931
ENSG000001	-	-	ENSG0000013143	PDLIM4	-	5:132184875-132395614	-	-	IGV_LINK	306.432	19.387	0	58.0284	OK	392.897	23.324	0	72.3814	OK	OK	0.266729
ENSG000001	-	-	ENSG0000013143	KIF3A	-	5:132688680-132737638	-	-	IGV_LINK	368.494	8.47321	0	18.1255	OK	273.275	5.71896	0	12.5319	OK	OK	-0.56716
ENSG000001	-	-	ENSG0000013144	MGAT1	-	5:180784781-180815652	-	-	IGV_LINK	3566.63	90.1704	15.7121	164.629	OK	5586.15	140.068	22.5336	257.602	OK	OK	0.635402
ENSG000001	-	-	ENSG0000013145	GFPT2	-	5:180300689-180353387	-	-	IGV_LINK	81.4565	1.57184	0	3.14375	OK	3175.14	63.7815	23.441	104.122	OK	OK	5.34261
ENSG000001	-	-	ENSG0000013146	TUBG1	-	17:42579512-42615234	-	-	IGV_LINK	190.065	7.58401	0	21.1524	OK	338.242	14.021	0	32.7717	OK	OK	0.886556
ENSG000001	-	-	ENSG0000013146	PSME3	-	17:42798791-42843758	-	-	IGV_LINK	1036.63	29.5321	0	59.9591	OK	1499.4	42.984	0	89.9938	OK	OK	0.541515
ENSG000001	-	-	ENSG0000013146	RPL27	-	17:42998272-43002959	-	-	IGV_LINK	7441.64	1322.52	491.271	2153.77	OK	5726.39	1015.87	401.196	1630.55	OK	OK	-0.38057
ENSG000001	-	-	ENSG0000013147	PSMC3IP	-	17:42567067-42577831	-	-	IGV_LINK	237.582	11.0501	0	24.9126	OK	219.651	10.3298	0	24.2068	OK	OK	-0.09725
ENSG000001	-	-	ENSG0000013147	AOC3	-	17:42851183-42858130	-	-	IGV_LINK	135.761	2.08233	0.31344	3.85122	OK	298.024	4.59463	1.38657	7.80268	OK	OK	1.14175
ENSG000001	-	-	ENSG0000013147	ACLY	-	17:41866907-41930542	-	-	IGV_LINK	5679.65	84.3976	26.7309	142.064	OK	6792.68	101.242	34.3965	168.088	OK	OK	0.262538
ENSG000001	-	-	ENSG0000013147	VPS25	-	17:42773435-42779599	-	-	IGV_LINK	962.932	59.1097	25.0794	93.14	OK	1236.44	76.513	32.0023	121.024	OK	OK	0.37231
ENSG000001	-	-	ENSG0000013147	RAMP2	-	17:42753913-42763041	-	-	IGV_LINK	360.736	34.0212	11.2483	56.794	OK	430.021	40.2033	13.8085	66.5982	OK	OK	0.240882
ENSG000001	-	-	ENSG0000013148	AOC2	-	17:42844599-42850707	-	-	IGV_LINK	20.3641	0.461922	0	0.974512	OK	88.6853	2.02419	0.186269	3.86211	OK	OK	2.13162
ENSG000001	-	-	ENSG0000013148	G6PC	-	17:42900796-42913369	-	-	IGV_LINK	3.87888	0.074773	0	0.344323	OK	9.28102	0.183534	0	0.574576	OK	NOTEST	1.29546
ENSG000001	-	-	ENSG0000013148	AC091132.1	-	17:45506740-45563230	-	-	IGV_LINK	11.6366	1.75118	0	8.49465	OK	9.28102	1.401	0	6.91854	OK	OK	-0.32187
ENSG000001	-	-	ENSG0000013149	NDUFA2	-	5:140638739-140662479	-	-	IGV_LINK	1137.48	104.038	0	245.834	OK	1241.59	114.381	0	242.662	OK	OK	0.13674
ENSG000001	-	-	ENSG0000013150	ANKHD1	-	5:140401813-140558252	-	-	IGV_LINK	2574.57	52.9146	0	123.508	OK	2110.59	46.936	0	113.121	OK	OK	-0.17297
ENSG000001	-	-	ENSG0000013150	DIAPH1	-	5:141515015-141636870	-	-	IGV_LINK	5164.73	63.1574	11.7177	114.597	OK	4806.54	58.3603	8.00879	108.712	OK	OK	-0.11397

ENSG000001	-	-	ENSG0000013150	'NDFIP1	-	5:142108504-142154443	-	-	IGV_LINK	1853.14	29.5253	10.6009	48.4496	OK	1924.27	29.6775	12.6726	46.6824	OK	OK	0.00742
ENSG000001	-	-	ENSG0000013150	'UBE2D2	-	5:139526430-139628433	-	-	IGV_LINK	1351.79	80.3747	13.5841	147.165	OK	1578.81	100.125	17.5308	182.718	OK	OK	0.316981
ENSG000001	-	-	ENSG0000013153	'TTTY6	-	Y:22438939-22485592	-	-	IGV_LINK	0	0	0	0	OK	0	0	0	0	OK	NOTEST	0
ENSG000001	-	-	ENSG0000013154	'TTTY6B	-	Y:22100813-22147484	-	-	IGV_LINK	0	0	0	0	OK	0	0	0	0	OK	NOTEST	0
ENSG000001	-	-	ENSG0000013155	'EXOC4	-	7:133253072-134066589	-	-	IGV_LINK	1382.82	21.999	4.51025	39.4878	OK	1739.68	30.4971	3.86882	57.1255	OK	OK	0.471236
ENSG000001	-	-	ENSG0000013158	'ACAP3	-	1:1292375-1324691	-	-	IGV_LINK	890.203	20.2654	0	60.1517	OK	744.544	17.2542	0	52.9635	OK	OK	-0.23207
ENSG000001	-	-	ENSG0000013159	'C1orf159	-	1:1081817-1116361	-	-	IGV_LINK	317.099	9.07397	1.34617	16.8018	OK	249.556	7.10574	0.413164	13.7983	OK	OK	-0.35275
ENSG000001	-	-	ENSG0000013162	'ANO1	-	11:70078301-70189528	-	-	IGV_LINK	803.898	14.6933	0.471351	28.9153	OK	276.368	5.95183	0	12.352	OK	OK	-1.30376
ENSG000001	-	-	ENSG0000013162	'PPFIA1	-	11:70270699-70436584	-	-	IGV_LINK	1153.97	20.218	0	62.1188	OK	1210.66	21.4887	0	74.9793	OK	OK	0.087938
ENSG000001	-	-	ENSG0000013163	'TMEM204	-	16:1493343-1612110	-	-	IGV_LINK	454.799	14.7471	0	33.5543	OK	385.678	12.425	0	29.7048	OK	OK	-0.24718
ENSG000001	-	-	ENSG0000013165	'KREMEN2	-	16:2963943-2980539	-	-	IGV_LINK	2.90916	0.075675	0	0.549605	OK	5.15612	0.14304	0	0.807614	OK	NOTEST	0.918537
ENSG000001	-	-	ENSG0000013165	'THOC6	-	16:3022619-3039133	-	-	IGV_LINK	345.22	17.0721	0	34.4417	OK	505.3	25.3036	0	52.9653	OK	OK	0.567707
ENSG000001	-	-	ENSG0000013165	'TRAF7	-	16:2155697-2196525	-	-	IGV_LINK	711.775	16.8701	0	35.1093	OK	787.856	18.2646	0	36.6019	OK	OK	0.114577
ENSG000001	-	-	ENSG0000013166	'BARX1	-	9:93951621-93955372	-	-	IGV_LINK	0	0	0	0	OK	0	0	0	0	OK	NOTEST	0
ENSG000001	-	-	ENSG0000013166	'NINJ1	-	9:93121488-93134288	-	-	IGV_LINK	1481.73	75.9902	33.0988	118.881	OK	2816.28	144.211	60.9719	227.449	OK	OK	0.924293
ENSG000001	-	-	ENSG0000013168	'CA6	-	1:8945866-8975092	-	-	IGV_LINK	0	0	0	0	OK	0	0	0	0	OK	NOTEST	0
ENSG000001	-	-	ENSG0000013169	'NPHP4	-	1:5862810-6101193	-	-	IGV_LINK	455.769	6.56402	0	16.5619	OK	574.392	8.5523	0	21.3467	OK	OK	0.381734
ENSG000001	-	-	ENSG0000013171	'MAP1B	-	5:72107233-72209570	-	-	IGV_LINK	285.098	1.35431	0.372635	2.33599	OK	368.147	1.74899	0.582858	2.91512	OK	OK	0.36896
ENSG000001	-	-	ENSG0000013172	'RHOXF2	-	X:120036235-120245267	-	-	IGV_LINK	0	0	0	0	OK	0	0	0	0	OK	NOTEST	0
ENSG000001	-	-	ENSG0000013172	'IL13RA1	-	X:118727571-118794539	-	-	IGV_LINK	1998.59	29.328	12.5792	46.0769	OK	2884.34	42.0855	18.3047	65.8663	OK	OK	0.521042
ENSG000001	-	-	ENSG0000013172	'WDR44	-	X:118346072-118449961	-	-	IGV_LINK	396.616	6.61979	1.99271	11.2469	OK	421.771	7.18645	2.37428	11.9986	OK	OK	0.118495
ENSG000001	-	-	ENSG0000013173	'CKMT2	-	5:80947696-81301560	-	-	IGV_LINK	5.81832	0.373471	0	2.51784	OK	0	0	0	0	OK	NOTEST	'-inf
ENSG000001	-	-	ENSG0000013173	'ZCCHC9	-	5:81301589-81313297	-	-	IGV_LINK	315.159	13.3332	1.78043	24.8859	OK	387.741	18.2803	4.32105	32.2396	OK	OK	0.45527
ENSG000001	-	-	ENSG0000013173	'KRT34	-	17:41377649-41382403	-	-	IGV_LINK	0	0	0	0	OK	0	0	0	0	OK	NOTEST	0
ENSG000001	-	-	ENSG0000013173	'KRT33B	-	17:41363493-41369800	-	-	IGV_LINK	0.969721	0.038097	0	0.149216	OK	1.03122	0.040546	0	0.151754	OK	NOTEST	0.089876
ENSG000001	-	-	ENSG0000013174	'TNS4	-	17:40475827-40501597	-	-	IGV_LINK	11.6366	0.398351	0	1.2028	OK	10.3122	0.339757	0	1.00113	OK	NOTEST	-0.22954
ENSG000001	-	-	ENSG0000013174	'TOP2A	-	17:40388515-40417950	-	-	IGV_LINK	568.256	6.04124	1.80847	10.274	OK	642.453	7.49288	1.32232	13.6634	OK	OK	0.310677
ENSG000001	-	-	ENSG0000013174	'STARD3	-	17:39637064-39663484	-	-	IGV_LINK	945.477	35.6487	10.0059	61.2915	OK	982.757	35.455	10.1892	60.7208	OK	OK	-0.00786
ENSG000001	-	-	ENSG0000013175	'RARA	-	17:40309191-40357643	-	-	IGV_LINK	732.139	17.7402	6.43838	29.042	OK	843.542	21.4115	7.63794	35.1851	OK	OK	0.271368
ENSG000001	-	-	ENSG0000013177	'PPP1R1B	-	17:39626739-39636626	-	-	IGV_LINK	0.969721	0.048856	0	0.426814	OK	3.09367	0.06445	0	0.445666	OK	NOTEST	0.399625
ENSG000001	-	-	ENSG0000013177	'KHDRBS3	-	8:135457456-135656722	-	-	IGV_LINK	278.31	22.2884	2.16425	42.4126	OK	208.307	15.2646	0.889979	29.6392	OK	OK	-0.5461
ENSG000001	-	-	ENSG0000013177	'CHD1L	-	1:147172770-147517875	-	-	IGV_LINK	584.741	12.8067	3.45316	22.1603	OK	550.674	13.042	0.169249	25.9148	OK	OK	0.026266
ENSG000001	-	-	ENSG0000013177	'PEX11B	-	1:145911349-145978848	-	-	IGV_LINK	558.559	20.2353	0	57.5382	OK	568.161	20.4421	0	47.3428	OK	OK	0.014672
ENSG000001	-	-	ENSG0000013178	'FMO5	-	1:147172770-147517875	-	-	IGV_LINK	126.064	4.15677	0	11.5207	OK	131.997	3.99184	0	10.7622	OK	OK	-0.05841
ENSG000001	-	-	ENSG0000013178	'PIAS3	-	1:145845628-145859836	-	-	IGV_LINK	757.352	21.1139	5.95655	36.2712	OK	603.267	17.5276	4.26641	30.7889	OK	OK	-0.26856
ENSG000001	-	-	ENSG0000013179	'PRKAB2	-	1:147155105-147172550	-	-	IGV_LINK	487.769	5.60711	1.06579	10.1484	OK	300.086	3.22534	1.01963	5.43104	OK	OK	-0.79781
ENSG000001	-	-	ENSG0000013179	'CLUHP3	-	16:31700589-31711986	-	-	IGV_LINK	591.53	12.6564	3.93467	21.3782	OK	155.715	3.46597	0.156392	6.77554	OK	OK	-1.86854
ENSG000001	-	-	ENSG0000013180	'FSHB	-	11:30231015-30235261	-	-	IGV_LINK	0	0	0	0	OK	0	0	0	0	OK	NOTEST	0
ENSG000001	-	-	ENSG0000013182	'PDHA1	-	X:19343892-19515261	-	-	IGV_LINK	1059.9	101.596	22.7701	180.422	OK	999.257	96.1393	18.9125	173.366	OK	OK	-0.07965
ENSG000001	-	-	ENSG0000013183	'RAI2	-	X:17800048-17861337	-	-	IGV_LINK	370.433	10.0982	2.94205	17.2542	OK	328.961	8.74971	2.8235	14.6759	OK	OK	-0.20678
ENSG000001	-	-	ENSG0000013184	'MCCC2	-	5:71587287-71658704	-	-	IGV_LINK	961.963	18.0937	5.52855	30.6588	OK	1247.78	23.783	7.33078	40.2353	OK	OK	0.394446
ENSG000001	-	-	ENSG0000013184	'ZNF304	-	19:57350847-57359898	-	-	IGV_LINK	272.491	3.9793	0.824856	7.13375	OK	260.9	3.78094	1.161	6.40089	OK	OK	-0.07377
ENSG000001	-	-	ENSG0000013184	'ZSCAN5A	-	19:56219669-56393545	-	-	IGV_LINK	97.9418	6.82762	0	18.1573	OK	107.247	9.48313	0	24.2544	OK	OK	0.47398
ENSG000001	-	-	ENSG0000013184	'ZNF132	-	19:58432813-58440222	-	-	IGV_LINK	73.6988	1.60001	0	3.22662	OK	60.8423	1.31898	0	2.63861	OK	OK	-0.27865
ENSG000001	-	-	ENSG0000013186	'USP29	-	19:57119137-57131926	-	-	IGV_LINK	0	0	0	0	OK	0	0	0	0	OK	NOTEST	0
ENSG000001	-	-	ENSG0000013187	'SELENOS	-	15:101270816-101277500	-	-	IGV_LINK	734.078	43.2279	15.7737	70.6821	OK	842.511	49.6775	18.4022	80.9528	OK	OK	0.200629
ENSG000001	-	-	ENSG0000013187	'CHSY1	-	15:101175722-101251932	-	-	IGV_LINK	524.619	10.4677	2.68947	18.2459	OK	707.42	14.1866	4.71739	23.6558	OK	OK	0.438587
ENSG000001	-	-	ENSG0000013187	'SNRPA1	-	15:101281509-101295282	-	-	IGV_LINK	623.53	36.4349	8.72087	64.149	OK	557.893	30.626	10.0896	51.1623	OK	OK	-0.25057
ENSG000001	-	-	ENSG0000013188	'KRT17P1	-	17:16840742-16845883	-	-	IGV_LINK	0	0	0	0	OK	0	0	0	0	OK	NOTEST	0
ENSG000001	-	-	ENSG0000013189	'LLGL1	-	17:18225586-18258916	-	-	IGV_LINK	505.224	6.92172	0	18.1663	OK	544.487	7.12094	0	18.4388	OK	OK	0.040938
ENSG000001	-	-	ENSG0000013191	'NR0B2	-	1:26890487-26946862	-	-	IGV_LINK	0	0	0	0	OK	0	0	0	0	OK	NOTEST	0
ENSG000001	-	-	ENSG0000013191	'LIN28A	-	1:26410777-26429722	-	-	IGV_LINK	2.90916	0.042745	0	0.122356	OK	1.03122	0.017694	0	0.074704	OK	NOTEST	-1.27252
ENSG000001	-	-	ENSG0000013193	'THAP1	-	8:42836673-42843325	-	-	IGV_LINK	130.912	3.72172	0.639078	6.80436	OK	100.029	3.05025	0.002242	6.09827	OK	OK	-0.28704
ENSG000001	-	-	ENSG0000013194	'RHPN2	-	19:32978592-33064888	-	-	IGV_LINK	712.745	16.5593	1.54773	31.5708	OK	596.048	15.1728	0.84021	29.5053	OK	OK	-0.12615
ENSG000001	-	-	ENSG0000013194	'C19orf12	-	19:29698885-29715789	-	-	IGV_LINK	575.044	15.6203	3.41684	27.8238	OK	765.169	21.7187	4.73976	38.6976	OK	OK	0.47551
ENSG000001	-	-	ENSG0000013194	'FAAP24	-	19:32972208-32978222	-	-	IGV_LINK	32.0008	1.1541	0	2.67597	OK	79.4043	3.77085	0	7.84915	OK	OK	1.70813
ENSG000001	-	-	ENSG0000013195	'LRRC9	-	14:59919422-60091783	-	-	IGV_LINK	0	0	0	0	OK	0	0	0	0	OK	NOTEST	0
ENSG000001	-	-	ENSG0000013196	'ACTR10	-	14:57999734-58298139	-	-	IGV_LINK	597.348	28.6426	0	68.3381	OK	632.141	28.3941	0	60.3005	OK	OK	-0.01257
ENSG000001	-	-	ENSG0000013196	'ABHD12B	-	14:50848121-50944736	-	-	IGV_LINK	2.90916	0.13969	0	1.82479	OK	0	0	0	0	OK	NOTEST	'-inf
ENSG000001	-	-	ENSG0000013197	'GCH1	-	14:54842007-54902852	-	-	IGV_LINK	324.856	6.708	2.18877	11.2272	OK	275.337	6.27689	1.58341	10.9704	OK	OK	-0.09583
ENSG000001	-	-	ENSG0000013198	'LGALS3	-	14:55124109-55145413	-	-	IGV_LINK	1611.68	98.1776	40.6581	155.697	OK	2554.34	156.628	62.1475	251.108	OK	OK	0.673876
ENSG000001	-	-	ENSG0000013198	'UBE2L2	-	12:20361731-20684381	-	-	IGV_LINK	0	0	0	0	OK	0	0	0	0	OK	NOTEST	0
ENSG000001	-	-	ENSG0000013200	'PODNL1	-	19:13931186-13961449	-	-	IGV_LINK	2.90916	0.135142	0	1.47998	OK	13.4059	1.07827	0	6.22065	OK	OK	2.99617
ENSG000001	-	-	ENSG0000013200	'DNAJB1	-	19:14514769-14565980	-	-	IGV_LINK	1068.63	30.1837	4.57019	55.7972	OK	1613.87	48.6173	6.45573	90.7789	OK	OK	0.6877
ENSG000001	-	-	ENSG0000013200	'ZSWIM4	-	19:13795459-13832230	-	-	IGV_LINK	230.793	3.65397	0.747505	6.56043	OK	271.212	4.37329	1.2238	7.52278	OK	OK	0.259255
ENSG000001	-	-	ENSG0000013200	'FBXW9	-	19:12687997-12696643	-	-	IGV_LINK	239.521	8.74513	2.44954	15.0407	OK	206.245	7.54812	2.03993	13.0563	OK	OK	-0.21236
ENSG000001	-	-	ENSG0000013200	'RFX1	-	19:13961537-14007039	-	-	IGV_LINK	318.068	4.87761	0.427859	9.32737	OK	365.054	5.80344	0.671954	10.9349	OK	OK	0.250734
ENSG000001	-	-	ENSG0000013201	'ZNF20	-	19:12092262-12156731	-	-	IGV_LINK	61.9317	3.79832	0	11.157	OK	23.0817	1.80345	0	7.2126	OK	OK	-1.0746
ENSG000001	-	-	ENSG0000013201	'C19orf57	-	19:13882347-13930879	-	-	IGV_LINK	34.9099	1.63284	0	7.8192	OK	25.7806	1.70625	0	8.98294	OK	OK	0.063445
ENSG000001	-	-	ENSG0000013201	'DCAF15	-	19:13931186-13961449	-	-	IGV_LINK	505.224	21.9523	3.29461	40.61	OK	587.798	27.0634	4.5858	49.541	OK	OK	0.301971
ENSG000001	-	-	ENSG0000013202	'CC2D1A	-	19:13882347-13930879	-	-	IGV_LINK	813.596	20.832	4.96495	36.699	OK	973.476	26.1465	6.29326	45.9997	OK	OK	0.327817
ENSG000001	-	-	ENSG0000013202	'RTBDN	-	19:12763002-12874951	-	-	IGV_LINK	0	0	0	0	OK	2.06245	0.193202	0	7.91207	OK	NOTEST	inf
ENSG000001	-	-	ENSG0000013203	'MATN3	-	2:19990216-20012694	-	-	IGV_LINK	2.90916	0.069722	0	0.287754	OK	10.3122	0.368154	0	0.893108	OK	NOTEST	2.40063
ENSG000001	-	-	ENSG0000013210	'TRIM21	-	11:4384896-4393696	-	-	IGV_LINK	843.657	27.1455	11.4488	42.8422	OK	728.045	24.5637	9.4147	39.7127	OK	OK	-0.14418
ENSG000001	-	-	ENSG0000013212	'SPATA6	-	1:48295371-48472208	-	-	IGV_LINK	314.189	5.24089	0.455459	10.0263	OK	287.712	7.40809	0	16.8662	OK	OK	0.499291
ENSG000001	-	-	ENSG0000013212	'LRRC41	-	1:46247687-46303608	-	-	IGV_LINK	4474.29	89.3861	33.81	144.962	OK	3387.57	69.1259	27.652	110.6	OK	OK	-0.37083
ENSG000001	-	-	ENSG0000013214	'CCT6B	-	17:34927858-35121522	-	-	IGV_LINK	45.5769	1.66745	0	8.54705	OK	80.4355	2.58889	0	11.1171	OK	OK	0.634695
ENSG000001	-	-	ENSG0000013215	'DHX30	-	3:47802908-47850195	-	-	IGV_LINK	823.293	15.0576	4.26414	25.8511	OK	984.82	18.2201	5.05263	31.3876	OK	OK	0.275038
ENSG000001	-	-	ENSG0000013215	'RAF1	-	3:12484431-12664226	-	-	IGV_LINK	1489.49	34.2904	8.45058	60.1302	OK	1939.73	44.2627	11.736	76.7895	OK	OK	0.368288
ENSG000001	-	-	ENSG0000013216	'SLC6A11	-	3:10816199-10940733	-	-	IGV_LINK	0.969721	0.137489	0	0.540889	OK	3.09367	0.042293	0	0.25711	OK	NOTEST	-1.70083
ENSG000001	-	-	ENSG0000013217	'PPARG	-	3:12287367-12434356	-	-	IGV_LINK	235.642	8.27343	2.31502	14.2318	OK	142.309	4.98566	0.826734	9.14458	OK	OK	-0.7307
ENSG000001	-	-	ENSG0000013218	'NUP210	-	3:13316234-13420309	-	-	IGV_LINK	1326.58	11.0397	4.37228	17.7071	OK	1086.91	8.93649	3.6037	14.2693	OK	OK	-0.30492
ENSG000001	-	-	ENSG0000013218	'FCRLA	-	1:161706971-161714352	-	-	IGV_LINK	17.455	0.542097	0	1.302	OK	37.1241	1.50991	0	3.37667	OK	OK	1.47784
ENSG000001	-	-	ENSG0000013219	'HSD17B7	-	1:162790701-162812817	-	-	IGV_LINK	236.612	10.8583	0.835073	20.8815	OK	588.829	28.4467	7.36553	49.5278	OK	OK	1.38946
ENSG000001	-	-	ENSG0000013219	'ENOSF1	-	18:596987-712676	-	-	IGV_LINK	3038.13	81.7723	0	173.479	OK	2855.46	73.3741	0	151.727	OK	OK	-0.15634
ENSG000001	-	-	ENSG0000013220	'LINC00470	-	18:1254382-1408344	-	-	IGV_LINK	1.93944	0.178406	0	0.956715	OK	2.06245	0.106109	0	0.833306	OK	NOTEST	-0.74962
ENSG000001	-	-	ENSG0000013220	'EMILIN2	-	18:2847029-2915993	-	-	IGV_LINK	654.561	17.9148	4.51579	31.3139	OK	1486	32.0904	8.75906	55.4218	OK	OK	0.840987
ENSG000001	-	-	ENSG0000013220	'SLX1A	-	16:30192933-30204310	-	-	IGV_LINK	15.4385	0.876685	0	2.90402	OK	26.3345	1.36257	0	3.83727	OK	OK	0.636195
ENSG000001	-	-	ENSG0000013225	'ARFIP2	-	11:6474682-6593758	-	-	IGV_LINK	974.569	40.7277	0	83.097	OK	1039.47	43.5001	2.63544	84.3648	OK	OK	0.095008
ENSG000001	-	-	ENSG0000013225	'TRIM5	-	11:5663556-5938619	-	-	IGV_LINK	537.225	13.5021	0	44.5189	OK	591.923	15.6208	0	46.6847	OK	OK	0.210289
ENSG000001	-	-	ENSG0000013225	'CNGA4	-	11:6234764-6244429	-	-	IGV_LINK	7.75776	0.258621	0	0.619177	OK	6.18735	0.206409	0	0.527686	OK	NOTEST	-0.32534
ENSG000001	-	-	ENSG0000013227	'TRIM22	-	11:5663556-5938619	-	-	IGV_LINK	4288.84	147.188	27.7446	266.631	OK	3805.22	127.914	24.3363	231.492	OK	OK	-0.20248
ENSG000001	-	-	ENSG0000013227	'RRP8	-	11:6595074-6603620	-	-	IGV_LINK	385.949	12.0713	0.723788	23.4188	OK	439.302	18.4638	3.11944	33.8081	OK	OK	0.613117
ENSG000001	-	-	ENSG0000013228	'TIMM10B	-	11:6474682-6593758	-	-	IGV_LINK	415.71	11.9422	0	31.9046	OK	491.894	14.5602	0	38.7573	OK	OK	0.28596
ENSG000001	-	-	ENSG0000013229	'EFR3A	-	8:131904087-132013642	-	-	IGV_LINK	1445.85	22.8808	4.21435	41.5473	OK	1624.18	24.3275	4.5891	44.0658	OK	OK	0.088446
ENSG000001	-	-	ENSG0000013229	'HHLA1	-	8:132024219-132111159	-	-	IGV_LINK	4.8486	0.130398	0	0.353011	OK	7.21857	0.194242	0	0.463325	OK	NOTEST	0.574937
ENSG000001	-	-	ENSG0000013230	'PTCD3	-	2:86106181-86142157	-	-	IGV_LINK	1084.15	36.3418	1.63808	71.0455	OK	1068.35	34.5041	0	74.9139	OK	OK	-0.07486
ENSG000001	-	-	ENSG0000013230	'IMMT	-	2:86143931-86196049	-	-	IGV_LINK	1217.97	29.0273	11.534	46.5206	OK	1472.59	34.4579	13.6842	55.2317	OK	OK	0.247426
ENSG000001	-	-	ENSG0000013231	'MRPL35	-	2:86199354-86213794	-	-	IGV_LINK	456.738	18.3327	2.07893	34.5864	OK	537.268	23.9455	2.19392	45.6971	OK	OK	0.38534
ENSG000001	-	-	ENSG0000013232	'IQCA1	-	2:236324146-236507542	-	-	IGV_LINK	195.884	5.31574	0	10.6953	OK	279.462	7.3876	0.39234	14.3829	OK	OK	0.474834
ENSG000001	-	-	ENSG0000013232	'ILKAP	-	2:238170400-238203729	-	-	IGV_LINK	448.011	25.2651	7.45838	43.0718	OK	341.335	19.2361	5.27608	33.1961	OK	OK	-0.39333
ENSG000001	-	-	ENSG0000013232	'PER2	-	2:238231683-238300620	-	-	IGV_LINK	640.016	6.34978	0.738078	11.9615	OK	677.515	6.9756	0.25987	13.6913	OK	OK	0.135611
ENSG000001	-	-	ENSG0000013232	'RAMP1	-	2:237858892-237912114	-	-	IGV_LINK	11.6366	0.937189	0	2.11832	OK	13.4059	1.02604	0	2.38397	OK	OK	0.130677
ENSG000001	-	-	ENSG0000013233	'SCLY	-	2:237966826-238099413	-	-	IGV_LINK	447.883	13.5125	0	29.2282	OK	247.472	6.49263	0	15.8526	OK	OK	-1.05742
ENSG000001	-	-	ENSG0000013233	'PTPRE	-	10:127907060-128085855	-	-	IGV_LINK	1124.88	16.4509	0	36.2853	OK	933.259	11.6621	1.23222	22.092	OK	OK	-0.49634
ENSG000001	-	-	ENSG0000013234	'RAN	-	12:130871878-130877678	-	-	IGV_LINK	2290.48	196.306	62.5861	330.025	OK	2701.81	233.627	85.1128	382.141	OK	OK	0.251106
ENSG000001	-	-	ENSG0000013235	'PRKAA1	-	5:40759378-40798374	-	-	IGV_LINK	936.75	27.1948	6.13195	48.2576	OK	1112.69	30.1264	5.94095	54.3119	OK	OK	0.147699
ENSG000001	-	-	ENSG0000013235	'CARD6	-	5:40841183-40860175	-	-	IGV_LINK	207.52	2.88871	0.78046	4.99696	OK	263.994	3.67587	1.14027	6.21148	OK	OK	0.347661
ENSG000001	-	-	ENSG0000013235	'RAP1GAP2	-	17:2755704-3037739	-	-	IGV_LINK	457.708	5.029	0	10.9003	OK	217.588	2.47087	0	5.82399	OK	OK	-1.02525
ENSG000001	-	-	ENSG0000013236	'CLUH	-	17:2689385-2712833	-	-	IGV_LINK	999.782	18.9684	0	38.2759	OK	1663.37	30.1365	0	60.6521	OK	OK	0.667913
ENSG000001	-	-	ENSG0000013237	'INPP5K	-	17:1494570-1516888	-	-	IGV_LINK	640.016	19.0429	3.28329	34.8024	OK	630.078	19.6656	2.56844	36.7627	OK	OK	0.046422
ENSG000001	-	-	ENSG0000013238	'MYBBP1A	-	17:4497393-4555631	-	-	IGV_LINK	544.013	13.013	0	29.0121	OK	583.673	11.3339	0.399861	22.2679	OK	OK	-0.19931
ENSG000001	-	-	ENSG0000013238	'RPA1	-	17:1779484-1900082	-	-	IGV_LINK	1025.96	24.5741	0	49.913	OK	965.227	24.8081	0	52.929	OK	OK	0.013669
ENSG000001	-	-	ENSG0000013238	'SERPINF1	-	17:1761958-1777574	-	-	IGV_LINK	617.712	62.0415	21.265	102.818	OK	678.546	50.9995	15.9698	86.0292	OK	OK	-0.28275
ENSG000001	-	-	ENSG0000013238	'UBE2G1	-	17:4269258-4366628	-	-	IGV_LINK	283.158	9.97626	0	20.4295	OK	299.055	10.6808	0.340589	21.021	OK	OK	0.098447
ENSG000001	-	-	ENSG0000013239	'EEFSEC	-	3:128051640-128408646	-	-	IGV_LINK	354.918	10.2131	0	33.1204	OK	329.992	9.11407	0	33.915	OK	OK	-0.16425
ENSG000001	-	-	ENSG0000013240	'TBC1D14	-	4:6909241-7057952	-	-	IGV_LINK	593.469	11.1583	0	30.9041	OK	808.48	16.9043	0	48.4804	OK	OK	0.599272
ENSG000001	-	-	ENSG0000013240	'TMEM128	-	4:4235541-4248212	-	-	IGV_LINK	299.644	15.4533	4.97015	25.9364	OK	374.335	19.6747	6.89588	32.4535	OK	OK	0.348428
ENSG000001	-	-	ENSG0000013242	'COQ3	-	6:99369399-99394204	-	-	IGV_LINK	77.5776	3.99804	0	8.10757	OK	53.6237	3.07529	0	6.33622	OK	OK	-0.37857
ENSG000001	-	-	ENSG0000013242	'PNISR	-	6:99398050-99431373	-	-	IGV_LINK	5207.4	107.412	34.5179	180.307	OK	2515.16	52.8688	15.3994	90.3382	OK	OK	-1.02267
ENSG000001	-	-	ENSG0000013242	'POPDC3	-	6:105096821-105179995	-	-	IGV_LINK	2.90916	0.226681	0	1.25809	OK	0	0	0	0	OK	NOTEST	'-inf
ENSG000001	-	-	ENSG0000013243	'SEC61G	-	7:54752249-54804928	-	-	IGV_LINK	432.495	80.4525	29.0161	131.889	OK	626.985	118.448	47.3339	189.561	OK	OK	0.558042
ENSG000001	-	-	ENSG0000013243	'LANCL2	-	7:55365447-55433742	-	-	IGV_LINK	293.825	6.69647	0	13.7506	OK	334.117	7.10836	1.05243	13.1643	OK	OK	0.086118
ENSG000001	-	-	ENSG0000013243	'FIGNL1	-	7:50444127-50565457	-	-	IGV_LINK	157.095	3.12379	0	8.03457	OK	129.934	2.36921	0	7.31311	OK	OK	-0.39889
ENSG000001	-	-	ENSG0000013243	'DDC	-	7:50444127-50565457	-	-	IGV_LINK	343.281	10.7631	2.03092	19.4954	OK	1113.72	34.0749	12.7892	55.3607	OK	OK	1.66261
ENSG000001	-	-	ENSG0000013244	'FTHL17	-	X:31071240-31072053	-	-	IGV_LINK	0	0	0	0	OK	0	0	0	0	OK	NOTEST	0
ENSG000001	-	-	ENSG0000013246	'GRSF1	-	4:70815781-70839945	-	-	IGV_LINK	1288.76	33.7824	10.5721	56.9926	OK	1507.65	41.2527	14.0586	68.4468	OK	OK	0.288218
ENSG000001	-	-	ENSG0000013246	'ENAM	-	4:70628743-70686816	-	-	IGV_LINK	101.821	2.41676	0	15.6239	OK	51.5612	1.17541	0	10.2746	OK	OK	-1.03991
ENSG000001	-	-	ENSG0000013246	'JCHAIN	-	4:70628743-70686816	-	-	IGV_LINK	2146.96	183.288	63.3001	303.277	OK	1611.8	144.399	54.438	234.36	OK	OK	-0.34405
ENSG000001	-	-	ENSG0000013246	'ANKRD17	-	4:73073375-73258798	-	-	IGV_LINK	1769.74	20.7495	0	48.7251	OK	2091.32	22.552	0	53.1263	OK	OK	0.120179
ENSG000001	-	-	ENSG0000013246	'UTP3	-	4:70688478-70690551	-	-	IGV_LINK	498.436	14.7803	5.57269	23.9879	OK	900.259	26.7119	11.303	42.1207	OK	OK	0.853805
ENSG000001	-	-	ENSG0000013247	'ITGB4	-	17:75721327-75765711	-	-	IGV_LINK	543.043	9.65202	0	24.5359	OK	308.336	5.5481	0	18.4618	OK	OK	-0.79884

ENSG000001	-	-	ENSG0000013247	'WBP2	-	17:75845698-75856507	-	-	IGV_LINK	3097.29	108.397	37.2454	179.548	OK	3105.02	106.218	39.2792	173.158	OK	OK	-0.02929
ENSG000001	-	-	ENSG0000013247	'H3F3B	-	17:75776433-75825799	-	-	IGV_LINK	5074.55	346.991	51.3091	642.673	OK	4690.01	353.774	83.8953	623.652	OK	OK	0.027929
ENSG000001	-	-	ENSG0000013247	'UNK	-	17:75776433-75825799	-	-	IGV_LINK	704.987	16.9649	0	60.6014	OK	655.859	13.3405	0	46.0049	OK	OK	-0.34675
ENSG000001	-	-	ENSG0000013248	'TRIM47	-	17:75874160-75879546	-	-	IGV_LINK	556.62	26.9169	6.03462	47.7992	OK	732.17	35.9397	7.44759	64.4317	OK	OK	0.417063
ENSG000001	-	-	ENSG0000013248	'ZRANB2	-	1:71048854-71081297	-	-	IGV_LINK	1449.73	50.829	8.67117	92.9869	OK	1285.94	45.0258	8.25063	81.801	OK	OK	-0.1749
ENSG000001	-	-	ENSG0000013250	'EIF5A	-	17:7306998-7329393	-	-	IGV_LINK	1643.68	80.7131	17.3748	144.051	OK	1611.8	78.9912	16.1542	141.828	OK	OK	-0.03111
ENSG000001	-	-	ENSG0000013251	'KDM6B	-	17:7839903-7854796	-	-	IGV_LINK	653.592	6.93823	0	14.4088	OK	729.076	8.14065	0	24.9863	OK	OK	0.230576
ENSG000001	-	-	ENSG0000013251	'CLEC10A	-	17:7074536-7080307	-	-	IGV_LINK	154.186	6.82013	0.589996	13.0503	OK	389.803	15.1853	4.66678	25.7039	OK	OK	1.1548
ENSG000001	-	-	ENSG0000013251	'SLC52A1	-	17:5032599-5052009	-	-	IGV_LINK	2.90916	0.064774	0	0.211467	OK	9.28102	0.219624	0	0.522833	OK	NOTEST	1.76156
ENSG000001	-	-	ENSG0000013251	'GUCY2D	-	17:8002593-8020339	-	-	IGV_LINK	0	0	0	0	OK	10.3122	1.28493	0	3.70804	OK	OK	inf
ENSG000001	-	-	ENSG0000013252	'GPS2	-	17:7306998-7329393	-	-	IGV_LINK	678.804	38.9096	0	89.3576	OK	625.732	32.6456	0	75.1038	OK	OK	-0.25324
ENSG000001	-	-	ENSG0000013253	'XAF1	-	17:6755446-6776116	-	-	IGV_LINK	1025.96	76.3114	0	168.546	OK	841.48	67.6169	0	147.058	OK	OK	-0.17452
ENSG000001	-	-	ENSG0000013253	'DLG4	-	17:7189889-7225273	-	-	IGV_LINK	313.22	8.97681	0	42.1484	OK	300.086	9.2888	0	53.5223	OK	OK	0.049289
ENSG000001	-	-	ENSG0000013254	'RIDA	-	8:98102343-98117241	-	-	IGV_LINK	962.932	64.073	26.153	101.993	OK	2165.57	142.811	60.5057	225.117	OK	OK	1.15632
ENSG000001	-	-	ENSG0000013254	'VPS13B	-	8:99013265-99894062	-	-	IGV_LINK	1284.88	9.4514	0	21.3914	OK	939.446	8.56277	0	21.6118	OK	OK	-0.14245
ENSG000001	-	-	ENSG0000013255	'RGS22	-	8:99960935-100131268	-	-	IGV_LINK	76.6079	2.28429	0	6.12175	OK	85.5917	1.51863	0	3.28221	OK	OK	-0.58897
ENSG000001	-	-	ENSG0000013256	'MATN2	-	8:97868839-98046469	-	-	IGV_LINK	127.033	2.68988	0	30.7502	OK	254.713	4.89681	0	32.3386	OK	OK	0.8643
ENSG000001	-	-	ENSG0000013256	'REEP2	-	5:138439016-138446969	-	-	IGV_LINK	10.6669	0.327839	0	1.10659	OK	4.1249	0.137765	0	0.801697	OK	NOTEST	-1.25078
ENSG000001	-	-	ENSG0000013257	'PCBD2	-	5:134904905-135011707	-	-	IGV_LINK	378.779	9.53887	0	20.1632	OK	413.67	13.5315	0	27.7549	OK	OK	0.504431
ENSG000001	-	-	ENSG0000013258	'SDF2	-	17:28648355-28702684	-	-	IGV_LINK	497.467	34.0646	0	93.2248	OK	520.769	33.0361	0	90.6924	OK	OK	-0.04423
ENSG000001	-	-	ENSG0000013258	'FLOT2	-	17:28879334-28897679	-	-	IGV_LINK	2636.67	66.0428	23.0011	109.085	OK	3139.05	78.1091	23.5899	132.628	OK	OK	0.242088
ENSG000001	-	-	ENSG0000013259	'ERAL1	-	17:28755977-28861067	-	-	IGV_LINK	719.533	26.9159	6.55675	47.2751	OK	937.384	33.3971	8.77861	58.0155	OK	OK	0.311261
ENSG000001	-	-	ENSG0000013260	'PRMT7	-	16:68310973-68448688	-	-	IGV_LINK	623.53	28.7603	3.14763	54.373	OK	512.519	23.798	2.37751	45.2186	OK	OK	-0.27324
ENSG000001	-	-	ENSG0000013260	'NIP7	-	16:69105563-69408571	-	-	IGV_LINK	252.127	7.86222	0	40.2642	OK	338.242	12.1432	0	62.5704	OK	OK	0.627137
ENSG000001	-	-	ENSG0000013260	'TERF2	-	16:69105563-69408571	-	-	IGV_LINK	465.466	10.254	0	37.3046	OK	465.082	11.4947	0	44.2929	OK	OK	0.164782
ENSG000001	-	-	ENSG0000013261	'VPS4A	-	16:69105563-69408571	-	-	IGV_LINK	1371.4	23.3979	0	61.5073	OK	1430.86	23.7283	0	62.6595	OK	OK	0.020231
ENSG000001	-	-	ENSG0000013261	'MTSS1L	-	16:70661203-70686066	-	-	IGV_LINK	215.278	7.54455	0	19.6216	OK	152.621	6.85887	0	17.6963	OK	OK	-0.13746
ENSG000001	-	-	ENSG0000013262	'HSPA12B	-	20:3732666-3753111	-	-	IGV_LINK	158.064	2.99079	0.660455	5.32113	OK	202.12	3.82584	1.02353	6.62814	OK	OK	0.355247
ENSG000001	-	-	ENSG0000013262	'ANKEF1	-	20:9986087-10368776	-	-	IGV_LINK	143.519	1.99175	0	4.33517	OK	272.243	4.09585	0	8.41891	OK	OK	1.04013
ENSG000001	-	-	ENSG0000013263	'SCP2D1	-	20:18809727-18830153	-	-	IGV_LINK	0	0	0	0	OK	0	0	0	0	OK	NOTEST	0
ENSG000001	-	-	ENSG0000013263	'PCED1A	-	20:2835313-3039076	-	-	IGV_LINK	1159.79	38.2757	0	81.689	OK	1129.19	36.8627	0	76.4952	OK	OK	-0.05427
ENSG000001	-	-	ENSG0000013263	'SNAP25	-	20:9986087-10368776	-	-	IGV_LINK	208.49	5.89926	0.823042	10.9755	OK	220.682	6.90429	0	14.9446	OK	OK	0.226959
ENSG000001	-	-	ENSG0000013264	'BTBD3	-	20:11890722-11926609	-	-	IGV_LINK	546.922	6.84933	1.57779	12.1209	OK	504.269	9.20695	0	19.3264	OK	OK	0.426759
ENSG000001	-	-	ENSG0000013264	'PCNA	-	20:5114952-5126626	-	-	IGV_LINK	746.685	35.8768	14.9047	56.8488	OK	752.794	36.191	14.0877	58.2942	OK	OK	0.01258
ENSG000001	-	-	ENSG0000013266	'NXT1	-	20:23346940-23354777	-	-	IGV_LINK	140.609	8.33701	1.57304	15.101	OK	156.746	9.30505	2.03935	16.5707	OK	OK	0.158484
ENSG000001	-	-	ENSG0000013266	'POLR3F	-	20:18383366-18484643	-	-	IGV_LINK	162.913	4.37271	0	9.02316	OK	185.62	4.85643	0	9.9328	OK	OK	0.151367
ENSG000001	-	-	ENSG0000013266	'RIN2	-	20:19886520-20002457	-	-	IGV_LINK	719.533	14.154	1.4763	26.8317	OK	1012.66	18.9123	5.2232	32.6015	OK	OK	0.418117
ENSG000001	-	-	ENSG0000013267	'PTPRA	-	20:2835313-3039076	-	-	IGV_LINK	1491.84	29.8852	0.542722	59.2277	OK	1375.67	28.5585	0	58.3701	OK	OK	-0.06551
ENSG000001	-	-	ENSG0000013267	'SSTR4	-	20:23031541-23039236	-	-	IGV_LINK	0	0	0	0	OK	0	0	0	0	OK	NOTEST	0
ENSG000001	-	-	ENSG0000013267	'DAP3	-	1:155609775-155739010	-	-	IGV_LINK	1187.91	57.6331	0	119.661	OK	1290.06	65.4052	0.056435	130.754	OK	OK	0.182508
ENSG000001	-	-	ENSG0000013267	'RHBG	-	1:156369211-156385219	-	-	IGV_LINK	0.969721	0.031372	0	0.473358	OK	0	0	0	0	OK	NOTEST	'-inf
ENSG000001	-	-	ENSG0000013268	'KIAA0907	-	1:155913042-155934400	-	-	IGV_LINK	2010.23	55.4903	7.91729	103.063	OK	2003.67	57.7976	8.53886	107.056	OK	OK	0.058775
ENSG000001	-	-	ENSG0000013268	'ATP1A4	-	1:160151569-160186977	-	-	IGV_LINK	12.6064	0.192459	0	0.537656	OK	8.2498	0.16663	0	0.539477	OK	NOTEST	-0.2079
ENSG000001	-	-	ENSG0000013268	'NES	-	1:156668762-156677397	-	-	IGV_LINK	806.807	8.44269	3.53391	13.3515	OK	562.018	5.88239	2.32524	9.43954	OK	OK	-0.5213
ENSG000001	-	-	ENSG0000013269	'BCAN	-	1:156641389-156661424	-	-	IGV_LINK	6.78804	0.116157	0	0.712253	OK	10.3122	0.177141	0	1.07515	OK	NOTEST	0.608826
ENSG000001	-	-	ENSG0000013269	'CRP	-	1:159712288-159714589	-	-	IGV_LINK	0	0	0	0	OK	2.06245	0.094424	0	0.622853	OK	NOTEST	inf
ENSG000001	-	-	ENSG0000013269	'ARHGEF11	-	1:156934839-157045370	-	-	IGV_LINK	1024.02	10.9838	1.47962	20.4879	OK	1204.47	16.032	0.387097	31.6769	OK	OK	0.545583
ENSG000001	-	-	ENSG0000013269	'RAB25	-	1:156061159-156070514	-	-	IGV_LINK	0	0	0	0	OK	0	0	0	0	OK	NOTEST	0
ENSG000001	-	-	ENSG0000013270	'HAPLN2	-	1:156619293-156625725	-	-	IGV_LINK	6.78804	0.321684	0	0.858784	OK	1.03122	0.106567	0	0.503135	OK	NOTEST	-1.59389
ENSG000001	-	-	ENSG0000013270	'APCS	-	1:159587824-159588865	-	-	IGV_LINK	0	0	0	0	OK	4.1249	0.30899	0	0.782754	OK	NOTEST	inf
ENSG000001	-	-	ENSG0000013270	'FCRL2	-	1:157745732-157777132	-	-	IGV_LINK	12.6064	0.289959	0	0.720403	OK	53.6237	0.966921	0	1.98431	OK	OK	1.73755
ENSG000001	-	-	ENSG0000013271	'DCAF8	-	1:160215714-160286348	-	-	IGV_LINK	4013.44	127.579	5.13112	250.027	OK	3492.51	110.454	0	228.845	OK	OK	-0.20794
ENSG000001	-	-	ENSG0000013271	'SYT11	-	1:155859508-155885199	-	-	IGV_LINK	620.621	6.90192	2.78439	11.0195	OK	512.519	5.70101	2.14825	9.25377	OK	OK	-0.27578
ENSG000001	-	-	ENSG0000013274	'IGHMBP2	-	11:68903841-68940602	-	-	IGV_LINK	336.493	7.90896	0	16.6344	OK	412.49	9.77462	0	20.8763	OK	OK	0.305553
ENSG000001	-	-	ENSG0000013274	'ACY3	-	11:67642554-67650659	-	-	IGV_LINK	583.772	30.5631	11.9032	49.2231	OK	437.239	22.5278	8.2755	36.7802	OK	OK	-0.44008
ENSG000001	-	-	ENSG0000013274	'ALDH3B2	-	11:67662161-67681200	-	-	IGV_LINK	0	0	0	0	OK	0	0	0	0	OK	NOTEST	0
ENSG000001	-	-	ENSG0000013274	'TESMIN	-	11:68707439-68751564	-	-	IGV_LINK	86.3051	3.29013	0	7.7645	OK	111.372	5.05372	0	10.8313	OK	OK	0.619204
ENSG000001	-	-	ENSG0000013276	'MMACHC	-	1:45493865-45523047	-	-	IGV_LINK	103.76	1.63966	0	8.90455	OK	97.9664	1.5043	0	11.5439	OK	OK	-0.1243
ENSG000001	-	-	ENSG0000013276	'DPH2	-	1:43969999-43973369	-	-	IGV_LINK	417.95	14.0893	3.1369	25.0417	OK	478.488	16.3291	3.36073	29.2975	OK	OK	0.212848
ENSG000001	-	-	ENSG0000013277	'TOE1	-	1:45329162-45491166	-	-	IGV_LINK	236.612	6.71754	0	14.7371	OK	244.4	6.62383	0	13.4015	OK	OK	-0.02027
ENSG000001	-	-	ENSG0000013278	'NASP	-	1:45583845-45618904	-	-	IGV_LINK	2322.48	77.7595	16.8068	138.712	OK	1885.08	63.896	17.1727	110.619	OK	OK	-0.28329
ENSG000001	-	-	ENSG0000013278	'MUTYH	-	1:45329162-45491166	-	-	IGV_LINK	262.794	16.3636	0	34.0213	OK	217.588	12.7115	0	26.4354	OK	OK	-0.36436
ENSG000001	-	-	ENSG0000013279	'CTNNBL1	-	20:37693954-37872129	-	-	IGV_LINK	637.106	22.4246	7.08068	37.7685	OK	587.798	20.6719	6.42589	34.918	OK	OK	-0.11741
ENSG000001	-	-	ENSG0000013279	'LPIN3	-	20:41340919-41366827	-	-	IGV_LINK	2081.02	47.9707	0.695022	95.2463	OK	3102.96	72.8992	0.013688	145.785	OK	OK	0.603751
ENSG000001	-	-	ENSG0000013280	'ZSWIM3	-	20:45857616-45879122	-	-	IGV_LINK	75.6382	1.63917	0	3.28503	OK	113.435	2.45935	0.245167	4.67354	OK	OK	0.585314
ENSG000001	-	-	ENSG0000013281	'RBM38	-	20:57384159-57409333	-	-	IGV_LINK	358.797	10.0516	2.69548	17.4077	OK	288.743	7.75316	2.20759	13.2987	OK	OK	-0.37457
ENSG000001	-	-	ENSG0000013282	'VSTM2L	-	20:37903103-37945350	-	-	IGV_LINK	0.969721	0.113034	0	0.473213	OK	180.464	6.08776	1.14418	11.0313	OK	OK	5.75108
ENSG000001	-	-	ENSG0000013282	'OSER1	-	20:44196495-44210791	-	-	IGV_LINK	921.234	37.07	15.6497	58.4902	OK	1120.94	45.1105	19.6515	70.5694	OK	OK	0.283211
ENSG000001	-	-	ENSG0000013282	'SERINC3	-	20:44496220-44522109	-	-	IGV_LINK	2365.15	40.8305	12.7759	68.8851	OK	3798	72.4305	14.2657	130.595	OK	OK	0.826951
ENSG000001	-	-	ENSG0000013282	'PPP1R3D	-	20:59933763-59948680	-	-	IGV_LINK	159.034	2.59219	0.013351	5.17103	OK	170.152	2.77433	0.030436	5.51822	OK	OK	0.097969
ENSG000001	-	-	ENSG0000013283	'LINC01260	-	20:44656450-44810338	-	-	IGV_LINK	2.76797	0.125447	0	0.488014	OK	1.17E-08	5.30E-10	0	0.090726	OK	NOTEST	-27.8172
ENSG000001	-	-	ENSG0000013283	'DMGDH	-	5:78997605-79236038	-	-	IGV_LINK	1123.91	23.7992	0	58.3135	OK	1521.06	32.0792	0	87.5286	OK	OK	0.430725
ENSG000001	-	-	ENSG0000013284	'BHMT2	-	5:78997605-79236038	-	-	IGV_LINK	2407.82	106.582	0	236.253	OK	2767.81	112.467	0	282.243	OK	OK	0.077547
ENSG000001	-	-	ENSG0000013284	'AP3B1	-	5:78000524-78294755	-	-	IGV_LINK	897.961	14.5706	2.93868	26.2026	OK	1164.25	18.0967	6.29382	29.8996	OK	OK	0.312664
ENSG000001	-	-	ENSG0000013284	'ZBED3	-	5:77072071-77166909	-	-	IGV_LINK	347.16	3.26101	0.867724	5.65429	OK	545.518	5.53963	0.102316	10.977	OK	OK	0.764474
ENSG000001	-	-	ENSG0000013284	'PATJ	-	1:61742476-62178675	-	-	IGV_LINK	2301.15	22.3156	0.826076	43.8052	OK	2371.82	27.7894	0	65.9489	OK	OK	0.316481
ENSG000001	-	-	ENSG0000013285	'KANK4	-	1:62236978-62319414	-	-	IGV_LINK	17.455	0.282864	0	0.642604	OK	3.09367	0.060787	0	0.195822	OK	NOTEST	-2.21826
ENSG000001	-	-	ENSG0000013285	'ANGPTL3	-	1:62454297-62688368	-	-	IGV_LINK	6.78804	0.145317	0	1.13406	OK	52.5925	1.73736	0	8.3641	OK	OK	3.57962
ENSG000001	-	-	ENSG0000013287	'SYT4	-	18:43267877-43277650	-	-	IGV_LINK	0	0	0	0	OK	3.09367	0.044298	0	0.520437	OK	NOTEST	inf
ENSG000001	-	-	ENSG0000013287	'SLC14A2	-	18:45212994-45752520	-	-	IGV_LINK	0.969721	0.016229	0	0.14349	OK	0	0	0	0	OK	NOTEST	'-inf
ENSG000001	-	-	ENSG0000013287	'FBXO44	-	1:11623557-11663327	-	-	IGV_LINK	1034.69	36.1709	12.3276	60.0142	OK	896.135	32.6115	8.50966	56.7134	OK	OK	-0.14945
ENSG000001	-	-	ENSG0000013288	'RSG1	-	1:16231699-16237162	-	-	IGV_LINK	86.3051	4.76928	0	11.3597	OK	58.7798	3.54823	0	9.00137	OK	OK	-0.42667
ENSG000001	-	-	ENSG0000013290	'CASP9	-	1:15456727-15526534	-	-	IGV_LINK	333.584	10.4689	2.65676	18.2811	OK	457.864	13.7825	4.4166	23.1483	OK	OK	0.396722
ENSG000001	-	-	ENSG0000013291	'NMUR2	-	5:152391531-152433368	-	-	IGV_LINK	0	0	0	0	OK	0	0	0	0	OK	NOTEST	0
ENSG000001	-	-	ENSG0000013291	'DCTN4	-	5:150708439-150759109	-	-	IGV_LINK	1290.7	23.1422	5.35386	40.9306	OK	1472.59	26.4903	6.45349	46.527	OK	OK	0.194934
ENSG000001	-	-	ENSG0000013291	'PDE6A	-	5:149857954-149944793	-	-	IGV_LINK	1.93944	0.020914	0	0.100654	OK	2.06245	0.039958	0	0.134141	OK	NOTEST	0.934005
ENSG000001	-	-	ENSG0000013293	'ATP8A2	-	13:25372070-26025851	-	-	IGV_LINK	83.396	0.499848	0	1.02116	OK	76.3106	0.45744	0	0.950564	OK	NOTEST	-0.12791
ENSG000001	-	-	ENSG0000013293	'MTUS2	-	13:28820347-29505947	-	-	IGV_LINK	9.69721	0.075322	0	0.479244	OK	4.1249	0.032036	0	0.41897	OK	NOTEST	-1.23339
ENSG000001	-	-	ENSG0000013295	'ZMYM5	-	13:19823481-19863636	-	-	IGV_LINK	255.036	5.72815	1.42171	10.0346	OK	232.026	5.84891	1.12059	10.5772	OK	OK	0.030097
ENSG000001	-	-	ENSG0000013295	'USPL1	-	13:30617692-30660770	-	-	IGV_LINK	597.348	10.0177	3.21089	16.8245	OK	527.987	8.64826	2.73928	14.5572	OK	OK	-0.21207
ENSG000001	-	-	ENSG0000013295	'XPO4	-	13:20777328-20903048	-	-	IGV_LINK	492.618	3.07237	0.45061	5.69412	OK	412.49	2.54916	0.395588	4.70274	OK	OK	-0.26933
ENSG000001	-	-	ENSG0000013295	'TPTE2	-	13:19422876-19536762	-	-	IGV_LINK	0.969721	0.039327	0	0.250507	OK	0	0	0	0	OK	NOTEST	'-inf
ENSG000001	-	-	ENSG0000013296	'POMP	-	13:28659103-28678925	-	-	IGV_LINK	950.326	46.256	18.8775	73.6344	OK	1010.6	49.2397	21.2649	77.2144	OK	OK	0.090183
ENSG000001	-	-	ENSG0000013296	'CDK8	-	13:26254103-26405238	-	-	IGV_LINK	159.034	3.29615	0.664992	5.92731	OK	97.9664	2.21467	0.002354	4.42698	OK	OK	-0.57369
ENSG000001	-	-	ENSG0000013296	'ALOX5AP	-	13:30713477-30764426	-	-	IGV_LINK	450.92	34.7355	12.4534	57.0175	OK	512.519	39.3926	15.0824	63.7029	OK	OK	0.181515
ENSG000001	-	-	ENSG0000013296	'HMGB1P5	-	3:22381818-22382929	-	-	IGV_LINK	4.8486	0.468994	0	1.2453	OK	5.15612	0.620459	0	1.53098	OK	OK	0.403767
ENSG000001	-	-	ENSG0000013297	'WASF3	-	13:26557702-26688948	-	-	IGV_LINK	96.0023	1.16739	0	2.40321	OK	98.9976	1.2058	0	2.46236	OK	OK	0.046703
ENSG000001	-	-	ENSG0000013297	'RNF17	-	13:24764151-24879921	-	-	IGV_LINK	0	0	0	0	OK	0	0	0	0	OK	NOTEST	0
ENSG000001	-	-	ENSG0000013297	'GPR12	-	13:26755199-26760785	-	-	IGV_LINK	0	0	0	0	OK	0	0	0	0	OK	NOTEST	0
ENSG000001	-	-	ENSG0000013301	'CHRM3	-	1:239386564-239916955	-	-	IGV_LINK	20.3641	0.197356	0	0.76062	OK	16.4996	0.131374	0	0.655552	OK	NOTEST	-0.58712
ENSG000001	-	-	ENSG0000013302	'MYH8	-	17:10383131-10623886	-	-	IGV_LINK	0.969721	0.009312	0	0.036474	OK	0	0	0	0	OK	NOTEST	'-inf
ENSG000001	-	-	ENSG0000013302	'MYH10	-	17:8413130-8630761	-	-	IGV_LINK	576.984	6.25683	0	16.608	OK	613.579	6.36406	0	16.39	OK	OK	0.024516
ENSG000001	-	-	ENSG0000013302	'PEMT	-	17:17505562-17610485	-	-	IGV_LINK	162.913	12.6194	0.999535	24.2393	OK	161.902	11.4521	1.78721	21.1169	OK	OK	-0.14004
ENSG000001	-	-	ENSG0000013302	'SCO1	-	17:10672473-10815164	-	-	IGV_LINK	579.893	9.99256	0	20.551	OK	620.797	13.3504	0	29.8657	OK	OK	0.417961
ENSG000001	-	-	ENSG0000013303	'MPRIP	-	17:17042544-17237188	-	-	IGV_LINK	3319.35	41.3863	0	107.26	OK	2604.87	33.2987	0	76.1887	OK	OK	-0.31369
ENSG000001	-	-	ENSG0000013304	'CHI3L1	-	1:203178930-203186749	-	-	IGV_LINK	1802.71	77.1962	29.8943	124.498	OK	1257.06	50.6144	20.4223	80.8066	OK	OK	-0.60898
ENSG000001	-	-	ENSG0000013305	'MYBPH	-	1:203167810-203175813	-	-	IGV_LINK	2.90916	0.102355	0	0.287349	OK	1.03122	0.036298	0	0.157439	OK	NOTEST	-1.49561
ENSG000001	-	-	ENSG0000013305	'PIK3C2B	-	1:204377849-204494724	-	-	IGV_LINK	450.92	4.84507	0	16.2606	OK	744.544	8.50976	0	26.6076	OK	OK	0.812601
ENSG000001	-	-	ENSG0000013305	'DSTYK	-	1:205142504-205211566	-	-	IGV_LINK	461.587	3.37775	1.20928	5.54622	OK	443.427	3.2522	1.20612	5.29828	OK	OK	-0.05464
ENSG000001	-	-	ENSG0000013306	'CHIT1	-	1:203212826-203273641	-	-	IGV_LINK	32.9705	1.32898	0	4.10876	OK	97.9664	3.71338	0	8.40234	OK	OK	1.48242
ENSG000001	-	-	ENSG0000013306	'SLC41A1	-	1:205789092-205813748	-	-	IGV_LINK	1057.97	13.8682	5.24422	22.4921	OK	1070.41	14.38	5.36789	23.3921	OK	OK	0.052289
ENSG000001	-	-	ENSG0000013306	'LGR6	-	1:202193900-202319781	-	-	IGV_LINK	24.243	0.42633	0	1.04019	OK	12.3747	0.301462	0	0.856948	OK	NOTEST	-0.49999
ENSG000001	-	-	ENSG0000013306	'TMCC2	-	1:205228175-205273343	-	-	IGV_LINK	38.7888	0.700432	0	1.55569	OK	40.2178	0.772123	0	1.69504	OK	OK	0.140586
ENSG000001	-	-	ENSG0000013308	'DCLK1	-	13:35768651-36131306	-	-	IGV_LINK	775.776	8.16577	2.94094	13.3906	OK	2771.93	29.1754	11.5681	46.7826	OK	OK	1.83709
ENSG000001	-	-	ENSG0000013310	'CCNA1	-	13:36431519-36442882	-	-	IGV_LINK	6.78804	0.224196	0	0.555913	OK	10.3122	0.380992	0	0.897618	OK	NOTEST	0.765
ENSG000001	-	-	ENSG0000013310	'COG6	-	13:39655626-39791665	-	-	IGV_LINK	396.616	6.63391	0.690783	12.577	OK	488.801	9.35571	0	19.2612	OK	OK	0.495988
ENSG000001	-	-	ENSG0000013310	'SPART	-	13:36301637-36370180	-	-	IGV_LINK	1761.01	51.6213	11.0904	92.1521	OK	1521.06	40.0251	9.43353	70.6166	OK	OK	-0.36706
ENSG000001	-	-	ENSG0000013310	'RXFP2	-	13:31739541-31803388	-	-	IGV_LINK	0	0	0	0	OK	0	0	0	0	OK	NOTEST	0
ENSG000001	-	-	ENSG0000013310	'EPSTI1	-	13:42886387-42992271	-	-	IGV_LINK	1511.79	45.7625	10.6706	80.8545	OK	2077.92	67.0971	11.1168	123.077	OK	OK	0.552083
ENSG000001	-	-	ENSG0000013310	'TRPC4	-	13:37636635-37870425	-	-	IGV_LINK	41.698	0.957269	0	2.61319	OK	19.5933	0.442421	0	1.10042	OK	OK	-1.1135
ENSG000001	-	-	ENSG0000013311	'POSTN	-	13:37562582-37598844	-	-	IGV_LINK	95.0326	1.88734	0.071847	3.70283	OK	370.21	7.07601	2.37857	11.7734	OK	OK	1.90658
ENSG000001	-	-	ENSG0000013311	'RFXAP	-	13:36819223-36829104	-	-	IGV_LINK	81.4565	2.15368	0.153714	4.15365	OK	87.6541	2.31879	0.204071	4.43352	OK	OK	0.106569
ENSG000001	-	-	ENSG0000013311	'TPT1	-	13:45333470-45420371	-	-	IGV_LINK	38801.4	2989.45	0	6211.87	OK	41938.9	3246.9	0	6826.88	OK	OK	0.119184
ENSG000001	-	-	ENSG0000013311	'GPALPP1	-	13:44939248-45083125	-	-	IGV_LINK	442.193	12.6382	2.67133	22.605	OK	411.459	12.1942	2.12077	22.2676	OK	OK	-0.05159
ENSG000001	-	-	ENSG0000013311	'STOML3	-	13:38965924-38991066	-	-	IGV_LINK	1.93944	0.059903	0	0.197295	OK	1.03122	0.031868	0	0.13701	OK	NOTEST	-0.9105
ENSG000001	-	-	ENSG0000013311	'KL	-	13:33016432-33066145	-	-	IGV_LINK	766.079	9.91156	4.05467	15.7684	OK	369.179	4.7097	1.37195	8.04745	OK	OK	-1.07348
ENSG000001	-	-	ENSG0000013311	'RFC3	-	13:33818048-33966558	-	-	IGV_LINK	110.548	3.52487	0	7.55585	OK	121.685	3.86171	0.257194	7.46623	OK	OK	0.131672
ENSG000001	-	-	ENSG0000013312	'STARD13	-	13:33103134-33350630	-	-	IGV_LINK	531.407	6.95665	0	14.3651	OK	1233.35	14.7171	1.43636	27.9978	OK	OK	1.08103
ENSG000001	-	-	ENSG0000013312	'IRS4	-	X:108719948-108738903	-	-	IGV_LINK	0	0	0	0	OK	0	0	0	0	OK	NOTEST	0
ENSG000001	-	-	ENSG0000013313	'MORC4	-	X:106802679-107000244	-	-	IGV_LINK	340.372	6.94613	0	27.7985	OK	272.243	5.85004	0	21.3651	OK	OK	-0.24776
ENSG000001	-	-	ENSG0000013313	'BEX2	-	X:103309345-103311046	-	-	IGV_LINK	144.488	11.8472	1.7101	21.9842	OK	81.4668	6.22825	0.130604	12.3259	OK	OK	-0.92764
ENSG000001	-	-	ENSG0000013313	'RNF128	-	X:106693793-106796993	-	-	IGV_LINK	819.414	17.4622	7.26191	27.6625	OK	2292.41	48.874	21.4207	76.3272	OK	OK	1.48483

ENSG0000013	-	-	ENSG0000013313	'GNG5P2	-	X:110194185-110456334	-	-	IGV_LINK	0	0	0	0	OK	0	0	0	0	OK	NOTEST	0
ENSG0000013	-	-	ENSG0000013313	'TBC1D8B	-	X:106802679-107000244	-	-	IGV_LINK	587.651	7.79466	0	27.3153	OK	1114.75	14.0422	0	33.438	OK	OK	0.849209
ENSG0000013	-	-	ENSG0000013314	'TCEAL4	-	X:103576230-103587736	-	-	IGV_LINK	834.929	50.741	18.694	82.788	OK	771.356	46.4911	17.5912	75.391	OK	OK	-0.1262
ENSG0000013	-	-	ENSG0000013316	'BEX1	-	X:103062650-103064240	-	-	IGV_LINK	1.93944	0.158065	0	0.484068	OK	0	0	0	0	OK	NOTEST	'-inf
ENSG0000013	-	-	ENSG0000013319	'FAM104A	-	17:73164976-73262352	-	-	IGV_LINK	565.347	12.9469	0	29.2327	OK	631.11	14.5897	0	33.4814	OK	OK	0.17234
ENSG0000013	-	-	ENSG0000013319	'SLC39A11	-	17:72645948-73092712	-	-	IGV_LINK	344.251	11.2739	0.525864	22.0219	OK	804.355	26.3957	3.67708	49.1143	OK	OK	1.22732
ENSG0000013	-	-	ENSG0000013321	'EPHB2	-	1:22710838-22921500	-	-	IGV_LINK	177.459	2.18493	0.18548	4.18437	OK	300.086	4.16869	0.472436	7.86495	OK	OK	0.93201
ENSG0000013	-	-	ENSG0000013322	'SRRM1	-	1:24631715-24673267	-	-	IGV_LINK	2135.32	61.4128	11.1878	111.638	OK	1836.61	49.7264	9.1609	90.2919	OK	OK	-0.30453
ENSG0000013	-	-	ENSG0000013324	'BTBD2	-	19:1985437-2034881	-	-	IGV_LINK	1693.02	42.9341	15.4313	70.4369	OK	1784.02	44.2148	17.2176	71.2119	OK	OK	0.042405
ENSG0000013	-	-	ENSG0000013324	'PRAM1	-	19:8490055-8503112	-	-	IGV_LINK	383.04	13.5128	2.97024	24.0553	OK	256.775	9.22772	1.03165	17.4238	OK	OK	-0.55028
ENSG0000013	-	-	ENSG0000013324	'KMT5C	-	19:55339852-55348120	-	-	IGV_LINK	252.127	12.0488	1.8416	22.2559	OK	216.557	10.297	1.16637	19.4277	OK	OK	-0.22665
ENSG0000013	-	-	ENSG0000013325	'ZNF414	-	19:8509677-8514164	-	-	IGV_LINK	140.609	13.3188	0	26.7997	OK	107.247	11.3885	0	24.79	OK	OK	-0.22588
ENSG0000013	-	-	ENSG0000013325	'PDE6B	-	4:625583-670782	-	-	IGV_LINK	497.467	16.1981	0.241303	32.1548	OK	287.712	13.8047	0	28.2669	OK	OK	-0.23066
ENSG0000013	-	-	ENSG0000013326	'HSPBP1	-	19:55262230-55280381	-	-	IGV_LINK	423.768	18.1134	5.32561	30.9012	OK	503.238	21.3634	7.49787	35.229	OK	OK	0.238086
ENSG0000013	-	-	ENSG0000013327	'CSNK1G2	-	19:1941148-1981338	-	-	IGV_LINK	1218.94	34.6616	9.60375	59.7195	OK	1554.06	48.9071	13.5285	84.2856	OK	OK	0.496704
ENSG0000013	-	-	ENSG0000013330	'SLF1	-	5:94152965-95284575	-	-	IGV_LINK	166.792	3.31767	0	8.50599	OK	105.185	1.70147	0	4.5054	OK	OK	-0.96339
ENSG0000013	-	-	ENSG0000013331	'CNDP2	-	18:74495815-74523454	-	-	IGV_LINK	15108.2	463.109	0	934.984	OK	27019.1	805.465	0	1733.46	OK	OK	0.79847
ENSG0000013	-	-	ENSG0000013331	'MACROD1	-	11:63974606-64166106	-	-	IGV_LINK	368.494	29.4867	0	105.349	OK	129.934	8.96904	0	40.6581	OK	OK	-1.71704
ENSG0000013	-	-	ENSG0000013331	'WDR74	-	11:62832233-62841809	-	-	IGV_LINK	579.506	37.9784	12.5908	63.3661	OK	545.06	35.3841	10.9189	59.8494	OK	OK	-0.10208
ENSG0000013	-	-	ENSG0000013331	'LGALS12	-	11:63506083-63516774	-	-	IGV_LINK	361.706	14.3728	4.03279	24.7128	OK	168.09	6.58629	1.52089	11.6517	OK	OK	-1.1258
ENSG0000013	-	-	ENSG0000013331	'RTN3	-	11:63681445-63759891	-	-	IGV_LINK	2304.06	56.2629	23.1485	89.3774	OK	3133.89	76.4603	31.7021	121.219	OK	OK	0.442527
ENSG0000013	-	-	ENSG0000013332	'RARRES3	-	11:63536808-63546462	-	-	IGV_LINK	2789.89	273.743	119.228	428.258	OK	1999.55	196.653	83.491	309.815	OK	OK	-0.47717
ENSG0000013	-	-	ENSG0000013332	'HRASLS2	-	11:63552769-63563383	-	-	IGV_LINK	0	0	0	0	OK	4.1249	0.409029	0	1.03618	OK	NOTEST	inf
ENSG0000013	-	-	ENSG0000013339	'MYH11	-	16:15643266-15857033	-	-	IGV_LINK	2068.41	20.6402	0.663708	40.6167	OK	467.145	5.01841	0	11.7108	OK	OK	-2.04015
ENSG0000013	-	-	ENSG0000013339	'FOPNL	-	16:15865719-15888625	-	-	IGV_LINK	560.498	20.1302	4.23263	36.0278	OK	557.893	20.6976	4.49861	36.8965	OK	OK	0.040098
ENSG0000013	-	-	ENSG0000013339	'MED10	-	5:6371880-6378594	-	-	IGV_LINK	343.281	21.6069	7.14987	36.0639	OK	423.833	26.1296	9.30605	42.9531	OK	OK	0.274194
ENSG0000013	-	-	ENSG0000013340	'PDZD2	-	5:31639409-32121941	-	-	IGV_LINK	416.01	3.0393	0	6.67728	OK	733.201	5.18383	0	11.2005	OK	OK	0.770281
ENSG0000013	-	-	ENSG0000013342	'MORC2	-	22:30922307-30968298	-	-	IGV_LINK	740.866	14.4776	1.13531	27.8198	OK	903.353	15.9878	1.70033	30.2753	OK	OK	0.143154
ENSG0000013	-	-	ENSG0000013342	'LARGE1	-	22:33162225-33922841	-	-	IGV_LINK	113.457	1.56619	0	3.26542	OK	105.185	1.4638	0	3.06161	OK	OK	-0.09754
ENSG0000013	-	-	ENSG0000013343	'GSTT2B	-	22:23957413-23961186	-	-	IGV_LINK	22.3036	1.36997	0	2.87229	OK	5.15612	0.359605	0	0.894025	OK	OK	-1.92966
ENSG0000013	-	-	ENSG0000013345	'MYO18B	-	22:25742143-26031041	-	-	IGV_LINK	0	0	0	0	OK	4.1249	0.072868	0	0.459399	OK	NOTEST	inf
ENSG0000013	-	-	ENSG0000013346	'SLC2A11	-	22:23856426-23898930	-	-	IGV_LINK	621.591	17.5599	0	62.8196	OK	547.58	15.7026	0	57.3488	OK	OK	-0.16128
ENSG0000013	-	-	ENSG0000013346	'C1QTNF6	-	22:37125837-37199385	-	-	IGV_LINK	476.133	9.05252	0	20.6441	OK	658.953	13.176	0.463776	25.8882	OK	OK	0.54152
ENSG0000013	-	-	ENSG0000013347	'GGT2	-	22:21207972-21227637	-	-	IGV_LINK	4.8486	0.124572	0	0.31619	OK	105.185	2.7928	0.36362	5.22198	OK	OK	4.48666
ENSG0000013	-	-	ENSG0000013347	'FAM83F	-	22:39994948-40044530	-	-	IGV_LINK	70.7896	0.313261	0	0.79815	OK	199.026	1.90308	0	4.36819	OK	OK	2.6029
ENSG0000013	-	-	ENSG0000013348	'SEC14L4	-	22:30475363-30505711	-	-	IGV_LINK	20.3641	0.48743	0	1.07381	OK	204.183	4.9208	1.20559	8.63601	OK	OK	3.33563
ENSG0000013	-	-	ENSG0000013351	'ZDHHC8P1	-	22:23390605-23402726	-	-	IGV_LINK	14.5458	0.624745	0	1.72426	OK	24.7494	0.755139	0	1.73611	OK	OK	0.273475
ENSG0000013	-	-	ENSG0000013356	'GIMAP6	-	7:150625374-150632648	-	-	IGV_LINK	1236.39	27.5455	7.75942	47.3316	OK	1074.54	22.3791	6.93392	37.8243	OK	OK	-0.29967
ENSG0000013	-	-	ENSG0000013357	'GIMAP4	-	7:150567276-150573955	-	-	IGV_LINK	1358.58	61.3194	22.9953	99.6436	OK	1118.88	51.1932	19.209	83.1775	OK	OK	-0.26039
ENSG0000013	-	-	ENSG0000013359	'ADCK2	-	7:140518419-140924764	-	-	IGV_LINK	770.928	21.8342	0	47.4662	OK	586.767	15.5319	0	37.4785	OK	OK	-0.49135
ENSG0000013	-	-	ENSG0000013360	'MKRN1	-	7:140453039-140479569	-	-	IGV_LINK	2285.63	60.0622	8.06926	112.055	OK	2073.79	50.077	9.38956	90.7644	OK	OK	-0.26231
ENSG0000013	-	-	ENSG0000013361	'AGAP3	-	7:151085830-151144436	-	-	IGV_LINK	1075.42	35.6894	5.30547	66.0733	OK	1143.63	37.4486	5.5672	69.33	OK	OK	0.069417
ENSG0000013	-	-	ENSG0000013361	'KRBA1	-	7:149714780-149734575	-	-	IGV_LINK	1078.33	27.2903	2.8361	51.7445	OK	657.922	15.1967	1.2204	29.173	OK	OK	-0.84463
ENSG0000013	-	-	ENSG0000013362	'ZNF767P	-	7:149547153-149624752	-	-	IGV_LINK	486.8	13.2139	0	27.0977	OK	500.144	11.8824	0.178095	23.5867	OK	OK	-0.15323
ENSG0000013	-	-	ENSG0000013362	'ACTR3B	-	7:152759748-152855378	-	-	IGV_LINK	81.4565	3.56337	0	9.61344	OK	82.498	2.5773	0.006396	5.14821	OK	OK	-0.46738
ENSG0000013	-	-	ENSG0000013363	'NTS	-	12:85874294-85882992	-	-	IGV_LINK	16.4852	0.872763	0	1.88485	OK	10.3122	0.546542	0	1.28695	OK	OK	-0.67526
ENSG0000013	-	-	ENSG0000013363	'BTG1	-	12:91984975-92189660	-	-	IGV_LINK	3035.22	87.1145	0	187.685	OK	3775.31	116.589	0	249.994	OK	OK	0.420446
ENSG0000013	-	-	ENSG0000013364	'LRRIQ1	-	12:85036313-85263224	-	-	IGV_LINK	140.609	4.1848	0	9.65773	OK	84.5604	2.70741	0	7.20463	OK	OK	-0.62825
ENSG0000013	-	-	ENSG0000013364	'C12orf29	-	12:87980034-88142216	-	-	IGV_LINK	161.943	8.02183	0	21.4555	OK	166.027	8.01931	0	20.449	OK	OK	-0.00045
ENSG0000013	-	-	ENSG0000013365	'ATP13A3	-	3:194402671-194518279	-	-	IGV_LINK	2270.76	38.9064	0	88.0754	OK	2482.16	34.1966	0	75.5681	OK	OK	-0.18615
ENSG0000013	-	-	ENSG0000013366	'SFTPD	-	10:79904897-79982614	-	-	IGV_LINK	14.5458	0.9049	0	2.25995	OK	12.3747	0.635136	0	1.8732	OK	OK	-0.51069
ENSG0000013	-	-	ENSG0000013366	'DYDC2	-	10:80336104-80368073	-	-	IGV_LINK	5.81832	0.211439	0	0.754073	OK	17.5308	1.59359	0	3.86122	OK	OK	2.91396
ENSG0000013	-	-	ENSG0000013367	'TMEM254	-	10:80046859-80092557	-	-	IGV_LINK	330.675	16.218	0	33.208	OK	256.775	13.0534	0	27.5159	OK	OK	-0.31317
ENSG0000013	-	-	ENSG0000013368	'TMTC1	-	12:29500839-29784759	-	-	IGV_LINK	422.798	3.51296	0.970636	6.05528	OK	332.054	2.66908	0.528365	4.8098	OK	OK	-0.39634
ENSG0000013	-	-	ENSG0000013370	'KRAS	-	12:25195215-25250936	-	-	IGV_LINK	410.192	4.25065	0.979177	7.52213	OK	421.771	4.37913	1.1119	7.64636	OK	OK	0.04296
ENSG0000013	-	-	ENSG0000013370	'IPO8	-	12:30628987-30695986	-	-	IGV_LINK	830.081	13.002	1.13905	24.865	OK	756.919	13.7051	0	31.2796	OK	OK	0.07598
ENSG0000013	-	-	ENSG0000013370	'LARS	-	5:146084385-146182660	-	-	IGV_LINK	2480.55	54.5453	9.54655	99.5441	OK	2139.79	46.2804	9.89498	82.6659	OK	OK	-0.23705
ENSG0000013	-	-	ENSG0000013371	'SPINK5	-	5:148025682-148137289	-	-	IGV_LINK	14.5458	0.261552	0	0.790076	OK	57.7486	1.01132	0	2.37027	OK	OK	1.95106
ENSG0000013	-	-	ENSG0000013373	'IMPA1	-	8:81657960-81686693	-	-	IGV_LINK	352.009	19.7311	4.68463	34.7776	OK	411.459	24.3551	6.0663	42.6438	OK	OK	0.303748
ENSG0000013	-	-	ENSG0000013373	'LRRCC1	-	8:85107146-85146076	-	-	IGV_LINK	205.581	4.20511	0.566372	7.84386	OK	144.371	2.857	0.474862	5.23913	OK	OK	-0.55765
ENSG0000013	-	-	ENSG0000013374	'E2F5	-	8:85177224-85220421	-	-	IGV_LINK	248.248	9.01428	0	23.0129	OK	203.151	6.54688	0	16.7076	OK	OK	-0.4614
ENSG0000013	-	-	ENSG0000013374	'CA1	-	8:85327607-85481493	-	-	IGV_LINK	0.969721	0.129662	0	3.57776	OK	2.06245	0.176557	0	4.61845	OK	NOTEST	0.445387
ENSG0000013	-	-	ENSG0000013377	'CCDC59	-	12:82223680-82479236	-	-	IGV_LINK	351.039	26.3249	4.84917	47.8007	OK	313.492	24.3391	4.99424	43.6839	OK	OK	-0.11316
ENSG0000013	-	-	ENSG0000013378	'SWAP70	-	11:9664076-9752991	-	-	IGV_LINK	1250.94	16.9047	6.96259	26.8467	OK	1391.12	19.8028	7.5984	32.0071	OK	OK	0.228281
ENSG0000013	-	-	ENSG0000013379	'ARNTL	-	11:13276651-13387266	-	-	IGV_LINK	303.523	9.71748	0	19.4832	OK	332.054	9.13934	0.703918	17.5748	OK	OK	-0.08849
ENSG0000013	-	-	ENSG0000013380	'LYVE1	-	11:10541271-10693988	-	-	IGV_LINK	196.853	4.96413	0	10.9547	OK	149.528	3.68672	0	8.03202	OK	OK	-0.4292
ENSG0000013	-	-	ENSG0000013380	'AMPD3	-	11:10308312-10507579	-	-	IGV_LINK	734.078	16.8204	0	40.7023	OK	721.857	17.0385	0	41.8262	OK	OK	0.018587
ENSG0000013	-	-	ENSG0000013380	'MICALCL	-	11:12276079-12359144	-	-	IGV_LINK	10.6669	0.209348	0	0.69296	OK	12.3747	0.242962	0	0.751044	OK	NOTEST	0.214827
ENSG0000013	-	-	ENSG0000013381	'SBF2	-	11:9754769-10294207	-	-	IGV_LINK	1072.51	17.1215	0	36.1356	OK	853.854	16.3839	0	33.2532	OK	OK	-0.06353
ENSG0000013	-	-	ENSG0000013381	'MICAL2	-	11:12094007-12263789	-	-	IGV_LINK	321.947	3.85503	0	8.73385	OK	425.896	6.56286	0	14.7847	OK	OK	0.767582
ENSG0000013	-	-	ENSG0000013382	'RRAS2	-	11:14277925-14364506	-	-	IGV_LINK	288.977	13.7817	3.78257	23.7808	OK	487.769	25.5099	7.92336	43.0964	OK	OK	0.888306
ENSG0000013	-	-	ENSG0000013383	'HSD17B4	-	5:119452442-119637199	-	-	IGV_LINK	1464.28	55.1705	5.49493	104.846	OK	2398.63	113.412	0	227.498	OK	OK	1.0396
ENSG0000013	-	-	ENSG0000013385	'ZFC3H1	-	12:71609471-71705046	-	-	IGV_LINK	1287.79	21.8989	0	55.1298	OK	1134.35	19.9979	0	49.2819	OK	OK	-0.13101
ENSG0000013	-	-	ENSG0000013386	'TEX15	-	8:30831543-30890606	-	-	IGV_LINK	36.8494	0.203424	0	0.490233	OK	21.6557	0.122823	0	0.361626	OK	NOTEST	-0.72791
ENSG0000013	-	-	ENSG0000013387	'SARAF	-	8:30063011-30083467	-	-	IGV_LINK	8391.96	342.035	82.275	601.795	OK	7546.5	304.839	78.9357	530.743	OK	OK	-0.1661
ENSG0000013	-	-	ENSG0000013387	'RNF122	-	8:33547754-33567125	-	-	IGV_LINK	142.549	4.73814	0.94718	8.52911	OK	203.151	6.75707	1.7272	11.7869	OK	OK	0.512077
ENSG0000013	-	-	ENSG0000013387	'DUSP26	-	8:33591331-33600106	-	-	IGV_LINK	12.6064	0.615338	0	1.48915	OK	6.18735	0.209888	0	0.577394	OK	OK	-1.55176
ENSG0000013	-	-	ENSG0000013388	'DPF2	-	11:65333753-65353249	-	-	IGV_LINK	1048.27	34.8253	8.63926	61.0113	OK	976.57	31.0487	7.14643	54.9511	OK	OK	-0.1656
ENSG0000013	-	-	ENSG0000013389	'MEN1	-	11:64803509-64811294	-	-	IGV_LINK	703.047	18.019	4.53706	31.5009	OK	782.7	21.1672	4.96275	37.3716	OK	OK	0.232314
ENSG0000013	-	-	ENSG0000013393	'ERG28	-	14:75574887-76084585	-	-	IGV_LINK	239.521	5.48605	0	12.7638	OK	312.461	7.16004	0	16.9029	OK	OK	0.3842
ENSG0000013	-	-	ENSG0000013393	'GSC	-	14:94768215-94770230	-	-	IGV_LINK	7.75776	0.400552	0	0.927101	OK	1.03122	0.053301	0	0.199493	OK	NOTEST	-2.90976
ENSG0000013	-	-	ENSG0000013394	'C14orf159	-	14:90847861-91225632	-	-	IGV_LINK	1026.93	37.7914	7.15928	68.4235	OK	1102.38	44.3465	9.91457	78.7783	OK	OK	0.230761
ENSG0000013	-	-	ENSG0000013395	'UNC79	-	14:93333218-93707876	-	-	IGV_LINK	17.455	0.138681	0	0.333192	OK	20.6245	0.147525	0	0.338926	OK	NOTEST	0.089191
ENSG0000013	-	-	ENSG0000013396	'NUMB	-	14:73275106-73477175	-	-	IGV_LINK	1920.05	42.1736	12.4071	71.94	OK	1697.4	37.7608	9.87074	65.6509	OK	OK	-0.15945
ENSG0000013	-	-	ENSG0000013396	'CATSPERB	-	14:91580695-91867536	-	-	IGV_LINK	26.1825	0.57362	0	2.12866	OK	30.9367	0.699922	0	2.45677	OK	OK	0.287098
ENSG0000013	-	-	ENSG0000013398	'VRTN	-	14:74303068-74360008	-	-	IGV_LINK	0	0	0	0	OK	0	0	0	0	OK	NOTEST	0
ENSG0000013	-	-	ENSG0000013398	'COX16	-	14:70325080-70417074	-	-	IGV_LINK	239.483	13.1774	0	31.0003	OK	250.393	12.4198	0	28.0023	OK	OK	-0.08542
ENSG0000013	-	-	ENSG0000013398	'TTC9	-	14:70641786-70675360	-	-	IGV_LINK	61.0924	0.682506	0.007859	1.35715	OK	41.249	0.460926	0	0.931308	OK	OK	-0.56631
ENSG0000013	-	-	ENSG0000013399	'MED6	-	14:70581256-70641298	-	-	IGV_LINK	196.853	10.0493	1.8685	18.2301	OK	193.87	10.0567	1.96609	18.1473	OK	OK	0.001059
ENSG0000013	-	-	ENSG0000013400	'EIF2S1	-	14:67241108-67386516	-	-	IGV_LINK	642.925	41.3984	0	94.6748	OK	665.14	41.4473	0	95.3377	OK	OK	0.001702
ENSG0000013	-	-	ENSG0000013400	'ADAM20	-	14:70425811-70547464	-	-	IGV_LINK	2.90916	0.062516	0	0.30941	OK	6.18735	0.133021	0	0.430917	OK	NOTEST	1.08935
ENSG0000013	-	-	ENSG0000013401	'LOXL2	-	8:23297188-23457695	-	-	IGV_LINK	1055.06	19.6842	0	40.6655	OK	2091.32	37.1838	5.11862	69.2489	OK	OK	0.917634
ENSG0000013	-	-	ENSG0000013401	'ELP3	-	8:28089672-28191156	-	-	IGV_LINK	651.652	17.4722	4.43338	30.5109	OK	868.291	31.1192	8.48875	53.7496	OK	OK	0.832745
ENSG0000013	-	-	ENSG0000013402	'PEBP4	-	8:22687658-23083619	-	-	IGV_LINK	1.93944	0.151283	0	3.73847	OK	1.03122	0.080567	0	3.31998	OK	NOTEST	-0.90898
ENSG0000013	-	-	ENSG0000013402	'ADAMDEC1	-	8:24294039-24912073	-	-	IGV_LINK	160.004	4.65698	0	11.9273	OK	755.888	21.6761	2.74743	40.6048	OK	OK	2.21864
ENSG0000013	-	-	ENSG0000013403	'CTIF	-	18:48539045-48863217	-	-	IGV_LINK	386.918	5.90106	0	13.4131	OK	455.801	5.77322	0.953786	10.5926	OK	OK	-0.0316
ENSG0000013	-	-	ENSG0000013404	'MRO	-	18:50795119-50825402	-	-	IGV_LINK	52.3649	0.977412	0	2.93453	OK	182.527	3.09785	0	6.61958	OK	OK	1.66423
ENSG0000013	-	-	ENSG0000013404	'MBD2	-	18:54151600-54224788	-	-	IGV_LINK	1442.94	66.027	0	164.599	OK	1350.9	65.1366	0	165.723	OK	OK	-0.01959
ENSG0000013	-	-	ENSG0000013404	'IER3IP1	-	18:47105945-47176374	-	-	IGV_LINK	343.251	9.88846	0	23.4324	OK	365.472	10.4714	0	24.6901	OK	OK	0.082639
ENSG0000013	-	-	ENSG0000013405	'MRPS36	-	5:69217759-69230129	-	-	IGV_LINK	285.098	17.7447	0.260309	35.229	OK	283.587	19.8017	0	40.8762	OK	OK	0.158235
ENSG0000013	-	-	ENSG0000013405	'CCNB1	-	5:69167009-69178245	-	-	IGV_LINK	188.126	8.1064	1.42609	14.7867	OK	293.899	14.3056	2.92555	25.6856	OK	OK	0.819445
ENSG0000013	-	-	ENSG0000013405	'CDK7	-	5:69234794-69277430	-	-	IGV_LINK	358.797	19.8422	4.77576	34.9086	OK	373.303	18.7447	5.30938	32.1801	OK	OK	-0.08209
ENSG0000013	-	-	ENSG0000013406	'CD180	-	5:67179612-67196799	-	-	IGV_LINK	377.221	4.07603	1.42792	6.72414	OK	386.709	4.34636	0.88945	7.80327	OK	OK	0.092643
ENSG0000013	-	-	ENSG0000013407	'IRAK2	-	3:10164864-10243743	-	-	IGV_LINK	228.854	3.89586	1.12592	6.66581	OK	213.464	3.63513	0.892274	6.37798	OK	OK	-0.09994
ENSG0000013	-	-	ENSG0000013407	'CAMK1	-	3:9749943-9855138	-	-	IGV_LINK	523.649	26.6094	0	92.2186	OK	629.047	31.1821	0	98.0318	OK	OK	0.228781
ENSG0000013	-	-	ENSG0000013407	'THUMPD3	-	3:8980590-9479240	-	-	IGV_LINK	346.19	13.5065	0	44.3472	OK	446.52	17.165	0	50.4849	OK	OK	0.34582
ENSG0000013	-	-	ENSG0000013408	'VHL	-	3:10141007-10152220	-	-	IGV_LINK	436.374	11.9057	3.02989	20.7815	OK	384.647	11.0427	1.63171	20.4538	OK	OK	-0.10855
ENSG0000013	-	-	ENSG0000013410	'BHLHE40	-	3:4896808-4985323	-	-	IGV_LINK	2359.33	47.059	10.477	83.641	OK	3862.97	79.2986	18.3481	140.249	OK	OK	0.752825
ENSG0000013	-	-	ENSG0000013410	'ARL8B	-	3:5122219-5219957	-	-	IGV_LINK	565.347	14.0696	0	44.4653	OK	615.641	13.8019	0.363873	27.24	OK	OK	-0.02771
ENSG0000013	-	-	ENSG0000013410	'EDEM1	-	3:5122219-5219957	-	-	IGV_LINK	1153	11.1451	2.41755	19.8726	OK	1126.1	10.821	2.93895	18.7031	OK	OK	-0.04257
ENSG0000013	-	-	ENSG0000013411	'CNTN6	-	3:1092575-1404217	-	-	IGV_LINK	0.969721	0.016329	0	0.286779	OK	0	0	0	0	OK	NOTEST	'-inf
ENSG0000013	-	-	ENSG0000013412	'CHL1	-	3:195757-409417	-	-	IGV_LINK	23.2733	0.462264	0	1.6479	OK	3.09367	0.1196	0	0.865247	OK	NOTEST	-1.9505
ENSG0000013	-	-	ENSG0000013413	'MEIS2	-	15:36889203-37101299	-	-	IGV_LINK	186.186	5.7778	0	11.7158	OK	202.12	8.78236	0	17.9263	OK	OK	0.604088
ENSG0000013	-	-	ENSG0000013414	'DPH6	-	15:35181798-35546193	-	-	IGV_LINK	106.669	10.8631	0	21.7365	OK	97.9664	10.545	0	21.2384	OK	OK	-0.04288
ENSG0000013	-	-	ENSG0000013415	'KATNBL1	-	15:34140673-34210096	-	-	IGV_LINK	230.793	12.571	1.69774	23.4443	OK	233.057	14.3059	2.31437	26.2975	OK	OK	0.186512
ENSG0000013	-	-	ENSG0000013415	'EMC7	-	15:34084016-34101948	-	-	IGV_LINK	965.842	57.5256	23.1338	91.9175	OK	1190.03	73.2394	29.3256	117.153	OK	OK	0.348415
ENSG0000013	-	-	ENSG0000013416	'TRPM1	-	15:31001060-31161273	-	-	IGV_LINK	7.75776	0.719528	0	1.99549	OK	3.09367	0.148943	0	0.604273	OK	OK	-2.27229
ENSG0000013	-	-	ENSG0000013418	'GNAT2	-	1:109548610-109632051	-	-	IGV_LINK	0.969721	0.04685	0	1.43432	OK	2.06245	0.099741	0	2.43562	OK	NOTEST	1.09014
ENSG0000013	-	-	ENSG0000013418	'GSTM1	-	1:109656080-109709551	-	-	IGV_LINK	890.677	76.2118	0	167.267	OK	788.616	64.284	0	135.751	OK	OK	-0.24555
ENSG0000013	-	-	ENSG0000013418	'PRPF38B	-	1:108692322-108701803	-	-	IGV_LINK	1544.76	30.3461	11.5566	49.1356	OK	1523.12	30.6947	11.5608	49.8285	OK	OK	0.016477
ENSG0000013	-	-	ENSG0000013419	'REG4	-	1:119794017-119811660	-	-	IGV_LINK	1.93944	0.092272	0	0.378073	OK	0	0	0	0	OK	NOTEST	'-inf
ENSG0000013	-	-	ENSG0000013419	'TSPAN2	-	1:115048010-115089500	-	-	IGV_LINK	64.9713	2.59209	0	5.94784	OK	43.3114	2.13318	0	5.21975	OK	OK	-0.28111
ENSG0000013	-	-	ENSG0000013420	'TSHB	-	1:115029823-115034309	-	-	IGV_LINK	0.969721	0.181215	0	0.797375	OK	0	0	0	0	OK	NOTEST	'-inf
ENSG0000013	-	-	ENSG0000013420	'GSTM5	-	1:109712254-109775428	-	-	IGV_LINK	42.6677	1.17944	0	3.72775	OK	21.6557	0.756944	0	3.70653	OK	OK	-0.63984
ENSG0000013	-	-	ENSG0000013420	'GSTM3	-	1:109712254-109775428	-	-	IGV_LINK	231.763	14.4103	0.403459	28.4172	OK	266.056	20.9375	1.0991	40.7759	OK	OK	0.538984
ENSG0000013	-	-	ENSG0000013420	'SYT6	-	1:114089290-114153919	-	-	IGV_LINK	75.6382	1.43852	0	3.59956	OK	19.5933	0.370004	0	0.953924	OK	OK	-1.95898
ENSG0000013	-	-	ENSG0000013421	'VAV3	-	1:107571159-107994607	-	-	IGV_LINK	980.387	19.2101	2.2825	36.1377	OK	1073.51	20.5711	1.6493	39.4928	OK	OK	0.098751
ENSG0000013	-	-	ENSG0000013421	'CHIA	-	1:111290861-111324075	-	-	IGV_LINK	0	0	0	0	OK	1.03122	0.050679	0	0.345351	OK	NOTEST	inf
ENSG0000013	-	-	ENSG0000013422	'PSRC1	-	1:109279555-109283186	-	-	IGV_LINK	92.1234	3.51088	0.159143	6.86261	OK	91.779	3.82984	0	7.97061	OK	OK	0.125452
ENSG0000013	-	-	ENSG0000013424	'HMGCS2	-	1:119747995-119768905	-	-	IGV_LINK	10.6669	0.387417	0	0.96487	OK	0	0	0	0	OK	NOTEST	'-inf
ENSG0000013	-	-	ENSG0000013424	'PTPN22	-	1:113761831-113914086	-	-	IGV_LINK	295.765	7.54929	0	18.9854	OK	229.963	5.38208	0	13.5888	OK	OK	-0.48818
ENSG0000013	-	-	ENSG0000013424	'SORT1	-	1:109309567-109397951	-	-	IGV_LINK	1046.33	8.61816	3.63903	13.5973	OK	777.544	6.91936	0	14.1501	OK	OK	-0.31674

ENSG000001	-	-	ENSG0000013424	'WNT2B	-	1:112466540-112671619	-	-	IGV_LINK	138.67	1.3535	0	5.07654	OK	364.022	3.59798	0	10.5773	OK	OK	1.41049
ENSG000001	-	-	ENSG0000013424	'PTGFRN	-	1:116910056-116990358	-	-	IGV_LINK	665.228	6.59347	1.41513	11.7718	OK	934.29	8.76906	3.59458	13.9435	OK	OK	0.411384
ENSG000001	-	-	ENSG0000013424	'LAMTOR5	-	1:110347115-110443817	-	-	IGV_LINK	1076.39	125.916	16.3555	235.477	OK	1435.47	167.632	0	353.49	OK	OK	0.412834
ENSG000001	-	-	ENSG0000013424	'ADAM30	-	1:119893532-119896495	-	-	IGV_LINK	0	0	0	0	OK	0	0	0	0	OK	NOTEST	0
ENSG000001	-	-	ENSG0000013425	'NOTCH2	-	1:119911552-120069626	-	-	IGV_LINK	2952.8	16.1019	0	32.4894	OK	2504.85	12.6803	5.44838	19.9123	OK	OK	-0.34464
ENSG000001	-	-	ENSG0000013425	'TRIM45	-	1:117111059-117122587	-	-	IGV_LINK	108.609	2.31804	0	4.65284	OK	93.8415	1.73802	0.038375	3.43766	OK	OK	-0.41546
ENSG000001	-	-	ENSG0000013425	'CEPT1	-	1:111117332-111185102	-	-	IGV_LINK	667.168	25.3887	0	58.5822	OK	714.639	32.8427	0	74.444	OK	OK	0.371386
ENSG000001	-	-	ENSG0000013425	'CD101	-	1:117001749-117059490	-	-	IGV_LINK	104.73	4.44673	0	11.6513	OK	84.5604	2.78358	0	7.01918	OK	OK	-0.6758
ENSG000001	-	-	ENSG0000013425	'VTCN1	-	1:117143586-117210960	-	-	IGV_LINK	0.969721	0.025819	0	0.362013	OK	3.09367	0.082418	0	0.433282	OK	NOTEST	1.67453
ENSG000001	-	-	ENSG0000013425	'NGF	-	1:115283033-115368072	-	-	IGV_LINK	108.609	6.99929	0.564887	13.4337	OK	30.9367	1.99634	0	4.10914	OK	OK	-1.80985
ENSG000001	-	-	ENSG0000013426	'AP4B1	-	1:113761831-113914086	-	-	IGV_LINK	381.1	14.0775	0	36.8506	OK	441.364	14.4729	0	36.6999	OK	OK	0.039971
ENSG000001	-	-	ENSG0000013426	'NAPG	-	18:10525904-10552761	-	-	IGV_LINK	308.371	7.70632	1.3995	14.0131	OK	459.926	11.635	1.39711	21.8728	OK	OK	0.594352
ENSG000001	-	-	ENSG0000013427	'SPIRE1	-	18:12438889-12725740	-	-	IGV_LINK	2691.94	40.9915	1.45629	80.5268	OK	2235.7	32.7705	2.40075	63.1402	OK	OK	-0.32293
ENSG000001	-	-	ENSG0000013428	'PPHLN1	-	12:42238446-42590355	-	-	IGV_LINK	960.023	27.406	2.83507	51.977	OK	964.619	28.9756	3.42488	54.5262	OK	OK	0.080342
ENSG000001	-	-	ENSG0000013428	'FKBP11	-	12:48903417-48957551	-	-	IGV_LINK	474.193	25.2375	0	82.3295	OK	732.17	50.1885	0	144.054	OK	OK	0.991787
ENSG000001	-	-	ENSG0000013428	'ARF3	-	12:48903417-48957551	-	-	IGV_LINK	3621.46	75.2257	4.78861	145.663	OK	3973.89	92.6792	0	188.228	OK	OK	0.301019
ENSG000001	-	-	ENSG0000013429	'TMEM106C	-	12:47963568-47968878	-	-	IGV_LINK	1101.6	70.526	23.481	117.571	OK	1227.16	74.6674	25.8059	123.529	OK	OK	0.082324
ENSG000001	-	-	ENSG0000013429	'SLC38A2	-	12:46358188-46373778	-	-	IGV_LINK	1595.19	24.6814	2.33089	47.0319	OK	1590.15	21.9816	5.65346	38.3098	OK	OK	-0.16713
ENSG000001	-	-	ENSG0000013429	'PLEKHA8P1	-	12:45173063-45440404	-	-	IGV_LINK	57.2135	2.62795	0	16.5594	OK	85.5917	7.65641	0	63.431	OK	OK	1.54273
ENSG000001	-	-	ENSG0000013430	'YWHAQ	-	2:9583971-9631014	-	-	IGV_LINK	2540.67	83.1944	29.1544	137.234	OK	3339.11	124.428	37.1053	211.75	OK	OK	0.580751
ENSG000001	-	-	ENSG0000013431	'KIDINS220	-	2:8721080-8837630	-	-	IGV_LINK	1842.47	16.6087	1.44484	31.7726	OK	1439.59	12.5069	1.84733	23.1665	OK	OK	-0.40922
ENSG000001	-	-	ENSG0000013431	'GRHL1	-	2:9951692-10002277	-	-	IGV_LINK	15.5155	0.457997	0	1.17641	OK	90.7478	2.907	0	6.7939	OK	OK	2.66612
ENSG000001	-	-	ENSG0000013431	'ROCK2	-	2:11179760-11348330	-	-	IGV_LINK	734.078	5.0952	2.0632	8.12721	OK	732.17	5.08256	2.05143	8.1137	OK	OK	-0.00358
ENSG000001	-	-	ENSG0000013432	'RSAD2	-	2:6840569-6898239	-	-	IGV_LINK	432.495	9.44719	0	19.9867	OK	603.267	11.5958	0.263774	22.9278	OK	OK	0.295642
ENSG000001	-	-	ENSG0000013432	'MYCN	-	2:15920398-15947007	-	-	IGV_LINK	5.81832	0.134967	0	0.338191	OK	2.06245	0.047867	0	0.166643	OK	NOTEST	-1.49551
ENSG000001	-	-	ENSG0000013432	'LPIN1	-	2:11677594-11827409	-	-	IGV_LINK	531.407	8.16047	0	17.4635	OK	432.083	7.67723	0	17.013	OK	OK	-0.08807
ENSG000001	-	-	ENSG0000013432	'CMPK2	-	2:6840569-6898239	-	-	IGV_LINK	349.099	10.433	0	22.6948	OK	379.491	10.9989	0	24.4965	OK	OK	0.076205
ENSG000001	-	-	ENSG0000013433	'IAH1	-	2:9473657-9555792	-	-	IGV_LINK	763.17	55.7392	10.0976	101.381	OK	952.852	66.3741	12.2313	120.517	OK	OK	0.251928
ENSG000001	-	-	ENSG0000013433	'LDHA	-	11:18394387-18408425	-	-	IGV_LINK	18648.7	837.186	125.167	1549.2	OK	36699.2	1753.79	0	3612.96	OK	OK	1.06686
ENSG000001	-	-	ENSG0000013433	'SAA2	-	11:18231348-18248643	-	-	IGV_LINK	0	0	0	0	OK	3528.29	381.892	87.8677	675.917	OK	OK	inf
ENSG000001	-	-	ENSG0000013434	'ANO3	-	11:26309598-26663288	-	-	IGV_LINK	37.8191	0.474416	0	1.06786	OK	4.1249	0.059327	0	0.296271	OK	NOTEST	-2.99939
ENSG000001	-	-	ENSG0000013435	'IL6ST	-	5:55935094-55994993	-	-	IGV_LINK	2965.41	34.8242	0	82.6456	OK	2562.59	23.9261	0	53.0658	OK	OK	-0.54151
ENSG000001	-	-	ENSG0000013436	'FST	-	5:53480408-53487134	-	-	IGV_LINK	0.969721	0.102632	0	0.488487	OK	2.06245	0.120661	0	0.541553	OK	NOTEST	0.233479
ENSG000001	-	-	ENSG0000013436	'CFHR4	-	1:196819744-196959226	-	-	IGV_LINK	0	0	0	0	OK	5.15612	0.203744	0	0.569346	OK	NOTEST	inf
ENSG000001	-	-	ENSG0000013436	'NAV1	-	1:201622884-201884294	-	-	IGV_LINK	806.807	5.3053	0	13.5912	OK	752.794	5.89397	0	22.17	OK	OK	0.151805
ENSG000001	-	-	ENSG0000013437	'CDC73	-	1:193122016-193253901	-	-	IGV_LINK	557.589	7.12879	1.75788	12.4997	OK	663.078	8.01229	2.7414	13.2832	OK	OK	0.168557
ENSG000001	-	-	ENSG0000013437	'TIMM17A	-	1:201955490-201970661	-	-	IGV_LINK	444.132	20.8713	4.01838	37.7242	OK	622.86	28.0813	8.48548	47.6771	OK	OK	0.428089
ENSG000001	-	-	ENSG0000013437	'CRB1	-	1:197268203-197478455	-	-	IGV_LINK	13.5761	0.221543	0	0.730571	OK	18.562	0.474614	0	1.23183	OK	NOTEST	1.09917
ENSG000001	-	-	ENSG0000013438	'CFHR5	-	1:196977555-197009674	-	-	IGV_LINK	0	0	0	0	OK	0	0	0	0	OK	NOTEST	0
ENSG000001	-	-	ENSG0000013439	'ERN2	-	16:23677655-23713500	-	-	IGV_LINK	0	0	0	0	OK	1.03122	0.027815	0	0.592605	OK	NOTEST	inf
ENSG000001	-	-	ENSG0000013441	'RPS15A	-	16:18781294-18801678	-	-	IGV_LINK	10672.9	1656.05	339.217	2972.88	OK	7814.03	1215.97	220.915	2211.03	OK	OK	-0.44563
ENSG000001	-	-	ENSG0000013443	'RAX	-	18:59267034-59274086	-	-	IGV_LINK	0	0	0	0	OK	0	0	0	0	OK	NOTEST	0
ENSG000001	-	-	ENSG0000013444	'NARS	-	18:57600655-57622213	-	-	IGV_LINK	2065.5	70.5539	20.9259	120.182	OK	2755.43	92.2002	30.1126	154.288	OK	OK	0.386043
ENSG000001	-	-	ENSG0000013444	'GRP	-	18:59220167-59230774	-	-	IGV_LINK	0	0	0	0	OK	0	0	0	0	OK	NOTEST	0
ENSG000001	-	-	ENSG0000013444	'KIAA1468	-	18:62187257-62307829	-	-	IGV_LINK	807.777	17.4276	0	35.795	OK	729.076	16.8969	0	34.9234	OK	OK	-0.04461
ENSG000001	-	-	ENSG0000013445	'FBXO18	-	10:5890202-5978187	-	-	IGV_LINK	1206.33	32.8388	0	69.0099	OK	955.946	25.0815	0	52.2142	OK	OK	-0.38878
ENSG000001	-	-	ENSG0000013445	'RBM17	-	10:6088986-6117457	-	-	IGV_LINK	1338.21	63.334	18.8842	107.784	OK	1107.54	59.4751	13.9121	105.038	OK	OK	-0.09069
ENSG000001	-	-	ENSG0000013446	'IL2RA	-	10:6010688-6062325	-	-	IGV_LINK	75.6382	2.01053	0	4.30607	OK	346.492	7.95964	1.96927	13.95	OK	OK	1.98513
ENSG000001	-	-	ENSG0000013446	'ANKRD16	-	10:5861616-5889906	-	-	IGV_LINK	105.508	4.06269	0	8.79547	OK	100.529	4.08002	0	8.5111	OK	OK	0.00614
ENSG000001	-	-	ENSG0000013446	'ECHDC3	-	10:11742365-11764070	-	-	IGV_LINK	680.744	32.1074	9.97119	54.2436	OK	373.303	16.1509	4.46445	27.8373	OK	OK	-0.99129
ENSG000001	-	-	ENSG0000013447	'IL15RA	-	10:5890202-5978187	-	-	IGV_LINK	453.973	21.1018	0	47.0685	OK	311.43	14.8511	0	33.9701	OK	OK	-0.5068
ENSG000001	-	-	ENSG0000013448	'CCNH	-	5:87267887-87413019	-	-	IGV_LINK	496.497	28.7657	0	58.5162	OK	662.046	31.8874	4.58047	59.1943	OK	OK	0.148635
ENSG000001	-	-	ENSG0000013448	'HRH4	-	18:24460628-24479957	-	-	IGV_LINK	7.75776	0.157052	0	0.425303	OK	9.28102	0.149683	0	0.399425	OK	NOTEST	-0.06934
ENSG000001	-	-	ENSG0000013449	'TMEM241	-	18:23134563-23437961	-	-	IGV_LINK	96.0023	2.98377	0	9.5203	OK	79.4043	2.97849	0	9.82255	OK	OK	-0.00256
ENSG000001	-	-	ENSG0000013450	'KCTD1	-	18:26454909-27190698	-	-	IGV_LINK	178.429	7.07822	0	16.5269	OK	188.714	8.15852	0	17.3661	OK	OK	0.204921
ENSG000001	-	-	ENSG0000013450	'CABLES1	-	18:23134563-23437961	-	-	IGV_LINK	554.68	9.44901	1.45006	17.448	OK	419.709	8.11412	0	16.7591	OK	OK	-0.21973
ENSG000001	-	-	ENSG0000013451	'DOCK2	-	5:169637246-170083382	-	-	IGV_LINK	2488.3	30.2782	8.0389	52.5174	OK	1957.27	22.4696	6.1812	38.7581	OK	OK	-0.4303
ENSG000001	-	-	ENSG0000013453	'EMP1	-	12:13196715-13219939	-	-	IGV_LINK	646.804	15.1404	0	32.0041	OK	1343.69	37.7199	0	75.6114	OK	OK	1.31692
ENSG000001	-	-	ENSG0000013453	'SOX5	-	12:23529499-23951032	-	-	IGV_LINK	28.1219	0.856278	0	2.38059	OK	29.9055	0.705372	0	2.13113	OK	OK	-0.2797
ENSG000001	-	-	ENSG0000013453	'RERG	-	12:15107782-15597399	-	-	IGV_LINK	818.444	23.9668	6.62321	41.3105	OK	1660.27	49.0038	19.0172	78.9904	OK	OK	1.03185
ENSG000001	-	-	ENSG0000013453	'SLCO1B1	-	12:20810701-21239246	-	-	IGV_LINK	0	0	0	0	OK	0	0	0	0	OK	NOTEST	0
ENSG000001	-	-	ENSG0000013453	'KLRD1	-	12:10226057-10329600	-	-	IGV_LINK	175.519	3.17659	0	7.91416	OK	213.464	4.57854	0	12.0059	OK	OK	0.527408
ENSG000001	-	-	ENSG0000013454	'KLRC1	-	12:10412311-10454685	-	-	IGV_LINK	31.0311	1.6	0	3.92722	OK	10.3122	0.471207	0	1.30971	OK	OK	-1.76364
ENSG000001	-	-	ENSG0000013454	'SPX	-	12:21526306-21604847	-	-	IGV_LINK	509.103	17.8734	5.61506	30.1317	OK	394.959	13.7134	3.99258	23.4343	OK	OK	-0.38222
ENSG000001	-	-	ENSG0000013455	'PRH2	-	12:10824959-11176016	-	-	IGV_LINK	0	0	0	0	OK	0	0	0	0	OK	NOTEST	0
ENSG000001	-	-	ENSG0000013456	'LRP4	-	11:46846411-46918642	-	-	IGV_LINK	145.458	1.55371	0	3.20578	OK	418.677	4.49816	0.525268	8.47104	OK	OK	1.53362
ENSG000001	-	-	ENSG0000013457	'MYBPC3	-	11:47331396-47352702	-	-	IGV_LINK	14.5458	0.202609	0	0.468162	OK	34.0304	0.474243	0	0.972105	OK	NOTEST	1.22693
ENSG000001	-	-	ENSG0000013457	'DDB2	-	11:47214464-47239240	-	-	IGV_LINK	1109.36	46.36	14.3552	78.3648	OK	1624.18	67.9262	22.627	113.225	OK	OK	0.551089
ENSG000001	-	-	ENSG0000013457	'ACP2	-	11:47239301-47269032	-	-	IGV_LINK	736.018	22.7532	1.61377	43.8927	OK	1549.93	48.4837	5.91232	91.0551	OK	OK	1.09143
ENSG000001	-	-	ENSG0000013458	'USP26	-	X:133024630-133097109	-	-	IGV_LINK	0	0	0	0	OK	0	0	0	0	OK	NOTEST	0
ENSG000001	-	-	ENSG0000013459	'RTL8C	-	X:135032365-135033546	-	-	IGV_LINK	913.477	51.5368	21.4482	81.6253	OK	977.601	55.7579	23.1913	88.3246	OK	OK	0.113575
ENSG000001	-	-	ENSG0000013459	'RAB33A	-	X:130171648-130184870	-	-	IGV_LINK	50.4255	2.61798	0.013796	5.22216	OK	35.0616	1.82226	0	3.71624	OK	OK	-0.52273
ENSG000001	-	-	ENSG0000013459	'SOX3	-	X:140502984-140505116	-	-	IGV_LINK	0	0	0	0	OK	0	0	0	0	OK	NOTEST	0
ENSG000001	-	-	ENSG0000013459	'RBMX2	-	X:130401968-130413343	-	-	IGV_LINK	320.008	17.6726	2.18996	33.1553	OK	360.929	18.2172	3.35122	33.0831	OK	OK	0.043782
ENSG000001	-	-	ENSG0000013460	'STK26	-	X:132023264-132075943	-	-	IGV_LINK	111.518	3.27009	0.225093	6.31508	OK	101.06	2.61918	0.069278	5.16908	OK	OK	-0.32021
ENSG000001	-	-	ENSG0000013461	'FOLH1B	-	11:89639226-89698718	-	-	IGV_LINK	0	0	0	0	OK	0	0	0	0	OK	NOTEST	0
ENSG000001	-	-	ENSG0000013462	'PIWIL4	-	11:94543839-94925521	-	-	IGV_LINK	286.253	8.71581	0	24.2894	OK	103.178	3.47044	0	11.2773	OK	OK	-1.32852
ENSG000001	-	-	ENSG0000013464	'MTNR1B	-	11:92969719-92985066	-	-	IGV_LINK	0	0	0	0	OK	0	0	0	0	OK	NOTEST	0
ENSG000001	-	-	ENSG0000013464	'PUM1	-	1:30931505-31065991	-	-	IGV_LINK	1661.13	25.966	3.85628	48.0756	OK	1688.12	24.5928	6.25204	42.9336	OK	OK	-0.07838
ENSG000001	-	-	ENSG0000013466	'SPOCD1	-	1:31789129-31816051	-	-	IGV_LINK	0.969721	0.030951	0	0.541757	OK	7.21857	0.185089	0	0.748737	OK	NOTEST	2.58014
ENSG000001	-	-	ENSG0000013468	'YARS	-	1:32775236-32858879	-	-	IGV_LINK	1140.39	38.3136	4.59987	72.0272	OK	1326.16	43.6282	9.62349	77.6329	OK	OK	0.187406
ENSG000001	-	-	ENSG0000013468	'PHC2	-	1:33306765-33431052	-	-	IGV_LINK	2712.31	69.2227	20.9772	117.468	OK	4711.67	128.531	28.9216	228.14	OK	OK	0.892798
ENSG000001	-	-	ENSG0000013469	'CDCA8	-	1:37692417-37709719	-	-	IGV_LINK	86.3051	2.22204	0	4.5075	OK	100.029	2.63146	0.330609	4.93232	OK	OK	0.243979
ENSG000001	-	-	ENSG0000013469	'GNL2	-	1:37556918-37595935	-	-	IGV_LINK	864.021	27.6532	0	57.8305	OK	949.758	29.4685	0.411294	58.5256	OK	OK	0.091725
ENSG000001	-	-	ENSG0000013469	'AGO4	-	1:35808171-35857890	-	-	IGV_LINK	705.957	8.26549	0	22.9118	OK	736.295	10.2597	0	30.0787	OK	OK	0.311814
ENSG000001	-	-	ENSG0000013470	'HOOK1	-	1:59814785-59876378	-	-	IGV_LINK	956.144	14.1968	2.63515	25.7585	OK	468.176	7.15199	0	14.7216	OK	OK	-0.98915
ENSG000001	-	-	ENSG0000013471	'CYP2J2	-	1:59893307-59926790	-	-	IGV_LINK	686.562	22.1225	8.62303	35.622	OK	268.118	8.7326	2.73581	14.7294	OK	OK	-1.34103
ENSG000001	-	-	ENSG0000013471	'BTF3L4	-	1:52020130-52090716	-	-	IGV_LINK	607.045	24.9114	0	53.507	OK	598.11	25.708	0	61.1943	OK	OK	0.045409
ENSG000001	-	-	ENSG0000013474	'ZCCHC11	-	1:52404563-52553487	-	-	IGV_LINK	627.409	16.3238	0	40.4102	OK	637.297	17.3815	0	40.6785	OK	OK	0.090575
ENSG000001	-	-	ENSG0000013474	'PRPF38A	-	1:52404563-52553487	-	-	IGV_LINK	887.294	21.0898	0	42.9594	OK	781.669	19.7488	0	40.6062	OK	OK	-0.09478
ENSG000001	-	-	ENSG0000013475	'DSC2	-	18:31058839-31162856	-	-	IGV_LINK	556.62	5.49613	1.95821	9.03404	OK	600.173	6.04548	2.23105	9.8599	OK	OK	0.137441
ENSG000001	-	-	ENSG0000013475	'DSG3	-	18:31447794-31478702	-	-	IGV_LINK	1.93944	0.02042	0	0.062535	OK	1.03122	0.01086	0	0.040646	OK	NOTEST	-0.91097
ENSG000001	-	-	ENSG0000013475	'RNF138	-	18:32018371-32470484	-	-	IGV_LINK	214.308	12.9593	0	40.7685	OK	199.026	9.79073	0	34.0018	OK	OK	-0.4045
ENSG000001	-	-	ENSG0000013475	'ELP2	-	18:36129443-36187448	-	-	IGV_LINK	1583.55	43.5139	5.82608	81.2017	OK	1730.4	48.337	6.6087	90.0652	OK	OK	0.151651
ENSG000001	-	-	ENSG0000013476	'DSG1	-	18:31318088-31426988	-	-	IGV_LINK	0	0	0	0	OK	1.03122	0.034524	0	0.132058	OK	NOTEST	inf
ENSG000001	-	-	ENSG0000013476	'DSC3	-	18:30990007-31042815	-	-	IGV_LINK	0	0	0	0	OK	0	0	0	0	OK	NOTEST	0
ENSG000001	-	-	ENSG0000013476	'DSC1	-	18:31058839-31162856	-	-	IGV_LINK	0.969721	0.013472	0	0.211883	OK	2.06245	0.028676	0	0.324292	OK	NOTEST	1.08985
ENSG000001	-	-	ENSG0000013476	'DTNA	-	18:34493289-34891844	-	-	IGV_LINK	145.458	2.37041	0	5.44689	OK	111.372	2.61049	0	6.39951	OK	OK	0.13918
ENSG000001	-	-	ENSG0000013477	'FHOD3	-	18:36297713-37232172	-	-	IGV_LINK	451.89	5.83341	0	12.0522	OK	892.01	11.3488	1.65326	21.0443	OK	OK	0.960124
ENSG000001	-	-	ENSG0000013477	'TPGS2	-	18:36297713-37232172	-	-	IGV_LINK	832.99	36.5641	1.24108	71.8871	OK	981.726	43.3612	0	88.1183	OK	OK	0.245977
ENSG000001	-	-	ENSG0000013478	'DAGLA	-	11:61680432-61867354	-	-	IGV_LINK	88.2446	0.890516	0	3.84392	OK	107.247	1.08252	0	5.0414	OK	OK	0.281675
ENSG000001	-	-	ENSG0000013480	'SLC43A3	-	11:57386793-57427580	-	-	IGV_LINK	1540.93	43.0556	12.3472	73.764	OK	2635.72	75.0993	20.86	129.339	OK	OK	0.802597
ENSG000001	-	-	ENSG0000013480	'TIMM10	-	11:57528462-57530803	-	-	IGV_LINK	391.767	45.4085	16.0138	74.8032	OK	416.615	47.4686	17.2089	77.7284	OK	OK	0.064013
ENSG000001	-	-	ENSG0000013481	'GIF	-	11:59829267-59845501	-	-	IGV_LINK	0.969721	0.067931	0	0.470689	OK	0	0	0	0	OK	NOTEST	'-inf
ENSG000001	-	-	ENSG0000013481	'DHX34	-	19:47349280-47382704	-	-	IGV_LINK	578.923	11.4686	0.035658	22.9015	OK	620.797	11.1534	1.49203	20.8147	OK	OK	-0.0402
ENSG000001	-	-	ENSG0000013481	'APLNR	-	11:57233576-57237314	-	-	IGV_LINK	256.976	4.8197	1.21595	8.42344	OK	643.484	11.5737	4.23022	18.9173	OK	OK	1.26384
ENSG000001	-	-	ENSG0000013482	'FADS2	-	11:61680432-61867354	-	-	IGV_LINK	1005.6	26.608	0	66.0358	OK	1798.46	49.7483	0	114.969	OK	OK	0.902789
ENSG000001	-	-	ENSG0000013482	'TMEM258	-	11:61680432-61867354	-	-	IGV_LINK	864.021	118.31	0	246.784	OK	949.758	129.041	0	299.433	OK	OK	0.125257
ENSG000001	-	-	ENSG0000013482	'TCN1	-	11:59852799-59866575	-	-	IGV_LINK	0.969721	0.106846	0	0.681131	OK	8.2498	0.585688	0	1.61002	OK	OK	2.4546
ENSG000001	-	-	ENSG0000013483	'C5AR2	-	19:47332146-47347327	-	-	IGV_LINK	80.4868	1.56749	0	3.57109	OK	138.184	2.63979	0	5.99757	OK	OK	0.75197
ENSG000001	-	-	ENSG0000013485	'TMEM165	-	4:55395956-55547889	-	-	IGV_LINK	886.325	36.7702	6.33599	67.2045	OK	1575.71	60.2857	17.028	103.543	OK	OK	0.713278
ENSG000001	-	-	ENSG0000013485	'CLOCK	-	4:55395956-55547889	-	-	IGV_LINK	503.285	3.24549	0	9.37264	OK	418.677	2.47286	0	6.22953	OK	OK	-0.39225
ENSG000001	-	-	ENSG0000013485	'PDGFRA	-	4:53377642-54298247	-	-	IGV_LINK	8.72748	0.076808	0	0.724351	OK	12.9842	0.360978	0	2.65813	OK	NOTEST	2.23258
ENSG000001	-	-	ENSG0000013486	'GGACT	-	13:100089014-100589528	-	-	IGV_LINK	84.3657	4.89384	0	20.2839	OK	142.21	6.47353	0	22.8032	OK	OK	0.403585
ENSG000001	-	-	ENSG0000013487	'COL4A2	-	13:110148962-110513027	-	-	IGV_LINK	9935.76	93.4839	0	212.672	OK	15445.7	145.113	0	365.476	OK	OK	0.634385
ENSG000001	-	-	ENSG0000013487	'CLDN10	-	13:95433603-95644703	-	-	IGV_LINK	286.068	13.4047	0	34.5133	OK	305.243	14.2263	0	36.1564	OK	OK	0.085823
ENSG000001	-	-	ENSG0000013487	'DZIP1	-	13:95433603-95644703	-	-	IGV_LINK	1451.67	29.3377	3.29843	55.3769	OK	1455.06	32.3354	2.25652	62.4143	OK	OK	0.140361
ENSG000001	-	-	ENSG0000013488	'UBAC2	-	13:99200773-99386434	-	-	IGV_LINK	1298.46	47.9008	17.745	78.0567	OK	1549.93	57.22	20.1511	94.2888	OK	OK	0.256468
ENSG000001	-	-	ENSG0000013488	'ARGLU1	-	13:106541672-106568164	-	-	IGV_LINK	2571.7	115.741	4.90342	226.578	OK	1899.52	88.7346	2.72852	174.741	OK	OK	-0.38333
ENSG000001	-	-	ENSG0000013489	'BIVM	-	13:102799048-102876001	-	-	IGV_LINK	1115.46	30.4797	0	80.5783	OK	618.418	15.5821	0	44.1406	OK	OK	-0.96796
ENSG000001	-	-	ENSG0000013489	'ERCC5	-	13:102799048-102876001	-	-	IGV_LINK	492.882	30.7105	0	91.9289	OK	475.353	29.0384	0	80.5006	OK	OK	-0.08077
ENSG000001	-	-	ENSG0000013490	'TPP2	-	13:102597002-102679958	-	-	IGV_LINK	994.933	14.3081	4.29066	24.3255	OK	1100.32	19.3734	4.15573	34.5911	OK	OK	0.437249
ENSG000001	-	-	ENSG0000013490	'KDELC1	-	13:102784280-102799007	-	-	IGV_LINK	252.127	7.52467	2.27129	12.7781	OK	211.401	6.31577	1.72375	10.9078	OK	OK	-0.25267
ENSG000001	-	-	ENSG0000013490	'CARS2	-	13:110641411-110723339	-	-	IGV_LINK	1322.7	55.197	11.3895	99.0045	OK	2404.82	95.275	22.2826	168.267	OK	OK	0.787506
ENSG000001	-	-	ENSG0000013490	'ARHGAP32	-	11:128965059-129279324	-	-	IGV_LINK	1174.33	9.5769	0.191439	18.9624	OK	917.79	7.2662	0.084107	14.4483	OK	OK	-0.39836
ENSG000001	-	-	ENSG0000013491	'STT3A	-	11:125569215-125681123	-	-	IGV_LINK	1877.38	52.3347	10.2869	94.3826	OK	2159.39	63.8609	3.80813	123.914	OK	OK	0.287164
ENSG000001	-	-	ENSG0000013491	'ADAMTS8	-	11:130404924-130428993	-	-	IGV_LINK	26.1825	0.57957	0	1.24218	OK	9.28102	0.227994	0	0.556488	OK	OK	-1.34598
ENSG000001	-	-	ENSG0000013494	'ACRV1	-	11:125569215-125681123	-	-	IGV_LINK	0.969721	0.062853	0	2.19521	OK	1.03122	0.093685	0	3.46491	OK	NOTEST	0.575841
ENSG000001	-	-	ENSG0000013495	'ETS1	-	11:128458760-128587558	-	-	IGV_LINK	4002.04	56.4655	12.6486	100.282	OK	3746.44	51.208	13.1545	89.2615	OK	OK	-0.141
ENSG000001	-	-	ENSG0000013495	'SLC37A2	-	11:125063066-125090312	-	-	IGV_LINK	783.534	16.5206	5.11577	27.9254	OK	1342.65	27.6026	9.99016	45.2151	OK	OK	0.740543
ENSG000001	-	-	ENSG0000013496	'KLB	-	4:39406852-39451536	-	-	IGV_LINK	0.969721	0.009248	0	0.036221	OK	0	0	0	0	OK	NOTEST	'-inf
ENSG000001	-	-	ENSG0000013497	'TMED7	-	5:115578641-115632992	-	-	IGV_LINK	643.067	23.2319	1.12832	45.3354	OK	791.721	25.385	1.60352	49.1664	OK	OK	0.127869

ENSG000001	-	-	ENSG0000013498	'APC	-	5:112707497-112922539	-	-	IGV_LINK	558.559	3.84333	0	15.7554	OK	531.081	3.18548	0	11.245	OK	OK	-0.27085
ENSG000001	-	-	ENSG0000013498	'NREP	-	5:111496032-112017309	-	-	IGV_LINK	1042.45	36.5499	7.73606	65.3638	OK	1030.19	37.6104	8.55654	66.6642	OK	OK	0.041263
ENSG000001	-	-	ENSG0000013498	'WDR36	-	5:111070061-111130502	-	-	IGV_LINK	373.342	4.46137	1.20372	7.71903	OK	357.835	3.98041	1.04406	6.91676	OK	OK	-0.16457
ENSG000001	-	-	ENSG0000013499	'OSTF1	-	9:75088542-75147265	-	-	IGV_LINK	554.68	27.2655	10.747	43.7841	OK	611.516	30.0895	12.0865	48.0926	OK	OK	0.142183
ENSG000001	-	-	ENSG0000013500	'RFK	-	9:76385516-76394517	-	-	IGV_LINK	298.674	12.0464	0.196538	23.8962	OK	350.616	14.9137	0.768025	29.0593	OK	OK	0.308039
ENSG000001	-	-	ENSG0000013501	'UBQLN1	-	9:83659962-83713378	-	-	IGV_LINK	1522.46	28.1507	3.22969	53.0718	OK	1654.08	29.2589	7.19723	51.3205	OK	OK	0.055702
ENSG000001	-	-	ENSG0000013504	'NAA35	-	9:85941145-86022298	-	-	IGV_LINK	332.614	7.82636	2.60207	13.0507	OK	366.085	8.41457	2.95455	13.8746	OK	OK	0.104549
ENSG000001	-	-	ENSG0000013504	'C9orf40	-	9:74946580-74952886	-	-	IGV_LINK	26.1825	0.684864	0	1.41914	OK	43.3114	1.13352	0	2.28387	OK	OK	0.726919
ENSG000001	-	-	ENSG0000013504	'ANXA1	-	9:73151756-73170393	-	-	IGV_LINK	3520.09	152.776	63.2331	242.32	OK	6289.44	272.762	103.794	441.731	OK	OK	0.836222
ENSG000001	-	-	ENSG0000013504	'CTSL	-	9:87725518-87731393	-	-	IGV_LINK	3581.18	159.193	64.903	253.483	OK	7398.01	337.879	120.801	554.956	OK	OK	1.08573
ENSG000001	-	-	ENSG0000013504	'TMEM2	-	9:71683365-71816690	-	-	IGV_LINK	1461.37	19.2467	4.79274	33.7006	OK	3774.28	54.2069	11.3279	97.0858	OK	OK	1.49386
ENSG000001	-	-	ENSG0000013504	'AGTPBP1	-	9:85546538-85742029	-	-	IGV_LINK	372.373	5.06323	1.58318	8.54327	OK	506.331	6.99417	2.06801	11.9203	OK	OK	0.466095
ENSG000001	-	-	ENSG0000013505	'GOLM1	-	9:86026145-86100173	-	-	IGV_LINK	3143.83	66.8562	17.7868	115.926	OK	5775.89	127.395	27.4757	227.314	OK	OK	0.930177
ENSG000001	-	-	ENSG0000013506	'FAM189A2	-	9:69324571-69392455	-	-	IGV_LINK	77.5776	2.71365	0	5.46686	OK	32.9992	1.19731	0	2.56225	OK	OK	-1.18043
ENSG000001	-	-	ENSG0000013506	'PSAT1	-	9:78297142-78330093	-	-	IGV_LINK	53.3346	1.49163	0	3.03566	OK	327.93	9.91714	3.16731	16.667	OK	OK	2.73304
ENSG000001	-	-	ENSG0000013507	'ISCA1	-	9:86264545-86283102	-	-	IGV_LINK	792.262	26.5821	10.119	43.0452	OK	1431.34	46.6338	20.1261	73.1415	OK	OK	0.81092
ENSG000001	-	-	ENSG0000013507	'ADAM19	-	5:157260121-157575775	-	-	IGV_LINK	448.981	4.18293	0	10.9065	OK	390.834	3.55584	0	8.46356	OK	OK	-0.23433
ENSG000001	-	-	ENSG0000013507	'HAVCR2	-	5:157085831-157255191	-	-	IGV_LINK	1161.73	26.0204	3.68087	48.3599	OK	923.978	22.5807	1.70839	43.453	OK	OK	-0.20455
ENSG000001	-	-	ENSG0000013508	'CCNJL	-	5:160251651-160345396	-	-	IGV_LINK	162.913	3.87109	0.264196	7.47799	OK	80.4355	2.24698	0	4.64234	OK	OK	-0.78476
ENSG000001	-	-	ENSG0000013509	'TAOK3	-	12:118149800-118372945	-	-	IGV_LINK	1043.42	27.053	0	67.8939	OK	1271.5	28.5137	0	68.4618	OK	OK	0.075867
ENSG000001	-	-	ENSG0000013509	'USP30	-	12:109023088-109088026	-	-	IGV_LINK	386.918	11.3362	0.100186	22.5722	OK	346.492	9.16996	0.002958	18.337	OK	OK	-0.30595
ENSG000001	-	-	ENSG0000013509	'SDS	-	12:113392444-113438276	-	-	IGV_LINK	481.951	20.8423	4.30695	37.3776	OK	900.259	38.7303	12.2839	65.1766	OK	OK	0.893948
ENSG000001	-	-	ENSG0000013509	'MSI1	-	12:120341329-120369180	-	-	IGV_LINK	21.3339	0.429014	0	1.01209	OK	5.15612	0.107129	0	0.479885	OK	NOTEST	-2.00167
ENSG000001	-	-	ENSG0000013510	'HNF1A	-	12:120941727-121016502	-	-	IGV_LINK	306.432	8.40929	0	18.7761	OK	676.484	18.5215	0	38.0193	OK	OK	1.13915
ENSG000001	-	-	ENSG0000013510	'FBXO21	-	12:117099480-117190531	-	-	IGV_LINK	849.475	13.4188	3.0707	23.7669	OK	878.604	16.2913	0.901973	31.6805	OK	OK	0.279844
ENSG000001	-	-	ENSG0000013511	'TBX3	-	12:114670253-114684164	-	-	IGV_LINK	128.973	1.92376	0	4.23677	OK	43.3114	0.61227	0	1.34836	OK	OK	-1.65169
ENSG000001	-	-	ENSG0000013511	'OASL	-	12:121019110-121039242	-	-	IGV_LINK	423.768	11.9584	2.68784	21.2289	OK	661.015	18.6453	5.62478	31.6658	OK	OK	0.640788
ENSG000001	-	-	ENSG0000013511	'HRK	-	12:116856143-116881441	-	-	IGV_LINK	5.81832	0.176376	0	0.718775	OK	4.1249	0.041398	0	0.466563	OK	NOTEST	-2.09104
ENSG000001	-	-	ENSG0000013511	'RNFT2	-	12:116738177-116853631	-	-	IGV_LINK	18.4247	0.702161	0	1.81278	OK	22.6869	0.734248	0	1.87908	OK	OK	0.064466
ENSG000001	-	-	ENSG0000013512	'P2RX4	-	12:121209856-121234106	-	-	IGV_LINK	1013.36	36.2016	12.3726	60.0306	OK	1125.07	41.2973	15.6916	66.903	OK	OK	0.189991
ENSG000001	-	-	ENSG0000013512	'BICDL1	-	12:119989868-120094494	-	-	IGV_LINK	1062.81	34.6744	5.24876	64.1001	OK	980.695	30.3259	4.67074	55.9811	OK	OK	-0.19332
ENSG000001	-	-	ENSG0000013514	'DTX1	-	12:113056708-113098028	-	-	IGV_LINK	37.8191	1.02639	0	2.27829	OK	25.7806	0.863875	0	2.01209	OK	OK	-0.24869
ENSG000001	-	-	ENSG0000013514	'TRAFD1	-	12:112125500-112153609	-	-	IGV_LINK	1828.89	44.6994	17.547	71.8518	OK	1118.88	28.2944	9.45766	47.1311	OK	OK	-0.65974
ENSG000001	-	-	ENSG0000013516	'DMTF1	-	7:87151422-87220587	-	-	IGV_LINK	2165.39	67.8454	4.61765	131.073	OK	1522.09	50.0033	1.76545	98.2412	OK	OK	-0.44023
ENSG000001	-	-	ENSG0000013517	'OCM2	-	7:97984683-97991169	-	-	IGV_LINK	0	0	0	0	OK	0	0	0	0	OK	NOTEST	0
ENSG000001	-	-	ENSG0000013518	'TMEM243	-	7:87151422-87220587	-	-	IGV_LINK	427.647	25.8555	0	62.4542	OK	625.954	38.2152	0	81.3866	OK	OK	0.563674
ENSG000001	-	-	ENSG0000013520	'CCDC146	-	7:76959834-77416400	-	-	IGV_LINK	3624.29	158.041	0	331.764	OK	260.526	8.75174	0	37.6579	OK	OK	-4.17458
ENSG000001	-	-	ENSG0000013521	'TMEM60	-	7:77793727-77798580	-	-	IGV_LINK	176.489	11.6948	2.83529	20.5543	OK	183.558	12.1797	3.04315	21.3162	OK	OK	0.05861
ENSG000001	-	-	ENSG0000013521	'CD36	-	7:80312573-80679277	-	-	IGV_LINK	345.22	9.86455	1.34885	18.3803	OK	1092.07	37.4437	7.21978	67.6675	OK	OK	1.9244
ENSG000001	-	-	ENSG0000013522	'UGT2A3	-	4:68928462-68951791	-	-	IGV_LINK	2452.42	57.261	23.5567	90.9654	OK	3146.27	79.5097	29.1526	129.867	OK	OK	0.473578
ENSG000001	-	-	ENSG0000013522	'CSN2	-	4:69955255-69961007	-	-	IGV_LINK	0	0	0	0	OK	0	0	0	0	OK	NOTEST	0
ENSG000001	-	-	ENSG0000013522	'UGT2B28	-	4:69280498-69295050	-	-	IGV_LINK	0	0	0	0	OK	0	0	0	0	OK	NOTEST	0
ENSG000001	-	-	ENSG0000013524	'PNPLA8	-	7:108470421-108574850	-	-	IGV_LINK	413.101	10.6542	0	24.6664	OK	426.927	9.46709	0	21.723	OK	OK	-0.17043
ENSG000001	-	-	ENSG0000013524	'HILPDA	-	7:128455839-128506602	-	-	IGV_LINK	1452.7	74.1574	26.3599	121.955	OK	7056.54	359.699	118.031	601.367	OK	OK	2.27813
ENSG000001	-	-	ENSG0000013524	'FAM71F1	-	7:128709060-128731743	-	-	IGV_LINK	3.87888	0.425901	0	1.32903	OK	5.15612	0.176729	0	0.677005	OK	NOTEST	-1.26898
ENSG000001	-	-	ENSG0000013524	'RINT1	-	7:105532084-105600875	-	-	IGV_LINK	311.28	11.2242	0.445809	22.0025	OK	362.991	11.4496	1.1012	21.798	OK	OK	0.028689
ENSG000001	-	-	ENSG0000013525	'SRPK2	-	7:105013424-105399308	-	-	IGV_LINK	416.01	10.6745	0	29.4705	OK	484.676	13.6588	0	34.5901	OK	OK	0.355662
ENSG000001	-	-	ENSG0000013525	'KCP	-	7:128830376-128910719	-	-	IGV_LINK	675.895	10.6398	0	23.7867	OK	1364.31	21.9248	0	44.6046	OK	OK	1.04309
ENSG000001	-	-	ENSG0000013526	'TES	-	7:116209233-116508541	-	-	IGV_LINK	760.261	22.9168	0	47.0208	OK	1120.94	37.8967	0.571619	75.2218	OK	OK	0.725668
ENSG000001	-	-	ENSG0000013527	'MDFIC	-	7:114922153-115019202	-	-	IGV_LINK	643.894	14.7873	1.37598	28.1986	OK	699.171	17.0364	0	34.9317	OK	OK	0.204259
ENSG000001	-	-	ENSG0000013529	'MTO1	-	6:73461577-73509236	-	-	IGV_LINK	541.104	17.7682	0.179901	35.3565	OK	342.367	10.7979	0	22.6215	OK	OK	-0.71854
ENSG000001	-	-	ENSG0000013529	'ADGRB3	-	6:68635366-69389511	-	-	IGV_LINK	108.609	1.49221	0	3.12826	OK	5.15612	0.157893	0	0.686171	OK	OK	-3.24043
ENSG000001	-	-	ENSG0000013529	'ANKRD6	-	6:89433169-89638753	-	-	IGV_LINK	604.136	17.3531	0	37.0841	OK	452.708	14.363	0	31.9058	OK	OK	-0.27284
ENSG000001	-	-	ENSG0000013531	'HTR1B	-	6:77461752-77463773	-	-	IGV_LINK	5.81832	0.177386	0	0.421286	OK	0	0	0	0	OK	NOTEST	'-inf
ENSG000001	-	-	ENSG0000013531	'KHDC1	-	6:73209082-73310365	-	-	IGV_LINK	76.6079	3.09443	0	7.37719	OK	40.2178	1.47262	0	4.00327	OK	OK	-1.07129
ENSG000001	-	-	ENSG0000013531	'CEP162	-	6:84124240-84227635	-	-	IGV_LINK	264.734	5.09908	0.849525	9.34863	OK	173.246	3.89494	0	7.81476	OK	OK	-0.38864
ENSG000001	-	-	ENSG0000013531	'SYNCRIP	-	6:85505495-85643792	-	-	IGV_LINK	2039.88	37.3683	0	79.667	OK	1495.69	27.9011	0	56.8291	OK	OK	-0.42149
ENSG000001	-	-	ENSG0000013531	'SNX14	-	6:85505495-85643792	-	-	IGV_LINK	903.595	42.8875	0	114.055	OK	578.34	21.6306	0	56.9163	OK	OK	-0.98749
ENSG000001	-	-	ENSG0000013531	'NT5E	-	6:85449583-85495791	-	-	IGV_LINK	176.489	4.02283	0.011788	8.03387	OK	192.839	4.2088	0.240419	8.17718	OK	OK	0.065198
ENSG000001	-	-	ENSG0000013532	'MRAP2	-	6:84033755-84090881	-	-	IGV_LINK	3.87888	0.110376	0	0.281109	OK	0	0	0	0	OK	NOTEST	'-inf
ENSG000001	-	-	ENSG0000013533	'EPHA7	-	6:93240019-93419547	-	-	IGV_LINK	939.659	8.98452	3.37449	14.5945	OK	423.833	4.17076	1.21408	7.12744	OK	OK	-1.10713
ENSG000001	-	-	ENSG0000013533	'AKIRIN2	-	6:87675071-87702209	-	-	IGV_LINK	376.252	16.5188	2.81564	30.222	OK	404.24	17.3415	3.69757	30.9854	OK	OK	0.070119
ENSG000001	-	-	ENSG0000013533	'ORC3	-	6:87590066-87667453	-	-	IGV_LINK	470.314	12.4532	2.95696	21.9495	OK	333.086	8.88354	1.56836	16.1987	OK	OK	-0.48731
ENSG000001	-	-	ENSG0000013533	'LCA5	-	6:79484990-79537458	-	-	IGV_LINK	240.491	3.12285	0.897617	5.34809	OK	103.122	1.33681	0.021548	2.65206	OK	OK	-1.22407
ENSG000001	-	-	ENSG0000013534	'MAP3K7	-	6:90513572-90587045	-	-	IGV_LINK	691.411	10.1785	2.52891	17.8281	OK	533.143	7.36858	2.59826	12.1389	OK	OK	-0.46607
ENSG000001	-	-	ENSG0000013534	'CGA	-	6:87085497-87095406	-	-	IGV_LINK	0	0	0	0	OK	0	0	0	0	OK	NOTEST	0
ENSG000001	-	-	ENSG0000013535	'GJA10	-	6:89894468-89921760	-	-	IGV_LINK	0	0	0	0	OK	0	0	0	0	OK	NOTEST	0
ENSG000001	-	-	ENSG0000013536	'PRR5L	-	11:36296287-36465204	-	-	IGV_LINK	335.523	17.2569	0	34.713	OK	241.307	8.56421	0	17.8478	OK	OK	-1.01078
ENSG000001	-	-	ENSG0000013536	'LMO2	-	11:33858575-33892289	-	-	IGV_LINK	484.86	18.946	6.41518	31.4768	OK	651.734	25.3777	8.84767	41.9076	OK	OK	0.421667
ENSG000001	-	-	ENSG0000013536	'PHF21A	-	11:45929322-46121178	-	-	IGV_LINK	1271.3	23.9206	0	51.6653	OK	1241.59	21.2388	1.0619	41.4157	OK	OK	-0.17155
ENSG000001	-	-	ENSG0000013537	'NAT10	-	11:34105601-34147670	-	-	IGV_LINK	1206.33	21.2391	3.55284	38.9253	OK	1366.37	23.3925	6.5266	40.2583	OK	OK	0.139322
ENSG000001	-	-	ENSG0000013537	'EHF	-	11:34621092-34661057	-	-	IGV_LINK	20.3641	0.498179	0	1.41295	OK	34.0304	0.708992	0	1.90375	OK	OK	0.509106
ENSG000001	-	-	ENSG0000013537	'ELF5	-	11:34478792-34513805	-	-	IGV_LINK	0	0	0	0	OK	2.06245	0.056878	0	0.363219	OK	NOTEST	inf
ENSG000001	-	-	ENSG0000013537	'PRRG4	-	11:32829942-32858123	-	-	IGV_LINK	310.311	3.26416	1.08523	5.4431	OK	575.424	6.05419	2.39914	9.70924	OK	OK	0.891221
ENSG000001	-	-	ENSG0000013538	'CAPRIN1	-	11:34051682-34101156	-	-	IGV_LINK	3050.74	55.2524	17.7051	92.7998	OK	3027.68	53.1978	21.0837	85.312	OK	OK	-0.05467
ENSG000001	-	-	ENSG0000013539	'ATP5G2	-	12:53632725-53677408	-	-	IGV_LINK	3867.25	427.559	167.927	687.19	OK	4033.12	441.975	177.773	706.176	OK	OK	0.047841
ENSG000001	-	-	ENSG0000013539	'DNAJC14	-	12:55743279-55842966	-	-	IGV_LINK	658.44	13.6859	0	33.0156	OK	748.83	15.0976	0	38.5022	OK	OK	0.141627
ENSG000001	-	-	ENSG0000013540	'CD63	-	12:55725322-55730852	-	-	IGV_LINK	9262.77	761.975	250.78	1273.17	OK	9261.43	759.578	224.073	1295.08	OK	OK	-0.00455
ENSG000001	-	-	ENSG0000013540	'PRPH	-	12:49292630-49331731	-	-	IGV_LINK	10.6669	0.744224	0	3.11557	OK	10.3122	0.377339	0	1.94387	OK	OK	-0.97988
ENSG000001	-	-	ENSG0000013540	'AVIL	-	12:57762333-57818704	-	-	IGV_LINK	169.701	7.48383	0	20.9091	OK	202.12	8.22662	0	27.3737	OK	OK	0.136523
ENSG000001	-	-	ENSG0000013540	'AMHR2	-	12:53423854-53431534	-	-	IGV_LINK	0.969721	0.076488	0	0.467427	OK	0	0	0	0	OK	NOTEST	'-inf
ENSG000001	-	-	ENSG0000013541	'LACRT	-	12:54630810-54634895	-	-	IGV_LINK	0	0	0	0	OK	0	0	0	0	OK	NOTEST	0
ENSG000001	-	-	ENSG0000013541	'GDF11	-	12:55743279-55842966	-	-	IGV_LINK	244.37	1.86573	0	6.40946	OK	149.528	1.23341	0	6.27948	OK	OK	-0.59709
ENSG000001	-	-	ENSG0000013542	'GLS2	-	12:56449501-56488414	-	-	IGV_LINK	41.698	2.41895	0	8.36947	OK	36.0929	2.60044	0	9.88085	OK	OK	0.104375
ENSG000001	-	-	ENSG0000013542	'ITGA7	-	12:55681545-55724705	-	-	IGV_LINK	800.989	17.8913	0	44.5799	OK	604.298	15.2788	0	45.0263	OK	OK	-0.22773
ENSG000001	-	-	ENSG0000013542	'TESPA1	-	12:54948017-54984746	-	-	IGV_LINK	219.157	5.57096	0	11.5425	OK	125.809	2.69453	0	5.7642	OK	OK	-1.04789
ENSG000001	-	-	ENSG0000013543	'FAM186B	-	12:49568217-49764934	-	-	IGV_LINK	22.3036	0.734616	0	21.6363	OK	12.3747	0.320625	0	14.1111	OK	OK	-1.1961
ENSG000001	-	-	ENSG0000013543	'RDH5	-	12:55681545-55724705	-	-	IGV_LINK	226.881	18.8125	0	59.0036	OK	626.828	47.924	0	116.109	OK	OK	1.34906
ENSG000001	-	-	ENSG0000013543	'AGAP2	-	12:57723760-57760407	-	-	IGV_LINK	297.704	4.29739	0	16.0029	OK	311.43	3.81027	0	11.5368	OK	OK	-0.17357
ENSG000001	-	-	ENSG0000013544	'BLOC1S1	-	12:55681545-55724705	-	-	IGV_LINK	1011.55	135.9	10.7143	261.086	OK	888.37	120.559	0	252.229	OK	OK	-0.17281
ENSG000001	-	-	ENSG0000013544	'KRT85	-	12:52360005-52367481	-	-	IGV_LINK	0.969721	0.044998	0	0.253574	OK	0	0	0	0	OK	NOTEST	'-inf
ENSG000001	-	-	ENSG0000013544	'CDK4	-	12:57723760-57760407	-	-	IGV_LINK	1399.31	56.6009	0	118.482	OK	1571.59	61.1935	0	130.932	OK	OK	0.112553
ENSG000001	-	-	ENSG0000013544	'PPP1R1A	-	12:54549349-54588659	-	-	IGV_LINK	21.3339	0.828098	0	3.98914	OK	1950.05	74.9356	23.3843	126.487	OK	OK	6.4997
ENSG000001	-	-	ENSG0000013545	'TROAP	-	12:49292630-49331731	-	-	IGV_LINK	109.578	3.70991	0	7.7479	OK	122.716	4.31452	0	9.18078	OK	OK	0.217817
ENSG000001	-	-	ENSG0000013545	'TSPAN31	-	12:57723760-57760407	-	-	IGV_LINK	895.052	58.3785	0	137.28	OK	1291.09	76.9715	0	179.821	OK	OK	0.398889
ENSG000001	-	-	ENSG0000013545	'B4GALNT1	-	12:57610179-57633355	-	-	IGV_LINK	43.6374	0.733726	0	2.27721	OK	107.247	2.6059	0	6.19994	OK	OK	1.82847
ENSG000001	-	-	ENSG0000013545	'TFCP2	-	12:51093662-51173134	-	-	IGV_LINK	518.8	12.2502	3.7775	20.723	OK	546.549	13.9322	3.27592	24.5884	OK	OK	0.185608
ENSG000001	-	-	ENSG0000013546	'COQ10A	-	12:56266857-56270966	-	-	IGV_LINK	252.127	12.4404	2.20195	22.6789	OK	257.806	13.4556	1.57256	25.3386	OK	OK	0.113167
ENSG000001	-	-	ENSG0000013547	'FAIM2	-	12:49866895-49904217	-	-	IGV_LINK	7.75776	0.13473	0	0.630483	OK	35.0616	1.02262	0	3.1366	OK	OK	2.92413
ENSG000001	-	-	ENSG0000013547	'PAN2	-	12:56271698-56334053	-	-	IGV_LINK	2545.52	79.6902	0	212.217	OK	2292.41	68.9243	0	181.233	OK	OK	-0.20939
ENSG000001	-	-	ENSG0000013547	'ESPL1	-	12:53268298-53293643	-	-	IGV_LINK	196.853	2.85741	0	6.42301	OK	360.929	5.52637	0	11.3084	OK	OK	0.951624
ENSG000001	-	-	ENSG0000013547	'KRT87P	-	12:52232519-52309163	-	-	IGV_LINK	7.75776	0.318316	0	1.72539	OK	0	0	0	0	OK	NOTEST	'-inf
ENSG000001	-	-	ENSG0000013548	'KRT7	-	12:52232519-52309163	-	-	IGV_LINK	187.156	18.1397	0.154892	36.1246	OK	41.249	4.46267	0	10.4497	OK	OK	-2.02317
ENSG000001	-	-	ENSG0000013548	'ZC3H10	-	12:56118158-56144671	-	-	IGV_LINK	206.192	2.09659	0	10.0308	OK	158.809	1.60188	0	7.25434	OK	OK	-0.38828
ENSG000001	-	-	ENSG0000013548	'HNRNPA1	-	12:54230939-54351849	-	-	IGV_LINK	13209.5	691.679	0	1518.25	OK	10341.1	557.419	0	1239.81	OK	OK	-0.31134
ENSG000001	-	-	ENSG0000013550	'SLC26A10	-	12:57610179-57633355	-	-	IGV_LINK	56.2438	3.27029	0	9.97167	OK	51.5612	2.13401	0	6.687	OK	OK	-0.61585
ENSG000001	-	-	ENSG0000013550	'ACVR1B	-	12:51951666-51997078	-	-	IGV_LINK	1290.7	19.1355	3.52224	34.7488	OK	1708.74	23.2852	7.63391	38.9365	OK	OK	0.283159
ENSG000001	-	-	ENSG0000013550	'OS9	-	12:57693954-57721557	-	-	IGV_LINK	6828.77	178.372	51.3533	305.39	OK	8629.29	224.77	60.8168	388.723	OK	OK	0.333562
ENSG000001	-	-	ENSG0000013551	'MIP	-	12:56449501-56488414	-	-	IGV_LINK	2.90916	0.155411	0	1.50671	OK	5.15612	0.306994	0	2.31454	OK	NOTEST	0.982116
ENSG000001	-	-	ENSG0000013551	'KCNH3	-	12:49539156-49568153	-	-	IGV_LINK	10.6669	0.162713	0	1.19575	OK	6.18735	0.11338	0	1.28562	OK	NOTEST	-0.52117
ENSG000001	-	-	ENSG0000013552	'LTV1	-	6:143536844-143938471	-	-	IGV_LINK	328.735	11.0638	1.19425	20.9333	OK	283.587	9.55084	1.01897	18.0827	OK	OK	-0.21214
ENSG000001	-	-	ENSG0000013552	'MAP7	-	6:136342280-136552554	-	-	IGV_LINK	674.925	12.9199	5.04676	20.793	OK	170.152	3.3707	0.732947	6.00846	OK	OK	-1.93847
ENSG000001	-	-	ENSG0000013553	'CD164	-	6:109366513-109382457	-	-	IGV_LINK	2889.77	97.4424	26.0825	168.802	OK	2230.54	79.7427	13.4333	146.052	OK	OK	-0.2892
ENSG000001	-	-	ENSG0000013553	'AFG1L	-	6:108294893-108526796	-	-	IGV_LINK	83.396	2.69611	0	6.21945	OK	45.3739	1.12198	0	2.78534	OK	OK	-1.26483
ENSG000001	-	-	ENSG0000013554	'NHSL1	-	6:138402584-138697288	-	-	IGV_LINK	247.279	2.25984	0	7.29547	OK	203.151	2.55517	0	9.72903	OK	OK	0.177194
ENSG000001	-	-	ENSG0000013554	'AHI1	-	6:135283531-135497776	-	-	IGV_LINK	590.56	18.5261	0	37.8418	OK	449.614	14.3375	0	29.587	OK	OK	-0.36977
ENSG000001	-	-	ENSG0000013554	'HEY2	-	6:125578557-125761269	-	-	IGV_LINK	243.4	5.59198	1.22946	9.9545	OK	94.8727	2.18606	0.26959	4.10253	OK	OK	-1.35503
ENSG000001	-	-	ENSG0000013554	'PKIB	-	6:122471916-122726373	-	-	IGV_LINK	113.381	9.43236	0.008715	18.856	OK	118.591	9.46784	0.406303	18.5294	OK	OK	0.005416
ENSG000001	-	-	ENSG0000013556	'TAAR5	-	6:132588672-132589686	-	-	IGV_LINK	0	0	0	0	OK	0	0	0	0	OK	NOTEST	0
ENSG000001	-	-	ENSG0000013557	'NMBR	-	6:142058329-142089219	-	-	IGV_LINK	3.87888	0.18502	0	0.584734	OK	1.03122	0.049237	0	0.36136	OK	NOTEST	-1.90988
ENSG000001	-	-	ENSG0000013558	'SMPD2	-	6:109390214-109443919	-	-	IGV_LINK	205.581	9.43659	0.539564	18.3336	OK	170.152	7.88488	0.629544	15.1402	OK	OK	-0.25918
ENSG000001	-	-	ENSG0000013559	'MICAL1	-	6:109444061-109483237	-	-	IGV_LINK	2797.6	56.9111	4.65959	109.163	OK	2338.7	45.0176	6.2004	83.8348	OK	OK	-0.33822
ENSG000001	-	-	ENSG0000013559	'REPS1	-	6:138795925-138988261	-	-	IGV_LINK	491.648	14.5328	0.779141	28.2865	OK	270.181	7.50328	0.156037	14.8505	OK	OK	-0.95372
ENSG000001	-	-	ENSG0000013560	'STX11	-	6:144150525-144188370	-	-	IGV_LINK	204.611	6.57605	1.66807	11.484	OK	140.247	4.51038	0.886664	8.1341	OK	OK	-0.54397
ENSG000001	-	-	ENSG0000013560	'TEC	-	4:48135782-48269864	-	-	IGV_LINK	85.3354	1.50639	0	3.1231	OK	115.497	2.07015	0.187203	3.95311	OK	OK	0.458648
ENSG000001	-	-	ENSG0000013561	'PRADC1	-	2:73228005-73233238	-	-	IGV_LINK	124.124	7.83431	1.26391	14.4047	OK	157.777	9.77828	2.13846	17.4181	OK	OK	0.319775
ENSG000001	-	-	ENSG0000013562	'SEMA4F	-	2:74654227-74683853	-	-	IGV_LINK	140.609	2.26874	0	4.69482	OK	164.996	3.56433	0	8.15755	OK	OK	0.651739
ENSG000001	-	-	ENSG0000013562	'CCT7	-	2:73233419-73253021	-	-	IGV_LINK	2769.52	96.6178	35.5068	157.729	OK	2822.46	100.419	37.4883	163.349	OK	OK	0.055666
ENSG000001	-	-	ENSG0000013562	'EGR4	-	2:73290928-73293705	-	-	IGV_LINK	0.969721	0.024818	0	0.09727	OK	1.03122	0.026406	0	0.098887	OK	NOTEST	0.089472
ENSG000001	-	-	ENSG0000013563	'RAB11FIP5	-	2:72942035-73156721	-	-	IGV_LINK	1431.31	18.932	5.62472	32.2393	OK	1854.14	23.6757	5.92318	41.4282	OK	OK	0.32258
ENSG000001	-	-	ENSG0000013563	'SMYD5	-	2:73214221-73227237	-	-	IGV_LINK	572.135	16.6807	0	33.4732	OK	680.608	20.1987	0.014167	40.3832	OK	OK	0.276084
ENSG000001	-	-	ENSG0000013563	'DYSF	-	2:71453721-71686768	-	-	IGV_LINK	1851.2	20.0182	7.64062	32.3957	OK	2394.5	24.9084	8.05101	41.7658	OK	OK	0.315323

ENSG000001	-	-	ENSG0000013563	'CCDC142	-	2:74471957-74494886	-	-	IGV_LINK	256.235	9.12867	0	27.2023	OK	288.747	11.6647	0	33.6679	OK	OK	0.353667
ENSG000001	-	-	ENSG0000013563	'EMX1	-	2:72916259-72936071	-	-	IGV_LINK	263.764	10.5865	2.17751	18.9954	OK	302.149	13.1403	2.92215	23.3584	OK	OK	0.311772
ENSG000001	-	-	ENSG0000013564	'KCNMB4	-	12:70366275-70434292	-	-	IGV_LINK	41.698	0.515503	0	1.16123	OK	47.4363	0.586757	0	1.30653	OK	OK	0.186782
ENSG000001	-	-	ENSG0000013565	'USP15	-	12:61708258-62417431	-	-	IGV_LINK	945.477	36.7651	0	73.8105	OK	907.478	28.9057	1.69802	56.1134	OK	OK	-0.34698
ENSG000001	-	-	ENSG0000013567	'GNS	-	12:64713444-64759447	-	-	IGV_LINK	5114.31	61.1334	21.9366	100.33	OK	6744.21	79.6775	26.0636	133.291	OK	OK	0.382211
ENSG000001	-	-	ENSG0000013567	'CPM	-	12:68808175-68971570	-	-	IGV_LINK	346.19	9.49184	0	41.8141	OK	629.047	12.8059	0	45.0451	OK	OK	0.432047
ENSG000001	-	-	ENSG0000013567	'MDM2	-	12:68808175-68971570	-	-	IGV_LINK	997.268	19.2148	0	49.7044	OK	2636.99	44.5845	0	110.226	OK	OK	1.21433
ENSG000001	-	-	ENSG0000013568	'KLHL36	-	16:84648524-84667686	-	-	IGV_LINK	1988.9	29.0401	8.48009	49.6001	OK	1462.28	22.0298	6.85399	37.2056	OK	OK	-0.39859
ENSG000001	-	-	ENSG0000013569	'BCO1	-	16:81238447-81291142	-	-	IGV_LINK	168.731	4.3631	0.902081	7.82412	OK	346.492	8.44756	2.75623	14.1389	OK	OK	0.953181
ENSG000001	-	-	ENSG0000013569	'MPHOSPH6	-	16:82147797-82170226	-	-	IGV_LINK	98.9115	6.47704	0.64524	12.3088	OK	129.934	9.03049	1.05002	17.011	OK	OK	0.47947
ENSG000001	-	-	ENSG0000013570	'CHST5	-	16:75528531-75623300	-	-	IGV_LINK	4.4322	0.152308	0	3.02769	OK	2.80693	0.051504	0	0.83988	OK	NOTEST	-1.56424
ENSG000001	-	-	ENSG0000013570	'KIAA0513	-	16:85027750-85094230	-	-	IGV_LINK	304.492	2.93028	0.437202	5.42335	OK	268.118	2.50159	0.230018	4.77317	OK	OK	-0.22819
ENSG000001	-	-	ENSG0000013572	'DYNC1LI2	-	16:66720892-66754740	-	-	IGV_LINK	2368.06	48.4039	0	99.8828	OK	2341.91	44.2876	0	90.2711	OK	OK	-0.12822
ENSG000001	-	-	ENSG0000013572	'FBXL8	-	16:67154179-67169945	-	-	IGV_LINK	253.562	14.6409	0	65.1876	OK	208.839	11.5609	0	45.2367	OK	OK	-0.34075
ENSG000001	-	-	ENSG0000013572	'FHOD1	-	16:67229386-67272190	-	-	IGV_LINK	749.594	20.7373	0.928091	40.5466	OK	735.263	24.0767	1.34295	46.8105	OK	OK	0.215409
ENSG000001	-	-	ENSG0000013573	'CCDC102A	-	16:57512177-57536599	-	-	IGV_LINK	192.974	5.79617	0.880646	10.7117	OK	189.745	6.51439	0.809638	12.2191	OK	OK	0.16853
ENSG000001	-	-	ENSG0000013574	'SLC9A5	-	16:67229386-67272190	-	-	IGV_LINK	71.7593	1.09176	0	3.59938	OK	56.7174	1.09174	0	5.03083	OK	OK	-2.18E-05
ENSG000001	-	-	ENSG0000013574	'AGT	-	1:230702522-230802003	-	-	IGV_LINK	897.961	23.8874	10.0825	37.6924	OK	2347.07	62.4703	27.2334	97.7072	OK	OK	1.38692
ENSG000001	-	-	ENSG0000013574	'ZNF670-ZNF695	-	1:246945546-247078811	-	-	IGV_LINK	4.13E-25	2.68E-26	0	0.136872	OK	2.11018	0.046141	0	0.268958	OK	NOTEST	80.5102
ENSG000001	-	-	ENSG0000013574	'PCNX2	-	1:232950604-233295727	-	-	IGV_LINK	952.266	12.3483	0	29.5351	OK	578.517	8.062	0	22.9517	OK	OK	-0.6151
ENSG000001	-	-	ENSG0000013575	'KCNK1	-	1:233614003-233672512	-	-	IGV_LINK	276.37	9.48319	1.98357	16.9828	OK	475.395	18.3869	3.93984	32.834	OK	OK	0.955235
ENSG000001	-	-	ENSG0000013576	'URB2	-	1:229626233-229660199	-	-	IGV_LINK	86.3051	1.01404	0	2.34923	OK	116.528	1.53797	0	3.68583	OK	OK	0.60091
ENSG000001	-	-	ENSG0000013576	'EGLN1	-	1:231363750-231425044	-	-	IGV_LINK	1205.36	16.947	0	33.9322	OK	1933.55	27.8904	0.215498	55.5652	OK	OK	0.718736
ENSG000001	-	-	ENSG0000013577	'CAPN9	-	1:230702522-230802003	-	-	IGV_LINK	0	0	0	0	OK	0	0	0	0	OK	NOTEST	0
ENSG000001	-	-	ENSG0000013577	'COG2	-	1:230642488-230693982	-	-	IGV_LINK	485.83	10.5136	2.18132	18.8458	OK	589.861	12.7194	3.56331	21.8755	OK	OK	0.274776
ENSG000001	-	-	ENSG0000013577	'ABCB10	-	1:229516581-229558695	-	-	IGV_LINK	258.915	4.55118	0.228187	8.87417	OK	263.994	5.36982	0	10.8777	OK	OK	0.238635
ENSG000001	-	-	ENSG0000013577	'NTPCR	-	1:232950604-233295727	-	-	IGV_LINK	716.623	37.8399	0	80.159	OK	1053.91	65.1945	11.2831	119.106	OK	OK	0.784842
ENSG000001	-	-	ENSG0000013580	'TAF5L	-	1:229593110-229626047	-	-	IGV_LINK	322.917	6.38133	1.38341	11.3792	OK	389.803	7.77544	1.9599	13.591	OK	OK	0.285067
ENSG000001	-	-	ENSG0000013582	'GLUL	-	1:182381703-182392206	-	-	IGV_LINK	6918.96	222.528	20.547	424.508	OK	10209.1	354.618	15.6445	693.591	OK	OK	0.672281
ENSG000001	-	-	ENSG0000013582	'STX6	-	1:180913153-181023121	-	-	IGV_LINK	930.571	11.4083	2.65588	20.1607	OK	798.049	10.2499	0.480982	20.0187	OK	OK	-0.15448
ENSG000001	-	-	ENSG0000013582	'RGS8	-	1:182641815-182684576	-	-	IGV_LINK	1.93944	0.086811	0	0.520212	OK	1.03122	0.010698	0	0.392743	OK	NOTEST	-3.02054
ENSG000001	-	-	ENSG0000013582	'RNASEL	-	1:182573633-182589256	-	-	IGV_LINK	556.62	7.79854	3.06556	12.5315	OK	538.299	7.50008	2.89946	12.1007	OK	OK	-0.0563
ENSG000001	-	-	ENSG0000013582	'DHX9	-	1:182839368-182887751	-	-	IGV_LINK	3010.98	57.3411	0	124.369	OK	2636.84	49.046	0.313463	97.7786	OK	OK	-0.22543
ENSG000001	-	-	ENSG0000013583	'KIAA1614	-	1:180913153-181023121	-	-	IGV_LINK	297.704	3.43583	0	11.607	OK	244.4	2.48473	0	9.01181	OK	OK	-0.46757
ENSG000001	-	-	ENSG0000013583	'CEP350	-	1:179954737-180114880	-	-	IGV_LINK	905.719	7.33316	0	15.6982	OK	664.109	5.58424	0.56863	10.5998	OK	OK	-0.39307
ENSG000001	-	-	ENSG0000013583	'NPL	-	1:182789292-182830384	-	-	IGV_LINK	1086.09	44.8062	1.68513	87.9273	OK	3520.6	131.301	14.3086	248.294	OK	OK	1.55111
ENSG000001	-	-	ENSG0000013584	'FAM129A	-	1:184790723-184974550	-	-	IGV_LINK	1265.49	11.1842	3.55786	18.8106	OK	899.228	8.07864	2.14979	14.0075	OK	OK	-0.46928
ENSG000001	-	-	ENSG0000013584	'PIGC	-	1:171841497-172468831	-	-	IGV_LINK	393.707	16.5715	4.86822	28.2748	OK	435.177	19.9073	5.62912	34.1855	OK	OK	0.264593
ENSG000001	-	-	ENSG0000013586	'LAMC1	-	1:183023459-183145592	-	-	IGV_LINK	3826.52	34.348	0	69.429	OK	4120.77	39.4465	0	82.044	OK	OK	0.199674
ENSG000001	-	-	ENSG0000013587	'RC3H1	-	1:173931213-174022297	-	-	IGV_LINK	483.891	2.61883	0.619701	4.61796	OK	489.832	2.58386	0.647673	4.52005	OK	OK	-0.01939
ENSG000001	-	-	ENSG0000013589	'GPR55	-	2:230907317-230961066	-	-	IGV_LINK	60.1227	0.926925	0	1.86867	OK	44.3427	1.16146	0	2.7692	OK	OK	0.32541
ENSG000001	-	-	ENSG0000013589	'SP110	-	2:230121369-230313215	-	-	IGV_LINK	818.444	30.9628	0	62.6925	OK	1126.1	42.6371	3.60761	81.6665	OK	OK	0.461571
ENSG000001	-	-	ENSG0000013590	'MRPL44	-	2:223957403-223967714	-	-	IGV_LINK	475.163	16.9968	6.42277	27.5708	OK	608.423	21.7794	8.40871	35.1502	OK	OK	0.357705
ENSG000001	-	-	ENSG0000013590	'CHRND	-	2:232525992-232536667	-	-	IGV_LINK	3.87888	0.098229	0	0.399007	OK	6.18735	0.142548	0	0.538833	OK	NOTEST	0.537224
ENSG000001	-	-	ENSG0000013590	'PAX3	-	2:222199887-222305217	-	-	IGV_LINK	0	0	0	0	OK	0	0	0	0	OK	NOTEST	0
ENSG000001	-	-	ENSG0000013590	'DOCK10	-	2:224765089-225042445	-	-	IGV_LINK	1762.95	25.3508	0	57.7782	OK	989.976	17.7853	0	41.9461	OK	OK	-0.51135
ENSG000001	-	-	ENSG0000013591	'TTLL4	-	2:218710844-218755416	-	-	IGV_LINK	415.04	12.325	0	25.4882	OK	458.895	16.2306	0.077241	32.3839	OK	OK	0.39713
ENSG000001	-	-	ENSG0000013591	'USP37	-	2:218419091-218568361	-	-	IGV_LINK	360.736	3.98925	0	9.60895	OK	291.837	2.36352	0	5.05686	OK	OK	-0.75518
ENSG000001	-	-	ENSG0000013591	'HTR2B	-	2:231056863-231172827	-	-	IGV_LINK	3.87888	0.105425	0	1.07325	OK	20.6245	0.560869	0	2.98223	OK	NOTEST	2.41145
ENSG000001	-	-	ENSG0000013591	'ITM2C	-	2:230864638-230879248	-	-	IGV_LINK	2229.39	70.7304	29.7366	111.724	OK	1039.47	32.6238	13.0864	52.1612	OK	OK	-1.11641
ENSG000001	-	-	ENSG0000013591	'SLC19A3	-	2:227685209-227718012	-	-	IGV_LINK	99.8812	2.6333	0	6.15405	OK	149.528	3.12662	0	6.40893	OK	OK	0.24773
ENSG000001	-	-	ENSG0000013591	'SERPINE2	-	2:223975111-224039319	-	-	IGV_LINK	636.137	18.1325	6.28357	29.9814	OK	877.572	28.647	8.93369	48.3602	OK	OK	0.659807
ENSG000001	-	-	ENSG0000013592	'DNAJB2	-	2:219279266-219286900	-	-	IGV_LINK	1543.8	46.2223	16.5496	75.8949	OK	1734.52	53.1222	18.4916	87.7527	OK	OK	0.200726
ENSG000001	-	-	ENSG0000013592	'WNT10A	-	2:218880362-218899581	-	-	IGV_LINK	57.2135	2.12174	0	5.41454	OK	19.5933	0.855727	0	2.50209	OK	OK	-1.31003
ENSG000001	-	-	ENSG0000013592	'TMBIM1	-	2:218270391-218368099	-	-	IGV_LINK	3510.39	96.6546	22.331	170.978	OK	4441.49	129.098	20.5384	237.657	OK	OK	0.417552
ENSG000001	-	-	ENSG0000013592	'CYP27A1	-	2:218781748-218815293	-	-	IGV_LINK	787.413	22.1979	8.17079	36.225	OK	854.886	24.2822	9.60541	38.959	OK	OK	0.129476
ENSG000001	-	-	ENSG0000013593	'EIF4E2	-	2:232547967-232682781	-	-	IGV_LINK	1019.18	36.8184	3.201	70.4359	OK	1153.94	41.296	7.58508	75.0069	OK	OK	0.165572
ENSG000001	-	-	ENSG0000013593	'ARMC9	-	2:231198545-231374837	-	-	IGV_LINK	253.097	4.3495	0.008482	8.69052	OK	294.93	7.09366	0	14.3565	OK	OK	0.705679
ENSG000001	-	-	ENSG0000013593	'CAB39	-	2:230712844-230821075	-	-	IGV_LINK	952.266	19.4539	3.4401	35.4678	OK	1173.53	23.5147	5.00044	42.029	OK	OK	0.273501
ENSG000001	-	-	ENSG0000013594	'COX5B	-	2:97646039-97648383	-	-	IGV_LINK	1988.9	207.876	92.1503	323.601	OK	1951.08	204.289	90.5894	317.989	OK	OK	-0.02511
ENSG000001	-	-	ENSG0000013594	'REV1	-	2:98997260-99490035	-	-	IGV_LINK	852.384	22.9867	0	70.0437	OK	630.078	16.4496	0	53.7054	OK	OK	-0.48274
ENSG000001	-	-	ENSG0000013595	'TSGA10	-	2:98997260-99490035	-	-	IGV_LINK	72.729	4.93471	0	45.6705	OK	64.9672	3.44298	0	39.7894	OK	OK	-0.51931
ENSG000001	-	-	ENSG0000013595	'MFSD9	-	2:102715839-102736888	-	-	IGV_LINK	214.308	5.28434	0	11.189	OK	282.556	8.46286	0	19.4256	OK	OK	0.679423
ENSG000001	-	-	ENSG0000013595	'TMEM127	-	2:96248515-96265994	-	-	IGV_LINK	2209.02	34.8724	10.5617	59.1832	OK	2781.21	45.4293	11.7677	79.091	OK	OK	0.381537
ENSG000001	-	-	ENSG0000013596	'EDAR	-	2:108894470-108989372	-	-	IGV_LINK	16.4852	0.229868	0	0.493696	OK	3.09367	0.043145	0	0.12042	OK	NOTEST	-2.41354
ENSG000001	-	-	ENSG0000013596	'TGFBRAP1	-	2:105264390-105330529	-	-	IGV_LINK	564.377	6.07695	2.25575	9.89815	OK	703.295	7.96467	2.93538	12.994	OK	OK	0.390268
ENSG000001	-	-	ENSG0000013596	'GCC2	-	2:108448560-108534196	-	-	IGV_LINK	1033.72	14.185	0.056289	28.3138	OK	1113.72	12.4635	1.43483	23.4921	OK	OK	-0.18666
ENSG000001	-	-	ENSG0000013597	'MRPS9	-	2:104936240-105102944	-	-	IGV_LINK	356.857	15.7486	5.17636	26.3208	OK	290.805	12.4288	3.89711	20.9606	OK	OK	-0.34153
ENSG000001	-	-	ENSG0000013597	'GPR45	-	2:105241742-105243049	-	-	IGV_LINK	2.90916	0.144514	0	0.387872	OK	0	0	0	0	OK	NOTEST	'-inf
ENSG000001	-	-	ENSG0000013597	'C2orf49	-	2:105334026-105349211	-	-	IGV_LINK	387.888	9.0415	0	18.412	OK	355.773	9.69778	0	22.1284	OK	OK	0.101092
ENSG000001	-	-	ENSG0000013597	'ANKRD36	-	2:97113495-97264521	-	-	IGV_LINK	228.854	3.01615	0.414242	5.61807	OK	121.685	1.89897	0	4.06054	OK	OK	-0.6675
ENSG000001	-	-	ENSG0000013599	'EPC2	-	2:148644439-148787568	-	-	IGV_LINK	416.98	7.37288	1.9679	12.7779	OK	422.802	7.31171	2.18384	12.4396	OK	OK	-0.01202
ENSG000001	-	-	ENSG0000013600	'ARHGEF4	-	2:130830977-131047263	-	-	IGV_LINK	3.87888	0.106614	0	0.55998	OK	6.18735	0.140682	0	0.681827	OK	NOTEST	0.400045
ENSG000001	-	-	ENSG0000013600	'ISCU	-	12:108562581-108569384	-	-	IGV_LINK	2041.26	117.751	46.0689	189.433	OK	2171.76	125.98	50.2528	201.707	OK	OK	0.097451
ENSG000001	-	-	ENSG0000013601	'ALDH1L2	-	12:104986309-105084577	-	-	IGV_LINK	37.8191	0.322302	0	1.08648	OK	35.0616	0.287224	0	0.95263	OK	NOTEST	-0.16624
ENSG000001	-	-	ENSG0000013601	'STAB2	-	12:103587272-103768858	-	-	IGV_LINK	1.93944	0.01353	0	0.633433	OK	8.2498	0.228982	0	1.47628	OK	NOTEST	4.08105
ENSG000001	-	-	ENSG0000013601	'USP44	-	12:95516559-95551490	-	-	IGV_LINK	0	0	0	0	OK	0	0	0	0	OK	NOTEST	0
ENSG000001	-	-	ENSG0000013602	'SCYL2	-	12:100267139-100341724	-	-	IGV_LINK	490.679	7.56922	0.919026	14.2194	OK	502.207	6.68064	2.05084	11.3104	OK	OK	-0.18016
ENSG000001	-	-	ENSG0000013602	'CKAP4	-	12:106237876-106347015	-	-	IGV_LINK	604.136	11.6578	3.36851	19.9471	OK	791.981	15.2886	5.2225	25.3547	OK	OK	0.391161
ENSG000001	-	-	ENSG0000013604	'PLXNC1	-	12:94148722-94459988	-	-	IGV_LINK	1295.55	13.6733	1.05185	26.2948	OK	1378.75	14.6264	3.17894	26.0739	OK	OK	0.097212
ENSG000001	-	-	ENSG0000013604	'APPL2	-	12:105173295-105396097	-	-	IGV_LINK	732.139	24.6168	0	52.6812	OK	809.512	25.2168	0	53.1153	OK	OK	0.034746
ENSG000001	-	-	ENSG0000013604	'PWP1	-	12:107685731-107713167	-	-	IGV_LINK	884.385	25.8985	10.1728	41.6242	OK	961.102	28.3452	11.0998	45.5907	OK	OK	0.130238
ENSG000001	-	-	ENSG0000013604	'DRAM1	-	12:101877350-102012130	-	-	IGV_LINK	1118.09	20.3318	5.88277	34.7808	OK	1359.15	27.9302	3.54439	52.316	OK	OK	0.458089
ENSG000001	-	-	ENSG0000013605	'WASHC4	-	12:105107323-105169134	-	-	IGV_LINK	1123.91	28.5475	0	69.7167	OK	1106.5	23.5648	0	57.8257	OK	OK	-0.27673
ENSG000001	-	-	ENSG0000013605	'SLC41A2	-	12:104802552-104958744	-	-	IGV_LINK	435.405	14.3252	0	30.7323	OK	722.889	19.3575	0	40.0257	OK	OK	0.434334
ENSG000001	-	-	ENSG0000013605	'VILL	-	3:37988058-38007188	-	-	IGV_LINK	463.526	14.6285	1.4171	27.84	OK	332.054	9.08399	1.99281	16.1752	OK	OK	-0.68739
ENSG000001	-	-	ENSG0000013606	'FLNB	-	3:58008399-58172251	-	-	IGV_LINK	3499.72	32.7778	5.13777	60.4178	OK	1194.16	10.4547	1.37487	19.5346	OK	OK	-1.64856
ENSG000001	-	-	ENSG0000013609	'NEK3	-	13:52033610-52159861	-	-	IGV_LINK	315.159	11.2377	0	22.6204	OK	306.274	12.7437	1.00935	24.4781	OK	OK	0.181444
ENSG000001	-	-	ENSG0000013609	'PCDH8	-	13:52842888-52848641	-	-	IGV_LINK	0	0	0	0	OK	0	0	0	0	OK	NOTEST	0
ENSG000001	-	-	ENSG0000013610	'VPS36	-	13:52412601-52450628	-	-	IGV_LINK	927.053	16.7493	0	33.7136	OK	907.478	15.1257	1.3882	28.8632	OK	OK	-0.1471
ENSG000001	-	-	ENSG0000013610	'RNASEH2B	-	13:50862171-50973745	-	-	IGV_LINK	352.009	15.0062	2.06829	27.9442	OK	281.524	11.7252	0	23.7491	OK	OK	-0.35595
ENSG000001	-	-	ENSG0000013610	'CKAP2	-	13:52455428-52476628	-	-	IGV_LINK	344.251	7.93824	0.818301	15.0582	OK	452.708	9.16887	1.95402	16.3837	OK	OK	0.207924
ENSG000001	-	-	ENSG0000013611	'CNMD	-	13:52703263-52739812	-	-	IGV_LINK	0.969721	0.044144	0	0.263517	OK	0	0	0	0	OK	NOTEST	'-inf
ENSG000001	-	-	ENSG0000013611	'TBC1D4	-	13:75284664-75482114	-	-	IGV_LINK	1022.09	11.4178	0	23.8016	OK	630.078	7.55452	0	15.8463	OK	OK	-0.59588
ENSG000001	-	-	ENSG0000013611	'THSD1	-	13:52377166-52406494	-	-	IGV_LINK	111.518	2.18617	0.254883	4.11745	OK	151.59	3.01094	0.577881	5.444	OK	OK	0.46181
ENSG000001	-	-	ENSG0000013612	'BORA	-	13:72727748-73016461	-	-	IGV_LINK	69.8199	2.74352	0	9.46919	OK	76.3106	2.98733	0	10.2295	OK	OK	0.122829
ENSG000001	-	-	ENSG0000013614	'LRCH1	-	13:46553167-46753040	-	-	IGV_LINK	1030.81	13.075	4.67043	21.4796	OK	1028.13	12.8447	5.35777	20.3316	OK	OK	-0.02564
ENSG000001	-	-	ENSG0000013614	'SUCLA2	-	13:47936490-48001354	-	-	IGV_LINK	602.196	25.2942	6.74025	43.8481	OK	549.643	20.6677	5.72054	35.6149	OK	OK	-0.29142
ENSG000001	-	-	ENSG0000013614	'RCBTB1	-	13:49531945-49585583	-	-	IGV_LINK	572.135	9.19607	0.867903	17.5242	OK	430.021	6.48084	2.03266	10.929	OK	OK	-0.50484
ENSG000001	-	-	ENSG0000013614	'MED4	-	13:48053322-48095131	-	-	IGV_LINK	532.377	31.1983	8.99396	53.4027	OK	446.52	26.2763	6.55558	45.997	OK	OK	-0.24771
ENSG000001	-	-	ENSG0000013614	'PHF11	-	13:49495609-49528987	-	-	IGV_LINK	762.2	45.1558	16.3957	73.9158	OK	586.767	35.393	12.5427	58.2433	OK	OK	-0.35145
ENSG000001	-	-	ENSG0000013614	'RPL13AP25	-	13:54440703-54441315	-	-	IGV_LINK	0	0	0	0	OK	1.03122	0.132585	0	0.496238	OK	NOTEST	inf
ENSG000001	-	-	ENSG0000013615	'COG3	-	13:45464897-45615739	-	-	IGV_LINK	628.379	10.0831	0	25.1981	OK	601.204	10.7983	0	27.3434	OK	OK	0.098863
ENSG000001	-	-	ENSG0000013615	'LMO7	-	13:75549479-75859870	-	-	IGV_LINK	1336.62	34.8957	4.70617	65.0853	OK	887.959	22.0132	2.97969	41.0468	OK	OK	-0.66468
ENSG000001	-	-	ENSG0000013615	'SCEL	-	13:77535673-77645263	-	-	IGV_LINK	0	0	0	0	OK	0	0	0	0	OK	NOTEST	0
ENSG000001	-	-	ENSG0000013615	'ITM2B	-	13:48233157-48270357	-	-	IGV_LINK	13385.1	1012.63	133.469	1891.79	OK	13383.2	1037.43	145.129	1929.74	OK	OK	0.034916
ENSG000001	-	-	ENSG0000013615	'SPRY2	-	13:80335975-80340951	-	-	IGV_LINK	210.429	5.92555	1.6088	10.2423	OK	155.715	4.40665	0.948072	7.86523	OK	OK	-0.42727
ENSG000001	-	-	ENSG0000013615	'NUDT15	-	13:48037566-48047222	-	-	IGV_LINK	179.398	5.25069	1.25773	9.24364	OK	207.276	6.07025	1.64586	10.4946	OK	OK	0.209251
ENSG000001	-	-	ENSG0000013616	'EDNRB	-	13:77895480-78659179	-	-	IGV_LINK	1059.9	19.588	3.427	35.749	OK	588.829	11.1351	1.47443	20.7958	OK	OK	-0.81486
ENSG000001	-	-	ENSG0000013616	'RCBTB2	-	13:48488958-48533256	-	-	IGV_LINK	1086.09	21.1967	7.54996	34.8435	OK	807.449	16.0079	5.95024	26.0656	OK	OK	-0.40506
ENSG000001	-	-	ENSG0000013616	'LCP1	-	13:46125919-46211871	-	-	IGV_LINK	7550.24	122.628	39.4335	205.823	OK	4942.66	77.1454	30.4636	123.827	OK	OK	-0.66864
ENSG000001	-	-	ENSG0000013616	'SETDB2	-	13:49444373-49495003	-	-	IGV_LINK	481.951	6.03448	1.3797	10.6893	OK	386.709	4.84885	1.01676	8.68094	OK	OK	-0.31559
ENSG000001	-	-	ENSG0000013619	'SCRN1	-	7:29920102-30026684	-	-	IGV_LINK	1249.97	14.6897	4.23198	25.1475	OK	853.854	9.85733	2.32574	17.3889	OK	OK	-0.57554
ENSG000001	-	-	ENSG0000013619	'C7orf25	-	7:42908725-42948958	-	-	IGV_LINK	16.3693	0.638791	0	6.21533	OK	22.9565	0.834767	0	6.72995	OK	OK	0.386029
ENSG000001	-	-	ENSG0000013620	'TNS3	-	7:47275153-47582558	-	-	IGV_LINK	5112.37	43.0781	10.7417	75.4145	OK	6491.56	57.2511	10.0168	104.485	OK	OK	0.41035
ENSG000001	-	-	ENSG0000013620	'SPDYE1	-	7:43926437-44019175	-	-	IGV_LINK	14.5458	0.331009	0	1.71447	OK	16.4996	0.375644	0	1.8817	OK	NOTEST	0.182498
ENSG000001	-	-	ENSG0000013621	'CHST12	-	7:2403559-2448483	-	-	IGV_LINK	417.95	8.61922	1.88638	15.3521	OK	501.175	11.8178	2.97657	20.6589	OK	OK	0.455328
ENSG000001	-	-	ENSG0000013623	'IGF2BP3	-	7:23298738-23470467	-	-	IGV_LINK	11.6366	0.591875	0	3.4652	OK	25.7806	1.08534	0	5.34401	OK	OK	0.874789
ENSG000001	-	-	ENSG0000013623	'GPNMB	-	7:23235966-23275108	-	-	IGV_LINK	7681.16	172.73	49.709	295.751	OK	9387.24	213.344	43.8518	382.836	OK	OK	0.304664
ENSG000001	-	-	ENSG0000013623	'RAPGEF5	-	7:22118237-22357144	-	-	IGV_LINK	916.386	12.7557	0	28.0282	OK	764.138	10.0095	0	21.5495	OK	OK	-0.34976
ENSG000001	-	-	ENSG0000013623	'RAC1	-	7:6374522-6403977	-	-	IGV_LINK	3471.6	146.889	7.50748	286.271	OK	3704.16	151.865	19.2251	284.504	OK	OK	0.048057
ENSG000001	-	-	ENSG0000013624	'KDELR2	-	7:6409125-6484242	-	-	IGV_LINK	1614.58	35.8929	8.12586	63.66	OK	2417.19	54.7922	12.0627	97.5217	OK	OK	0.61027
ENSG000001	-	-	ENSG0000013624	'NUPL2	-	7:23181826-23201011	-	-	IGV_LINK	352.978	22.4591	2.60239	42.3158	OK	309.367	19.7249	0.340285	39.1094	OK	OK	-0.18728
ENSG000001	-	-	ENSG0000013624	'IL6	-	7:22725394-22732002	-	-	IGV_LINK	7.75776	0.89267	0	2.2489	OK	196.964	17.9378	3.47857	32.397	OK	OK	4.32873
ENSG000001	-	-	ENSG0000013624	'ZDHHC4	-	7:6577433-6589374	-	-	IGV_LINK	683.653	38.7911	13.4946	64.0877	OK	719.795	36.4436	13.308	59.5791	OK	OK	-0.09006
ENSG000001	-	-	ENSG0000013625	'AOAH	-	7:36512948-36724549	-	-	IGV_LINK	1494.34	47.6898	13.3378	82.0417	OK	1456.09	42.7546	13.6642	71.8451	OK	OK	-0.1576
ENSG000001	-	-	ENSG0000013626	'BZW2	-	7:16646130-16719898	-	-	IGV_LINK	304.492	10.8978	3.26605	18.5295	OK	319.68	10.9738	3.53114	18.4164	OK	OK	0.010025
ENSG000001	-	-	ENSG0000013626	'DGKB	-	7:14145048-14974777	-	-	IGV_LINK	10.6669	0.145556	0	0.714489	OK	11.3435	0.150311	0	0.728657	OK	NOTEST	0.046379
ENSG000001	-	-	ENSG0000013627	'TBRG4	-	7:45100099-45112047	-	-	IGV_LINK	883.415	37.3778	10.9122	63.8435	OK	1140.53	44.4554	13.3271	75.5837	OK	OK	0.250176
ENSG000001	-	-	ENSG0000013627	'DDX56	-	7:44565416-44575051	-	-	IGV_LINK	1523.43	46.0773	14.4075	77.747	OK	1567.46	47.296	15.4955	79.0965	OK	OK	0.037664
ENSG000001	-	-	ENSG0000013627	'HUS1	-	7:47695729-47979581	-	-	IGV_LINK	296.734	14.1642	1.29702	27.0314	OK	252.65	10.7362	0.636995	20.8354	OK	OK	-0.39977
ENSG000001	-	-	ENSG0000013627	'NACAD	-	7:45080437-45088914	-	-	IGV_LINK	26.1825	0.661116	0	2.71541	OK	14.4371	0.176629	0	0.591685	OK	OK	-1.90418

ENSG000001	-	-	ENSG0000013627	'C7orf69	-	7:47695729-47979581	-	-	IGV_LINK	0	0	0	0	OK	0	0	0	0	OK	NOTEST	0
ENSG000001	-	-	ENSG0000013627	'DBNL	-	7:44044639-44069456	-	-	IGV_LINK	2679.98	60.4628	16.9561	103.969	OK	2988.87	70.9508	21.1314	120.77	OK	OK	0.230773
ENSG000001	-	-	ENSG0000013628	'CCM2	-	7:44999474-45076469	-	-	IGV_LINK	1203.42	35.6584	12.6168	58.7001	OK	1476.71	46.0923	16.8141	75.3706	OK	OK	0.370284
ENSG000001	-	-	ENSG0000013628	'MYO1G	-	7:44962661-44979098	-	-	IGV_LINK	947.417	16.3054	6.53896	26.0718	OK	967.289	16.7239	6.73596	26.7119	OK	OK	0.036566
ENSG000001	-	-	ENSG0000013629	'TTYH3	-	7:2631950-2664802	-	-	IGV_LINK	2213.87	33.8513	5.79057	61.912	OK	5516.02	79.3328	12.0758	146.59	OK	OK	1.22871
ENSG000001	-	-	ENSG0000013629	'MMD2	-	7:4905988-4959213	-	-	IGV_LINK	0	0	0	0	OK	0	0	0	0	OK	NOTEST	0
ENSG000001	-	-	ENSG0000013630	'CIDEB	-	14:24299861-24318036	-	-	IGV_LINK	750.564	29.8135	0	64.8737	OK	1238.5	49.2586	0	104.4	OK	OK	0.724408
ENSG000001	-	-	ENSG0000013631	'AL355922.1	-	14:20897984-20936255	-	-	IGV_LINK	0	0	0	0	OK	1.03122	0.3529	0	1.32083	OK	NOTEST	inf
ENSG000001	-	-	ENSG0000013631	'TTC5	-	14:20256557-20306811	-	-	IGV_LINK	293.825	7.66661	0.473799	14.8594	OK	249.201	6.53336	0	13.5571	OK	OK	-0.23077
ENSG000001	-	-	ENSG0000013632	'NKX2-8	-	14:36320752-37173811	-	-	IGV_LINK	0	0	0	0	OK	3.09367	0.160344	0	0.790877	OK	NOTEST	inf
ENSG000001	-	-	ENSG0000013635	'NKX2-1	-	14:36320752-37173811	-	-	IGV_LINK	0	0	0	0	OK	0	0	0	0	OK	NOTEST	0
ENSG000001	-	-	ENSG0000013636	'ZFHX2	-	14:23511759-23578800	-	-	IGV_LINK	45.5769	0.342399	0	2.42062	OK	46.4051	0.381095	0	4.72633	OK	NOTEST	0.154474
ENSG000001	-	-	ENSG0000013637	'MTHFS	-	15:79833584-79926993	-	-	IGV_LINK	265.583	26.0197	0	53.6075	OK	533.037	49.2679	6.31271	92.2231	OK	OK	0.921044
ENSG000001	-	-	ENSG0000013637	'ADAMTS7	-	15:78759202-78898133	-	-	IGV_LINK	132.852	1.79937	0	9.79498	OK	171.183	2.13268	0	10.2617	OK	OK	0.245181
ENSG000001	-	-	ENSG0000013637	'ABHD17C	-	15:80679683-80755621	-	-	IGV_LINK	92.1234	2.71384	0	5.53355	OK	59.811	1.55483	0	3.17323	OK	OK	-0.80358
ENSG000001	-	-	ENSG0000013638	'IREB2	-	15:78437430-78501456	-	-	IGV_LINK	1104.51	14.0419	1.05042	27.0333	OK	993.07	10.6861	1.15695	20.2153	OK	OK	-0.39399
ENSG000001	-	-	ENSG0000013638	'ALPK3	-	15:84816679-84873482	-	-	IGV_LINK	4370.53	22.9238	9.56652	36.281	OK	7371.2	40.5029	0.689403	80.3163	OK	OK	0.821179
ENSG000001	-	-	ENSG0000013640	'TM6SF1	-	15:83107406-83439445	-	-	IGV_LINK	181.338	10.2869	0	23.5245	OK	143.34	7.65995	0	17.0908	OK	OK	-0.42541
ENSG000001	-	-	ENSG0000013642	'CIB2	-	15:78077807-78245688	-	-	IGV_LINK	43.6374	2.87433	0	10.6916	OK	27.8431	1.55276	0	6.83888	OK	OK	-0.88839
ENSG000001	-	-	ENSG0000013643	'CALCOCO2	-	17:48830987-48866522	-	-	IGV_LINK	2479.58	95.8984	20.1967	171.6	OK	2513.1	100.562	21.7155	179.409	OK	OK	0.068508
ENSG000001	-	-	ENSG0000013644	'RSAD1	-	17:50478799-50485975	-	-	IGV_LINK	961.963	31.7644	7.49859	56.0301	OK	892.01	30.4774	7.18359	53.7711	OK	OK	-0.05967
ENSG000001	-	-	ENSG0000013644	'NMT1	-	17:45023339-45144181	-	-	IGV_LINK	2313.75	43.9397	0	94.7561	OK	2954.46	76.0243	0	309.302	OK	OK	0.790937
ENSG000001	-	-	ENSG0000013644	'MYCBPAP	-	17:50508383-50531501	-	-	IGV_LINK	32.0008	1.48959	0	3.99747	OK	32.9992	1.20436	0	3.20945	OK	OK	-0.30664
ENSG000001	-	-	ENSG0000013645	'SRSF1	-	17:57989037-58008187	-	-	IGV_LINK	2906.12	103.621	12.707	194.535	OK	2451.39	87.7007	9.35533	166.046	OK	OK	-0.24066
ENSG000001	-	-	ENSG0000013645	'VEZF1	-	17:57971546-57988760	-	-	IGV_LINK	939.659	18.8177	6.04121	31.5942	OK	749.701	15.4731	5.31529	25.6308	OK	OK	-0.28233
ENSG000001	-	-	ENSG0000013645	'CHAD	-	17:50426157-50474845	-	-	IGV_LINK	13.5761	0.473305	0	2.0461	OK	6.18735	0.222751	0	1.2662	OK	NOTEST	-1.08734
ENSG000001	-	-	ENSG0000013646	'TACO1	-	17:63600871-63608365	-	-	IGV_LINK	231.763	9.54918	2.7077	16.3907	OK	399.084	17.0182	5.66743	28.3689	OK	OK	0.833625
ENSG000001	-	-	ENSG0000013647	'TEX2	-	17:64147226-64263301	-	-	IGV_LINK	669.107	8.0201	2.94834	13.0919	OK	1034.32	13.2663	3.468	23.0646	OK	OK	0.726074
ENSG000001	-	-	ENSG0000013648	'DCAF7	-	17:63550460-63594266	-	-	IGV_LINK	1794.95	18.7101	5.67651	31.7436	OK	1686.05	19.1337	3.9035	34.3639	OK	OK	0.032301
ENSG000001	-	-	ENSG0000013648	'GH2	-	17:63880214-63881935	-	-	IGV_LINK	0	0	0	0	OK	0	0	0	0	OK	NOTEST	0
ENSG000001	-	-	ENSG0000013648	'CSH1	-	17:63894908-63896661	-	-	IGV_LINK	0	0	0	0	OK	0	0	0	0	OK	NOTEST	0
ENSG000001	-	-	ENSG0000013649	'LIMD2	-	17:63622414-63819317	-	-	IGV_LINK	1369.25	61.2846	0	165.178	OK	727.014	28.6979	0	95.1133	OK	OK	-1.09458
ENSG000001	-	-	ENSG0000013649	'BRIP1	-	17:61681265-61863521	-	-	IGV_LINK	66.9107	0.70869	0	1.6283	OK	58.7798	0.594504	0	1.32385	OK	OK	-0.25347
ENSG000001	-	-	ENSG0000013650	'KAT7	-	17:49700942-49836454	-	-	IGV_LINK	951.296	17.0805	0	36.5658	OK	843.542	14.0612	0.192805	27.9295	OK	OK	-0.28063
ENSG000001	-	-	ENSG0000013651	'RTP4	-	3:187368331-187372076	-	-	IGV_LINK	823.293	33.5851	14.2011	52.9691	OK	266.056	10.8624	3.35487	18.37	OK	OK	-1.62847
ENSG000001	-	-	ENSG0000013651	'ACTL6A	-	3:179562879-179604654	-	-	IGV_LINK	556.62	23.3642	3.34465	43.3838	OK	485.707	19.6501	1.59123	37.7089	OK	OK	-0.24977
ENSG000001	-	-	ENSG0000013652	'NDUFB5	-	3:179604689-179627647	-	-	IGV_LINK	1110.33	75.2899	0	182.031	OK	1033.29	62.2084	3.52546	120.891	OK	OK	-0.27535
ENSG000001	-	-	ENSG0000013652	'MRPL47	-	3:179562879-179604654	-	-	IGV_LINK	263.764	18.3999	0	38.8017	OK	292.868	19.2399	0	39.4649	OK	OK	0.064408
ENSG000001	-	-	ENSG0000013652	'TRA2B	-	3:185915905-185938136	-	-	IGV_LINK	1665.01	55.5408	18.8098	92.2718	OK	1402.47	52.0503	16.1361	87.9645	OK	OK	-0.09364
ENSG000001	-	-	ENSG0000013653	'SCN2A	-	2:164653623-165392310	-	-	IGV_LINK	11.6366	0.10783	0	0.775211	OK	10.3122	0.087112	0	0.534117	OK	NOTEST	-0.30782
ENSG000001	-	-	ENSG0000013653	'TBR1	-	2:161416093-161985282	-	-	IGV_LINK	0	0	0	0	OK	0	0	0	0	OK	NOTEST	0
ENSG000001	-	-	ENSG0000013653	'MARCH7	-	2:159712456-159904749	-	-	IGV_LINK	851.415	24.2853	0	49.4999	OK	849.729	27.4968	0	58.6857	OK	OK	0.179183
ENSG000001	-	-	ENSG0000013654	'ERMN	-	2:157318624-157327713	-	-	IGV_LINK	46.5466	1.25074	0	3.33942	OK	26.8118	0.417479	0	1.13896	OK	OK	-1.583
ENSG000001	-	-	ENSG0000013654	'GALNT5	-	2:157257597-157314211	-	-	IGV_LINK	0	0	0	0	OK	0	0	0	0	OK	NOTEST	0
ENSG000001	-	-	ENSG0000013654	'SCN7A	-	2:166403572-166494247	-	-	IGV_LINK	0.969721	0.010581	0	0.179058	OK	4.1249	0.046534	0	0.218872	OK	NOTEST	2.13675
ENSG000001	-	-	ENSG0000013656	'TANK	-	2:161096230-161411717	-	-	IGV_LINK	832.99	29.4396	0.687244	58.1919	OK	956.977	31.9803	3.30569	60.6549	OK	OK	0.119428
ENSG000001	-	-	ENSG0000013657	'BLK	-	8:11494000-11564604	-	-	IGV_LINK	17.455	0.459678	0	1.09271	OK	41.249	1.21201	0	2.82947	OK	OK	1.39871
ENSG000001	-	-	ENSG0000013657	'GATA4	-	8:11676958-11760002	-	-	IGV_LINK	0	0	0	0	OK	0	0	0	0	OK	NOTEST	0
ENSG000001	-	-	ENSG0000013660	'SKIL	-	3:170357677-170396835	-	-	IGV_LINK	517.831	8.06246	0.468189	15.6567	OK	438.271	7.54483	0	15.496	OK	OK	-0.09573
ENSG000001	-	-	ENSG0000013662	'EPRS	-	1:219968600-220046658	-	-	IGV_LINK	1999.56	25.8446	3.88935	47.7999	OK	2086.17	29.5305	0.074969	58.9861	OK	OK	0.192344
ENSG000001	-	-	ENSG0000013663	'HLX	-	1:220832762-220885059	-	-	IGV_LINK	565.347	18.2108	2.4584	33.9633	OK	486.738	16.0646	0.268738	31.8605	OK	OK	-0.18091
ENSG000001	-	-	ENSG0000013663	'VPS45	-	1:150067292-150145327	-	-	IGV_LINK	637.106	18.6144	5.73101	31.4978	OK	754.857	22.565	6.92586	38.2042	OK	OK	0.27767
ENSG000001	-	-	ENSG0000013663	'IL10	-	1:206767601-206772494	-	-	IGV_LINK	17.1805	0.874129	0	2.37504	OK	49.4982	4.13318	0	10.0719	OK	OK	2.24134
ENSG000001	-	-	ENSG0000013663	'KCTD3	-	1:215567391-215621807	-	-	IGV_LINK	2647.34	52.8614	8.84108	96.8818	OK	3723.75	70.1087	11.6651	128.552	OK	OK	0.407378
ENSG000001	-	-	ENSG0000013664	'RPS6KC1	-	1:213051232-213274773	-	-	IGV_LINK	374.312	5.50414	1.27298	9.73531	OK	367.116	5.49076	1.05954	9.92198	OK	OK	-0.00351
ENSG000001	-	-	ENSG0000013668	'CBWD2	-	2:113437690-113496189	-	-	IGV_LINK	224.005	7.73578	1.32029	14.1513	OK	215.526	7.99035	1.3034	14.6773	OK	OK	0.046711
ENSG000001	-	-	ENSG0000013668	'IL36G	-	2:112973202-112985665	-	-	IGV_LINK	0	0	0	0	OK	1.03122	0.296448	0	1.12157	OK	NOTEST	inf
ENSG000001	-	-	ENSG0000013668	'IL1RN	-	2:113107213-113134016	-	-	IGV_LINK	44.6071	2.41393	0	5.56686	OK	131.997	7.11622	0.325746	13.9067	OK	OK	1.55972
ENSG000001	-	-	ENSG0000013669	'IL36A	-	2:113005460-113008044	-	-	IGV_LINK	0	0	0	0	OK	0	0	0	0	OK	NOTEST	0
ENSG000001	-	-	ENSG0000013669	'IL36RN	-	2:113058637-113065382	-	-	IGV_LINK	0	0	0	0	OK	0	0	0	0	OK	NOTEST	0
ENSG000001	-	-	ENSG0000013669	'IL36B	-	2:113022090-113052867	-	-	IGV_LINK	0	0	0	0	OK	0	0	0	0	OK	NOTEST	0
ENSG000001	-	-	ENSG0000013669	'IL1F10	-	2:113067969-113075850	-	-	IGV_LINK	0	0	0	0	OK	0	0	0	0	OK	NOTEST	0
ENSG000001	-	-	ENSG0000013669	'CFC1	-	2:130592167-130599575	-	-	IGV_LINK	0	0	0	0	OK	0	0	0	0	OK	NOTEST	0
ENSG000001	-	-	ENSG0000013669	'SMPD4	-	2:130151391-130190729	-	-	IGV_LINK	1130.69	27.5958	0	64.5232	OK	1204.47	31.6502	0	71.0221	OK	OK	0.197764
ENSG000001	-	-	ENSG0000013670	'WDR33	-	2:127701021-127811187	-	-	IGV_LINK	1575.8	17.3581	2.5547	32.1615	OK	1369.47	15.061	0.828655	29.2933	OK	OK	-0.20479
ENSG000001	-	-	ENSG0000013671	'CCDC115	-	2:130338240-130347810	-	-	IGV_LINK	1011.42	38.8358	6.1594	71.5122	OK	865.198	33.8833	5.98583	61.7809	OK	OK	-0.19681
ENSG000001	-	-	ENSG0000013671	'SAP130	-	2:127941216-128028120	-	-	IGV_LINK	442.193	6.29025	2.24828	10.3322	OK	350.616	5.26103	1.41369	9.10837	OK	OK	-0.25777
ENSG000001	-	-	ENSG0000013671	'BIN1	-	2:127048026-127107355	-	-	IGV_LINK	847.536	21.1694	7.76121	34.5776	OK	669.265	18.0565	5.79176	30.3212	OK	OK	-0.22947
ENSG000001	-	-	ENSG0000013671	'IMP4	-	2:130338240-130347810	-	-	IGV_LINK	803.898	44.6958	0	91.861	OK	839.417	48.7707	0	97.658	OK	OK	0.125875
ENSG000001	-	-	ENSG0000013672	'HS6ST1	-	2:128236715-128318577	-	-	IGV_LINK	512.012	7.67518	2.91333	12.437	OK	441.364	6.61817	2.34006	10.8963	OK	OK	-0.21377
ENSG000001	-	-	ENSG0000013673	'UGGT1	-	2:128091199-128195677	-	-	IGV_LINK	1036.63	9.64566	0	22.8125	OK	1157.03	9.34602	0	21.5753	OK	OK	-0.04553
ENSG000001	-	-	ENSG0000013673	'GYPC	-	2:126655932-126696675	-	-	IGV_LINK	707.896	40.2523	15.8629	64.6417	OK	520.769	28.5479	10.3293	46.7665	OK	OK	-0.49569
ENSG000001	-	-	ENSG0000013673	'STAM	-	10:17644124-17715914	-	-	IGV_LINK	344.251	7.379	1.86128	12.8967	OK	267.087	6.57874	0	13.2611	OK	OK	-0.16562
ENSG000001	-	-	ENSG0000013675	'GAD2	-	10:26216306-26304558	-	-	IGV_LINK	3.87888	0.038449	0	0.297787	OK	4.1249	0.040895	0	0.30125	OK	NOTEST	0.089006
ENSG000001	-	-	ENSG0000013675	'ABI1	-	10:26697658-26861087	-	-	IGV_LINK	1054.09	28.7737	9.46378	48.0836	OK	769.294	20.8001	7.0087	34.5916	OK	OK	-0.46816
ENSG000001	-	-	ENSG0000013675	'YME1L1	-	10:27110111-27186924	-	-	IGV_LINK	1836.65	30.6221	11.2456	49.9986	OK	1399.37	24.8327	7.85322	41.8122	OK	OK	-0.30233
ENSG000001	-	-	ENSG0000013677	'DNAJC1	-	10:21756536-22003769	-	-	IGV_LINK	703.047	20.5378	8.44284	32.6327	OK	384.647	11.3199	3.97289	18.6668	OK	OK	-0.85943
ENSG000001	-	-	ENSG0000013678	'NIPSNAP3A	-	9:104747687-104760122	-	-	IGV_LINK	305.462	11.9829	3.93251	20.0332	OK	245.432	9.52166	2.75359	16.2897	OK	OK	-0.33169
ENSG000001	-	-	ENSG0000013680	'LRRC8A	-	9:128832941-128918039	-	-	IGV_LINK	1062.81	14.743	4.9397	24.5464	OK	1096.19	14.914	5.84389	23.9841	OK	OK	0.016634
ENSG000001	-	-	ENSG0000013680	'CDK9	-	9:127785678-127790787	-	-	IGV_LINK	1112.27	38.1962	8.12277	68.2696	OK	1077.63	34.7573	9.21564	60.2989	OK	OK	-0.13611
ENSG000001	-	-	ENSG0000013681	'TXN	-	9:110243810-110256640	-	-	IGV_LINK	1721.25	139.869	61.3415	218.397	OK	2614.16	213.137	93.0117	333.262	OK	OK	0.607704
ENSG000001	-	-	ENSG0000013681	'ODF2	-	9:128455185-128501292	-	-	IGV_LINK	882.446	20.4588	6.19648	34.7211	OK	879.635	19.7191	6.50698	32.9311	OK	OK	-0.05313
ENSG000001	-	-	ENSG0000013681	'KIAA0368	-	9:111360691-111484745	-	-	IGV_LINK	2395.21	30.7688	0	65.5199	OK	1886.11	25.2945	0	54.3955	OK	OK	-0.28265
ENSG000001	-	-	ENSG0000013681	'TOR1B	-	9:129803152-129811281	-	-	IGV_LINK	424.738	10.9674	1.98155	19.9532	OK	608.423	14.423	4.16394	24.682	OK	OK	0.395154
ENSG000001	-	-	ENSG0000013681	'C9orf78	-	9:129827289-129881838	-	-	IGV_LINK	881.476	37.0374	2.45937	71.6154	OK	979.664	45.8057	2.78001	88.8313	OK	OK	0.306544
ENSG000001	-	-	ENSG0000013682	'SMC2	-	9:104094259-104141417	-	-	IGV_LINK	455.769	5.31507	1.54601	9.08413	OK	389.803	4.27537	1.45366	7.09708	OK	OK	-0.31404
ENSG000001	-	-	ENSG0000013682	'KLF4	-	9:107484851-107490482	-	-	IGV_LINK	225.945	5.11365	1.42463	8.80267	OK	647.609	15.7343	5.52087	25.9478	OK	OK	1.62149
ENSG000001	-	-	ENSG0000013682	'TOR1A	-	9:129812943-129824134	-	-	IGV_LINK	630.318	20.4214	7.67898	33.1638	OK	831.167	25.8176	10.6201	41.0151	OK	OK	0.338277
ENSG000001	-	-	ENSG0000013682	'RALGPS1	-	9:126914773-127223166	-	-	IGV_LINK	145.458	2.46173	0	7.85823	OK	117.56	2.61007	0	11.2459	OK	OK	0.084418
ENSG000001	-	-	ENSG0000013683	'FAM129B	-	9:127505338-127578989	-	-	IGV_LINK	1606.83	26.1793	9.54388	42.8147	OK	2776.06	49.0478	9.08283	89.0128	OK	OK	0.905762
ENSG000001	-	-	ENSG0000013683	'OR1J1	-	9:122476957-122477926	-	-	IGV_LINK	0	0	0	0	OK	0	0	0	0	OK	NOTEST	0
ENSG000001	-	-	ENSG0000013683	'OR13C9	-	9:104617247-104618204	-	-	IGV_LINK	0	0	0	0	OK	0	0	0	0	OK	NOTEST	0
ENSG000001	-	-	ENSG0000013684	'ST6GALNAC4	-	9:127907885-127917038	-	-	IGV_LINK	220.127	9.20972	1.98961	16.4298	OK	360.929	14.8348	3.81763	25.852	OK	OK	0.687758
ENSG000001	-	-	ENSG0000013684	'TMOD1	-	9:97501179-97633575	-	-	IGV_LINK	27.1522	0.49576	0	1.80898	OK	502.207	8.93	0.993909	16.8661	OK	OK	4.17095
ENSG000001	-	-	ENSG0000013684	'DAB2IP	-	9:121567056-121785530	-	-	IGV_LINK	1549.61	15.1941	3.33972	27.0484	OK	2135.67	21.1162	4.5917	37.6407	OK	OK	0.47484
ENSG000001	-	-	ENSG0000013685	'STXBP1	-	9:127579369-127735317	-	-	IGV_LINK	2366.12	59.0243	2.14935	115.899	OK	1956.23	53.8875	1.79059	105.984	OK	OK	-0.13136
ENSG000001	-	-	ENSG0000013685	'SLC2A8	-	9:127397137-127408424	-	-	IGV_LINK	294.795	12.0769	2.65371	21.5001	OK	366.085	15.3892	2.7035	28.0748	OK	OK	0.349661
ENSG000001	-	-	ENSG0000013685	'ANGPTL2	-	9:126914773-127223166	-	-	IGV_LINK	1041.48	19.1774	5.16865	33.1861	OK	644.516	11.7349	0	23.708	OK	OK	-0.7086
ENSG000001	-	-	ENSG0000013686	'CDK5RAP2	-	9:120388868-120580170	-	-	IGV_LINK	1185.97	17.548	2.38826	32.7078	OK	1735.55	27.1879	4.93874	49.437	OK	OK	0.631654
ENSG000001	-	-	ENSG0000013686	'ZFP37	-	9:112957721-113056759	-	-	IGV_LINK	88.2446	2.16005	0	4.60478	OK	67.0296	1.78226	0	4.03235	OK	OK	-0.27736
ENSG000001	-	-	ENSG0000013686	'SLC31A2	-	9:113150941-113221361	-	-	IGV_LINK	77.5776	3.47678	0	16.1123	OK	139.215	6.2772	0	25.0107	OK	OK	0.852371
ENSG000001	-	-	ENSG0000013686	'SLC31A1	-	9:113221561-113292905	-	-	IGV_LINK	487.769	6.1817	0	12.7736	OK	743.513	9.70837	0	20.9713	OK	OK	0.651225
ENSG000001	-	-	ENSG0000013686	'TLR4	-	9:117704331-117716871	-	-	IGV_LINK	945.477	12.6704	4.36104	20.9798	OK	1133.32	14.9465	6.16987	23.7231	OK	OK	0.238345
ENSG000001	-	-	ENSG0000013687	'ZNF189	-	9:101398872-101410660	-	-	IGV_LINK	774.807	15.0892	6.2193	23.9591	OK	757.95	14.7119	5.96968	23.4541	OK	OK	-0.03653
ENSG000001	-	-	ENSG0000013687	'ALDOB	-	9:101420577-101435823	-	-	IGV_LINK	22.3036	0.551675	0	1.3486	OK	13.4059	0.331756	0	1.01345	OK	NOTEST	-0.7337
ENSG000001	-	-	ENSG0000013687	'STX17	-	9:99906632-99970341	-	-	IGV_LINK	437.344	10.898	2.90963	18.8864	OK	326.898	7.82543	2.16544	13.4854	OK	OK	-0.47782
ENSG000001	-	-	ENSG0000013687	'PRPF4	-	9:113221561-113292905	-	-	IGV_LINK	434.435	8.96025	0	19.0461	OK	672.359	13.9446	0	28.417	OK	OK	0.638094
ENSG000001	-	-	ENSG0000013687	'FPGS	-	9:127794596-127814327	-	-	IGV_LINK	672.986	27.4159	6.72227	48.1096	OK	735.263	31.8539	6.51763	57.1902	OK	OK	0.216457
ENSG000001	-	-	ENSG0000013687	'USP20	-	9:129827289-129881838	-	-	IGV_LINK	769.958	15.055	0	33.4258	OK	800.231	14.7261	0	33.3027	OK	OK	-0.03187
ENSG000001	-	-	ENSG0000013688	'BAAT	-	9:101358931-101383519	-	-	IGV_LINK	8.72748	0.153488	0	0.361298	OK	30.9367	0.616146	0	1.2859	OK	OK	2.00514
ENSG000001	-	-	ENSG0000013688	'KIF12	-	9:114086125-114099291	-	-	IGV_LINK	4211.5	126.088	24.4657	227.71	OK	2375.94	69.5957	18.1707	121.021	OK	OK	-0.85736
ENSG000001	-	-	ENSG0000013688	'ATP6V1G1	-	9:114587745-114598373	-	-	IGV_LINK	1313	81.7992	35.9818	127.617	OK	1473.62	91.6301	40.6381	142.622	OK	OK	0.163735
ENSG000001	-	-	ENSG0000013689	'TEX10	-	9:100302076-100352939	-	-	IGV_LINK	453.829	7.85803	2.5875	13.1286	OK	419.709	7.4908	2.18535	12.7963	OK	OK	-0.06905
ENSG000001	-	-	ENSG0000013689	'GARNL3	-	9:127224264-127393660	-	-	IGV_LINK	219.157	7.94857	0	18.1351	OK	103.122	2.5051	0	6.12877	OK	OK	-1.66583
ENSG000001	-	-	ENSG0000013689	'MRPL50	-	9:101387632-101398614	-	-	IGV_LINK	360.736	6.43443	2.24409	10.6248	OK	374.335	6.67942	2.3028	11.056	OK	OK	0.053911
ENSG000001	-	-	ENSG0000013690	'DPM2	-	9:127934502-127980513	-	-	IGV_LINK	696.259	30.3259	6.25136	54.4004	OK	761.044	34.0962	0	69.8636	OK	OK	0.169063
ENSG000001	-	-	ENSG0000013691	'WDR38	-	9:124853416-124861981	-	-	IGV_LINK	26.1825	1.29944	0	16.341	OK	19.5933	1.00648	0	13.3471	OK	OK	-0.36857
ENSG000001	-	-	ENSG0000013692	'TSTD2	-	9:97501179-97633575	-	-	IGV_LINK	476.133	8.8048	1.25328	16.3563	OK	633.172	12.0297	0.193739	23.8656	OK	OK	0.450236
ENSG000001	-	-	ENSG0000013692	'GABBR2	-	9:98288081-98709197	-	-	IGV_LINK	0.969721	0.023073	0	1.49993	OK	1.03122	0.010351	0	1.48334	OK	NOTEST	-1.15643
ENSG000001	-	-	ENSG0000013692	'HEMGN	-	9:97926790-97944856	-	-	IGV_LINK	3.87888	0.102508	0	0.270099	OK	2.06245	0.057583	0	0.181679	OK	NOTEST	-0.83204
ENSG000001	-	-	ENSG0000013693	'PSMB7	-	9:124353465-124415444	-	-	IGV_LINK	1906.47	133.84	59.1856	208.495	OK	2053.17	145.373	63.9347	226.811	OK	OK	0.119247
ENSG000001	-	-	ENSG0000013693	'NR5A1	-	9:124481235-124507430	-	-	IGV_LINK	0	0	0	0	OK	0	0	0	0	OK	NOTEST	0
ENSG000001	-	-	ENSG0000013693	'TRMO	-	9:97904488-97922570	-	-	IGV_LINK	252.127	10.355	1.62393	19.0861	OK	305.243	12.0981	2.80492	21.3913	OK	OK	0.224453
ENSG000001	-	-	ENSG0000013693	'RABEPK	-	9:125200541-125234158	-	-	IGV_LINK	204.611	8.95256	2.40919	15.4959	OK	258.837	11.0986	3.30763	18.8896	OK	OK	0.31001
ENSG000001	-	-	ENSG0000013693	'GOLGA1	-	9:124878356-125143506	-	-	IGV_LINK	699.115	9.76642	1.91944	17.6134	OK	830.544	12.3162	1.84605	22.7864	OK	OK	0.334661
ENSG000001	-	-	ENSG0000013693	'XPA	-	9:97674908-97697357	-	-	IGV_LINK	200.732	9.05245	2.20548	15.8994	OK	205.214	9.11215	2.24725	15.9771	OK	OK	0.009484
ENSG000001	-	-	ENSG0000013693	'NCBP1	-	9:97633625-97673748	-	-	IGV_LINK	497.467	7.10452	0.297412	13.9116	OK	543.456	6.72088	1.22176	12.22	OK	OK	-0.08009
ENSG000001	-	-	ENSG0000013693	'ANP32B	-	9:97983360-98015943	-	-	IGV_LINK	1671.8	74.3287	31.1549	117.502	OK	1421.03	62.1956	27.525	96.8663	OK	OK	-0.25711
ENSG000001	-	-	ENSG0000013693	'OR1L4	-	9:122723989-122724925	-	-	IGV_LINK	0	0	0	0	OK	0	0	0	0	OK	NOTEST	0
ENSG000001	-	-	ENSG0000013694	'PDCL	-	9:122798388-122828631	-	-	IGV_LINK	286.068	7.10459	0.986993	13.2222	OK	293.899	6.90588	1.31011	12.5017	OK	OK	-0.04093
ENSG000001	-	-	ENSG0000013694	'RPL35	-	9:124853416-124861981	-	-	IGV_LINK	8545.18	1699.12	609.99	2788.25	OK	7504.22	1499.42	559.79	2439.05	OK	OK	-0.18039
ENSG000001	-	-	ENSG0000013694	'CTSV	-	9:97029678-97039643	-	-	IGV_LINK	10.6669	0.399754	0	0.979443	OK	25.7806	1.0587	0	2.23785	OK	OK	1.40511

ENSG000001	-	-	ENSG0000013694	LMX1B	-	9:126614442-126701032	-	-	IGV_LINK	0	0	0	0	OK	0	0	0	0	OK	NOTEST	0
ENSG000001	-	-	ENSG0000013695	ARPC5L	-	9:124862129-124877733	-	-	IGV_LINK	513.952	21.7359	7.41936	36.0524	OK	454.77	19.6197	6.67625	32.5632	OK	OK	-0.14777
ENSG000001	-	-	ENSG0000013696	ENPP2	-	8:119557085-119673453	-	-	IGV_LINK	4604.23	113.704	43.881	183.527	OK	5353.09	133.8	48.9418	218.658	OK	OK	0.234796
ENSG000001	-	-	ENSG0000013698	DSCC1	-	8:119833975-119856010	-	-	IGV_LINK	39.7585	1.0463	0	2.26735	OK	54.6549	1.84409	0	4.10592	OK	OK	0.817617
ENSG000001	-	-	ENSG0000013698	DERL1	-	8:123002559-123152153	-	-	IGV_LINK	3864.34	124.69	4.92716	244.453	OK	6281.19	250.964	0	502.599	OK	OK	1.00913
ENSG000001	-	-	ENSG0000013699	MYC	-	8:127735433-127741434	-	-	IGV_LINK	2034.47	71.1496	25.3534	116.946	OK	2454.32	87.4004	30.8353	143.966	OK	OK	0.296784
ENSG000001	-	-	ENSG0000013699	NOV	-	8:119416305-119462350	-	-	IGV_LINK	218.187	5.23352	1.45864	9.0084	OK	116.528	2.79647	0.419707	5.17323	OK	OK	-0.90418
ENSG000001	-	-	ENSG0000013703	IL33	-	9:6215785-6257983	-	-	IGV_LINK	20.3641	0.558123	0	1.35622	OK	131.997	3.06698	0.495248	5.6387	OK	OK	2.45816
ENSG000001	-	-	ENSG0000013703	DMAC1	-	9:7796489-7888380	-	-	IGV_LINK	596.378	35.8224	1.54849	70.0964	OK	599.142	40.7538	3.43179	78.0758	OK	OK	0.18607
ENSG000001	-	-	ENSG0000013704	RANBP6	-	9:6011042-6015625	-	-	IGV_LINK	438.314	6.19406	1.6292	10.7589	OK	378.46	5.54594	1.11453	9.97735	OK	OK	-0.15945
ENSG000001	-	-	ENSG0000013705	POLR1E	-	9:37485934-37503697	-	-	IGV_LINK	458.678	15.2388	5.73736	24.7403	OK	365.054	12.1174	4.26834	19.9666	OK	OK	-0.33067
ENSG000001	-	-	ENSG0000013705	PLAA	-	9:26904082-27062930	-	-	IGV_LINK	363.645	7.6013	0	17.0342	OK	575.424	13.7843	0	28.9131	OK	OK	0.858709
ENSG000001	-	-	ENSG0000013707	IL11RA	-	9:34638132-34681298	-	-	IGV_LINK	463.02	18.8563	0	44.3167	OK	258.397	11.4135	0	30.7882	OK	OK	-0.72431
ENSG000001	-	-	ENSG0000013707	UBAP2	-	9:33921692-34048949	-	-	IGV_LINK	534.316	8.68994	2.81243	14.5675	OK	590.892	9.20206	3.24406	15.1601	OK	OK	0.08261
ENSG000001	-	-	ENSG0000013707	APTX	-	9:32972605-33025168	-	-	IGV_LINK	400.495	18.4138	3.62585	33.2017	OK	501.175	23.4072	5.30626	41.5082	OK	OK	0.346168
ENSG000001	-	-	ENSG0000013707	RNF38	-	9:36336395-36487548	-	-	IGV_LINK	785.474	9.70038	2.43503	16.9657	OK	639.359	8.06593	1.46082	14.671	OK	OK	-0.2662
ENSG000001	-	-	ENSG0000013707	TLN1	-	9:35696947-35749228	-	-	IGV_LINK	7450.36	75.6555	0	211.53	OK	8382.83	87.5168	0	251.722	OK	OK	0.210116
ENSG000001	-	-	ENSG0000013707	CCL21	-	9:34709004-34710124	-	-	IGV_LINK	5.81832	0.482577	0	1.21155	OK	12.3747	1.0281	0	2.3725	OK	OK	1.09114
ENSG000001	-	-	ENSG0000013707	SIT1	-	9:35649294-35650950	-	-	IGV_LINK	195.884	10.2652	2.5156	18.0148	OK	88.6853	4.7665	0.151378	9.38163	OK	OK	-1.10676
ENSG000001	-	-	ENSG0000013708	IFNA21	-	9:21165636-21166660	-	-	IGV_LINK	1.93944	0.128363	0	0.393107	OK	1.03122	0.068345	0	0.2558	OK	NOTEST	-0.90933
ENSG000001	-	-	ENSG0000013709	DMRT1	-	9:841689-969090	-	-	IGV_LINK	0	0	0	0	OK	0	0	0	0	OK	NOTEST	0
ENSG000001	-	-	ENSG0000013709	DNAJB5	-	9:34989640-34998900	-	-	IGV_LINK	81.4565	2.16377	0	4.40155	OK	141.278	3.95495	0.009819	7.90008	OK	OK	0.870115
ENSG000001	-	-	ENSG0000013709	SPAG8	-	9:35792153-35812272	-	-	IGV_LINK	190.065	10.5552	0	21.4534	OK	119.622	5.12898	0	12.1562	OK	OK	-1.04121
ENSG000001	-	-	ENSG0000013710	DCTN3	-	9:34613544-34620523	-	-	IGV_LINK	810.686	67.5639	24.886	110.242	OK	798.168	67.7213	26.2139	109.229	OK	OK	0.003357
ENSG000001	-	-	ENSG0000013710	CD72	-	9:35605304-35647099	-	-	IGV_LINK	425.707	15.5024	0.809783	30.1951	OK	338.242	12.0454	0	24.6168	OK	OK	-0.36401
ENSG000001	-	-	ENSG0000013710	TMEM8B	-	9:35812959-35865518	-	-	IGV_LINK	421.828	6.11941	0	12.7394	OK	304.211	4.45828	0	10.3903	OK	OK	-0.45691
ENSG000001	-	-	ENSG0000013710	GRHPR	-	9:37422665-37436990	-	-	IGV_LINK	1937.26	96.8759	37.8616	155.89	OK	2034.42	102.637	34.8944	170.38	OK	OK	0.083347
ENSG000001	-	-	ENSG0000013712	ALDH1B1	-	9:38392663-38398661	-	-	IGV_LINK	226.915	4.41955	1.2555	7.58361	OK	818.793	17.0339	5.54693	28.5208	OK	OK	1.94643
ENSG000001	-	-	ENSG0000013713	HINT2	-	9:35812959-35865518	-	-	IGV_LINK	361.706	42.2012	5.01942	79.3829	OK	515.612	60.1863	15.2899	105.083	OK	OK	0.512153
ENSG000001	-	-	ENSG0000013713	ARHGEF39	-	9:35658289-35681159	-	-	IGV_LINK	279.28	5.97355	0	20.6062	OK	336.179	7.7088	0	29.2069	OK	OK	0.367916
ENSG000001	-	-	ENSG0000013714	IGFBPL1	-	9:38408993-38424447	-	-	IGV_LINK	0	0	0	0	OK	0	0	0	0	OK	NOTEST	0
ENSG000001	-	-	ENSG0000013714	DENND4C	-	9:19230434-19373545	-	-	IGV_LINK	1024.99	12.1324	4.68379	19.5809	OK	952.852	12.9622	4.63608	21.2884	OK	OK	0.095453
ENSG000001	-	-	ENSG0000013715	RPS6	-	9:19375450-19380254	-	-	IGV_LINK	24280.8	1612.9	176.294	3049.51	OK	20079	1314.13	210.654	2417.61	OK	OK	-0.29555
ENSG000001	-	-	ENSG0000013716	CNPY3	-	6:42929191-42939287	-	-	IGV_LINK	1506.95	55.5473	24.4919	86.6028	OK	1148.78	42.3049	18.4498	66.16	OK	OK	-0.39289
ENSG000001	-	-	ENSG0000013716	FOXP4	-	6:41494852-41602384	-	-	IGV_LINK	468.375	7.33396	2.18304	12.4849	OK	275.337	4.55467	0	9.26622	OK	OK	-0.68724
ENSG000001	-	-	ENSG0000013716	PPIL1	-	6:36854826-36928964	-	-	IGV_LINK	220.127	8.56698	0	25.2726	OK	178.402	6.4232	0	17.8896	OK	OK	-0.4155
ENSG000001	-	-	ENSG0000013717	KLC4	-	6:43037616-43075099	-	-	IGV_LINK	1235.42	48.3866	0	106.417	OK	1163.22	41.8957	0	86.8414	OK	OK	-0.20781
ENSG000001	-	-	ENSG0000013717	KIF13A	-	6:17759182-17987623	-	-	IGV_LINK	790.322	9.50559	2.84921	16.162	OK	431.052	5.36235	1.11048	9.61421	OK	OK	-0.82591
ENSG000001	-	-	ENSG0000013718	ZSCAN9	-	6:28224885-28233482	-	-	IGV_LINK	322.917	12.2528	3.65451	20.8511	OK	192.839	6.90731	1.61294	12.2017	OK	OK	-0.82692
ENSG000001	-	-	ENSG0000013719	PIM1	-	6:37170202-37175426	-	-	IGV_LINK	748.624	24.1536	6.02775	42.2794	OK	826.011	24.2034	6.46461	41.9421	OK	OK	0.002971
ENSG000001	-	-	ENSG0000013719	GMPR	-	6:16238579-16295549	-	-	IGV_LINK	97.9418	4.24297	0	8.54338	OK	97.9664	4.40619	0.253716	8.55867	OK	OK	0.054457
ENSG000001	-	-	ENSG0000013720	CMTR1	-	6:37433218-37482827	-	-	IGV_LINK	1617.49	30.5083	7.5176	53.4989	OK	1053.91	19.6685	4.86629	34.4707	OK	OK	-0.63331
ENSG000001	-	-	ENSG0000013720	TFAP2A	-	6:10393185-10419659	-	-	IGV_LINK	10.6669	0.342994	0	1.27216	OK	63.9359	1.82908	0	4.28368	OK	OK	2.41486
ENSG000001	-	-	ENSG0000013720	SLC22A7	-	6:43295693-43369478	-	-	IGV_LINK	1.93944	0.208794	0	2.82914	OK	0	0	0	0	OK	NOTEST	'-inf
ENSG000001	-	-	ENSG0000013720	YIPF3	-	6:43506968-43576075	-	-	IGV_LINK	2204.17	94.397	21.9207	166.873	OK	1648.93	68.5645	20.247	116.882	OK	OK	-0.46128
ENSG000001	-	-	ENSG0000013721	TMEM14B	-	6:10747758-10979320	-	-	IGV_LINK	691.411	56.7338	18.4673	95.0003	OK	492.926	39.8202	12.6077	67.0328	OK	OK	-0.51071
ENSG000001	-	-	ENSG0000013721	TMEM63B	-	6:44113453-44155519	-	-	IGV_LINK	893.113	19.6478	3.93145	35.3641	OK	573.361	11.4032	2.83819	19.9683	OK	OK	-0.78492
ENSG000001	-	-	ENSG0000013721	FRS3	-	6:41770175-41895361	-	-	IGV_LINK	126.064	3.74802	0	12.4653	OK	98.9976	2.98945	0	9.6703	OK	OK	-0.32625
ENSG000001	-	-	ENSG0000013722	TJAP1	-	6:43477522-43506556	-	-	IGV_LINK	874.688	23.0735	5.17162	40.9754	OK	580.58	15.1917	2.68109	27.7024	OK	OK	-0.60295
ENSG000001	-	-	ENSG0000013722	CAPN11	-	6:44158810-44184402	-	-	IGV_LINK	8.72748	0.192578	0	0.600061	OK	22.6869	0.860543	0	1.95577	OK	OK	2.1598
ENSG000001	-	-	ENSG0000013725	TINAG	-	6:54307858-54390152	-	-	IGV_LINK	345.22	16.1216	3.69976	28.5434	OK	305.243	14.2765	3.0963	25.4567	OK	OK	-0.17535
ENSG000001	-	-	ENSG0000013725	HCRTR2	-	6:55106459-55282620	-	-	IGV_LINK	0	0	0	0	OK	0	0	0	0	OK	NOTEST	0
ENSG000001	-	-	ENSG0000013726	KIAA0319	-	6:24544103-24646155	-	-	IGV_LINK	37.8191	0.350809	0	0.716622	OK	49.4988	0.485438	0	1.03608	OK	NOTEST	0.468601
ENSG000001	-	-	ENSG0000013726	IRF4	-	6:391738-411447	-	-	IGV_LINK	209.46	3.48053	0	9.72358	OK	183.558	2.35977	0	4.9647	OK	OK	-0.56066
ENSG000001	-	-	ENSG0000013726	SLC22A23	-	6:3224260-3457022	-	-	IGV_LINK	320.977	3.38607	0	7.57206	OK	667.203	7.46222	0.361619	14.5628	OK	OK	1.13999
ENSG000001	-	-	ENSG0000013726	TUBB2A	-	6:3153668-3157526	-	-	IGV_LINK	33.9402	1.35943	0	2.79007	OK	44.3427	1.90808	0	3.91399	OK	OK	0.48912
ENSG000001	-	-	ENSG0000013726	LRRC1	-	6:53794496-53924121	-	-	IGV_LINK	156.125	3.30334	0	6.74514	OK	176.339	4.4392	0	9.49103	OK	OK	0.426372
ENSG000001	-	-	ENSG0000013727	GCM1	-	6:53126963-53148829	-	-	IGV_LINK	1.93944	0.04216	0	0.129112	OK	2.06245	0.044854	0	0.131844	OK	NOTEST	0.089359
ENSG000001	-	-	ENSG0000013727	FOXF2	-	6:1389833-1395597	-	-	IGV_LINK	0.969721	0.027128	0	0.106254	OK	4.1249	0.115461	0	0.292493	OK	NOTEST	2.08954
ENSG000001	-	-	ENSG0000013727	BPHL	-	6:3118373-3153578	-	-	IGV_LINK	325.826	12.2969	1.18168	23.4122	OK	193.87	7.15614	0	14.6931	OK	OK	-0.78104
ENSG000001	-	-	ENSG0000013727	RIPK1	-	6:3063990-3115187	-	-	IGV_LINK	982.327	14.7681	6.07455	23.4616	OK	808.48	12.1412	4.78387	19.4985	OK	OK	-0.28257
ENSG000001	-	-	ENSG0000013728	TUBB2B	-	6:3224260-3457022	-	-	IGV_LINK	8.72748	0.280976	0	1.71918	OK	6.18735	0.199323	0	1.58708	OK	NOTEST	-0.49534
ENSG000001	-	-	ENSG0000013728	UQCC2	-	6:33620364-33711727	-	-	IGV_LINK	532.377	28.7714	0	89.5707	OK	448.583	17.7568	0	60.2022	OK	OK	-0.69626
ENSG000001	-	-	ENSG0000013730	HMGA1	-	6:34236872-34246231	-	-	IGV_LINK	787.413	25.8064	10.2182	41.3945	OK	459.926	14.9935	5.60029	24.3866	OK	OK	-0.78339
ENSG000001	-	-	ENSG0000013731	TCF19	-	6:31158541-31180731	-	-	IGV_LINK	704.017	13.6075	0	33.3605	OK	445.489	8.69138	0	20.597	OK	OK	-0.64674
ENSG000001	-	-	ENSG0000013731	FLOT1	-	6:30727708-30742733	-	-	IGV_LINK	4393.8	161.071	57.4335	264.709	OK	3195.77	125.248	46.9959	203.499	OK	OK	-0.36292
ENSG000001	-	-	ENSG0000013733	IER3	-	6:30742928-30744554	-	-	IGV_LINK	819.414	42.5042	16.2877	68.7207	OK	681.64	35.4516	13.9935	56.9097	OK	OK	-0.26176
ENSG000001	-	-	ENSG0000013733	MDC1	-	6:30699806-30717889	-	-	IGV_LINK	1122.94	16.7202	0	35.1386	OK	571.299	8.75296	0	22.3251	OK	OK	-0.93375
ENSG000001	-	-	ENSG0000013733	PGBD1	-	6:28267009-28302549	-	-	IGV_LINK	94.0629	1.81076	0	5.44122	OK	46.4051	0.893673	0	2.45222	OK	OK	-1.01877
ENSG000001	-	-	ENSG0000013734	ATAT1	-	6:30626841-30646823	-	-	IGV_LINK	286.068	8.47967	1.79881	15.1605	OK	202.12	6.24009	1.04402	11.4362	OK	OK	-0.44244
ENSG000001	-	-	ENSG0000013736	TPMT	-	6:18128310-18155074	-	-	IGV_LINK	983.297	18.4193	7.95822	28.8804	OK	948.727	17.7785	7.65179	27.9053	OK	OK	-0.05108
ENSG000001	-	-	ENSG0000013739	CLPS	-	6:35794981-35797344	-	-	IGV_LINK	0	0	0	0	OK	0	0	0	0	OK	NOTEST	0
ENSG000001	-	-	ENSG0000013739	RNF144B	-	6:18387349-18468874	-	-	IGV_LINK	1306.21	15.1357	6.67305	23.5984	OK	1184.88	13.733	5.97016	21.4958	OK	OK	-0.14031
ENSG000001	-	-	ENSG0000013740	NRM	-	6:30688046-30691420	-	-	IGV_LINK	305.462	15.2204	3.67092	26.7698	OK	196.964	9.10031	1.81848	16.3821	OK	OK	-0.74202
ENSG000001	-	-	ENSG0000013740	MTCH1	-	6:36968140-36986298	-	-	IGV_LINK	2868.43	96.6479	30.0463	163.249	OK	1859.3	62.8217	22.1437	103.5	OK	OK	-0.62147
ENSG000001	-	-	ENSG0000013741	VARS2	-	6:30908183-30926459	-	-	IGV_LINK	1015.95	20.1131	3.3331	36.8931	OK	578.166	12.155	1.0576	23.2524	OK	OK	-0.72659
ENSG000001	-	-	ENSG0000013741	TAF8	-	6:42050512-42087461	-	-	IGV_LINK	551.771	9.84604	0.47311	19.219	OK	403.209	8.53258	0	17.5727	OK	OK	-0.20656
ENSG000001	-	-	ENSG0000013741	FAM8A1	-	6:17600354-17611719	-	-	IGV_LINK	640.985	8.01935	3.25525	12.7834	OK	546.549	6.83961	2.68717	10.992	OK	OK	-0.22957
ENSG000001	-	-	ENSG0000013743	C6orf52	-	6:10671417-10709782	-	-	IGV_LINK	3.87888	0.848234	0	5.30035	OK	5.15612	0.890846	0	4.66477	OK	OK	0.070714
ENSG000001	-	-	ENSG0000013744	FGFBP1	-	4:15935568-15938740	-	-	IGV_LINK	0	0	0	0	OK	0	0	0	0	OK	NOTEST	0
ENSG000001	-	-	ENSG0000013744	FGFBP2	-	4:15960242-16084378	-	-	IGV_LINK	97.9418	5.44465	0	24.5421	OK	58.7798	3.33148	0	6.73022	OK	OK	-0.73493
ENSG000001	-	-	ENSG0000013744	CPEB2	-	4:15002673-15446166	-	-	IGV_LINK	1197.6	18.2606	2.6963	33.8248	OK	1718.02	26.784	3.55245	50.0155	OK	OK	0.552638
ENSG000001	-	-	ENSG0000013746	FHDC1	-	4:152936351-152979696	-	-	IGV_LINK	55.2741	0.493851	0	1.00497	OK	49.4988	0.435231	0	0.874387	OK	NOTEST	-0.1823
ENSG000001	-	-	ENSG0000013746	TLR2	-	4:153701499-153705699	-	-	IGV_LINK	1245.12	17.4226	7.63321	27.2121	OK	1143.63	16.0071	6.97458	25.0395	OK	OK	-0.12226
ENSG000001	-	-	ENSG0000013746	MGARP	-	4:139266162-139476609	-	-	IGV_LINK	29.874	1.44329	0	8.47373	OK	13.4059	0.648315	0	5.29191	OK	OK	-1.1546
ENSG000001	-	-	ENSG0000013747	TTC29	-	4:146706637-146945882	-	-	IGV_LINK	7.75776	0.280695	0	0.858049	OK	18.562	0.652363	0	1.64734	OK	OK	1.21667
ENSG000001	-	-	ENSG0000013747	MYO7A	-	11:77128263-77215239	-	-	IGV_LINK	768.019	9.51912	0.671547	18.3667	OK	358.866	5.26608	0.094256	10.4379	OK	OK	-0.8541
ENSG000001	-	-	ENSG0000013747	FCHSD2	-	11:72814307-73142261	-	-	IGV_LINK	1387.67	24.7102	0	58.52	OK	1249.84	24.2246	0	58.0591	OK	OK	-0.02864
ENSG000001	-	-	ENSG0000013748	ARRB1	-	11:75264181-75351705	-	-	IGV_LINK	440.253	11.0653	3.34432	18.7863	OK	411.459	11.932	0.857348	23.0067	OK	OK	0.108795
ENSG000001	-	-	ENSG0000013749	SLCO2B1	-	11:75099171-75206549	-	-	IGV_LINK	2969.28	42.4027	12.2985	72.507	OK	5669.67	82.3746	19.5117	145.237	OK	OK	0.958042
ENSG000001	-	-	ENSG0000013749	THAP12	-	11:76349955-76380971	-	-	IGV_LINK	559.529	11.3835	3.15911	19.6079	OK	574.392	11.5053	3.5113	19.4993	OK	OK	0.015351
ENSG000001	-	-	ENSG0000013749	ANKRD42	-	11:83156987-83423516	-	-	IGV_LINK	418.09	10.9655	0	29.6204	OK	519.471	13.5072	0	34.3828	OK	OK	0.30075
ENSG000001	-	-	ENSG0000013749	IL18BP	-	11:71998540-72112780	-	-	IGV_LINK	946.447	39.2269	0	141.215	OK	844.573	34.8339	0	132.701	OK	OK	-0.17135
ENSG000001	-	-	ENSG0000013749	NUMA1	-	11:71998540-72112780	-	-	IGV_LINK	8424.93	144.847	0	317.423	OK	8611.76	145.398	0	355.579	OK	OK	0.005481
ENSG000001	-	-	ENSG0000013750	CCDC90B	-	11:83156987-83423516	-	-	IGV_LINK	408.252	21.4301	0	58.9979	OK	461.989	27.024	0	69.5336	OK	OK	0.334598
ENSG000001	-	-	ENSG0000013750	SYTL2	-	11:85694223-85811159	-	-	IGV_LINK	1473.01	61.3141	15.6356	106.993	OK	6276.04	268.941	58.2529	479.63	OK	OK	2.133
ENSG000001	-	-	ENSG0000013750	RAB30	-	11:82823501-83071923	-	-	IGV_LINK	226.915	3.73678	0	19.7598	OK	315.555	4.7156	0	24.0742	OK	OK	0.335645
ENSG000001	-	-	ENSG0000013750	CREBZF	-	11:85659707-85682908	-	-	IGV_LINK	1896.77	35.1601	10.8618	59.4583	OK	1712.86	32.5973	7.84843	57.3461	OK	OK	-0.10919
ENSG000001	-	-	ENSG0000013750	LRRC32	-	11:76657055-76670747	-	-	IGV_LINK	1145.24	21.4052	1.69099	41.1193	OK	902.322	15.4641	2.00906	28.9191	OK	OK	-0.46904
ENSG000001	-	-	ENSG0000013750	PRCP	-	11:82823501-83071923	-	-	IGV_LINK	2612.43	69.9801	27.305	112.655	OK	3192.67	88.4517	30.7828	146.121	OK	OK	0.337944
ENSG000001	-	-	ENSG0000013751	NARS2	-	11:78215296-78574874	-	-	IGV_LINK	290.916	10.0183	0	24.4783	OK	303.18	8.90441	0	23.9004	OK	OK	-0.17005
ENSG000001	-	-	ENSG0000013752	RNF121	-	11:71928700-71997597	-	-	IGV_LINK	589.59	24.2092	6.27661	42.1419	OK	601.204	25.6105	5.80803	45.413	OK	OK	0.081178
ENSG000001	-	-	ENSG0000013754	MRPL15	-	8:54135209-54147901	-	-	IGV_LINK	668.137	39.5742	16.0276	63.1208	OK	788.887	46.1739	19.1862	73.1615	OK	OK	0.222517
ENSG000001	-	-	ENSG0000013755	PI15	-	8:74599774-74866939	-	-	IGV_LINK	3.87888	0.044363	0	0.14725	OK	6.18735	0.053168	0	0.190744	OK	NOTEST	0.261206
ENSG000001	-	-	ENSG0000013756	TTPA	-	8:63048552-63086053	-	-	IGV_LINK	0	0	0	0	OK	3.09367	0.080554	0	0.500017	OK	NOTEST	inf
ENSG000001	-	-	ENSG0000013756	GGH	-	8:63015078-63039171	-	-	IGV_LINK	99.8812	4.24765	0.280037	8.21527	OK	295.962	13.1964	3.42411	22.9686	OK	OK	1.6354
ENSG000001	-	-	ENSG0000013757	SLCO5A1	-	8:69667046-69854971	-	-	IGV_LINK	44.6071	0.389089	0	0.944793	OK	12.3747	0.117311	0	0.454047	OK	NOTEST	-1.72976
ENSG000001	-	-	ENSG0000013757	SULF1	-	8:69466623-69660915	-	-	IGV_LINK	1310.09	18.3403	2.9044	33.7762	OK	1926.33	34.9579	2.25209	67.6638	OK	OK	0.930603
ENSG000001	-	-	ENSG0000013757	TGS1	-	8:55696423-55825448	-	-	IGV_LINK	310.311	5.0145	0.938271	9.09073	OK	302.149	4.82555	0.710088	8.94102	OK	OK	-0.05541
ENSG000001	-	-	ENSG0000013757	SDCBP	-	8:58552923-58582860	-	-	IGV_LINK	2518.36	117.317	43.1279	191.506	OK	3562.88	169.075	46.6838	291.467	OK	OK	0.527255
ENSG000001	-	-	ENSG0000013760	NEK1	-	4:169392856-169612629	-	-	IGV_LINK	361.706	4.11008	0.995054	7.2251	OK	417.646	4.44562	1.37207	7.51918	OK	OK	0.11322
ENSG000001	-	-	ENSG0000013762	DDX60	-	4:168216292-168318807	-	-	IGV_LINK	828.141	16.1371	0	34.4416	OK	1085.88	17.9308	0	37.774	OK	OK	0.152059
ENSG000001	-	-	ENSG0000013763	NXPE4	-	11:114570590-114595762	-	-	IGV_LINK	0.969721	0.03608	0	0.161725	OK	1.03122	0.038398	0	0.163976	OK	NOTEST	0.089826
ENSG000001	-	-	ENSG0000013764	SORL1	-	11:121447330-121633693	-	-	IGV_LINK	5412.01	35.5513	4.0418	67.0608	OK	2547.13	16.6292	1.83678	31.4216	OK	OK	-1.09618
ENSG000001	-	-	ENSG0000013764	TMPRSS4	-	11:118003633-118121890	-	-	IGV_LINK	0	0	0	0	OK	1.03122	0.017914	0	0.728711	OK	NOTEST	inf
ENSG000001	-	-	ENSG0000013765	BUD13	-	11:116748169-116772988	-	-	IGV_LINK	416.98	11.8985	4.24883	19.5481	OK	376.397	10.5361	3.73936	17.3329	OK	OK	-0.17543
ENSG000001	-	-	ENSG0000013767	TRPC6	-	11:101451563-101872562	-	-	IGV_LINK	64.0016	1.06693	0	2.51534	OK	73.217	1.43777	0	3.50515	OK	OK	0.430368
ENSG000001	-	-	ENSG0000013767	MMP7	-	11:102520507-102530753	-	-	IGV_LINK	3632.57	214.748	91.4339	338.063	OK	24146.1	1429.46	269.547	2589.38	OK	OK	2.73475
ENSG000001	-	-	ENSG0000013767	MMP20	-	11:102576834-102628070	-	-	IGV_LINK	0	0	0	0	OK	0	0	0	0	OK	NOTEST	0
ENSG000001	-	-	ENSG0000013767	MMP27	-	11:102691486-102705806	-	-	IGV_LINK	0	0	0	0	OK	3.09367	0.100237	0	0.258968	OK	NOTEST	inf
ENSG000001	-	-	ENSG0000013769	C11orf70	-	11:102047442-102084560	-	-	IGV_LINK	28.1219	1.29904	0	3.15572	OK	30.9367	2.12913	0	5.19044	OK	OK	0.712818
ENSG000001	-	-	ENSG0000013769	DCUN1D5	-	11:103062075-103092215	-	-	IGV_LINK	155.155	7.73519	1.53364	13.9367	OK	214.495	11.2724	2.72468	19.82	OK	OK	0.543281
ENSG000001	-	-	ENSG0000013769	YAP1	-	11:102110460-102233423	-	-	IGV_LINK	879.536	15.8459	4.08275	27.6091	OK	889.947	17.1573	3.17453	31.1401	OK	OK	0.114709
ENSG000001	-	-	ENSG0000013769	TRIM29	-	11:120111274-120185529	-	-	IGV_LINK	9.69721	0.30068	0	1.40531	OK	60.8423	2.41098	0	7.91759	OK	OK	3.00332
ENSG000001	-	-	ENSG0000013770	SLC37A4	-	11:118998141-119030906	-	-	IGV_LINK	3549.18	92.4404	0	253.295	OK	5341.75	148.408	0	337.452	OK	OK	0.682976
ENSG000001	-	-	ENSG0000013770	BTG4	-	11:111352251-111512354	-	-	IGV_LINK	0	0	0	0	OK	0	0	0	0	OK	NOTEST	0
ENSG000001	-	-	ENSG0000013770	POU2F3	-	11:120236639-120319944	-	-	IGV_LINK	11.6366	0.32524	0	0.943662	OK	7.21857	0.225947	0	0.651814	OK	NOTEST	-0.52552
ENSG000001	-	-	ENSG0000013771	RDX	-	11:110174879-110296722	-	-	IGV_LINK	1421.61	33.091	5.7079	60.4741	OK	1477.75	34.7815	6.72701	62.836	OK	OK	0.071882
ENSG000001	-	-	ENSG0000013771	PPP2R1B	-	11:111602390-111766427	-	-	IGV_LINK	422.798	8.05665	0	19.4768	OK	572.33	12.2737	0	27.0312	OK	OK	0.607321
ENSG000001	-	-	ENSG0000013771	FDX1	-	11:110429882-110464881	-	-	IGV_LINK	264.734	4.91818	1.51625	8.3201	OK	307.305	5.71123	1.89811	9.52434	OK	OK	0.215677
ENSG000001	-	-	ENSG0000013772	C11orf1	-	11:111782194-111885975	-	-	IGV_LINK	624.5	71.2461	4.07388	138.418	OK	494.988	56.0395	3.98488	108.094	OK	OK	-0.34637
ENSG000001	-	-	ENSG0000013772	FXYD6	-	11:117427772-117877486	-	-	IGV_LINK	605.809	24.3615	0	125.244	OK	325.856	15.2831	0	72.8367	OK	OK	-0.67267

ENSG000001	-	-	ENSG0000013772	'ARHGAP20	-	11:110577041-110713189	-	-	IGV_LINK	16.4852	0.201081	0	0.453505	OK	17.5308	0.173129	0	0.375964	OK	NOTEST	-0.21593
ENSG000001	-	-	ENSG0000013773	'FXYD2	-	11:117427772-117877486	-	-	IGV_LINK	13993.8	1901.5	401.753	3401.24	OK	7355.08	990.466	302.737	1678.19	OK	OK	-0.94096
ENSG000001	-	-	ENSG0000013774	'MMP13	-	11:102942994-102955734	-	-	IGV_LINK	0	0	0	0	OK	3.09367	0.178207	0	0.48005	OK	NOTEST	inf
ENSG000001	-	-	ENSG0000013774	'TMPRSS13	-	11:117900642-117929459	-	-	IGV_LINK	52.3649	1.03863	0	2.16858	OK	38.1553	0.771048	0	1.66213	OK	OK	-0.42979
ENSG000001	-	-	ENSG0000013775	'CASP1	-	11:105025442-105035250	-	-	IGV_LINK	1498.22	72.6936	25.9779	119.409	OK	1488.06	72.6159	26.0895	119.142	OK	OK	-0.00154
ENSG000001	-	-	ENSG0000013775	'CASP5	-	11:104994234-105023168	-	-	IGV_LINK	10.6669	0.564883	0	1.43829	OK	31.968	2.21266	0	4.81558	OK	OK	1.96976
ENSG000001	-	-	ENSG0000013776	'ALKBH8	-	11:107502725-107565746	-	-	IGV_LINK	192.974	3.70409	0.282128	7.12605	OK	174.277	2.66684	0.559021	4.77465	OK	OK	-0.47399
ENSG000001	-	-	ENSG0000013776	'MAP2K5	-	15:67542708-67807123	-	-	IGV_LINK	323.887	9.56803	1.28781	17.8482	OK	309.367	8.58334	2.1347	15.032	OK	OK	-0.15668
ENSG000001	-	-	ENSG0000013776	'UNC13C	-	15:54012903-54633414	-	-	IGV_LINK	0.969721	0.004278	0	0.398061	OK	3.09367	0.310372	0	1.03864	OK	NOTEST	6.18081
ENSG000001	-	-	ENSG0000013776	'SQOR	-	15:45587122-45691294	-	-	IGV_LINK	2193.51	76.671	16.3355	137.006	OK	2048.01	71.1438	15.7237	126.564	OK	OK	-0.10794
ENSG000001	-	-	ENSG0000013777	'CTDSPL2	-	15:44427233-44536923	-	-	IGV_LINK	467.405	10.1808	0	22.236	OK	436.208	7.13008	0	15.3625	OK	OK	-0.51386
ENSG000001	-	-	ENSG0000013777	'SLTM	-	15:58771191-59097419	-	-	IGV_LINK	1985.02	53.0256	0.382596	105.669	OK	1930.45	52.9971	0	106.996	OK	OK	-0.00078
ENSG000001	-	-	ENSG0000013780	'THBS1	-	15:39581078-39599466	-	-	IGV_LINK	7799.46	87.8275	0	204.122	OK	2487.31	26.7308	0	57.2258	OK	OK	-1.71617
ENSG000001	-	-	ENSG0000013780	'MAPKBP1	-	15:41774433-41827936	-	-	IGV_LINK	600.257	7.48543	0	17.429	OK	593.986	8.20371	0	19.2351	OK	OK	0.13219
ENSG000001	-	-	ENSG0000013780	'NUSAP1	-	15:41332693-41381050	-	-	IGV_LINK	599.287	27.542	3.72529	51.3588	OK	1053.91	53.5271	9.12031	97.9338	OK	OK	0.958633
ENSG000001	-	-	ENSG0000013780	'NDUFAF1	-	15:41387348-41402519	-	-	IGV_LINK	200.732	10.9911	0.793909	21.1883	OK	308.336	18.2487	3.25844	33.239	OK	OK	0.73146
ENSG000001	-	-	ENSG0000013780	'KIF23	-	15:69298946-69448427	-	-	IGV_LINK	137.7	3.41362	0	9.47831	OK	128.903	3.44476	0	9.22623	OK	OK	0.0131
ENSG000001	-	-	ENSG0000013780	'AC027088.1	-	15:68778534-69062743	-	-	IGV_LINK	0.641933	0.038303	0	1.20756	OK	7.37E-75	4.40E-76	0	0.119483	OK	NOTEST	-245.622
ENSG000001	-	-	ENSG0000013780	'ITGA11	-	15:68296532-68432162	-	-	IGV_LINK	52.3649	0.568268	0	1.50868	OK	49.4988	0.498802	0	1.37511	OK	OK	-0.1881
ENSG000001	-	-	ENSG0000013781	'KNL1	-	15:40594019-40664342	-	-	IGV_LINK	119.276	1.20805	0	3.72428	OK	89.7166	1.62093	0	4.89745	OK	OK	0.424141
ENSG000001	-	-	ENSG0000013781	'HAUS2	-	15:42548809-42569994	-	-	IGV_LINK	226.915	6.34593	0	13.7075	OK	246.463	7.08961	0	15.6877	OK	OK	0.159874
ENSG000001	-	-	ENSG0000013781	'RTF1	-	15:41408407-41483563	-	-	IGV_LINK	823.293	10.324	1.85912	18.7889	OK	910.572	15.3893	0	36.6893	OK	OK	0.575926
ENSG000001	-	-	ENSG0000013781	'PARP6	-	15:72241180-72378788	-	-	IGV_LINK	2240.05	72.112	0	162.464	OK	1901.58	58.4543	0	144.16	OK	OK	-0.30293
ENSG000001	-	-	ENSG0000013781	'RPLP1	-	15:69452783-69456194	-	-	IGV_LINK	14420.7	1247.8	0	2515.37	OK	11603.3	949.686	65.0564	1834.32	OK	OK	-0.39387
ENSG000001	-	-	ENSG0000013781	'PAQR5	-	15:69298946-69448427	-	-	IGV_LINK	517.831	7.59863	0	15.5085	OK	244.4	3.61429	0	8.7384	OK	OK	-1.07203
ENSG000001	-	-	ENSG0000013782	'LRRC49	-	15:70829129-71053657	-	-	IGV_LINK	209.46	4.2401	0	9.38048	OK	143.34	3.77322	0	9.30418	OK	OK	-0.1683
ENSG000001	-	-	ENSG0000013782	'TUBGCP4	-	15:43358171-43510728	-	-	IGV_LINK	333.584	3.74728	0	11.1633	OK	360.929	4.92345	0	19.7479	OK	OK	0.393826
ENSG000001	-	-	ENSG0000013782	'RMDN3	-	15:40735883-40755851	-	-	IGV_LINK	811.656	25.1969	8.91082	41.483	OK	949.758	27.8736	10.7895	44.9576	OK	OK	0.14565
ENSG000001	-	-	ENSG0000013782	'ITPKA	-	15:41493392-41503551	-	-	IGV_LINK	6.78804	0.334846	0	0.910801	OK	37.1241	1.44144	0	3.16394	OK	OK	2.10594
ENSG000001	-	-	ENSG0000013783	'UACA	-	15:70654553-70763593	-	-	IGV_LINK	1715.44	27.145	2.49074	51.7992	OK	1818.05	33.1232	0.972721	65.2737	OK	OK	0.287156
ENSG000001	-	-	ENSG0000013783	'SMAD6	-	15:66702227-66782848	-	-	IGV_LINK	104.73	2.24755	0	5.56138	OK	90.7478	2.22033	0	5.70668	OK	OK	-0.01758
ENSG000001	-	-	ENSG0000013784	'PLCB2	-	15:40278175-40307935	-	-	IGV_LINK	2906.25	73.9192	5.1364	142.702	OK	2469.78	57.4724	0.940873	114.004	OK	OK	-0.36308
ENSG000001	-	-	ENSG0000013784	'TMEM62	-	15:43106224-43221283	-	-	IGV_LINK	427.647	13.4209	0	35.9105	OK	393.928	10.8178	0	30.1331	OK	OK	-0.31107
ENSG000001	-	-	ENSG0000013784	'PAK6	-	15:40161022-40277487	-	-	IGV_LINK	9.69721	0.314397	0	2.11044	OK	5.15612	0.142915	0	1.52457	OK	NOTEST	-1.13743
ENSG000001	-	-	ENSG0000013784	'ADAM10	-	15:58587506-58749978	-	-	IGV_LINK	1887.08	43.9131	8.37153	79.4547	OK	2528.56	57.3324	14.3919	100.273	OK	OK	0.384699
ENSG000001	-	-	ENSG0000013785	'DUOX1	-	15:45092649-45201175	-	-	IGV_LINK	886.325	13.9944	0	29.7872	OK	157.777	2.96528	0	7.28021	OK	OK	-2.2386
ENSG000001	-	-	ENSG0000013786	'SLC28A2	-	15:45251579-45279251	-	-	IGV_LINK	2.90916	0.039021	0	0.312989	OK	1.03122	0.013836	0	0.272411	OK	NOTEST	-1.49585
ENSG000001	-	-	ENSG0000013786	'STRA6	-	15:74179465-74336472	-	-	IGV_LINK	2.90916	0.53191	0	1.62065	OK	3.09367	0.084699	0	0.703695	OK	NOTEST	-2.65077
ENSG000001	-	-	ENSG0000013786	'CYP19A1	-	15:51037487-51338610	-	-	IGV_LINK	0	0	0	0	OK	0	0	0	0	OK	NOTEST	0
ENSG000001	-	-	ENSG0000013787	'ZNF280D	-	15:56630180-56918571	-	-	IGV_LINK	779.655	18.3898	2.92067	33.859	OK	607.392	17.7137	0	36.6796	OK	OK	-0.05404
ENSG000001	-	-	ENSG0000013787	'SEMA6D	-	15:47184100-47774223	-	-	IGV_LINK	75.6382	0.980863	0	2.50472	OK	59.811	0.888423	0	2.67901	OK	OK	-0.1428
ENSG000001	-	-	ENSG0000013787	'BCL2L10	-	15:52109262-52112775	-	-	IGV_LINK	19.3944	1.01534	0	2.15516	OK	8.2498	0.432355	0	1.02712	OK	OK	-1.23167
ENSG000001	-	-	ENSG0000013787	'RSL24D1	-	15:55180805-55197067	-	-	IGV_LINK	1095.78	33.5165	14.5709	52.4622	OK	890.978	27.6892	11.0767	44.3017	OK	OK	-0.27555
ENSG000001	-	-	ENSG0000013787	'SPTBN5	-	15:41828084-41972578	-	-	IGV_LINK	314.189	2.15198	0	8.94425	OK	229.963	1.96367	0	9.01009	OK	OK	-0.13211
ENSG000001	-	-	ENSG0000013787	'GCOM1	-	15:57591907-57782762	-	-	IGV_LINK	12.9431	0.821539	0	4.48858	OK	35.543	1.09868	0	4.33516	OK	OK	0.41937
ENSG000001	-	-	ENSG0000013788	'GCHFR	-	15:40764019-40815084	-	-	IGV_LINK	159.034	14.8494	0	39.279	OK	137.153	12.8875	0	34.3602	OK	OK	-0.20443
ENSG000001	-	-	ENSG0000013793	'BCAR3	-	1:93561785-93847150	-	-	IGV_LINK	331.644	7.70859	1.13014	14.287	OK	239.244	5.85653	0.035521	11.6775	OK	OK	-0.39642
ENSG000001	-	-	ENSG0000013794	'TTLL7	-	1:83865027-83999150	-	-	IGV_LINK	266.673	6.33731	0	14.6774	OK	236.151	5.15611	0	11.5095	OK	OK	-0.29759
ENSG000001	-	-	ENSG0000013794	'FNBP1L	-	1:93448130-93554661	-	-	IGV_LINK	1188.88	16.4729	3.31447	29.6313	OK	801.262	9.82803	3.57967	16.0764	OK	OK	-0.74512
ENSG000001	-	-	ENSG0000013794	'KYAT3	-	1:88935772-88992960	-	-	IGV_LINK	389.081	12.785	2.03897	23.531	OK	320.582	10.2091	1.50738	18.9108	OK	OK	-0.3246
ENSG000001	-	-	ENSG0000013794	'GTF2B	-	1:88852931-88891944	-	-	IGV_LINK	453.829	22.494	7.77048	37.2175	OK	454.77	21.7687	7.79196	35.7454	OK	OK	-0.04729
ENSG000001	-	-	ENSG0000013794	'BRDT	-	1:91949370-92014426	-	-	IGV_LINK	0	0	0	0	OK	0	0	0	0	OK	NOTEST	0
ENSG000001	-	-	ENSG0000013795	'RABGGTB	-	1:75724346-75795079	-	-	IGV_LINK	1541.86	70.9781	5.58819	136.368	OK	1333.37	60.65	5.24502	116.055	OK	OK	-0.22687
ENSG000001	-	-	ENSG0000013795	'IFI44L	-	1:78619921-78646145	-	-	IGV_LINK	4650.78	58.9828	7.51124	110.454	OK	2842.06	38.8002	6.24205	71.3583	OK	OK	-0.60423
ENSG000001	-	-	ENSG0000013796	'GIPC2	-	1:77979174-78138449	-	-	IGV_LINK	279.28	4.28331	0	8.66569	OK	328.961	5.04685	0.517965	9.57573	OK	OK	0.236658
ENSG000001	-	-	ENSG0000013796	'ARHGAP29	-	1:94148987-94334848	-	-	IGV_LINK	1995.68	19.4852	8.0493	30.9211	OK	1832.49	18.1117	7.53399	28.6894	OK	OK	-0.10546
ENSG000001	-	-	ENSG0000013796	'IFI44	-	1:78649795-78664078	-	-	IGV_LINK	2695.82	133.042	48.7785	217.306	OK	2494.53	129.003	42.9467	215.06	OK	OK	-0.04448
ENSG000001	-	-	ENSG0000013796	'SLC44A5	-	1:75202130-75611116	-	-	IGV_LINK	1.93944	0.029354	0	0.19994	OK	1.03122	0.015612	0	0.181446	OK	NOTEST	-0.91094
ENSG000001	-	-	ENSG0000013797	'RPL7P9	-	1:96678873-96679620	-	-	IGV_LINK	237.582	23.3066	6.87854	39.7347	OK	202.12	19.8676	5.02958	34.7057	OK	OK	-0.23032
ENSG000001	-	-	ENSG0000013797	'CLCA2	-	1:86424085-86456558	-	-	IGV_LINK	0.969721	0.014185	0	0.291495	OK	0	0	0	0	OK	NOTEST	'-inf
ENSG000001	-	-	ENSG0000013797	'DNASE2B	-	1:84398531-84415018	-	-	IGV_LINK	1.93944	0.114149	0	0.372616	OK	2.06245	0.121534	0	0.380052	OK	NOTEST	0.090444
ENSG000001	-	-	ENSG0000013799	'DBT	-	1:100186918-100249834	-	-	IGV_LINK	410.192	2.99591	0.09265	5.89917	OK	435.177	3.18728	0.150355	6.2242	OK	OK	0.089329
ENSG000001	-	-	ENSG0000013799	'RTCA	-	1:100266206-100292769	-	-	IGV_LINK	580.863	22.1739	6.73803	37.6098	OK	736.295	25.7132	9.10412	42.3222	OK	OK	0.213643
ENSG000001	-	-	ENSG0000013800	'IFT172	-	2:27442365-27489789	-	-	IGV_LINK	935.78	24.8983	0	52.7933	OK	984.82	17.088	0	37.5747	OK	OK	-0.54306
ENSG000001	-	-	ENSG0000013801	'SELENOI	-	2:26308172-26395891	-	-	IGV_LINK	490.679	3.48771	1.19636	5.77905	OK	536.237	4.03671	0	8.07613	OK	OK	0.2109
ENSG000001	-	-	ENSG0000013802	'CGREF1	-	2:27086746-27119115	-	-	IGV_LINK	526.558	23.6518	0	49.7352	OK	934.29	42.3856	0	112.856	OK	OK	0.841624
ENSG000001	-	-	ENSG0000013802	'HADHB	-	2:26190634-26290468	-	-	IGV_LINK	2866.49	87.5002	0	175.248	OK	3445.32	104.769	0	218.604	OK	OK	0.259859
ENSG000001	-	-	ENSG0000013803	'KHK	-	2:27086746-27119115	-	-	IGV_LINK	1222.82	50.0426	9.95248	90.1328	OK	5252.03	219.863	28.3527	411.372	OK	OK	2.13537
ENSG000001	-	-	ENSG0000013803	'ADCY3	-	2:24789733-24919839	-	-	IGV_LINK	1032.75	33.2493	3.79091	62.7076	OK	904.384	29.4541	1.22052	57.6876	OK	OK	-0.17486
ENSG000001	-	-	ENSG0000013803	'PPM1B	-	2:44167624-44244384	-	-	IGV_LINK	510.073	12.382	3.53183	21.2321	OK	448.583	12.3695	2.51304	22.2259	OK	OK	-0.00146
ENSG000001	-	-	ENSG0000013803	'PNPT1	-	2:55634264-55693910	-	-	IGV_LINK	359.766	5.77743	1.25225	10.3026	OK	457.864	8.3063	0.939778	15.6728	OK	OK	0.523777
ENSG000001	-	-	ENSG0000013803	'DYNC2LI1	-	2:43774038-43810010	-	-	IGV_LINK	982.327	50.5597	19.3892	81.7302	OK	654.828	33.1138	12.2779	53.9498	OK	OK	-0.61056
ENSG000001	-	-	ENSG0000013803	'LHCGR	-	2:48529382-48776517	-	-	IGV_LINK	0	0	0	0	OK	0	0	0	0	OK	NOTEST	0
ENSG000001	-	-	ENSG0000013805	'THUMPD2	-	2:39736059-39779267	-	-	IGV_LINK	185.217	7.21662	0.284058	14.1492	OK	193.87	5.76278	1.03641	10.4892	OK	OK	-0.32456
ENSG000001	-	-	ENSG0000013806	'CYP1B1	-	2:37923186-38239590	-	-	IGV_LINK	108.609	1.46341	0	3.3975	OK	1095.16	16.8545	2.8467	30.8624	OK	OK	3.52574
ENSG000001	-	-	ENSG0000013806	'SULT6B1	-	2:37167819-37324808	-	-	IGV_LINK	0	0	0	0	OK	0	0	0	0	OK	NOTEST	0
ENSG000001	-	-	ENSG0000013806	'RAB1A	-	2:65056365-65130106	-	-	IGV_LINK	1368.28	44.3721	13.3017	75.4424	OK	2137.73	72.2186	19.1176	125.32	OK	OK	0.702718
ENSG000001	-	-	ENSG0000013807	'ACTR2	-	2:65227752-65271253	-	-	IGV_LINK	3045.89	63.7828	22.303	105.263	OK	3408.2	75.1405	18.7197	131.561	OK	OK	0.236423
ENSG000001	-	-	ENSG0000013807	'PREB	-	2:27123788-27134675	-	-	IGV_LINK	919.295	26.0866	10.1846	41.9885	OK	1120.94	36.0457	12.3231	59.7684	OK	OK	0.466521
ENSG000001	-	-	ENSG0000013807	'SLC5A6	-	2:27199586-27217178	-	-	IGV_LINK	457.708	12.5149	0	35.0099	OK	618.735	18.0083	0	50.4341	OK	OK	0.525015
ENSG000001	-	-	ENSG0000013807	'ABCG5	-	2:43812471-43838865	-	-	IGV_LINK	5.81832	0.114629	0	0.29246	OK	0	0	0	0	OK	NOTEST	'-inf
ENSG000001	-	-	ENSG0000013807	'PREPL	-	2:44275457-44361862	-	-	IGV_LINK	4448.11	196.566	0	575.525	OK	2819.37	107.255	0	298.402	OK	OK	-0.87397
ENSG000001	-	-	ENSG0000013807	'SLC3A1	-	2:44275457-44361862	-	-	IGV_LINK	8792.46	305.52	37.7172	573.322	OK	5134.47	181.035	35.415	326.655	OK	OK	-0.755
ENSG000001	-	-	ENSG0000013808	'EMILIN1	-	2:27078566-27086408	-	-	IGV_LINK	284.128	4.62812	1.19023	8.06601	OK	147.465	2.21531	0.354452	4.07617	OK	OK	-1.06292
ENSG000001	-	-	ENSG0000013808	'FBXO11	-	2:47695529-47907810	-	-	IGV_LINK	903.78	18.5593	0	38.8442	OK	683.702	14.0081	0	29.7233	OK	OK	-0.40588
ENSG000001	-	-	ENSG0000013808	'SIX3	-	2:44941897-44946077	-	-	IGV_LINK	0	0	0	0	OK	0	0	0	0	OK	NOTEST	0
ENSG000001	-	-	ENSG0000013808	'ATRAID	-	2:27199586-27217178	-	-	IGV_LINK	1998.59	138.677	50.7718	226.582	OK	2352.22	163.353	51.6018	275.103	OK	OK	0.236259
ENSG000001	-	-	ENSG0000013809	'CENPO	-	2:24789733-24919839	-	-	IGV_LINK	169.701	2.76906	0	8.32149	OK	211.401	4.78897	0	16.1378	OK	OK	0.790319
ENSG000001	-	-	ENSG0000013809	'LRPPRC	-	2:43886507-43996005	-	-	IGV_LINK	2402	24.2409	5.75107	42.7307	OK	1993.36	19.2939	6.09823	32.4896	OK	OK	-0.3293
ENSG000001	-	-	ENSG0000013810	'TRIM54	-	2:27282391-27308445	-	-	IGV_LINK	47.5163	1.56442	0	3.44771	OK	788.887	25.1116	10.0781	40.1451	OK	OK	4.00466
ENSG000001	-	-	ENSG0000013810	'DTNB	-	2:25377197-25673647	-	-	IGV_LINK	222.066	6.83997	1.05038	12.6295	OK	171.183	6.7136	0	13.5359	OK	OK	-0.0269
ENSG000001	-	-	ENSG0000013810	'ACTR1A	-	10:102461394-102502711	-	-	IGV_LINK	1399.31	30.0578	11.1692	48.9465	OK	1245.72	27.4169	9.42056	45.4132	OK	OK	-0.13268
ENSG000001	-	-	ENSG0000013810	'CYP2C9	-	10:94938657-94989390	-	-	IGV_LINK	8.72748	0.293737	0	0.775168	OK	483.645	16.3309	5.37464	27.2871	OK	OK	5.79694
ENSG000001	-	-	ENSG0000013811	'MFSD13A	-	10:102461394-102502711	-	-	IGV_LINK	128.003	4.50352	0	19.5659	OK	138.184	4.32185	0	17.0218	OK	OK	-0.0594
ENSG000001	-	-	ENSG0000013811	'CYP2C8	-	10:95036771-95069497	-	-	IGV_LINK	154.186	6.2468	0.129953	12.3636	OK	128.903	4.33912	0.175601	8.50265	OK	OK	-0.52571
ENSG000001	-	-	ENSG0000013811	'MYOF	-	10:93306428-93482317	-	-	IGV_LINK	4508.23	51.6264	0	104.279	OK	2436.78	26.5573	2.56659	50.5481	OK	OK	-0.959
ENSG000001	-	-	ENSG0000013813	'LOXL4	-	10:98247689-98268250	-	-	IGV_LINK	373.342	6.04511	2.12347	9.96675	OK	329.992	5.34495	1.79138	8.89853	OK	OK	-0.17759
ENSG000001	-	-	ENSG0000013813	'STAMBPL1	-	10:88879733-89015785	-	-	IGV_LINK	722.442	19.5713	0	41.1875	OK	247.494	6.89625	0	19.2037	OK	OK	-1.50485
ENSG000001	-	-	ENSG0000013813	'CH25H	-	10:89205628-89207314	-	-	IGV_LINK	8.72748	0.324975	0	0.739615	OK	13.4059	0.49956	0	1.11903	OK	NOTEST	0.62033
ENSG000001	-	-	ENSG0000013813	'LBX1	-	10:101226194-101270148	-	-	IGV_LINK	0	0	0	0	OK	0	0	0	0	OK	NOTEST	0
ENSG000001	-	-	ENSG0000013813	'ATAD1	-	10:87751511-87845612	-	-	IGV_LINK	646.804	11.3826	4.33975	18.4255	OK	360.929	7.26637	1.23644	13.2963	OK	OK	-0.64753
ENSG000001	-	-	ENSG0000013815	'BTBD16	-	10:122271305-122338162	-	-	IGV_LINK	33.9402	2.07952	0	4.77615	OK	96.9351	5.73369	0	12.0076	OK	OK	1.46321
ENSG000001	-	-	ENSG0000013816	'KIF11	-	10:92593285-92655395	-	-	IGV_LINK	190.065	2.28507	0.515566	4.05457	OK	108.279	1.3021	0.117393	2.48681	OK	OK	-0.81139
ENSG000001	-	-	ENSG0000013816	'CUZD1	-	10:122832148-122879641	-	-	IGV_LINK	62.0278	1.37145	0	3.10015	OK	23.2152	0.504115	0	1.40059	OK	OK	-1.44387
ENSG000001	-	-	ENSG0000013816	'TACC2	-	10:121989173-122254545	-	-	IGV_LINK	706.926	15.118	1.11912	29.1168	OK	234.088	5.79221	0	12.1682	OK	OK	-1.38408
ENSG000001	-	-	ENSG0000013816	'DUSP5	-	10:110497837-110511544	-	-	IGV_LINK	215.278	5.08795	1.41113	8.76477	OK	270.181	6.38862	2.00557	10.7717	OK	OK	0.328421
ENSG000001	-	-	ENSG0000013817	'CALHM2	-	10:103446785-103452402	-	-	IGV_LINK	398.555	16.1658	4.93125	27.4003	OK	334.117	13.9366	3.79143	24.0817	OK	OK	-0.21407
ENSG000001	-	-	ENSG0000013817	'ARL3	-	10:102673730-102714407	-	-	IGV_LINK	483.891	7.42749	2.83209	12.0229	OK	223.776	3.43593	0.980003	5.89185	OK	OK	-1.11217
ENSG000001	-	-	ENSG0000013818	'CEP55	-	10:93496631-93529092	-	-	IGV_LINK	125.094	3.20316	0.165777	6.24054	OK	102.091	2.50854	0.055948	4.96113	OK	OK	-0.35265
ENSG000001	-	-	ENSG0000013818	'KIF20B	-	10:89701609-89774939	-	-	IGV_LINK	172.61	1.74645	0.303778	3.18911	OK	112.404	1.07004	0.133064	2.00702	OK	OK	-0.70675
ENSG000001	-	-	ENSG0000013818	'ENTPD1	-	10:95711778-96090238	-	-	IGV_LINK	1716.29	32.3827	6.43032	58.3352	OK	1384.71	31.3304	4.04732	58.6134	OK	OK	-0.04766
ENSG000001	-	-	ENSG0000013819	'EXOC6	-	10:92834712-93059493	-	-	IGV_LINK	158.064	11.1339	0	39.8361	OK	196.964	7.42486	0	28.3004	OK	OK	-0.58453
ENSG000001	-	-	ENSG0000013819	'PLCE1	-	10:94030811-94332823	-	-	IGV_LINK	405.343	3.58322	0	7.48906	OK	163.965	1.20582	0.159039	2.2526	OK	OK	-1.57124
ENSG000001	-	-	ENSG0000013820	'RBP4	-	10:93566664-93604480	-	-	IGV_LINK	1.93944	0.170369	0	1.08047	OK	0	0	0	0	OK	NOTEST	'-inf
ENSG000001	-	-	ENSG0000013823	'DBR1	-	3:138161011-138174949	-	-	IGV_LINK	287.037	6.8671	1.62213	12.1121	OK	287.712	6.7777	1.7549	11.8005	OK	OK	-0.01891
ENSG000001	-	-	ENSG0000013824	'DNAJC13	-	3:132417525-132539032	-	-	IGV_LINK	1286.82	10.9025	0.936366	20.8687	OK	1172.5	9.16416	2.82765	15.5007	OK	OK	-0.25059
ENSG000001	-	-	ENSG0000013827	'GPR87	-	3:151085696-151458709	-	-	IGV_LINK	0	0	0	0	OK	0	0	0	0	OK	NOTEST	0
ENSG000001	-	-	ENSG0000013827	'ANXA7	-	10:73375100-73414076	-	-	IGV_LINK	2228.42	86.019	23.2606	148.777	OK	1634.49	67.5129	23.1765	111.849	OK	OK	-0.34949
ENSG000001	-	-	ENSG0000013828	'FAM149B1	-	10:73110374-73358859	-	-	IGV_LINK	493.588	14.6924	0	38.6904	OK	307.305	8.178	0	21.1185	OK	OK	-0.84525
ENSG000001	-	-	ENSG0000013830	'ASCC1	-	10:72096031-72235860	-	-	IGV_LINK	518.8	21.2906	0	55.6992	OK	393.928	15.7739	0	36.0242	OK	OK	-0.43268
ENSG000001	-	-	ENSG0000013830	'PLA2G12B	-	10:72935169-72954778	-	-	IGV_LINK	37.8191	2.23063	0	4.53016	OK	188.714	11.1441	2.84219	19.446	OK	OK	2.32075
ENSG000001	-	-	ENSG0000013831	'ZNF365	-	10:62374191-62672011	-	-	IGV_LINK	4.8486	0.070913	0	0.571757	OK	42.2802	0.853698	0	2.5377	OK	OK	3.58961
ENSG000001	-	-	ENSG0000013831	'OIT3	-	10:72893580-72933033	-	-	IGV_LINK	5.81832	0.149554	0	0.369965	OK	43.3114	1.11386	0	2.24777	OK	OK	2.89683
ENSG000001	-	-	ENSG0000013831	'ADAMTS14	-	10:70672802-70762441	-	-	IGV_LINK	36.8494	0.408124	0	0.869913	OK	79.4043	0.879715	0.071139	1.68829	OK	OK	1.10803
ENSG000001	-	-	ENSG0000013832	'RPS24	-	10:78033759-78056812	-	-	IGV_LINK	16968.2	2777.22	524.89	5029.55	OK	8208.55	1350.32	426.312	2274.32	OK	OK	-1.04034
ENSG000001	-	-	ENSG0000013833	'TET1	-	10:68560655-68694482	-	-	IGV_LINK	32.0008	0.197841	0	0.405481	OK	4.1249	0.025505	0	0.064611	OK	NOTEST	-2.95549
ENSG000001	-	-	ENSG0000013834	'DNA2	-	10:68414063-68472121	-	-	IGV_LINK	126.064	2.21193	0.150061	4.2738	OK	79.4043	1.75705	0	4.18365	OK	OK	-0.33215
ENSG000001	-	-	ENSG0000013834	'MYPN	-	10:68106116-68212017	-	-	IGV_LINK	1.93944	0.024708	0	0.105529	OK	0	0	0	0	OK	NOTEST	'-inf
ENSG000001	-	-	ENSG0000013835	'AOX1	-	2:200585867-200677064	-	-	IGV_LINK	146.428	3.24273	0	7.55811	OK	1251.91	18.6282	4.05417	33.2022	OK	OK	2.52221
ENSG000001	-	-	ENSG0000013836	'ATIC	-	2:215311816-215349773	-	-	IGV_LINK	933.841	41.6513	7.9698	75.3328	OK	986.882	39.0854	10.5958	67.575	OK	OK	-0.09173
ENSG000001	-	-	ENSG0000013837	'SMARCAL1	-	2:216385287-216579180	-	-	IGV_LINK	432.495	9.21133	0	70.8662	OK	558.924	12.4017	0	62.0159	OK	OK	0.429056
ENSG000001	-	-	ENSG0000013837	'BARD1	-	2:214725645-214809711	-	-	IGV_LINK	88.2446	1.27993	0	3.00064	OK	59.811	2.50096	0	17.7922	OK	OK	0.966414

ENSG0000013	-	-	ENSG0000013837	'STAT4	-	2:191021525-191151596	-	-	IGV_LINK	270.552	7.05701	0.825277	13.2888	OK	303.18	7.65466	0.790449	14.5189	OK	OK	0.11728
ENSG0000013	-	-	ENSG0000013837	'MSTN	-	2:189879608-190371665	-	-	IGV_LINK	32.9705	0.701296	0	2.72992	OK	23.7182	0.504714	0	2.30935	OK	OK	-0.47456
ENSG0000013	-	-	ENSG0000013838	'CARF	-	2:202874781-203014798	-	-	IGV_LINK	371.403	8.74809	0	19.0944	OK	241.307	6.38583	0	16.1019	OK	OK	-0.4541
ENSG0000013	-	-	ENSG0000013838	'ASNSD1	-	2:189661384-189670831	-	-	IGV_LINK	453.829	12.9181	3.0095	22.8267	OK	485.707	12.9678	4.0602	21.8753	OK	OK	0.005536
ENSG0000013	-	-	ENSG0000013838	'METTL5	-	2:169791932-169824931	-	-	IGV_LINK	346.19	27.4359	0	58.2067	OK	302.149	24.1836	0	54.0737	OK	OK	-0.18204
ENSG0000013	-	-	ENSG0000013838	'SSB	-	2:169791932-169824931	-	-	IGV_LINK	1019.18	39.3388	12.7046	65.973	OK	1017.82	39.8236	11.8407	67.8064	OK	OK	0.017669
ENSG0000013	-	-	ENSG0000013838	'NAB1	-	2:190607659-190708716	-	-	IGV_LINK	941.599	21.4219	3.29092	39.5529	OK	658.953	13.5515	3.44621	23.6569	OK	OK	-0.66063
ENSG0000013	-	-	ENSG0000013839	'CDK15	-	2:201790460-201895550	-	-	IGV_LINK	6.78804	0.148434	0	0.597148	OK	2.06245	0.033348	0	0.415381	OK	NOTEST	-2.15413
ENSG0000013	-	-	ENSG0000013839	'PPIG	-	2:169584339-169641406	-	-	IGV_LINK	939.659	22.1743	6.42202	37.9266	OK	921.915	23.8102	6.54848	41.0719	OK	OK	0.102691
ENSG0000013	-	-	ENSG0000013839	'FASTKD1	-	2:169529748-169573875	-	-	IGV_LINK	377.221	16.9764	0.387426	33.5653	OK	278.431	12.715	0	26.0433	OK	OK	-0.41699
ENSG0000013	-	-	ENSG0000013840	'MDH1B	-	2:206737762-206792509	-	-	IGV_LINK	178.429	6.26436	0	13.7849	OK	156.746	5.27677	0	12.0441	OK	OK	-0.24751
ENSG0000013	-	-	ENSG0000013841	'HECW2	-	2:196194369-196593692	-	-	IGV_LINK	232.733	1.54905	0	3.17894	OK	371.241	2.27217	0.255514	4.28882	OK	OK	0.552684
ENSG0000013	-	-	ENSG0000013841	'IDH1	-	2:208236226-208266074	-	-	IGV_LINK	751.533	21.2849	7.87916	34.6907	OK	2874.02	79.1581	31.9204	126.396	OK	OK	1.89491
ENSG0000013	-	-	ENSG0000013843	'OLA1	-	2:174072446-174248698	-	-	IGV_LINK	1006.57	47.5213	12.9568	82.0859	OK	1163.22	56.2765	19.8462	92.7069	OK	OK	0.243957
ENSG0000013	-	-	ENSG0000013843	'CIR1	-	2:174348021-174395715	-	-	IGV_LINK	849.475	30.7127	9.59099	51.8343	OK	783.731	27.0665	9.31532	44.8177	OK	OK	-0.18232
ENSG0000013	-	-	ENSG0000013843	'SSFA2	-	2:181891832-181930738	-	-	IGV_LINK	2302.12	44.8725	3.93922	85.8058	OK	2288.29	47.033	0	108.083	OK	OK	0.06784
ENSG0000013	-	-	ENSG0000013843	'CHRNA1	-	2:174547140-174787935	-	-	IGV_LINK	6.78804	0.212295	0	2.65036	OK	2.06245	0.089939	0	1.89764	OK	NOTEST	-1.23906
ENSG0000013	-	-	ENSG0000013843	'FAM117B	-	2:202635187-202769757	-	-	IGV_LINK	219.157	2.46851	0.104107	4.83291	OK	142.309	1.77015	0	3.90223	OK	OK	-0.47976
ENSG0000013	-	-	ENSG0000013844	'WDR12	-	2:202874781-203014798	-	-	IGV_LINK	422.798	20.0479	0	50.7203	OK	490.863	16.9529	0	41.6497	OK	OK	-0.24192
ENSG0000013	-	-	ENSG0000013844	'ABI2	-	2:203328218-203535410	-	-	IGV_LINK	907.658	13.4236	0	30.4254	OK	712.576	13.7763	0	29.4319	OK	OK	0.037421
ENSG0000013	-	-	ENSG0000013844	'ITGAV	-	2:186590064-186765965	-	-	IGV_LINK	3798.4	41.5083	7.88928	75.1274	OK	5352.06	51.7299	15.3503	88.1095	OK	OK	0.317597
ENSG0000013	-	-	ENSG0000013844	'SLC40A1	-	2:189560578-189583758	-	-	IGV_LINK	2262.36	39.4142	16.7906	62.0378	OK	4211.52	72.7801	30.2935	115.267	OK	OK	0.884828
ENSG0000013	-	-	ENSG0000013845	'SLC35A5	-	3:112532508-112585577	-	-	IGV_LINK	520.74	18.7976	0	45.1638	OK	590.892	22.1294	0	46.5794	OK	OK	0.235418
ENSG0000013	-	-	ENSG0000013846	'DIRC2	-	3:122794794-122881139	-	-	IGV_LINK	177.459	4.47091	0.585141	8.35668	OK	267.087	7.18985	1.87599	12.5037	OK	OK	0.685393
ENSG0000013	-	-	ENSG0000013846	'SENP7	-	3:101324204-101513241	-	-	IGV_LINK	533.346	11.0869	2.73578	19.4381	OK	312.461	6.28872	0.600448	11.977	OK	OK	-0.81803
ENSG0000013	-	-	ENSG0000013847	'GUCA1C	-	3:108907791-108953895	-	-	IGV_LINK	0	0	0	0	OK	0	0	0	0	OK	NOTEST	0
ENSG0000013	-	-	ENSG0000013848	'CCDC54	-	3:107240691-107421304	-	-	IGV_LINK	0	0	0	0	OK	0	0	0	0	OK	NOTEST	0
ENSG0000013	-	-	ENSG0000013849	'COX17	-	3:119636456-119677454	-	-	IGV_LINK	452.859	52.4161	16.052	88.7802	OK	400.29	46.5381	14.08	78.9962	OK	OK	-0.1716
ENSG0000013	-	-	ENSG0000013849	'PARP9	-	3:122527923-122575203	-	-	IGV_LINK	1883.2	34.6651	2.08934	67.2408	OK	2127.42	39.9756	4.65717	75.294	OK	OK	0.205635
ENSG0000013	-	-	ENSG0000013858	'MNS1	-	15:56244008-56465137	-	-	IGV_LINK	192.974	6.22656	0	12.9023	OK	139.215	4.61202	0	9.96954	OK	OK	-0.43304
ENSG0000013	-	-	ENSG0000013859	'USP8	-	15:50424379-50546708	-	-	IGV_LINK	996.873	18.7387	2.0107	35.4668	OK	1182.82	22.9102	4.02607	41.7944	OK	OK	0.289968
ENSG0000013	-	-	ENSG0000013859	'SECISBP2L	-	15:48988475-49046563	-	-	IGV_LINK	1187.91	11.823	0	25.6074	OK	1366.37	11.9166	4.27451	19.5586	OK	OK	0.011378
ENSG0000013	-	-	ENSG0000013859	'TMOD3	-	15:51829627-52100523	-	-	IGV_LINK	850.445	9.89052	0	23.8188	OK	1136.41	14.2208	0	39.1902	OK	OK	0.523882
ENSG0000013	-	-	ENSG0000013860	'SPPL2A	-	15:50702265-50765808	-	-	IGV_LINK	747.655	27.4725	1.52786	53.4171	OK	982.757	42.3167	6.64854	77.9848	OK	OK	0.62324
ENSG0000013	-	-	ENSG0000013860	'GLCE	-	15:69160583-69272217	-	-	IGV_LINK	398.555	6.07741	1.30984	10.845	OK	554.799	8.66589	2.05257	15.2792	OK	OK	0.51189
ENSG0000013	-	-	ENSG0000013860	'SHF	-	15:45092649-45201175	-	-	IGV_LINK	64.9713	2.06497	0	7.09139	OK	43.1286	2.39347	0	7.00529	OK	OK	0.212982
ENSG0000013	-	-	ENSG0000013861	'APH1B	-	15:63276017-63309126	-	-	IGV_LINK	389.828	6.86814	1.58176	12.1545	OK	491.894	12.1809	0	24.4382	OK	OK	0.826624
ENSG0000013	-	-	ENSG0000013861	'INTS14	-	15:65578752-65611289	-	-	IGV_LINK	606.075	17.9899	4.82404	31.1558	OK	685.765	18.2209	6.22918	30.2125	OK	OK	0.018401
ENSG0000013	-	-	ENSG0000013861	'CILP	-	15:65194757-65211488	-	-	IGV_LINK	32.0008	0.326559	0	0.669292	OK	17.5308	0.178935	0	0.398234	OK	NOTEST	-0.86791
ENSG0000013	-	-	ENSG0000013861	'PARP16	-	15:65234459-65300618	-	-	IGV_LINK	321.947	9.46879	1.50189	17.4357	OK	299.055	7.60308	1.6339	13.5723	OK	OK	-0.3166
ENSG0000013	-	-	ENSG0000013862	'PPCDC	-	15:75023554-75117462	-	-	IGV_LINK	297.704	19.4158	3.40682	35.4248	OK	303.18	19.5933	4.37063	34.8159	OK	OK	0.013126
ENSG0000013	-	-	ENSG0000013862	'HCN4	-	15:73319858-73369264	-	-	IGV_LINK	5.81832	0.046474	0	0.110373	OK	7.21857	0.057668	0	0.134373	OK	NOTEST	0.311346
ENSG0000013	-	-	ENSG0000013862	'SEMA7A	-	15:74409288-74434467	-	-	IGV_LINK	188.126	5.32442	0	11.8721	OK	163.965	4.37153	0	9.80775	OK	OK	-0.28449
ENSG0000013	-	-	ENSG0000013862	'UBL7	-	15:74445976-74461182	-	-	IGV_LINK	663.289	33.0841	11.0917	55.0766	OK	645.547	32.8057	11	54.6114	OK	OK	-0.01219
ENSG0000013	-	-	ENSG0000013863	'ARHGAP24	-	4:85475113-86815171	-	-	IGV_LINK	1226.7	36.6493	0	74.9261	OK	944.602	28.5049	0	58.0667	OK	OK	-0.36258
ENSG0000013	-	-	ENSG0000013864	'FAM13A	-	4:88709788-89111398	-	-	IGV_LINK	3318.38	56.4095	1.13051	111.688	OK	4718.89	74.2055	21.4587	126.952	OK	OK	0.395588
ENSG0000013	-	-	ENSG0000013864	'HERC3	-	4:88520984-88708542	-	-	IGV_LINK	1225.73	38.3749	5.50382	71.2459	OK	1411.75	40.2896	6.74078	73.8384	OK	OK	0.070246
ENSG0000013	-	-	ENSG0000013864	'HERC6	-	4:88378738-88443111	-	-	IGV_LINK	428.616	8.4141	1.05477	15.7734	OK	739.388	13.0434	3.45876	22.6281	OK	OK	0.632442
ENSG0000013	-	-	ENSG0000013864	'HERC5	-	4:88457116-88506163	-	-	IGV_LINK	306.432	5.8116	1.4344	10.1888	OK	501.175	9.28923	3.32958	15.2489	OK	OK	0.676623
ENSG0000013	-	-	ENSG0000013865	'PCDH10	-	4:133149314-133208606	-	-	IGV_LINK	118.306	1.05399	0	2.21491	OK	100.029	1.20217	0	2.72323	OK	OK	0.189782
ENSG0000013	-	-	ENSG0000013865	'NDST4	-	4:114827762-115113876	-	-	IGV_LINK	0	0	0	0	OK	0	0	0	0	OK	NOTEST	0
ENSG0000013	-	-	ENSG0000013865	'ZGRF1	-	4:112513515-112657592	-	-	IGV_LINK	146.428	2.83173	0	7.82738	OK	83.5292	1.3326	0	4.03199	OK	OK	-1.08744
ENSG0000013	-	-	ENSG0000013866	'AP1AR	-	4:112231736-112270047	-	-	IGV_LINK	411.161	9.17059	3.0016	15.3396	OK	419.709	10.1031	1.28965	18.9165	OK	OK	0.139707
ENSG0000013	-	-	ENSG0000013866	'COPS4	-	4:83034446-83075818	-	-	IGV_LINK	488.739	20.9608	5.58512	36.3365	OK	435.177	18.9008	4.67373	33.1278	OK	OK	-0.14925
ENSG0000013	-	-	ENSG0000013866	'HNRNPD	-	4:82352497-82384027	-	-	IGV_LINK	3193.29	172.133	58.6561	285.61	OK	2952.4	160.128	51.0869	269.169	OK	OK	-0.1043
ENSG0000013	-	-	ENSG0000013866	'PRKG2	-	4:81087369-81215117	-	-	IGV_LINK	192.974	4.57233	0	9.54503	OK	128.903	3.56076	0	7.73741	OK	OK	-0.36075
ENSG0000013	-	-	ENSG0000013867	'RASGEF1B	-	4:81426392-82044244	-	-	IGV_LINK	287.037	6.80136	1.30087	12.3019	OK	170.152	3.95533	0.760563	7.1501	OK	OK	-0.78203
ENSG0000013	-	-	ENSG0000013867	'SEC31A	-	4:82818660-83012926	-	-	IGV_LINK	3190	75.1737	5.67398	144.673	OK	4231.46	115.76	0	233.618	OK	OK	0.622838
ENSG0000013	-	-	ENSG0000013867	'FGF5	-	4:80266598-80963756	-	-	IGV_LINK	2.90916	0.257519	0	1.30858	OK	38.3218	0.849199	0	3.07664	OK	OK	1.72142
ENSG0000013	-	-	ENSG0000013867	'GPAT3	-	4:83535913-83605875	-	-	IGV_LINK	151.276	3.47233	0.486863	6.45779	OK	161.902	3.7163	0.590721	6.84188	OK	OK	0.097965
ENSG0000013	-	-	ENSG0000013868	'IL21	-	4:122612627-122689156	-	-	IGV_LINK	0	0	0	0	OK	0	0	0	0	OK	NOTEST	0
ENSG0000013	-	-	ENSG0000013868	'FGF2	-	4:122826707-122922968	-	-	IGV_LINK	179.398	2.41815	0	6.12842	OK	71.1545	1.07247	0	3.30805	OK	OK	-1.17297
ENSG0000013	-	-	ENSG0000013868	'BBS7	-	4:121824439-121870497	-	-	IGV_LINK	270.552	6.66612	0.764396	12.5678	OK	249.556	6.921	0.672186	13.1698	OK	OK	0.054135
ENSG0000013	-	-	ENSG0000013868	'KIAA1109	-	4:122152332-122362758	-	-	IGV_LINK	2343.81	26.045	8.79388	43.2962	OK	1496.31	18.4558	5.2679	31.6437	OK	OK	-0.49693
ENSG0000013	-	-	ENSG0000013869	'BMPR1B	-	4:94757967-95158448	-	-	IGV_LINK	29.0916	0.703463	0	1.81011	OK	13.4059	0.256677	0	0.710217	OK	OK	-1.45452
ENSG0000013	-	-	ENSG0000013869	'RAP1GDS1	-	4:98251687-98443861	-	-	IGV_LINK	500.376	13.0957	1.80141	24.3899	OK	413.521	11.1286	0.862996	21.3941	OK	OK	-0.23482
ENSG0000013	-	-	ENSG0000013870	'LARP1B	-	4:128061267-128222931	-	-	IGV_LINK	229.824	5.21018	1.02749	9.39287	OK	269.15	6.84298	1.59594	12.09	OK	OK	0.393292
ENSG0000013	-	-	ENSG0000013872	'MMRN1	-	4:89879531-89954629	-	-	IGV_LINK	26.1825	0.428627	0	0.934373	OK	209.339	2.4941	0.491753	4.49644	OK	OK	2.54072
ENSG0000013	-	-	ENSG0000013873	'PDE5A	-	4:119454790-119628991	-	-	IGV_LINK	158.064	3.10106	0	10.2305	OK	111.372	1.82851	0	5.69682	OK	OK	-0.7621
ENSG0000013	-	-	ENSG0000013873	'PRDM5	-	4:120684918-120922870	-	-	IGV_LINK	81.4565	1.27843	0	2.77113	OK	42.2802	0.766685	0	2.00753	OK	OK	-0.73767
ENSG0000013	-	-	ENSG0000013874	'TRPC3	-	4:121879026-121951754	-	-	IGV_LINK	8.72748	0.157726	0	0.450729	OK	6.18735	0.103332	0	0.347007	OK	NOTEST	-0.61013
ENSG0000013	-	-	ENSG0000013874	'NAAA	-	4:75913656-76007488	-	-	IGV_LINK	613.833	29.2628	0	97.1011	OK	642.453	31.3233	0	64.1472	OK	OK	0.098169
ENSG0000013	-	-	ENSG0000013875	'NUP54	-	4:76114658-76148515	-	-	IGV_LINK	445.102	19.0573	4.02876	34.0859	OK	403.209	16.8523	2.48289	31.2217	OK	OK	-0.1774
ENSG0000013	-	-	ENSG0000013875	'CXCL9	-	4:75913656-76007488	-	-	IGV_LINK	5144.37	112.917	36.3961	189.439	OK	509.425	11.1868	0	22.5746	OK	OK	-3.33541
ENSG0000013	-	-	ENSG0000013875	'BMP2K	-	4:78776341-78939438	-	-	IGV_LINK	286.068	7.35445	0	20.056	OK	358.866	10.5673	0	28.358	OK	OK	0.522913
ENSG0000013	-	-	ENSG0000013875	'G3BP2	-	4:75642781-75724525	-	-	IGV_LINK	1382.82	29.5701	6.76762	52.3725	OK	1740.71	34.586	9.64223	59.5299	OK	OK	0.226052
ENSG0000013	-	-	ENSG0000013875	'SEPT11	-	4:76949702-77040384	-	-	IGV_LINK	1640.77	21.4153	6.53569	36.295	OK	1513.84	19.6648	3.58849	35.7411	OK	OK	-0.12303
ENSG0000013	-	-	ENSG0000013875	'FRAS1	-	4:78057569-78544269	-	-	IGV_LINK	229.824	0.838224	0.165581	1.51087	OK	75.2794	0.274583	0	0.619556	OK	OK	-1.61009
ENSG0000013	-	-	ENSG0000013876	'SCARB2	-	4:76158732-76311599	-	-	IGV_LINK	3778.03	69.4493	1.72603	137.173	OK	4851.91	99.1896	0	198.423	OK	OK	0.514229
ENSG0000013	-	-	ENSG0000013876	'CCNG2	-	4:77157150-77494286	-	-	IGV_LINK	1818.23	47.756	14.7184	80.7936	OK	1271.5	35.0668	10.423	59.7107	OK	OK	-0.44557
ENSG0000013	-	-	ENSG0000013876	'CNOT6L	-	4:77713386-77819615	-	-	IGV_LINK	803.898	9.92751	0.433555	19.4215	OK	635.235	9.4071	0.080585	18.7336	OK	OK	-0.07768
ENSG0000013	-	-	ENSG0000013876	'USO1	-	4:75724592-75814286	-	-	IGV_LINK	1677.62	24.9095	8.49889	41.3202	OK	1555.09	23.2297	7.29093	39.1684	OK	OK	-0.10073
ENSG0000013	-	-	ENSG0000013876	'CDKL2	-	4:75578004-75630716	-	-	IGV_LINK	93.0932	1.70214	0.052876	3.3514	OK	68.0608	1.30119	0	2.66341	OK	OK	-0.38752
ENSG0000013	-	-	ENSG0000013877	'SHROOM3	-	4:76435099-76801964	-	-	IGV_LINK	289.946	1.65687	0.279941	3.0338	OK	553.768	3.15809	1.02118	5.295	OK	OK	0.930592
ENSG0000013	-	-	ENSG0000013877	'ANXA3	-	4:78551518-78610451	-	-	IGV_LINK	4.8486	0.336481	0	1.24341	OK	14.4371	0.569453	0	1.50727	OK	OK	0.759052
ENSG0000013	-	-	ENSG0000013877	'PPA2	-	4:105369076-105474081	-	-	IGV_LINK	460.617	33.1247	10.72	55.5294	OK	517.675	38.946	13.2032	64.6889	OK	OK	0.233569
ENSG0000013	-	-	ENSG0000013877	'CENPE	-	4:103105805-103198409	-	-	IGV_LINK	139.64	0.964999	0	2.00012	OK	79.4043	0.550413	0	1.31195	OK	OK	-0.81001
ENSG0000013	-	-	ENSG0000013878	'GSTCD	-	4:105552619-106022478	-	-	IGV_LINK	233.703	4.6206	0	10.1065	OK	228.932	4.44969	0	11.3739	OK	OK	-0.05438
ENSG0000013	-	-	ENSG0000013878	'INTS12	-	4:105552619-106022478	-	-	IGV_LINK	310.311	14.5356	0	30.0677	OK	280.493	12.3447	0	25.6196	OK	OK	-0.2357
ENSG0000013	-	-	ENSG0000013879	'ENPEP	-	4:110365732-110565285	-	-	IGV_LINK	10910.3	132.039	0	315.493	OK	15522	186.244	0	465.485	OK	OK	0.49623
ENSG0000013	-	-	ENSG0000013879	'CASP6	-	4:109560204-109703583	-	-	IGV_LINK	190.065	14.6205	0	31.3238	OK	298.024	18.6254	0.689841	36.5609	OK	OK	0.349276
ENSG0000013	-	-	ENSG0000013879	'LEF1	-	4:108047544-108256836	-	-	IGV_LINK	104.73	3.93034	0	8.65487	OK	119.622	6.64147	0	13.7365	OK	OK	0.756848
ENSG0000013	-	-	ENSG0000013879	'HADH	-	4:107989713-108035175	-	-	IGV_LINK	603.166	16.5722	5.79122	27.3532	OK	666.171	19.9913	7.0605	32.922	OK	OK	0.270602
ENSG0000013	-	-	ENSG0000013879	'EGF	-	4:109912883-110012266	-	-	IGV_LINK	98.9115	2.29175	0	5.54283	OK	192.839	3.34554	0	7.45791	OK	OK	0.54579
ENSG0000013	-	-	ENSG0000013880	'PAPSS1	-	4:107590275-107720452	-	-	IGV_LINK	618.682	14.9329	4.30852	25.5574	OK	551.705	12.9964	4.46985	21.523	OK	OK	-0.20039
ENSG0000013	-	-	ENSG0000013880	'SEC24B	-	4:109347474-109540896	-	-	IGV_LINK	820.384	11.3802	4.47931	18.2812	OK	775.481	10.9936	4.29504	17.6921	OK	OK	-0.04987
ENSG0000013	-	-	ENSG0000013881	'C4orf17	-	4:99511003-99542303	-	-	IGV_LINK	0	0	0	0	OK	0	0	0	0	OK	NOTEST	0
ENSG0000013	-	-	ENSG0000013881	'PPP3CA	-	4:101023408-101348883	-	-	IGV_LINK	240.491	6.22711	0	13.7214	OK	331.023	8.98684	0	18.9655	OK	OK	0.529251
ENSG0000013	-	-	ENSG0000013882	'SLC39A8	-	4:102251040-102450010	-	-	IGV_LINK	372.373	7.12273	1.84865	12.3968	OK	338.242	6.44902	1.50353	11.3945	OK	OK	-0.14335
ENSG0000013	-	-	ENSG0000013882	'MTTP	-	4:99546708-99625913	-	-	IGV_LINK	60.1227	1.22855	0	3.61066	OK	162.934	2.53259	0.125922	4.93926	OK	OK	1.04365
ENSG0000013	-	-	ENSG0000013882	'FBN2	-	5:128257908-129033642	-	-	IGV_LINK	25.2127	0.142307	0	0.385621	OK	94.8727	0.518145	0.006148	1.03014	OK	NOTEST	1.86435
ENSG0000013	-	-	ENSG0000013883	'MAPK8IP3	-	16:1706182-1771730	-	-	IGV_LINK	4883.51	91.0025	0	197.855	OK	3607.22	71.993	0	151.679	OK	OK	-0.33805
ENSG0000013	-	-	ENSG0000013883	'RGS3	-	9:113444730-113597743	-	-	IGV_LINK	625.47	21.2368	5.8719	36.6017	OK	1094.13	34.2099	11.1958	57.2239	OK	OK	0.687846
ENSG0000013	-	-	ENSG0000013886	'GUCD1	-	22:24540422-24629005	-	-	IGV_LINK	2475.7	61.4557	0	166.582	OK	3893.91	96.032	0	259.977	OK	OK	0.643968
ENSG0000013	-	-	ENSG0000013889	'TTLL8	-	22:50015122-50056935	-	-	IGV_LINK	0	0	0	0	OK	1.03122	0.024914	0	0.40131	OK	NOTEST	inf
ENSG0000013	-	-	ENSG0000013894	'RNF185	-	22:31160182-31207019	-	-	IGV_LINK	1342.09	25.9928	8.97633	43.0093	OK	1407.62	27.4592	9.57986	45.3386	OK	OK	0.079177
ENSG0000013	-	-	ENSG0000013894	'KIAA1644	-	22:44243666-44312851	-	-	IGV_LINK	2.90916	0.024922	0	0.066889	OK	4.1249	0.035343	0	0.089532	OK	NOTEST	0.504008
ENSG0000013	-	-	ENSG0000013896	'PARVG	-	22:44172955-44229512	-	-	IGV_LINK	2272.06	58.4827	10.7316	106.234	OK	1888.17	48.1708	12.3762	83.9654	OK	OK	-0.27985
ENSG0000013	-	-	ENSG0000013904	'B4GALNT3	-	12:460363-563509	-	-	IGV_LINK	29.0916	0.36229	0	0.870847	OK	49.4988	0.877158	0	2.34511	OK	OK	1.27569
ENSG0000013	-	-	ENSG0000013905	'PDE6H	-	12:14973021-14981865	-	-	IGV_LINK	0.969721	0.095129	0	0.372596	OK	1.03122	0.101365	0	0.379389	OK	NOTEST	0.09161
ENSG0000013	-	-	ENSG0000013905	'ERP27	-	12:14914034-14939082	-	-	IGV_LINK	23.2733	0.681909	0	1.52531	OK	27.8431	1.12566	0	2.49278	OK	OK	0.723117
ENSG0000013	-	-	ENSG0000013908	'ETV6	-	12:11649853-11895402	-	-	IGV_LINK	979.418	10.4213	1.99236	18.8503	OK	1407.62	14.353	4.44072	24.2652	OK	OK	0.461811
ENSG0000013	-	-	ENSG0000013911	'GABARAPL1	-	12:10212457-10223130	-	-	IGV_LINK	1087.06	37.9105	13.2792	62.5417	OK	2207.85	75.7754	27.331	124.22	OK	OK	0.999134
ENSG0000013	-	-	ENSG0000013911	'KIF21A	-	12:39293227-39443390	-	-	IGV_LINK	743.776	11.2884	3.59862	18.9783	OK	901.291	12.9686	4.92506	21.0121	OK	OK	0.200173
ENSG0000013	-	-	ENSG0000013911	'CPNE8	-	12:38646821-38909592	-	-	IGV_LINK	589.59	13.9497	3.17752	24.7218	OK	634.203	15.044	1.82538	28.2626	OK	OK	0.108958
ENSG0000013	-	-	ENSG0000013913	'YARS2	-	12:32679199-32755902	-	-	IGV_LINK	217.217	8.49573	0	23.0382	OK	298.024	10.514	0	23.951	OK	OK	0.307499
ENSG0000013	-	-	ENSG0000013913	'FGD4	-	12:32399528-32646050	-	-	IGV_LINK	543.043	5.46491	0.398071	10.5317	OK	899.228	7.79307	1.25928	14.3269	OK	OK	0.511994
ENSG0000013	-	-	ENSG0000013913	'ALG10	-	12:34022280-34056740	-	-	IGV_LINK	65.941	2.59661	0	6.32799	OK	31.968	0.836575	0	1.85811	OK	OK	-1.63406
ENSG0000013	-	-	ENSG0000013914	'PIK3C2G	-	12:18080868-18648416	-	-	IGV_LINK	0	0	0	0	OK	1.03122	0.01325	0	0.439287	OK	NOTEST	inf
ENSG0000013	-	-	ENSG0000013914	'FAM60A	-	12:31280421-31327058	-	-	IGV_LINK	636.137	18.4841	1.95435	35.0139	OK	680.608	19.2617	2.9974	35.5261	OK	OK	0.059451
ENSG0000013	-	-	ENSG0000013915	'PLCZ1	-	12:18683168-18738057	-	-	IGV_LINK	0	0	0	0	OK	1.03122	0.093234	0	0.882581	OK	NOTEST	inf
ENSG0000013	-	-	ENSG0000013915	'AEBP2	-	12:19404044-19720801	-	-	IGV_LINK	319.038	12.8665	1.6379	24.0952	OK	371.241	15.7235	1.99539	29.4516	OK	OK	0.2893
ENSG0000013	-	-	ENSG0000013915	'SLCO1C1	-	12:20695354-20753386	-	-	IGV_LINK	12.6064	3.1019	0	13.8037	OK	20.6245	1.30017	0	3.24106	OK	OK	-1.25445
ENSG0000013	-	-	ENSG0000013916	'ETFBKMT	-	12:31647159-31731204	-	-	IGV_LINK	77.5776	1.06976	0	2.68079	OK	70.1233	1.72988	0	4.58677	OK	OK	0.693376
ENSG0000013	-	-	ENSG0000013916	'ETNK1	-	12:22625074-22690665	-	-	IGV_LINK	672.986	7.12745	0.129699	14.1252	OK	684.733	9.85753	0	22.5249	OK	OK	0.46784
ENSG0000013	-	-	ENSG0000013916	'ZCRB1	-	12:42238446-42590355	-	-	IGV_LINK	692.38	38.6258	0	77.4469	OK	757.95	43.1538	0	86.9607	OK	OK	0.15992
ENSG0000013	-	-	ENSG0000013917	'TMEM117	-	12:43835966-44389762	-	-	IGV_LINK	28.1219	1.1388	0	2.94061	OK	43.3114	1.85459	0	4.33018	OK	OK	0.70359
ENSG0000013	-	-	ENSG0000013917	'PRICKLE1	-	12:42238446-42590355	-	-	IGV_LINK	30.0613	2.30962	0	18.0525	OK	26.8118	0.976925	0	8.10573	OK	OK	-1.24134
ENSG0000013	-	-	ENSG0000013917	'C1RL	-	12:7080208-7122501	-	-	IGV_LINK	1240.32	34.4854	0	130.584	OK	2393.16	60.7049	0	289.039	OK	OK	0.815825
ENSG0000013	-	-	ENSG0000013918	'NDUFA9	-	12:4562203-4851927	-	-	IGV_LINK	858.008	53.6218	7.4282	99.8154	OK	812.426	49.7823	6.27827	93.2864	OK	OK	-0.10719
ENSG0000013	-	-	ENSG0000013918	'CLSTN3	-	12:7129078-7158945	-	-	IGV_LINK	3979.73	80.0057	3.7509	156.261	OK	5394.34	116.591	0	236.291	OK	OK	0.543284
ENSG0000013	-	-	ENSG0000013918	'KLRG1	-	12:8950043-9010760	-	-	IGV_LINK	52.3649	3.87333	0	8.40241	OK	42.2802	2.79925	0	5.94031	OK	OK	-0.46854

ENSG0000013	-	-	ENSG0000013919	'VAMP1	-	12:6439000-6470987	-	-	IGV_LINK	633.227	11.1852	0	27.6459	OK	451.677	8.46239	0	25.8421	OK	OK	-0.40245
ENSG0000013	-	-	ENSG0000013919	'TAPBPL	-	12:6439000-6470987	-	-	IGV_LINK	1715.44	67.0867	3.38827	130.785	OK	1577.77	64.6799	5.25047	124.109	OK	OK	-0.05271
ENSG0000013	-	-	ENSG0000013919	'CD27	-	12:6439000-6470987	-	-	IGV_LINK	562.438	27.9779	0	76.8621	OK	210.37	10.4952	0	33.1664	OK	OK	-1.41456
ENSG0000013	-	-	ENSG0000013919	'RBP5	-	12:7123683-7128942	-	-	IGV_LINK	3783.85	277.373	110.613	444.133	OK	6470.94	474.665	173.054	776.276	OK	OK	0.775082
ENSG0000013	-	-	ENSG0000013919	'PEX5	-	12:7166673-7218574	-	-	IGV_LINK	928.992	21.3609	4.51915	38.2027	OK	1262.22	28.6304	7.49405	49.7668	OK	OK	0.422574
ENSG0000013	-	-	ENSG0000013920	'PIANP	-	12:6693791-6700800	-	-	IGV_LINK	10.6669	0.243333	0	0.797541	OK	6.18735	0.138297	0	0.651691	OK	NOTEST	-0.81516
ENSG0000013	-	-	ENSG0000013920	'SLC38A4	-	12:46383678-46876159	-	-	IGV_LINK	2.90916	0.045308	0	0.424521	OK	3.09367	0.095912	0	0.722795	OK	NOTEST	1.08194
ENSG0000013	-	-	ENSG0000013921	'AMIGO2	-	12:47075706-47236662	-	-	IGV_LINK	242.43	4.88249	0	10.6136	OK	125.809	2.56959	0	6.7882	OK	OK	-0.92608
ENSG0000013	-	-	ENSG0000013921	'SCAF11	-	12:45919130-45992120	-	-	IGV_LINK	2256.54	38.1507	0	79.0916	OK	2074.82	38.2211	0	80.3219	OK	OK	0.002658
ENSG0000013	-	-	ENSG0000013921	'COL2A1	-	12:47972964-48004486	-	-	IGV_LINK	0	0	0	0	OK	0	0	0	0	OK	NOTEST	0
ENSG0000013	-	-	ENSG0000013922	'PPFIA2	-	12:80792519-81759553	-	-	IGV_LINK	17.455	0.619434	0	5.48219	OK	10.3122	0.144625	0	1.3459	OK	OK	-2.09863
ENSG0000013	-	-	ENSG0000013922	'ANP32D	-	12:48472664-48473624	-	-	IGV_LINK	0	0	0	0	OK	0	0	0	0	OK	NOTEST	0
ENSG0000013	-	-	ENSG0000013923	'LLPH	-	12:66116554-66170072	-	-	IGV_LINK	320.381	9.71202	0	24.7845	OK	340.866	11.0402	0	28.7011	OK	OK	0.184917
ENSG0000013	-	-	ENSG0000013923	'AC078814.1	-	12:62965324-62965969	-	-	IGV_LINK	9.69721	1.15666	0	2.60878	OK	9.28102	1.10972	0	2.50412	OK	OK	-0.05977
ENSG0000013	-	-	ENSG0000013926	'LRIG3	-	12:58872148-58920522	-	-	IGV_LINK	654.561	12.0245	2.11372	21.9353	OK	1085.88	18.4946	6.01175	30.9775	OK	OK	0.621127
ENSG0000013	-	-	ENSG0000013926	'MARCH9	-	12:57723760-57760407	-	-	IGV_LINK	436.374	14.8434	0	39.1191	OK	488.801	16.0819	0	44.5998	OK	OK	0.115618
ENSG0000013	-	-	ENSG0000013926	'INHBE	-	12:57452322-57459280	-	-	IGV_LINK	2.90916	0.072656	0	0.340195	OK	28.8743	0.752916	0	1.67362	OK	OK	3.37333
ENSG0000013	-	-	ENSG0000013927	'GLIPR1	-	12:75480679-75511636	-	-	IGV_LINK	876.627	28.88	0	64.1717	OK	977.601	45.3381	0	92.0854	OK	OK	0.650656
ENSG0000013	-	-	ENSG0000013928	'TPH2	-	12:71938845-72670757	-	-	IGV_LINK	1.93944	0.179627	0	2.16014	OK	5.15612	0.478453	0	3.62797	OK	NOTEST	1.41338
ENSG0000013	-	-	ENSG0000013928	'PHLDA1	-	12:76025446-76033932	-	-	IGV_LINK	908.628	8.93093	3.56773	14.2941	OK	1175.6	11.7749	4.73969	18.81	OK	OK	0.398826
ENSG0000013	-	-	ENSG0000013929	'TMEM19	-	12:71609471-71705046	-	-	IGV_LINK	419.152	16.7479	0	51.3288	OK	645.435	29.709	0	76.1653	OK	OK	0.82692
ENSG0000013	-	-	ENSG0000013929	'LGR5	-	12:71125084-71586310	-	-	IGV_LINK	2.90916	0.04382	0	0.475428	OK	3.09367	0.039291	0	0.474832	OK	NOTEST	-0.1574
ENSG0000013	-	-	ENSG0000013930	'PTPRQ	-	12:80402177-80680234	-	-	IGV_LINK	0.969721	0.006833	0	0.446946	OK	0	0	0	0	OK	NOTEST	'-inf
ENSG0000013	-	-	ENSG0000013931	'DUSP6	-	12:89347231-89353271	-	-	IGV_LINK	717.593	26.9281	7.05809	46.798	OK	851.792	32.5885	7.16929	58.0077	OK	OK	0.275253
ENSG0000013	-	-	ENSG0000013932	'POC1B	-	12:89419717-89548005	-	-	IGV_LINK	261.558	6.49496	0	15.3738	OK	227.087	5.23974	0	11.2278	OK	OK	-0.30983
ENSG0000013	-	-	ENSG0000013932	'TMTC3	-	12:88142295-88199887	-	-	IGV_LINK	255.036	2.46329	0	5.06654	OK	296.993	2.38105	0.717539	4.04455	OK	OK	-0.04899
ENSG0000013	-	-	ENSG0000013932	'LUM	-	12:91102628-91111831	-	-	IGV_LINK	12.6064	0.904351	0	2.15358	OK	45.3739	1.97083	0	4.49306	OK	OK	1.12385
ENSG0000013	-	-	ENSG0000013933	'KERA	-	12:91050490-91057983	-	-	IGV_LINK	0.969721	0.027469	0	0.107589	OK	0	0	0	0	OK	NOTEST	'-inf
ENSG0000013	-	-	ENSG0000013934	'SNRPF	-	12:95858927-95942974	-	-	IGV_LINK	304.492	15.2348	4.23079	26.2389	OK	293.899	14.6251	3.92954	25.3207	OK	OK	-0.05893
ENSG0000013	-	-	ENSG0000013934	'AMDHD1	-	12:95943292-95968716	-	-	IGV_LINK	122.185	4.33705	0	9.07509	OK	167.058	5.66931	0.324137	11.0145	OK	OK	0.386458
ENSG0000013	-	-	ENSG0000013935	'NEDD1	-	12:96907222-96953777	-	-	IGV_LINK	289.946	5.89012	1.67723	10.103	OK	233.057	5.74368	0.691439	10.7959	OK	OK	-0.03632
ENSG0000013	-	-	ENSG0000013935	'SYCP3	-	12:101696946-101744140	-	-	IGV_LINK	8.72748	0.330692	0	2.42994	OK	11.3435	0.690579	0	4.25256	OK	OK	1.06232
ENSG0000013	-	-	ENSG0000013935	'ASCL1	-	12:102836884-102960516	-	-	IGV_LINK	1.93944	0.047532	0	0.329107	OK	0	0	0	0	OK	NOTEST	'-inf
ENSG0000013	-	-	ENSG0000013935	'GAS2L3	-	12:100573682-100628286	-	-	IGV_LINK	757.352	18.0986	2.77765	33.4195	OK	365.054	8.81253	0	18.4496	OK	OK	-1.03825
ENSG0000013	-	-	ENSG0000013936	'TMEM132B	-	12:125186835-125662377	-	-	IGV_LINK	3.87888	0.298652	0	1.02709	OK	14.4371	0.180546	0	0.799151	OK	NOTEST	-0.7261
ENSG0000013	-	-	ENSG0000013937	'SLC15A4	-	12:128793190-128823983	-	-	IGV_LINK	2372.91	68.3893	23.0402	113.739	OK	1730.4	48.0873	16.7794	79.3952	OK	OK	-0.50812
ENSG0000013	-	-	ENSG0000013937	'TDG	-	12:103965803-103988874	-	-	IGV_LINK	498.436	14.1048	3.57214	24.6375	OK	560.986	14.628	4.06487	25.1911	OK	OK	0.052542
ENSG0000013	-	-	ENSG0000013940	'RITA1	-	12:113185525-113192368	-	-	IGV_LINK	339.402	10.6965	2.45887	18.934	OK	392.897	12.3623	3.17907	21.5456	OK	OK	0.208819
ENSG0000013	-	-	ENSG0000013941	'SDSL	-	12:113392444-113438276	-	-	IGV_LINK	225.945	11.0678	0	24.0282	OK	239.244	11.6875	0	28.1752	OK	OK	0.078595
ENSG0000013	-	-	ENSG0000013942	'MMAB	-	12:109553736-109598117	-	-	IGV_LINK	511.043	27.684	2.33054	53.0374	OK	692.983	34.7366	0	69.5864	OK	OK	0.327404
ENSG0000013	-	-	ENSG0000013943	'GLTP	-	12:109850942-109880488	-	-	IGV_LINK	468.375	12.3418	4.18266	20.501	OK	601.204	15.7007	6.15601	25.2453	OK	OK	0.347271
ENSG0000013	-	-	ENSG0000013943	'GIT2	-	12:109900263-109996389	-	-	IGV_LINK	2876.19	85.4231	0	185.725	OK	1845.89	53.7041	0	114.104	OK	OK	-0.6696
ENSG0000013	-	-	ENSG0000013943	'TCHP	-	12:109900263-109996389	-	-	IGV_LINK	2778.25	51.4382	0.204986	102.672	OK	1838.67	34.2895	1.84901	66.7299	OK	OK	-0.58508
ENSG0000013	-	-	ENSG0000013943	'FAM222A	-	12:109714227-109773508	-	-	IGV_LINK	14.5458	0.320399	0	0.777332	OK	65.9984	1.29598	0	2.73465	OK	OK	2.01611
ENSG0000013	-	-	ENSG0000013944	'FOXN4	-	12:109277978-109309220	-	-	IGV_LINK	0.969721	0.038472	0	0.194346	OK	5.15612	0.091485	0	0.274645	OK	NOTEST	1.24972
ENSG0000013	-	-	ENSG0000013949	'NUP58	-	13:25301523-25349800	-	-	IGV_LINK	897.961	12.6317	3.81892	21.4445	OK	1013.69	15.0028	3.05778	26.9478	OK	OK	0.248182
ENSG0000013	-	-	ENSG0000013950	'MTMR6	-	13:25246200-25288009	-	-	IGV_LINK	514.922	5.28911	1.95749	8.62073	OK	574.392	5.90121	2.24899	9.55343	OK	OK	0.157986
ENSG0000013	-	-	ENSG0000013950	'SLC46A3	-	13:28700063-28718970	-	-	IGV_LINK	928.023	19.8277	8.23863	31.4168	OK	933.259	19.2511	7.97995	30.5223	OK	OK	-0.04258
ENSG0000013	-	-	ENSG0000013951	'SLC7A1	-	13:29509409-29595688	-	-	IGV_LINK	926.083	7.50851	2.41554	12.6015	OK	1003.38	8.16801	2.56025	13.7758	OK	OK	0.121459
ENSG0000013	-	-	ENSG0000013951	'PDX1	-	13:27920019-27926231	-	-	IGV_LINK	0	0	0	0	OK	0	0	0	0	OK	NOTEST	0
ENSG0000013	-	-	ENSG0000013951	'LNX2	-	13:27545910-27620404	-	-	IGV_LINK	288.977	3.65596	1.18547	6.12645	OK	316.586	4.00629	1.3464	6.66618	OK	OK	0.132017
ENSG0000013	-	-	ENSG0000013953	'SUOX	-	12:55997179-56006641	-	-	IGV_LINK	741.836	23.0724	3.6779	42.467	OK	770.325	21.5414	5.27327	37.8096	OK	OK	-0.09906
ENSG0000013	-	-	ENSG0000013953	'CCDC65	-	12:48903417-48957551	-	-	IGV_LINK	47.5163	2.72792	0	21.8034	OK	87.6541	2.86148	0	15.3703	OK	OK	0.06896
ENSG0000013	-	-	ENSG0000013954	'SLC39A5	-	12:56230048-56258391	-	-	IGV_LINK	413.101	23.8648	0	56.4432	OK	802.293	49.4199	0	104.331	OK	OK	1.05021
ENSG0000013	-	-	ENSG0000013954	'TARBP2	-	12:53500161-53506431	-	-	IGV_LINK	372.373	20.2678	4.92543	35.6102	OK	361.96	21.4288	4.99348	37.8642	OK	OK	0.080361
ENSG0000013	-	-	ENSG0000013954	'RDH16	-	12:56951430-56959374	-	-	IGV_LINK	21.3339	0.505014	0	1.06249	OK	21.6557	0.52348	0	1.10506	OK	NOTEST	0.051813
ENSG0000013	-	-	ENSG0000013954	'DHH	-	12:49089420-49094819	-	-	IGV_LINK	0	0	0	0	OK	4.1249	0.131906	0	0.334151	OK	NOTEST	inf
ENSG0000013	-	-	ENSG0000013956	'ACVRL1	-	12:51906907-51923361	-	-	IGV_LINK	637.106	12.2273	1.71553	22.7391	OK	868.291	16.1755	4.86188	27.4892	OK	OK	0.403706
ENSG0000013	-	-	ENSG0000013957	'GPR84	-	12:54353660-54497688	-	-	IGV_LINK	76.6079	2.90711	0	13.3561	OK	83.5292	3.35416	0	18.8969	OK	OK	0.206363
ENSG0000013	-	-	ENSG0000013957	'NPFF	-	12:53506687-53626410	-	-	IGV_LINK	1.01E-37	1.36E-38	0	0.268695	OK	7.36E-109	9.92E-110	0	0.269435	OK	NOTEST	-236.316
ENSG0000013	-	-	ENSG0000013957	'NABP2	-	12:56222014-56229854	-	-	IGV_LINK	429.586	19.2076	6.42734	31.9879	OK	592.954	29.081	9.82159	48.3405	OK	OK	0.598401
ENSG0000013	-	-	ENSG0000013959	'N4BP2L1	-	13:32400722-32538885	-	-	IGV_LINK	887.325	36.7703	0	84.7273	OK	512.948	18.9004	0	48.237	OK	OK	-0.96013
ENSG0000013	-	-	ENSG0000013961	'CELA1	-	12:51328442-51346679	-	-	IGV_LINK	1.93944	0.14022	0	0.429418	OK	1.03122	0.074667	0	0.279463	OK	NOTEST	-0.90915
ENSG0000013	-	-	ENSG0000013961	'SMARCC2	-	12:56150795-56190284	-	-	IGV_LINK	3152.56	55.5607	0	176.652	OK	2890.52	51.6178	0	165.124	OK	OK	-0.1062
ENSG0000013	-	-	ENSG0000013961	'BRCA2	-	13:32315473-32400266	-	-	IGV_LINK	119.276	3.85375	0	10.6063	OK	118.591	2.31803	0	7.27523	OK	OK	-0.73337
ENSG0000013	-	-	ENSG0000013962	'KANSL2	-	12:48653400-48682238	-	-	IGV_LINK	611.894	21.558	6.31448	36.8015	OK	550.674	19.2015	5.16554	33.2374	OK	OK	-0.16701
ENSG0000013	-	-	ENSG0000013962	'CERS5	-	12:50111978-50167533	-	-	IGV_LINK	704.987	24.9045	1.5638	48.2452	OK	674.421	25.251	0	52.6812	OK	OK	0.019931
ENSG0000013	-	-	ENSG0000013962	'MAP3K12	-	12:53441604-53500063	-	-	IGV_LINK	1039.54	21.8829	0	102.073	OK	1102.38	25.16	0	126.559	OK	OK	0.20133
ENSG0000013	-	-	ENSG0000013962	'ITGB7	-	12:53157662-53207307	-	-	IGV_LINK	785.474	23.5354	0	56.3805	OK	683.702	23.8968	0	59.3802	OK	OK	0.02199
ENSG0000013	-	-	ENSG0000013962	'GALNT6	-	12:51351246-51515763	-	-	IGV_LINK	189.095	4.41606	0	12.6056	OK	144.371	2.47954	0	7.15201	OK	OK	-0.83269
ENSG0000013	-	-	ENSG0000013963	'CSAD	-	12:53157662-53207307	-	-	IGV_LINK	786.443	46.4515	0	107.968	OK	823.949	46.4424	0	105.087	OK	OK	-0.00028
ENSG0000013	-	-	ENSG0000013963	'LMBR1L	-	12:49096550-49110900	-	-	IGV_LINK	1113.24	59.269	14.6491	103.889	OK	1035.35	58.7859	14.7544	102.817	OK	OK	-0.01181
ENSG0000013	-	-	ENSG0000013963	'C12orf10	-	12:53295290-53307177	-	-	IGV_LINK	671.047	41.5081	0	134.37	OK	593.986	39.1479	0	120.911	OK	OK	-0.08446
ENSG0000013	-	-	ENSG0000013964	'ESYT1	-	12:56118158-56144671	-	-	IGV_LINK	3256.68	54.3456	5.94652	102.745	OK	2607.97	42.6519	5.44902	79.8547	OK	OK	-0.34956
ENSG0000013	-	-	ENSG0000013964	'TMBIM6	-	12:49568217-49764934	-	-	IGV_LINK	17423	424.94	20.3399	829.541	OK	26971.7	672.857	0	1411.64	OK	OK	0.663039
ENSG0000013	-	-	ENSG0000013964	'ANKRD52	-	12:56230048-56258391	-	-	IGV_LINK	1192.76	8.32887	0.437428	16.2203	OK	1313.78	8.95373	0.940014	16.9674	OK	OK	0.104368
ENSG0000013	-	-	ENSG0000013964	'KRT71	-	12:52543908-52553147	-	-	IGV_LINK	0	0	0	0	OK	0	0	0	0	OK	NOTEST	0
ENSG0000013	-	-	ENSG0000013965	'ZNF740	-	12:53157662-53207307	-	-	IGV_LINK	942.568	14.3595	0	45.9053	OK	929.134	13.136	0	41.8349	OK	OK	-0.12848
ENSG0000013	-	-	ENSG0000013965	'SMIM2	-	13:44106377-44243192	-	-	IGV_LINK	2.90916	0.252431	0	2.4159	OK	7.21857	0.627522	0	5.43096	OK	OK	1.31378
ENSG0000013	-	-	ENSG0000013966	'WDFY2	-	13:51584454-51767707	-	-	IGV_LINK	539.165	4.45622	0	12.4005	OK	381.553	2.36457	0.815378	3.91377	OK	OK	-0.91424
ENSG0000013	-	-	ENSG0000013967	'HNRNPA1L2	-	13:52600041-52643796	-	-	IGV_LINK	189.007	3.75256	0.612378	6.89275	OK	177.54	3.52632	0.654793	6.39785	OK	OK	-0.08971
ENSG0000013	-	-	ENSG0000013967	'LPAR6	-	13:48303750-48481986	-	-	IGV_LINK	1203.42	58.6296	0	123.203	OK	840.448	34.8763	0	77.4121	OK	OK	-0.74938
ENSG0000013	-	-	ENSG0000013968	'ESD	-	13:46771255-46797232	-	-	IGV_LINK	2087.81	113.746	50.0383	177.453	OK	2018.11	110.119	47.4339	172.804	OK	OK	-0.04675
ENSG0000013	-	-	ENSG0000013968	'RB1	-	13:48303750-48481986	-	-	IGV_LINK	1081.24	30.6132	0	69.195	OK	1142.6	30.2618	0	66.1535	OK	OK	-0.01665
ENSG0000013	-	-	ENSG0000013969	'SBNO1	-	12:123289108-123366113	-	-	IGV_LINK	901.84	5.43865	1.99386	8.88344	OK	908.509	5.2342	1.84233	8.62607	OK	OK	-0.05528
ENSG0000013	-	-	ENSG0000013971	'MORN3	-	12:121648741-121672631	-	-	IGV_LINK	58.1832	1.14574	0	2.58497	OK	67.0296	1.98837	0	4.57449	OK	OK	0.795306
ENSG0000013	-	-	ENSG0000013971	'SETD1B	-	12:121804179-121832584	-	-	IGV_LINK	834.929	6.50454	2.65009	10.359	OK	845.604	6.56831	2.68385	10.4528	OK	OK	0.014075
ENSG0000013	-	-	ENSG0000013971	'VPS33A	-	12:122203542-122266521	-	-	IGV_LINK	478.072	9.62019	0	20.6543	OK	624.922	13.2668	0	29.9736	OK	OK	0.463681
ENSG0000013	-	-	ENSG0000013972	'VPS37B	-	12:122865327-122896444	-	-	IGV_LINK	302.865	6.26398	1.19661	11.3314	OK	317.547	6.78066	1.18077	12.3805	OK	OK	0.114344
ENSG0000013	-	-	ENSG0000013972	'RHOF	-	12:121712751-121803906	-	-	IGV_LINK	388.926	10.6341	0	24.0759	OK	376.599	10.4782	0	24.2254	OK	OK	-0.02131
ENSG0000013	-	-	ENSG0000013972	'DENR	-	12:122752773-122771064	-	-	IGV_LINK	691.411	18.0505	5.06367	31.0374	OK	833.23	22.4051	6.52525	38.285	OK	OK	0.311787
ENSG0000013	-	-	ENSG0000013973	'DIAPH3	-	13:59665582-60163987	-	-	IGV_LINK	48.486	0.752495	0	1.70157	OK	60.8423	0.897048	0	1.87893	OK	OK	0.253504
ENSG0000013	-	-	ENSG0000013973	'SLAIN1	-	13:77697853-77764242	-	-	IGV_LINK	135.761	6.44237	0.413276	12.4715	OK	62.9047	3.21903	0	6.86088	OK	OK	-1.00096
ENSG0000013	-	-	ENSG0000013974	'RBM26	-	13:79311823-79424328	-	-	IGV_LINK	922.204	13.8895	5.24478	22.5342	OK	791.981	11.93	4.53562	19.3243	OK	OK	-0.21941
ENSG0000013	-	-	ENSG0000013976	'SRRM4	-	12:118981494-119163051	-	-	IGV_LINK	9.69721	0.065807	0	0.446522	OK	4.1249	0.027996	0	0.398405	OK	NOTEST	-1.23301
ENSG0000013	-	-	ENSG0000013978	'METTL21C	-	13:102685743-102694504	-	-	IGV_LINK	0	0	0	0	OK	0	0	0	0	OK	NOTEST	0
ENSG0000013	-	-	ENSG0000013979	'MBNL2	-	13:97221433-97394120	-	-	IGV_LINK	776.746	11.6811	4.07035	19.2919	OK	885.822	16.013	1.92627	30.0998	OK	OK	0.455066
ENSG0000013	-	-	ENSG0000013979	'RNF113B	-	13:98142561-98578830	-	-	IGV_LINK	0	0	0	0	OK	1.03122	0.048336	0	1.95805	OK	NOTEST	inf
ENSG0000013	-	-	ENSG0000013980	'ZIC5	-	13:99962963-99971909	-	-	IGV_LINK	0	0	0	0	OK	4.1249	0.052059	0	0.131879	OK	NOTEST	inf
ENSG0000013	-	-	ENSG0000013982	'ABHD13	-	13:108218378-108234255	-	-	IGV_LINK	205.581	2.24289	0.570169	3.9156	OK	213.464	2.32941	0.571773	4.08704	OK	OK	0.054606
ENSG0000013	-	-	ENSG0000013983	'RAB20	-	13:110523065-110561733	-	-	IGV_LINK	448.981	18.8056	7.04283	30.5684	OK	652.765	27.3646	11.1458	43.5834	OK	OK	0.541145
ENSG0000013	-	-	ENSG0000013983	'GRTP1	-	13:113324163-113364148	-	-	IGV_LINK	84.3657	3.39657	0	7.12658	OK	120.653	5.13388	0.353602	9.91417	OK	OK	0.595971
ENSG0000013	-	-	ENSG0000013984	'CUL4A	-	13:113177538-113267108	-	-	IGV_LINK	1138.45	27.7474	0	56.6448	OK	1211.69	25.6755	1.50675	49.8442	OK	OK	-0.11196
ENSG0000013	-	-	ENSG0000013986	'TTC6	-	14:37589983-38202923	-	-	IGV_LINK	0.969721	0.016099	0	0.542571	OK	2.06245	0.020538	0	0.545308	OK	NOTEST	0.351283
ENSG0000013	-	-	ENSG0000013987	'SSTR1	-	14:38207998-38213067	-	-	IGV_LINK	20.3641	0.278383	0	0.616149	OK	25.7806	0.352527	0	0.731474	OK	NOTEST	0.34066
ENSG0000013	-	-	ENSG0000013988	'CDH24	-	14:23047061-23057538	-	-	IGV_LINK	156.125	2.82497	0.352336	5.29761	OK	128.903	2.3707	0.192956	4.54844	OK	OK	-0.25292
ENSG0000013	-	-	ENSG0000013989	'REM2	-	14:22883164-22887686	-	-	IGV_LINK	18.4247	0.739358	0	1.747	OK	11.3435	0.392913	0	0.876399	OK	OK	-0.91206
ENSG0000013	-	-	ENSG0000013989	'CBLN3	-	14:24426531-24442905	-	-	IGV_LINK	623.53	16.6983	0	40.0792	OK	480.551	13.6849	0	33.7242	OK	OK	-0.28712
ENSG0000013	-	-	ENSG0000013990	'TSSK4	-	14:24171852-24239242	-	-	IGV_LINK	51.3952	2.83509	0	19.6432	OK	47.4363	2.53994	0	19.5255	OK	OK	-0.1586
ENSG0000013	-	-	ENSG0000013991	'NOVA1	-	14:26443092-26597754	-	-	IGV_LINK	234.672	4.65473	0	9.79731	OK	303.18	6.11416	0.216382	12.0119	OK	OK	0.393455
ENSG0000013	-	-	ENSG0000013991	'FITM1	-	14:24080106-24132849	-	-	IGV_LINK	13.5761	0.948374	0	7.34287	OK	2.06245	0.185576	0	3.03324	OK	OK	-2.35345
ENSG0000013	-	-	ENSG0000013991	'MDGA2	-	14:46840091-47674954	-	-	IGV_LINK	1.93944	0.104649	0	0.588684	OK	0	0	0	0	OK	NOTEST	'-inf
ENSG0000013	-	-	ENSG0000013992	'TMX1	-	14:51240161-51257546	-	-	IGV_LINK	517.831	11.3845	3.78907	18.9799	OK	630.078	14.6938	5.0649	24.3227	OK	OK	0.368137
ENSG0000013	-	-	ENSG0000013992	'FRMD6	-	14:51454511-51730727	-	-	IGV_LINK	119.276	2.27083	0	5.70916	OK	73.217	1.5001	0	3.87125	OK	OK	-0.59816
ENSG0000013	-	-	ENSG0000013994	'PELI2	-	14:56117315-56301526	-	-	IGV_LINK	124.124	1.51852	0	3.45547	OK	162.934	1.99619	0	4.36959	OK	OK	0.394586
ENSG0000013	-	-	ENSG0000013997	'RTN1	-	14:59595975-59870966	-	-	IGV_LINK	385.949	8.90915	2.31721	15.5011	OK	358.866	7.79223	2.57659	13.0079	OK	OK	-0.19325
ENSG0000013	-	-	ENSG0000013997	'C14orf37	-	14:57999734-58298139	-	-	IGV_LINK	4.8486	0.099124	0	1.5621	OK	8.94565	0.401407	0	5.59152	OK	NOTEST	2.01776
ENSG0000013	-	-	ENSG0000013997	'SYT16	-	14:61811973-62130962	-	-	IGV_LINK	0.969721	0.003959	0	0.352402	OK	0	0	0	0	OK	NOTEST	'-inf
ENSG0000013	-	-	ENSG0000013997	'SLC38A6	-	14:60734741-61083733	-	-	IGV_LINK	167.762	9.76737	0	22.9995	OK	138.184	7.78793	0	18.8277	OK	OK	-0.32673
ENSG0000013	-	-	ENSG0000013997	'NAA30	-	14:57390543-57415917	-	-	IGV_LINK	200.732	2.17254	0.286154	4.05893	OK	185.62	1.8887	0.051983	3.72541	OK	OK	-0.20199
ENSG0000013	-	-	ENSG0000013998	'ADAM21	-	14:70425811-70547464	-	-	IGV_LINK	7.75776	0.175899	0	0.596437	OK	2.06245	0.046785	0	0.228261	OK	NOTEST	-1.91061
ENSG0000013	-	-	ENSG0000013998	'RDH12	-	14:67701885-67816590	-	-	IGV_LINK	7.75776	0.263281	0	2.12683	OK	18.562	0.613415	0	3.24732	OK	OK	1.22026
ENSG0000013	-	-	ENSG0000013999	'DCAF5	-	14:69050880-69153150	-	-	IGV_LINK	1192.76	18.4654	5.69052	31.2403	OK	1367.4	21.418	8.23339	34.6027	OK	OK	0.214001
ENSG0000013	-	-	ENSG0000013999	'RAB15	-	14:64914360-65102695	-	-	IGV_LINK	145.458	3.12442	0	13.5983	OK	217.588	5.28609	0	20.8003	OK	OK	0.75861
ENSG0000014	-	-	ENSG0000014000	'WDR89	-	14:63597038-63641861	-	-	IGV_LINK	121.215	3.34802	0.219616	6.47643	OK	76.3106	1.81265	0	3.65659	OK	OK	-0.88521
ENSG0000014	-	-	ENSG0000014000	'ESR2	-	14:63852982-64338599	-	-	IGV_LINK	17.1547	0.234177	0	2.27955	OK	17.9436	0.422902	0	3.85071	OK	NOTEST	0.852724
ENSG0000014	-	-	ENSG0000014001	'KCNH5	-	14:62699453-63102037	-	-	IGV_LINK	0	0	0	0	OK	0	0	0	0	OK	NOTEST	0
ENSG0000014	-	-	ENSG0000014002	'STON2	-	14:81260655-81436465	-	-	IGV_LINK	42.6677	1.10268	0	2.95563	OK	62.9047	1.29607	0	3.37631	OK	OK	0.233119
ENSG0000014	-	-	ENSG0000014002	'EFCAB11	-	14:89124870-89954777	-	-	IGV_LINK	94.0629	5.13062	0	21.0691	OK	112.404	7.97452	0	26.8797	OK	OK	0.636266
ENSG0000014	-	-	ENSG0000014003	'GPR65	-	14:88005123-88015611	-	-	IGV_LINK	432.495	5.60376	2.02867	9.17885	OK	281.524	3.64863	1.16201	6.13524	OK	OK	-0.61904
ENSG0000014	-	-	ENSG0000014004	'PTGR2	-	14:73851843-74084493	-	-	IGV_LINK	397.299	12.5404	0	38.1051	OK	328.851	9.68987	0	31.9374	OK	OK	-0.37204
ENSG0000014	-	-	ENSG0000014004	'JDP2	-	14:75423682-75474111	-	-	IGV_LINK	217.217	8.24741	1.06987	15.425	OK	143.34	5.44001	0	11.2247	OK	OK	-0.60033

ENSG0000014	-	-	ENSG0000014005	'AK7	-	14:96392110-96489427	-	-	IGV_LINK	70.7896	1.97354	0	4.51851	OK	76.3106	1.91549	0	4.20067	OK	OK	-0.04307
ENSG0000014	-	-	ENSG0000014006	'FAM181A	-	14:93904729-93929608	-	-	IGV_LINK	0	0	0	0	OK	0	0	0	0	OK	NOTEST	0
ENSG0000014	-	-	ENSG0000014009	'SLC24A4	-	14:92322580-92501483	-	-	IGV_LINK	75.6382	0.618977	0	1.57154	OK	57.7486	0.440129	0	1.02226	OK	OK	-0.49196
ENSG0000014	-	-	ENSG0000014009	'FBLN5	-	14:91869411-91947987	-	-	IGV_LINK	134.791	3.72117	0.294903	7.14745	OK	237.182	6.19773	1.67145	10.724	OK	OK	0.735983
ENSG0000014	-	-	ENSG0000014009	'SERPINA10	-	14:94280454-94293271	-	-	IGV_LINK	0	0	0	0	OK	0	0	0	0	OK	NOTEST	0
ENSG0000014	-	-	ENSG0000014010	'C14orf79	-	14:104985774-105021148	-	-	IGV_LINK	331.644	9.8078	0.57104	19.0446	OK	351.648	10.4815	0	21.4938	OK	OK	0.095847
ENSG0000014	-	-	ENSG0000014010	'WARS	-	14:100333787-100530303	-	-	IGV_LINK	3532.69	129.639	18.1207	241.158	OK	1378.75	53.3757	0	108.753	OK	OK	-1.28025
ENSG0000014	-	-	ENSG0000014010	'SLC25A47	-	14:100323336-100330378	-	-	IGV_LINK	1.93944	0.079229	0	0.259467	OK	1.03122	0.042161	0	0.178882	OK	NOTEST	-0.91013
ENSG0000014	-	-	ENSG0000014015	'WDR20	-	14:102080737-102305200	-	-	IGV_LINK	264.734	6.68861	0	28.2336	OK	265.025	7.11959	0	32.9066	OK	OK	0.090086
ENSG0000014	-	-	ENSG0000014015	'NIPA2	-	15:22838640-22981063	-	-	IGV_LINK	387.888	15.5952	0	42.6195	OK	565.111	21.8878	0	59.6538	OK	OK	0.48903
ENSG0000014	-	-	ENSG0000014019	'SLC12A6	-	15:34224998-34338060	-	-	IGV_LINK	960.993	15.5237	0	39.9662	OK	1008.54	16.7132	0	43.4354	OK	OK	0.106511
ENSG0000014	-	-	ENSG0000014025	'DUOXA1	-	15:45092649-45201175	-	-	IGV_LINK	4.8486	0.421891	0	4.18558	OK	1.03122	0.02315	0	0.713147	OK	NOTEST	-4.18776
ENSG0000014	-	-	ENSG0000014025	'MFAP1	-	15:43804491-43824802	-	-	IGV_LINK	581.832	16.7059	6.4259	26.9859	OK	647.609	18.4201	7.48984	29.3504	OK	OK	0.140928
ENSG0000014	-	-	ENSG0000014026	'TCF12	-	15:56918622-57299281	-	-	IGV_LINK	1190.82	20.5248	3.01267	38.037	OK	1083.82	17.2372	4.3295	30.145	OK	OK	-0.25184
ENSG0000014	-	-	ENSG0000014026	'SORD	-	15:45023103-45077185	-	-	IGV_LINK	286.068	7.62395	1.21165	14.0363	OK	668.234	18.1226	4.82861	31.4166	OK	OK	1.24918
ENSG0000014	-	-	ENSG0000014026	'SERF2	-	15:43726917-43803043	-	-	IGV_LINK	5858.54	549.184	0	1189.73	OK	6285.55	596.328	0	1283.2	OK	OK	0.118816
ENSG0000014	-	-	ENSG0000014026	'ZSCAN29	-	15:43358171-43510728	-	-	IGV_LINK	629.349	11.7063	0	35.3954	OK	629.047	10.9646	0	32.5205	OK	OK	-0.09443
ENSG0000014	-	-	ENSG0000014027	'DUOXA2	-	15:45092649-45201175	-	-	IGV_LINK	2.90916	0.105672	0	1.24711	OK	0	0	0	0	OK	NOTEST	'-inf
ENSG0000014	-	-	ENSG0000014027	'DUOX2	-	15:45092649-45201175	-	-	IGV_LINK	50.4255	0.616696	0	4.01205	OK	7.21857	0.064193	0	0.491329	OK	OK	-3.26407
ENSG0000014	-	-	ENSG0000014028	'LYSMD2	-	15:51723010-51816368	-	-	IGV_LINK	263.764	15.9794	0	32.3105	OK	209.339	13.5511	0	28.3454	OK	OK	-0.23781
ENSG0000014	-	-	ENSG0000014028	'SLC27A2	-	15:49858237-50236395	-	-	IGV_LINK	343.281	8.75177	1.56776	15.9358	OK	35.0616	0.889921	0	2.74185	OK	OK	-3.29783
ENSG0000014	-	-	ENSG0000014028	'FGF7	-	15:49106067-49620931	-	-	IGV_LINK	12.6064	0.159734	0	1.20743	OK	11.3435	0.154444	0	1.2977	OK	NOTEST	-0.04859
ENSG0000014	-	-	ENSG0000014028	'HDC	-	15:50241946-50266026	-	-	IGV_LINK	38.7888	1.2979	0	2.81947	OK	36.0929	1.41603	0	3.02706	OK	OK	0.125674
ENSG0000014	-	-	ENSG0000014029	'GCNT3	-	15:59594874-59689534	-	-	IGV_LINK	271.522	7.24854	0	20.1852	OK	1081.75	31.4886	0	63.8399	OK	OK	2.11907
ENSG0000014	-	-	ENSG0000014029	'BNIP2	-	15:59594874-59689534	-	-	IGV_LINK	811.656	23.9409	0	49.1769	OK	844.573	24.6564	0	55.0109	OK	OK	0.042482
ENSG0000014	-	-	ENSG0000014030	'GTF2A2	-	15:59594874-59689534	-	-	IGV_LINK	267.643	29.6538	0	77.7258	OK	255.744	28.5567	0	85.654	OK	OK	-0.05439
ENSG0000014	-	-	ENSG0000014031	'SRP14	-	15:40035738-40039188	-	-	IGV_LINK	3189.41	214.703	89.8942	339.513	OK	3479.35	242.039	95.0435	389.034	OK	OK	0.172891
ENSG0000014	-	-	ENSG0000014032	'BAHD1	-	15:40439720-40468242	-	-	IGV_LINK	527.528	7.73941	1.05552	14.4233	OK	466.114	6.8865	0.853252	12.9197	OK	OK	-0.16845
ENSG0000014	-	-	ENSG0000014032	'DISP2	-	15:40358234-40378639	-	-	IGV_LINK	11.6366	0.15874	0	0.874881	OK	9.28102	0.522584	0	2.04506	OK	NOTEST	1.71899
ENSG0000014	-	-	ENSG0000014032	'CDAN1	-	15:42723558-42737126	-	-	IGV_LINK	213.339	4.56703	0	13.0304	OK	298.024	5.0433	0	10.9133	OK	OK	0.143111
ENSG0000014	-	-	ENSG0000014033	'TLE3	-	15:70047789-70098176	-	-	IGV_LINK	1265.49	23.3944	0	48.6764	OK	1325.12	24.9311	0	50.015	OK	OK	0.091782
ENSG0000014	-	-	ENSG0000014035	'ANP32A	-	15:68778534-69062743	-	-	IGV_LINK	1909.38	90.2811	24.4206	156.142	OK	2536.81	121.923	36.8631	206.983	OK	OK	0.433477
ENSG0000014	-	-	ENSG0000014036	'COMMD4	-	15:75335890-75343224	-	-	IGV_LINK	787.413	50.7544	17.7405	83.7683	OK	917.79	61.4527	20.4575	102.448	OK	OK	0.275943
ENSG0000014	-	-	ENSG0000014036	'UBE2Q2	-	15:75843284-75901078	-	-	IGV_LINK	363.645	8.74593	1.20702	16.2848	OK	478.488	10.8172	3.1087	18.5256	OK	OK	0.306638
ENSG0000014	-	-	ENSG0000014036	'PSTPIP1	-	15:76993358-77037332	-	-	IGV_LINK	490.679	23.2877	5.73439	40.841	OK	304.211	15.6451	2.63186	28.6584	OK	OK	-0.57386
ENSG0000014	-	-	ENSG0000014037	'ETFA	-	15:76059836-76311472	-	-	IGV_LINK	1189.85	72.6481	23.3658	121.93	OK	1493.21	91.3475	27.6653	155.03	OK	OK	0.33044
ENSG0000014	-	-	ENSG0000014037	'BCL2A1	-	15:79960888-79971446	-	-	IGV_LINK	120.245	8.66261	0.795408	16.5298	OK	107.247	7.22727	1.00037	13.4542	OK	OK	-0.26135
ENSG0000014	-	-	ENSG0000014038	'HMG20A	-	15:77420411-77485607	-	-	IGV_LINK	562.438	19.7348	0	41.543	OK	541.393	18.3592	0	38.2925	OK	OK	-0.10424
ENSG0000014	-	-	ENSG0000014038	'SCAPER	-	15:76347903-76905444	-	-	IGV_LINK	329.705	9.93065	0	21.4867	OK	232.026	6.50315	0	14.9921	OK	OK	-0.61075
ENSG0000014	-	-	ENSG0000014039	'TSPAN3	-	15:77041403-77083984	-	-	IGV_LINK	2356.42	85.4525	27.5158	143.389	OK	3910.41	168.038	55.3029	280.774	OK	OK	0.975596
ENSG0000014	-	-	ENSG0000014039	'WDR61	-	15:78250501-78299924	-	-	IGV_LINK	738.927	37.4322	8.11839	66.746	OK	769.294	40.8538	5.96336	75.7441	OK	OK	0.126189
ENSG0000014	-	-	ENSG0000014039	'NCOA2	-	8:70109761-70403805	-	-	IGV_LINK	1168.51	13.5664	0.442491	26.6903	OK	1045.66	9.27024	1.05305	17.4874	OK	OK	-0.54936
ENSG0000014	-	-	ENSG0000014039	'NEIL1	-	15:75346743-75455842	-	-	IGV_LINK	278.31	10.9708	0	48.4858	OK	265.025	12.4089	0	55.6242	OK	OK	0.177708
ENSG0000014	-	-	ENSG0000014040	'MAN2C1	-	15:75346743-75455842	-	-	IGV_LINK	3379.48	166.82	5.79726	327.842	OK	3419.54	160.208	0	320.721	OK	OK	-0.05834
ENSG0000014	-	-	ENSG0000014040	'DNAJA4	-	15:78250501-78299924	-	-	IGV_LINK	445.102	11.1453	0	23.7425	OK	633.172	15.6841	0.683875	30.6843	OK	OK	0.49287
ENSG0000014	-	-	ENSG0000014040	'TLNRD1	-	15:81000943-81005788	-	-	IGV_LINK	294.795	3.55556	1.16247	5.94866	OK	408.365	4.92656	1.79714	8.05598	OK	OK	0.470503
ENSG0000014	-	-	ENSG0000014041	'TPM1	-	15:63042631-63071915	-	-	IGV_LINK	11545.5	506.28	70.2583	942.302	OK	5735.67	243.424	72.3192	414.529	OK	OK	-1.05646
ENSG0000014	-	-	ENSG0000014044	'IGF1R	-	15:98648970-99135593	-	-	IGV_LINK	848.505	4.96589	0	13.6657	OK	847.667	4.34745	0.119228	8.57568	OK	OK	-0.19188
ENSG0000014	-	-	ENSG0000014045	'ARRDC4	-	15:97960697-97973838	-	-	IGV_LINK	872.748	12.613	5.37479	19.8512	OK	948.727	13.7151	5.9029	21.5273	OK	OK	0.120853
ENSG0000014	-	-	ENSG0000014045	'PIF1	-	15:64815631-64825668	-	-	IGV_LINK	85.3354	4.22404	0	12.2543	OK	69.0921	3.22869	0	8.78208	OK	OK	-0.38768
ENSG0000014	-	-	ENSG0000014045	'USP3	-	15:63504510-63602428	-	-	IGV_LINK	659.41	21.3902	2.31813	40.4622	OK	574.392	18.9569	2.4477	35.4662	OK	OK	-0.17422
ENSG0000014	-	-	ENSG0000014045	'CYP11A1	-	15:74337758-74371211	-	-	IGV_LINK	2.90916	0.157835	0	0.6817	OK	0	0	0	0	OK	NOTEST	'-inf
ENSG0000014	-	-	ENSG0000014046	'BBS4	-	15:72686178-72738476	-	-	IGV_LINK	668.137	24.4009	6.58771	42.2141	OK	716.701	26.3424	6.94339	45.7414	OK	OK	0.110449
ENSG0000014	-	-	ENSG0000014046	'PML	-	15:73994672-74047812	-	-	IGV_LINK	2479.58	46.4484	8.68933	84.2075	OK	3075.11	53.7609	14.1102	93.4116	OK	OK	0.210927
ENSG0000014	-	-	ENSG0000014046	'CYP1A1	-	15:74719541-74725610	-	-	IGV_LINK	0	0	0	0	OK	0	0	0	0	OK	NOTEST	0
ENSG0000014	-	-	ENSG0000014047	'ADAMTS17	-	15:99970214-100342005	-	-	IGV_LINK	50.4255	0.918583	0	2.45477	OK	50.53	0.742873	0	2.07958	OK	OK	-0.30629
ENSG0000014	-	-	ENSG0000014047	'LINS1	-	15:100553528-100651705	-	-	IGV_LINK	305.462	10.028	0	22.1588	OK	260.9	10.3193	0	25.3033	OK	OK	0.041319
ENSG0000014	-	-	ENSG0000014047	'ULK3	-	15:74836115-74843346	-	-	IGV_LINK	1198.57	32.4163	9.43181	55.4007	OK	1158.07	29.9696	9.2958	50.6435	OK	OK	-0.11322
ENSG0000014	-	-	ENSG0000014047	'GOLGA6D	-	15:75282834-75295807	-	-	IGV_LINK	11.6366	0.215899	0	0.49739	OK	11.3435	0.210539	0	0.487101	OK	NOTEST	-0.03627
ENSG0000014	-	-	ENSG0000014047	'PCSK6	-	15:101295418-101525202	-	-	IGV_LINK	764.346	13.7574	1.79002	25.7247	OK	3230.64	51.3476	13.0099	89.6853	OK	OK	1.90009
ENSG0000014	-	-	ENSG0000014048	'CCDC33	-	15:74179465-74336472	-	-	IGV_LINK	0	0	0	0	OK	0	0	0	0	OK	NOTEST	0
ENSG0000014	-	-	ENSG0000014048	'CELF6	-	15:72241180-72378788	-	-	IGV_LINK	173.932	3.32352	0	15.6729	OK	172.281	3.26916	0	16.3672	OK	OK	-0.02379
ENSG0000014	-	-	ENSG0000014049	'SCAMP2	-	15:74843729-74873365	-	-	IGV_LINK	2346.72	69.4132	23.4448	115.382	OK	2565.69	75.6287	25.6565	125.601	OK	OK	0.123724
ENSG0000014	-	-	ENSG0000014050	'CYP1A2	-	15:74748843-74756202	-	-	IGV_LINK	0	0	0	0	OK	0	0	0	0	OK	NOTEST	0
ENSG0000014	-	-	ENSG0000014050	'LMAN1L	-	15:74812715-74831802	-	-	IGV_LINK	2.60602	0.298016	0	1.26078	OK	1.03122	0.148422	0	0.840061	OK	NOTEST	-1.00568
ENSG0000014	-	-	ENSG0000014051	'HAPLN3	-	15:88877287-88895626	-	-	IGV_LINK	616.742	16.9068	5.35492	28.4586	OK	255.744	7.37039	1.15899	13.5818	OK	OK	-1.19779
ENSG0000014	-	-	ENSG0000014051	'RHCG	-	15:89471397-89496613	-	-	IGV_LINK	0	0	0	0	OK	1.03122	0.069157	0	0.38114	OK	NOTEST	inf
ENSG0000014	-	-	ENSG0000014052	'POLG	-	15:89243948-89334861	-	-	IGV_LINK	1673.74	38.6112	0	77.6638	OK	2695.62	57.4882	0.596766	114.38	OK	OK	0.574248
ENSG0000014	-	-	ENSG0000014052	'RLBP1	-	15:89209868-89221751	-	-	IGV_LINK	0.969721	0.033374	0	0.434836	OK	0	0	0	0	OK	NOTEST	'-inf
ENSG0000014	-	-	ENSG0000014052	'FANCI	-	15:89243948-89334861	-	-	IGV_LINK	376.252	7.18248	0	23.1001	OK	378.46	5.83598	0	19.9836	OK	OK	-0.29951
ENSG0000014	-	-	ENSG0000014052	'ABHD2	-	15:89087077-89202360	-	-	IGV_LINK	1895.8	16.6512	0	39.2349	OK	2792.56	19.9934	1.73272	38.2541	OK	OK	0.263901
ENSG0000014	-	-	ENSG0000014052	'WDR93	-	15:89690796-89743638	-	-	IGV_LINK	101.821	2.77296	0.279243	5.26668	OK	90.7478	2.54039	0	5.35556	OK	OK	-0.12638
ENSG0000014	-	-	ENSG0000014053	'TICRR	-	15:89505276-89655451	-	-	IGV_LINK	53.3346	0.739471	0	1.91559	OK	91.779	1.15374	0	2.81688	OK	OK	0.641755
ENSG0000014	-	-	ENSG0000014053	'NTRK3	-	15:87859750-88271066	-	-	IGV_LINK	125.094	2.26064	0	7.38644	OK	8.2498	0.28859	0	1.29611	OK	OK	-2.96964
ENSG0000014	-	-	ENSG0000014054	'DET1	-	15:88459475-88546675	-	-	IGV_LINK	188.126	4.46434	0	10.9374	OK	100.029	2.52689	0	8.79736	OK	OK	-0.82108
ENSG0000014	-	-	ENSG0000014054	'MFGE8	-	15:88898682-88913411	-	-	IGV_LINK	994.933	44.0189	12.5247	75.5131	OK	750.732	29.7719	7.50094	52.0428	OK	OK	-0.56417
ENSG0000014	-	-	ENSG0000014054	'ZNF710	-	15:90001391-90082207	-	-	IGV_LINK	666.198	16.379	0	39.2569	OK	575.424	12.3574	0	27.8608	OK	OK	-0.40648
ENSG0000014	-	-	ENSG0000014055	'UNC45A	-	15:90929963-90963125	-	-	IGV_LINK	1473.98	34.4367	6.8845	61.9889	OK	1641.71	41.7918	6.54114	77.0425	OK	OK	0.279275
ENSG0000014	-	-	ENSG0000014055	'ST8SIA2	-	15:92393827-92468728	-	-	IGV_LINK	0	0	0	0	OK	4.1249	0.091027	0	0.401634	OK	NOTEST	inf
ENSG0000014	-	-	ENSG0000014056	'MCTP2	-	15:94231537-94483952	-	-	IGV_LINK	631.288	10.7167	0	21.502	OK	440.333	6.79301	0	14.2777	OK	OK	-0.65774
ENSG0000014	-	-	ENSG0000014056	'FURIN	-	15:90868591-90883458	-	-	IGV_LINK	1300.4	18.5193	6.84119	30.1973	OK	1724.21	24.7353	8.64384	40.8267	OK	OK	0.417544
ENSG0000014	-	-	ENSG0000014057	'IQGAP1	-	15:90388217-90502243	-	-	IGV_LINK	4239.62	62.5578	0	138.732	OK	4684.86	72.2815	0	161.282	OK	OK	0.208437
ENSG0000014	-	-	ENSG0000014057	'CRTC3	-	15:90529924-90717141	-	-	IGV_LINK	1014.33	14.6696	2.34392	26.9952	OK	1114.75	16.3181	3.21509	29.4211	OK	OK	0.153644
ENSG0000014	-	-	ENSG0000014059	'EFL1	-	15:82130229-82262763	-	-	IGV_LINK	448.981	8.22451	0.810968	15.6381	OK	529.018	13.4939	0	71.5586	OK	OK	0.714308
ENSG0000014	-	-	ENSG0000014060	'SH3GL3	-	15:83447227-83618743	-	-	IGV_LINK	1.93944	0.066916	0	0.424143	OK	1.03122	0.058328	0	0.411302	OK	NOTEST	-0.19815
ENSG0000014	-	-	ENSG0000014061	'SEC11A	-	15:84669537-84716716	-	-	IGV_LINK	1847.32	103.565	39.6699	167.46	OK	1612.84	88.415	28.6785	148.152	OK	OK	-0.22817
ENSG0000014	-	-	ENSG0000014062	'SEPT12	-	16:4777668-4796532	-	-	IGV_LINK	0	0	0	0	OK	1.03122	0.04602	0	0.538325	OK	NOTEST	inf
ENSG0000014	-	-	ENSG0000014063	'GLYR1	-	16:4803202-4882360	-	-	IGV_LINK	1981.14	40.46	0	84.8075	OK	2201.67	44.605	0	90.8037	OK	OK	0.14071
ENSG0000014	-	-	ENSG0000014065	'PMM2	-	16:8674564-8869012	-	-	IGV_LINK	566.109	15.4393	0	37.3537	OK	1246.89	33.5113	0	74.4032	OK	OK	1.11804
ENSG0000014	-	-	ENSG0000014067	'SLC5A2	-	16:31483001-31509309	-	-	IGV_LINK	1.93944	0.047635	0	1.08372	OK	3.09367	0.170181	0	3.25957	OK	NOTEST	1.83697
ENSG0000014	-	-	ENSG0000014067	'ITGAX	-	16:31355133-31382997	-	-	IGV_LINK	2674.49	48.7934	0.944247	96.6425	OK	2690.47	48.4142	1.97452	94.8538	OK	OK	-0.01126
ENSG0000014	-	-	ENSG0000014068	'TGFB1I1	-	16:31470203-31477960	-	-	IGV_LINK	192.005	6.81515	0.7374	12.8929	OK	149.528	5.93855	0	11.9069	OK	OK	-0.19863
ENSG0000014	-	-	ENSG0000014068	'C16orf58	-	16:31483001-31509309	-	-	IGV_LINK	2035.44	49.5739	18.5629	80.5849	OK	1964.48	49.0059	17.608	80.4038	OK	OK	-0.01663
ENSG0000014	-	-	ENSG0000014069	'ARMC5	-	16:31456710-31467166	-	-	IGV_LINK	273.461	4.30937	0.609495	8.00925	OK	321.742	5.88519	0.239789	11.5306	OK	OK	0.449611
ENSG0000014	-	-	ENSG0000014069	'PARN	-	16:14435700-14632728	-	-	IGV_LINK	703.047	22.723	0.167327	45.2786	OK	887.885	27.2165	2.03426	52.3987	OK	OK	0.260327
ENSG0000014	-	-	ENSG0000014071	'FTO	-	16:53597682-54158512	-	-	IGV_LINK	2576.55	84.2452	21.8365	146.654	OK	1764.43	58.0172	18.5494	97.4849	OK	OK	-0.53811
ENSG0000014	-	-	ENSG0000014074	'UQCRC2	-	16:21952659-21983660	-	-	IGV_LINK	3361.05	148.742	46.8333	250.651	OK	3154.52	136.501	44.2457	228.756	OK	OK	-0.12391
ENSG0000014	-	-	ENSG0000014074	'CDR2	-	16:22345935-22536521	-	-	IGV_LINK	541.809	16.1799	0	39.2071	OK	720.999	18.8021	0	37.7562	OK	OK	0.216685
ENSG0000014	-	-	ENSG0000014074	'IGSF6	-	16:21597217-21657473	-	-	IGV_LINK	812.626	21.7452	0	59.2296	OK	581.611	22.6176	0	75.5938	OK	OK	0.056745
ENSG0000014	-	-	ENSG0000014075	'ARHGAP17	-	16:24919384-25015666	-	-	IGV_LINK	994.933	24.3018	7.25171	41.3519	OK	833.23	20.5895	5.39388	35.7852	OK	OK	-0.23915
ENSG0000014	-	-	ENSG0000014079	'MYLK3	-	16:46703368-46792040	-	-	IGV_LINK	13.5761	0.350937	0	1.298	OK	8.2498	0.069007	0	0.503377	OK	NOTEST	-2.3464
ENSG0000014	-	-	ENSG0000014079	'ABCC12	-	16:48082972-48156018	-	-	IGV_LINK	0	0	0	0	OK	0	0	0	0	OK	NOTEST	0
ENSG0000014	-	-	ENSG0000014080	'NKD1	-	16:50548329-50649249	-	-	IGV_LINK	22.3036	0.165657	0	0.925216	OK	25.7806	0.206052	0	0.878574	OK	NOTEST	0.314812
ENSG0000014	-	-	ENSG0000014082	'DHX38	-	16:72044288-72176878	-	-	IGV_LINK	1374.09	33.5306	0	70.3291	OK	1580.87	35.1608	0	307.979	OK	OK	0.068488
ENSG0000014	-	-	ENSG0000014083	'TXNL4B	-	16:72044288-72176878	-	-	IGV_LINK	233.703	7.05165	0	22.367	OK	383.616	10.0349	0	110.194	OK	OK	0.508997
ENSG0000014	-	-	ENSG0000014083	'MARVELD3	-	16:71623707-71724701	-	-	IGV_LINK	72.729	2.43861	0	6.79506	OK	67.0296	1.73309	0	4.5665	OK	OK	-0.49272
ENSG0000014	-	-	ENSG0000014083	'CHST4	-	16:71447596-71565089	-	-	IGV_LINK	8.72748	0.311917	0	1.8906	OK	0	0	0	0	OK	NOTEST	'-inf
ENSG0000014	-	-	ENSG0000014083	'ZFHX3	-	16:72665169-73144447	-	-	IGV_LINK	671.047	3.08276	0	7.56949	OK	523.862	2.00158	0.474171	3.529	OK	OK	-0.62308
ENSG0000014	-	-	ENSG0000014083	'CLEC18B	-	16:74408269-74421953	-	-	IGV_LINK	310.311	21.8953	0.096937	43.6938	OK	163.965	10.3163	0	22.9366	OK	OK	-1.08569
ENSG0000014	-	-	ENSG0000014084	'CPNE2	-	16:57092536-57148367	-	-	IGV_LINK	745.715	25.2587	7.72305	42.7944	OK	876.541	27.9597	10.1904	45.7291	OK	OK	0.14657
ENSG0000014	-	-	ENSG0000014085	'NLRC5	-	16:56989484-57083531	-	-	IGV_LINK	3295.11	71.7119	0	144.893	OK	1453	32.0094	0	64.3999	OK	OK	-1.16372
ENSG0000014	-	-	ENSG0000014085	'KATNB1	-	16:57735729-57757250	-	-	IGV_LINK	367.524	11.8643	2.07878	21.6498	OK	427.958	14.1051	2.53404	25.6761	OK	OK	0.249589
ENSG0000014	-	-	ENSG0000014085	'KIFC3	-	16:57758216-57863053	-	-	IGV_LINK	1322.7	38.4069	6.61108	70.2026	OK	1277.69	39.6853	5.63952	73.7312	OK	OK	0.047242
ENSG0000014	-	-	ENSG0000014087	'ADAMTS18	-	16:77190834-77444336	-	-	IGV_LINK	15.5155	0.252874	0	2.50966	OK	31.968	0.447413	0	3.31096	OK	NOTEST	0.823192
ENSG0000014	-	-	ENSG0000014087	'NUDT7	-	16:77722491-77743000	-	-	IGV_LINK	53.3346	4.5055	0	9.92796	OK	70.1233	6.08733	0	13.0542	OK	OK	0.434123
ENSG0000014	-	-	ENSG0000014090	'GCSH	-	16:80966447-81096425	-	-	IGV_LINK	296.149	16.9088	0	46.6265	OK	330.321	16.6548	0	44.0374	OK	OK	-0.02183
ENSG0000014	-	-	ENSG0000014093	'CMTM3	-	16:66603873-66613892	-	-	IGV_LINK	873.718	36.5149	12.3224	60.7074	OK	609.454	27.3739	8.87939	45.8683	OK	OK	-0.41569
ENSG0000014	-	-	ENSG0000014093	'CMTM2	-	16:66579447-66588275	-	-	IGV_LINK	5.81832	0.718443	0	1.83282	OK	64.9672	9.8657	0	20.0243	OK	OK	3.77948
ENSG0000014	-	-	ENSG0000014093	'CDH11	-	16:64943752-65126112	-	-	IGV_LINK	268.613	9.10003	0	19.296	OK	1060.1	35.0864	3.84719	66.3256	OK	OK	1.94697
ENSG0000014	-	-	ENSG0000014093	'NOL3	-	16:67170153-67184040	-	-	IGV_LINK	2248.78	132.594	25.7914	239.397	OK	2332.63	136.787	31.8929	241.682	OK	OK	0.044915
ENSG0000014	-	-	ENSG0000014094	'MAP1LC3B	-	16:87083561-87404779	-	-	IGV_LINK	1274.21	29.3718	7.70812	51.0355	OK	1549.93	37.8835	9.80169	65.9653	OK	OK	0.367138
ENSG0000014	-	-	ENSG0000014094	'MBTPS1	-	16:84053760-84116906	-	-	IGV_LINK	2333.15	39.7962	7.35466	72.2378	OK	2457.41	38.5036	10.7434	66.2639	OK	OK	-0.04764
ENSG0000014	-	-	ENSG0000014094	'CDH13	-	16:82626802-83807834	-	-	IGV_LINK	808.747	15.8489	3.66757	28.0303	OK	955.946	21.3041	5.3542	37.254	OK	OK	0.426746
ENSG0000014	-	-	ENSG0000014094	'ZCCHC14	-	16:87406245-87492045	-	-	IGV_LINK	859.172	9.0715	0	19.0116	OK	450.645	4.79438	0	10.4421	OK	OK	-0.92
ENSG0000014	-	-	ENSG0000014095	'TLDC1	-	16:84476420-84554033	-	-	IGV_LINK	326.796	7.46592	0	16.1084	OK	375.366	9.62037	0.08501	19.1557	OK	OK	0.365773
ENSG0000014	-	-	ENSG0000014095	'ADAD2	-	16:84191137-84197168	-	-	IGV_LINK	0.969721	0.023025	0	0.466966	OK	0	0	0	0	OK	NOTEST	'-inf
ENSG0000014	-	-	ENSG0000014096	'OSGIN1	-	16:83899125-83966332	-	-	IGV_LINK	81.4565	2.7959	0	7.74484	OK	420.74	14.6081	0	30.2056	OK	OK	2.38538
ENSG0000014	-	-	ENSG0000014096	'IRF8	-	16:85898802-85922609	-	-	IGV_LINK	1843.44	46.8643	9.43191	84.2968	OK	1060.1	30.9886	6.24304	55.7342	OK	OK	-0.59675
ENSG0000014	-	-	ENSG0000014098	'RHOT2	-	16:668085-674174	-	-	IGV_LINK	1837.62	61.0128	18.6364	103.389	OK	1993.36	66.3068	18.6037	114.01	OK	OK	0.120046
ENSG0000014	-	-	ENSG0000014098	'RPL3L	-	16:1943973-1957606	-	-	IGV_LINK	0	0	0	0	OK	6.18735	0.173623	0	0.54731	OK	NOTEST	inf
ENSG0000014	-	-	ENSG0000014098	'ZSCAN32	-	16:3365098-3577400	-	-	IGV_LINK	265.703	7.65529	0	21.8878	OK	374.335	11.1082	0	28.9998	OK	OK	0.53709
ENSG0000014	-	-	ENSG0000014098	'RPS2	-	16:1962051-1964860	-	-	IGV_LINK	17490.7	1437.54	188.583	2686.49	OK	14955	1186.41	280.521	2092.3	OK	OK	-0.277

ENSG0000014	-	-	ENSG0000014099	NDUFB10	-	16:1959507-1961975	-	-	IGV_LINK	932.871	96.8469	38.9592	154.735	OK	935.321	99.9726	41.76	158.185	OK	OK	0.045827
ENSG0000014	-	-	ENSG0000014099	PDPK1	-	16:2537963-2603188	-	-	IGV_LINK	1085.12	16.3303	0	41.5667	OK	1210.66	18.9955	0	47.7609	OK	OK	0.218103
ENSG0000014	-	-	ENSG0000014099	TIGD7	-	16:3263742-3318852	-	-	IGV_LINK	201.702	4.74356	0	16.8239	OK	176.339	3.6113	0	12.7126	OK	OK	-0.39345
ENSG0000014	-	-	ENSG0000014099	DEF8	-	16:89947924-89968060	-	-	IGV_LINK	885.355	25.3175	2.91821	47.7167	OK	1172.5	33.9284	6.64197	61.2149	OK	OK	0.422362
ENSG0000014	-	-	ENSG0000014100	TCF25	-	16:89873569-89938761	-	-	IGV_LINK	2273.53	94.8335	16.8272	172.84	OK	2660.03	106.938	24.1155	189.76	OK	OK	0.173302
ENSG0000014	-	-	ENSG0000014101	GALNS	-	16:88813733-88862686	-	-	IGV_LINK	521.71	16.1353	0	38.8395	OK	730.107	22.2048	0	47.1533	OK	OK	0.460653
ENSG0000014	-	-	ENSG0000014101	GAS8	-	16:90004864-90047773	-	-	IGV_LINK	690.441	26.8168	0	55.5489	OK	740.42	30.2912	0.820323	59.762	OK	OK	0.175758
ENSG0000014	-	-	ENSG0000014102	MED9	-	17:17476985-17493226	-	-	IGV_LINK	398.555	10.9404	3.59582	18.2849	OK	480.551	12.9231	4.78351	21.0627	OK	OK	0.240291
ENSG0000014	-	-	ENSG0000014102	NCOR1	-	17:15976559-16353656	-	-	IGV_LINK	2658.97	43.8933	0	104.635	OK	2148.04	38.5685	0	91.691	OK	OK	-0.18658
ENSG0000014	-	-	ENSG0000014102	CDRT15P1	-	17:13756477-14069495	-	-	IGV_LINK	0.969721	0.139596	0	1.52037	OK	2.06245	0.297777	0	2.19131	OK	NOTEST	1.09297
ENSG0000014	-	-	ENSG0000014103	COPS3	-	17:17246819-17281293	-	-	IGV_LINK	511.043	23.2651	5.31635	41.2139	OK	559.955	23.8247	7.83151	39.8179	OK	OK	0.034289
ENSG0000014	-	-	ENSG0000014103	GID4	-	17:18039291-18068404	-	-	IGV_LINK	256.006	3.85387	0.431779	7.27595	OK	234.088	3.84871	0	7.93844	OK	OK	-0.00193
ENSG0000014	-	-	ENSG0000014104	ZNF287	-	17:16551386-16569206	-	-	IGV_LINK	70.7896	2.08293	0	5.56281	OK	79.4043	2.55474	0	6.53241	OK	OK	0.29456
ENSG0000014	-	-	ENSG0000014105	MYOCD	-	17:12665889-12991643	-	-	IGV_LINK	17.455	0.152362	0	0.649374	OK	21.6557	0.216378	0	0.819725	OK	NOTEST	0.506051
ENSG0000014	-	-	ENSG0000014106	KSR1	-	17:27456713-27649560	-	-	IGV_LINK	2203.49	52.2433	0	142.957	OK	2020.62	46.4583	0	127.31	OK	OK	-0.16931
ENSG0000014	-	-	ENSG0000014107	UTP4	-	16:69105563-69408571	-	-	IGV_LINK	806.807	23.6704	0	72.7912	OK	694.014	21.545	0	73.123	OK	OK	-0.13574
ENSG0000014	-	-	ENSG0000014108	RANBP10	-	16:67723065-67806652	-	-	IGV_LINK	1178.21	16.811	4.7813	28.8406	OK	1020.91	13.9809	4.1475	23.8144	OK	OK	-0.26594
ENSG0000014	-	-	ENSG0000014108	CTRL	-	16:67893271-67937087	-	-	IGV_LINK	59.1572	4.0338	0	27.866	OK	31.803	2.93291	0	24.4228	OK	OK	-0.4598
ENSG0000014	-	-	ENSG0000014109	DPEP3	-	16:67975662-67980829	-	-	IGV_LINK	36.8494	1.12528	0	2.34789	OK	72.1857	2.20572	0.098257	4.31318	OK	OK	0.970968
ENSG0000014	-	-	ENSG0000014109	GFOD2	-	16:67674530-67719421	-	-	IGV_LINK	431.526	9.04755	2.58291	15.5122	OK	491.894	11.4465	2.1722	20.7208	OK	OK	0.339308
ENSG0000014	-	-	ENSG0000014110	NOB1	-	16:69727012-69754940	-	-	IGV_LINK	1063.78	41.3842	16.6155	66.1528	OK	1061.13	40.1215	15.8767	64.3663	OK	OK	-0.0447
ENSG0000014	-	-	ENSG0000014112	PRPSAP2	-	17:18835706-18931287	-	-	IGV_LINK	439.283	20.0116	5.5086	34.5146	OK	387.741	16.8587	4.23113	29.4863	OK	OK	-0.24734
ENSG0000014	-	-	ENSG0000014116	UNC45B	-	17:35147816-35191702	-	-	IGV_LINK	0.969721	0.009981	0	0.066619	OK	1.03122	0.024425	0	0.098216	OK	NOTEST	1.29105
ENSG0000014	-	-	ENSG0000014117	PCTP	-	17:55750978-55872939	-	-	IGV_LINK	379.161	12.1023	3.60548	20.5991	OK	659.984	20.8477	7.68485	34.0105	OK	OK	0.784607
ENSG0000014	-	-	ENSG0000014119	OR4D1	-	17:58148448-58159555	-	-	IGV_LINK	3.87888	0.063372	0	0.21189	OK	0	0	0	0	OK	NOTEST	'-inf
ENSG0000014	-	-	ENSG0000014119	TOM1L1	-	17:54899386-55173632	-	-	IGV_LINK	958.084	47.6088	0	99.7424	OK	1066.29	48.489	0.632004	96.346	OK	OK	0.02643
ENSG0000014	-	-	ENSG0000014120	KIF2B	-	17:53822900-53825213	-	-	IGV_LINK	0	0	0	0	OK	0	0	0	0	OK	NOTEST	0
ENSG0000014	-	-	ENSG0000014121	C17orf80	-	17:73164976-73262352	-	-	IGV_LINK	516.861	9.17175	0	21.2209	OK	541.393	10.3945	0	23.872	OK	OK	0.180548
ENSG0000014	-	-	ENSG0000014123	TOB1	-	17:50862222-50910232	-	-	IGV_LINK	308.371	13.2884	2.0907	24.4862	OK	522.831	22.3518	7.15156	37.552	OK	OK	0.750218
ENSG0000014	-	-	ENSG0000014125	VPS53	-	17:503170-721717	-	-	IGV_LINK	555.65	7.92528	0	20.6651	OK	463.02	5.75203	0	14.9654	OK	OK	-0.46239
ENSG0000014	-	-	ENSG0000014125	SPATA22	-	17:3439922-3557995	-	-	IGV_LINK	8.18E-16	6.11E-16	0	1.56226	OK	1.72E-37	6.44E-39	0	0.631246	OK	NOTEST	-76.3285
ENSG0000014	-	-	ENSG0000014125	SGSM2	-	17:2054153-2381058	-	-	IGV_LINK	1088.03	25.2246	0	60.2228	OK	1055.97	21.464	0	51.8315	OK	OK	-0.23291
ENSG0000014	-	-	ENSG0000014127	NPEPPS	-	17:47522941-47649420	-	-	IGV_LINK	2366.12	57.6999	0	116.608	OK	2965.8	72.2879	0	144.578	OK	OK	0.325185
ENSG0000014	-	-	ENSG0000014129	SKAP1	-	17:48133439-48430275	-	-	IGV_LINK	634.197	26.2502	10.5788	41.9215	OK	448.583	19.2	6.07427	32.3258	OK	OK	-0.45122
ENSG0000014	-	-	ENSG0000014129	LRRC46	-	17:47831626-47841333	-	-	IGV_LINK	50.4255	1.51344	0	5.13923	OK	57.7486	1.89408	0	6.81589	OK	OK	0.323667
ENSG0000014	-	-	ENSG0000014129	SCRN2	-	17:47831626-47841333	-	-	IGV_LINK	804.868	34.6867	10.9204	58.4529	OK	1015.76	45.2911	14.9116	75.6706	OK	OK	0.384846
ENSG0000014	-	-	ENSG0000014129	SSH2	-	17:29560546-30186475	-	-	IGV_LINK	1385.73	13.8612	0	48.5737	OK	1769.58	19.2032	0	68.0576	OK	OK	0.470292
ENSG0000014	-	-	ENSG0000014131	RHBDL3	-	17:32266175-32381886	-	-	IGV_LINK	9.69721	0.127165	0	1.50102	OK	2.06245	0.027051	0	0.646073	OK	NOTEST	-2.23297
ENSG0000014	-	-	ENSG0000014131	SPACA3	-	17:32970375-32997877	-	-	IGV_LINK	0	0	0	0	OK	0	0	0	0	OK	NOTEST	0
ENSG0000014	-	-	ENSG0000014133	ARSG	-	17:68259181-68763882	-	-	IGV_LINK	252.015	5.38116	0	22.5607	OK	202.601	4.80774	0	31.5619	OK	OK	-0.16256
ENSG0000014	-	-	ENSG0000014133	ABCA8	-	17:68867291-69061064	-	-	IGV_LINK	21.3339	0.34062	0	1.24002	OK	31.968	0.968912	0	4.23719	OK	OK	1.5082
ENSG0000014	-	-	ENSG0000014134	G6PC3	-	17:44070734-44076344	-	-	IGV_LINK	811.656	43.3444	16.9182	69.7706	OK	1107.54	55.9337	21.9708	89.8965	OK	OK	0.367871
ENSG0000014	-	-	ENSG0000014136	CLTC	-	17:59618552-59842255	-	-	IGV_LINK	4249.32	54.3291	0	122.54	OK	5956.36	77.4947	0	193.509	OK	OK	0.512372
ENSG0000014	-	-	ENSG0000014137	C17orf64	-	17:60179093-60431421	-	-	IGV_LINK	3.87888	0.281162	0	2.61236	OK	10.3122	1.08332	0	7.88476	OK	OK	1.94599
ENSG0000014	-	-	ENSG0000014137	BCAS3	-	17:60677452-61411555	-	-	IGV_LINK	504.255	9.40286	0	25.8825	OK	461.989	9.62156	0	24.4561	OK	OK	0.033172
ENSG0000014	-	-	ENSG0000014137	PTRH2	-	17:59618552-59842255	-	-	IGV_LINK	250.188	14.4317	0	76.0181	OK	360.929	21.0764	0	138.975	OK	OK	0.546391
ENSG0000014	-	-	ENSG0000014138	SS18	-	18:26016252-26091217	-	-	IGV_LINK	1169.48	29.7544	8.73398	50.7748	OK	1246.75	30.2742	9.3044	51.2441	OK	OK	0.024989
ENSG0000014	-	-	ENSG0000014138	TAF4B	-	18:26225935-26391685	-	-	IGV_LINK	277.34	3.66155	0.942992	6.3801	OK	278.431	3.68101	0.963605	6.39841	OK	OK	0.007648
ENSG0000014	-	-	ENSG0000014138	AFG3L2	-	18:12307668-12377314	-	-	IGV_LINK	1794.95	34.6886	8.87028	60.5069	OK	2782.24	55.9619	7.01507	104.909	OK	OK	0.689983
ENSG0000014	-	-	ENSG0000014139	PRELID3A	-	18:12407895-12432238	-	-	IGV_LINK	64.9713	3.70377	0	7.87063	OK	90.7478	4.85769	0	10.1201	OK	OK	0.391277
ENSG0000014	-	-	ENSG0000014140	IMPA2	-	18:11981024-12030883	-	-	IGV_LINK	2487.33	130.205	45.6467	214.762	OK	3004.99	157.582	57.6296	257.535	OK	OK	0.275327
ENSG0000014	-	-	ENSG0000014140	GNAL	-	18:11688955-11909223	-	-	IGV_LINK	160.974	2.1062	0	9.17931	OK	168.09	2.79718	0	11.9819	OK	OK	0.409332
ENSG0000014	-	-	ENSG0000014142	SLC39A6	-	18:36108531-36129385	-	-	IGV_LINK	1133.6	26.1529	6.80168	45.5042	OK	1563.34	38.6008	10.0328	67.1688	OK	OK	0.561659
ENSG0000014	-	-	ENSG0000014142	RPRD1A	-	18:35984386-36067576	-	-	IGV_LINK	700.138	15.2642	4.03522	26.4931	OK	699.171	14.342	4.25293	24.4311	OK	OK	-0.0899
ENSG0000014	-	-	ENSG0000014142	C18orf21	-	18:35972082-35979286	-	-	IGV_LINK	247.015	18.2276	4.28212	32.1731	OK	246.457	17.847	3.92242	31.7717	OK	OK	-0.03044
ENSG0000014	-	-	ENSG0000014142	GALNT1	-	18:35581116-35711834	-	-	IGV_LINK	654.561	16.8786	3.22394	30.5333	OK	880.666	23.1934	5.58749	40.7994	OK	OK	0.458518
ENSG0000014	-	-	ENSG0000014143	ASXL3	-	18:33578576-33751192	-	-	IGV_LINK	97.9418	0.49166	0	1.40623	OK	47.4363	0.367884	0	1.60198	OK	NOTEST	-0.41841
ENSG0000014	-	-	ENSG0000014143	ADCYAP1	-	18:894434-912172	-	-	IGV_LINK	0.969721	0.073308	0	0.37601	OK	4.1249	0.260952	0	0.795003	OK	NOTEST	1.83175
ENSG0000014	-	-	ENSG0000014143	MEP1B	-	18:32018371-32470484	-	-	IGV_LINK	2.90916	0.076722	0	1.0403	OK	0	0	0	0	OK	NOTEST	'-inf
ENSG0000014	-	-	ENSG0000014143	SLC25A52	-	18:31759561-31760880	-	-	IGV_LINK	2.90916	0.143001	0	0.383813	OK	1.03122	0.050741	0	0.189913	OK	NOTEST	-1.4948
ENSG0000014	-	-	ENSG0000014144	GAREM1	-	18:32018371-32470484	-	-	IGV_LINK	820.384	8.40703	0.605459	16.2086	OK	1067.32	10.5071	1.87061	19.1436	OK	OK	0.321699
ENSG0000014	-	-	ENSG0000014144	ESCO1	-	18:21529280-21600884	-	-	IGV_LINK	288.977	6.4783	0	13.6384	OK	280.493	6.4379	0	14.0001	OK	OK	-0.00902
ENSG0000014	-	-	ENSG0000014144	OSBPL1A	-	18:24138955-24397880	-	-	IGV_LINK	1059.04	28.4125	8.4561	48.3688	OK	890.599	25.39	7.02941	43.7505	OK	OK	-0.16227
ENSG0000014	-	-	ENSG0000014144	GATA6	-	18:22169442-22205229	-	-	IGV_LINK	11.6366	0.182333	0	0.41911	OK	32.9992	0.526754	0	1.13699	OK	NOTEST	1.53055
ENSG0000014	-	-	ENSG0000014144	GREB1L	-	18:21242241-21525417	-	-	IGV_LINK	30.0613	0.325532	0	1.22899	OK	97.9664	0.998201	0	3.12672	OK	OK	1.61653
ENSG0000014	-	-	ENSG0000014145	C18orf8	-	18:23503469-23586898	-	-	IGV_LINK	541.104	23.8758	0	60.0357	OK	545.518	20.4486	0	59.7952	OK	OK	-0.22355
ENSG0000014	-	-	ENSG0000014145	PELP1	-	17:4669773-4705529	-	-	IGV_LINK	543.043	13.146	0	26.5234	OK	501.175	11.4975	0.098582	22.8965	OK	OK	-0.1933
ENSG0000014	-	-	ENSG0000014145	NPC1	-	18:23503469-23586898	-	-	IGV_LINK	1480.76	33.7523	0	69.0923	OK	2353.26	54.6	0	113.002	OK	OK	0.693913
ENSG0000014	-	-	ENSG0000014146	SLC14A1	-	18:45212994-45752520	-	-	IGV_LINK	37.8191	1.33334	0	3.73763	OK	53.6237	1.14939	0	3.22866	OK	OK	-0.21417
ENSG0000014	-	-	ENSG0000014148	ARRB2	-	17:4710488-4721499	-	-	IGV_LINK	1537.01	76.1003	23.141	129.06	OK	1683.99	87.3443	26.6646	148.024	OK	OK	0.19881
ENSG0000014	-	-	ENSG0000014148	SLC13A5	-	17:6651717-6713567	-	-	IGV_LINK	2.90916	0.031375	0	0.561884	OK	4.1249	0.0445	0	0.577335	OK	NOTEST	0.504204
ENSG0000014	-	-	ENSG0000014149	ZMYND15	-	17:4740014-4746119	-	-	IGV_LINK	250.188	6.62715	1.6158	11.6385	OK	219.651	6.50774	1.13406	11.8814	OK	OK	-0.02623
ENSG0000014	-	-	ENSG0000014149	WRAP53	-	17:7661778-7703502	-	-	IGV_LINK	167.762	4.23839	0	10.2604	OK	153.653	4.48038	0	11.5571	OK	OK	0.080106
ENSG0000014	-	-	ENSG0000014150	MINK1	-	17:4833387-4903074	-	-	IGV_LINK	2189.63	39.3889	2.39662	76.3811	OK	2172.79	38.4691	1.85753	75.0807	OK	OK	-0.03409
ENSG0000014	-	-	ENSG0000014150	SAT2	-	17:7561874-7633383	-	-	IGV_LINK	760.261	54.3259	0	223.075	OK	532.112	38.7828	0	220.921	OK	OK	-0.48623
ENSG0000014	-	-	ENSG0000014150	ASGR1	-	17:7173430-7179564	-	-	IGV_LINK	40.7283	2.61791	0	5.96534	OK	37.1241	2.09656	0	4.40589	OK	OK	-0.32039
ENSG0000014	-	-	ENSG0000014150	PIK3R5	-	17:8878910-8965712	-	-	IGV_LINK	709.835	15.7183	1.91253	29.5241	OK	884.791	17.4909	3.41061	31.5712	OK	OK	0.154155
ENSG0000014	-	-	ENSG0000014151	TP53	-	17:7661778-7703502	-	-	IGV_LINK	443.162	10.5443	1.41196	19.6766	OK	423.833	10.1475	2.50324	17.7918	OK	OK	-0.05533
ENSG0000014	-	-	ENSG0000014151	CCDC40	-	17:80036631-80100613	-	-	IGV_LINK	244.37	8.82522	0	18.2354	OK	276.368	9.75269	0	19.5982	OK	OK	0.144168
ENSG0000014	-	-	ENSG0000014152	ARHGDIA	-	17:81867720-81871406	-	-	IGV_LINK	2757.89	107.51	30.4597	184.56	OK	3114.3	128.054	39.8145	216.293	OK	OK	0.25228
ENSG0000014	-	-	ENSG0000014152	TMC6	-	17:77959239-78142968	-	-	IGV_LINK	1027.41	31.9333	0	81.6052	OK	1178.48	39.5421	0	102.977	OK	OK	0.308328
ENSG0000014	-	-	ENSG0000014152	SLC16A3	-	17:82228396-82273731	-	-	IGV_LINK	3380.45	122.318	0	251.22	OK	6347.19	232.606	0	482.152	OK	OK	0.927258
ENSG0000014	-	-	ENSG0000014152	CARD14	-	17:80169991-80253500	-	-	IGV_LINK	91.1537	1.50284	0	5.92119	OK	741.451	17.6566	0	50.5399	OK	OK	3.55445
ENSG0000014	-	-	ENSG0000014154	TTYH2	-	17:74213513-74262020	-	-	IGV_LINK	290.916	6.81692	0	14.3638	OK	273.275	6.89519	0	14.9259	OK	OK	0.016471
ENSG0000014	-	-	ENSG0000014154	RAB40B	-	17:82654972-82698728	-	-	IGV_LINK	301.583	11.7159	0.795673	22.6361	OK	181.496	7.09144	0	14.5201	OK	OK	-0.72431
ENSG0000014	-	-	ENSG0000014154	EIF4A3	-	17:80135213-80147183	-	-	IGV_LINK	836.869	30.459	12.1711	48.747	OK	1064.22	38.1015	15.878	60.325	OK	OK	0.32298
ENSG0000014	-	-	ENSG0000014155	CSNK1D	-	17:82228396-82273731	-	-	IGV_LINK	2766.61	75.3561	0	187.351	OK	3553.6	98.7702	0	278.162	OK	OK	0.39035
ENSG0000014	-	-	ENSG0000014155	ANAPC11	-	17:81887843-81911464	-	-	IGV_LINK	523.649	56.3766	7.03718	105.716	OK	570.267	62.8525	9.10758	116.597	OK	OK	0.156872
ENSG0000014	-	-	ENSG0000014155	TBCD	-	17:82752063-83051810	-	-	IGV_LINK	1261.61	41.6702	6.21565	77.1247	OK	1223.03	42.9659	5.87063	80.0611	OK	OK	0.044175
ENSG0000014	-	-	ENSG0000014156	FN3KRP	-	17:82716682-82734143	-	-	IGV_LINK	769.958	29.0546	10.0297	48.0796	OK	823.949	29.7779	10.4029	49.153	OK	OK	0.035475
ENSG0000014	-	-	ENSG0000014156	NARF	-	17:82454272-82490537	-	-	IGV_LINK	927.053	34.6386	8.79989	60.4774	OK	808.48	32.3427	7.51536	57.17	OK	OK	-0.09894
ENSG0000014	-	-	ENSG0000014156	RPTOR	-	17:80544818-80971213	-	-	IGV_LINK	441.223	5.02234	1.22909	8.81558	OK	658.953	7.54352	2.40489	12.6822	OK	OK	0.586879
ENSG0000014	-	-	ENSG0000014156	FOXK2	-	17:82519712-82648553	-	-	IGV_LINK	493.588	9.53179	0	24.2115	OK	549.643	11.2146	0	28.5652	OK	OK	0.234558
ENSG0000014	-	-	ENSG0000014156	TRIM65	-	17:75880334-75897003	-	-	IGV_LINK	399.525	9.15073	0	19.0479	OK	394.959	8.43272	0.363801	16.5016	OK	OK	-0.11789
ENSG0000014	-	-	ENSG0000014157	CBX8	-	17:79792131-79802529	-	-	IGV_LINK	243.4	7.48726	0	16.2855	OK	241.307	7.77749	0	17.5259	OK	OK	0.054865
ENSG0000014	-	-	ENSG0000014157	SECTM1	-	17:82321023-82334074	-	-	IGV_LINK	620.621	23.1949	6.10933	40.2805	OK	582.642	23.6954	6.1753	41.2154	OK	OK	0.030796
ENSG0000014	-	-	ENSG0000014157	RNF157	-	17:76136332-76240373	-	-	IGV_LINK	304.492	3.96832	0.045561	7.89108	OK	383.616	4.62205	1.33885	7.90525	OK	OK	0.220004
ENSG0000014	-	-	ENSG0000014157	CEP131	-	17:81189592-81222999	-	-	IGV_LINK	359.766	8.43409	0.451327	16.4168	OK	313.492	9.41101	0.128061	18.694	OK	OK	0.158118
ENSG0000014	-	-	ENSG0000014157	ZNF750	-	17:82752063-83051810	-	-	IGV_LINK	5.81832	0.092613	0	0.938295	OK	2.06245	0.03284	0	0.54078	OK	NOTEST	-1.49578
ENSG0000014	-	-	ENSG0000014158	WDR45B	-	17:82519712-82648553	-	-	IGV_LINK	1400.28	38.2599	1.59157	74.9282	OK	1531.37	41.527	0	86.0277	OK	OK	0.118216
ENSG0000014	-	-	ENSG0000014158	CBX4	-	17:79833155-79839429	-	-	IGV_LINK	268.613	6.93145	0.575673	13.2872	OK	323.805	8.64381	0.824706	16.4629	OK	OK	0.31851
ENSG0000014	-	-	ENSG0000014162	RNF165	-	18:46326808-46463140	-	-	IGV_LINK	264.734	2.53166	0.426113	4.6372	OK	379.491	3.77788	0.902018	6.65375	OK	OK	0.577498
ENSG0000014	-	-	ENSG0000014162	DYM	-	18:49023702-49461347	-	-	IGV_LINK	892.143	21.8369	1.63015	42.0436	OK	629.047	17.2115	1.09685	33.3261	OK	OK	-0.3434
ENSG0000014	-	-	ENSG0000014163	MAPK4	-	18:50560077-50731824	-	-	IGV_LINK	0	0	0	0	OK	1.03122	0.013981	0	0.272784	OK	NOTEST	inf
ENSG0000014	-	-	ENSG0000014164	ELAC1	-	18:50967990-51085045	-	-	IGV_LINK	84.0206	4.33752	0	14.6441	OK	121.685	5.32299	0	16.1519	OK	OK	0.295367
ENSG0000014	-	-	ENSG0000014164	MBD1	-	18:50266881-50281774	-	-	IGV_LINK	1704.77	42.3274	2.95865	81.6961	OK	1642.74	39.9241	4.84731	75.0009	OK	OK	-0.08433
ENSG0000014	-	-	ENSG0000014164	SMAD4	-	18:50967990-51085045	-	-	IGV_LINK	979.793	25.3272	1.30755	49.3468	OK	1004.41	29.6694	0.766018	58.5728	OK	OK	0.22829
ENSG0000014	-	-	ENSG0000014165	TNFRSF11A	-	18:62325286-62391292	-	-	IGV_LINK	153.216	1.85871	0	4.10437	OK	227.901	2.4149	0	4.93429	OK	OK	0.377661
ENSG0000014	-	-	ENSG0000014166	ZCCHC2	-	18:62523006-62587709	-	-	IGV_LINK	534.316	7.79758	1.13079	14.4644	OK	767.231	11.1111	2.94343	19.2788	OK	OK	0.510907
ENSG0000014	-	-	ENSG0000014166	FBXO15	-	18:74073352-74147865	-	-	IGV_LINK	51.3952	2.26451	0	4.98142	OK	61.8735	3.66581	0	8.25473	OK	OK	0.694933
ENSG0000014	-	-	ENSG0000014166	CBLN2	-	18:72536679-72638521	-	-	IGV_LINK	0	0	0	0	OK	0	0	0	0	OK	NOTEST	0
ENSG0000014	-	-	ENSG0000014168	PMAIP1	-	18:59899947-59904306	-	-	IGV_LINK	44.6071	1.72283	0	3.74394	OK	95.9039	3.25931	0.297404	6.22122	OK	OK	0.919791
ENSG0000014	-	-	ENSG0000014169	P3H4	-	17:41801946-41812604	-	-	IGV_LINK	382.07	12.2753	1.91351	22.6371	OK	548.612	18.0219	3.17085	32.873	OK	OK	0.553993
ENSG0000014	-	-	ENSG0000014169	NT5C3B	-	17:41825180-41848384	-	-	IGV_LINK	735.048	48.3404	17.0233	79.6576	OK	616.673	37.4152	12.3924	62.438	OK	OK	-0.36961
ENSG0000014	-	-	ENSG0000014169	RETREG3	-	17:42579512-42615234	-	-	IGV_LINK	1272.27	25.4231	1.93531	48.9108	OK	1362.25	25.7627	2.19124	49.3342	OK	OK	0.019148
ENSG0000014	-	-	ENSG0000014173	ERBB2	-	17:39671121-39730787	-	-	IGV_LINK	1812.41	29.717	0	60.3483	OK	1593.24	26.0465	0	54.6745	OK	OK	-0.1902
ENSG0000014	-	-	ENSG0000014173	GRB7	-	17:39737926-39747291	-	-	IGV_LINK	412.131	17.1481	2.74332	31.5529	OK	442.396	19.0244	3.63405	34.4147	OK	OK	0.149801
ENSG0000014	-	-	ENSG0000014174	MIEN1	-	17:39671121-39730787	-	-	IGV_LINK	601.227	44.0902	0	112.879	OK	600.173	42.8283	0	95.8036	OK	OK	-0.0419
ENSG0000014	-	-	ENSG0000014174	PNMT	-	17:39667980-39670475	-	-	IGV_LINK	0	0	0	0	OK	1.03122	0.089777	0	0.408481	OK	NOTEST	inf
ENSG0000014	-	-	ENSG0000014174	ARL5C	-	17:39156893-39167849	-	-	IGV_LINK	0	0	0	0	OK	1.03122	0.15864	0	0.863974	OK	NOTEST	inf
ENSG0000014	-	-	ENSG0000014175	STAC2	-	17:39210535-39225872	-	-	IGV_LINK	2.90916	0.052782	0	0.154966	OK	1.03122	0.027976	0	0.112769	OK	NOTEST	-0.91587
ENSG0000014	-	-	ENSG0000014175	IGFBP4	-	17:40443460-40457731	-	-	IGV_LINK	6258.58	173.962	67.682	280.243	OK	13595.7	378.118	112.749	643.486	OK	OK	1.12006
ENSG0000014	-	-	ENSG0000014175	FKBP10	-	17:41812679-41823217	-	-	IGV_LINK	4610.05	138.618	36.7725	240.463	OK	7438.23	212.741	46.8767	378.606	OK	OK	0.617988
ENSG0000014	-	-	ENSG0000014175	TXNL4A	-	18:79964560-80033949	-	-	IGV_LINK	537.225	51.4885	7.99115	94.9857	OK	576.455	56.5784	9.90681	103.25	OK	OK	0.136003
ENSG0000014	-	-	ENSG0000014183	CACNA1A	-	19:13206441-13633025	-	-	IGV_LINK	51.3952	0.964977	0	2.46011	OK	49.4988	1.07228	0	2.84037	OK	OK	0.152113
ENSG0000014	-	-	ENSG0000014185	MISP3	-	19:14072535-14075062	-	-	IGV_LINK	64.9713	2.3717	0	5.28295	OK	94.8727	3.35122	0	6.95185	OK	OK	0.498766
ENSG0000014	-	-	ENSG0000014185	SAMD1	-	19:14087839-14091036	-	-	IGV_LINK	318.068	12.5926	1.91098	23.2743	OK	280.493	12.1168	0	24.7859	OK	OK	-0.05557
ENSG0000014	-	-	ENSG0000014186	BRD4	-	19:15235518-15332545	-	-	IGV_LINK	1181.12	16.9288	0	35.409	OK	1295.22	16.4466	0.662286	32.2309	OK	OK	-0.04169
ENSG0000014	-	-	ENSG0000014187	SLC39A3	-	19:2714566-2740152	-	-	IGV_LINK	192.974	8.38015	0.800525	15.9598	OK	278.669	15.1962	1.41256	28.9798	OK	OK	0.858663
ENSG0000014	-	-	ENSG0000014190	NFIC	-	19:3314402-3469217	-	-	IGV_LINK	1613.61	24.8703	4.21527	45.5253	OK	2090.29	31.1207	4.69741	57.544	OK	OK	0.323452
ENSG0000014	-	-	ENSG0000014193	TPGS1	-	19:489175-519654	-	-	IGV_LINK	175.519	10.0701	1.98893	18.1512	OK	153.653	7.97612	1.27095	14.6813	OK	OK	-0.33632
ENSG0000014	-	-	ENSG0000014193	PLPP2	-	19:281039-291504	-	-	IGV_LINK	321.947	15.7981	4.18647	27.4097	OK	671.327	31.7361	11.3828	52.0893	OK	OK	1.00637

ENSG0000014	-	-	ENSG0000014194	ZIM3	-	19:57134095-57145202	-	-	IGV_LINK	0	0	0	0	OK	0	0	0	0	OK	NOTEST	0
ENSG0000014	-	-	ENSG0000014195	PRDM15	-	21:41798224-41879482	-	-	IGV_LINK	241.46	3.04758	0	6.62822	OK	184.589	1.85343	0.117764	3.58909	OK	OK	-0.71747
ENSG0000014	-	-	ENSG0000014195	PFKL	-	21:44300050-44327376	-	-	IGV_LINK	6141.24	119.823	39.6426	200.003	OK	6686.46	130.234	38.1952	222.272	OK	OK	0.120201
ENSG0000014	-	-	ENSG0000014196	FEM1A	-	19:4791680-4801273	-	-	IGV_LINK	56.2438	0.336463	0	0.982874	OK	36.0929	0.215942	0	0.739304	OK	NOTEST	-0.6398
ENSG0000014	-	-	ENSG0000014196	VAV1	-	19:6772713-6857366	-	-	IGV_LINK	678.804	16.2156	4.95282	27.4783	OK	774.45	17.8712	6.86675	28.8757	OK	OK	0.14026
ENSG0000014	-	-	ENSG0000014197	MVB12A	-	19:17405693-17433724	-	-	IGV_LINK	641.602	43.8572	8.2433	79.471	OK	567.516	38.8889	7.55201	70.2257	OK	OK	-0.17346
ENSG0000014	-	-	ENSG0000014197	CIB3	-	19:16161367-16173525	-	-	IGV_LINK	1.93944	0.258704	0	0.96297	OK	0	0	0	0	OK	NOTEST	'-inf
ENSG0000014	-	-	ENSG0000014197	AC008764.1	-	19:16479056-16660442	-	-	IGV_LINK	2.56E-154	6.02E-156	0	0.047071	OK	1.6338	0.038471	0	1.27193	OK	NOTEST	510.931
ENSG0000014	-	-	ENSG0000014198	SH3GL1	-	19:4360369-4400547	-	-	IGV_LINK	1052.15	26.7171	10.999	42.4352	OK	1851.05	46.2122	19.6968	72.7276	OK	OK	0.790511
ENSG0000014	-	-	ENSG0000014199	DUS3L	-	19:5782959-5791238	-	-	IGV_LINK	424.738	20.5864	2.63276	38.5401	OK	423.833	18.7088	3.00556	34.4119	OK	OK	-0.13798
ENSG0000014	-	-	ENSG0000014200	DPP9	-	19:4675223-4724673	-	-	IGV_LINK	1724.16	38.2504	0.199828	76.301	OK	2156.29	44.6838	4.33478	85.0327	OK	OK	0.224276
ENSG0000014	-	-	ENSG0000014202	DMRTC2	-	19:41844742-41852333	-	-	IGV_LINK	0	0	0	0	OK	0	0	0	0	OK	NOTEST	0
ENSG0000014	-	-	ENSG0000014203	CCDC97	-	19:41262495-41425005	-	-	IGV_LINK	603.166	13.338	0	67.6589	OK	613.579	13.0748	0	62.5512	OK	OK	-0.02875
ENSG0000014	-	-	ENSG0000014204	TMEM91	-	19:41262495-41425005	-	-	IGV_LINK	1558.3	107.847	0	314.672	OK	958.684	70.0881	0	225.338	OK	OK	-0.62175
ENSG0000014	-	-	ENSG0000014206	ZFP14	-	19:36334452-36379199	-	-	IGV_LINK	283.158	3.89187	0	9.43656	OK	188.714	1.9809	0	4.85622	OK	OK	-0.97431
ENSG0000014	-	-	ENSG0000014208	SIRT3	-	11:215457-252984	-	-	IGV_LINK	499.406	18.0166	0	48.1274	OK	532.112	22.3035	0	58.4944	OK	OK	0.307944
ENSG0000014	-	-	ENSG0000014208	IFITM3	-	11:318639-330122	-	-	IGV_LINK	5704.87	506.754	200.509	812.999	OK	6331.72	563.624	218.775	908.472	OK	OK	0.153447
ENSG0000014	-	-	ENSG0000014210	PGGHG	-	11:289134-296107	-	-	IGV_LINK	5321.83	120.239	22.934	217.544	OK	4013.53	89.6908	19.6947	159.687	OK	OK	-0.42288
ENSG0000014	-	-	ENSG0000014214	HUNK	-	21:31873314-32044633	-	-	IGV_LINK	179.398	1.55456	0	3.36924	OK	250.588	1.95841	0.45749	3.45932	OK	OK	0.333177
ENSG0000014	-	-	ENSG0000014215	COL6A1	-	21:45981736-46005050	-	-	IGV_LINK	2682.25	40.5284	11.5308	69.526	OK	2359.44	34.5238	11.6917	57.3559	OK	OK	-0.23134
ENSG0000014	-	-	ENSG0000014216	OR1E3	-	17:3116426-3117374	-	-	IGV_LINK	0	0	0	0	OK	0	0	0	0	OK	NOTEST	0
ENSG0000014	-	-	ENSG0000014216	IFNAR1	-	21:33324476-33359862	-	-	IGV_LINK	1260.64	12.6823	2.60152	22.7631	OK	1593.24	23.6871	0	56.0417	OK	OK	0.901286
ENSG0000014	-	-	ENSG0000014216	SOD1	-	21:31659621-31668931	-	-	IGV_LINK	3184.56	225.109	96.6657	353.553	OK	4367.24	309.244	128.33	490.159	OK	OK	0.458121
ENSG0000014	-	-	ENSG0000014217	COL6A2	-	21:46098096-46132849	-	-	IGV_LINK	5278.19	105.679	9.14492	202.214	OK	4851.91	98.474	6.92563	190.022	OK	OK	-0.10188
ENSG0000014	-	-	ENSG0000014217	SIK1	-	21:43414514-43427128	-	-	IGV_LINK	4.8486	0.207712	0	0.783661	OK	5.15612	0.52764	0	1.46458	OK	NOTEST	1.34497
ENSG0000014	-	-	ENSG0000014218	DNMT3L	-	21:44246338-44262216	-	-	IGV_LINK	0	0	0	0	OK	0	0	0	0	OK	NOTEST	0
ENSG0000014	-	-	ENSG0000014218	TRPM2	-	21:44350162-44443081	-	-	IGV_LINK	736.018	11.9189	2.40594	21.4318	OK	647.609	9.6094	1.0942	18.1246	OK	OK	-0.31073
ENSG0000014	-	-	ENSG0000014218	SCYL1	-	11:65525076-65538704	-	-	IGV_LINK	1552.52	43.468	14.9956	71.9404	OK	1708.74	46.9775	16.447	77.5079	OK	OK	0.112016
ENSG0000014	-	-	ENSG0000014218	TMEM50B	-	21:33402895-33480011	-	-	IGV_LINK	510.073	17.9477	0	47.7579	OK	423.833	13.3341	0	36.7378	OK	OK	-0.42869
ENSG0000014	-	-	ENSG0000014219	APP	-	21:25880549-26217381	-	-	IGV_LINK	22165.9	458.648	81.7397	835.556	OK	24895.8	522.919	64.5566	981.282	OK	OK	0.189202
ENSG0000014	-	-	ENSG0000014219	DOPEY2	-	21:36131766-36294274	-	-	IGV_LINK	1344.03	11.3889	1.94766	20.8301	OK	1630.37	14.5161	0.383224	28.649	OK	OK	0.350026
ENSG0000014	-	-	ENSG0000014220	URB1	-	21:32291812-32393026	-	-	IGV_LINK	605.106	3.33838	0.406765	6.27	OK	833.23	4.40566	1.80008	7.01125	OK	OK	0.40021
ENSG0000014	-	-	ENSG0000014220	AKT1	-	14:104769348-104795751	-	-	IGV_LINK	1981.14	49.7607	11.6868	87.8347	OK	1911.89	48.0765	13.8113	82.3417	OK	OK	-0.04968
ENSG0000014	-	-	ENSG0000014222	IL19	-	1:206798869-206842981	-	-	IGV_LINK	0	0	0	0	OK	1.03122	0.072513	0	0.337708	OK	NOTEST	inf
ENSG0000014	-	-	ENSG0000014222	EMP3	-	19:48296456-48330553	-	-	IGV_LINK	1187.91	104.717	42.1068	167.327	OK	922.946	88.1227	33.8036	142.442	OK	OK	-0.24891
ENSG0000014	-	-	ENSG0000014223	SAE1	-	19:47064186-47210636	-	-	IGV_LINK	860.142	27.7813	5.06215	50.5005	OK	950.789	30.2935	6.36978	54.2172	OK	OK	0.124891
ENSG0000014	-	-	ENSG0000014223	NTN5	-	19:48637941-48682245	-	-	IGV_LINK	14.5458	0.479508	0	2.34774	OK	8.2498	0.239005	0	1.1474	OK	NOTEST	-1.00452
ENSG0000014	-	-	ENSG0000014223	LMTK3	-	19:48485270-48513189	-	-	IGV_LINK	84.3657	0.990617	0.09028	1.89095	OK	127.872	1.50183	0.266237	2.73741	OK	OK	0.600318
ENSG0000014	-	-	ENSG0000014225	GEMIN7	-	19:45071499-45305283	-	-	IGV_LINK	173.58	10.8004	0	38.5472	OK	211.401	17.0501	0	54.4421	OK	OK	0.658704
ENSG0000014	-	-	ENSG0000014227	CBLC	-	19:44777868-44800634	-	-	IGV_LINK	7.75776	0.310844	0	0.728828	OK	5.15612	0.229279	0	0.571164	OK	NOTEST	-0.43909
ENSG0000014	-	-	ENSG0000014227	WTIP	-	19:34481637-34512304	-	-	IGV_LINK	90.184	2.19604	0	6.15497	OK	49.4988	1.28377	0	3.81484	OK	OK	-0.77452
ENSG0000014	-	-	ENSG0000014230	ADAMTS10	-	19:8580241-8610735	-	-	IGV_LINK	1781.38	71.3844	14.8698	127.899	OK	2034.61	70.6115	15.27	125.953	OK	OK	-0.01571
ENSG0000014	-	-	ENSG0000014231	SLC6A3	-	5:1392789-1445430	-	-	IGV_LINK	3540.45	85.3992	19.6798	151.119	OK	1082.79	22.696	5.84153	39.5505	OK	OK	-1.91179
ENSG0000014	-	-	ENSG0000014232	RNPEPL1	-	2:240479421-240581372	-	-	IGV_LINK	1519.55	37.1729	1.8012	72.5446	OK	1940.77	48.8078	3.878	93.7377	OK	OK	0.392862
ENSG0000014	-	-	ENSG0000014233	CAPN10	-	2:240586715-240631259	-	-	IGV_LINK	469.154	17.5395	2.1501	32.929	OK	448.747	15.8848	1.71532	30.0543	OK	OK	-0.14297
ENSG0000014	-	-	ENSG0000014234	MYO1F	-	19:8520789-8577577	-	-	IGV_LINK	2176.05	53.1421	0.286464	105.998	OK	2813.18	71.3809	4.8779	137.884	OK	OK	0.425682
ENSG0000014	-	-	ENSG0000014239	AC020915.1	-	19:58228593-58326933	-	-	IGV_LINK	442.713	20.2134	0	44.9066	OK	397.376	19.4629	0	43.1629	OK	OK	-0.05458
ENSG0000014	-	-	ENSG0000014240	NLRP12	-	19:53793602-53824394	-	-	IGV_LINK	35.8797	0.566908	0	1.16054	OK	53.6237	0.853597	0	1.7222	OK	OK	0.590441
ENSG0000014	-	-	ENSG0000014240	CACNG8	-	19:53963039-53990215	-	-	IGV_LINK	3.87888	0.049614	0	0.198309	OK	5.15612	0.033893	0	0.134731	OK	NOTEST	-0.54975
ENSG0000014	-	-	ENSG0000014240	ZNF787	-	19:56087365-56121280	-	-	IGV_LINK	382.07	12.335	4.39935	20.2707	OK	414.552	13.3906	4.91416	21.8669	OK	OK	0.118453
ENSG0000014	-	-	ENSG0000014244	TIMM29	-	19:10928732-10933535	-	-	IGV_LINK	186.186	9.32191	1.39944	17.2444	OK	190.777	9.40651	1.90075	16.9123	OK	OK	0.013035
ENSG0000014	-	-	ENSG0000014244	FBN3	-	19:8065401-8149846	-	-	IGV_LINK	0	0	0	0	OK	2.06245	0.210755	0	1.01394	OK	NOTEST	inf
ENSG0000014	-	-	ENSG0000014245	CARM1	-	19:10871512-10928681	-	-	IGV_LINK	452.859	14.2988	0	31.4549	OK	471.27	14.7497	0	33.4852	OK	OK	0.044794
ENSG0000014	-	-	ENSG0000014245	EVI5L	-	19:7830232-7864976	-	-	IGV_LINK	185.217	3.17648	0.484325	5.86864	OK	220.682	3.84614	0.691223	7.00105	OK	OK	0.275981
ENSG0000014	-	-	ENSG0000014248	TM4SF5	-	17:4771883-4783213	-	-	IGV_LINK	25.2127	2.54325	0	5.36175	OK	101.06	10.3096	1.28466	19.3345	OK	OK	2.01924
ENSG0000014	-	-	ENSG0000014249	SLC47A1	-	17:19495384-19597922	-	-	IGV_LINK	3361.05	73.1027	21.5222	124.683	OK	2504.85	54.3413	17.6651	91.0175	OK	OK	-0.42788
ENSG0000014	-	-	ENSG0000014250	PSMB6	-	17:4796143-4798503	-	-	IGV_LINK	818.444	69.45	29.0902	109.81	OK	923.978	79.0007	33.2992	124.702	OK	OK	0.18589
ENSG0000014	-	-	ENSG0000014251	GPR32	-	19:50770463-50771732	-	-	IGV_LINK	0	0	0	0	OK	0	0	0	0	OK	NOTEST	0
ENSG0000014	-	-	ENSG0000014251	SIGLEC10	-	19:51410020-51417803	-	-	IGV_LINK	771.898	17.677	6.09191	29.262	OK	850.761	20.8374	6.44605	35.2288	OK	OK	0.237306
ENSG0000014	-	-	ENSG0000014251	ACP4	-	19:50776140-50795224	-	-	IGV_LINK	0	0	0	0	OK	0	0	0	0	OK	NOTEST	0
ENSG0000014	-	-	ENSG0000014251	KLK3	-	19:50854914-50860764	-	-	IGV_LINK	0	0	0	0	OK	0	0	0	0	OK	NOTEST	0
ENSG0000014	-	-	ENSG0000014252	ZNF473	-	19:49976466-50066793	-	-	IGV_LINK	164.852	3.2397	0	9.34468	OK	173.246	3.02115	0	9.61497	OK	OK	-0.10076
ENSG0000014	-	-	ENSG0000014253	FAM71E1	-	19:50466784-50490870	-	-	IGV_LINK	9.69721	0.493011	0	5.36176	OK	25.7806	1.44439	0	9.71767	OK	OK	1.55076
ENSG0000014	-	-	ENSG0000014253	RPS11	-	19:49496364-49499689	-	-	IGV_LINK	19953.9	2545.05	515.12	4574.98	OK	14370.1	1835.76	527.807	3143.71	OK	OK	-0.47132
ENSG0000014	-	-	ENSG0000014253	PTH2	-	19:49422413-49423441	-	-	IGV_LINK	0	0	0	0	OK	0	0	0	0	OK	NOTEST	0
ENSG0000014	-	-	ENSG0000014253	AC020909.1	-	19:50384203-50431313	-	-	IGV_LINK	1.98E-05	1.02E-06	0	0.103171	OK	5.4839	0.283186	0	1.89646	OK	NOTEST	18.0778
ENSG0000014	-	-	ENSG0000014254	RPL13A	-	19:49446297-49492308	-	-	IGV_LINK	29941.1	1861.13	132.2	3590.07	OK	20421.3	1225.74	185.267	2266.22	OK	OK	-0.60253
ENSG0000014	-	-	ENSG0000014254	CTU1	-	19:51097605-51108370	-	-	IGV_LINK	48.486	1.42726	0	2.85485	OK	57.7486	1.70094	0.026647	3.37523	OK	OK	0.253084
ENSG0000014	-	-	ENSG0000014254	NOSIP	-	19:49555710-49591015	-	-	IGV_LINK	679.774	44.0849	17.3839	70.7859	OK	580.58	36.506	12.2991	60.713	OK	OK	-0.27215
ENSG0000014	-	-	ENSG0000014254	IGLON5	-	19:51311847-51330354	-	-	IGV_LINK	1.93944	0.044914	0	0.137548	OK	17.5308	0.406178	0	0.903983	OK	NOTEST	3.17686
ENSG0000014	-	-	ENSG0000014255	RCN3	-	19:49527617-49546962	-	-	IGV_LINK	467.405	21.5227	4.79902	38.2465	OK	480.551	23.063	4.81046	41.3156	OK	OK	0.09972
ENSG0000014	-	-	ENSG0000014255	ZNF614	-	19:52012764-52030240	-	-	IGV_LINK	160.974	2.52649	0.076526	4.97646	OK	140.247	1.98826	0.031493	3.94502	OK	OK	-0.34563
ENSG0000014	-	-	ENSG0000014258	SLC2A5	-	1:9035106-9088478	-	-	IGV_LINK	362.675	7.95433	0	16.0802	OK	3954.75	87.5005	9.18063	165.82	OK	OK	3.45948
ENSG0000014	-	-	ENSG0000014259	RERE	-	1:8352396-8817643	-	-	IGV_LINK	3104.08	36.5856	0	95.1823	OK	4713.73	54.961	0	134.408	OK	OK	0.587133
ENSG0000014	-	-	ENSG0000014260	MMEL1	-	1:2586490-2801717	-	-	IGV_LINK	76.6079	11.2424	0	34.2439	OK	41.249	6.00111	0	20.3986	OK	OK	-0.90565
ENSG0000014	-	-	ENSG0000014260	CFAP74	-	1:1921950-2003837	-	-	IGV_LINK	13.5761	0.81345	0	2.42986	OK	20.6245	0.769451	0	2.18144	OK	OK	-0.08022
ENSG0000014	-	-	ENSG0000014261	PRDM16	-	1:3069167-3438621	-	-	IGV_LINK	12.6064	0.271389	0	1.01636	OK	30.9367	0.299932	0	0.884706	OK	NOTEST	0.144271
ENSG0000014	-	-	ENSG0000014261	CELA2A	-	1:15456727-15526534	-	-	IGV_LINK	0	0	0	0	OK	0	0	0	0	OK	NOTEST	0
ENSG0000014	-	-	ENSG0000014261	PADI3	-	1:17249097-17284233	-	-	IGV_LINK	0	0	0	0	OK	1.03122	0.019273	0	0.072136	OK	NOTEST	inf
ENSG0000014	-	-	ENSG0000014262	FHAD1	-	1:15247271-15400283	-	-	IGV_LINK	102.79	2.38142	0	6.34209	OK	103.122	2.42656	0	6.2604	OK	OK	0.027087
ENSG0000014	-	-	ENSG0000014262	PADI1	-	1:17205125-17246005	-	-	IGV_LINK	191.035	3.81898	0	8.16575	OK	131.997	2.68304	0	6.18332	OK	OK	-0.50932
ENSG0000014	-	-	ENSG0000014262	EPHA2	-	1:16124336-16157329	-	-	IGV_LINK	457.708	6.99916	2.35042	11.6479	OK	350.616	5.21305	1.67055	8.75555	OK	OK	-0.42505
ENSG0000014	-	-	ENSG0000014263	ARHGEF19	-	1:16197853-16212609	-	-	IGV_LINK	161.943	5.4498	0.335217	10.5644	OK	229.963	7.58687	1.12981	14.0439	OK	OK	0.477301
ENSG0000014	-	-	ENSG0000014263	EFHD2	-	1:15409894-15430343	-	-	IGV_LINK	1740.65	43.7781	19.2136	68.3427	OK	1846.92	46.4839	20.5699	72.3979	OK	OK	0.086521
ENSG0000014	-	-	ENSG0000014265	PEX14	-	1:10456521-10630758	-	-	IGV_LINK	384.009	14.1319	0	29.167	OK	441.364	16.3502	0	33.3753	OK	OK	0.210347
ENSG0000014	-	-	ENSG0000014265	PGD	-	1:10398591-10420144	-	-	IGV_LINK	911.537	31.6521	11.7088	51.5953	OK	1183.85	42.3635	14.5096	70.2175	OK	OK	0.420523
ENSG0000014	-	-	ENSG0000014266	MYOM3	-	1:24040834-24112175	-	-	IGV_LINK	1134.57	13.4034	3.16575	23.641	OK	2348.1	26.3997	9.2412	43.5582	OK	OK	0.977926
ENSG0000014	-	-	ENSG0000014266	SH3BGRL3	-	1:26279175-26320523	-	-	IGV_LINK	1657.25	94.0865	0	202.462	OK	1574.68	90.6773	0	198.597	OK	OK	-0.05325
ENSG0000014	-	-	ENSG0000014267	CNKSR1	-	1:26177402-26189886	-	-	IGV_LINK	25.2127	1.32031	0	3.45335	OK	18.562	0.48845	0	1.45402	OK	OK	-1.43459
ENSG0000014	-	-	ENSG0000014267	RPL11	-	1:23691778-23696425	-	-	IGV_LINK	16747.1	2038.7	524.953	3552.45	OK	15005.4	1831.45	515.588	3147.32	OK	OK	-0.15466
ENSG0000014	-	-	ENSG0000014267	IL22RA1	-	1:24119770-24143121	-	-	IGV_LINK	71.7593	1.5403	0	3.08506	OK	686.796	14.7484	6.04816	23.4487	OK	OK	3.25928
ENSG0000014	-	-	ENSG0000014268	ZNF593	-	1:26169515-26171821	-	-	IGV_LINK	29.0916	3.26281	0	9.78711	OK	59.811	6.72357	0	14.5194	OK	OK	1.04311
ENSG0000014	-	-	ENSG0000014268	C1orf216	-	1:35713874-35719472	-	-	IGV_LINK	246.309	4.68473	1.05267	8.3168	OK	238.213	4.8055	0.647569	8.96343	OK	OK	0.036721
ENSG0000014	-	-	ENSG0000014268	KIAA0319L	-	1:35433489-35567274	-	-	IGV_LINK	2113.99	39.3201	0	93.9857	OK	2811.12	44.8644	0	99.4516	OK	OK	0.190305
ENSG0000014	-	-	ENSG0000014269	EVA1B	-	1:36306386-36329221	-	-	IGV_LINK	368.494	27.5471	0.694929	54.3994	OK	293.899	22.0689	0	57.1059	OK	OK	-0.31989
ENSG0000014	-	-	ENSG0000014269	C1orf94	-	1:34166882-34219131	-	-	IGV_LINK	0	0	0	0	OK	0	0	0	0	OK	NOTEST	0
ENSG0000014	-	-	ENSG0000014270	DMRTA2	-	1:50417549-50423500	-	-	IGV_LINK	0	0	0	0	OK	0	0	0	0	OK	NOTEST	0
ENSG0000014	-	-	ENSG0000014273	PLK4	-	4:127880860-127899195	-	-	IGV_LINK	88.2446	3.89621	0	9.09225	OK	61.8735	2.89749	0	7.10858	OK	OK	-0.42727
ENSG0000014	-	-	ENSG0000014273	MAP3K6	-	1:27355183-27366892	-	-	IGV_LINK	455.769	13.5933	0	28.1534	OK	623.891	12.4706	0	25.0765	OK	OK	-0.12437
ENSG0000014	-	-	ENSG0000014274	FCN3	-	1:27369111-27374824	-	-	IGV_LINK	154.186	10.1826	2.18139	18.1838	OK	664.109	43.8429	17.8894	69.7964	OK	OK	2.10624
ENSG0000014	-	-	ENSG0000014275	GPN2	-	1:26876132-26890297	-	-	IGV_LINK	594.439	15.9164	0.735464	31.0974	OK	662.046	22.0056	1.2374	42.7738	OK	OK	0.467354
ENSG0000014	-	-	ENSG0000014276	SYTL1	-	1:27342019-27353937	-	-	IGV_LINK	323.887	10.3148	2.33599	18.2936	OK	332.054	9.92648	2.0988	17.7542	OK	OK	-0.05536
ENSG0000014	-	-	ENSG0000014278	WDTC1	-	1:27234515-27308633	-	-	IGV_LINK	1360.52	20.6175	6.16045	35.0745	OK	1295.22	19.3823	7.28848	31.4761	OK	OK	-0.08913
ENSG0000014	-	-	ENSG0000014278	CELA3A	-	1:22001655-22012539	-	-	IGV_LINK	0	0	0	0	OK	0	0	0	0	OK	NOTEST	0
ENSG0000014	-	-	ENSG0000014279	NBPF3	-	1:21440127-21485005	-	-	IGV_LINK	291.886	5.97883	0.422489	11.5352	OK	259.869	4.89255	0.669912	9.11518	OK	OK	-0.28928
ENSG0000014	-	-	ENSG0000014279	HSPG2	-	1:21812264-21937297	-	-	IGV_LINK	4351.14	36.0048	0	75.4516	OK	6084.23	41.6202	0	94.2854	OK	OK	0.209093
ENSG0000014	-	-	ENSG0000014285	ITGB3BP	-	1:63440769-63660245	-	-	IGV_LINK	221.096	16.1268	0	41.3085	OK	223.776	15.5957	0	53.089	OK	OK	-0.04831
ENSG0000014	-	-	ENSG0000014286	SERBP1	-	1:67407809-67430415	-	-	IGV_LINK	3211.71	94.2309	26.7529	161.709	OK	3289.61	103.471	29.3015	177.641	OK	OK	0.134957
ENSG0000014	-	-	ENSG0000014286	BCL10	-	1:85266247-85578363	-	-	IGV_LINK	204.611	7.6071	0	19.8568	OK	213.464	6.3587	0	18.7937	OK	OK	-0.25862
ENSG0000014	-	-	ENSG0000014287	CYR61	-	1:85580760-85583962	-	-	IGV_LINK	675.895	18.556	6.88952	30.2224	OK	824.98	24.3246	7.8674	40.7819	OK	OK	0.390534
ENSG0000014	-	-	ENSG0000014287	PRKACB	-	1:84078061-84238498	-	-	IGV_LINK	578.923	10.5348	2.34018	18.7294	OK	514.581	9.70923	1.9958	17.4227	OK	OK	-0.11773
ENSG0000014	-	-	ENSG0000014289	PIGK	-	1:77088989-77219430	-	-	IGV_LINK	489.709	16.8659	3.40718	30.3247	OK	584.705	19.5446	4.59701	34.4922	OK	OK	0.212659
ENSG0000014	-	-	ENSG0000014291	TINAGL1	-	1:31500084-31587686	-	-	IGV_LINK	1719.31	58.6661	18.7662	98.5659	OK	2440.91	78.6501	25.9481	131.352	OK	OK	0.422923
ENSG0000014	-	-	ENSG0000014292	AZIN2	-	1:33081103-33120530	-	-	IGV_LINK	514.922	12.8702	0.964362	24.776	OK	277.4	6.72685	0.798975	12.6547	OK	OK	-0.93603
ENSG0000014	-	-	ENSG0000014293	RPS8	-	1:44739817-44778779	-	-	IGV_LINK	19573.8	1582.73	355.145	2810.31	OK	13718.4	1110.43	277.054	1943.81	OK	OK	-0.51129
ENSG0000014	-	-	ENSG0000014294	KIF2C	-	1:44739817-44778779	-	-	IGV_LINK	137.7	3.33917	0	45.9481	OK	150.559	3.71953	0	35.7621	OK	OK	0.155631
ENSG0000014	-	-	ENSG0000014294	PTPRF	-	1:43525186-43623666	-	-	IGV_LINK	3755.73	35.4504	10.7688	60.1321	OK	7466.07	69.7419	22.4843	116.999	OK	OK	0.976223
ENSG0000014	-	-	ENSG0000014295	BEST4	-	1:44783584-44787705	-	-	IGV_LINK	29.0916	0.874629	0	1.80226	OK	24.7494	0.744539	0	1.54726	OK	OK	-0.23232
ENSG0000014	-	-	ENSG0000014296	MOB3C	-	1:46538695-46616891	-	-	IGV_LINK	872.748	15.6032	2.1466	29.0597	OK	861.073	15.1625	0.322824	30.0021	OK	OK	-0.04133
ENSG0000014	-	-	ENSG0000014297	CYP4B1	-	1:46757837-46819413	-	-	IGV_LINK	2.90916	0.253634	0	0.948215	OK	29.9055	0.927908	0	2.1854	OK	OK	1.87123
ENSG0000014	-	-	ENSG0000014300	TMEM61	-	1:54980791-54992293	-	-	IGV_LINK	0	0	0	0	OK	0	0	0	0	OK	NOTEST	0
ENSG0000014	-	-	ENSG0000014300	DMRTB1	-	1:53459398-53467488	-	-	IGV_LINK	0	0	0	0	OK	0	0	0	0	OK	NOTEST	0
ENSG0000014	-	-	ENSG0000014301	LMO4	-	1:87328467-87348923	-	-	IGV_LINK	419.889	17.741	5.60114	29.8808	OK	299.055	13.3866	3.05216	23.7209	OK	OK	-0.4063
ENSG0000014	-	-	ENSG0000014302	SYPL2	-	1:109466627-109482137	-	-	IGV_LINK	182.307	3.44314	0	7.11699	OK	64.9672	1.06113	0	2.20568	OK	OK	-1.69812
ENSG0000014	-	-	ENSG0000014303	BARHL2	-	1:90711538-90717237	-	-	IGV_LINK	0.969721	0.030252	0	0.118488	OK	0	0	0	0	OK	NOTEST	'-inf
ENSG0000014	-	-	ENSG0000014303	MTF2	-	1:93079234-93139081	-	-	IGV_LINK	392.737	11.7496	1.51323	21.986	OK	335.148	9.65028	2.34854	16.952	OK	OK	-0.28397
ENSG0000014	-	-	ENSG0000014303	SLC44A3	-	1:94613813-94895246	-	-	IGV_LINK	826.202	25.8836	6.99922	44.7681	OK	454.77	14.7668	1.70872	27.8249	OK	OK	-0.80968
ENSG0000014	-	-	ENSG0000014306	IGSF3	-	1:116574398-116667755	-	-	IGV_LINK	668.137	5.31115	1.89692	8.72538	OK	829.105	6.59235	2.60423	10.5805	OK	OK	0.311769
ENSG0000014	-	-	ENSG0000014306	ZNF697	-	1:119619421-119647773	-	-	IGV_LINK	227.884	2.63789	0.760506	4.51527	OK	195.933	2.26857	0.592965	3.94417	OK	OK	-0.2176
ENSG0000014	-	-	ENSG0000014307	CTTNBP2NL	-	1:112396180-112463456	-	-	IGV_LINK	782.564	10.8651	1.97047	19.7597	OK	1033.29	14.7843	2.64111	26.9275	OK	OK	0.444368
ENSG0000014	-	-	ENSG0000014309	STRIP1	-	1:110031576-110074641	-	-	IGV_LINK	548.862	13.7925	0	28.0572	OK	544.487	13.4491	0	27.4139	OK	OK	-0.03637
ENSG0000014	-	-	ENSG0000014310	KCNA10	-	1:110517216-110519175	-	-	IGV_LINK	0	0	0	0	OK	0	0	0	0	OK	NOTEST	0

ENSG0000014	-	-	ENSG0000014310	'PSMA5	-	1:109399030-109426427	-	-	IGV_LINK	966.811	36.1701	14.8204	57.5199	OK	1072.47	40.8027	16.8531	64.7523	OK	OK	0.173866
ENSG0000014	-	-	ENSG0000014310	'FNDC7	-	1:108712656-108742740	-	-	IGV_LINK	1.93944	0.073135	0	0.228025	OK	1.03122	0.018401	0	0.102276	OK	NOTEST	-1.9908
ENSG0000014	-	-	ENSG0000014311	'C1orf162	-	1:111473791-111478512	-	-	IGV_LINK	1082.21	67.2298	27.7161	106.743	OK	701.233	45.1818	16.6648	73.6987	OK	OK	-0.57336
ENSG0000014	-	-	ENSG0000014311	'CD53	-	1:110873153-110899928	-	-	IGV_LINK	2298.24	102.282	41.0094	163.556	OK	2412.04	105.86	43.0634	168.657	OK	OK	0.049603
ENSG0000014	-	-	ENSG0000014312	'PROK1	-	1:110451199-110457354	-	-	IGV_LINK	0	0	0	0	OK	1.03122	0.049913	0	0.186814	OK	NOTEST	inf
ENSG0000014	-	-	ENSG0000014312	'CELSR2	-	1:109250018-109275750	-	-	IGV_LINK	1045.36	7.60609	0.770525	14.4416	OK	839.417	5.95736	0	12.0642	OK	OK	-0.35248
ENSG0000014	-	-	ENSG0000014312	'ITGA10	-	1:145891207-145910189	-	-	IGV_LINK	106.669	2.0324	0	4.8332	OK	82.498	1.21902	0	2.85868	OK	OK	-0.73746
ENSG0000014	-	-	ENSG0000014314	'GPR161	-	1:168079542-168137667	-	-	IGV_LINK	128.003	1.18933	0	2.38905	OK	86.6229	0.894355	0	1.93976	OK	OK	-0.41123
ENSG0000014	-	-	ENSG0000014314	'ALDH9A1	-	1:165662215-165709968	-	-	IGV_LINK	1488.52	33.9577	14.462	53.4534	OK	1674.71	38.8598	15.5935	62.126	OK	OK	0.194538
ENSG0000014	-	-	ENSG0000014315	'ATP1B1	-	1:169105696-169367967	-	-	IGV_LINK	9809.69	292.566	72.1246	513.007	OK	9137.68	273.937	80.7665	467.107	OK	OK	-0.09492
ENSG0000014	-	-	ENSG0000014315	'TIPRL	-	1:168178932-168202114	-	-	IGV_LINK	412.131	11.6446	1.75021	21.5391	OK	445.489	12.9498	1.92728	23.9723	OK	OK	0.153261
ENSG0000014	-	-	ENSG0000014315	'NME7	-	1:169105696-169367967	-	-	IGV_LINK	486.8	40.1527	0	230.637	OK	411.459	31.3013	0	187.619	OK	OK	-0.35927
ENSG0000014	-	-	ENSG0000014315	'POGK	-	1:166839446-166856344	-	-	IGV_LINK	1024.02	10.3441	3.35298	17.3351	OK	824.98	8.18513	2.89295	13.4773	OK	OK	-0.33773
ENSG0000014	-	-	ENSG0000014315	'MPC2	-	1:167916728-168075843	-	-	IGV_LINK	973.599	72.4424	5.2797	139.605	OK	1229.22	92.1298	9.08541	175.174	OK	OK	0.346834
ENSG0000014	-	-	ENSG0000014316	'CREG1	-	1:167529676-167553767	-	-	IGV_LINK	1989.87	62.7672	27.3052	98.2293	OK	3144.2	99.0368	39.727	158.347	OK	OK	0.657953
ENSG0000014	-	-	ENSG0000014316	'DCAF6	-	1:167916728-168075843	-	-	IGV_LINK	1346.94	43.0539	0	87.1102	OK	1538.59	49.6012	0	102.158	OK	OK	0.204231
ENSG0000014	-	-	ENSG0000014316	'GPA33	-	1:167052550-167166479	-	-	IGV_LINK	8.72748	0.274079	0	1.00574	OK	3.09367	0.073397	0	0.40887	OK	NOTEST	-1.9008
ENSG0000014	-	-	ENSG0000014317	'RXRG	-	1:165400916-165445355	-	-	IGV_LINK	0.969721	0.127246	0	0.534255	OK	1.03122	0.094293	0	0.421229	OK	NOTEST	-0.4324
ENSG0000014	-	-	ENSG0000014317	'TBX19	-	1:168281039-168314426	-	-	IGV_LINK	277.34	6.4848	1.70525	11.2644	OK	139.215	3.3674	0.438702	6.2961	OK	OK	-0.94543
ENSG0000014	-	-	ENSG0000014317	'UCK2	-	1:165724292-165911618	-	-	IGV_LINK	251.158	7.99917	0	23.8204	OK	289.774	8.34942	0	27.2751	OK	OK	0.061826
ENSG0000014	-	-	ENSG0000014318	'TMCO1	-	1:165724292-165911618	-	-	IGV_LINK	1296.52	74.9945	15.6425	134.347	OK	1876.83	117.337	28.7826	205.892	OK	OK	0.645806
ENSG0000014	-	-	ENSG0000014318	'XCL1	-	1:168576472-168582077	-	-	IGV_LINK	123.155	5.81044	0.930095	10.6908	OK	16.4996	0.779203	0	1.74118	OK	OK	-2.89858
ENSG0000014	-	-	ENSG0000014318	'XCL2	-	1:168540764-168543997	-	-	IGV_LINK	82.4262	11.8355	1.02524	22.6457	OK	19.5933	2.82165	0	5.95038	OK	OK	-2.0685
ENSG0000014	-	-	ENSG0000014319	'POU2F1	-	1:167220828-167427345	-	-	IGV_LINK	621.591	2.6173	0.831281	4.40331	OK	428.99	1.85448	0.394106	3.31485	OK	OK	-0.49706
ENSG0000014	-	-	ENSG0000014319	'MAEL	-	1:166975581-167022214	-	-	IGV_LINK	28.1219	1.0181	0	2.2789	OK	37.1241	1.34036	0	2.81235	OK	OK	0.396733
ENSG0000014	-	-	ENSG0000014319	'ILDR2	-	1:166895710-166975482	-	-	IGV_LINK	177.459	2.19958	0	6.28947	OK	226.869	1.54258	0	4.1149	OK	OK	-0.51188
ENSG0000014	-	-	ENSG0000014319	'DPT	-	1:168695458-168729264	-	-	IGV_LINK	3.87888	0.135482	0	0.345049	OK	1.03122	0.036044	0	0.134906	OK	NOTEST	-1.91025
ENSG0000014	-	-	ENSG0000014319	'MGST3	-	1:165630860-165661796	-	-	IGV_LINK	1633.01	138.407	56.2366	220.577	OK	1242.63	103.903	41.3746	166.431	OK	OK	-0.41368
ENSG0000014	-	-	ENSG0000014319	'ADCY10	-	1:167809387-167914215	-	-	IGV_LINK	56.2438	0.995498	0	2.46331	OK	57.7486	1.35335	0	3.59064	OK	OK	0.443047
ENSG0000014	-	-	ENSG0000014320	'RFWD2	-	1:175944830-176207493	-	-	IGV_LINK	421.828	12.8431	2.30865	23.3774	OK	521.8	14.0246	4.72174	23.3275	OK	OK	0.126975
ENSG0000014	-	-	ENSG0000014321	'NECTIN4	-	1:161070994-161089599	-	-	IGV_LINK	0.969721	0.016414	0	0.15476	OK	83.5292	2.90959	0	6.52857	OK	OK	7.46977
ENSG0000014	-	-	ENSG0000014322	'UFC1	-	1:161152775-161159349	-	-	IGV_LINK	1819.2	117.7	48.4656	186.934	OK	1826.3	118.307	48.574	188.041	OK	OK	0.007429
ENSG0000014	-	-	ENSG0000014322	'PPOX	-	1:161166409-161178013	-	-	IGV_LINK	597.348	33.554	0.440692	66.6673	OK	529.018	27.0346	0	55.8144	OK	OK	-0.31168
ENSG0000014	-	-	ENSG0000014322	'FCGR2A	-	1:161505429-161605662	-	-	IGV_LINK	2010.23	74.7655	0	204.355	OK	4004.25	137.261	0	314.66	OK	OK	0.876478
ENSG0000014	-	-	ENSG0000014322	'NUF2	-	1:163111120-163355764	-	-	IGV_LINK	75.6382	2.45798	0	39.9125	OK	52.5925	1.88124	0	13.9734	OK	OK	-0.38579
ENSG0000014	-	-	ENSG0000014324	'RGS5	-	1:163111120-163355764	-	-	IGV_LINK	25563	718.955	0	1851.07	OK	5312.72	168.918	0	359.489	OK	OK	-2.08958
ENSG0000014	-	-	ENSG0000014325	'SDHC	-	1:161314256-161375340	-	-	IGV_LINK	2462.12	118.585	15.7986	221.371	OK	2577.03	158.596	33.2211	283.971	OK	OK	0.419437
ENSG0000014	-	-	ENSG0000014325	'PFDN2	-	1:161100555-161132688	-	-	IGV_LINK	292.856	33.2481	0	80.9041	OK	356.804	40.3416	0	96.6037	OK	OK	0.278997
ENSG0000014	-	-	ENSG0000014325	'NR1I3	-	1:161225938-161238302	-	-	IGV_LINK	40.7283	3.58353	0	11.204	OK	34.0304	3.30031	0	11.127	OK	OK	-0.11878
ENSG0000014	-	-	ENSG0000014325	'USP21	-	1:161159449-161165723	-	-	IGV_LINK	516.861	18.9036	5.14173	32.6654	OK	521.8	19.3085	5.03213	33.5849	OK	OK	0.030582
ENSG0000014	-	-	ENSG0000014327	'F13B	-	1:197039190-197067267	-	-	IGV_LINK	0	0	0	0	OK	1.03122	0.137608	0	0.518959	OK	NOTEST	inf
ENSG0000014	-	-	ENSG0000014329	'PRCC	-	1:156742106-156800817	-	-	IGV_LINK	712.745	26.1173	0	78.9621	OK	1162.19	39.499	0	116.064	OK	OK	0.596811
ENSG0000014	-	-	ENSG0000014329	'FCRL5	-	1:157513376-157552520	-	-	IGV_LINK	66.9107	1.28335	0.002238	2.56446	OK	149.528	2.21498	0.073093	4.35688	OK	OK	0.787383
ENSG0000014	-	-	ENSG0000014330	'RRNAD1	-	1:156721890-156736960	-	-	IGV_LINK	510.073	29.5994	0	62.0396	OK	548.612	29.261	0	62.3259	OK	OK	-0.01659
ENSG0000014	-	-	ENSG0000014331	'MRPL24	-	1:156737302-156741590	-	-	IGV_LINK	760.261	60.7885	25.1967	96.3803	OK	866.229	71.5723	29.754	113.391	OK	OK	0.235603
ENSG0000014	-	-	ENSG0000014331	'PIGM	-	1:159998650-160079821	-	-	IGV_LINK	270.552	3.67711	0.659016	6.69521	OK	266.056	3.61701	0.876631	6.35739	OK	OK	-0.02378
ENSG0000014	-	-	ENSG0000014331	'CASQ1	-	1:160190555-160201886	-	-	IGV_LINK	20.3641	0.696301	0	1.60882	OK	13.4059	0.438401	0	1.08842	OK	OK	-0.66746
ENSG0000014	-	-	ENSG0000014331	'ISG20L2	-	1:156721890-156736960	-	-	IGV_LINK	537.225	15.7901	0	33.4648	OK	694.014	22.815	0	47.9784	OK	OK	0.530961
ENSG0000014	-	-	ENSG0000014332	'CRABP2	-	1:156699605-156705816	-	-	IGV_LINK	11.6366	0.800254	0	1.88128	OK	13.4059	1.3209	0	2.95303	OK	OK	0.72299
ENSG0000014	-	-	ENSG0000014332	'HDGF	-	1:156742106-156800817	-	-	IGV_LINK	4422.9	157.549	36.7631	278.335	OK	5921.29	218.065	42.4483	393.682	OK	OK	0.468958
ENSG0000014	-	-	ENSG0000014332	'ABL2	-	1:179099326-179229684	-	-	IGV_LINK	556.62	3.24259	0	8.28429	OK	828.074	4.61123	0	10.3202	OK	OK	0.508002
ENSG0000014	-	-	ENSG0000014332	'XPR1	-	1:180632003-180890251	-	-	IGV_LINK	785.474	9.6006	0.684524	18.5167	OK	1537.56	22.1557	0	44.8823	OK	OK	1.20648
ENSG0000014	-	-	ENSG0000014333	'RGS16	-	1:182598622-182604408	-	-	IGV_LINK	102.79	2.56946	0.309379	4.82955	OK	101.06	2.5275	0.318261	4.73674	OK	OK	-0.02376
ENSG0000014	-	-	ENSG0000014333	'TOR1AIP1	-	1:179881606-179925000	-	-	IGV_LINK	1264.52	26.6052	6.73186	46.4786	OK	1303.47	28.3661	7.3225	49.4097	OK	OK	0.092457
ENSG0000014	-	-	ENSG0000014334	'FAM163A	-	1:179743162-179818191	-	-	IGV_LINK	25.2127	0.519235	0	1.08698	OK	12.3747	0.254954	0	0.576064	OK	NOTEST	-1.02615
ENSG0000014	-	-	ENSG0000014334	'HMCN1	-	1:185734550-186190949	-	-	IGV_LINK	384.009	4.15785	0	11.2623	OK	184.589	3.87016	0	11.8487	OK	OK	-0.10344
ENSG0000014	-	-	ENSG0000014334	'RGL1	-	1:183636084-183928531	-	-	IGV_LINK	1274.21	15.6033	6.52861	24.6779	OK	1860.33	22.9044	10.2163	35.5926	OK	OK	0.55378
ENSG0000014	-	-	ENSG0000014335	'LYPLAL1	-	1:219086601-219212865	-	-	IGV_LINK	396.616	29.9147	0	61.0517	OK	344.429	24.2949	1.27719	47.3127	OK	OK	-0.3002
ENSG0000014	-	-	ENSG0000014335	'LHX9	-	1:197911901-197935478	-	-	IGV_LINK	0	0	0	0	OK	0	0	0	0	OK	NOTEST	0
ENSG0000014	-	-	ENSG0000014336	'PRUNE1	-	1:151008419-151035713	-	-	IGV_LINK	492.618	13.6799	3.81704	23.5428	OK	703.295	18.7405	6.05497	31.426	OK	OK	0.454096
ENSG0000014	-	-	ENSG0000014336	'RORC	-	1:151800263-151831872	-	-	IGV_LINK	1837.62	37.5964	15.2032	59.9896	OK	2010.89	41.1703	16.7603	65.5804	OK	OK	0.131009
ENSG0000014	-	-	ENSG0000014336	'TUFT1	-	1:151540304-151583583	-	-	IGV_LINK	260.855	5.13951	1.57745	8.70157	OK	624.922	13.5047	3.90254	23.1069	OK	OK	1.39376
ENSG0000014	-	-	ENSG0000014336	'SF3B4	-	1:149923316-149928344	-	-	IGV_LINK	734.078	21.6405	8.98875	34.2923	OK	807.449	26.3118	9.69008	42.9335	OK	OK	0.281974
ENSG0000014	-	-	ENSG0000014336	'ECM1	-	1:150508061-150513789	-	-	IGV_LINK	178.429	5.02872	0.868656	9.18879	OK	179.433	4.66312	0.748154	8.57809	OK	OK	-0.1089
ENSG0000014	-	-	ENSG0000014337	'ZNF687	-	1:151280023-151327715	-	-	IGV_LINK	671.047	8.92482	0	22.3563	OK	732.17	9.33852	0	21.9298	OK	OK	0.06537
ENSG0000014	-	-	ENSG0000014337	'TARS2	-	1:150487363-150507609	-	-	IGV_LINK	602.196	17.4396	3.45273	31.4264	OK	739.388	19.9444	5.81216	34.0767	OK	OK	0.19362
ENSG0000014	-	-	ENSG0000014337	'CGN	-	1:151510509-151538692	-	-	IGV_LINK	53.3346	1.16846	0	3.87598	OK	610.485	12.1597	0	28.6583	OK	OK	3.37942
ENSG0000014	-	-	ENSG0000014337	'SNX27	-	1:151612037-151699091	-	-	IGV_LINK	1239.3	10.9469	3.59845	18.2954	OK	1309.66	11.9062	3.38795	20.4244	OK	OK	0.12118
ENSG0000014	-	-	ENSG0000014337	'SETDB1	-	1:150926262-150975534	-	-	IGV_LINK	1307.18	32.0285	0	96.543	OK	1408.65	33.7608	0	115.12	OK	OK	0.075994
ENSG0000014	-	-	ENSG0000014338	'ADAMTSL4	-	1:150548561-150579738	-	-	IGV_LINK	287.037	5.99535	0	23.1412	OK	417.646	8.97114	0	31.8403	OK	OK	0.581447
ENSG0000014	-	-	ENSG0000014338	'MCL1	-	1:150548561-150579738	-	-	IGV_LINK	5278.19	103.719	24.7206	182.716	OK	5826.42	115.438	25.3262	205.55	OK	OK	0.154446
ENSG0000014	-	-	ENSG0000014338	'CTSK	-	1:150796207-150808323	-	-	IGV_LINK	176.489	7.09053	1.40462	12.7764	OK	180.464	6.69827	1.55397	11.8426	OK	OK	-0.0821
ENSG0000014	-	-	ENSG0000014339	'RFX5	-	1:151340639-151348027	-	-	IGV_LINK	2010.23	49.963	5.76208	94.164	OK	1481.87	40.5347	4.14331	76.9261	OK	OK	-0.3017
ENSG0000014	-	-	ENSG0000014339	'PI4KB	-	1:151280023-151327715	-	-	IGV_LINK	2179.93	50.5608	4.44429	96.6773	OK	2280.04	46.5494	6.47632	86.6225	OK	OK	-0.11926
ENSG0000014	-	-	ENSG0000014339	'PIP5K1A	-	1:151197948-151249536	-	-	IGV_LINK	748.624	17.889	1.83066	33.9474	OK	986.882	21.2469	1.9591	40.5347	OK	OK	0.248179
ENSG0000014	-	-	ENSG0000014340	'ANP32E	-	1:150218416-150236156	-	-	IGV_LINK	664.259	15.8464	3.95705	27.7358	OK	602.235	16.6276	2.19718	31.0579	OK	OK	0.069418
ENSG0000014	-	-	ENSG0000014340	'MINDY1	-	1:150996085-151008375	-	-	IGV_LINK	722.442	19.4164	4.4234	34.4094	OK	1220.97	33.3165	9.63071	57.0024	OK	OK	0.778963
ENSG0000014	-	-	ENSG0000014341	'ANXA9	-	1:150982016-150995634	-	-	IGV_LINK	78.5474	2.67713	0.18424	5.17002	OK	180.464	6.15505	1.407	10.9031	OK	OK	1.20108
ENSG0000014	-	-	ENSG0000014341	'SELENBP1	-	1:151364301-151372733	-	-	IGV_LINK	508.134	17.2061	6.30291	28.1093	OK	1190.03	42.1492	16.6489	67.6494	OK	OK	1.29258
ENSG0000014	-	-	ENSG0000014341	'CERS2	-	1:150926262-150975534	-	-	IGV_LINK	3933.19	168.569	0	347.093	OK	5735.67	248.812	0	535.216	OK	OK	0.561714
ENSG0000014	-	-	ENSG0000014342	'ENSA	-	1:150600850-150629612	-	-	IGV_LINK	2177.02	86.0486	25.6571	146.44	OK	2324.38	103.136	21.0795	185.192	OK	OK	0.26132
ENSG0000014	-	-	ENSG0000014342	'AC116050.1	-	2:91589493-91659972	-	-	IGV_LINK	43.6374	4.38773	0	10.324	OK	113.435	10.7502	0.283439	21.217	OK	OK	1.29282
ENSG0000014	-	-	ENSG0000014343	'SEMA6C	-	1:151131684-151146664	-	-	IGV_LINK	173.58	3.11188	0	8.1435	OK	346.492	6.36562	0	15.0537	OK	OK	1.03251
ENSG0000014	-	-	ENSG0000014343	'MRPL9	-	1:151755540-151794402	-	-	IGV_LINK	519.77	30.4488	6.38612	54.5114	OK	477.457	27.7111	4.65598	50.7662	OK	OK	-0.13592
ENSG0000014	-	-	ENSG0000014343	'ARNT	-	1:150809704-150876768	-	-	IGV_LINK	1277.12	20.4236	5.76033	35.0868	OK	1123	18.1941	5.75179	30.6364	OK	OK	-0.16676
ENSG0000014	-	-	ENSG0000014344	'POGZ	-	1:151402723-151459465	-	-	IGV_LINK	2356.42	32.8381	0	69.8501	OK	1782.99	22.8453	0	72.9095	OK	OK	-0.52347
ENSG0000014	-	-	ENSG0000014344	'C1orf56	-	1:151047739-151070325	-	-	IGV_LINK	54.3043	2.38211	0	16.6955	OK	122.716	3.59757	0	14.1011	OK	OK	0.59478
ENSG0000014	-	-	ENSG0000014345	'OAZ3	-	1:151755540-151794402	-	-	IGV_LINK	45.8845	3.64578	0	12.9063	OK	61.8735	4.76818	0	15.1135	OK	OK	0.387211
ENSG0000014	-	-	ENSG0000014345	'HORMAD1	-	1:150698059-150720888	-	-	IGV_LINK	10.6669	0.612672	0	1.57896	OK	2.06245	0.076552	0	0.477801	OK	OK	-3.00061
ENSG0000014	-	-	ENSG0000014345	'GOLPH3L	-	1:150646224-150697144	-	-	IGV_LINK	574.075	16.7276	2.36348	31.0918	OK	790.95	23.7461	6.1914	41.3009	OK	OK	0.505459
ENSG0000014	-	-	ENSG0000014345	'GABPB2	-	1:151070577-151125542	-	-	IGV_LINK	411.161	5.59084	0	11.8376	OK	399.084	5.79817	0	11.6879	OK	OK	0.052533
ENSG0000014	-	-	ENSG0000014346	'SYT14	-	1:209938173-210171389	-	-	IGV_LINK	0	0	0	0	OK	0	0	0	0	OK	NOTEST	0
ENSG0000014	-	-	ENSG0000014347	'KCNH1	-	1:210676822-211207138	-	-	IGV_LINK	1.93944	0.032	0	0.342445	OK	3.09367	0.053277	0	0.369829	OK	NOTEST	0.735453
ENSG0000014	-	-	ENSG0000014347	'DTL	-	1:212035552-212107400	-	-	IGV_LINK	96.9721	2.1208	0	5.3808	OK	94.8727	1.87777	0	4.89609	OK	OK	-0.17559
ENSG0000014	-	-	ENSG0000014347	'DYRK3	-	1:206634648-206684419	-	-	IGV_LINK	137.7	3.91839	0.709306	7.12748	OK	201.089	5.84044	1.52564	10.1552	OK	OK	0.575814
ENSG0000014	-	-	ENSG0000014348	'EIF2D	-	1:206507529-206612463	-	-	IGV_LINK	1598.1	48.2668	0.526824	96.0067	OK	1265.31	39.2146	0.968074	77.4612	OK	OK	-0.29964
ENSG0000014	-	-	ENSG0000014349	'INTS7	-	1:211940398-212035542	-	-	IGV_LINK	224.005	3.76812	0.028046	7.50819	OK	284.618	5.86828	0	11.8132	OK	OK	0.639094
ENSG0000014	-	-	ENSG0000014349	'VASH2	-	1:212950519-212992037	-	-	IGV_LINK	47.5163	0.924558	0	2.4163	OK	23.7182	0.943663	0	2.53915	OK	OK	0.029508
ENSG0000014	-	-	ENSG0000014349	'TAF1A	-	1:222557901-222593032	-	-	IGV_LINK	65.941	2.58641	0	5.60128	OK	41.249	1.43303	0	3.28271	OK	OK	-0.85188
ENSG0000014	-	-	ENSG0000014349	'SMYD2	-	1:214281101-214337131	-	-	IGV_LINK	1124.88	39.2416	11.4935	66.9896	OK	887.885	30.6083	9.67989	51.5366	OK	OK	-0.35846
ENSG0000014	-	-	ENSG0000014350	'SUSD4	-	1:223220818-223364202	-	-	IGV_LINK	29.0916	0.674781	0	1.67526	OK	43.3114	1.15604	0	2.58427	OK	OK	0.776706
ENSG0000014	-	-	ENSG0000014350	'DUSP10	-	1:221701423-221742176	-	-	IGV_LINK	174.55	5.41542	0.665134	10.1657	OK	156.746	4.67362	0.315951	9.03129	OK	OK	-0.21253
ENSG0000014	-	-	ENSG0000014351	'HHIPL2	-	1:222522257-222548103	-	-	IGV_LINK	4.8486	0.230181	0	0.827729	OK	2.06245	0.057115	0	0.492108	OK	NOTEST	-2.01083
ENSG0000014	-	-	ENSG0000014351	'TP53BP2	-	1:223779898-223845972	-	-	IGV_LINK	637.106	9.79387	2.38817	17.1996	OK	793.012	11.1423	3.35815	18.9264	OK	OK	0.186091
ENSG0000014	-	-	ENSG0000014351	'ATP8B2	-	1:154325552-154351307	-	-	IGV_LINK	560.498	7.55026	0.798984	14.3015	OK	486.738	6.99098	0.607726	13.3742	OK	OK	-0.11103
ENSG0000014	-	-	ENSG0000014352	'FLG2	-	1:152122533-152445456	-	-	IGV_LINK	5.81832	0.03663	0	0.165037	OK	14.4371	0.090903	0	0.476378	OK	NOTEST	1.3113
ENSG0000014	-	-	ENSG0000014353	'CRNN	-	1:152122533-152445456	-	-	IGV_LINK	0	0	0	0	OK	2.06245	0.06725	0	0.822156	OK	NOTEST	inf
ENSG0000014	-	-	ENSG0000014353	'ADAM15	-	1:155033823-155062775	-	-	IGV_LINK	1018.21	31.2315	4.38365	58.0794	OK	1658.21	40.8515	10.9175	70.7854	OK	OK	0.387385
ENSG0000014	-	-	ENSG0000014354	'JTB	-	1:153959098-153977688	-	-	IGV_LINK	1512.76	87.3809	0	196.711	OK	1813.92	100.815	0	209.895	OK	OK	0.206321
ENSG0000014	-	-	ENSG0000014354	'RAB13	-	1:153981616-153986358	-	-	IGV_LINK	1684.4	94.0715	40.1021	148.041	OK	2280.04	127.268	55.761	198.774	OK	OK	0.436036
ENSG0000014	-	-	ENSG0000014354	'S100A8	-	1:153390031-153391188	-	-	IGV_LINK	118.306	18.8987	2.98026	34.8171	OK	546.549	87.7243	34.3561	141.092	OK	OK	2.21469
ENSG0000014	-	-	ENSG0000014354	'TPM3	-	1:154155303-154194648	-	-	IGV_LINK	4717.69	176.065	51.3196	300.811	OK	4900.38	193.631	53.1228	334.139	OK	OK	0.137199
ENSG0000014	-	-	ENSG0000014355	'NUP210L	-	1:153992684-154155116	-	-	IGV_LINK	4.8486	0.109408	0	0.295236	OK	3.09367	0.033206	0	0.112654	OK	NOTEST	-1.72022
ENSG0000014	-	-	ENSG0000014355	'SNAPIN	-	1:153658653-153671048	-	-	IGV_LINK	494.557	32.4044	0	70.822	OK	593.986	39.6436	0	89.4352	OK	OK	0.290901
ENSG0000014	-	-	ENSG0000014355	'SLC27A3	-	1:153728066-153780157	-	-	IGV_LINK	568.502	23.9719	0	82.3314	OK	660.021	26.9155	0	85.7379	OK	OK	0.167091
ENSG0000014	-	-	ENSG0000014355	'S100A7	-	1:153457743-153460701	-	-	IGV_LINK	1.93944	0.405316	0	1.32005	OK	1.03122	0.216458	0	0.911143	OK	NOTEST	-0.90496
ENSG0000014	-	-	ENSG0000014356	'UBAP2L	-	1:154206705-154271510	-	-	IGV_LINK	2313.75	60.5911	0	147.787	OK	2536.69	60.553	0	145.643	OK	OK	-0.00091
ENSG0000014	-	-	ENSG0000014357	'SLC39A1	-	1:153959098-153977688	-	-	IGV_LINK	2949.89	85.5341	19.7185	151.35	OK	3127.71	90.814	20.2858	161.342	OK	OK	0.086415
ENSG0000014	-	-	ENSG0000014357	'HAX1	-	1:154272510-154275875	-	-	IGV_LINK	1396.4	79.1258	33.4053	124.846	OK	1609.74	90.7416	38.5835	142.9	OK	OK	0.197616
ENSG0000014	-	-	ENSG0000014357	'CREB3L4	-	1:153959098-153977688	-	-	IGV_LINK	492.618	24.008	0	72.8237	OK	357.835	16.4244	0	58.3766	OK	OK	-0.54768
ENSG0000014	-	-	ENSG0000014359	'EFNA3	-	1:155063730-155087538	-	-	IGV_LINK	93.0932	4.93864	0	10.8489	OK	120.653	6.01909	0	12.5755	OK	OK	0.285433
ENSG0000014	-	-	ENSG0000014359	'AQP10	-	1:154321089-154325325	-	-	IGV_LINK	0	0	0	0	OK	0	0	0	0	OK	NOTEST	0
ENSG0000014	-	-	ENSG0000014360	'KCNN3	-	1:154697454-154870280	-	-	IGV_LINK	254.067	1.45581	0	2.99597	OK	183.558	1.09173	0	2.32528	OK	OK	-0.4152
ENSG0000014	-	-	ENSG0000014361	'C1orf43	-	1:154206705-154271510	-	-	IGV_LINK	4146.52	163.567	23.4345	303.7	OK	4130.06	163.392	27.4405	299.343	OK	OK	-0.00155
ENSG0000014	-	-	ENSG0000014361	'GATAD2B	-	1:153789029-153946696	-	-	IGV_LINK	655.531	5.54937	0	14.9745	OK	599.142	5.63092	0	16.6062	OK	OK	0.021046
ENSG0000014	-	-	ENSG0000014362	'ILF2	-	1:153658653-153671048	-	-	IGV_LINK	1486.58	57.4213	19.9652	94.8774	OK	1978.92	77.9426	26.4567	129.428	OK	OK	0.440825
ENSG0000014	-	-	ENSG0000014362	'RIT1	-	1:155897807-155911404	-	-	IGV_LINK	1256.76	39.4633	9.51391	69.4128	OK	1410.72	52.0357	13.4715	90.5998	OK	OK	0.398988
ENSG0000014	-	-	ENSG0000014362	'INTS3	-	1:153728066-153780157	-	-	IGV_LINK	3444.2	48.0652	5.71906	90.4114	OK	2910.08	40.2888	7.23592	73.3417	OK	OK	-0.25462
ENSG0000014	-	-	ENSG0000014362	'PKLR	-	1:155262867-155301434	-	-	IGV_LINK	3.87888	0.11442	0	1.45491	OK	14.4371	0.352927	0	2.27534	OK	NOTEST	1.62503
ENSG0000014	-	-	ENSG0000014363	'HCN3	-	1:155262867-155301434	-	-	IGV_LINK	211.399	5.02975	0	17.7664	OK	168.09	3.14413	0	9.9274	OK	OK	-0.67783
ENSG0000014	-	-	ENSG0000014363	'FLG	-	1:152122533-152445456	-	-	IGV_LINK	20.3641	0.091266	0	0.262278	OK	348.554	1.56227	0.187609	2.93692	OK	OK	4.09741
ENSG0000014	-	-	ENSG0000014363	'ACTA1	-	1:229431244-229434098	-	-	IGV_LINK	2.90916	0.124346	0	0.363602	OK	4.1249	0.245126	0	0.63183	OK	NOTEST	0.979164

ENSG0000014	-	-	ENSG0000014363	'C1orf131	-	1:231223762-231241187	-	-	IGV_LINK	217.217	10.7197	0.752072	20.6872	OK	221.713	12.1854	0.651583	23.7192	OK	OK	0.184893
ENSG0000014	-	-	ENSG0000014364	'GALNT2	-	1:230057989-230282124	-	-	IGV_LINK	1197.6	20.4845	2.61002	38.359	OK	6478.16	111.191	2.87013	219.512	OK	OK	2.44043
ENSG0000014	-	-	ENSG0000014364	'TTC13	-	1:230906242-230978875	-	-	IGV_LINK	365.585	9.66599	0	19.3558	OK	296.993	8.3021	0	17.3838	OK	OK	-0.21944
ENSG0000014	-	-	ENSG0000014365	'SCCPDH	-	1:246724046-246768137	-	-	IGV_LINK	578.923	13.9995	5.50803	22.4909	OK	1118.88	27.07	11.7793	42.3607	OK	OK	0.951323
ENSG0000014	-	-	ENSG0000014366	'LYST	-	1:235661040-235883640	-	-	IGV_LINK	1130.69	10.1156	0	23.4373	OK	535.206	5.53692	0	12.8802	OK	OK	-0.86943
ENSG0000014	-	-	ENSG0000014367	'MAP3K21	-	1:233327767-233385148	-	-	IGV_LINK	622.561	8.41549	3.34722	13.4838	OK	464.051	5.88645	1.99184	9.78106	OK	OK	-0.51565
ENSG0000014	-	-	ENSG0000014370	'CEP170	-	1:243124427-243255348	-	-	IGV_LINK	769.958	15.2941	0	33.7924	OK	624.922	12.5572	0	26.8748	OK	OK	-0.28446
ENSG0000014	-	-	ENSG0000014372	'ACP1	-	2:217729-278283	-	-	IGV_LINK	1173.36	58.1119	10.345	105.879	OK	1324.09	65.8283	12.6158	119.041	OK	OK	0.179875
ENSG0000014	-	-	ENSG0000014374	'SNAP47	-	1:227728538-227781231	-	-	IGV_LINK	590.56	18.9557	4.16663	33.7447	OK	578.517	18.3597	2.49403	34.2254	OK	OK	-0.04609
ENSG0000014	-	-	ENSG0000014374	'SRP9	-	1:225777812-225790466	-	-	IGV_LINK	1263.55	104.656	14.838	194.475	OK	1421.03	117.846	17.3651	218.328	OK	OK	0.171247
ENSG0000014	-	-	ENSG0000014374	'NVL	-	1:224219612-224330387	-	-	IGV_LINK	431.526	11.4838	1.57824	21.3894	OK	385.678	10.3788	1.21903	19.5386	OK	OK	-0.14597
ENSG0000014	-	-	ENSG0000014375	'SDE2	-	1:225982701-225999331	-	-	IGV_LINK	350.069	5.18827	1.80486	8.57168	OK	426.927	6.32928	2.33263	10.3259	OK	OK	0.286787
ENSG0000014	-	-	ENSG0000014375	'DEGS1	-	1:224175755-224193441	-	-	IGV_LINK	1194.7	37.0907	11.2379	62.9435	OK	3881.53	125.348	39.1077	211.588	OK	OK	1.75681
ENSG0000014	-	-	ENSG0000014375	'FBXO28	-	1:224114086-224162047	-	-	IGV_LINK	542.074	8.63668	1.33205	15.9413	OK	762.075	11.7983	2.98419	20.6124	OK	OK	0.450031
ENSG0000014	-	-	ENSG0000014376	'ARF1	-	1:228082659-228099212	-	-	IGV_LINK	5211.28	196.736	65.251	328.22	OK	6121.35	242.409	75.8547	408.963	OK	OK	0.301184
ENSG0000014	-	-	ENSG0000014376	'LEFTY2	-	1:225936410-225941489	-	-	IGV_LINK	0.969721	0.028572	0	0.267384	OK	0	0	0	0	OK	NOTEST	'-inf
ENSG0000014	-	-	ENSG0000014377	'CNIH4	-	1:224356849-224379459	-	-	IGV_LINK	618.682	22.0558	2.96195	41.1497	OK	714.639	31.2156	8.23958	54.1917	OK	OK	0.501109
ENSG0000014	-	-	ENSG0000014377	'ITPKB	-	1:226631689-226739323	-	-	IGV_LINK	740.866	7.69601	3.03349	12.3585	OK	608.423	6.55263	2.5505	10.5548	OK	OK	-0.23204
ENSG0000014	-	-	ENSG0000014377	'GUK1	-	1:228139961-228148984	-	-	IGV_LINK	3424.08	240.268	85.5753	394.96	OK	2801.84	196.431	72.7975	320.064	OK	OK	-0.29062
ENSG0000014	-	-	ENSG0000014377	'CDC42BPA	-	1:226989864-227318474	-	-	IGV_LINK	1039.54	6.83309	2.50343	11.1628	OK	1841.77	13.8809	1.49744	26.2644	OK	OK	1.02249
ENSG0000014	-	-	ENSG0000014378	'CNIH3	-	1:224385142-224740549	-	-	IGV_LINK	109.578	5.90111	0	25.5634	OK	64.9672	4.88857	0	25.5575	OK	OK	-0.27157
ENSG0000014	-	-	ENSG0000014379	'C1orf35	-	1:228100725-228105411	-	-	IGV_LINK	301.583	12.0982	3.767	20.4294	OK	285.649	12.3107	3.11003	21.5114	OK	OK	0.025122
ENSG0000014	-	-	ENSG0000014379	'MBOAT2	-	2:8852689-9003813	-	-	IGV_LINK	138.67	4.17196	0	10.1779	OK	89.7166	2.42405	0	5.88227	OK	OK	-0.78331
ENSG0000014	-	-	ENSG0000014379	'PARP1	-	1:226360690-226408079	-	-	IGV_LINK	2112.05	33.7309	10.0021	57.4597	OK	2638.9	44.6372	8.20911	81.0654	OK	OK	0.404177
ENSG0000014	-	-	ENSG0000014380	'PSEN2	-	1:226870183-226896105	-	-	IGV_LINK	353.948	13.7327	2.40953	25.0558	OK	357.835	15.6466	2.6966	28.5965	OK	OK	0.188236
ENSG0000014	-	-	ENSG0000014381	'PYCR2	-	1:225886281-225924340	-	-	IGV_LINK	1125.44	40.3503	13.1592	67.5415	OK	1197.25	42.7078	15.6978	69.7178	OK	OK	0.081917
ENSG0000014	-	-	ENSG0000014381	'LBR	-	1:225401501-225428925	-	-	IGV_LINK	726.321	15.7266	0.606993	30.8462	OK	662.046	12.5552	1.89259	23.2177	OK	OK	-0.32493
ENSG0000014	-	-	ENSG0000014381	'WNT9A	-	1:227918655-227947898	-	-	IGV_LINK	32.0008	0.474774	0	0.973062	OK	26.8118	0.397909	0	0.823921	OK	NOTEST	-0.2548
ENSG0000014	-	-	ENSG0000014381	'EPHX1	-	1:225810091-225882369	-	-	IGV_LINK	548.862	20.7914	0	47.7078	OK	1279.75	50.4235	0	101.537	OK	OK	1.27811
ENSG0000014	-	-	ENSG0000014383	'REN	-	1:204154818-204190324	-	-	IGV_LINK	275.401	12.1778	3.8663	20.4893	OK	6.18735	0.273843	0	0.658544	OK	OK	-5.47476
ENSG0000014	-	-	ENSG0000014384	'SOX13	-	1:204073114-204127743	-	-	IGV_LINK	846.566	18.4061	4.08315	32.729	OK	696.077	16.3345	3.02028	29.6486	OK	OK	-0.17226
ENSG0000014	-	-	ENSG0000014384	'ETNK2	-	1:204131061-204152003	-	-	IGV_LINK	247.279	6.70761	1.00243	12.4128	OK	384.647	10.734	1.99862	19.4695	OK	OK	0.678322
ENSG0000014	-	-	ENSG0000014384	'PPFIA4	-	1:203026497-203078740	-	-	IGV_LINK	1962.71	34.5098	0	72.1958	OK	1808.77	34.9823	0	75.0024	OK	OK	0.019619
ENSG0000014	-	-	ENSG0000014385	'PLEKHA6	-	1:204218850-204377665	-	-	IGV_LINK	893.113	14.2336	0	45.7623	OK	1401.43	22.7589	0	71.0956	OK	OK	0.677124
ENSG0000014	-	-	ENSG0000014385	'PTPN7	-	1:202147012-202161588	-	-	IGV_LINK	838.808	22.5606	6.27863	38.8426	OK	446.52	13.2126	2.68674	23.7384	OK	OK	-0.77189
ENSG0000014	-	-	ENSG0000014385	'SYT2	-	1:202348698-202710417	-	-	IGV_LINK	34.9099	0.264911	0	1.01075	OK	17.5308	0.133028	0	0.624129	OK	NOTEST	-0.99378
ENSG0000014	-	-	ENSG0000014386	'ARL8A	-	1:202122857-202144743	-	-	IGV_LINK	592.499	23.6883	4.88036	42.4963	OK	628.016	24.0278	5.81805	42.2375	OK	OK	0.020527
ENSG0000014	-	-	ENSG0000014386	'OSR1	-	2:19351484-19358653	-	-	IGV_LINK	16.4852	0.39449	0	0.923112	OK	52.5925	1.30798	0	2.88469	OK	OK	1.72928
ENSG0000014	-	-	ENSG0000014386	'GDF7	-	2:20666663-20679245	-	-	IGV_LINK	61.0924	0.359515	0	0.730021	OK	59.811	0.352016	0	0.710016	OK	NOTEST	-0.03041
ENSG0000014	-	-	ENSG0000014387	'PDIA6	-	2:10721648-10837977	-	-	IGV_LINK	3694.64	96.1305	40.6267	151.634	OK	3637.13	94.7942	40.2163	149.372	OK	OK	-0.0202
ENSG0000014	-	-	ENSG0000014387	'RHOB	-	2:20447073-20449445	-	-	IGV_LINK	3234.02	82.8627	35.8064	129.919	OK	2989.52	76.6382	33.1872	120.089	OK	OK	-0.11266
ENSG0000014	-	-	ENSG0000014388	'ATP6V1C2	-	2:10721648-10837977	-	-	IGV_LINK	40.7283	0.783504	0	5.103	OK	28.8743	0.579265	0	4.43533	OK	OK	-0.43572
ENSG0000014	-	-	ENSG0000014388	'HNRNPLL	-	2:38561977-38603586	-	-	IGV_LINK	434.435	12.6129	3.28103	21.9447	OK	311.43	9.8535	1.44173	18.2653	OK	OK	-0.35619
ENSG0000014	-	-	ENSG0000014389	'GALM	-	2:38665909-38741237	-	-	IGV_LINK	764.14	20.2487	7.21838	33.279	OK	930.165	25.7051	9.0613	42.3489	OK	OK	0.344227
ENSG0000014	-	-	ENSG0000014391	'CAMKMT	-	2:44361949-44772592	-	-	IGV_LINK	69.8199	4.59085	0	9.6672	OK	46.4051	2.70717	0	5.90294	OK	OK	-0.76198
ENSG0000014	-	-	ENSG0000014392	'ABCG8	-	2:43838963-43878466	-	-	IGV_LINK	2.90916	0.065777	0	0.176544	OK	1.03122	0.023327	0	0.087308	OK	NOTEST	-1.49558
ENSG0000014	-	-	ENSG0000014392	'EML4	-	2:42143237-42332548	-	-	IGV_LINK	1555.43	18.1103	6.95244	29.2682	OK	1410.72	17.0298	6.45997	27.5996	OK	OK	-0.08875
ENSG0000014	-	-	ENSG0000014393	'CALM2	-	2:46899274-47176601	-	-	IGV_LINK	4222.16	239.181	19.0183	459.344	OK	4705.48	265.852	10.739	520.964	OK	OK	0.152519
ENSG0000014	-	-	ENSG0000014394	'CHAC2	-	2:53532671-53860160	-	-	IGV_LINK	12.6064	0.618592	0	3.87943	OK	13.4059	0.658486	0	4.51863	OK	OK	0.090164
ENSG0000014	-	-	ENSG0000014394	'RPS27A	-	2:55174790-55235853	-	-	IGV_LINK	18543	1947.61	315.428	3579.79	OK	14269.1	1505.59	284.53	2726.64	OK	OK	-0.37138
ENSG0000014	-	-	ENSG0000014395	'WDPCP	-	2:63121382-63827843	-	-	IGV_LINK	325.826	6.63746	0	18.3843	OK	325.867	7.04312	0	21.3892	OK	OK	0.085584
ENSG0000014	-	-	ENSG0000014395	'VPS54	-	2:63892145-64019072	-	-	IGV_LINK	687.532	13.0991	2.69466	23.5035	OK	670.296	12.2699	3.74259	20.7972	OK	OK	-0.09434
ENSG0000014	-	-	ENSG0000014395	'REG3G	-	2:79025685-79028505	-	-	IGV_LINK	1.93944	0.15991	0	0.63163	OK	900.259	80.1855	28.493	131.878	OK	OK	8.96994
ENSG0000014	-	-	ENSG0000014397	'ASXL2	-	2:25733752-25878516	-	-	IGV_LINK	1014.33	6.68501	2.41815	10.9519	OK	777.544	4.44448	1.67832	7.21064	OK	OK	-0.58891
ENSG0000014	-	-	ENSG0000014397	'ETAA1	-	2:67397318-67410545	-	-	IGV_LINK	242.43	4.74834	0.234103	9.26258	OK	265.025	5.30707	0.153332	10.4608	OK	OK	0.160492
ENSG0000014	-	-	ENSG0000014397	'SNRPG	-	2:70281361-70293771	-	-	IGV_LINK	487.769	62.193	23.3817	101.004	OK	551.705	69.3901	26.9121	111.868	OK	OK	0.157978
ENSG0000014	-	-	ENSG0000014399	'ABHD1	-	2:27123788-27134675	-	-	IGV_LINK	23.2733	1.03483	0	5.66664	OK	39.1865	1.54669	0	7.02456	OK	OK	0.579798
ENSG0000014	-	-	ENSG0000014399	'MEIS1	-	2:66426734-66573869	-	-	IGV_LINK	246.309	4.81736	0	10.0793	OK	141.278	2.62604	0	5.37253	OK	OK	-0.87536
ENSG0000014	-	-	ENSG0000014401	'TRIM43B	-	2:95476966-95484731	-	-	IGV_LINK	0	0	0	0	OK	0	0	0	0	OK	NOTEST	0
ENSG0000014	-	-	ENSG0000014401	'TRIM43	-	2:95592017-95599778	-	-	IGV_LINK	0	0	0	0	OK	0	0	0	0	OK	NOTEST	0
ENSG0000014	-	-	ENSG0000014402	'CIAO1	-	2:96266131-96273349	-	-	IGV_LINK	1538.95	42.682	14.9976	70.3664	OK	1842.8	50.709	17.968	83.45	OK	OK	0.248614
ENSG0000014	-	-	ENSG0000014402	'ZNF514	-	2:95087206-95165413	-	-	IGV_LINK	1058.09	19.5522	0	40.4262	OK	630.137	13.2395	0	31.9299	OK	OK	-0.56249
ENSG0000014	-	-	ENSG0000014402	'SNRNP200	-	2:96274335-96305515	-	-	IGV_LINK	5940.51	74.3754	7.73527	141.016	OK	5688.24	73.6936	14.1687	133.218	OK	OK	-0.01329
ENSG0000014	-	-	ENSG0000014402	'MRPS5	-	2:95087206-95165413	-	-	IGV_LINK	1063.66	40.6057	5.77387	75.4376	OK	957.95	36.5758	7.92529	65.2262	OK	OK	-0.15079
ENSG0000014	-	-	ENSG0000014403	'ANKRD53	-	2:70935881-71079805	-	-	IGV_LINK	43.6374	1.92809	0	16.6973	OK	16.4996	0.605172	0	6.77776	OK	OK	-1.67175
ENSG0000014	-	-	ENSG0000014403	'TPRKB	-	2:73729103-73737400	-	-	IGV_LINK	215.278	24.085	6.18028	41.9896	OK	176.339	19.6984	4.17199	35.2248	OK	OK	-0.29005
ENSG0000014	-	-	ENSG0000014403	'NAT8	-	2:73640831-73642393	-	-	IGV_LINK	2322.48	168.998	73.7484	264.247	OK	911.603	66.4325	28.4002	104.465	OK	OK	-1.34704
ENSG0000014	-	-	ENSG0000014403	'EXOC6B	-	2:72175983-72826041	-	-	IGV_LINK	518.8	5.14841	1.91375	8.38307	OK	468.176	4.74987	0.997626	8.5021	OK	OK	-0.11624
ENSG0000014	-	-	ENSG0000014404	'SFXN5	-	2:72942035-73156721	-	-	IGV_LINK	222.066	4.78821	0	17.8592	OK	423.833	9.74876	0	34.7459	OK	OK	1.02573
ENSG0000014	-	-	ENSG0000014404	'TEX261	-	2:70935881-71079805	-	-	IGV_LINK	2033.33	36.886	0	75.1343	OK	2316.13	43.1317	0	88.0488	OK	OK	0.225676
ENSG0000014	-	-	ENSG0000014404	'DQX1	-	2:74518130-74526336	-	-	IGV_LINK	5.81832	0.193221	0	1.42174	OK	9.28102	0.287722	0	1.59358	OK	NOTEST	0.574426
ENSG0000014	-	-	ENSG0000014404	'DUSP11	-	2:73762183-73780157	-	-	IGV_LINK	308.371	11.5187	3.58511	19.4522	OK	418.677	15.5219	5.59093	25.4529	OK	OK	0.430335
ENSG0000014	-	-	ENSG0000014405	'ST6GAL2	-	2:106801599-106887108	-	-	IGV_LINK	1.93944	0.01635	0	0.433482	OK	16.4996	0.264064	0	0.911108	OK	NOTEST	4.01354
ENSG0000014	-	-	ENSG0000014406	'NPHP1	-	2:110122310-110205066	-	-	IGV_LINK	451.89	9.03827	0	18.8725	OK	411.459	9.43459	1.14205	17.7271	OK	OK	0.061912
ENSG0000014	-	-	ENSG0000014406	'MALL	-	2:110083869-110116566	-	-	IGV_LINK	119.276	2.32716	0.348697	4.30563	OK	436.208	8.55537	3.02726	14.0835	OK	OK	1.87826
ENSG0000014	-	-	ENSG0000014411	'THNSL2	-	2:88170294-88186636	-	-	IGV_LINK	709.835	20.3872	5.70158	35.0727	OK	722.889	20.1561	2.50214	37.81	OK	OK	-0.01645
ENSG0000014	-	-	ENSG0000014411	'RALB	-	2:120240063-120294713	-	-	IGV_LINK	1265.49	38.397	11.8619	64.9321	OK	1828.36	53.6114	21.3889	85.8339	OK	OK	0.481547
ENSG0000014	-	-	ENSG0000014411	'C1QL2	-	2:119156242-119158889	-	-	IGV_LINK	0	0	0	0	OK	5.15612	0.155444	0	0.381047	OK	NOTEST	inf
ENSG0000014	-	-	ENSG0000014412	'TMEM177	-	2:119679166-119686507	-	-	IGV_LINK	150.307	7.49552	1.20509	13.786	OK	293.899	15.1385	4.09895	26.1781	OK	OK	1.01412
ENSG0000014	-	-	ENSG0000014413	'NT5DC4	-	2:112721485-112764677	-	-	IGV_LINK	12.6064	0.397162	0	1.67606	OK	5.15612	0.169492	0	1.22855	OK	NOTEST	-1.22851
ENSG0000014	-	-	ENSG0000014413	'RABL2A	-	2:113627228-113643396	-	-	IGV_LINK	481.951	22.6675	5.24168	40.0933	OK	435.177	21.8631	5.19824	38.528	OK	OK	-0.05213
ENSG0000014	-	-	ENSG0000014413	'SLC20A1	-	2:112645856-112663827	-	-	IGV_LINK	832.99	18.0756	3.65448	32.4967	OK	882.729	18.6903	4.06774	33.3129	OK	OK	0.048247
ENSG0000014	-	-	ENSG0000014415	'FBLN7	-	2:112138384-112188216	-	-	IGV_LINK	64.9713	2.19507	0	4.57319	OK	24.7494	0.665822	0	1.45358	OK	OK	-1.72106
ENSG0000014	-	-	ENSG0000014415	'AL078621.1	-	2:113657907-113658675	-	-	IGV_LINK	0	0	0	0	OK	0	0	0	0	OK	NOTEST	0
ENSG0000014	-	-	ENSG0000014416	'ZC3H8	-	2:112211524-112255136	-	-	IGV_LINK	213.339	4.89768	0	10.5799	OK	192.839	4.20927	0	8.68222	OK	OK	-0.21853
ENSG0000014	-	-	ENSG0000014418	'LIPT1	-	2:98997260-99490035	-	-	IGV_LINK	97.765	5.79101	0	31.2526	OK	83.9049	5.19233	0	28.5538	OK	OK	-0.15743
ENSG0000014	-	-	ENSG0000014418	'TRIM43CP	-	2:97025980-97031636	-	-	IGV_LINK	0	0	0	0	OK	0	0	0	0	OK	NOTEST	0
ENSG0000014	-	-	ENSG0000014419	'CNGA3	-	2:98331388-98398601	-	-	IGV_LINK	0	0	0	0	OK	0	0	0	0	OK	NOTEST	0
ENSG0000014	-	-	ENSG0000014419	'FAHD2B	-	2:97083582-97094882	-	-	IGV_LINK	95.0326	5.96794	0.152542	11.7833	OK	120.653	8.02244	0.415904	15.629	OK	OK	0.426809
ENSG0000014	-	-	ENSG0000014421	'LYG1	-	2:98997260-99490035	-	-	IGV_LINK	490.679	33.7902	0	93.1411	OK	665.14	45.9184	0	111.263	OK	OK	0.442468
ENSG0000014	-	-	ENSG0000014421	'AFF3	-	2:99545418-100142739	-	-	IGV_LINK	371.403	3.60304	0	8.64879	OK	103.122	1.10926	0	3.22849	OK	OK	-1.69962
ENSG0000014	-	-	ENSG0000014422	'UBXN4	-	2:135741618-135785055	-	-	IGV_LINK	2255.57	35.8551	13.5945	58.1156	OK	2465.66	39.116	14.7248	63.5072	OK	OK	0.125581
ENSG0000014	-	-	ENSG0000014422	'NXPH2	-	2:138670771-138780348	-	-	IGV_LINK	0	0	0	0	OK	0	0	0	0	OK	NOTEST	0
ENSG0000014	-	-	ENSG0000014422	'SPOPL	-	2:138501800-138574458	-	-	IGV_LINK	482.631	7.68967	0	15.4575	OK	354.741	5.30162	0.161942	10.4413	OK	OK	-0.53649
ENSG0000014	-	-	ENSG0000014422	'THSD7B	-	2:136765544-137677717	-	-	IGV_LINK	15.5155	0.147241	0	0.598507	OK	24.7494	0.234916	0	0.729337	OK	NOTEST	0.67397
ENSG0000014	-	-	ENSG0000014423	'GPR17	-	2:127638380-127681786	-	-	IGV_LINK	23.2733	0.727739	0	3.03941	OK	43.3114	1.20011	0	3.74889	OK	OK	0.721675
ENSG0000014	-	-	ENSG0000014423	'POLR2D	-	2:127843550-127858157	-	-	IGV_LINK	428.723	8.791	0.975853	16.6061	OK	470.058	11.1248	2.01796	20.2317	OK	OK	0.339682
ENSG0000014	-	-	ENSG0000014423	'AMMECR1L	-	2:127861629-127885922	-	-	IGV_LINK	472.254	5.93338	2.25152	9.61524	OK	501.175	6.29837	2.42549	10.1712	OK	OK	0.086123
ENSG0000014	-	-	ENSG0000014427	'GALNT13	-	2:153871912-154457438	-	-	IGV_LINK	0	0	0	0	OK	1.03122	0.187834	0	0.945396	OK	NOTEST	inf
ENSG0000014	-	-	ENSG0000014428	'PKP4	-	2:158456963-158753775	-	-	IGV_LINK	1647.56	26.8769	2.58637	51.1675	OK	2566.72	40.0113	4.17159	75.851	OK	OK	0.57404
ENSG0000014	-	-	ENSG0000014428	'SCN1A	-	2:165957398-166375993	-	-	IGV_LINK	2.90916	0.040031	0	1.46664	OK	0	0	0	0	OK	NOTEST	'-inf
ENSG0000014	-	-	ENSG0000014429	'SLC4A10	-	2:161416093-161985282	-	-	IGV_LINK	3.87888	0.050752	0	0.440729	OK	61.8735	0.774608	0	1.66157	OK	OK	3.93193
ENSG0000014	-	-	ENSG0000014430	'SCRN3	-	2:174395729-174429575	-	-	IGV_LINK	268.613	13.6407	1.93997	25.3415	OK	305.243	15.5872	2.01212	29.1622	OK	OK	0.19244
ENSG0000014	-	-	ENSG0000014432	'LNPK	-	2:175923891-176002839	-	-	IGV_LINK	375.282	5.72056	0	11.6057	OK	510.456	8.56135	0	22.0043	OK	OK	0.581681
ENSG0000014	-	-	ENSG0000014433	'ZNF385B	-	2:179441981-179861505	-	-	IGV_LINK	124.124	3.52561	0.022742	7.02848	OK	4.1249	0.120115	0	0.840413	OK	OK	-4.87538
ENSG0000014	-	-	ENSG0000014433	'TMEFF2	-	2:191834301-192195709	-	-	IGV_LINK	0	0	0	0	OK	0	0	0	0	OK	NOTEST	0
ENSG0000014	-	-	ENSG0000014435	'CDCA7	-	2:173338218-173368997	-	-	IGV_LINK	115.397	3.06697	0.21934	5.91461	OK	102.091	2.80545	0.031798	5.57911	OK	OK	-0.12858
ENSG0000014	-	-	ENSG0000014435	'DLX1	-	2:172084739-172089677	-	-	IGV_LINK	26.1825	0.840276	0	1.96824	OK	7.21857	0.401282	0	1.14661	OK	OK	-1.06625
ENSG0000014	-	-	ENSG0000014435	'UBR3	-	2:169827457-170084131	-	-	IGV_LINK	598.318	6.96262	0.757417	13.1678	OK	578.517	7.57311	0.036633	15.1096	OK	OK	0.121256
ENSG0000014	-	-	ENSG0000014436	'PHOSPHO2	-	2:169694453-169776989	-	-	IGV_LINK	26.8904	1.63109	0	5.01936	OK	27.8427	1.7848	0	5.04348	OK	OK	0.129932
ENSG0000014	-	-	ENSG0000014436	'GULP1	-	2:187712815-188595931	-	-	IGV_LINK	108.609	4.63804	0	10.2127	OK	10.3122	0.369202	0	1.30141	OK	OK	-3.65103
ENSG0000014	-	-	ENSG0000014436	'FAM171B	-	2:186590064-186765965	-	-	IGV_LINK	47.5163	0.503896	0	3.56521	OK	61.8735	0.616897	0	4.07415	OK	OK	0.291905
ENSG0000014	-	-	ENSG0000014438	'HSPD1	-	2:197486580-197553699	-	-	IGV_LINK	4421.93	126.383	21.561	231.204	OK	5700.61	166.895	27.5678	306.222	OK	OK	0.401144
ENSG0000014	-	-	ENSG0000014439	'CCDC150	-	2:196639553-196799725	-	-	IGV_LINK	300.613	8.89589	0	20.0641	OK	122.716	5.61275	0	17.2907	OK	OK	-0.66443
ENSG0000014	-	-	ENSG0000014440	'METTL21A	-	2:207239649-207625928	-	-	IGV_LINK	342.311	9.6567	0	23.021	OK	280.493	7.8594	0	18.8925	OK	OK	-0.29711
ENSG0000014	-	-	ENSG0000014440	'UNC80	-	2:209771992-209999300	-	-	IGV_LINK	7.75776	0.04986	0	0.376435	OK	1.03122	0.023132	0	0.344548	OK	NOTEST	-1.10799
ENSG0000014	-	-	ENSG0000014440	'PTH2R	-	2:208359713-208854503	-	-	IGV_LINK	507.164	56.2228	19.4972	92.9485	OK	228.932	25.7132	6.77634	44.65	OK	OK	-1.12865
ENSG0000014	-	-	ENSG0000014441	'CPO	-	2:206939553-206969474	-	-	IGV_LINK	24.243	1.31815	0	2.92138	OK	25.7806	1.40331	0	2.91179	OK	OK	0.09032
ENSG0000014	-	-	ENSG0000014442	'NBEAL1	-	2:203014878-203226378	-	-	IGV_LINK	101.85	1.58544	0	5.01273	OK	54.2448	0.999178	0	3.48667	OK	OK	-0.66607
ENSG0000014	-	-	ENSG0000014444	'KANSL1L	-	2:210002564-210171383	-	-	IGV_LINK	1045.36	20.5204	1.32264	39.7182	OK	896.135	19.5595	0	40.4556	OK	OK	-0.06919
ENSG0000014	-	-	ENSG0000014445	'SPAG16	-	2:213284378-214684246	-	-	IGV_LINK	222.066	10.6581	2.4274	18.8889	OK	216.557	11.5223	2.68978	20.3547	OK	OK	0.112469
ENSG0000014	-	-	ENSG0000014445	'ABCA12	-	2:214810228-215138428	-	-	IGV_LINK	176.489	1.11411	0.192526	2.0357	OK	777.544	5.54226	0	11.2313	OK	OK	2.31458
ENSG0000014	-	-	ENSG0000014445	'SUMF1	-	3:3700813-4467281	-	-	IGV_LINK	527.528	15.4977	5.04028	25.9552	OK	670.296	19.2922	7.22944	31.355	OK	OK	0.315962
ENSG0000014	-	-	ENSG0000014446	'NYAP2	-	2:225399709-225654018	-	-	IGV_LINK	1.93944	0.024742	0	0.08034	OK	2.06245	0.021983	0	0.07149	OK	NOTEST	-0.17056
ENSG0000014	-	-	ENSG0000014446	'RHBDD1	-	2:226835580-226999215	-	-	IGV_LINK	671.047	11.3656	0	23.5245	OK	637.297	13.6294	0	28.8177	OK	OK	0.262048
ENSG0000014	-	-	ENSG0000014447	'ACKR3	-	2:236567786-236582358	-	-	IGV_LINK	939.659	32.1037	10.3061	53.9013	OK	828.074	30.8966	7.92896	53.8643	OK	OK	-0.05529
ENSG0000014	-	-	ENSG0000014448	'TRPM8	-	2:233917397-234019522	-	-	IGV_LINK	0.969721	0.027734	0	0.694859	OK	25.7806	0.631465	0	1.94622	OK	OK	4.50898
ENSG0000014	-	-	ENSG0000014448	'HES6	-	2:238231683-238300620	-	-	IGV_LINK	27.1522	1.32603	0	5.28077	OK	45.3739	2.40521	0	8.42988	OK	OK	0.859048
ENSG0000014	-	-	ENSG0000014448	'ESPNL	-	2:238100156-238133287	-	-	IGV_LINK	17.455	0.261824	0	0.652939	OK	15.4684	0.230561	0	0.602517	OK	NOTEST	-0.18345
ENSG0000014	-	-	ENSG0000014450	'ANKMY1	-	2:240479421-240581372	-	-	IGV_LINK	447.041	13.2338	0	50.9299	OK	401.147	9.03349	0	33.741	OK	OK	-0.55088
ENSG0000014	-	-	ENSG0000014452	'COPS7B	-	2:231732424-231809254	-	-	IGV_LINK	745.715	28.9311	4.57519	53.2871	OK	621.829	23.9222	3.70782	44.1366	OK	OK	-0.27427
ENSG0000014	-	-	ENSG0000014453	'DIS3L2	-	2:231961244-232344350	-	-	IGV_LINK	471.609	15.0509	1.1544	28.9474	OK	423.043	13.2356	0.98212	25.489	OK	OK	-0.18543

ENSG0000014-	-	ENSG0000014455	CPNE9	-	3:9703806-9729908	-	-	IGV_LINK	3.87888	0.134954	0	0.528126	OK	6.18735	0.262262	0	0.834414	OK	NOTEST	0.958545
ENSG0000014-	-	ENSG0000014455	FANCD2	-	3:10026413-10108231	-	-	IGV_LINK	266.673	5.85171	0	14.1282	OK	191.808	4.55158	0	11.1563	OK	OK	-0.36249
ENSG0000014-	-	ENSG0000014455	TAMM41	-	3:11790441-11846919	-	-	IGV_LINK	83.396	4.90254	0	10.1468	OK	80.4355	5.01583	0.032243	9.99942	OK	OK	0.032959
ENSG0000014-	-	ENSG0000014456	VGLL4	-	3:11272308-11771350	-	-	IGV_LINK	974.569	19.3327	3.376	35.2895	OK	464.051	8.91931	0.707244	17.1314	OK	OK	-1.11604
ENSG0000014-	-	ENSG0000014456	RAB5A	-	3:19947078-20012330	-	-	IGV_LINK	509.103	15.9852	5.08106	26.8894	OK	557.893	17.3127	5.28549	29.3399	OK	OK	0.115094
ENSG0000014-	-	ENSG0000014456	RETREG2	-	2:219171896-219185479	-	-	IGV_LINK	2879.1	86.0574	4.85636	167.258	OK	2795.65	85.7112	2.15117	169.271	OK	OK	-0.00582
ENSG0000014-	-	ENSG0000014457	CTDSP1	-	2:218398255-218405941	-	-	IGV_LINK	2663.82	91.6464	29.8985	153.394	OK	2824.53	97.0954	28.0224	166.168	OK	OK	0.083325
ENSG0000014-	-	ENSG0000014458	CNOT9	-	2:218568579-218597080	-	-	IGV_LINK	692.38	15.296	2.46095	28.1311	OK	699.171	16.2899	2.31534	30.2644	OK	OK	0.09082
ENSG0000014-	-	ENSG0000014458	MARCH4	-	2:216257864-216372027	-	-	IGV_LINK	11.6366	0.153419	0	0.349268	OK	10.3122	0.135995	0	0.30897	OK	NOTEST	-0.17393
ENSG0000014-	-	ENSG0000014458	STK11IP	-	2:219597859-219616451	-	-	IGV_LINK	487.769	14.5935	1.38654	27.8005	OK	617.704	19.1487	2.28279	36.0147	OK	OK	0.391922
ENSG0000014-	-	ENSG0000014459	GMPPA	-	2:219434845-219538772	-	-	IGV_LINK	812.626	27.9481	8.32427	47.5719	OK	1127.13	41.2676	14.2547	68.2806	OK	OK	0.562262
ENSG0000014-	-	ENSG0000014459	GRIP2	-	3:14402575-14556075	-	-	IGV_LINK	143.519	2.12412	0	6.50449	OK	47.4363	0.879349	0	6.23156	OK	OK	-1.27236
ENSG0000014-	-	ENSG0000014459	EAF1	-	3:15381274-15521751	-	-	IGV_LINK	301.583	4.19602	0	8.50213	OK	484.676	6.19821	0.886215	11.5102	OK	OK	0.562829
ENSG0000014-	-	ENSG0000014461	CNTN4	-	3:2098812-3126613	-	-	IGV_LINK	101.821	3.06306	0	7.40744	OK	14.4371	0.251054	0	0.950388	OK	OK	-3.6089
ENSG0000014-	-	ENSG0000014463	DYNC1LI1	-	3:32525970-32570874	-	-	IGV_LINK	395.646	11.4054	1.6739	21.1369	OK	345.46	10.3973	1.17953	19.6151	OK	OK	-0.13351
ENSG0000014-	-	ENSG0000014464	RBMS3	-	3:28349145-30010391	-	-	IGV_LINK	236.348	4.03829	0	8.99979	OK	152.099	1.79213	0	4.10948	OK	OK	-1.17207
ENSG0000014-	-	ENSG0000014464	GADL1	-	3:30726199-30894765	-	-	IGV_LINK	2.90916	0.037807	0	0.201901	OK	1.03122	0.013405	0	0.169169	OK	NOTEST	-1.49586
ENSG0000014-	-	ENSG0000014464	OSBPL10	-	3:31657889-32077580	-	-	IGV_LINK	399.525	8.47346	0	17.9951	OK	1047.72	27.0807	0.837389	53.324	OK	OK	1.67624
ENSG0000014-	-	ENSG0000014464	POMGNT2	-	3:43079231-43106076	-	-	IGV_LINK	162.913	3.87144	0.87879	6.86409	OK	175.308	4.16803	1.00495	7.3311	OK	OK	0.106493
ENSG0000014-	-	ENSG0000014464	ACKR2	-	3:42692662-43060211	-	-	IGV_LINK	4.8486	0.12114	0	1.33098	OK	7.21857	0.181281	0	1.99004	OK	NOTEST	0.581554
ENSG0000014-	-	ENSG0000014464	FAM198A	-	3:42692662-43060211	-	-	IGV_LINK	8.53659	0.179316	0	1.10358	OK	11.3435	0.486304	0	3.58963	OK	NOTEST	1.43935
ENSG0000014-	-	ENSG0000014465	CSRNP1	-	3:39141854-39172952	-	-	IGV_LINK	244.37	4.62836	1.32645	7.93026	OK	331.023	6.27211	2.05137	10.4928	OK	OK	0.438451
ENSG0000014-	-	ENSG0000014465	SLC25A38	-	3:39383347-39397351	-	-	IGV_LINK	434.435	12.6739	4.46607	20.8817	OK	366.085	10.5836	3.73169	17.4355	OK	OK	-0.26003
ENSG0000014-	-	ENSG0000014466	ITGA9	-	3:37452114-37861780	-	-	IGV_LINK	186.186	1.3645	0.163316	2.56569	OK	565.111	4.12777	1.45085	6.80468	OK	OK	1.59698
ENSG0000014-	-	ENSG0000014467	SLC22A14	-	3:38282293-38318575	-	-	IGV_LINK	10.6669	0.314076	0	0.849695	OK	10.3122	0.285272	0	0.699938	OK	NOTEST	-0.13877
ENSG0000014-	-	ENSG0000014467	GOLGA4	-	3:37241788-37366751	-	-	IGV_LINK	1039.54	10.0062	0.846145	19.1663	OK	1195.19	10.3592	1.74905	18.9693	OK	OK	0.050008
ENSG0000014-	-	ENSG0000014467	CTDSPL	-	3:37861959-37984469	-	-	IGV_LINK	896.022	14.8755	2.11836	27.6326	OK	435.177	6.76691	1.04361	12.4902	OK	OK	-1.13637
ENSG0000014-	-	ENSG0000014468	STAC	-	3:36380343-36548007	-	-	IGV_LINK	28.1219	0.598475	0	1.61032	OK	77.3419	1.91359	0	4.80994	OK	OK	1.67692
ENSG0000014-	-	ENSG0000014471	IQSEC1	-	3:12897219-13073117	-	-	IGV_LINK	775.776	9.5835	3.39657	15.7704	OK	810.543	10.3974	3.24444	17.5504	OK	OK	0.117599
ENSG0000014-	-	ENSG0000014471	CAND2	-	3:12796471-12871916	-	-	IGV_LINK	21.3339	0.273239	0	4.28979	OK	21.6557	0.27741	0	3.3854	OK	NOTEST	0.021855
ENSG0000014-	-	ENSG0000014471	RPL32	-	3:12796471-12871916	-	-	IGV_LINK	10328.5	1308.35	342.98	2273.73	OK	6891.68	871.341	258.525	1484.16	OK	OK	-0.58644
ENSG0000014-	-	ENSG0000014472	PTPRG	-	3:61561568-62369330	-	-	IGV_LINK	838.808	7.61152	2.58408	12.6389	OK	815.699	7.92731	2.55144	13.3032	OK	OK	0.058648
ENSG0000014-	-	ENSG0000014473	IL17RD	-	3:57089981-57170306	-	-	IGV_LINK	186.186	1.22757	0.195157	2.25999	OK	73.217	0.4828	0	1.1157	OK	OK	-1.34631
ENSG0000014-	-	ENSG0000014473	SHQ1	-	3:72749276-72861914	-	-	IGV_LINK	174.55	3.58389	0.680318	6.48746	OK	189.745	4.09579	0.861321	7.33027	OK	OK	0.192617
ENSG0000014-	-	ENSG0000014474	SLC25A26	-	3:66133609-66501263	-	-	IGV_LINK	226.915	11.2655	0	28.3779	OK	251.619	12.7729	0	34.2821	OK	OK	0.18117
ENSG0000014-	-	ENSG0000014474	UBA3	-	3:68975213-69080408	-	-	IGV_LINK	525.589	20.5199	0	42.7065	OK	564.08	20.4864	0	41.8667	OK	OK	-0.00235
ENSG0000014-	-	ENSG0000014474	ARL6IP5	-	3:69084943-69106066	-	-	IGV_LINK	2193.51	79.9487	23.9483	135.949	OK	2776.06	99.3839	30.074	168.694	OK	OK	0.313937
ENSG0000014-	-	ENSG0000014474	TMF1	-	3:68975213-69080408	-	-	IGV_LINK	634.197	8.16501	0	19.6973	OK	606.36	7.51072	0	15.1583	OK	OK	-0.1205
ENSG0000014-	-	ENSG0000014474	LRIG1	-	3:66133609-66501263	-	-	IGV_LINK	816.505	12.5941	0	25.3961	OK	789.918	9.9984	2.92649	17.0703	OK	OK	-0.33297
ENSG0000014-	-	ENSG0000014477	LRTM1	-	3:54122546-55074557	-	-	IGV_LINK	0	0	0	0	OK	0	0	0	0	OK	NOTEST	0
ENSG0000014-	-	ENSG0000014478	AC073896.1	-	12:56271698-56334053	-	-	IGV_LINK	11.9997	0.884681	0	13.2229	OK	1.49E-09	2.42E-10	0	0.462778	OK	OK	-31.7659
ENSG0000014-	-	ENSG0000014479	LIMD1	-	3:45555393-45745424	-	-	IGV_LINK	563.408	6.65147	0	19.5309	OK	628.016	6.93395	0	18.2	OK	OK	0.060005
ENSG0000014-	-	ENSG0000014479	ZNF660	-	3:44555192-44685653	-	-	IGV_LINK	47.5163	0.472986	0	1.5214	OK	56.7174	1.13458	0	5.01875	OK	OK	1.26229
ENSG0000014-	-	ENSG0000014480	NFKBIZ	-	3:101827990-101861022	-	-	IGV_LINK	565.347	15.8558	0.548401	31.1631	OK	929.134	23.458	1.78982	45.1261	OK	OK	0.565073
ENSG0000014-	-	ENSG0000014481	COL8A1	-	3:99638474-99799226	-	-	IGV_LINK	478.072	9.16372	0	31.5954	OK	367.116	5.20753	1.07456	9.34051	OK	OK	-0.81533
ENSG0000014-	-	ENSG0000014481	NXPE3	-	3:101779201-101827392	-	-	IGV_LINK	202.672	2.73703	0	5.65669	OK	173.246	2.7122	0.021322	5.40307	OK	OK	-0.01315
ENSG0000014-	-	ENSG0000014482	ADGRG7	-	3:100609588-100695479	-	-	IGV_LINK	0	0	0	0	OK	2.06245	0.062445	0	0.381489	OK	NOTEST	inf
ENSG0000014-	-	ENSG0000014482	MYH15	-	3:108380368-108529322	-	-	IGV_LINK	20.3641	0.166287	0	0.452247	OK	16.4996	0.161165	0	0.453748	OK	NOTEST	-0.04514
ENSG0000014-	-	ENSG0000014482	PHLDB2	-	3:111674675-111976517	-	-	IGV_LINK	1105.48	15.6202	4.58227	26.658	OK	1805.67	26.3499	4.4468	48.253	OK	OK	0.754388
ENSG0000014-	-	ENSG0000014482	ABHD10	-	3:111979009-111993363	-	-	IGV_LINK	608.015	19.3581	5.14015	33.5761	OK	612.548	18.0073	5.37849	30.6361	OK	OK	-0.10436
ENSG0000014-	-	ENSG0000014483	TAGLN3	-	3:111998663-112013887	-	-	IGV_LINK	0	0	0	0	OK	0	0	0	0	OK	NOTEST	0
ENSG0000014-	-	ENSG0000014483	PLA1A	-	3:119597841-119629811	-	-	IGV_LINK	1620.4	57.3865	25.19	89.5829	OK	2950.33	105.632	43.3214	167.942	OK	OK	0.880258
ENSG0000014-	-	ENSG0000014484	RABL3	-	3:120686680-120783069	-	-	IGV_LINK	343.281	8.09436	0.231138	15.9576	OK	403.209	12.0486	0.258511	23.8386	OK	OK	0.573873
ENSG0000014-	-	ENSG0000014484	ADPRH	-	3:119579211-119589945	-	-	IGV_LINK	370.433	6.7521	1.93183	11.5724	OK	394.959	7.54947	1.64447	13.4545	OK	OK	0.16104
ENSG0000014-	-	ENSG0000014484	IGSF11	-	3:118900556-119146068	-	-	IGV_LINK	20.3641	0.345588	0	0.912601	OK	7.21857	0.227116	0	0.818303	OK	NOTEST	-0.60562
ENSG0000014-	-	ENSG0000014484	ATG3	-	3:112532508-112585577	-	-	IGV_LINK	833.96	36.4917	6.989	65.9943	OK	776.512	34.619	6.47345	62.7646	OK	OK	-0.076
ENSG0000014-	-	ENSG0000014485	NR1I2	-	3:119780483-119818485	-	-	IGV_LINK	42.6677	1.27324	0	2.80025	OK	53.6237	1.72694	0	3.74956	OK	OK	0.439712
ENSG0000014-	-	ENSG0000014485	BOC	-	3:113211002-113441610	-	-	IGV_LINK	191.035	4.96351	0	16.3171	OK	155.715	3.145	0	8.55014	OK	OK	-0.6583
ENSG0000014-	-	ENSG0000014486	SRPRB	-	3:133784032-133895836	-	-	IGV_LINK	661.349	19.946	0	42.9549	OK	862.104	28.9747	0	61.4662	OK	OK	0.538694
ENSG0000014-	-	ENSG0000014486	TMEM108	-	3:133038390-133397792	-	-	IGV_LINK	16.4852	0.515127	0	1.51784	OK	23.7182	0.662314	0	1.69809	OK	OK	0.362589
ENSG0000014-	-	ENSG0000014489	AGTR1	-	3:148697783-148743008	-	-	IGV_LINK	54.3043	1.55507	0	3.13038	OK	7.21857	0.238109	0	0.632603	OK	OK	-2.70728
ENSG0000014-	-	ENSG0000014489	MED12L	-	3:151085696-151458709	-	-	IGV_LINK	13.5761	0.427578	0	4.08796	OK	2.06245	0.018665	0	0.574591	OK	NOTEST	-4.51782
ENSG0000014-	-	ENSG0000014489	EIF2A	-	3:150541993-150630445	-	-	IGV_LINK	1889.99	62.8708	1.85592	123.886	OK	1582.93	52.2055	0	108.231	OK	OK	-0.26819
ENSG0000014-	-	ENSG0000014490	ALDH1L1	-	3:126103561-126210169	-	-	IGV_LINK	3687.85	78.4797	17.1268	139.833	OK	6463.72	131.884	30.2896	233.479	OK	OK	0.748882
ENSG0000014-	-	ENSG0000014490	OSBPL11	-	3:125528857-125595090	-	-	IGV_LINK	408.252	5.72536	2.08839	9.36233	OK	499.113	7.0016	2.63707	11.3661	OK	OK	0.290318
ENSG0000014-	-	ENSG0000014493	TRPC1	-	3:142724073-142807888	-	-	IGV_LINK	268.613	4.7095	0.065804	9.35319	OK	232.026	4.33617	0	9.00825	OK	OK	-0.11915
ENSG0000014-	-	ENSG0000014495	NCEH1	-	3:172630248-172711218	-	-	IGV_LINK	3217.53	43.0753	15.4447	70.7059	OK	3714.47	50.611	15.8241	85.3979	OK	OK	0.23259
ENSG0000014-	-	ENSG0000014496	SPATA16	-	3:172889357-173141268	-	-	IGV_LINK	0	0	0	0	OK	0	0	0	0	OK	NOTEST	0
ENSG0000014-	-	ENSG0000014500	FAM86B2	-	8:12425613-12436343	-	-	IGV_LINK	4.8486	0.431344	0	1.12147	OK	15.4684	1.22548	0	2.69117	OK	OK	1.50643
ENSG0000014-	-	ENSG0000014501	LPP	-	3:188151205-188890671	-	-	IGV_LINK	2340.91	8.26805	0	22.6969	OK	2009.86	7.12469	0	18.1557	OK	OK	-0.21472
ENSG0000014-	-	ENSG0000014501	TMEM44	-	3:194584010-194672477	-	-	IGV_LINK	602.196	23.4814	0.57015	46.3927	OK	994.101	42.0753	5.03684	79.1137	OK	OK	0.841454
ENSG0000014-	-	ENSG0000014501	RUBCN	-	3:197671392-197787596	-	-	IGV_LINK	691.411	9.46823	0	25.8772	OK	634.203	9.07179	0	24.6402	OK	OK	-0.06171
ENSG0000014-	-	ENSG0000014502	AMT	-	3:49416774-49429326	-	-	IGV_LINK	360.766	10.3432	0	21.0038	OK	514.109	16.1735	1.49014	30.8569	OK	OK	0.644956
ENSG0000014-	-	ENSG0000014502	TCTA	-	3:49359144-49416475	-	-	IGV_LINK	703.047	21.4505	0	60.6302	OK	976.57	29.5168	0	74.0057	OK	OK	0.460522
ENSG0000014-	-	ENSG0000014502	NICN1	-	3:49416774-49429326	-	-	IGV_LINK	300.164	9.80368	0	20.5214	OK	288.679	9.55115	0	20.5933	OK	OK	-0.03765
ENSG0000014-	-	ENSG0000014504	UCN2	-	3:48517683-48563773	-	-	IGV_LINK	2.90916	0.125296	0	1.19426	OK	1.03122	0.044454	0	0.866892	OK	NOTEST	-1.49498
ENSG0000014-	-	ENSG0000014504	DCAF1	-	3:51391267-51500002	-	-	IGV_LINK	631.288	7.07809	1.27744	12.8787	OK	517.675	5.71069	0.492136	10.9292	OK	OK	-0.3097
ENSG0000014-	-	ENSG0000014505	MANF	-	3:51385046-51389397	-	-	IGV_LINK	309.341	26.1514	0.852043	51.4508	OK	305.243	26.8579	0	54.3848	OK	OK	0.038455
ENSG0000014-	-	ENSG0000014506	AC062028.1	-	2:11105316-11132821	-	-	IGV_LINK	0	0	0	0	OK	4.1249	0.284773	0	0.866697	OK	NOTEST	inf
ENSG0000014-	-	ENSG0000014507	CCDC39	-	3:180602129-180982753	-	-	IGV_LINK	33.9402	1.41588	0	14.0588	OK	36.0929	2.14919	0	18.336	OK	OK	0.602093
ENSG0000014-	-	ENSG0000014508	STXBP5L	-	3:120908071-121424761	-	-	IGV_LINK	0	0	0	0	OK	0	0	0	0	OK	NOTEST	0
ENSG0000014-	-	ENSG0000014508	EAF2	-	3:121835182-121886526	-	-	IGV_LINK	49.4557	3.7235	0	8.06672	OK	46.4051	3.38891	0	6.84146	OK	OK	-0.13584
ENSG0000014-	-	ENSG0000014510	ILDR1	-	3:121987322-122022204	-	-	IGV_LINK	119.276	2.83229	0.376653	5.28792	OK	184.589	4.50526	0.69886	8.31167	OK	OK	0.669644
ENSG0000014-	-	ENSG0000014510	TM4SF19	-	3:196211486-196338503	-	-	IGV_LINK	10.6669	0.60056	0	5.78632	OK	23.8373	1.40396	0	8.67266	OK	OK	1.22512
ENSG0000014-	-	ENSG0000014511	MUC4	-	3:195746764-195812277	-	-	IGV_LINK	47.5163	1.25666	0	3.09611	OK	25.7806	0.661114	0	1.62104	OK	OK	-0.92662
ENSG0000014-	-	ENSG0000014514	SLIT2	-	4:20253259-20620561	-	-	IGV_LINK	28.1219	0.716907	0	2.75601	OK	38.1553	0.365565	0	1.17204	OK	OK	-0.97166
ENSG0000014-	-	ENSG0000014519	EIF2B5	-	3:184134018-184145311	-	-	IGV_LINK	1580.64	52.9905	11.989	93.9921	OK	1498.37	51.6803	11.638	91.7227	OK	OK	-0.03612
ENSG0000014-	-	ENSG0000014519	AHSG	-	3:186538440-186773476	-	-	IGV_LINK	0	0	0	0	OK	0	0	0	0	OK	NOTEST	0
ENSG0000014-	-	ENSG0000014519	ECE2	-	3:184249649-184293031	-	-	IGV_LINK	28.1219	0.953077	0	2.15057	OK	53.6237	1.83226	0	4.00379	OK	OK	0.942958
ENSG0000014-	-	ENSG0000014519	VWA5B2	-	3:184230428-184249548	-	-	IGV_LINK	3.87888	0.06965	0	0.569696	OK	3.09367	0.05557	0	0.565025	OK	NOTEST	-0.32584
ENSG0000014-	-	ENSG0000014521	DGKQ	-	4:958886-1004506	-	-	IGV_LINK	346.19	4.6097	0	9.32226	OK	376.397	5.11325	0	10.6895	OK	OK	0.149569
ENSG0000014-	-	ENSG0000014521	FIP1L1	-	4:53377642-54298247	-	-	IGV_LINK	555.65	22.8927	0	48.3207	OK	508.794	20.8323	0	42.7282	OK	OK	-0.13606
ENSG0000014-	-	ENSG0000014521	SLC26A1	-	4:958886-1004506	-	-	IGV_LINK	130.912	4.89759	0	25.2048	OK	331.023	6.85362	0	20.1515	OK	OK	0.484795
ENSG0000014-	-	ENSG0000014522	LYAR	-	4:4267700-4290169	-	-	IGV_LINK	149.337	6.24319	1.08216	11.4042	OK	187.683	7.91443	1.82554	14.0033	OK	OK	0.342202
ENSG0000014-	-	ENSG0000014524	CENPC	-	4:67468747-67545606	-	-	IGV_LINK	417.95	6.90597	1.17479	12.6371	OK	386.709	7.99098	1.64732	14.3346	OK	OK	0.210529
ENSG0000014-	-	ENSG0000014524	EPHA5	-	4:65319562-65698029	-	-	IGV_LINK	0	0	0	0	OK	0	0	0	0	OK	NOTEST	0
ENSG0000014-	-	ENSG0000014524	CORIN	-	4:47593997-48040173	-	-	IGV_LINK	16.4852	0.319136	0	2.02054	OK	12.3747	0.244481	0	1.35329	OK	NOTEST	-0.38445
ENSG0000014-	-	ENSG0000014524	ATP10D	-	4:47485287-47593486	-	-	IGV_LINK	834.929	13.6736	0	28.1797	OK	748.669	13.3803	0	28.4302	OK	OK	-0.03129
ENSG0000014-	-	ENSG0000014524	OCIAD2	-	4:48885018-48906937	-	-	IGV_LINK	1973.38	150.437	48.1398	252.735	OK	2207.85	175.393	65.0263	285.759	OK	OK	0.221426
ENSG0000014-	-	ENSG0000014524	SLC10A4	-	4:48483342-48489196	-	-	IGV_LINK	4.8486	0.168933	0	0.410323	OK	0	0	0	0	OK	NOTEST	'-inf
ENSG0000014-	-	ENSG0000014528	SLC10A6	-	4:86823467-86849263	-	-	IGV_LINK	6.78804	0.28774	0	0.707505	OK	30.9367	1.31252	0	2.71645	OK	OK	2.1895
ENSG0000014-	-	ENSG0000014528	SCD5	-	4:82629538-82798857	-	-	IGV_LINK	473.224	9.10668	3.45731	14.7561	OK	49.4988	0.952926	0	1.93718	OK	OK	-3.25649
ENSG0000014-	-	ENSG0000014528	PLAC8	-	4:83090047-83137075	-	-	IGV_LINK	233.703	18.8239	1.36664	36.2812	OK	136.122	12.4147	0.028847	24.8005	OK	OK	-0.60052
ENSG0000014-	-	ENSG0000014529	ENOPH1	-	4:82430561-82461091	-	-	IGV_LINK	576.014	17.7783	6.24046	29.3162	OK	491.894	15.2376	5.81312	24.662	OK	OK	-0.22249
ENSG0000014-	-	ENSG0000014530	CABS1	-	4:70334965-70337116	-	-	IGV_LINK	0	0	0	0	OK	0	0	0	0	OK	NOTEST	0
ENSG0000014-	-	ENSG0000014532	GC	-	4:71741692-71804041	-	-	IGV_LINK	6.78804	0.295739	0	0.975391	OK	146.434	5.49163	0.617331	10.3659	OK	OK	4.21484
ENSG0000014-	-	ENSG0000014533	TRMT10A	-	4:99546708-99625913	-	-	IGV_LINK	124.124	5.63886	0	16.6156	OK	119.622	4.28337	0	10.011	OK	OK	-0.39666
ENSG0000014-	-	ENSG0000014533	KLHL8	-	4:87160102-87240314	-	-	IGV_LINK	305.462	3.83928	1.15723	6.52133	OK	478.488	6.43007	0.825781	12.0344	OK	OK	0.743997
ENSG0000014-	-	ENSG0000014533	SNCA	-	4:89551355-89841978	-	-	IGV_LINK	88.2446	6.15845	0	12.5997	OK	157.777	10.2399	0.295214	20.1846	OK	OK	0.733566
ENSG0000014-	-	ENSG0000014533	PYURF	-	4:88520984-88708542	-	-	IGV_LINK	34.3392	1.62831	0	7.47212	OK	34.3035	1.62819	0	7.8124	OK	OK	-0.0001
ENSG0000014-	-	ENSG0000014534	TBCK	-	4:106041598-106349226	-	-	IGV_LINK	585.711	14.3988	0	34.3252	OK	516.644	10.9829	0	25.3227	OK	OK	-0.3907
ENSG0000014-	-	ENSG0000014534	CAMK2D	-	4:113451031-113761927	-	-	IGV_LINK	932.871	19.9906	5.69322	34.288	OK	427.958	10.2995	1.77076	18.8283	OK	OK	-0.95675
ENSG0000014-	-	ENSG0000014535	CISD2	-	4:102868977-103019739	-	-	IGV_LINK	545.953	15.1371	4.41143	25.8627	OK	762.075	22.9384	5.88169	39.995	OK	OK	0.599675
ENSG0000014-	-	ENSG0000014535	DDIT4L	-	4:99948085-100215505	-	-	IGV_LINK	277.34	8.03795	0	22.8558	OK	555.83	15.8288	0	38.0686	OK	OK	0.977652
ENSG0000014-	-	ENSG0000014536	ANK2	-	4:112818108-113383740	-	-	IGV_LINK	2309.87	33.8512	0	68.5962	OK	3126.67	41.959	4.36122	79.5568	OK	OK	0.309775
ENSG0000014-	-	ENSG0000014536	TIFA	-	4:112274541-112442620	-	-	IGV_LINK	379.161	12.1691	0	32.0422	OK	305.243	9.59066	0	26.5543	OK	OK	-0.34352
ENSG0000014-	-	ENSG0000014537	SPATA5	-	4:122923073-123319450	-	-	IGV_LINK	140.609	1.51267	0.061114	2.96423	OK	92.8102	0.857984	0	1.78558	OK	OK	-0.81808
ENSG0000014-	-	ENSG0000014538	FABP2	-	4:119317249-119322390	-	-	IGV_LINK	0	0	0	0	OK	0	0	0	0	OK	NOTEST	0
ENSG0000014-	-	ENSG0000014538	CCNA2	-	4:121801316-121823933	-	-	IGV_LINK	141.579	3.04013	0	7.0962	OK	104.154	2.23747	0	5.41694	OK	OK	-0.44226
ENSG0000014-	-	ENSG0000014538	METTL14	-	4:118685367-118715433	-	-	IGV_LINK	392.737	15.6949	0	34.863	OK	393.928	15.199	0	33.9364	OK	OK	-0.04632
ENSG0000014-	-	ENSG0000014539	USP53	-	4:119192772-119295517	-	-	IGV_LINK	340.372	3.93507	0.652448	7.2177	OK	376.397	4.68166	0	9.41645	OK	OK	0.250629
ENSG0000014-	-	ENSG0000014539	SETD7	-	4:139495940-139606699	-	-	IGV_LINK	1412.88	11.6493	4.50935	18.7892	OK	2098.54	18.12	5.30603	30.9339	OK	OK	0.637342
ENSG0000014-	-	ENSG0000014541	NAF1	-	4:163108784-163166921	-	-	IGV_LINK	127.033	4.86546	0.12181	9.60911	OK	128.903	5.13118	0.429805	9.83256	OK	OK	0.076715
ENSG0000014-	-	ENSG0000014541	MARCH1	-	4:163524297-164384050	-	-	IGV_LINK	590.56	10.3077	1.6383	18.9771	OK	570.267	12.4565	1.69425	23.2187	OK	OK	0.273174
ENSG0000014-	-	ENSG0000014542	SFRP2	-	4:153780591-153789120	-	-	IGV_LINK	8.72748	0.264505	0	0.601992	OK	5.15612	0.156364	0	0.383303	OK	NOTEST	-0.75839
ENSG0000014-	-	ENSG0000014542	RPS3A	-	4:151099572-151325632	-	-	IGV_LINK	17526.7	1307.29	252.707	2361.87	OK	12427.3	924.592	227.06	1622.12	OK	OK	-0.49969
ENSG0000014-	-	ENSG0000014542	RNF175	-	4:153710124-153760235	-	-	IGV_LINK	27.1522	1.03992	0	2.47961	OK	16.4996	0.719591	0	1.9973	OK	OK	-0.53123
ENSG0000014-	-	ENSG0000014543	PDGFC	-	4:156760453-156971394	-	-	IGV_LINK	570.196	20.1659	3.49453	36.8374	OK	611.516	19.9607	3.86101	36.0604	OK	OK	-0.01476
ENSG0000014-	-	ENSG0000014543	CBR4	-	4:168356734-169010275	-	-	IGV_LINK	366.554	8.85184	0	21.8428	OK	308.336	6.1094	0	15.2882	OK	OK	-0.53495
ENSG0000014-	-	ENSG0000014545	GLRA3	-	4:174636913-174829314	-	-	IGV_LINK	0	0	0	0	OK	0	0	0	0	OK	NOTEST	0
ENSG0000014-	-	ENSG0000014547	CYP4V2	-	4:186191519-186258471	-	-	IGV_LINK	1417.35	25.8744	9.04978	42.699	OK	910.572	17.0033	5.78942	28.2172	OK	OK	-0.60571
ENSG0000014-	-	ENSG0000014549	ROPN1L	-	5:10441289-10472029	-	-	IGV_LINK	24.243	1.54146	0	3.50989	OK	43.3114	3.04666	0	6.3139	OK	OK	0.982933

ENSG0000014	-	-	ENSG0000014549	'NDUFS6	-	5:1801399-1816605	-	-	IGV_LINK	412.131	26.5207	9.27074	43.7707	OK		473.332	33.4055	10.6625	56.1484	OK		OK	0.332965
ENSG0000014	-	-	ENSG0000014549	'MARCH6	-	5:10353702-10440388	-	-	IGV_LINK	2879.1	50.5586	9.87873	91.2384	OK		2467.72	45.4067	8.86446	81.9488	OK		OK	-0.15505
ENSG0000014	-	-	ENSG0000014550	'NKD2	-	5:1008828-1038943	-	-	IGV_LINK	116.366	5.00975	0	10.5818	OK		123.747	5.70761	0	11.6041	OK		OK	0.18815
ENSG0000014	-	-	ENSG0000014552	'CDH18	-	5:19472950-20575873	-	-	IGV_LINK	0	0	0	0	OK		1.03122	0.080123	0	0.537621	OK		NOTEST	inf
ENSG0000014	-	-	ENSG0000014553	'ADAMTS16	-	5:5140329-5320304	-	-	IGV_LINK	3.87888	0.045481	0	0.659573	OK		1.03122	0.019033	0	0.627123	OK		NOTEST	-1.25673
ENSG0000014	-	-	ENSG0000014554	'SRD5A1	-	5:6633342-6674386	-	-	IGV_LINK	636.137	9.01589	0.837319	17.1945	OK		837.355	13.1063	0.151563	26.0611	OK		OK	0.539723
ENSG0000014	-	-	ENSG0000014555	'MYO10	-	5:16661913-16936276	-	-	IGV_LINK	480.012	3.76539	1.21742	6.31337	OK		1040.51	9.14868	2.35681	15.9406	OK		OK	1.28076
ENSG0000014	-	-	ENSG0000014556	'FAM105A	-	5:14581774-14615007	-	-	IGV_LINK	530.437	12.5288	0	26.5609	OK		430.021	10.1709	0	22.2836	OK		OK	-0.3008
ENSG0000014	-	-	ENSG0000014559	'RPL37	-	5:40825261-40835335	-	-	IGV_LINK	13942.6	1964.55	388.368	3540.74	OK		10927.9	1575.82	408.658	2742.99	OK		OK	-0.3181
ENSG0000014	-	-	ENSG0000014560	'SKP2	-	5:36151988-36184319	-	-	IGV_LINK	191.035	4.92787	0.677816	9.17792	OK		163.965	4.96198	0.630618	9.29334	OK		OK	0.009952
ENSG0000014	-	-	ENSG0000014562	'OSMR	-	5:38845857-39074408	-	-	IGV_LINK	2369.03	28.1687	3.98292	52.3545	OK		5828.48	66.4408	16.4593	116.422	OK		OK	1.23798
ENSG0000014	-	-	ENSG0000014562	'UGT3A1	-	5:35951009-36001028	-	-	IGV_LINK	307.401	5.55536	0	11.3527	OK		359.898	6.47028	0.40757	12.533	OK		OK	0.219949
ENSG0000014	-	-	ENSG0000014563	'PLK2	-	5:58453981-58460260	-	-	IGV_LINK	1491.43	61.8846	0	127.072	OK		1817.02	82.8849	0	169.145	OK		OK	0.421527
ENSG0000014	-	-	ENSG0000014564	'FAM159B	-	5:64690307-64718090	-	-	IGV_LINK	0	0	0	0	OK		0	0	0	0	OK		NOTEST	0
ENSG0000014	-	-	ENSG0000014564	'GZMA	-	5:55102647-55110252	-	-	IGV_LINK	607.045	47.4173	19.0456	75.7889	OK		243.369	19.0403	5.69235	32.3883	OK		OK	-1.31636
ENSG0000014	-	-	ENSG0000014567	'PIK3R1	-	5:68215719-68301821	-	-	IGV_LINK	952.266	10.1877	3.11871	17.2568	OK		4432.2	65.4777	14.8672	116.088	OK		OK	2.68417
ENSG0000014	-	-	ENSG0000014568	'HAPLN1	-	5:83637804-83721613	-	-	IGV_LINK	77.5776	1.85497	0	5.25045	OK		284.618	9.08123	0	19.8033	OK		OK	2.29149
ENSG0000014	-	-	ENSG0000014568	'LHFPL2	-	5:78485214-78770021	-	-	IGV_LINK	875.658	10.8727	4.02622	17.7192	OK		2534.75	32.4344	10.734	54.1348	OK		OK	1.57681
ENSG0000014	-	-	ENSG0000014568	'SSBP2	-	5:81413020-81751797	-	-	IGV_LINK	351.039	18.7412	3.54614	33.9363	OK		273.275	17.3768	2.84041	31.9132	OK		OK	-0.10905
ENSG0000014	-	-	ENSG0000014569	'BHMT	-	5:78997605-79236038	-	-	IGV_LINK	2374.85	60.099	0	122.754	OK		5199.44	130.109	9.89393	250.324	OK		OK	1.11431
ENSG0000014	-	-	ENSG0000014570	'ANKRD31	-	5:75068274-75236878	-	-	IGV_LINK	14.5458	0.164048	0	0.371884	OK		10.3122	0.09913	0	0.234492	OK		NOTEST	-0.72673
ENSG0000014	-	-	ENSG0000014570	'IQGAP2	-	5:76403248-76735781	-	-	IGV_LINK	692.38	13.1283	0	29.1803	OK		822.918	14.2088	0	31.0989	OK		OK	0.114103
ENSG0000014	-	-	ENSG0000014570	'CRHBP	-	5:76952712-76981158	-	-	IGV_LINK	20.3641	1.24211	0	3.09561	OK		30.9367	2.86587	0	6.82127	OK		OK	1.20618
ENSG0000014	-	-	ENSG0000014571	'RASA1	-	5:87267887-87413019	-	-	IGV_LINK	832.02	13.5979	2.98159	24.2143	OK		727.014	11.4807	2.14075	20.8206	OK		OK	-0.24418
ENSG0000014	-	-	ENSG0000014572	'LIX1	-	5:97089074-97437217	-	-	IGV_LINK	1.93944	0.028364	0	0.349859	OK		0	0	0	0	OK		NOTEST	'-inf
ENSG0000014	-	-	ENSG0000014572	'GIN1	-	5:103085999-103212799	-	-	IGV_LINK	111.518	2.55226	0	8.827	OK		62.9047	1.49382	0	5.42883	OK		OK	-0.77277
ENSG0000014	-	-	ENSG0000014572	'PPIP5K2	-	5:103085999-103212799	-	-	IGV_LINK	877.597	19.247	0	42.9458	OK		863.135	23.101	0	49.7197	OK		OK	0.263327
ENSG0000014	-	-	ENSG0000014573	'PAM	-	5:102753980-103031105	-	-	IGV_LINK	3108.92	85.0148	19.0531	150.976	OK		6240.97	159.223	27.2427	291.202	OK		OK	0.90526
ENSG0000014	-	-	ENSG0000014573	'BDP1	-	5:71455614-71567820	-	-	IGV_LINK	683.653	4.98501	1.27844	8.69158	OK		392.897	3.6053	1.18747	6.02313	OK		OK	-0.46748
ENSG0000014	-	-	ENSG0000014573	'GTF2H2	-	5:71032669-71067689	-	-	IGV_LINK	44.6071	1.84168	0	3.97222	OK		43.3114	1.87804	0	4.05192	OK		OK	0.028205
ENSG0000014	-	-	ENSG0000014574	'SLC30A5	-	5:69093645-69136394	-	-	IGV_LINK	568.256	19.7735	0	42.3492	OK		744.544	19.6078	0	41.682	OK		OK	-0.01214
ENSG0000014	-	-	ENSG0000014574	'BTF3	-	5:73498407-73505635	-	-	IGV_LINK	4657.57	361.974	148.23	575.719	OK		4577.61	358.543	147.717	569.369	OK		OK	-0.01374
ENSG0000014	-	-	ENSG0000014574	'FBXL17	-	5:107859034-108382098	-	-	IGV_LINK	416.01	14.161	0	32.4553	OK		443.427	14.0825	0	30.8517	OK		OK	-0.00801
ENSG0000014	-	-	ENSG0000014575	'SPATA9	-	5:95646753-95698711	-	-	IGV_LINK	2.90916	0.222374	0	0.867695	OK		7.21857	0.46497	0	1.41346	OK		NOTEST	1.06415
ENSG0000014	-	-	ENSG0000014577	'TSLP	-	5:111070061-111130502	-	-	IGV_LINK	29.0916	1.09935	0	4.34719	OK		40.2178	1.32808	0	4.58039	OK		OK	0.272697
ENSG0000014	-	-	ENSG0000014577	'TNFAIP8	-	5:119268691-119399688	-	-	IGV_LINK	962.932	28.7618	4.89937	52.6241	OK		1189	42.5136	7.53021	77.4971	OK		OK	0.563774
ENSG0000014	-	-	ENSG0000014578	'FEM1C	-	5:115520907-115544894	-	-	IGV_LINK	279.28	2.78887	0.840342	4.73739	OK		412.49	4.11994	1.50729	6.73259	OK		OK	0.562943
ENSG0000014	-	-	ENSG0000014578	'COMMD10	-	5:116083806-116412762	-	-	IGV_LINK	315.159	18.4799	3.68129	33.2785	OK		360.929	22.1646	5.11422	39.2151	OK		OK	0.262304
ENSG0000014	-	-	ENSG0000014578	'ATG12	-	5:115828199-115914081	-	-	IGV_LINK	670.077	24.1678	0	49.8132	OK		664.109	26.3072	0	58.0852	OK		OK	0.122372
ENSG0000014	-	-	ENSG0000014579	'MEGF10	-	5:127290830-127465737	-	-	IGV_LINK	1.93944	0.026713	0	0.549452	OK		5.15612	0.137249	0	0.766699	OK		NOTEST	2.36121
ENSG0000014	-	-	ENSG0000014580	'ADAMTS19	-	5:129459558-130186634	-	-	IGV_LINK	0	0	0	0	OK		0	0	0	0	OK		NOTEST	0
ENSG0000014	-	-	ENSG0000014581	'YIPF5	-	5:144158158-144170714	-	-	IGV_LINK	742.806	31.2057	5.37917	57.0323	OK		879.635	37.1715	10.5586	63.7844	OK		OK	0.252385
ENSG0000014	-	-	ENSG0000014581	'ARHGAP26	-	5:142770383-143229011	-	-	IGV_LINK	2333.15	24.8923	0	56.8763	OK		1140.53	12.6564	0	30.0693	OK		OK	-0.97583
ENSG0000014	-	-	ENSG0000014582	'CXCL14	-	5:135559576-135634874	-	-	IGV_LINK	12420.2	440.813	129.806	751.82	OK		26668.5	947.383	88.0473	1806.72	OK		OK	1.10378
ENSG0000014	-	-	ENSG0000014582	'LECT2	-	5:135922278-135955034	-	-	IGV_LINK	9.69721	0.885957	0	3.69426	OK		8.2498	0.746295	0	3.20316	OK		OK	-0.24749
ENSG0000014	-	-	ENSG0000014583	'SLC25A48	-	5:135834648-135888637	-	-	IGV_LINK	17.455	1.593	0	3.96317	OK		18.562	1.29123	0	3.12808	OK		OK	-0.303
ENSG0000014	-	-	ENSG0000014583	'DDX46	-	5:134758770-134859737	-	-	IGV_LINK	1484.64	25.4993	0	53.5641	OK		1485.7	27.4955	0	59.3085	OK		OK	0.108737
ENSG0000014	-	-	ENSG0000014583	'CDKN2AIPNLP2	-	2:26827168-26827520	-	-	IGV_LINK	0	0	0	0	OK		0	0	0	0	OK		NOTEST	0
ENSG0000014	-	-	ENSG0000014583	'IL9	-	5:135892245-135895827	-	-	IGV_LINK	0.969721	0.130591	0	0.511492	OK		0	0	0	0	OK		NOTEST	'-inf
ENSG0000014	-	-	ENSG0000014585	'TIMD4	-	5:156919281-156963255	-	-	IGV_LINK	134.791	6.47712	1.19672	11.7575	OK		106.216	5.00782	0.381779	9.63386	OK		OK	-0.37117
ENSG0000014	-	-	ENSG0000014586	'RNF145	-	5:159157408-159248796	-	-	IGV_LINK	1589.37	40.4346	13.9139	66.9553	OK		2970.96	76.7074	27.3127	126.102	OK		OK	0.923776
ENSG0000014	-	-	ENSG0000014586	'C1QTNF2	-	5:160347750-160370641	-	-	IGV_LINK	10.6669	0.268189	0	0.601658	OK		9.28102	0.233464	0	0.52682	OK		NOTEST	-0.20005
ENSG0000014	-	-	ENSG0000014586	'GABRA6	-	5:161288428-161702593	-	-	IGV_LINK	0	0	0	0	OK		0	0	0	0	OK		NOTEST	0
ENSG0000014	-	-	ENSG0000014586	'GABRB2	-	5:161288428-161702593	-	-	IGV_LINK	0	0	0	0	OK		0	0	0	0	OK		NOTEST	0
ENSG0000014	-	-	ENSG0000014586	'FBXO38	-	5:148383934-148442836	-	-	IGV_LINK	946.447	16.4587	3.11994	29.7975	OK		1011.63	23.0772	0.309977	45.8445	OK		OK	0.487616
ENSG0000014	-	-	ENSG0000014587	'SPINK7	-	5:148268179-148383783	-	-	IGV_LINK	0	0	0	0	OK		1.03122	0.799119	0	10.122	OK		OK	inf
ENSG0000014	-	-	ENSG0000014588	'PCYOX1L	-	5:149358006-149369653	-	-	IGV_LINK	339.402	6.22929	1.4495	11.0091	OK		301.118	5.68166	1.16081	10.2025	OK		OK	-0.13276
ENSG0000014	-	-	ENSG0000014588	'GLRA1	-	5:151822512-151924842	-	-	IGV_LINK	0.969721	0.035614	0	0.191435	OK		0	0	0	0	OK		NOTEST	'-inf
ENSG0000014	-	-	ENSG0000014590	'TNIP1	-	5:151029944-151093577	-	-	IGV_LINK	9615.75	239.155	46.3057	432.005	OK		9851.29	237.79	46.6464	428.934	OK		OK	-0.00826
ENSG0000014	-	-	ENSG0000014590	'G3BP1	-	5:151742315-151812785	-	-	IGV_LINK	2169.26	37.093	0	74.51	OK		2834.84	55.2551	1.84513	108.665	OK		OK	0.57496
ENSG0000014	-	-	ENSG0000014590	'ZNF300	-	5:150846522-150904983	-	-	IGV_LINK	486.8	8.0673	2.75746	13.3771	OK		312.461	5.03272	1.60764	8.45781	OK		OK	-0.68075
ENSG0000014	-	-	ENSG0000014591	'N4BP3	-	5:178113442-178126087	-	-	IGV_LINK	104.73	0.999088	0.118361	1.87981	OK		75.2794	0.71828	0	1.44145	OK		OK	-0.47606
ENSG0000014	-	-	ENSG0000014591	'NHP2	-	5:178130995-178153967	-	-	IGV_LINK	1153.97	97.7304	24.8914	170.569	OK		1342.65	113.584	32.9676	194.201	OK		OK	0.216887
ENSG0000014	-	-	ENSG0000014591	'RMND5B	-	5:178130995-178153967	-	-	IGV_LINK	813.596	22.2445	0	45.4857	OK		775.481	21.0136	0	47.1224	OK		OK	-0.08212
ENSG0000014	-	-	ENSG0000014591	'BOD1	-	5:173607513-173616660	-	-	IGV_LINK	748.624	39.6531	11.5737	67.7325	OK		777.544	39.9902	14.0544	65.926	OK		OK	0.012213
ENSG0000014	-	-	ENSG0000014592	'CPLX2	-	5:175796309-175884020	-	-	IGV_LINK	0	0	0	0	OK		1.03122	0.011984	0	0.620501	OK		NOTEST	inf
ENSG0000014	-	-	ENSG0000014593	'TENM2	-	5:167284798-168264157	-	-	IGV_LINK	20.3641	0.891839	0	3.12267	OK		22.6869	0.155255	0	0.743466	OK		OK	-2.52214
ENSG0000014	-	-	ENSG0000014593	'KCNMB1	-	5:170353486-170736632	-	-	IGV_LINK	223.036	4.23577	0.555752	7.91579	OK		81.4668	1.52893	0	3.2737	OK		OK	-1.4701
ENSG0000014	-	-	ENSG0000014594	'FAM50B	-	6:3831932-3855737	-	-	IGV_LINK	397.585	14.9485	5.03836	24.8586	OK		421.771	15.8595	5.12736	26.5916	OK		OK	0.08535
ENSG0000014	-	-	ENSG0000014594	'MYLK4	-	6:2663628-2750966	-	-	IGV_LINK	52.3649	0.523841	0.005578	1.0421	OK		23.7182	0.237317	0	0.523983	OK		NOTEST	-1.14231
ENSG0000014	-	-	ENSG0000014597	'FAM217A	-	6:4021266-4157385	-	-	IGV_LINK	0.969721	0.031113	0	1.13971	OK		0	0	0	0	OK		NOTEST	'-inf
ENSG0000014	-	-	ENSG0000014597	'TBC1D7	-	6:12716804-13328583	-	-	IGV_LINK	204.611	12.5117	0	27.3159	OK		128.376	7.33766	0	16.9073	OK		OK	-0.76989
ENSG0000014	-	-	ENSG0000014598	'FARS2	-	6:5261043-5771580	-	-	IGV_LINK	367.524	14.6076	3.77606	25.4391	OK		240.275	9.46495	1.8967	17.0332	OK		OK	-0.62605
ENSG0000014	-	-	ENSG0000014599	'GFOD1	-	6:13357829-13487662	-	-	IGV_LINK	174.55	1.34253	0.166707	2.51836	OK		124.778	0.859423	0.051744	1.6671	OK		OK	-0.64352
ENSG0000014	-	-	ENSG0000014599	'CDKAL1	-	6:20534456-21232404	-	-	IGV_LINK	260.855	4.89722	1.41944	8.37499	OK		213.464	4.21564	1.07837	7.3529	OK		OK	-0.21621
ENSG0000014	-	-	ENSG0000014600	'PCDHB18P	-	5:141100241-141249365	-	-	IGV_LINK	12.6064	0.301062	0	2.81116	OK		5.15612	0.166333	0	2.57908	OK		NOTEST	-0.85598
ENSG0000014	-	-	ENSG0000014600	'PSD2	-	5:139795820-139844466	-	-	IGV_LINK	156.125	2.02708	0.380894	3.67326	OK		68.0608	0.883915	0.000437	1.76739	OK		OK	-1.19742
ENSG0000014	-	-	ENSG0000014600	'LRRTM2	-	5:138610966-138935034	-	-	IGV_LINK	5.81832	0.056103	0	1.23781	OK		1.03122	0.03257	0	1.97884	OK		NOTEST	-0.78455
ENSG0000014	-	-	ENSG0000014600	'ZMAT2	-	5:140664675-140706676	-	-	IGV_LINK	1579.64	79.7124	0	185.377	OK		2240.28	112.994	0	243.316	OK		OK	0.503372
ENSG0000014	-	-	ENSG0000014601	'GFRA3	-	5:138252378-138274671	-	-	IGV_LINK	3.87888	0.131328	0	0.341974	OK		0	0	0	0	OK		NOTEST	'-inf
ENSG0000014	-	-	ENSG0000014602	'KLHL3	-	5:137617499-137736090	-	-	IGV_LINK	91.1537	0.938553	0	2.14905	OK		71.1545	0.680863	0	1.66191	OK		OK	-0.46307
ENSG0000014	-	-	ENSG0000014603	'DCDC2	-	6:24171755-24358284	-	-	IGV_LINK	658.44	8.23028	3.00038	13.4602	OK		58.7798	0.730713	0	1.55624	OK		OK	-3.49356
ENSG0000014	-	-	ENSG0000014603	'SLC17A4	-	6:25754698-25781191	-	-	IGV_LINK	111.518	2.02234	0.143918	3.90076	OK		21.6557	0.382461	0	0.817617	OK		OK	-2.40264
ENSG0000014	-	-	ENSG0000014604	'HIST1H2BA	-	6:25726776-25727292	-	-	IGV_LINK	2.90916	0.47728	0	1.28101	OK		0	0	0	0	OK		NOTEST	'-inf
ENSG0000014	-	-	ENSG0000014604	'KAAG1	-	6:24171755-24358284	-	-	IGV_LINK	76.6079	3.56954	0	10.03	OK		11.3435	0.529052	0	1.77355	OK		OK	-2.75426
ENSG0000014	-	-	ENSG0000014605	'TRIM7	-	5:181191923-181205293	-	-	IGV_LINK	97.9418	3.06074	0	7.12987	OK		32.9992	0.598733	0	1.3207	OK		OK	-2.35389
ENSG0000014	-	-	ENSG0000014606	'TRIM41	-	5:181222498-181235809	-	-	IGV_LINK	1332.4	18.9975	6.33261	31.6624	OK		1560.24	21.7477	6.34736	37.1481	OK		OK	0.195055
ENSG0000014	-	-	ENSG0000014606	'HIGD2A	-	5:176365467-176416569	-	-	IGV_LINK	1258.7	143.165	15.8312	270.499	OK		1695.33	193.277	22.5592	363.995	OK		OK	0.432991
ENSG0000014	-	-	ENSG0000014606	'FAM193B	-	5:177519787-177554541	-	-	IGV_LINK	2942.13	115.491	12.8269	218.155	OK		1959.33	78.0765	11.8918	144.261	OK		OK	-0.56482
ENSG0000014	-	-	ENSG0000014607	'PLA2G7	-	6:46652914-46735693	-	-	IGV_LINK	139.64	4.74547	0	11.4505	OK		505.3	17.2183	5.00839	29.4282	OK		OK	1.85932
ENSG0000014	-	-	ENSG0000014607	'TNFRSF21	-	6:47231531-47309905	-	-	IGV_LINK	884.385	14.5625	6.20168	22.9234	OK		1688.12	27.8063	12.1583	43.4543	OK		OK	0.933152
ENSG0000014	-	-	ENSG0000014608	'RNF44	-	5:176526696-176538025	-	-	IGV_LINK	2308.9	60.2235	8.08391	112.363	OK		2501.75	60.4663	7.54134	113.391	OK		OK	0.005803
ENSG0000014	-	-	ENSG0000014608	'MUT	-	6:49430359-49463191	-	-	IGV_LINK	566.317	8.92359	3.53323	14.3139	OK		399.084	6.29049	2.27942	10.3016	OK		OK	-0.50445
ENSG0000014	-	-	ENSG0000014609	'RASGEF1C	-	5:180100790-180209153	-	-	IGV_LINK	0	0	0	0	OK		13.4059	0.365098	0	0.928064	OK		NOTEST	inf
ENSG0000014	-	-	ENSG0000014609	'DOK3	-	5:177483393-177511274	-	-	IGV_LINK	614.803	22.6655	1.52873	43.8024	OK		863.135	31.892	4.47511	59.3089	OK		OK	0.492694
ENSG0000014	-	-	ENSG0000014610	'ABT1	-	6:26596951-26600744	-	-	IGV_LINK	467.405	9.48714	3.58871	15.3856	OK		280.493	5.69566	1.71919	9.67212	OK		OK	-0.73611
ENSG0000014	-	-	ENSG0000014611	'PPP1R18	-	6:30676388-30687895	-	-	IGV_LINK	2242.96	52.6187	18.7873	86.4501	OK		2024.29	48.2534	15.1671	81.3397	OK		OK	-0.12495
ENSG0000014	-	-	ENSG0000014612	'DAAM2	-	6:39792297-39934551	-	-	IGV_LINK	898.931	9.04108	0.124774	17.9574	OK		2198.57	26.4673	0	77.2328	OK		OK	1.54964
ENSG0000014	-	-	ENSG0000014614	'PRIM2	-	6:57314804-57646849	-	-	IGV_LINK	193.944	5.11832	1.09431	9.14233	OK		114.466	3.0224	0.333406	5.7114	OK		OK	-0.75997
ENSG0000014	-	-	ENSG0000014614	'MLIP	-	6:53929981-54266280	-	-	IGV_LINK	84.7946	6.84395	0	15.0299	OK		93.8465	8.95286	0	21.4761	OK		OK	0.38752
ENSG0000014	-	-	ENSG0000014615	'HMGCLL1	-	6:55434368-55579214	-	-	IGV_LINK	1.93944	0.185098	0	0.763681	OK		0	0	0	0	OK		NOTEST	'-inf
ENSG0000014	-	-	ENSG0000014616	'LGSN	-	6:63275950-63319977	-	-	IGV_LINK	5.81832	0.12044	0	0.508493	OK		1.03122	0.133806	0	0.512968	OK		NOTEST	0.151832
ENSG0000014	-	-	ENSG0000014619	'FGD2	-	6:37005645-37029070	-	-	IGV_LINK	1168.51	32.2533	7.02894	57.4776	OK		859.01	22.89	5.9449	39.835	OK		OK	-0.49473
ENSG0000014	-	-	ENSG0000014619	'SCUBE3	-	6:35214418-35253079	-	-	IGV_LINK	15.5155	0.121721	0	0.41901	OK		13.4059	0.105188	0	0.318633	OK		NOTEST	-0.21061
ENSG0000014	-	-	ENSG0000014620	'ANO7	-	2:241188508-241225377	-	-	IGV_LINK	64.0016	2.16019	0	4.7426	OK		75.2794	2.53705	0	5.72283	OK		OK	0.231994
ENSG0000014	-	-	ENSG0000014621	'CRIP3	-	6:43295693-43369478	-	-	IGV_LINK	43.4152	4.33981	0	16.7283	OK		24.6065	2.22301	0	9.02097	OK		OK	-0.96512
ENSG0000014	-	-	ENSG0000014621	'TTBK1	-	6:43243679-43288259	-	-	IGV_LINK	32.0008	0.331862	0	0.742698	OK		15.4684	0.20016	0	0.472897	OK		NOTEST	-0.72943
ENSG0000014	-	-	ENSG0000014622	'TCTE1	-	6:44270465-44378957	-	-	IGV_LINK	3.87888	0.075522	0	0.656453	OK		2.06245	0.040172	0	0.463414	OK		NOTEST	-0.91071
ENSG0000014	-	-	ENSG0000014622	'RPL7L1	-	6:42879617-42889925	-	-	IGV_LINK	1744.53	33.9443	5.21505	62.6735	OK		1477.75	26.2695	5.96594	46.573	OK		OK	-0.36978
ENSG0000014	-	-	ENSG0000014623	'NFKBIE	-	6:44258165-44265788	-	-	IGV_LINK	1024.02	40.3542	9.73205	70.9763	OK		651.734	25.256	5.07267	45.4394	OK		OK	-0.67609
ENSG0000014	-	-	ENSG0000014623	'CYP39A1	-	6:46549579-46652830	-	-	IGV_LINK	30.0613	1.10261	0	2.93516	OK		20.6245	1.12694	0	3.22946	OK		OK	0.031493
ENSG0000014	-	-	ENSG0000014624	'TPBG	-	6:82363205-82370828	-	-	IGV_LINK	246.309	4.7739	1.40106	8.14675	OK		216.557	4.25173	1.01378	7.48969	OK		OK	-0.16712
ENSG0000014	-	-	ENSG0000014624	'IRAK1BP1	-	6:78867471-79078236	-	-	IGV_LINK	90.184	1.33396	0	4.33081	OK		38.1553	0.6028	0	2.08383	OK		OK	-1.14596
ENSG0000014	-	-	ENSG0000014624	'PHIP	-	6:78867471-79078236	-	-	IGV_LINK	789.352	4.66238	1.77155	7.5532	OK		302.149	1.76024	0.502126	3.01836	OK		OK	-1.40529
ENSG0000014	-	-	ENSG0000014625	'PRSS35	-	6:83512537-83525704	-	-	IGV_LINK	0	0	0	0	OK		22.6869	0.564143	0	1.24287	OK		OK	inf
ENSG0000014	-	-	ENSG0000014626	'MMS22L	-	6:97142160-97283217	-	-	IGV_LINK	191.035	2.38932	0	6.81565	OK		80.4355	0.775992	0	2.12392	OK		OK	-1.62249
ENSG0000014	-	-	ENSG0000014626	'FAXC	-	6:99271168-99350062	-	-	IGV_LINK	1.93944	0.071358	0	0.372182	OK		3.09367	0.015382	0	0.264367	OK		NOTEST	-2.21383
ENSG0000014	-	-	ENSG0000014627	'GABRR1	-	6:89177500-89231278	-	-	IGV_LINK	0	0	0	0	OK		0	0	0	0	OK		NOTEST	0
ENSG0000014	-	-	ENSG0000014627	'PNRC1	-	6:89080750-89085160	-	-	IGV_LINK	1756.16	70.1904	17.4255	122.955	OK		1233.35	49.0105	12.5902	85.4308	OK		OK	-0.51818
ENSG0000014	-	-	ENSG0000014628	'PM20D2	-	6:89146049-89165565	-	-	IGV_LINK	410.192	5.09682	1.86162	8.33202	OK		194.902	2.42236	0.627822	4.21689	OK		OK	-1.07319
ENSG0000014	-	-	ENSG0000014628	'RARS2	-	6:87514377-87590003	-	-	IGV_LINK	496.497	17.945	6.24057	29.6495	OK		321.742	11.7298	3.48253	19.9771	OK		OK	-0.6134
ENSG0000014	-	-	ENSG0000014628	'SCML4	-	6:107704103-107824317	-	-	IGV_LINK	102.79	2.97956	0	6.56503	OK		85.5917	2.2537	0	4.59257	OK		OK	-0.4028
ENSG0000014	-	-	ENSG0000014635	'TBC1D32	-	6:121079493-121334745	-	-	IGV_LINK	76.6079	1.64184	0	3.72544	OK		55.6861	1.76119	0	4.38016	OK		OK	0.101233
ENSG0000014	-	-	ENSG0000014635	'CLVS2	-	6:122995970-123072927	-	-	IGV_LINK	1.93944	0.009662	0	0.094727	OK		0	0	0	0	OK		NOTEST	'-inf
ENSG0000014	-	-	ENSG0000014636	'GPR6	-	6:109978255-109980718	-	-	IGV_LINK	0	0	0	0	OK		0	0	0	0	OK		NOTEST	0
ENSG0000014	-	-	ENSG0000014637	'RNF217	-	6:124909092-125092633	-	-	IGV_LINK	360.736	10.3782	0	24.1666	OK		207.276	5.17302	0	12.4624	OK		OK	-1.00448
ENSG0000014	-	-	ENSG0000014637	'RSPO3	-	6:127118603-127197765	-	-	IGV_LINK	0	0	0	0	OK		3.09367	0.103264	0	0.396011	OK		NOTEST	inf
ENSG0000014	-	-	ENSG0000014637	'ARHGAP18	-	6:129576131-129710225	-	-	IGV_LINK	900.87	16.1933	0	34.0264	OK		956.977	15.5077	0	31.088	OK		OK	-0.06241
ENSG0000014	-	-	ENSG0000014637	'TAAR2	-	6:132617021-132624275	-	-	IGV_LINK	0	0	0	0	OK		0	0	0	0	OK		NOTEST	0
ENSG0000014	-	-	ENSG0000014638	'TAAR6	-	6:132570321-132571359	-	-	IGV_LINK	0	0	0	0	OK		0	0	0	0	OK		NOTEST	0
ENSG0000014	-	-	ENSG0000014638	'TAAR8	-	6:132552692-132553721	-	-	IGV_LINK	0	0	0	0	OK		0	0	0	0	OK		NOTEST	0
ENSG0000014	-	-	ENSG0000014638	'ABRACL	-	6:139028681-139043302	-	-	IGV_LINK	210.429	17.7461	4.82192	30.6703	OK		181.496	15.3552	3.60131	27.109	OK		OK	-0.20878

ENSG0000014	-	-	ENSG0000014639	'TAAR1	-	6:132644983-132646003	-	-	IGV_LINK	0.969721	0.064485	0	0.25257	OK	2.06245	0.137336	0	0.40369	OK	NOTEST	1.09068
ENSG0000014	-	-	ENSG0000014640	'SLC18B1	-	6:132769369-132798553	-	-	IGV_LINK	336.493	8.6374	2.796	14.4788	OK	370.21	9.50786	3.33826	15.6775	OK	OK	0.138523
ENSG0000014	-	-	ENSG0000014641	'MTFR2	-	6:136231023-136250335	-	-	IGV_LINK	32.0008	1.65515	0	3.83418	OK	28.8743	1.50829	0	3.38629	OK	OK	-0.13405
ENSG0000014	-	-	ENSG0000014641	'SLC2A12	-	6:133952169-134052636	-	-	IGV_LINK	16.4852	0.216282	0	0.756584	OK	16.4996	0.216528	0	0.691146	OK	NOTEST	0.001642
ENSG0000014	-	-	ENSG0000014641	'SHPRH	-	6:145793501-145964423	-	-	IGV_LINK	417.95	3.8095	0	9.0983	OK	220.682	2.23557	0	6.75103	OK	OK	-0.76896
ENSG0000014	-	-	ENSG0000014641	'AIG1	-	6:143060495-143340304	-	-	IGV_LINK	1383.79	77.8116	27.4226	128.201	OK	768.263	43.2784	14.0642	72.4926	OK	OK	-0.84634
ENSG0000014	-	-	ENSG0000014642	'DYNLT1	-	6:158636473-158644739	-	-	IGV_LINK	1426.46	131.606	55.5279	207.683	OK	1032.26	95.4088	37.4673	153.35	OK	OK	-0.46403
ENSG0000014	-	-	ENSG0000014642	'TIAM2	-	6:154733324-155314493	-	-	IGV_LINK	152.833	4.42844	0	14.2865	OK	60.2252	2.91924	0	10.4995	OK	OK	-0.6012
ENSG0000014	-	-	ENSG0000014643	'TMEM181	-	6:158536435-158635428	-	-	IGV_LINK	374.312	4.04305	1.28235	6.80374	OK	459.926	4.96888	1.76103	8.17673	OK	OK	0.297479
ENSG0000014	-	-	ENSG0000014645	'PNLDC1	-	6:159800248-159820704	-	-	IGV_LINK	0.969721	0.033247	0	0.439471	OK	1.03122	0.03538	0	0.442536	OK	NOTEST	0.089707
ENSG0000014	-	-	ENSG0000014645	'WTAP	-	6:159669056-159789749	-	-	IGV_LINK	1611.68	48.9019	0	249.423	OK	1336.47	43.5606	0	380.374	OK	OK	-0.16687
ENSG0000014	-	-	ENSG0000014646	'ZMYM4	-	1:35268966-35422058	-	-	IGV_LINK	1098.69	10.9616	0	22.7118	OK	1154.97	12.4983	0	27.3385	OK	OK	0.189269
ENSG0000014	-	-	ENSG0000014646	'VIP	-	6:152750797-152759765	-	-	IGV_LINK	31.0311	1.29102	0	2.72922	OK	13.4059	0.54948	0	1.21463	OK	OK	-1.23237
ENSG0000014	-	-	ENSG0000014647	'ARMT1	-	6:151452257-151470101	-	-	IGV_LINK	389.828	9.28076	3.20562	15.3559	OK	321.742	7.54765	2.41393	12.6814	OK	OK	-0.29822
ENSG0000014	-	-	ENSG0000014647	'SLC22A3	-	6:160348267-160452581	-	-	IGV_LINK	64.9713	1.15305	0.011379	2.29472	OK	101.06	1.79417	0.225921	3.36242	OK	OK	0.637862
ENSG0000014	-	-	ENSG0000014652	'LINC01558	-	6:167784536-167798248	-	-	IGV_LINK	7.75776	0.393356	0	1.04589	OK	7.21857	0.364838	0	0.975171	OK	NOTEST	-0.10858
ENSG0000014	-	-	ENSG0000014653	'VWDE	-	7:12330884-12403941	-	-	IGV_LINK	12.6064	0.344336	0	1.10823	OK	8.2498	0.235398	0	0.826337	OK	NOTEST	-0.54871
ENSG0000014	-	-	ENSG0000014653	'GNA12	-	7:2679521-2844324	-	-	IGV_LINK	722.442	10.9985	4.01972	17.9773	OK	952.852	14.3365	5.00303	23.67	OK	OK	0.382386
ENSG0000014	-	-	ENSG0000014654	'C7orf50	-	7:996985-1138260	-	-	IGV_LINK	683.653	38.4972	8.1072	68.8872	OK	705.358	39.4701	9.593	69.3471	OK	OK	0.036005
ENSG0000014	-	-	ENSG0000014655	'SDK1	-	7:3264031-4269000	-	-	IGV_LINK	152.246	0.927765	0	2.02412	OK	391.865	3.16013	0	7.25633	OK	OK	1.76815
ENSG0000014	-	-	ENSG0000014655	'WASH2P	-	2:113588549-113601261	-	-	IGV_LINK	548.862	12.6029	4.78419	20.4216	OK	484.676	11.0484	4.04434	18.0525	OK	OK	-0.18991
ENSG0000014	-	-	ENSG0000014657	'CCZ1B	-	7:6754108-6826770	-	-	IGV_LINK	192.005	9.72455	0	19.7617	OK	237.182	10.6099	0.259777	20.9601	OK	OK	0.125711
ENSG0000014	-	-	ENSG0000014657	'C7orf26	-	7:6590016-6608726	-	-	IGV_LINK	352.009	12.5352	2.09921	22.9712	OK	362.991	12.7802	2.28904	23.2713	OK	OK	0.027926
ENSG0000014	-	-	ENSG0000014658	'RBAK	-	7:4973987-5073223	-	-	IGV_LINK	385.373	3.55526	0.303675	6.80684	OK	420.122	3.90977	0.504121	7.31543	OK	OK	0.137131
ENSG0000014	-	-	ENSG0000014659	'CREB5	-	7:28299320-28825894	-	-	IGV_LINK	843.657	8.48453	0	18.7	OK	1101.35	16.6723	0	36.4368	OK	OK	0.974549
ENSG0000014	-	-	ENSG0000014661	'FERD3L	-	7:19144292-19146253	-	-	IGV_LINK	0	0	0	0	OK	0	0	0	0	OK	NOTEST	0
ENSG0000014	-	-	ENSG0000014664	'EGFR	-	7:55019020-55211628	-	-	IGV_LINK	4285.19	27.5157	2.58619	52.4452	OK	3361.79	20.6819	3.83293	37.531	OK	OK	-0.41188
ENSG0000014	-	-	ENSG0000014666	'LINC00525	-	7:47695729-47979581	-	-	IGV_LINK	0.969721	0.053816	0	0.701013	OK	0	0	0	0	OK	NOTEST	'-inf
ENSG0000014	-	-	ENSG0000014667	'CDCA5	-	11:65066299-65084164	-	-	IGV_LINK	121.215	3.47807	0.126407	6.82973	OK	159.84	4.09993	0.475781	7.72407	OK	OK	0.237312
ENSG0000014	-	-	ENSG0000014667	'IGFBP3	-	7:45912244-45921874	-	-	IGV_LINK	9773.81	262.979	81.7924	444.165	OK	14647.5	416.633	64.0499	769.216	OK	OK	0.66383
ENSG0000014	-	-	ENSG0000014667	'PURB	-	7:44876292-44886393	-	-	IGV_LINK	1055.06	6.68332	2.80424	10.5624	OK	820.855	5.20044	2.14794	8.25294	OK	OK	-0.36193
ENSG0000014	-	-	ENSG0000014667	'AC004453.1	-	7:44379120-44490880	-	-	IGV_LINK	0	0	0	0	OK	0	0	0	0	OK	NOTEST	0
ENSG0000014	-	-	ENSG0000014667	'IGFBP1	-	7:45888356-45893668	-	-	IGV_LINK	0.969721	0.045673	0	0.217999	OK	97.9664	3.75349	0.119808	7.38718	OK	OK	6.36076
ENSG0000014	-	-	ENSG0000014670	'SSC4D	-	7:76389333-76442071	-	-	IGV_LINK	17.455	0.675897	0	2.13759	OK	30.9367	1.32693	0	3.77019	OK	OK	0.97322
ENSG0000014	-	-	ENSG0000014670	'MDH2	-	7:76048050-76067508	-	-	IGV_LINK	1902.59	70.3976	19.0898	121.705	OK	2635.81	101.925	26.1674	177.682	OK	OK	0.533908
ENSG0000014	-	-	ENSG0000014670	'POMZP3	-	7:76549359-76627982	-	-	IGV_LINK	65.941	3.29315	0	7.40466	OK	68.0608	3.37993	0	7.67065	OK	OK	0.037525
ENSG0000014	-	-	ENSG0000014672	'AC211486.1	-	7:75344014-75486271	-	-	IGV_LINK	10.6669	0.744469	0	7.48167	OK	13.4059	0.936965	0	8.69742	OK	OK	0.331783
ENSG0000014	-	-	ENSG0000014672	'NIPSNAP2	-	7:55887276-56000181	-	-	IGV_LINK	782.564	26.7976	6.46721	47.1279	OK	668.234	22.3108	5.90601	38.7157	OK	OK	-0.26436
ENSG0000014	-	-	ENSG0000014673	'CCT6A	-	7:56051629-56063989	-	-	IGV_LINK	1774.59	54.2793	14.2885	94.2702	OK	1814.96	60.6429	15.6717	105.614	OK	OK	0.159935
ENSG0000014	-	-	ENSG0000014673	'PSPH	-	7:56011050-56051604	-	-	IGV_LINK	160.004	7.92516	0	16.4432	OK	125.809	8.19475	0.226434	16.1631	OK	OK	0.048261
ENSG0000014	-	-	ENSG0000014675	'TRIM50	-	7:73312538-73328082	-	-	IGV_LINK	2.90916	0.11493	0	0.471891	OK	11.3435	0.341248	0	0.899628	OK	NOTEST	1.57006
ENSG0000014	-	-	ENSG0000014675	'ZNF92	-	7:65373798-65401135	-	-	IGV_LINK	84.3657	1.59661	0.119037	3.07419	OK	79.4043	1.4868	0.031147	2.94246	OK	OK	-0.1028
ENSG0000014	-	-	ENSG0000014677	'ATXN7L1	-	7:105605066-105876604	-	-	IGV_LINK	310.311	8.47135	0	18.6931	OK	377.428	11.2071	0	23.9506	OK	OK	0.403755
ENSG0000014	-	-	ENSG0000014680	'TMEM168	-	7:112762381-112790592	-	-	IGV_LINK	362.675	12.2578	0.571582	23.9441	OK	411.459	12.8654	0.867965	24.8628	OK	OK	0.069787
ENSG0000014	-	-	ENSG0000014680	'ASB15	-	7:123567009-123639481	-	-	IGV_LINK	0	0	0	0	OK	0	0	0	0	OK	NOTEST	0
ENSG0000014	-	-	ENSG0000014682	'C7orf43	-	7:100148906-100158715	-	-	IGV_LINK	422.798	17.4516	0	42.3046	OK	509.425	19.1103	0	44.5573	OK	OK	0.130993
ENSG0000014	-	-	ENSG0000014682	'SLC12A9	-	7:100802564-100867009	-	-	IGV_LINK	1181.12	21.9256	2.39808	41.4532	OK	1325.12	28.5471	3.21683	53.8774	OK	OK	0.380728
ENSG0000014	-	-	ENSG0000014683	'GIGYF1	-	7:100679506-100689448	-	-	IGV_LINK	2524.18	25.9465	0.921274	50.9718	OK	1784.02	20.092	0	41.8233	OK	OK	-0.36892
ENSG0000014	-	-	ENSG0000014683	'TRIM4	-	7:99875986-99919600	-	-	IGV_LINK	654.561	13.5048	4.33337	22.6761	OK	719.795	15.9091	4.6889	27.1293	OK	OK	0.236382
ENSG0000014	-	-	ENSG0000014683	'MEPCE	-	7:100400825-100434126	-	-	IGV_LINK	860.142	20.3994	7.25232	33.5465	OK	1073.51	25.0681	9.05176	41.0845	OK	OK	0.297327
ENSG0000014	-	-	ENSG0000014683	'ZAN	-	7:100733625-100797797	-	-	IGV_LINK	0.969721	0.00665	0	0.045405	OK	0	0	0	0	OK	NOTEST	'-inf
ENSG0000014	-	-	ENSG0000014684	'TMEM209	-	7:130164714-130207770	-	-	IGV_LINK	250.188	5.43699	1.27339	9.60058	OK	415.584	9.07457	2.31904	15.8301	OK	OK	0.739022
ENSG0000014	-	-	ENSG0000014685	'AGBL3	-	7:134986507-135170795	-	-	IGV_LINK	264.734	8.58827	0	39.1403	OK	215.526	5.98896	0	33.5408	OK	OK	-0.52006
ENSG0000014	-	-	ENSG0000014685	'STRA8	-	7:135231978-135317752	-	-	IGV_LINK	2.90916	0.19982	0	1.27274	OK	1.03122	0.070931	0	0.918943	OK	NOTEST	-1.49422
ENSG0000014	-	-	ENSG0000014685	'ZC3HAV1L	-	7:139025705-139036029	-	-	IGV_LINK	105.7	3.76896	0.459603	7.07831	OK	109.31	3.90053	0.427037	7.37402	OK	OK	0.049505
ENSG0000014	-	-	ENSG0000014685	'TMEM140	-	7:134986507-135170795	-	-	IGV_LINK	2684.19	82.9107	23.3268	142.495	OK	3810.38	117.771	33.9622	201.58	OK	OK	0.506358
ENSG0000014	-	-	ENSG0000014687	'TLK2	-	17:62458657-62615481	-	-	IGV_LINK	532.377	13.0864	0	26.6428	OK	550.674	16.3638	0	34.1884	OK	OK	0.322435
ENSG0000014	-	-	ENSG0000014690	'EPHA1	-	7:143381079-143523449	-	-	IGV_LINK	128.973	4.84233	0	21.2411	OK	87.6541	3.13172	0	17.5314	OK	OK	-0.62875
ENSG0000014	-	-	ENSG0000014690	'NOM1	-	7:156949722-156973182	-	-	IGV_LINK	408.252	8.81455	0	19.7172	OK	421.771	7.47203	0	16.1009	OK	OK	-0.23839
ENSG0000014	-	-	ENSG0000014691	'CNPY1	-	7:155474205-155781485	-	-	IGV_LINK	0.005693	0.000219	0	0.240843	OK	0	0	0	0	OK	NOTEST	'-inf
ENSG0000014	-	-	ENSG0000014691	'NCAPG2	-	7:158631310-158704829	-	-	IGV_LINK	324.856	5.28162	1.45385	9.10939	OK	323.805	5.21901	1.35443	9.08358	OK	OK	-0.01721
ENSG0000014	-	-	ENSG0000014692	'ASB10	-	7:151175697-151187832	-	-	IGV_LINK	0	0	0	0	OK	0	0	0	0	OK	NOTEST	0
ENSG0000014	-	-	ENSG0000014693	'NLGN4X	-	X:5840636-6228863	-	-	IGV_LINK	28.1219	0.47461	0	1.21516	OK	19.5933	0.232169	0	0.712906	OK	NOTEST	-1.03157
ENSG0000014	-	-	ENSG0000014695	'SHROOM2	-	X:9786455-9949443	-	-	IGV_LINK	73.6988	0.592554	0	1.20522	OK	97.9664	0.891649	0.0324	1.7509	OK	OK	0.58953
ENSG0000014	-	-	ENSG0000014695	'RAB19	-	7:140293692-140426250	-	-	IGV_LINK	25.2127	1.82304	0	7.78583	OK	3.09367	0.369907	0	2.83182	OK	OK	-2.30111
ENSG0000014	-	-	ENSG0000014696	'LUC7L2	-	7:139339456-139423457	-	-	IGV_LINK	1101.32	26.0606	3.63439	48.4868	OK	881.018	20.8909	1.90049	39.8813	OK	OK	-0.319
ENSG0000014	-	-	ENSG0000014696	'DENND2A	-	7:140518419-140924764	-	-	IGV_LINK	260.855	4.45206	0	12.867	OK	163.965	2.98985	0	11.1935	OK	OK	-0.5744
ENSG0000014	-	-	ENSG0000014700	'TMEM27	-	X:15627317-15665031	-	-	IGV_LINK	1456.52	58.4022	25.9024	90.902	OK	1139.5	45.7282	19.9075	71.5489	OK	OK	-0.35294
ENSG0000014	-	-	ENSG0000014701	'SH3KBP1	-	X:19533974-19887601	-	-	IGV_LINK	1227.67	25.373	7.11557	43.6304	OK	912.634	18.6903	5.44872	31.9319	OK	OK	-0.441
ENSG0000014	-	-	ENSG0000014702	'TMEM47	-	X:34627063-34657288	-	-	IGV_LINK	523.649	7.60289	2.83793	12.3679	OK	262.962	3.8191	1.18509	6.45311	OK	OK	-0.99332
ENSG0000014	-	-	ENSG0000014703	'LANCL3	-	X:37349274-38688920	-	-	IGV_LINK	17.455	0.186552	0	2.62972	OK	36.0929	0.28073	0	2.53199	OK	NOTEST	0.589609
ENSG0000014	-	-	ENSG0000014704	'SYTL5	-	X:37349274-38688920	-	-	IGV_LINK	0	0	0	0	OK	0	0	0	0	OK	NOTEST	0
ENSG0000014	-	-	ENSG0000014704	'CASK	-	X:41514933-41923463	-	-	IGV_LINK	461.587	7.15036	0	16.976	OK	360.929	6.50993	0	17.6043	OK	OK	-0.13538
ENSG0000014	-	-	ENSG0000014705	'KDM6A	-	X:44873176-45112602	-	-	IGV_LINK	476.133	10.5911	0	21.4282	OK	433.114	7.51115	0	15.076	OK	OK	-0.49575
ENSG0000014	-	-	ENSG0000014705	'SPIN2A	-	X:57134529-57137625	-	-	IGV_LINK	16.4852	0.404724	0	0.887949	OK	14.4371	0.354619	0	0.835003	OK	NOTEST	-0.19067
ENSG0000014	-	-	ENSG0000014706	'MSN	-	X:65588376-65741931	-	-	IGV_LINK	10821.1	163.19	49.9809	276.398	OK	11415.7	173.166	48.9731	297.359	OK	OK	0.085609
ENSG0000014	-	-	ENSG0000014708	'AKAP4	-	X:50190767-50201013	-	-	IGV_LINK	0	0	0	0	OK	0	0	0	0	OK	NOTEST	0
ENSG0000014	-	-	ENSG0000014708	'CCNB3	-	X:50202712-50351910	-	-	IGV_LINK	14.5458	0.261686	0	0.904791	OK	29.9055	0.375501	0	1.05032	OK	NOTEST	0.52098
ENSG0000014	-	-	ENSG0000014709	'HDAC8	-	X:72329515-72573103	-	-	IGV_LINK	227.884	8.25969	0.283436	16.236	OK	337.211	15.1432	0.60311	29.6833	OK	OK	0.874512
ENSG0000014	-	-	ENSG0000014710	'SLC16A2	-	X:74421460-74533917	-	-	IGV_LINK	490.679	7.01067	2.51599	11.5053	OK	283.587	4.6272	1.29862	7.95579	OK	OK	-0.59941
ENSG0000014	-	-	ENSG0000014711	'CXorf36	-	X:45148373-45333917	-	-	IGV_LINK	601.227	8.69253	2.26959	15.1155	OK	691.952	10.3523	3.80222	16.9024	OK	OK	0.252105
ENSG0000014	-	-	ENSG0000014711	'ZNF157	-	X:47370582-47414305	-	-	IGV_LINK	10.6669	0.280189	0	0.634298	OK	4.1249	0.108407	0	0.274624	OK	NOTEST	-1.36994
ENSG0000014	-	-	ENSG0000014711	'ZNF182	-	X:47836901-48071658	-	-	IGV_LINK	446.933	7.57352	1.8256	13.3214	OK	364.022	6.16217	1.51041	10.8139	OK	OK	-0.29753
ENSG0000014	-	-	ENSG0000014711	'CHST7	-	X:46573783-46598408	-	-	IGV_LINK	76.6079	2.03984	0	4.1043	OK	72.1857	1.92314	0	3.85103	OK	OK	-0.085
ENSG0000014	-	-	ENSG0000014712	'KRBOX4	-	X:46446856-46497422	-	-	IGV_LINK	224.005	9.80169	1.09241	18.511	OK	220.682	8.50302	1.4282	15.5778	OK	OK	-0.20506
ENSG0000014	-	-	ENSG0000014712	'NDUFB11	-	X:47142215-47186813	-	-	IGV_LINK	883.415	50.6914	6.49857	94.8843	OK	959.039	55.1621	5.57048	104.754	OK	OK	0.121934
ENSG0000014	-	-	ENSG0000014712	'ZNF41	-	X:47445878-47482946	-	-	IGV_LINK	292.856	4.40933	0.579477	8.23918	OK	309.367	4.58791	0.5991	8.57672	OK	OK	0.057278
ENSG0000014	-	-	ENSG0000014712	'RAB41	-	X:70282092-70285002	-	-	IGV_LINK	3.87888	0.318631	0	0.921242	OK	1.03122	0.064658	0	0.327869	OK	NOTEST	-2.30099
ENSG0000014	-	-	ENSG0000014713	'ZMYM3	-	X:71239623-71255146	-	-	IGV_LINK	1185.97	12.7503	4.69019	20.8103	OK	953.883	11.9439	1.54443	22.3433	OK	OK	-0.09426
ENSG0000014	-	-	ENSG0000014713	'TAF1	-	X:71366238-71532374	-	-	IGV_LINK	1108.39	15.6115	0	33.8128	OK	912.634	14.0625	0	29.4156	OK	OK	-0.15076
ENSG0000014	-	-	ENSG0000014713	'GPR174	-	X:79170971-79172229	-	-	IGV_LINK	134.791	6.99806	1.30412	12.692	OK	36.0929	1.87585	0	3.81772	OK	OK	-1.89941
ENSG0000014	-	-	ENSG0000014714	'NONO	-	X:71283191-71301168	-	-	IGV_LINK	4817.57	177.813	10.6505	344.975	OK	4246.58	157.749	15.5629	299.936	OK	OK	-0.17273
ENSG0000014	-	-	ENSG0000014714	'CCDC120	-	X:49053571-49069857	-	-	IGV_LINK	230.793	4.58108	0.338347	8.82381	OK	237.182	4.29673	0.812095	7.78136	OK	OK	-0.09245
ENSG0000014	-	-	ENSG0000014714	'LPAR4	-	X:78747708-78757094	-	-	IGV_LINK	0	0	0	0	OK	4.1249	0.17416	0	0.601927	OK	NOTEST	inf
ENSG0000014	-	-	ENSG0000014715	'EBP	-	X:48521157-48528716	-	-	IGV_LINK	250.188	21.1604	5.73619	36.5845	OK	290.805	24.7556	6.44767	43.0636	OK	OK	0.226392
ENSG0000014	-	-	ENSG0000014716	'AWAT2	-	X:70040541-70049938	-	-	IGV_LINK	0	0	0	0	OK	1.03122	0.029798	0	0.268987	OK	NOTEST	inf
ENSG0000014	-	-	ENSG0000014716	'OGT	-	X:71533082-71575897	-	-	IGV_LINK	10692.1	134.096	0	311.064	OK	10388.6	127.969	0	275.215	OK	OK	-0.06748
ENSG0000014	-	-	ENSG0000014716	'SNX12	-	X:71056331-71073426	-	-	IGV_LINK	684.623	17.7047	7.17323	28.2362	OK	683.702	20.1786	6.19569	34.1614	OK	OK	0.188688
ENSG0000014	-	-	ENSG0000014716	'ITGB1BP2	-	X:71301733-71305371	-	-	IGV_LINK	11.6366	0.64306	0	1.57168	OK	12.3747	0.680524	0	1.60745	OK	OK	0.081692
ENSG0000014	-	-	ENSG0000014716	'IL2RG	-	X:71107403-71112108	-	-	IGV_LINK	1478.82	60.4136	25.7586	95.0687	OK	998.226	42.0812	16.8023	67.3601	OK	OK	-0.5217
ENSG0000014	-	-	ENSG0000014717	'GCNA	-	X:71578410-71613583	-	-	IGV_LINK	84.3657	2.27671	0	4.94228	OK	69.0921	1.4207	0.009357	2.83204	OK	OK	-0.68035
ENSG0000014	-	-	ENSG0000014718	'ZNF711	-	X:85244031-85273362	-	-	IGV_LINK	510.073	8.57448	2.75031	14.3986	OK	29.9055	0.544469	0	1.19647	OK	OK	-3.97713
ENSG0000014	-	-	ENSG0000014718	'CPXCR1	-	X:88747224-88754785	-	-	IGV_LINK	0	0	0	0	OK	0	0	0	0	OK	NOTEST	0
ENSG0000014	-	-	ENSG0000014720	'DIAPH2	-	X:96684662-97642589	-	-	IGV_LINK	554.68	4.96648	1.00078	8.93219	OK	458.895	3.62675	0.781528	6.47197	OK	OK	-0.45355
ENSG0000014	-	-	ENSG0000014720	'NXF3	-	X:103075809-103093125	-	-	IGV_LINK	1.93944	0.151649	0	0.728036	OK	13.4059	0.507804	0	1.42999	OK	NOTEST	1.74353
ENSG0000014	-	-	ENSG0000014722	'RIPPLY1	-	X:106802679-107000244	-	-	IGV_LINK	3.87888	0.264885	0	6.85154	OK	8.2498	0.759275	0	10.5882	OK	OK	1.51925
ENSG0000014	-	-	ENSG0000014722	'PRPS1	-	X:107628423-107651026	-	-	IGV_LINK	633.227	21.7333	7.74432	35.7223	OK	785.793	25.9313	9.47062	42.392	OK	OK	0.254789
ENSG0000014	-	-	ENSG0000014723	'CXorf57	-	X:106611929-106679442	-	-	IGV_LINK	427.647	11.3839	0	23.6278	OK	411.459	10.6047	0.015565	21.1938	OK	OK	-0.10229
ENSG0000014	-	-	ENSG0000014723	'FRMPD3	-	X:107512982-107605251	-	-	IGV_LINK	23.2733	0.207976	0	0.473186	OK	51.5612	0.408442	0.001955	0.81493	OK	NOTEST	0.973713
ENSG0000014	-	-	ENSG0000014724	'HTR2C	-	X:114584077-114910061	-	-	IGV_LINK	0	0	0	0	OK	0	0	0	0	OK	NOTEST	0
ENSG0000014	-	-	ENSG0000014725	'DOCK11	-	X:118495897-118686163	-	-	IGV_LINK	716.623	6.9973	1.19821	12.7964	OK	544.487	5.02476	1.80952	8.24	OK	OK	-0.47774
ENSG0000014	-	-	ENSG0000014725	'IGSF1	-	X:131273505-131578899	-	-	IGV_LINK	28.1219	0.550367	0	1.49511	OK	42.2802	0.617183	0	1.49098	OK	OK	0.165303
ENSG0000014	-	-	ENSG0000014725	'ARHGAP36	-	X:131058241-131089883	-	-	IGV_LINK	0	0	0	0	OK	0	0	0	0	OK	NOTEST	0
ENSG0000014	-	-	ENSG0000014725	'GPC3	-	X:133535744-133985895	-	-	IGV_LINK	9.69721	0.503665	0	1.23745	OK	25.7806	0.840699	0	1.85093	OK	OK	0.739125
ENSG0000014	-	-	ENSG0000014726	'GPR119	-	X:130384439-130385447	-	-	IGV_LINK	0	0	0	0	OK	0	0	0	0	OK	NOTEST	0
ENSG0000014	-	-	ENSG0000014727	'RBMX	-	X:136840930-136880764	-	-	IGV_LINK	4348.23	141.488	45.081	237.896	OK	3041.08	99.1824	33.4053	164.96	OK	OK	-0.51253
ENSG0000014	-	-	ENSG0000014731	'MCPH1	-	8:6403550-6708209	-	-	IGV_LINK	360.736	8.13587	0	23.1168	OK	333.086	8.82211	0	23.4476	OK	OK	0.116827
ENSG0000014	-	-	ENSG0000014732	'MFHAS1	-	8:8783353-8893645	-	-	IGV_LINK	466.436	4.88265	0	10.3218	OK	436.208	4.44253	0.053392	8.83166	OK	OK	-0.13628
ENSG0000014	-	-	ENSG0000014736	'FBXO25	-	8:406427-477967	-	-	IGV_LINK	651.652	12.405	1.91126	22.8987	OK	1461.25	33.8927	3.86629	63.919	OK	OK	1.45005
ENSG0000014	-	-	ENSG0000014737	'FATE1	-	X:151716034-151723194	-	-	IGV_LINK	28.1219	1.76194	0	3.6818	OK	21.6557	1.46596	0	3.32223	OK	OK	-0.26532
ENSG0000014	-	-	ENSG0000014738	'MAGEA4	-	X:151904430-151925170	-	-	IGV_LINK	0	0	0	0	OK	0	0	0	0	OK	NOTEST	0
ENSG0000014	-	-	ENSG0000014738	'NSDHL	-	X:152830966-152869729	-	-	IGV_LINK	320.977	11.4063	3.68321	19.1293	OK	315.555	11.0614	3.58393	18.5388	OK	OK	-0.04429
ENSG0000014	-	-	ENSG0000014739	'ZNF185	-	X:152914441-152973480	-	-	IGV_LINK	444.132	6.5459	2.35049	10.7413	OK	259.869	4.2783	1.23835	7.31825	OK	OK	-0.61355
ENSG0000014	-	-	ENSG0000014740	'CETN2	-	X:152826972-152830777	-	-	IGV_LINK	961.963	42.6067	17.4046	67.8089	OK	951.821	41.8958	18.0365	65.7552	OK	OK	-0.02427
ENSG0000014	-	-	ENSG0000014740	'RPL10	-	X:154389954-154421726	-	-	IGV_LINK	29468.8	3280.44	0	6640.12	OK	20768.9	2322.89	335.28	4310.49	OK	OK	-0.49797
ENSG0000014	-	-	ENSG0000014740	'CSGALNACT1	-	8:19404160-19758029	-	-	IGV_LINK	221.096	4.36835	0	9.30036	OK	681.64	13.9553	2.09724	25.8135	OK	OK	1.67566
ENSG0000014	-	-	ENSG0000014741	'ATP6V1B2	-	8:20197366-20226819	-	-	IGV_LINK	3517.18	77.676	25.459	129.893	OK	3579.38	79.2724	26.2056	132.339	OK	OK	0.02935
ENSG0000014	-	-	ENSG0000014741	'CCDC25	-	8:27733310-27838095	-	-	IGV_LINK	710.805	19.2533	4.00776	34.4989	OK	738.357	20.4495	2.49888	38.4001	OK	OK	0.086957
ENSG0000014	-	-	ENSG0000014742	'HMBOX1	-	8:28890393-29064764	-	-	IGV_LINK	312.25	16.6475	3.0046	30.2904	OK	213.464	13.9361	2.59198	25.2803	OK	OK	-0.25648
ENSG0000014	-	-	ENSG0000014743	'CHRNB3	-	8:42697375-42737407	-	-	IGV_LINK	0	0	0	0	OK	0	0	0	0	OK	NOTEST	0
ENSG0000014	-	-	ENSG0000014743	'CHRNA6	-	8:42752619-42796392	-	-	IGV_LINK	106.669	2.975	0	5.95642	OK	36.0929	0.913749	0	1.99049	OK	OK	-1.70302
ENSG0000014	-	-	ENSG0000014743	'GNRH1	-	8:25419259-25424654	-	-	IGV_LINK	146.428	6.41296	0	12.9124	OK	95.9039	4.9576	0	10.8945	OK	OK	-0.37135

ENSG0000014	-	-	ENSG0000014743	'BIN3	-	8:22604631-22669148	-	-	IGV_LINK	763.17	28.9919	0	67.0679	OK	772.388	31.6987	0	74.8701	OK	OK	0.128772
ENSG0000014	-	-	ENSG0000014744	'DOK2	-	8:21908872-21913860	-	-	IGV_LINK	357.827	12.7006	3.90257	21.4987	OK	345.46	13.1023	3.06087	23.1438	OK	OK	0.044923
ENSG0000014	-	-	ENSG0000014745	'SLC25A37	-	8:23528804-23575463	-	-	IGV_LINK	2271.09	35.606	10.4285	60.7834	OK	4033.12	65.3242	17.3921	113.256	OK	OK	0.875497
ENSG0000014	-	-	ENSG0000014745	'CHMP7	-	8:23243636-23262000	-	-	IGV_LINK	969.721	23.4897	6.92631	40.0531	OK	1019.88	26.9133	7.44747	46.3792	OK	OK	0.196291
ENSG0000014	-	-	ENSG0000014745	'DOCK5	-	8:25184722-25418082	-	-	IGV_LINK	808.747	6.58397	0	13.5922	OK	2168.67	18.674	1.01123	36.3368	OK	OK	1.504
ENSG0000014	-	-	ENSG0000014746	'STAR	-	8:38105241-38213301	-	-	IGV_LINK	7.75776	0.248903	0	2.16588	OK	22.6869	0.78626	0	4.93489	OK	OK	1.65942
ENSG0000014	-	-	ENSG0000014747	'PLPBP	-	8:37762592-37779767	-	-	IGV_LINK	1068.63	42.0737	0	94.3885	OK	908.509	36.9933	0	82.7858	OK	OK	-0.18565
ENSG0000014	-	-	ENSG0000014747	'ERLIN2	-	8:37734760-37759101	-	-	IGV_LINK	1262.58	23.1419	3.03291	43.2509	OK	1430.31	24.9804	2.89056	47.0702	OK	OK	0.110289
ENSG0000014	-	-	ENSG0000014748	'SNTG1	-	8:49909788-50794118	-	-	IGV_LINK	0	0	0	0	OK	1.03122	0.037919	0	0.533784	OK	NOTEST	inf
ENSG0000014	-	-	ENSG0000014748	'PXDNL	-	8:51257687-51809445	-	-	IGV_LINK	8.72748	0.12449	0	0.364635	OK	42.2802	0.567736	0	1.20424	OK	OK	2.18919
ENSG0000014	-	-	ENSG0000014748	'ST18	-	8:52110838-52460959	-	-	IGV_LINK	20.3641	0.456634	0	1.69363	OK	31.968	0.778038	0	2.6055	OK	OK	0.768803
ENSG0000014	-	-	ENSG0000014750	'RGS20	-	8:53851807-53959303	-	-	IGV_LINK	2.90916	0.321122	0	1.07855	OK	2.06245	0.227029	0	0.808199	OK	NOTEST	-0.50025
ENSG0000014	-	-	ENSG0000014752	'TACC1	-	8:38728185-38853028	-	-	IGV_LINK	2258.48	26.463	0	52.9611	OK	1636.55	18.1388	1.78459	34.493	OK	OK	-0.5449
ENSG0000014	-	-	ENSG0000014753	'GOLGA7	-	8:41490395-41510980	-	-	IGV_LINK	871.779	41.2532	11.1369	71.3695	OK	864.167	43.3866	12.4376	74.3356	OK	OK	0.072744
ENSG0000014	-	-	ENSG0000014753	'PLPP5	-	8:38225217-38382272	-	-	IGV_LINK	632.258	28.3641	0	73.1063	OK	872.416	40.7811	0	93.3488	OK	OK	0.523834
ENSG0000014	-	-	ENSG0000014753	'GINS4	-	8:41529205-41625001	-	-	IGV_LINK	68.8502	3.65548	0	20.6692	OK	78.3731	3.60281	0	22.8609	OK	OK	-0.02094
ENSG0000014	-	-	ENSG0000014754	'NSD3	-	8:38225217-38382272	-	-	IGV_LINK	1428.4	20.2631	0	45.994	OK	1110.63	14.1942	0	34.3692	OK	OK	-0.51356
ENSG0000014	-	-	ENSG0000014757	'DNAJC5B	-	8:66021559-66100526	-	-	IGV_LINK	36.8494	3.37839	0	7.87818	OK	21.6557	1.68436	0	4.10949	OK	OK	-1.00413
ENSG0000014	-	-	ENSG0000014757	'CRH	-	8:66176381-66178725	-	-	IGV_LINK	0	0	0	0	OK	0	0	0	0	OK	NOTEST	0
ENSG0000014	-	-	ENSG0000014757	'TRIM55	-	8:66126895-66175487	-	-	IGV_LINK	565.347	14.6045	4.84404	24.3651	OK	1041.54	24.5859	10.5516	38.6201	OK	OK	0.751411
ENSG0000014	-	-	ENSG0000014757	'ADHFE1	-	8:66429027-66518524	-	-	IGV_LINK	254.185	12.6539	0	28.6722	OK	241.193	11.1895	0	25.5857	OK	OK	-0.17744
ENSG0000014	-	-	ENSG0000014758	'MRPS28	-	8:79918716-80231232	-	-	IGV_LINK	428.309	70.9495	0	144.91	OK	455.169	72.726	0	155.174	OK	OK	0.035677
ENSG0000014	-	-	ENSG0000014758	'PMP2	-	8:81439435-81533275	-	-	IGV_LINK	0	0	0	0	OK	0	0	0	0	OK	NOTEST	0
ENSG0000014	-	-	ENSG0000014759	'LACTB2	-	8:70608576-70669174	-	-	IGV_LINK	405.343	25.7753	9.03561	42.515	OK	832.199	52.8458	21.8422	83.8494	OK	OK	1.0358
ENSG0000014	-	-	ENSG0000014759	'PRDM14	-	8:70051650-70071693	-	-	IGV_LINK	0	0	0	0	OK	1.03122	0.026777	0	0.303995	OK	NOTEST	inf
ENSG0000014	-	-	ENSG0000014760	'TERF1	-	8:73008863-73048122	-	-	IGV_LINK	586.681	15.7674	4.54636	26.9884	OK	711.545	20.4654	7.01907	33.9118	OK	OK	0.376248
ENSG0000014	-	-	ENSG0000014760	'RPL7	-	8:73290266-73356461	-	-	IGV_LINK	22553.8	1729.65	84.6901	3374.62	OK	19411.8	1490.75	275.698	2705.8	OK	OK	-0.21444
ENSG0000014	-	-	ENSG0000014760	'SLC26A7	-	8:91101831-91398152	-	-	IGV_LINK	26.1825	0.415463	0	1.29282	OK	38.1553	0.550435	0	1.49412	OK	NOTEST	0.405851
ENSG0000014	-	-	ENSG0000014761	'PSKH2	-	8:85987322-86154228	-	-	IGV_LINK	0	0	0	0	OK	0	0	0	0	OK	NOTEST	0
ENSG0000014	-	-	ENSG0000014761	'ATP6V0D2	-	8:85987322-86154228	-	-	IGV_LINK	0.969721	0.024038	0	0.366027	OK	11.3435	0.281327	0	0.780071	OK	NOTEST	3.54888
ENSG0000014	-	-	ENSG0000014764	'SYBU	-	8:109573977-109691791	-	-	IGV_LINK	85.3354	1.99692	0	4.18079	OK	1708.74	54.6167	11.3407	97.8927	OK	OK	4.7735
ENSG0000014	-	-	ENSG0000014764	'DPYS	-	8:104330323-104467053	-	-	IGV_LINK	1156.88	53.7115	8.76256	98.6605	OK	2459.47	105.043	31.2415	178.845	OK	OK	0.967678
ENSG0000014	-	-	ENSG0000014764	'MTDH	-	8:97644178-97728770	-	-	IGV_LINK	2070.35	47.7917	3.83097	91.7525	OK	2651.28	62.9672	4.466	121.468	OK	OK	0.397838
ENSG0000014	-	-	ENSG0000014765	'LRP12	-	8:104489230-104589024	-	-	IGV_LINK	133.821	3.41639	0	7.30533	OK	167.058	2.689	0.216398	5.1616	OK	OK	-0.3454
ENSG0000014	-	-	ENSG0000014765	'EBAG9	-	8:109539710-109565996	-	-	IGV_LINK	696.259	42.4816	12.9408	72.0223	OK	658.953	41.1619	14.5477	67.7761	OK	OK	-0.04553
ENSG0000014	-	-	ENSG0000014765	'RSPO2	-	8:107899315-108083648	-	-	IGV_LINK	0	0	0	0	OK	0	0	0	0	OK	NOTEST	0
ENSG0000014	-	-	ENSG0000014766	'POLR2K	-	8:100150583-100154002	-	-	IGV_LINK	707.896	48.3178	19.9501	76.6856	OK	739.388	50.8076	20.868	80.7472	OK	OK	0.07249
ENSG0000014	-	-	ENSG0000014767	'MAL2	-	8:119165033-119246848	-	-	IGV_LINK	296.734	8.89287	0.123281	17.6625	OK	85.5917	3.03211	0	7.07263	OK	OK	-1.55233
ENSG0000014	-	-	ENSG0000014767	'EIF3H	-	8:116642129-116876868	-	-	IGV_LINK	4790.42	300.98	30.0276	571.932	OK	4563.17	296.047	28.9502	563.144	OK	OK	-0.02384
ENSG0000014	-	-	ENSG0000014767	'UTP23	-	8:116642129-116876868	-	-	IGV_LINK	250.188	6.2119	0	37.8626	OK	260.9	7.53853	0	50.2462	OK	OK	0.279249
ENSG0000014	-	-	ENSG0000014768	'NDUFB9	-	8:124488484-124728429	-	-	IGV_LINK	1657.25	173.855	25.0615	322.648	OK	1480.84	155.783	38.1362	273.429	OK	OK	-0.15835
ENSG0000014	-	-	ENSG0000014768	'TATDN1	-	8:124488484-124728429	-	-	IGV_LINK	414.071	29.116	0	79.0854	OK	248.525	17.985	0	52.3049	OK	OK	-0.69502
ENSG0000014	-	-	ENSG0000014768	'FAM83A	-	8:123178959-123210079	-	-	IGV_LINK	2.90916	0.083246	0	0.51679	OK	1.03122	0.040629	0	0.459954	OK	NOTEST	-1.03488
ENSG0000014	-	-	ENSG0000014769	'GSDMC	-	8:129748195-129786888	-	-	IGV_LINK	2.90916	0.109241	0	0.565436	OK	3.09367	0.105196	0	0.546436	OK	NOTEST	-0.05444
ENSG0000014	-	-	ENSG0000014772	'FAM135B	-	8:138130022-138496822	-	-	IGV_LINK	68.8502	0.695967	0	2.04679	OK	150.559	1.43291	0	3.42898	OK	OK	1.04186
ENSG0000014	-	-	ENSG0000014775	'TTTY7	-	Y:6449467-6457906	-	-	IGV_LINK	0	0	0	0	OK	0	0	0	0	OK	NOTEST	0
ENSG0000014	-	-	ENSG0000014776	'TTTY7B	-	Y:9706823-9715262	-	-	IGV_LINK	0	0	0	0	OK	0	0	0	0	OK	NOTEST	0
ENSG0000014	-	-	ENSG0000014778	'ZNF7	-	8:144827463-144853736	-	-	IGV_LINK	624.5	22.6004	2.35358	42.8472	OK	547.58	20.084	2.37589	37.7921	OK	OK	-0.1703
ENSG0000014	-	-	ENSG0000014779	'ARHGAP39	-	8:144529178-144605816	-	-	IGV_LINK	76.6079	0.958908	0	1.94011	OK	62.9047	0.7879	0	1.65945	OK	OK	-0.28338
ENSG0000014	-	-	ENSG0000014780	'SLC39A4	-	8:144409491-144416895	-	-	IGV_LINK	125.094	12.8576	1.07575	24.6394	OK	144.371	15.1799	2.1511	28.2086	OK	OK	0.239542
ENSG0000014	-	-	ENSG0000014781	'NAPRT	-	8:143573489-143603224	-	-	IGV_LINK	920.265	48.3372	0	185.821	OK	1146.72	59.7322	0	184.593	OK	OK	0.305374
ENSG0000014	-	-	ENSG0000014785	'VLDLR	-	9:2422701-2660053	-	-	IGV_LINK	763.17	11.731	2.08668	21.3753	OK	1622.12	25.0728	8.45307	41.6926	OK	OK	1.0958
ENSG0000014	-	-	ENSG0000014785	'AK3	-	9:4709558-4742043	-	-	IGV_LINK	998.812	28.4194	7.44517	49.3936	OK	1895.39	49.197	14.5549	83.8392	OK	OK	0.791694
ENSG0000014	-	-	ENSG0000014785	'UHRF2	-	9:6413150-6507054	-	-	IGV_LINK	761.231	15.3805	0	33.2148	OK	662.046	13.8375	0	30.5963	OK	OK	-0.15251
ENSG0000014	-	-	ENSG0000014786	'NFIB	-	9:14081842-14398983	-	-	IGV_LINK	1142.33	9.30648	2.6711	15.9419	OK	1061.13	8.36711	2.14646	14.5878	OK	OK	-0.15351
ENSG0000014	-	-	ENSG0000014786	'CER1	-	9:14719723-14722717	-	-	IGV_LINK	0	0	0	0	OK	0	0	0	0	OK	NOTEST	0
ENSG0000014	-	-	ENSG0000014787	'PLIN2	-	9:19108374-19149290	-	-	IGV_LINK	28815.2	942.539	99.1437	1785.93	OK	80361.3	2617	0	6214.92	OK	OK	1.47329
ENSG0000014	-	-	ENSG0000014787	'IFNA5	-	9:21304325-21305312	-	-	IGV_LINK	1.93944	0.134195	0	0.410965	OK	1.03122	0.071454	0	0.267437	OK	NOTEST	-0.90924
ENSG0000014	-	-	ENSG0000014787	'HAUS6	-	9:19053142-19103119	-	-	IGV_LINK	412.131	5.63856	0	11.8478	OK	405.271	4.73647	0.004548	9.4684	OK	OK	-0.25151
ENSG0000014	-	-	ENSG0000014788	'CDKN2B	-	9:21802542-22121097	-	-	IGV_LINK	106.669	3.35877	0	10.5349	OK	170.152	5.72138	0	15.87	OK	OK	0.76843
ENSG0000014	-	-	ENSG0000014788	'IFNA16	-	9:21216372-21217311	-	-	IGV_LINK	0	0	0	0	OK	0	0	0	0	OK	NOTEST	0
ENSG0000014	-	-	ENSG0000014788	'CDKN2A	-	9:21802542-22121097	-	-	IGV_LINK	183.277	14.1723	0	34.1621	OK	224.807	19.1308	0	44.777	OK	OK	0.432823
ENSG0000014	-	-	ENSG0000014789	'C9orf72	-	9:27546544-27573866	-	-	IGV_LINK	560.498	10.6957	3.625	17.7663	OK	401.147	8.79253	1.94942	15.6356	OK	OK	-0.28267
ENSG0000014	-	-	ENSG0000014789	'IFNK	-	9:27325208-27529781	-	-	IGV_LINK	0	0	0	0	OK	0	0	0	0	OK	NOTEST	0
ENSG0000014	-	-	ENSG0000014790	'ZCCHC7	-	9:37120538-37358149	-	-	IGV_LINK	313.22	10.2299	2.10885	18.3509	OK	320.711	9.6947	1.93308	17.4563	OK	OK	-0.07752
ENSG0000014	-	-	ENSG0000014791	'FBXO10	-	9:37510891-38069211	-	-	IGV_LINK	128.099	1.86308	0	7.07591	OK	123.141	2.04555	0	9.55427	OK	OK	0.134799
ENSG0000014	-	-	ENSG0000014795	'SIGMAR1	-	9:34634721-34637809	-	-	IGV_LINK	526.558	22.7472	7.07108	38.4234	OK	636.266	25.554	9.51735	41.5906	OK	OK	0.167857
ENSG0000014	-	-	ENSG0000014799	'CBWD5	-	9:65668804-65734041	-	-	IGV_LINK	183.277	6.52413	0.530932	12.5173	OK	217.588	7.90498	1.01192	14.798	OK	OK	0.276976
ENSG0000014	-	-	ENSG0000014801	'CEP78	-	9:78236061-78279690	-	-	IGV_LINK	213.339	4.43026	0	9.26527	OK	189.745	3.33829	0	6.77792	OK	OK	-0.40828
ENSG0000014	-	-	ENSG0000014805	'NTRK2	-	9:84668550-85027070	-	-	IGV_LINK	141.579	1.20352	0.223345	2.1837	OK	52.5925	0.449566	0.002233	0.896899	OK	OK	-1.42066
ENSG0000014	-	-	ENSG0000014805	'IDNK	-	9:83623048-83644130	-	-	IGV_LINK	108.609	7.81788	0	15.7618	OK	127.872	9.52649	0	19.9627	OK	OK	0.285168
ENSG0000014	-	-	ENSG0000014808	'SHC3	-	9:89005770-89178767	-	-	IGV_LINK	24.243	0.142382	0	0.402564	OK	9.28102	0.054515	0	0.268331	OK	NOTEST	-1.38504
ENSG0000014	-	-	ENSG0000014809	'AUH	-	9:91213814-91361913	-	-	IGV_LINK	494.557	21.5272	7.55547	35.4989	OK	795.074	33.991	12.795	55.187	OK	OK	0.658993
ENSG0000014	-	-	ENSG0000014811	'MFSD14B	-	9:94374550-94461042	-	-	IGV_LINK	737.957	12.9384	5.34402	20.5327	OK	645.547	11.2065	4.54962	17.8634	OK	OK	-0.20732
ENSG0000014	-	-	ENSG0000014812	'C9orf3	-	9:94726700-95087218	-	-	IGV_LINK	480.012	25.3171	6.79228	43.8419	OK	570.267	28.648	6.26945	51.0265	OK	OK	0.178323
ENSG0000014	-	-	ENSG0000014812	'PLPPR1	-	9:101028708-101325135	-	-	IGV_LINK	0	0	0	0	OK	0	0	0	0	OK	NOTEST	0
ENSG0000014	-	-	ENSG0000014813	'OR13C4	-	9:104520828-104537180	-	-	IGV_LINK	0	0	0	0	OK	0	0	0	0	OK	NOTEST	0
ENSG0000014	-	-	ENSG0000014814	'ZNF462	-	9:106863096-107102988	-	-	IGV_LINK	531.407	5.36152	0	11.0474	OK	385.678	4.0531	0	8.41265	OK	OK	-0.40362
ENSG0000014	-	-	ENSG0000014815	'INIP	-	9:112683925-112718236	-	-	IGV_LINK	351.039	7.30666	0.611525	14.0018	OK	406.303	8.09067	0	17.0921	OK	OK	0.147047
ENSG0000014	-	-	ENSG0000014815	'UGCG	-	9:111896765-111935369	-	-	IGV_LINK	2137.26	33.034	10.6037	55.4642	OK	2630.65	40.9249	13.4606	68.3892	OK	OK	0.309029
ENSG0000014	-	-	ENSG0000014815	'ACTL7B	-	9:108854588-108856967	-	-	IGV_LINK	0	0	0	0	OK	1.03122	0.026352	0	0.098631	OK	NOTEST	inf
ENSG0000014	-	-	ENSG0000014815	'SNX30	-	9:112750837-112890913	-	-	IGV_LINK	1279.06	10.3021	4.27074	16.3335	OK	1512.81	12.4847	5.00816	19.9613	OK	OK	0.277224
ENSG0000014	-	-	ENSG0000014817	'STOM	-	9:121338987-121500027	-	-	IGV_LINK	4195.98	120.657	29.3344	211.979	OK	6094.54	179.449	40.0703	318.827	OK	OK	0.572661
ENSG0000014	-	-	ENSG0000014818	'GSN	-	9:121178136-121332843	-	-	IGV_LINK	7193.39	197.603	41.1261	354.081	OK	5092.19	141.19	36.0023	246.378	OK	OK	-0.48497
ENSG0000014	-	-	ENSG0000014818	'MRRF	-	9:122237623-122331343	-	-	IGV_LINK	947.417	33.3027	5.71751	60.888	OK	866.229	31.0409	4.30332	57.7785	OK	OK	-0.10147
ENSG0000014	-	-	ENSG0000014820	'NR6A1	-	9:124517274-124772927	-	-	IGV_LINK	235.642	2.03293	0.471673	3.59418	OK	207.276	1.70347	0.431755	2.97518	OK	OK	-0.25509
ENSG0000014	-	-	ENSG0000014820	'CRB2	-	9:123356169-123930152	-	-	IGV_LINK	178.429	1.88719	0	4.38237	OK	16.4996	0.172954	0	0.842538	OK	OK	-3.44778
ENSG0000014	-	-	ENSG0000014821	'OR5C1	-	9:122788932-122789895	-	-	IGV_LINK	0	0	0	0	OK	1.03122	0.073627	0	0.275568	OK	NOTEST	inf
ENSG0000014	-	-	ENSG0000014821	'ALAD	-	9:113386316-113401333	-	-	IGV_LINK	1009.48	26.0614	5.71456	46.4082	OK	1069.38	26.4774	5.45044	47.5044	OK	OK	0.022851
ENSG0000014	-	-	ENSG0000014821	'ASTN2	-	9:116425224-117415070	-	-	IGV_LINK	274.431	5.57059	0.206893	10.9343	OK	380.522	8.57052	1.00141	16.1396	OK	OK	0.621553
ENSG0000014	-	-	ENSG0000014822	'WDR31	-	9:113313221-113340298	-	-	IGV_LINK	221.096	4.00559	0	8.2324	OK	196.964	2.92111	0.518135	5.32408	OK	OK	-0.4555
ENSG0000014	-	-	ENSG0000014822	'POLE3	-	9:113407234-113429684	-	-	IGV_LINK	845.596	25.5724	9.0453	42.0994	OK	1003.38	29.1655	12.1201	46.2109	OK	OK	0.189677
ENSG0000014	-	-	ENSG0000014824	'SURF4	-	9:133361448-133376166	-	-	IGV_LINK	2285.63	46.6341	19.6366	73.6316	OK	3582.48	73.3961	29.3088	117.483	OK	OK	0.654317
ENSG0000014	-	-	ENSG0000014828	'GBGT1	-	9:133152947-133163945	-	-	IGV_LINK	456.738	16.3083	4.71359	27.903	OK	315.555	9.99591	2.29738	17.6944	OK	OK	-0.70619
ENSG0000014	-	-	ENSG0000014829	'SURF1	-	9:133351754-133361169	-	-	IGV_LINK	663.289	43.9493	14.9852	72.9135	OK	763.106	49.8162	17.4284	82.2039	OK	OK	0.180773
ENSG0000014	-	-	ENSG0000014829	'SURF2	-	9:133351754-133361169	-	-	IGV_LINK	147.398	12.9005	0	31.301	OK	95.9039	8.60027	0	24.5383	OK	OK	-0.58497
ENSG0000014	-	-	ENSG0000014829	'SURF6	-	9:133328773-133336398	-	-	IGV_LINK	639.046	9.60657	2.6078	16.6053	OK	674.421	11.3622	2.4896	20.2348	OK	OK	0.242149
ENSG0000014	-	-	ENSG0000014829	'MED22	-	9:133338322-133348131	-	-	IGV_LINK	815.535	12.9279	4.45608	21.3998	OK	859.01	14.8162	4.19456	25.4379	OK	OK	0.196689
ENSG0000014	-	-	ENSG0000014830	'REXO4	-	9:133376366-133459402	-	-	IGV_LINK	612.863	16.2603	5.20549	27.3151	OK	665.14	18.4014	5.48642	31.3163	OK	OK	0.178461
ENSG0000014	-	-	ENSG0000014830	'RPL7A	-	9:133348213-133351426	-	-	IGV_LINK	20228.4	1599.36	330.866	2867.86	OK	15931.4	1261.4	340.216	2182.59	OK	OK	-0.34247
ENSG0000014	-	-	ENSG0000014830	'GTF3C5	-	9:133030674-133058503	-	-	IGV_LINK	866.93	33.7032	9.21063	58.1958	OK	810.543	27.4965	7.76012	47.2328	OK	OK	-0.29364
ENSG0000014	-	-	ENSG0000014833	'ASB6	-	9:129608883-129642169	-	-	IGV_LINK	705.957	11.261	3.34361	19.1784	OK	637.297	11.0102	3.23828	18.782	OK	OK	-0.0325
ENSG0000014	-	-	ENSG0000014833	'PTGES2	-	9:128120692-128128462	-	-	IGV_LINK	655.531	30.4277	8.3117	52.5438	OK	686.796	31.3461	10.1287	52.5635	OK	OK	0.0429
ENSG0000014	-	-	ENSG0000014833	'NTMT1	-	9:129608883-129642169	-	-	IGV_LINK	173.58	17.7507	0	41.9729	OK	185.62	18.0813	0	41.7814	OK	OK	0.026622
ENSG0000014	-	-	ENSG0000014833	'CIZ1	-	9:128166063-128255248	-	-	IGV_LINK	1981.14	66.6292	7.90355	125.355	OK	1837.64	60.5119	12.62	108.404	OK	OK	-0.13893
ENSG0000014	-	-	ENSG0000014833	'SLC25A25	-	9:128061232-128118693	-	-	IGV_LINK	185.217	3.6062	0	9.43978	OK	164.996	3.35769	0	8.58206	OK	OK	-0.10301
ENSG0000014	-	-	ENSG0000014834	'SH3GLB2	-	9:128920894-129028303	-	-	IGV_LINK	1888.05	71.1052	2.34357	139.867	OK	1460.21	55.0705	0.984664	109.156	OK	OK	-0.36867
ENSG0000014	-	-	ENSG0000014834	'MIGA2	-	9:129036620-129072082	-	-	IGV_LINK	512.982	12.8212	0	25.8969	OK	466.114	11.9395	0.064579	23.8143	OK	OK	-0.1028
ENSG0000014	-	-	ENSG0000014834	'PTGES	-	9:129738330-129753047	-	-	IGV_LINK	65.941	2.30761	0.107238	4.50798	OK	872.416	30.6261	13.0218	48.2305	OK	OK	3.73029
ENSG0000014	-	-	ENSG0000014834	'LCN2	-	9:128149070-128153455	-	-	IGV_LINK	0	0	0	0	OK	7.21857	0.627474	0	1.73679	OK	OK	inf
ENSG0000014	-	-	ENSG0000014835	'LRSAM1	-	9:127451485-127503501	-	-	IGV_LINK	659.41	14.4901	4.68891	24.2913	OK	500.144	10.3278	2.84944	17.8063	OK	OK	-0.48853
ENSG0000014	-	-	ENSG0000014835	'HMCN2	-	9:130265881-130434123	-	-	IGV_LINK	11.6366	0.148561	0	0.419339	OK	70.1233	0.567622	0	1.5001	OK	OK	1.93388
ENSG0000014	-	-	ENSG0000014835	'GPR107	-	9:130053425-130140169	-	-	IGV_LINK	2398.12	20.473	6.3022	34.6437	OK	3650.54	30.2739	12.7007	47.8472	OK	OK	0.564356
ENSG0000014	-	-	ENSG0000014836	'PAXX	-	9:136992417-136993984	-	-	IGV_LINK	593.469	47.9634	18.1978	77.729	OK	413.521	33.4606	10.7809	56.1403	OK	OK	-0.51947
ENSG0000014	-	-	ENSG0000014837	'IDI2	-	10:971145-1049170	-	-	IGV_LINK	25.2127	1.19756	0	5.23463	OK	25.7806	1.22572	0	4.98984	OK	OK	0.033534
ENSG0000014	-	-	ENSG0000014838	'INPP5E	-	9:136428618-136439823	-	-	IGV_LINK	466.436	7.91524	2.96184	12.8686	OK	419.709	7.14687	2.61049	11.6833	OK	OK	-0.14732
ENSG0000014	-	-	ENSG0000014838	'LCN9	-	9:135663321-135666422	-	-	IGV_LINK	0	0	0	0	OK	0	0	0	0	OK	NOTEST	0
ENSG0000014	-	-	ENSG0000014839	'SEC16A	-	9:136440095-136486067	-	-	IGV_LINK	1466.22	14.5473	3.50253	25.5922	OK	2167.63	19.2902	5.55318	33.0272	OK	OK	0.407112
ENSG0000014	-	-	ENSG0000014839	'DPH7	-	9:137554903-137578935	-	-	IGV_LINK	534.316	25.746	8.7918	42.7002	OK	402.178	19.8844	4.96973	34.7992	OK	OK	-0.37271
ENSG0000014	-	-	ENSG0000014840	'NOTCH1	-	9:136494443-136545862	-	-	IGV_LINK	1338.21	9.23772	0.600893	17.8746	OK	1407.62	9.03595	2.29187	15.78	OK	OK	-0.03186
ENSG0000014	-	-	ENSG0000014840	'CACNA1B	-	9:137605653-138124624	-	-	IGV_LINK	0	0	0	0	OK	1.03122	0.008321	0	0.314416	OK	NOTEST	inf
ENSG0000014	-	-	ENSG0000014841	'NACC2	-	9:136006536-136095285	-	-	IGV_LINK	366.554	3.10749	0.934018	5.28096	OK	616.673	5.2163	2.04368	8.38892	OK	OK	0.747276
ENSG0000014	-	-	ENSG0000014842	'PROSER2	-	10:11823338-11894700	-	-	IGV_LINK	61.0924	2.37941	0	6.74448	OK	89.7166	3.38242	0	7.9816	OK	OK	0.507453
ENSG0000014	-	-	ENSG0000014842	'USP6NL	-	10:11453945-11612227	-	-	IGV_LINK	316.129	3.77212	0.899279	6.64495	OK	222.745	2.85018	0.511668	5.1887	OK	OK	-0.40432
ENSG0000014	-	-	ENSG0000014844	'COMMD3	-	10:22315973-22331484	-	-	IGV_LINK	529.613	36.2252	2.78734	69.663	OK	377.404	25.5324	0	54.5682	OK	OK	-0.50467
ENSG0000014	-	-	ENSG0000014845	'MSRB2	-	10:23095505-23122013	-	-	IGV_LINK	490.679	27.7665	2.48945	53.0436	OK	261.931	16.1603	0.161352	32.1593	OK	OK	-0.78089
ENSG0000014	-	-	ENSG0000014845	'PDSS1	-	10:26697658-26861087	-	-	IGV_LINK	60.1227	3.34815	0	12.3861	OK	42.2802	1.79238	0	6.55604	OK	OK	-0.90149
ENSG0000014	-	-	ENSG0000014846	'FAM171A1	-	10:15211642-15371062	-	-	IGV_LINK	650.682	9.92935	3.54636	16.3123	OK	188.714	3.06725	0.141083	5.99342	OK	OK	-1.69475
ENSG0000014	-	-	ENSG0000014848	'MINDY3	-	10:15778169-15860520	-	-	IGV_LINK	264.734	9.40998	0	18.8542	OK	196.964	7.82762	0	16.594	OK	OK	-0.26562
ENSG0000014	-	-	ENSG0000014848	'SLC39A12	-	10:17951838-18043292	-	-	IGV_LINK	0	0	0	0	OK	1.03122	0.025405	0	0.129348	OK	NOTEST	inf
ENSG0000014	-	-	ENSG0000014848	'TMEM236	-	10:17752251-17800868	-	-	IGV_LINK	3.87888	0.041311	0	0.105212	OK	10.3122	0.109852	0	0.246838	OK	NOTEST	1.41096
ENSG0000014	-	-	ENSG0000014848	'RSU1	-	10:16590610-16817528	-	-	IGV_LINK	1446.82	57.1809	18.1698	96.192	OK	869.323	34.8019	10.3914	59.2124	OK	OK	-0.71637

ENSG0000014	-	-	ENSG0000014848	'ST8SIA6	-	10:17318382-17454330	-	-	IGV_LINK	5.81832	0.344774	0	0.937326	OK	0	0	0	0	OK	NOTEST	'-inf
ENSG0000014	-	-	ENSG0000014849	'PARD3	-	10:34109559-34815325	-	-	IGV_LINK	1570.95	22.1394	4.31111	39.9677	OK	746.607	13.1556	2.11476	24.1965	OK	OK	-0.75094
ENSG0000014	-	-	ENSG0000014851	'ANKRD30A	-	10:37125787-37384111	-	-	IGV_LINK	0	0	0	0	OK	0	0	0	0	OK	NOTEST	0
ENSG0000014	-	-	ENSG0000014851	'ZEB1	-	10:31187882-31529814	-	-	IGV_LINK	569.226	8.78116	1.87751	15.6848	OK	513.55	9.04587	1.01416	17.0776	OK	OK	0.042848
ENSG0000014	-	-	ENSG0000014854	'FAM13C	-	10:59176589-59363181	-	-	IGV_LINK	90.184	2.19697	0	5.69339	OK	83.5292	2.55846	0	7.06116	OK	OK	0.219759
ENSG0000014	-	-	ENSG0000014857	'NRBF2	-	10:63133246-63155031	-	-	IGV_LINK	318.068	10.6733	3.58744	17.7591	OK	309.367	10.3819	3.40446	17.3593	OK	OK	-0.03993
ENSG0000014	-	-	ENSG0000014858	'A1CF	-	10:50739317-50885675	-	-	IGV_LINK	361.706	2.97193	0.291118	5.65274	OK	345.46	2.75026	0.33114	5.16937	OK	OK	-0.11183
ENSG0000014	-	-	ENSG0000014860	'CDHR1	-	10:84194634-84219621	-	-	IGV_LINK	431.526	6.74496	0	13.5885	OK	57.7486	1.38891	0	3.89624	OK	OK	-2.27985
ENSG0000014	-	-	ENSG0000014860	'LRIT1	-	10:84231592-84241461	-	-	IGV_LINK	0	0	0	0	OK	0	0	0	0	OK	NOTEST	0
ENSG0000014	-	-	ENSG0000014860	'RGR	-	10:84245052-84259960	-	-	IGV_LINK	0	0	0	0	OK	0	0	0	0	OK	NOTEST	0
ENSG0000014	-	-	ENSG0000014860	'POLR3A	-	10:77969250-78029545	-	-	IGV_LINK	592.499	5.34837	1.97454	8.72221	OK	408.365	3.70777	1.21855	6.19698	OK	OK	-0.52855
ENSG0000014	-	-	ENSG0000014863	'HERC4	-	10:67921898-68075348	-	-	IGV_LINK	748.624	13.7964	1.9025	25.6903	OK	497.05	8.04966	1.78699	14.3123	OK	OK	-0.77729
ENSG0000014	-	-	ENSG0000014865	'LRMDA	-	10:75430570-76560994	-	-	IGV_LINK	260.855	34.6827	4.90369	64.4617	OK	191.808	22.629	1.10119	44.1569	OK	OK	-0.61604
ENSG0000014	-	-	ENSG0000014866	'CAMK2G	-	10:73812500-73874591	-	-	IGV_LINK	718.563	14.6223	4.461	24.7836	OK	560.986	11.3098	3.25748	19.362	OK	OK	-0.3706
ENSG0000014	-	-	ENSG0000014867	'ADIRF	-	10:86935539-87024732	-	-	IGV_LINK	2775.19	327.486	0	680.947	OK	585.633	69.611	0	211.313	OK	OK	-2.23405
ENSG0000014	-	-	ENSG0000014867	'GLUD1	-	10:87050485-87191468	-	-	IGV_LINK	2900.43	61.3133	9.99707	112.63	OK	1802.58	38.554	7.17631	69.9316	OK	OK	-0.66932
ENSG0000014	-	-	ENSG0000014867	'ANKRD1	-	10:90912095-90921276	-	-	IGV_LINK	0	0	0	0	OK	3.09367	0.096573	0	0.2495	OK	NOTEST	inf
ENSG0000014	-	-	ENSG0000014868	'HTR7	-	10:90740822-90857698	-	-	IGV_LINK	89.2143	1.76012	0.186431	3.33381	OK	45.3739	0.895343	0	1.79864	OK	OK	-0.97516
ENSG0000014	-	-	ENSG0000014868	'RPP30	-	10:90871715-90908553	-	-	IGV_LINK	321.947	21.175	5.36734	36.9828	OK	186.652	13.077	2.11689	24.037	OK	OK	-0.69534
ENSG0000014	-	-	ENSG0000014869	'FRA10AC1	-	10:93667882-93702572	-	-	IGV_LINK	412.131	9.04569	0.590918	17.5005	OK	181.496	5.63099	0	16.2772	OK	OK	-0.68384
ENSG0000014	-	-	ENSG0000014870	'ADD3	-	10:109940103-110135565	-	-	IGV_LINK	2309.87	56.473	4.8581	108.088	OK	1872.7	42.7995	0	90.0981	OK	OK	-0.39997
ENSG0000014	-	-	ENSG0000014870	'HABP2	-	10:113550836-113664127	-	-	IGV_LINK	93.0932	1.84959	0.178637	3.52053	OK	129.934	2.71371	0.167462	5.25995	OK	OK	0.553063
ENSG0000014	-	-	ENSG0000014870	'VAX1	-	10:117128520-117138301	-	-	IGV_LINK	0	0	0	0	OK	0	0	0	0	OK	NOTEST	0
ENSG0000014	-	-	ENSG0000014871	'DNAJB12	-	10:72332829-72355230	-	-	IGV_LINK	988.145	21.6357	7.17656	36.0949	OK	760.013	16.6297	5.90465	27.3548	OK	OK	-0.37965
ENSG0000014	-	-	ENSG0000014873	'EIF4EBP2	-	10:70404378-70428618	-	-	IGV_LINK	4120.34	32.7764	13.5037	52.049	OK	2506.91	19.9451	8.73625	31.154	OK	OK	-0.71662
ENSG0000014	-	-	ENSG0000014873	'NPFFR1	-	10:70247328-70283676	-	-	IGV_LINK	335.523	2.16135	0.741304	3.5814	OK	70.1233	0.451775	0.001246	0.902304	OK	OK	-2.25826
ENSG0000014	-	-	ENSG0000014873	'PLEKHS1	-	10:113751453-113783429	-	-	IGV_LINK	10.6669	0.38736	0	1.09381	OK	0	0	0	0	OK	NOTEST	'-inf
ENSG0000014	-	-	ENSG0000014873	'TCF7L2	-	10:112950249-113167678	-	-	IGV_LINK	357.827	10.3574	0	22.0144	OK	197.995	6.68911	0	14.9052	OK	OK	-0.63077
ENSG0000014	-	-	ENSG0000014877	'MKI67	-	10:128096658-128126385	-	-	IGV_LINK	839.778	6.00362	0	14.1185	OK	461.989	3.7015	0	9.28092	OK	OK	-0.69772
ENSG0000014	-	-	ENSG0000014879	'CYP17A1	-	10:102830530-102837533	-	-	IGV_LINK	17.455	0.596967	0	1.49888	OK	8.2498	0.226093	0	0.841137	OK	OK	-1.40073
ENSG0000014	-	-	ENSG0000014879	'INA	-	10:103277162-103290351	-	-	IGV_LINK	0.969721	0.017868	0	0.069985	OK	0	0	0	0	OK	NOTEST	'-inf
ENSG0000014	-	-	ENSG0000014880	'FUOM	-	10:133355153-133358035	-	-	IGV_LINK	377.221	40.2342	13.4482	67.0202	OK	154.684	16.866	1.77189	31.96	OK	OK	-1.25431
ENSG0000014	-	-	ENSG0000014881	'LRRC27	-	10:132332153-132379918	-	-	IGV_LINK	516.861	18.6753	4.02068	33.3298	OK	266.056	11.1737	0.442377	21.9049	OK	OK	-0.74103
ENSG0000014	-	-	ENSG0000014882	'MTG1	-	10:133379233-133424572	-	-	IGV_LINK	997.815	46.9221	9.40294	84.4413	OK	536.237	26.3961	3.7441	49.0482	OK	OK	-0.82994
ENSG0000014	-	-	ENSG0000014882	'NKX6-2	-	10:132537819-132786052	-	-	IGV_LINK	20.3641	3.25351	0	12.0638	OK	11.3435	2.12104	0	8.49013	OK	OK	-0.61722
ENSG0000014	-	-	ENSG0000014883	'PAOX	-	10:133379233-133424572	-	-	IGV_LINK	209.46	7.91744	0	19.5265	OK	126.841	4.87255	0	12.077	OK	OK	-0.70036
ENSG0000014	-	-	ENSG0000014883	'GSTO1	-	10:104235355-104267459	-	-	IGV_LINK	2087.81	149.002	65.6779	232.326	OK	1574.68	111.716	49.0191	174.414	OK	OK	-0.41549
ENSG0000014	-	-	ENSG0000014883	'TAF5	-	10:103367966-103396466	-	-	IGV_LINK	106.669	1.94199	0	5.58651	OK	82.498	1.50249	0	4.38065	OK	OK	-0.37018
ENSG0000014	-	-	ENSG0000014884	'PPRC1	-	10:102132993-102150331	-	-	IGV_LINK	583.772	10.2731	0.404052	20.1421	OK	468.176	7.33518	0.382834	14.2875	OK	OK	-0.48597
ENSG0000014	-	-	ENSG0000014884	'ITPRIP	-	10:104309697-104338404	-	-	IGV_LINK	1043.42	13.8137	5.77596	21.8514	OK	1019.88	13.4407	5.62361	21.2578	OK	OK	-0.03949
ENSG0000014	-	-	ENSG0000014884	'CNNM2	-	10:102918292-103193306	-	-	IGV_LINK	282.189	2.81733	0	7.0322	OK	158.809	1.77302	0	4.91222	OK	OK	-0.66812
ENSG0000014	-	-	ENSG0000014884	'PDCD11	-	10:103396647-103446292	-	-	IGV_LINK	968.751	9.46201	2.05945	16.8646	OK	640.391	7.32588	0.030357	14.6214	OK	OK	-0.36914
ENSG0000014	-	-	ENSG0000014884	'ADAM12	-	10:126012380-126388455	-	-	IGV_LINK	36.8494	0.283833	0	0.89363	OK	189.745	2.04344	0	4.46004	OK	OK	2.84788
ENSG0000014	-	-	ENSG0000014890	'RGS10	-	10:119499827-119542708	-	-	IGV_LINK	1046.33	93.3602	39.9757	146.745	OK	567.174	51.1934	19.3278	83.059	OK	OK	-0.86685
ENSG0000014	-	-	ENSG0000014892	'BTBD10	-	11:13388000-13463297	-	-	IGV_LINK	408.252	12.4217	3.71452	21.1288	OK	449.614	12.7666	4.01884	21.5143	OK	OK	0.039512
ENSG0000014	-	-	ENSG0000014892	'ADM	-	11:10304679-10307397	-	-	IGV_LINK	2501.88	126.425	42.2709	210.58	OK	2675	134.106	33.1548	235.057	OK	OK	0.085086
ENSG0000014	-	-	ENSG0000014893	'GAS2	-	11:22622518-22813055	-	-	IGV_LINK	187.156	6.66897	0	19.6308	OK	193.87	9.46756	0	25.8503	OK	OK	0.505529
ENSG0000014	-	-	ENSG0000014894	'SLC5A12	-	11:26667018-26723427	-	-	IGV_LINK	47.5163	0.672479	0	1.50077	OK	68.0608	1.47085	0	3.45189	OK	OK	1.12909
ENSG0000014	-	-	ENSG0000014894	'LIN7C	-	11:27494575-27506773	-	-	IGV_LINK	217.217	2.71098	0.753202	4.66876	OK	237.182	2.96092	0.841976	5.07985	OK	OK	0.127228
ENSG0000014	-	-	ENSG0000014894	'LRRC4C	-	11:40114202-41459773	-	-	IGV_LINK	75.6382	1.47513	0	3.07358	OK	46.4051	0.842195	0	1.76733	OK	OK	-0.80861
ENSG0000014	-	-	ENSG0000014895	'IMMP1L	-	11:31432400-31509645	-	-	IGV_LINK	73.6988	31.4724	0	165.128	OK	73.217	16.9459	0	35.6886	OK	OK	-0.89315
ENSG0000014	-	-	ENSG0000014896	'SAA4	-	11:18231348-18248643	-	-	IGV_LINK	0	0	0	0	OK	258.24	26.3015	0	90.339	OK	OK	inf
ENSG0000014	-	-	ENSG0000014898	'PGAP2	-	11:3665586-3826371	-	-	IGV_LINK	377.221	17.4969	0	41.4877	OK	365.054	17.3728	0	43.575	OK	OK	-0.01027
ENSG0000014	-	-	ENSG0000014901	'TUT1	-	11:62559600-62592177	-	-	IGV_LINK	413.467	10.7301	0	89.2827	OK	588.87	15.8765	0	101.427	OK	OK	0.565226
ENSG0000014	-	-	ENSG0000014902	'SCGB1A1	-	11:62405102-62426724	-	-	IGV_LINK	0	0	0	0	OK	0	0	0	0	OK	NOTEST	0
ENSG0000014	-	-	ENSG0000014904	'SYT8	-	11:1828306-1837521	-	-	IGV_LINK	0.969721	0.02328	0	0.638768	OK	1.03122	0.275623	0	1.24073	OK	NOTEST	3.56554
ENSG0000014	-	-	ENSG0000014905	'ZNF214	-	11:6821941-7020368	-	-	IGV_LINK	120.245	3.46835	0	7.71297	OK	124.778	3.27271	0	7.25537	OK	OK	-0.08376
ENSG0000014	-	-	ENSG0000014905	'ZNF215	-	11:6821941-7020368	-	-	IGV_LINK	25.2127	0.713455	0	2.62787	OK	65.9984	1.23382	0	2.8552	OK	OK	0.790233
ENSG0000014	-	-	ENSG0000014908	'HSD17B12	-	11:43556435-43880726	-	-	IGV_LINK	553.71	26.9828	1.4191	52.5464	OK	687.985	39.0523	2.9233	75.1813	OK	OK	0.533369
ENSG0000014	-	-	ENSG0000014908	'APIP	-	11:34853093-35020591	-	-	IGV_LINK	240.491	11.7621	0.82095	22.7033	OK	260.9	12.2323	0.701203	23.7634	OK	OK	0.056548
ENSG0000014	-	-	ENSG0000014909	'PAMR1	-	11:35431822-35530300	-	-	IGV_LINK	103.76	2.49733	0	5.39162	OK	114.466	2.65735	0.175701	5.139	OK	OK	0.089601
ENSG0000014	-	-	ENSG0000014909	'DGKZ	-	11:46332904-46380554	-	-	IGV_LINK	2350.6	47.0757	7.64149	86.5098	OK	2573.94	49.152	14.1447	84.1593	OK	OK	0.062268
ENSG0000014	-	-	ENSG0000014910	'EIF3M	-	11:32583797-32794658	-	-	IGV_LINK	1613.61	121.78	44.8556	198.704	OK	1272.53	103.498	34.251	172.746	OK	OK	-0.23467
ENSG0000014	-	-	ENSG0000014911	'TNKS1BP1	-	11:57299637-57324952	-	-	IGV_LINK	1696.04	28.779	1.50741	56.0507	OK	3903.19	67.0107	4.99969	129.022	OK	OK	1.21937
ENSG0000014	-	-	ENSG0000014912	'GLYAT	-	11:58640425-58731974	-	-	IGV_LINK	806.807	44.5684	14.7495	74.3872	OK	1405.56	81.514	33.7986	129.229	OK	OK	0.871028
ENSG0000014	-	-	ENSG0000014913	'SERPING1	-	11:57597386-57614853	-	-	IGV_LINK	10888	425.227	106.821	743.633	OK	17078.1	660.418	131.585	1189.25	OK	OK	0.635146
ENSG0000014	-	-	ENSG0000014913	'OR5F1	-	11:55993680-55994625	-	-	IGV_LINK	0	0	0	0	OK	0	0	0	0	OK	NOTEST	0
ENSG0000014	-	-	ENSG0000014913	'SSRP1	-	11:57325602-57335877	-	-	IGV_LINK	2451.45	50.2003	19.6245	80.7761	OK	2754.4	55.6424	23.9332	87.3516	OK	OK	0.148489
ENSG0000014	-	-	ENSG0000014915	'SLC43A1	-	11:57484533-57515786	-	-	IGV_LINK	88.2446	2.81484	0	5.87627	OK	71.1545	1.70781	0	3.44337	OK	OK	-0.72091
ENSG0000014	-	-	ENSG0000014917	'PTPRJ	-	11:47980557-48170841	-	-	IGV_LINK	1600.04	12.8672	2.86721	22.8673	OK	1227.16	9.70296	3.35111	16.0548	OK	OK	-0.40721
ENSG0000014	-	-	ENSG0000014917	'C11orf49	-	11:46936688-47177125	-	-	IGV_LINK	632.258	27.3924	0	61.2323	OK	899.228	39.7265	0	84.1996	OK	OK	0.536326
ENSG0000014	-	-	ENSG0000014918	'ARFGAP2	-	11:46936688-47177125	-	-	IGV_LINK	1710.1	46.6583	3.1095	90.207	OK	1763.02	46.0084	1.24246	90.7744	OK	OK	-0.02023
ENSG0000014	-	-	ENSG0000014918	'CELF1	-	11:47465932-47584562	-	-	IGV_LINK	2283.69	29.6124	0	76.4511	OK	2401.72	30.6453	0	76.3875	OK	OK	0.049465
ENSG0000014	-	-	ENSG0000014919	'HIKESHI	-	11:86302210-86345931	-	-	IGV_LINK	441.223	36.8082	6.08526	67.5311	OK	364.022	29.9839	6.95119	53.0166	OK	OK	-0.29584
ENSG0000014	-	-	ENSG0000014920	'CCDC81	-	11:86374735-86423109	-	-	IGV_LINK	10.6669	0.341068	0	1.04369	OK	7.21857	0.252034	0	0.879594	OK	NOTEST	-0.43644
ENSG0000014	-	-	ENSG0000014921	'SESN3	-	11:95150538-95234104	-	-	IGV_LINK	730.2	4.81507	0.255489	9.37465	OK	719.795	4.47456	0.817464	8.13165	OK	OK	-0.10581
ENSG0000014	-	-	ENSG0000014921	'ENDOD1	-	11:95089809-95132645	-	-	IGV_LINK	741.836	9.26053	3.80079	14.7203	OK	1098.25	13.7133	5.97847	21.4481	OK	OK	0.566408
ENSG0000014	-	-	ENSG0000014923	'CCDC82	-	11:96352768-96389923	-	-	IGV_LINK	507.164	19.2254	0.824769	37.6261	OK	535.206	20.9152	4.47107	37.3593	OK	OK	0.121534
ENSG0000014	-	-	ENSG0000014924	'KLHL35	-	11:75422393-75430629	-	-	IGV_LINK	5.81832	0.156122	0	0.515316	OK	19.5933	0.836812	0	2.20908	OK	OK	2.42223
ENSG0000014	-	-	ENSG0000014925	'TENM4	-	11:78652830-79440948	-	-	IGV_LINK	499.406	3.59671	0	11.4917	OK	717.733	4.49512	0	12.407	OK	OK	0.321684
ENSG0000014	-	-	ENSG0000014925	'SERPINH1	-	11:75562055-75572783	-	-	IGV_LINK	1590.34	47.2756	17.6654	76.8858	OK	2184.13	63.4977	23.5708	103.425	OK	OK	0.425609
ENSG0000014	-	-	ENSG0000014926	'CAPN5	-	11:77066931-77126155	-	-	IGV_LINK	144.488	2.35194	0.330211	4.37367	OK	281.524	4.26376	0.884905	7.64262	OK	OK	0.858277
ENSG0000014	-	-	ENSG0000014926	'INTS4	-	11:77821108-77994678	-	-	IGV_LINK	507.164	17.3194	0	35.0013	OK	542.424	18.1236	0.512209	35.735	OK	OK	0.06548
ENSG0000014	-	-	ENSG0000014926	'PAK1	-	11:77321706-77477030	-	-	IGV_LINK	1768.77	43.6749	10.1154	77.2345	OK	1230.25	34.0808	7.51104	60.6505	OK	OK	-0.35785
ENSG0000014	-	-	ENSG0000014927	'RPS3	-	11:75399485-75422280	-	-	IGV_LINK	12847.8	1348.52	345.077	2351.97	OK	10639.1	1118.89	317.949	1919.83	OK	OK	-0.26931
ENSG0000014	-	-	ENSG0000014928	'ZC3H12C	-	11:110093360-110171841	-	-	IGV_LINK	350.069	3.37504	0.797831	5.95224	OK	287.712	1.96827	0.53507	3.40148	OK	OK	-0.77797
ENSG0000014	-	-	ENSG0000014929	'TTC12	-	11:113314528-113383544	-	-	IGV_LINK	486.8	20.0853	0.263071	39.9076	OK	457.932	16.0105	1.95741	30.0636	OK	OK	-0.32712
ENSG0000014	-	-	ENSG0000014929	'NCAM1	-	11:112959278-113278436	-	-	IGV_LINK	59.153	1.16802	0	3.51178	OK	523.862	7.53959	0.594123	14.4851	OK	OK	2.69042
ENSG0000014	-	-	ENSG0000014929	'DRD2	-	11:113405320-113475691	-	-	IGV_LINK	0	0	0	0	OK	7.21857	0.375852	0	1.22005	OK	NOTEST	inf
ENSG0000014	-	-	ENSG0000014930	'C11orf52	-	11:111908564-111926872	-	-	IGV_LINK	240.141	13.9738	0	56.538	OK	398.053	25.1638	0	143.853	OK	OK	0.848627
ENSG0000014	-	-	ENSG0000014930	'HTR3B	-	11:113904676-113946565	-	-	IGV_LINK	0	0	0	0	OK	0	0	0	0	OK	NOTEST	0
ENSG0000014	-	-	ENSG0000014930	'NPAT	-	11:108157214-108467531	-	-	IGV_LINK	456.738	8.35924	0	29.5886	OK	372.272	6.26039	0	19.2114	OK	OK	-0.41712
ENSG0000014	-	-	ENSG0000014931	'ATM	-	11:108157214-108467531	-	-	IGV_LINK	3471.6	57.0627	0	130.266	OK	2457.41	46.4363	0	103.411	OK	OK	-0.2973
ENSG0000014	-	-	ENSG0000014931	'AASDHPPT	-	11:106051097-106101975	-	-	IGV_LINK	463.526	12.4683	0.536608	24.3999	OK	640.391	16.8025	2.17099	31.434	OK	OK	0.430415
ENSG0000014	-	-	ENSG0000014932	'GLB1L2	-	11:134331873-134378341	-	-	IGV_LINK	787.413	15.1817	5.81639	24.5469	OK	381.553	7.65	2.39256	12.9075	OK	OK	-0.9888
ENSG0000014	-	-	ENSG0000014934	'SLX4IP	-	20:10435302-10636829	-	-	IGV_LINK	253.097	2.42184	0.643887	4.1998	OK	235.119	2.38482	0.185605	4.58403	OK	OK	-0.02222
ENSG0000014	-	-	ENSG0000014935	'LAMTOR1	-	11:71998540-72112780	-	-	IGV_LINK	1119.06	63.6153	0	203.635	OK	1134.35	64.075	0	204.211	OK	OK	0.010389
ENSG0000014	-	-	ENSG0000014938	'P4HA3	-	11:74012713-74324705	-	-	IGV_LINK	46.5466	1.24824	0	5.14509	OK	23.7182	0.6364	0	3.70134	OK	OK	-0.97189
ENSG0000014	-	-	ENSG0000014940	'GRIK4	-	11:120511699-120988904	-	-	IGV_LINK	3.87888	0.063913	0	1.05226	OK	27.8431	0.392622	0	1.47607	OK	NOTEST	2.61896
ENSG0000014	-	-	ENSG0000014941	'ST14	-	11:130159561-130210376	-	-	IGV_LINK	880.506	15.2136	5.8584	24.5688	OK	1757.21	29.9264	12.6941	47.1587	OK	OK	0.976055
ENSG0000014	-	-	ENSG0000014942	'HYOU1	-	11:119044187-119057202	-	-	IGV_LINK	1650.46	24.0149	6.97794	41.0518	OK	1649.96	24.4842	7.58865	41.3797	OK	OK	0.027923
ENSG0000014	-	-	ENSG0000014943	'GGTLC1	-	20:23985049-23988779	-	-	IGV_LINK	0.969721	0.068175	0	0.340524	OK	0	0	0	0	OK	NOTEST	'-inf
ENSG0000014	-	-	ENSG0000014944	'C20orf78	-	20:18809727-18830153	-	-	IGV_LINK	0	0	0	0	OK	0	0	0	0	OK	NOTEST	0
ENSG0000014	-	-	ENSG0000014945	'ADAM33	-	20:3667964-3682246	-	-	IGV_LINK	0.969721	0.030385	0	0.166167	OK	12.3747	0.217495	0	0.496534	OK	NOTEST	2.83956
ENSG0000014	-	-	ENSG0000014945	'SLC22A8	-	11:62989153-63015839	-	-	IGV_LINK	0	0	0	0	OK	1.03122	0.03768	0	0.432844	OK	NOTEST	inf
ENSG0000014	-	-	ENSG0000014947	'KAT14	-	20:18137872-18188387	-	-	IGV_LINK	368.7	6.76612	0.925983	12.6063	OK	326.791	6.0522	0.647991	11.4564	OK	OK	-0.16087
ENSG0000014	-	-	ENSG0000014947	'TKFC	-	11:61299450-61369509	-	-	IGV_LINK	816.46	46.8032	0	153.545	OK	942.309	51.4659	0	161.313	OK	OK	0.137011
ENSG0000014	-	-	ENSG0000014948	'MTA2	-	11:62593213-62601840	-	-	IGV_LINK	1226.7	33.5272	6.69157	60.3628	OK	1280.78	34.2031	8.53452	59.8717	OK	OK	0.028797
ENSG0000014	-	-	ENSG0000014948	'TMEM138	-	11:61299450-61369509	-	-	IGV_LINK	589.59	28.1099	0	103.476	OK	633.172	38.2046	0	140.605	OK	OK	0.442667
ENSG0000014	-	-	ENSG0000014948	'FADS1	-	11:61680432-61867354	-	-	IGV_LINK	605.106	9.85581	0	30.7015	OK	785.793	12.4793	0	40.1393	OK	OK	0.340487
ENSG0000014	-	-	ENSG0000014948	'TMC2	-	20:2536606-2641784	-	-	IGV_LINK	4.8486	0.091189	0	0.228694	OK	3.09367	0.058206	0	0.159369	OK	NOTEST	-0.64769
ENSG0000014	-	-	ENSG0000014948	'ROM1	-	11:62602217-62615120	-	-	IGV_LINK	79.5171	13.1478	0	65.2464	OK	92.8102	7.53609	0	39.8217	OK	OK	-0.80293
ENSG0000014	-	-	ENSG0000014949	'EML3	-	11:62602217-62615120	-	-	IGV_LINK	1413.85	44.7239	7.72842	81.7193	OK	1382.87	42.3835	6.92663	77.8404	OK	OK	-0.07754
ENSG0000014	-	-	ENSG0000014950	'INCENP	-	11:62123972-62153163	-	-	IGV_LINK	329.705	8.77423	0	18.1104	OK	262.962	8.9688	0	18.9743	OK	OK	0.031644
ENSG0000014	-	-	ENSG0000014950	'ZP1	-	11:60867561-60875693	-	-	IGV_LINK	2.90916	0.117065	0	0.595881	OK	14.4371	0.581431	0	1.47668	OK	OK	2.31229
ENSG0000014	-	-	ENSG0000014950	'OOSP2	-	11:60040274-60048044	-	-	IGV_LINK	0	0	0	0	OK	0	0	0	0	OK	NOTEST	0
ENSG0000014	-	-	ENSG0000014951	'MS4A3	-	11:60056586-60071128	-	-	IGV_LINK	0.969721	0.174297	0	0.86097	OK	0	0	0	0	OK	NOTEST	'-inf
ENSG0000014	-	-	ENSG0000014952	'PLCH2	-	1:2425979-2505530	-	-	IGV_LINK	114.427	1.42269	0	3.65755	OK	109.31	1.28197	0.007985	2.55596	OK	OK	-0.15026
ENSG0000014	-	-	ENSG0000014953	'FRG1BP	-	20:30377371-30399257	-	-	IGV_LINK	90.184	8.63652	0.903786	16.3693	OK	112.404	10.7855	1.12514	20.4458	OK	OK	0.320566
ENSG0000014	-	-	ENSG0000014953	'CPSF7	-	11:61402640-61490931	-	-	IGV_LINK	2725.88	57.6667	9.63717	105.696	OK	2631.69	54.2839	10.546	98.0218	OK	OK	-0.08721
ENSG0000014	-	-	ENSG0000014953	'MS4A2	-	11:60088260-60098466	-	-	IGV_LINK	24.243	0.69417	0	1.62808	OK	4.1249	0.121415	0	0.450985	OK	OK	-2.51534
ENSG0000014	-	-	ENSG0000014954	'B3GAT3	-	11:62615295-62622175	-	-	IGV_LINK	695.29	29.1861	10.2614	48.1109	OK	585.736	24.1471	8.46322	39.831	OK	OK	-0.27343
ENSG0000014	-	-	ENSG0000014954	'EI24	-	11:125569215-125681123	-	-	IGV_LINK	1342.09	39.9777	0	82.9061	OK	1553.02	46.347	0.823029	91.871	OK	OK	0.21328
ENSG0000014	-	-	ENSG0000014954	'CCDC15	-	11:124954120-125041489	-	-	IGV_LINK	49.4557	0.857488	0	1.85635	OK	28.8743	0.437504	0	0.956761	OK	OK	-0.97082
ENSG0000014	-	-	ENSG0000014955	'CHEK1	-	11:125569215-125681123	-	-	IGV_LINK	129.943	4.66711	0	23.5202	OK	252.65	7.64648	0	31.2482	OK	OK	0.712267
ENSG0000014	-	-	ENSG0000014955	'FEZ1	-	11:125445744-125499528	-	-	IGV_LINK	107.639	5.01165	0	10.3557	OK	205.214	10.4448	0.361997	20.5275	OK	OK	1.05942
ENSG0000014	-	-	ENSG0000014956	'ESAM	-	11:124752582-124765936	-	-	IGV_LINK	1304.27	44.0298	18.3393	69.7203	OK	1285.94	43.5784	18.8747	68.2822	OK	OK	-0.01487
ENSG0000014	-	-	ENSG0000014957	'KIRREL3	-	11:126355639-127006058	-	-	IGV_LINK	2.90916	0.19299	0	2.03556	OK	7.21857	0.290585	0	2.2264	OK	NOTEST	0.590439
ENSG0000014	-	-	ENSG0000014957	'MPZL2	-	11:118253402-118264536	-	-	IGV_LINK	287.037	10.9853	2.01417	19.9564	OK	656.89	23.932	7.7387	40.1252	OK	OK	1.12337
ENSG0000014	-	-	ENSG0000014957	'SCN2B	-	11:118161950-118176673	-	-	IGV_LINK	0	0	0	0	OK	1.03122	0.010282	0	0.038482	OK	NOTEST	inf
ENSG0000014	-	-	ENSG0000014957	'SIDT2	-	11:117178732-117197445	-	-	IGV_LINK	765.109	13.8822	3.85079	23.9136	OK	1080.72	21.4994	2.95381	40.0449	OK	OK	0.631057
ENSG0000014	-	-	ENSG0000014958	'TMEM25	-	11:118436489-118603033	-	-	IGV_LINK	216.248	6.5275	0	28.4387	OK	203.151	6.7422	0	31.5049	OK	OK	0.046689

ENSG0000014	-	-	ENSG0000014959	'TAGLN	-	11:117199320-117232525	-	-	IGV_LINK	516.861	35.2056	0	80.4672	OK	452.708	30.5134	0	70.1126	OK	OK	-0.20636
ENSG0000014	-	-	ENSG0000014959	'JPH2	-	20:44111694-44187578	-	-	IGV_LINK	303.523	4.1365	1.09572	7.17729	OK	1346.78	17.6263	7.0727	28.1799	OK	OK	2.09125
ENSG0000014	-	-	ENSG0000014959	'DUSP15	-	20:31847636-31952092	-	-	IGV_LINK	48.486	2.48	0	7.44039	OK	42.2802	2.00537	0	6.16723	OK	OK	-0.30648
ENSG0000014	-	-	ENSG0000014960	'COMMD7	-	20:32702690-32743997	-	-	IGV_LINK	985.236	42.2258	16.6398	67.8118	OK	1085.88	47.1863	20.1371	74.2354	OK	OK	0.160242
ENSG0000014	-	-	ENSG0000014960	'C20orf144	-	20:33657086-33674463	-	-	IGV_LINK	3.87888	0.10502	0	0.711046	OK	2.06245	0.145515	0	1.57646	OK	NOTEST	0.470511
ENSG0000014	-	-	ENSG0000014963	'KIAA1755	-	20:38210487-38337505	-	-	IGV_LINK	80.4868	1.8569	0	4.10117	OK	74.2482	1.82161	0	3.9934	OK	OK	-0.02769
ENSG0000014	-	-	ENSG0000014963	'SPATA25	-	20:45886488-45887635	-	-	IGV_LINK	37.8191	3.64655	0	7.40573	OK	63.9359	6.17692	0.056603	12.2972	OK	OK	0.760354
ENSG0000014	-	-	ENSG0000014963	'OCSTAMP	-	20:46540945-46550574	-	-	IGV_LINK	0	0	0	0	OK	1.03122	0.031089	0	0.116359	OK	NOTEST	inf
ENSG0000014	-	-	ENSG0000014963	'DSN1	-	20:36751790-36773818	-	-	IGV_LINK	275.401	7.7349	2.09637	13.3734	OK	312.461	9.2275	2.76832	15.6867	OK	OK	0.254557
ENSG0000014	-	-	ENSG0000014963	'SOGA1	-	20:36777441-36863686	-	-	IGV_LINK	966.811	4.03069	0.807792	7.25358	OK	965.227	3.91857	1.58622	6.25092	OK	OK	-0.0407
ENSG0000014	-	-	ENSG0000014964	'CNBD2	-	20:35953616-36030700	-	-	IGV_LINK	72.729	7.99169	0	21.2422	OK	57.7486	6.28941	0	17.9124	OK	OK	-0.34558
ENSG0000014	-	-	ENSG0000014965	'SPINT4	-	20:45722350-45725830	-	-	IGV_LINK	0	0	0	0	OK	0	0	0	0	OK	NOTEST	0
ENSG0000014	-	-	ENSG0000014965	'CDH22	-	20:46173732-46308498	-	-	IGV_LINK	162.913	2.62186	0.564228	4.6795	OK	7.21857	0.109113	0	0.27862	OK	OK	-4.58669
ENSG0000014	-	-	ENSG0000014965	'LINC00266-1	-	20:64290186-64313132	-	-	IGV_LINK	0	0	0	0	OK	0	0	0	0	OK	NOTEST	0
ENSG0000014	-	-	ENSG0000014965	'LSM14B	-	20:62122460-62135378	-	-	IGV_LINK	555.65	15.7746	5.47549	26.0736	OK	543.456	14.4228	5.19031	23.6552	OK	OK	-0.12925
ENSG0000014	-	-	ENSG0000014965	'YTHDF1	-	20:63195428-63216234	-	-	IGV_LINK	520.74	9.49277	3.5395	15.446	OK	645.547	11.633	4.71777	18.5481	OK	OK	0.293317
ENSG0000014	-	-	ENSG0000014967	'CABLES2	-	20:62388631-62407285	-	-	IGV_LINK	124.124	1.99217	0.051146	3.9332	OK	86.6229	1.52246	0	3.33929	OK	OK	-0.38793
ENSG0000014	-	-	ENSG0000014971	'ORAOV1	-	11:69641086-69675416	-	-	IGV_LINK	877.597	47.335	0	158.038	OK	669.265	35.3394	0	118.951	OK	OK	-0.42163
ENSG0000014	-	-	ENSG0000014973	'GPHA2	-	11:64917552-64935888	-	-	IGV_LINK	6.78804	0.665903	0	3.81726	OK	10.3122	1.27974	0	6.79451	OK	OK	0.942464
ENSG0000014	-	-	ENSG0000014974	'SLC22A9	-	11:63369788-63410294	-	-	IGV_LINK	0	0	0	0	OK	0	0	0	0	OK	NOTEST	0
ENSG0000014	-	-	ENSG0000014974	'TRPT1	-	11:64206677-64234352	-	-	IGV_LINK	490.679	34.6639	0	90.285	OK	467.145	33.4909	0	89.6385	OK	OK	-0.04966
ENSG0000014	-	-	ENSG0000014976	'NUDT22	-	11:64206677-64234352	-	-	IGV_LINK	682.511	48.0273	0	119.474	OK	712.025	49.3755	0	110.929	OK	OK	0.039943
ENSG0000014	-	-	ENSG0000014978	'FERMT3	-	11:64206677-64234352	-	-	IGV_LINK	1148.15	36.7972	0	78.9515	OK	1123	35.8035	0	78.7053	OK	OK	-0.0395
ENSG0000014	-	-	ENSG0000014978	'PLCB3	-	11:64251522-64269150	-	-	IGV_LINK	481.951	7.19413	1.30936	13.0789	OK	466.114	8.24556	0	17.2405	OK	OK	0.196797
ENSG0000014	-	-	ENSG0000014979	'MRPL49	-	11:65120627-65134533	-	-	IGV_LINK	1015.3	33.4224	0	100.601	OK	1190.03	36.7102	0	104.353	OK	OK	0.135365
ENSG0000014	-	-	ENSG0000014979	'CDC42EP2	-	11:65314817-65322429	-	-	IGV_LINK	82.4262	2.51026	0.213802	4.80672	OK	102.091	3.11108	0.2671	5.95506	OK	OK	0.30958
ENSG0000014	-	-	ENSG0000014980	'FAU	-	11:65120627-65134533	-	-	IGV_LINK	6112.01	887.7	111.637	1663.76	OK	4327.91	594.228	111.127	1077.33	OK	OK	-0.57906
ENSG0000014	-	-	ENSG0000014980	'TM7SF2	-	11:65084222-65116384	-	-	IGV_LINK	184.247	9.12831	0	29.1824	OK	100.029	4.99039	0	19.2199	OK	OK	-0.87119
ENSG0000014	-	-	ENSG0000014982	'VPS51	-	11:65084222-65116384	-	-	IGV_LINK	1319.79	60.3091	0	121.095	OK	1206.53	55.293	0	110.836	OK	OK	-0.12528
ENSG0000014	-	-	ENSG0000014992	'TBX6	-	16:30085792-30091887	-	-	IGV_LINK	114.427	3.69206	0.473547	6.91056	OK	82.498	2.69977	0.063815	5.33572	OK	OK	-0.45159
ENSG0000014	-	-	ENSG0000014992	'PPP4C	-	16:30075977-30085377	-	-	IGV_LINK	1150.09	59.4439	24.3755	94.5123	OK	1272.53	63.521	24.6083	102.434	OK	OK	0.095704
ENSG0000014	-	-	ENSG0000014992	'ALDOA	-	16:30053089-30070457	-	-	IGV_LINK	26810.8	1501.08	75.1475	2927.01	OK	32665.1	1750.25	9.12852	3491.36	OK	OK	0.221557
ENSG0000014	-	-	ENSG0000014992	'FAM57B	-	16:30023333-30052978	-	-	IGV_LINK	8.72748	0.396624	0	1.02016	OK	9.28102	0.432459	0	1.16095	OK	NOTEST	0.124794
ENSG0000014	-	-	ENSG0000014992	'DOC2A	-	16:29992320-30023270	-	-	IGV_LINK	1082.21	37.6144	0	82.202	OK	1199.31	43.3163	0	94.125	OK	OK	0.203627
ENSG0000014	-	-	ENSG0000014992	'HIRIP3	-	16:29992320-30023270	-	-	IGV_LINK	451.89	14.2881	0	43.5804	OK	443.427	15.4705	0	47.9197	OK	OK	0.114699
ENSG0000014	-	-	ENSG0000014993	'TAOK2	-	16:29973640-29992261	-	-	IGV_LINK	1686.34	24.213	8.81108	39.6149	OK	1716.99	24.3953	9.03957	39.751	OK	OK	0.010821
ENSG0000014	-	-	ENSG0000014993	'TMEM219	-	16:29940884-29973052	-	-	IGV_LINK	1979.2	139.626	58.9327	220.32	OK	1986.14	141.052	58.2415	223.862	OK	OK	0.014651
ENSG0000014	-	-	ENSG0000014994	'HMGA2	-	12:65758019-65966295	-	-	IGV_LINK	0	0	0	0	OK	0	0	0	0	OK	NOTEST	0
ENSG0000014	-	-	ENSG0000014996	'MMP3	-	11:102746967-102843803	-	-	IGV_LINK	0	0	0	0	OK	0	0	0	0	OK	NOTEST	0
ENSG0000014	-	-	ENSG0000014997	'CNKSR2	-	X:21374417-21658330	-	-	IGV_LINK	0.969721	0.013062	0	0.436784	OK	4.1249	0.149659	0	0.717337	OK	NOTEST	3.51828
ENSG0000014	-	-	ENSG0000014997	'CNTN5	-	11:99020952-100358885	-	-	IGV_LINK	0	0	0	0	OK	0	0	0	0	OK	NOTEST	0
ENSG0000015	-	-	ENSG0000015004	'KLRF1	-	12:9827480-9845007	-	-	IGV_LINK	38.7888	2.21108	0	4.54586	OK	49.4988	2.68136	0	5.41339	OK	OK	0.278211
ENSG0000015	-	-	ENSG0000015004	'CLEC1A	-	12:10069553-10111627	-	-	IGV_LINK	132.852	4.9592	0.393375	9.52503	OK	117.56	4.28084	0.042721	8.51896	OK	OK	-0.21222
ENSG0000015	-	-	ENSG0000015005	'MKX	-	10:27672874-27767794	-	-	IGV_LINK	6.78804	0.130965	0	0.487648	OK	2.06245	0.033548	0	0.326656	OK	NOTEST	-1.96489
ENSG0000015	-	-	ENSG0000015005	'MPP7	-	10:28050992-28334486	-	-	IGV_LINK	56.2438	1.53176	0	3.75224	OK	27.8431	0.396187	0	0.97953	OK	OK	-1.95093
ENSG0000015	-	-	ENSG0000015009	'ITGB1	-	10:32900318-33082102	-	-	IGV_LINK	8063.23	142.553	30.3563	254.75	OK	6430.72	115.357	22.7721	207.943	OK	OK	-0.30539
ENSG0000015	-	-	ENSG0000015017	'FRMPD2B	-	10:46870857-46894562	-	-	IGV_LINK	25.2127	1.2098	0	2.51156	OK	19.5933	0.941076	0	2.08246	OK	OK	-0.36238
ENSG0000015	-	-	ENSG0000015020	'FXYD4	-	10:43371641-43376335	-	-	IGV_LINK	0	0	0	0	OK	0	0	0	0	OK	NOTEST	0
ENSG0000015	-	-	ENSG0000015024	'TRIM48	-	11:55262181-55271119	-	-	IGV_LINK	0	0	0	0	OK	0	0	0	0	OK	NOTEST	0
ENSG0000015	-	-	ENSG0000015026	'OR8K1	-	11:56345944-56347031	-	-	IGV_LINK	0	0	0	0	OK	0	0	0	0	OK	NOTEST	0
ENSG0000015	-	-	ENSG0000015026	'OR5M9	-	11:56459220-56473352	-	-	IGV_LINK	0	0	0	0	OK	0	0	0	0	OK	NOTEST	0
ENSG0000015	-	-	ENSG0000015027	'PCDH15	-	10:53802770-55627942	-	-	IGV_LINK	0	0	0	0	OK	1.03122	0.045143	0	0.462927	OK	NOTEST	inf
ENSG0000015	-	-	ENSG0000015027	'PPIAP26	-	13:52820496-52821018	-	-	IGV_LINK	0	0	0	0	OK	0	0	0	0	OK	NOTEST	0
ENSG0000015	-	-	ENSG0000015028	'CTF1	-	16:30896606-30903560	-	-	IGV_LINK	73.6988	2.78473	0.181113	5.38835	OK	61.8735	2.34167	0	4.73239	OK	OK	-0.25
ENSG0000015	-	-	ENSG0000015031	'CWC15	-	11:94962621-94973612	-	-	IGV_LINK	1041.48	56.3557	18.9909	93.7204	OK	1045.66	55.6323	19.3447	91.9199	OK	OK	-0.01864
ENSG0000015	-	-	ENSG0000015033	'FCGR1A	-	1:149782670-149812373	-	-	IGV_LINK	460.617	33.3131	0	68.6808	OK	480.551	28.8021	0	60.4086	OK	OK	-0.20991
ENSG0000015	-	-	ENSG0000015034	'ARID5B	-	10:61901299-62096944	-	-	IGV_LINK	1203.42	10.8477	2.97507	18.7204	OK	683.702	5.60278	2.06755	9.13801	OK	OK	-0.95318
ENSG0000015	-	-	ENSG0000015036	'KLHL1	-	13:69700593-70139429	-	-	IGV_LINK	0	0	0	0	OK	0	0	0	0	OK	NOTEST	0
ENSG0000015	-	-	ENSG0000015039	'CDH8	-	16:61647241-62037035	-	-	IGV_LINK	5.81832	0.160556	0	0.976234	OK	8.2498	0.470196	0	1.56088	OK	NOTEST	1.55019
ENSG0000015	-	-	ENSG0000015040	'DCUN1D2	-	13:113455818-113490952	-	-	IGV_LINK	208.49	6.63863	0	14.0077	OK	163.965	6.8652	0	15.0578	OK	OK	0.048416
ENSG0000015	-	-	ENSG0000015040	'TMCO3	-	13:113490994-113554590	-	-	IGV_LINK	720.502	16.1298	1.63345	30.6261	OK	1169.41	24.919	5.83554	44.0024	OK	OK	0.627517
ENSG0000015	-	-	ENSG0000015043	'TMEM218	-	11:125096544-125111763	-	-	IGV_LINK	310.311	16.7269	3.09347	30.3604	OK	317.617	19.0276	3.73638	34.3188	OK	OK	0.18592
ENSG0000015	-	-	ENSG0000015045	'TIRAP	-	11:126283064-126355587	-	-	IGV_LINK	172.61	5.42646	0	12.2506	OK	194.902	6.87404	0	16.1754	OK	OK	0.341148
ENSG0000015	-	-	ENSG0000015045	'EEF1AKMT1	-	13:20728730-20773958	-	-	IGV_LINK	64.0016	4.59072	0	10.1971	OK	83.5292	6.8414	0	14.8924	OK	OK	0.57557
ENSG0000015	-	-	ENSG0000015045	'LATS2	-	13:20973031-21061547	-	-	IGV_LINK	433.465	4.58473	1.58641	7.58305	OK	407.334	4.30108	1.53547	7.06669	OK	OK	-0.09214
ENSG0000015	-	-	ENSG0000015045	'SAP18	-	13:21140513-21149084	-	-	IGV_LINK	2012.17	132.415	21.1922	243.638	OK	2323.35	165.972	17.7465	314.197	OK	OK	0.32587
ENSG0000015	-	-	ENSG0000015047	'ADGRL3	-	4:61201257-62165554	-	-	IGV_LINK	10.6669	0.283641	0	1.06206	OK	7.21857	0.077277	0	0.431463	OK	NOTEST	-1.87596
ENSG0000015	-	-	ENSG0000015047	'KIAA1328	-	18:36297713-37232172	-	-	IGV_LINK	280.249	6.9859	0	17.5403	OK	215.526	5.70771	0	17.0696	OK	OK	-0.29154
ENSG0000015	-	-	ENSG0000015051	'FAM124A	-	13:51222333-51284241	-	-	IGV_LINK	39.7585	0.505332	0	1.06699	OK	113.435	1.46639	0.121977	2.8108	OK	OK	1.53697
ENSG0000015	-	-	ENSG0000015052	'CTAGE5	-	14:39230230-39388513	-	-	IGV_LINK	951.296	20.0207	3.90343	36.1379	OK	1228.19	24.4126	6.20854	42.6166	OK	OK	0.286136
ENSG0000015	-	-	ENSG0000015054	'HNMT	-	2:137964019-138016364	-	-	IGV_LINK	2404.91	130.319	34.4382	226.199	OK	3247.33	173.204	48.3882	298.019	OK	OK	0.410424
ENSG0000015	-	-	ENSG0000015055	'LYPD1	-	2:132416573-132671579	-	-	IGV_LINK	75.6382	2.48047	0	5.08198	OK	128.903	4.28832	0.429832	8.14681	OK	OK	0.789801
ENSG0000015	-	-	ENSG0000015055	'LYPD6B	-	2:149038106-149215262	-	-	IGV_LINK	0	0	0	0	OK	0	0	0	0	OK	NOTEST	0
ENSG0000015	-	-	ENSG0000015059	'PDCD4	-	10:110869742-110919274	-	-	IGV_LINK	1190.82	84.7422	18.2121	151.272	OK	850.761	57.619	13.2125	102.025	OK	OK	-0.55654
ENSG0000015	-	-	ENSG0000015059	'ADRA2A	-	10:111077162-111080907	-	-	IGV_LINK	10.6669	0.168269	0	0.377497	OK	12.3747	0.195272	0	0.435807	OK	NOTEST	0.214714
ENSG0000015	-	-	ENSG0000015062	'GPM6A	-	4:175632933-176002664	-	-	IGV_LINK	10.6669	0.808644	0	2.44649	OK	1.03122	0.15361	0	1.02006	OK	OK	-2.39624
ENSG0000015	-	-	ENSG0000015062	'WDR17	-	4:176065833-176182818	-	-	IGV_LINK	208.49	3.31728	0	7.50819	OK	121.685	1.60921	0	3.33304	OK	OK	-1.04365
ENSG0000015	-	-	ENSG0000015062	'SPATA4	-	4:176184637-176195671	-	-	IGV_LINK	31.0311	2.06835	0	5.02292	OK	34.0304	1.78357	0	3.83821	OK	OK	-0.21371
ENSG0000015	-	-	ENSG0000015063	'VEGFC	-	4:176669620-176792727	-	-	IGV_LINK	138.67	4.43025	0.618554	8.24196	OK	287.712	8.78601	2.69714	14.8749	OK	OK	0.987818
ENSG0000015	-	-	ENSG0000015063	'CCDC102B	-	18:68673687-69055189	-	-	IGV_LINK	146.428	5.70641	0	15.664	OK	110.341	5.27293	0	16.7532	OK	OK	-0.11398
ENSG0000015	-	-	ENSG0000015063	'CD226	-	18:69401054-69961803	-	-	IGV_LINK	195.884	4.12311	0	9.40079	OK	147.465	3.54195	0	10.1231	OK	OK	-0.21919
ENSG0000015	-	-	ENSG0000015065	'CNDP1	-	18:74534439-74587212	-	-	IGV_LINK	17.455	0.571222	0	1.46412	OK	58.7798	2.23676	0	5.07633	OK	OK	1.96929
ENSG0000015	-	-	ENSG0000015066	'FSIP1	-	15:39600030-39920892	-	-	IGV_LINK	24.243	0.52977	0	2.71428	OK	13.4059	0.287116	0	1.85329	OK	NOTEST	-0.88373
ENSG0000015	-	-	ENSG0000015067	'DLG2	-	11:83455011-85627922	-	-	IGV_LINK	63.0318	0.932901	0	3.06946	OK	59.811	1.16209	0	3.37609	OK	OK	0.31693
ENSG0000015	-	-	ENSG0000015067	'CCDC83	-	11:85855100-85923682	-	-	IGV_LINK	0	0	0	0	OK	1.03122	0.067945	0	0.420992	OK	NOTEST	inf
ENSG0000015	-	-	ENSG0000015068	'RGS18	-	1:192158456-192185815	-	-	IGV_LINK	236.612	7.68471	1.18797	14.1815	OK	235.119	7.57363	1.12348	14.0238	OK	OK	-0.02101
ENSG0000015	-	-	ENSG0000015068	'PRSS23	-	11:86791058-86955391	-	-	IGV_LINK	1642.71	36.585	0	87.975	OK	1285.94	35.5908	0	80.4538	OK	OK	-0.03975
ENSG0000015	-	-	ENSG0000015071	'MTMR12	-	5:32226993-32313009	-	-	IGV_LINK	735.048	8.91683	3.33425	14.4994	OK	810.543	9.72195	3.81239	15.6315	OK	OK	0.124715
ENSG0000015	-	-	ENSG0000015072	'PPP1R1C	-	2:181954240-182131398	-	-	IGV_LINK	7.75776	0.977715	0	2.6427	OK	34.0304	5.11193	0	10.9322	OK	OK	2.38638
ENSG0000015	-	-	ENSG0000015073	'AL136372.1	-	1:188067297-188067525	-	-	IGV_LINK	0	0	0	0	OK	0	0	0	0	OK	NOTEST	0
ENSG0000015	-	-	ENSG0000015075	'C11orf53	-	11:111245804-111286401	-	-	IGV_LINK	0	0	0	0	OK	0	0	0	0	OK	NOTEST	0
ENSG0000015	-	-	ENSG0000015075	'CCT5	-	5:10249920-10267146	-	-	IGV_LINK	1592.28	72.0065	19.819	124.194	OK	1837.64	86.0021	20.1817	151.822	OK	OK	0.256244
ENSG0000015	-	-	ENSG0000015075	'FAM173B	-	5:10226329-10249915	-	-	IGV_LINK	120.245	6.81231	0	14.1236	OK	129.934	6.26361	0.095207	12.432	OK	OK	-0.12115
ENSG0000015	-	-	ENSG0000015076	'DOCK1	-	10:126905408-127452517	-	-	IGV_LINK	1208.27	10.6992	3.84861	17.5498	OK	697.108	7.57746	0	19.0083	OK	OK	-0.49772
ENSG0000015	-	-	ENSG0000015076	'DIXDC1	-	11:111927143-112022584	-	-	IGV_LINK	953.235	14.6786	3.11705	26.2401	OK	1167.35	17.4669	3.05071	31.8831	OK	OK	0.250908
ENSG0000015	-	-	ENSG0000015076	'DLAT	-	11:112024813-112224699	-	-	IGV_LINK	509.103	11.726	0	33.9007	OK	631.11	14.7942	0	40.296	OK	OK	0.335326
ENSG0000015	-	-	ENSG0000015077	'PIH1D2	-	11:112024813-112224699	-	-	IGV_LINK	134.791	6.32452	0	29.4422	OK	145.403	7.72536	0	36.625	OK	OK	0.288647
ENSG0000015	-	-	ENSG0000015077	'C11orf57	-	11:112024813-112224699	-	-	IGV_LINK	868.87	21.883	0	66.4425	OK	819.824	20.8836	0	67.531	OK	OK	-0.06744
ENSG0000015	-	-	ENSG0000015077	'TIMM8B	-	11:112024813-112224699	-	-	IGV_LINK	473.224	38.0328	0	102.221	OK	512.519	41.7462	0	112.869	OK	OK	0.134402
ENSG0000015	-	-	ENSG0000015078	'IL18	-	11:112024813-112224699	-	-	IGV_LINK	580.863	31.9032	0	80.7893	OK	443.427	23.6824	0	67.6501	OK	OK	-0.42988
ENSG0000015	-	-	ENSG0000015078	'TEX12	-	11:112024813-112224699	-	-	IGV_LINK	0	0	0	0	OK	0	0	0	0	OK	NOTEST	0
ENSG0000015	-	-	ENSG0000015078	'PTS	-	11:112226364-112269955	-	-	IGV_LINK	213.339	18.6309	4.64202	32.6198	OK	236.151	19.388	5.18248	33.5935	OK	OK	0.057465
ENSG0000015	-	-	ENSG0000015086	'PIP4K2A	-	10:22534848-22714555	-	-	IGV_LINK	717.593	16.0157	0	32.1841	OK	656.89	20.3114	0	43.3436	OK	OK	0.342802
ENSG0000015	-	-	ENSG0000015087	'C2orf50	-	2:11133052-11146790	-	-	IGV_LINK	8.72748	0.320135	0	0.81171	OK	5.15612	0.14177	0	0.392269	OK	NOTEST	-1.17512
ENSG0000015	-	-	ENSG0000015089	'FREM2	-	13:38687128-38887131	-	-	IGV_LINK	640.016	2.89751	0	7.04904	OK	475.395	1.68926	0.604074	2.77444	OK	OK	-0.77843
ENSG0000015	-	-	ENSG0000015090	'FOXO1	-	13:40079105-40666597	-	-	IGV_LINK	362.675	3.67439	1.18371	6.16507	OK	321.742	3.26035	0.969508	5.5512	OK	OK	-0.17248
ENSG0000015	-	-	ENSG0000015093	'CRIM1	-	2:36355925-36646087	-	-	IGV_LINK	2944.07	31.6477	0	67.7714	OK	2461.53	28.7088	0	60.8473	OK	OK	-0.14061
ENSG0000015	-	-	ENSG0000015096	'SEC24D	-	4:118722822-118838683	-	-	IGV_LINK	543.043	10.14	2.4269	17.8532	OK	764.138	13.7514	4.27975	23.223	OK	OK	0.439513
ENSG0000015	-	-	ENSG0000015096	'ABCB9	-	12:122920950-122982913	-	-	IGV_LINK	158.064	4.49448	0	20.5971	OK	223.776	4.97296	0	19.5035	OK	OK	0.145949
ENSG0000015	-	-	ENSG0000015097	'RILPL2	-	12:123410682-123436717	-	-	IGV_LINK	595.408	5.62195	2.24936	8.99453	OK	697.108	6.58348	2.68731	10.4797	OK	OK	0.227781
ENSG0000015	-	-	ENSG0000015099	'DHX37	-	12:124946824-124989122	-	-	IGV_LINK	571.165	8.74884	1.85203	15.6456	OK	687.827	10.7458	2.13322	19.3584	OK	OK	0.296608
ENSG0000015	-	-	ENSG0000015099	'UBC	-	12:124911603-124917368	-	-	IGV_LINK	22394.7	747.488	0.972191	1494	OK	26092.1	849.572	0	1731.8	OK	OK	0.184686
ENSG0000015	-	-	ENSG0000015099	'ITPR1	-	3:4493347-4887293	-	-	IGV_LINK	1715.44	21.2354	0	45.3465	OK	1261.19	20.3255	0	40.9226	OK	OK	-0.06318
ENSG0000015	-	-	ENSG0000015100	'TKTL2	-	4:163471092-163473746	-	-	IGV_LINK	0.969721	0.022023	0	0.086258	OK	4.1249	0.093722	0	0.237422	OK	NOTEST	2.08939
ENSG0000015	-	-	ENSG0000015100	'PRSS53	-	16:31074421-31095980	-	-	IGV_LINK	173.205	2.0647	0	6.49088	OK	178.798	2.44532	0	8.68417	OK	OK	0.24409
ENSG0000015	-	-	ENSG0000015101	'SLC7A11	-	4:138027421-138242349	-	-	IGV_LINK	27.1522	0.16154	0	0.40944	OK	60.8423	0.362018	0	0.773983	OK	NOTEST	1.16417
ENSG0000015	-	-	ENSG0000015101	'NOCT	-	4:139015788-139177218	-	-	IGV_LINK	84.3657	4.3533	0	13.651	OK	302.149	12.7645	0	30.188	OK	OK	1.55196
ENSG0000015	-	-	ENSG0000015102	'ENKUR	-	10:24981978-25062279	-	-	IGV_LINK	67.8804	2.24204	0	5.48471	OK	67.0296	2.40555	0	6.42888	OK	OK	0.101556
ENSG0000015	-	-	ENSG0000015102	'GPR158	-	10:25113057-25602226	-	-	IGV_LINK	0.969721	0.011843	0	0.165737	OK	0	0	0	0	OK	NOTEST	'-inf
ENSG0000015	-	-	ENSG0000015103	'LYZL2	-	10:30611778-30629762	-	-	IGV_LINK	0	0	0	0	OK	0	0	0	0	OK	NOTEST	0
ENSG0000015	-	-	ENSG0000015106	'CACNA2D4	-	12:1791956-1922867	-	-	IGV_LINK	205.581	8.0228	0	16.5787	OK	328.961	13.1404	0.309866	25.9709	OK	OK	0.71183
ENSG0000015	-	-	ENSG0000015106	'DCP1B	-	12:1946053-2941140	-	-	IGV_LINK	332.614	9.7392	0	30.7635	OK	376.397	11.016	0	32.5264	OK	OK	0.177727
ENSG0000015	-	-	ENSG0000015106	'CACNA1C	-	12:1946053-2941140	-	-	IGV_LINK	335.523	2.80637	0	13.6595	OK	243.369	1.84575	0	9.66253	OK	OK	-0.6045
ENSG0000015	-	-	ENSG0000015107	'AC005833.1	-	12:4562203-4851927	-	-	IGV_LINK	1.13855	0.015786	0	0.310568	OK	9.20E-92	1.28E-93	0	0.027739	OK	NOTEST	-302.603
ENSG0000015	-	-	ENSG0000015109	'THRB	-	3:24117159-24681711	-	-	IGV_LINK	78.5474	1.58927	0	4.79061	OK	148.496	2.54638	0	6.75793	OK	OK	0.680082
ENSG0000015	-	-	ENSG0000015109	'NGLY1	-	3:25718943-25794534	-	-	IGV_LINK	421.828	11.8453	2.34467	21.346	OK	453.739	12.6832	3.79269	21.5738	OK	OK	0.098604
ENSG0000015	-	-	ENSG0000015109	'OXSM	-	3:25718943-25794534	-	-	IGV_LINK	120.245	6.7406	0	18.8963	OK	124.778	6.7618	0	17.1724	OK	OK	0.004531
ENSG0000015	-	-	ENSG0000015111	'UEVLD	-	11:18529608-18588747	-	-	IGV_LINK	264.734	6.50652	0.38653	12.6265	OK	342.367	8.08444	1.3673	14.8016	OK	OK	0.313262
ENSG0000015	-	-	ENSG0000015111	'TMEM86A	-	11:18693121-18792721	-	-	IGV_LINK	344.251	5.66852	1.77128	9.56577	OK	531.081	9.56635	1.9445	17.1882	OK	OK	0.754996
ENSG0000015	-	-	ENSG0000015113	'C12orf45	-	12:104986309-105084577	-	-	IGV_LINK	191.035	6.02903	0	15.6299	OK	173.246	6.91744	0	16.1851	OK	OK	0.198313
ENSG0000015	-	-	ENSG0000015113	'TMEM263	-	12:106955718-106987166	-	-	IGV_LINK	455.769	13.3033	0	27.2408	OK	607.392	18.5247	1.76808	35.2814	OK	OK	0.477671
ENSG0000015	-	-	ENSG0000015113	'BTBD11	-	12:107318412-107659642	-	-	IGV_LINK	42.6677	0.513945	0	1.23184	OK	321.742	3.48134	1.05067	5.91202	OK	OK	2.75996

ENSG0000015	-	-	ENSG0000015114	'UBE3B	-	12:109347902-109536705	-	-	IGV_LINK	1138.45	13.9017	0.621857	27.1815	OK	1374.62	16.5211	0.797785	32.2444	OK	OK	0.24905
ENSG0000015	-	-	ENSG0000015115	'ANK3	-	10:60026297-60733490	-	-	IGV_LINK	1690.22	30.4547	1.23125	59.6781	OK	807.449	15.131	2.04338	28.2186	OK	OK	-1.00916
ENSG0000015	-	-	ENSG0000015115	'IPMK	-	10:58191516-58267934	-	-	IGV_LINK	193.944	1.83371	0.40796	3.25946	OK	173.246	1.63833	0.391338	2.88532	OK	OK	-0.16254
ENSG0000015	-	-	ENSG0000015116	'RAD9B	-	12:110491096-110532086	-	-	IGV_LINK	22.4513	0.806115	0	5.08155	OK	21.6557	0.69038	0	4.86581	OK	OK	-0.2236
ENSG0000015	-	-	ENSG0000015117	'PLBD2	-	12:113298758-113391625	-	-	IGV_LINK	1209.24	15.203	2.05528	28.3508	OK	1758.24	21.6238	5.52759	37.7201	OK	OK	0.508264
ENSG0000015	-	-	ENSG0000015120	'DLG5	-	10:77782865-77926526	-	-	IGV_LINK	812.626	8.73876	0	19.1134	OK	653.797	5.71027	1.68147	9.73907	OK	OK	-0.61387
ENSG0000015	-	-	ENSG0000015122	'MAT1A	-	10:80271819-80289684	-	-	IGV_LINK	0	0	0	0	OK	11.3435	0.285596	0	0.833882	OK	NOTEST	inf
ENSG0000015	-	-	ENSG0000015122	'SLC2A13	-	12:39626166-40106089	-	-	IGV_LINK	318.068	3.0773	0.653431	5.50116	OK	222.745	2.08315	0.4766	3.68971	OK	OK	-0.56289
ENSG0000015	-	-	ENSG0000015123	'GXYLT1	-	12:42081844-42144879	-	-	IGV_LINK	327.766	2.52188	0.80208	4.24168	OK	439.302	3.59963	1.14745	6.05182	OK	OK	0.513351
ENSG0000015	-	-	ENSG0000015123	'TWF1	-	12:43793722-43806375	-	-	IGV_LINK	536.255	19.3957	1.92943	36.8621	OK	821.886	27.7259	4.77297	50.6788	OK	OK	0.515493
ENSG0000015	-	-	ENSG0000015124	'DIP2C	-	10:274189-689668	-	-	IGV_LINK	952.266	7.51088	1.6982	13.3236	OK	328.961	2.5155	0.616915	4.41409	OK	OK	-1.57813
ENSG0000015	-	-	ENSG0000015124	'EIF4E	-	4:98871683-98994994	-	-	IGV_LINK	529.467	34.7147	8.9266	60.5027	OK	506.331	33.8519	9.08363	58.6203	OK	OK	-0.03631
ENSG0000015	-	-	ENSG0000015127	'MAGI1	-	3:65353524-66038834	-	-	IGV_LINK	304.492	3.27445	0	6.75245	OK	390.834	4.7454	0	10.1654	OK	OK	0.535277
ENSG0000015	-	-	ENSG0000015128	'TEX30	-	13:102765989-102773811	-	-	IGV_LINK	230.793	12.9538	2.91562	22.9919	OK	145.403	8.28009	1.59229	14.9679	OK	OK	-0.64566
ENSG0000015	-	-	ENSG0000015129	'CSNK1G3	-	5:123512098-123617045	-	-	IGV_LINK	346.19	7.20736	1.69552	12.7192	OK	399.084	8.8029	0	19.4338	OK	OK	0.288508
ENSG0000015	-	-	ENSG0000015130	'AL136982.1	-	10:86935539-87024732	-	-	IGV_LINK	41.8019	0.536929	0	3.9349	OK	11.7654	0.151161	0	1.57352	OK	NOTEST	-1.82865
ENSG0000015	-	-	ENSG0000015130	'SRFBP1	-	5:121961960-122078360	-	-	IGV_LINK	213.339	4.89274	0	11.3115	OK	202.12	4.84767	0	31.5061	OK	OK	-0.01335
ENSG0000015	-	-	ENSG0000015132	'AKAP6	-	14:32329272-32837681	-	-	IGV_LINK	170.671	1.68998	0	5.8733	OK	81.4668	0.490648	0	1.71248	OK	OK	-1.78424
ENSG0000015	-	-	ENSG0000015132	'NPAS3	-	14:32934932-33804176	-	-	IGV_LINK	13.5761	0.312618	0	1.3445	OK	4.1249	0.208399	0	0.991698	OK	NOTEST	-0.58506
ENSG0000015	-	-	ENSG0000015132	'FAM177A1	-	14:35044906-35317493	-	-	IGV_LINK	319.038	22.2088	0	64.3896	OK	379.491	26.4838	0	70.2788	OK	OK	0.253979
ENSG0000015	-	-	ENSG0000015133	'MBIP	-	14:36298557-36320676	-	-	IGV_LINK	166.792	8.65653	0.792462	16.5206	OK	163.965	8.43788	1.02383	15.8519	OK	OK	-0.03691
ENSG0000015	-	-	ENSG0000015133	'MIPOL1	-	14:37197912-37552361	-	-	IGV_LINK	63.0318	1.16553	0	2.89283	OK	43.3114	0.841111	0	2.14353	OK	OK	-0.47062
ENSG0000015	-	-	ENSG0000015134	'EXT2	-	11:44095548-44245429	-	-	IGV_LINK	1041.48	19.2348	8.06375	30.4058	OK	1413.81	26.5664	11.4029	41.7299	OK	OK	0.465884
ENSG0000015	-	-	ENSG0000015135	'TMEM18	-	2:667334-697371	-	-	IGV_LINK	585.711	21.7798	4.03251	39.5272	OK	585.736	27.5946	5.231	49.9583	OK	OK	0.341395
ENSG0000015	-	-	ENSG0000015136	'ALLC	-	2:3658194-3847411	-	-	IGV_LINK	7.75776	0.287581	0	0.716931	OK	9.28102	0.340538	0	0.888907	OK	NOTEST	0.243849
ENSG0000015	-	-	ENSG0000015136	'KCTD14	-	11:78015714-78080219	-	-	IGV_LINK	121.196	4.56518	0	13.051	OK	119.299	4.43209	0	12.3026	OK	OK	-0.04268
ENSG0000015	-	-	ENSG0000015136	'THRSP	-	11:78015714-78080219	-	-	IGV_LINK	1.93944	0.109137	0	1.45498	OK	3.09367	0.174289	0	1.94152	OK	NOTEST	0.675339
ENSG0000015	-	-	ENSG0000015136	'NDUFC2	-	11:78015714-78080219	-	-	IGV_LINK	693.216	77.9484	16.9587	138.938	OK	747.939	83.2193	21.6829	144.756	OK	OK	0.094398
ENSG0000015	-	-	ENSG0000015137	'ME3	-	11:86431589-86672636	-	-	IGV_LINK	490.679	15.089	3.35383	26.8242	OK	549.643	16.3965	5.11152	27.6814	OK	OK	0.119887
ENSG0000015	-	-	ENSG0000015137	'MSGN1	-	2:17816495-17817101	-	-	IGV_LINK	0	0	0	0	OK	0	0	0	0	OK	NOTEST	0
ENSG0000015	-	-	ENSG0000015138	'ADAMTS12	-	5:33523534-33892192	-	-	IGV_LINK	4.8486	0.031767	0	0.28478	OK	67.0296	0.470536	0	1.0432	OK	NOTEST	3.8887
ENSG0000015	-	-	ENSG0000015141	'NUBPL	-	14:31489955-31861224	-	-	IGV_LINK	160.974	4.79545	0	10.8912	OK	144.371	4.7903	0	10.5434	OK	OK	-0.00155
ENSG0000015	-	-	ENSG0000015141	'NEK7	-	1:198156962-198322420	-	-	IGV_LINK	727.29	15.8746	3.32278	28.4264	OK	610.485	11.7427	2.31949	21.1659	OK	OK	-0.43496
ENSG0000015	-	-	ENSG0000015141	'ATP6V1G3	-	1:198523221-198540945	-	-	IGV_LINK	0	0	0	0	OK	0	0	0	0	OK	NOTEST	0
ENSG0000015	-	-	ENSG0000015142	'FER	-	5:108747821-109196841	-	-	IGV_LINK	265.703	2.09858	0	5.7664	OK	220.682	1.59249	0	4.69227	OK	OK	-0.39813
ENSG0000015	-	-	ENSG0000015144	'VIPAS39	-	14:77426674-77469474	-	-	IGV_LINK	464.496	12.1723	0	25.6113	OK	410.428	10.1988	0	21.0175	OK	OK	-0.2552
ENSG0000015	-	-	ENSG0000015145	'ANKRD50	-	4:124664051-124712732	-	-	IGV_LINK	239.521	1.84172	0.459861	3.22358	OK	245.432	1.84452	0.458941	3.2301	OK	OK	0.002195
ENSG0000015	-	-	ENSG0000015146	'UPF2	-	10:11920021-12043170	-	-	IGV_LINK	943.538	10.5913	4.38942	16.7932	OK	509.425	5.70416	2.19437	9.21396	OK	OK	-0.89279
ENSG0000015	-	-	ENSG0000015146	'CDC123	-	10:12129636-12250589	-	-	IGV_LINK	559.529	26.4813	1.16956	51.7931	OK	493.957	23.7894	0.369912	47.209	OK	OK	-0.15466
ENSG0000015	-	-	ENSG0000015146	'SCLT1	-	4:128809622-129116640	-	-	IGV_LINK	206.55	4.90874	0	13.092	OK	203.151	4.5051	0	11.3495	OK	OK	-0.12379
ENSG0000015	-	-	ENSG0000015146	'CCDC3	-	10:12896624-13138308	-	-	IGV_LINK	72.729	1.56795	0	7.81203	OK	135.09	2.93608	0	9.58195	OK	OK	0.905011
ENSG0000015	-	-	ENSG0000015147	'C4orf33	-	4:128809622-129116640	-	-	IGV_LINK	236.612	10.2566	0	24.8309	OK	253.681	12.7025	0	33.025	OK	OK	0.308562
ENSG0000015	-	-	ENSG0000015147	'FRMD4A	-	10:13586926-14462142	-	-	IGV_LINK	666.198	9.43299	0	23.6858	OK	430.021	7.30861	0	19.4808	OK	OK	-0.36812
ENSG0000015	-	-	ENSG0000015147	'SLC25A31	-	4:127730377-127774299	-	-	IGV_LINK	0.969721	0.032754	0	0.128289	OK	1.03122	0.034855	0	0.130456	OK	NOTEST	0.089713
ENSG0000015	-	-	ENSG0000015149	'PTPRO	-	12:15107782-15597399	-	-	IGV_LINK	425.707	8.43876	0	21.1849	OK	232.026	4.95802	0	15.3018	OK	OK	-0.76727
ENSG0000015	-	-	ENSG0000015149	'EPS8	-	12:15620157-15882329	-	-	IGV_LINK	2180.9	53.345	8.70527	97.9847	OK	3022.52	71.3792	13.6913	129.067	OK	OK	0.420151
ENSG0000015	-	-	ENSG0000015149	'ACAD8	-	11:134253494-134265855	-	-	IGV_LINK	645.834	16.8809	4.52105	29.2407	OK	649.672	17.465	5.35735	29.5727	OK	OK	0.04908
ENSG0000015	-	-	ENSG0000015150	'THYN1	-	11:134248278-134253370	-	-	IGV_LINK	484.86	34.7439	11.5199	57.9679	OK	470.239	34.7933	12.5393	57.0472	OK	OK	0.002049
ENSG0000015	-	-	ENSG0000015150	'VPS26B	-	11:134068924-134247792	-	-	IGV_LINK	1083.18	25.7027	0	52.2503	OK	1046.69	21.6839	0.909434	42.4585	OK	OK	-0.24529
ENSG0000015	-	-	ENSG0000015150	'NCAPD3	-	11:134068924-134247792	-	-	IGV_LINK	516.861	11.5257	0	38.4362	OK	390.834	7.93418	0	26.1598	OK	OK	-0.5387
ENSG0000015	-	-	ENSG0000015153	'VTI1A	-	10:112446997-112818744	-	-	IGV_LINK	318.068	7.91744	0	16.3128	OK	286.681	7.13284	0	15.7923	OK	OK	-0.15056
ENSG0000015	-	-	ENSG0000015155	'QDPR	-	4:17460260-17512234	-	-	IGV_LINK	378.191	17.1731	4.41285	29.9334	OK	631.11	27.3955	10.1457	44.6453	OK	OK	0.673788
ENSG0000015	-	-	ENSG0000015155	'FAM160B1	-	10:114821743-114899832	-	-	IGV_LINK	454.799	5.12036	1.74672	8.49401	OK	329.992	3.77538	0	7.64088	OK	OK	-0.43963
ENSG0000015	-	-	ENSG0000015157	'ANO4	-	12:100717525-101128641	-	-	IGV_LINK	201.702	4.64417	0.262551	9.02578	OK	12.3747	0.354541	0	1.1919	OK	OK	-3.7114
ENSG0000015	-	-	ENSG0000015157	'TEX9	-	15:56244008-56465137	-	-	IGV_LINK	191.035	7.88763	0	17.1598	OK	187.683	7.86763	0.419104	15.3161	OK	OK	-0.00366
ENSG0000015	-	-	ENSG0000015157	'QTRT2	-	3:113947900-114088422	-	-	IGV_LINK	377.221	6.45801	0	14.3444	OK	521.8	10.072	0	23.742	OK	OK	0.641184
ENSG0000015	-	-	ENSG0000015157	'DRD3	-	3:114128651-114199407	-	-	IGV_LINK	0	0	0	0	OK	0	0	0	0	OK	NOTEST	0
ENSG0000015	-	-	ENSG0000015161	'MMAA	-	4:145618262-145660035	-	-	IGV_LINK	308.371	8.12415	0.134754	16.1136	OK	213.464	5.89277	0	12.0336	OK	OK	-0.46327
ENSG0000015	-	-	ENSG0000015161	'ZNF827	-	4:145680114-145938635	-	-	IGV_LINK	617.712	8.28466	0	18.232	OK	722.889	9.33513	0	20.8577	OK	OK	0.172228
ENSG0000015	-	-	ENSG0000015161	'POU4F2	-	4:146638892-146642474	-	-	IGV_LINK	0	0	0	0	OK	0	0	0	0	OK	NOTEST	0
ENSG0000015	-	-	ENSG0000015161	'EDNRA	-	4:147480916-147544954	-	-	IGV_LINK	267.643	4.61272	0.777461	8.44799	OK	197.995	3.69491	0.517983	6.87185	OK	OK	-0.32008
ENSG0000015	-	-	ENSG0000015162	'NR3C2	-	4:148078761-148444698	-	-	IGV_LINK	131.882	1.78404	0	4.15303	OK	82.498	2.03731	0	5.96887	OK	OK	0.191513
ENSG0000015	-	-	ENSG0000015163	'AKR1C6P	-	10:4871900-4891975	-	-	IGV_LINK	0	0	0	0	OK	0	0	0	0	OK	NOTEST	0
ENSG0000015	-	-	ENSG0000015163	'AKR1C2	-	10:4987399-5018031	-	-	IGV_LINK	24.243	0.960162	0	2.64634	OK	11.3435	0.644657	0	2.00976	OK	OK	-0.57475
ENSG0000015	-	-	ENSG0000015164	'DPYSL4	-	10:132186899-132205776	-	-	IGV_LINK	0	0	0	0	OK	3.09367	0.232422	0	0.769061	OK	NOTEST	inf
ENSG0000015	-	-	ENSG0000015165	'VENTX	-	10:133237403-133241929	-	-	IGV_LINK	227.884	4.68966	1.35203	8.02728	OK	269.15	5.54119	1.69417	9.38821	OK	OK	0.240713
ENSG0000015	-	-	ENSG0000015165	'ADAM8	-	10:133262402-133276868	-	-	IGV_LINK	395.646	10.019	0	20.0712	OK	655.859	17.0676	0.258888	33.8762	OK	OK	0.768523
ENSG0000015	-	-	ENSG0000015165	'ITIH2	-	10:7703268-7749520	-	-	IGV_LINK	2.90916	0.132329	0	0.59117	OK	1.03122	0.019248	0	0.387051	OK	NOTEST	-2.78137
ENSG0000015	-	-	ENSG0000015165	'KIN	-	10:7750961-7787981	-	-	IGV_LINK	267.643	6.65678	0	14.9952	OK	192.839	5.51135	0	12.7446	OK	OK	-0.27242
ENSG0000015	-	-	ENSG0000015166	'PIGF	-	2:46490749-46625742	-	-	IGV_LINK	333.584	20.0642	0	44.7456	OK	416.615	22.7413	0	50.7409	OK	OK	0.180693
ENSG0000015	-	-	ENSG0000015168	'ANKAR	-	2:189674289-189765556	-	-	IGV_LINK	99.8812	3.63102	0	10.8806	OK	70.1233	2.99199	0	8.98015	OK	OK	-0.27927
ENSG0000015	-	-	ENSG0000015168	'INPP1	-	2:189879608-190371665	-	-	IGV_LINK	320.008	10.8987	0.240103	21.5572	OK	431.052	14.5529	1.61979	27.486	OK	OK	0.417156
ENSG0000015	-	-	ENSG0000015169	'MFSD6	-	2:190408354-190534722	-	-	IGV_LINK	418.919	7.13326	0	16.1068	OK	338.242	6.12006	0	13.8905	OK	OK	-0.22102
ENSG0000015	-	-	ENSG0000015169	'RNF144A	-	2:6912276-7077880	-	-	IGV_LINK	217.217	2.51887	0	5.43291	OK	124.778	1.26264	0	2.56828	OK	OK	-0.99633
ENSG0000015	-	-	ENSG0000015169	'ASAP2	-	2:9206764-9423547	-	-	IGV_LINK	207.52	4.90631	0	16.713	OK	234.088	5.4381	0	18.0549	OK	OK	0.148466
ENSG0000015	-	-	ENSG0000015169	'ADAM17	-	2:9473657-9555792	-	-	IGV_LINK	541.104	7.39413	0.914102	13.8742	OK	690.921	9.59202	0.984334	18.1997	OK	OK	0.375455
ENSG0000015	-	-	ENSG0000015170	'FLI1	-	11:128629652-128813267	-	-	IGV_LINK	862.082	20.8698	2.07512	39.6645	OK	1005.44	23.7407	2.35147	45.13	OK	OK	0.185946
ENSG0000015	-	-	ENSG0000015170	'KCNJ1	-	11:128836314-128867373	-	-	IGV_LINK	19.3944	0.440733	0	1.04549	OK	9.28102	0.310081	0	0.877189	OK	NOTEST	-0.50726
ENSG0000015	-	-	ENSG0000015171	'TMEM45B	-	11:129815818-129860003	-	-	IGV_LINK	105.7	3.52667	0.180152	6.87319	OK	240.275	8.37678	1.75659	14.997	OK	OK	1.24809
ENSG0000015	-	-	ENSG0000015171	'WWC2	-	4:183099292-183320777	-	-	IGV_LINK	859.172	12.7893	1.14543	24.4333	OK	679.577	9.82322	1.05258	18.5939	OK	OK	-0.38067
ENSG0000015	-	-	ENSG0000015172	'CENPU	-	4:184649612-184734133	-	-	IGV_LINK	107.639	5.57754	0	12.821	OK	201.089	7.40472	0.582059	14.2274	OK	OK	0.408816
ENSG0000015	-	-	ENSG0000015172	'ACSL1	-	4:184755594-184826818	-	-	IGV_LINK	3372.69	58.7173	24.0311	93.4035	OK	11522.9	207.793	38.3362	377.25	OK	OK	1.82329
ENSG0000015	-	-	ENSG0000015172	'SLC25A4	-	4:185143240-185150382	-	-	IGV_LINK	726.321	20.182	7.1071	33.2569	OK	893.041	25.6424	9.54929	41.7356	OK	OK	0.345463
ENSG0000015	-	-	ENSG0000015174	'AMN1	-	12:31647159-31731204	-	-	IGV_LINK	83.396	4.23596	0	10.7356	OK	64.9672	2.85133	0	7.3789	OK	OK	-0.57106
ENSG0000015	-	-	ENSG0000015174	'BICD1	-	12:32104116-32383633	-	-	IGV_LINK	806.807	12.7902	0	26.1637	OK	464.051	9.4973	0	19.3335	OK	OK	-0.42944
ENSG0000015	-	-	ENSG0000015174	'SAV1	-	14:50418500-50668331	-	-	IGV_LINK	274.431	5.75392	0	12.7703	OK	256.775	5.92555	0	15.2587	OK	OK	0.042403
ENSG0000015	-	-	ENSG0000015177	'CCDC122	-	13:43823908-44030461	-	-	IGV_LINK	66.9107	3.19627	0	9.74219	OK	31.968	1.92243	0	8.29892	OK	OK	-0.73346
ENSG0000015	-	-	ENSG0000015177	'SERP2	-	13:44373664-44397714	-	-	IGV_LINK	78.5474	6.05399	0.052335	12.0557	OK	81.4668	6.48067	0.481247	12.4801	OK	OK	0.098257
ENSG0000015	-	-	ENSG0000015177	'NBAS	-	2:15166908-15561330	-	-	IGV_LINK	997.842	13.9107	1.37332	26.4481	OK	781.669	10.2685	1.41501	19.122	OK	OK	-0.43798
ENSG0000015	-	-	ENSG0000015178	'ZNF385D	-	3:21412221-22373321	-	-	IGV_LINK	41.698	1.55741	0	4.73187	OK	397.022	18.9891	0	38.977	OK	OK	3.60796
ENSG0000015	-	-	ENSG0000015179	'TDO2	-	4:155829728-155920406	-	-	IGV_LINK	2.90916	0.26188	0	1.14505	OK	20.6245	0.717071	0	1.82441	OK	OK	1.45321
ENSG0000015	-	-	ENSG0000015180	'GUF1	-	4:44622064-44726595	-	-	IGV_LINK	423.768	7.9986	1.04472	14.9525	OK	491.894	9.07255	1.30876	16.8364	OK	OK	0.181762
ENSG0000015	-	-	ENSG0000015181	'SLC35F4	-	14:57563921-57982194	-	-	IGV_LINK	0.969721	0.038067	0	0.572247	OK	0	0	0	0	OK	NOTEST	'-inf
ENSG0000015	-	-	ENSG0000015183	'GABRA2	-	4:46248426-46475230	-	-	IGV_LINK	0	0	0	0	OK	0	0	0	0	OK	NOTEST	0
ENSG0000015	-	-	ENSG0000015183	'SACS	-	13:23328825-23411513	-	-	IGV_LINK	505.224	1.88875	0.635738	3.14176	OK	353.71	1.32657	0.391158	2.26197	OK	OK	-0.50974
ENSG0000015	-	-	ENSG0000015183	'CCDC175	-	14:59429021-59576831	-	-	IGV_LINK	1.93944	0.044731	0	0.73493	OK	1.03122	0.025122	0	0.63017	OK	NOTEST	-0.83232
ENSG0000015	-	-	ENSG0000015184	'PABPC3	-	13:25095867-25099254	-	-	IGV_LINK	0	0	0	0	OK	1.03122	0.018086	0	0.067691	OK	NOTEST	inf
ENSG0000015	-	-	ENSG0000015184	'CENPJ	-	13:24882283-24922889	-	-	IGV_LINK	192.005	3.70128	0	8.38873	OK	1264.28	24.2805	0.878334	47.6828	OK	OK	2.7137
ENSG0000015	-	-	ENSG0000015187	'FBXO4	-	5:41925253-41941743	-	-	IGV_LINK	153.216	7.55429	1.02056	14.088	OK	173.246	8.04836	1.31427	14.7824	OK	OK	0.091399
ENSG0000015	-	-	ENSG0000015188	'TMEM267	-	5:43444251-43483893	-	-	IGV_LINK	125.094	7.07516	0	15.6498	OK	96.9351	5.91517	0	12.5588	OK	OK	-0.25834
ENSG0000015	-	-	ENSG0000015188	'CCL28	-	5:43376644-43412391	-	-	IGV_LINK	736.018	20.4956	6.68478	34.3063	OK	424.865	12.0553	3.11429	20.9963	OK	OK	-0.76564
ENSG0000015	-	-	ENSG0000015188	'PARP8	-	5:50665898-50846522	-	-	IGV_LINK	1177.24	31.2582	3.24399	59.2724	OK	1080.72	25.3372	4.65269	46.0217	OK	OK	-0.30298
ENSG0000015	-	-	ENSG0000015189	'GFRA1	-	10:116056924-116273467	-	-	IGV_LINK	76.6079	0.483101	0	1.2479	OK	789.918	5.73938	0	12.3394	OK	OK	3.5705
ENSG0000015	-	-	ENSG0000015189	'CACUL1	-	10:118674166-118755249	-	-	IGV_LINK	681.714	7.68313	0	21.245	OK	611.516	6.76828	0	16.2053	OK	OK	-0.18291
ENSG0000015	-	-	ENSG0000015191	'DST	-	6:56457986-56954628	-	-	IGV_LINK	2289.51	36.3141	0	79.0233	OK	1756.18	31.9872	0	68.3205	OK	OK	-0.18304
ENSG0000015	-	-	ENSG0000015191	'BEND6	-	6:56955125-57027342	-	-	IGV_LINK	32.0008	0.717383	0	1.5953	OK	31.968	1.31418	0	3.48891	OK	OK	0.873344
ENSG0000015	-	-	ENSG0000015192	'TIAL1	-	10:119571801-119597029	-	-	IGV_LINK	1654.34	58.0377	12.003	104.072	OK	989.976	33.6899	7.72074	59.6591	OK	OK	-0.78467
ENSG0000015	-	-	ENSG0000015192	'BAG3	-	10:119651369-119677819	-	-	IGV_LINK	425.707	10.2524	3.66431	16.8404	OK	540.362	13.4037	4.73478	22.0726	OK	OK	0.386673
ENSG0000015	-	-	ENSG0000015194	'GLT1D1	-	12:128853426-128984968	-	-	IGV_LINK	16.4852	0.558322	0	1.28654	OK	23.7182	0.480002	0	1.03951	OK	NOTEST	-0.21806
ENSG0000015	-	-	ENSG0000015195	'TMEM132D	-	12:129071724-129903666	-	-	IGV_LINK	4.8486	0.048764	0	0.121702	OK	14.4371	0.145228	0	0.322534	OK	NOTEST	1.57444
ENSG0000015	-	-	ENSG0000015196	'RBM46	-	4:154754755-154828813	-	-	IGV_LINK	21.3339	0.438299	0	1.10832	OK	27.8431	0.61043	0	1.38828	OK	OK	0.477912
ENSG0000015	-	-	ENSG0000015196	'ZNF37CP	-	10:37883593-37884109	-	-	IGV_LINK	7.75776	5.7311	0	13.265	OK	10.3122	7.71853	0	17.3437	OK	OK	0.429513
ENSG0000015	-	-	ENSG0000015196	'SCHIP1	-	3:158962234-159897366	-	-	IGV_LINK	94.9814	4.23962	0	18.024	OK	73.7993	2.62993	0	7.44219	OK	OK	-0.68891
ENSG0000015	-	-	ENSG0000015203	'MCHR2	-	6:99919909-100076413	-	-	IGV_LINK	0	0	0	0	OK	0	0	0	0	OK	NOTEST	0
ENSG0000015	-	-	ENSG0000015204	'KCNE4	-	2:223051813-223198399	-	-	IGV_LINK	326.796	5.90568	1.90426	9.90709	OK	98.9976	1.85019	0.078329	3.62205	OK	OK	-1.67443
ENSG0000015	-	-	ENSG0000015205	'AP1S3	-	2:223751685-223838027	-	-	IGV_LINK	168.731	8.34581	0	17.7624	OK	87.6541	4.33533	0	9.46464	OK	OK	-0.94491
ENSG0000015	-	-	ENSG0000015206	'RABGAP1L	-	1:174115299-174995308	-	-	IGV_LINK	786.443	14.615	2.04661	27.1833	OK	521.8	11.7294	0.079903	23.3788	OK	OK	-0.31732
ENSG0000015	-	-	ENSG0000015207	'CCDC74B	-	2:130129620-130145134	-	-	IGV_LINK	565.347	20.2769	4.90926	35.6445	OK	525.925	18.7862	5.0073	32.5651	OK	OK	-0.11016
ENSG0000015	-	-	ENSG0000015207	'TMEM56	-	1:95117337-95278940	-	-	IGV_LINK	33.5941	0.351183	0	1.38722	OK	24.0315	0.203706	0	0.841904	OK	NOTEST	-0.78574
ENSG0000015	-	-	ENSG0000015208	'MZT2B	-	2:130151391-130190729	-	-	IGV_LINK	1392.52	157.536	15.2521	299.82	OK	1102.38	128.671	0	275.789	OK	OK	-0.29199
ENSG0000015	-	-	ENSG0000015208	'TUBA3E	-	2:130191742-130198461	-	-	IGV_LINK	0.969721	0.039905	0	0.156298	OK	9.28102	0.382246	0	0.86255	OK	NOTEST	3.25986
ENSG0000015	-	-	ENSG0000015209	'ASTN1	-	1:176857301-177164973	-	-	IGV_LINK	0	0	0	0	OK	1.03122	0.020627	0	0.115045	OK	NOTEST	inf
ENSG0000015	-	-	ENSG0000015209	'CFC1B	-	2:130515996-130528604	-	-	IGV_LINK	0	0	0	0	OK	0	0	0	0	OK	NOTEST	0
ENSG0000015	-	-	ENSG0000015210	'FAM168B	-	2:131047875-131093460	-	-	IGV_LINK	1822.1	23.6714	6.49944	40.8433	OK	1892.3	25.2878	6.6128	43.9627	OK	OK	0.095297
ENSG0000015	-	-	ENSG0000015210	'PTPN14	-	1:214344171-214552449	-	-	IGV_LINK	1222.82	5.46104	2.29353	8.62855	OK	1164.25	5.39122	0.672946	10.1095	OK	OK	-0.01856
ENSG0000015	-	-	ENSG0000015211	'AC073869.1	-	2:131492812-131521573	-	-	IGV_LINK	415.04	6.57859	2.4001	10.7571	OK	441.364	6.88757	2.47379	11.3014	OK	OK	0.066218
ENSG0000015	-	-	ENSG0000015212	'MGAT5	-	2:134119982-134454621	-	-	IGV_LINK	1436.16	11.8501	0	25.4198	OK	1432.37	11.6323	0	25.2043	OK	OK	-0.02676
ENSG0000015	-	-	ENSG0000015212	'TMEM163	-	2:134455758-134719000	-	-	IGV_LINK	28.1219	0.891611	0	1.84446	OK	74.2482	2.07957	0	4.30046	OK	OK	1.2218
ENSG0000015	-	-	ENSG0000015213	'GPATCH11	-	2:37084450-37157065	-	-	IGV_LINK	276.37	4.82706	0	13.3445	OK	292.868	4.69208	0	12.3432	OK	OK	-0.04092
ENSG0000015	-	-	ENSG0000015213	'HSPB8	-	12:119178641-119221131	-	-	IGV_LINK	3881.79	111.485	44.6368	178.333	OK	6593.65	188.754	67.0739	310.435	OK	OK	0.759661
ENSG0000015	-	-	ENSG0000015214	'GEMIN6	-	2:38751533-38785000	-	-	IGV_LINK	236.612	10.1541	1.00885	19.2993	OK	250.588	12.0308	1.63081	22.4309	OK	OK	0.244679
ENSG0000015	-	-	ENSG0000015215	'TMEM178A	-	2:39249265-39717963	-	-	IGV_LINK	47.5163	2.45654	0	7.79359	OK	40.2178	2.08065	0	6.83531	OK	OK	-0.23959

ENSG0000015	-	-	ENSG0000015219	'POU4F1	-	13:77895480-78659179	-	-	IGV_LINK	0	0	0	0	OK	2.06245	0.026577	0	0.35053	OK	NOTEST	inf
ENSG0000015	-	-	ENSG0000015219	'RNF219	-	13:77895480-78659179	-	-	IGV_LINK	333.584	5.60089	0	11.9806	OK	343.398	5.76764	0	11.5649	OK	OK	0.042326
ENSG0000015	-	-	ENSG0000015220	'CYSLTR2	-	13:48653710-48711226	-	-	IGV_LINK	179.398	2.71647	0	5.48789	OK	64.9672	0.859164	0	1.84716	OK	OK	-1.66073
ENSG0000015	-	-	ENSG0000015220	'GRID2	-	4:92297250-93810157	-	-	IGV_LINK	0	0	0	0	OK	0	0	0	0	OK	NOTEST	0
ENSG0000015	-	-	ENSG0000015221	'ARL11	-	13:49628298-49633872	-	-	IGV_LINK	107.639	2.64724	0	11.7412	OK	137.153	4.22044	0	18.273	OK	OK	0.672906
ENSG0000015	-	-	ENSG0000015221	'RIT2	-	18:42743226-43115691	-	-	IGV_LINK	0	0	0	0	OK	0	0	0	0	OK	NOTEST	0
ENSG0000015	-	-	ENSG0000015221	'SETBP1	-	18:44680172-45068510	-	-	IGV_LINK	519.77	3.2316	0.955378	5.50782	OK	177.371	1.02781	0.222558	1.83306	OK	OK	-1.65267
ENSG0000015	-	-	ENSG0000015221	'ARL14EP	-	11:30323050-30338227	-	-	IGV_LINK	303.523	10.7014	0	28.2651	OK	248.525	7.70422	0	18.6673	OK	OK	-0.47408
ENSG0000015	-	-	ENSG0000015222	'EPG5	-	18:45847608-45967274	-	-	IGV_LINK	953.235	9.52809	0	21.7898	OK	683.702	6.22141	0.356377	12.0864	OK	OK	-0.61495
ENSG0000015	-	-	ENSG0000015222	'PSTPIP2	-	18:45983535-46072272	-	-	IGV_LINK	376.252	9.53999	0.745133	18.3348	OK	301.118	8.01287	0.159717	15.866	OK	OK	-0.25167
ENSG0000015	-	-	ENSG0000015223	'ATP5A1	-	18:46080247-46128333	-	-	IGV_LINK	6398.22	210.565	72.0515	349.079	OK	5817.14	191.572	66.1892	316.956	OK	OK	-0.13638
ENSG0000015	-	-	ENSG0000015224	'HAUS1	-	18:46080247-46128333	-	-	IGV_LINK	199.762	16.5577	0	90.0506	OK	227.901	17.6158	0	85.3848	OK	OK	0.089362
ENSG0000015	-	-	ENSG0000015224	'C18orf25	-	18:46173552-46266991	-	-	IGV_LINK	585.711	7.65726	0.594592	14.7199	OK	547.58	8.53245	0	17.645	OK	OK	0.156132
ENSG0000015	-	-	ENSG0000015225	'SPC25	-	2:168455861-168913371	-	-	IGV_LINK	29.0916	1.38765	0	6.46379	OK	36.0929	1.72328	0	7.25853	OK	OK	0.312516
ENSG0000015	-	-	ENSG0000015225	'G6PC2	-	2:168455861-168913371	-	-	IGV_LINK	20.3641	0.594352	0	3.48063	OK	9.28102	0.258892	0	2.11209	OK	OK	-1.19897
ENSG0000015	-	-	ENSG0000015225	'PDK1	-	2:172427353-172608669	-	-	IGV_LINK	962.932	16.0257	0	44.0885	OK	1123	19.0529	0	49.336	OK	OK	0.249624
ENSG0000015	-	-	ENSG0000015226	'PTH	-	11:13492054-13496181	-	-	IGV_LINK	0	0	0	0	OK	1.03122	0.092888	0	0.40429	OK	NOTEST	inf
ENSG0000015	-	-	ENSG0000015227	'PDE3B	-	11:14643722-14872044	-	-	IGV_LINK	169.701	2.14124	0	4.58739	OK	210.37	2.3712	0.499676	4.24272	OK	OK	0.147169
ENSG0000015	-	-	ENSG0000015228	'TCF7L1	-	2:85133409-85310388	-	-	IGV_LINK	132.852	3.52462	0	7.44998	OK	126.841	3.07746	0	6.24604	OK	OK	-0.19573
ENSG0000015	-	-	ENSG0000015229	'TGOLN2	-	2:85318019-85328425	-	-	IGV_LINK	8150.5	82.133	23.7816	140.484	OK	10530.9	104.2	25.9493	182.451	OK	OK	0.343326
ENSG0000015	-	-	ENSG0000015229	'SH2D6	-	2:85418720-85437029	-	-	IGV_LINK	0.969721	0.142508	0	0.740792	OK	4.1249	0.32128	0	1.02956	OK	NOTEST	1.17279
ENSG0000015	-	-	ENSG0000015231	'KCNK13	-	14:90061764-90185857	-	-	IGV_LINK	62.0621	1.48865	0	3.04122	OK	56.7174	1.36111	0	2.77744	OK	OK	-0.12922
ENSG0000015	-	-	ENSG0000015233	'UHMK1	-	1:162497250-162529629	-	-	IGV_LINK	1317.85	8.97014	3.98297	13.9573	OK	1256.03	8.64239	3.76459	13.5202	OK	OK	-0.0537
ENSG0000015	-	-	ENSG0000015234	'ATG10	-	5:81972024-82278577	-	-	IGV_LINK	156.125	8.27404	0	74.0332	OK	125.809	6.72492	0	54.6636	OK	OK	-0.29908
ENSG0000015	-	-	ENSG0000015235	'POC5	-	5:75674123-75717481	-	-	IGV_LINK	147.398	5.231	0	10.8189	OK	166.027	5.10853	0.497979	9.71908	OK	OK	-0.03418
ENSG0000015	-	-	ENSG0000015237	'SPOCK1	-	5:136975297-137598379	-	-	IGV_LINK	140.609	3.61549	0	9.30255	OK	1249.84	27.3124	0	57.8684	OK	OK	2.91729
ENSG0000015	-	-	ENSG0000015238	'FAM151B	-	5:80482292-80542563	-	-	IGV_LINK	11.6366	0.766534	0	2.21766	OK	21.6557	1.47362	0	3.68225	OK	OK	0.942945
ENSG0000015	-	-	ENSG0000015238	'TADA1	-	1:166856509-166876327	-	-	IGV_LINK	217.217	6.00883	1.34423	10.6734	OK	176.339	4.95912	1.17649	8.74175	OK	OK	-0.277
ENSG0000015	-	-	ENSG0000015240	'GUCY1A2	-	11:106674011-107018524	-	-	IGV_LINK	61.0924	0.462941	0	1.131	OK	81.4668	0.618152	0	1.44598	OK	OK	0.417134
ENSG0000015	-	-	ENSG0000015240	'CWF19L2	-	11:107326344-107457844	-	-	IGV_LINK	328.735	6.19198	2.04986	10.3341	OK	296.993	5.97686	1.69015	10.2636	OK	OK	-0.05101
ENSG0000015	-	-	ENSG0000015240	'JMY	-	5:79236188-79327215	-	-	IGV_LINK	160.974	1.02288	0.088708	1.95705	OK	157.777	1.00285	0.0851	1.9206	OK	OK	-0.02853
ENSG0000015	-	-	ENSG0000015241	'HOMER1	-	5:79372635-79514217	-	-	IGV_LINK	20.3641	0.72732	0	1.97941	OK	29.9055	0.358012	0	1.01322	OK	OK	-1.02258
ENSG0000015	-	-	ENSG0000015242	'XRCC4	-	5:83052845-83353787	-	-	IGV_LINK	184.247	7.96333	0	18.8774	OK	238.213	10.5036	0	26.1139	OK	OK	0.39944
ENSG0000015	-	-	ENSG0000015243	'BOLL	-	2:197693105-197786762	-	-	IGV_LINK	0.969721	0.021263	0	0.417705	OK	0	0	0	0	OK	NOTEST	'-inf
ENSG0000015	-	-	ENSG0000015243	'ZNF547	-	19:57363476-57477570	-	-	IGV_LINK	46.5537	2.35744	0	10.303	OK	48.9342	1.773	0	7.55722	OK	OK	-0.41103
ENSG0000015	-	-	ENSG0000015243	'ZNF773	-	19:57487710-57518404	-	-	IGV_LINK	81.4565	3.16749	0	8.95237	OK	35.0616	1.44065	0	5.44587	OK	OK	-1.13662
ENSG0000015	-	-	ENSG0000015244	'ZNF776	-	19:57681968-57758159	-	-	IGV_LINK	512.433	7.25106	0.098617	14.4035	OK	488.443	7.99671	0	18.7006	OK	OK	0.141215
ENSG0000015	-	-	ENSG0000015245	'ZNF256	-	19:57940832-57947675	-	-	IGV_LINK	72.729	2.14351	0	4.42154	OK	79.4043	2.23932	0.176942	4.30169	OK	OK	0.063087
ENSG0000015	-	-	ENSG0000015245	'SUV39H2	-	10:14878819-14954432	-	-	IGV_LINK	54.3043	1.76451	0	4.74946	OK	39.1865	0.79286	0	2.30024	OK	OK	-1.15413
ENSG0000015	-	-	ENSG0000015245	'DCLRE1C	-	10:14878819-14954432	-	-	IGV_LINK	220.127	5.74778	0.560773	10.9348	OK	149.528	4.17234	0	8.37699	OK	OK	-0.46215
ENSG0000015	-	-	ENSG0000015246	'OLAH	-	10:15032226-15073852	-	-	IGV_LINK	0	0	0	0	OK	0	0	0	0	OK	NOTEST	0
ENSG0000015	-	-	ENSG0000015246	'RPP38	-	10:15095384-15168693	-	-	IGV_LINK	128.973	7.09325	0	15.8132	OK	82.498	4.48683	0	10.6707	OK	OK	-0.66075
ENSG0000015	-	-	ENSG0000015246	'NMT2	-	10:15095384-15168693	-	-	IGV_LINK	340.372	10.6656	0.926338	20.4049	OK	269.15	7.60609	0.532146	14.68	OK	OK	-0.48774
ENSG0000015	-	-	ENSG0000015246	'ZSCAN1	-	19:58034031-58054631	-	-	IGV_LINK	0	0	0	0	OK	0	0	0	0	OK	NOTEST	0
ENSG0000015	-	-	ENSG0000015247	'ZNF837	-	19:58367617-58381060	-	-	IGV_LINK	24.243	0.737596	0	1.5383	OK	34.0304	1.02868	0	2.10372	OK	OK	0.479897
ENSG0000015	-	-	ENSG0000015248	'USP12	-	13:27066141-27171896	-	-	IGV_LINK	865.96	11.6299	4.14142	19.1185	OK	1348.84	18.6699	4.2096	33.1302	OK	OK	0.68287
ENSG0000015	-	-	ENSG0000015249	'CCDC50	-	3:191267167-191398670	-	-	IGV_LINK	1730.95	20.6144	3.01698	38.2119	OK	1871.67	24.1652	7.13666	41.1938	OK	OK	0.229277
ENSG0000015	-	-	ENSG0000015249	'CAMK4	-	5:111223652-111494886	-	-	IGV_LINK	128.973	1.61238	0	3.90386	OK	97.9664	0.917791	0	2.70006	OK	OK	-0.81296
ENSG0000015	-	-	ENSG0000015250	'TRIM36	-	5:115124761-115180546	-	-	IGV_LINK	55.2741	1.2847	0	3.01411	OK	44.3427	1.02306	0	2.51419	OK	OK	-0.32854
ENSG0000015	-	-	ENSG0000015251	'ZFP36L2	-	2:43222401-43226609	-	-	IGV_LINK	3786.76	60.5664	25.6628	95.47	OK	2516.19	40.2577	17.6217	62.8938	OK	OK	-0.58925
ENSG0000015	-	-	ENSG0000015252	'PAN3	-	13:28138505-28295335	-	-	IGV_LINK	884.385	11.8535	3.75805	19.9489	OK	714.639	9.22678	3.50409	14.9495	OK	OK	-0.36141
ENSG0000015	-	-	ENSG0000015252	'PLEKHH2	-	2:43637272-43767987	-	-	IGV_LINK	3172.93	37.6026	0	81.626	OK	135.09	1.84115	0	5.13467	OK	OK	-4.35216
ENSG0000015	-	-	ENSG0000015255	'PFKM	-	12:48042897-48146404	-	-	IGV_LINK	1480.76	37.4328	5.15832	69.7073	OK	1354	37.3592	3.47136	71.2471	OK	OK	-0.00284
ENSG0000015	-	-	ENSG0000015255	'TMEM123	-	11:102396331-102498801	-	-	IGV_LINK	3756.7	135.593	0	296.7	OK	6045.04	236.482	0	542.717	OK	OK	0.802447
ENSG0000015	-	-	ENSG0000015257	'GRIA4	-	11:105609993-105982092	-	-	IGV_LINK	0.969721	0.160494	0	0.852372	OK	0	0	0	0	OK	NOTEST	'-inf
ENSG0000015	-	-	ENSG0000015258	'IGSF10	-	3:151085696-151458709	-	-	IGV_LINK	17.455	0.090292	0	0.78205	OK	11.3435	0.147962	0	1.08107	OK	NOTEST	0.712558
ENSG0000015	-	-	ENSG0000015258	'SPEF2	-	5:35617843-35827018	-	-	IGV_LINK	515.891	8.31394	0	17.1534	OK	387.741	6.74442	0	13.7403	OK	OK	-0.30184
ENSG0000015	-	-	ENSG0000015258	'SPARCL1	-	4:87473334-87531061	-	-	IGV_LINK	4378.29	91.2984	34.2957	148.301	OK	4342.49	89.4546	37.02	141.889	OK	OK	-0.02943
ENSG0000015	-	-	ENSG0000015259	'DSPP	-	4:87568034-87733956	-	-	IGV_LINK	0	0	0	0	OK	0	0	0	0	OK	NOTEST	0
ENSG0000015	-	-	ENSG0000015259	'DMP1	-	4:87568034-87733956	-	-	IGV_LINK	0	0	0	0	OK	1.03122	0.023609	0	0.102654	OK	NOTEST	inf
ENSG0000015	-	-	ENSG0000015259	'MEPE	-	4:87821410-87846817	-	-	IGV_LINK	0	0	0	0	OK	0	0	0	0	OK	NOTEST	0
ENSG0000015	-	-	ENSG0000015260	'MBNL1	-	3:152243827-152496813	-	-	IGV_LINK	2459.21	46.0768	1.87564	90.278	OK	1953.14	35.4375	0	71.5499	OK	OK	-0.37877
ENSG0000015	-	-	ENSG0000015261	'CAPSL	-	5:35904294-35938779	-	-	IGV_LINK	1.93944	0.137211	0	0.534437	OK	0	0	0	0	OK	NOTEST	'-inf
ENSG0000015	-	-	ENSG0000015262	'NADK2	-	5:36192591-36242279	-	-	IGV_LINK	352.978	10.6068	2.24067	18.973	OK	306.274	8.95087	2.29527	15.6065	OK	OK	-0.24489
ENSG0000015	-	-	ENSG0000015264	'GPD1L	-	3:32105688-32168713	-	-	IGV_LINK	188.126	2.82358	0.376682	5.27048	OK	178.402	3.10308	0	6.5375	OK	OK	0.136174
ENSG0000015	-	-	ENSG0000015266	'GJA1	-	6:121435691-121449727	-	-	IGV_LINK	961.963	18.9256	8.15791	29.6932	OK	802.293	15.7906	6.61016	24.971	OK	OK	-0.26127
ENSG0000015	-	-	ENSG0000015266	'CCNO	-	5:55231151-55233680	-	-	IGV_LINK	4.8486	0.199292	0	0.49609	OK	17.5308	0.737787	0	1.62183	OK	OK	1.88832
ENSG0000015	-	-	ENSG0000015267	'DDX4	-	5:55625844-55817157	-	-	IGV_LINK	0	0	0	0	OK	0	0	0	0	OK	NOTEST	0
ENSG0000015	-	-	ENSG0000015267	'CLEC4F	-	2:70808642-70820600	-	-	IGV_LINK	127.033	3.10927	0.52741	5.69113	OK	42.2802	1.03539	0	2.0916	OK	OK	-1.58641
ENSG0000015	-	-	ENSG0000015268	'SLC30A6	-	2:32165840-32224379	-	-	IGV_LINK	422.798	5.75019	1.00428	10.4961	OK	504.269	6.6646	2.04664	11.2826	OK	OK	0.212908
ENSG0000015	-	-	ENSG0000015268	'PELO	-	5:52675192-53094779	-	-	IGV_LINK	563.609	14.3176	0	43.5778	OK	640.809	16.1274	0	42.0571	OK	OK	0.171729
ENSG0000015	-	-	ENSG0000015268	'RASGRP3	-	2:33436323-33564750	-	-	IGV_LINK	771.898	15.7603	0	32.1079	OK	804.355	15.5712	0.245688	30.8967	OK	OK	-0.01741
ENSG0000015	-	-	ENSG0000015270	'SAR1B	-	5:134601143-134727823	-	-	IGV_LINK	822.323	28.686	0	66.5241	OK	1454.03	47.069	0	95.7193	OK	OK	0.714428
ENSG0000015	-	-	ENSG0000015270	'CATSPER3	-	5:134904905-135011707	-	-	IGV_LINK	46.9286	2.21409	0	6.61302	OK	36.975	1.74617	0	5.76336	OK	OK	-0.34252
ENSG0000015	-	-	ENSG0000015274	'GPR180	-	13:94601902-94634645	-	-	IGV_LINK	159.034	1.03618	0.193664	1.8787	OK	160.871	1.04829	0.192358	1.90422	OK	OK	0.01676
ENSG0000015	-	-	ENSG0000015276	'TCTEX1D1	-	1:66752458-66778787	-	-	IGV_LINK	9.69721	0.760376	0	2.21801	OK	28.8743	2.88927	0	7.23091	OK	OK	1.92592
ENSG0000015	-	-	ENSG0000015276	'WDR78	-	1:66812884-66924887	-	-	IGV_LINK	217.217	4.61271	0	9.83269	OK	144.371	2.94135	0	6.38432	OK	OK	-0.64914
ENSG0000015	-	-	ENSG0000015276	'ANKRD22	-	10:88822131-88851818	-	-	IGV_LINK	190.065	8.63758	0.952748	16.3224	OK	58.7798	2.32899	0	4.75874	OK	OK	-1.89093
ENSG0000015	-	-	ENSG0000015276	'FARP1	-	13:98142561-98578830	-	-	IGV_LINK	3197.17	65.4285	0	168.754	OK	2371.82	46.362	0	125.002	OK	OK	-0.49698
ENSG0000015	-	-	ENSG0000015277	'IFIT5	-	10:89414585-89421001	-	-	IGV_LINK	606.075	8.88896	3.56927	14.2086	OK	400.115	5.87002	2.12864	9.61139	OK	OK	-0.59865
ENSG0000015	-	-	ENSG0000015277	'SLC16A12	-	10:89430298-89556641	-	-	IGV_LINK	1249	15.8372	6.90918	24.7651	OK	711.545	9.36894	1.75512	16.9828	OK	OK	-0.75736
ENSG0000015	-	-	ENSG0000015278	'PANK1	-	10:89579496-89650822	-	-	IGV_LINK	249.762	6.48084	0	13.3046	OK	225.45	4.90933	0.368651	9.45001	OK	OK	-0.40066
ENSG0000015	-	-	ENSG0000015278	'PRDM8	-	4:80183279-80204329	-	-	IGV_LINK	41.698	0.823507	0	2.28728	OK	803.324	16.5189	5.43919	27.5987	OK	OK	4.3262
ENSG0000015	-	-	ENSG0000015278	'BMP3	-	4:81030964-81057531	-	-	IGV_LINK	3.87888	0.039305	0	0.100104	OK	11.3435	0.114969	0	0.25763	OK	NOTEST	1.54845
ENSG0000015	-	-	ENSG0000015279	'HNRNPDL	-	4:82422563-82430408	-	-	IGV_LINK	5074.55	185.078	65.4189	304.738	OK	4195.02	161.945	60.1193	263.77	OK	OK	-0.19263
ENSG0000015	-	-	ENSG0000015280	'HHEX	-	10:92689950-92695646	-	-	IGV_LINK	186.186	7.81981	1.32762	14.312	OK	223.776	9.41687	1.98585	16.8479	OK	OK	0.268114
ENSG0000015	-	-	ENSG0000015281	'UTRN	-	6:144285700-144853034	-	-	IGV_LINK	2209.02	29.6742	0	70.5015	OK	1219.94	20.6557	0	44.7004	OK	OK	-0.52266
ENSG0000015	-	-	ENSG0000015282	'GRM1	-	6:146027645-146437598	-	-	IGV_LINK	1.93944	0.016943	0	0.133828	OK	3.09367	0.027033	0	0.147687	OK	NOTEST	0.674043
ENSG0000015	-	-	ENSG0000015289	'PTPRK	-	6:127968778-128520674	-	-	IGV_LINK	1986.96	26.4649	2.80402	50.1259	OK	1071.44	13.0142	2.48219	23.5462	OK	OK	-1.024
ENSG0000015	-	-	ENSG0000015290	'GGPS1	-	1:235131182-235344532	-	-	IGV_LINK	384.009	20.1318	0	51.6875	OK	418.677	20.5514	0	49.2664	OK	OK	0.029757
ENSG0000015	-	-	ENSG0000015291	'CNTNAP4	-	16:76277277-76928169	-	-	IGV_LINK	0	0	0	0	OK	0	0	0	0	OK	NOTEST	0
ENSG0000015	-	-	ENSG0000015292	'ZNF117	-	7:64971775-65006743	-	-	IGV_LINK	731.816	8.05784	0.31826	15.7974	OK	477.085	5.16452	0.374969	9.95407	OK	OK	-0.64176
ENSG0000015	-	-	ENSG0000015293	'PART1	-	5:58969037-60547657	-	-	IGV_LINK	0	0	0	0	OK	0	0	0	0	OK	NOTEST	0
ENSG0000015	-	-	ENSG0000015293	'RAB3C	-	5:58582220-58863414	-	-	IGV_LINK	10.6669	0.132035	0	0.649837	OK	18.562	0.557844	0	2.12306	OK	NOTEST	2.07895
ENSG0000015	-	-	ENSG0000015293	'LMNTD1	-	12:25409292-25648579	-	-	IGV_LINK	0.969721	0.194056	0	0.934845	OK	0	0	0	0	OK	NOTEST	'-inf
ENSG0000015	-	-	ENSG0000015293	'MARVELD2	-	5:69415111-69444330	-	-	IGV_LINK	28.1219	0.677687	0	1.54173	OK	18.562	0.709814	0	1.91522	OK	OK	0.066822
ENSG0000015	-	-	ENSG0000015294	'RAD17	-	5:69350983-69414801	-	-	IGV_LINK	496.497	15.1905	0	33.5072	OK	455.801	12.5929	0	28.4241	OK	OK	-0.27056
ENSG0000015	-	-	ENSG0000015294	'MED21	-	12:27022545-27066343	-	-	IGV_LINK	214.308	11.8158	0	23.9571	OK	157.777	8.534	0	17.9007	OK	OK	-0.46942
ENSG0000015	-	-	ENSG0000015295	'PLOD2	-	3:146064041-146163653	-	-	IGV_LINK	2040.29	36.6553	14.2263	59.0843	OK	8302.39	147.866	37.3133	258.419	OK	OK	2.0122
ENSG0000015	-	-	ENSG0000015295	'STK32B	-	4:5051441-5500998	-	-	IGV_LINK	706.926	12.6238	4.74206	20.5056	OK	136.122	2.67047	0	5.38347	OK	OK	-2.24099
ENSG0000015	-	-	ENSG0000015295	'NRSN1	-	6:24126121-24154900	-	-	IGV_LINK	0.969721	0.025041	0	0.465873	OK	0	0	0	0	OK	NOTEST	'-inf
ENSG0000015	-	-	ENSG0000015296	'JAKMIP1	-	4:6026198-6200591	-	-	IGV_LINK	105.7	2.7802	0.331067	5.22933	OK	39.1865	1.07817	0	2.34241	OK	OK	-1.3666
ENSG0000015	-	-	ENSG0000015297	'ZIC1	-	3:147386045-147510293	-	-	IGV_LINK	0	0	0	0	OK	0	0	0	0	OK	NOTEST	0
ENSG0000015	-	-	ENSG0000015299	'ADGRA3	-	4:22345070-22516054	-	-	IGV_LINK	623.53	9.76841	2.35113	17.1857	OK	384.647	5.88609	0.990344	10.7818	OK	OK	-0.73082
ENSG0000015	-	-	ENSG0000015300	'CPB1	-	3:148791101-148960112	-	-	IGV_LINK	2.90916	0.208738	0	1.61114	OK	8.2498	0.673192	0	2.90891	OK	OK	1.68932
ENSG0000015	-	-	ENSG0000015300	'SREK1IP1	-	5:64718143-64768685	-	-	IGV_LINK	329.705	7.10553	0	19.0312	OK	277.4	6.07319	0	15.6045	OK	OK	-0.22649
ENSG0000015	-	-	ENSG0000015301	'LGI2	-	4:24998846-25030879	-	-	IGV_LINK	16.4852	0.148515	0	0.37244	OK	36.0929	0.325263	0	0.713777	OK	NOTEST	1.131
ENSG0000015	-	-	ENSG0000015301	'CWC27	-	5:64768929-65018763	-	-	IGV_LINK	357.827	10.5835	3.52865	17.6384	OK	290.805	8.60524	2.78907	14.4214	OK	OK	-0.29853
ENSG0000015	-	-	ENSG0000015302	'MR1	-	1:181033424-181061938	-	-	IGV_LINK	1480.76	16.4384	3.05846	29.8183	OK	2283.13	32.1756	2.71583	61.6354	OK	OK	0.968901
ENSG0000015	-	-	ENSG0000015303	'SRP19	-	5:112707497-112922539	-	-	IGV_LINK	266.523	29.5823	0	94.5596	OK	248.807	26.3547	0	89.2624	OK	OK	-0.16667
ENSG0000015	-	-	ENSG0000015304	'CENPH	-	5:69189547-69210357	-	-	IGV_LINK	55.2741	2.99945	0	6.51414	OK	35.0616	1.62584	0	3.41118	OK	OK	-0.88351
ENSG0000015	-	-	ENSG0000015304	'CDYL	-	6:4706158-4955551	-	-	IGV_LINK	268.613	6.14559	1.42091	10.8703	OK	216.557	4.69507	0.907095	8.48305	OK	OK	-0.3884
ENSG0000015	-	-	ENSG0000015304	'CARHSP1	-	16:8674564-8869012	-	-	IGV_LINK	1517.61	83.3107	6.76061	159.861	OK	2343.97	153.21	6.60852	299.812	OK	OK	0.878938
ENSG0000015	-	-	ENSG0000015306	'TEKT5	-	16:10627500-10694945	-	-	IGV_LINK	5.81832	0.36475	0	1.01593	OK	7.21857	0.325864	0	0.851936	OK	NOTEST	-0.16264
ENSG0000015	-	-	ENSG0000015306	'BANK1	-	4:101411285-102074812	-	-	IGV_LINK	319.038	7.60301	1.92689	13.2791	OK	287.712	6.6226	1.95002	11.2952	OK	OK	-0.19917
ENSG0000015	-	-	ENSG0000015306	'TXNDC11	-	16:11668413-11744506	-	-	IGV_LINK	1191.79	36.0947	0.653115	71.5362	OK	1453	41.4034	5.76617	77.0406	OK	OK	0.197962
ENSG0000015	-	-	ENSG0000015307	'DAB2	-	5:39284261-39462300	-	-	IGV_LINK	6680.4	113.275	9.01537	217.534	OK	15066.2	269.061	0	560.683	OK	OK	1.24811
ENSG0000015	-	-	ENSG0000015308	'ACMSD	-	2:134735463-134959342	-	-	IGV_LINK	686.562	33.7423	3.5863	63.8982	OK	1572.62	76.7937	18.3086	135.279	OK	OK	1.18643
ENSG0000015	-	-	ENSG0000015309	'ACOXL	-	2:110732572-111118222	-	-	IGV_LINK	1.93944	0.05195	0	0.635379	OK	10.3122	0.266667	0	0.929442	OK	NOTEST	2.35985
ENSG0000015	-	-	ENSG0000015309	'BCL2L11	-	2:111119377-111168447	-	-	IGV_LINK	631.288	8.23733	1.10784	15.3668	OK	981.726	12.1948	2.49415	21.8955	OK	OK	0.566017
ENSG0000015	-	-	ENSG0000015310	'ANAPC1	-	2:111766270-111884690	-	-	IGV_LINK	494.557	8.26081	0	19.2811	OK	430.021	6.77752	0	16.2369	OK	OK	-0.28553
ENSG0000015	-	-	ENSG0000015311	'CAST	-	5:95885097-96808100	-	-	IGV_LINK	3129.29	72.1192	0	145.491	OK	3259.7	74.3923	0	150.146	OK	OK	0.044769
ENSG0000015	-	-	ENSG0000015313	'SCOC	-	4:140257285-140385726	-	-	IGV_LINK	372.373	12.4835	4.06951	20.8974	OK	434.146	14.8192	5.03835	24.6	OK	OK	0.247446
ENSG0000015	-	-	ENSG0000015313	'CLGN	-	4:140388454-140427968	-	-	IGV_LINK	32.0008	0.628265	0	1.32309	OK	52.5925	1.03297	0	2.11497	OK	OK	0.717351
ENSG0000015	-	-	ENSG0000015314	'CETN3	-	5:90392260-90409786	-	-	IGV_LINK	217.217	17.0024	3.23307	30.7718	OK	188.714	15.1905	1.95271	28.4284	OK	OK	-0.16257
ENSG0000015	-	-	ENSG0000015314	'SMARCA5	-	4:143513462-143557486	-	-	IGV_LINK	1218.94	11.5162	0	30.1362	OK	1387	12.2337	0	30.3857	OK	OK	0.087187
ENSG0000015	-	-	ENSG0000015315	'SYCP2L	-	6:10747758-10979320	-	-	IGV_LINK	54.3043	2.48491	0	13.9929	OK	30.9366	1.34616	0	8.91775	OK	OK	-0.88435
ENSG0000015	-	-	ENSG0000015316	'BMP6	-	6:7726796-7881422	-	-	IGV_LINK	21.3339	0.461089	0	1.02033	OK	55.6861	1.20408	0.022923	2.38523	OK	OK	1.38481
ENSG0000015	-	-	ENSG0000015316	'RGPD3	-	2:106382170-106468376	-	-	IGV_LINK	46.5486	0.483895	0	0.983391	OK	16.4996	0.174241	0	0.397695	OK	NOTEST	-1.47361
ENSG0000015	-	-	ENSG0000015317	'RASSF3	-	12:64507000-64697567	-	-	IGV_LINK	503.285	12.768	0.956636	24.5794	OK	595.017	15.704	0.671626	30.7364	OK	OK	0.2986
ENSG0000015	-	-	ENSG0000015318	'HNRNPU	-	1:244835321-244864560	-	-	IGV_LINK	7702.49	129.537	35.3978	223.676	OK	6942.21	125.552	28.6306	222.472	OK	OK	-0.04509
ENSG0000015	-	-	ENSG0000015320	'RANBP2	-	2:108719480-108785811	-	-	IGV_LINK	1144.27	5.58876	2.2908	8.88671	OK	991.007	4.84068	1.90771	7.77366	OK	OK	-0.20732

ENSG0000015	-	-	ENSG0000015320	'AHCTF1	-	1:246771836-246931978	-	-	IGV_LINK	737.957	7.50801	0	16.4201	OK	861.073	10.5834	0	27.7326	OK	OK	0.495302
ENSG0000015	-	-	ENSG0000015320	'MERTK	-	2:111898478-112029561	-	-	IGV_LINK	538.195	9.53519	2.56366	16.5067	OK	1344.72	24.2688	8.64584	39.8917	OK	OK	1.34777
ENSG0000015	-	-	ENSG0000015321	'TMEM87B	-	2:112055222-112119318	-	-	IGV_LINK	419.889	4.84356	1.29353	8.39358	OK	618.735	7.31308	1.85751	12.7687	OK	OK	0.594413
ENSG0000015	-	-	ENSG0000015323	'OR14K1	-	1:247507057-247747062	-	-	IGV_LINK	0	0	0	0	OK	0	0	0	0	OK	NOTEST	0
ENSG0000015	-	-	ENSG0000015323	'PTPRR	-	12:70638072-70920843	-	-	IGV_LINK	4.8486	0.175562	0	0.538997	OK	9.28102	0.336285	0	0.830798	OK	NOTEST	0.937707
ENSG0000015	-	-	ENSG0000015323	'NR4A2	-	2:156324431-156342348	-	-	IGV_LINK	168.731	4.10768	0.493851	7.7215	OK	153.653	3.02927	0.512371	5.54617	OK	OK	-0.43935
ENSG0000015	-	-	ENSG0000015323	'CCDC148	-	2:158166649-158456753	-	-	IGV_LINK	37.8191	1.66538	0	3.988	OK	100.029	3.35767	0	6.75493	OK	OK	1.01161
ENSG0000015	-	-	ENSG0000015324	'PLA2R1	-	2:159932005-160062610	-	-	IGV_LINK	211.399	1.89727	0	5.33447	OK	200.058	1.65943	0	4.76073	OK	OK	-0.19324
ENSG0000015	-	-	ENSG0000015325	'RBMS1	-	2:160272150-160493794	-	-	IGV_LINK	774.807	15.8424	3.19843	28.4863	OK	697.108	15.3632	2.50762	28.2187	OK	OK	-0.04431
ENSG0000015	-	-	ENSG0000015325	'SCN3A	-	2:164653623-165392310	-	-	IGV_LINK	0.969721	0.013753	0	0.397166	OK	10.3122	0.243215	0	2.46623	OK	NOTEST	4.14437
ENSG0000015	-	-	ENSG0000015326	'FEZF2	-	3:62369680-62374324	-	-	IGV_LINK	0	0	0	0	OK	0	0	0	0	OK	NOTEST	0
ENSG0000015	-	-	ENSG0000015328	'CD96	-	3:111292718-111665750	-	-	IGV_LINK	547.892	14.2653	0	29.1143	OK	288.743	6.45371	0	13.4204	OK	OK	-1.14431
ENSG0000015	-	-	ENSG0000015329	'SLC25A27	-	6:46652914-46735693	-	-	IGV_LINK	378.191	25.677	0	52.1976	OK	134.059	11.5538	0	32.8635	OK	OK	-1.15211
ENSG0000015	-	-	ENSG0000015329	'ADGRF1	-	6:46997702-47042363	-	-	IGV_LINK	5.81832	0.191288	0	0.751525	OK	20.6245	0.35483	0	1.12086	OK	NOTEST	0.891378
ENSG0000015	-	-	ENSG0000015329	'ADGRF4	-	6:47656435-47722021	-	-	IGV_LINK	0	0	0	0	OK	0	0	0	0	OK	NOTEST	0
ENSG0000015	-	-	ENSG0000015330	'FRMD1	-	6:168055744-168081557	-	-	IGV_LINK	256.976	3.9613	1.11916	6.80345	OK	158.809	2.50972	0.370986	4.64845	OK	OK	-0.65845
ENSG0000015	-	-	ENSG0000015331	'FAM49B	-	8:129832300-130017129	-	-	IGV_LINK	1469.13	47.9569	12.6719	83.242	OK	1458.15	50.1168	12.345	87.8886	OK	OK	0.063555
ENSG0000015	-	-	ENSG0000015331	'ASAP1	-	8:130052103-130443660	-	-	IGV_LINK	1405.13	21.0749	3.70066	38.4491	OK	1463.31	23.8466	2.16364	45.5296	OK	OK	0.178259
ENSG0000015	-	-	ENSG0000015333	'TRAPPC8	-	18:31829172-31953136	-	-	IGV_LINK	651.652	10.7631	0.653684	20.8726	OK	609.454	11.3194	0.385651	22.2532	OK	OK	0.072701
ENSG0000015	-	-	ENSG0000015334	'FAM81B	-	5:95391343-95450454	-	-	IGV_LINK	41.698	2.71584	0	6.08769	OK	20.6245	1.06427	0	2.62625	OK	OK	-1.35153
ENSG0000015	-	-	ENSG0000015336	'LINC00467	-	1:211382802-211435333	-	-	IGV_LINK	139.64	5.61514	0	12.8458	OK	201.089	10.3039	0.072233	20.5356	OK	OK	0.875802
ENSG0000015	-	-	ENSG0000015339	'INO80C	-	18:35443868-35497991	-	-	IGV_LINK	227.884	16.2204	3.92574	28.515	OK	312.461	25.457	6.49555	44.4184	OK	OK	0.650252
ENSG0000015	-	-	ENSG0000015339	'LPCAT1	-	5:1456479-1523977	-	-	IGV_LINK	1926.83	33.3736	7.47572	59.2715	OK	2662.62	45.1127	11.9749	78.2505	OK	OK	0.434825
ENSG0000015	-	-	ENSG0000015340	'PLEKHG4B	-	5:92150-189972	-	-	IGV_LINK	25.2127	0.12527	0	0.297632	OK	24.7494	0.112135	0	0.267373	OK	NOTEST	-0.1598
ENSG0000015	-	-	ENSG0000015340	'NMRAL1	-	16:4461679-4510347	-	-	IGV_LINK	643.894	40.6055	4.84741	76.3635	OK	794.043	45.0133	7.0337	82.9929	OK	OK	0.148678
ENSG0000015	-	-	ENSG0000015344	'UBALD1	-	16:4608882-4615027	-	-	IGV_LINK	245.339	9.95809	1.53593	18.3802	OK	204.183	8.20914	0.90815	15.5101	OK	OK	-0.27864
ENSG0000015	-	-	ENSG0000015344	'C16orf89	-	16:4882506-5097808	-	-	IGV_LINK	2.90916	0.246927	0	4.07131	OK	3.09367	0.210764	0	5.27557	OK	NOTEST	-0.22846
ENSG0000015	-	-	ENSG0000015348	'TMEM251	-	14:93184950-93229215	-	-	IGV_LINK	138.67	9.11554	0	22.9059	OK	175.308	10.7601	0	25.3382	OK	OK	0.239292
ENSG0000015	-	-	ENSG0000015348	'ING1	-	13:110641411-110723339	-	-	IGV_LINK	228.854	8.66751	0	23.5028	OK	241.307	8.38611	0	26.9788	OK	OK	-0.04762
ENSG0000015	-	-	ENSG0000015349	'TEX29	-	13:111316183-111344249	-	-	IGV_LINK	2.90916	0.305602	0	0.849597	OK	2.06245	0.175755	0	0.574917	OK	NOTEST	-0.79809
ENSG0000015	-	-	ENSG0000015349	'SPACA7	-	13:112376318-112434689	-	-	IGV_LINK	0	0	0	0	OK	0	0	0	0	OK	NOTEST	0
ENSG0000015	-	-	ENSG0000015353	'ADPRHL1	-	13:113399609-113453524	-	-	IGV_LINK	41.698	1.56392	0	3.33576	OK	76.3106	3.02756	0	6.22074	OK	OK	0.952994
ENSG0000015	-	-	ENSG0000015355	'CMTM7	-	3:32391670-32503408	-	-	IGV_LINK	287.037	13.5377	0	32.2167	OK	348.554	18.3365	0	44.45	OK	OK	0.437741
ENSG0000015	-	-	ENSG0000015355	'FBXL2	-	3:33277024-33441371	-	-	IGV_LINK	174.55	6.36295	0	18.2083	OK	164.996	6.08549	0	16.3261	OK	OK	-0.06432
ENSG0000015	-	-	ENSG0000015356	'UBP1	-	3:33277024-33441371	-	-	IGV_LINK	848.505	17.1652	0	34.8796	OK	679.577	11.9647	0.59845	23.3309	OK	OK	-0.52071
ENSG0000015	-	-	ENSG0000015356	'RMND5A	-	2:86503430-86778041	-	-	IGV_LINK	789.352	8.66823	0	24.4205	OK	648.641	7.00852	0	21.4134	OK	OK	-0.30663
ENSG0000015	-	-	ENSG0000015356	'CD8A	-	2:86784609-86808396	-	-	IGV_LINK	1521.49	41.6399	17.5563	65.7235	OK	494.988	13.5196	4.97673	22.0624	OK	OK	-1.62292
ENSG0000015	-	-	ENSG0000015357	'RPIA	-	2:88691643-88750935	-	-	IGV_LINK	268.613	9.03492	2.77196	15.2979	OK	169.121	5.69237	1.33284	10.0519	OK	OK	-0.66648
ENSG0000015	-	-	ENSG0000015368	'GOLGA8F	-	15:28378620-28392018	-	-	IGV_LINK	5.81832	0.064744	0	0.499446	OK	1.03122	0.011478	0	0.433556	OK	NOTEST	-2.49592
ENSG0000015	-	-	ENSG0000015370	'PTPRD	-	9:8314245-10612723	-	-	IGV_LINK	433.465	8.6703	0	20.3717	OK	276.368	4.92893	0	13.8198	OK	OK	-0.81481
ENSG0000015	-	-	ENSG0000015371	'LURAP1L	-	9:12685438-12822131	-	-	IGV_LINK	253.097	9.71717	2.59451	16.8398	OK	186.652	6.96923	1.56767	12.3708	OK	OK	-0.47954
ENSG0000015	-	-	ENSG0000015372	'CNKSR3	-	6:154387503-154510659	-	-	IGV_LINK	1628.16	25.2794	6.41234	44.1465	OK	902.322	16.8891	4.94035	28.8379	OK	OK	-0.58187
ENSG0000015	-	-	ENSG0000015376	'GTF2E1	-	3:120686680-120783069	-	-	IGV_LINK	153.216	3.04089	0	6.51313	OK	169.121	4.76004	0	12.9406	OK	OK	0.646478
ENSG0000015	-	-	ENSG0000015377	'CFDP1	-	16:75293697-75465497	-	-	IGV_LINK	1048.27	56.2909	17.4366	95.1452	OK	1115.79	76.9511	9.33818	144.564	OK	OK	0.451041
ENSG0000015	-	-	ENSG0000015377	'TGIF2LX	-	X:89921881-89922883	-	-	IGV_LINK	0	0	0	0	OK	0	0	0	0	OK	NOTEST	0
ENSG0000015	-	-	ENSG0000015378	'ZDHHC7	-	16:84974180-85011535	-	-	IGV_LINK	966.811	22.865	3.55332	42.1766	OK	1281.81	30.0078	4.88221	55.1334	OK	OK	0.392198
ENSG0000015	-	-	ENSG0000015378	'FAM92B	-	16:85098357-85112508	-	-	IGV_LINK	1.93944	0.121338	0	0.418084	OK	5.15612	0.403505	0	1.05059	OK	NOTEST	1.73356
ENSG0000015	-	-	ENSG0000015379	'C7orf31	-	7:25134696-25180356	-	-	IGV_LINK	131.882	3.58523	0	7.21352	OK	110.341	2.96001	0	6.03189	OK	OK	-0.27646
ENSG0000015	-	-	ENSG0000015380	'TMPRSS11D	-	4:67701279-68129880	-	-	IGV_LINK	0.969721	0.024017	0	0.814802	OK	0	0	0	0	OK	NOTEST	'-inf
ENSG0000015	-	-	ENSG0000015381	'JAZF1	-	7:27733063-28243917	-	-	IGV_LINK	348.13	8.30797	0	27.4627	OK	253.681	5.9569	0	21.0899	OK	OK	-0.47994
ENSG0000015	-	-	ENSG0000015381	'CMIP	-	16:81445169-81711762	-	-	IGV_LINK	1259.67	19.8625	7.26503	32.4599	OK	1970.67	37.4252	4.17603	70.6744	OK	OK	0.913967
ENSG0000015	-	-	ENSG0000015382	'SPHKAP	-	2:227979949-228181645	-	-	IGV_LINK	0.969721	0.008236	0	0.217656	OK	1.03122	0.00876	0	0.21799	OK	NOTEST	0.088973
ENSG0000015	-	-	ENSG0000015382	'KCNJ16	-	17:70017323-70135608	-	-	IGV_LINK	1578.7	32.9979	8.23444	57.7613	OK	3501.01	70.5904	16.092	125.089	OK	OK	1.0971
ENSG0000015	-	-	ENSG0000015382	'PID1	-	2:228850525-229271285	-	-	IGV_LINK	46.5466	1.29112	0	2.91284	OK	78.3731	2.50471	0	5.31815	OK	OK	0.956021
ENSG0000015	-	-	ENSG0000015382	'TRIP12	-	2:229763837-230013109	-	-	IGV_LINK	2888.8	36.5965	0	77.1671	OK	3343.23	52.208	0	116.423	OK	OK	0.512564
ENSG0000015	-	-	ENSG0000015383	'FBXO36	-	2:229763837-230013109	-	-	IGV_LINK	118.306	2.50591	0	9.67361	OK	90.7478	2.07695	0	9.47581	OK	OK	-0.27087
ENSG0000015	-	-	ENSG0000015387	'CEBPG	-	19:33373329-33382686	-	-	IGV_LINK	526.558	7.70093	2.84622	12.5557	OK	558.924	8.00403	3.14356	12.8645	OK	OK	0.055694
ENSG0000015	-	-	ENSG0000015388	'KCTD15	-	19:33795932-33815763	-	-	IGV_LINK	482.921	11.7744	0.892738	22.6561	OK	428.99	10.381	0.734856	20.0271	OK	OK	-0.18171
ENSG0000015	-	-	ENSG0000015389	'ZNF599	-	19:34758075-34773229	-	-	IGV_LINK	190.065	7.30627	0.449184	14.1634	OK	126.841	5.11395	0.024639	10.2033	OK	OK	-0.5147
ENSG0000015	-	-	ENSG0000015389	'MCOLN2	-	1:84925582-84997113	-	-	IGV_LINK	175.519	6.89599	0	14.5444	OK	117.56	4.8731	0	10.8334	OK	OK	-0.50092
ENSG0000015	-	-	ENSG0000015390	'LGI4	-	19:35124512-35154301	-	-	IGV_LINK	207.52	5.28595	0.701611	9.87028	OK	124.778	3.06228	0	6.40578	OK	OK	-0.78756
ENSG0000015	-	-	ENSG0000015390	'DDAH1	-	1:85266247-85578363	-	-	IGV_LINK	810.686	12.1913	4.4277	19.9549	OK	1171.47	17.6236	6.91214	28.3351	OK	OK	0.531661
ENSG0000015	-	-	ENSG0000015391	'SREK1	-	5:66139970-66183615	-	-	IGV_LINK	1832.77	42.4023	7.32026	77.4844	OK	1537.56	33.7309	9.86745	57.5944	OK	OK	-0.33007
ENSG0000015	-	-	ENSG0000015392	'CHD1	-	5:98853984-98928957	-	-	IGV_LINK	799.05	18.7462	0	38.8431	OK	725.982	18.9777	1.41457	36.5409	OK	OK	0.017707
ENSG0000015	-	-	ENSG0000015392	'CLCA3P	-	1:86547077-86693203	-	-	IGV_LINK	0	0	0	0	OK	0	0	0	0	OK	NOTEST	0
ENSG0000015	-	-	ENSG0000015393	'ANKFN1	-	17:55882300-56511659	-	-	IGV_LINK	0	0	0	0	OK	2.06245	0.1138	0	0.645652	OK	NOTEST	inf
ENSG0000015	-	-	ENSG0000015393	'DGKE	-	17:56791912-56869567	-	-	IGV_LINK	194.914	1.70332	0	3.9681	OK	234.088	1.8596	0	4.01036	OK	OK	0.126639
ENSG0000015	-	-	ENSG0000015393	'HS2ST1	-	1:86914647-87169204	-	-	IGV_LINK	718.155	8.14688	0	16.7901	OK	1220.55	15.8418	0.55276	31.1308	OK	OK	0.959413
ENSG0000015	-	-	ENSG0000015394	'MSI2	-	17:57255850-57684685	-	-	IGV_LINK	1216.03	23.7449	0	52.2056	OK	755.888	16.5352	0	37.4244	OK	OK	-0.52207
ENSG0000015	-	-	ENSG0000015395	'CACNA2D1	-	7:81946443-82443798	-	-	IGV_LINK	145.458	1.62106	0	3.69743	OK	106.216	1.51257	0	4.16486	OK	OK	-0.09993
ENSG0000015	-	-	ENSG0000015397	'ZUFSP	-	6:116635617-116668794	-	-	IGV_LINK	121.215	3.99248	0.329981	7.65499	OK	116.528	3.6873	0.310557	7.06405	OK	OK	-0.11472
ENSG0000015	-	-	ENSG0000015397	'HS3ST3A1	-	17:13495688-13601927	-	-	IGV_LINK	0	0	0	0	OK	4.1249	0.10879	0	0.351523	OK	NOTEST	inf
ENSG0000015	-	-	ENSG0000015398	'GDPD1	-	17:59220466-59275967	-	-	IGV_LINK	48.486	2.44374	0	5.25346	OK	40.2178	2.12228	0	4.5289	OK	OK	-0.20347
ENSG0000015	-	-	ENSG0000015398	'NUS1	-	6:117675501-117710640	-	-	IGV_LINK	793.231	9.9263	4.03364	15.819	OK	574.392	7.18964	2.84346	11.5358	OK	OK	-0.46534
ENSG0000015	-	-	ENSG0000015399	'SEMA3D	-	7:84995552-85186855	-	-	IGV_LINK	5.81832	0.053822	0	0.22168	OK	4.1249	0.171755	0	0.45394	OK	NOTEST	1.67409
ENSG0000015	-	-	ENSG0000015400	'PPP2R5E	-	14:63371356-63543374	-	-	IGV_LINK	343.281	5.16822	1.02377	9.31266	OK	339.273	4.78474	1.14439	8.42509	OK	OK	-0.11123
ENSG0000015	-	-	ENSG0000015400	'ASB17	-	1:75918872-75932431	-	-	IGV_LINK	0.969721	0.058481	0	0.229055	OK	0	0	0	0	OK	NOTEST	'-inf
ENSG0000015	-	-	ENSG0000015401	'GRAP	-	17:18950344-19047637	-	-	IGV_LINK	270.552	6.15179	0.595631	11.708	OK	260.9	5.93508	0.11211	11.758	OK	OK	-0.05174
ENSG0000015	-	-	ENSG0000015402	'SLC5A10	-	17:18950344-19047637	-	-	IGV_LINK	187.156	5.44632	0	11.0748	OK	317.617	8.76761	0.771498	16.7637	OK	OK	0.686901
ENSG0000015	-	-	ENSG0000015402	'AK5	-	1:77282050-77559969	-	-	IGV_LINK	3.87888	0.599573	0	2.05069	OK	3.09367	0.281799	0	1.31187	OK	OK	-1.08927
ENSG0000015	-	-	ENSG0000015404	'CABYR	-	18:24138955-24397880	-	-	IGV_LINK	13.5761	1.30325	0	9.52939	OK	20.6245	1.22601	0	6.85707	OK	OK	-0.08814
ENSG0000015	-	-	ENSG0000015405	'IMPACT	-	18:24426615-24453535	-	-	IGV_LINK	554.68	12.2359	0.514906	23.9568	OK	705.358	16.5524	1.04242	32.0625	OK	OK	0.43593
ENSG0000015	-	-	ENSG0000015406	'ANKRD29	-	18:23598925-23662885	-	-	IGV_LINK	288.977	11.285	0.455647	22.1143	OK	224.807	7.35963	0	14.8037	OK	OK	-0.6167
ENSG0000015	-	-	ENSG0000015407	'SDHAF4	-	6:70566916-70589569	-	-	IGV_LINK	165.822	9.70245	1.19325	18.2116	OK	126.841	7.29886	0.621114	13.9766	OK	OK	-0.41068
ENSG0000015	-	-	ENSG0000015408	'CHST9	-	18:26454909-27190698	-	-	IGV_LINK	407.283	5.04233	0	10.5182	OK	77.3419	1.08893	0	3.05267	OK	OK	-2.21117
ENSG0000015	-	-	ENSG0000015409	'THY1	-	11:119381777-119526664	-	-	IGV_LINK	1451.67	58.4164	11.0243	105.809	OK	1947.98	84.186	12.3276	156.044	OK	OK	0.527207
ENSG0000015	-	-	ENSG0000015409	'DNAAF1	-	16:84145286-84187070	-	-	IGV_LINK	225.945	4.79317	0	15.1336	OK	189.745	4.01765	0	13.0482	OK	OK	-0.25463
ENSG0000015	-	-	ENSG0000015410	'C16orf74	-	16:85690083-85751129	-	-	IGV_LINK	32.0008	2.86574	0	6.2802	OK	144.371	12.668	1.73068	23.6053	OK	OK	2.14421
ENSG0000015	-	-	ENSG0000015411	'TBCEL	-	11:121024071-121090775	-	-	IGV_LINK	364.615	5.519	1.00054	10.0375	OK	368.147	5.80812	0.379458	11.2368	OK	OK	0.073665
ENSG0000015	-	-	ENSG0000015411	'JPH3	-	16:87600525-87765992	-	-	IGV_LINK	5.81832	0.086006	0	0.680633	OK	10.3122	0.150975	0	0.95041	OK	NOTEST	0.811806
ENSG0000015	-	-	ENSG0000015412	'ANKH	-	5:14704803-14871778	-	-	IGV_LINK	1009.48	13.2898	0	29.5783	OK	1088.97	16.2261	0	38.0707	OK	OK	0.288005
ENSG0000015	-	-	ENSG0000015412	'OTULIN	-	5:14664663-14699711	-	-	IGV_LINK	735.048	10.3897	0	23.7063	OK	966.258	17.846	0	39.0015	OK	OK	0.780458
ENSG0000015	-	-	ENSG0000015412	'UBASH3B	-	11:122655674-122814473	-	-	IGV_LINK	128.003	1.60062	0	4.21959	OK	157.777	1.43106	0	2.92154	OK	OK	-0.16155
ENSG0000015	-	-	ENSG0000015413	'ROBO4	-	11:124883690-124898500	-	-	IGV_LINK	558.559	8.22117	2.06262	14.3797	OK	657.922	10.1516	2.80129	17.502	OK	OK	0.304296
ENSG0000015	-	-	ENSG0000015413	'ROBO3	-	11:124865385-124881470	-	-	IGV_LINK	612.863	28.114	6.76057	49.4674	OK	574.392	29.303	5.37654	53.2294	OK	OK	0.05976
ENSG0000015	-	-	ENSG0000015414	'PANX3	-	11:124611489-124620356	-	-	IGV_LINK	0.969721	0.036567	0	0.143225	OK	0	0	0	0	OK	NOTEST	'-inf
ENSG0000015	-	-	ENSG0000015414	'TBRG1	-	11:124622835-124697518	-	-	IGV_LINK	744.745	26.3895	0	59.2712	OK	609.454	21.9991	0	48.8537	OK	OK	-0.26252
ENSG0000015	-	-	ENSG0000015414	'NRGN	-	11:124739845-124747210	-	-	IGV_LINK	138.67	7.91129	1.43453	14.3881	OK	75.2794	3.97372	0.138754	7.80868	OK	OK	-0.99343
ENSG0000015	-	-	ENSG0000015415	'RETREG1	-	5:16473037-16629969	-	-	IGV_LINK	781.595	22.9322	5.07451	40.7899	OK	2036.67	61.6391	19.966	103.312	OK	OK	1.42647
ENSG0000015	-	-	ENSG0000015416	'CDH12	-	5:21616261-22853622	-	-	IGV_LINK	0.969721	0.027441	0	0.374021	OK	0	0	0	0	OK	NOTEST	'-inf
ENSG0000015	-	-	ENSG0000015416	'GPR15	-	3:98497603-98593723	-	-	IGV_LINK	2.90916	0.151877	0	1.96337	OK	23.7182	1.23956	0	6.69588	OK	OK	3.02886
ENSG0000015	-	-	ENSG0000015417	'TOMM70	-	3:100363430-100456319	-	-	IGV_LINK	1913.26	26.6701	9.33767	44.0026	OK	2491.44	34.391	12.4506	56.3314	OK	OK	0.366806
ENSG0000015	-	-	ENSG0000015417	'ABI3BP	-	3:100749155-100993515	-	-	IGV_LINK	1453.61	41.1468	1.88348	80.4101	OK	1092.07	30.3715	0.725428	60.0175	OK	OK	-0.43806
ENSG0000015	-	-	ENSG0000015418	'ANGPT1	-	8:107249481-107498055	-	-	IGV_LINK	102.79	2.10357	0	4.24128	OK	63.9359	1.08478	0	2.30552	OK	OK	-0.95543
ENSG0000015	-	-	ENSG0000015419	'CYP4Z2P	-	1:46843094-46900437	-	-	IGV_LINK	0.969721	0.043246	0	0.194421	OK	0	0	0	0	OK	NOTEST	'-inf
ENSG0000015	-	-	ENSG0000015421	'PITPNC1	-	17:67377280-67697261	-	-	IGV_LINK	672.986	22.0806	5.88921	38.272	OK	461.989	15.6519	3.80428	27.4995	OK	OK	-0.49644
ENSG0000015	-	-	ENSG0000015422	'CC2D1B	-	1:52142093-52366193	-	-	IGV_LINK	1043.42	20.35	0	42.082	OK	1124.04	22.63	0	45.9117	OK	OK	0.153207
ENSG0000015	-	-	ENSG0000015422	'CERS3	-	15:100372938-100544995	-	-	IGV_LINK	5.81832	0.147652	0	0.699772	OK	3.09367	0.069034	0	0.570763	OK	NOTEST	-1.09682
ENSG0000015	-	-	ENSG0000015422	'PRKCA	-	17:66302635-66810743	-	-	IGV_LINK	716.623	4.81856	1.87427	7.76284	OK	842.511	5.73164	2.22458	9.23869	OK	OK	0.250346
ENSG0000015	-	-	ENSG0000015423	'LRRK1	-	15:100849560-101086066	-	-	IGV_LINK	1624.28	15.2495	0	33.2453	OK	1410.72	12.8576	0	27.5776	OK	OK	-0.24614
ENSG0000015	-	-	ENSG0000015424	'CEP112	-	17:65528562-66192084	-	-	IGV_LINK	329.705	13.5702	0	27.9687	OK	412.385	16.3963	0	32.9017	OK	OK	0.272925
ENSG0000015	-	-	ENSG0000015425	'GAL3ST2	-	2:241776824-241804208	-	-	IGV_LINK	1.93944	0.091274	0	0.279521	OK	3.09367	0.145734	0	0.377454	OK	NOTEST	0.675067
ENSG0000015	-	-	ENSG0000015425	'ABCA9	-	17:68867291-69061064	-	-	IGV_LINK	78.5474	0.844546	0	2.28512	OK	194.902	2.41459	0	6.09415	OK	OK	1.51553
ENSG0000015	-	-	ENSG0000015426	'ABCA6	-	17:69078701-69141888	-	-	IGV_LINK	24.243	0.40085	0	1.05926	OK	265.025	4.45017	0.850682	8.04966	OK	OK	3.47273
ENSG0000015	-	-	ENSG0000015426	'ABCA10	-	17:69147213-69327244	-	-	IGV_LINK	61.0925	1.72037	0	7.99954	OK	45.3739	1.62296	0	7.21597	OK	OK	-0.08409
ENSG0000015	-	-	ENSG0000015426	'ABCA5	-	17:69147213-69327244	-	-	IGV_LINK	756.382	16.5757	2.84774	30.3038	OK	509.425	10.7408	2.47286	19.0087	OK	OK	-0.62598
ENSG0000015	-	-	ENSG0000015426	'ENPP3	-	6:131628441-131747418	-	-	IGV_LINK	7933.28	158.372	49.9921	266.752	OK	4220.8	82.1256	31.676	132.575	OK	OK	-0.94742
ENSG0000015	-	-	ENSG0000015427	'C4orf19	-	4:37453940-37686376	-	-	IGV_LINK	173.58	5.89137	0.109311	11.6734	OK	336.179	11.1845	2.3754	19.9937	OK	OK	0.924829
ENSG0000015	-	-	ENSG0000015427	'UCHL1	-	4:41220073-41268455	-	-	IGV_LINK	97.9418	7.30928	0.705764	13.9128	OK	926.04	65.5394	26.5469	104.532	OK	OK	3.16456
ENSG0000015	-	-	ENSG0000015430	'MIA3	-	1:222618085-222668012	-	-	IGV_LINK	1279.06	36.3146	0	83.4628	OK	1346.78	38.9669	0	86.9964	OK	OK	0.1017
ENSG0000015	-	-	ENSG0000015430	'DISP1	-	1:222815021-223005995	-	-	IGV_LINK	327.766	4.29994	0.992648	7.60723	OK	511.488	6.64852	1.92359	11.3734	OK	OK	0.628717
ENSG0000015	-	-	ENSG0000015431	'TNIK	-	3:171061338-171460408	-	-	IGV_LINK	661.349	10.6332	0	23.8119	OK	463.02	6.47568	0	14.6789	OK	OK	-0.71547
ENSG0000015	-	-	ENSG0000015431	'TDH	-	8:11339636-11474715	-	-	IGV_LINK	0.969721	0.086401	0	0.7446	OK	2.06245	0.093281	0	0.680865	OK	NOTEST	0.110543
ENSG0000015	-	-	ENSG0000015431	'FAM167A	-	8:11339636-11474715	-	-	IGV_LINK	45.5769	0.654983	0	1.3987	OK	68.0608	1.09251	0	2.34074	OK	OK	0.73811
ENSG0000015	-	-	ENSG0000015432	'NEIL2	-	8:11769638-11787346	-	-	IGV_LINK	289.946	8.0005	1.79998	14.201	OK	334.117	10.1086	2.79893	17.4182	OK	OK	0.337418
ENSG0000015	-	-	ENSG0000015433	'PGM5	-	9:68306527-68531061	-	-	IGV_LINK	291.886	7.32395	0	16.2311	OK	124.778	4.05167	0	10.8515	OK	OK	-0.8541
ENSG0000015	-	-	ENSG0000015434	'WNT3A	-	1:228007050-228061260	-	-	IGV_LINK	6.78804	0.138629	0	0.325804	OK	6.18735	0.126414	0	0.299777	OK	NOTEST	-0.13307
ENSG0000015	-	-	ENSG0000015435	'OBSCN	-	1:228203505-228378874	-	-	IGV_LINK	2201.27	22.5669	0	52.3083	OK	2351.19	21.8402	0	51.506	OK	OK	-0.04722
ENSG0000015	-	-	ENSG0000015435	'LONRF1	-	8:12721893-12756073	-	-	IGV_LINK	217.217	6.11834	0.240434	11.9962	OK	307.305	10.7017	1.12713	20.2763	OK	OK	0.806629
ENSG0000015	-	-	ENSG0000015437	'TRIM11	-	1:228393672-228406840	-	-	IGV_LINK	399.525	8.36833	1.47901	15.2577	OK	375.366	7.72413	2.03095	13.4173	OK	OK	-0.11557
ENSG0000015	-	-	ENSG0000015438	'ENAH	-	1:225486834-225653142	-	-	IGV_LINK	473.224	7.67871	0.752617	14.6048	OK	550.674	10.0926	0.715572	19.4696	OK	OK	0.394361
ENSG0000015	-	-	ENSG0000015441	'PPP1R3A	-	7:113876776-114075920	-	-	IGV_LINK	0	0	0	0	OK	0	0	0	0	OK	NOTEST	0

ENSG0000015	-	-	ENSG0000015442	'CCSAP	-	1:229319402-229343294	-	-	IGV_LINK	236.612	2.75339	0.803554	4.70323	OK	191.808	2.24284	0.576073	3.9096	OK	OK	-0.29589
ENSG0000015	-	-	ENSG0000015443	'ASZ1	-	7:117363221-117428123	-	-	IGV_LINK	0	0	0	0	OK	0	0	0	0	OK	NOTEST	0
ENSG0000015	-	-	ENSG0000015444	'SH3RF1	-	4:169094255-169271105	-	-	IGV_LINK	343.281	3.79424	1.18503	6.40344	OK	315.555	4.33659	0	9.64848	OK	OK	0.192751
ENSG0000015	-	-	ENSG0000015445	'GBP5	-	1:89258949-89272804	-	-	IGV_LINK	3103.11	75.9531	21.6888	130.217	OK	1411.75	34.2728	10.2271	58.3185	OK	OK	-1.14805
ENSG0000015	-	-	ENSG0000015447	'BUB3	-	10:123154276-123170467	-	-	IGV_LINK	1114.21	47.5197	14.9752	80.0642	OK	794.043	33.4394	8.29148	58.5874	OK	OK	-0.50698
ENSG0000015	-	-	ENSG0000015447	'GPR26	-	10:123666354-123694607	-	-	IGV_LINK	9.69721	0.074439	0	0.167892	OK	6.18735	0.047503	0	0.112649	OK	NOTEST	-0.64802
ENSG0000015	-	-	ENSG0000015447	'CCDC173	-	2:169645424-169694433	-	-	IGV_LINK	23.2733	0.653041	0	1.49643	OK	16.4996	0.463224	0	1.12146	OK	OK	-0.49546
ENSG0000015	-	-	ENSG0000015448	'MMP21	-	10:125766452-125775821	-	-	IGV_LINK	6.78804	0.219037	0	0.514779	OK	5.15612	0.166488	0	0.408119	OK	NOTEST	-0.39576
ENSG0000015	-	-	ENSG0000015449	'C10orf90	-	10:126424996-126670446	-	-	IGV_LINK	0	0	0	0	OK	0	0	0	0	OK	NOTEST	0
ENSG0000015	-	-	ENSG0000015451	'FAM69A	-	1:92832024-92961522	-	-	IGV_LINK	102.79	2.41218	0	23.2711	OK	121.685	3.08403	0	24.1123	OK	OK	0.354477
ENSG0000015	-	-	ENSG0000015451	'ATP5G3	-	2:175176257-175184607	-	-	IGV_LINK	1736.77	129.621	51.7941	207.447	OK	2061.42	155.892	62.1832	249.601	OK	OK	0.26625
ENSG0000015	-	-	ENSG0000015452	'CNTNAP3B	-	9:41890313-42129510	-	-	IGV_LINK	388.858	7.41198	0	14.9967	OK	658.953	12.8887	0	26.153	OK	OK	0.798172
ENSG0000015	-	-	ENSG0000015453	'FAM27C	-	9:61854161-61855331	-	-	IGV_LINK	107.639	13.6785	1.50667	25.8503	OK	95.9039	12.2189	1.43205	23.0058	OK	OK	-0.16279
ENSG0000015	-	-	ENSG0000015454	'MAGED4	-	X:52184822-52192268	-	-	IGV_LINK	0	0	0	0	OK	0	0	0	0	OK	NOTEST	0
ENSG0000015	-	-	ENSG0000015454	'SRSF12	-	6:89095958-89118081	-	-	IGV_LINK	23.2733	0.616005	0	1.70542	OK	13.4059	0.469938	0	1.36753	OK	OK	-0.39047
ENSG0000015	-	-	ENSG0000015455	'PDLIM3	-	4:185500659-185535612	-	-	IGV_LINK	235.642	8.81022	1.67819	15.9423	OK	388.772	14.4925	4.66204	24.323	OK	OK	0.718058
ENSG0000015	-	-	ENSG0000015455	'SORBS2	-	4:185585443-185956652	-	-	IGV_LINK	807.777	25.32	1.37602	49.2639	OK	1147.75	34.2513	4.04067	64.462	OK	OK	0.435885
ENSG0000015	-	-	ENSG0000015458	'ELOC	-	8:73939168-73972287	-	-	IGV_LINK	644.864	57.6171	21.4903	93.744	OK	817.761	74.7551	27.3835	122.127	OK	OK	0.375674
ENSG0000015	-	-	ENSG0000015458	'LY96	-	8:73991351-74029087	-	-	IGV_LINK	97.9418	14.7074	1.37697	28.0378	OK	145.403	20.097	3.77249	36.4216	OK	OK	0.450442
ENSG0000015	-	-	ENSG0000015460	'CEP170P1	-	4:118467589-118554100	-	-	IGV_LINK	3.87888	0.049518	0	0.126114	OK	0	0	0	0	OK	NOTEST	'-inf
ENSG0000015	-	-	ENSG0000015461	'PSMA8	-	18:26133851-26193355	-	-	IGV_LINK	0.969721	0.049344	0	0.276993	OK	4.1249	0.152036	0	0.462941	OK	NOTEST	1.62346
ENSG0000015	-	-	ENSG0000015462	'TMSB4Y	-	Y:13703566-13706024	-	-	IGV_LINK	8.72748	0.328667	0	0.748017	OK	11.3435	0.42751	0	0.957991	OK	NOTEST	0.379334
ENSG0000015	-	-	ENSG0000015463	'CXADR	-	21:17512381-17593579	-	-	IGV_LINK	267.643	7.96109	0.995169	14.927	OK	159.84	4.57405	0.11517	9.03293	OK	OK	-0.79949
ENSG0000015	-	-	ENSG0000015464	'BTG3	-	21:17593652-17633199	-	-	IGV_LINK	397.585	17.9322	5.52094	30.3434	OK	420.74	18.7498	6.13884	31.3607	OK	OK	0.064324
ENSG0000015	-	-	ENSG0000015464	'C21orf91	-	21:17763314-17819386	-	-	IGV_LINK	180.368	3.87631	0	8.17243	OK	142.309	3.60928	0	7.91208	OK	OK	-0.10297
ENSG0000015	-	-	ENSG0000015464	'CHODL	-	21:17901262-18267373	-	-	IGV_LINK	3.87888	0.149265	0	0.420184	OK	3.09367	0.079642	0	0.27265	OK	NOTEST	-0.90628
ENSG0000015	-	-	ENSG0000015464	'TMPRSS15	-	21:18269115-18486599	-	-	IGV_LINK	0	0	0	0	OK	0	0	0	0	OK	NOTEST	0
ENSG0000015	-	-	ENSG0000015465	'NCAM2	-	21:20998314-21543329	-	-	IGV_LINK	26.1825	0.791828	0	3.24997	OK	39.1865	0.979531	0	2.87638	OK	OK	0.306903
ENSG0000015	-	-	ENSG0000015465	'L3MBTL4	-	18:5954705-6415237	-	-	IGV_LINK	458.678	7.99057	1.85748	14.1237	OK	481.582	8.10958	2.28805	13.9311	OK	OK	0.021329
ENSG0000015	-	-	ENSG0000015467	'PDE1C	-	7:31751178-32299329	-	-	IGV_LINK	121.215	1.84427	0	4.79392	OK	127.872	1.55505	0	3.56406	OK	OK	-0.24609
ENSG0000015	-	-	ENSG0000015471	'RABGEF1	-	7:66628766-66844826	-	-	IGV_LINK	338.271	9.13488	0	20.2025	OK	477.051	13.3464	0	27.7693	OK	OK	0.546997
ENSG0000015	-	-	ENSG0000015471	'MRPL39	-	21:25585655-25607517	-	-	IGV_LINK	331.644	20.1696	6.90483	33.4343	OK	331.023	19.9501	6.79116	33.109	OK	OK	-0.01579
ENSG0000015	-	-	ENSG0000015472	'JAM2	-	21:25639271-25772460	-	-	IGV_LINK	151.276	4.53827	0	17.8064	OK	155.715	3.8314	0	12.6359	OK	OK	-0.24427
ENSG0000015	-	-	ENSG0000015472	'ATP5J	-	21:25639271-25772460	-	-	IGV_LINK	1089.97	131.509	33.667	229.351	OK	1216.85	147.193	45.1392	249.247	OK	OK	0.162548
ENSG0000015	-	-	ENSG0000015472	'GABPA	-	21:25639271-25772460	-	-	IGV_LINK	396.616	4.78964	0	11.2047	OK	347.523	4.16214	0	9.32343	OK	OK	-0.20259
ENSG0000015	-	-	ENSG0000015473	'ADAMTS1	-	21:26835746-26845409	-	-	IGV_LINK	1847.32	25.678	2.53998	48.8161	OK	3068.93	51.5288	0	108.553	OK	OK	1.00484
ENSG0000015	-	-	ENSG0000015473	'ADAMTS5	-	21:26889375-26966513	-	-	IGV_LINK	474.193	3.00821	1.14269	4.87373	OK	381.553	2.42083	0.865413	3.97624	OK	OK	-0.3134
ENSG0000015	-	-	ENSG0000015474	'TSEN2	-	3:12484431-12664226	-	-	IGV_LINK	142.549	5.82322	0	20.9025	OK	123.747	3.92564	0	15.5186	OK	OK	-0.56889
ENSG0000015	-	-	ENSG0000015476	'SLFN13	-	17:35435095-35448837	-	-	IGV_LINK	2397.15	51.0285	0	104.571	OK	1992.33	41.7822	0.042884	83.5215	OK	OK	-0.28841
ENSG0000015	-	-	ENSG0000015476	'WNT7A	-	3:13816257-13880121	-	-	IGV_LINK	0	0	0	0	OK	0	0	0	0	OK	NOTEST	0
ENSG0000015	-	-	ENSG0000015476	'XPC	-	3:14124939-14201119	-	-	IGV_LINK	766.079	21.9224	0	58.6915	OK	824.98	22.4653	0	61.7612	OK	OK	0.035293
ENSG0000015	-	-	ENSG0000015478	'CCDC174	-	3:14651745-14672659	-	-	IGV_LINK	371.403	13.7651	0	27.9071	OK	347.523	10.924	0.331702	21.5163	OK	OK	-0.33351
ENSG0000015	-	-	ENSG0000015478	'FGD5	-	3:14799330-15065335	-	-	IGV_LINK	440.253	5.09904	0	14.3512	OK	613.579	6.86544	0	18.3808	OK	OK	0.429126
ENSG0000015	-	-	ENSG0000015480	'FLCN	-	17:17042544-17237188	-	-	IGV_LINK	1159.26	28.2859	0	89.7671	OK	847.391	20.3324	0	60.5693	OK	OK	-0.4763
ENSG0000015	-	-	ENSG0000015481	'DPH3	-	3:16257977-16264972	-	-	IGV_LINK	186.186	3.73447	0.605704	6.86323	OK	199.026	4.12363	0.464923	7.78234	OK	OK	0.143012
ENSG0000015	-	-	ENSG0000015481	'OXNAD1	-	3:16265159-16514026	-	-	IGV_LINK	171.641	4.83527	0	15.0231	OK	138.184	4.46188	0	13.6699	OK	OK	-0.11594
ENSG0000015	-	-	ENSG0000015482	'PLCL2	-	3:16802650-17090594	-	-	IGV_LINK	448.011	6.8233	2.35332	11.2933	OK	258.837	4.02423	1.20144	6.84703	OK	OK	-0.76176
ENSG0000015	-	-	ENSG0000015483	'CXXC1	-	18:50282342-50288304	-	-	IGV_LINK	1407.06	65.5067	16.1648	114.849	OK	1127.13	53.0188	13.1676	92.87	OK	OK	-0.30514
ENSG0000015	-	-	ENSG0000015483	'SKA1	-	18:50374994-50394173	-	-	IGV_LINK	33.9402	0.940893	0	2.2704	OK	37.1241	0.986526	0	2.24715	OK	OK	0.068326
ENSG0000015	-	-	ENSG0000015484	'PPP4R1	-	18:9546790-9615240	-	-	IGV_LINK	929.962	20.4215	1.60157	39.2414	OK	1141.57	20.9267	3.57479	38.2787	OK	OK	0.035259
ENSG0000015	-	-	ENSG0000015485	'APCDD1	-	18:10454627-10489948	-	-	IGV_LINK	345.22	10.4816	0.434966	20.5283	OK	420.74	17.8147	1.68865	33.9408	OK	OK	0.765207
ENSG0000015	-	-	ENSG0000015486	'PIEZO2	-	18:10661932-11148762	-	-	IGV_LINK	210.429	2.42092	0	5.94669	OK	161.902	2.27337	0	6.09173	OK	OK	-0.09072
ENSG0000015	-	-	ENSG0000015487	'CCDC144B	-	17:18511220-18625617	-	-	IGV_LINK	66.3035	1.11835	0	5.59166	OK	24.0833	0.365657	0	2.36435	OK	OK	-1.61281
ENSG0000015	-	-	ENSG0000015488	'MPPE1	-	18:11688955-11909223	-	-	IGV_LINK	1118.09	52.6187	0	105.689	OK	1234.38	57.6553	0	117.391	OK	OK	0.131877
ENSG0000015	-	-	ENSG0000015489	'CCDC144CP	-	17:20009343-20403557	-	-	IGV_LINK	2.90916	0.032436	0	0.292566	OK	3.09367	0.034501	0	0.307218	OK	NOTEST	0.089047
ENSG0000015	-	-	ENSG0000015491	'USP43	-	17:9576626-9729691	-	-	IGV_LINK	198.793	3.34823	0.682237	6.01422	OK	234.088	4.17551	0.570918	7.78009	OK	OK	0.318553
ENSG0000015	-	-	ENSG0000015491	'RAB6B	-	3:133784032-133895836	-	-	IGV_LINK	637.106	13.2421	0	31.2157	OK	680.608	15.4979	0	37.1695	OK	OK	0.226936
ENSG0000015	-	-	ENSG0000015492	'EME1	-	17:50373219-50397888	-	-	IGV_LINK	53.3346	1.70748	0	5.98937	OK	65.9984	2.97049	0	11.6674	OK	OK	0.798829
ENSG0000015	-	-	ENSG0000015492	'EPHB1	-	3:134597800-135260467	-	-	IGV_LINK	241.46	3.29538	0	6.62771	OK	96.9351	3.46928	0	8.84723	OK	OK	0.074194
ENSG0000015	-	-	ENSG0000015493	'ACSS1	-	20:25006229-25058980	-	-	IGV_LINK	2512.55	39.2676	16.7081	61.8271	OK	2116.07	33.2611	14.575	51.9471	OK	OK	-0.23951
ENSG0000015	-	-	ENSG0000015494	'ANKRD40	-	17:50693189-50707924	-	-	IGV_LINK	697.229	10.8272	2.76821	18.8862	OK	777.544	12.1336	3.04311	21.2241	OK	OK	0.164348
ENSG0000015	-	-	ENSG0000015495	'ZNF18	-	17:11598430-11997510	-	-	IGV_LINK	224.975	6.20708	0.452424	11.9617	OK	300.086	8.559	1.26716	15.8508	OK	OK	0.463527
ENSG0000015	-	-	ENSG0000015497	'CA10	-	17:51630312-52160017	-	-	IGV_LINK	0	0	0	0	OK	1.03122	0.027642	0	0.437808	OK	NOTEST	inf
ENSG0000015	-	-	ENSG0000015497	'VOPP1	-	7:55436055-55572988	-	-	IGV_LINK	2118.84	44.0851	17.9245	70.2458	OK	2094.42	43.2805	17.3556	69.2055	OK	OK	-0.02657
ENSG0000015	-	-	ENSG0000015499	'SEPT14	-	7:55793543-55862789	-	-	IGV_LINK	0.969721	0.015128	0	0.221137	OK	1.03122	0.016092	0	0.222227	OK	NOTEST	0.089178
ENSG0000015	-	-	ENSG0000015500	'APOOL	-	X:85003825-85109048	-	-	IGV_LINK	322.917	4.731	0.18823	9.27376	OK	322.773	4.66016	0.240858	9.07946	OK	OK	-0.02176
ENSG0000015	-	-	ENSG0000015501	'DKK2	-	4:106921801-107301870	-	-	IGV_LINK	1.93944	0.075698	0	0.336659	OK	8.2498	0.153277	0	0.475187	OK	NOTEST	1.01781
ENSG0000015	-	-	ENSG0000015501	'CYP2U1	-	4:107824562-107978799	-	-	IGV_LINK	242.43	3.11635	0	6.28319	OK	160.871	1.93808	0	4.67699	OK	OK	-0.68523
ENSG0000015	-	-	ENSG0000015502	'RSPH10B	-	7:5898724-5970683	-	-	IGV_LINK	49.4557	1.02903	0	3.12555	OK	60.8423	1.26649	0	3.04368	OK	OK	0.299549
ENSG0000015	-	-	ENSG0000015503	'FBXL18	-	7:5431334-5513798	-	-	IGV_LINK	48.486	0.849266	0	1.94216	OK	86.6229	1.59408	0	3.48708	OK	OK	0.908439
ENSG0000015	-	-	ENSG0000015505	'CNTNAP5	-	2:124025286-124915287	-	-	IGV_LINK	0	0	0	0	OK	2.06245	0.022744	0	0.405647	OK	NOTEST	inf
ENSG0000015	-	-	ENSG0000015506	'PROM2	-	2:95274452-95291308	-	-	IGV_LINK	29.0916	0.502737	0	1.65806	OK	4714.76	90.4335	0	186.264	OK	OK	7.49091
ENSG0000015	-	-	ENSG0000015507	'UNC93B2	-	7:6855484-6859830	-	-	IGV_LINK	0	0	0	0	OK	0	0	0	0	OK	NOTEST	0
ENSG0000015	-	-	ENSG0000015508	'AK9	-	6:109487905-109691217	-	-	IGV_LINK	233.703	5.09913	0.157426	10.0408	OK	75.2794	2.18402	0	4.81334	OK	OK	-1.22327
ENSG0000015	-	-	ENSG0000015508	'ODF1	-	8:102551571-102561017	-	-	IGV_LINK	0.969721	0.065022	0	0.425505	OK	0	0	0	0	OK	NOTEST	'-inf
ENSG0000015	-	-	ENSG0000015509	'KLF10	-	8:102648778-102655902	-	-	IGV_LINK	1131.66	21.6906	9.25534	34.1259	OK	1359.15	26.0965	11.5592	40.6338	OK	OK	0.266785
ENSG0000015	-	-	ENSG0000015509	'PTPRN2	-	7:157539055-158587788	-	-	IGV_LINK	427.647	5.33248	1.69378	8.97118	OK	235.119	2.93251	0.546035	5.31899	OK	OK	-0.86267
ENSG0000015	-	-	ENSG0000015509	'AZIN1	-	8:102826356-102977876	-	-	IGV_LINK	1127.78	21.1897	3.52803	38.8513	OK	1143.63	19.5062	5.0037	34.0087	OK	OK	-0.11943
ENSG0000015	-	-	ENSG0000015509	'ATP6V1C1	-	8:103020186-103073051	-	-	IGV_LINK	1851.2	43.022	4.87592	81.1682	OK	1795.36	40.5922	5.23348	75.9508	OK	OK	-0.08388
ENSG0000015	-	-	ENSG0000015509	'TMEM55A	-	8:90993795-91041064	-	-	IGV_LINK	243.4	21.6383	0	60.4598	OK	181.496	13.0919	2.15919	24.0245	OK	OK	-0.72491
ENSG0000015	-	-	ENSG0000015510	'OTUD6B	-	8:91070195-91087095	-	-	IGV_LINK	152.246	5.0189	0.340559	9.69724	OK	183.558	5.99383	0.760784	11.2269	OK	OK	0.256106
ENSG0000015	-	-	ENSG0000015511	'CDK19	-	6:110609977-110815958	-	-	IGV_LINK	831.05	9.18447	2.20253	16.1664	OK	473.332	5.6056	0.708879	10.5023	OK	OK	-0.71233
ENSG0000015	-	-	ENSG0000015511	'GTF3C6	-	6:110958559-110967890	-	-	IGV_LINK	563.408	43.6846	17.0446	70.3247	OK	531.081	39.113	15.1305	63.0954	OK	OK	-0.15948
ENSG0000015	-	-	ENSG0000015515	'TTC39B	-	9:15163621-15307360	-	-	IGV_LINK	382.07	3.73295	0.259412	7.2065	OK	365.054	3.33411	0.000666	6.66755	OK	OK	-0.16302
ENSG0000015	-	-	ENSG0000015518	'AGPAT5	-	8:6708356-6759666	-	-	IGV_LINK	811.656	17.2258	4.20305	30.2485	OK	748.669	16.1517	3.98917	28.3143	OK	OK	-0.09288
ENSG0000015	-	-	ENSG0000015522	'MMS19	-	10:97458323-97498794	-	-	IGV_LINK	1329.49	33.5862	9.88087	57.2915	OK	828.074	20.0959	5.61696	34.5748	OK	OK	-0.74097
ENSG0000015	-	-	ENSG0000015524	'OR4K1	-	14:19930916-19936757	-	-	IGV_LINK	0	0	0	0	OK	0	0	0	0	OK	NOTEST	0
ENSG0000015	-	-	ENSG0000015525	'PI4K2A	-	10:97584322-97676434	-	-	IGV_LINK	623.467	8.74789	2.21388	15.2819	OK	756.919	10.6234	2.92892	18.3179	OK	OK	0.28024
ENSG0000015	-	-	ENSG0000015525	'MARVELD1	-	10:97713172-97718152	-	-	IGV_LINK	599.287	12.8504	3.29259	22.4083	OK	477.457	9.32904	2.88781	15.7703	OK	OK	-0.46202
ENSG0000015	-	-	ENSG0000015525	'ZFYVE27	-	10:97737120-97760907	-	-	IGV_LINK	871.779	20.6639	4.07574	37.252	OK	988.945	23.3479	4.90181	41.7939	OK	OK	0.176179
ENSG0000015	-	-	ENSG0000015526	'GOLGA7B	-	10:97850238-98030828	-	-	IGV_LINK	162.913	1.46323	0.30264	2.62383	OK	217.588	1.95476	0	6.4068	OK	OK	0.417832
ENSG0000015	-	-	ENSG0000015526	'GPR78	-	4:8558724-8619761	-	-	IGV_LINK	25.3402	0.402556	0	1.30728	OK	1.03122	0.074191	0	0.492236	OK	NOTEST	-2.43988
ENSG0000015	-	-	ENSG0000015527	'TRMT44	-	4:8366281-8512610	-	-	IGV_LINK	266.673	8.85309	0	19.7978	OK	216.557	7.30785	0	18.2909	OK	OK	-0.27673
ENSG0000015	-	-	ENSG0000015528	'SLC25A28	-	10:99610521-99620609	-	-	IGV_LINK	733.109	51.251	14.3293	88.1726	OK	459.926	29.451	9.2811	49.6209	OK	OK	-0.79926
ENSG0000015	-	-	ENSG0000015530	'HSPA13	-	21:14371114-14383484	-	-	IGV_LINK	439.283	6.49171	2.30388	10.6795	OK	447.552	6.6159	2.36076	10.871	OK	OK	0.027338
ENSG0000015	-	-	ENSG0000015530	'SAMSN1	-	21:14485227-14658821	-	-	IGV_LINK	329.705	10.9024	3.61727	18.1876	OK	333.086	11.0379	3.5587	18.5171	OK	OK	0.017815
ENSG0000015	-	-	ENSG0000015531	'USP25	-	21:15730024-15880069	-	-	IGV_LINK	883.415	15.0503	3.15449	26.9462	OK	818.793	14.5485	2.52416	26.5728	OK	OK	-0.04893
ENSG0000015	-	-	ENSG0000015532	'GRAMD2B	-	5:126360131-126498604	-	-	IGV_LINK	798.08	26.5295	7.16789	45.8912	OK	966.597	31.6409	9.47164	53.8102	OK	OK	0.254192
ENSG0000015	-	-	ENSG0000015532	'ZCCHC10	-	5:132996984-133026604	-	-	IGV_LINK	180.368	15.9936	0	33.654	OK	168.09	14.284	0	31.9136	OK	OK	-0.1631
ENSG0000015	-	-	ENSG0000015533	'C16orf87	-	16:46796606-46831411	-	-	IGV_LINK	118.306	3.60436	0.154339	7.05437	OK	148.496	4.23693	0.400207	8.07366	OK	OK	0.233278
ENSG0000015	-	-	ENSG0000015536	'MOV10	-	1:112673140-112700746	-	-	IGV_LINK	1935.56	46.2651	6.91732	85.6129	OK	2205.79	53.4688	3.77827	103.159	OK	OK	0.208772
ENSG0000015	-	-	ENSG0000015536	'RHOC	-	1:112701105-112715477	-	-	IGV_LINK	2860.24	172.243	65.1837	279.302	OK	4440.05	270.277	96.1081	444.447	OK	OK	0.649999
ENSG0000015	-	-	ENSG0000015536	'PPM1J	-	1:112701105-112715477	-	-	IGV_LINK	21.6316	0.723682	0	5.86184	OK	18.3053	0.692514	0	7.44056	OK	OK	-0.06351
ENSG0000015	-	-	ENSG0000015536	'DBI	-	2:119366920-119372560	-	-	IGV_LINK	2104.29	254.546	112.377	396.716	OK	2594.56	315.189	137.379	492.999	OK	OK	0.30829
ENSG0000015	-	-	ENSG0000015538	'SLC16A1	-	1:112911846-113073105	-	-	IGV_LINK	1008.51	16.4793	4.64501	28.3137	OK	1302.44	23.2445	4.12852	42.3604	OK	OK	0.496228
ENSG0000015	-	-	ENSG0000015539	'HEATR3	-	16:50046428-50121943	-	-	IGV_LINK	247.279	4.99995	0	10.8079	OK	256.775	5.22937	0	11.1082	OK	OK	0.064723
ENSG0000015	-	-	ENSG0000015542	'TRIM74	-	7:72959484-72969466	-	-	IGV_LINK	2.90916	0.139236	0	0.395759	OK	8.2498	0.395232	0	0.92242	OK	NOTEST	1.50517
ENSG0000015	-	-	ENSG0000015543	'NIFK	-	2:121649649-121736923	-	-	IGV_LINK	468.375	30.0814	2.23997	57.9228	OK	566.143	39.2514	3.84711	74.6556	OK	OK	0.383872
ENSG0000015	-	-	ENSG0000015546	'OXA1L	-	14:22702142-22773041	-	-	IGV_LINK	1695.07	69.0446	26.3484	111.741	OK	1833.52	74.8802	31.3717	118.389	OK	OK	0.117056
ENSG0000015	-	-	ENSG0000015546	'SLC7A7	-	14:22773221-22829820	-	-	IGV_LINK	925.113	27.1103	9.80971	44.4109	OK	2118.14	64.398	22.5767	106.219	OK	OK	1.24818
ENSG0000015	-	-	ENSG0000015549	'MAGEC1	-	X:141903893-141909388	-	-	IGV_LINK	0	0	0	0	OK	0	0	0	0	OK	NOTEST	0
ENSG0000015	-	-	ENSG0000015550	'LARP1	-	5:154712901-154817607	-	-	IGV_LINK	4847.63	52.0698	0.841475	103.298	OK	5534.58	61.8649	0	133.296	OK	OK	0.248675
ENSG0000015	-	-	ENSG0000015550	'CNOT8	-	5:154818490-154876793	-	-	IGV_LINK	1023.06	29.8375	3.51149	56.1636	OK	988.945	27.5521	6.32773	48.7764	OK	OK	-0.11497
ENSG0000015	-	-	ENSG0000015551	'GRIA1	-	5:153489614-153813869	-	-	IGV_LINK	0	0	0	0	OK	1.03122	0.011486	0	0.575214	OK	NOTEST	inf
ENSG0000015	-	-	ENSG0000015553	'LRGUK	-	7:134127298-134264591	-	-	IGV_LINK	63.0318	1.41176	0	3.29191	OK	77.3419	1.45878	0.077941	2.83962	OK	OK	0.047265
ENSG0000015	-	-	ENSG0000015554	'SETD9	-	5:56900040-56971675	-	-	IGV_LINK	80.4868	5.22443	0	12.8781	OK	70.1233	5.16707	0	14.0323	OK	OK	-0.01593
ENSG0000015	-	-	ENSG0000015554	'MIER3	-	5:56900040-56971675	-	-	IGV_LINK	233.703	5.54332	0	15.3292	OK	262.962	5.40453	0	13.8804	OK	OK	-0.03658
ENSG0000015	-	-	ENSG0000015556	'NUP205	-	7:135557918-135648757	-	-	IGV_LINK	848.505	11.5961	1.67969	21.5126	OK	669.265	8.6467	0.517692	16.7757	OK	OK	-0.42342
ENSG0000015	-	-	ENSG0000015559	'ZKSCAN2	-	16:25236000-25257931	-	-	IGV_LINK	139.64	1.07061	0.195584	1.94563	OK	140.247	1.07543	0.200377	1.95048	OK	OK	0.00648
ENSG0000015	-	-	ENSG0000015562	'C9orf85	-	9:71911509-71986054	-	-	IGV_LINK	99.8812	3.97546	0.202734	7.74818	OK	119.622	6.06325	0.48043	11.6461	OK	OK	0.608971
ENSG0000015	-	-	ENSG0000015562	'XAGE2	-	X:52369029-52375680	-	-	IGV_LINK	0	0	0	0	OK	0	0	0	0	OK	NOTEST	0
ENSG0000015	-	-	ENSG0000015562	'PIK3AP1	-	10:96593311-96720514	-	-	IGV_LINK	1667.92	24.7415	8.00032	41.4826	OK	1482.9	23.0484	6.94556	39.1512	OK	OK	-0.10227
ENSG0000015	-	-	ENSG0000015563	'RBM45	-	2:178112423-178139011	-	-	IGV_LINK	261.825	8.58413	1.37993	15.7883	OK	238.213	7.62117	1.20553	14.0368	OK	OK	-0.17166
ENSG0000015	-	-	ENSG0000015565	'TTN	-	2:178521182-179050086	-	-	IGV_LINK	380.13	0.591422	0	3.40752	OK	135.09	0.139985	0	0.760475	OK	OK	-2.07892
ENSG0000015	-	-	ENSG0000015565	'VSIG4	-	X:66021737-66040125	-	-	IGV_LINK	550.801	20.0508	7.69023	32.4113	OK	1476.71	53.8592	22.4763	85.242	OK	OK	1.42553
ENSG0000015	-	-	ENSG0000015566	'PDIA4	-	7:149003061-149028641	-	-	IGV_LINK	1523.43	35.1772	11.5346	58.8198	OK	2738.93	62.051	20.0204	104.082	OK	OK	0.818813
ENSG0000015	-	-	ENSG0000015566	'KDM8	-	16:27203494-27221768	-	-	IGV_LINK	120.245	5.12194	0	11.3685	OK	131.997	4.99483	0	10.6067	OK	OK	-0.03626
ENSG0000015	-	-	ENSG0000015571	'PDZD9	-	16:21983864-22001110	-	-	IGV_LINK	0	0	0	0	OK	1.03122	0.06498	0	0.293894	OK	NOTEST	inf
ENSG0000015	-	-	ENSG0000015571	'OTOA	-	16:21678513-21760729	-	-	IGV_LINK	26.1825	0.43857	0	1.00275	OK	19.5933	0.418115	0	0.955394	OK	NOTEST	-0.06891
ENSG0000015	-	-	ENSG0000015572	'KCTD18	-	2:200488951-200583782	-	-	IGV_LINK	382.07	9.68586	2.14606	17.2257	OK	362.991	9.46608	1.60875	17.3234	OK	OK	-0.03311
ENSG0000015	-	-	ENSG0000015574	'FAM126B	-	2:200908972-201085750	-	-	IGV_LINK	319.038	3.32208	0	12.0555	OK	239.244	1.72701	0	5.67652	OK	OK	-0.94381
ENSG0000015	-	-	ENSG0000015574	'ALS2CR12	-	2:201288270-201357398	-	-	IGV_LINK	14.5458	1.56533	0	3.89249	OK	8.2498	0.370342	0	1.20271	OK	OK	-2.07954

ENSG0000015	-	-	ENSG0000015575	C2CD6	-	2:201487424-201619178	-	-	IGV_LINK	10.6669	0.112584	0	0.697062	OK	4.1249	0.043546	0	0.613413	OK	NOTEST	-1.3704
ENSG0000015	-	-	ENSG0000015575	TMEM237	-	2:201620183-201643570	-	-	IGV_LINK	258.649	6.38294	0.336788	12.4291	OK	253.903	6.89949	0.33498	13.464	OK	OK	0.112268
ENSG0000015	-	-	ENSG0000015576	FZD7	-	2:202034586-202038445	-	-	IGV_LINK	65.941	1.00804	0.00916	2.00693	OK	159.84	2.44424	0.453613	4.43488	OK	OK	1.27783
ENSG0000015	-	-	ENSG0000015576	SPAG17	-	1:117929719-118185223	-	-	IGV_LINK	1301.36	24.9203	0	60.3733	OK	617.704	13.6021	0	36.4288	OK	OK	-0.87349
ENSG0000015	-	-	ENSG0000015579	DEPTOR	-	8:119867418-120050913	-	-	IGV_LINK	361.706	9.9116	1.73604	18.0872	OK	1371.53	35.6349	12.4506	58.8193	OK	OK	1.8461
ENSG0000015	-	-	ENSG0000015581	FMN2	-	1:240014347-240475189	-	-	IGV_LINK	0	0	0	0	OK	0	0	0	0	OK	NOTEST	0
ENSG0000015	-	-	ENSG0000015582	RNF20	-	9:101533850-101563344	-	-	IGV_LINK	1039.54	16.92	3.30817	30.5317	OK	1014.73	15.9268	5.45864	26.395	OK	OK	-0.08727
ENSG0000015	-	-	ENSG0000015583	CYLC2	-	9:102995310-103018488	-	-	IGV_LINK	0	0	0	0	OK	0	0	0	0	OK	NOTEST	0
ENSG0000015	-	-	ENSG0000015584	PPARGC1B	-	5:149730297-149855022	-	-	IGV_LINK	246.309	1.42019	0.332983	2.5074	OK	230.994	1.37258	0.224342	2.52082	OK	OK	-0.0492
ENSG0000015	-	-	ENSG0000015584	ELMO1	-	7:36854360-37449249	-	-	IGV_LINK	829.111	22.1212	2.47581	41.7666	OK	794.043	23.0849	3.84652	42.3232	OK	OK	0.061517
ENSG0000015	-	-	ENSG0000015585	SLC26A2	-	5:149960736-150053142	-	-	IGV_LINK	217.217	1.54807	0	4.46669	OK	201.089	1.43334	0	4.353	OK	OK	-0.1111
ENSG0000015	-	-	ENSG0000015585	LSM11	-	5:157743694-157760709	-	-	IGV_LINK	197.823	1.73882	0.387467	3.09017	OK	150.559	1.32361	0.208647	2.43858	OK	OK	-0.39362
ENSG0000015	-	-	ENSG0000015586	MED7	-	5:157085831-157255191	-	-	IGV_LINK	302.553	10.9043	0	27.905	OK	280.493	10.99	0	26.2408	OK	OK	0.011293
ENSG0000015	-	-	ENSG0000015587	SAXO1	-	9:18927657-19049354	-	-	IGV_LINK	1.93944	0.07031	0	0.343253	OK	4.1249	0.149782	0	0.538822	OK	NOTEST	1.09107
ENSG0000015	-	-	ENSG0000015587	RRAGA	-	9:19049394-19050983	-	-	IGV_LINK	1192.76	47.4546	20.6627	74.2466	OK	2158.35	85.9414	37.529	134.354	OK	OK	0.856804
ENSG0000015	-	-	ENSG0000015588	SLC24A2	-	9:19507451-19786928	-	-	IGV_LINK	0	0	0	0	OK	0	0	0	0	OK	NOTEST	0
ENSG0000015	-	-	ENSG0000015589	TRIM42	-	3:140678038-140701150	-	-	IGV_LINK	1.93944	0.046186	0	0.141442	OK	1.03122	0.02457	0	0.091959	OK	NOTEST	-0.91058
ENSG0000015	-	-	ENSG0000015589	PXYLP1	-	3:141228725-141449792	-	-	IGV_LINK	307.401	7.29827	0	18.1589	OK	355.773	9.53547	0	27.6991	OK	OK	0.38575
ENSG0000015	-	-	ENSG0000015589	ADCY8	-	8:130780300-131042426	-	-	IGV_LINK	0	0	0	0	OK	21.6557	0.364366	0	0.811285	OK	NOTEST	inf
ENSG0000015	-	-	ENSG0000015590	RASA2	-	3:141487046-141615342	-	-	IGV_LINK	366.554	4.72894	0.331418	9.12647	OK	270.181	2.93322	0.190055	5.67638	OK	OK	-0.68903
ENSG0000015	-	-	ENSG0000015590	RMND1	-	6:151404762-151452181	-	-	IGV_LINK	442.193	15.5305	5.65393	25.4072	OK	284.618	9.8668	3.08714	16.6465	OK	OK	-0.65445
ENSG0000015	-	-	ENSG0000015591	RAET1L	-	6:150018333-150025532	-	-	IGV_LINK	0.969721	0.049124	0	0.249973	OK	0	0	0	0	OK	NOTEST	'-inf
ENSG0000015	-	-	ENSG0000015592	SLA	-	8:132866957-133134903	-	-	IGV_LINK	1185	28.7711	7.38847	50.1537	OK	1007.51	23.3149	5.25406	41.3757	OK	OK	-0.30337
ENSG0000015	-	-	ENSG0000015595	TMBIM4	-	12:66116554-66170072	-	-	IGV_LINK	1010.68	79.8199	16.4343	143.206	OK	1169.83	93.6958	16.0714	171.32	OK	OK	0.231237
ENSG0000015	-	-	ENSG0000015595	VBP1	-	X:155197006-155239817	-	-	IGV_LINK	356.857	16.6215	3.82401	29.4191	OK	344.429	15.7207	3.67033	27.771	OK	OK	-0.08039
ENSG0000015	-	-	ENSG0000015596	RAB39B	-	X:155258240-155264589	-	-	IGV_LINK	38.7888	0.655956	0	1.32985	OK	25.7806	0.436126	0	0.904938	OK	OK	-0.58886
ENSG0000015	-	-	ENSG0000015596	CLIC2	-	X:155276210-155334657	-	-	IGV_LINK	330.675	12.5165	0.512283	24.5207	OK	390.834	13.9697	1.62336	26.316	OK	OK	0.158465
ENSG0000015	-	-	ENSG0000015596	AFF2	-	X:148500618-149000663	-	-	IGV_LINK	104.73	0.625561	0.052662	1.19846	OK	145.403	0.889431	0	1.90384	OK	OK	0.507731
ENSG0000015	-	-	ENSG0000015597	MICU3	-	8:17027237-17122644	-	-	IGV_LINK	104.73	3.21598	0	9.98144	OK	48.4676	1.12808	0	3.27219	OK	OK	-1.51139
ENSG0000015	-	-	ENSG0000015597	GRIP1	-	12:66302544-66804186	-	-	IGV_LINK	95.0326	3.68665	0	18.9275	OK	100.029	3.20825	0	9.34747	OK	OK	-0.20052
ENSG0000015	-	-	ENSG0000015597	VPS37A	-	8:17224963-17413528	-	-	IGV_LINK	555.65	21.466	0	50.3818	OK	814.668	31.3464	0	68.4591	OK	OK	0.546247
ENSG0000015	-	-	ENSG0000015598	KIF5A	-	12:57549997-57586632	-	-	IGV_LINK	18.4247	0.3196	0	1.08835	OK	5.15612	0.051427	0	0.273441	OK	NOTEST	-2.63568
ENSG0000015	-	-	ENSG0000015600	NAT2	-	8:18391244-18401218	-	-	IGV_LINK	0	0	0	0	OK	6.18735	0.303652	0	0.77579	OK	NOTEST	inf
ENSG0000015	-	-	ENSG0000015600	MAGEA8	-	X:149881140-149885835	-	-	IGV_LINK	1.93944	0.095779	0	0.38434	OK	1.03122	0.036223	0	0.294445	OK	NOTEST	-1.40282
ENSG0000015	-	-	ENSG0000015601	PSD3	-	8:18527300-19084730	-	-	IGV_LINK	80.4868	0.731857	0	2.91992	OK	193.87	1.48866	0	4.54943	OK	OK	1.02438
ENSG0000015	-	-	ENSG0000015601	CARNMT1	-	9:74952967-75028423	-	-	IGV_LINK	174.55	2.56519	0.494106	4.63627	OK	180.464	3.04509	0.012859	6.07731	OK	OK	0.247419
ENSG0000015	-	-	ENSG0000015602	MCU	-	10:72692130-72887694	-	-	IGV_LINK	292.856	6.70892	1.4028	12.015	OK	219.651	5.62835	0.238947	11.0177	OK	OK	-0.25337
ENSG0000015	-	-	ENSG0000015603	ELMSAN1	-	14:73715121-73803270	-	-	IGV_LINK	943.538	11.6536	0	25.3622	OK	829.105	9.76407	0	20.06	OK	OK	-0.25522
ENSG0000015	-	-	ENSG0000015603	CFAP70	-	10:73110374-73358859	-	-	IGV_LINK	538.195	10.4371	0	27.5662	OK	207.276	4.58893	0	14.1806	OK	OK	-1.1855
ENSG0000015	-	-	ENSG0000015604	GNA14	-	9:77423078-77648307	-	-	IGV_LINK	41.698	1.18604	0	2.61326	OK	127.872	3.12187	0.539514	5.70422	OK	OK	1.39625
ENSG0000015	-	-	ENSG0000015605	FAM161B	-	14:73851843-74084493	-	-	IGV_LINK	315.159	11.3663	0	29.1423	OK	352.641	12.3796	0	32.1116	OK	OK	0.123205
ENSG0000015	-	-	ENSG0000015605	GNAQ	-	9:77716086-78031458	-	-	IGV_LINK	1231.55	15.1473	0	32.5161	OK	1230.25	15.1688	0	32.6082	OK	OK	0.002049
ENSG0000015	-	-	ENSG0000015607	WIF1	-	12:65017467-65121566	-	-	IGV_LINK	0	0	0	0	OK	0	0	0	0	OK	NOTEST	0
ENSG0000015	-	-	ENSG0000015609	UGT2B4	-	4:69480164-69526014	-	-	IGV_LINK	0	0	0	0	OK	0	0	0	0	OK	NOTEST	0
ENSG0000015	-	-	ENSG0000015609	GPR61	-	1:109539871-109548406	-	-	IGV_LINK	10.6669	1.29412	0	2.95617	OK	3.09367	0.277031	0	0.85058	OK	OK	-2.22385
ENSG0000015	-	-	ENSG0000015610	MMP16	-	8:88032008-88737134	-	-	IGV_LINK	7.75776	0.04129	0	0.338973	OK	13.4059	0.130692	0	0.509168	OK	NOTEST	1.6623
ENSG0000015	-	-	ENSG0000015611	ADK	-	10:74151184-74709303	-	-	IGV_LINK	259.885	12.7673	2.58831	22.9463	OK	210.37	10.2139	2.35488	18.0728	OK	OK	-0.32193
ENSG0000015	-	-	ENSG0000015611	KCNMA1	-	10:76869600-77638369	-	-	IGV_LINK	815.535	16.045	3.13759	28.9524	OK	703.295	14.1252	2.36557	25.8849	OK	OK	-0.18385
ENSG0000015	-	-	ENSG0000015612	BATF	-	14:75522424-75547015	-	-	IGV_LINK	182.307	12.7237	2.92143	22.5259	OK	94.8727	6.63084	0.60064	12.661	OK	OK	-0.94025
ENSG0000015	-	-	ENSG0000015613	DCK	-	4:70902325-71030914	-	-	IGV_LINK	486.8	11.4538	0	23.5232	OK	311.43	7.33756	0	16.2408	OK	OK	-0.64246
ENSG0000015	-	-	ENSG0000015614	ADAMTS3	-	4:72280968-72569386	-	-	IGV_LINK	79.5171	1.29236	0	3.1017	OK	16.4996	0.149651	0	0.420678	OK	OK	-3.11034
ENSG0000015	-	-	ENSG0000015615	ALX3	-	1:110031576-110074641	-	-	IGV_LINK	46.5466	1.55541	0	5.18963	OK	48.4676	1.6207	0	5.3533	OK	OK	0.059329
ENSG0000015	-	-	ENSG0000015616	DPY19L4	-	8:94719702-94793836	-	-	IGV_LINK	460.617	13.4792	0	29.7037	OK	407.334	9.62856	0	22.8874	OK	OK	-0.48534
ENSG0000015	-	-	ENSG0000015617	NDUFAF6	-	8:94813310-95116455	-	-	IGV_LINK	249.218	15.2388	0	45.027	OK	266.056	16.3841	0	54.4709	OK	OK	0.104544
ENSG0000015	-	-	ENSG0000015617	DRAM2	-	1:111117332-111185102	-	-	IGV_LINK	1066.69	65.806	7.05105	124.561	OK	1036.38	65.5882	3.75279	127.424	OK	OK	-0.00478
ENSG0000015	-	-	ENSG0000015617	C8orf37	-	8:95204455-95810136	-	-	IGV_LINK	130.912	2.3336	0.39822	4.26897	OK	142.309	2.53768	0.44804	4.62731	OK	OK	0.120952
ENSG0000015	-	-	ENSG0000015619	PPEF2	-	4:75859863-75902571	-	-	IGV_LINK	8.72748	0.13641	0	0.820096	OK	3.09367	0.073995	0	0.712089	OK	NOTEST	-0.88245
ENSG0000015	-	-	ENSG0000015620	CFAP161	-	15:81007032-81149175	-	-	IGV_LINK	2.90916	0.295269	0	0.939981	OK	4.1249	0.341834	0	1.01042	OK	NOTEST	0.211265
ENSG0000015	-	-	ENSG0000015621	ADAMTSL3	-	15:83654085-84039842	-	-	IGV_LINK	353.948	3.22367	0	6.53235	OK	506.331	5.05045	0	11.1514	OK	OK	0.647709
ENSG0000015	-	-	ENSG0000015621	ART3	-	4:76011183-76112802	-	-	IGV_LINK	11.6366	1.22695	0	16.2248	OK	2.06245	0.144248	0	2.17303	OK	OK	-3.08846
ENSG0000015	-	-	ENSG0000015622	SLC28A1	-	15:84884653-84945796	-	-	IGV_LINK	3612.21	97.1994	37.3919	157.007	OK	4077.46	111.46	40.1916	182.728	OK	OK	0.197506
ENSG0000015	-	-	ENSG0000015623	WHAMM	-	15:82809627-82836108	-	-	IGV_LINK	457.708	6.11239	0.574259	11.6505	OK	633.172	8.21554	1.37392	15.0572	OK	OK	0.426619
ENSG0000015	-	-	ENSG0000015623	CXCL13	-	4:77511752-77611834	-	-	IGV_LINK	866.93	49.7086	20.1363	79.2809	OK	89.7166	4.91198	0.486277	9.33769	OK	OK	-3.33912
ENSG0000015	-	-	ENSG0000015623	N6AMT1	-	21:28872190-28885371	-	-	IGV_LINK	243.4	4.68785	0.049332	9.32637	OK	234.088	3.92507	0.32212	7.52801	OK	OK	-0.25621
ENSG0000015	-	-	ENSG0000015625	RWDD2B	-	21:29004383-29019378	-	-	IGV_LINK	313.22	11.4695	3.29718	19.6417	OK	329.992	11.9852	3.26315	20.7073	OK	OK	0.063462
ENSG0000015	-	-	ENSG0000015625	USP16	-	21:29024254-29054488	-	-	IGV_LINK	791.292	17.0172	6.60034	27.4342	OK	738.357	15.7002	6.31106	25.0894	OK	OK	-0.11621
ENSG0000015	-	-	ENSG0000015626	CCT8	-	21:29055804-29073797	-	-	IGV_LINK	2462.12	77.9723	33.636	122.309	OK	2352.22	73.4336	31.7273	115.14	OK	OK	-0.08652
ENSG0000015	-	-	ENSG0000015626	MAP3K7CL	-	21:29077470-29175889	-	-	IGV_LINK	490.679	25.9921	6.05593	45.9283	OK	2062.45	114.325	42.1457	186.505	OK	OK	2.137
ENSG0000015	-	-	ENSG0000015626	NAA11	-	4:78971747-79326050	-	-	IGV_LINK	0	0	0	0	OK	0	0	0	0	OK	NOTEST	0
ENSG0000015	-	-	ENSG0000015627	BACH1	-	21:29193479-29940033	-	-	IGV_LINK	732.139	9.88871	0.282178	19.4952	OK	1658.21	18.2245	5.24607	31.203	OK	OK	0.882027
ENSG0000015	-	-	ENSG0000015628	CLDN17	-	21:30165563-30166756	-	-	IGV_LINK	0	0	0	0	OK	0	0	0	0	OK	NOTEST	0
ENSG0000015	-	-	ENSG0000015628	CLDN8	-	21:30214005-30216073	-	-	IGV_LINK	0	0	0	0	OK	0	0	0	0	OK	NOTEST	0
ENSG0000015	-	-	ENSG0000015629	TSPAN7	-	X:37349274-38688920	-	-	IGV_LINK	290.998	9.44484	0	30.8791	OK	152.862	5.64833	0	24.5267	OK	OK	-0.7417
ENSG0000015	-	-	ENSG0000015629	TIAM1	-	21:31118415-31560487	-	-	IGV_LINK	570.196	7.05555	1.00951	13.1016	OK	286.681	3.63586	0.251799	7.01992	OK	OK	-0.95646
ENSG0000015	-	-	ENSG0000015630	SCAF4	-	21:31671032-31732075	-	-	IGV_LINK	597.348	9.83075	2.66368	16.9978	OK	692.983	10.9827	3.82902	18.1364	OK	OK	0.159861
ENSG0000015	-	-	ENSG0000015631	RPGR	-	X:37349274-38688920	-	-	IGV_LINK	119.276	4.58755	0	33.0608	OK	104.154	4.78188	0	37.354	OK	OK	0.059854
ENSG0000015	-	-	ENSG0000015634	CDK20	-	9:87966440-87974753	-	-	IGV_LINK	690.441	14.6814	5.34869	24.0141	OK	625.954	14.9444	4.35421	25.5345	OK	OK	0.025613
ENSG0000015	-	-	ENSG0000015637	PCGF6	-	10:103302795-103351134	-	-	IGV_LINK	72.729	2.21854	0	4.44721	OK	65.9984	1.96375	0	4.27817	OK	OK	-0.176
ENSG0000015	-	-	ENSG0000015638	ANKRD9	-	14:102362962-102509799	-	-	IGV_LINK	102.79	1.59843	0	4.57594	OK	127.872	2.03948	0	5.31284	OK	OK	0.351543
ENSG0000015	-	-	ENSG0000015638	SFR1	-	10:104122057-104126385	-	-	IGV_LINK	171.641	7.52929	1.72631	13.3323	OK	83.5292	3.82032	0.126604	7.51404	OK	OK	-0.97882
ENSG0000015	-	-	ENSG0000015639	SORCS3	-	10:104641100-105265235	-	-	IGV_LINK	833.96	8.56946	3.56634	13.5726	OK	262.962	2.65475	0.7827	4.5268	OK	OK	-1.69063
ENSG0000015	-	-	ENSG0000015639	SFXN2	-	10:102714537-102743492	-	-	IGV_LINK	208.49	6.4111	0	13.6488	OK	174.277	5.01879	0	10.8363	OK	OK	-0.35323
ENSG0000015	-	-	ENSG0000015641	C14orf2	-	14:103912287-103928269	-	-	IGV_LINK	606.075	71.301	22.8858	119.716	OK	614.61	71.3614	26.0823	116.64	OK	OK	0.001221
ENSG0000015	-	-	ENSG0000015641	FUT6	-	19:5830609-5839731	-	-	IGV_LINK	286.068	10.1208	1.66467	18.5769	OK	239.244	9.27982	0.056538	18.5031	OK	OK	-0.12515
ENSG0000015	-	-	ENSG0000015641	TDRD9	-	14:103928461-104052667	-	-	IGV_LINK	17.455	0.520649	0	1.55728	OK	16.4996	0.499341	0	1.46288	OK	NOTEST	-0.06028
ENSG0000015	-	-	ENSG0000015642	FGF18	-	5:171419655-171457623	-	-	IGV_LINK	0	0	0	0	OK	11.3435	0.352733	0	0.790425	OK	NOTEST	inf
ENSG0000015	-	-	ENSG0000015645	PCDH1	-	5:141853110-141879246	-	-	IGV_LINK	231.763	3.51879	0.944965	6.09261	OK	537.268	9.53884	2.15827	16.9194	OK	OK	1.43873
ENSG0000015	-	-	ENSG0000015646	SH3RF2	-	5:145858520-146081791	-	-	IGV_LINK	7.75776	0.132527	0	0.635633	OK	433.114	7.16056	0.522014	13.7991	OK	OK	5.75571
ENSG0000015	-	-	ENSG0000015646	GDF6	-	8:96142329-96160792	-	-	IGV_LINK	190.065	3.13984	0.612563	5.66712	OK	8.2498	0.169309	0	0.478309	OK	OK	-4.21296
ENSG0000015	-	-	ENSG0000015646	UQCRB	-	8:96225919-96238034	-	-	IGV_LINK	5054.18	407.943	98.9979	716.887	OK	3911.44	316.28	89.3965	543.163	OK	OK	-0.36717
ENSG0000015	-	-	ENSG0000015646	MTERF3	-	8:96239397-96261610	-	-	IGV_LINK	204.611	9.97587	2.04623	17.9055	OK	253.681	12.0751	2.87321	21.2769	OK	OK	0.275518
ENSG0000015	-	-	ENSG0000015647	PTDSS1	-	8:96261714-96336995	-	-	IGV_LINK	996.873	37.6766	11.7502	63.603	OK	1161.16	44.7901	13.5366	76.0437	OK	OK	0.249512
ENSG0000015	-	-	ENSG0000015647	PPP2R2B	-	5:146563225-147084784	-	-	IGV_LINK	62.0621	1.9553	0	4.91439	OK	25.7806	0.788238	0	2.16691	OK	OK	-1.31069
ENSG0000015	-	-	ENSG0000015648	RPL30	-	8:97868839-98046469	-	-	IGV_LINK	17214.5	2642.58	533.857	4751.29	OK	13956.6	2156.2	568.716	3743.68	OK	OK	-0.29346
ENSG0000015	-	-	ENSG0000015648	KCNS2	-	8:98401402-98942827	-	-	IGV_LINK	5.81832	0.064975	0	0.440984	OK	4.1249	0.046074	0	0.359425	OK	NOTEST	-0.49592
ENSG0000015	-	-	ENSG0000015650	FAM122C	-	X:134769565-134854610	-	-	IGV_LINK	91.1537	5.89111	0	17.8752	OK	87.6541	5.42443	0	16.0269	OK	OK	-0.11907
ENSG0000015	-	-	ENSG0000015650	SUPV3L1	-	10:69180230-69209099	-	-	IGV_LINK	409.222	18.1061	2.97501	33.2372	OK	271.212	12.3608	1.1785	23.543	OK	OK	-0.55071
ENSG0000015	-	-	ENSG0000015650	FAM122B	-	X:134769565-134854610	-	-	IGV_LINK	637.106	18.1701	1.74677	34.5935	OK	682.671	19.015	1.25417	36.7757	OK	OK	0.065566
ENSG0000015	-	-	ENSG0000015650	EEF1A1	-	6:73515749-73570596	-	-	IGV_LINK	212262	6320.01	0	19029.9	OK	98626.4	2931.23	0	7158.14	OK	OK	-1.10842
ENSG0000015	-	-	ENSG0000015650	FBXO43	-	8:100133359-100145800	-	-	IGV_LINK	38.7888	0.789502	0	1.61779	OK	36.0929	0.721793	0	1.58658	OK	OK	-0.12936
ENSG0000015	-	-	ENSG0000015651	HKDC1	-	10:69215332-69268148	-	-	IGV_LINK	950.326	17.3817	4.04703	30.7165	OK	610.485	11.385	2.04391	20.7261	OK	OK	-0.61044
ENSG0000015	-	-	ENSG0000015651	HK1	-	10:69269983-69401882	-	-	IGV_LINK	2290.48	38.0263	15.2416	60.8111	OK	1813.92	31.6017	12.1621	51.0413	OK	OK	-0.267
ENSG0000015	-	-	ENSG0000015652	TYSND1	-	10:70137980-70146676	-	-	IGV_LINK	385.949	6.88989	2.43489	11.3449	OK	250.588	4.34822	1.27957	7.41686	OK	OK	-0.66406
ENSG0000015	-	-	ENSG0000015653	PHF6	-	X:134373252-134428791	-	-	IGV_LINK	224.005	2.91735	0.586655	5.24804	OK	265.025	3.84212	0.728067	6.95617	OK	OK	0.397244
ENSG0000015	-	-	ENSG0000015653	CD109	-	6:73693902-73828316	-	-	IGV_LINK	1051.18	6.60371	2.63071	10.5767	OK	968.32	6.07808	2.56174	9.59442	OK	OK	-0.11966
ENSG0000015	-	-	ENSG0000015656	LRFN2	-	6:40391585-40587465	-	-	IGV_LINK	0.969721	0.017643	0	0.069103	OK	1.03122	0.018769	0	0.070249	OK	NOTEST	0.089254
ENSG0000015	-	-	ENSG0000015657	NODAL	-	10:70432314-70447951	-	-	IGV_LINK	21.3339	0.805561	0	2.33889	OK	3.09367	0.116907	0	0.44558	OK	OK	-2.78463
ENSG0000015	-	-	ENSG0000015657	PRG3	-	11:57376768-57381150	-	-	IGV_LINK	0	0	0	0	OK	0	0	0	0	OK	NOTEST	0
ENSG0000015	-	-	ENSG0000015658	UBE2L6	-	11:57551655-57568284	-	-	IGV_LINK	2528.06	121.763	51.6776	191.848	OK	2138.76	102.208	44.6136	159.803	OK	OK	-0.25256
ENSG0000015	-	-	ENSG0000015659	ZDHHC5	-	11:57667746-57701187	-	-	IGV_LINK	1668.89	23.1486	6.99125	39.306	OK	2461.53	34.5364	10.5944	58.4784	OK	OK	0.577191
ENSG0000015	-	-	ENSG0000015660	MED19	-	11:57703713-57712323	-	-	IGV_LINK	162.913	8.80346	1.44667	16.1602	OK	237.182	13.372	3.80136	22.9427	OK	OK	0.603075
ENSG0000015	-	-	ENSG0000015663	ZFAND3	-	6:37819498-38154624	-	-	IGV_LINK	1412.88	56.9263	0	123.904	OK	1093.1	49.0468	6.41057	91.683	OK	OK	-0.21494
ENSG0000015	-	-	ENSG0000015664	NPTN	-	15:73443157-73634134	-	-	IGV_LINK	1616.52	96.6066	36.2152	156.998	OK	1744.83	103.048	39.8294	166.266	OK	OK	0.093118
ENSG0000015	-	-	ENSG0000015665	KAT6B	-	10:74825581-75032622	-	-	IGV_LINK	668.137	5.78989	2.14013	9.43966	OK	418.677	3.5818	1.1988	5.96481	OK	OK	-0.69285
ENSG0000015	-	-	ENSG0000015667	SAMD8	-	10:75094431-75182123	-	-	IGV_LINK	408.252	4.10241	0.955767	7.24906	OK	368.147	3.13734	0.987581	5.2871	OK	OK	-0.38693
ENSG0000015	-	-	ENSG0000015667	RAB11FIP1	-	8:37858617-37899467	-	-	IGV_LINK	513.952	8.47633	0.071327	16.8813	OK	538.299	8.12954	0.876993	15.3821	OK	OK	-0.06027
ENSG0000015	-	-	ENSG0000015668	UNC5D	-	8:35235456-35796550	-	-	IGV_LINK	75.6382	0.565541	0	1.16434	OK	3.09367	0.025108	0	0.130403	OK	OK	-4.49343
ENSG0000015	-	-	ENSG0000015668	GLYATL2	-	11:58834064-58904215	-	-	IGV_LINK	22.3036	1.14009	0	2.64255	OK	23.7182	1.64952	0	3.93504	OK	OK	0.532889
ENSG0000015	-	-	ENSG0000015669	UTP14A	-	X:129869063-129957528	-	-	IGV_LINK	397.585	9.80161	3.32909	16.2741	OK	342.367	9.05998	2.75679	15.3632	OK	OK	-0.11351
ENSG0000015	-	-	ENSG0000015670	AIFM1	-	X:130129361-130165887	-	-	IGV_LINK	834.929	23.5609	9.23876	37.883	OK	1314.81	36.958	15.3715	58.5445	OK	OK	0.649493
ENSG0000015	-	-	ENSG0000015671	MAPK13	-	6:36127808-36144524	-	-	IGV_LINK	991.054	17.3186	4.70252	29.9347	OK	963.164	17.7224	3.59864	31.8462	OK	OK	0.033251
ENSG0000015	-	-	ENSG0000015673	BAG4	-	8:38105241-38213301	-	-	IGV_LINK	223.036	5.12151	0	16.2282	OK	278.431	5.0994	0	13.8573	OK	OK	-0.00624
ENSG0000015	-	-	ENSG0000015673	MS4A1	-	11:60455751-60470760	-	-	IGV_LINK	128.003	6.05035	0	13.8967	OK	284.618	12.589	0	25.9962	OK	OK	1.05708
ENSG0000015	-	-	ENSG0000015675	IGKV1OR-2	-	9:64765053-64765535	-	-	IGV_LINK	0	0	0	0	OK	0	0	0	0	OK	NOTEST	0
ENSG0000015	-	-	ENSG0000015678	TBC1D31	-	8:123002559-123152153	-	-	IGV_LINK	305.462	24.7222	0	86.4907	OK	509.425	40.5092	0	146.979	OK	OK	0.712439
ENSG0000015	-	-	ENSG0000015679	WDYHV1	-	8:123416724-123467230	-	-	IGV_LINK	174.55	10.7874	2.03584	19.539	OK	141.278	7.52238	1.31918	13.7256	OK	OK	-0.52009
ENSG0000015	-	-	ENSG0000015680	ATAD2	-	8:123319849-123416350	-	-	IGV_LINK	376.252	4.03218	1.35652	6.70783	OK	334.117	3.60939	1.02947	6.18931	OK	OK	-0.1598
ENSG0000015	-	-	ENSG0000015680	FBXO32	-	8:123497888-123541206	-	-	IGV_LINK	261.825	4.83033	0	10.2549	OK	294.93	5.54082	0	11.8696	OK	OK	0.197977
ENSG0000015	-	-	ENSG0000015683	NSMCE2	-	8:125024259-125367120	-	-	IGV_LINK	471.284	29.5782	0	61.4522	OK	333.086	21.841	0	50.407	OK	OK	-0.4375
ENSG0000015	-	-	ENSG0000015685	ZNF689	-	16:30585906-30624012	-	-	IGV_LINK	213.339	3.63273	0.924525	6.34094	OK	197.995	4.25472	0.080443	8.429	OK	OK	0.228009
ENSG0000015	-	-	ENSG0000015685	PRR14	-	16:30650716-30656440	-	-	IGV_LINK	886.325	31.2562	10.0906	52.4218	OK	712.576	27.4956	8.4536	46.5376	OK	OK	-0.18494
ENSG0000015	-	-	ENSG0000015686	FBRS	-	16:30658430-30670814	-	-	IGV_LINK	1857.01	42.6199	4.67023	80.5695	OK	1842.8	42.5486	2.35849	82.7387	OK	OK	-0.00241

ENSG0000015	-	-	ENSG0000015686	'FRRS1	-	1:99708702-99766631	-	-	IGV_LINK	103.76	2.33389	0	5.31015	OK	123.747	3.85471	0	8.94828	OK	OK	0.723887
ENSG0000015	-	-	ENSG0000015687	'PHKG2	-	16:30697706-30776307	-	-	IGV_LINK	626.623	25.3264	0	73.6577	OK	650.883	26.6696	0	84.2139	OK	OK	0.074553
ENSG0000015	-	-	ENSG0000015687	'MFSD14A	-	1:99968382-100132955	-	-	IGV_LINK	635.674	14.0855	1.5187	26.6524	OK	566.98	12.2737	1.17978	23.3676	OK	OK	-0.19865
ENSG0000015	-	-	ENSG0000015687	'SASS6	-	1:99968382-100132955	-	-	IGV_LINK	95.0326	1.44454	0	4.51025	OK	35.0616	0.533119	0	2.3597	OK	OK	-1.43808
ENSG0000015	-	-	ENSG0000015688	'COX6A2	-	16:31427730-31428646	-	-	IGV_LINK	1.93944	0.602013	0	2.26419	OK	3.09367	0.654248	0	2.22503	OK	OK	0.120043
ENSG0000015	-	-	ENSG0000015688	'ITGAD	-	16:31393311-31426505	-	-	IGV_LINK	54.3043	0.917714	0	1.96552	OK	61.8735	1.08947	0	2.76384	OK	OK	0.24751
ENSG0000015	-	-	ENSG0000015692	'ADGRG4	-	X:136300962-136416888	-	-	IGV_LINK	0.969721	0.006211	0	0.030773	OK	27.8431	0.165167	0	0.341537	OK	NOTEST	4.73305
ENSG0000015	-	-	ENSG0000015692	'ZIC3	-	X:137566141-137981763	-	-	IGV_LINK	0.969721	0.016578	0	0.333238	OK	1.03122	0.032388	0	0.354244	OK	NOTEST	0.96619
ENSG0000015	-	-	ENSG0000015692	'MALSU1	-	7:23298738-23470467	-	-	IGV_LINK	300.613	34.5522	4.91817	64.1863	OK	365.054	40.2176	5.55133	74.8839	OK	OK	0.219049
ENSG0000015	-	-	ENSG0000015693	'VPS8	-	3:184812142-185052614	-	-	IGV_LINK	864.021	13.1473	0.595443	25.6991	OK	966.258	16.8896	0	34.7774	OK	OK	0.361378
ENSG0000015	-	-	ENSG0000015695	'GALK2	-	15:49106067-49620931	-	-	IGV_LINK	346.19	17.8042	0	44.2466	OK	356.804	22.5844	0	57.0521	OK	OK	0.343107
ENSG0000015	-	-	ENSG0000015695	'LHFPL4	-	3:9498360-9553802	-	-	IGV_LINK	0.969721	0.011609	0	0.464868	OK	1.03122	0.012348	0	0.46646	OK	NOTEST	0.089071
ENSG0000015	-	-	ENSG0000015696	'B3GNT7	-	2:231395542-231401164	-	-	IGV_LINK	22.3036	0.36804	0	0.827305	OK	80.4355	1.28329	0.078496	2.48808	OK	OK	1.80191
ENSG0000015	-	-	ENSG0000015696	'MPV17L	-	16:15395753-15643166	-	-	IGV_LINK	554.68	11.4006	0	42.2922	OK	2488.73	48.7542	0	120.789	OK	OK	2.09641
ENSG0000015	-	-	ENSG0000015697	'BUB1B	-	15:40161022-40277487	-	-	IGV_LINK	152.246	3.53614	0	8.34273	OK	174.277	3.98312	0	9.17917	OK	OK	0.171726
ENSG0000015	-	-	ENSG0000015697	'PDE6D	-	2:231732424-231809254	-	-	IGV_LINK	278.31	16.0761	0	33.8695	OK	315.555	17.9164	0.20292	35.6298	OK	OK	0.156363
ENSG0000015	-	-	ENSG0000015697	'EIF4A2	-	3:186781779-186807058	-	-	IGV_LINK	8739.12	293.766	69.5487	517.983	OK	7093.76	236.302	52.2397	420.365	OK	OK	-0.31403
ENSG0000015	-	-	ENSG0000015698	'BRPF1	-	3:9731728-9748018	-	-	IGV_LINK	420.859	6.61878	0.020562	13.217	OK	353.71	5.27099	0.181669	10.3603	OK	OK	-0.32849
ENSG0000015	-	-	ENSG0000015699	'RPUSD3	-	3:9749943-9855138	-	-	IGV_LINK	293.825	15.6038	0	64.5693	OK	318.649	17.6517	0	70.347	OK	OK	0.17791
ENSG0000015	-	-	ENSG0000015700	'SST	-	3:187668905-187670399	-	-	IGV_LINK	0	0	0	0	OK	0	0	0	0	OK	NOTEST	0
ENSG0000015	-	-	ENSG0000015701	'TATDN2	-	3:10248022-10321178	-	-	IGV_LINK	924.905	15.7196	0	32.6991	OK	900.476	15.3556	0	34.4214	OK	OK	-0.0338
ENSG0000015	-	-	ENSG0000015701	'GHRL	-	3:10248022-10321178	-	-	IGV_LINK	54.3043	4.00045	0	18.9276	OK	42.2802	2.95431	0	15.4636	OK	OK	-0.43734
ENSG0000015	-	-	ENSG0000015702	'SEC13	-	3:10248022-10321178	-	-	IGV_LINK	750.564	35.897	0	72.5405	OK	906.447	43.3457	4.14328	82.5481	OK	OK	0.272025
ENSG0000015	-	-	ENSG0000015702	'FAM92A1P1	-	15:41163161-41164374	-	-	IGV_LINK	0	0	0	0	OK	0	0	0	0	OK	NOTEST	0
ENSG0000015	-	-	ENSG0000015703	'EXOG	-	3:38496126-38542161	-	-	IGV_LINK	104.73	3.25704	0	6.74731	OK	128.903	4.77574	0	15.3469	OK	OK	0.55216
ENSG0000015	-	-	ENSG0000015704	'NTAN1	-	16:14974590-15154564	-	-	IGV_LINK	596.378	41.8476	0	123.151	OK	592.954	36.0935	0	110.25	OK	OK	-0.21341
ENSG0000015	-	-	ENSG0000015706	'SHCBP1L	-	1:182899864-182953525	-	-	IGV_LINK	0	0	0	0	OK	0	0	0	0	OK	NOTEST	0
ENSG0000015	-	-	ENSG0000015706	'NMNAT2	-	1:183248236-183418602	-	-	IGV_LINK	21.3339	0.36537	0	1.12006	OK	9.28102	0.143135	0	0.524178	OK	NOTEST	-1.35198
ENSG0000015	-	-	ENSG0000015707	'ZFYVE9	-	1:52142093-52366193	-	-	IGV_LINK	417.95	4.94053	0	10.5201	OK	420.74	5.18432	0	12.3469	OK	OK	0.069488
ENSG0000015	-	-	ENSG0000015708	'ATP2B2	-	3:10324022-10708031	-	-	IGV_LINK	324.856	4.31275	0	11.2086	OK	27.8431	0.418418	0	1.56412	OK	OK	-3.36559
ENSG0000015	-	-	ENSG0000015709	'LYZL4	-	3:42397077-42410600	-	-	IGV_LINK	0	0	0	0	OK	0	0	0	0	OK	NOTEST	0
ENSG0000015	-	-	ENSG0000015710	'SLC6A1	-	3:10992723-11039247	-	-	IGV_LINK	170.671	2.64798	0	6.08021	OK	107.247	1.51908	0	3.12389	OK	OK	-0.8017
ENSG0000015	-	-	ENSG0000015710	'SMG1	-	16:18803082-18926454	-	-	IGV_LINK	2526.12	10.4028	0	21.022	OK	1847.96	8.61418	0	20.5667	OK	OK	-0.27219
ENSG0000015	-	-	ENSG0000015710	'FCHO2	-	5:72955980-73090522	-	-	IGV_LINK	790.322	12.4472	4.39748	20.4969	OK	963.164	17.3011	2.9493	31.653	OK	OK	0.475048
ENSG0000015	-	-	ENSG0000015711	'RBPMS	-	8:30382118-30572261	-	-	IGV_LINK	3659.73	156.887	34.3732	279.401	OK	3474.2	156.661	38.9484	274.374	OK	OK	-0.00208
ENSG0000015	-	-	ENSG0000015711	'TMEM171	-	5:73120291-73131817	-	-	IGV_LINK	180.368	8.41387	1.45183	15.3759	OK	161.902	7.5808	1.49597	13.6656	OK	OK	-0.15042
ENSG0000015	-	-	ENSG0000015711	'KLHL40	-	3:42685518-42692544	-	-	IGV_LINK	0	0	0	0	OK	0	0	0	0	OK	NOTEST	0
ENSG0000015	-	-	ENSG0000015713	'C8A	-	1:56854805-56918221	-	-	IGV_LINK	0	0	0	0	OK	1.03122	0.02663	0	0.09967	OK	NOTEST	inf
ENSG0000015	-	-	ENSG0000015715	'TIMP4	-	3:12004401-12191400	-	-	IGV_LINK	0	0	0	0	OK	17.5308	0.669201	0	1.49853	OK	OK	inf
ENSG0000015	-	-	ENSG0000015715	'SYN2	-	3:12004401-12191400	-	-	IGV_LINK	1.93944	0.055892	0	0.384352	OK	5.15612	0.148682	0	0.608654	OK	NOTEST	1.41151
ENSG0000015	-	-	ENSG0000015716	'NRG1	-	8:31639385-32767959	-	-	IGV_LINK	97.9418	1.9547	0	4.95394	OK	501.175	11.1744	0.919152	21.4296	OK	OK	2.51518
ENSG0000015	-	-	ENSG0000015718	'C1orf27	-	1:186375837-186421378	-	-	IGV_LINK	571.165	10.9019	0	22.8857	OK	636.266	11.7186	0.884051	22.5531	OK	OK	0.104225
ENSG0000015	-	-	ENSG0000015718	'CPT2	-	1:53196428-53220617	-	-	IGV_LINK	467.405	10.219	0	20.5717	OK	713.608	16.4026	0	33.3929	OK	OK	0.682672
ENSG0000015	-	-	ENSG0000015719	'NECAP2	-	1:16440671-16460078	-	-	IGV_LINK	1813.38	58.2657	21.0647	95.4667	OK	1799.49	58.4706	20.7776	96.1635	OK	OK	0.005063
ENSG0000015	-	-	ENSG0000015719	'LRP8	-	1:53226891-53328070	-	-	IGV_LINK	202.672	2.21172	0	5.46806	OK	106.216	1.64358	0	5.67231	OK	OK	-0.42833
ENSG0000015	-	-	ENSG0000015721	'CDCP2	-	1:54132685-54413479	-	-	IGV_LINK	1.3778	0.030444	0	0.733378	OK	2.75219	0.06084	0	1.08164	OK	NOTEST	0.998866
ENSG0000015	-	-	ENSG0000015721	'PAXIP1	-	7:154928497-155003084	-	-	IGV_LINK	352.009	5.67618	0.711377	10.641	OK	290.805	4.48609	0.739825	8.23236	OK	OK	-0.33946
ENSG0000015	-	-	ENSG0000015721	'STEAP2	-	7:89882352-90238137	-	-	IGV_LINK	124.124	1.81433	0	4.19256	OK	68.0608	0.739	0	1.67876	OK	OK	-1.29579
ENSG0000015	-	-	ENSG0000015721	'SSBP3	-	1:54132685-54413479	-	-	IGV_LINK	551.771	15.8599	0	37.9841	OK	519.737	16.0468	0	39.9498	OK	OK	0.016895
ENSG0000015	-	-	ENSG0000015721	'HTR5A	-	7:155067066-155085749	-	-	IGV_LINK	0	0	0	0	OK	0	0	0	0	OK	NOTEST	0
ENSG0000015	-	-	ENSG0000015722	'CLDN12	-	7:90335222-91210590	-	-	IGV_LINK	505.224	12.7975	0	34.9805	OK	834.261	19.9946	0	48.1917	OK	OK	0.643744
ENSG0000015	-	-	ENSG0000015722	'MMP14	-	14:22836556-22849027	-	-	IGV_LINK	3203.96	54.9467	15.3026	94.5907	OK	5145.81	91.1989	26.3629	156.035	OK	OK	0.730984
ENSG0000015	-	-	ENSG0000015724	'FZD1	-	7:91264363-91271326	-	-	IGV_LINK	1617.49	13.4239	5.86339	20.9843	OK	903.353	7.49836	3.18317	11.8135	OK	OK	-0.84015
ENSG0000015	-	-	ENSG0000015725	'GATAD1	-	7:92412549-92528531	-	-	IGV_LINK	910.568	19.3681	3.12378	35.6124	OK	988.945	19.4373	4.83616	34.0384	OK	OK	0.005146
ENSG0000015	-	-	ENSG0000015730	'SUSD3	-	9:93058687-93085138	-	-	IGV_LINK	171.641	10.5882	1.30967	19.8668	OK	214.495	13.0107	2.51897	23.5025	OK	OK	0.29724
ENSG0000015	-	-	ENSG0000015730	'ZFHX2-AS1	-	14:23511759-23578800	-	-	IGV_LINK	93.5366	2.44581	0	10.2064	OK	77.25	2.02644	0	9.01096	OK	OK	-0.27137
ENSG0000015	-	-	ENSG0000015731	'TMED6	-	16:69105563-69408571	-	-	IGV_LINK	14.5458	1.17627	0	16.3617	OK	13.4059	1.00922	0	15.1475	OK	OK	-0.22097
ENSG0000015	-	-	ENSG0000015732	'CLEC18A	-	16:69950704-69964452	-	-	IGV_LINK	291.886	8.30474	2.31716	14.2923	OK	137.153	3.71738	0.108534	7.32622	OK	OK	-1.15965
ENSG0000015	-	-	ENSG0000015732	'DHRS4	-	14:23938730-24006408	-	-	IGV_LINK	339.402	17.5438	1.51742	33.5702	OK	353.71	18.6858	1.83082	35.5408	OK	OK	0.090983
ENSG0000015	-	-	ENSG0000015733	'C1orf158	-	1:12746214-12763699	-	-	IGV_LINK	0	0	0	0	OK	0	0	0	0	OK	NOTEST	0
ENSG0000015	-	-	ENSG0000015733	'CLEC18C	-	16:70113625-70187361	-	-	IGV_LINK	93.0932	3.52115	0	14.742	OK	90.7478	3.93116	0	15.7672	OK	OK	0.158907
ENSG0000015	-	-	ENSG0000015734	'ARMC12	-	6:35737031-35749079	-	-	IGV_LINK	18.4247	1.04201	0	2.27528	OK	19.5933	1.32942	0	3.07243	OK	OK	0.351434
ENSG0000015	-	-	ENSG0000015734	'DDX19B	-	16:70289662-70373383	-	-	IGV_LINK	557.589	18.1778	0	38.5681	OK	505.611	17.1549	0	35.8517	OK	OK	-0.08356
ENSG0000015	-	-	ENSG0000015735	'ST3GAL2	-	16:70375977-70439237	-	-	IGV_LINK	591.53	7.93367	1.44459	14.4228	OK	625.954	11.2444	0	22.5807	OK	OK	0.503141
ENSG0000015	-	-	ENSG0000015735	'FUK	-	16:70454420-70480274	-	-	IGV_LINK	840.748	18.9081	4.25704	33.5592	OK	777.544	14.9637	4.40643	25.5209	OK	OK	-0.33755
ENSG0000015	-	-	ENSG0000015736	'IL34	-	16:70579894-70660682	-	-	IGV_LINK	251.158	12.2495	2.74416	21.7548	OK	261.931	12.5491	2.38069	22.7175	OK	OK	0.03486
ENSG0000015	-	-	ENSG0000015737	'DHRS1	-	14:24290597-24299833	-	-	IGV_LINK	462.557	20.0444	5.79323	34.2956	OK	473.332	20.17	6.58917	33.7509	OK	OK	0.009012
ENSG0000015	-	-	ENSG0000015738	'CACNA1D	-	3:53283427-53846390	-	-	IGV_LINK	176.489	4.01082	0	16.6802	OK	85.5917	2.40138	0	14.5021	OK	OK	-0.74004
ENSG0000015	-	-	ENSG0000015739	'ARSE	-	X:2934631-2968310	-	-	IGV_LINK	1596.16	59.741	19.2456	100.236	OK	1996.45	70.8838	25.4925	116.275	OK	OK	0.246735
ENSG0000015	-	-	ENSG0000015740	'KIT	-	4:54657917-54740715	-	-	IGV_LINK	96.0023	1.07914	0.031052	2.12723	OK	73.217	0.842795	0	1.76457	OK	OK	-0.35663
ENSG0000015	-	-	ENSG0000015742	'HYDIN	-	16:70807377-71230722	-	-	IGV_LINK	232.733	2.32661	0	5.28416	OK	153.653	1.6355	0	3.84169	OK	OK	-0.5085
ENSG0000015	-	-	ENSG0000015742	'AASDH	-	4:56338286-56387508	-	-	IGV_LINK	263.764	4.69039	1.35611	8.02467	OK	281.524	5.1204	0.971406	9.26939	OK	OK	0.126549
ENSG0000015	-	-	ENSG0000015742	'ZNF19	-	16:71447596-71565089	-	-	IGV_LINK	169.38	8.21704	0	20.3797	OK	149.688	6.9539	0	18.0345	OK	OK	-0.2408
ENSG0000015	-	-	ENSG0000015744	'CACNA2D3	-	3:54122546-55074557	-	-	IGV_LINK	109.578	3.83394	0	8.93061	OK	131.997	5.37182	0	11.7893	OK	OK	0.486582
ENSG0000015	-	-	ENSG0000015745	'RNF111	-	15:58771191-59097419	-	-	IGV_LINK	573.105	13.2964	0	46.6396	OK	648.641	9.4197	0	29.8753	OK	OK	-0.49728
ENSG0000015	-	-	ENSG0000015745	'CCNB2	-	15:59105077-59523549	-	-	IGV_LINK	133.821	4.51791	0	11.34	OK	125.809	4.40549	0	11.7757	OK	OK	-0.03635
ENSG0000015	-	-	ENSG0000015747	'FAM81A	-	15:59105077-59523549	-	-	IGV_LINK	56.2438	1.95574	0	9.02489	OK	34.0304	1.31792	0	8.58104	OK	OK	-0.56946
ENSG0000015	-	-	ENSG0000015748	'MYO1E	-	15:59105077-59523549	-	-	IGV_LINK	676.865	14.252	0	33.3184	OK	831.167	21.5893	0	48.1297	OK	OK	0.599153
ENSG0000015	-	-	ENSG0000015750	'APPL1	-	3:57227736-57292682	-	-	IGV_LINK	596.378	6.73728	2.12449	11.3501	OK	514.581	5.47045	1.02302	9.91789	OK	OK	-0.30051
ENSG0000015	-	-	ENSG0000015750	'MUM1L1	-	X:106168304-106208956	-	-	IGV_LINK	6.78804	0.10108	0	0.243316	OK	46.4051	0.675766	0	1.4671	OK	OK	2.74103
ENSG0000015	-	-	ENSG0000015751	'AFAP1L1	-	5:149141482-149341802	-	-	IGV_LINK	371.403	5.95292	0	20.1661	OK	519.737	8.34968	0	28.0859	OK	OK	0.488124
ENSG0000015	-	-	ENSG0000015751	'TSC22D3	-	X:107713220-107795829	-	-	IGV_LINK	1577.74	52.3593	18.9401	85.7784	OK	2497.63	86.4138	27.536	145.292	OK	OK	0.722816
ENSG0000015	-	-	ENSG0000015753	'DSCR3	-	21:37208502-37268497	-	-	IGV_LINK	1317.85	30.5894	7.7176	53.4613	OK	1346.78	29.8774	7.95957	51.7953	OK	OK	-0.03398
ENSG0000015	-	-	ENSG0000015754	'DYRK1A	-	21:37365476-37517450	-	-	IGV_LINK	966.811	16.5528	0.998331	32.1072	OK	879.635	13.4246	2.10476	24.7444	OK	OK	-0.30219
ENSG0000015	-	-	ENSG0000015754	'KCNJ6	-	21:37607375-37916446	-	-	IGV_LINK	0.969721	0.002806	0	0.010992	OK	3.09367	0.008954	0	0.023191	OK	NOTEST	1.67377
ENSG0000015	-	-	ENSG0000015755	'KCNJ15	-	21:38121450-38307357	-	-	IGV_LINK	3036.19	60.6309	12.4632	108.799	OK	178.402	4.62079	0	12.4412	OK	OK	-3.71384
ENSG0000015	-	-	ENSG0000015755	'ERG	-	21:38380026-38661780	-	-	IGV_LINK	299.644	4.98758	1.0452	8.92996	OK	410.428	7.80828	1.77435	13.8422	OK	OK	0.646665
ENSG0000015	-	-	ENSG0000015755	'ETS2	-	21:38805306-38824955	-	-	IGV_LINK	1518.58	26.4189	9.50254	43.3352	OK	2340.88	39.2152	15.0557	63.3746	OK	OK	0.569843
ENSG0000015	-	-	ENSG0000015757	'TSPAN18	-	11:44719391-44951306	-	-	IGV_LINK	930.932	16.2821	0	35.0763	OK	875.51	15.3335	0	31.6725	OK	OK	-0.0866
ENSG0000015	-	-	ENSG0000015757	'LCA5L	-	21:39380243-39445805	-	-	IGV_LINK	59.153	2.51832	0	7.68239	OK	70.1233	2.60879	0	7.61503	OK	OK	0.050917
ENSG0000015	-	-	ENSG0000015759	'SLC35B2	-	6:44254095-44257890	-	-	IGV_LINK	933.841	28.2105	12.2363	44.1848	OK	772.388	23.4962	9.39836	37.594	OK	OK	-0.26381
ENSG0000015	-	-	ENSG0000015760	'TMEM164	-	X:110002630-110182734	-	-	IGV_LINK	468.375	5.93795	0.857337	11.0186	OK	589.861	9.59546	0	27.7944	OK	OK	0.692389
ENSG0000015	-	-	ENSG0000015760	'MX1	-	21:41420303-41459214	-	-	IGV_LINK	2415.57	60.9146	14.564	107.265	OK	3164.83	78.4405	17.8775	139.004	OK	OK	0.364811
ENSG0000015	-	-	ENSG0000015761	'CREB3L1	-	11:46277660-46321422	-	-	IGV_LINK	0	0	0	0	OK	2.06245	0.046247	0	0.479418	OK	NOTEST	inf
ENSG0000015	-	-	ENSG0000015761	'C2CD2	-	21:41885111-41953890	-	-	IGV_LINK	794.201	11.2614	0.773221	21.7495	OK	800.231	11.2676	0	22.8247	OK	OK	0.00079
ENSG0000015	-	-	ENSG0000015762	'TAB3	-	X:30827441-30975084	-	-	IGV_LINK	696.259	7.58004	1.31783	13.8422	OK	646.578	7.70321	0.451657	14.9548	OK	OK	0.023255
ENSG0000015	-	-	ENSG0000015763	'SLC38A10	-	17:81244999-81295547	-	-	IGV_LINK	2086.84	37.4398	11.0436	63.836	OK	2376.97	41.5226	13.2419	69.8033	OK	OK	0.149323
ENSG0000015	-	-	ENSG0000015765	'C9orf43	-	9:113407234-113429684	-	-	IGV_LINK	30.0613	1.01963	0	4.74802	OK	15.4684	0.490652	0	2.97043	OK	OK	-1.05527
ENSG0000015	-	-	ENSG0000015765	'PALM2-AKAP2	-	9:109640787-110172512	-	-	IGV_LINK	204.405	2.12182	0	7.8045	OK	20.4035	0.553003	0	4.42643	OK	OK	-1.93995
ENSG0000015	-	-	ENSG0000015765	'ZNF618	-	9:113876281-114056591	-	-	IGV_LINK	936.75	6.33787	2.31673	10.359	OK	467.145	3.07523	1.03612	5.11433	OK	OK	-1.04331
ENSG0000015	-	-	ENSG0000015768	'DGKI	-	7:137381036-137847092	-	-	IGV_LINK	83.396	0.698721	0	2.08131	OK	63.9359	0.461701	0	1.59025	OK	OK	-0.59776
ENSG0000015	-	-	ENSG0000015769	'TMEM268	-	9:114611205-114646422	-	-	IGV_LINK	610.924	10.9659	1.77043	20.1613	OK	654.828	13.8687	2.28602	25.4514	OK	OK	0.338812
ENSG0000015	-	-	ENSG0000015770	'SVOPL	-	7:138594284-138701352	-	-	IGV_LINK	0	0	0	0	OK	8.2498	0.188975	0	0.640854	OK	NOTEST	inf
ENSG0000015	-	-	ENSG0000015773	'SNX22	-	15:64151714-64163205	-	-	IGV_LINK	122.185	2.81139	0	14.8091	OK	113.435	2.06815	0	10.7331	OK	OK	-0.44295
ENSG0000015	-	-	ENSG0000015774	'UBN2	-	7:139230355-139308236	-	-	IGV_LINK	658.44	2.66292	0.989297	4.33655	OK	490.863	1.98138	0.673522	3.28923	OK	OK	-0.42651
ENSG0000015	-	-	ENSG0000015776	'BRAF	-	7:140518419-140924764	-	-	IGV_LINK	850.445	9.05708	0	22.7765	OK	563.049	5.75099	0	15.142	OK	OK	-0.65524
ENSG0000015	-	-	ENSG0000015776	'SLC34A2	-	4:25655300-25678748	-	-	IGV_LINK	2190.6	39.8096	7.36468	72.2546	OK	7111.33	128.695	13.583	243.808	OK	OK	1.69277
ENSG0000015	-	-	ENSG0000015776	'ACAN	-	15:88803442-88875354	-	-	IGV_LINK	232.733	2.48984	0	6.06655	OK	31.968	0.260582	0	0.864013	OK	OK	-3.25624
ENSG0000015	-	-	ENSG0000015777	'PSMG3	-	7:1567329-1589626	-	-	IGV_LINK	302.553	24.7801	5.1823	44.3778	OK	222.745	18.3798	1.90384	34.8558	OK	OK	-0.43106
ENSG0000015	-	-	ENSG0000015778	'CABP1	-	12:120629610-120667324	-	-	IGV_LINK	131.882	5.34512	0.243187	10.4471	OK	74.2482	3.67351	0	7.69563	OK	OK	-0.54106
ENSG0000015	-	-	ENSG0000015779	'WDR19	-	4:39182403-39285810	-	-	IGV_LINK	1516.64	36.3033	5.19948	67.4071	OK	1243.66	29.372	3.74905	54.9949	OK	OK	-0.30566
ENSG0000015	-	-	ENSG0000015780	'SLC37A3	-	7:140293692-140426250	-	-	IGV_LINK	764.14	19.6892	3.32959	36.0487	OK	296.993	7.57595	0.466944	14.685	OK	OK	-1.3779
ENSG0000015	-	-	ENSG0000015782	'AP3S2	-	15:89830598-89912956	-	-	IGV_LINK	788.295	23.428	0	58.9083	OK	906.601	27.8649	0	71.1047	OK	OK	0.250216
ENSG0000015	-	-	ENSG0000015782	'FMNL2	-	2:152335236-152649834	-	-	IGV_LINK	1882.23	26.7534	0.838925	52.6679	OK	1495.28	18.2363	2.83694	33.6357	OK	OK	-0.55291
ENSG0000015	-	-	ENSG0000015783	'GAREM2	-	2:26173090-26189663	-	-	IGV_LINK	153.216	2.25817	0	5.20294	OK	262.962	5.34423	0	16.3847	OK	OK	1.24283
ENSG0000015	-	-	ENSG0000015783	'SPPL3	-	12:120762509-120904371	-	-	IGV_LINK	713.714	17.2058	2.99561	31.4161	OK	828.074	24.407	4.4209	44.3932	OK	OK	0.504398
ENSG0000015	-	-	ENSG0000015785	'DPYSL5	-	2:26847746-27027196	-	-	IGV_LINK	0	0	0	0	OK	1.03122	0.014502	0	0.389909	OK	NOTEST	inf
ENSG0000015	-	-	ENSG0000015785	'DRC1	-	2:26401915-26456711	-	-	IGV_LINK	0.969721	0.023571	0	0.458946	OK	0	0	0	0	OK	NOTEST	'-inf
ENSG0000015	-	-	ENSG0000015786	'RAB28	-	4:13361353-13484365	-	-	IGV_LINK	219.157	8.20848	1.47184	14.9451	OK	215.526	7.83197	1.19313	14.4708	OK	OK	-0.06774
ENSG0000015	-	-	ENSG0000015787	'FAM213B	-	1:2586490-2801717	-	-	IGV_LINK	664.259	17.6302	3.13121	32.1293	OK	367.116	9.08051	0.890479	17.2705	OK	OK	-0.95721
ENSG0000015	-	-	ENSG0000015787	'TNFRSF14	-	1:2549919-2565382	-	-	IGV_LINK	8426.87	306.929	55.9194	557.938	OK	3880.5	137.07	43.6821	230.457	OK	OK	-1.16299
ENSG0000015	-	-	ENSG0000015788	'PANK4	-	1:2508532-2526628	-	-	IGV_LINK	467.405	17.3992	2.36519	32.4332	OK	326.898	12.4796	0.273757	24.6855	OK	OK	-0.47945
ENSG0000015	-	-	ENSG0000015788	'CIB4	-	2:26581201-26641368	-	-	IGV_LINK	2.90916	0.392709	0	1.1189	OK	21.6557	2.03634	0	4.30864	OK	OK	2.37444
ENSG0000015	-	-	ENSG0000015789	'MEGF11	-	15:65895078-66253747	-	-	IGV_LINK	365.585	9.05437	0	20.0404	OK	1219.94	30.9315	0.017311	61.8458	OK	OK	1.77239
ENSG0000015	-	-	ENSG0000015789	'C12orf43	-	12:120941727-121016502	-	-	IGV_LINK	608.015	10.8262	0	23.4943	OK	894.072	13.2738	0	27.1314	OK	OK	0.294049
ENSG0000015	-	-	ENSG0000015791	'PEX10	-	1:2391774-2413797	-	-	IGV_LINK	1070.57	25.6402	0	52.7619	OK	1150.85	27.5104	0	57.1896	OK	OK	0.101567
ENSG0000015	-	-	ENSG0000015791	'RER1	-	1:2391774-2413797	-	-	IGV_LINK	1835.68	103.189	10.0019	196.375	OK	2464.63	154.606	21.9348	287.278	OK	OK	0.583315
ENSG0000015	-	-	ENSG0000015792	'RADIL	-	7:4797054-4883719	-	-	IGV_LINK	18.4247	0.391305	0	1.18822	OK	0	0	0	0	OK	NOTEST	'-inf
ENSG0000015	-	-	ENSG0000015793	'SKI	-	1:2228694-2310119	-	-	IGV_LINK	1133.6	12.3977	3.25934	21.5361	OK	947.696	10.9479	1.41581	20.48	OK	OK	-0.17942
ENSG0000015	-	-	ENSG0000015795	'WIPI2	-	7:5190187-5233826	-	-	IGV_LINK	1278.09	35.7965	9.94236	61.6506	OK	1643.77	45.2315	15.6117	74.8514	OK	OK	0.337511
ENSG0000015	-	-	ENSG0000015796	'SSX8	-	X:52624997-52632895	-	-	IGV_LINK	0	0	0	0	OK	0	0	0	0	OK	NOTEST	0
ENSG0000015	-	-	ENSG0000015797	'LDLRAP1	-	1:25543579-25568886	-	-	IGV_LINK	255.036	4.99006	0.882671	9.09745	OK	324.836	6.50567	1.89697	11.1144	OK	OK	0.382641

ENSG0000015	-	-	ENSG0000015798	AGAP1	-	2:235494088-236131800	-	-	IGV_LINK	909.598	7.99662	0.602177	15.3911	OK	900.259	8.27342	0.421315	16.1255	OK	OK	0.049094
ENSG0000015	-	-	ENSG0000015799	KRTCAP3	-	2:27442365-27489789	-	-	IGV_LINK	104.73	10.2634	0	29.8789	OK	176.339	18.5722	0	47.2346	OK	OK	0.855633
ENSG0000015	-	-	ENSG0000015799	ANKRD61	-	7:6009244-6059230	-	-	IGV_LINK	15.5155	0.806275	0	5.65443	OK	10.3122	0.536452	0	4.84509	OK	OK	-0.58782
ENSG0000015	-	-	ENSG0000015800	PAFAH2	-	1:25959766-25998157	-	-	IGV_LINK	427.647	7.73291	2.26549	13.2003	OK	596.048	11.6427	2.08746	21.198	OK	OK	0.590348
ENSG0000015	-	-	ENSG0000015800	EXTL1	-	1:26019883-26036464	-	-	IGV_LINK	2.90916	0.198448	0	0.629762	OK	10.3122	0.249595	0	0.771909	OK	NOTEST	0.330829
ENSG0000015	-	-	ENSG0000015801	SLC30A2	-	1:26037251-26046133	-	-	IGV_LINK	61.0924	1.45386	0.031469	2.87624	OK	456.833	10.7953	3.96293	17.6277	OK	OK	2.89245
ENSG0000015	-	-	ENSG0000015801	BABAM2	-	2:27771716-28338901	-	-	IGV_LINK	1308.32	49.1866	14.553	83.8202	OK	1868.38	72.9812	14.5491	131.413	OK	OK	0.56926
ENSG0000015	-	-	ENSG0000015802	TRIM63	-	1:26051303-26068436	-	-	IGV_LINK	0	0	0	0	OK	0	0	0	0	OK	NOTEST	0
ENSG0000015	-	-	ENSG0000015802	WDR66	-	12:121888730-122003927	-	-	IGV_LINK	554.584	11.5101	0	24.7038	OK	540.256	12.6108	0	28.507	OK	OK	0.131763
ENSG0000015	-	-	ENSG0000015804	MRPL17	-	11:6680781-6683401	-	-	IGV_LINK	570.196	29.0357	7.22229	50.8492	OK	769.294	42.4575	5.5917	79.3234	OK	OK	0.548191
ENSG0000015	-	-	ENSG0000015805	DUSP2	-	2:96143165-96145440	-	-	IGV_LINK	214.308	8.12975	2.18947	14.07	OK	90.7478	3.50644	0.284404	6.72847	OK	OK	-1.2132
ENSG0000015	-	-	ENSG0000015805	GRHL3	-	1:24307555-24472976	-	-	IGV_LINK	15.5155	0.374856	0	2.61279	OK	9.28102	0.219968	0	2.18609	OK	NOTEST	-0.76904
ENSG0000015	-	-	ENSG0000015806	UBXN11	-	1:26279175-26320523	-	-	IGV_LINK	2292.42	64.714	2.1258	127.302	OK	3157.61	92.0878	9.00657	175.169	OK	OK	0.508933
ENSG0000015	-	-	ENSG0000015807	NLRP14	-	11:7020445-7071308	-	-	IGV_LINK	14.5458	0.237228	0	0.528485	OK	2.06245	0.033648	0	0.098906	OK	NOTEST	-2.81769
ENSG0000015	-	-	ENSG0000015807	PTPDC1	-	9:94030793-94109856	-	-	IGV_LINK	274.431	4.2081	1.07196	7.34424	OK	158.809	2.48654	0.36814	4.60495	OK	OK	-0.75903
ENSG0000015	-	-	ENSG0000015808	GALNT14	-	2:30910466-31155202	-	-	IGV_LINK	5801.84	172.914	48.52	297.308	OK	4180.59	119.508	35.8381	203.177	OK	OK	-0.53295
ENSG0000015	-	-	ENSG0000015809	NCK1	-	3:136862207-137011085	-	-	IGV_LINK	920.265	47.8702	7.66131	88.079	OK	1107.54	60.2081	12.4462	107.97	OK	OK	0.330831
ENSG0000015	-	-	ENSG0000015810	HPD	-	12:121839526-121863596	-	-	IGV_LINK	0.969721	0.043843	0	0.323314	OK	88.6853	3.98156	0.384608	7.57852	OK	OK	6.50485
ENSG0000015	-	-	ENSG0000015810	RHPN1	-	8:143368886-143384220	-	-	IGV_LINK	715.654	17.5988	6.47624	28.7213	OK	750.732	18.0819	6.61706	29.5467	OK	OK	0.039069
ENSG0000015	-	-	ENSG0000015810	TPRG1L	-	1:3625001-3630127	-	-	IGV_LINK	896.991	22.6106	9.54984	35.6714	OK	1123	28.2492	12.2847	44.2137	OK	OK	0.321209
ENSG0000015	-	-	ENSG0000015811	LRRC43	-	12:122167737-122203471	-	-	IGV_LINK	2.90916	0.371733	0	1.1675	OK	8.2498	0.705454	0	1.95059	OK	OK	0.924286
ENSG0000015	-	-	ENSG0000015812	AAED1	-	9:96639576-96655303	-	-	IGV_LINK	202.672	6.35571	0	13.9542	OK	246.463	6.59372	0	13.4598	OK	OK	0.053038
ENSG0000015	-	-	ENSG0000015812	XDH	-	2:31334320-31414715	-	-	IGV_LINK	0	0	0	0	OK	10.3122	0.105389	0	0.449326	OK	NOTEST	inf
ENSG0000015	-	-	ENSG0000015815	XKR8	-	1:27934992-27968096	-	-	IGV_LINK	179.398	5.28645	0	15.317	OK	218.62	4.8923	0	9.88406	OK	OK	-0.11179
ENSG0000015	-	-	ENSG0000015815	CNNM4	-	2:96760901-96811891	-	-	IGV_LINK	462.557	5.63323	1.99172	9.27474	OK	608.423	7.50963	2.74942	12.2698	OK	OK	0.41478
ENSG0000015	-	-	ENSG0000015816	EYA3	-	1:27970343-28088696	-	-	IGV_LINK	830.081	8.98626	3.25562	14.7169	OK	568.205	6.11379	2.01216	10.2154	OK	OK	-0.55565
ENSG0000015	-	-	ENSG0000015816	DZIP1L	-	3:138061989-138115818	-	-	IGV_LINK	365.585	11.6356	0	23.6117	OK	364.022	9.77703	0	19.6626	OK	OK	-0.25107
ENSG0000015	-	-	ENSG0000015816	TMSB15A	-	X:102513675-102516784	-	-	IGV_LINK	1.93944	0.213276	0	0.653147	OK	0	0	0	0	OK	NOTEST	'-inf
ENSG0000015	-	-	ENSG0000015816	FANCC	-	9:95099053-95426796	-	-	IGV_LINK	391.767	5.74751	1.12983	10.3652	OK	237.182	3.68587	0.264674	7.10706	OK	OK	-0.64093
ENSG0000015	-	-	ENSG0000015818	MRAS	-	3:138347647-138405534	-	-	IGV_LINK	1232.51	30.1549	0	61.4041	OK	675.452	16.8637	0	36.8248	OK	OK	-0.83847
ENSG0000015	-	-	ENSG0000015819	WASF2	-	1:27404225-27490158	-	-	IGV_LINK	4243.5	43.4533	18.2121	68.6945	OK	3947.53	40.4311	16.9789	63.8833	OK	OK	-0.104
ENSG0000015	-	-	ENSG0000015820	ABHD3	-	18:21650896-21704805	-	-	IGV_LINK	273.461	8.93972	1.04803	16.8314	OK	311.43	10.5692	2.81923	18.3191	OK	OK	0.241558
ENSG0000015	-	-	ENSG0000015822	ESYT3	-	3:138434585-138481686	-	-	IGV_LINK	28.1219	0.380887	0	1.04674	OK	8.2498	0.261907	0	0.962183	OK	NOTEST	-0.54031
ENSG0000015	-	-	ENSG0000015823	FAIM	-	3:138608605-138633376	-	-	IGV_LINK	181.338	11.5345	0.740139	22.3289	OK	266.056	18.3345	3.32408	33.3449	OK	OK	0.668602
ENSG0000015	-	-	ENSG0000015824	FAM46B	-	1:27005019-27012836	-	-	IGV_LINK	3.87888	0.099567	0	0.25358	OK	4.1249	0.105937	0	0.268366	OK	NOTEST	0.089474
ENSG0000015	-	-	ENSG0000015825	CLSTN2	-	3:139935184-140577397	-	-	IGV_LINK	11.6366	0.046744	0	0.148995	OK	8.2498	0.033142	0	0.111358	OK	NOTEST	-0.49613
ENSG0000015	-	-	ENSG0000015827	COLEC12	-	18:316736-500722	-	-	IGV_LINK	16.4852	0.168733	0	0.476616	OK	139.215	1.45542	0.217451	2.6934	OK	OK	3.10863
ENSG0000015	-	-	ENSG0000015828	RNF207	-	1:6181268-6235972	-	-	IGV_LINK	687.532	21.2242	0	98.3841	OK	705.358	25.2845	0	126.99	OK	OK	0.252543
ENSG0000015	-	-	ENSG0000015829	CUL4B	-	X:120524608-120575794	-	-	IGV_LINK	1159.79	16.9019	4.91169	28.8922	OK	1004.41	14.5839	4.81133	24.3565	OK	OK	-0.21281
ENSG0000015	-	-	ENSG0000015829	GPR153	-	1:6247345-6260975	-	-	IGV_LINK	57.2135	0.824737	0.010565	1.63891	OK	69.0921	0.99626	0	1.99485	OK	OK	0.272589
ENSG0000015	-	-	ENSG0000015829	SLC13A3	-	20:46557822-46690289	-	-	IGV_LINK	54.3043	1.11328	0	3.25512	OK	25.7806	0.430009	0	1.38809	OK	OK	-1.37238
ENSG0000015	-	-	ENSG0000015830	GPRASP2	-	X:102599167-102717733	-	-	IGV_LINK	308.316	5.80375	0	13.5994	OK	207.604	4.03302	0	10.0712	OK	OK	-0.52512
ENSG0000015	-	-	ENSG0000015831	RHBDL2	-	1:38838197-38941799	-	-	IGV_LINK	32.9705	1.3923	0	6.25831	OK	145.403	7.42386	0	21.1229	OK	OK	2.4147
ENSG0000015	-	-	ENSG0000015832	AUTS2	-	7:69598918-70793068	-	-	IGV_LINK	932.871	23.1252	0	47.4351	OK	803.324	17.0641	0	34.572	OK	OK	-0.4385
ENSG0000015	-	-	ENSG0000015835	SHROOM4	-	X:50591646-50814302	-	-	IGV_LINK	442.193	3.47123	0.004479	6.93797	OK	367.116	2.85723	0	5.73112	OK	OK	-0.28083
ENSG0000015	-	-	ENSG0000015837	HIST1H2BD	-	6:26158145-26184655	-	-	IGV_LINK	384.009	44.9367	8.51689	81.3565	OK	617.704	67.686	23.3645	112.007	OK	OK	0.590963
ENSG0000015	-	-	ENSG0000015840	CDC25C	-	5:138285264-138349729	-	-	IGV_LINK	22.3036	0.880604	0	4.45117	OK	50.53	1.68221	0	6.6593	OK	OK	0.93379
ENSG0000015	-	-	ENSG0000015840	HIST1H4H	-	6:26277608-26285638	-	-	IGV_LINK	93.0932	4.03674	0	9.19195	OK	207.276	9.24557	1.77295	16.7182	OK	OK	1.19557
ENSG0000015	-	-	ENSG0000015841	MITD1	-	2:98997260-99490035	-	-	IGV_LINK	608.015	43.8536	0	127.793	OK	398.053	27.342	0	87.1152	OK	OK	-0.68158
ENSG0000015	-	-	ENSG0000015841	EIF5B	-	2:98997260-99490035	-	-	IGV_LINK	1420.64	19.1967	0	47.9652	OK	1331.31	19.6207	0	51.4544	OK	OK	0.031516
ENSG0000015	-	-	ENSG0000015842	RIBC1	-	X:53374148-53431120	-	-	IGV_LINK	89.2143	3.7465	0	13.1835	OK	105.185	4.73628	0	15.3959	OK	OK	0.338212
ENSG0000015	-	-	ENSG0000015842	TMSB15B	-	X:103917410-103966712	-	-	IGV_LINK	42.6677	1.63003	0	4.03441	OK	6.18735	0.213547	0	0.684365	OK	OK	-2.93227
ENSG0000015	-	-	ENSG0000015842	CATIP	-	2:218270391-218368099	-	-	IGV_LINK	59.153	3.62938	0	23.4309	OK	86.6229	4.87218	0	31.5502	OK	OK	0.424846
ENSG0000015	-	-	ENSG0000015843	CNOT11	-	2:100820151-101270316	-	-	IGV_LINK	1012.39	35.7808	0	177.736	OK	1030.19	40.2384	0	173.626	OK	OK	0.169391
ENSG0000015	-	-	ENSG0000015844	KCNB1	-	20:49219294-49484297	-	-	IGV_LINK	111.518	0.630457	0	3.51975	OK	300.086	1.49842	0	5.32969	OK	OK	1.24897
ENSG0000015	-	-	ENSG0000015845	TSPAN33	-	7:129144891-129169697	-	-	IGV_LINK	1357.61	47.4175	18.3619	76.473	OK	807.449	29.0122	9.98517	48.0392	OK	OK	-0.70876
ENSG0000015	-	-	ENSG0000015845	NRG2	-	5:139846778-140043299	-	-	IGV_LINK	20.3641	0.431295	0	1.03595	OK	44.3427	1.23799	0	3.07904	OK	OK	1.52126
ENSG0000015	-	-	ENSG0000015846	AHCYL2	-	7:129225022-129430211	-	-	IGV_LINK	650.682	10.1136	0.904271	19.3229	OK	4853.98	88.3595	4.63934	172.08	OK	OK	3.12709
ENSG0000015	-	-	ENSG0000015847	B4GALT5	-	20:49632944-49713878	-	-	IGV_LINK	1881.26	23.3036	10.3468	36.2603	OK	3134.92	38.8428	16.8297	60.856	OK	OK	0.737098
ENSG0000015	-	-	ENSG0000015847	CD1D	-	1:158179946-158184896	-	-	IGV_LINK	279.28	7.56506	2.2795	12.8506	OK	304.211	8.24496	2.72638	13.7635	OK	OK	0.124162
ENSG0000015	-	-	ENSG0000015847	CD1A	-	1:158254136-158258269	-	-	IGV_LINK	3.87888	0.113646	0	0.289439	OK	5.15612	0.151158	0	0.370541	OK	NOTEST	0.411509
ENSG0000015	-	-	ENSG0000015848	SPATA2	-	20:49903390-49915508	-	-	IGV_LINK	276.37	4.04599	1.28648	6.80551	OK	342.367	5.01366	1.58498	8.44234	OK	OK	0.309371
ENSG0000015	-	-	ENSG0000015848	CD1C	-	1:158289785-158293630	-	-	IGV_LINK	64.9713	3.08193	0.012501	6.15136	OK	89.7166	4.00856	0.371738	7.64537	OK	OK	0.379249
ENSG0000015	-	-	ENSG0000015848	SNX29P1	-	16:21376647-21386064	-	-	IGV_LINK	1.93944	0.130978	0	0.401115	OK	3.09367	0.209217	0	0.540522	OK	NOTEST	0.675671
ENSG0000015	-	-	ENSG0000015848	FAM86C1	-	11:71787509-71821548	-	-	IGV_LINK	70.7896	2.6436	0	5.55231	OK	106.216	3.54428	0.23804	6.85052	OK	OK	0.42299
ENSG0000015	-	-	ENSG0000015848	CD1B	-	1:158327950-158331531	-	-	IGV_LINK	5.81832	0.268222	0	0.664583	OK	8.2498	0.411661	0	1.01786	OK	NOTEST	0.618029
ENSG0000015	-	-	ENSG0000015848	DNAH3	-	16:20933110-21180616	-	-	IGV_LINK	0.969721	0.018841	0	0.40701	OK	5.15612	0.049532	0	0.481296	OK	NOTEST	1.39446
ENSG0000015	-	-	ENSG0000015848	CD1E	-	1:158353695-158357553	-	-	IGV_LINK	88.2446	4.07204	0.021253	8.12283	OK	70.1233	3.90623	0	8.21671	OK	OK	-0.05997
ENSG0000015	-	-	ENSG0000015849	HMHB1	-	5:143605627-143828772	-	-	IGV_LINK	3.87888	1.03079	0	2.83889	OK	0	0	0	0	OK	OK	'-inf
ENSG0000015	-	-	ENSG0000015851	CPA2	-	7:130266826-130289798	-	-	IGV_LINK	0	0	0	0	OK	1.03122	0.048741	0	0.350071	OK	NOTEST	inf
ENSG0000015	-	-	ENSG0000015851	NCF1	-	7:74773961-74789315	-	-	IGV_LINK	272.491	14.6381	3.24608	26.0301	OK	335.148	18.4943	4.74172	32.2469	OK	OK	0.337356
ENSG0000015	-	-	ENSG0000015852	CPA5	-	7:130344815-130368730	-	-	IGV_LINK	3.87888	0.237115	0	0.904217	OK	0	0	0	0	OK	NOTEST	'-inf
ENSG0000015	-	-	ENSG0000015852	TSR2	-	X:54440400-54496166	-	-	IGV_LINK	632.258	26.171	9.70536	42.6367	OK	600.173	24.8639	8.79138	40.9365	OK	OK	-0.07392
ENSG0000015	-	-	ENSG0000015852	PPP1R9A	-	7:94907201-95296415	-	-	IGV_LINK	256.976	1.63435	0.361022	2.90767	OK	126.841	0.777232	0.036606	1.51786	OK	OK	-1.0723
ENSG0000015	-	-	ENSG0000015854	ZC3H18	-	16:88570380-88631966	-	-	IGV_LINK	672.016	15.0482	2.12769	27.9687	OK	802.293	17.482	2.62032	32.3436	OK	OK	0.216277
ENSG0000015	-	-	ENSG0000015855	ZFAND2B	-	2:219195236-219209651	-	-	IGV_LINK	704.017	38.5522	12.8229	64.2814	OK	609.454	32.6349	10.5389	54.7308	OK	OK	-0.2404
ENSG0000015	-	-	ENSG0000015855	POM121L2	-	6:27285902-27312170	-	-	IGV_LINK	0.969721	0.018617	0	0.269123	OK	0	0	0	0	OK	NOTEST	'-inf
ENSG0000015	-	-	ENSG0000015855	GDPD5	-	11:75434639-75525903	-	-	IGV_LINK	230.793	5.5867	0	11.3687	OK	323.805	8.08749	0	16.282	OK	OK	0.533697
ENSG0000015	-	-	ENSG0000015856	DYNC1I1	-	7:95772505-96110322	-	-	IGV_LINK	49.4557	1.56128	0	3.65625	OK	92.8102	2.94694	0	6.43027	OK	OK	0.916495
ENSG0000015	-	-	ENSG0000015857	PFKFB1	-	X:54932960-54998534	-	-	IGV_LINK	9.69721	0.419645	0	0.990352	OK	4.1249	0.147816	0	0.424604	OK	NOTEST	-1.50537
ENSG0000015	-	-	ENSG0000015857	ALAS2	-	X:55000356-55031064	-	-	IGV_LINK	1.93944	0.138972	0	1.52743	OK	0	0	0	0	OK	NOTEST	'-inf
ENSG0000015	-	-	ENSG0000015860	TMED4	-	7:44577893-44582287	-	-	IGV_LINK	2727.82	74.9487	31.9182	117.979	OK	3123.58	83.8331	36.1716	131.495	OK	OK	0.161616
ENSG0000015	-	-	ENSG0000015861	PPP1R15B	-	1:204377849-204494724	-	-	IGV_LINK	1023.06	11.4067	3.78356	19.0297	OK	1294.19	14.4329	4.34587	24.52	OK	OK	0.339491
ENSG0000015	-	-	ENSG0000015862	COPG2	-	7:130486041-130687432	-	-	IGV_LINK	337.463	6.42175	1.7542	11.0893	OK	346.492	6.88258	1.49966	12.2655	OK	OK	0.099984
ENSG0000015	-	-	ENSG0000015863	EMSY	-	11:76444922-76553025	-	-	IGV_LINK	348.13	7.35302	0	16.5218	OK	284.618	5.69367	0	12.1221	OK	OK	-0.36898
ENSG0000015	-	-	ENSG0000015863	PAGE5	-	X:55220354-55224108	-	-	IGV_LINK	0.969721	0.213712	0	0.885738	OK	0	0	0	0	OK	NOTEST	'-inf
ENSG0000015	-	-	ENSG0000015866	GPAT4	-	8:41529205-41625001	-	-	IGV_LINK	1441.97	58.4157	12.3181	104.513	OK	1523.12	56.787	10.7369	102.837	OK	OK	-0.0408
ENSG0000015	-	-	ENSG0000015868	PKD1L1	-	7:47695729-47979581	-	-	IGV_LINK	11.6366	0.095998	0	0.78402	OK	26.8118	0.246039	0	1.67013	OK	NOTEST	1.35782
ENSG0000015	-	-	ENSG0000015869	ZSCAN12	-	6:28378954-28399734	-	-	IGV_LINK	276.37	3.84847	1.09523	6.60171	OK	152.621	2.17512	0.380071	3.97016	OK	OK	-0.82319
ENSG0000015	-	-	ENSG0000015871	TAGLN2	-	1:159918106-159925732	-	-	IGV_LINK	6489.37	284.176	100.003	468.35	OK	13979.3	630.097	149.472	1110.72	OK	OK	1.14879
ENSG0000015	-	-	ENSG0000015871	ELK4	-	1:205568884-205631962	-	-	IGV_LINK	982.327	6.52046	0.703937	12.337	OK	800.231	4.67932	1.49924	7.85941	OK	OK	-0.47867
ENSG0000015	-	-	ENSG0000015871	SLAMF8	-	1:159826749-159900163	-	-	IGV_LINK	822.323	23.353	2.45429	44.2518	OK	635.235	19.692	1.1933	38.1907	OK	OK	-0.246
ENSG0000015	-	-	ENSG0000015871	SLC45A3	-	1:205657850-205680459	-	-	IGV_LINK	75.6382	1.43354	0.08674	2.78034	OK	74.2482	1.4486	0.004081	2.89313	OK	OK	0.015081
ENSG0000015	-	-	ENSG0000015871	DUSP23	-	1:159780931-159782543	-	-	IGV_LINK	666.198	69.3367	27.0519	111.622	OK	1980.98	200.737	87.8125	313.661	OK	OK	1.53361
ENSG0000015	-	-	ENSG0000015871	RNF166	-	16:88696494-88706421	-	-	IGV_LINK	1135.54	48.3721	13.6275	83.1166	OK	1078.66	43.9163	11.7212	76.1113	OK	OK	-0.13942
ENSG0000015	-	-	ENSG0000015873	OR10J6P	-	1:159598297-159599227	-	-	IGV_LINK	0	0	0	0	OK	1.03122	0.076839	0	0.287592	OK	NOTEST	inf
ENSG0000015	-	-	ENSG0000015874	NBL1	-	1:19596976-19658456	-	-	IGV_LINK	919.152	32.4786	1.28397	63.6733	OK	2848.93	108.52	18.9246	198.115	OK	OK	1.7404
ENSG0000015	-	-	ENSG0000015874	HTR6	-	1:19665286-19679562	-	-	IGV_LINK	67.8804	2.11178	0	4.23105	OK	88.6853	2.76078	0.279731	5.24182	OK	OK	0.386616
ENSG0000015	-	-	ENSG0000015876	ITLN2	-	1:160931738-160954799	-	-	IGV_LINK	0	0	0	0	OK	0	0	0	0	OK	NOTEST	0
ENSG0000015	-	-	ENSG0000015876	F11R	-	1:160995210-161038990	-	-	IGV_LINK	1899.59	27.6088	0.004035	55.2135	OK	2998.29	53.7492	0	112.48	OK	OK	0.961117
ENSG0000015	-	-	ENSG0000015877	USF1	-	1:161039250-161045977	-	-	IGV_LINK	1181.12	60.3769	23.5599	97.1939	OK	1106.5	56.0013	20.1654	91.8371	OK	OK	-0.10854
ENSG0000015	-	-	ENSG0000015878	PLA2G2F	-	1:20139325-20150386	-	-	IGV_LINK	1.93944	0.03524	0	0.109184	OK	0	0	0	0	OK	NOTEST	'-inf
ENSG0000015	-	-	ENSG0000015879	SPATA2L	-	16:89680736-89701705	-	-	IGV_LINK	107.639	2.95211	0	11.947	OK	135.09	3.52241	0	12.5874	OK	OK	0.254818
ENSG0000015	-	-	ENSG0000015879	NIT1	-	1:161100555-161132688	-	-	IGV_LINK	978.448	50.5326	7.20461	93.8607	OK	1279.75	65.7791	13.6871	117.871	OK	OK	0.380413
ENSG0000015	-	-	ENSG0000015879	DEDD	-	1:161100555-161132688	-	-	IGV_LINK	638.076	23.8345	0	52.9097	OK	567.174	21.922	0	49.6443	OK	OK	-0.12067
ENSG0000015	-	-	ENSG0000015880	ZNF276	-	16:89707133-89816657	-	-	IGV_LINK	913.477	32.1818	0	71.052	OK	758.982	24.7195	0	56.8191	OK	OK	-0.38059
ENSG0000015	-	-	ENSG0000015880	NPM2	-	8:22024124-22036897	-	-	IGV_LINK	4.4846	0.414039	0	1.74	OK	42.2802	3.78857	0	10.2356	OK	OK	3.19382
ENSG0000015	-	-	ENSG0000015881	EDA	-	X:69616066-70039469	-	-	IGV_LINK	243.4	3.17205	0.616655	5.72745	OK	76.3106	1.12947	0	2.51396	OK	OK	-1.48977
ENSG0000015	-	-	ENSG0000015881	FGF17	-	8:22042397-22048809	-	-	IGV_LINK	11.6366	0.35138	0	0.919817	OK	3.09367	0.113697	0	0.359401	OK	NOTEST	-1.62783
ENSG0000015	-	-	ENSG0000015881	VWA5B1	-	1:20290918-20354894	-	-	IGV_LINK	4.4846	0.101931	0	0.452818	OK	0	0	0	0	OK	NOTEST	'-inf
ENSG0000015	-	-	ENSG0000015882	CDA	-	1:20588947-20618908	-	-	IGV_LINK	24.243	2.03108	0	4.25881	OK	515.612	37.8159	14.5328	61.0991	OK	OK	4.21867
ENSG0000015	-	-	ENSG0000015882	PINK1	-	1:20633454-20661544	-	-	IGV_LINK	1590.34	41.772	0	90.1308	OK	2038.73	52.0777	0	119.792	OK	OK	0.318132
ENSG0000015	-	-	ENSG0000015885	B4GALT3	-	1:161166409-161178013	-	-	IGV_LINK	907.658	30.5244	0	62.4213	OK	883.76	29.7704	0.639355	58.9015	OK	OK	-0.03608
ENSG0000015	-	-	ENSG0000015885	DMTN	-	8:22048994-22082527	-	-	IGV_LINK	1525.37	44.0709	12.6676	75.4742	OK	2054.2	65.17	18.9345	111.405	OK	OK	0.564381
ENSG0000015	-	-	ENSG0000015885	ADAMTS4	-	1:161184307-161214395	-	-	IGV_LINK	591.53	7.63338	0	29.589	OK	1039.47	15.4581	0	56.3895	OK	OK	1.01797
ENSG0000015	-	-	ENSG0000015886	FAM160B2	-	8:22089158-22104898	-	-	IGV_LINK	1065.72	36.7001	6.27924	67.1209	OK	1192.1	35.2011	5.34577	65.0565	OK	OK	-0.06016
ENSG0000015	-	-	ENSG0000015886	NDUFS2	-	1:161184307-161214395	-	-	IGV_LINK	1654.34	76.6511	15.1892	138.113	OK	2183.1	98.9007	17.8787	179.923	OK	OK	0.367674
ENSG0000015	-	-	ENSG0000015886	SLC5A11	-	16:24845840-24911628	-	-	IGV_LINK	2.90916	0.063834	0	0.469985	OK	4.1249	0.183989	0	0.814209	OK	NOTEST	1.52722
ENSG0000015	-	-	ENSG0000015886	FCER1G	-	1:161215233-161220699	-	-	IGV_LINK	1568.04	212.486	93.197	331.775	OK	1589.12	215.838	94.8603	336.816	OK	OK	0.022583
ENSG0000015	-	-	ENSG0000015887	APOA2	-	1:161222291-161223631	-	-	IGV_LINK	0	0	0	0	OK	2.06245	0.592307	0	2.60825	OK	OK	inf
ENSG0000015	-	-	ENSG0000015888	TOMM40L	-	1:161225938-161238302	-	-	IGV_LINK	213.339	4.82908	1.10562	8.55255	OK	247.494	6.76212	0.646716	12.8775	OK	OK	0.485726
ENSG0000015	-	-	ENSG0000015888	MPZ	-	1:161304734-161309972	-	-	IGV_LINK	79.5171	3.25418	0	6.52694	OK	48.4676	2.36214	0	5.03092	OK	OK	-0.4622
ENSG0000015	-	-	ENSG0000015890	WFDC8	-	20:45551152-45579326	-	-	IGV_LINK	0	0	0	0	OK	0	0	0	0	OK	NOTEST	0
ENSG0000015	-	-	ENSG0000015894	CCAR2	-	8:22604631-22669148	-	-	IGV_LINK	1472.04	60.3168	0	145.14	OK	1609.74	59.9981	0	146.719	OK	OK	-0.00764
ENSG0000015	-	-	ENSG0000015895	WNT9B	-	17:46833200-46886730	-	-	IGV_LINK	1.93944	0.045335	0	0.285086	OK	2.06245	0.071766	0	0.327941	OK	NOTEST	0.662689
ENSG0000015	-	-	ENSG0000015896	CACHD1	-	1:64470791-64693058	-	-	IGV_LINK	59.153	0.653327	0	1.38859	OK	151.59	1.6559	0.311007	3.00079	OK	OK	1.34173
ENSG0000015	-	-	ENSG0000015898	CDC42SE2	-	5:131245492-131398447	-	-	IGV_LINK	2200.3	40.4701	1.99224	79.039	OK	1672.65	29.6036	5.35197	53.8553	OK	OK	-0.45108
ENSG0000015	-	-	ENSG0000015898	RAPGEF6	-	5:131423920-131797063	-	-	IGV_LINK	637.106	5.46357	0.500948	10.4262	OK	480.551	4.11283	0.274301	7.95135	OK	OK	-0.40972
ENSG0000015	-	-	ENSG0000015902	EPB41	-	1:28887090-29123935	-	-	IGV_LINK	2001.5	28.9666	0	64.3608	OK	2534.75	42.8897	0	120.689	OK	OK	0.566242
ENSG0000015	-	-	ENSG0000015905	MIS18A	-	21:32268218-32280988	-	-	IGV_LINK	90.184	3.96953	0	8.32921	OK	104.154	4.18863	0.329251	8.04801	OK	OK	0.077511
ENSG0000015	-	-	ENSG0000015906	ALG8	-	11:78100935-78139660	-	-	IGV_LINK	385.949	19.0103	4.88937	33.1311	OK	466.114	21.3557	6.49823	36.2132	OK	OK	0.167844
ENSG0000015	-	-	ENSG0000015906	FBXW5	-	9:136940434-136944696	-	-	IGV_LINK	2288.54	65.3572	24.7611	105.953	OK	2634.78	77.1053	29.9775	124.233	OK	OK	0.238485

ENSG0000015	-	-	ENSG0000015907	'C21orf59	-	21:32572237-32612866	-	-	IGV_LINK	593.351	22.5022	7.39597	37.6085	OK	719.168	30.1445	10.6985	49.5905	OK	OK	0.421828
ENSG0000015	-	-	ENSG0000015908	'SYNJ1	-	21:32628758-32728048	-	-	IGV_LINK	419.889	4.98918	1.36788	8.61048	OK	416.615	5.01038	1.40623	8.61454	OK	OK	0.006118
ENSG0000015	-	-	ENSG0000015908	'PAXBP1	-	21:32728114-32771858	-	-	IGV_LINK	736.018	17.0256	0	35.9257	OK	487.769	9.80181	0	20.0723	OK	OK	-0.79658
ENSG0000015	-	-	ENSG0000015911	'IFNAR2	-	21:33229900-33310187	-	-	IGV_LINK	738.123	25.4075	0	54.0565	OK	866.417	27.0271	0	57.9482	OK	OK	0.089156
ENSG0000015	-	-	ENSG0000015911	'MRPL10	-	17:47823271-47831534	-	-	IGV_LINK	688.502	26.2742	10.316	42.2323	OK	905.416	35.157	12.7882	57.5257	OK	OK	0.420165
ENSG0000015	-	-	ENSG0000015912	'IFNGR2	-	21:33402895-33480011	-	-	IGV_LINK	2188.66	85.4241	27.8855	142.963	OK	2199.6	84.8858	30.6551	139.116	OK	OK	-0.00912
ENSG0000015	-	-	ENSG0000015913	'GART	-	21:33503930-33977691	-	-	IGV_LINK	905.719	23.0973	0	106.284	OK	918.821	21.6817	0	91.9268	OK	OK	-0.09125
ENSG0000015	-	-	ENSG0000015914	'SON	-	21:33503930-33977691	-	-	IGV_LINK	6356.52	112.238	0	323.829	OK	4686.92	79.6515	0	228.208	OK	OK	-0.49479
ENSG0000015	-	-	ENSG0000015914	'DONSON	-	21:33503930-33977691	-	-	IGV_LINK	154.186	7.43934	0	63.3451	OK	188.714	8.44151	0	62.4542	OK	OK	0.182326
ENSG0000015	-	-	ENSG0000015916	'SV2A	-	1:149903317-149917882	-	-	IGV_LINK	86.3051	1.14617	0.109023	2.18332	OK	27.8431	0.388998	0	0.838242	OK	OK	-1.55899
ENSG0000015	-	-	ENSG0000015916	'LAD1	-	1:201359007-201401190	-	-	IGV_LINK	199.762	5.94551	0	12.5905	OK	34.0304	1.20795	0	3.26673	OK	OK	-2.29924
ENSG0000015	-	-	ENSG0000015916	'STC1	-	8:23841914-23854807	-	-	IGV_LINK	1742.59	27.2005	11.7452	42.6558	OK	2916.3	46.401	19.4098	73.3922	OK	OK	0.770521
ENSG0000015	-	-	ENSG0000015917	'TNNI1	-	1:201403767-201429866	-	-	IGV_LINK	10.6669	0.100705	0	0.479153	OK	1.03122	0.011091	0	0.36963	OK	NOTEST	-3.18263
ENSG0000015	-	-	ENSG0000015917	'CSRP1	-	1:201483529-201534784	-	-	IGV_LINK	1535.07	47.0983	15.8652	78.3315	OK	2006.76	64.8494	20.9313	108.768	OK	OK	0.461418
ENSG0000015	-	-	ENSG0000015918	'PRAC1	-	17:48721718-48722522	-	-	IGV_LINK	0	0	0	0	OK	0	0	0	0	OK	NOTEST	0
ENSG0000015	-	-	ENSG0000015918	'HOXB13	-	17:48724762-48729178	-	-	IGV_LINK	2.90916	0.049764	0	0.133566	OK	25.7806	0.441158	0	0.91538	OK	NOTEST	3.14812
ENSG0000015	-	-	ENSG0000015918	'AC007383.1	-	2:206175315-206266243	-	-	IGV_LINK	0	0	0	0	OK	0	0	0	0	OK	NOTEST	0
ENSG0000015	-	-	ENSG0000015918	'C1QC	-	1:22643629-22648110	-	-	IGV_LINK	6598.95	376.175	142.793	609.557	OK	9503.77	542.04	182.266	901.814	OK	OK	0.526994
ENSG0000015	-	-	ENSG0000015919	'KCNE2	-	21:34364023-34375348	-	-	IGV_LINK	3.87888	0.248836	0	0.794026	OK	9.28102	0.596173	0	1.47693	OK	OK	1.26053
ENSG0000015	-	-	ENSG0000015919	'ATP5G1	-	17:48874859-48929056	-	-	IGV_LINK	679.774	98.1847	0	246.121	OK	944.602	145.449	0	368.752	OK	OK	0.566938
ENSG0000015	-	-	ENSG0000015920	'RCAN1	-	21:34513141-34615142	-	-	IGV_LINK	521.71	13.3804	5.1085	21.6524	OK	1369.47	33.932	14.6963	53.1677	OK	OK	1.34252
ENSG0000015	-	-	ENSG0000015920	'UBE2Z	-	17:48874859-48929056	-	-	IGV_LINK	2820.92	62.2087	7.27825	117.139	OK	3433.98	74.4712	11.5177	137.425	OK	OK	0.259566
ENSG0000015	-	-	ENSG0000015920	'CIART	-	1:150282542-150287093	-	-	IGV_LINK	90.184	4.75816	0	9.71269	OK	63.9359	3.24524	0.063363	6.42712	OK	OK	-0.55208
ENSG0000015	-	-	ENSG0000015921	'SNF8	-	17:48929315-48945117	-	-	IGV_LINK	895.052	59.6299	20.4081	98.8517	OK	1087.94	81.7791	27.883	135.675	OK	OK	0.455696
ENSG0000015	-	-	ENSG0000015921	'CLIC6	-	21:34669388-34718227	-	-	IGV_LINK	10.6669	0.165434	0	0.380106	OK	262.962	4.07984	1.26823	6.89145	OK	OK	4.62418
ENSG0000015	-	-	ENSG0000015921	'CCDC24	-	1:43991358-44115913	-	-	IGV_LINK	535.286	23.0184	4.44796	41.5888	OK	442.396	20.144	4.54434	35.7437	OK	OK	-0.19243
[truncated: 8,228,020 more chars]
